# Expression of immune-related genes and possible regulatory mechanisms in ulcerative colitis

**DOI:** 10.3389/fmolb.2026.1621643

**Published:** 2026-03-05

**Authors:** Fanfan Qu, Baoqing Xu, Yi Zhou, Yang He, Yanda Wang, Jiaxin Li, Jiayin Li, Aihua Shen

**Affiliations:** 1 First Teaching Hospital of Tianjin University of Traditional Chinese Medicine, National Clinical Research Center for Chinese Medicine Acupuncture and Moxibustion, Tianjin, China; 2 Tianjin University of Traditional Chinese Medicine, Tianjin, China; 3 Medical College, Yanbian University, Yanji, China

**Keywords:** biomarker genes, immune-related genes, single-cell sequencing, ulcerative colitis, weighted gene co-expression network analysis

## Abstract

**Background:**

The abnormal immune response may lead to lesions of the intestinal mucosal layer in ulcerative colitis (UC). Immune-related genes (IRGs) are crucial for the immunological reaction in UC. However, the IRGs and their regulatory mechanisms in UC remain incompletely understood. Identification of IRGs in UC is essential for understanding its pathogenesis and developing new targeted therapeutic modalities.

**Methods:**

In this study, we combined the R package “SingleR” with manual inspection methods to annotate single-cell RNA-seq data. We then performed differentially expressed genes (DEGs) analysis and pseudo-time analysis. Additionally, we performed weighted gene co-expression network analysis (WGCNA) and identified IRGs in bulk sequencing of UC intestinal tissues. Afterward, GO and KEGG analyses were performed on scRNA and bulk sequencing data. From the Human TFDB database, pertinent regulatory transcription factors (TFs) were found. Using the STRING database, the protein-protein interaction (PPI) network of important TFs was created. Finally, candidate IRGs were validated experimentally by qRT-PCR and immunohistochemistry in colon tissues of DSS-induced UC mouse models.

**Results:**

We verified that the relevant IRGs were highly expressed in T and B cells of UC patients by the single-cell technique. Moreover, analysis of IRGs’ regulatory TFs revealed that 11 TFs were associated with the expression of IRGs. Through co-expression analysis and database screening, HNF4A was identified as a key transcription factor among them, and PPI network analysis further indicated its central regulatory role. Immune checkpoint analysis showed significant differences in PVR, ICOS, and CD28 (*P* < 0.001). Experimental validation confirmed that CD28 was significantly upregulated at both mRNA (*P* < 0.05) and protein levels (*P* < 0.001) in DSS-induced colitis mouse models.

**Conclusion:**

Our findings suggest that HNF4A may be associated with T cell activation and potential regulation of CD28 in UC. Importantly, we improved our understanding of the immune landscape in UC inflammatory tissue using scRNA-seq and bulk sequencing data.

## 1 Introduction

Ulcerative colitis (UC) is a complex, chronic, immune-mediated inflammatory condition of the colon. Pathologically, UC patients’ aberrant immune responses most likely cause lesions of the intestinal mucosal layer, such as extensive epithelial destruction, immune cell infiltration, crypt abscesses, and persistent inflammation (Schirmer et al., 2018). Although the pathogenesis of UC is not fully known, immune-mediated processes that cause dysregulated immune responses to luminal antigens in people with genetic susceptibility constitute a significant etiology (Koch et al., 2016).

While many medicines are effective in inducing and sustaining remission, some UC patients are resistant to or no longer respond to existing therapy. As a result, there is an urgent need to expand UC treatment options (Lamb et al., 2019). Improved understanding of inflammatory landscapes in tissue could lead to the identification of new therapeutic targets. Currently, the majority of UC analysis is focused on studying discrete cell populations and signaling pathways; a deeper comprehension of cell specificity in human tissues is lacking. Single-cell analysis in UC broadens research paths by capturing a far more comprehensive cellular environment (Gudiño et al., 2025). Additionally, the single-cell analysis provides new ideas for diagnosis and personalized disease treatment of UC.

Disturbances in the intestinal immune system play a crucial role in the development of UC, where dysregulation of T and B lymphocytes contributes to intestinal inflammation (Roh et al., 2019; Kamble et al., 2025). In particular, CD4^+^ T cells drive intestinal inflammation, and activated T cells accumulate in the inflamed areas of UC (Yeung et al., 2000). While immune-related genes (IRGs) are known to regulate the activation and homeostasis of T and B cells (Della Corte et al., 2020), their specific roles in UC remain insufficiently explored. CD28, as a co-stimulatory molecule for T cell activation, plays a role in autoimmune diseases. Genome-wide association studies (GWAS) suggest that it may be a susceptibility gene for UC, but its role in the pathogenesis of UC remains unclear (Liu et al., 2015).

Accordingly, the present study aims to identify IRGs associated with UC and potential diagnostic biomarkers through bioinformatics analysis, and to validate key genes (such as CD28) and their regulatory roles in UC through experimental validation. We anticipate that these findings will enhance the understanding of UC pathogenesis and provide valuable insights for the development of early diagnostic and therapeutic strategies.

## 2 Materials and methods

### 2.1 ScRNA-seq data processing

We obtained three UC single-cell RNA-sequencing datasets [GSE116222; GSE114374; GSE95459] and one UC RNA bulk dataset [GSE165512] from GEO (http://www.ncbi.nlm.nih.gov/geo). Following standardization, samples deficient in clinical information were discarded (Guo et al., 2023). The following are nine samples in GSE116222 (3 Healthy, three adjacent non-inflamed areas of UC, and three inflamed areas of UC samples), four samples in GSE114374 (2 Healthy, 2 UC samples), and 10 samples in GSE95459 (5 Healthy, 5 UC samples). Additionally, 86 samples were obtained from GSE165512 (46 Healthy and 40 UC samples). Dataset GSE95459 is specifically designed for analyzing epithelial cell heterogeneity and gene expression in UC. All analyses related to immune cell populations (e.g., T cells, B cells) and IRGs (e.g., *CD28*) were conducted using the GSE116222 and GSE114374 datasets, which contain full-thickness mucosal biopsies encompassing diverse cell types. Table 1 displays the features of the four datasets. The clinical information of GSE116222 samples is provided in Supplementary Table S1.

**TABLE 1:** Characteristics of the four datasets.

Datasets	Healthy	UC		Platform	Year	Source
		Adjacent non-inflamed area	Inflamed area			
GSE116222	3	3	3	GPL24676	2019	United Kingdom
GSE114374	2	0	2	GPL20301	2020	United Kingdom
GSE95459	5	0	5	GPL16791 GPL20301	2019	United Kingdom
GSE165512	46	40	0	GPL16791	2021	Italy

### 2.2 Single-cell quality control and dimensional reduction

We retained cells that expressed above 200 but no more than 4,000 genes. Meanwhile, a cutoff value of 5% of mitochondrial genes was established for further filtering. To produce cell clusters that could be shown and annotated using the t-SNE diagram, the number of principal components (PCs) was increased to 20 after 1,500 hypervariable genes were identified for study. The top 10 unique expression genes in each cluster were then selected using the “FindAllMarkers” function of the Seurat R package. Then a total of 11 clusters were found (Supplementary Table S2).

### 2.3 Differentially expressed genes (DEGs) analysis and cell type annotation

To automatically label our single-cell RNA-seq data, we used the R package “SingleR”. Between the expression profiles of each cell and those of the reference sample, Spearman’s correlation was calculated. According to a prior study, cell type identification is based on differentially expressed genes (DEGs) in each cluster with manual verification (Zhang et al., 2019). We applied this strategy repeatedly for each label, annotating the cell with the label that received the highest score. We found the genes that were differentially expressed between UC and normal cells using the “FindMarkers” approach. A list of each major indication that separates UC from normal cells may be seen in Supplementary Table S3. The reference marker gene list used for manual annotation is provided in Supplementary Table S4.

### 2.4 Pseudotime analysis

After each cell had been annotated, the inflammatory cell objects were extracted, and cells with mean expression >0.1 and dispersion empirical >1 * dispersion fit for the next stage of the pseudotime study (Xiong et al., 2022). We then used the “DDRTree” technique to decrease the dimension of the cells, using the reduceDimension function to identify the type of cell differentiation state. Finally, we used the plot_cell_trajectory function to depict the cell differentiation trajectory.

### 2.5 Bulk sequencing data processing

We used the limma package (version 3.8) to identify DEGs on the raw data of GSE165512 (Ritchie et al., 2015; Tang et al., 2022). Significantly differentially expressed genes (DEGs) are those with an absolute |log_2_FC| >1 and adjusted *p*-value <0.05. With the help of the ggplot2 program, volcano and heatmap plots were created (version 3.6.3). After that, we carried out GO and KEGG analyses as well as ranking analyses of the DEGs. Table 1 provides a comprehensive list of all datasets used in this investigation.

### 2.6 Weighted gene Co-Expression network analysis (WGCNA)

On GSE165512, we ran a Weighted Gene Co-Expression Network Analysis (WGCNA) analysis (Guo et al., 2024) and then used a subset of genes with an expression standard deviation greater than 0 for additional analysis, removing outlier data. The data were separated into distinct modules by selecting an optimum soft threshold (Supplementary Table S5) and concurrently identifying the modules that were most closely related to UC.

### 2.7 Consensus clustering and immune-related genes (IRGs) analysis

The R program ConsensusClusterPlus was used to group the inflammatory cells into various categories. When the clustered index “k” increases from two to 9, choose an appropriate value of k that maximizes the difference between clusters. Then, we draw a box diagram with the R package ggplot2 and the R package reshape2. To explore whether there is a correlation between genes in branches and clusters. Using the CIBERSORT algorithm, we summarized the distribution of immune cell subsets in inflammation samples in UC. In addition, we further performed an analysis of immune cells and IRGs among different types.

### 2.8 GO and KEGG analysis

For GO and KEGG analysis, the DEGs for the T cluster in scRNA sequencing data and DEGs in bulk sequencing data were separately imported into xiantao, an online bioinformatic analysis platform (https://www.xiantao.love/) (Zhou et al., 2022). Based on *p*-value ranking, the top 10 paths were chosen.

### 2.9 PPI network construction

Protein-protein interaction (PPI) network analysis was performed using STRING (https://string-db.org/).

### 2.10 Quantitative Real-Time PCR analysis

Colitis was induced in male C57BL/6 mice by treatment with 3% dextran sulfate sodium (DSS) for 7 days. Successful modeling was confirmed when the disease activity index (DAI) score exceeded 3, indicating weight loss, diarrhea, and bloody stools. Colon tissues were collected from DSS-induced colitis model mice (n = 6) and healthy control mice (n = 6), and total RNA was extracted using Trizol reagent (Thermo Fisher Scientific, MA, USA). 5 μg of total RNA was reverse-transcribed into cDNA following the manufacturer’s instructions. CD28 mRNA expression was measured using SYBR Green Master Mix (Promega, WA, USA) and the CFX 96 Real-Time PCR System (Bio-Rad Laboratories, CA, USA). Primer sequences are listed in Table 2 and were synthesized by Wuhan Service Biotech. GAPDH served as an internal reference, and relative expression levels were calculated using the 2 (−ΔΔCt) method.

**TABLE 2:** Primer sequence.

Primer name	Sequence
CD28	forward:5′-GACACTCAGGCTGCTGTTCTTG-3′, reverse: 5′-GAGGCTGACCTCGTTGCTATCT-3′
GAPDH	forward:5′-CCTCGTCCCGTAGACAAAATG-3′, reverse:5′-TGAGGTCAATGAAGGGGTCGT-3′

### 2.11 Immunohistochemical analysis

Colon tissue slices from the colitis model mice (n = 6) and the healthy control mice (n = 6) were collected. After sacrificing the mice, tissue was washed with ice-cold phosphate-buffered saline (PBS), fixed with 4% formaldehyde, and embedded in paraffin. Tissue sections (4 μm thick) were stained with anti-CD28 antibody (clone number, EPR22076, Abcam) using immunohistochemistry (Fujii et al., 2021). Two researchers independently evaluated each section. Sections from each mouse were randomly examined using four different visual fields to calculate the average optical density value for each tissue section.

### 2.12 Statistical analysis

RNA-Seq differential expression was analyzed using limma with Benjamini–Hochberg FDR correction. WGCNA used Pearson correlation for module-clinical trait associations. IRGs and DEG intersections were analyzed with GO and KEGG enrichment (*P* < 0.01). Single-cell analysis used Seurat and SingleR, and immune cell infiltration was assessed with CIBERSORT. Group differences were tested with an unpaired t-test (*P* < 0.05).

## 3 Results

### 3.1 Single-cell quality control and identified high-variable genes: TPSB2 and TPSAB1 are the top two HVGs

**FIGURE 1:**
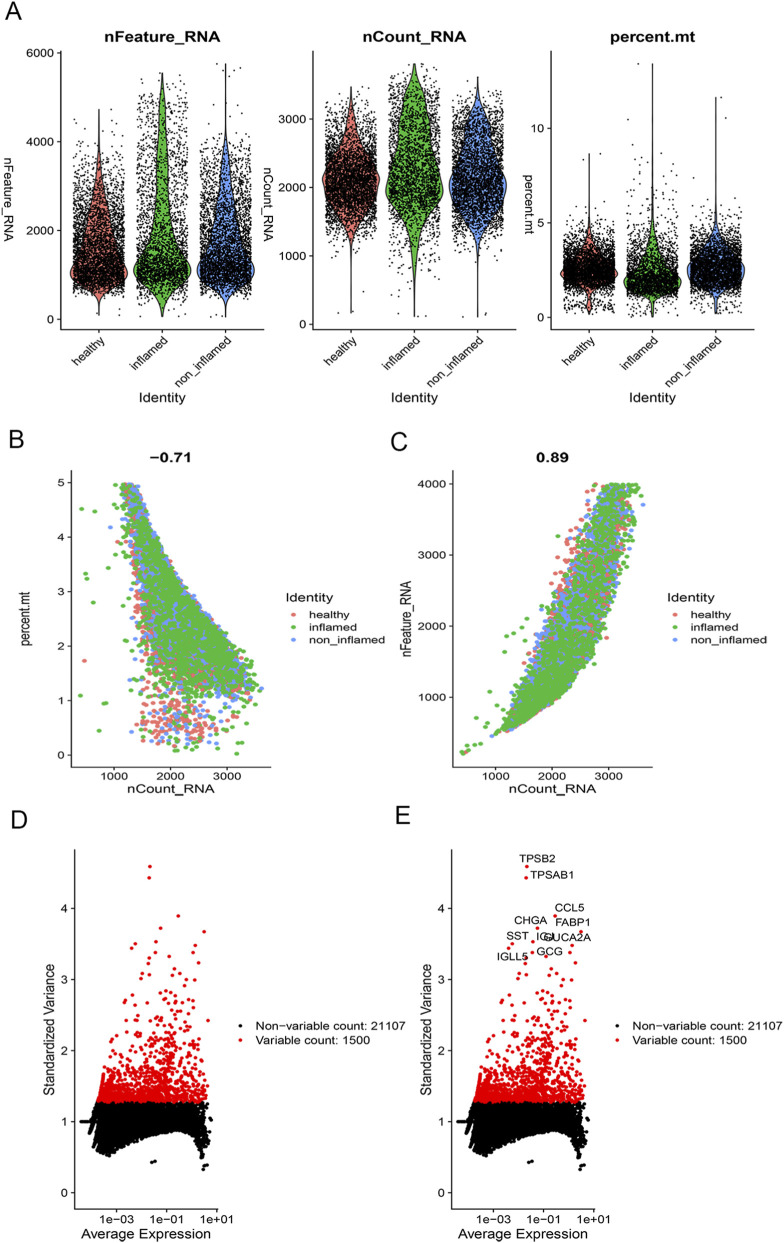
Clustering of GSE116222 single cells for quality control and identified high variable genes. **(A)** The genes (features), counts, and mitochondrial gene percentage of each sample; **(B)** Correlation between mitochondrial gene percentage and total gene count; **(C)** Correlation between the nCount_RNA and nFeature_RNA in each sample; **(D)** 1500 highly variable genes (HVGs) were colored in red; **(E)** The top 10 HVGs were labeled.

An exercise in quality control was done on the single-cell dataset. To ensure the quality of the cell samples utilized in the research, as indicated in Figure 1A, we excluded specific cells with <200 genes, >4,000 genes, and controlled the fraction of mitochondrial genes. With a correlation value of 0.71, the nCount RNA, which indicates the number of distinct molecular identifiers, is inversely linked with the percentage of mitochondrial genes (Figure 1B). With a correlation coefficient of 0.89, the number of genes represented by nFeature RNA and nCount RNA is positively associated (Figure 1C). Thereafter, we identified 1,500 genes with high variability, all of which are indicated in red, and marked the 10 most important genes (Figures 1D,E). TPSB2 and TPSAB1 are the top two HVGs, which are neutral proteases present in mast cells. They are elevated in the colon of inflammatory bowel disease patients and may be involved in the innate immune response (Cenac et al., 2002). TPSB2 and TPSAB1 fold changes and significance are shown in Supplementary Table S6.

### 3.2 Single-cell dimension reduction Clustering: 20 principal components dimensionality reduction analysis

**FIGURE 2:**
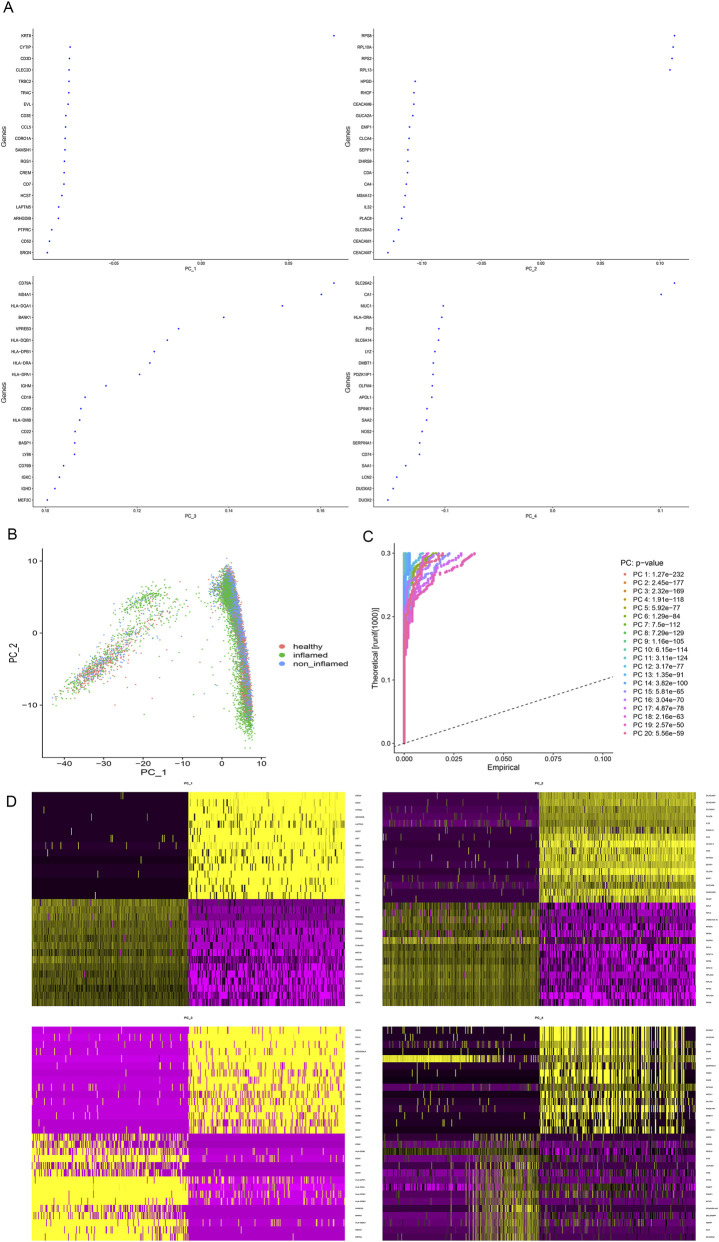
scRNA analysis of PCA dimensionality reduction. **(A)** Each gene associated with a principal component; **(B)** The coordinates of each cell in PC-1 and PC_2; **(C)** PCs selection using JackStraw function; **(D)** Heat map of highly expressed in each PC.

As shown in Figure 2A, we first performed PCA dimensionality reduction analysis to obtain the genes associated with each PC. Then we calculated the coordinates of each cell in PC-1 and PC-2 by PC correlation coefficient, and labeled them (Figure 2B). Using JackStrawPlot, PCA detected all 20 PCs with a *p*-value <0.05 (Figure 2C), and then the 20 PCs were analyzed by TSNE dimensionality reduction. The genes highly expressed in each PC were marked in yellow in the heat map (Figure 2D).

### 3.3 DEGs analysis and cell type annotation: T cell marker gene expression

**FIGURE 3:**
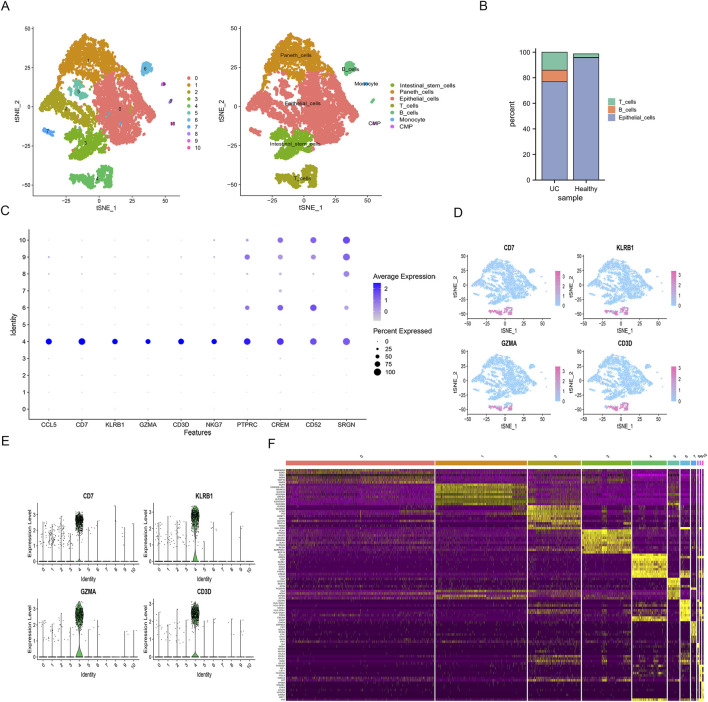
Marker gene expression of each cluster. **(A)** tSNE reduced dimensionality and cluster analysis. Different cell types were colored with unique colors; **(B)** T cells and B cells Cluster distribution in each sample; **(C)** Dot plot of cell type marker genes. The color of dots represents average expression, and size of dots represents average percent of cells expressing selected gene; **(D)** Expression distribution of CD7, KLRB1, GZMA, CD3D. The darker the color, the higher the expression; **(E)** Violin plot depicts distributions of cell type marker genes in T cells cluster. The width of each violin plot corresponds with the frequency of cells with relevant gene expression level; **(F)** Heatmap of top 10 DEGs in each cluster (Cluster 0,2,5,7 is Epithelial cells, Cluster 1 is Paneth cells, Cluster 3 is Intestinal stem cells, Cluster 4 T cells, Cluster 6,8 is B cells, Cluster 9 is Monocyte, Cluster 10 is CMP cells). The top 10 DEGs were labeled in yellow color.

We found the genes that were significantly different between UC and normal cells using the “FindMarkers” approach. A previous study suggested that these clusters could be connected to known cell lineages using marker genes (Sinha et al., 2018). Visualization of 11 clusters using the t-SNE analysis (Figure 3A). T cells and B cells cluster revealed an increased percentage in the UC group (Figure 3B), and the T cells cluster was the main focus of the analysis that followed. The dot plot (Figure 3C) and violin plot (Figure 3E) display the expression of T cell type marker genes. In addition, we mapped t-SNE plots of T cell type marker genes in all clusters (Figure 3D). The DEGs in each cell cluster were heatmapped, and the top 10 DEGs were marked with a yellow sticker (Figure 3F).

### 3.4 Pseudotime analysis: T cells and B cells differentiate later

**FIGURE 4:**
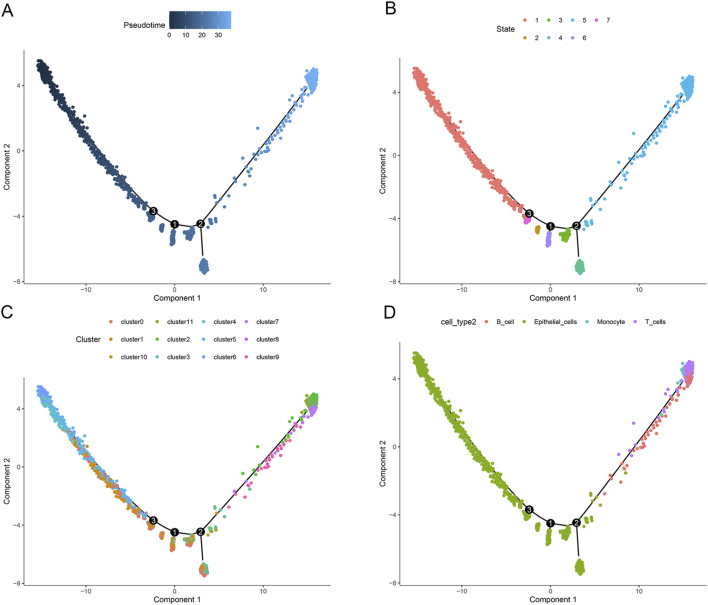
Analyses of pseudotime. **(A)** Timing differences in cell differentiation. Darker blue represents an earlier stage of differentiation, while a lighter blue indicates a later stage of differentiation; **(B)** Six stages of inflammatory cells differentiation. State 1 is the earliest stage of differentiation; **(C)** The differentiation process of different cell clusters; **(D)** Cell type of all analyzed cells.

We used simulation to analyze the cell trajectory differentiation of UC cells. We found that the darker the blue, the earlier the cell differentiation, and the lightest blue denoting the most recently differentiated cells (Figure 4A), indicating that inflammatory cells gradually differentiate from left to right. All inflammatory cells had six distinct developed states, each designated with a different hue, as seen in Figure 4B, with the red type being the earliest differentiated type. After that, we looked into how various cell clusters differentiate (Figure 4C). All cells were analyzed, including B cells, T cells, monocytes, and epithelial cells (Figure 4D). We discovered that T cells and B cells differentiated later than normal epithelial cells.

### 3.5 DEGs of UC from bulk sequencing data: Bile secretion, fat digestion and absorption, etc are enriched in DEGs

**FIGURE 5:**
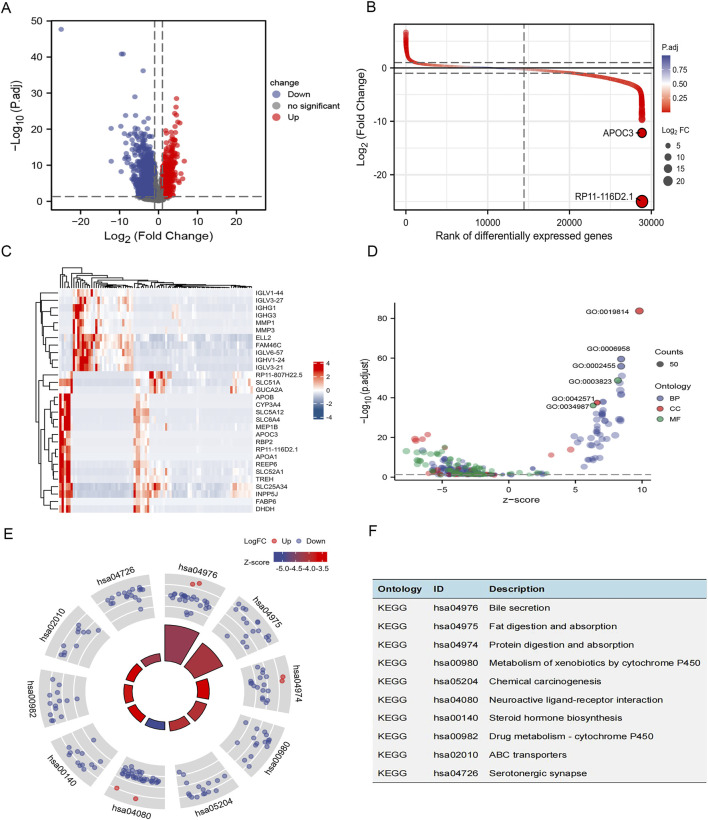
DEGs of UC from GSE165512 dataset. **(A)** Volcano plot of DEGs. Up-regulated genes were colored in red and downregulated genes were colored in blue; **(B)** The difference ranking chart. The abscissa is the position of differentially expressed genes sorted according to their multiples, and the ordinate is the differentially expressed multiples. The closer the points are to the left and right, the greater the absolute value of the difference; **(C)** A heatmap of the top 30 regulated DEGs is shown; **(D)** GO of DEGs in GSE165512. The top 2 BPs include complement activation, classical pathway (GO:0006958), humoral immune response mediated by circulating immunoglobulin (GO:0002455). The top 2 CCs include immunoglobulin complex (GO:0019814), immunoglobulin complex, circulating (GO:0042571). The top 2 CCs include antigen binding (GO:0003823), immunoglobulin receptor binding (GO:0034987); **(E)** Enrichment analysis of DEGs between UC and normal samples using the KEGG; **(F)** The top 10 KEGGs were analyzed.

The bulk RNA sequencing dataset GSE165512, which includes 40 UC patients and 46 healthy controls, was used to examine the gene expression characteristics in UC. After the null value is eliminated, there are 28,864 IDs remaining. DEGs with |log_2_FC| >1 and adjusted *p*-values <0.05 were chosen (Supplementary Table S7). Following that, 947 upregulated and 4,633 downregulated DEGs were kept (Figure 5A). The difference multiples and adjusted *p*-values were obtained after the difference analysis. The difference ranking chart was made by the size of the final difference multiples data to show the results of difference analysis (Figure 5B). Relative consistency was seen within groups in the heatmap of the top 30 upregulated and top 30 downregulated DEGs (Figure 5C). Next, we used the GO database to obtain the association of differentially expressed genes at three levels: biological process (BP), cellular component (CC), and molecular function (MF). Interestingly, the top two terms of BP, CC, and MF of DEGs in UC were mainly focused on immune response (Figure 5D). The KEGG pathway was enriched for Bile secretion, Fat digestion and absorption, Protein digestion and absorption, Metabolism of xenobiotics by cytochrome P450, Chemical carcinogenesis, Neuroactive ligand-receptor interaction, Steroid hormone biosynthesis, Drug metabolism cytochrome P450, ABC transporters, Serotonergic synapse (Figures 5E,F).

### 3.6 WGCNA: related genes in immune response pathways

**FIGURE 6:**
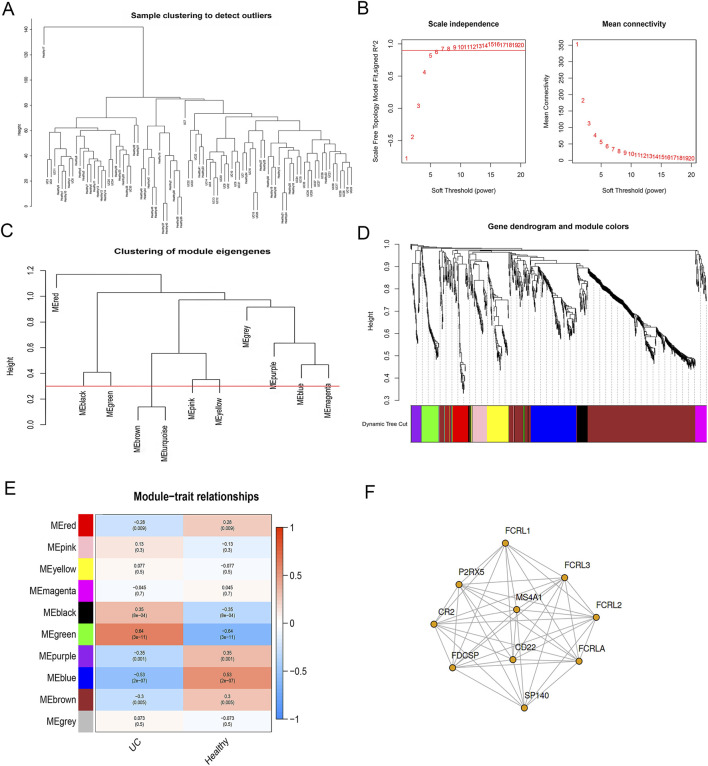
Analysis of the weighted co-expression network in GSE165512. **(A)** Sample clustering of dataset GSE165512; **(B)** Selection of optimal thresholds. The threshold is 15; **(C)** Set the threshold to 0.3 to merge modules that are comparable in the cluster tree; **(D)** Different modules are produced and shown in different colors by aggregating genes with strong correlations into a same module. Brown modules make up a greater proportion; **(E)** Analysis of correlations between modules and UC; **(F)** The top 10 WGCNA-hub genes were provided to the genes in the green module. UC, ulcerative colitis.

First, we discovered 993 genes using WGCNA on GSE165512. Second, the cluster analysis was carried out using the “flashClust” utility package; the samples were grouped into four statistically different groups. The clusters with a small number were removed, and the other three clusters were used for further analysis (Figure 6A). The “choose Soft Threshold” function of the “WGCNA” package was then used to filter the power parameter range of 1–20. For the purpose of building a scale-free network, we chose a power of b = 15 as the soft threshold (Figure 6B). We set the threshold to 0.3 (Figure 6C) and the minimum number of modules to 30 to combine related modules in cluster 3. Ten modules encompassing genes with comparable co-expression characteristics were created (Figure 6D). Multiple modules were related to UC, with the green module being the most important and encompassing 69 genes, as shown by module-trait association analyses (Figure 6E). The green module showed a significant association with both healthy samples and UC (COR = 0.64, *P* < 0.001). In the green module, we analyzed the top 10 WGCNA-hub genes (Figure 6F).

### 3.7 Typing of inflammation-related genes and immune cell analysis: Significant differences in PVR, ICOS, and CD28

**FIGURE 7:**
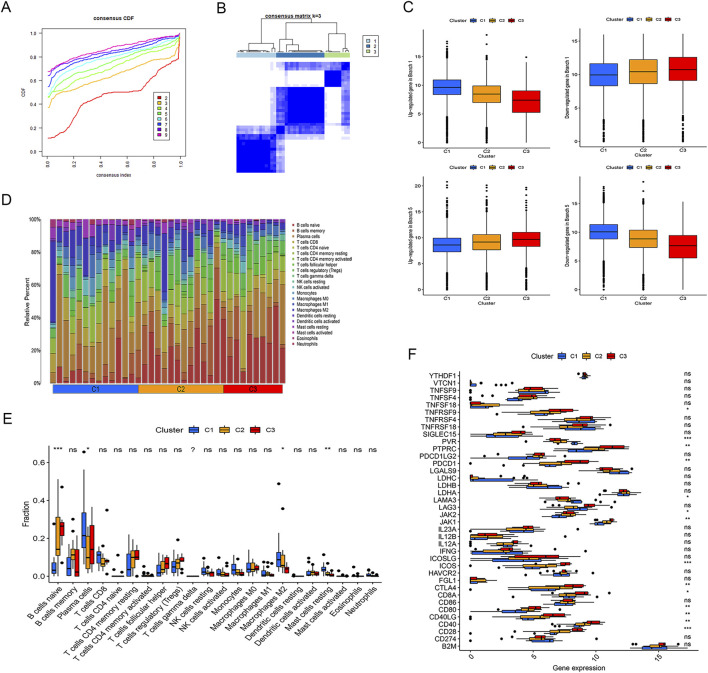
The type of Inflammation-related genes and immune cell analysis. **(A)** The Item-consensus plot shows the relationship between each cluster; **(B)** Consensus clustering matrix shows the optimal three clusters; **(C)** The correlation analysis between the genes in the branches and the typing clusters; **(D)** Heat map of the 22 immune cell subpopulations in inflammation samples; **(E)** Box diagram illustrating the proportion of 22 different kinds of immune cells indifferent clusters. (Cluster 1 was denoted by blue color, cluster 2 was denoted by saffron yellow color, cluster 2 was denoted by red color. *P*-values <0.05 were considered statistically significant); **(F)** Immune checkpoint analysis, the Y-axis represents immune checkpoint-related genes, and the X-axis represents gene expression levels. PVR, ICOS, CD28 is the most significant difference (*P <* 0.001). (*P < 0.05; **P < 0.01; ***P < 0.001).

To explore the heterogeneity of UC, we clustered inflammatory cells in the published dataset GSE165512 using a previously developed consensus unsupervised clustering technique. When K = 3, there is a flattest middle portion of the CDF curve in the consensus matrix (Figure 7A). Additionally, we discovered that when K = 3 was chosen for the consensus clustering analysis, the interference between subgroups could be minimized. Therefore, the analysis defined three clusters with the most robust classification (Figure 7B). Next, we performed expression analysis of the typing genes. We aimed to observe the correlation between the genes in the branches of the pseudotime analysis in the single-cell analysis and the clusters in the genotyping. We found that genes upregulated in branch 1 were highly expressed in cluster 1, and genes downregulated in branch 1 were also under-expressed in cluster 1. A similar pattern was found in branch 5 and cluster 3 (Figure 7C). We initially compiled the findings from UC samples using the CIBERSORT method. The correlation heatmap of the 22 immune cell subpopulations in inflammatory samples shows this (Figure 7D). By immune cell analysis, we found that there were significant differences in naive B cells (*P* < 0.001), Plasma cells (*P* < 0.05), M2 Macrophages (*P* < 0.05), and resting Mast cells (*P* < 0.01) between different types of clusters (Figure 7E). In addition, we further performed immune checkpoint analysis to observe whether there were differences in immune checkpoint-related genes among different types. We found differences in these immune checkpoints, such as TNFRSF9, PVR, PTPRC, PDCD1, ICOS, CTLA4, CD8A, CD80, CD40LG, CD40, CD28, among which PVR, ICOS, CD28 was the most significant difference (*P <* 0.001) (Figure 7F).

### 3.8 IRGs related regulatory transcription factors: 11 overlapping transcription factors

**FIGURE 8:**
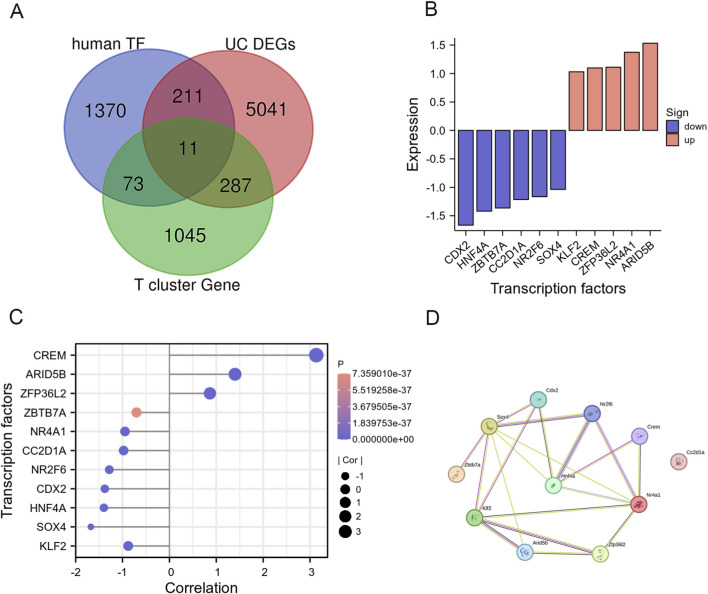
IRGs relevant regulatory transcription factors. **(A)** Venn plot showed TFs in T cell cluster from single cell data of UC in GSE116222, human TF database, and TFs in DEGs of UC intestinal tissue in GES165512; **(B)** The expression of common TFs in DEG of GES165512. Upregulated TFs were colored in pink, and downregulated TFs were colored in blue; **(C)** The expression of common TFs in T cell cluster from single cell data in UC. The size of the circle represents the degree of correlation, and the higher the degree of correlation, the larger the circle. The longer the rod, the higher the degree of correlation; **(D)** The PPI network of the common TFs illustrated using STRING. HNF4A serves as a hub gene.

To study the transcriptionally regulated activity of IRGs, we downloaded a list of human transcription factors from Human TFDB (http://bioinfo.life.hust.edu.cn/Human TFDB/#!/) (Hu et al., 2019). We screened 84 TFs in the UC T cluster in single-cell data and 222 TFs in the UC bulk data. Furthermore, the UC T cluster and the UC intestinal tissue both expressed 11 shared TFs (Figure 8A). With the default settings (only.pos = FALSE, min. pct = 0.25, and logfc. threshold = 0.5), the FindAllMarkers function was run. Across all clusters, 7,723 different marker genes were discovered. There were 1,416 marker genes found for the T cluster in total, however only 84 of these genes corresponded to TFs. There were only 11 overlapping TFs between the T marker genes, DEGs in the UC intestinal tract, and the TF database. The expression of 11 TFs in the data set GSE165512 and the single-cell T cluster is shown in Figures 8B,C. 5 TFs were upregulated in the UC intestinal, and 3 TFs were raised in T cells. As a hub gene for the transcriptional control of IRGs, the PPI network predicts that HNF4A may play a significant role (Figure 8D). The names of the 11 co-expressed TFs and their expression trends in UC and healthy samples are provided in Supplementary Table S8.

### 3.9 Verification of novel biomarker gene: CD28 is highly expressed in colon tissue

**FIGURE 9:**
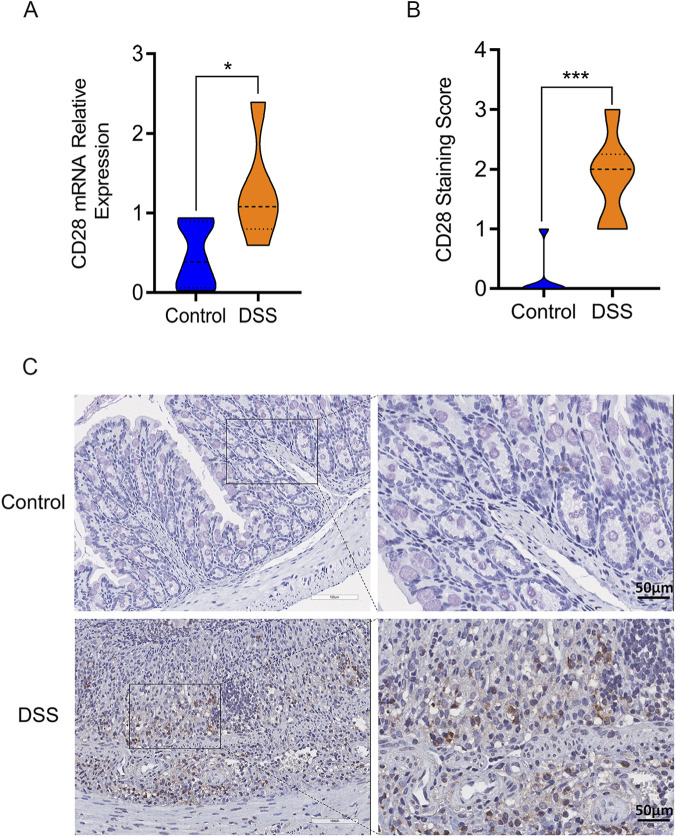
Verification of CD28 expression level. **(A)** CD28 showed upregulation in DSS-induced colitis mice by qRT-PCR; **(B)** Statistical analysis of IHC; **(C)** CD28 showed highly expressed in colonic tissues of DSS-induced colitis mice by IHC (scale bars: left, 100 μm; right, 50 μm). *P < 0.05, **P < 0.01, ***P < 0.001.

By using qRT-PCR, we were able to determine the biomarkers’ expression levels. CD28 showed a significant upregulation in DSS-induced model mice colonic tissues (*P* < 0.05) (Figure 9A). After that, the expression level of CD28 was detected by immunohistochemistry. CD28 was highly expressed in colonic tissues of DSS-induced colitis mice (*P* < 0.001) (Figures 9B,C).

## 4 Discussion

UC is a chronic inflammatory disorder caused by dysregulated immune responses in genetically predisposed individuals. It is possible to distinguish between genes that support innate and adaptive immune responses when analyzing the primary IRGs in UC. In general, it is believed that the pathophysiology of UC is mostly influenced by the adaptive immune system. T and B cells, which make up the lymphocytes of the adaptive immune response, produce effector responses when activated (cytokines and antibodies). After activation, native T-cells (Th0) can develop into Th1, Th2, or Th17 cells (Wallace et al., 2014). In particular, UC has been considered a Th2-dominant condition mediated by specialized cells such as natural killer T cells (Shih et al., 2008).

Recent research has started to use single-cell techniques to look into the processes underlying the complicated immune system dysregulation in UC (Martin et al., 2019). We conducted comprehensive investigations of its immunopathogenesis using single-cell transcriptomic and bulk sequencing data. First, we identified 7 cell clusters and found that the proportion of adaptive immune cells (T and B cells) increased in the UC group compared with healthy controls (Figure 3B). These results confirmed the abnormal activation of effector immune cells and the response of immune cells to inflammatory signals at the inflammatory site of UC. Consistent with previous reports, inappropriate immune activation is thought to underlie the pathogenesis of UC (Hudson et al., 2017; van der Post et al., 2019). We then performed an annotation analysis of the Marker genes for each cluster and found some genes were highly expressed in the T cell clusters, such as “CCL5”, “CD7”, “KLRB1”, “GZMA”, “CD3D”, “NKG7”, “PTPRC”, “CREM”, “CD52”, and “SRGN”. Recent studies reported that C-C chemokine ligand 5 (CCL5), a key proinflammatory chemokine, plays an important role in inflammation and in immune responses (Marques et al., 2013). Some researchers found that the CCL5 is upregulated in IBD tissues showing eosinophilia (Jeziorska et al., 2001). Further study is needed to determine whether CCL5 could serve as a new diagnostic biomarker for UC. Next, we used a pseudotime analysis to comprehend the temporal connections between the various cells. We discovered that T cells and B cells differentiated later than normal epithelial cells. When inflammation arises, the innate immune system is the initial line of defense and also aids in the start of the adaptive immune response. The epithelial cells and innate immune cells predate the emergence of adaptive immunity (Kennedy et al., 2020).

In the bulk RNA sequencing data of the UC human intestine, we identified 947 upregulated and 4,633 downregulated DEGs. The GO and WGCNA analysis of bulk RNA sequencing data focused on immune response, which was similar to single-cell analysis. These data are consistent with literature reports that it is important for immune response in the process of UC (Koch et al., 2016).

For a more thorough examination, we also combined data from bulk RNA sequencing and single-cell data analysis. We aimed to investigate these typical IRGs’ putative regulation mechanisms. We defined three clusters with the most robust classification in inflammatory cells in GSE165512. After pseudotime Analysis, we found that branch 5 mainly included B cells and T cells, and it had a positive correlation with cluster three in the inflammatory cell cluster. Therefore, we speculated that cluster three is mainly composed of B and T immune cells. To prove this hypothesis, we went on to analyze the correlation between the immune cells and the inflammation samples. To our excitement, there was indeed a significant increase in B cells in cluster 3, which was significantly different from the other clusters (Figures 7D,E). Then we continued to analyze IRGs in different clusters, and found that CD28 was highly expressed in cluster 3 (Figure 7F). CD28 is a costimulatory molecule expressed for T cells activation (Hui et al., 2017), and its high expression in cluster three indicates involvement in UC immune responses. CD38 expression has been linked to colitis development (Schneider et al., 2015), with CD38neg effector T cells showing reduced IFNγ and IL-17 under CD3/CD28 stimulation (Joosse et al., 2019).‌ Furthermore, the imbalance of CD8^+^CD28^+^ and CD8^+^CD28-T cells is associated with experimental colitis and may serve as an early indicator (Dai et al., 2017). These findings support our results and highlight a critical role of CD28 in UC pathogenesis.

By analyzing at TF DEGs, we further investigated the potential regulatory mechanisms of these widespread IRGs. We found that the UC T cluster and the UC intestinal tissue both expressed 11 shared TFs. HNF4A may serve as the hub TF in the regulation of IRGs, according to PPI network research. Studies have shown that HNF4A expression in intestinal epithelial cells is necessary for the normal development and composition of intraepithelial lymphocyte compartments. Moreover, HNF4A is directly involved in the regulation of immune signaling molecules in UC (Lei et al., 2022). The risk of UC may rise if HNF4A expression is reduced (Ahn et al., 2008). These data imply that HNF4A may be essential for T cell activation and intestinal infiltration in UC. Our study also found that HNF4A expression was decreased in intestinal tissue and T cell clusters in UC (Figures 8B,C). Currently, studies utilizing the GSE116222 dataset have shown that the frequency of IFNG^+^TNF^+^ EM T cells in UCa mucosa was significantly higher than that in UCin and HC mucosa (Luo et al., 2022). Furthermore, another study based on the GSE114374 dataset indicated that S1PR1/S1pr1 was mainly expressed by endothelial cells and showed a positive correlation with endothelial cell markers (Zheng et al., 2025).

Taken together, single-cell data and bulk RNA sequencing data may indicate that IRGs are highly expressed on adaptive immune cells such as T and B cells. Our data provide a novel insight into the potential role of HNF4A in the regulation of IRGs, particularly its association with CD28, in the context of UC pathogenesis. We integrated two mucosal biopsy scRNA-seq datasets (GSE116222, GSE114374) for immune cell analysis. Although batch effects were reduced, they were not completely eliminated, highlighting limitations in data integration. Additionally, while the purified epithelial dataset (GSE95459) served its purpose it differs fundamentally from mucosal biopsy samples in cellular composition, which may affect the interpretation of the results. Although our study suggests that HNF4A might contribute to T cell activation, infiltration, and potential regulation of CD28, the functional validation was limited to mouse models and lacks human primary T cell experiments. Therefore, further *in vivo* studies are needed to verify the regulatory mechanisms of HNF4A and CD28. Future studies should focus on larger, harmonized cohorts and advanced integration methods to address these issues.

## 5 Conclusion

In conclusion, using bioinformatic analysis incorporating both scRNA and bulk sequencing data, we suggest that adaptive immune cells are primary contributors to disease etiology in UC. Additionally, we discovered that IRGs are crucial for immune stimulation and identified CD28 to be a potential UC biomarker. Our findings suggest that HNF4A, a key transcription factor, may regulate CD28 expression in UC.

## Data Availability

The datasets presented in this study can be found in online repositories. The names of the repository/repositories and accession number(s) can be found in the article/Supplementary Material.
