# Supplementary material for: Expression of immune-related genes and possible regulatory mechanisms in ulcerative colitis
Source: Front Mol Biosci. 2026 Mar 5;13:1621643. doi: 10.3389/fmolb.2026.1621643 (PMC12999447; doi:10.3389/fmolb.2026.1621643)
Supplement: Supplementary file 1 [file Table5.pdf]

Supplementary Table 5 Modules with different data of WGCNA

Genes	Module Color
ACTR2	black
ARF1	black
ARHGDIB	black
ARPC2	black
BTF3	black
CEBPD	black
DSTN	black
EEF1A1	black
EEF2	black
EIF4B	black
EIF4H	black
FOSB	black
FTH1	black
FUS	black
GADD45B	black
GAPDH	black
GNAI2	black
H3-3A	black
H3-3B	black
HLA-DPA1	black
HLA-DQB1	black
HLA-DRA ENSG00000204287	black
HMGB1	black
HMGB2	black
HNRNPA1	black
HNRNPA2B1	black
HNRNPA3	black
HNRNPC	black
HNRNPD	black
HNRNPK	black
HSP90AB1	black
HSPA8	black
KLF2	black
LDHA	black
LENG8	black
MALAT1	black
MCL1 ENSG00000143384	black
MGP	black
NCL	black
NEAT1	black

NFKBIA	black
NPM1	black
PTMA	black
RHOA	black
RNF145	black
RPS2P5	black
SRRM2 ENSG00000167978	black
SRSF5 ENSG00000100650	black
STOM	black
TXNIP	black
UBB	black
VIM	black
YBX1	black
YWHAZ	black
ACADS	blue
ADH1C	blue
AGPAT2	blue
AGR2	blue
AKR1B10	blue
AMN	blue
ANPEP	blue
AOC1	blue
ASS1	blue
ATP1A1	blue
ATP5F1A	blue
B4GALNT3	blue
C11orf86	blue
C1orf115	blue
CACFD1	blue
CAMK2N1	blue
CBLC	blue
CDH1	blue
CDH17	blue
CDHR2	blue
CDHR5	blue
CDX2	blue
CEACAM1 ENSG00000079385	blue
CEACAM5	blue
CEACAM6	blue
CES2	blue
CHGA	blue
CHP2	blue
CISD3	blue

CLCA1	blue
CLCA4	blue
CLDN3	blue
CLDN4	blue
CLDN7	blue
CMBL	blue
CXCL14	blue
CYP3A5	blue
CYP4F12	blue
DEPP1 ENSG00000165507	blue
DGAT1	blue
DHRS11	blue
DMBT1	blue
DSP	blue
ECHS1	blue
ELMO3	blue
EPCAM	blue
EPS8L2	blue
ERBB3	blue
FABP1	blue
FABP2	blue
FCGBP	blue
FCGRT	blue
FRMD1	blue
FUT6	blue
GCNT3	blue
GMDS	blue
GNA11	blue
GPA33	blue
GPRC5A	blue
GPT	blue
GUCA2A	blue
GUCA2B	blue
HADHA	blue
HDHD3	blue
HMGCS2	blue
HSD11B2	blue
ID1	blue
ITLN1	blue
JUP	blue
KRT18	blue
KRT19	blue
KRT20	blue

KRT8	blue
LAD1	blue
LGALS3BP ENSG00000108679	blue
LGALS4	blue
LINC01133	blue
LLGL2	blue
LRP1	blue
LSR	blue
LYPD8	blue
MAOA	blue
MDK	blue
MEP1A ENSG00000112818	blue
MFGE8	blue
MISP	blue
MMP15	blue
MPST	blue
MSN	blue
MT1G	blue
MUC13	blue
MUC2	blue
MVP	blue
MYH14	blue
MYO15B	blue
MYO1A	blue
MYO1C	blue
MYO7B	blue
PADI2	blue
PCK1	blue
PDZK1IP1	blue
PEBP1	blue
PHGR1	blue
PIGR	blue
PKP3	blue
PLCB3	blue
PLEK	blue
PLS1	blue
PPP1R14D	blue
PPP1R1B	blue
PRSS8	blue
PTPRF	blue
PYGB	blue
PYY	blue
RBM47	blue

S100A14	blue
SDC1	blue
SDCBP2	blue
SELENBP1 ENSG00000143416	blue
SEMA3B	blue
SERINC2	blue
SLC22A18	blue
SLC26A2	blue
SLC26A3	blue
SLC27A4	blue
SLC39A4	blue
SLC39A5	blue
SLC44A4	blue
SLC5A1	blue
SLC6A8	blue
SLC9A3R1	blue
SMIM24	blue
SPINK1	blue
SPINK4	blue
SPINT1	blue
SRI	blue
ST14	blue
STAP2	blue
SULT1A1	blue
TFF1	blue
TFF3	blue
TMBIM6	blue
TMC4	blue
TMEM54 ENSG00000121900	blue
TMPRSS2	blue
TPRN	blue
TRIM31	blue
TRPM4	blue
TSPAN1	blue
TSPAN8	blue
TST	blue
UGT2B17	blue
UQCRC1	blue
USH1C	blue
VIL1	blue
VILL	blue
ZG16	blue
A2M	brown

ABCA7	brown
ABHD14B	brown
ACKR1	brown
ACP5	brown
ACTA2	brown
ACTG2	brown
ACTN1	brown
ADAM15	brown
ADAMDEC1	brown
ADAMTS1	brown
ADAP1	brown
ADH1B	brown
AHNAK	brown
ALDH1A1	brown
ALKBH7	brown
ANKRD22	brown
ANO9	brown
ANXA4	brown
ANXA5	brown
AP2S1	brown
APLP2	brown
APOE	brown
APP	brown
APRT ENSG00000198931	brown
AQP8	brown
ARFGAP1	brown
ARFRP1	brown
ARL2	brown
ARPC4	brown
ASPSCR1	brown
ATOX1	brown
ATP2A3	brown
ATP5F1D	brown
ATP5F1E	brown
ATP5MC1	brown
ATP6V0A1	brown
ATP6V0B	brown
ATP6V0D1	brown
AURKAIP1	brown
BCAM	brown
BCAP31	brown
BSG	brown
BST2	brown

C11orf96	brown
C1QA	brown
C1QB	brown
C1S	brown
C4orf48	brown
C7	brown
C7orf50	brown
C9orf16	brown
C9orf78	brown
CA1	brown
CA2	brown
CA4	brown
CALD1	brown
CALM2	brown
CANX	brown
CAPG	brown
CAPNS1	brown
CAVIN1	brown
CCDC80	brown
CCDC85B	brown
CCN1	brown
CCN2	brown
CCS	brown
CD14	brown
CD151	brown
CD164	brown
CD24	brown
CD320	brown
CD81	brown
CD9	brown
CDC42EP5	brown
CEACAM7	brown
CEBPZOS	brown
CFD	brown
CHCHD10	brown
CHMP1A	brown
CHMP2A	brown
CIB1	brown
CIRBP	brown
CKB	brown
CLDN5	brown
CLIC1	brown
CLTB	brown

CLUH	brown
CNN1	brown
COL14A1	brown
COL1A1 ENSG00000108821	brown
COL1A2	brown
COL3A1	brown
COL4A1	brown
COL4A2	brown
COL5A1	brown
COL6A1	brown
COL6A2	brown
COL6A3	brown
COMTD1	brown
COPE	brown
COX4I1	brown
COX5B	brown
COX6B1	brown
CRIP2	brown
CSDE1	brown
CSRP1	brown
CST3	brown
CTNNA1	brown
CTNNB1	brown
CTSD	brown
CUTA	brown
CXCL12	brown
CYBA	brown
CYBRD1	brown
DCN	brown
DCXR	brown
DDX17	brown
DDX3X ENSG00000215301	brown
DDX5	brown
DES	brown
DPP7	brown
DUSP1	brown
DYNC2I2	brown
EDF1	brown
EFHD2	brown
EGFL7 ENSG00000172889	brown
EGR1	brown
EHBP1L1	brown
EIF3B	brown

EIF3K	brown
EIF4A2	brown
EIF4G2	brown
EIF5A	brown
EIF5B ENSG00000158417	brown
ELOB	brown
EMILIN1	brown
EMP1	brown
ENO1 ENSG00000074800	brown
EPAS1	brown
EPN1	brown
ERAP2	brown
ETHE1	brown
ETS1	brown
EZR	brown
F13A1	brown
FAAP20	brown
FAM174C	brown
FAM234A	brown
FASTK	brown
FAU	brown
FBLN2	brown
FBN1	brown
FBXL15	brown
FBXW5	brown
FHL1	brown
FIS1	brown
FKBP5	brown
FKBP8 ENSG00000105701	brown
FLNA	brown
FLNC	brown
FLOT2	brown
FN1	brown
FOS	brown
FOSL2	brown
FSTL1	brown
FTL	brown
GADD45GIP1	brown
GIPC1	brown
GLUL	brown
GMPPA	brown
GNAS	brown
GNB1	brown

GNB2	brown
GPAA1	brown
GPX1	brown
GPX2	brown
GPX3	brown
GPX4	brown
GREM1	brown
GSDMD	brown
GSN	brown
GSTP1	brown
GUK1	brown
H1-0	brown
H1-10	brown
H2AJ	brown
HBA2	brown
HM13	brown
HMGA1	brown
HNRNPH1	brown
HSP90AA1	brown
HSPB1	brown
HSPG2	brown
HTRA3	brown
IER2	brown
IFI27	brown
IFITM3	brown
IGFBP2	brown
IGFBP4	brown
IGFBP5	brown
IGFBP6	brown
IGFBP7	brown
IGHA1	brown
IGHA2	brown
IGLC7	brown
IL32	brown
INF2	brown
INTS1	brown
ISG15	brown
ITGB1	brown
ITM2B	brown
JOSD2	brown
JPT1	brown
JSRP1	brown
JUN	brown

JUNB	brown
JUND	brown
KCNK6	brown
KDELR1 ENSG00000105438	brown
KLF4	brown
KLF6	brown
LAMB2	brown
LAMTOR4	brown
LASP1	brown
LGALS1	brown
LGALS9	brown
LIMS2	brown
LMAN2	brown
LMNA	brown
LMOD1	brown
LRRC45	brown
LSM7	brown
LTBP3	brown
LUM	brown
LY6E	brown
LYPLA2	brown
LYZ	brown
MAF	brown
MAN1B1	brown
MAP2K2	brown
MAP4	brown
MAVS	brown
MCAM	brown
MCRIP1	brown
MCRIP2	brown
MED16	brown
METTL16	brown
MFAP4	brown
MICOS13	brown
MIR23AHG	brown
MMP14	brown
MMP2	brown
MPDU1	brown
MRPL12	brown
MRPL23 ENSG00000214026	brown
MRPL27	brown
MRPL28	brown
MRPL41	brown

MRPS34	brown
MS4A12	brown
MUC1	brown
MUC12	brown
MUC5B	brown
MVD	brown
MYH11	brown
MYH9	brown
MYL6	brown
MYL9	brown
MYLK	brown
NAMPT	brown
NBL1	brown
NCLN	brown
NDUFA10	brown
NDUFA3	brown
NDUFB11	brown
NDUFB7	brown
NDUFS5	brown
NDUFS6	brown
NDUFS8	brown
NDUFV1	brown
NEGR1	brown
NFIC	brown
NNMT	brown
NORAD	brown
NOSIP	brown
NOTCH3	brown
NR4A1	brown
NUCB1	brown
NUPR1	brown
OAT ENSG00000065154	brown
OAZ1	brown
OTUB1 ENSG00000167770	brown
PABPC1	brown
PALLD	brown
PARP10	brown
PCYT2	brown
PDLIM7	brown
PDXK	brown
PEPD	brown
PFDN5	brown
PFKL	brown

PGLS	brown
PIGQ	brown
PITX1	brown
PKN1	brown
PLAC8	brown
PLEC	brown
PLEKHJ1	brown
PNPLA6	brown
POLR2L	brown
PPDPF	brown
PPP1R12B	brown
PPP2R1A	brown
PPP4C	brown
PRDX5	brown
PRDX6	brown
PRKCSH	brown
PRMT1	brown
PRR13	brown
PSMB3	brown
PTPRC	brown
RABL6	brown
RAC1	brown
RALY	brown
RANGAP1	brown
RARRES2	brown
RASSF7	brown
REX1BD	brown
RGS5	brown
RHOB	brown
RHOC	brown
RN7SL1	brown
RNPEPL1	brown
ROMO1	brown
RPL13	brown
RPL13A	brown
RPL13P12	brown
RPL18	brown
RPL18A	brown
RPL21	brown
RPL27A	brown
RPL28	brown
RPL29	brown
RPL3	brown

RPL32	brown
RPL36	brown
RPL8	brown
RPL9	brown
RPLP0	brown
RPLP1	brown
RPLP2	brown
RPS11	brown
RPS15 ENSG00000115268	brown
RPS19	brown
RPS21	brown
RPS26	brown
RPS28	brown
RPS3A	brown
RPS5	brown
RPS9	brown
RPSA	brown
RRP7A	brown
S100A11	brown
S100A16	brown
S100A4	brown
S100A6	brown
S100P	brown
SAMHD1	brown
SCAND1	brown
SDF2L1	brown
SECTM1	brown
SELENOM	brown
SELENOW	brown
SEPTIN2	brown
SERF2	brown
SERPINB6	brown
SF3B5	brown
SFN	brown
SFRP2	brown
SGK1	brown
SIL1	brown
SIRT6	brown
SLC25A1	brown
SLC37A2	brown
SLC40A1	brown
SLC66A2	brown
SMIM22	brown

SMTN ENSG00000183963	brown
SNORC	brown
SNRNP70	brown
SNRPD2	brown
SOCS3	brown
SOD1	brown
SOD2	brown
SOD3	brown
SPARC	brown
SPARCL1	brown
SPTBN1	brown
SQOR	brown
STARD10	brown
STAT6	brown
STXBP2	brown
SYMPK	brown
SYNM	brown
SYNPO2	brown
TAGLN	brown
TAPBP	brown
TCF25	brown
TCIRG1 ENSG00000110719	brown
TECR	brown
THBS1	brown
THY1	brown
TIMM13	brown
TIMP1	brown
TIMP2	brown
TIMP3	brown
TLN1	brown
TMEM141	brown
TMEM160	brown
TMEM176A	brown
TMEM219	brown
TMEM238	brown
TMSB10	brown
TMUB1	brown
TNC	brown
TNFRSF14	brown
TNIP1	brown
TNS1	brown
TNXB	brown
TP53I13	brown

TPI1	brown
TPM1	brown
TPM2	brown
TPM4	brown
TPSAB1	brown
TPSB2	brown
TRIM28	brown
TRIR	brown
TSPO	brown
TSSC4	brown
TTC39B	brown
TUFM	brown
TYMP ENSG00000025708	brown
TYROBP ENSG00000011600	brown
UBA52	brown
VAMP8	brown
VPS28	brown
WDR13	brown
WIPF2	brown
XRCC6	brown
ZBTB7A	brown
ZFP36	brown
ZFP36L1	brown
ZFP36L2	brown
ZNF358	brown
ZNHIT1	brown
AEBP1	green
AQP1 ENSG00000240583	green
B2M	green
BTG2 ENSG00000159388	green
C1QC	green
C1R	green
CALR	green
CHI3L1	green
COL18A1	green
CSF3	green
CXCL1	green
CXCL8	green
HBB	green
HERPUD1	green
HLA-DRB5	green
IGHV1-18	green
IGHV1-2	green

IGHV1-24	green
IGHV1-3	green
IGHV1-69D	green
IGHV3-11 ENSG00000211941	green
IGHV3-15	green
IGHV3-21	green
IGHV3-23	green
IGHV3-30	green
IGHV3-33	green
IGHV3-48	green
IGHV3-7 ENSG00000211938	green
IGHV3-74	green
IGHV4-34	green
IGHV4-39	green
IGHV4-4	green
IGHV4-59	green
IGHV5-10-1	green
IGHV5-51	green
IGKV1-12	green
IGKV1-17	green
IGKV1-5	green
IGKV1-9	green
IGKV3-11	green
IGKV3-15	green
IGKV3-20	green
IGKV4-1	green
IGLV1-40	green
IGLV1-44	green
IGLV1-47	green
IGLV2-11	green
IGLV2-14	green
IGLV2-23	green
IGLV3-1	green
IGLV3-10	green
IGLV3-19	green
IGLV3-21	green
IGLV3-25	green
IGLV6-57	green
IL1B	green
JCHAIN	green
LUC7L3	green
MAPK8IP1P2	green
MMP1	green

MMP3	green
P4HB	green
PECAM1	green
PIM2 ENSG00000102096	green
SERPING1	green
SRGN	green
TSC22D3	green
WARS1	green
XBP1	green
RPS4Y1	grey
ACADVL	magenta
ACSL5	magenta
ANXA11	magenta
ANXA2	magenta
ATP5F1B	magenta
CALM1	magenta
CALM3	magenta
CDKN1A	magenta
CHMP4B	magenta
CNDP2	magenta
CTSZ	magenta
GRN	magenta
HDLBP	magenta
HLA-A	magenta
HLA-B	magenta
HLA-C	magenta
HLA-E	magenta
ITM2C	magenta
LGALS3	magenta
MGAT4B	magenta
MT1E	magenta
MT2A	magenta
MYL12B	magenta
MYO1D	magenta
NDRG1	magenta
NIBAN2	magenta
PDIA3	magenta
PDLIM1	magenta
PLD3	magenta
RNASE1	magenta
S100A10	magenta
SAT1	magenta
SLC25A5	magenta

TMEM176B	magenta
TSPAN3	magenta
TUBB4B	magenta
UBC	magenta
ACAP1	pink
ACTN4	pink
ARHGAP4	pink
ARHGAP45	pink
ARHGEF1	pink
CCDC88B	pink
CCL19	pink
CCL21	pink
CD19	pink
CD22	pink
CD37	pink
CD52	pink
CD7	pink
CD79A	pink
CD79B	pink
CLU	pink
CORO1A	pink
COTL1	pink
CR2	pink
CSK	pink
CXCL13	pink
CXCR4	pink
FDCSP	pink
FXYD5	pink
GPSM3	pink
GRK2	pink
HCLS1	pink
HLA-DRB1	pink
IGHD	pink
IGHM	pink
KIF1C	pink
LAPTM5	pink
LCP1	pink
LIMD2	pink
LTB	pink
LTBP4	pink
LTF	pink
MS4A1	pink
MYBL2	pink

MYO1G	pink
NAPSB	pink
PGGHG	pink
POU2AF1	pink
PTPN6	pink
RAC2	pink
RASGRP2	pink
TBC1D10C	pink
TCL1A	pink
TMC8	pink
TRBC1	pink
TRBC2	pink
UCP2	pink
ACE	purple
ALDOB	purple
ALPI	purple
APOA1 ENSG00000118137	purple
APOA4	purple
APOB	purple
APOC3	purple
CCL25	purple
CYP3A4	purple
DEFA5	purple
DEFA6	purple
DPEP1 ENSG00000015413	purple
ENPEP	purple
FABP6	purple
GSTA1	purple
MGAM	purple
MTTP	purple
OLFM4	purple
PCK2	purple
PGC ENSG00000096088	purple
PLA2G2A	purple
PRSS2	purple
RBP2	purple
REG1A	purple
REG1B	purple
REG3A	purple
REG4	purple
SERPINA1	purple
SI	purple
SLC15A1	purple

SLC6A19	purple
XPNPEP2	purple
BGN	red
C3	red
CCNI	red
CD63	red
CTSB	red
DUOX2	red
FBLN1	red
HSP90B1	red
HSPA5	red
IFI6	red
IGHG1	red
IGHG2	red
IGHG3	red
IGHG4	red
IGHGP	red
IGKC	red
IGLC2	red
IGLC3	red
IL1RN	red
LCN2	red
MT-ATP6	red
MTATP6P1	red
MT-ATP8	red
MT-CO1	red
MTCO1P12	red
MTCO1P40	red
MT-CO2	red
MTCO2P12	red
MT-CO3	red
MTCO3P12	red
MT-CYB	red
MT-ND1	red
MTND1P23	red
MT-ND2	red
MTND2P28	red
MT-ND3	red
MT-ND4	red
MT-ND4L	red
MTND4P12	red
MT-ND5	red
MT-ND6	red

MT-RNR1	red
MT-RNR2	red
MTRNR2L12	red
MT-TP	red
MZB1	red
PDIA4 ENSG00000155660	red
PI3	red
PLVAP	red
PSAP	red
QSOX1	red
RPN1 ENSG00000163902	red
RRBP1	red
S100A8	red
S100A9	red
SEC61A1	red
SERPINE1 ENSG00000106366	red
SSR4	red
VWF ENSG00000110799	red
ACTB	yellow
ACTG1	yellow
ANP32B	yellow
ATF4	yellow
ATP5MC2	yellow
CAPZB	yellow
CD74	yellow
CFL1	yellow
COX7C	yellow
EEF1B2	yellow
EIF1	yellow
HLA-DPB1	yellow
HMGN2	yellow
IFITM2	yellow
ISG20	yellow
LITAF	yellow
LPP	yellow
MARCKSL1	yellow
NACA	yellow
NAP1L1	yellow
NOP53	yellow
PFN1	yellow
PKM	yellow
PPIA	yellow
PTMS ENSG00000159335	yellow

RACK1	yellow
RPL10	yellow
RPL10A	yellow
RPL11	yellow
RPL12	yellow
RPL14	yellow
RPL15	yellow
RPL19	yellow
RPL23	yellow
RPL24	yellow
RPL27	yellow
RPL31	yellow
RPL34	yellow
RPL35	yellow
RPL35A	yellow
RPL36AL ENSG00000165502	yellow
RPL37 ENSG00000145592	yellow
RPL37A	yellow
RPL38	yellow
RPL39	yellow
RPL4	yellow
RPL41	yellow
RPL5	yellow
RPL6	yellow
RPL7	yellow
RPL7A	yellow
RPS12	yellow
RPS13	yellow
RPS14	yellow
RPS16	yellow
RPS18	yellow
RPS2	yellow
RPS20	yellow
RPS24	yellow
RPS25	yellow
RPS27	yellow
RPS27A	yellow
RPS29	yellow
RPS3	yellow
RPS4X	yellow
RPS6	yellow
RPS7	yellow
RPS8	yellow

SEPTIN9	yellow
SH3BGRL3	yellow
SNHG5	yellow
TAGLN2	yellow
TMSB4X	yellow
TPT1	yellow
TUBB	yellow
