# Supplementary material for: Expression of immune-related genes and possible regulatory mechanisms in ulcerative colitis
Source: Front Mol Biosci. 2026 Mar 5;13:1621643. doi: 10.3389/fmolb.2026.1621643 (PMC12999447; doi:10.3389/fmolb.2026.1621643)
Supplement: Supplementary file 2 [file Table4.pdf]

Supplementary Table 4 Reference marker gene list used for manual cell type annotation

Cell type	gene
Epithelial_cells	SMIM22
Epithelial_cells	C19orf33
Epithelial_cells	KRT18
Epithelial_cells	CLDN7
Epithelial_cells	ELF3
Epithelial_cells	S100A14
Epithelial_cells	EPCAM
Epithelial_cells	TMEM54
Epithelial_cells	CLDN3
Epithelial_cells	C10orf99
Epithelial_cells	SFN
Epithelial_cells	FAM3D
Epithelial_cells	SELENBP1
Epithelial_cells	AMN
Epithelial_cells	TSPAN8
Epithelial_cells	MISP
Epithelial_cells	CLDN4
Epithelial_cells	PRSS3
Epithelial_cells	TSPAN1
Epithelial_cells	CA2
Epithelial_cells	CDC42EP5
Epithelial_cells	KRT19
Epithelial_cells	IFI27
Epithelial_cells	LGALS4
Epithelial_cells	C15orf48
Epithelial_cells	PIGR
Epithelial_cells	S100A16
Epithelial_cells	LGALS3BP
Epithelial_cells	TPM1
Epithelial_cells	FXYD3
Epithelial_cells	CKB
Epithelial_cells	CST3
Epithelial_cells	KRT8
Epithelial_cells	HES1
Epithelial_cells	PHLDA2
Epithelial_cells	KLF4
Epithelial_cells	IER3
Epithelial_cells	CD24
Epithelial_cells	LGALS3

Epithelial_cells	TMEM141
Epithelial_cells	AGR2
Epithelial_cells	RRBP1
Epithelial_cells	CD9
Epithelial_cells	FABP1
Epithelial_cells	PHGR1
Epithelial_cells	CHCHD10
Epithelial_cells	NDUFB7
Epithelial_cells	ITM2C
Epithelial_cells	TXN
Epithelial_cells	ATP5I
Epithelial_cells	UQCRQ
Epithelial_cells	COX5B
Epithelial_cells	S100A10
Epithelial_cells	S100A6
Epithelial_cells	ATP5G3
Epithelial_cells	BTG1
Epithelial_cells	RPL4
Epithelial_cells	PABPC1
Epithelial_cells	SRSF2
Epithelial_cells	NPM1
Epithelial_cells	CCNI
Epithelial_cells	PPP1R18
Epithelial_cells	ANXA6
Epithelial_cells	RAB8B
Epithelial_cells	EIF3E
Epithelial_cells	EMB
Epithelial_cells	TBC1D10C
Epithelial_cells	ELOVL5
Epithelial_cells	ZEB2
Epithelial_cells	P2RY10
Epithelial_cells	RASSF5
Epithelial_cells	PRKCH
Epithelial_cells	DOCK8
Epithelial_cells	LPXN
Epithelial_cells	LBH
Epithelial_cells	IL2RB
Epithelial_cells	PRMT9
Epithelial_cells	ITGA4
Epithelial_cells	SLA2
Epithelial_cells	PTPN7
Epithelial_cells	AMICA1
Epithelial_cells	PNRC1
Epithelial_cells	DOK2

Epithelial_cells	CBLB
Epithelial_cells	LINC00152
Epithelial_cells	BST2
Epithelial_cells	SERPINB9
Epithelial_cells	TNFRSF18
Epithelial_cells	ITM2A
Epithelial_cells	PDE4B
Epithelial_cells	ITGA1
Epithelial_cells	CD8B
Epithelial_cells	MAP3K8
Epithelial_cells	RP11-138A9.2
Epithelial_cells	GZMM
Epithelial_cells	ARID5B
Epithelial_cells	ARHGAP9
Epithelial_cells	RGS10
Epithelial_cells	CTSW
Epithelial_cells	TRAT1
Epithelial_cells	CLDND1
Epithelial_cells	TGFB1
Epithelial_cells	ELF1
Epithelial_cells	UCP2
Epithelial_cells	PCED1B-AS1
Epithelial_cells	HCLS1
Epithelial_cells	LY6E
Epithelial_cells	MIR142
Epithelial_cells	TUBA1A
Epithelial_cells	LGALS1
Epithelial_cells	REL
Epithelial_cells	EVI2A
Epithelial_cells	IFI16
Epithelial_cells	STK17B
Epithelial_cells	TNFRSF1B
Epithelial_cells	FMNL1
Epithelial_cells	TNFAIP3
Epithelial_cells	AC016831.7
Epithelial_cells	SLA
Epithelial_cells	COTL1
Epithelial_cells	MSN
Epithelial_cells	FNBP1
Epithelial_cells	ENTPD1
Epithelial_cells	IDS
Epithelial_cells	SLC2A3
Epithelial_cells	EMP3
Epithelial_cells	SPOCK2

Epithelial_cells	SH2D2A
Epithelial_cells	CD247
Epithelial_cells	FYB
Epithelial_cells	LEPROTL1
Epithelial_cells	TMEM66
Epithelial_cells	IFITM2
Epithelial_cells	NR3C1
Epithelial_cells	KLRD1
Epithelial_cells	LINC-PINT
Epithelial_cells	DUSP2
Epithelial_cells	LCK
Epithelial_cells	TIGIT
Epithelial_cells	LDLRAD4
Epithelial_cells	YPEL5
Epithelial_cells	HLA-DQA1
Epithelial_cells	GPR183
Epithelial_cells	NAP1L1
Epithelial_cells	ARL4C
Epithelial_cells	NR4A2
Epithelial_cells	LCP1
Epithelial_cells	PTPN22
Epithelial_cells	HLA-DPA1
Epithelial_cells	IL7R
Epithelial_cells	CD3G
Epithelial_cells	6-Sep
Epithelial_cells	CD44
Epithelial_cells	GLIPR1
Epithelial_cells	CNOT6L
Epithelial_cells	GNG2
Epithelial_cells	CCL4
Epithelial_cells	CD8A
Epithelial_cells	EVI2B
Epithelial_cells	RUNX3
Epithelial_cells	GPSM3
Epithelial_cells	DUSP4
Epithelial_cells	GZMB
Epithelial_cells	HLA-DPB1
Epithelial_cells	GYPC
Epithelial_cells	RORA
Epithelial_cells	CD53
Epithelial_cells	TRBV28
Epithelial_cells	LIMD2
Epithelial_cells	GMFG
Epithelial_cells	CD2

Epithelial_cells	CD96
Epithelial_cells	ALOX5AP
Epithelial_cells	ACAP1
Epithelial_cells	GPR65
Epithelial_cells	RHOH
Epithelial_cells	RGCC
Epithelial_cells	FXYD5
Epithelial_cells	WIPF1
Epithelial_cells	RP11-138A9.1
Epithelial_cells	HOPX
Epithelial_cells	STK4
Epithelial_cells	SYTL3
Epithelial_cells	CLEC2B
Epithelial_cells	RAC2
Epithelial_cells	GNLY
Epithelial_cells	AC092580.4
Epithelial_cells	LTB
Epithelial_cells	CST7
Epithelial_cells	FYN
Epithelial_cells	CD69
Epithelial_cells	ZNF331
Epithelial_cells	LSP1
Epithelial_cells	CD48
Epithelial_cells	TSC22D3
Epithelial_cells	EVL
Epithelial_cells	S100A4
Epithelial_cells	CD3E
Epithelial_cells	CLEC2D
Epithelial_cells	TRAC
Epithelial_cells	CYTIP
Epithelial_cells	NKG7
Epithelial_cells	SAMSN1
Epithelial_cells	TRBC2
Epithelial_cells	CD3D
Epithelial_cells	GZMA
Epithelial_cells	CORO1A
Epithelial_cells	HCST
Epithelial_cells	CD37
Epithelial_cells	VIM
Epithelial_cells	KLRB1
Epithelial_cells	ARHGDIB
Epithelial_cells	PTPRC
Epithelial_cells	CD7
Epithelial_cells	LAPTM5

Epithelial_cells	RGS1
Epithelial_cells	CXCR4
Epithelial_cells	CREM
Epithelial_cells	CD52
Epithelial_cells	CCL5
Epithelial_cells	SRGN
Epithelial_cells	SLC44A4
Epithelial_cells	RILPL2
Epithelial_cells	MT1G
Epithelial_cells	AKNA
Epithelial_cells	TCEB2
Epithelial_cells	MT1E
Epithelial_cells	RPSAP58
Epithelial_cells	MUC13
Epithelial_cells	AGR3
Epithelial_cells	SPINT2
Epithelial_cells	PCK1
Epithelial_cells	CD151
Epithelial_cells	CEACAM5
Epithelial_cells	ATPIF1
Epithelial_cells	PRNP
Epithelial_cells	GPX2
Epithelial_cells	BIRC3
Epithelial_cells	STARD10
Epithelial_cells	CIRBP
Epithelial_cells	ANKRD28
Epithelial_cells	ANXA2
Epithelial_cells	ADIRF
Epithelial_cells	ARL14
Epithelial_cells	NDUFA1
Epithelial_cells	ID1
Epithelial_cells	CD83
Epithelial_cells	CAMK2N1
Epithelial_cells	SNRPN
Epithelial_cells	RGS2
Epithelial_cells	RND3
Epithelial_cells	AGPAT2
Epithelial_cells	ETS1
Epithelial_cells	ROMO1
Epithelial_cells	TST
Epithelial_cells	ATF3
Epithelial_cells	CISD3
Epithelial_cells	DSP
Epithelial_cells	CYC1

Epithelial_cells	UQCRC1
Epithelial_cells	KRT20
Epithelial_cells	RHOB
Epithelial_cells	CES2
Epithelial_cells	ST14
Epithelial_cells	SUMO2
Epithelial_cells	HSPA1B
Epithelial_cells	CDHR5
Epithelial_cells	SMAP2
Epithelial_cells	CD74
Epithelial_cells	MUC12
Epithelial_cells	ETHE1
Epithelial_cells	1-Sep
Epithelial_cells	PIK3R1
Epithelial_cells	MGST1
Epithelial_cells	PPP2R5C
Epithelial_cells	CYSTM1
Epithelial_cells	SERINC2
Epithelial_cells	SPINK1
Epithelial_cells	PKP3
Epithelial_cells	GPA33
Epithelial_cells	BLVRB
Epithelial_cells	TFF3
Epithelial_cells	EGR1
Epithelial_cells	H2AFJ
Epithelial_cells	ARID5A
Epithelial_cells	SDCBP2
Epithelial_cells	MARCKS
Epithelial_cells	COX5A
Epithelial_cells	ANXA1
Epithelial_cells	HSD11B2
Epithelial_cells	VSIG2
Epithelial_cells	ATP1B1
Epithelial_cells	MPST
Epithelial_cells	KLF5
Epithelial_cells	BSG
Epithelial_cells	HLA-DQB1
Epithelial_cells	CDX1
Epithelial_cells	TSPO
Epithelial_cells	SPINT1
Epithelial_cells	CASP4
Epithelial_cells	SRI
Epithelial_cells	PRAP1
Epithelial_cells	PDE4D

Epithelial_cells	FCGRT
Epithelial_cells	GNAI2
Epithelial_cells	ACTN4
Epithelial_cells	ACADVL
Epithelial_cells	AURKAIP1
Epithelial_cells	TSC22D1
Epithelial_cells	CA1
Epithelial_cells	TXNDC17
Epithelial_cells	ALKBH7
Epithelial_cells	SPRY1
Epithelial_cells	AREG
Epithelial_cells	AOC1
Epithelial_cells	FKBP2
Epithelial_cells	PNISR
Epithelial_cells	JUP
Epithelial_cells	HSPB1
Epithelial_cells	GIPC1
Epithelial_cells	CA12
Epithelial_cells	PDLIM1
Epithelial_cells	ETFB
Epithelial_cells	PRSS8
Epithelial_cells	NDUFS6
Epithelial_cells	COMTD1
Epithelial_cells	PDE3B
Epithelial_cells	TMEM176B
Epithelial_cells	MYH14
Epithelial_cells	RSL24D1
Epithelial_cells	STRA13
Epithelial_cells	TMEM176A
Epithelial_cells	CDH17
Epithelial_cells	ATP5J
Epithelial_cells	RN7SK
Epithelial_cells	SLC22A18
Epithelial_cells	RBM47
Epithelial_cells	DDX24
Epithelial_cells	LIMS1
Epithelial_cells	PLA2G2A
Epithelial_cells	NUPR1
Epithelial_cells	ARID4B
Epithelial_cells	CELF2
Epithelial_cells	DSTN
Epithelial_cells	RARRES3
Epithelial_cells	LLGL2
Epithelial_cells	MVP

Epithelial_cells	ASL
Epithelial_cells	NDUFB3
Epithelial_cells	TERF2IP
Epithelial_cells	RP11-357H14.17
Epithelial_cells	PKIB
Epithelial_cells	PAIP2
Epithelial_cells	LMO7
Epithelial_cells	MAL2
Epithelial_cells	GUCA2A
Epithelial_cells	DSC2
Epithelial_cells	PPP1R2
Epithelial_cells	EPS8L3
Epithelial_cells	ACADS
Epithelial_cells	KMT2E
Epithelial_cells	MRPL12
Epithelial_cells	LDLR
Epithelial_cells	IL2RG
Epithelial_cells	DSG2
Epithelial_cells	GOLM1
Epithelial_cells	RAB25
Epithelial_cells	OSBPL8
Epithelial_cells	HMGCS2
Epithelial_cells	CYP3A5
Epithelial_cells	WFDC2
Epithelial_cells	CCDC64B
Epithelial_cells	STAP2
Epithelial_cells	CDH1
Epithelial_cells	CYLD
Epithelial_cells	PRAC1
Epithelial_cells	MAPK3
Epithelial_cells	LIPH
Epithelial_cells	PFKFB3
Epithelial_cells	GPRC5A
Epithelial_cells	RASSF7
Epithelial_cells	GRN
Epithelial_cells	TMPRSS2
Epithelial_cells	CDC42SE2
Epithelial_cells	ATP5G1
Epithelial_cells	MYCBP2
Epithelial_cells	CLDN23
Epithelial_cells	ABCC3
Epithelial_cells	S100P
Epithelial_cells	ALDH2
Epithelial_cells	PDE4C

Epithelial_cells	SPATS2L
Epithelial_cells	LAMB3
Epithelial_cells	ATP8B1
Epithelial_cells	CLTB
Epithelial_cells	LIMA1
Epithelial_cells	NDUFB10
Epithelial_cells	GABARAPL1
Epithelial_cells	MRPL41
Epithelial_cells	SLC26A3
Epithelial_cells	FAM49B
Epithelial_cells	ASS1
Epithelial_cells	TSPYL2
Epithelial_cells	PNKD
Epithelial_cells	NAPRT1
Epithelial_cells	PHF20
Epithelial_cells	TIMM13
Epithelial_cells	MALL
Epithelial_cells	UGDH
Epithelial_cells	LSR
Epithelial_cells	MT-ND4L
Epithelial_cells	TCEA3
Epithelial_cells	MUC1
Epithelial_cells	TMSB4XP4
Epithelial_cells	VIL1
Epithelial_cells	PPP1R1B
Epithelial_cells	FAM162A
Epithelial_cells	PLS1
Epithelial_cells	FCGBP
Epithelial_cells	RPS4Y1
Epithelial_cells	TSPAN3
Epithelial_cells	SLC26A2
Epithelial_cells	C19orf70
Epithelial_cells	ABHD11
Epithelial_cells	NDUFS7
Epithelial_cells	HIGD1A
Epithelial_cells	ISOC2
Epithelial_cells	APP
Epithelial_cells	CCL15
Epithelial_cells	JAK1
Epithelial_cells	CD99
Epithelial_cells	FAM46A
Epithelial_cells	MAOA
Epithelial_cells	SOX4
Epithelial_cells	FAM195A

Epithelial_cells	FHL2
Epithelial_cells	7-Sep
Epithelial_cells	MTATP6P1
Epithelial_cells	C6orf48
Epithelial_cells	EPS8
Epithelial_cells	ETS2
Epithelial_cells	TMEM243
Epithelial_cells	C11orf58
Epithelial_cells	CTNND1
Epithelial_cells	DHX36
Epithelial_cells	NDUFB1
Epithelial_cells	FLNB
Epithelial_cells	TCF7L2
Epithelial_cells	MGLL
Epithelial_cells	TRPM4
Epithelial_cells	SEPP1
Epithelial_cells	PPP1R14D
Epithelial_cells	DST
Epithelial_cells	ICAM3
Epithelial_cells	FABP5
Epithelial_cells	NET1
Epithelial_cells	C2orf82
Epithelial_cells	PBXIP1
Epithelial_cells	SLC39A5
Epithelial_cells	HBEGF
Epithelial_cells	MUC5B
Epithelial_cells	RNASE1
Epithelial_cells	INF2
Epithelial_cells	SELT
Epithelial_cells	MGST2
Epithelial_cells	CEACAM1
Epithelial_cells	CHP2
Epithelial_cells	CHMP2A
Epithelial_cells	SULT1A1
Epithelial_cells	CKLF
Epithelial_cells	GGT6
Epithelial_cells	SNHG16
Epithelial_cells	CEACAM7
Epithelial_cells	SH3BGRL
Epithelial_cells	GSN
Epithelial_cells	CKMT1B
Epithelial_cells	ITGA6
Epithelial_cells	CDX2
Epithelial_cells	HLA-DMA

Epithelial_cells	MXD1
Epithelial_cells	PHF1
Epithelial_cells	USH1C
Epithelial_cells	CXADR
Epithelial_cells	CTNNA1
Epithelial_cells	EDN1
Epithelial_cells	HNF4A
Epithelial_cells	MUC4
Epithelial_cells	RP11-467L13.7
Epithelial_cells	UGT2B17
Epithelial_cells	FAM84A
Epithelial_cells	SLPI
Epithelial_cells	CTTN
Epithelial_cells	TUBA1C
Epithelial_cells	PFKL
Epithelial_cells	LAD1
Epithelial_cells	TJP3
Epithelial_cells	FBLIM1
Epithelial_cells	PPAP2C
Epithelial_cells	UGP2
Epithelial_cells	CRB3
Epithelial_cells	TRIB1
Epithelial_cells	ECHS1
Epithelial_cells	SQRDL
Epithelial_cells	ODF2L
Epithelial_cells	LINC01133
Epithelial_cells	SSFA2
Epithelial_cells	MGST3
Epithelial_cells	TMX4
Epithelial_cells	EFNA1
Epithelial_cells	TFF1
Epithelial_cells	SPTBN1
Epithelial_cells	GOLIM4
Epithelial_cells	GDF15
Epithelial_cells	RP11-532F12.5
Epithelial_cells	EMP2
Epithelial_cells	FABP2
Epithelial_cells	SLC4A4
Epithelial_cells	HNRNPA1P48
Epithelial_cells	GPT
Epithelial_cells	EMP1
Epithelial_cells	CMBL
Epithelial_cells	ANXA4
Epithelial_cells	SLIRP

Epithelial_cells	LGALS2
Epithelial_cells	MT1M
Epithelial_cells	STK17A
Epithelial_cells	MUC3A
Epithelial_cells	FBXW5
Epithelial_cells	VAMP2
Epithelial_cells	FUT3
Epithelial_cells	NDUFC1
Epithelial_cells	MS4A12
Epithelial_cells	PHB
Epithelial_cells	CHMP4B
Epithelial_cells	COA3
Epithelial_cells	TP53I3
Epithelial_cells	GNA11
Epithelial_cells	GMDS
Epithelial_cells	ROCK1
Epithelial_cells	IGFBP2
Epithelial_cells	NBL1
Epithelial_cells	FGFR1OP2
Epithelial_cells	NR2F6
Epithelial_cells	DHRS11
Epithelial_cells	NEDD4L
Epithelial_cells	GUCA2B
Epithelial_cells	PVRL2
Epithelial_cells	EID1
Epithelial_cells	RARRES2
Epithelial_cells	C6orf222
Epithelial_cells	TRIM31
Epithelial_cells	WBP5
Epithelial_cells	LPIN1
Epithelial_cells	ADM
Epithelial_cells	S100A13
Epithelial_cells	EIF3L
Epithelial_cells	QTRT1
Epithelial_cells	C8orf4
Epithelial_cells	PLA2G10
Epithelial_cells	RBMX
Epithelial_cells	LINC00035
Epithelial_cells	AP1M2
Epithelial_cells	RPL7P9
Epithelial_cells	ECI1
Epithelial_cells	CFDP1
Epithelial_cells	DGAT1
Epithelial_cells	TANK

Epithelial_cells	PTPRF
Epithelial_cells	MYO1C
Epithelial_cells	ERBB3
Epithelial_cells	EML4
Epithelial_cells	AHCYL2
Epithelial_cells	MYO1A
Epithelial_cells	CDA
Epithelial_cells	TMEM123
Epithelial_cells	SULT1B1
Epithelial_cells	CDKN2B-AS1
Epithelial_cells	PLEC
Epithelial_cells	TMPRSS4
Epithelial_cells	TMEM45B
Epithelial_cells	DDAH2
Epithelial_cells	GCNT3
Epithelial_cells	TINAGL1
Epithelial_cells	ABHD17C
Epithelial_cells	TIMM8B
Epithelial_cells	ACAA1
Epithelial_cells	CHD2
Epithelial_cells	PLAUR
Epithelial_cells	MDK
Epithelial_cells	SUCLG1
Epithelial_cells	FDPS
Epithelial_cells	LAMA3
Epithelial_cells	CD177
Epithelial_cells	SLC44A1
Epithelial_cells	ST6GALNAC1
Epithelial_cells	SLC25A1
Epithelial_cells	ACAA2
Epithelial_cells	FAM46C
Epithelial_cells	TRIM15
Epithelial_cells	AK1
Epithelial_cells	LGALS9
Epithelial_cells	SERPINB6
Epithelial_cells	C11orf83
Epithelial_cells	EFNB2
Epithelial_cells	SHROOM3
Epithelial_cells	CKMT1A
Epithelial_cells	VILL
Epithelial_cells	CAPN5
Epithelial_cells	BLOC1S2
Epithelial_cells	LITAF
Epithelial_cells	AKR1B10

Epithelial_cells	PPIC
Epithelial_cells	PLXNB2
Epithelial_cells	SLC51B
Epithelial_cells	HADH
Epithelial_cells	GABPB1
Epithelial_cells	TMC4
Epithelial_cells	PARM1
Epithelial_cells	ARHGEF1
Epithelial_cells	MGAT4B
Epithelial_cells	PDCL3
Epithelial_cells	PDLIM5
Epithelial_cells	ERRFI1
Epithelial_cells	TDP2
Epithelial_cells	CHP1
Epithelial_cells	SNHG18
Epithelial_cells	CEACAM6
Epithelial_cells	EPN1
Epithelial_cells	KIF1C
Epithelial_cells	PPP1R16A
Epithelial_cells	PLCD3
Epithelial_cells	GTF3A
Epithelial_cells	SMCHD1
Epithelial_cells	TAX1BP3
Epithelial_cells	CBLC
Epithelial_cells	DHRS9
Epithelial_cells	GNG12
Epithelial_cells	AKR7A3
Epithelial_cells	PRR15
Epithelial_cells	RABL6
Epithelial_cells	CTSA
Epithelial_cells	ADAM15
Epithelial_cells	CCNH
Epithelial_cells	H1F0
Epithelial_cells	HDHD3
Epithelial_cells	EPS8L2
Epithelial_cells	EHF
Epithelial_cells	MARVELD3
Epithelial_cells	CHD1
Epithelial_cells	RAB13
Epithelial_cells	B3GNT5
Epithelial_cells	SCNN1A
Epithelial_cells	PAWR
Epithelial_cells	HLA-DRA
Epithelial_cells	HOXB7

Epithelial_cells	ERN2
Epithelial_cells	NPDC1
Epithelial_cells	SIRT6
Epithelial_cells	SHD
Epithelial_cells	CXCL3
Epithelial_cells	BCAS1
Epithelial_cells	C2orf88
Epithelial_cells	GALE
Epithelial_cells	TMC5
Epithelial_cells	POF1B
Epithelial_cells	ESPN
Epithelial_cells	ARHGEF16
Epithelial_cells	PLAC8
Epithelial_cells	MYO15B
Epithelial_cells	SATB2
Epithelial_cells	ACSS2
Epithelial_cells	WASL
Epithelial_cells	USP53
Epithelial_cells	BMP2
Epithelial_cells	PRR15L
Epithelial_cells	HOOK2
Epithelial_cells	PADI2
Epithelial_cells	RHOG
Epithelial_cells	CA4
Epithelial_cells	ZG16
Epithelial_cells	SH3BGRL2
Epithelial_cells	KRTCAP3
Epithelial_cells	ATP10B
Epithelial_cells	NQO1
Epithelial_cells	PKP2
Epithelial_cells	HK2
Epithelial_cells	HRCT1
Epithelial_cells	PXMP2
Epithelial_cells	TSPAN15
Epithelial_cells	CGN
Epithelial_cells	TICAM1
Epithelial_cells	MTMR11
Epithelial_cells	WHSC1L1
Epithelial_cells	SDC4
Epithelial_cells	FAM118A
Epithelial_cells	FAM129B
Epithelial_cells	MT1X
Epithelial_cells	LYST
Epithelial_cells	BAIAP2L1

Epithelial_cells	NDRG2
Epithelial_cells	MYO1D
Epithelial_cells	HEPH
Epithelial_cells	EPHA2
Epithelial_cells	TJP1
Epithelial_cells	C1orf21
Epithelial_cells	TM4SF1
Epithelial_cells	ANPEP
Epithelial_cells	TPRN
Epithelial_cells	ENTPD5
Epithelial_cells	ACVRL1
Epithelial_cells	CD320
Epithelial_cells	CDKN1B
Epithelial_cells	PRDM2
Epithelial_cells	PTK6
Epithelial_cells	TNFRSF1A
Epithelial_cells	G3BP2
Epithelial_cells	HLA-DRB1
Epithelial_cells	SMPDL3A
Epithelial_cells	FAM213A
Epithelial_cells	ANKRD9
Epithelial_cells	DDAH1
Epithelial_cells	F2RL1
Epithelial_cells	MAP7
Epithelial_cells	ENTPD8
Epithelial_cells	SLC22A18AS
Epithelial_cells	ST3GAL4
Epithelial_cells	MYO7B
Epithelial_cells	SCNN1B
Epithelial_cells	ADH1C
Epithelial_cells	RETSAT
Epithelial_cells	PCNP
Epithelial_cells	AKR1C3
Epithelial_cells	ZG16B
Epithelial_cells	CDKN2B
Epithelial_cells	EPS8L1
Epithelial_cells	PIGZ
Epithelial_cells	KCNK1
Epithelial_cells	LYPD8
Epithelial_cells	CAPN8
Epithelial_cells	UPP1
Epithelial_cells	SLC6A8
Epithelial_cells	SEMA3B
Epithelial_cells	CCNDBP1

Epithelial_cells	OSTF1
Epithelial_cells	ISCA1
Epithelial_cells	AQP8
Epithelial_cells	VPS37B
Epithelial_cells	ITGB4
Epithelial_cells	RP11-670E13.6
Epithelial_cells	RBPJ
Epithelial_cells	RNF19A
Epithelial_cells	BCAS2
Epithelial_cells	NUDT8
Epithelial_cells	PTGER4
Epithelial_cells	CLCA4
Epithelial_cells	KLK1
Epithelial_cells	CXCL2
Epithelial_cells	PDZK1IP1
Epithelial_cells	MUC2
Epithelial_cells	PI3
Epithelial_cells	SPINK4
T_cells	CCL5
T_cells	SRGN
T_cells	CD7
T_cells	KLRB1
T_cells	GZMA
T_cells	CD3D
T_cells	NKG7
T_cells	PTPRC
T_cells	CREM
T_cells	CD52
T_cells	RGS1
T_cells	TRAC
T_cells	CD3E
T_cells	HCST
T_cells	TRBC2
T_cells	ARHGDIB
T_cells	CST7
T_cells	FYN
T_cells	VIM
T_cells	SAMSN1
T_cells	GNLY
T_cells	AC092580.4
T_cells	CLEC2D
T_cells	EVL
T_cells	S100A4
T_cells	HOPX

T_cells	SYTL3
T_cells	CXCR4
T_cells	CD96
T_cells	CD2
T_cells	TRBV28
T_cells	LAPTM5
T_cells	CORO1A
T_cells	RORA
T_cells	LSP1
T_cells	GZMB
T_cells	CD8A
T_cells	CYTIP
T_cells	GPR65
T_cells	CLEC2B
T_cells	CD3G
T_cells	RGCC
T_cells	ZNF331
T_cells	IL7R
T_cells	CD48
T_cells	CCL4
T_cells	TIGIT
T_cells	TSC22D3
T_cells	WIPF1
T_cells	ALOX5AP
T_cells	KLRD1
T_cells	ACAP1
T_cells	DUSP4
T_cells	CD37
T_cells	RUNX3
T_cells	RP11-138A9.1
T_cells	LCK
T_cells	CD247
T_cells	PTPN22
T_cells	SH2D2A
T_cells	CNOT6L
T_cells	ARL4C
T_cells	LEPROTL1
T_cells	RAC2
T_cells	FYB
T_cells	CD69
T_cells	GMFG
T_cells	STK4
T_cells	SLA
T_cells	GNG2

T_cells	GLIPR1
T_cells	FXYD5
T_cells	GYPC
T_cells	TNFAIP3
T_cells	DUSP2
T_cells	CD53
T_cells	SPOCK2
T_cells	LTB
T_cells	TNFRSF1B
T_cells	ENTPD1
T_cells	TRAT1
T_cells	6-Sep
T_cells	PIK3R1
T_cells	GZMM
T_cells	LCP1
T_cells	ITGA1
T_cells	CTSW
T_cells	LINC-PINT
T_cells	CD8B
T_cells	NR3C1
T_cells	LIMD2
T_cells	EVI2A
T_cells	LDLRAD4
T_cells	RHOH
T_cells	XCL2
T_cells	KLRC2
T_cells	CLDND1
T_cells	IFITM2
T_cells	TMEM66
T_cells	CD44
T_cells	YPEL5
T_cells	COTL1
T_cells	EVI2B
T_cells	SLA2
T_cells	STK17B
T_cells	ITM2A
T_cells	FNBP1
T_cells	AC016831.7
T_cells	MSN
T_cells	PRKCH
T_cells	CBLB
T_cells	NAP1L1
T_cells	GPSM3
T_cells	IL2RB

T_cells	ARHGAP9
T_cells	TNFRSF18
T_cells	DOK2
T_cells	AMICA1
T_cells	TRGC2
T_cells	IFI16
T_cells	GPR171
T_cells	PCED1B-AS1
T_cells	LY6E
T_cells	PDE4D
T_cells	SPRY1
T_cells	TMIGD2.1
T_cells	LINC00152
T_cells	LAG3
T_cells	RGS10
T_cells	GIMAP7
T_cells	PRMT9
T_cells	LBH
T_cells	ITGA4
T_cells	HCLS1
T_cells	STAT4
T_cells	PRF1
T_cells	FMNL1
T_cells	TRGC1
T_cells	ETS1
T_cells	TGFB1
T_cells	PTPN7
T_cells	BATF
T_cells	SKAP1
T_cells	CXCR6
T_cells	MIR142
T_cells	PYHIN1
T_cells	CD6
T_cells	DOCK8
T_cells	ZEB2
T_cells	P2RY10
T_cells	PIK3IP1
T_cells	CD160
T_cells	SLC2A3
T_cells	PNRC1
T_cells	CAMK4
T_cells	RAB8B
T_cells	TBC1D10C
T_cells	APBB1IP

T_cells	EMB
T_cells	PDE4B
T_cells	RASSF5
T_cells	LPXN
T_cells	PPP1R18
T_cells	IKZF1
T_cells	APOBEC3G
T_cells	PABPC1
T_cells	BTG1
T_cells	S100A6
T_cells	COX5B
T_cells	PHGR1
T_cells	CHCHD10
T_cells	AGR2
T_cells	LGALS3
T_cells	KRT8
T_cells	FXYD3
T_cells	LGALS4
T_cells	CLDN4
T_cells	PIGR
T_cells	KRT19
T_cells	C15orf48
T_cells	TSPAN8
T_cells	CLDN3
T_cells	CST3
T_cells	EPCAM
T_cells	CLDN7
T_cells	S100A14
T_cells	ELF3
T_cells	KRT18
T_cells	C19orf33
T_cells	SMIM22
T_cells	FAM3D
T_cells	BST2
T_cells	PDE3B
T_cells	CHST12
T_cells	NPM1
T_cells	FLNA
T_cells	PIP4K2A
T_cells	TUBA1A
T_cells	IDS
T_cells	RP11-138A9.2
T_cells	PPP2R5C
T_cells	IER3

T_cells	CD24
T_cells	TXN
T_cells	NR4A2
T_cells	AKNA
T_cells	S100A10
T_cells	HES1
T_cells	GPR183
T_cells	ANXA6
T_cells	ELF1
T_cells	ANKRD28
T_cells	C10orf99
T_cells	TMEM54
T_cells	IFI27
T_cells	SRSF2
T_cells	ANXA1
T_cells	TTC39C
T_cells	LGALS1
T_cells	NDUFB7
T_cells	SFN
T_cells	RARRES3
T_cells	ATP5I
T_cells	CDC42EP5
T_cells	REL
T_cells	LGALS3BP
T_cells	UQCRQ
T_cells	1-Sep
T_cells	SERPINB9
T_cells	CCNI
T_cells	MISP
T_cells	RRBP1
T_cells	CD9
T_cells	AMN
T_cells	TPM1
T_cells	MAP3K8
T_cells	CKB
T_cells	GABARAPL1
T_cells	SPINT2
T_cells	BIN2
T_cells	TMEM141
T_cells	SELENBP1
T_cells	EMP3
T_cells	KLF4
T_cells	ELOVL5
T_cells	S100A16

T_cells	PHLDA2
T_cells	DDX24
T_cells	TSPAN1
T_cells	CA2
T_cells	FABP1
T_cells	ATP5G3
T_cells	PRSS3
T_cells	JUN
T_cells	SLC44A4
T_cells	LIMS1
T_cells	ITM2C
T_cells	SLC7A5
T_cells	MARCKS
T_cells	SNRPN
T_cells	EIF3E
T_cells	MT1G
T_cells	MUC13
T_cells	GPX2
T_cells	AGR3
T_cells	CELF2
T_cells	MT1E
T_cells	PCK1
T_cells	CEACAM5
T_cells	ROMO1
T_cells	ADIRF
T_cells	ANXA2
T_cells	STARD10
T_cells	PDLIM1
T_cells	HSPA1B
T_cells	TSPYL2
T_cells	IL2RG
T_cells	DSP
T_cells	TERF2IP
T_cells	RHOB
T_cells	CD99
T_cells	CYC1
T_cells	ST14
T_cells	CYSTM1
T_cells	UCP2
T_cells	CIRBP
T_cells	ARL14
T_cells	CD151
T_cells	PFKFB3
T_cells	ID1

T_cells	BIRC3
T_cells	RND3
T_cells	TST
T_cells	ARID4B
T_cells	MUC12
T_cells	KRT20
T_cells	GNAI2
T_cells	CES2
T_cells	RGS2
T_cells	SUMO2
T_cells	UQCRC1
T_cells	ATF3
T_cells	AGPAT2
T_cells	CDHR5
T_cells	SPINK1
T_cells	SRSF7
T_cells	CAMK2N1
T_cells	NDUFA1
T_cells	ATPIF1
T_cells	FCGRT
T_cells	TSPO
T_cells	BLVRB
T_cells	CISD3
T_cells	FKBP2
T_cells	CDC42SE2
T_cells	CKLF
T_cells	MGST1
T_cells	EGR1
T_cells	TFF3
T_cells	RNF125
T_cells	COX5A
T_cells	PKP3
T_cells	SERINC2
T_cells	ODF2L
T_cells	ETHE1
T_cells	TSC22D1
T_cells	SDCBP2
T_cells	AREG
T_cells	SPINT1
T_cells	GPA33
T_cells	ARID5B
T_cells	HSD11B2
T_cells	TMEM176B
T_cells	ALKBH7

T_cells	VSIG2
T_cells	CDX1
T_cells	GRN
T_cells	RBM47
T_cells	MPST
T_cells	KLF5
T_cells	ATP1B1
T_cells	AURKAIP1
T_cells	CASP4
T_cells	PRAP1
T_cells	PNISR
T_cells	PLA2G2A
T_cells	JAK1
T_cells	ATP5J
T_cells	TMEM176A
T_cells	JUP
T_cells	ACADVL
T_cells	SRI
T_cells	PPP1R2
T_cells	AOC1
T_cells	PBXIP1
T_cells	H2AFJ
T_cells	TMSB4XP4
T_cells	PRSS8
T_cells	FAM118A
T_cells	PAIP2
T_cells	CA1
T_cells	TXNDC17
T_cells	NUPR1
T_cells	CA12
T_cells	TNIP3
T_cells	STRA13
T_cells	LMNA
T_cells	ARID5A
T_cells	MYH14
T_cells	RSL24D1
T_cells	GIPC1
T_cells	PDCL3
T_cells	BSG
T_cells	NDUFS6
T_cells	RN7SK
T_cells	PKIB
T_cells	ETFB
T_cells	ACTN4

T_cells	HSPB1
T_cells	CYLD
T_cells	NDUFB3
T_cells	AKAP13
T_cells	ASL
T_cells	SLC22A18
T_cells	DHX36
T_cells	LAMTOR4
T_cells	COMTD1
T_cells	MAL2
T_cells	MVP
T_cells	CDH17
T_cells	ATP5G1
T_cells	SNHG16
T_cells	LMO7
T_cells	DSC2
T_cells	EPS8L3
T_cells	DSTN
T_cells	MRPL12
T_cells	7-Sep
T_cells	ALDH2
T_cells	EID1
T_cells	LLGL2
T_cells	RAB25
T_cells	RP11-357H14.17
T_cells	CCDC64B
T_cells	WFDC2
T_cells	TSPAN3
T_cells	KMT2E
T_cells	LDLR
T_cells	CDH1
T_cells	GOLM1
T_cells	ASS1
T_cells	TIMM13
T_cells	ISOC2
T_cells	STAP2
T_cells	DSG2
T_cells	ACADS
T_cells	ICAM3
T_cells	HMGCS2
T_cells	EML4
T_cells	GUCA2A
T_cells	PRAC1
T_cells	TMPRSS2

T_cells	MUC1
T_cells	LAMB3
T_cells	CYP3A5
T_cells	LIPH
T_cells	GPRC5A
T_cells	S100P
T_cells	SLC26A3
T_cells	STK17A
T_cells	MAPK3
T_cells	PTGER4
T_cells	ATP8B1
T_cells	RASSF7
T_cells	PHF1
T_cells	SPATS2L
T_cells	MALL
T_cells	ABCC3
T_cells	TMX4
T_cells	MT-ND4L
T_cells	PNKD
T_cells	NAPRT1
T_cells	PDE4C
T_cells	ABHD11
T_cells	CLDN23
T_cells	APP
T_cells	LSR
T_cells	FAM162A
T_cells	UGDH
T_cells	MBNL1
T_cells	ARHGEF1
T_cells	VIL1
T_cells	C19orf70
T_cells	CTNND1
T_cells	LYAR
T_cells	G3BP2
T_cells	HIGD1A
T_cells	FAM46A
T_cells	PHF20
T_cells	NDUFS7
T_cells	SOX4
T_cells	SLC26A2
T_cells	MRPL41
T_cells	TCEA3
T_cells	OSBPL8
T_cells	GSN

T_cells	PPP1R1B
T_cells	RPS4Y1
T_cells	PLS1
T_cells	FGFR1OP2
T_cells	INF2
T_cells	CCL15
T_cells	NET1
T_cells	MAOA
T_cells	FCGBP
T_cells	MTATP6P1
T_cells	SEPP1
T_cells	FAM195A
T_cells	LIMA1
T_cells	ETS2
T_cells	EPS8
T_cells	TRPM4
T_cells	MUC5B
T_cells	HBEGF
T_cells	FHL2
T_cells	CEACAM1
T_cells	C6orf48
T_cells	RNF19A
T_cells	RCAN3
T_cells	PHLDA1
T_cells	TCF7L2
T_cells	NDUFA3
T_cells	FLNB
T_cells	PFKL
T_cells	TANK
T_cells	RNASE1
T_cells	DST
T_cells	PPP1R14D
T_cells	SELT
T_cells	SULT1A1
T_cells	FAM49B
T_cells	SMIM14
T_cells	TRIB1
T_cells	MGLL
T_cells	C2orf82
T_cells	SLC39A5
T_cells	C11orf58
T_cells	MGST2
T_cells	CEACAM7
T_cells	GABPB1

T_cells	DDAH2
T_cells	CHP2
T_cells	LPIN1
T_cells	RP11-467L13.7
T_cells	LGALS2
T_cells	GGT6
T_cells	ITGA6
T_cells	MRPL23
T_cells	CDX2
T_cells	ROCK1
T_cells	MXD1
T_cells	ANXA4
T_cells	MUC4
T_cells	CTNNA1
T_cells	CHD2
T_cells	VAMP2
T_cells	LGALS9
T_cells	CHMP2A
T_cells	GBP2
T_cells	CKMT1B
T_cells	HNF4A
T_cells	FABP5
T_cells	SLPI
T_cells	PLAUR
T_cells	CCNH
T_cells	USH1C
T_cells	CXADR
T_cells	FAM46C
T_cells	FAM84A
T_cells	TJP3
T_cells	SQRDL
T_cells	PVRL2
T_cells	FBLIM1
T_cells	CTTN
T_cells	ECHS1
T_cells	EDN1
T_cells	GPT
T_cells	RP11-532F12.5
T_cells	UGT2B17
T_cells	TP53I3
T_cells	GOLIM4
T_cells	LAD1
T_cells	PPAP2C
T_cells	TFF1

T_cells	CCSER2
T_cells	LINC01133
T_cells	FABP2
T_cells	PIM1
T_cells	NDUFC1
T_cells	EFNA1
T_cells	CRB3
T_cells	ADM
T_cells	WBP5
T_cells	PLAC8
T_cells	COA3
T_cells	EMP2
T_cells	FUT3
T_cells	SLC4A4
T_cells	MGST3
T_cells	COX6A1P2
T_cells	GNA11
T_cells	OSTF1
T_cells	CHMP4B
T_cells	GMDS
T_cells	PHB
T_cells	MT1M
T_cells	RBPJ
T_cells	NR2F6
T_cells	SH3BGRL
T_cells	RP1-313I6.12
T_cells	MUC3A
T_cells	GDF15
T_cells	CMBL
T_cells	NEDD4L
T_cells	SSFA2
T_cells	MS4A12
T_cells	LITAF
T_cells	MYCBP2
T_cells	EMP1
T_cells	GUCA2B
T_cells	IGFBP2
T_cells	MYO1C
T_cells	QTRT1
T_cells	DGAT1
T_cells	ECI1
T_cells	SLC44A1
T_cells	DHRS11
T_cells	CTSZ

T_cells	USP36
T_cells	C6orf222
T_cells	POLD4
T_cells	C8orf4
T_cells	TRIM31
T_cells	SERPINB6
T_cells	RAB13
T_cells	AP1M2
T_cells	RARRES2
T_cells	PLA2G10
T_cells	HERPUD2
T_cells	SPTBN1
T_cells	FBXW5
T_cells	ACAA1
T_cells	C11orf83
T_cells	LINC00035
T_cells	SMCHD1
T_cells	TMEM45B
T_cells	BTN3A2
T_cells	PARM1
T_cells	PLEC
T_cells	CDKN1B
T_cells	TIMM8B
T_cells	PTPRF
T_cells	ERBB3
T_cells	CDA
T_cells	CHP1
T_cells	TINAGL1
T_cells	AHCYL2
T_cells	HNRNPA1P48
T_cells	CFDP1
T_cells	MDK
T_cells	TMPRSS4
T_cells	ISCA1
T_cells	VPS37B
T_cells	GCNT3
T_cells	FDPS
T_cells	CXCL3
T_cells	CDKN2B-AS1
T_cells	MYO1A
T_cells	CHD1
T_cells	SULT1B1
T_cells	LAMA3
T_cells	HADH

T_cells	S100A13
T_cells	TRIM15
T_cells	CD177
T_cells	SHROOM3
T_cells	WHSC1L1
T_cells	TMC4
T_cells	EPN1
T_cells	ABHD17C
T_cells	SOCS1
T_cells	B3GNT5
T_cells	EFNB2
T_cells	PPIC
T_cells	CEACAM6
T_cells	DHRS9
T_cells	CKMT1A
T_cells	CAPN5
T_cells	SLC25A1
T_cells	PLXNB2
T_cells	PAWR
T_cells	EMD
T_cells	SNHG18
T_cells	PDLIM5
T_cells	ST6GALNAC1
T_cells	SLC51B
T_cells	AKR1B10
T_cells	HOOK2
T_cells	PLCD3
T_cells	AKR7A3
T_cells	METTL9
T_cells	HK2
T_cells	VILL
T_cells	TAX1BP3
T_cells	CHIC2
T_cells	KIF1C
T_cells	SIRT6
T_cells	C2orf88
T_cells	AK1
T_cells	H1F0
T_cells	ACAA2
T_cells	MARVELD3
T_cells	GTF3A
T_cells	ERRFI1
T_cells	PPP1R16A
T_cells	MYO15B

T_cells	WASL
T_cells	HDHD3
T_cells	CTSA
T_cells	ADAM15
T_cells	MGAT4B
T_cells	ACSS2
T_cells	PRR15
T_cells	LYST
T_cells	CBLC
T_cells	SCNN1A
T_cells	EHF
T_cells	PKP2
T_cells	ERN2
T_cells	GNG12
T_cells	HOXB7
T_cells	FLJ31306
T_cells	TDP2
T_cells	CCNDBP1
T_cells	POF1B
T_cells	RABL6
T_cells	BUB3
T_cells	ESPN
T_cells	ARHGEF16
T_cells	BMP2
T_cells	TMC5
T_cells	PADI2
T_cells	BCAS1
T_cells	GALE
T_cells	SATB2
T_cells	TSPAN15
T_cells	RNF149
T_cells	SHD
T_cells	ATP10B
T_cells	KRTCAP3
T_cells	SH3BGRL2
T_cells	BCL10
T_cells	PRR15L
T_cells	FAM213A
T_cells	EPHA2
T_cells	GLUL
T_cells	TM4SF1
T_cells	TIPARP
T_cells	NDRG2
T_cells	MYO1D

T_cells	ANPEP
T_cells	HEPH
T_cells	NQO1
T_cells	NPDC1
T_cells	BAIAP2L1
T_cells	HRCT1
T_cells	TSPAN13
T_cells	FAM129B
T_cells	USP53
T_cells	CGN
T_cells	ENTPD5
T_cells	TJP1
T_cells	MTMR11
T_cells	CA4
T_cells	TICAM1
T_cells	ACVRL1
T_cells	TPRN
T_cells	SMPDL3A
T_cells	CD320
T_cells	PTK6
T_cells	ORMDL1
T_cells	CXCL2
T_cells	SLC22A18AS
T_cells	RANBP2
T_cells	IFITM3
T_cells	PLIN3
T_cells	ST3GAL4
T_cells	EPS8L1
T_cells	AKR1C3
T_cells	CDKN2B
T_cells	ADH1C
T_cells	MYO7B
T_cells	KLK1
T_cells	MT1X
T_cells	RP11-670E13.6
T_cells	ZG16
T_cells	CLCA4
T_cells	AQP8
T_cells	LCN2
T_cells	PI3
T_cells	MUC2
B_cell	IGKC
B_cell	CD79A
B_cell	HLA-DQA1

B_cell	HLA-DPB1
B_cell	MS4A1
B_cell	HLA-DPA1
B_cell	HLA-DRA
B_cell	HLA-DQB1
B_cell	CXCR4
B_cell	CD37
B_cell	CD83
B_cell	LAPTM5
B_cell	IGHM
B_cell	BANK1
B_cell	LTB
B_cell	MEF2C
B_cell	VPREB3
B_cell	LY9
B_cell	RHOH
B_cell	CD52
B_cell	CYTIP
B_cell	CORO1A
B_cell	GPR183
B_cell	CD79B
B_cell	ADAM28
B_cell	HLA-DMB
B_cell	CCR7
B_cell	ORAI2
B_cell	IGHD
B_cell	BASP1
B_cell	CD19
B_cell	CTA-250D10.23
B_cell	GNG7
B_cell	SELL
B_cell	CD22
B_cell	POU2F2
B_cell	PHACTR1
B_cell	TCL1A
B_cell	FCER2
B_cell	AC079767.4
B_cell	TMEM156
B_cell	LY86
B_cell	TCF4
B_cell	NCF1C
B_cell	NAPSB
B_cell	RASGRP2
B_cell	BTK

B_cell	PRKCB
B_cell	LIMD2
B_cell	IL16
B_cell	CD48
B_cell	FAM65B
B_cell	FMNL1
B_cell	SLC2A3
B_cell	EVI2B
B_cell	IGJ
B_cell	SESN3
B_cell	MIR142
B_cell	HLA-DMA
B_cell	GPSM3
B_cell	EMP3
B_cell	RCSD1
B_cell	CD74
B_cell	LAT2
B_cell	CD69
B_cell	CD40
B_cell	PDE4B
B_cell	ARHGDIB
B_cell	NFKBID
B_cell	IGLC2
B_cell	GYPC
B_cell	LYN
B_cell	HLA-DRB1
B_cell	STK4
B_cell	TSC22D3
B_cell	SP140
B_cell	ARID5B
B_cell	CD53
B_cell	RAC2
B_cell	RILPL2
B_cell	ZNF331
B_cell	SMAP2
B_cell	LSP1
B_cell	TAGAP
B_cell	FXYD5
B_cell	RP11-138A9.1
B_cell	BCL2A1
B_cell	PIM2
B_cell	CREM
B_cell	LGALS4
B_cell	UCP2

B_cell	RAB30
B_cell	KRT8
B_cell	KRT18
B_cell	PPM1K
B_cell	CLDN3
B_cell	ACAP1
B_cell	S100A6
B_cell	CLDN4
B_cell	WIPF1
B_cell	PTPRC
B_cell	GNB5
B_cell	PHGR1
B_cell	ANKRD44
B_cell	SRGN
B_cell	TMEM243
B_cell	6-Sep
B_cell	TGFB1
B_cell	NAP1L1
B_cell	ELF3
B_cell	CST3
B_cell	SMIM22
B_cell	C15orf48
B_cell	PIGR
B_cell	S100A10
B_cell	HCLS1
B_cell	CLDN7
B_cell	LGALS3
B_cell	C19orf33
B_cell	PRNP
B_cell	ATM
B_cell	IRF8
B_cell	CLEC2D
B_cell	TUBA1A
B_cell	KRT19
B_cell	RPSAP58
B_cell	EPCAM
B_cell	FXYD3
B_cell	LINC-PINT
B_cell	KIAA0226L
B_cell	DENND4A
B_cell	ELOVL5
B_cell	COX5B
B_cell	S100A14
B_cell	TXN

B_cell	GNG2
B_cell	RPL4
B_cell	TSPAN8
B_cell	PDE7A
B_cell	FABP1
B_cell	LPXN
B_cell	ZCCHC11
B_cell	YPEL5
B_cell	NCOA3
B_cell	TMEM66
B_cell	HLA-DRB5
B_cell	AC016831.7
B_cell	GLIPR1
B_cell	IDS
B_cell	UBE2Q2P6
B_cell	IER3
B_cell	CD9
B_cell	VIM
B_cell	FAM3D
B_cell	PNRC1
B_cell	GMFG
B_cell	MYCBP2
B_cell	PCED1B-AS1
B_cell	SERPINB9
B_cell	SPIB
B_cell	ATP5I
B_cell	RASSF5
B_cell	DOCK8
B_cell	TMEM54
B_cell	LGALS3BP
B_cell	LDLRAD4
B_cell	IGHA1
B_cell	UQCRQ
B_cell	C10orf99
B_cell	EIF3E
B_cell	CD63
B_cell	CDC42EP5
B_cell	IFI27
B_cell	TPM1
B_cell	CKB
B_cell	REL
B_cell	AMN
B_cell	SKIL
B_cell	SFN

B_cell	SNX2
B_cell	NPM1
B_cell	CLEC2B
B_cell	PHLDA2
B_cell	CD151
B_cell	CTSD
B_cell	CCNI
B_cell	RRBP1
B_cell	ST6GAL1
B_cell	CD44
B_cell	ARID5A
B_cell	AGR2
B_cell	MISP
B_cell	SELENBP1
B_cell	TCEB2
B_cell	IFI16
B_cell	ATPIF1
B_cell	ITM2C
B_cell	RGS1
B_cell	HES1
B_cell	HSPA1B
B_cell	MT1G
B_cell	MT1E
B_cell	PRSS3
B_cell	NDUFA1
B_cell	ATF3
B_cell	NR3C1
B_cell	H2AFJ
B_cell	NDUFB7
B_cell	CHCHD10
B_cell	BSG
B_cell	BIRC3
B_cell	EVL
B_cell	ACP5
B_cell	HSPB1
B_cell	BST2
B_cell	TSPAN1
B_cell	ATP5G3
B_cell	CA2
B_cell	CIRBP
B_cell	ANXA2
B_cell	GGA2
B_cell	KDM4B
B_cell	S100A16

B_cell	PRDM2
B_cell	CISD3
B_cell	SLC44A4
B_cell	LCP1
B_cell	TMEM123
B_cell	EGR1
B_cell	NR4A2
B_cell	MAP3K8
B_cell	AGPAT2
B_cell	RPL10P3
B_cell	TMEM141
B_cell	PCK1
B_cell	FNBP1
B_cell	AGR3
B_cell	RP11-138A9.2
B_cell	MSN
B_cell	ACTN4
B_cell	CAMK2N1
B_cell	STARD10
B_cell	MUC13
B_cell	ADIRF
B_cell	TSC22D1
B_cell	ID1
B_cell	USMG5
B_cell	SRI
B_cell	KLF4
B_cell	ETHE1
B_cell	ARL14
B_cell	ATP1B1
B_cell	UQCRC1
B_cell	SAMSN1
B_cell	TST
B_cell	NBL1
B_cell	ELF1
B_cell	PNISR
B_cell	RHOC
B_cell	HSPA1A
B_cell	RND3
B_cell	CEACAM5
B_cell	CDHR5
B_cell	CD24
B_cell	CASP4
B_cell	ACADVL
B_cell	TRBC2

B_cell	SWAP70
B_cell	RP11-51O6.1
B_cell	BLVRB
B_cell	CTSH
B_cell	DSTN
B_cell	CLTB
B_cell	FOS
B_cell	CA1
B_cell	GPX2
B_cell	GPA33
B_cell	CES2
B_cell	COMTD1
B_cell	MGST1
B_cell	ETFB
B_cell	MPST
B_cell	KMT2E
B_cell	ROMO1
B_cell	NGLY1
B_cell	PKP3
B_cell	IL32
B_cell	TXNDC17
B_cell	SERINC2
B_cell	CDH17
B_cell	RSL24D1
B_cell	NDUFS6
B_cell	PRAP1
B_cell	KLF5
B_cell	S100A11
B_cell	IFITM2
B_cell	CYC1
B_cell	SDCBP2
B_cell	ID2
B_cell	KRT20
B_cell	VSIG2
B_cell	GUCA2A
B_cell	HSD11B2
B_cell	TSPO
B_cell	RGS2
B_cell	FCGRT
B_cell	LLGL2
B_cell	SLC22A18
B_cell	ST14
B_cell	SPATS2L
B_cell	TMEM176A

B_cell	GIPC1
B_cell	ACADS
B_cell	TFF3
B_cell	AURKAIP1
B_cell	SNRPN
B_cell	RPL7P9
B_cell	AOC1
B_cell	TMEM176B
B_cell	MVP
B_cell	LIMA1
B_cell	CA12
B_cell	SPINK1
B_cell	CDX1
B_cell	MUC12
B_cell	PRSS8
B_cell	TUBA1C
B_cell	DSP
B_cell	RP11-357H14.17
B_cell	SPINT1
B_cell	PHF20
B_cell	FABP5
B_cell	MYH14
B_cell	JUP
B_cell	PKIB
B_cell	NDUFB10
B_cell	CYSTM1
B_cell	STRA13
B_cell	LDLR
B_cell	ASL
B_cell	RHOB
B_cell	SP110
B_cell	LMO7
B_cell	SEPW1
B_cell	HNRNPA1P48
B_cell	BLOC1S1.1
B_cell	MRPL41
B_cell	RN7SK
B_cell	FAM49B
B_cell	ADPGK
B_cell	MIS18BP1
B_cell	CMTM6
B_cell	C4orf48
B_cell	ETS2
B_cell	CSTB

B_cell	CLK4
B_cell	HMGCS2
B_cell	DSC2
B_cell	NDUFB3
B_cell	EIF3L
B_cell	DSG2
B_cell	FAM107B
B_cell	CHMP2A
B_cell	NDUFB1
B_cell	SLC26A2
B_cell	MT2A
B_cell	EPS8L3
B_cell	CLDN23
B_cell	CD55
B_cell	CD47
B_cell	STK17B
B_cell	RASSF7
B_cell	UGDH
B_cell	UGP2
B_cell	MAP4K4
B_cell	PERP
B_cell	SPINT2
B_cell	NDUFS7
B_cell	MAL2
B_cell	CYP3A5
B_cell	LIPH
B_cell	PDE4C
B_cell	OSBPL8
B_cell	GOLM1
B_cell	MAPK3
B_cell	PAIP2
B_cell	FAM162A
B_cell	RALGPS2
B_cell	TCEA3
B_cell	PRAC1
B_cell	RPS4Y1
B_cell	MRPL12
B_cell	GPRC5A
B_cell	RAB25
B_cell	ABCC3
B_cell	PNKD
B_cell	PRDX2
B_cell	FAM46A
B_cell	AREG

B_cell	PTPN1
B_cell	ALKBH7
B_cell	BLOC1S2
B_cell	C6orf48
B_cell	MGST2
B_cell	PLS1
B_cell	LY6E
B_cell	C11orf58
B_cell	LAMB3
B_cell	STAP2
B_cell	FYTTD1
B_cell	GSN
B_cell	NUPR1
B_cell	RBM47
B_cell	CDH1
B_cell	ALDH2
B_cell	FBXW5
B_cell	FAM195A
B_cell	PPP1R1B
B_cell	HIGD1A
B_cell	FCGBP
B_cell	LSR
B_cell	ATP8B1
B_cell	MXD1
B_cell	PARP14
B_cell	S100P
B_cell	NAPRT1
B_cell	SLC26A3
B_cell	CCL15
B_cell	TMPRSS2
B_cell	PHPT1
B_cell	GRB2
B_cell	CCDC64B
B_cell	SLIRP
B_cell	VIL1
B_cell	SPTBN1
B_cell	FHL2
B_cell	MGST3
B_cell	C19orf70
B_cell	WFDC2
B_cell	MGLL
B_cell	AC093106.7
B_cell	DST
B_cell	ICAM3

B_cell	PPP1R14D
B_cell	TCF7L2
B_cell	NXT1
B_cell	SSFA2
B_cell	GDF15
B_cell	TRPM4
B_cell	CTNND1
B_cell	MALL
B_cell	TIMM13
B_cell	ALG13
B_cell	SLC39A5
B_cell	SOX4
B_cell	C2orf82
B_cell	RNASE1
B_cell	PLAUR
B_cell	EEF1G
B_cell	MAOA
B_cell	LGALS2
B_cell	ZG16
B_cell	CORO1B
B_cell	HBEGF
B_cell	RASGEF1B
B_cell	SQRDL
B_cell	EFHD2
B_cell	RGCC
B_cell	FLNB
B_cell	S100A13
B_cell	EMP1
B_cell	EDN1
B_cell	CTNNA1
B_cell	ACAA2
B_cell	SULT1A1
B_cell	SH3BGRL
B_cell	TRIM38
B_cell	ABHD11
B_cell	EPS8
B_cell	ECHS1
B_cell	CHP2
B_cell	CTD-2192J16.15
B_cell	UGT2B17
B_cell	PLA2G2A
B_cell	CKMT1B
B_cell	GOLIM4
B_cell	USH1C

B_cell	SOCS3
B_cell	MT1X
B_cell	ISOC2
B_cell	CRB3
B_cell	PVRL2
B_cell	APP
B_cell	APLP2
B_cell	CXADR
B_cell	NFKB2
B_cell	MT1M
B_cell	SEPP1
B_cell	NDUFS8
B_cell	CDX2
B_cell	CMBL
B_cell	TYMP
B_cell	VMP1
B_cell	GGT6
B_cell	LINC01133
B_cell	PPP1R2
B_cell	HNF4A
B_cell	MARCKS
B_cell	LAD1
B_cell	EMP2
B_cell	DRAM2
B_cell	UPP1
B_cell	TJP3
B_cell	CEACAM7
B_cell	GUCA2B
B_cell	SLC4A4
B_cell	FAM84A
B_cell	CHMP4B
B_cell	ITGA6
B_cell	SELT
B_cell	MS4A12
B_cell	EFNA1
B_cell	PHB
B_cell	MUC1
B_cell	MUC3A
B_cell	CTTN
B_cell	PPAP2C
B_cell	CEACAM1
B_cell	SUCLG1
B_cell	TFF1
B_cell	RARRES2

B_cell	CFDP1
B_cell	TIMM8B
B_cell	FBLIM1
B_cell	IGFBP2
B_cell	TDP2
B_cell	NET1
B_cell	DHRS11
B_cell	LINC00035
B_cell	TNFRSF1A
B_cell	TRIM31
B_cell	FABP2
B_cell	ANXA4
B_cell	RP11-467L13.7
B_cell	ASS1
B_cell	NDUFV1
B_cell	C6orf222
B_cell	PLEC
B_cell	AK1
B_cell	CNPY3
B_cell	WBP5
B_cell	MUC5B
B_cell	MAPK1IP1L
B_cell	EPS8L2
B_cell	MUC4
B_cell	NPDC1
B_cell	SDC4
B_cell	CAPNS1
B_cell	PLA2G10
B_cell	PTPRF
B_cell	CDA
B_cell	CTSA
B_cell	SLPI
B_cell	MYO1A
B_cell	RNMT
B_cell	SERPINB6
B_cell	RP11-532F12.5
B_cell	CDKN2B-AS1
B_cell	AHCYL2
B_cell	PPP1R16A
B_cell	ERBB3
B_cell	FUT3
B_cell	GPT
B_cell	PDLIM5
B_cell	HDLBP

B_cell	ABHD17C
B_cell	SULT1B1
B_cell	SLC25A1
B_cell	TP53I3
B_cell	AP1M2
B_cell	PKIG
B_cell	INF2
B_cell	HEBP2
B_cell	MAFF
B_cell	SMCHD1
B_cell	TRIB1
B_cell	TINAGL1
B_cell	ADM
B_cell	TMPRSS4
B_cell	GTF3A
B_cell	EIF1B
B_cell	ST6GALNAC1
B_cell	GALE
B_cell	C8orf4
B_cell	USP53
B_cell	MGAT4B
B_cell	ERRFI1
B_cell	TAX1BP3
B_cell	APOBR
B_cell	C1orf21
B_cell	CA4
B_cell	NEDD4L
B_cell	VILL
B_cell	DNM2
B_cell	ANKRD9
B_cell	GNA11
B_cell	RIOK3
B_cell	SLC51B
B_cell	ZFAND2A
B_cell	CKMT1A
B_cell	TNRC6B
B_cell	EFNB2
B_cell	KDM6B
B_cell	SHD
B_cell	NDRG2
B_cell	CD177
B_cell	AVPI1
B_cell	GNG12
B_cell	NR2F6

B_cell	ACAA1
B_cell	SLC20A1
B_cell	GMDS
B_cell	PRR15
B_cell	GCNT3
B_cell	RABL6
B_cell	CCDC50
B_cell	SLC44A1
B_cell	THBS1
B_cell	KIF1C
B_cell	AKR1B10
B_cell	CBLC
B_cell	SS18L2
B_cell	CAPN5
B_cell	NQO1
B_cell	FAM129B
B_cell	ECI1
B_cell	LAMA3
B_cell	PYGB
B_cell	ST3GAL4
B_cell	BCAS1
B_cell	ANPEP
B_cell	B3GNT5
B_cell	PLXNB2
B_cell	TMEM45B
B_cell	HEXIM1
B_cell	TRIM15
B_cell	H1F0
B_cell	FAM46C
B_cell	MDK
B_cell	EHF
B_cell	SNHG18
B_cell	AQP8
B_cell	HOXB7
B_cell	SCNN1A
B_cell	ADAM15
B_cell	PRR15L
B_cell	PXMP2
B_cell	TMC5
B_cell	SHROOM3
B_cell	PADI2
B_cell	ADAP1
B_cell	ERN2
B_cell	NDFIP2

B_cell	ARHGEF16
B_cell	ESPN
B_cell	SATB2
B_cell	PLCD3
B_cell	HRCT1
B_cell	TMC4
B_cell	AKR7A3
B_cell	HSBP1L1
B_cell	BOLA3
B_cell	MARVELD3
B_cell	MT1F
B_cell	DHRS9
B_cell	ATP10B
B_cell	CXCL3
B_cell	TICAM1
B_cell	QSOX1
B_cell	BMP2
B_cell	CEACAM6
B_cell	SIRT6
B_cell	BAIAP2L1
B_cell	TPRN
B_cell	HPGD
B_cell	RETSAT
B_cell	RP11-670E13.6
B_cell	HK2
B_cell	C2orf88
B_cell	EPS8L1
B_cell	IL4R
B_cell	ZMYM2
B_cell	NUDT8
B_cell	LGALS1
B_cell	CXCL2
B_cell	CLCA4
B_cell	BCAS2
B_cell	MUC2
Monocyte	TYROBP
Monocyte	AIF1
Monocyte	LST1
Monocyte	C1QA
Monocyte	MS4A7
Monocyte	C1QB
Monocyte	PTGS2
Monocyte	SPI1
Monocyte	C1QC

Monocyte	PLEK
Monocyte	IGSF6
Monocyte	TNFAIP2
Monocyte	MS4A6A
Monocyte	CYBB
Monocyte	FAM26F
Monocyte	OLR1
Monocyte	CLEC7A
Monocyte	CFP
Monocyte	CD86
Monocyte	PLA2G7
Monocyte	FAM49A
Monocyte	SDS
Monocyte	CLEC10A
Monocyte	TNFSF13B
Monocyte	MMP12
Monocyte	FPR3
Monocyte	S100B
Monocyte	LILRB4
Monocyte	MPEG1
Monocyte	FCGR3A
Monocyte	FCGR2A
Monocyte	CSF2RA
Monocyte	CD4
Monocyte	MNDA
Monocyte	CSF1R
Monocyte	NCF2
Monocyte	GPNMB
Monocyte	CLEC4A
Monocyte	LILRB2
Monocyte	SLC8A1
Monocyte	CPVL
Monocyte	TFEC
Monocyte	ITGAX
Monocyte	SEMA6B
Monocyte	HCK
Monocyte	AXL
Monocyte	CD36
Monocyte	GGTA1P
Monocyte	CSF3R
Monocyte	LAMP3
Monocyte	CLEC4E
Monocyte	FCGR1A
Monocyte	FPR1

Monocyte	VASH1
Monocyte	LILRB1
Monocyte	KYNU
Monocyte	FGR
Monocyte	ENG
Monocyte	S100A8
Monocyte	FCER1G
Monocyte	LY96
Monocyte	DNASE1L3
Monocyte	PTAFR
Monocyte	VMO1
Monocyte	ITGB2
Monocyte	CCDC88A
Monocyte	HLA-DQA1
Monocyte	ZNF385A
Monocyte	SLAMF7
Monocyte	CD300A
Monocyte	CCL3
Monocyte	PIK3R5
Monocyte	BCL2A1
Monocyte	THEMIS2
Monocyte	ICAM1
Monocyte	PHACTR1
Monocyte	PLAU
Monocyte	SLC7A7
Monocyte	QKI
Monocyte	NAPSB
Monocyte	MIR155HG
Monocyte	DSE
Monocyte	GPR183
Monocyte	ENPP2
Monocyte	C5AR1
Monocyte	C10orf128
Monocyte	C1orf162
Monocyte	IL4I1
Monocyte	CSTA
Monocyte	NRP1
Monocyte	TNF
Monocyte	BASP1
Monocyte	HLA-DMB
Monocyte	SPHK1
Monocyte	LY86
Monocyte	STX11
Monocyte	SIRPA

Monocyte	LAIR1
Monocyte	RNASE6
Monocyte	LCP2
Monocyte	RASGRP3
Monocyte	EMILIN2
Monocyte	PSTPIP2
Monocyte	SERPINB9
Monocyte	SLC15A3
Monocyte	LCP1
Monocyte	HLA-DPB1
Monocyte	ZEB2
Monocyte	OGFRL1
Monocyte	GPR132
Monocyte	CCL3L3
Monocyte	FGL2
Monocyte	CXCL8
Monocyte	IL1B
Monocyte	TUBB6
Monocyte	PLEKHO1
Monocyte	IL18BP
Monocyte	HLA-DPA1
Monocyte	CD40
Monocyte	CECR1
Monocyte	TGFBI
Monocyte	RNF130
Monocyte	ADAP2
Monocyte	GNA15
Monocyte	RGS19
Monocyte	ST8SIA4
Monocyte	ACP5
Monocyte	VIM
Monocyte	TBXAS1
Monocyte	NCF1C
Monocyte	MRC1
Monocyte	PLXNC1
Monocyte	HLA-DQB1
Monocyte	NCKAP1L
Monocyte	SAMHD1
Monocyte	CD83
Monocyte	IFI30
Monocyte	LYZ
Monocyte	NINJ1
Monocyte	LIPA
Monocyte	UBE2E2

Monocyte	ASAP1
Monocyte	IL1RN
Monocyte	SLC2A3
Monocyte	CTSH
Monocyte	RAB31
Monocyte	DRAM1
Monocyte	HLA-DMA
Monocyte	CD53
Monocyte	C1orf54
Monocyte	NRIP3
Monocyte	SRGN
Monocyte	BST2
Monocyte	LPXN
Monocyte	PPT1
Monocyte	AMICA1
Monocyte	S100A9
Monocyte	IDO1
Monocyte	LSP1
Monocyte	SNX10
Monocyte	PARVG
Monocyte	SLC43A2
Monocyte	TRAF1
Monocyte	TNFAIP8
Monocyte	EMP3
Monocyte	RGS10
Monocyte	GPR137B
Monocyte	PTPRE
Monocyte	S100A4
Monocyte	HLA-DRA
Monocyte	DAPK1
Monocyte	ARL4C
Monocyte	ARRB2
Monocyte	LAPTM5
Monocyte	FAM110A
Monocyte	FLNA
Monocyte	RGS1
Monocyte	HAVCR2
Monocyte	LIMS1
Monocyte	LGALS1
Monocyte	RASSF5
Monocyte	CCL4L1
Monocyte	CCL4
Monocyte	IL10RA
Monocyte	NR4A3

Monocyte	CTSL
Monocyte	PMP22
Monocyte	ADA
Monocyte	GPSM3
Monocyte	TNFRSF1B
Monocyte	GLIPR1
Monocyte	LAT2
Monocyte	GMFG
Monocyte	CD48
Monocyte	FYB
Monocyte	GM2A
Monocyte	APOC1
Monocyte	ARHGDIB
Monocyte	LYN
Monocyte	MSN
Monocyte	ABCA1
Monocyte	NCF4
Monocyte	ABI3
Monocyte	HLA-DRB1
Monocyte	ANXA5
Monocyte	FXYD5
Monocyte	SAMSN1
Monocyte	PEA15
Monocyte	MYO1F
Monocyte	HCLS1
Monocyte	CORO1A
Monocyte	PTPRC
Monocyte	NFKBID
Monocyte	CPPED1
Monocyte	UCP2
Monocyte	AP1S2
Monocyte	COTL1
Monocyte	GNAI2
Monocyte	TIMP1
Monocyte	CXCL16
Monocyte	CD74
Monocyte	IGFLR1
Monocyte	ZNF267
Monocyte	HCST
Monocyte	SH2B3
Monocyte	RILPL2
Monocyte	G0S2
Monocyte	DOK2
Monocyte	TGFB1

Monocyte	LPCAT1
Monocyte	ZFYVE16
Monocyte	TUBA1A
Monocyte	RASGEF1B
Monocyte	PTGER2
Monocyte	CCRL2
Monocyte	ATP6V1B2
Monocyte	CAMK1
Monocyte	CHST11
Monocyte	FERMT3
Monocyte	HLA-DRB5
Monocyte	AAED1
Monocyte	RUNX3
Monocyte	CSGALNACT2
Monocyte	SLC6A6
Monocyte	ENTPD1
Monocyte	PFKFB3
Monocyte	WARS
Monocyte	MAP3K8
Monocyte	LY6E
Monocyte	ABL2
Monocyte	RELT
Monocyte	TPP1
Monocyte	FAM49B
Monocyte	RHOQ
Monocyte	GRINA
Monocyte	PTPN1
Monocyte	SLC31A2
Monocyte	NAMPT
Monocyte	BLOC1S6
Monocyte	PDE4B
Monocyte	BID
Monocyte	ARID5A
Monocyte	DPYSL2
Monocyte	CD52
Monocyte	PLXDC2
Monocyte	LITAF
Monocyte	MGAT1
Monocyte	ODF3B
Monocyte	WIPF1
Monocyte	M6PR
Monocyte	CD44
Monocyte	PIK3AP1
Monocyte	RNF149

Monocyte	EPB41L3
Monocyte	SMAP2
Monocyte	NFKB1
Monocyte	TYMP
Monocyte	CTSB
Monocyte	ATP6V1F
Monocyte	JARID2
Monocyte	ZNF331
Monocyte	RGCC
Monocyte	ANXA1
Monocyte	C9orf72
Monocyte	GPX1
Monocyte	KRT8
Monocyte	NPC2
Monocyte	AOAH
Monocyte	PDE4DIP
Monocyte	LGALS4
Monocyte	OSBPL8
Monocyte	KRT18
Monocyte	PHGR1
Monocyte	IL7R
Monocyte	CLDN3
Monocyte	SOCS3
Monocyte	STARD3NL
Monocyte	CLDN4
Monocyte	PIGR
Monocyte	RDX
Monocyte	LINC00152
Monocyte	ATF5
Monocyte	ARPC1B
Monocyte	IRF8
Monocyte	OAZ2
Monocyte	ELF3
Monocyte	GNA13
Monocyte	CTD-2336O2.1
Monocyte	IL6R
Monocyte	VAMP5
Monocyte	NAP1L1
Monocyte	KRT19
Monocyte	ICOSLG
Monocyte	EVI2B
Monocyte	MALT1
Monocyte	ARL8B
Monocyte	NUDT3

Monocyte	DUSP2
Monocyte	CMTM6
Monocyte	NFKBIE
Monocyte	CLN8
Monocyte	CTSC
Monocyte	BHLHE40
Monocyte	FXYD3
Monocyte	PNRC1
Monocyte	PURB
Monocyte	ATP13A3
Monocyte	NQO2
Monocyte	CLDN7
Monocyte	LPAR6
Monocyte	CXCR4
Monocyte	IFNGR1
Monocyte	SMIM22
Monocyte	AHR
Monocyte	EPCAM
Monocyte	C19orf33
Monocyte	CEBPB
Monocyte	ITM2C
Monocyte	PSAP
Monocyte	AGR2
Monocyte	SMIM3
Monocyte	RHOG
Monocyte	MMP14
Monocyte	SOD2
Monocyte	PPDPF
Monocyte	MYO9B
Monocyte	FNIP2
Monocyte	GLIPR2
Monocyte	S100A14
Monocyte	PPIF
Monocyte	CREM
Monocyte	STAT1
Monocyte	NAGK
Monocyte	RB1
Monocyte	TNFAIP3
Monocyte	ETV3
Monocyte	SH3BGRL
Monocyte	GRB2
Monocyte	CD24
Monocyte	TSPAN8
Monocyte	KDM6B

Monocyte	CHMP1B
Monocyte	TMEM54
Monocyte	FAM3D
Monocyte	INSIG1
Monocyte	HIF1A
Monocyte	WTAP
Monocyte	GABARAP
Monocyte	CKB
Monocyte	FABP1
Monocyte	C10orf99
Monocyte	REL
Monocyte	ANKRD28
Monocyte	ATPIF1
Monocyte	CREG1
Monocyte	MTHFD2
Monocyte	MOB1A
Monocyte	NR4A2
Monocyte	CHCHD10
Monocyte	SLC7A11
Monocyte	AMN
Monocyte	CDC42EP5
Monocyte	GNS
Monocyte	CTSS
Monocyte	IFI27
Monocyte	GDI2
Monocyte	PHLDA1
Monocyte	LAP3
Monocyte	RGS2
Monocyte	GPCPD1
Monocyte	VOPP1
Monocyte	TSC22D3
Monocyte	C10orf54
Monocyte	GABARAPL1
Monocyte	PLSCR1
Monocyte	ARFGAP3
Monocyte	UQCRQ
Monocyte	AKIRIN2
Monocyte	SELENBP1
Monocyte	ZMIZ1
Monocyte	GLUL
Monocyte	NFE2L2
Monocyte	STK4
Monocyte	PIM3
Monocyte	RAB20

Monocyte	CFLAR
Monocyte	ASAH1
Monocyte	PRMT2
Monocyte	CAPZA1
Monocyte	S100A16
Monocyte	ALCAM
Monocyte	TMSB4XP4
Monocyte	GNB1
Monocyte	SDCBP
Monocyte	NDUFB7
Monocyte	PRSS3
Monocyte	STARD10
Monocyte	CEACAM5
Monocyte	NUMB
Monocyte	ATP6V1H
Monocyte	MT1E
Monocyte	AGR3
Monocyte	MAP3K2
Monocyte	VMA21
Monocyte	SFN
Monocyte	CTSZ
Monocyte	TSPAN1
Monocyte	CAMK2N1
Monocyte	MISP
Monocyte	SGK1
Monocyte	ACTN4
Monocyte	TPM1
Monocyte	GK
Monocyte	NBN
Monocyte	DSP
Monocyte	UGP2
Monocyte	CD14
Monocyte	GLA
Monocyte	KRT20
Monocyte	PCK1
Monocyte	ACTN1
Monocyte	MT1G
Monocyte	HSD11B2
Monocyte	RALA
Monocyte	PLAUR
Monocyte	ATOX1
Monocyte	TFF3
Monocyte	ARL14
Monocyte	PRDX2

Monocyte	NBL1
Monocyte	TMEM141
Monocyte	EREG
Monocyte	PRAP1
Monocyte	MAT2A
Monocyte	RAPGEF2
Monocyte	USP12
Monocyte	GPA33
Monocyte	PDLIM1
Monocyte	RRBP1
Monocyte	SDCBP2
Monocyte	VSIG2
Monocyte	GIPC1
Monocyte	RND3
Monocyte	CES2
Monocyte	SERINC2
Monocyte	SLC44A4
Monocyte	GPX2
Monocyte	ACADVL
Monocyte	FNDC3B
Monocyte	GUCA2A
Monocyte	BSG
Monocyte	ADIRF
Monocyte	PKP3
Monocyte	H2AFJ
Monocyte	DSTN
Monocyte	ETHE1
Monocyte	CD151
Monocyte	BRI3
Monocyte	SRI
Monocyte	MUC13
Monocyte	LLGL2
Monocyte	LGALS3BP
Monocyte	ID1
Monocyte	RHOB
Monocyte	PLIN2
Monocyte	CA1
Monocyte	CDX1
Monocyte	SPTBN1
Monocyte	KLF5
Monocyte	FCGBP
Monocyte	ROMO1
Monocyte	TXNDC17
Monocyte	UQCRC1

Monocyte	ETFB
Monocyte	DUSP6
Monocyte	SPINK1
Monocyte	RP11-357H14.17
Monocyte	BACH1
Monocyte	CA12
Monocyte	MGST1
Monocyte	MAPK3
Monocyte	PERP
Monocyte	MUC12
Monocyte	STAP2
Monocyte	FAM195A
Monocyte	MYH14
Monocyte	JUP
Monocyte	ASS1
Monocyte	LTB
Monocyte	COMTD1
Monocyte	CXCL2
Monocyte	CYP3A5
Monocyte	ID3
Monocyte	HIGD1A
Monocyte	CISD3
Monocyte	AOC1
Monocyte	PNKD
Monocyte	CDHR5
Monocyte	PPP1R1B
Monocyte	OCIAD2
Monocyte	ASL
Monocyte	MAL2
Monocyte	LIMA1
Monocyte	FLNB
Monocyte	LSR
Monocyte	MPST
Monocyte	CA2
Monocyte	WFDC2
Monocyte	PDE4C
Monocyte	MRPL41
Monocyte	ALKBH7
Monocyte	AGPAT2
Monocyte	GOLM1
CMP	TPSB2
CMP	TPSAB1
CMP	CPA3
CMP	LTC4S

CMP	HPGDS
CMP	GATA2
CMP	KRT1
CMP	MS4A2
CMP	SLC18A2
CMP	RP11-354E11.2
CMP	IL1RL1
CMP	PTGS1
CMP	RGS13
CMP	RENBP
CMP	SIGLEC17P
CMP	GCSAML
CMP	FCER1A
CMP	CATSPER1
CMP	C1orf186
CMP	PLAT
CMP	HDC
CMP	CNRIP1
CMP	CALB2
CMP	NTRK1
CMP	CD33
CMP	CDK15
CMP	ARHGEF6
CMP	P2RX1
CMP	MAOB
CMP	KIT
CMP	TYROBP
CMP	FCER1G
CMP	COL18A1
CMP	FHL3
CMP	BTK
CMP	ACSL4
CMP	CSF2RB
CMP	ALOX5
CMP	APOE
CMP	BCL2A1
CMP	KLRG1
CMP	EMR2
CMP	C1orf162
CMP	PTGS2
CMP	BLVRA
CMP	CSF1
CMP	DUSP14
CMP	ALOX5AP

CMP	C1orf228
CMP	BST2
CMP	CTTNBP2
CMP	C10orf128
CMP	SVOPL
CMP	TESC
CMP	STX11
CMP	SLC2A3
CMP	VIM
CMP	PHACTR1
CMP	PTRF
CMP	RP11-465B22.3
CMP	APOC1
CMP	CD22
CMP	TESPA1
CMP	TUBA1A
CMP	RGS1
CMP	GRAP2
CMP	SAMSN1
CMP	ANXA1
CMP	S100A4
CMP	CLU
CMP	LAPTM5
CMP	LAT2
CMP	SRGN
CMP	EMP3
CMP	FXYD5
CMP	VWA5A
CMP	CD84
CMP	CD69
CMP	RAC2
CMP	SLC45A3
CMP	CD83
CMP	TNFAIP8
CMP	AKAP12
CMP	TNFRSF4
CMP	LYL1
CMP	LGALS1
CMP	GMPR
CMP	PARVB
CMP	CD37
CMP	ICAM1
CMP	ADRB2
CMP	ITM2A

CMP	DUSP10
CMP	GALNT6
CMP	RASSF5
CMP	CMTM3
CMP	CD44
CMP	PTPN7
CMP	CD52
CMP	RAB34
CMP	HAVCR2
CMP	ARHGDIB
CMP	TMEM154
CMP	NR4A3
CMP	LCP2
CMP	CD53
CMP	GMFG
CMP	CD38
CMP	LCP1
CMP	GPR65
CMP	HS3ST1
CMP	LAX1
CMP	SMYD3
CMP	TBXAS1
CMP	TIMP1
CMP	HCST
CMP	ANKRD28
CMP	MAML1
CMP	TGFB1
CMP	CD82
CMP	DUSP6
CMP	RAB27B
CMP	CDC42EP3
CMP	WASH6P
CMP	CHST11
CMP	CKLF
CMP	ZNF331
CMP	GLUL
CMP	ZEB2
CMP	RASAL3
CMP	TCEAL3
CMP	PMP22
CMP	NSMCE1
CMP	BEX4
CMP	LPCAT2
CMP	ELOVL5

CMP	CAPG
CMP	MATK
CMP	LAPTM4A
CMP	LEO1
CMP	DEGS1
CMP	RGS10
CMP	NFKBID
CMP	ABCC4
CMP	PRNP
CMP	ACP5
CMP	MSRA
CMP	CREM
CMP	ITM2B
CMP	LMNA
CMP	RP11-347P5.1
CMP	RGS2
CMP	ATP6V1F
CMP	CLDN3
CMP	SLC7A5
CMP	KDM6B
CMP	CLDN4
CMP	ELL2
CMP	KRT18
CMP	NR4A2
CMP	IFITM2
CMP	PIGR
CMP	PRKD3
CMP	BHLHE40
CMP	MSN
CMP	IDS
CMP	SDCBP
CMP	MPP1
CMP	PRKX
CMP	NFKBIA
CMP	ELF3
CMP	KRT8
CMP	HPGD
CMP	LGALS4
CMP	AP1S2
CMP	CLDN7
CMP	SUMO2
CMP	C15orf48
CMP	CTSW
CMP	RHBDD2

CMP	FAM110A
CMP	FERMT3
CMP	SKIL
CMP	SMIM22
CMP	MORF4L1
CMP	SEC11A
CMP	RGS16
CMP	PHGR1
CMP	2-Sep
CMP	FXYD3
CMP	EIF3E
CMP	CTNNBL1
CMP	REL
CMP	CTD-3252C9.4
CMP	TSPAN8
CMP	HIF1A
CMP	AGR2
CMP	TNFRSF18
CMP	BIRC3
CMP	FAM3D
CMP	KRT19
CMP	EPCAM
CMP	HINT1
CMP	CD24
CMP	GPX1
CMP	TIPARP
CMP	DNAJA1
CMP	TWISTNB
CMP	S100A10
CMP	AAED1
CMP	AURKA
CMP	CNIH1
CMP	AHR
CMP	C19orf33
CMP	CIRBP
CMP	GPSM3
CMP	GALC
CMP	ZBTB20
CMP	RHOH
CMP	NXT1
CMP	STMN1
CMP	AMN
CMP	MAGED2
CMP	OSBPL8

CMP	DRAP1
CMP	S100A14
CMP	SFN
CMP	TMEM54
CMP	PHF1
CMP	LGALS3BP
CMP	CHCHD10
CMP	RHOG
CMP	C10orf99
CMP	CKB
CMP	NFKB1
CMP	ARHGAP18
CMP	LMO4
CMP	SMIM3
CMP	NFKBIZ
CMP	TNFSF10
CMP	SLC44A4
CMP	NAP1L1
CMP	ANXA2
CMP	HSPH1
CMP	B4GALT5
CMP	MYADM
CMP	SWAP70
CMP	PPT1
CMP	PDLIM1
CMP	PRSS3
CMP	STK17B
CMP	TSC22D3
CMP	ACTR3
CMP	MARCKS
CMP	PLIN2
CMP	CDC42EP5
CMP	MISP
CMP	IFI27
CMP	AGR3
CMP	SH3BGRL
CMP	SNX3
CMP	EZR
CMP	CNBP
CMP	LIMS1
CMP	DSP
CMP	RHOB
CMP	CAMK2N1
CMP	CEACAM5

CMP	MAPK6
CMP	GPX2
CMP	TSPAN1
CMP	PRDX5
CMP	BLOC1S2
CMP	ASAH1
CMP	PAG1
CMP	RAB11FIP1
CMP	NDUFB7
CMP	AGPAT2
CMP	ATP1B1
CMP	NR3C1
CMP	NFE2L2
CMP	DYNLT3
CMP	ID1
CMP	SELENBP1
CMP	KLF5
CMP	CA2
CMP	NCOA4
CMP	ATP5I
CMP	SERINC2
CMP	LDHB
CMP	ARL14
CMP	TNFAIP3
CMP	RRBP1
CMP	AGO3
CMP	COMTD1
CMP	TMEM141
CMP	COTL1
CMP	PHLDA1
CMP	CDX1
CMP	FHOD1
CMP	ETHE1
CMP	MUC12
CMP	KRT20
CMP	PBX1
CMP	MPST
CMP	GPA33
CMP	MT1G
CMP	CLTB
CMP	VSIG2
CMP	MUC13
CMP	GADD45B
CMP	EID1

CMP	ARL6IP5
CMP	MT1E
CMP	MAPK1
CMP	BCAS2
CMP	SDCBP2
CMP	SPINK1
CMP	TMEM176A
CMP	S100A16
CMP	ID3
CMP	SUPT4H1
CMP	ATPIF1
CMP	LY6E
CMP	PLA2G2A
CMP	FOXP1
CMP	TPD52
CMP	BLVRB
CMP	TPM1
CMP	RND3
CMP	MAP3K8
CMP	OSTF1
CMP	ACOT7
CMP	RP11-357H14.17
CMP	CA12
CMP	ZNF267
CMP	ST14
CMP	FABP1
CMP	RASGEF1B
CMP	LEPROTL1
CMP	AURKAIP1
CMP	RBM47
CMP	DNAJB9
CMP	MIR4435-1HG
CMP	CYP3A5
CMP	ATF3
CMP	FAM195B
CMP	PCK1
CMP	C11orf58
CMP	PLAC8
CMP	BMP2K
CMP	DAD1
CMP	RBBP8
CMP	HSD17B12
CMP	IER3
CMP	RBM8A

CMP	SPINT1
CMP	TERF2IP
CMP	MAPK3
CMP	STARD10
CMP	AOC1
CMP	H2AFJ
CMP	SLC4A2
CMP	DSC2
CMP	PRKAR1A
CMP	NUPR1
CMP	M6PR
CMP	AREG
CMP	ALKBH7
CMP	TFF3
CMP	BNIP2
CMP	TSNAX
CMP	GPRC5A
CMP	TXNDC17
CMP	RN7SK
CMP	PRAC1
CMP	TST
CMP	DDIT4
CMP	CYC1
CMP	GOLM1
CMP	FABP5
CMP	CDH1
CMP	VAMP2
