# Supplementary material for: Expression of immune-related genes and possible regulatory mechanisms in ulcerative colitis
Source: Front Mol Biosci. 2026 Mar 5;13:1621643. doi: 10.3389/fmolb.2026.1621643 (PMC12999447; doi:10.3389/fmolb.2026.1621643)
Supplement: Supplementary file 3 [file Table1.pdf]

Supplementary Table 1 Clinical information of GSE116222 samples

| Patient group | N | Age (years) (median [range]) | Gender (n (%) male) |
|---|---|---|---|
| healthy | 3 | 50 [47-74] | 1 (33%) |
| adjacent non-inflamed areas of UC | 3 | 55 [36-80] | 2 (66%) |
| inflamed areas of UC samples | 3 | 55 [36-80] | 2 (66%) |
