# Supplementary material for: Expression of immune-related genes and possible regulatory mechanisms in ulcerative colitis
Source: Front Mol Biosci. 2026 Mar 5;13:1621643. doi: 10.3389/fmolb.2026.1621643 (PMC12999447; doi:10.3389/fmolb.2026.1621643)
Supplement: Supplementary file 4 [file Table2.pdf]

Supplementary Table 2 Cell Type Annotation

Cluster ID	Cell type
0	Epithelial_cells
1	Paneth_cells
2	Epithelial_cells
3	Intestinal_stem_cells
4	T_cells
5	Epithelial_cells
6	B_cells
7	Epithelial_cells
8	B_cells
9	Monocyte
10	CMP

We use SingleR to define the score for each cluster as 0.8.
