# Supplementary material for: Expression of immune-related genes and possible regulatory mechanisms in ulcerative colitis
Source: Front Mol Biosci. 2026 Mar 5;13:1621643. doi: 10.3389/fmolb.2026.1621643 (PMC12999447; doi:10.3389/fmolb.2026.1621643)
Supplement: Supplementary file 5 [file Table6.pdf]

Supplementary Table 6 Fold changes and statistical significance of TPSB2 and TPSAB1 between UC inflammation and healthy samples

p_val	avg_log2FC	p_val_adj	gene
0	4.6650864185043	0	TPSAB1
0	4.70040011411171	0	TPSB2
