# Supplementary material for: Expression of immune-related genes and possible regulatory mechanisms in ulcerative colitis
Source: Front Mol Biosci. 2026 Mar 5;13:1621643. doi: 10.3389/fmolb.2026.1621643 (PMC12999447; doi:10.3389/fmolb.2026.1621643)
Supplement: Supplementary file 6 [file Table3.pdf]

Supplementary Table 3 Main indication of separating UC from normal cells

Gene
RARRES2
EDN1
ADH1C
MAFB
PPP1R1B
GMNN
GDF15
HES1
CD320
SOX4
NUPR1
LEFTY1
TOMM40
LGALS2
C2orf82
MRPL12
METTL12
RN7SK
STARD10
QTRT1
RP11-395A13.2
MLEC
DDX21
GAS6
HEXIM1
TRABD2A
IGFBP2
PYCARD
GADD45GIP1
IMPDH2
NHP2
DNPH1
PHB
ATP5G1
FBL
MRPS12
CXCL3
RANBP1
C1QBP
TIMM13

FABP5
RHOV
BOLA3
PLK2
NME1
PA2G4
C19orf48
UQCC2
NCL
ZNF593
NDRG2
FAM84A
CYC1
SURF6
TRAP1
RPL13P12
SNRPF
MACROD1
CLUH
WBP5
TLE4
NUDT8
MRPS26
C1orf35
RP11-234A1.1
SNHG8
HIST1H4C
NAA38
CXCL2
C17orf89
MCM7
SNHG5
RPL36A
NUCKS1
LRRC75A-AS1
MRPL4
NGFRAP1
DANCR
NDUFS8
TIMM10
APRT
SLC25A10.1
CDC42EP1

TRIM28
TMEM261
MIF
GPX2
REPIN1
MZT2A
SNRPD2
NOP56
UGT2A3
PDCD5
PRAC1
MRPS25
RP11-466H18.1
TCEA3
IGFBP4
NOP16
CISD3
ILF3
HMGCS2
C19orf60
EIF3B
DCTPP1
MUC5B
MRPL11
GGCT
EIF3J
RPL10A
HSPE1
TIMM44
DUS1L
METAP2
MRPS34
GCHFR
POLD2
ECSIT
GAS5
GNL3
C19orf70
RPS2
ECI1
ABCF1
MRPS7
TUBA1B

EIF3A
SNORA76C
PFDN6
RPS8
RPS21
NDUFV1
C16orf13
RPL22
RND3
SUCLG1
SNRPD1
EBPL
RP11-467L13.7
ISOC2
ATP2C2
HSPD1
NSA2
STOML2
RPS17L
RPL35
EIF5B
LINC00116
RPS19
PNKD
SET
TKT
COMT
NDUFS7
DUT
PHB2
RPL18AP3
HSPBP1
RPLP0
RPS5
CLTB
PLEC
NQO1
PRR15
UBE2D3
PRR15L
SH3GLB1
SERPINB1
CD164

LINC01207
ACOX1
GABARAP
CCL28
SUMO1
SPPL2A
ANXA4
N4BP2L2
FOSL2
PSENEN
RAB5C
CTSZ
LASP1
TNFRSF1A
RBCK1
TMEM45B
TSPAN3
ATP6V1G1
AHNAK
TNNC2
KCNK1
ACTR3
ELF1
ZNF706
MAP1LC3B
TSTD1
CAB39
GNG12
CCDC107
PNPLA2
CCL20
AHCYL2
RAP1A
ZG16
TDP2
PAIP2
HTATIP2
SYTL2
SSFA2
PRAP1
PPP2R5C
TNIP1
MAX

IFNGR2
ESPN
DSC2
UBE2B
DYNLT1
STK17A
JAK1
ARPC5
STK24
SRI
HLA-A
CHMP1B
CHP1
VAPA
SMIM14
IFNGR1
PPP1R11
CDHR5
MCL1
CHMP2B
RNF213
CFLAR
CASP7
TMPRSS2
HK2
CAPZB
SH3BGRL3
LRP10
PPP1CB
MSMO1
ATP6V0E1
GABARAPL2
LDHA
MIER3
MAL2
FAM107B
TMEM120A
CAPN8
SULT1A1
CIB1
TMEM50A
CCL15
LMO7

SMPD1
CDC42SE2
FABP2
RFK
DDAH2
CAPN2
AAK1
CCNI
SMCHD1
BCAS1
ARPC1B
B3GNT5
RAP1B
CFDP1
AP3S1
TXNIP
LINC00483
AK1
MYL12B
ACAA1
ARPC3
GDPD3
HIST1H2AC
PPP1R14D
DDIT4
P2RX4
MYL12A
DST
NT5C3A
ABHD3
GPRC5A
GBP3
PRR13
SLC16A3
CAP1
RCAN1
SIRT6
ABRACL
FTH1P10
RHOG
ARPC2
ARL6IP5
LCN2

CLDN23
FHL2
INSIG1
FBXO32
TM4SF1
HLA-B
VAMP8
TAX1BP3
ITM2B
PLS1
OCIAD2
UBE2A
LEPROTL1
MGLL
SLC6A8
B2M
SMPDL3A
TNFAIP3
PLA2G10
B4GALT1
GLRX
OPTN
IFITM2
TRIM15
CD59
UPP1
LINC00035
MXD1
PRDX6
SCNN1A
SPATS2L
PKIB
SCNN1B
SDCBP2
CDKN1A
HLA-C
MALL
TNFSF10
TFF1
TMEM66
CHMP5
POLD4
HHLA2

MUC2
HIGD1A
PLIN3
PSMB9
IFI27
HSD17B2
SLC9A3R1
HLA-F
TSC22D3
HRCT1
SLC40A1
SULT1A2
ANPEP
LITAF
TSPAN1
TRIM31
IL2RG
TMSB4XP4
RHOC
HLA-E
ATP1B3
ENTPD8
ASS1
GCNT3
C12orf75
ST3GAL4
BIRC3
KRT20
CDHR2
PRDM1
C2orf88
YPEL5
SLC51B
HIST1H1C
CDKN2B-AS1
CEACAM5
SERPINA1
GSN
TMIGD1
OAS1
PLAUR
MDK
FCGBP

PI3
MYO15B
ITLN1
SLC26A3
EMP1
CD177
SPINK4
CD74
HLA-DRB1
LINC01133
HPGD
IL32
C11orf86
RHOF
GUCA2A
HLA-DRA
MS4A12
CLCA4
RARRES3
CDA
DHRS9
PLAC8
LYPD8
GUCA2B
CEACAM1
CEACAM6
AQP8
ISG15
ISG20
SEPP1
CA4
CEACAM7
AQP8
CDKN2B-AS1
MS4A12
SLC51B
GUCA2A
ANPEP
SLC26A3
CEACAM7
TMIGD1
GUCA2B
LINC01133

C11orf86
PRAP1
CEACAM1
SULT1A2
EMP1
CD177
PKIB
CDA
C2orf88
HSD17B2
CA4
SLC26A2
PLAC8
DHRS9
HPGD
SEPP1
CLDN23
ABCG2
KRT20
SULT1A1
MALL
SCNN1B
LINC00035
FLJ22763
PRDX6
SDCBP2
HIST1H1C
LYPD8
DDAH2
CA1
TMEM37
PLAUR
CLCA4
HIGD1A
C12orf75
ACAA1
SLC9A3R1
RHOF
AKR1B10
PPP1R14D
AHCYL2
PRR15
RHOC

PRSS3
TSPAN1
TAX1BP3
HRCT1
SIRT6
IL32
CTSA
HHLA2
DEFB1
SLC51A
CA2
LINC00483
ESPN
TNNC2
MYO15B
TST
SMPDL3A
MEP1A
PRR13
ETHE1
TM4SF1
GCNT3
GPRC5A
SRI
NQO1
RSAD2
OAS1
UPP1
ABCB1
CHP1
CEACAM6
CFDP1
AGPAT2
GIPC1
FABP1
PLA2G10
CD59
SFN
CDHR5
FAM132A
POLD4
DHRS11
SERINC2

CEACAM5
TMEM54
ATP1B3
SGK2
RCAN1
GDPD3
MGLL
HRASLS2
CHP2
PADI2
SLC22A18
TRIM31
ADIRF
CTSZ
BTNL3
TDP2
PRDM1
MIER3
TMEM120A
ACAA2
CHMP5
SLC17A4
MXI1
CDKN1A
VAMP8
TMEM45B
SULT1B1
PLS1
PDLIM2
APPL2
SSFA2
UBE2A
FXYD3
CRB3
CYSTM1
CES2
IFI27
MISP
CKB
ACOX1
FBXO32
LMO7
SELENBP1

AK1
CAPN2
DSC2
OASL
RHOD
HDHD3
TRPM6
CHMP2A
MSMO1
PLCD1
MYL12B
MXD1
GNG12
FTH1P10
LAMA1
ACOT8
RETSAT
TSTD1
ENTPD8
VILL
PDZD3
SPATS2L
COL17A1
EPS8
SLC22A18AS
FHL2
CDHR2
HSD17B11
CCL15
ANKRD9
CLTB
TJP3
AOC1
HMGCS1
TMPRSS2
HIST1H2AC
FCGRT
BTNL8
THOP1
C1orf115
HN1
TMCC3
GLRX

PPARG
MT1M
SLC16A1
MAL2
BSG
PRSS23
FLNB
SPINT1
GPA33
PDE9A
FBLIM1
MGST3
HSD11B2
PLIN3
PLCD3
LDHD
TRIM15
CLCN2
SLC20A1
FDPS
CLIC5
PEX26
PPAP2A
TMEM176A
CTD-2228K2.5
CHMP4B
PLEKHG6
MMP24-AS1
STAP2
CNIH4
FABP2
C1orf210
IGSF9
AMN
TMEM176B
COX6A1P2
PDLIM1
HTATIP2
CYP3A5
FUCA1
CGN
INO80C
OCIAD2

EIF6
MYL12A
LRRC19
PIGZ
TSPAN8
B3GNT5
TMEM82
PAG1
TSPAN3
CRIP1
DST
C19orf33
PRSS8
ARRDC4
TNFRSF1A
SMPD1
S100A16
CSTB
CDKN2B
CD9
LAMA3
PDE4C
SMIM6
SLC16A9
GNA11
MYH14
CDH17
MVP
PMM1
CAPG
H2AFJ
ST3GAL4
SLC25A23
ARF3
CTSD
B3GNT8
MPST
TMEM56
ACADS
TICAM1
USH1C
SPINT2
MAPRE3

HMOX1
ARL14
CA12
TNIP1
LRP10
C4orf48
AIFM2
DOK4
GDA
ABRACL
