# Supplementary material for: Expression of immune-related genes and possible regulatory mechanisms in ulcerative colitis
Source: Front Mol Biosci. 2026 Mar 5;13:1621643. doi: 10.3389/fmolb.2026.1621643 (PMC12999447; doi:10.3389/fmolb.2026.1621643)
Supplement: Supplementary file 8 [file Table8.pdf]

Supplementary Table 8 11 co-expressed TFs in UC and healthy samples

gene	Correlation
KLF2	-0.878740905
SOX4	-1.677940736
HNF4A	-1.399068255
CDX2	-1.37986464
NR2F6	-1.283419854
CC2D1A	-0.976315743
NR4A1	-0.947361623
ZBTB7A	-0.70199089
ZFP36L2	0.864849065
ARID5B	1.399688757
CREM	3.133738466
