# Supplementary material for: Expression of immune-related genes and possible regulatory mechanisms in ulcerative colitis
Source: Front Mol Biosci. 2026 Mar 5;13:1621643. doi: 10.3389/fmolb.2026.1621643 (PMC12999447; doi:10.3389/fmolb.2026.1621643)
Supplement: Supplementary file 7 [file Table7.pdf]

**Supplementary Table 7 DEGs of UC From Bulk Sequencing Data**

| gene_name | log2FoldChange | pvalue | padj | |
|---|---|---|---|---|
| RP11-116D2.1 | -24.99937375 | 6.97E-53 | 2.09E-48 | down |
| APOC3 | -12.16721067 | 3.94E-24 | 6.23E-21 | down |
| APOA4 | -12.14460842 | 5.21E-14 | 8.15E-12 | down |
| APOB | -9.671594922 | 8.76E-25 | 1.64E-21 | down |
| SULT2A1 | -9.595071376 | 8.44E-11 | 5.85E-09 | down |
| APOA1 | -9.514660136 | 1.43E-45 | 1.43E-41 | down |
| FABP6 | -9.089282466 | 1.31E-45 | 1.43E-41 | down |
| CYP3A4 | -8.762384735 | 1.65E-22 | 1.77E-19 | down |
| SLC10A2 | -8.649179013 | 8.50E-15 | 1.60E-12 | down |
| SLC5A12 | -8.447335574 | 5.72E-28 | 1.63E-24 | down |
| MS4A10 | -8.385096386 | 3.13E-15 | 6.35E-13 | down |
| CPO | -8.383188523 | 1.28E-19 | 6.86E-17 | down |
| C17orf78 | -8.270167283 | 2.00E-16 | 5.51E-14 | down |
| NA | -8.198428355 | 6.08E-14 | 9.37E-12 | down |
| SLC2A2 | -8.059142954 | 1.54E-13 | 2.17E-11 | down |
| MTTP | -7.797565186 | 2.44E-15 | 5.10E-13 | down |
| LINC00955 | -7.693807604 | 7.01E-22 | 6.38E-19 | down |
| G6PC | -7.658445695 | 1.23E-14 | 2.25E-12 | down |
| RBP2 | -7.227679308 | 2.24E-22 | 2.24E-19 | down |
| ERICH4 | -7.019225897 | 4.25E-13 | 5.19E-11 | down |
| HNF4A-AS1 | -6.836646658 | 2.76E-12 | 2.73E-10 | down |
| SLC13A1 | -6.798673907 | 1.64E-08 | 6.09E-07 | down |
| SLC6A19 | -6.774673824 | 1.39E-20 | 9.29E-18 | down |
| SLC7A9 | -6.752522897 | 1.22E-17 | 4.22E-15 | down |
| KCNJ13 | -6.535671182 | 3.05E-08 | 1.02E-06 | down |
| IGSF23 | -6.470275414 | 2.57E-19 | 1.33E-16 | down |
| SMLR1 | -6.466777324 | 1.56E-12 | 1.64E-10 | down |
| GSTA2 | -6.429241431 | 6.77E-11 | 4.81E-09 | down |
| HSD3B1 | -6.340069797 | 1.04E-11 | 9.06E-10 | down |
| UGT3A1 | -6.333175538 | 2.54E-08 | 8.65E-07 | down |
| RP11-132E11.2 | -6.324976597 | 2.05E-09 | 9.67E-08 | down |
| RP11-641C17.1 | -6.263809159 | 1.71E-07 | 4.52E-06 | down |
| SP8 | -6.222785088 | 1.30E-09 | 6.44E-08 | down |
| ADGRD2 | -6.134692912 | 1.66E-08 | 6.14E-07 | down |
| CEACAM20 | -6.124408364 | 1.42E-16 | 4.02E-14 | down |
| RP11-807H22.5 | -6.103068984 | 5.73E-23 | 7.18E-20 | down |
| DHDH | -6.0671118 | 1.82E-33 | 1.09E-29 | down |
| CREB3L3 | -6.021442221 | 2.55E-22 | 2.39E-19 | down |
| LCT | -6.005822059 | 7.29E-16 | 1.80E-13 | down |
| MEP1B | -6.002420275 | 2.95E-24 | 4.92E-21 | down |
| SLC28A1 | -5.968661038 | 8.19E-18 | 3.04E-15 | down |
| RP11-159K7.2 | -5.909613559 | 1.16E-06 | 2.30E-05 | down |
| AADAC | -5.904051698 | 2.41E-09 | 1.12E-07 | down |
| NA | -5.902717182 | 7.67E-07 | 1.62E-05 | down |
| SLC6A4 | -5.89662911 | 5.97E-28 | 1.63E-24 | down |
| LINC00479 | -5.876500764 | 4.09E-12 | 3.91E-10 | down |
| AC007126.1 | -5.795891497 | 1.02E-06 | 2.06E-05 | down |
| CYP4F2 | -5.754337659 | 6.04E-19 | 2.93E-16 | down |

| | | | | |
|---|---|---|---|---|
| EGFR-AS1 | -5.737640697 | 5.00E-13 | 6.03E-11 | down |
| AC008271.1 | -5.736737326 | 1.03E-07 | 2.91E-06 | down |
| RP3-368B9.2 | -5.723269667 | 1.09E-05 | 0.000151623 | down |
| LINC00837 | -5.714273494 | 6.68E-07 | 1.45E-05 | down |
| CTB-127C13.1 | -5.703938174 | 2.86E-07 | 7.01E-06 | down |
| CYB5AP4 | -5.688013012 | 0.020809156 | 0.063925386 | down |
| GATA5 | -5.612801542 | 5.74E-19 | 2.83E-16 | down |
| ALPI | -5.594673658 | 8.95E-22 | 7.68E-19 | down |
| LINC01595 | -5.496639535 | 3.10E-06 | 5.34E-05 | down |
| RP11-515017.2 | -5.487089725 | 5.28E-08 | 1.65E-06 | down |
| ALDOB | -5.438797554 | 2.34E-21 | 1.76E-18 | down |
| RP11-436D23.1 | -5.426307562 | 4.54E-08 | 1.45E-06 | down |
| PKLR | -5.401521745 | 1.57E-17 | 5.36E-15 | down |
| FADS6 | -5.369061472 | 4.97E-14 | 7.82E-12 | down |
| CTD-2291D10.2 | -5.354441579 | 2.80E-06 | 4.93E-05 | down |
| RP11-64D22.5 | -5.345439052 | 2.45E-08 | 8.41E-07 | down |
| REG3G | -5.339323347 | 2.70E-08 | 9.09E-07 | down |
| GUCA2A | -5.330736142 | 5.93E-24 | 8.48E-21 | down |
| CABP2 | -5.325739898 | 0.000145258 | 0.0012847 | down |
| RP11-71N10.1 | -5.32456076 | 7.92E-06 | 0.000116283 | down |
| USH1G | -5.321212303 | 4.39E-11 | 3.31E-09 | down |
| USP29 | -5.244444557 | 0.000385883 | 0.002840141 | down |
| FLT1P1 | -5.232661419 | 9.04E-06 | 0.00013008 | down |
| RNA5SP432 | -5.205795167 | 0.006582892 | 0.026733768 | down |
| RN7SL478P | -5.197779214 | 0.000459223 | 0.003250154 | down |
| FLJ33581 | -5.189770416 | 0.00683631 | 0.027506373 | down |
| RP11-158M9.1 | -5.173722785 | 0.000735501 | 0.004751823 | down |
| PLA2G12B | -5.155208322 | 2.91E-11 | 2.32E-09 | down |
| RP11-1K3.1 | -5.140838785 | 0.001455107 | 0.008140237 | down |
| FGF19 | -5.138387809 | 2.09E-05 | 0.00026029 | down |
| RP1-101G11.3 | -5.124657129 | 9.76E-06 | 0.0001385 | down |
| MGAM | -5.124161238 | 3.16E-17 | 1.02E-14 | down |
| IRX4 | -5.118680044 | 3.46E-05 | 0.000392906 | down |
| SPDYE22P | -5.096334648 | 8.36E-05 | 0.000815799 | down |
| RP11-433J20.1 | -5.089390574 | 1.03E-05 | 0.000144218 | down |
| HNRNPCP9 | -5.085179922 | 0.003375849 | 0.015887219 | down |
| NAT8 | -5.073918139 | 2.95E-07 | 7.16E-06 | down |
| TCP10 | -5.067645736 | 1.18E-12 | 1.30E-10 | down |
| C18orf15 | -5.063104453 | 1.66E-05 | 0.000215188 | down |
| AC113617.1 | -5.056267858 | 0.001914548 | 0.010125098 | down |
| AC006019.3 | -5.055383785 | 0.000926096 | 0.005709755 | down |
| SLC15A1 | -5.054826485 | 3.25E-17 | 1.03E-14 | down |
| OTOP3 | -5.054267463 | 6.92E-14 | 1.05E-11 | down |
| PDZK1 | -5.048616435 | 1.42E-20 | 9.29E-18 | down |
| MCHR2-AS1 | -5.045349336 | 0.000825693 | 0.005211292 | down |
| RP11-339A7.1 | -5.030779069 | 4.36E-06 | 7.10E-05 | down |
| TAAR5 | -5.022851846 | 0.000524753 | 0.00361847 | down |
| RP11-146E23.2 | -5.022599252 | 0.000118463 | 0.001091832 | down |
| RP1-228P16.9 | -5.013440594 | 0.00087684 | 0.005450812 | down |
| LIPF | -5.002179598 | 0.011166386 | 0.039911522 | down |
| CTD-2118P12.1 | -4.996755333 | 0.00015602 | 0.001362636 | down |

| | | | | |
|---|---|---|---|---|
| GAPDHP51 | -4.976325525 | 2.99E-05 | 0.000349574 | down |
| RP11-394A14.2 | -4.970690922 | 0.087804595 | 0.184217964 | down |
| SLC35G4 | -4.969133479 | 0.000292215 | 0.002282377 | down |
| GUSBP6 | -4.963229085 | 7.36E-05 | 0.000730878 | down |
| RP11-646E18.2 | -4.949867462 | 0.00066163 | 0.004358256 | down |
| ABCG8 | -4.947656618 | 5.69E-18 | 2.22E-15 | down |
| RP11-167N24.3 | -4.944858494 | 2.08E-06 | 3.83E-05 | down |
| NANOGP1 | -4.942153159 | 0.001146738 | 0.006763366 | down |
| AC125238.2 | -4.926671054 | 0.000539327 | 0.003701127 | down |
| SLC34A2 | -4.925642303 | 8.16E-13 | 9.32E-11 | down |
| A3GALT2 | -4.910029782 | 4.73E-06 | 7.59E-05 | down |
| RHBG | -4.906529688 | 6.08E-07 | 1.34E-05 | down |
| CCL25 | -4.893154187 | 9.08E-14 | 1.34E-11 | down |
| CYP2T3P | -4.889481009 | 1.32E-07 | 3.59E-06 | down |
| SLC6A18 | -4.870514366 | 6.93E-14 | 1.05E-11 | down |
| SLC34A3 | -4.847642342 | 4.16E-18 | 1.69E-15 | down |
| C3P1 | -4.833560059 | 3.30E-06 | 5.63E-05 | down |
| NA | -4.832201359 | 3.77E-08 | 1.23E-06 | down |
| NA | -4.829002184 | 0.0012096 | 0.007060659 | down |
| PIK3C2G | -4.826618367 | 1.10E-07 | 3.06E-06 | down |
| RPS6P21 | -4.826522756 | 0.000159023 | 0.001383635 | down |
| GRAMD4P3 | -4.825852766 | 0.000752926 | 0.004838694 | down |
| CTC-543D15.1 | -4.824438849 | 0.000176577 | 0.001510124 | down |
| PACRG-AS3 | -4.818886358 | 0.000566849 | 0.003849548 | down |
| WIF1 | -4.811270659 | 1.43E-07 | 3.87E-06 | down |
| AC000367.1 | -4.808068558 | 0.000196939 | 0.001649592 | down |
| NA | -4.798204293 | 5.85E-09 | 2.45E-07 | down |
| AP005901.1 | -4.790478009 | 0.001451635 | 0.008126925 | down |
| SELV | -4.776103184 | 5.56E-05 | 0.000583034 | down |
| MALRD1 | -4.770113376 | 2.34E-12 | 2.36E-10 | down |
| TBX5 | -4.748006777 | 0.001035547 | 0.006239867 | down |
| RBP3 | -4.740049127 | 5.51E-06 | 8.57E-05 | down |
| ANKRD20A7P | -4.738362369 | 3.37E-07 | 8.01E-06 | down |
| CTD-2544M6.1 | -4.732512931 | 0.004290925 | 0.019247022 | down |
| SLC17A8 | -4.732043341 | 2.00E-13 | 2.68E-11 | down |
| RPL39P40 | -4.7270128 | 2.24E-08 | 7.82E-07 | down |
| RN7SL200P | -4.718522143 | 0.006108942 | 0.025301498 | down |
| PCAT5 | -4.717330886 | 8.03E-05 | 0.000788189 | down |
| NAT8B | -4.699869486 | 2.32E-18 | 9.63E-16 | down |
| GSTA1 | -4.693506768 | 6.93E-16 | 1.74E-13 | down |
| UGT1A5 | -4.69318695 | 0.000809027 | 0.005126615 | down |
| AC009276.4 | -4.69085035 | 0.000449624 | 0.003198033 | down |
| RP1-228P16.4 | -4.684087915 | 4.08E-05 | 0.000451818 | down |
| RNU2-47P | -4.683754494 | 0.0584504 | 0.137151187 | down |
| RP11-136I13.1 | -4.681686239 | 4.29E-07 | 9.83E-06 | down |
| RP11-63A11.1 | -4.67553237 | 0.000340484 | 0.002583205 | down |
| PNLIPRP2 | -4.673038294 | 1.05E-12 | 1.17E-10 | down |
| NA | -4.663920202 | 0.000199582 | 0.001667479 | down |
| ZP2 | -4.653808385 | 6.19E-07 | 1.36E-05 | down |
| SNRPGP17 | -4.652655081 | 0.003677012 | 0.017035028 | down |
| RP11-403P17.4 | -4.647100327 | 1.39E-05 | 0.000185869 | down |

| | | | | |
|---|---|---|---|---|
| RP5-1049N15.2 | -4.64675319 | 0.000245808 | 0.001983099 | down |
| ZBBX | -4.646233196 | 0.000136365 | 0.001222242 | down |
| RP11-174M13.2 | -4.643164676 | 0.000605124 | 0.004062647 | down |
| MIR4516 | -4.637697834 | 0.001929175 | 0.010186317 | down |
| RP4-539M6.14 | -4.631497294 | 0.00189544 | 0.010048808 | down |
| A2ML1 | -4.617924099 | 2.34E-08 | 8.10E-07 | down |
| DUX4L32 | -4.614193742 | 0.010501635 | 0.0380784 | down |
| NA | -4.612445907 | 0.005981505 | 0.024893801 | down |
| TPM3P2 | -4.609704598 | 0.00643861 | 0.026318771 | down |
| RP11-358L22.2 | -4.604197262 | 0.000825265 | 0.00520997 | down |
| NA | -4.598892535 | 0.000479929 | 0.003362959 | down |
| CTA-212A2.3 | -4.595791907 | 2.11E-06 | 3.87E-05 | down |
| RP11-403I13.4 | -4.595277744 | 0.000372029 | 0.002758448 | down |
| LINC00658 | -4.593633523 | 5.10E-06 | 8.08E-05 | down |
| SMYD1 | -4.592038142 | 0.0006346 | 0.004215325 | down |
| ZIM3 | -4.589756642 | 0.006219116 | 0.025655241 | down |
| NA | -4.586756761 | 5.01E-05 | 0.000536372 | down |
| RP11-343J18.1 | -4.586604841 | 0.001640599 | 0.008963476 | down |
| KRT8P23 | -4.585055518 | 0.004861214 | 0.021225158 | down |
| DPY19L2P1 | -4.58033623 | 0.000479975 | 0.003362959 | down |
| LINC01620 | -4.579795546 | 5.97E-05 | 0.00061746 | down |
| NUS1P3 | -4.578763567 | 0.000273629 | 0.00216625 | down |
| RP11-449L23.2 | -4.572500597 | 1.64E-06 | 3.12E-05 | down |
| ATP5G1P7 | -4.571994589 | 0.011672627 | 0.041330868 | down |
| NA | -4.56774154 | 0.000206032 | 0.001713267 | down |
| RN7SKP213 | -4.566925263 | 0.013670296 | 0.046492005 | down |
| SERPINB13 | -4.554547735 | 0.004492501 | 0.019975239 | down |
| RP11-139I14.2 | -4.549273009 | 0.044183083 | 0.111755896 | down |
| CTD-2085J24.5 | -4.54301202 | 0.003048474 | 0.01467762 | down |
| AC079776.3 | -4.541675453 | 6.49E-05 | 0.000660485 | down |
| RP11-666A20.4 | -4.540785 | 2.30E-18 | 9.63E-16 | down |
| OR52A1 | -4.540358969 | 0.007330339 | 0.028974176 | down |
| AC151960.1 | -4.538622631 | 0.008915617 | 0.033595985 | down |
| RP11-168E17.1 | -4.53073689 | 0.000238554 | 0.001935063 | down |
| RP11-697E22.2 | -4.524091912 | 5.23E-08 | 1.64E-06 | down |
| RP11-766F14.1 | -4.522681351 | 0.00027038 | 0.002146746 | down |
| RP11-640N11.2 | -4.522400397 | 0.001126667 | 0.006675133 | down |
| RP11-13K12.2 | -4.521711251 | 0.000101813 | 0.000959794 | down |
| CFHR4 | -4.519832042 | 0.002278258 | 0.011680542 | down |
| GBA3 | -4.514631555 | 1.02E-18 | 4.56E-16 | down |
| EIF3KP3 | -4.513356559 | 0.000897653 | 0.005553764 | down |
| RP11-156L14.1 | -4.507722709 | 3.06E-05 | 0.000355422 | down |
| ENPEP | -4.501223521 | 2.36E-16 | 6.40E-14 | down |
| EXD1 | -4.496590071 | 0.000560342 | 0.003814843 | down |
| TREH | -4.493123471 | 9.94E-23 | 1.15E-19 | down |
| RP11-158D2.1 | -4.482169658 | 0.007457207 | 0.029367456 | down |
| NA | -4.479762975 | 0.000392048 | 0.002868497 | down |
| KCNH6 | -4.479634014 | 2.19E-12 | 2.23E-10 | down |
| SEC14L4 | -4.469782413 | 7.89E-07 | 1.66E-05 | down |
| FAHD2P1 | -4.46353856 | 7.71E-10 | 4.09E-08 | down |
| ZIC1 | -4.46311842 | 1.24E-06 | 2.45E-05 | down |

| | | | | |
|---|---|---|---|---|
| NA | -4.448380363 | 0.003099448 | 0.014867661 | down |
| CYP4F8 | -4.447148897 | 0.000114948 | 0.001064905 | down |
| DDR1-AS1 | -4.447065834 | 0.001408686 | 0.007935976 | down |
| ACOT12 | -4.44167422 | 5.39E-06 | 8.43E-05 | down |
| KIAA1210 | -4.431103264 | 0.000277813 | 0.002191864 | down |
| UGT1A1 | -4.430648455 | 5.95E-08 | 1.83E-06 | down |
| RP11-40H20.4 | -4.427048539 | 0.002057803 | 0.010735308 | down |
| RP11-114G22.1 | -4.422942675 | 0.001613785 | 0.008850778 | down |
| AQP8 | -4.421703983 | 5.64E-09 | 2.37E-07 | down |
| RP11-168K9.2 | -4.420801619 | 0.000829936 | 0.005231768 | down |
| PSMC1P12 | -4.419259793 | 0.001094066 | 0.006519991 | down |
| RP11-15H20.2 | -4.413980051 | 0.02275318 | 0.068270902 | down |
| FGF14-IT1 | -4.409781759 | 2.41E-05 | 0.000291538 | down |
| ARL2BPP6 | -4.409583404 | 0.000756869 | 0.004857802 | down |
| NA | -4.40919806 | 0.000368196 | 0.002740175 | down |
| RPS3AP54 | -4.399129721 | 0.000298913 | 0.002323844 | down |
| BSND | -4.39792176 | 0.000215236 | 0.001774572 | down |
| RP11-116O18.1 | -4.397190962 | 1.81E-13 | 2.47E-11 | down |
| OR9M1P | -4.396970139 | 0.003287723 | 0.015548647 | down |
| RP4-614C10.3 | -4.391121234 | 0.004267997 | 0.019175671 | down |
| LRRC74B | -4.389738463 | 1.66E-06 | 3.16E-05 | down |
| GUCA2B | -4.386191738 | 6.51E-16 | 1.64E-13 | down |
| NA | -4.385909094 | 0.000825243 | 0.00520997 | down |
| RP11-133K1.9 | -4.381362743 | 0.002667079 | 0.013218366 | down |
| AC004906.3 | -4.375772733 | 2.91E-06 | 5.07E-05 | down |
| RBM46 | -4.371670762 | 0.00029774 | 0.002317435 | down |
| RP1-102K2.6 | -4.371402175 | 1.53E-05 | 0.000201404 | down |
| RP11-271K21.12 | -4.370706843 | 0.001266685 | 0.007310169 | down |
| MIR4500HG | -4.370472599 | 1.98E-10 | 1.23E-08 | down |
| GIP | -4.370467948 | 0.000473309 | 0.003327227 | down |
| DCDC1 | -4.369877601 | 3.89E-05 | 0.000434287 | down |
| XXyac-YM21GA2.7 | -4.361803909 | 1.37E-12 | 1.47E-10 | down |
| RP1-232P20.1 | -4.354118053 | 0.001588419 | 0.00873558 | down |
| CYP1A2 | -4.337529826 | 0.000677291 | 0.004440001 | down |
| RP11-23E19.2 | -4.336433957 | 0.01221581 | 0.042755336 | down |
| LYZL1 | -4.335773556 | 0.0247954 | 0.072848914 | down |
| CHRND | -4.333719871 | 3.83E-05 | 0.000429766 | down |
| CTD-2201G3.1 | -4.316899257 | 3.75E-05 | 0.000422043 | down |
| RNA5SP371 | -4.314736961 | 0.005724189 | 0.02405953 | down |
| RP11-675F6.3 | -4.313886512 | 2.42E-07 | 6.10E-06 | down |
| CTC-523E23.10 | -4.313416172 | 0.001462279 | 0.00817353 | down |
| RP5-930J4.5 | -4.306806169 | 0.013627839 | 0.046408781 | down |
| RP11-485F13.1 | -4.297644355 | 0.010182716 | 0.037186035 | down |
| LINC00330 | -4.295470867 | 7.35E-08 | 2.21E-06 | down |
| LINC01416 | -4.290377881 | 0.001398317 | 0.00789023 | down |
| RP11-109I13.2 | -4.281156388 | 0.000207433 | 0.001722534 | down |
| OTOP2 | -4.275716933 | 1.13E-11 | 9.82E-10 | down |
| AC008132.13 | -4.271359784 | 0.003691611 | 0.017092118 | down |
| RP11-346D14.1 | -4.269656333 | 9.22E-05 | 0.00088307 | down |
| OR4K2 | -4.268822741 | 0.000151254 | 0.001328348 | down |
| RP11-793H13.12 | -4.267259185 | 0.000840825 | 0.005280464 | down |

| | | | | |
|---|---|---|---|---|
| HNRNPA1P57 | -4.266477263 | 0.013206829 | 0.045264199 | down |
| VAX1 | -4.26399437 | 0.000932814 | 0.00573822 | down |
| RP11-286E11.2 | -4.259554978 | 5.32E-07 | 1.19E-05 | down |
| HTR3C | -4.254324348 | 6.26E-11 | 4.53E-09 | down |
| NA | -4.239184182 | 0.001929906 | 0.010186597 | down |
| DPEP1 | -4.22963198 | 1.39E-13 | 1.98E-11 | down |
| NA | -4.229315971 | 3.94E-05 | 0.000439248 | down |
| BEST3 | -4.227497496 | 5.41E-05 | 0.000569176 | down |
| FAM3B | -4.225673969 | 7.85E-22 | 6.94E-19 | down |
| NA | -4.222256276 | 1.62E-05 | 0.000211405 | down |
| AP001626.2 | -4.221110406 | 7.11E-10 | 3.84E-08 | down |
| SGCZ | -4.218195113 | 0.007476899 | 0.029418259 | down |
| NA | -4.216059012 | 0.001312452 | 0.007513588 | down |
| RP11-805F19.3 | -4.215581166 | 0.000582812 | 0.003937487 | down |
| SERPINB10 | -4.215358591 | 8.48E-06 | 0.000123237 | down |
| FAM151A | -4.215202794 | 2.69E-21 | 1.97E-18 | down |
| RP11-10D7.5 | -4.212705514 | 0.005765445 | 0.024175439 | down |
| Metazoa_SRP | -4.210964127 | 3.39E-05 | 0.000386544 | down |
| OR9H1P | -4.208517449 | 0.013052403 | 0.044883415 | down |
| RP13-122B23.9 | -4.206073296 | 1.75E-05 | 0.000224397 | down |
| MYO16-AS1 | -4.198723705 | 0.004492351 | 0.019975239 | down |
| RP11-294N21.2 | -4.19856848 | 0.00063793 | 0.00423447 | down |
| RP11-451N19.3 | -4.195026793 | 0.003420485 | 0.016077136 | down |
| CTD-2620I22.3 | -4.194896681 | 0.000946309 | 0.005796311 | down |
| RP11-704C2.1 | -4.193036479 | 0.045904384 | 0.114843143 | down |
| SSX3 | -4.191555724 | 0.00675424 | 0.02727112 | down |
| RP11-397A16.1 | -4.189058642 | 0.007737158 | 0.030204979 | down |
| CEACAM18 | -4.18618026 | 1.23E-05 | 0.000167524 | down |
| LINC00898 | -4.185909943 | 0.018539733 | 0.058702419 | down |
| MS4A18 | -4.183882967 | 0.000333674 | 0.002543103 | down |
| RP11-98D18.7 | -4.183298131 | 0.002433276 | 0.012300632 | down |
| RP11-421P23.2 | -4.182337394 | 0.029504317 | 0.082859198 | down |
| RP11-814E24.1 | -4.182090415 | 0.009283862 | 0.034648986 | down |
| GOLGA8EP | -4.180335776 | 0.003131741 | 0.014978766 | down |
| MORC1 | -4.174956685 | 0.000159706 | 0.00138837 | down |
| OTOG | -4.171895874 | 6.55E-05 | 0.000664832 | down |
| CNN2P10 | -4.171697286 | 0.065181047 | 0.148254931 | down |
| C1orf105 | -4.165061667 | 0.000157552 | 0.001372819 | down |
| RP11-503G7.1 | -4.164840498 | 0.000641355 | 0.004249858 | down |
| NA | -4.164703242 | 5.78E-06 | 8.95E-05 | down |
| RP3-333H23.8 | -4.163047648 | 0.000207159 | 0.001720731 | down |
| CTD-2010I22.2 | -4.158810336 | 0.000917933 | 0.005668733 | down |
| AP000472.2 | -4.153566872 | 1.47E-07 | 3.95E-06 | down |
| NDUFB4P3 | -4.151975303 | 0.013102946 | 0.045005706 | down |
| RP11-67K19.3 | -4.14990408 | 0.008767848 | 0.033163947 | down |
| RP4-701O16.5 | -4.149077551 | 0.034456684 | 0.093069912 | down |
| CTD-2384A14.1 | -4.149018531 | 0.001694966 | 0.00919696 | down |
| MRE11B | -4.146532095 | 0.000850953 | 0.005331813 | down |
| CNTNAP5 | -4.142212931 | 0.004352602 | 0.019456863 | down |
| RP11-298J20.3 | -4.141212248 | 3.32E-05 | 0.000380024 | down |
| SOCS5P2 | -4.141037897 | 5.45E-08 | 1.70E-06 | down |

| | | | | |
|---|---|---|---|---|
| UNC93A | -4.134608905 | 9.85E-07 | 2.00E-05 | down |
| RP11-570J4.1 | -4.134053926 | 0.001436749 | 0.008063378 | down |
| SULT1E1 | -4.133449059 | 2.20E-05 | 0.00027023 | down |
| RP11-762H8.3 | -4.132715088 | 0.005052948 | 0.021874751 | down |
| MIR141 | -4.131126138 | 0.001007005 | 0.006098462 | down |
| RP11-794P6.2 | -4.130816113 | 0.015464678 | 0.051147159 | down |
| OR6W1P | -4.121935224 | 0.000632368 | 0.004207398 | down |
| Y_RNA | -4.120449932 | 0.000397795 | 0.002905701 | down |
| NA | -4.117667469 | 0.030663833 | 0.085182465 | down |
| NA | -4.116568916 | 1.41E-05 | 0.000188281 | down |
| EDN2 | -4.115434597 | 7.95E-15 | 1.51E-12 | down |
| SLC22A6 | -4.108817572 | 0.02318776 | 0.069208531 | down |
| RP11-179H18.5 | -4.108162487 | 0.000857776 | 0.005360028 | down |
| P2RY4 | -4.106922552 | 8.17E-06 | 0.000119573 | down |
| RP11-327O17.2 | -4.101657148 | 0.002396804 | 0.012159671 | down |
| CH17-360D5.1 | -4.101556927 | 1.20E-06 | 2.38E-05 | down |
| RP11-177B4.2 | -4.098948316 | 0.000872739 | 0.005429811 | down |
| RP11-329B9.1 | -4.097377323 | 4.61E-05 | 0.000499528 | down |
| NA | -4.097356033 | 0.001563801 | 0.008620706 | down |
| Metazoa_SRP | -4.094886448 | 0.00038773 | 0.002846263 | down |
| RP11-319E16.2 | -4.094156245 | 0.0143813 | 0.048368047 | down |
| RP11-467K18.2 | -4.093329899 | 0.0003379 | 0.002567491 | down |
| SLC5A11 | -4.092535858 | 2.10E-17 | 6.93E-15 | down |
| CTD-2013N17.7 | -4.091640216 | 0.001148802 | 0.006774212 | down |
| LINC01309 | -4.090237161 | 0.000139821 | 0.001248374 | down |
| AC096775.2 | -4.088807806 | 0.001472105 | 0.008213172 | down |
| AC007326.10 | -4.087769778 | 0.000249958 | 0.002010638 | down |
| GS1-594A7.3 | -4.083544112 | 0.001395919 | 0.007881785 | down |
| RP11-2K6.2 | -4.078345499 | 0.02672402 | 0.077023835 | down |
| AGXT2 | -4.077990607 | 2.65E-05 | 0.000316732 | down |
| RP11-252A24.8 | -4.077526074 | 0.004474038 | 0.01991673 | down |
| Y_RNA | -4.075976858 | 0.023012507 | 0.068781114 | down |
| RP11-67O4.5 | -4.075297569 | 0.020488911 | 0.063234415 | down |
| Y_RNA | -4.073632983 | 0.000641689 | 0.004251135 | down |
| RP11-115J23.1 | -4.073279505 | 0.004165722 | 0.018834453 | down |
| RP11-47P18.1 | -4.071636271 | 0.001423785 | 0.008004529 | down |
| RPL7P61 | -4.070116373 | 0.010905838 | 0.039202559 | down |
| AC027612.3 | -4.06827288 | 0.009466104 | 0.035150529 | down |
| BOD1L2 | -4.067796018 | 0.104557369 | 0.208393365 | down |
| RP11-333A23.2 | -4.067263172 | 0.073977195 | 0.162706504 | down |
| RP11-329N22.2 | -4.06493459 | 0.038636597 | 0.101220607 | down |
| FLJ22763 | -4.063984631 | 1.13E-18 | 5.00E-16 | down |
| RP11-848P1.4 | -4.063837679 | 1.52E-09 | 7.36E-08 | down |
| CTA-243E7.4 | -4.061806434 | 0.000210834 | 0.001746426 | down |
| RP11-334E15.1 | -4.055765865 | 0.009301046 | 0.034691575 | down |
| CPS1 | -4.055366798 | 1.20E-12 | 1.31E-10 | down |
| RPS4XP8 | -4.054575576 | 1.67E-05 | 0.000216319 | down |
| SEPT14 | -4.054008859 | 0.040214573 | 0.104349898 | down |
| NA | -4.05345483 | 0.014432143 | 0.048490193 | down |
| NA | -4.051562014 | 0.003481501 | 0.016307799 | down |
| RP4-550H1.4 | -4.046059213 | 9.44E-05 | 0.00090098 | down |

| | | | | |
|---|---|---|---|---|
| RPS23P7 | -4.043503089 | 0.000912356 | 0.005636609 | down |
| CTC-513N18.7 | -4.040518365 | 0.02455771 | 0.072334493 | down |
| RP11-65J3.3 | -4.040250653 | 0.053980669 | 0.129249282 | down |
| DAB1 | -4.037087806 | 3.27E-11 | 2.58E-09 | down |
| CTD-3022L24.1 | -4.031079116 | 0.000455435 | 0.003230151 | down |
| ABCG5 | -4.03017275 | 1.81E-13 | 2.47E-11 | down |
| NTS | -4.028769379 | 2.17E-08 | 7.62E-07 | down |
| CENPUP1 | -4.025001402 | 0.076723988 | 0.167125663 | down |
| SLC13A2 | -4.02301355 | 3.19E-12 | 3.12E-10 | down |
| C1QTNF8 | -4.021936479 | 0.000490963 | 0.003427612 | down |
| PANCR | -4.02090326 | 8.49E-06 | 0.000123317 | down |
| AJ003147.8 | -4.020839452 | 0.004337526 | 0.019399936 | down |
| TRPC5OS | -4.019237783 | 0.009016586 | 0.033887205 | down |
| RP11-25P11.2 | -4.016753023 | 0.025250245 | 0.073845845 | down |
| AC009502.4 | -4.013957149 | 0.000307501 | 0.002379231 | down |
| XPNPEP2 | -4.011683212 | 1.15E-19 | 6.30E-17 | down |
| SYNJ2-IT1 | -4.008669816 | 0.002436998 | 0.012311612 | down |
| RP11-103J17.1 | -4.007797664 | 0.02058131 | 0.063400481 | down |
| Z69666.2 | -4.004842659 | 3.30E-06 | 5.64E-05 | down |
| NA | -4.002430669 | 0.01035466 | 0.037664 | down |
| RP11-255I10.2 | -3.998353155 | 0.010465011 | 0.037985149 | down |
| NA | -3.995566781 | 0.000787155 | 0.005010444 | down |
| KRT32 | -3.99552565 | 0.054013354 | 0.129304957 | down |
| RP11-319E12.2 | -3.995161717 | 0.015696416 | 0.051703848 | down |
| RN7SKP204 | -3.993790728 | 0.040333239 | 0.104580291 | down |
| CELA3B | -3.991720684 | 1.77E-08 | 6.43E-07 | down |
| F11 | -3.990169332 | 1.83E-06 | 3.44E-05 | down |
| LINC01435 | -3.98816908 | 0.000613016 | 0.004107374 | down |
| RP11-618M23.1 | -3.986801086 | 0.010705165 | 0.038638391 | down |
| BPIFB5P | -3.985651265 | 0.057666821 | 0.135768511 | down |
| NA | -3.985120276 | 6.31E-11 | 4.54E-09 | down |
| BSPH1 | -3.985099 | 0.038809216 | 0.101542717 | down |
| NA | -3.98437998 | 0.00454699 | 0.020166781 | down |
| RPS4XP5 | -3.98036239 | 0.005140162 | 0.022159712 | down |
| RP5-908M14.5 | -3.979497702 | 1.08E-07 | 3.02E-06 | down |
| ZIC4 | -3.979244829 | 0.001309728 | 0.00750228 | down |
| SLC14A2 | -3.978712713 | 2.62E-20 | 1.55E-17 | down |
| RP11-121L11.3 | -3.978697972 | 0.043436078 | 0.110516886 | down |
| CTB-180A7.8 | -3.978626938 | 0.003735212 | 0.01726207 | down |
| RP11-22903.1 | -3.977682375 | 3.21E-05 | 0.000369855 | down |
| AF121898.3 | -3.977624979 | 0.000352294 | 0.002652715 | down |
| ADGRG4 | -3.965179759 | 3.89E-07 | 9.03E-06 | down |
| RP11-326L17.1 | -3.964722438 | 8.29E-07 | 1.73E-05 | down |
| RP11-378I6.1 | -3.962990873 | 0.039983239 | 0.103914916 | down |
| AC007461.2 | -3.962566247 | 0.011989875 | 0.042180774 | down |
| XRCC6P3 | -3.962244643 | 0.01406231 | 0.04753185 | down |
| AC072062.3 | -3.962119715 | 0.001738979 | 0.00939553 | down |
| LINC01297 | -3.961728967 | 0.000323582 | 0.002478127 | down |
| RP11-742B18.1 | -3.960013669 | 0.045372659 | 0.113856903 | down |
| RP11-297H3.3 | -3.958064813 | 0.026607628 | 0.076798884 | down |
| NDUFA5P6 | -3.957568815 | 0.018376236 | 0.058305591 | down |

| | | | | |
|---|---|---|---|---|
| HMGN1P32 | -3.956646321 | 0.056107625 | 0.133014872 | down |
| RP4-695O20.1 | -3.953918502 | 3.60E-07 | 8.44E-06 | down |
| MORN5 | -3.953585085 | 1.32E-05 | 0.000177635 | down |
| RP11-313E4.1 | -3.95222724 | 0.01099609 | 0.039460887 | down |
| SLC51A | -3.951630234 | 8.44E-41 | 6.34E-37 | down |
| RP11-166A12.1 | -3.951314972 | 6.85E-05 | 0.000689063 | down |
| RP11-589N15.1 | -3.950923495 | 0.033966182 | 0.092051232 | down |
| GGT3P | -3.94838523 | 5.31E-05 | 0.000560533 | down |
| RP5-1041C10.3 | -3.943322931 | 6.73E-07 | 1.46E-05 | down |
| NA | -3.942804692 | 0.008032079 | 0.031058658 | down |
| AC003989.4 | -3.939785376 | 0.004707757 | 0.020714999 | down |
| NPM1P40 | -3.938815161 | 0.000106365 | 0.000995833 | down |
| EFTUD1P1 | -3.938476269 | 0.003564101 | 0.016622143 | down |
| PRODH | -3.937018122 | 1.82E-14 | 3.20E-12 | down |
| RP5-896L10.1 | -3.935645437 | 0.060225411 | 0.140101606 | down |
| RP11-125D12.1 | -3.932675361 | 0.058639529 | 0.137412527 | down |
| NA | -3.93233554 | 0.000623662 | 0.004161069 | down |
| NA | -3.930688135 | 0.000158794 | 0.001382042 | down |
| RP11-718B12.5 | -3.928345795 | 0.001191678 | 0.006979101 | down |
| PNLIP | -3.927548513 | 0.008516469 | 0.032421285 | down |
| AC002463.3 | -3.926592979 | 4.35E-06 | 7.08E-05 | down |
| RP11-213G2.5 | -3.923300301 | 0.001136417 | 0.006722289 | down |
| NA | -3.922350222 | 0.006457199 | 0.02637304 | down |
| MAGEB16 | -3.921946014 | 0.041910486 | 0.107647998 | down |
| RP1-215K18.4 | -3.921861268 | 0.029135612 | 0.082169372 | down |
| ANPEP | -3.919894516 | 5.41E-20 | 3.06E-17 | down |
| RP1-149M18.4 | -3.919630813 | 0.015317123 | 0.050754124 | down |
| CD24P2 | -3.917747603 | 0.027386758 | 0.078493724 | down |
| RP11-96O20.2 | -3.917038565 | 0.008305524 | 0.03182519 | down |
| CUBN | -3.916209279 | 3.37E-19 | 1.72E-16 | down |
| AC092066.6 | -3.91216787 | 0.002539804 | 0.012723702 | down |
| RP11-310P5.2 | -3.908767877 | 4.61E-09 | 1.99E-07 | down |
| WDR95P | -3.908083846 | 0.005480246 | 0.023258726 | down |
| GSTA3 | -3.90751513 | 0.030477686 | 0.084792257 | down |
| FAM99A | -3.902182123 | 0.063367002 | 0.145494705 | down |
| RP5-892K4.1 | -3.901062362 | 4.69E-09 | 2.02E-07 | down |
| AC018880.1 | -3.897692165 | 0.063202898 | 0.1452289 | down |
| RP11-278L15.6 | -3.895007372 | 0.018923975 | 0.059538007 | down |
| ITIH6 | -3.894795535 | 0.000583777 | 0.003942237 | down |
| RP11-29B9.2 | -3.893933289 | 0.009354134 | 0.034850644 | down |
| CTD-2572N17.1 | -3.893385072 | 0.122722129 | 0.233654224 | down |
| NA | -3.888961001 | 6.75E-05 | 0.000682293 | down |
| HOXC11 | -3.888521158 | 9.46E-06 | 0.000134932 | down |
| RPL6P21 | -3.885456885 | 0.020379033 | 0.062979803 | down |
| AC002115.5 | -3.881808561 | 0.010614132 | 0.038402094 | down |
| ODF4 | -3.881069609 | 0.013893483 | 0.047107075 | down |
| RP11-299H22.3 | -3.877947448 | 0.002665576 | 0.013215273 | down |
| CYP2F1 | -3.877661338 | 7.70E-05 | 0.000760227 | down |
| CYP2A7 | -3.876684505 | 1.06E-06 | 2.14E-05 | down |
| RP5-898I4.1 | -3.873449933 | 0.046195064 | 0.115332029 | down |
| ITLN2 | -3.87244493 | 8.39E-06 | 0.000122107 | down |

| | | | | |
|---|---|---|---|---|
| NF1P7 | -3.871646488 | 0.020939361 | 0.064239053 | down |
| NA | -3.866786796 | 0.003016895 | 0.014569941 | down |
| TOPAZ1 | -3.864834033 | 0.002042019 | 0.010672656 | down |
| SOAT2 | -3.864412856 | 4.99E-15 | 9.68E-13 | down |
| RP5-1027G4.3 | -3.860306032 | 1.02E-06 | 2.06E-05 | down |
| AC093802.1 | -3.86026862 | 0.005267085 | 0.022580522 | down |
| LINC01565 | -3.859999945 | 0.170119269 | 0.293891284 | down |
| BTBD9-AS1 | -3.859938689 | 0.002762689 | 0.013600236 | down |
| NA | -3.855942107 | 1.53E-05 | 0.000201429 | down |
| LINC01586 | -3.854204102 | 0.001545065 | 0.008537378 | down |
| RS1 | -3.851798531 | 0.025399681 | 0.074150527 | down |
| THAP12P3 | -3.8505593 | 0.000934943 | 0.005744787 | down |
| AC079779.4 | -3.849300911 | 2.89E-06 | 5.05E-05 | down |
| CTA-243E7.1 | -3.84692061 | 4.92E-11 | 3.66E-09 | down |
| KHK | -3.844827508 | 2.47E-22 | 2.39E-19 | down |
| PABPC1P2 | -3.842067565 | 0.048321574 | 0.119399077 | down |
| LINC01606 | -3.839792379 | 0.000438979 | 0.003134192 | down |
| LCN15 | -3.839491375 | 1.49E-12 | 1.58E-10 | down |
| TMPRSS7 | -3.83614734 | 2.46E-07 | 6.17E-06 | down |
| LYPD8 | -3.835830215 | 2.04E-13 | 2.71E-11 | down |
| RP11-400N13.1 | -3.835793637 | 0.005828246 | 0.024397915 | down |
| CTD-2119L1.1 | -3.833848875 | 0.006574831 | 0.026711862 | down |
| DLX6 | -3.832674887 | 0.000144146 | 0.001277879 | down |
| Y_RNA | -3.830306677 | 0.002077241 | 0.010814179 | down |
| NA | -3.828635359 | 0.070690754 | 0.157481252 | down |
| RP5-945F2.3 | -3.828532853 | 0.012738614 | 0.04410451 | down |
| AC020956.3 | -3.828204101 | 0.00283207 | 0.013860003 | down |
| LPO | -3.826285277 | 4.00E-05 | 0.000444266 | down |
| RP11-667M19.2 | -3.823964508 | 0.000190468 | 0.001606481 | down |
| AC004461.4 | -3.822889901 | 2.86E-05 | 0.000336822 | down |
| RP11-849N15.5 | -3.821358322 | 0.005754485 | 0.024146333 | down |
| RP11-686G8.1 | -3.820709371 | 0.000222176 | 0.00182329 | down |
| RP11-255M2.2 | -3.820445001 | 0.004031755 | 0.01835582 | down |
| AC087430.1 | -3.81930676 | 0.00332719 | 0.015702499 | down |
| TEDDM1 | -3.818400561 | 6.40E-05 | 0.000652954 | down |
| TNNI3 | -3.818321201 | 1.40E-06 | 2.73E-05 | down |
| CELP | -3.815805919 | 1.99E-14 | 3.41E-12 | down |
| TMED7-TICAM2 | -3.815239849 | 0.009776404 | 0.035995377 | down |
| Y_RNA | -3.814875688 | 0.004846789 | 0.021190556 | down |
| AC002550.5 | -3.814396034 | 0.047356633 | 0.117546487 | down |
| RP11-802H3.1 | -3.813837979 | 0.004758764 | 0.02089322 | down |
| ABCC11 | -3.813358821 | 5.91E-08 | 1.83E-06 | down |
| NA | -3.812916655 | 0.014637778 | 0.049005729 | down |
| MLIP-AS1 | -3.812762347 | 0.022178208 | 0.067025563 | down |
| NA | -3.812321458 | 0.038493381 | 0.100945042 | down |
| DGKK | -3.810137671 | 0.004715461 | 0.020739467 | down |
| TUBA3C | -3.809274673 | 0.029066241 | 0.082027629 | down |
| OR7E55P | -3.805880243 | 0.004724264 | 0.020769064 | down |
| RP11-805I24.1 | -3.80549305 | 0.00018588 | 0.001576229 | down |
| AC018737.3 | -3.805487471 | 0.002645933 | 0.013141745 | down |
| RP11-260O18.1 | -3.805328172 | 0.048673086 | 0.120060279 | down |

| | | | | |
|---|---|---|---|---|
| AC007731.1 | -3.805148971 | 0.070694488 | 0.157481252 | down |
| OR7E25P | -3.804488702 | 0.013676737 | 0.046508644 | down |
| RP1-232N11.2 | -3.802936509 | 0.013818133 | 0.046893934 | down |
| RGPD4-AS1 | -3.801021293 | 0.050617507 | 0.123347585 | down |
| VENTXP7 | -3.800087871 | 0.003615494 | 0.016807192 | down |
| NA | -3.799135228 | 0.008219131 | 0.03157744 | down |
| RPS26P45 | -3.798688129 | 5.88E-09 | 2.46E-07 | down |
| NA | -3.797670075 | 3.70E-06 | 6.18E-05 | down |
| RP11-544M22.1 | -3.796827842 | 0.000125524 | 0.001142454 | down |
| RP11-3B12.3 | -3.794938832 | 0.009576977 | 0.035451447 | down |
| CYP2G2P | -3.794717716 | 0.099480301 | 0.201224411 | down |
| MT-TF | -3.791461809 | 1.13E-10 | 7.57E-09 | down |
| RP11-44M6.3 | -3.790035044 | 5.19E-12 | 4.88E-10 | down |
| UGT1A4 | -3.788002289 | 0.007401252 | 0.029196851 | down |
| KRT83 | -3.787353131 | 0.000151953 | 0.001334094 | down |
| GPR1-AS | -3.786677251 | 0.008936684 | 0.033645831 | down |
| CCDC140 | -3.786463963 | 0.134433753 | 0.248932368 | down |
| TMPRSS15 | -3.786410636 | 2.47E-05 | 0.000297635 | down |
| GSG1L | -3.786264973 | 1.60E-13 | 2.23E-11 | down |
| SNORA57 | -3.783801784 | 0.006955298 | 0.027862018 | down |
| TATDN2P1 | -3.781204569 | 0.007311466 | 0.028907185 | down |
| RP11-213G6.2 | -3.78119527 | 0.073046272 | 0.161272941 | down |
| ANKFN1 | -3.781156169 | 9.99E-10 | 5.07E-08 | down |
| RP5-1028L10.1 | -3.777051786 | 0.180674338 | 0.306037087 | down |
| RP11-314P15.2 | -3.77662004 | 0.011676517 | 0.04133977 | down |
| SLC30A10 | -3.776412665 | 3.34E-14 | 5.40E-12 | down |
| RP11-20F18.1 | -3.775120897 | 0.00047525 | 0.003339195 | down |
| snoZ6 | -3.775117898 | 7.02E-06 | 0.000105322 | down |
| CTD-2545H1.2 | -3.774903752 | 0.001756458 | 0.009470753 | down |
| Y_RNA | -3.77393607 | 0.0086268 | 0.0327252 | down |
| SI | -3.772659126 | 5.91E-13 | 7.02E-11 | down |
| RP11-98J9.2 | -3.772427172 | 0.002937646 | 0.014265173 | down |
| RP3-417L20.4 | -3.771470141 | 2.92E-06 | 5.10E-05 | down |
| RN7SL619P | -3.76992854 | 0.000105262 | 0.000987972 | down |
| KCNMA1-AS1 | -3.769928288 | 1.79E-06 | 3.36E-05 | down |
| PTCSC2 | -3.769246708 | 0.000274591 | 0.00217167 | down |
| GGT4P | -3.76893284 | 0.00014627 | 0.00129137 | down |
| TMIGD1 | -3.76801468 | 1.82E-13 | 2.47E-11 | down |
| C16orf78 | -3.767930254 | 0.034079287 | 0.092249581 | down |
| SEPHS1P6 | -3.766027237 | 0.000282527 | 0.002222189 | down |
| FAM9B | -3.765184898 | 0.008527558 | 0.032455275 | down |
| NHLH2 | -3.762246972 | 6.09E-06 | 9.36E-05 | down |
| RP11-1028N23.4 | -3.760165727 | 0.000402605 | 0.002928465 | down |
| RP11-368L12.1 | -3.760018804 | 0.001975379 | 0.010377388 | down |
| PRDX2P2 | -3.759259109 | 0.054706503 | 0.130641617 | down |
| NA | -3.756659149 | 0.136464392 | 0.251699686 | down |
| RP11-33504.3 | -3.755847413 | 2.28E-09 | 1.07E-07 | down |
| AP000432.3 | -3.755006771 | 0.00987644 | 0.036283657 | down |
| RPSAP76 | -3.754109995 | 0.013468323 | 0.04597163 | down |
| RP11-71L14.4 | -3.754084596 | 0.000245682 | 0.001983099 | down |
| EPB42 | -3.753697386 | 0.018830065 | 0.059325763 | down |

| | | | | |
|---|---|---|---|---|
| ASNSP1 | -3.7526285 | 0.005496772 | 0.023295953 | down |
| SAG | -3.747776071 | 3.16E-08 | 1.05E-06 | down |
| RP11-958J22.1 | -3.747521755 | 0.041769507 | 0.107382079 | down |
| RP11-242P2.1 | -3.745266844 | 0.074625271 | 0.163736336 | down |
| MS4A15 | -3.742136967 | 6.33E-08 | 1.93E-06 | down |
| RP11-497D6.5 | -3.742037013 | 0.047686031 | 0.118137948 | down |
| LINC00534 | -3.741993702 | 0.000134836 | 0.001211428 | down |
| OR10K2 | -3.741813789 | 0.021816872 | 0.066195325 | down |
| NA | -3.740291241 | 0.007217237 | 0.028621259 | down |
| SLC52A1 | -3.740256908 | 4.47E-26 | 1.03E-22 | down |
| NA | -3.737980213 | 0.026914499 | 0.077453948 | down |
| RP11-680B3.2 | -3.737895248 | 0.000125577 | 0.001142595 | down |
| MIR3616 | -3.734911833 | 0.035301711 | 0.094561752 | down |
| AC011298.2 | -3.734005934 | 2.75E-16 | 7.31E-14 | down |
| RP11-614F17.2 | -3.733731889 | 0.014414157 | 0.048451435 | down |
| DMRT1 | -3.731039542 | 0.001171175 | 0.006875106 | down |
| TMEM229A | -3.730260422 | 1.78E-07 | 4.68E-06 | down |
| LINC01087 | -3.729736695 | 0.073146616 | 0.161458888 | down |
| LINC01206 | -3.728499921 | 5.21E-06 | 8.19E-05 | down |
| AC005281.1 | -3.727995005 | 2.70E-05 | 0.000320818 | down |
| SMIM24 | -3.724484687 | 7.90E-18 | 2.97E-15 | down |
| RN7SL208P | -3.724170358 | 2.65E-08 | 8.94E-07 | down |
| RP11-339B9.1 | -3.724145084 | 0.039471905 | 0.102920916 | down |
| VN1R8P | -3.722944373 | 0.103789106 | 0.207371145 | down |
| LRRC9 | -3.721476378 | 0.000375271 | 0.002778022 | down |
| ZNF90P2 | -3.721382368 | 0.106693241 | 0.211765325 | down |
| RP11-418I22.2 | -3.721264829 | 1.69E-07 | 4.49E-06 | down |
| FABP2 | -3.72117151 | 2.63E-14 | 4.34E-12 | down |
| RP1-297M16.2 | -3.715965324 | 0.000261267 | 0.002085413 | down |
| GGTLC2 | -3.715538777 | 0.003321999 | 0.015681244 | down |
| NA | -3.715314258 | 0.002200005 | 0.011335438 | down |
| RP11-598D12.4 | -3.713874591 | 0.053690172 | 0.12876291 | down |
| CTB-159G17.3 | -3.71248174 | 0.011672007 | 0.041330868 | down |
| LINC00927 | -3.711719708 | 0.202718623 | 0.332069043 | down |
| RP3-331H24.6 | -3.711180313 | 2.58E-06 | 4.57E-05 | down |
| LINC01047 | -3.70902057 | 0.078674186 | 0.170135121 | down |
| RP11-615I2.3 | -3.708695846 | 0.017245865 | 0.055433269 | down |
| RNU6ATAC11P | -3.706058202 | 0.058575574 | 0.137326925 | down |
| AC005162.5 | -3.705796207 | 0.054684327 | 0.130599041 | down |
| RP11-856B14.10 | -3.704190728 | 0.022408241 | 0.06749205 | down |
| RP6-91H8.1 | -3.703914924 | 0.05261922 | 0.126825583 | down |
| RP11-223P11.3 | -3.703643495 | 5.69E-07 | 1.26E-05 | down |
| BEAN1-AS1 | -3.701733241 | 0.009679201 | 0.035729442 | down |
| LMO1 | -3.701356637 | 7.88E-07 | 1.66E-05 | down |
| RP11-447B18.1 | -3.700278462 | 0.143151602 | 0.260310297 | down |
| C8orf46 | -3.699117756 | 0.003223919 | 0.015323432 | down |
| RP5-836J3.1 | -3.699085332 | 1.62E-06 | 3.09E-05 | down |
| OR11A1 | -3.698640062 | 0.14427255 | 0.261756051 | down |
| C17orf112 | -3.696933855 | 0.003280612 | 0.015526576 | down |
| RP11-297A16.4 | -3.691781518 | 0.012666617 | 0.043944091 | down |
| RP11-361M4.1 | -3.691751157 | 0.076381247 | 0.166506907 | down |

| | | | | |
|---|---|---|---|---|
| AC004941.3 | -3.69157682 | 0.005614585 | 0.023701643 | down |
| C1orf158 | -3.69086003 | 0.022388282 | 0.067445721 | down |
| RN7SKP96 | -3.688851474 | 0.018580086 | 0.058764718 | down |
| RPL23AP10 | -3.688420591 | 0.000650127 | 0.004297561 | down |
| AC016831.6 | -3.684588106 | 0.042663568 | 0.109013799 | down |
| LINC01280 | -3.683114061 | 0.013025678 | 0.044806901 | down |
| AC006130.4 | -3.682853273 | 0.056606183 | 0.1339326 | down |
| CTC-492K19.7 | -3.682311653 | 0.014383171 | 0.048368925 | down |
| NA | -3.681896642 | 0.021313365 | 0.06505524 | down |
| RN7SL272P | -3.679731041 | 2.83E-06 | 4.97E-05 | down |
| RP11-138H8.4 | -3.679488609 | 0.030186939 | 0.084186509 | down |
| RP11-370I10.6 | -3.678498465 | 0.005124801 | 0.022109353 | down |
| ADAD1 | -3.67805503 | 0.021423927 | 0.065299835 | down |
| AC007491.1 | -3.678005376 | 0.013101405 | 0.045005706 | down |
| SLC5A9 | -3.677297264 | 9.08E-19 | 4.14E-16 | down |
| PRAMEF17 | -3.675757761 | 0.043814804 | 0.111114373 | down |
| ANKRD20A21P | -3.675297046 | 0.000133935 | 0.001204776 | down |
| CNGB3 | -3.672980162 | 7.06E-06 | 0.000105822 | down |
| XXbac-BPG27H4.8 | -3.672908697 | 0.006572229 | 0.026708515 | down |
| BAGE2 | -3.672777539 | 0.111884763 | 0.219002269 | down |
| PRKG2 | -3.670728164 | 5.36E-18 | 2.12E-15 | down |
| PWRN2 | -3.669872142 | 0.000478129 | 0.00335472 | down |
| RP11-503C24.4 | -3.667361737 | 2.13E-05 | 0.000263827 | down |
| RP1-166H1.2 | -3.666933735 | 0.110237405 | 0.21676632 | down |
| RP11-452I5.2 | -3.66673932 | 0.000444917 | 0.003169808 | down |
| SCN10A | -3.665373621 | 0.010240128 | 0.0373367 | down |
| RP11-126M14.1 | -3.663579152 | 0.018563555 | 0.05873692 | down |
| RP11-234K24.3 | -3.66324944 | 2.58E-05 | 0.000308724 | down |
| RP11-649A18.3 | -3.661358966 | 8.84E-11 | 6.09E-09 | down |
| Metazoa_SRP | -3.661222667 | 6.52E-06 | 9.92E-05 | down |
| MRLN | -3.660929986 | 2.64E-05 | 0.000315229 | down |
| LINC01500 | -3.658259556 | 8.75E-06 | 0.000126543 | down |
| CDH9 | -3.657953474 | 0.001223817 | 0.007124268 | down |
| RP11-19G24.2 | -3.656041202 | 0.001133305 | 0.006706521 | down |
| NA | -3.655867281 | 0.11097802 | 0.217794998 | down |
| TMEM225 | -3.654521204 | 0.000376217 | 0.002782635 | down |
| C8orf17 | -3.65446995 | 0.009332549 | 0.034781482 | down |
| HIST1H1A | -3.654377574 | 0.009474397 | 0.035167875 | down |
| RP11-466F5.6 | -3.652098793 | 9.39E-06 | 0.000134236 | down |
| KRT14 | -3.651203897 | 4.05E-11 | 3.10E-09 | down |
| RP11-398J10.2 | -3.65090299 | 0.007406301 | 0.029212934 | down |
| RP11-399E6.1 | -3.65034601 | 3.13E-07 | 7.52E-06 | down |
| RP11-777B9.4 | -3.649768514 | 0.024050213 | 0.071209677 | down |
| RP11-10F11.3 | -3.648495391 | 0.011701661 | 0.041399519 | down |
| NA | -3.648348155 | 3.93E-11 | 3.02E-09 | down |
| RP4-668E10.4 | -3.64772479 | 0.008626357 | 0.0327252 | down |
| RP11-64C12.1 | -3.647673796 | 4.87E-05 | 0.000523902 | down |
| LA16c-431H6.6 | -3.647607566 | 3.97E-08 | 1.29E-06 | down |
| ETF1P3 | -3.646518302 | 0.002061317 | 0.010748043 | down |
| RP11-412P11.1 | -3.645978638 | 0.000569157 | 0.003864348 | down |
| CTD-2143L24.1 | -3.644264161 | 0.02530333 | 0.073965095 | down |

| | | | | |
|---|---|---|---|---|
| CYP2B6 | -3.643577507 | 1.51E-15 | 3.32E-13 | down |
| AP004550.1 | -3.642657857 | 8.66E-05 | 0.000836904 | down |
| RP11-114018.1 | -3.642324578 | 0.033735249 | 0.09152645 | down |
| RP11-794C22.2 | -3.639184605 | 0.115147014 | 0.223422686 | down |
| RP11-521D12.2 | -3.638487713 | 0.030868348 | 0.085633301 | down |
| RP11-190J1.3 | -3.636954341 | 0.002261098 | 0.011602466 | down |
| OCM2 | -3.636188965 | 0.043655064 | 0.11088192 | down |
| RARRES2P3 | -3.634353653 | 0.085724144 | 0.181245333 | down |
| RP11-261N11.8 | -3.634020741 | 0.006753413 | 0.02727112 | down |
| GPR32 | -3.633828598 | 0.112932852 | 0.220235921 | down |
| Metazoa_SRP | -3.632448126 | 0.000662691 | 0.004363331 | down |
| RPL13AP17 | -3.631848231 | 0.01116683 | 0.039911522 | down |
| AC144836.1 | -3.631676484 | 0.002435094 | 0.012304066 | down |
| TARM1 | -3.630576477 | 0.000261234 | 0.002085413 | down |
| OTC | -3.629543993 | 4.49E-14 | 7.13E-12 | down |
| RP11-449P1.1 | -3.627160381 | 0.006540291 | 0.026621936 | down |
| RNU6-358P | -3.62587546 | 0.020785324 | 0.063864899 | down |
| LIN28B-AS1 | -3.625326888 | 0.024044258 | 0.071209677 | down |
| LINC01060 | -3.625000912 | 5.07E-05 | 0.000541054 | down |
| KC6 | -3.62441339 | 9.62E-06 | 0.000136754 | down |
| TRIM60 | -3.623377521 | 0.010697687 | 0.038629963 | down |
| RP11-318C2.1 | -3.622128389 | 0.008200196 | 0.031520815 | down |
| NA | -3.620621144 | 0.013129043 | 0.045054584 | down |
| ATP4B | -3.620608359 | 0.000928425 | 0.005717076 | down |
| NA | -3.61987338 | 0.021548952 | 0.065601044 | down |
| CTD-2540B15.6 | -3.61899958 | 0.045166063 | 0.113525408 | down |
| NA | -3.618941758 | 0.033313752 | 0.090610026 | down |
| DRD5 | -3.617173278 | 1.42E-07 | 3.84E-06 | down |
| AC097374.2 | -3.616069265 | 0.052920371 | 0.127337788 | down |
| RP11-363J17.1 | -3.615671538 | 0.015470365 | 0.051154704 | down |
| U82695.5 | -3.614910898 | 0.119829808 | 0.229954442 | down |
| CACYBPP1 | -3.611645926 | 0.001702119 | 0.009234104 | down |
| RP4-595K12.2 | -3.611276322 | 0.001316102 | 0.007530066 | down |
| ANKRD33 | -3.611092166 | 0.001186152 | 0.006952153 | down |
| RP11-90P5.1 | -3.609825503 | 0.010554405 | 0.038232039 | down |
| OR9N1P | -3.609153537 | 0.025706804 | 0.074838685 | down |
| TBPL2 | -3.608981584 | 0.000393914 | 0.002880203 | down |
| HMGN2P7 | -3.608780509 | 0.024821897 | 0.072905375 | down |
| LINC00939 | -3.608630744 | 7.43E-05 | 0.000736871 | down |
| LCE2A | -3.608493625 | 0.001569332 | 0.008646432 | down |
| CYP2C19 | -3.608275856 | 5.25E-08 | 1.64E-06 | down |
| RP11-496I2.4 | -3.607210617 | 0.007875722 | 0.030619789 | down |
| NEUROD2 | -3.606540454 | 4.61E-08 | 1.47E-06 | down |
| RP11-731C17.1 | -3.60580551 | 0.013309714 | 0.045544082 | down |
| NA | -3.604427196 | 0.034567695 | 0.093260762 | down |
| MAGEB3 | -3.601378749 | 0.029321723 | 0.082492916 | down |
| NA | -3.601239456 | 9.10E-08 | 2.63E-06 | down |
| Y_RNA | -3.596259105 | 0.015373148 | 0.050906078 | down |
| RP11-359M6.2 | -3.59317394 | 3.92E-08 | 1.27E-06 | down |
| RP11-21L23.4 | -3.590390485 | 0.00043849 | 0.00313219 | down |
| RPS6P12 | -3.590280446 | 0.002074163 | 0.01080377 | down |

| | | | | |
|---|---|---|---|---|
| RP11-542M13.1 | -3.590083661 | 0.090447741 | 0.188212489 | down |
| TBC1D3P1 | -3.589789593 | 0.066104902 | 0.149868373 | down |
| CTD-2134A5.4 | -3.589276483 | 9.93E-06 | 0.000140305 | down |
| RP11-465I4.3 | -3.589231057 | 0.06677814 | 0.150973168 | down |
| PRAMEF10 | -3.586536928 | 0.15606546 | 0.276389884 | down |
| LINC01044 | -3.584826113 | 0.158545989 | 0.279621701 | down |
| ENPP7 | -3.582767414 | 3.24E-09 | 1.46E-07 | down |
| RP11-491F9.2 | -3.582714258 | 0.031277872 | 0.08646599 | down |
| MRO | -3.581400303 | 3.27E-13 | 4.12E-11 | down |
| DEFA6 | -3.580186431 | 3.50E-07 | 8.22E-06 | down |
| TBR1 | -3.58008717 | 0.008827926 | 0.033345008 | down |
| MIR3646 | -3.578926475 | 2.18E-05 | 0.000268155 | down |
| SHANK2-AS3 | -3.577731964 | 0.117013486 | 0.226007919 | down |
| RN7SL372P | -3.576440195 | 0.064393156 | 0.14707526 | down |
| RP11-817J15.3 | -3.576417663 | 0.023310093 | 0.069511508 | down |
| LINC00397 | -3.575293008 | 0.161406019 | 0.28310365 | down |
| RP11-568G20.2 | -3.574703337 | 0.158818328 | 0.279893214 | down |
| FNTAP2 | -3.572862935 | 0.009152388 | 0.034277531 | down |
| AC002551.1 | -3.572427272 | 3.78E-05 | 0.000425254 | down |
| KIF28P | -3.5719122 | 2.02E-07 | 5.22E-06 | down |
| HOXC-AS2 | -3.571378439 | 0.000354471 | 0.002664428 | down |
| Y_RNA | -3.570951797 | 0.041947793 | 0.107706991 | down |
| NA | -3.569914659 | 0.008443952 | 0.032210525 | down |
| RP11-123O10.3 | -3.569753114 | 0.051800822 | 0.12541735 | down |
| HSPD1P8 | -3.56948888 | 0.011128171 | 0.039806496 | down |
| ACE2 | -3.568911805 | 0.00049485 | 0.003447084 | down |
| RP11-545A16.1 | -3.567083697 | 6.73E-07 | 1.46E-05 | down |
| LA16c-60G3.7 | -3.566704093 | 0.120159172 | 0.230395186 | down |
| OR10Q2P | -3.566540508 | 0.121062762 | 0.231577679 | down |
| POLR3GP2 | -3.565287408 | 0.208849244 | 0.339024566 | down |
| HNRNPA1P72 | -3.564924908 | 0.0314757 | 0.086908918 | down |
| RP11-515O17.3 | -3.564790254 | 0.002747236 | 0.013546359 | down |
| OR10G3 | -3.564189523 | 0.043172111 | 0.110018964 | down |
| PCA3 | -3.561880253 | 3.09E-07 | 7.44E-06 | down |
| RP11-552F3.12 | -3.561586762 | 2.42E-06 | 4.34E-05 | down |
| MIR219A2 | -3.559400683 | 0.033093481 | 0.090166004 | down |
| SULT1A2 | -3.558386953 | 4.04E-21 | 2.89E-18 | down |
| SLC4A9 | -3.558308771 | 1.68E-07 | 4.47E-06 | down |
| AKR1B10P1 | -3.557938116 | 7.25E-05 | 0.000722108 | down |
| LINC01085 | -3.55741349 | 0.000939044 | 0.005763562 | down |
| CTD-2037L6.2 | -3.557386547 | 0.018381777 | 0.058316244 | down |
| NA | -3.556547618 | 0.087168081 | 0.183330615 | down |
| SEC14L3 | -3.555453967 | 0.000284829 | 0.002235729 | down |
| CFL1P6 | -3.553978782 | 4.47E-05 | 0.000487391 | down |
| NA | -3.553593868 | 1.89E-05 | 0.00023921 | down |
| LA16c-380H5.6 | -3.552815847 | 2.27E-06 | 4.11E-05 | down |
| RP11-63A23.2 | -3.552046862 | 0.013319763 | 0.045568088 | down |
| RP11-132A1.3 | -3.55018988 | 0.053841613 | 0.129017181 | down |
| RP11-572C21.1 | -3.549863836 | 0.087383743 | 0.183629934 | down |
| CTD-2139B15.1 | -3.54815191 | 0.00962034 | 0.035555849 | down |
| LINC00486 | -3.547641152 | 3.01E-06 | 5.20E-05 | down |

| | | | | |
|---|---|---|---|---|
| IZUMO3 | -3.545255823 | 0.011431761 | 0.040650469 | down |
| NA | -3.542655891 | 0.009413357 | 0.035019178 | down |
| RP11-96H17.1 | -3.541740355 | 6.16E-05 | 0.00063469 | down |
| RP11-578F21.13 | -3.541372998 | 0.053451461 | 0.128307422 | down |
| NA | -3.541330332 | 0.050909116 | 0.123910474 | down |
| LINC01241 | -3.539882732 | 0.125277844 | 0.236958421 | down |
| RP11-550A18.1 | -3.539529137 | 0.165421709 | 0.288212121 | down |
| D21S2088E | -3.538481243 | 0.024209905 | 0.071583742 | down |
| LINC00958 | -3.535354347 | 0.000387851 | 0.002846263 | down |
| NA | -3.535001897 | 0.000543352 | 0.003721946 | down |
| NA | -3.533450428 | 0.225330861 | 0.357426896 | down |
| RP11-463I20.4 | -3.531211531 | 0.091326927 | 0.189412275 | down |
| RP3-467K16.2 | -3.530326074 | 0.014931596 | 0.049765111 | down |
| RP5-1037N22.2 | -3.528332677 | 0.003187964 | 0.015183764 | down |
| RP11-699F16.2 | -3.528019563 | 0.093108156 | 0.192032774 | down |
| SALL4P5 | -3.527993409 | 0.126550113 | 0.238733618 | down |
| RP11-634B7.4 | -3.527858591 | 0.0142458 | 0.048036007 | down |
| NA | -3.527480865 | 0.001643776 | 0.008977569 | down |
| NA | -3.52685636 | 0.006726403 | 0.027180638 | down |
| MYH6 | -3.526707028 | 8.90E-05 | 0.000858499 | down |
| SLC30A2 | -3.526682543 | 5.33E-13 | 6.40E-11 | down |
| RP11-4104.2 | -3.526235383 | 0.000743707 | 0.004788891 | down |
| NA | -3.525288933 | 0.013650324 | 0.04644511 | down |
| SLC36A2 | -3.52516041 | 0.000449491 | 0.003197847 | down |
| RP11-44D19.1 | -3.523601437 | 0.002302019 | 0.011766226 | down |
| ALOX15P2 | -3.522300901 | 0.023297234 | 0.069486955 | down |
| TAS1R2 | -3.521944895 | 0.069740258 | 0.155921737 | down |
| GALR3 | -3.520574539 | 4.26E-07 | 9.79E-06 | down |
| RNU1-17P | -3.519635043 | 0.008974886 | 0.0337685 | down |
| WI2-2118C23.2 | -3.51938889 | 0.000983582 | 0.005978303 | down |
| AC073069.2 | -3.518999017 | 0.095461256 | 0.195490284 | down |
| CTD-2528A14.5 | -3.518359855 | 9.15E-05 | 0.000878202 | down |
| C6orf10 | -3.516681185 | 0.000140305 | 0.001251954 | down |
| ABCG2 | -3.516617681 | 1.89E-20 | 1.16E-17 | down |
| SLC47A2 | -3.515849858 | 2.86E-11 | 2.29E-09 | down |
| RPS2P52 | -3.515735821 | 0.12709399 | 0.239383852 | down |
| RP11-568J23.1 | -3.514206875 | 0.000128721 | 0.001165899 | down |
| CTB-167G5.6 | -3.513652197 | 0.000472194 | 0.003321758 | down |
| AC010132.10 | -3.512738298 | 0.220422434 | 0.352085258 | down |
| RP11-13J10.1 | -3.512652362 | 0.006280998 | 0.025815397 | down |
| ZNF534 | -3.511555569 | 0.000945728 | 0.005793933 | down |
| KRT18P40 | -3.511370473 | 0.170552334 | 0.294413702 | down |
| RP11-515C16.7 | -3.510814564 | 0.090945206 | 0.188894418 | down |
| SPATA4 | -3.510438204 | 0.003934305 | 0.017993764 | down |
| RN7SL186P | -3.50973426 | 0.025242459 | 0.07383745 | down |
| SLC23A1 | -3.509720403 | 9.65E-17 | 2.87E-14 | down |
| OR4D9 | -3.509513282 | 0.013411937 | 0.045834081 | down |
| CICP8 | -3.509394916 | 0.068230481 | 0.153436868 | down |
| snoU109 | -3.508536156 | 4.92E-05 | 0.000528101 | down |
| RP11-443B9.1 | -3.507368246 | 0.168625226 | 0.292065969 | down |
| RN7SKP75 | -3.506216852 | 0.042355578 | 0.108457426 | down |

| | | | | |
|---|---|---|---|---|
| MEP1A | -3.505984967 | 1.75E-14 | 3.11E-12 | down |
| CLPSL1 | -3.505797165 | 0.040125833 | 0.104186373 | down |
| LINC01582 | -3.503439283 | 0.169546195 | 0.293184904 | down |
| LINC00635 | -3.501992748 | 0.001100953 | 0.006553801 | down |
| AP1B1P1 | -3.500578895 | 0.021555765 | 0.065615138 | down |
| NA | -3.499990074 | 0.003246306 | 0.015403034 | down |
| RAX2 | -3.499093946 | 0.059117597 | 0.138241678 | down |
| NA | -3.494721137 | 0.010721306 | 0.038687355 | down |
| NKX2-5 | -3.49375686 | 0.000728287 | 0.004710585 | down |
| AC010096.1 | -3.493533706 | 0.044964143 | 0.113161195 | down |
| AP000936.5 | -3.49297432 | 0.003777102 | 0.017410132 | down |
| GALP | -3.490942119 | 0.043856018 | 0.111189782 | down |
| AC009518.2 | -3.488817437 | 0.097901212 | 0.199042249 | down |
| RP11-140C5.3 | -3.487486513 | 0.023797866 | 0.070622588 | down |
| AC009967.3 | -3.487469008 | 0.001593994 | 0.008756621 | down |
| RN7SKP202 | -3.486794741 | 0.032096388 | 0.08816427 | down |
| RP11-1217F2.15 | -3.486551396 | 0.001190107 | 0.006971255 | down |
| NR2E1 | -3.484582531 | 0.058677128 | 0.137457748 | down |
| RESP18 | -3.484501739 | 0.006068628 | 0.025165752 | down |
| RP11-34C15.2 | -3.483950901 | 0.022590679 | 0.067912183 | down |
| RP11-700F16.2 | -3.483672404 | 0.000887783 | 0.005502902 | down |
| RP11-855A2.1 | -3.483629194 | 2.61E-08 | 8.85E-07 | down |
| NA | -3.483476712 | 2.16E-06 | 3.96E-05 | down |
| RP11-60E8.4 | -3.482232912 | 0.0931738 | 0.192141784 | down |
| Y_RNA | -3.481960852 | 0.000207069 | 0.001720458 | down |
| MLN | -3.480328453 | 0.002874061 | 0.014015304 | down |
| RP11-249L21.4 | -3.48007185 | 1.13E-05 | 0.000156393 | down |
| NA | -3.479948634 | 0.032628559 | 0.08922294 | down |
| SSX1 | -3.479941652 | 0.080683397 | 0.17325795 | down |
| RP11-7M10.2 | -3.479048053 | 0.036948076 | 0.097846452 | down |
| RP11-62H20.1 | -3.478428337 | 0.00829522 | 0.03179472 | down |
| RP11-268G12.1 | -3.478332885 | 0.003866157 | 0.017747108 | down |
| Metazoa_SRP | -3.477714655 | 0.131796222 | 0.245683793 | down |
| AC018892.3 | -3.476885715 | 0.022891437 | 0.068514677 | down |
| LINC00326 | -3.476729947 | 0.007936432 | 0.030774672 | down |
| NA | -3.473362544 | 0.002967117 | 0.014380394 | down |
| RN7SL524P | -3.47329528 | 0.000145806 | 0.00128879 | down |
| LL22NC01-81G9.3 | -3.472604255 | 0.028382683 | 0.080651596 | down |
| RP11-101B14.1 | -3.471966714 | 0.012098122 | 0.042447271 | down |
| AC012499.1 | -3.47194629 | 0.001728921 | 0.009357537 | down |
| FRMD1 | -3.471777966 | 6.40E-18 | 2.46E-15 | down |
| RPL9P31 | -3.471696768 | 0.13249384 | 0.24652955 | down |
| NA | -3.470986665 | 0.000495795 | 0.003452066 | down |
| RP11-384J4.2 | -3.470798564 | 0.009761288 | 0.035961255 | down |
| HMGN2P32 | -3.470738535 | 0.010754723 | 0.038784646 | down |
| LINC01030 | -3.47000409 | 0.023850386 | 0.070750494 | down |
| CYP17A1 | -3.469583145 | 0.031884428 | 0.087706992 | down |
| NA | -3.468651112 | 0.017828513 | 0.056998098 | down |
| OR51M1 | -3.468393767 | 0.024793352 | 0.072848914 | down |
| CDH12 | -3.46831473 | 0.005113796 | 0.022077726 | down |
| NA | -3.467488634 | 0.025587849 | 0.074557398 | down |

| | | | | |
|---|---|---|---|---|
| RP11-382D8.5 | -3.467352955 | 0.023734005 | 0.070467877 | down |
| KRT89P | -3.467056857 | 0.000103952 | 0.00097843 | down |
| MYH16 | -3.466472336 | 2.89E-07 | 7.05E-06 | down |
| REEP6 | -3.466077985 | 2.05E-22 | 2.13E-19 | down |
| RP11-14I2.2 | -3.465287324 | 0.130930141 | 0.244496043 | down |
| ELF5 | -3.465068932 | 0.007389828 | 0.029155613 | down |
| DPPA3 | -3.465037633 | 0.000977121 | 0.00594352 | down |
| NA | -3.463547044 | 0.097914358 | 0.199042249 | down |
| AC073316.2 | -3.462424081 | 0.009105419 | 0.034139923 | down |
| RP4-811M8.1 | -3.461670952 | 8.10E-07 | 1.69E-05 | down |
| RP1-90J20.10 | -3.460889554 | 0.17394064 | 0.29842907 | down |
| CTD-2530N21.5 | -3.460664233 | 0.037226772 | 0.098436995 | down |
| RP11-103J17.2 | -3.460552457 | 9.35E-05 | 0.000893506 | down |
| NA | -3.460430347 | 0.131831849 | 0.245716489 | down |
| DAW1 | -3.459015019 | 0.001200177 | 0.007011106 | down |
| AKAIN1 | -3.45863424 | 0.002144512 | 0.011100918 | down |
| TRPC7 | -3.458633136 | 0.000127631 | 0.001157078 | down |
| AC002543.2 | -3.458165768 | 0.005459361 | 0.023202866 | down |
| SLCO1B7 | -3.457286807 | 0.015842599 | 0.052036506 | down |
| RP1-272L16.1 | -3.45572917 | 0.057073896 | 0.134742112 | down |
| RP11-145G20.1 | -3.455558819 | 0.003182685 | 0.015161026 | down |
| IL1RAPL2 | -3.45504234 | 0.025210133 | 0.073764438 | down |
| AC005863.1 | -3.454704152 | 0.077154588 | 0.167694602 | down |
| AF241726.2 | -3.453561167 | 0.034825588 | 0.093700205 | down |
| RNU6-140P | -3.452314779 | 7.71E-08 | 2.30E-06 | down |
| RP11-432M8.6 | -3.45201431 | 0.083159185 | 0.177181374 | down |
| RP11-19N8.4 | -3.451041332 | 0.056874817 | 0.134388282 | down |
| RP11-80F22.4 | -3.448940937 | 0.014165139 | 0.047817689 | down |
| KIR3DL3 | -3.447375304 | 0.028076141 | 0.079992373 | down |
| RP11-85B7.4 | -3.446801815 | 0.0453351 | 0.113816976 | down |
| RHBDF1P1 | -3.445210714 | 0.102608324 | 0.205727391 | down |
| ERVH-1 | -3.444262745 | 1.71E-06 | 3.24E-05 | down |
| PHBP7 | -3.443356515 | 0.011401537 | 0.040591038 | down |
| RP11-849I19.1 | -3.442733205 | 0.101218181 | 0.203779337 | down |
| NA | -3.441071546 | 0.020277235 | 0.062756228 | down |
| RP11-592N21.2 | -3.440978966 | 0.008996486 | 0.033832824 | down |
| KRT17P3 | -3.440871185 | 0.177734749 | 0.302709555 | down |
| LINC01289 | -3.440473514 | 0.014654493 | 0.049056222 | down |
| RNU1-8P | -3.439877434 | 0.005441729 | 0.02316069 | down |
| FAM81B | -3.439856875 | 0.001785528 | 0.009584491 | down |
| LINC01021 | -3.439334778 | 0.007299058 | 0.028869525 | down |
| NA | -3.438411321 | 0.137829606 | 0.25353289 | down |
| RP11-464C19.2 | -3.437375137 | 0.083747933 | 0.17799398 | down |
| RN7SL785P | -3.436527451 | 0.178574393 | 0.303623141 | down |
| SMPD4P2 | -3.436155696 | 0.231227454 | 0.363734734 | down |
| NA | -3.435549063 | 6.40E-07 | 1.40E-05 | down |
| RP11-165J3.5 | -3.433234111 | 0.231233051 | 0.363734734 | down |
| PGA3 | -3.431422998 | 0.012066491 | 0.042356078 | down |
| LINC01304 | -3.430134763 | 0.003768291 | 0.017377521 | down |
| RP11-179K3.2 | -3.429210036 | 0.010908641 | 0.039207946 | down |
| MTND4P11 | -3.427137757 | 0.042769349 | 0.109237636 | down |

| | | | | |
|---|---|---|---|---|
| RP11-143A12.3 | -3.426695815 | 0.026189912 | 0.075877504 | down |
| CTD-2145A24.4 | -3.42396914 | 3.14E-06 | 5.39E-05 | down |
| FOLH1B | -3.423386796 | 0.009366582 | 0.034884043 | down |
| NA | -3.423335779 | 0.00120748 | 0.007052396 | down |
| GOLGA6L6 | -3.421930665 | 0.075488759 | 0.165112426 | down |
| CTD-2315E11.1 | -3.42126593 | 0.001585948 | 0.008723586 | down |
| RP11-27M9.1 | -3.419130657 | 0.233015366 | 0.365725298 | down |
| NA | -3.419111845 | 0.051649469 | 0.125214903 | down |
| LINC01441 | -3.418559248 | 0.234298032 | 0.367048184 | down |
| SLC1A6 | -3.416769046 | 0.000271821 | 0.002153831 | down |
| NA | -3.416526655 | 0.023353169 | 0.069605417 | down |
| CTD-2015A6.2 | -3.41648165 | 0.003285115 | 0.015542992 | down |
| AC009518.4 | -3.415208782 | 0.001519416 | 0.008423941 | down |
| CTA-256D12.11 | -3.414862885 | 0.007143467 | 0.028411215 | down |
| AC010468.3 | -3.41382278 | 0.062350898 | 0.143799062 | down |
| TSPYL6 | -3.413111577 | 0.0336312 | 0.091300562 | down |
| AJ006995.3 | -3.411919085 | 0.040108281 | 0.104149801 | down |
| RP11-231I16.1 | -3.409924723 | 7.75E-06 | 0.000114358 | down |
| RP11-131J4.12 | -3.40941162 | 0.183267749 | 0.309036104 | down |
| RPL21P12 | -3.408791008 | 0.010056822 | 0.036847214 | down |
| RP11-776A13.3 | -3.408017882 | 0.009333255 | 0.034781482 | down |
| NA | -3.407388219 | 0.23677477 | 0.369851202 | down |
| RP11-348H3.8 | -3.407372116 | 0.008085361 | 0.031179128 | down |
| NA | -3.40657603 | 0.025239721 | 0.073836629 | down |
| CTB-58E17.2 | -3.406545947 | 0.236971227 | 0.370078155 | down |
| SLC17A1 | -3.406309752 | 3.25E-08 | 1.08E-06 | down |
| RP11-292B8.2 | -3.405520893 | 0.004358409 | 0.01947413 | down |
| CNBD1 | -3.404912237 | 0.001991833 | 0.010452863 | down |
| OR2Z1 | -3.404685326 | 0.182235645 | 0.307806701 | down |
| OR1X1P | -3.402768084 | 0.061541319 | 0.142449873 | down |
| AC013470.8 | -3.402644396 | 0.047531621 | 0.117844531 | down |
| NA | -3.402515365 | 0.009214146 | 0.034457282 | down |
| SSX7 | -3.4022 | 0.140019632 | 0.256321584 | down |
| HSPA8P14 | -3.40163623 | 0.045529172 | 0.114161113 | down |
| CLDN19 | -3.401406918 | 1.76E-05 | 0.000225308 | down |
| DYDC1 | -3.400649988 | 0.029318844 | 0.08249254 | down |
| RP11-123C21.2 | -3.400603928 | 0.001778753 | 0.009556663 | down |
| REXO1L1P | -3.399137613 | 0.238581452 | 0.372012723 | down |
| SUB1P4 | -3.398754003 | 0.000216701 | 0.001785671 | down |
| PROP1 | -3.398075822 | 0.006574317 | 0.026711862 | down |
| RP11-511B23.3 | -3.397574598 | 0.000510495 | 0.003536391 | down |
| RNASEH1P2 | -3.397111784 | 0.143037128 | 0.26017382 | down |
| CHN2 | -3.396273297 | 1.45E-09 | 7.14E-08 | down |
| RP11-490K7.1 | -3.394365022 | 0.021303384 | 0.065033237 | down |
| CTB-186G2.1 | -3.394202729 | 0.001676755 | 0.009116253 | down |
| RP11-475A13.1 | -3.394187171 | 0.001822175 | 0.009742634 | down |
| AF131215.3 | -3.393397167 | 0.000151001 | 0.001326517 | down |
| CATSPERD | -3.393256065 | 0.001341585 | 0.007642505 | down |
| RP1-17K7.2 | -3.39225117 | 0.023658394 | 0.070285058 | down |
| RP11-767C6.1 | -3.391883824 | 0.035460813 | 0.094904787 | down |
| RP11-244C20.2 | -3.391559014 | 0.055873886 | 0.13267012 | down |

| | | | | |
|---|---|---|---|---|
| TNRC18P2 | -3.391252187 | 0.00959693 | 0.035486788 | down |
| VTRNA2-2P | -3.390439346 | 0.010622501 | 0.038418493 | down |
| CTC-463N11.1 | -3.390226714 | 0.140867005 | 0.257433595 | down |
| AC009508.1 | -3.389829242 | 0.143463929 | 0.260736209 | down |
| AC004257.1 | -3.385669038 | 0.043570206 | 0.110755839 | down |
| RP11-413N13.1 | -3.385276998 | 0.082117494 | 0.175672031 | down |
| PITX2 | -3.384072662 | 2.18E-10 | 1.35E-08 | down |
| RP11-317M11.1 | -3.383812113 | 0.000686444 | 0.004487278 | down |
| RN7SKP89 | -3.3821557 | 0.147291093 | 0.265666903 | down |
| RP11-85B7.2 | -3.379781566 | 0.00072749 | 0.00470644 | down |
| PCDHA9 | -3.37773527 | 0.025494063 | 0.074334592 | down |
| LRRC72 | -3.377040084 | 0.046698722 | 0.116279783 | down |
| MIR5000 | -3.37593411 | 0.026356062 | 0.076233902 | down |
| EGFEM1P | -3.375562533 | 5.31E-05 | 0.000560533 | down |
| TDRD12 | -3.375328288 | 4.86E-11 | 3.62E-09 | down |
| UNC5D | -3.373144116 | 7.73E-09 | 3.14E-07 | down |
| AC004603.4 | -3.372714002 | 0.011642765 | 0.041254303 | down |
| RPL27AP5 | -3.372175281 | 0.000336416 | 0.002559452 | down |
| SLC2A7 | -3.371282942 | 0.000126764 | 0.001150954 | down |
| NLRP6 | -3.369444633 | 1.63E-13 | 2.27E-11 | down |
| VWA5B1 | -3.369124521 | 3.51E-06 | 5.93E-05 | down |
| MRPS21P9 | -3.365791525 | 0.109344638 | 0.215589336 | down |
| ALDOAP1 | -3.365758993 | 0.062742754 | 0.144436696 | down |
| PSG6 | -3.363879802 | 0.06638277 | 0.150302917 | down |
| SPATA31D3 | -3.36368567 | 0.017878038 | 0.057090535 | down |
| MS4A8 | -3.363355108 | 9.32E-13 | 1.05E-10 | down |
| RPL17P40 | -3.363144849 | 0.01030098 | 0.037504356 | down |
| RP11-122F24.1 | -3.363067625 | 0.04869845 | 0.120111441 | down |
| RP11-838N2.3 | -3.36263253 | 0.06385192 | 0.146157353 | down |
| CTD-2343P13.1 | -3.361273824 | 0.188253175 | 0.315108272 | down |
| NOS1 | -3.359808204 | 1.19E-08 | 4.64E-07 | down |
| LINC00692 | -3.359310198 | 0.143707655 | 0.261100194 | down |
| NA | -3.358630207 | 1.62E-08 | 6.05E-07 | down |
| RP11-120I21.3 | -3.357262268 | 0.003102171 | 0.014876556 | down |
| GOLGA2P4 | -3.356778161 | 0.064081456 | 0.146545645 | down |
| RP11-496B10.3 | -3.356400843 | 0.002205835 | 0.01135185 | down |
| CTC-360J11.4 | -3.355334607 | 0.000703129 | 0.004582449 | down |
| RP11-644F5.16 | -3.355301666 | 0.082838189 | 0.17675259 | down |
| RNU6-419P | -3.354466902 | 0.049272979 | 0.121092628 | down |
| IQCA1L | -3.354246118 | 0.039088332 | 0.102163536 | down |
| NA | -3.35408156 | 0.000318276 | 0.002444974 | down |
| RP11-539L10.5 | -3.35405546 | 0.001055753 | 0.006332604 | down |
| LINC01255 | -3.353361337 | 0.08602706 | 0.181706762 | down |
| RP11-474D1.1 | -3.352210974 | 0.024540606 | 0.072294116 | down |
| MARK2P5 | -3.351210872 | 0.049002889 | 0.120586683 | down |
| GOLGA8UP | -3.350446563 | 0.004846941 | 0.021190556 | down |
| XXyac-YM21GA2.3 | -3.350349601 | 0.002978909 | 0.014421259 | down |
| RNU6-222P | -3.350243717 | 0.0908092 | 0.188677151 | down |
| TM6SF2 | -3.34999746 | 7.23E-13 | 8.36E-11 | down |
| RP1-190J20.2 | -3.348241793 | 0.04681178 | 0.11647604 | down |
| NA | -3.347899245 | 0.086110718 | 0.181819552 | down |

| | | | | |
|---|---|---|---|---|
| CKM | -3.347805926 | 5.10E-08 | 1.61E-06 | down |
| NA | -3.347407885 | 0.006251452 | 0.025739157 | down |
| RP11-435I10.6 | -3.347268823 | 0.034884209 | 0.093828217 | down |
| MYADML | -3.346610958 | 0.002955836 | 0.014332657 | down |
| RP11-549L6.2 | -3.345840057 | 0.07143914 | 0.158669144 | down |
| ELOVL2-AS1 | -3.345663029 | 0.065061035 | 0.148071641 | down |
| LINC01288 | -3.343310503 | 0.148317086 | 0.267032379 | down |
| NA | -3.342904242 | 0.052817595 | 0.127162678 | down |
| RP11-419M24.1 | -3.342581727 | 0.040538167 | 0.104946154 | down |
| AMER3 | -3.340776092 | 6.94E-07 | 1.49E-05 | down |
| RP4-738P15.1 | -3.340684717 | 0.000181004 | 0.001540971 | down |
| NA | -3.340522989 | 0.001444423 | 0.008091786 | down |
| ATP6V0A4 | -3.339656824 | 0.000690236 | 0.004511085 | down |
| RP11-798L4.1 | -3.337806199 | 0.051279301 | 0.124577292 | down |
| RP11-314D7.2 | -3.33750627 | 0.00501099 | 0.021740097 | down |
| OR51L1 | -3.337463537 | 0.14832367 | 0.267032379 | down |
| VTRNA1-2 | -3.336931474 | 0.003044729 | 0.014667576 | down |
| AC009158.1 | -3.336883286 | 0.191656657 | 0.319360361 | down |
| RP11-552E4.2 | -3.336847179 | 0.090806956 | 0.188677151 | down |
| RP11-1112G13.3 | -3.335300984 | 0.014765541 | 0.049350973 | down |
| LINC01080 | -3.334137383 | 0.001712846 | 0.009285589 | down |
| DSCAM | -3.333983971 | 3.99E-05 | 0.000443691 | down |
| HNF1A-AS1 | -3.333699062 | 1.99E-14 | 3.41E-12 | down |
| MUC19 | -3.333596523 | 5.99E-05 | 0.000618786 | down |
| PDE11A | -3.333082303 | 6.36E-11 | 4.56E-09 | down |
| RP11-219E7.3 | -3.331459858 | 0.000531816 | 0.003662131 | down |
| SULT6B1 | -3.331427057 | 0.009609478 | 0.035524445 | down |
| DPPA2P2 | -3.331206461 | 0.044537703 | 0.112366146 | down |
| RP11-459J23.1 | -3.330711051 | 0.191756129 | 0.319494295 | down |
| LRAT | -3.330672303 | 4.93E-10 | 2.77E-08 | down |
| KNOP1P5 | -3.330058685 | 0.045671622 | 0.114394182 | down |
| Y_RNA | -3.329443638 | 0.014529542 | 0.048719372 | down |
| RNA5SP97 | -3.327621641 | 0.020260011 | 0.062725863 | down |
| SLC22A14 | -3.327383644 | 9.51E-07 | 1.94E-05 | down |
| NKX6-1 | -3.32733153 | 0.017679189 | 0.056614106 | down |
| RP11-127I20.7 | -3.32718751 | 0.111332717 | 0.218291468 | down |
| RP11-367J11.2 | -3.326490554 | 8.32E-05 | 0.000811672 | down |
| RPS3AP4 | -3.324722911 | 0.010328357 | 0.037599073 | down |
| RN7SL18P | -3.323169399 | 0.030011018 | 0.083788776 | down |
| NCAPGP2 | -3.322664561 | 0.005796115 | 0.024287095 | down |
| RP11-415C15.2 | -3.319869558 | 0.005435197 | 0.023136165 | down |
| ANKRD20A19P | -3.319394128 | 0.022457526 | 0.06761338 | down |
| ASS1P2 | -3.31679316 | 0.007856279 | 0.030566439 | down |
| RP11-411G2.2 | -3.316581428 | 0.152500661 | 0.271961172 | down |
| NA | -3.31582346 | 0.000202961 | 0.001691006 | down |
| RP11-388G22.1 | -3.315459507 | 0.063430412 | 0.145606914 | down |
| RP11-221N13.2 | -3.314628493 | 0.152440979 | 0.271903157 | down |
| RP11-20I23.10 | -3.314465685 | 4.42E-10 | 2.52E-08 | down |
| RP11-370P15.2 | -3.31369816 | 0.150010535 | 0.268973294 | down |
| UBA52P5 | -3.312137991 | 8.39E-05 | 0.000817212 | down |
| FUT9 | -3.311591853 | 0.028468762 | 0.080840463 | down |

| | | | | |
|---|---|---|---|---|
| CTB-95D12.1 | -3.310839604 | 0.017486173 | 0.056079695 | down |
| Y_RNA | -3.310779722 | 0.00081104 | 0.005134194 | down |
| RP11-10017.1 | -3.309972705 | 7.20E-08 | 2.17E-06 | down |
| RN7SL340P | -3.307338218 | 0.115597391 | 0.224093949 | down |
| GRAMD4P2 | -3.306550564 | 0.153131609 | 0.272759861 | down |
| AC006262.5 | -3.306444137 | 0.014154143 | 0.047801577 | down |
| RP11-1100L3.4 | -3.305024465 | 2.41E-06 | 4.33E-05 | down |
| LINC00836 | -3.304807747 | 0.000400709 | 0.002919236 | down |
| RP11-16C1.2 | -3.304378355 | 0.000430975 | 0.003088789 | down |
| RPL21P122 | -3.304237108 | 8.01E-06 | 0.000117274 | down |
| KNCN | -3.303711488 | 0.052899011 | 0.127296587 | down |
| AL133249.1 | -3.303682554 | 0.199054207 | 0.328051815 | down |
| RP11-316A16.1 | -3.303353368 | 0.115268032 | 0.223585303 | down |
| AC103881.1 | -3.303168512 | 0.257133445 | 0.392150638 | down |
| EEF1A1P33 | -3.302678314 | 0.004265835 | 0.019168821 | down |
| SPATA31C1 | -3.302476489 | 0.003061728 | 0.014724917 | down |
| NA | -3.302279304 | 0.024857885 | 0.072989668 | down |
| AC098828.2 | -3.300870386 | 0.00022791 | 0.001863726 | down |
| VSX1 | -3.300740124 | 0.02359697 | 0.070158078 | down |
| UBTFL7 | -3.300183188 | 0.024287099 | 0.0717428 | down |
| PANX3 | -3.299130683 | 0.029264963 | 0.082394953 | down |
| NA | -3.299088695 | 0.253497247 | 0.388436339 | down |
| CDH16 | -3.298968265 | 2.24E-07 | 5.67E-06 | down |
| KISS1 | -3.298085263 | 2.07E-07 | 5.31E-06 | down |
| ACY1 | -3.297946305 | 8.39E-11 | 5.82E-09 | down |
| CYP3A7 | -3.297192751 | 0.004480948 | 0.019944034 | down |
| RP11-44K6.5 | -3.297033997 | 0.016870726 | 0.054513238 | down |
| NA | -3.295579707 | 0.046812947 | 0.11647604 | down |
| CTB-184G21.3 | -3.294576836 | 0.022038703 | 0.066706709 | down |
| SRRM1P3 | -3.29457504 | 4.70E-05 | 0.000507264 | down |
| AD001527.7 | -3.294127975 | 1.91E-10 | 1.20E-08 | down |
| CLEC19A | -3.29382035 | 0.000482608 | 0.003378488 | down |
| AP003025.2 | -3.292644897 | 0.002365552 | 0.012026731 | down |
| RP11-893F2.15 | -3.292527113 | 0.120054449 | 0.23023847 | down |
| RP4-724E13.2 | -3.292196633 | 3.81E-05 | 0.000427264 | down |
| GRM7-AS1 | -3.29057856 | 0.02036173 | 0.062943494 | down |
| LINC01549 | -3.290473637 | 0.028179768 | 0.080196357 | down |
| RP1-242N11.1 | -3.290029995 | 0.04010187 | 0.104142991 | down |
| RP11-438P9.1 | -3.289635911 | 0.069151001 | 0.154930349 | down |
| RP11-178L8.6 | -3.289234824 | 0.120513386 | 0.230838646 | down |
| LINC00343 | -3.287683115 | 0.118019108 | 0.227482267 | down |
| CYCSP35 | -3.287610201 | 0.15421337 | 0.27395852 | down |
| NA | -3.287590774 | 0.257610871 | 0.392640671 | down |
| NA | -3.286038177 | 0.005312099 | 0.022737812 | down |
| CTD-2128A3.2 | -3.285481801 | 0.067336247 | 0.151960506 | down |
| RP11-259A24.1 | -3.285013309 | 0.011408382 | 0.040603594 | down |
| Metazoa_SRP | -3.283926326 | 0.001216543 | 0.007091541 | down |
| AC092638.2 | -3.283809404 | 0.08957109 | 0.186932052 | down |
| NR1H4 | -3.283556524 | 2.05E-15 | 4.37E-13 | down |
| VN2R3P | -3.281781677 | 0.257135121 | 0.392150638 | down |
| RP11-282K24.3 | -3.280744054 | 9.00E-09 | 3.60E-07 | down |

| | | | | |
|---|---|---|---|---|
| NA | -3.280643283 | 0.005705049 | 0.023989153 | down |
| RP11-278J20.2 | -3.280423281 | 0.027538558 | 0.078796995 | down |
| RPSAP4 | -3.280403195 | 0.000668794 | 0.004394851 | down |
| ATG4AP1 | -3.280310745 | 0.087924948 | 0.184418956 | down |
| LSM3P5 | -3.278210312 | 0.119150112 | 0.2290158 | down |
| FAM41C | -3.276232391 | 0.086337563 | 0.182091846 | down |
| AL121657.4 | -3.274292165 | 0.022748422 | 0.068269071 | down |
| RN7SL602P | -3.273680256 | 0.061199802 | 0.141895884 | down |
| RP11-145E17.3 | -3.272989664 | 0.061826401 | 0.142904404 | down |
| RP11-83B20.5 | -3.272081493 | 0.068630238 | 0.154070602 | down |
| RP11-166P13.4 | -3.271691469 | 1.42E-07 | 3.84E-06 | down |
| LINC01419 | -3.271381021 | 0.261756961 | 0.39688247 | down |
| ASB15 | -3.27129071 | 0.003918184 | 0.017942069 | down |
| AKIRIN1P2 | -3.270396963 | 0.011165952 | 0.039911522 | down |
| CTD-2336H13.2 | -3.270057918 | 0.08508606 | 0.18020059 | down |
| RP11-404K5.2 | -3.269573769 | 0.046844196 | 0.11653449 | down |
| RP11-123B3.2 | -3.269457765 | 0.00208974 | 0.010859019 | down |
| CHRM4 | -3.268910795 | 4.59E-06 | 7.40E-05 | down |
| RP5-906A24.1 | -3.268544523 | 0.095556575 | 0.19563994 | down |
| RP5-1107A17.2 | -3.268244361 | 0.002336672 | 0.011912943 | down |
| RP11-328L11.1 | -3.267383762 | 0.000257182 | 0.002058792 | down |
| DLGAP1-AS4 | -3.267185642 | 0.032148331 | 0.088263225 | down |
| KRTAP13-2 | -3.26670451 | 0.016124449 | 0.052725778 | down |
| RP11-531H8.1 | -3.26646412 | 0.022103294 | 0.066834879 | down |
| OSBPL10-AS1 | -3.266418276 | 8.65E-05 | 0.000836606 | down |
| RP4-601P9.2 | -3.265426044 | 0.000357542 | 0.002682816 | down |
| RP11-639E23.1 | -3.264954206 | 0.009039812 | 0.033950305 | down |
| C8orf34-AS1 | -3.264159601 | 0.000129125 | 0.001169184 | down |
| RNY4P34 | -3.263985839 | 0.01023839 | 0.0373367 | down |
| NA | -3.263540366 | 6.81E-05 | 0.000686103 | down |
| CTC-518B2.10 | -3.263259298 | 0.027256001 | 0.078211968 | down |
| CHAT | -3.263112826 | 0.000943434 | 0.005781445 | down |
| MROH7 | -3.262118115 | 8.80E-18 | 3.19E-15 | down |
| RP11-639B1.1 | -3.261917861 | 0.205266018 | 0.335072657 | down |
| RPS11P6 | -3.261514614 | 0.000399507 | 0.002915422 | down |
| SPRR2F | -3.259080238 | 0.157320113 | 0.278047265 | down |
| LINC00376 | -3.258396635 | 0.009040156 | 0.033950305 | down |
| LIN28B | -3.257320936 | 0.125468674 | 0.237229758 | down |
| KCNA7 | -3.257217297 | 0.005180774 | 0.022283634 | down |
| RP11-159D12.10 | -3.256903569 | 0.070954305 | 0.157825656 | down |
| ZSWIM5P3 | -3.256654429 | 0.012201703 | 0.042715911 | down |
| RP11-338I21.1 | -3.256216535 | 6.24E-09 | 2.59E-07 | down |
| RP11-153K11.3 | -3.255614369 | 0.001281715 | 0.007368513 | down |
| RP11-380D11.2 | -3.254717586 | 0.034327956 | 0.09277225 | down |
| RPL7L1P2 | -3.254094614 | 0.16031953 | 0.281691324 | down |
| GUCY2GP | -3.254023553 | 1.42E-05 | 0.000189557 | down |
| RP11-32D16.1 | -3.252270541 | 0.072405219 | 0.160269829 | down |
| NAT16 | -3.251963817 | 0.000768818 | 0.004916551 | down |
| RP1-12803.4 | -3.250833054 | 0.161965829 | 0.283886668 | down |
| BANF2 | -3.250748998 | 0.130803624 | 0.244379334 | down |
| RP11-775H9.2 | -3.250675619 | 0.120490595 | 0.230809706 | down |

| | | | | |
|---|---|---|---|---|
| CABP5 | -3.250423556 | 0.010431972 | 0.037898208 | down |
| LINC00964 | -3.249165466 | 1.29E-06 | 2.53E-05 | down |
| RPL10P2 | -3.249048304 | 0.067425363 | 0.152070235 | down |
| RP13-492C18.1 | -3.248906484 | 0.018196673 | 0.057851993 | down |
| SCGB1D2 | -3.248308208 | 0.005636563 | 0.023768443 | down |
| ADGRV1 | -3.247586202 | 8.27E-10 | 4.34E-08 | down |
| SLC6A5 | -3.247448497 | 0.002988696 | 0.014455385 | down |
| CT45A1 | -3.247423538 | 0.159280237 | 0.28042282 | down |
| KARSP3 | -3.247265419 | 0.075877515 | 0.165649184 | down |
| RP4-660H19.1 | -3.246986339 | 0.052122027 | 0.125935321 | down |
| RP5-1024C24.1 | -3.246281267 | 0.01094641 | 0.039334282 | down |
| RP5-1061H20.5 | -3.246025453 | 0.013732667 | 0.046646043 | down |
| SLC37A2 | -3.24596478 | 6.99E-19 | 3.23E-16 | down |
| AC007163.3 | -3.244385591 | 0.032151551 | 0.088263998 | down |
| RPS27AP3 | -3.243824561 | 0.097917869 | 0.199042249 | down |
| CASC23 | -3.243213889 | 0.125311546 | 0.236998486 | down |
| NA | -3.242923289 | 0.009516371 | 0.03528346 | down |
| RP11-404O13.5 | -3.242857112 | 0.001857546 | 0.009905118 | down |
| AGGF1P10 | -3.242535464 | 0.008907705 | 0.033575364 | down |
| MYH4 | -3.242476495 | 0.000113702 | 0.001054668 | down |
| ANKRD45 | -3.242450114 | 5.86E-06 | 9.06E-05 | down |
| USH2A | -3.241027154 | 2.03E-06 | 3.75E-05 | down |
| MEP1AP4 | -3.240790013 | 2.20E-08 | 7.69E-07 | down |
| AC019118.3 | -3.240502452 | 0.094347857 | 0.193990351 | down |
| OR6C66P | -3.239964009 | 0.127829135 | 0.240361145 | down |
| RP11-345P4.11 | -3.239712057 | 0.266420098 | 0.40210617 | down |
| PCK1 | -3.239097055 | 9.90E-12 | 8.70E-10 | down |
| NA | -3.238934908 | 0.02901695 | 0.081927563 | down |
| RP11-321E2.3 | -3.23860684 | 0.00014191 | 0.001263271 | down |
| GJD2 | -3.238542288 | 0.003049655 | 0.014678601 | down |
| GJD4 | -3.236874954 | 0.051672193 | 0.125237123 | down |
| GSG1 | -3.235420262 | 0.000628009 | 0.004184497 | down |
| GAPDHP68 | -3.235171074 | 0.017195769 | 0.055296515 | down |
| RP5-1121H13.1 | -3.233574585 | 0.122720923 | 0.233654224 | down |
| TRIM80P | -3.232871638 | 7.32E-10 | 3.92E-08 | down |
| RP11-625H11.2 | -3.232031385 | 0.018350554 | 0.058236405 | down |
| NA | -3.231334353 | 0.127582126 | 0.240062465 | down |
| RP11-715G15.2 | -3.230653865 | 0.123817477 | 0.235128771 | down |
| AC024028.1 | -3.229985794 | 0.021327619 | 0.065085524 | down |
| CTD-3007L5.1 | -3.229242419 | 0.129520394 | 0.242600418 | down |
| RP11-551L14.4 | -3.227910903 | 2.34E-12 | 2.36E-10 | down |
| NA | -3.225072768 | 0.002262022 | 0.011605221 | down |
| AC142119.1 | -3.224568822 | 0.005642519 | 0.023782808 | down |
| AP000253.1 | -3.223508231 | 4.32E-06 | 7.04E-05 | down |
| RP11-639F1.2 | -3.223353886 | 7.91E-07 | 1.66E-05 | down |
| AC079630.2 | -3.22222612 | 1.36E-05 | 0.000182343 | down |
| RP11-1223D19.5 | -3.22144933 | 0.000104496 | 0.000982008 | down |
| RP11-440I14.2 | -3.220628879 | 6.19E-08 | 1.89E-06 | down |
| RPL21P106 | -3.220241309 | 0.005646678 | 0.02378785 | down |
| CTC-774J1.1 | -3.219954731 | 0.127450286 | 0.239874484 | down |
| KRT18P57 | -3.219774541 | 0.001385743 | 0.007836111 | down |

| | | | | |
|---|---|---|---|---|
| LINC00615 | -3.217289844 | 0.269756075 | 0.405755058 | down |
| NA | -3.216272755 | 2.47E-05 | 0.000297608 | down |
| RP4-680D5.9 | -3.215321101 | 0.003616236 | 0.016807192 | down |
| RP11-300M24.1 | -3.215224437 | 0.050785412 | 0.123676494 | down |
| RP5-1051H14.2 | -3.214709171 | 0.003899118 | 0.017876561 | down |
| CYP4F26P | -3.214460786 | 0.022050306 | 0.066735105 | down |
| AC093585.6 | -3.213834293 | 0.000165685 | 0.001431649 | down |
| RP13-631K18.3 | -3.213751762 | 0.05697734 | 0.134556453 | down |
| CTA-315H11.2 | -3.212937732 | 5.28E-06 | 8.29E-05 | down |
| SSX9 | -3.212835421 | 0.270422204 | 0.406431451 | down |
| CTD-2130O13.1 | -3.212342578 | 0.000346588 | 0.002620257 | down |
| RP11-240D10.4 | -3.21224246 | 0.014370309 | 0.048346958 | down |
| CST8 | -3.211084623 | 0.04686662 | 0.11658062 | down |
| RP1-21O18.3 | -3.210804042 | 0.001141855 | 0.006741184 | down |
| RP11-680E19.1 | -3.210168574 | 0.016882012 | 0.054537975 | down |
| CDH7 | -3.210045955 | 0.000386104 | 0.002841078 | down |
| CTD-2540B15.9 | -3.209206628 | 0.000185249 | 0.001571763 | down |
| RP11-65N13.8 | -3.207963351 | 0.054237713 | 0.129723262 | down |
| CRYGD | -3.206679234 | 0.053748173 | 0.128865224 | down |
| NPM1P30 | -3.206446875 | 0.007296615 | 0.02886366 | down |
| NA | -3.206414636 | 0.094514529 | 0.194266574 | down |
| TMPOP1 | -3.20614997 | 0.00106473 | 0.006376073 | down |
| C17orf50 | -3.203862038 | 9.93E-08 | 2.82E-06 | down |
| NA | -3.203738237 | 0.079174607 | 0.17089386 | down |
| NA | -3.202856154 | 0.09984306 | 0.201686607 | down |
| RP11-498D10.3 | -3.202202809 | 0.007892206 | 0.030658578 | down |
| CTB-39G8.2 | -3.202090064 | 0.04070838 | 0.105271351 | down |
| GUCY2F | -3.201003625 | 0.01154819 | 0.040991336 | down |
| HDAC1P1 | -3.200853376 | 0.074234524 | 0.163093384 | down |
| PRSS56 | -3.200766012 | 0.024727878 | 0.072693185 | down |
| CTB-118P15.2 | -3.200550524 | 0.074823543 | 0.164075507 | down |
| NA | -3.199989216 | 0.016068259 | 0.052587667 | down |
| AC027612.4 | -3.199839644 | 0.001329171 | 0.00758444 | down |
| NA | -3.19870467 | 0.028601145 | 0.081088308 | down |
| RP11-115J16.2 | -3.198308037 | 0.000809107 | 0.005126615 | down |
| NA | -3.198185383 | 0.000484951 | 0.003393867 | down |
| RP11-404L6.2 | -3.196513643 | 0.131669884 | 0.245509215 | down |
| ZNF488 | -3.196438813 | 8.13E-09 | 3.30E-07 | down |
| Y_RNA | -3.196112451 | 0.038665209 | 0.101271394 | down |
| RP11-72I2.1 | -3.195360706 | 0.100373184 | 0.202498787 | down |
| A2ML1-AS1 | -3.194853753 | 0.073591558 | 0.162185459 | down |
| OR7A17 | -3.194133379 | 0.033382032 | 0.090708447 | down |
| RP4-715N11.2 | -3.194020347 | 0.001681808 | 0.00913546 | down |
| NPHS2 | -3.193486006 | 0.001887132 | 0.010019448 | down |
| PRB4 | -3.192904473 | 0.008454346 | 0.032241987 | down |
| MBD3L1 | -3.192660193 | 0.068105211 | 0.15329285 | down |
| RP11-654D12.3 | -3.192455956 | 0.022426019 | 0.067538824 | down |
| ATP2B2 | -3.192278078 | 2.48E-08 | 8.49E-07 | down |
| NCR2 | -3.191664894 | 7.27E-05 | 0.00072385 | down |
| RP11-193J6.1 | -3.189934848 | 0.000292804 | 0.00228497 | down |
| TRPM1 | -3.189166696 | 0.001708893 | 0.009265834 | down |

| | | | | |
|---|---|---|---|---|
| NUP58P1 | -3.187486609 | 0.106436816 | 0.211333037 | down |
| KLC3 | -3.187359153 | 0.000499077 | 0.003469291 | down |
| LINC00411 | -3.186661162 | 0.017993078 | 0.057369795 | down |
| AC144450.2 | -3.185740109 | 0.128540565 | 0.241412312 | down |
| AP000459.7 | -3.185535556 | 0.060564157 | 0.140775941 | down |
| RP11-448N11.2 | -3.185218375 | 0.098243976 | 0.199489188 | down |
| LINC00456 | -3.184364445 | 7.16E-05 | 0.000714563 | down |
| RP11-744H18.1 | -3.184080025 | 0.21710113 | 0.348354324 | down |
| RP11-341D18.5 | -3.184050291 | 0.029914102 | 0.083588104 | down |
| EIF3EP2 | -3.183954516 | 0.015019669 | 0.049989026 | down |
| PKHD1 | -3.182677284 | 3.97E-08 | 1.28E-06 | down |
| RP3-499B10.3 | -3.182293164 | 0.021228206 | 0.064900806 | down |
| RP11-767I20.1 | -3.181085022 | 0.019566756 | 0.061019681 | down |
| RP11-26J3.1 | -3.180826614 | 0.000411604 | 0.002974081 | down |
| CTB-32H22.1 | -3.180300283 | 0.218836543 | 0.350259715 | down |
| KPRP | -3.179884653 | 0.27349945 | 0.409764996 | down |
| RP11-627G18.4 | -3.179219669 | 0.005842238 | 0.024453079 | down |
| SERPINA7 | -3.179152389 | 0.010456622 | 0.037964805 | down |
| RP11-7F17.5 | -3.178242227 | 2.77E-07 | 6.81E-06 | down |
| TUBB3P1 | -3.178137386 | 0.098498419 | 0.199870761 | down |
| RP11-565A3.1 | -3.17657534 | 0.217919356 | 0.349312763 | down |
| ASIC2 | -3.17497696 | 3.91E-08 | 1.27E-06 | down |
| PLG | -3.173567474 | 0.00304395 | 0.014667576 | down |
| RN7SL442P | -3.172880861 | 0.008553328 | 0.032526561 | down |
| NA | -3.172050237 | 0.100366671 | 0.202498787 | down |
| KRT40 | -3.171961858 | 7.58E-05 | 0.000751001 | down |
| ZNF587P1 | -3.171852073 | 0.000168974 | 0.001454626 | down |
| PPP4R3CP | -3.171599767 | 0.276642293 | 0.412867769 | down |
| CHRM5 | -3.171526589 | 7.08E-07 | 1.52E-05 | down |
| RP11-244B22.11 | -3.171233659 | 0.044776897 | 0.112795973 | down |
| RP11-231E4.2 | -3.171220508 | 0.055180849 | 0.131369878 | down |
| TGIF2LX | -3.170984601 | 0.276735819 | 0.412966322 | down |
| RP4-640H8.2 | -3.170955347 | 0.01545873 | 0.051133115 | down |
| RN7SL580P | -3.170325802 | 0.020452373 | 0.063139241 | down |
| RP11-925D8.1 | -3.169108137 | 0.024287579 | 0.0717428 | down |
| RP4-530I15.6 | -3.169024655 | 0.00020983 | 0.001739071 | down |
| MTCO1P49 | -3.168111421 | 0.131838297 | 0.245716489 | down |
| RN7SL814P | -3.167896692 | 0.2771268 | 0.413299028 | down |
| LINC01230 | -3.167830538 | 0.00016316 | 0.001413896 | down |
| RP11-449H3.2 | -3.16775945 | 0.01463369 | 0.049002964 | down |
| LINC00691 | -3.166557776 | 0.136664432 | 0.251973927 | down |
| SNORA3 | -3.166528187 | 0.07383536 | 0.16251352 | down |
| MARK2P12 | -3.166511173 | 0.03674691 | 0.097528637 | down |
| FREM2 | -3.166096013 | 3.06E-05 | 0.000355422 | down |
| CLEC2A | -3.165953182 | 0.277461128 | 0.413617254 | down |
| TAT | -3.165098036 | 1.44E-08 | 5.51E-07 | down |
| NA | -3.164163648 | 0.001584322 | 0.008717831 | down |
| RP11-407B7.1 | -3.164067232 | 1.35E-08 | 5.20E-07 | down |
| CTA-414D7.1 | -3.163891752 | 7.57E-07 | 1.60E-05 | down |
| CTB-191D16.1 | -3.162452665 | 0.075118677 | 0.164482583 | down |
| RP13-60M5.2 | -3.161380318 | 0.011022538 | 0.039519981 | down |

| | | | | |
|---|---|---|---|---|
| LA16c-381G6.1 | -3.161349508 | 0.101456288 | 0.204135596 | down |
| HOXC6 | -3.160619015 | 3.23E-07 | 7.74E-06 | down |
| ELMO2P1 | -3.159940201 | 0.066871119 | 0.151149254 | down |
| CTC-265F19.2 | -3.159247877 | 4.93E-06 | 7.85E-05 | down |
| RP11-729I10.2 | -3.158658562 | 0.138372606 | 0.254282621 | down |
| RP11-136O12.2 | -3.158553368 | 0.004290913 | 0.019247022 | down |
| GOLGA8G | -3.157914068 | 0.003372786 | 0.015878641 | down |
| RP11-60C6.5 | -3.157784988 | 0.065104281 | 0.148125181 | down |
| NA | -3.157205585 | 0.035115235 | 0.094297652 | down |
| HLA-N | -3.156098909 | 0.016716513 | 0.054148886 | down |
| RN7SL697P | -3.155992634 | 0.219518367 | 0.350958376 | down |
| UGT1A3 | -3.155530158 | 0.051249396 | | down |
| CTD-2623N2.5 | -3.155516546 | 0.00598569 | 0.024907767 | down |
| NA | -3.154939371 | 0.133116205 | 0.24728385 | down |
| RP11-86H7.6 | -3.154433666 | 0.026969383 | 0.077574739 | down |
| Y_RNA | -3.154424862 | 0.065150104 | 0.14820699 | down |
| LETM1P2 | -3.153463887 | 0.048859046 | 0.120378251 | down |
| AC068538.4 | -3.153247466 | 0.05743056 | 0.135283047 | down |
| MYBPHL | -3.152047339 | 0.009670425 | 0.035705819 | down |
| RP11-700H13.1 | -3.15197626 | 0.279556605 | 0.415812597 | down |
| RP11-60H5.1 | -3.151339921 | 0.108705419 | 0.21470946 | down |
| RP11-78O7.1 | -3.150030785 | 0.006301226 | 0.025876713 | down |
| PGAM1P3 | -3.149439866 | 0.083023959 | 0.177059185 | down |
| NA | -3.148952825 | 0.023981313 | 0.071061699 | down |
| RP11-613F22.7 | -3.148888937 | 0.090047937 | 0.187573762 | down |
| RP11-522B15.1 | -3.148855153 | 0.002907624 | 0.014144535 | down |
| NA | -3.148852003 | 0.006959689 | 0.027875871 | down |
| NALCN-AS1 | -3.148220129 | 0.00218827 | 0.011276911 | down |
| AF224669.3 | -3.147069552 | 0.060726903 | 0.140995292 | down |
| CARD18 | -3.146642013 | 0.173715157 | | down |
| TRIML2 | -3.146283592 | 0.007913751 | 0.030714471 | down |
| Y_RNA | -3.145952979 | 0.280559768 | | down |
| NIPA2P4 | -3.145604232 | 0.141758058 | 0.258460262 | down |
| OR2M3 | -3.145297713 | 0.072119869 | 0.159755905 | down |
| RP11-389J22.1 | -3.144637686 | 0.046600065 | 0.116083028 | down |
| RP11-55L4.1 | -3.144194735 | 1.18E-06 | 2.34E-05 | down |
| NA | -3.142938916 | 5.44E-12 | 5.08E-10 | down |
| RNA5SP427 | -3.142852465 | 0.102984642 | 0.206225662 | down |
| OR1I1 | -3.142461702 | 0.000522228 | 0.00360437 | down |
| ESPNP | -3.142272554 | 4.97E-07 | 1.12E-05 | down |
| ATP6V1G1P4 | -3.140598307 | 0.10973199 | 0.216125392 | down |
| OR51I2 | -3.138683157 | 0.280914042 | | down |
| RP1-270O5.3 | -3.13861923 | 0.013463811 | 0.045961452 | down |
| AC011288.2 | -3.137559562 | 0.000121355 | 0.001112598 | down |
| OR1D2 | -3.136857825 | 0.058600085 | 0.137357204 | down |
| RP11-260N14.1 | -3.136738388 | 0.001433808 | 0.008054621 | down |
| RP11-113K21.1 | -3.136688427 | 0.03489499 | 0.093840412 | down |
| RP11-778J15.1 | -3.136339637 | 0.05959744 | 0.138974347 | down |
| RP3-329A5.1 | -3.136277439 | 0.011062762 | 0.039629144 | down |
| RP11-81H3.2 | -3.13620973 | 0.137522721 | 0.25304585 | down |
| RP11-59H7.1 | -3.136153259 | 0.002872439 | 0.014014218 | down |

| | | | | |
|---|---|---|---|---|
| RP11-484L7.1 | -3.135414331 | 0.020084615 | 0.062291955 | down |
| RP11-661D19.3 | -3.135403027 | 0.016735158 | 0.054191753 | down |
| CTD-2659N19.4 | -3.13484947 | 5.48E-05 | 0.000575858 | down |
| CTD-2012B7.1 | -3.134148925 | 0.029993173 | 0.083777881 | down |
| RP11-444A22.1 | -3.134026106 | 0.001945662 | 0.010237383 | down |
| RP11-241G9.3 | -3.133968685 | 0.225273778 | 0.357405038 | down |
| CPA2 | -3.133527323 | 1.91E-07 | 4.97E-06 | down |
| GNAT3 | -3.133388235 | 0.07386301 | | down |
| AC072062.1 | -3.133342662 | 6.61E-06 | 0.000100371 | down |
| RP11-55609.2 | -3.131927735 | 7.59E-05 | 0.000751085 | down |
| RPL23AP12 | -3.131635007 | 0.003949727 | 0.018056238 | down |
| LINC01499 | -3.130941843 | 0.180360085 | 0.305642641 | down |
| OR1L8 | -3.129379272 | 9.60E-05 | 0.000913577 | down |
| RP11-784B15.1 | -3.129032132 | 0.064407905 | 0.147097764 | down |
| Metazoa_SRP | -3.128652402 | 0.004616228 | 0.020398581 | down |
| OR2C3 | -3.127893003 | 0.008500795 | 0.032373925 | down |
| RP11-238K6.1 | -3.127847171 | 0.000243329 | 0.001967322 | down |
| AC103564.6 | -3.1271294 | 0.108627316 | 0.214625746 | down |
| RP4-552O12.1 | -3.12690356 | 0.282776292 | | down |
| LINC01602 | -3.126386193 | 0.143935527 | 0.261371955 | down |
| OR4C1P | -3.125931269 | 0.108679448 | 0.214700508 | down |
| RP11-791G16.2 | -3.125711689 | 0.000282544 | 0.002222189 | down |
| Y_RNA | -3.125577885 | 0.111788272 | 0.218927505 | down |
| SATL1 | -3.125422979 | 0.0119534 | 0.042091883 | down |
| KCNJ9 | -3.125153979 | 2.40E-05 | 0.000291335 | down |
| RP11-396O20.2 | -3.122788889 | 1.38E-09 | 6.83E-08 | down |
| RP11-35G22.1 | -3.122778219 | 0.003211553 | 0.015274323 | down |
| LINC01551 | -3.121763017 | 0.011363627 | 0.040480056 | down |
| RP11-162A12.3 | -3.121580872 | 0.023428274 | 0.069766979 | down |
| NA | -3.119611796 | 0.012788351 | 0.044208172 | down |
| RN7SL455P | -3.118041459 | 0.011717075 | 0.041444291 | down |
| IL1RAPL1 | -3.117789201 | 1.16E-05 | 0.000159722 | down |
| DLX3 | -3.116963029 | 1.25E-06 | 2.47E-05 | down |
| NA | -3.116478983 | 0.003576581 | 0.01666224 | down |
| MT-TY | -3.116271229 | 7.55E-07 | 1.60E-05 | down |
| RP11-826N14.1 | -3.115890691 | 0.004031215 | 0.01835582 | down |
| RP11-243M5.4 | -3.115390063 | 0.026226752 | 0.075962768 | down |
| SPATS1 | -3.114343479 | 0.000251693 | 0.002022429 | down |
| LAMB4 | -3.114287445 | 4.34E-07 | 9.94E-06 | down |
| AC009961.5 | -3.113489622 | 0.000141537 | 0.001260329 | down |
| RNU11-2P | -3.11305527 | 0.181274231 | 0.306724656 | down |
| AC007919.19 | -3.112977365 | 0.147119777 | 0.265469464 | down |
| RP11-420J11.1 | -3.112945688 | 0.109343834 | 0.215589336 | down |
| UMOD | -3.112752637 | 0.073605224 | 0.162185459 | down |
| RP11-338E21.2 | -3.111751312 | 1.05E-07 | 2.96E-06 | down |
| RP11-476K15.1 | -3.111416609 | 0.004311558 | 0.019322263 | down |
| CTC-277H1.6 | -3.111286413 | 2.98E-09 | 1.36E-07 | down |
| CTA-221G9.11 | -3.110814173 | 0.007936155 | 0.030774672 | down |
| RP5-1004I9.1 | -3.110148218 | 0.012485389 | 0.043430708 | down |
| RP11-649A16.1 | -3.110033632 | 0.021495912 | 0.065459475 | down |
| RP11-3P22.2 | -3.110012672 | 0.013559277 | 0.046238616 | down |

| | | | | |
|---|---|---|---|---|
| RNU7-10P | -3.109834396 | 0.0036799 | 0.017045778 | down |
| RP11-638L3.4 | -3.109236772 | 0.032699137 | 0.089350875 | down |
| LINC01225 | -3.108833526 | 0.072810072 | 0.160930086 | down |
| OR2AT1P | -3.107919363 | 0.05105415 | 0.124200071 | down |
| OR7G2 | -3.107837744 | 0.112013127 | 0.219121564 | down |
| NA | -3.106088909 | 0.000532498 | 0.003663469 | down |
| RP11-589M2.1 | -3.105942443 | 0.28674622 | | down |
| RP1-125N5.2 | -3.105642495 | 0.016149842 | 0.052797155 | down |
| AC002306.1 | -3.105128313 | 0.000251459 | 0.002021091 | down |
| AP000230.1 | -3.10494884 | 2.93E-05 | 0.000343318 | down |
| RP11-717F1.1 | -3.104919617 | 0.286905569 | | down |
| FAM230C | -3.104399814 | 0.01291964 | 0.044543078 | down |
| RP11-357H14.17 | -3.104128031 | 1.59E-13 | 2.23E-11 | down |
| RP11-527L4.2 | -3.103878766 | 1.02E-05 | 0.000143051 | down |
| RP11-1072C15.7 | -3.103685846 | 0.144750395 | 0.262312617 | down |
| RPL31P35 | -3.102227793 | 0.085207782 | 0.18035529 | down |
| RP11-33E15.1 | -3.102039208 | 0.287354637 | | down |
| NUTM2F | -3.102001101 | 0.010960806 | 0.03936718 | down |
| RP11-63E9.1 | -3.101690674 | 0.086182688 | 0.181857241 | down |
| PDXDC2P | -3.10166135 | 7.90E-06 | 0.000116052 | down |
| RAP2CP1 | -3.10153316 | 0.046264227 | 0.115435798 | down |
| GCSHP2 | -3.101259793 | 0.047703689 | 0.118158037 | down |
| NA | -3.101058282 | 0.008595493 | 0.032655918 | down |
| DPP10-AS1 | -3.10015642 | 1.36E-08 | 5.23E-07 | down |
| LINC00320 | -3.099743016 | 0.011905906 | 0.041944306 | down |
| KCNJ6 | -3.098693258 | 6.66E-11 | 4.74E-09 | down |
| AACSP1 | -3.098580326 | 0.010072846 | 0.03688793 | down |
| LINC01107 | -3.098260159 | 0.182530506 | 0.308222628 | down |
| NA | -3.098127244 | 0.006148894 | 0.025424908 | down |
| UGT2B26P | -3.097377385 | 0.288082439 | | down |
| ABCB10P1 | -3.096993454 | 0.231351924 | | down |
| MSGN1 | -3.096040127 | 0.070274766 | 0.156801713 | down |
| CDHR5 | -3.095687937 | 4.93E-12 | 4.66E-10 | down |
| NR1I3 | -3.095572067 | 1.28E-12 | 1.38E-10 | down |
| AC017006.2 | -3.0955053 | 0.025234457 | 0.073828417 | down |
| CTB-78F1.1 | -3.095163291 | 0.005430207 | 0.023120115 | down |
| RP11-624D20.1 | -3.094684662 | 0.021322435 | 0.065076315 | down |
| RP11-61L19.1 | -3.093820047 | 3.91E-05 | 0.000436672 | down |
| MYL1 | -3.093701011 | 0.18116367 | 0.306658477 | down |
| AC010150.1 | -3.093435285 | 1.02E-06 | 2.06E-05 | down |
| KCNJ3 | -3.093251396 | 1.27E-14 | 2.29E-12 | down |
| U47924.29 | -3.092780746 | 0.000323844 | 0.002479505 | down |
| UGT2A3 | -3.091435331 | 4.90E-06 | 7.81E-05 | down |
| CTD-2085J24.3 | -3.090415848 | 0.016679081 | 0.054062607 | down |
| RPL21P136 | -3.090254515 | 0.001726501 | 0.009349495 | down |
| NA | -3.089855733 | 0.005645933 | 0.02378785 | down |
| EN2 | -3.089498481 | 0.000626008 | 0.00417394 | down |
| RP11-636N17.1 | -3.088905484 | 0.182765035 | 0.308517401 | down |
| OR5H14 | -3.087715175 | 0.289594859 | | down |
| ANKRD18B | -3.087703023 | 0.007958116 | 0.030846813 | down |
| LINC01285 | -3.087171226 | 6.31E-07 | 1.38E-05 | down |

| | | | | |
|---|---|---|---|---|
| LINC00453 | -3.086660589 | 0.288466368 | 0.425609368 | down |
| AC092625.1 | -3.085966405 | 0.035602698 | 0.095206716 | down |
| Y_RNA | -3.08426952 | 0.006110707 | 0.025305316 | down |
| CTD-2116N17.1 | -3.083144751 | 0.007921599 | 0.030740961 | down |
| RP11-90D4.3 | -3.083142146 | 0.019419331 | 0.060736454 | down |
| RP11-396C23.3 | -3.083126133 | 0.003838115 | 0.017642644 | down |
| CTD-2184D3.3 | -3.082455489 | 0.006438655 | 0.026318771 | down |
| RNA5SP303 | -3.08186116 | 0.089378135 | 0.186671782 | down |
| RP11-27G24.3 | -3.081573741 | 0.234291795 | 0.367048184 | down |
| CTD-2621I17.6 | -3.081363893 | 0.051945954 | 0.125627001 | down |
| RP11-53A1.3 | -3.08131569 | 0.143026964 | 0.26017382 | down |
| BHLHA9 | -3.080826377 | 0.044378999 | 0.112119293 | down |
| RP11-366G5.1 | -3.079816436 | 0.09470008 | 0.194490562 | down |
| NA | -3.079567902 | 0.000251973 | 0.002024138 | down |
| LINC00485 | -3.079401452 | 0.141888751 | 0.258655762 | down |
| SIX6 | -3.078923174 | 0.290975705 | | down |
| RP11-23E10.3 | -3.078708497 | 0.020750637 | 0.063810864 | down |
| AC012363.13 | -3.078282289 | 0.000964519 | 0.005885057 | down |
| RP11-71L14.3 | -3.077089828 | 1.09E-06 | 2.18E-05 | down |
| AP003900.6 | -3.077061927 | 0.291268594 | | down |
| SOWAHA | -3.076743521 | 8.56E-18 | 3.14E-15 | down |
| HIGD1AP9 | -3.076415164 | 0.066863569 | 0.15114356 | down |
| CTC-575N7.1 | -3.076197562 | 0.001462157 | 0.00817353 | down |
| RP5-1107A17.3 | -3.075994715 | 0.001144322 | 0.006754422 | down |
| RP11-69C17.4 | -3.075874076 | 0.141337732 | 0.258042709 | down |
| NA | -3.07553975 | 0.01812795 | 0.057706732 | down |
| RP11-716H6.2 | -3.074960019 | 0.116903461 | 0.225853488 | down |
| GABRR3 | -3.074775234 | 0.019295066 | 0.060461094 | down |
| TM4SF5 | -3.074510303 | 9.03E-12 | 7.98E-10 | down |
| RNU6-35P | -3.0744417 | 0.013562988 | 0.046242216 | down |
| RP11-49006.2 | -3.074182972 | 3.02E-07 | 7.30E-06 | down |
| NA | -3.073749155 | 0.065259643 | 0.148377532 | down |
| POTEM | -3.073182209 | 0.00096528 | 0.005887535 | down |
| ADAM30 | -3.072789215 | 0.289077984 | 0.426344261 | down |
| RPL29P30 | -3.072240163 | 0.03684034 | 0.097655829 | down |
| LYPD2 | -3.070061595 | 0.011512901 | 0.040880935 | down |
| OR7C1 | -3.069641502 | 3.79E-06 | 6.30E-05 | down |
| C4orf51 | -3.069488511 | 0.005639535 | 0.023773565 | down |
| RP11-1079K10.1 | -3.069111737 | 0.043412283 | 0.110479801 | down |
| LINC01520 | -3.068265052 | 0.081501037 | 0.174527239 | down |
| WNT8A | -3.068235484 | 0.026174718 | 0.075848111 | down |
| RP11-557F20.2 | -3.065932045 | 0.293024151 | | down |
| RP11-359K18.4 | -3.065569078 | 3.99E-06 | 6.60E-05 | down |
| CTSLP8 | -3.065371911 | 0.00029736 | 0.00231628 | down |
| RP11-620J15.2 | -3.06486176 | 1.03E-06 | 2.08E-05 | down |
| GMCL1P1 | -3.064374878 | 0.010239832 | 0.0373367 | down |
| AC012497.2 | -3.063827705 | 0.293356873 | | down |
| NA | -3.063425572 | 0.077006886 | 0.167506145 | down |
| CTD-2311A18.1 | -3.063372816 | 0.186217855 | 0.312502331 | down |
| SNORA70 | -3.063249298 | 0.040506361 | 0.104929567 | down |
| RP11-379I19.1 | -3.062853734 | 0.153598273 | 0.273301619 | down |

| | | | | |
|---|---|---|---|---|
| RP11-408H1.3 | -3.06222324 | 5.94E-05 | 0.000614979 | down |
| CTB-52I2.3 | -3.060328814 | 0.005365232 | 0.022896737 | down |
| C14orf39 | -3.060316656 | 0.013308518 | 0.045544082 | down |
| RP13-379O24.3 | -3.060026995 | 0.021368974 | | down |
| ATP5LP3 | -3.059949018 | 0.004014298 | 0.018298521 | down |
| SSTR5 | -3.059102571 | 0.002487969 | 0.012518596 | down |
| RNU6-662P | -3.057796535 | 0.121068786 | 0.231577679 | down |
| AC009313.2 | -3.055231915 | 0.236510118 | | down |
| NA | -3.055100512 | 2.45E-05 | 0.00029555 | down |
| RP11-309H21.3 | -3.054323011 | 0.095225385 | 0.195193524 | down |
| AC016700.2 | -3.053110619 | 0.239858317 | | down |
| CTD-2586B10.1 | -3.052343749 | 0.029407339 | 0.082664117 | down |
| RP5-921G16.1 | -3.052186358 | 0.001310416 | 0.007503648 | down |
| GRM5-AS1 | -3.052105263 | 0.009436283 | 0.035069729 | down |
| CACNA1S | -3.051590921 | 5.07E-05 | 0.000541172 | down |
| CTC-448F2.7 | -3.048775116 | 0.295744268 | | down |
| LINC01320 | -3.048410425 | 4.01E-05 | 0.000444861 | down |
| RP11-259K15.2 | -3.048269107 | 0.020850253 | 0.064018907 | down |
| C1orf168 | -3.046421241 | 2.20E-05 | 0.000270056 | down |
| GOLGA6A | -3.045587086 | 0.011999841 | 0.042201008 | down |
| NA | -3.045238529 | 0.00036375 | 0.002713806 | down |
| RP11-46H11.2 | -3.044552535 | 0.013077816 | 0.044946862 | down |
| RSPH6A | -3.042857478 | 0.005346622 | 0.022833532 | down |
| CTD-2147F2.2 | -3.042646859 | 0.004482395 | 0.019944034 | down |
| NA | -3.042493817 | 0.053680598 | 0.12876291 | down |
| RP11-728G15.1 | -3.042393174 | 0.025427195 | 0.074204433 | down |
| RP11-753A21.1 | -3.042206523 | 0.150091298 | 0.26906993 | down |
| DKFZP434K028 | -3.041491745 | 0.00012978 | 0.001174431 | down |
| MGAT4EP | -3.041203795 | 0.031795458 | 0.087517813 | down |
| LINC01114 | -3.040705043 | 0.296985216 | | down |
| NA | -3.040548766 | 0.03300252 | 0.089983456 | down |
| RPS29P24 | -3.040303873 | 0.039965318 | 0.103899449 | down |
| KCNU1 | -3.039194374 | 0.010602055 | 0.038372261 | down |
| SIGLEC21P | -3.039147507 | 0.297278045 | | down |
| FDPSP7 | -3.038840464 | 0.043135823 | 0.109942071 | down |
| AP003385.2 | -3.038645422 | 0.002735948 | 0.013503998 | down |
| RP11-30L3.2 | -3.038570012 | 0.000172738 | 0.001481512 | down |
| NA | -3.038205127 | 0.058768073 | 0.137638603 | down |
| Z83001.1 | -3.037718698 | 1.88E-06 | 3.52E-05 | down |
| FLJ36000 | -3.036698078 | 0.23967685 | | down |
| NA | -3.036670997 | 0.007649071 | 0.029962922 | down |
| CTD-2210P24.1 | -3.035543615 | 0.007982411 | 0.030925025 | down |
| AC006004.1 | -3.034554315 | 0.242916421 | | down |
| NDUFS5P5 | -3.032287013 | 0.091464028 | 0.189631167 | down |
| RP11-91I20.4 | -3.031859448 | 0.024953233 | 0.073183808 | down |
| NA | -3.031527112 | 0.241599639 | | down |
| NA | -3.031376089 | 0.002158756 | 0.01116311 | down |
| RP1-172I22.1 | -3.030721877 | 0.298624692 | | down |
| NA | -3.030469945 | 9.87E-06 | 0.000139688 | down |
| LHX9 | -3.029692399 | 0.000617399 | 0.004129369 | down |
| RPL36P16 | -3.028997897 | 0.068118039 | 0.153310238 | down |

| | | | | |
|---|---|---|---|---|
| CTC-218B8.3 | -3.028070144 | 0.002556826 | 0.012796482 | down |
| APOA5 | -3.027004921 | 0.112513962 | | down |
| MTATP6P21 | -3.026468577 | 0.118837311 | 0.228516908 | down |
| NA | -3.025131267 | 8.18E-05 | 0.000800919 | down |
| KRT4 | -3.024416096 | 0.116478741 | | down |
| PLCE1-AS1 | -3.023684032 | 0.008256152 | 0.031675115 | down |
| RP11-532F6.4 | -3.022020877 | 0.000103091 | 0.000970926 | down |
| FANCD2P2 | -3.022015049 | 0.040391287 | 0.104703695 | down |
| CATSPER4 | -3.021816389 | 8.58E-05 | 0.000831945 | down |
| NA | -3.020937367 | 0.077064568 | 0.167583084 | down |
| FTH1P1 | -3.019994778 | 5.90E-05 | 0.000612057 | down |
| AP000470.2 | -3.019902158 | 0.295663599 | 0.433153653 | down |
| CTA-992D9.11 | -3.019619844 | 0.004136626 | 0.018733703 | down |
| THAP12P8 | -3.019016872 | 0.243318319 | | down |
| CTB-186H2.3 | -3.017994419 | 0.000252956 | 0.002029863 | down |
| RP11-76N22.1 | -3.016798582 | 0.083299087 | 0.177416544 | down |
| AC010148.1 | -3.015179309 | 3.71E-05 | 0.00041945 | down |
| NA | -3.015109703 | 0.151230583 | | down |
| Y_RNA | -3.014840245 | 0.301174075 | | down |
| CDH20 | -3.014682053 | 2.38E-05 | 0.000288526 | down |
| NDUFA3P6 | -3.013800961 | 0.082414064 | 0.176080794 | down |
| SH2D4B | -3.013222502 | 9.40E-05 | 0.000897865 | down |
| RP11-497G19.1 | -3.012780879 | 0.006173774 | 0.025503215 | down |
| RP11-9D8.1 | -3.012774871 | 0.009167855 | 0.03431407 | down |
| CASP16P | -3.012619721 | 5.95E-11 | 4.33E-09 | down |
| RNA5SP30 | -3.012438191 | 0.197645251 | 0.326316781 | down |
| AC114783.1 | -3.01237218 | 0.242318325 | 0.376238334 | down |
| RP11-363J20.1 | -3.011764122 | 0.000314395 | 0.002421346 | down |
| SLC25A15P3 | -3.011351608 | 0.010380829 | | down |
| MIR7515HG | -3.009866704 | 0.08991717 | | down |
| SMIM22 | -3.009639876 | 6.27E-09 | 2.60E-07 | down |
| RP11-7F17.1 | -3.009530596 | 0.002293337 | 0.01174182 | down |
| NA | -3.009499802 | 0.024921514 | 0.073112193 | down |
| RP11-447M12.3 | -3.00803416 | 0.197380066 | 0.325990144 | down |
| RP11-203E14.1 | -3.00781361 | 0.195226209 | | down |
| NA | -3.006586764 | 1.46E-06 | 2.84E-05 | down |
| AQP4-AS1 | -3.00630347 | 0.00123049 | 0.007150647 | down |
| PRR23A | -3.005178319 | 0.123740206 | 0.2350266 | down |
| RP11-109E12.5 | -3.004621028 | 0.0019274 | | down |
| RALYL | -3.004225812 | 1.27E-06 | 2.49E-05 | down |
| IGF2BP1 | -3.003953923 | 5.49E-08 | 1.71E-06 | down |
| PRDM13 | -3.003104652 | 0.032206922 | 0.08837563 | down |
| RP11-34P1.4 | -3.002256564 | 0.09807112 | 0.199232451 | down |
| RN7SL513P | -3.001509223 | 0.199973546 | 0.32900746 | down |
| MME | -3.0014615 | 4.32E-11 | 3.28E-09 | down |
| RP11-90E5.1 | -3.000762357 | 0.16351745 | 0.285756064 | down |
| SMYD3-IT1 | -2.999618523 | 0.059354189 | 0.138600752 | down |
| NA | -2.998930435 | 6.63E-07 | 1.44E-05 | down |
| HOXD12 | -2.998874616 | 0.01241372 | 0.043241485 | down |
| NA | -2.998592458 | 0.200865204 | 0.329945933 | down |
| RPL12P21 | -2.99801418 | 0.000376444 | 0.002783515 | down |

| | | | | |
|---|---|---|---|---|
| TYR | -2.997974518 | 0.119980942 | | down |
| COX7A2P2 | -2.997258769 | 0.122476841 | 0.233320285 | down |
| CTC-359M8.1 | -2.995090976 | 0.126792884 | 0.238982317 | down |
| CACNG2 | -2.993429025 | 0.004022427 | 0.018318904 | down |
| CSNK1A1P1 | -2.992304187 | 0.005727464 | 0.024067152 | down |
| TPT1P3 | -2.99209885 | 0.155203157 | 0.275293638 | down |
| C17orf105 | -2.992066393 | 0.014625214 | 0.048985499 | down |
| GABRA5 | -2.991465784 | 0.002750121 | 0.013553909 | down |
| LINC01104 | -2.991448337 | 0.006454216 | 0.026371614 | down |
| NA | -2.991377707 | 0.121714183 | 0.232397897 | down |
| RP11-475D12.2 | -2.990459835 | 0.044301591 | 0.111961391 | down |
| MOG | -2.990441496 | 0.010448943 | 0.037946094 | down |
| RP11-810P12.5 | -2.990392541 | 0.043574419 | 0.110757178 | down |
| LINC01214 | -2.989349661 | 0.000277461 | 0.002189905 | down |
| NA | -2.989089256 | 0.248195918 | | down |
| RP11-382N13.2 | -2.98842418 | 0.126647928 | 0.238882639 | down |
| NA | -2.988274567 | 3.39E-09 | 1.52E-07 | down |
| RP11-845C23.2 | -2.987514238 | 0.004858454 | 0.021219273 | down |
| AC002984.2 | -2.987242021 | 0.00245613 | 0.012397846 | down |
| NA | -2.987164841 | 0.000264483 | 0.002105493 | down |
| Y_RNA | -2.986565039 | 9.43E-06 | 0.000134712 | down |
| RP11-700A24.1 | -2.986502635 | 2.93E-05 | 0.000343527 | down |
| CTC-523E23.15 | -2.985639369 | 0.305853137 | | down |
| LINC00575 | -2.985330199 | 0.30594954 | | down |
| KRT8P15 | -2.984959074 | 0.043093127 | 0.109859134 | down |
| GIMD1 | -2.983753136 | 0.047926644 | 0.118589038 | down |
| RP11-214J9.1 | -2.983730319 | 0.002202187 | 0.011340846 | down |
| NA | -2.983472003 | 0.123318072 | 0.234447141 | down |
| LRP4-AS1 | -2.983430712 | 9.87E-07 | 2.00E-05 | down |
| CCDC26 | -2.983410716 | 5.84E-08 | 1.81E-06 | down |
| CTD-2194L12.2 | -2.9832481 | 0.198445486 | 0.327209404 | down |
| SCT | -2.982941275 | 3.75E-15 | 7.51E-13 | down |
| CTA-125H2.2 | -2.98291987 | 0.200689135 | 0.329753575 | down |
| RP11-278H7.5 | -2.98270842 | 0.001103807 | 0.006566885 | down |
| RN7SL318P | -2.981922909 | 0.017049774 | 0.054955842 | down |
| RP11-24D15.1 | -2.981836628 | 0.005661492 | 0.023835257 | down |
| NA | -2.981189176 | 0.306623657 | | down |
| GDPD2 | -2.980890558 | 1.26E-11 | 1.08E-09 | down |
| AC016735.3 | -2.980861739 | 0.25132055 | | down |
| GATA4 | -2.980844693 | 0.000590648 | 0.00398237 | down |
| NA | -2.979762314 | 0.033608305 | 0.091262464 | down |
| RNU6-993P | -2.979661886 | 0.249620751 | 0.384236697 | down |
| DEFB131 | -2.979246118 | 0.063585273 | | down |
| CYP4Z2P | -2.97895227 | 0.057679468 | 0.135787645 | down |
| NA | -2.977718635 | 0.007666825 | 0.03000418 | down |
| LINC01413 | -2.977205562 | 0.00554701 | 0.023482622 | down |
| AP000266.7 | -2.974696079 | 0.047936388 | 0.11860338 | down |
| HOXC-AS1 | -2.974588122 | 1.05E-05 | 0.000147155 | down |
| RP11-798K3.2 | -2.974319807 | 1.15E-11 | 9.96E-10 | down |
| THSD4-AS1 | -2.973823168 | 0.078591345 | 0.169994397 | down |
| RP11-554D20.1 | -2.973720809 | 3.38E-05 | 0.000385338 | down |

| | | | | |
|---|---|---|---|---|
| RP11-85I21.1 | -2.973715879 | 0.051333898 | 0.124678709 | down |
| RP11-271F18.3 | -2.973565965 | 0.020520582 | 0.06330428 | down |
| SLC7A15P | -2.973283326 | 0.161596937 | | down |
| RP11-676F20.4 | -2.972949764 | 0.019125515 | 0.060036252 | down |
| RP11-351A20.1 | -2.971932819 | 0.252727029 | | down |
| RP11-14206.1 | -2.971411799 | 3.07E-05 | 0.000356861 | down |
| RNU6-1046P | -2.971081112 | 0.008832768 | 0.033354911 | down |
| RN7SL45P | -2.970802084 | 0.159670094 | 0.280872201 | down |
| ADAMTS20 | -2.970568048 | 0.00252457 | 0.012674588 | down |
| LINC01077 | -2.970472236 | 0.092087837 | | down |
| NA | -2.970140191 | 0.202646427 | 0.331986981 | down |
| AC009120.4 | -2.969748024 | 0.00077046 | 0.004922573 | down |
| CRAT40 | -2.969718768 | 0.157546493 | | down |
| RNU6-264P | -2.969370611 | 0.099333417 | 0.201062669 | down |
| C9orf62 | -2.969115183 | 0.252530098 | | down |
| CTD-2147F2.1 | -2.967844502 | 0.000339803 | 0.002579345 | down |
| RP11-231E6.1 | -2.967582807 | 5.51E-06 | 8.58E-05 | down |
| RP11-279F6.2 | -2.967021112 | 0.038458488 | 0.100912385 | down |
| RP11-471M2.3 | -2.966865599 | 0.043316309 | 0.110269037 | down |
| CTA-243E7.2 | -2.966775542 | 0.0019262 | 0.010177763 | down |
| GPT | -2.965822963 | 8.92E-12 | 7.93E-10 | down |
| RP11-115A15.2 | -2.965683907 | 0.162167065 | 0.284093794 | down |
| RP11-543G18.1 | -2.96561594 | 0.036593847 | 0.097204339 | down |
| NA | -2.965563543 | 0.047051383 | | down |
| VIPR1-AS1 | -2.965382827 | 2.29E-11 | 1.85E-09 | down |
| MYO1A | -2.965099898 | 4.23E-15 | 8.37E-13 | down |
| SUCLA2P3 | -2.964421563 | 0.023220058 | 0.069298046 | down |
| DIAPH3-AS1 | -2.964411529 | 0.309231245 | 0.44765732 | down |
| CSTL1 | -2.964345641 | 0.059638662 | 0.139038098 | down |
| PSMC1P7 | -2.963133514 | 0.023538061 | 0.070024509 | down |
| RNA5SP125 | -2.962638895 | 0.085845687 | 0.181451515 | down |
| RP11-410N8.3 | -2.961912911 | 0.011780904 | 0.041601489 | down |
| RP11-760D2.9 | -2.961894935 | 0.115309862 | 0.223637564 | down |
| RP11-1079J22.1 | -2.961851604 | 0.25214694 | | down |
| RP11-759A24.2 | -2.961663369 | 0.204036832 | 0.333519183 | down |
| AC114755.7 | -2.961348149 | 0.007706831 | 0.030122638 | down |
| Y_RNA | -2.960670662 | 0.015001984 | 0.049963375 | down |
| RP1-101D8.1 | -2.960659616 | 0.002601715 | 0.012973597 | down |
| NA | -2.960409202 | 0.010079786 | 0.036904349 | down |
| OR52U1P | -2.960132206 | 0.310066738 | | down |
| TMEM82 | -2.959550388 | 5.54E-10 | 3.07E-08 | down |
| VN1R10P | -2.95942912 | 0.121964765 | 0.23265911 | down |
| SP7 | -2.958555297 | 9.82E-05 | 0.000930286 | down |
| LINC01111 | -2.958197738 | 0.017236946 | 0.05541053 | down |
| DAO | -2.956895468 | 0.000100305 | 0.000947958 | down |
| ABCF2P2 | -2.956376238 | 0.093057398 | 0.191965945 | down |
| CCNB3P1 | -2.955262067 | 0.057958629 | 0.136305992 | down |
| CTD-3187F8.12 | -2.955178025 | 0.13860808 | 0.254559641 | down |
| CTD-2008L17.2 | -2.954982664 | 0.001637314 | 0.008948785 | down |
| NA | -2.954923366 | 0.168475026 | 0.291906786 | down |
| CTD-2330K9.2 | -2.954333887 | 9.49E-05 | 0.000904441 | down |

| | | | | |
|---|---|---|---|---|
| RP11-96D1.3 | -2.954244216 | 0.009502875 | 0.035247557 | down |
| MYRFL | -2.954116115 | 8.82E-08 | 2.56E-06 | down |
| CTD-2203A3.1 | -2.954110407 | 0.204815404 | 0.334518836 | down |
| HSPE1P8 | -2.954068051 | 0.02068321 | 0.063655639 | down |
| PDE6A | -2.954020112 | 6.60E-09 | 2.72E-07 | down |
| GOLT1A | -2.953791547 | 9.20E-15 | 1.70E-12 | down |
| RP11-154H17.1 | -2.953717569 | 0.034612427 | 0.093361532 | down |
| TSIX | -2.95371359 | 0.002082277 | 0.010832887 | down |
| SLC51B | -2.9535164 | 4.95E-16 | 1.26E-13 | down |
| GPX5 | -2.953089478 | 0.256435248 | | down |
| KCNQ1DN | -2.952962449 | 0.034953318 | 0.09395522 | down |
| RP3-405J10.2 | -2.952816201 | 6.48E-06 | 9.87E-05 | down |
| RPS2P45 | -2.951969813 | 0.001852073 | 0.009878159 | down |
| DEFA5 | -2.951956752 | 3.03E-06 | 5.24E-05 | down |
| RN7SKP85 | -2.951659677 | 0.311459279 | | down |
| OFCC1 | -2.951128179 | 0.205135076 | 0.334968109 | down |
| FOXE3 | -2.951012698 | 8.26E-06 | 0.000120607 | down |
| RP11-326K13.3 | -2.950841702 | 0.015607951 | 0.051496296 | down |
| NPAP1P6 | -2.950482574 | 0.311560838 | | down |
| RP4-633I8.4 | -2.949973532 | 3.34E-06 | 5.69E-05 | down |
| ASNSP6 | -2.949949896 | 0.081991647 | 0.175427791 | down |
| RP11-750H9.7 | -2.949879647 | 0.002181553 | 0.011250011 | down |
| NA | -2.949641069 | 0.102693263 | 0.205866176 | down |
| KCNQ5-AS1 | -2.949627144 | 0.005013176 | 0.021743305 | down |
| SOX11 | -2.949586786 | 4.67E-07 | 1.05E-05 | down |
| AC020550.7 | -2.949214138 | 0.008042311 | 0.031084933 | down |
| RN7SL426P | -2.949156796 | 0.002718412 | 0.013432892 | down |
| SLC5A1 | -2.948662007 | 4.39E-10 | 2.51E-08 | down |
| RN7SL377P | -2.948593722 | 0.124700128 | | down |
| CTC-512J14.7 | -2.948423941 | 0.016188306 | 0.052905632 | down |
| RP11-1101K5.1 | -2.948388743 | 0.010613405 | 0.038402094 | down |
| RP11-383J24.1 | -2.948077406 | 0.000217291 | 0.001789554 | down |
| RP11-264L1.4 | -2.947836266 | 0.020288647 | 0.062782173 | down |
| NA | -2.94770761 | 0.042118404 | 0.10802504 | down |
| LINC00303 | -2.947609848 | 0.005751501 | 0.024140555 | down |
| AC104389.28 | -2.947546388 | 0.048328377 | 0.119406065 | down |
| RPL36P20 | -2.946607824 | 0.165273856 | 0.288038037 | down |
| NA | -2.946362577 | 0.078368034 | 0.169644011 | down |
| RP11-255M6.1 | -2.946118425 | 0.090061885 | | down |
| SPATA31A7 | -2.946064565 | 0.312288628 | | down |
| PDZD7 | -2.946005758 | 2.20E-14 | 3.69E-12 | down |
| RP11-1902.2 | -2.944600252 | 0.040666694 | 0.105190716 | down |
| CTD-2335A18.2 | -2.943680949 | 0.312774426 | | down |
| RP11-158M2.6 | -2.941895733 | 0.01855665 | 0.058729169 | down |
| HNRNPKP5 | -2.941664211 | 0.001073031 | 0.006414272 | down |
| RP11-134L4.2 | -2.941536089 | 0.022807374 | 0.068358415 | down |
| NBPF7 | -2.941292989 | 1.86E-06 | 3.48E-05 | down |
| TLX1 | -2.941192128 | 4.14E-06 | 6.81E-05 | down |
| RPS27P25 | -2.941094123 | 0.006598914 | 0.026777117 | down |
| RP11-536C10.12 | -2.941048294 | 0.165576124 | | down |
| CDC20B | -2.940731134 | 0.001380647 | 0.007813176 | down |

| | | | | |
|---|---|---|---|---|
| XIAP-AS1 | -2.940602895 | 0.003214126 | 0.015281724 | down |
| AKR1B1P6 | -2.940520543 | 0.04507834 | 0.113389258 | down |
| RN7SL101P | -2.940375709 | 0.071126123 | 0.158123338 | down |
| AC018634.9 | -2.939805161 | 0.167325901 | 0.290442908 | down |
| DISC1FP1 | -2.939785434 | 0.002426537 | 0.012278112 | down |
| AC007364.1 | -2.939742949 | 0.005461589 | 0.023209051 | down |
| HMGB1P48 | -2.939384433 | 0.208314514 | 0.338504044 | down |
| RP11-671J11.7 | -2.939379608 | 0.000487221 | 0.003406576 | down |
| CTC-379B2.4 | -2.938788386 | 0.081086635 | 0.17391254 | down |
| RP11-153F5.2 | -2.937399225 | 0.047152836 | 0.117166472 | down |
| RP11-4M23.7 | -2.936855332 | 0.029196234 | 0.082274088 | down |
| IQCJ | -2.93670008 | 0.313928089 | | down |
| RP5-849H19.3 | -2.93617502 | 0.060962384 | 0.141454576 | down |
| RP1-155D22.2 | -2.936157644 | 0.06010195 | 0.139868549 | down |
| NA | -2.936074123 | 0.027226986 | 0.078151101 | down |
| NA | -2.935865482 | 0.208089514 | 0.33830454 | down |
| RP11-1105G2.4 | -2.935712731 | 0.002318771 | 0.011841775 | down |
| LINC01205 | -2.93535995 | 0.034645561 | 0.093395356 | down |
| RPL23AP23 | -2.934517849 | 0.07337667 | | down |
| AC007679.1 | -2.93298111 | 0.038645375 | 0.101234777 | down |
| CYP4F12 | -2.932639399 | 8.19E-16 | 1.98E-13 | down |
| RP11-636021.2 | -2.932178016 | 0.027354363 | 0.07841932 | down |
| CCKBR | -2.931923439 | 0.071296068 | 0.158409931 | down |
| RP11-482M8.1 | -2.930928415 | 0.014703249 | 0.049170127 | down |
| RN7SL183P | -2.930883802 | 0.314891427 | | down |
| GULOP | -2.930700309 | 2.10E-05 | 0.000260796 | down |
| ENPP7P8 | -2.930561245 | 0.000143931 | 0.001276753 | down |
| NA | -2.930405116 | 0.100138484 | 0.202128464 | down |
| RP11-158L12.6 | -2.929557895 | 0.000389909 | 0.002857179 | down |
| RP11-561N12.6 | -2.929486982 | 0.042511537 | 0.108732929 | down |
| RP11-46802.1 | -2.929309061 | 0.063552759 | 0.14575413 | down |
| RNU6-1114P | -2.929200404 | 0.086782635 | 0.182746665 | down |
| RP1-27K12.2 | -2.928150092 | 0.208235196 | 0.338414597 | down |
| RP11-356K23.2 | -2.92786294 | 0.132478374 | 0.24652955 | down |
| MIR205HG | -2.927238896 | 0.208628617 | 0.338794561 | down |
| FMR1-AS1 | -2.927134458 | 0.043743113 | 0.111032494 | down |
| RP11-569G13.3 | -2.926833839 | 0.003571951 | 0.016648417 | down |
| NA | -2.925475835 | 1.69E-05 | 0.00021835 | down |
| PTPRQ | -2.924660598 | 0.01563388 | 0.051542223 | down |
| OR1K1 | -2.924556384 | 0.211172229 | | down |
| LINC00390 | -2.924214889 | 2.83E-09 | 1.30E-07 | down |
| IFNK | -2.924106681 | 0.037134884 | 0.098246477 | down |
| RP11-628J14.1 | -2.924019585 | 0.010515277 | 0.03811788 | down |
| RP11-1033H12.1 | -2.922995452 | 0.053831936 | 0.129004281 | down |
| RP11-54606.4 | -2.92236 | 0.095365565 | | down |
| RP5-855F16.1 | -2.92223962 | 1.12E-05 | 0.000155576 | down |
| RAB28P3 | -2.922049632 | 0.137036653 | 0.252367826 | down |
| LPA | -2.921991693 | 0.0021507 | 0.011131028 | down |
| CTD-2535P7.2 | -2.921841454 | 0.316392946 | | down |
| RP11-148E17.1 | -2.92141778 | 0.054349931 | 0.129924358 | down |
| RP11-191F9.4 | -2.92132711 | 0.316478495 | | down |

| | | | | |
|---|---|---|---|---|
| AC092155.4 | -2.921009838 | 0.00406329 | 0.018471402 | down |
| RP11-210K20.6 | -2.920927681 | 0.027755628 | 0.079267119 | down |
| MIR554 | -2.920760116 | 0.022086832 | 0.066791826 | down |
| RP11-44N21.3 | -2.920186623 | 0.000345185 | 0.002612937 | down |
| NA | -2.920183993 | 0.060651228 | 0.140884914 | down |
| RP11-146N23.4 | -2.919965069 | 0.004210733 | 0.018991523 | down |
| CH17-76K2.6 | -2.919882587 | 0.034042382 | 0.092182905 | down |
| IL6RP1 | -2.919683077 | 0.316752046 | | down |
| NA | -2.919085742 | 0.057927472 | | down |
| UPK1B | -2.918938298 | 0.031253369 | | down |
| RP11-1035H13.2 | -2.91887643 | 5.82E-07 | 1.29E-05 | down |
| OR3A3 | -2.918469254 | 0.06231324 | 0.143734279 | down |
| AC006116.13 | -2.917924817 | 0.085523668 | 0.180923325 | down |
| LINC00507 | -2.917400026 | 0.000283575 | 0.002228103 | down |
| IZUMO2 | -2.917106799 | 0.011290287 | 0.040249456 | down |
| RP11-673F18.1 | -2.915044588 | 0.208413071 | 0.338590943 | down |
| RPL12P49 | -2.914871089 | 0.317553605 | | down |
| RP11-307P22.1 | -2.914554338 | 0.002046975 | 0.010695532 | down |
| NA | -2.914281995 | 0.001438906 | 0.00806991 | down |
| BMP3 | -2.91423909 | 9.12E-15 | 1.69E-12 | down |
| RP11-159M11.2 | -2.914064459 | 0.004273951 | 0.01919094 | down |
| RP11-340I6.8 | -2.913790098 | 7.11E-05 | 0.000711226 | down |
| GUCY2EP | -2.913294922 | 0.014932515 | 0.049765111 | down |
| DNAI1 | -2.912953722 | 5.68E-05 | 0.000593655 | down |
| GOLGA2P9 | -2.912706249 | 0.261618542 | 0.396772714 | down |
| RP11-407P18.1 | -2.912247388 | 0.0026327 | 0.013093336 | down |
| CRISP1 | -2.911476338 | 0.08737565 | 0.183625772 | down |
| RP11-713H12.1 | -2.911289209 | 0.043413811 | 0.110479801 | down |
| CACNA1D | -2.911275136 | 1.26E-14 | 2.29E-12 | down |
| CCNB2P1 | -2.910680244 | 0.080999071 | 0.17381159 | down |
| MAS1 | -2.910551177 | 0.00109257 | 0.006514222 | down |
| C1orf195 | -2.910310181 | 0.001628294 | 0.008909209 | down |
| PWWP2AP1 | -2.910282686 | 0.211267044 | | down |
| CTA-243E7.3 | -2.910267691 | 0.102716353 | | down |
| RNU6ATAC27P | -2.909954809 | 0.004210988 | 0.018991523 | down |
| C1orf64 | -2.909472517 | 0.057444553 | 0.135283047 | down |
| LINC00309 | -2.909465259 | 0.000413301 | 0.00298491 | down |
| RP11-354P11.8 | -2.909424677 | 0.033157828 | 0.090251294 | down |
| RP11-109G23.3 | -2.908350899 | 0.000297653 | 0.002317362 | down |
| SRD5A2 | -2.908234414 | 0.011889088 | 0.041895154 | down |
| COX7BP2 | -2.908190114 | 0.070241029 | 0.15674972 | down |
| CTC-340A15.2 | -2.908098999 | 0.001542755 | 0.008526589 | down |
| RP11-509E10.3 | -2.907735649 | 0.000143934 | 0.001276753 | down |
| NA | -2.906824869 | 0.052420624 | 0.12645939 | down |
| NA | -2.906401478 | 0.003130281 | 0.01497789 | down |
| SLC39A5 | -2.906128831 | 2.26E-13 | 2.97E-11 | down |
| AC012370.3 | -2.906103377 | 0.004754916 | 0.020882428 | down |
| RN7SL297P | -2.904938964 | 0.109309046 | 0.215580148 | down |
| RP11-527L4.9 | -2.904354645 | 0.063456469 | 0.145644474 | down |
| RP3-425C14.5 | -2.903853654 | 0.001262921 | 0.007295368 | down |
| NA | -2.903850088 | 0.319345621 | | down |

| | | | | |
|---|---|---|---|---|
| NA | -2.903658379 | 0.006016101 | 0.025006603 | down |
| ADCY10 | -2.903631004 | 1.61E-06 | 3.08E-05 | down |
| MRPL57P3 | -2.902929642 | 0.042786313 | 0.109271675 | down |
| DUSP8P4 | -2.902644168 | 0.169695474 | | down |
| PPP1R27 | -2.902561559 | 1.21E-06 | 2.40E-05 | down |
| CRX | -2.90246514 | 0.002151661 | 0.011134088 | down |
| AC114755.5 | -2.902130045 | 0.027864945 | 0.079533955 | down |
| MTCYBP35 | -2.902040777 | 0.000971095 | 0.005910772 | down |
| CFTRP2 | -2.901922617 | 0.035193736 | 0.094424059 | down |
| RP3-340H11.2 | -2.900427586 | 0.217828686 | 0.349241958 | down |
| UBE2CP4 | -2.900404843 | 0.050605978 | 0.1233395 | down |
| UGT2A1 | -2.899686984 | 0.064746557 | 0.147579512 | down |
| AC007131.1 | -2.898893851 | 0.170772201 | | down |
| OR4X2 | -2.898630369 | 0.210084512 | | down |
| ATP5G1P3 | -2.898623693 | 0.00247585 | 0.012472239 | down |
| RP11-426L16.9 | -2.898555961 | 0.320232197 | | down |
| AC009120.10 | -2.898245078 | 0.021779235 | 0.066114512 | down |
| RP3-412A9.17 | -2.897483835 | 0.000111374 | 0.001035628 | down |
| LIN28A | -2.897464173 | 0.068501342 | 0.153919252 | down |
| CTD-2561B21.5 | -2.897425194 | 3.45E-06 | 5.85E-05 | down |
| SSXP3 | -2.896915854 | 0.123091642 | 0.234150012 | down |
| CTAGE11P | -2.896884887 | 0.169692745 | | down |
| AP000318.2 | -2.896532001 | 0.049631517 | 0.121645398 | down |
| AC007182.6 | -2.896155878 | 1.37E-10 | 9.04E-09 | down |
| NA | -2.895340254 | 0.17486618 | 0.299269696 | down |
| LILRP1 | -2.895280411 | 0.13982077 | 0.256051153 | down |
| FAM71F1 | -2.89431133 | 0.014925428 | 0.049763574 | down |
| CICP3 | -2.89414995 | 0.001480618 | 0.008246883 | down |
| NPM1P5 | -2.893556475 | 0.059725914 | 0.139219904 | down |
| CLUHP11 | -2.893288999 | 0.321164992 | | down |
| AC005863.2 | -2.893240086 | 0.321173207 | | down |
| SLCO6A1 | -2.892276894 | 0.173732879 | 0.298269098 | down |
| RP11-108H9.1 | -2.891436355 | 0.267271183 | | down |
| RP11-789F5.1 | -2.891302241 | 0.094701186 | 0.194490562 | down |
| RP11-855O10.2 | -2.890766964 | 0.00013724 | 0.001229347 | down |
| AC004158.3 | -2.890345652 | 0.030542592 | 0.084912286 | down |
| AC016700.4 | -2.889398275 | 0.321722667 | | down |
| GRK1 | -2.888478634 | 0.000165203 | 0.001428304 | down |
| TRPM6 | -2.888004892 | 8.47E-16 | 2.03E-13 | down |
| ANP32BP3 | -2.887415101 | 0.097294187 | 0.198149848 | down |
| CTC-467M3.3 | -2.887302198 | 0.006177237 | 0.025510503 | down |
| CDKN2B-AS1 | -2.886660665 | 4.42E-13 | 5.38E-11 | down |
| RP11-39H3.1 | -2.885960632 | 0.062600666 | 0.144197991 | down |
| BOLA3P2 | -2.885518546 | 0.012461468 | 0.043370954 | down |
| HSD3B2 | -2.885470569 | 4.61E-06 | 7.42E-05 | down |
| SNORA26 | -2.885154339 | 0.119661235 | 0.229704309 | down |
| AC007292.4 | -2.884970174 | 0.013078334 | 0.044946862 | down |
| CRNN | -2.884779024 | 0.175363204 | 0.299864092 | down |
| AC009501.4 | -2.884486517 | 1.50E-07 | 4.03E-06 | down |
| RP11-562I5.2 | -2.884374211 | 4.11E-05 | 0.000454091 | down |
| NA | -2.884336601 | 0.001102268 | 0.006560331 | down |

| | | | | |
|---|---|---|---|---|
| PAUPAR | -2.884088289 | 0.000756472 | 0.004857331 | down |
| MYO1H | -2.883897022 | 3.46E-05 | 0.000393473 | down |
| GLYATL3 | -2.883573296 | 0.064566795 | 0.147315066 | down |
| AC005262.4 | -2.882935517 | 0.001094439 | 0.006520194 | down |
| RP11-333E13.4 | -2.882549235 | 0.000947388 | 0.005800555 | down |
| LINC00424 | -2.882258738 | 0.109783158 | 0.216156052 | down |
| PKN2-AS1 | -2.882185882 | 1.52E-05 | 0.000201404 | down |
| RP11-278H7.1 | -2.8818686 | 0.003586108 | 0.016693681 | down |
| RP11-316E14.2 | -2.881757478 | 0.093803088 | 0.193214039 | down |
| Metazoa_SRP | -2.881620304 | 0.001313026 | 0.007515444 | down |
| DPP6 | -2.881562759 | 5.32E-08 | 1.66E-06 | down |
| LINC01399 | -2.881478333 | 2.72E-05 | 0.000322463 | down |
| RP11-84A19.2 | -2.880421607 | 0.056093489 | 0.133002352 | down |
| CTD-2313J17.3 | -2.880270026 | 0.015121761 | 0.050261996 | down |
| TLR12P | -2.880140264 | 0.003955317 | 0.018079043 | down |
| AC064874.1 | -2.879869894 | 0.17333728 | | down |
| RP11-449H3.1 | -2.879736218 | 0.029888146 | 0.083546657 | down |
| AC073218.3 | -2.879731336 | 0.268356464 | | down |
| RP11-697K23.3 | -2.879461273 | 0.001074872 | 0.006423999 | down |
| COX6CP3 | -2.878664293 | 0.269741929 | | down |
| TMEM246-AS1 | -2.878560373 | 0.031649186 | 0.087243614 | down |
| POU3F4 | -2.877807249 | 0.090896681 | 0.18881974 | down |
| SEPT14P1 | -2.877576964 | 0.172612811 | 0.296901546 | down |
| RNU6-176P | -2.877490207 | 0.015901804 | 0.052207826 | down |
| SIGLEC29P | -2.876739286 | 0.177700317 | 0.302671826 | down |
| MYH2 | -2.876567569 | 0.00073407 | 0.004744922 | down |
| RP11-134D3.1 | -2.87654988 | 0.112736705 | 0.219981916 | down |
| RP11-54A9.1 | -2.876501135 | 6.99E-05 | 0.000701637 | down |
| AC073934.6 | -2.876457403 | 0.010588119 | 0.038340297 | down |
| NR2E3 | -2.876183518 | 9.98E-08 | 2.83E-06 | down |
| CTD-2290C23.1 | -2.876034379 | 0.00364462 | 0.016911036 | down |
| NA | -2.875668285 | 0.075301588 | 0.16482303 | down |
| CYP4F36P | -2.875609486 | 0.137524397 | | down |
| RP11-155D18.13 | -2.87481924 | 7.83E-08 | 2.32E-06 | down |
| RPL12P44 | -2.874183193 | 0.00157926 | 0.008694758 | down |
| INPP5J | -2.873805924 | 4.96E-24 | 7.45E-21 | down |
| NEUROG1 | -2.872887687 | 0.324603364 | | down |
| NA | -2.872720684 | 0.270590978 | | down |
| NA | -2.871896124 | 0.039269152 | 0.10252471 | down |
| NA | -2.870425393 | 0.076262232 | 0.166319868 | down |
| NA | -2.870402957 | 0.086810706 | 0.182770908 | down |
| RNA5SP206 | -2.870182198 | 0.031684997 | 0.087306546 | down |
| RN7SL794P | -2.870135211 | 0.004964576 | 0.021579243 | down |
| NA | -2.869634689 | 0.217331056 | | down |
| NA | -2.869518332 | 0.179514981 | 0.304691717 | down |
| RP11-643A5.2 | -2.869247877 | 0.010068803 | 0.036877619 | down |
| RP11-110L15.1 | -2.868196441 | 0.325397387 | | down |
| HMGN1P33 | -2.867532891 | 0.146866909 | 0.265140572 | down |
| CTD-2013N17.4 | -2.867073433 | 0.018572261 | 0.058747631 | down |
| RP11-320N7.2 | -2.867016159 | 0.002900806 | 0.014121268 | down |
| OTX2 | -2.866567299 | 0.185245703 | 0.311382213 | down |

| | | | | |
|---|---|---|---|---|
| LINC01351 | -2.86625998 | 0.001145177 | 0.006756835 | down |
| NA | -2.86574232 | 0.000650539 | 0.004299338 | down |
| UBE2R2-AS1 | -2.865379305 | 3.45E-07 | 8.14E-06 | down |
| NA | -2.865354493 | 0.088795849 | 0.185736747 | down |
| CTB-120L21.1 | -2.865235569 | 0.325801376 | | down |
| PLGLA | -2.864671458 | 0.173977738 | 0.298440703 | down |
| RP11-640I15.1 | -2.864655139 | 1.16E-06 | 2.30E-05 | down |
| RP11-292B1.2 | -2.864563515 | 0.267722874 | 0.403484627 | down |
| NA | -2.864283102 | 0.026586424 | 0.076766961 | down |
| FBXO47 | -2.86391416 | 0.069416996 | 0.155372438 | down |
| RP11-626G3.1 | -2.863685701 | 2.41E-05 | 0.000291983 | down |
| LINC00449 | -2.863642354 | 0.002068796 | 0.010783293 | down |
| RP11-196E1.3 | -2.863556771 | 0.000597921 | 0.004022375 | down |
| TDGF1P5 | -2.863441154 | 0.064978709 | 0.147951523 | down |
| SPDYE16 | -2.86284129 | 0.00023124 | 0.00188429 | down |
| RP1-56K13.2 | -2.862408449 | 0.001183184 | 0.006937467 | down |
| RP11-261B23.2 | -2.862122707 | 0.043260946 | 0.110184047 | down |
| SLC25A51P1 | -2.861760719 | 0.141450449 | 0.258193996 | down |
| ACA64 | -2.861418824 | 0.002740962 | 0.01351986 | down |
| FAM71B | -2.861154663 | 0.217881665 | | down |
| RP4-594I10.3 | -2.861109347 | 0.00709222 | 0.028274767 | down |
| MOGAT2 | -2.861095413 | 4.40E-15 | 8.65E-13 | down |
| CICP16 | -2.860673178 | 0.00015956 | 0.001387502 | down |
| RP11-146N18.1 | -2.860067881 | 0.012991335 | 0.044719485 | down |
| HMGB1P28 | -2.859464122 | 0.027788559 | 0.079346082 | down |
| SLC25A47P1 | -2.858387165 | 0.000376605 | 0.002783515 | down |
| AC004386.4 | -2.858173855 | 0.0042217 | 0.019027414 | down |
| SLC22A9 | -2.858049297 | 0.000943497 | 0.005781445 | down |
| RN7SL180P | -2.857348251 | 1.67E-05 | 0.000215648 | down |
| MTCO1P22 | -2.857159092 | 0.000203931 | 0.001698615 | down |
| DPP10 | -2.856717397 | 8.87E-08 | 2.57E-06 | down |
| RPS26P31 | -2.856515196 | 0.000831759 | 0.005240579 | down |
| UNC13C | -2.855867125 | 4.28E-05 | 0.00046907 | down |
| Y_RNA | -2.855576557 | 0.010866123 | 0.039091625 | down |
| RP11-568A7.3 | -2.854652644 | 0.010148368 | | down |
| PRAMEF15 | -2.854508634 | 0.32762283 | | down |
| MTND4P20 | -2.854472188 | 0.000337376 | 0.002564162 | down |
| AC098973.2 | -2.854332517 | 0.001601225 | 0.008788309 | down |
| MYO18B | -2.853921511 | 4.78E-06 | 7.64E-05 | down |
| RNU6-611P | -2.853425061 | 0.000128429 | 0.001163606 | down |
| KRTAP5-4 | -2.852649904 | 0.015251164 | 0.050557869 | down |
| POU5F2 | -2.852049403 | 1.36E-06 | 2.65E-05 | down |
| RP11-665C16.1 | -2.851963027 | 0.00319571 | 0.015211013 | down |
| NA | -2.85182703 | 0.115778626 | 0.224315021 | down |
| CTD-2619J13.13 | -2.850943876 | 0.016247317 | 0.053018017 | down |
| CLRN2 | -2.850725258 | 0.145307152 | 0.263004281 | down |
| RP11-779O18.4 | -2.85068293 | 0.107630113 | | down |
| RP13-43E11.1 | -2.850653778 | 0.016044213 | 0.052537584 | down |
| bP-21201H5.1 | -2.850362447 | 0.001401818 | 0.007906177 | down |
| RP11-142L1.3 | -2.850053624 | 0.153600922 | 0.273301619 | down |
| MARCKSL1P2 | -2.849945349 | 0.047797202 | 0.118346723 | down |

| | | | | |
|---|---|---|---|---|
| KRT18P16 | -2.849476768 | 0.001108253 | 0.006586813 | down |
| CTC-204F22.1 | -2.849229157 | 2.97E-08 | 9.95E-07 | down |
| TCP10L2 | -2.847913077 | 5.09E-05 | 0.00054282 | down |
| Y_RNA | -2.847577206 | 0.187044477 | 0.313521773 | down |
| NA | -2.847174965 | 0.225421619 | | down |
| RP11-281P23.2 | -2.846804281 | 2.67E-06 | 4.73E-05 | down |
| NA | -2.845811777 | 0.008079208 | 0.031163401 | down |
| RPS20P2 | -2.845404898 | 0.001754012 | 0.009460957 | down |
| VDAC1P9 | -2.845060777 | 0.019072004 | 0.059887048 | down |
| OR10D3 | -2.844863548 | 0.181077736 | 0.306547558 | down |
| CLUHP8 | -2.844254582 | 0.271795193 | 0.40798475 | down |
| NA | -2.84387324 | 0.000413557 | 0.00298604 | down |
| NA | -2.843783473 | 0.180915051 | | down |
| NA | -2.84357618 | 0.005344758 | 0.022831063 | down |
| NPM1P32 | -2.843372456 | 0.001056644 | 0.00633649 | down |
| LINC01133 | -2.843206671 | 4.78E-12 | 4.53E-10 | down |
| RP11-108K3.3 | -2.842990461 | 0.058410201 | 0.137110406 | down |
| NA | -2.84295194 | 0.275895683 | | down |
| RP11-321E2.12 | -2.842423333 | 0.113850193 | 0.221594787 | down |
| B3GNT8 | -2.842111606 | 1.00E-16 | 2.96E-14 | down |
| RP11-396N11.1 | -2.841739435 | 0.046406996 | 0.115730183 | down |
| SLC13A5 | -2.841446377 | 3.49E-06 | 5.90E-05 | down |
| SYCP3 | -2.840887918 | 9.69E-07 | 1.98E-05 | down |
| ETNPPL | -2.840863866 | 0.081019621 | 0.173815232 | down |
| RP11-133K1.6 | -2.840703735 | 0.01688921 | 0.054555361 | down |
| CTD-2165H16.3 | -2.840690297 | 0.009523022 | 0.035300393 | down |
| AQP10 | -2.84065705 | 0.000104939 | 0.000985559 | down |
| SPATA3 | -2.840499671 | 0.038111384 | 0.100229203 | down |
| RP4-659I19.1 | -2.84006612 | 0.002262648 | 0.011606451 | down |
| RP11-215E13.1 | -2.840017768 | 0.000624425 | 0.004165235 | down |
| SNORA7 | -2.838843898 | 0.023640562 | 0.070252922 | down |
| EIF4EP3 | -2.838836145 | 0.139978759 | | down |
| RPL12P45 | -2.838655511 | 0.095515222 | 0.195587468 | down |
| CTA-747E2.10 | -2.8378782 | 0.003526117 | 0.016471205 | down |
| PAPOLB | -2.836500346 | 0.225781654 | | down |
| ABCA4 | -2.836368229 | 2.69E-10 | 1.62E-08 | down |
| RP11-154F14.2 | -2.836053746 | 0.278993557 | | down |
| ARHGAP36 | -2.835958974 | 0.002996358 | 0.014480069 | down |
| NA | -2.835950346 | 0.179270088 | | down |
| SNORA31 | -2.835648049 | 0.275643292 | | down |
| RP11-238K6.2 | -2.835123562 | 0.224583178 | 0.356739823 | down |
| POU5F1P4 | -2.834819364 | 0.022147548 | 0.066955215 | down |
| DUSP12P1 | -2.834485807 | 0.183367268 | 0.309133914 | down |
| CHAD | -2.834068059 | 3.93E-12 | 3.79E-10 | down |
| LINC01568 | -2.833522481 | 0.030271031 | 0.08435793 | down |
| NA | -2.832858226 | 0.004718949 | 0.020751772 | down |
| KRT18P36 | -2.832624055 | 0.225363096 | | down |
| RP11-243A14.1 | -2.832513412 | 6.34E-08 | 1.94E-06 | down |
| RP1-91J24.1 | -2.832470872 | 0.088058143 | 0.184608111 | down |
| RP11-137L10.5 | -2.832454069 | 0.044062661 | 0.111548749 | down |
| PART1 | -2.832135567 | 1.20E-07 | 3.32E-06 | down |

| | | | | |
|---|---|---|---|---|
| MIR4664 | -2.830639264 | 0.000709869 | 0.004613305 | down |
| NA | -2.830481242 | 0.018507811 | 0.058630184 | down |
| RN7SL456P | -2.829616668 | 0.054197742 | 0.129663717 | down |
| RABGAP1L-IT1 | -2.829277907 | 0.001042501 | 0.006270448 | down |
| NIFKP6 | -2.829134823 | 0.027379475 | 0.078483823 | down |
| SLC17A4 | -2.828390643 | 1.52E-11 | 1.28E-09 | down |
| RP11-220C2.1 | -2.828241265 | 0.003672459 | 0.017016555 | down |
| GDA | -2.827502446 | 2.41E-13 | 3.14E-11 | down |
| C10orf67 | -2.827365983 | 4.78E-08 | 1.51E-06 | down |
| AC009264.1 | -2.827246561 | 0.026464317 | 0.076480755 | down |
| Y_RNA | -2.826633596 | 0.092395755 | 0.191022437 | down |
| VN1R90P | -2.826145607 | 0.149992101 | 0.268972345 | down |
| LINC01608 | -2.825403673 | 0.095767784 | 0.195957727 | down |
| ACTG1P19 | -2.824969346 | 0.181551201 | 0.307089533 | down |
| SLC38A8 | -2.824027834 | 0.018248506 | 0.057989633 | down |
| RP11-463C8.5 | -2.824008797 | 0.181259638 | 0.306724656 | down |
| RN7SL479P | -2.82337484 | 0.225619392 | | down |
| RP11-483E23.2 | -2.823202205 | 0.079570067 | 0.171455433 | down |
| SLC25A6P3 | -2.822994193 | 0.333061251 | | down |
| RP11-568J23.7 | -2.822445763 | 0.333207145 | | down |
| RN7SL832P | -2.822325306 | 1.73E-09 | 8.29E-08 | down |
| MIR3687-1 | -2.820928069 | 0.001088826 | 0.00649448 | down |
| RP11-498P14.4 | -2.820430144 | 0.228504851 | 0.360821203 | down |
| AC005307.3 | -2.820017788 | 0.089210986 | 0.186426574 | down |
| RP11-626H12.2 | -2.819900206 | 0.00017046 | 0.001465745 | down |
| RP11-59H7.3 | -2.819797502 | 5.47E-05 | 0.000575644 | down |
| CTD-2568A17.1 | -2.819338756 | 0.000117294 | 0.001083299 | down |
| RP1-131F15.3 | -2.819058842 | 0.130314724 | 0.243723579 | down |
| RP11-274B18.4 | -2.819027206 | 7.70E-05 | 0.000759751 | down |
| NHEG1 | -2.817871256 | 0.053379086 | 0.12818257 | down |
| NA | -2.817437868 | 0.001578426 | 0.00869176 | down |
| RP11-269G24.4 | -2.817346335 | 0.002432499 | 0.012300387 | down |
| ALPP | -2.816984208 | 0.068958538 | 0.154636431 | down |
| RPS26P35 | -2.816805567 | 0.11863094 | 0.228236934 | down |
| OPRPN | -2.81611401 | 0.334297442 | | down |
| AC024162.2 | -2.815770952 | 0.225660478 | | down |
| SLC30A8 | -2.815655774 | 3.33E-06 | 5.67E-05 | down |
| VWC2 | -2.815525537 | 1.03E-08 | 4.08E-07 | down |
| RP11-542M13.2 | -2.815054927 | 4.53E-05 | 0.000493047 | down |
| SCIN | -2.814751245 | 2.00E-17 | 6.69E-15 | down |
| RP11-213G2.2 | -2.814682593 | 0.000437741 | 0.003129075 | down |
| NA | -2.814565823 | 0.111648641 | | down |
| OSTM1-AS1 | -2.814482959 | 0.334430528 | | down |
| RP11-587H10.2 | -2.814029221 | 0.279924615 | 0.416256382 | down |
| RP13-977E11.1 | -2.813336434 | 0.182009279 | | down |
| AC011753.6 | -2.813116985 | 0.281366911 | | down |
| RP11-255P5.2 | -2.813006182 | 0.148586078 | | down |
| CSRP3 | -2.812576443 | 0.038255399 | 0.100572734 | down |
| RP5-1022P6.7 | -2.812374347 | 0.02769172 | 0.079139519 | down |
| LINC00393 | -2.812177081 | 0.016578317 | 0.05382481 | down |
| Y_RNA | -2.811294513 | 0.003992803 | 0.018211595 | down |

| | | | | |
|---|---|---|---|---|
| ESRRG | -2.811227441 | 4.18E-09 | 1.82E-07 | down |
| RP5-1185I7.1 | -2.810905882 | 1.06E-07 | 2.98E-06 | down |
| RP11-774D14.1 | -2.810558184 | 0.010236852 | 0.0373367 | down |
| OR11G2 | -2.810236765 | 0.019719612 | 0.061381686 | down |
| CDHR2 | -2.80894465 | 1.44E-11 | 1.22E-09 | down |
| RP11-795A2.2 | -2.808694285 | 0.016487424 | 0.05359171 | down |
| RP11-688I9.5 | -2.808638078 | 0.024597494 | 0.072409083 | down |
| SLC7A13 | -2.808105484 | 0.117010477 | | down |
| RNU6-833P | -2.808072154 | 0.001432894 | 0.008051216 | down |
| RP11-83M16.5 | -2.807894597 | 0.087623092 | | down |
| RP11-1070N10.4 | -2.807699387 | 0.153474461 | 0.273162719 | down |
| RP11-517M22.1 | -2.807258901 | 0.232273636 | | down |
| NA | -2.806631952 | 1.16E-07 | 3.21E-06 | down |
| RP1-166H4.2 | -2.806097277 | 0.284941057 | | down |
| ELK2BP | -2.804827201 | 0.036359273 | 0.096773388 | down |
| RP11-2E11.6 | -2.804624034 | 0.058809971 | 0.137715258 | down |
| RP11-1134I14.2 | -2.804605277 | 0.163225441 | 0.285378559 | down |
| NA | -2.804371723 | 0.001369726 | 0.007766002 | down |
| CTC-457E21.4 | -2.804298391 | 0.121145603 | 0.231665616 | down |
| RN7SL88P | -2.804227962 | 0.129778655 | 0.242893026 | down |
| RNU6-758P | -2.803966494 | 0.001038573 | 0.006254339 | down |
| C2orf82 | -2.803787373 | 1.37E-10 | 9.00E-09 | down |
| AC135999.2 | -2.803287423 | 0.101620112 | 0.204383092 | down |
| RP1-240K6.3 | -2.802890249 | 4.11E-07 | 9.45E-06 | down |
| AC144838.3 | -2.802375758 | 0.01267135 | 0.043950363 | down |
| RP11-354P11.4 | -2.802355008 | 0.003965892 | 0.018119107 | down |
| Y_RNA | -2.802079985 | 0.119603274 | 0.229649856 | down |
| RP1-90K10.4 | -2.80161585 | 0.024909797 | 0.073092094 | down |
| BRINP2 | -2.801512889 | 3.35E-05 | 0.0003828 | down |
| RP5-947P14.1 | -2.801351803 | 0.051723006 | 0.125306109 | down |
| OR1M1 | -2.800860499 | 0.33450512 | 0.472757165 | down |
| RP5-958B11.2 | -2.80080346 | 0.122409579 | 0.233271697 | down |
| CFAP206 | -2.800629911 | 0.152220065 | 0.271670408 | down |
| RP11-429D19.1 | -2.800620043 | 1.03E-07 | 2.90E-06 | down |
| RP11-648L3.2 | -2.800459499 | 0.156120915 | 0.276448209 | down |
| GOT1L1 | -2.800402704 | 0.162495372 | 0.284473015 | down |
| CTC-43909.1 | -2.800235674 | 0.005945939 | 0.024786982 | down |
| MIR663AHG | -2.799821398 | 4.17E-06 | 6.83E-05 | down |
| AC007036.4 | -2.799653342 | 0.000319223 | 0.002450605 | down |
| RN7SL386P | -2.799537898 | 0.021491649 | 0.065458748 | down |
| NA | -2.799279943 | 0.232855515 | | down |
| RP11-3J1.1 | -2.798698823 | 0.148922656 | 0.267645647 | down |
| MTCO2P20 | -2.798543643 | 0.088336861 | 0.18505295 | down |
| RNU6-777P | -2.798515344 | 0.284151753 | | down |
| NA | -2.797955587 | 0.00385423 | 0.017699081 | down |
| LINC00868 | -2.797738548 | 0.009188909 | 0.034373791 | down |
| FLG2 | -2.797473009 | 0.185484554 | | down |
| RP11-503C24.6 | -2.797444476 | 0.001233817 | 0.007165458 | down |
| PRSS55 | -2.797395218 | 0.024323289 | 0.071810794 | down |
| RP11-720N19.1 | -2.797218857 | 0.001006448 | 0.006096314 | down |
| RP11-312A15.3 | -2.796483481 | 0.090842321 | 0.188732916 | down |

| | | | | |
|---|---|---|---|---|
| CTC-308K20.1 | -2.795840098 | 5.68E-05 | 0.000593354 | down |
| NA | -2.795226377 | 0.000206363 | 0.001715473 | down |
| SLC15A5 | -2.795065766 | 0.15321179 | 0.272856847 | down |
| CPB2 | -2.794679319 | 0.063024051 | 0.144895516 | down |
| SPDYE20P | -2.79463668 | 0.024590685 | 0.072403228 | down |
| AGMO | -2.794444733 | 2.29E-09 | 1.07E-07 | down |
| RP11-153I24.5 | -2.794217938 | 0.011191012 | 0.039969421 | down |
| OR8D1 | -2.793210792 | 0.05676099 | 0.134214322 | down |
| KCNC2 | -2.792746371 | 0.061657078 | 0.142636887 | down |
| AGBL1 | -2.792140032 | 0.003405262 | 0.016008086 | down |
| NA | -2.791440957 | 0.072265608 | 0.160043338 | down |
| RP11-371I20.2 | -2.791106311 | 0.007055642 | 0.028147616 | down |
| RPL10P13 | -2.790251085 | 0.018996567 | 0.059703082 | down |
| RNU6-899P | -2.790148881 | 0.15877313 | 0.279892107 | down |
| CASR | -2.790104488 | 1.15E-06 | 2.29E-05 | down |
| IMMP1LP3 | -2.789754524 | 0.044026421 | 0.111519246 | down |
| NA | -2.78817545 | 0.001150122 | 0.006780663 | down |
| NA | -2.787885303 | 3.70E-06 | 6.17E-05 | down |
| EEF1DP6 | -2.787773332 | 0.339205709 | | down |
| KRTAP16-1 | -2.787721095 | 0.041108949 | 0.106024318 | down |
| NXF5 | -2.787533458 | 0.042195934 | 0.108168485 | down |
| RP11-271F18.4 | -2.787297329 | 0.020706314 | 0.063720218 | down |
| RP11-118K6.2 | -2.787277567 | 0.153769799 | 0.273429204 | down |
| MKRN9P | -2.787260778 | 0.008863066 | 0.033452508 | down |
| LINC00462 | -2.786994447 | 0.00161994 | 0.008878051 | down |
| RP11-167P20.1 | -2.786798243 | 6.80E-05 | 0.000685963 | down |
| RP11-616M22.1 | -2.786678445 | 0.003447456 | 0.016177154 | down |
| RP5-983L19.2 | -2.786452972 | 0.001495306 | 0.008316361 | down |
| AC063980.3 | -2.786406795 | 0.047441889 | | down |
| NLRP11 | -2.786219312 | 4.39E-06 | 7.12E-05 | down |
| CTD-2194F4.2 | -2.786206165 | 0.052965194 | 0.127425232 | down |
| RP3-462C17.1 | -2.785636637 | 0.062461181 | 0.143964996 | down |
| TFAP2B | -2.785509626 | 0.034398974 | 0.092947457 | down |
| RNU6-898P | -2.784737944 | 0.062168143 | 0.143509773 | down |
| ABCC2 | -2.78464886 | 3.83E-10 | 2.23E-08 | down |
| RP1-146A15.1 | -2.784642772 | 0.286946145 | | down |
| Metazoa_SRP | -2.784624171 | 0.057138116 | 0.134851791 | down |
| GOLGA6B | -2.784517602 | 0.019460884 | 0.06082484 | down |
| MTHFD2P1 | -2.784108857 | 0.001363134 | 0.00773739 | down |
| PTH2 | -2.784049883 | 0.149598629 | | down |
| MGC15885 | -2.783853574 | 0.150828367 | | down |
| PLEKHD1 | -2.783400351 | 1.74E-08 | 6.36E-07 | down |
| DUXAP7 | -2.7833428 | 0.101811278 | 0.204657971 | down |
| NA | -2.783011124 | 0.049923484 | 0.122171635 | down |
| CTC-435M10.10 | -2.782924968 | 0.012012673 | 0.042231308 | down |
| IGSF9 | -2.782852894 | 1.38E-12 | 1.48E-10 | down |
| AC073842.19 | -2.782788932 | 0.004260352 | 0.019158511 | down |
| NPM1P10 | -2.782384572 | 0.157411001 | 0.278142452 | down |
| OPALIN | -2.782230977 | 0.044710773 | 0.112687643 | down |
| RP1-81D8.3 | -2.781940553 | 0.096222704 | 0.196647501 | down |
| RP11-415C15.3 | -2.781913072 | 0.083112562 | 0.177116973 | down |

| | | | | |
|---|---|---|---|---|
| LINC01618 | -2.781821738 | 0.005621291 | 0.023719954 | down |
| MMP26 | -2.781601267 | 0.285257285 | | down |
| CIR1P1 | -2.780717302 | 0.196034366 | 0.324534741 | down |
| PRAMEF30P | -2.780623879 | 0.039150895 | 0.10227367 | down |
| RPSAP71 | -2.780391214 | 0.032323737 | 0.088615234 | down |
| TRIM71 | -2.780276253 | 0.050280362 | 0.122804797 | down |
| RN7SL733P | -2.780048614 | 0.285319922 | | down |
| RP3-404F18.5 | -2.780034264 | 1.15E-06 | 2.29E-05 | down |
| GS1-25M2.1 | -2.77993592 | 0.236327072 | | down |
| LINC00838 | -2.779744964 | 0.069637904 | 0.155750851 | down |
| RP11-223J15.2 | -2.779594079 | 0.021340234 | 0.065110795 | down |
| MGC32805 | -2.779444667 | 5.90E-11 | 4.30E-09 | down |
| TMEM235 | -2.779091188 | 0.007533319 | 0.029578023 | down |
| RP11-63G10.2 | -2.778457009 | 0.01883269 | 0.059327811 | down |
| C1orf87 | -2.77803174 | 0.156654066 | 0.27716358 | down |
| RP11-308B16.2 | -2.777051725 | 0.340876911 | 0.479237525 | down |
| HNRNPRP1 | -2.776584904 | 0.158120689 | | down |
| FAM170B-AS1 | -2.776270353 | 0.079230059 | 0.170955753 | down |
| AC005336.5 | -2.775611392 | 0.098177314 | | down |
| LINC01055 | -2.775045167 | 3.33E-06 | 5.68E-05 | down |
| NUCKS1P1 | -2.774969485 | 0.289051416 | | down |
| AC023115.2 | -2.774322647 | 0.002514406 | 0.012634691 | down |
| AF001550.9 | -2.774265805 | 0.017963193 | 0.057309777 | down |
| RP11-459F1.2 | -2.773798928 | 0.054568598 | 0.130384839 | down |
| RP11-393N4.1 | -2.773498753 | 0.174029046 | 0.298466072 | down |
| RP11-344E13.4 | -2.773446517 | 0.005072777 | 0.021944782 | down |
| RP11-34D15.2 | -2.773293111 | 0.027917302 | 0.079645563 | down |
| RNU4-15P | -2.773083701 | 0.072717342 | 0.160809953 | down |
| RP11-1081M5.1 | -2.772747541 | 0.000582563 | 0.00393669 | down |
| PSMD8P1 | -2.772670027 | 0.007008407 | 0.028028175 | down |
| LINC01524 | -2.772169501 | 0.001982011 | 0.01040859 | down |
| RP3-522D1.1 | -2.771962322 | 0.011999049 | 0.042201008 | down |
| NA | -2.77178813 | 0.287584423 | | down |
| HOXC9 | -2.771747955 | 9.13E-08 | 2.63E-06 | down |
| FAM163B | -2.77172996 | 4.58E-06 | 7.38E-05 | down |
| GJA10 | -2.771606699 | 0.28861843 | | down |
| RP11-264L1.1 | -2.771008965 | 0.022284488 | 0.067217188 | down |
| GDNF-AS1 | -2.76835823 | 1.10E-07 | 3.07E-06 | down |
| GNAS-AS1 | -2.768239667 | 1.49E-06 | 2.88E-05 | down |
| TET1P1 | -2.767756755 | 0.288534774 | 0.425689391 | down |
| AP000889.1 | -2.7675499 | 0.342736275 | | down |
| RP11-192P3.4 | -2.767085031 | 3.39E-05 | 0.000386206 | down |
| RP1-319M7.2 | -2.766951765 | 0.291117728 | | down |
| RP5-906A24.2 | -2.766495626 | 0.342816517 | | down |
| PHYHIPL | -2.766278465 | 1.63E-05 | 0.000212606 | down |
| CDCA4P2 | -2.766139943 | 0.024904586 | 0.073084918 | down |
| RP11-723D22.3 | -2.766100398 | 1.31E-07 | 3.57E-06 | down |
| RP11-890B15.2 | -2.765707683 | 1.90E-05 | 0.000240031 | down |
| RP11-445F12.1 | -2.765592033 | 0.022707061 | 0.068188923 | down |
| OR2AT4 | -2.765559208 | 0.032554716 | 0.089081709 | down |
| RP11-510N19.2 | -2.765376134 | 0.188791925 | | down |

| | | | | |
|---|---|---|---|---|
| NA | -2.765295295 | 0.190396295 | | down |
| ASPG | -2.764864665 | 2.03E-10 | 1.26E-08 | down |
| OR10AA1P | -2.764797159 | 0.343218652 | | down |
| SDAD1P2 | -2.764559447 | 0.193324547 | | down |
| RP11-597M12.2 | -2.763551132 | 0.002463005 | 0.012422114 | down |
| HTR3E-AS1 | -2.763518586 | 0.150077446 | | down |
| BHMT | -2.763344316 | 0.000470402 | 0.003314499 | down |
| RP11-203H2.1 | -2.763046283 | 0.002076838 | 0.010813958 | down |
| CTC-50503.3 | -2.762832637 | 0.035531271 | 0.095041088 | down |
| RP11-338E21.3 | -2.762781676 | 0.104540916 | 0.208388222 | down |
| PAX6 | -2.762475685 | 2.14E-05 | 0.000264444 | down |
| CTA-992D9.8 | -2.762018976 | 0.158066944 | 0.278967596 | down |
| AP000439.1 | -2.761438061 | 0.000412078 | 0.002976789 | down |
| TTC36 | -2.7614135 | 0.000940365 | 0.005770492 | down |
| DAOA | -2.761379119 | 0.192695241 | 0.320703291 | down |
| C8G | -2.760804474 | 4.76E-15 | 9.28E-13 | down |
| GRIFIN | -2.760788411 | 0.13439645 | 0.248878633 | down |
| OR5AS1 | -2.760403853 | 0.343989412 | | down |
| RP11-474C8.5 | -2.759669891 | 0.046803858 | 0.116472716 | down |
| MTND4P31 | -2.758594376 | 0.106969572 | 0.212159613 | down |
| CTA-228A9.3 | -2.758482978 | 5.56E-10 | 3.07E-08 | down |
| RP11-209E8.1 | -2.758099607 | 0.004109908 | 0.018638202 | down |
| NA | -2.75729803 | 0.193733219 | 0.321842456 | down |
| RP11-131K5.2 | -2.756844443 | 0.129154257 | 0.242125951 | down |
| KRT9 | -2.756607796 | 0.000294506 | 0.00229643 | down |
| RP4-533D7.4 | -2.756321859 | 0.000504893 | 0.003503911 | down |
| RP11-637O19.2 | -2.755794874 | 0.000140778 | 0.001255055 | down |
| KIAA0087 | -2.755721232 | 0.002854337 | 0.013946284 | down |
| RP11-405M12.2 | -2.755294011 | 0.344887268 | | down |
| RP11-432I5.8 | -2.755199961 | 8.44E-07 | 1.75E-05 | down |
| DEFA11P | -2.755115391 | 0.084766469 | | down |
| AC008067.2 | -2.754814862 | 0.034938375 | 0.093931859 | down |
| SLC22A2 | -2.754758972 | 0.003867746 | 0.017751689 | down |
| NA | -2.754156546 | 0.345087336 | | down |
| ANKRD20A4 | -2.753205728 | 3.35E-06 | 5.69E-05 | down |
| ZG16 | -2.753148173 | 3.42E-09 | 1.53E-07 | down |
| RP11-192C21.3 | -2.753111773 | 0.167232532 | 0.29038018 | down |
| KRT18P59 | -2.752018786 | 0.06441633 | 0.147105824 | down |
| RP11-107E5.4 | -2.751995086 | 0.008021326 | 0.031027772 | down |
| CTD-2542C24.9 | -2.751709178 | 0.157501312 | | down |
| NA | -2.75152361 | 0.29126172 | 0.428345347 | down |
| RP5-1120P11.3 | -2.750832725 | 1.40E-06 | 2.73E-05 | down |
| RP11-958N24.4 | -2.750390071 | 0.195941814 | 0.324457787 | down |
| AC022819.3 | -2.749968627 | 2.11E-05 | 0.00026205 | down |
| RP11-640N20.6 | -2.749829118 | 0.076148021 | | down |
| SLC3A1 | -2.74953124 | 2.23E-06 | 4.06E-05 | down |
| RP5-966M1.4 | -2.748935126 | 0.153050264 | | down |
| AC079779.7 | -2.748720266 | 0.030109395 | 0.084016593 | down |
| AC114755.3 | -2.748462279 | 0.346035854 | | down |
| AC114755.2 | -2.748359216 | 0.346108176 | | down |
| Y_RNA | -2.747700515 | 0.13157643 | 0.245395882 | down |

| | | | | |
|---|---|---|---|---|
| RP11-444P10.1 | -2.747403702 | 0.346075114 | | down |
| RP11-84D1.2 | -2.74688643 | 0.014534888 | 0.048731859 | down |
| RPL30P3 | -2.746686003 | 0.099850301 | 0.201687672 | down |
| AC099799.1 | -2.746531466 | 0.162499197 | 0.284473015 | down |
| RP11-420B22.1 | -2.746363001 | 0.240703935 | | down |
| RP11-115D19.4 | -2.7459548 | 0.012659768 | 0.043930478 | down |
| NA | -2.745457043 | 0.142027817 | 0.25884645 | down |
| SLC23A3 | -2.745334541 | 3.98E-15 | 7.92E-13 | down |
| KIF4CP | -2.745128223 | 0.002746698 | 0.013545927 | down |
| RP11-538D16.2 | -2.744788493 | 0.014738173 | 0.049270465 | down |
| NA | -2.743796117 | 5.36E-05 | 0.000564956 | down |
| TTLL7-IT1 | -2.743532108 | 0.063264997 | 0.145327132 | down |
| RP5-849L7.1 | -2.743352244 | 0.242744854 | | down |
| TBC1D3P5 | -2.742965241 | 0.120732169 | 0.231095647 | down |
| RP1-273N12.4 | -2.742366047 | 0.204680831 | 0.334317218 | down |
| RARRES2P4 | -2.741912211 | 0.049530105 | 0.121486037 | down |
| RP11-405A12.2 | -2.741847948 | 0.075956201 | 0.165753209 | down |
| NA | -2.741241274 | 0.125244117 | | down |
| TM4SF19 | -2.740958779 | 0.005131841 | 0.022133367 | down |
| RP11-589B3.6 | -2.740791245 | 0.003116784 | 0.014927573 | down |
| GOLGA8IP | -2.740507281 | 0.007658655 | 0.029983925 | down |
| SCTR | -2.740425642 | 1.67E-08 | 6.17E-07 | down |
| RP11-401P9.7 | -2.740182709 | 2.06E-06 | 3.80E-05 | down |
| RN7SL732P | -2.740043356 | 0.243230277 | 0.377283827 | down |
| RP11-309N24.1 | -2.738977034 | 0.097413887 | 0.198272667 | down |
| PTF1A | -2.738959828 | 0.061052247 | 0.141597477 | down |
| RP11-532N4.2 | -2.738815652 | 0.026732017 | 0.077039494 | down |
| RP1-230L10.1 | -2.738610964 | 0.054161865 | 0.129588203 | down |
| TMEM252 | -2.738418682 | 5.69E-05 | 0.000594104 | down |
| AC079150.3 | -2.738345117 | 0.295490963 | | down |
| NA | -2.738231466 | 0.086296781 | 0.182044898 | down |
| DEGS2 | -2.737506538 | 8.16E-14 | 1.22E-11 | down |
| RP11-2N5.2 | -2.737405512 | 0.121831036 | | down |
| RP11-467L13.4 | -2.737389966 | 5.19E-05 | 0.000551733 | down |
| RPL35P9 | -2.737260996 | 0.010002985 | 0.036685753 | down |
| CTD-2026G6.3 | -2.737242779 | 0.006777912 | 0.027337304 | down |
| HNRNPA1P44 | -2.73711793 | 0.079092572 | 0.170781747 | down |
| RP11-249C24.12 | -2.736854377 | 0.000580719 | 0.003927765 | down |
| FRMPD2B | -2.735868453 | 0.107062912 | 0.212288683 | down |
| RP11-638F5.2 | -2.735844773 | 0.002158408 | 0.01116311 | down |
| RP11-1112J20.2 | -2.735641175 | 0.004981554 | 0.021633392 | down |
| AP000477.3 | -2.735581062 | 0.24014279 | | down |
| DMBT1P1 | -2.735295123 | 0.091611328 | 0.189844857 | down |
| NA | -2.7352715 | 5.16E-06 | 8.14E-05 | down |
| SPINK9 | -2.735264037 | 0.129420584 | 0.242450593 | down |
| UBE2F-SCLY | -2.73519945 | 0.001753924 | 0.009460957 | down |
| RPL21P9 | -2.734840422 | 0.348496133 | | down |
| ATP6V0E1P2 | -2.734640346 | 0.1299549 | | down |
| CTC-479C5.6 | -2.73461575 | 0.244174524 | | down |
| CTB-107G13.1 | -2.734323919 | 0.197605288 | | down |
| DNAH7 | -2.733806555 | 9.93E-13 | 1.12E-10 | down |

| | | | | |
|---|---|---|---|---|
| SNORA51 | -2.733632193 | 0.029715145 | 0.083241195 | down |
| AC104534.2 | -2.733612326 | 1.77E-08 | 6.42E-07 | down |
| NA | -2.733569818 | 0.101199468 | 0.203755315 | down |
| RP5-1096D14.6 | -2.733247854 | 0.019471521 | 0.06082484 | down |
| SLC35G3 | -2.732245106 | 0.000830578 | 0.005234712 | down |
| RP11-58A11.2 | -2.732129712 | 1.15E-05 | 0.000158618 | down |
| FGB | -2.731900993 | 0.032434477 | 0.088837762 | down |
| RPL15P4 | -2.730887983 | 0.295827672 | | down |
| RP13-395E19.2 | -2.730710014 | 0.163223141 | 0.285378559 | down |
| RP11-533K9.3 | -2.730453207 | 0.002218184 | 0.011405634 | down |
| HAUS6P2 | -2.730153684 | 0.349326435 | | down |
| SLC25A47 | -2.729949192 | 1.68E-08 | 6.20E-07 | down |
| GOLGA8S | -2.729898245 | 0.000399689 | 0.002916039 | down |
| RP11-817J15.2 | -2.729836814 | 0.012315312 | 0.043003399 | down |
| PRELID3BP11 | -2.72979203 | 0.030628235 | | down |
| NA | -2.729769474 | 0.084582819 | 0.17932441 | down |
| RP11-356M20.3 | -2.729478841 | 0.00462666 | 0.020438667 | down |
| ADAM2 | -2.729408696 | 0.087607598 | 0.18394901 | down |
| RP11-380J14.1 | -2.72927079 | 0.349428155 | | down |
| FKBP6 | -2.728792962 | 0.004423927 | 0.019733978 | down |
| RP11-324D17.2 | -2.727940217 | 0.097477647 | 0.198341297 | down |
| ALK | -2.727939184 | 6.12E-08 | 1.88E-06 | down |
| HNRNPLP1 | -2.72752983 | 0.003107257 | 0.014894116 | down |
| SHANK2-AS1 | -2.727525767 | 0.011069148 | 0.039642564 | down |
| NA | -2.727078213 | 0.011243573 | | down |
| MYCNOS | -2.726635811 | 0.005806741 | 0.024324835 | down |
| NA | -2.725993839 | 0.041342985 | 0.106481667 | down |
| RP11-606M12.1 | -2.725958364 | 0.244375509 | 0.378337513 | down |
| RP11-538D16.3 | -2.725742343 | 0.008063265 | 0.031125883 | down |
| RP11-299H22.1 | -2.725623242 | 0.138032276 | 0.253781389 | down |
| CTC-428G20.2 | -2.725501431 | 0.02278956 | 0.068318652 | down |
| RNU6-116P | -2.725340261 | 0.295414144 | | down |
| NA | -2.724660074 | 0.159858047 | | down |
| RP11-430B1.1 | -2.724526995 | 1.17E-05 | 0.000159885 | down |
| CTC-297N7.7 | -2.724203184 | 0.298713131 | | down |
| RP11-124D2.7 | -2.723588297 | 0.000783183 | 0.004989387 | down |
| UNC79 | -2.723564972 | 1.82E-09 | 8.71E-08 | down |
| RP11-717H13.1 | -2.723240685 | 0.350446007 | | down |
| MIR519A1 | -2.723217213 | 0.095412554 | 0.19540387 | down |
| HNRNPA1P5 | -2.723047839 | 0.06979554 | 0.155968687 | down |
| NA | -2.722903473 | 0.350613351 | | down |
| RP11-1281K21.8 | -2.722022389 | 0.350769948 | | down |
| RP11-546B8.6 | -2.721473822 | 0.35081236 | | down |
| ERI3-IT1 | -2.721397932 | 0.000112016 | 0.001040846 | down |
| NA | -2.720992542 | 0.000684537 | 0.004478709 | down |
| PRR35 | -2.720566443 | 0.046833374 | 0.116517214 | down |
| DNAH17-AS1 | -2.720295649 | 0.000736512 | 0.00475457 | down |
| CTA-228A9.4 | -2.720252054 | 1.03E-06 | 2.08E-05 | down |
| RP11-197P3.1 | -2.719419346 | 0.203642357 | 0.333064627 | down |
| RP11-152C17.1 | -2.718849807 | 0.351334186 | | down |
| DUSP5P2 | -2.718848751 | 0.089472315 | 0.186780623 | down |

| | | | | |
|---|---|---|---|---|
| RP11-605F20.1 | -2.718163471 | 0.351456325 | | down |
| NA | -2.717808947 | 0.000271169 | 0.002150738 | down |
| KCNH7 | -2.717717538 | 0.009920312 | 0.036413665 | down |
| AC024592.9 | -2.717475395 | 3.35E-07 | 7.96E-06 | down |
| RP11-565F19.3 | -2.717381779 | 0.248772218 | 0.383268167 | down |
| RP11-63M22.1 | -2.717079186 | 8.88E-05 | 0.000856421 | down |
| ABCA13 | -2.716900593 | 2.59E-07 | 6.42E-06 | down |
| RP11-561023.9 | -2.716715579 | 0.103955398 | 0.207587742 | down |
| AC090505.5 | -2.716197098 | 0.126851853 | | down |
| ASH1L-IT1 | -2.715649765 | 0.078702659 | 0.170172197 | down |
| AMN | -2.715416666 | 6.29E-12 | 5.75E-10 | down |
| FRG2KP | -2.715159338 | 0.200077693 | | down |
| KB-1247B1.1 | -2.714786668 | 3.55E-06 | 5.96E-05 | down |
| NA | -2.714786183 | 0.199485589 | | down |
| NA | -2.714766926 | 0.069122137 | 0.154897106 | down |
| CCT8L2 | -2.714105009 | 0.203306076 | 0.332764309 | down |
| NA | -2.713406752 | 0.010259154 | 0.037392457 | down |
| DAND5 | -2.713330463 | 2.88E-06 | 5.03E-05 | down |
| AF064860.5 | -2.712780938 | 0.200043194 | | down |
| AL591893.1 | -2.712699373 | 0.000138306 | 0.001237426 | down |
| RNA5SP447 | -2.71262627 | 0.057369069 | 0.135184024 | down |
| NA | -2.712417553 | 0.246634333 | | down |
| TMEM78 | -2.712280889 | 0.013590657 | 0.046320782 | down |
| RP11-1267H10.4 | -2.71190642 | 0.000812262 | 0.005139767 | down |
| UNC93B8 | -2.711796113 | 0.302756728 | | down |
| RANP8 | -2.711652206 | 0.005583028 | 0.023604912 | down |
| CYP4A22 | -2.711534619 | 0.0048084 | 0.021068041 | down |
| SNORA40 | -2.711353746 | 0.203217295 | | down |
| RP11-362F19.2 | -2.710726158 | 0.130931195 | 0.244496043 | down |
| AC010969.1 | -2.710515521 | 0.165403421 | 0.288202987 | down |
| SLC2A3P4 | -2.710384269 | 0.000442803 | 0.003156248 | down |
| RP11-56I23.1 | -2.709920687 | 0.039725366 | 0.103409966 | down |
| PIEZO1P2 | -2.709027068 | 0.025894092 | 0.075230838 | down |
| RP13-16H11.8 | -2.708811243 | 0.000794261 | 0.005047117 | down |
| RP11-262H14.3 | -2.707676108 | 0.000339182 | 0.002575278 | down |
| CTD-2349P21.5 | -2.706886336 | 5.90E-05 | 0.000612057 | down |
| RP11-565A3.2 | -2.706699683 | 0.088652599 | 0.185530698 | down |
| OR7E91P | -2.706399848 | 0.002130529 | 0.011043758 | down |
| NA | -2.706361607 | 0.007248824 | 0.028704846 | down |
| TMEM213 | -2.706199486 | 0.012224404 | 0.042770465 | down |
| CTD-2299N12.1 | -2.705835384 | 0.353654788 | | down |
| Y_RNA | -2.705820386 | 3.00E-06 | 5.20E-05 | down |
| RP11-433M22.1 | -2.705745595 | 0.249157329 | | down |
| RP11-724M22.1 | -2.705584453 | 0.166589777 | | down |
| RP11-680G24.1 | -2.70554427 | 0.043174131 | 0.110018964 | down |
| RP11-1223D19.1 | -2.704633969 | 0.008103658 | 0.031225757 | down |
| RP11-460M2.1 | -2.70432292 | 0.031875367 | 0.087690094 | down |
| RP11-1250I15.3 | -2.703879075 | 0.022967299 | | down |
| RP11-149I9.2 | -2.703455816 | 0.009850589 | 0.036210829 | down |
| PGDP1 | -2.703451435 | 0.005178631 | 0.022280794 | down |
| ELAVL2 | -2.703435123 | 1.49E-05 | 0.000197391 | down |

| | | | | |
|---|---|---|---|---|
| NA | -2.702633471 | 7.18E-10 | 3.86E-08 | down |
| NUTM1 | -2.702569519 | 0.00031686 | 0.002435965 | down |
| CTC-304I17.6 | -2.702558131 | 3.03E-07 | 7.32E-06 | down |
| NA | -2.702463911 | 0.049439264 | 0.121372222 | down |
| RNU6-1066P | -2.7024155 | 0.133321739 | 0.247401072 | down |
| RPL21P8 | -2.701325397 | 0.020125262 | 0.062392258 | down |
| DKKL1 | -2.70088587 | 1.64E-07 | 4.36E-06 | down |
| RP11-435O5.7 | -2.700616925 | 4.09E-08 | 1.32E-06 | down |
| RP11-615I2.1 | -2.700558972 | 2.41E-05 | 0.000291846 | down |
| RP11-107I14.4 | -2.700524531 | 0.070095323 | 0.156517569 | down |
| RP11-2E11.5 | -2.700510147 | 0.013020082 | 0.044793737 | down |
| RP11-197N18.8 | -2.700172384 | 0.000107043 | 0.001000718 | down |
| NEUROD1 | -2.700131359 | 1.22E-07 | 3.36E-06 | down |
| NA | -2.699997038 | 0.00300396 | 0.014512135 | down |
| RBFOX3 | -2.69995509 | 1.78E-08 | 6.43E-07 | down |
| NA | -2.699905373 | 0.201913195 | | down |
| CTD-2337I7.1 | -2.699771145 | 0.354683523 | | down |
| NA | -2.699492522 | 0.24661732 | | down |
| C6orf222 | -2.698719833 | 3.37E-13 | 4.20E-11 | down |
| OPTC | -2.698646314 | 0.1340406 | | down |
| Z99756.1 | -2.698286316 | 0.164908866 | | down |
| FAM205C | -2.697626142 | 0.100948617 | | down |
| RP11-547D24.3 | -2.697535458 | 0.003493445 | 0.016350997 | down |
| AC068535.2 | -2.697534984 | 0.1691162 | 0.292612711 | down |
| TGM7 | -2.697343357 | 0.302033111 | | down |
| TUBAL3 | -2.697160976 | 1.37E-11 | 1.17E-09 | down |
| RP11-244N9.6 | -2.696867543 | 0.06783962 | 0.152786623 | down |
| RP11-554L12.1 | -2.696503599 | 0.303310468 | | down |
| CALCB | -2.696445242 | 7.59E-05 | 0.000751001 | down |
| RP11-355I22.2 | -2.696363934 | 0.007882431 | 0.030636449 | down |
| RP11-454H13.1 | -2.696097859 | 0.137398366 | 0.252863492 | down |
| PNLIPRP1 | -2.695909362 | 4.46E-05 | 0.000485902 | down |
| NA | -2.695303532 | 4.03E-12 | 3.85E-10 | down |
| RNU6-933P | -2.695183538 | 0.002762219 | 0.01360015 | down |
| RP11-641A6.8 | -2.694890567 | 0.355613835 | | down |
| NWD1 | -2.694788373 | 9.40E-08 | 2.69E-06 | down |
| NA | -2.694318601 | 0.3557164 | | down |
| RP11-478K15.6 | -2.694286384 | 2.41E-06 | 4.32E-05 | down |
| ST7-AS2 | -2.694025758 | 0.136917231 | | down |
| RP11-242G20.1 | -2.693666845 | 0.008919342 | 0.033605806 | down |
| ATP12A | -2.692758643 | 0.016257857 | 0.053030248 | down |
| RN7SKP104 | -2.692185297 | 0.356099112 | | down |
| CBLC | -2.691649195 | 3.64E-11 | 2.83E-09 | down |
| MAP3K15 | -2.691581608 | 9.54E-05 | 0.000908749 | down |
| PDCL2 | -2.691560017 | 0.160133139 | | down |
| RP11-70O5.2 | -2.691077372 | 0.007765194 | 0.030294441 | down |
| SPO11 | -2.690712417 | 0.205282478 | | down |
| RP11-543E8.1 | -2.690603865 | 0.356382986 | | down |
| ITCH-IT1 | -2.690502918 | 0.006194877 | 0.025576319 | down |
| RP11-515C16.1 | -2.69048127 | 0.304283033 | | down |
| TTLL13P | -2.690448312 | 0.010881353 | 0.039128588 | down |

| | | | | |
|---|---|---|---|---|
| RP11-451B8.1 | -2.690184964 | 0.019599077 | 0.061095111 | down |
| GABBR2 | -2.690008523 | 9.75E-06 | 0.000138333 | down |
| LINC01517 | -2.689901684 | 0.013311357 | 0.045544519 | down |
| Y_RNA | -2.689798972 | 0.254182796 | 0.389245421 | down |
| NA | -2.68965533 | 0.012219978 | 0.042763557 | down |
| RP1-261G23.5 | -2.689161186 | 0.074439623 | 0.163436424 | down |
| NLRP8 | -2.688527083 | 0.30553295 | | down |
| AC012593.1 | -2.688524787 | 0.356700127 | | down |
| AC011752.1 | -2.688350156 | 0.026077933 | | down |
| GAPDHP45 | -2.688087147 | 0.11566332 | 0.224163901 | down |
| KRT128P | -2.687979522 | 0.163416622 | | down |
| NA | -2.687698024 | 0.11592963 | 0.224477301 | down |
| RP11-756P10.7 | -2.686214522 | 0.302774638 | | down |
| HNF4G | -2.685943652 | 2.17E-12 | 2.22E-10 | down |
| ACSM2B | -2.685583987 | 2.49E-05 | 0.000299449 | down |
| HES3 | -2.685150525 | 0.357362976 | | down |
| RP11-480I12.9 | -2.684469457 | 1.44E-08 | 5.49E-07 | down |
| FAM166AP1 | -2.684359045 | 0.357505349 | | down |
| HYDIN | -2.684244385 | 0.000272769 | 0.002160579 | down |
| RP11-190D6.1 | -2.68411929 | 0.141266373 | 0.257928101 | down |
| RP11-142A12.1 | -2.684039731 | 0.054993298 | 0.13109725 | down |
| CTA-305I2.1 | -2.683058193 | 0.175609745 | 0.30021732 | down |
| LINC01180 | -2.68280257 | 0.008876003 | 0.033488715 | down |
| RP11-242F24.1 | -2.682650394 | 0.202410004 | | down |
| RP11-744O11.2 | -2.682332161 | 0.08230051 | | down |
| CALCR | -2.682274612 | 0.001769232 | 0.009514156 | down |
| AC092415.1 | -2.681878081 | 0.138394984 | | down |
| IRGC | -2.681869667 | 0.072163268 | 0.159840256 | down |
| RP11-747H7.1 | -2.681848293 | 0.038561864 | 0.101077705 | down |
| OLA1P2 | -2.681527155 | 0.009013927 | 0.03388145 | down |
| RP4-663N10.2 | -2.681172029 | 0.358078999 | | down |
| NA | -2.68072029 | 0.151965172 | 0.271344445 | down |
| RNU6-937P | -2.680544252 | 0.086142145 | 0.181841811 | down |
| NA | -2.680128977 | 0.202736624 | | down |
| RP11-51J9.4 | -2.680024608 | 0.02462123 | 0.072464756 | down |
| Y_RNA | -2.678943554 | 0.30180294 | 0.439629941 | down |
| AC004448.5 | -2.678830814 | 0.004213139 | 0.018994529 | down |
| DNAH12 | -2.678598439 | 5.09E-05 | 0.000542531 | down |
| RP11-219B4.5 | -2.677895288 | 0.001637207 | 0.008948785 | down |
| UBE2V1P5 | -2.677350837 | 0.358710882 | | down |
| L29074.3 | -2.677218707 | 0.207759054 | | down |
| Y_RNA | -2.677062179 | 0.000577529 | 0.003908828 | down |
| CTA-360L10.1 | -2.676945219 | 1.16E-05 | 0.000159722 | down |
| PLCZ1 | -2.676930658 | 0.358843324 | | down |
| Y_RNA | -2.676821995 | 0.165201459 | | down |
| MIR4505 | -2.67673492 | 0.053520054 | 0.128400263 | down |
| AC002429.5 | -2.676544374 | 0.039642507 | 0.103270569 | down |
| ARSF | -2.67632135 | 0.004156138 | 0.018796781 | down |
| DOCK11P1 | -2.675950157 | 0.359020164 | | down |
| SPDYE7P | -2.675297036 | 0.034048489 | 0.092191132 | down |
| CTA-392E5.1 | -2.675204644 | 0.062863958 | 0.144638134 | down |

| | | | | |
|---|---|---|---|---|
| C8orf49 | -2.674773792 | 0.087151203 | 0.183307949 | down |
| RP11-415D17.3 | -2.674720912 | 0.033752618 | 0.091555042 | down |
| RNU6-1092P | -2.674602074 | 0.204113007 | | down |
| MLXIPL | -2.673478846 | 6.72E-14 | 1.03E-11 | down |
| MIR200C | -2.673239706 | 0.131520641 | | down |
| RP11-307I14.2 | -2.673058317 | 0.036428192 | 0.096888155 | down |
| TRIM42 | -2.673006494 | 0.308270783 | | down |
| RP11-246E12.2 | -2.672988364 | 0.013478804 | 0.045991732 | down |
| KB-1568E2.1 | -2.672607896 | 0.084152305 | 0.178613341 | down |
| PAICSP2 | -2.672081882 | 0.134658338 | | down |
| NA | -2.671809915 | 0.000631084 | 0.004203123 | down |
| ERICH3 | -2.671486101 | 1.29E-06 | 2.53E-05 | down |
| LINC01158 | -2.670816782 | 7.22E-05 | 0.00071931 | down |
| AP000997.2 | -2.670805327 | 0.307854128 | | down |
| RPL12P8 | -2.670251694 | 0.010409235 | 0.037833905 | down |
| RP11-16N2.1 | -2.670109165 | 0.208615256 | | down |
| SUN5 | -2.669810843 | 0.360017537 | | down |
| KRT8P20 | -2.668940613 | 0.171650536 | | down |
| RPL13AP2 | -2.668797006 | 0.000241397 | 0.00195539 | down |
| PLCH1-AS1 | -2.668287384 | 0.087476379 | | down |
| RP11-12K11.2 | -2.667888583 | 0.36047619 | | down |
| SDR42E2 | -2.667833487 | 1.75E-06 | 3.30E-05 | down |
| NA | -2.667591177 | 3.32E-06 | 5.66E-05 | down |
| GS1-124K5.13 | -2.66742405 | 0.003150113 | 0.01503447 | down |
| CACNA1C-IT3 | -2.667311704 | 0.257421239 | | down |
| RP11-128P17.2 | -2.667294905 | 0.360583562 | | down |
| Metazoa_SRP | -2.666555953 | 0.042306143 | 0.108367785 | down |
| LINC00621 | -2.666431977 | 0.000212297 | 0.001756613 | down |
| AMBN | -2.66508254 | 0.007239419 | 0.028682725 | down |
| PRDM9 | -2.664048879 | 0.031416368 | 0.086776995 | down |
| RNU6-1035P | -2.664007321 | 0.03443115 | 0.093009303 | down |
| DKFZP434A062 | -2.66400489 | 1.65E-06 | 3.13E-05 | down |
| LINC01194 | -2.663578972 | 0.044691536 | 0.112662571 | down |
| Y_RNA | -2.663354553 | 0.041965843 | 0.107736074 | down |
| RP1-163M9.7 | -2.663003297 | 0.06628014 | 0.150141033 | down |
| B4GALNT2 | -2.662700852 | 2.33E-07 | 5.88E-06 | down |
| CTB-181H17.1 | -2.662110442 | 0.008379224 | 0.032024953 | down |
| SLC25A39P2 | -2.662091615 | 0.083175441 | 0.177203444 | down |
| NDUFA12P1 | -2.661652518 | 0.208226678 | | down |
| NYAP2 | -2.661310996 | 0.000319254 | 0.002450605 | down |
| C2orf54 | -2.661262858 | 6.29E-11 | 4.53E-09 | down |
| AC079779.5 | -2.660881225 | 0.017394362 | 0.055838896 | down |
| NA | -2.660476772 | 3.96E-07 | 9.15E-06 | down |
| NA | -2.660241102 | 0.003141715 | 0.015008467 | down |
| C2orf91 | -2.660130086 | 0.000205391 | 0.001709351 | down |
| LINC01398 | -2.660114055 | 0.009463712 | 0.035150529 | down |
| RP11-451J24.1 | -2.660034734 | 0.361840956 | | down |
| Y_RNA | -2.658877569 | 0.156512647 | 0.277027569 | down |
| RP11-56104.1 | -2.658734513 | 0.043883488 | 0.111232222 | down |
| FGF3 | -2.658673049 | 0.257649624 | | down |
| AC005336.4 | -2.65865003 | 0.001216476 | 0.007091541 | down |

| | | | | |
|---|---|---|---|---|
| CCT4P2 | -2.658640964 | 0.005263758 | 0.022569482 | down |
| SLC2A14 | -2.658512003 | 0.000381122 | 0.002810613 | down |
| Y_RNA | -2.658155593 | 0.067229265 | 0.151764672 | down |
| RP11-9G1.3 | -2.657438606 | 0.021703622 | 0.065944946 | down |
| RP11-418B12.1 | -2.657323084 | 0.362390055 | | down |
| RP11-964E11.2 | -2.657284244 | 0.224068917 | 0.356322554 | down |
| SCb-64M4.1 | -2.656806975 | 0.097469822 | 0.198341297 | down |
| LINC00844 | -2.656471684 | 0.000434794 | 0.003110228 | down |
| RP11-380P13.1 | -2.656058434 | 0.011677966 | 0.041340026 | down |
| RP11-419C5.3 | -2.655998642 | 0.091171405 | 0.189207274 | down |
| AC116609.1 | -2.6555065 | 0.05904171 | 0.138139487 | down |
| CHORDC2P | -2.655273985 | 0.006045425 | 0.025090308 | down |
| CDRT7 | -2.654774609 | 0.031557063 | 0.087045575 | down |
| DUSP9 | -2.654562338 | 0.000996741 | 0.006047273 | down |
| CTD-2291D10.3 | -2.653905212 | 0.129322677 | 0.242365606 | down |
| RP11-66N5.2 | -2.653009214 | 0.090669035 | 0.188503252 | down |
| NA | -2.652897591 | 0.363136035 | | down |
| RP11-10K17.6 | -2.652734408 | 0.312206863 | | down |
| LINC01078 | -2.652256092 | 0.176839562 | | down |
| NXF4 | -2.652230274 | 0.02120816 | 0.064857311 | down |
| METTL7B | -2.651746548 | 4.23E-13 | 5.18E-11 | down |
| NA | -2.65169865 | 5.69E-05 | 0.000594159 | down |
| TERF1P2 | -2.651114881 | 0.259391244 | | down |
| RP5-875O13.1 | -2.651089135 | 0.00158101 | 0.008702799 | down |
| RP13-726E6.2 | -2.651050695 | 0.001023057 | 0.006180722 | down |
| NA | -2.651012045 | 0.001122066 | 0.006653115 | down |
| NA | -2.650954605 | 0.053865313 | | down |
| RP11-10N16.3 | -2.650416667 | 0.140093804 | | down |
| RNU6-56P | -2.650183117 | 0.310752046 | | down |
| RP11-483E23.4 | -2.650085406 | 0.08121671 | 0.174116944 | down |
| STOML3 | -2.650082826 | 0.005625007 | 0.023728968 | down |
| RP13-452N2.1 | -2.650076739 | 6.21E-05 | 0.000638208 | down |
| NA | -2.649847547 | 0.309307593 | | down |
| RNU6-673P | -2.649423271 | 0.20853688 | | down |
| RP11-373D7.1 | -2.648802383 | 0.109738812 | 0.216125392 | down |
| LINC01118 | -2.647246934 | 0.000407346 | 0.002951123 | down |
| SLC5A5 | -2.647052145 | 1.61E-06 | 3.07E-05 | down |
| NA | -2.646903414 | 0.012985519 | 0.04470971 | down |
| NA | -2.646523856 | 0.001125085 | 0.00666707 | down |
| AP000648.4 | -2.646168159 | 0.261801133 | 0.396916106 | down |
| TRDN | -2.646082666 | 6.35E-05 | 0.000650003 | down |
| LINC00488 | -2.645865084 | 0.002334917 | 0.011906014 | down |
| NA | -2.645259193 | 0.364583104 | | down |
| ERVMER61-1 | -2.64420092 | 2.63E-05 | 0.000313946 | down |
| SLC25A34 | -2.644004022 | 4.18E-27 | 1.05E-23 | down |
| SLC39A12-AS1 | -2.64397416 | 0.170899466 | | down |
| C20orf173 | -2.643682857 | 0.212372886 | | down |
| RN7SL89P | -2.643616263 | 0.213817895 | | down |
| NHEJ1 | -2.643606207 | 0.000441076 | 0.003146919 | down |
| AC105398.3 | -2.643439604 | 0.262217935 | | down |
| NA | -2.6434119 | 0.316280939 | | down |

| | | | | |
|---|---|---|---|---|
| WI2-2373I1.2 | -2.643317611 | 0.069559525 | 0.155645072 | down |
| NA | -2.64188783 | 0.000520541 | 0.003594374 | down |
| PGAM4P1 | -2.641464219 | 0.170691869 | | down |
| NA | -2.641385 | 0.031746759 | 0.087440378 | down |
| NA | -2.64100804 | 0.212933872 | | down |
| ZNRF3-AS1 | -2.640583726 | 0.006108472 | 0.025301498 | down |
| RP11-130C19.3 | -2.640563728 | 0.048650367 | 0.120033802 | down |
| ALB | -2.640415509 | 0.000829378 | 0.005229893 | down |
| RP1-81D8.4 | -2.640389053 | 0.260990139 | | down |
| GALNTL5 | -2.640030578 | 0.035211935 | 0.094447583 | down |
| RP11-615I2.6 | -2.639727996 | 6.75E-05 | 0.000681829 | down |
| RP11-706C16.8 | -2.639537795 | 0.365626065 | | down |
| RP11-608O21.1 | -2.639244408 | 0.210856929 | | down |
| LINC00301 | -2.63917756 | 0.093265141 | 0.192277356 | down |
| KRTAP10-1 | -2.639174082 | 0.317400738 | | down |
| LLNLF-136C3.1 | -2.639043062 | 0.365716338 | | down |
| HERC2P8 | -2.638322096 | 0.088338084 | 0.18505295 | down |
| RN7SL233P | -2.638200811 | 0.000329582 | 0.002517662 | down |
| RP11-197P3.5 | -2.637867109 | 1.55E-06 | 2.98E-05 | down |
| AC092646.2 | -2.63764479 | 0.068544784 | 0.153947782 | down |
| NA | -2.637436029 | 0.011594114 | 0.041106155 | down |
| GS1-124K5.9 | -2.635294883 | 0.102173359 | 0.205146961 | down |
| BAAT | -2.635191654 | 0.00109324 | 0.006516928 | down |
| RP11-486E2.1 | -2.634882932 | 0.316314212 | | down |
| DCDC2C | -2.634774262 | 0.004827623 | 0.021127719 | down |
| RP11-110H1.9 | -2.634459208 | 0.314585361 | | down |
| RIMS1 | -2.633809496 | 9.16E-05 | 0.000878515 | down |
| FMO11P | -2.633580147 | 0.366655819 | | down |
| TMPRSS12 | -2.633575561 | 0.12171418 | 0.232397897 | down |
| RP11-43A14.2 | -2.633500687 | 0.265555165 | | down |
| AC004125.3 | -2.633499574 | 0.022871632 | 0.068482101 | down |
| AC007251.2 | -2.633090058 | 0.261121439 | | down |
| Y_RNA | -2.633045222 | 0.184426621 | 0.310432708 | down |
| CTB-11I22.2 | -2.632897831 | 0.096550824 | 0.197157137 | down |
| NA | -2.632885248 | 0.260653327 | | down |
| RP11-346J10.2 | -2.632599563 | 0.091790553 | 0.190150685 | down |
| PSG11 | -2.632464433 | 0.222863777 | 0.354871704 | down |
| CICP6 | -2.632263293 | 0.007677612 | 0.03002332 | down |
| RP5-1181K21.4 | -2.632193352 | 0.315569711 | | down |
| SNORA27 | -2.632088556 | 0.31542036 | | down |
| NA | -2.632055701 | 0.018863043 | 0.059392294 | down |
| RP11-255H23.4 | -2.631540526 | 0.215914267 | | down |
| ELDR | -2.631008473 | 0.213711094 | | down |
| LINC01467 | -2.630519558 | 0.367215425 | | down |
| AC092569.2 | -2.630224992 | 0.010187531 | 0.03719518 | down |
| RP11-708L7.6 | -2.630130496 | 0.264042312 | | down |
| CTA-796E4.4 | -2.630077861 | 0.170655959 | | down |
| MYH7 | -2.629634627 | 1.00E-05 | 0.000141604 | down |
| LINC01625 | -2.629629555 | 3.17E-05 | 0.000366481 | down |
| RP11-408E5.4 | -2.629457248 | 0.141690416 | | down |
| CDK8P2 | -2.628187743 | 0.098344467 | 0.199625781 | down |

| | | | | |
|---|---|---|---|---|
| CTD-2316B1.2 | -2.628066046 | 0.367664416 | | down |
| NA | -2.627993683 | 0.001449764 | 0.008120192 | down |
| RP5-1067M6.3 | -2.627764805 | 0.213591209 | | down |
| APCDD1L-AS1 | -2.627692004 | 5.31E-06 | 8.32E-05 | down |
| RP11-588H7.1 | -2.627604608 | 0.072869916 | | down |
| RP11-154B12.3 | -2.626793618 | 0.017991853 | 0.057369795 | down |
| RP11-1C8.4 | -2.626600158 | 0.063467098 | 0.145646617 | down |
| NA | -2.626429215 | 1.70E-05 | 0.000219104 | down |
| MS4A12 | -2.626213085 | 1.04E-05 | 0.000145371 | down |
| NR1H5P | -2.625729327 | 0.263746835 | 0.39914406 | down |
| PRSS37 | -2.62510995 | 0.106236124 | 0.21102594 | down |
| RP11-618M23.4 | -2.625088395 | 0.262741165 | | down |
| GTF2IP7 | -2.624857962 | 9.86E-06 | 0.000139608 | down |
| ARF4P4 | -2.623644412 | 0.36853304 | | down |
| RP11-258F1.2 | -2.623435642 | 0.083095374 | 0.177116973 | down |
| BRWD1-AS1 | -2.623335769 | 0.017613565 | 0.056434034 | down |
| NA | -2.623300079 | 0.001652253 | 0.009015672 | down |
| DPPA3P2 | -2.622681903 | 0.05454848 | 0.130347136 | down |
| NA | -2.622678172 | 0.261398874 | | down |
| NA | -2.622432138 | 0.26488065 | | down |
| RP11-122C21.1 | -2.622297617 | 0.071958724 | 0.159528329 | down |
| RP11-76908.1 | -2.622277596 | 0.005656159 | 0.023816909 | down |
| LINC00207 | -2.622184463 | 0.368742131 | | down |
| PFN1P3 | -2.622148274 | 0.053720569 | | down |
| LINC00364 | -2.621757836 | 0.139189342 | | down |
| RP13-379024.2 | -2.621675277 | 0.368894202 | | down |
| DEFB1 | -2.621098324 | 2.31E-10 | 1.42E-08 | down |
| CAPN13 | -2.620511452 | 1.56E-09 | 7.56E-08 | down |
| NA | -2.620290982 | 0.264347318 | | down |
| CH507-216K13.2 | -2.619926762 | 0.112651361 | 0.219872506 | down |
| SCARNA15 | -2.619478123 | 0.000364521 | 0.002718884 | down |
| CTD-2296D1.2 | -2.618971552 | 0.369390454 | | down |
| RP11-473J6.1 | -2.618152749 | 0.029884506 | 0.083544253 | down |
| RPL12P37 | -2.61812491 | 0.184502757 | 0.310526067 | down |
| MIR519A2 | -2.618102438 | 0.079279653 | 0.171025347 | down |
| AKR1C4 | -2.617855285 | 0.001874731 | 0.009970687 | down |
| SETP3 | -2.617606954 | 0.016082473 | 0.052622719 | down |
| RP11-941H19.3 | -2.617577095 | 0.013620217 | 0.046389954 | down |
| CCT8L1P | -2.617509845 | 0.075565406 | 0.165206021 | down |
| RP11-648K4.2 | -2.616687923 | 0.369751064 | | down |
| RNA5SP222 | -2.616531845 | 0.264101424 | | down |
| RPL27AP | -2.616479788 | 0.00238942 | 0.012128357 | down |
| C10orf90 | -2.616279344 | 0.00155979 | 0.008603331 | down |
| RMST | -2.616071062 | 0.03414002 | 0.092380686 | down |
| RP11-307N16.7 | -2.615587909 | 0.100195533 | 0.202221859 | down |
| NA | -2.614888519 | 0.08731627 | | down |
| RN7SL399P | -2.614725037 | 0.054432173 | 0.130100254 | down |
| LRRC3B | -2.614658375 | 7.91E-05 | 0.000778042 | down |
| RFPL3S | -2.614312378 | 5.82E-06 | 9.01E-05 | down |
| TCF4-AS2 | -2.613482245 | 0.03148109 | 0.086915813 | down |
| WBSCR28 | -2.61327872 | 0.002583569 | 0.012908824 | down |

| | | | | |
|---|---|---|---|---|
| TMEM236 | -2.612982765 | 5.00E-12 | 4.71E-10 | down |
| RP11-254F7.1 | -2.612908679 | 0.265157 | | down |
| RP3-407E4.3 | -2.612746899 | 0.002868926 | 0.014001628 | down |
| RP11-73G16.1 | -2.612247525 | 0.069449305 | 0.155421595 | down |
| GRM1 | -2.61164699 | 0.007504183 | 0.029502181 | down |
| RN7SL563P | -2.611512233 | 0.370761714 | | down |
| RP11-463H12.2 | -2.611412564 | 0.126250341 | 0.2383754 | down |
| LINC00971 | -2.611014315 | 0.059488829 | 0.138839617 | down |
| RP11-46I1.2 | -2.610804844 | 0.11537418 | | down |
| BPIFA2 | -2.61070497 | 0.034523078 | 0.093190597 | down |
| RP11-301G19.1 | -2.610679294 | 0.034643046 | 0.093395356 | down |
| MGC39584 | -2.610209721 | 0.371001481 | | down |
| ADGRA1 | -2.609707848 | 0.006782494 | 0.027345901 | down |
| OR5BT1P | -2.609582076 | 0.21573323 | | down |
| RP11-140I24.2 | -2.609330662 | 0.154357525 | 0.274117359 | down |
| GSTTP1 | -2.609314925 | 0.039569971 | 0.10312632 | down |
| AC074121.4 | -2.609060538 | 0.041667915 | 0.107180723 | down |
| RP11-370I10.2 | -2.608874952 | 0.113022695 | | down |
| MROH2B | -2.608429037 | 0.000137473 | 0.00123107 | down |
| Y_RNA | -2.608130389 | 0.07541192 | 0.165016439 | down |
| NA | -2.607939455 | 0.049507963 | 0.121455871 | down |
| TRBV23OR9-2 | -2.607764478 | 0.321292487 | | down |
| ACE | -2.60763951 | 1.84E-14 | 3.21E-12 | down |
| RP11-849I19.2 | -2.606653784 | 0.002617673 | 0.013038024 | down |
| Xbac-BPG252P9.1 | -2.606631669 | 0.005116389 | 0.02208404 | down |
| LMNTD1 | -2.606118243 | 0.176731773 | | down |
| ZNF847P | -2.605891514 | 0.018941497 | 0.059580135 | down |
| AF003626.1 | -2.605865487 | 0.166957177 | 0.290113443 | down |
| LINC01548 | -2.605787234 | 0.179326731 | | down |
| LINC00463 | -2.605715142 | 0.006414954 | 0.026265264 | down |
| CTA-280A3.2 | -2.60544598 | 0.153322296 | 0.272956568 | down |
| NOTO | -2.60498868 | 0.024947349 | 0.073173695 | down |
| RP11-702F3.4 | -2.604671918 | 0.32230431 | | down |
| APOL5 | -2.604389622 | 0.020310974 | 0.062816609 | down |
| CTD-2270P14.2 | -2.604329108 | 0.018522997 | 0.058659735 | down |
| GJA8 | -2.603938143 | 0.318302586 | | down |
| HNRNPA1P14 | -2.603772062 | 0.027493596 | | down |
| PPP1R26P1 | -2.603307741 | 0.00040481 | 0.002937197 | down |
| RP11-60O8.2 | -2.603131748 | 0.037670057 | 0.09935556 | down |
| RP3-358H7.1 | -2.602402361 | 0.217818162 | | down |
| UBE3AP2 | -2.600850902 | 0.13357966 | 0.247747842 | down |
| RNU6-1010P | -2.600779483 | 4.83E-05 | 0.000519514 | down |
| SRRM4 | -2.600639109 | 1.67E-05 | 0.000215648 | down |
| HMGN2P18 | -2.599576246 | 0.323438819 | | down |
| RP11-147L13.2 | -2.599459705 | 2.66E-08 | 8.98E-07 | down |
| AC114730.8 | -2.598592767 | 0.012180377 | 0.04266114 | down |
| RP11-432J24.2 | -2.598475093 | 3.15E-05 | 0.000365393 | down |
| XRCC6P5 | -2.598280207 | 0.373201936 | | down |
| RP11-106M7.1 | -2.598150189 | 9.77E-05 | 0.000927358 | down |
| Y_RNA | -2.597954722 | 0.051902824 | 0.125557104 | down |
| NA | -2.597795053 | 0.02271391 | 0.068193936 | down |

| | | | | |
|---|---|---|---|---|
| SAMD11P1 | -2.597724782 | 0.042925427 | 0.109524547 | down |
| RP11-989E6.13 | -2.597571964 | 0.001346332 | 0.007665187 | down |
| RNU6-97P | -2.597189266 | 0.111711432 | 0.218851528 | down |
| RP1-34P24.3 | -2.5966454 | 0.373504111 | | down |
| RP11-862G15.2 | -2.596554778 | 0.012599029 | 0.043755082 | down |
| GOLGA6D | -2.596269306 | 0.035278085 | 0.094540611 | down |
| RP11-138I18.1 | -2.595785289 | 0.02939243 | 0.082645407 | down |
| ZNF259P1 | -2.59576143 | 0.016293083 | 0.053115169 | down |
| CTD-2194D22.3 | -2.595675609 | 0.373623818 | | down |
| RP5-1029K10.2 | -2.595585914 | 0.034599801 | 0.093338997 | down |
| RP1-293L6.1 | -2.595326189 | 0.12375563 | 0.235041037 | down |
| AC078842.3 | -2.59501577 | 0.144170842 | | down |
| SERPINA3 | -2.59462513 | 0.000336619 | 0.002560353 | down |
| CHRNG | -2.594625075 | 5.11E-05 | 0.000543927 | down |
| VCX | -2.594461921 | 0.046130935 | 0.11525369 | down |
| MIR133A1HG | -2.594421745 | 0.000331018 | 0.002527636 | down |
| CTSLP1 | -2.594203323 | 0.14200067 | | down |
| APOBEC2 | -2.594168318 | 1.01E-06 | 2.05E-05 | down |
| CTA-481E9.4 | -2.593557796 | 0.180517875 | | down |
| RP11-297L17.4 | -2.593385603 | 0.147345526 | | down |
| CASC4P1 | -2.593060635 | 0.000105368 | 0.000988347 | down |
| RPS10P18 | -2.592966129 | 0.119848422 | | down |
| NA | -2.592563426 | 0.374259278 | | down |
| GPD1 | -2.59249544 | 1.26E-09 | 6.25E-08 | down |
| RP11-218F4.1 | -2.591901445 | 0.009339559 | 0.034800658 | down |
| SSXP9 | -2.591718431 | 0.111918798 | 0.219015767 | down |
| RP11-709A23.1 | -2.59166695 | 0.163898681 | 0.286205868 | down |
| NA | -2.590986711 | 0.182820067 | | down |
| CA5A | -2.590590779 | 0.058491298 | 0.137200682 | down |
| RP11-20A20.2 | -2.589711278 | 0.267254237 | | down |
| SLC32A1 | -2.589468972 | 0.029911909 | 0.083588104 | down |
| HHATL-AS1 | -2.589205855 | 0.015648751 | 0.051579933 | down |
| HNRNPA1P54 | -2.58897222 | 0.00105176 | 0.006314765 | down |
| NA | -2.588830854 | 0.001936606 | 0.010211196 | down |
| LINC01227 | -2.588057207 | 0.006139348 | 0.025395922 | down |
| CTD-3149D2.3 | -2.587906453 | 0.028390656 | 0.080666622 | down |
| POTEA | -2.587906097 | 0.375062135 | | down |
| MRPS17P9 | -2.587702963 | 0.375159694 | | down |
| NPY | -2.587317425 | 1.59E-09 | 7.68E-08 | down |
| AC010525.4 | -2.587314924 | 3.73E-07 | 8.71E-06 | down |
| RN7SL160P | -2.586805994 | 0.119678532 | 0.229722835 | down |
| RP11-16F15.4 | -2.586707242 | 0.123605893 | 0.234860578 | down |
| ESPN | -2.586540141 | 1.21E-15 | 2.75E-13 | down |
| SIGLEC18P | -2.586367498 | 0.030706243 | 0.085278088 | down |
| Y_RNA | -2.585860478 | 0.180669252 | | down |
| RBPMS2P1 | -2.585660134 | 0.156061686 | 0.276389884 | down |
| TCP11 | -2.58565693 | 0.000903164 | 0.005582114 | down |
| NA | -2.58532767 | 0.000611342 | 0.004099855 | down |
| TNNC2 | -2.583496025 | 2.05E-13 | 2.72E-11 | down |
| NA | -2.582571759 | 0.033010033 | 0.089995774 | down |
| CACNG7 | -2.582135529 | 0.000255122 | 0.002045606 | down |

| | | | | |
|---|---|---|---|---|
| ADAM5 | -2.581656702 | 0.22480084 | | down |
| NPM1P31 | -2.581245252 | 0.0461337 | 0.11525369 | down |
| RP11-113D6.10 | -2.581083565 | 0.046445852 | 0.115792813 | down |
| RPS7P7 | -2.581069536 | 0.14570299 | | down |
| RP11-90P16.1 | -2.580927703 | 0.376417061 | | down |
| PCAT1 | -2.58072591 | 1.86E-08 | 6.68E-07 | down |
| RP11-401G5.1 | -2.580679574 | 0.322955025 | 0.461513545 | down |
| RP11-696L21.2 | -2.580631184 | 0.016473234 | 0.053568768 | down |
| CHP2 | -2.580598101 | 8.13E-11 | 5.67E-09 | down |
| NA | -2.580095467 | 0.016222644 | 0.052960241 | down |
| CTD-2192J16.26 | -2.579828975 | 3.20E-07 | 7.65E-06 | down |
| RP11-95I16.6 | -2.579619895 | 4.37E-07 | 1.00E-05 | down |
| AC005522.7 | -2.579593856 | 0.034691973 | 0.093453387 | down |
| RP11-363N22.2 | -2.57958129 | 0.000150803 | 0.001325552 | down |
| RP11-557H15.3 | -2.579087449 | 0.000875846 | 0.005446886 | down |
| NA | -2.578887883 | 0.326174845 | | down |
| RP11-433J8.2 | -2.578812034 | 0.066171629 | 0.149985697 | down |
| NLRP5 | -2.578383591 | 0.061034596 | 0.141597477 | down |
| DCD | -2.57833773 | 0.042012064 | 0.107824905 | down |
| SPACA6P-AS | -2.577947228 | 0.003357845 | 0.015819838 | down |
| NA | -2.577755912 | 0.000213167 | 0.001762357 | down |
| LHFPL3-AS1 | -2.577688697 | 0.0099084 | 0.036383276 | down |
| RN7SL323P | -2.57717213 | 0.043810821 | 0.111114373 | down |
| NA | -2.577171992 | 0.377115172 | | down |
| LINC00536 | -2.577069171 | 0.229478781 | 0.361888492 | down |
| UGT1A7 | -2.576966357 | 0.013450391 | 0.045931296 | down |
| RP11-106M3.3 | -2.576952334 | 0.000956094 | 0.00584315 | down |
| CD177P1 | -2.576198826 | 0.043752952 | 0.111032494 | down |
| RP11-48F14.1 | -2.576040709 | 0.37732561 | | down |
| RP11-317N12.1 | -2.576027691 | 0.04356171 | 0.110743613 | down |
| DSCAM-IT1 | -2.575872074 | 0.269840878 | | down |
| GACAT1 | -2.575862161 | 0.041156115 | 0.106115847 | down |
| RP13-685P2.7 | -2.575827969 | 0.060643338 | 0.140884914 | down |
| NA | -2.575579276 | 0.013239951 | 0.045336346 | down |
| CTD-3028N15.1 | -2.575219465 | 0.273355566 | | down |
| NTSR2 | -2.574784055 | 0.228458145 | | down |
| CICP10 | -2.574656518 | 0.104570196 | 0.208405106 | down |
| ARHGEF10 | -2.574298675 | 2.67E-06 | 4.71E-05 | down |
| FLJ46284 | -2.574003635 | 1.26E-05 | 0.000171032 | down |
| LVCAT5 | -2.573796492 | 0.138721935 | 0.254671477 | down |
| RP11-222O23.1 | -2.573508963 | 0.042607193 | 0.108925332 | down |
| OR11H2 | -2.573309231 | 0.186638261 | 0.313015514 | down |
| RP1-196A12.1 | -2.572551752 | 0.157541478 | 0.278274806 | down |
| AC012494.1 | -2.572246782 | 0.223676655 | | down |
| AC015923.1 | -2.572205192 | 0.129888173 | 0.243031717 | down |
| RP11-209K10.2 | -2.571991908 | 0.274329481 | | down |
| NA | -2.571964644 | 0.047310884 | 0.117462044 | down |
| SLC7A11-AS1 | -2.571777059 | 0.030195794 | 0.084187309 | down |
| RP11-111E14.1 | -2.57171307 | 0.01251317 | 0.043495548 | down |
| KRT8P8 | -2.571614864 | 0.006779707 | 0.027340876 | down |
| RP11-161D15.1 | -2.571245019 | 0.129684782 | 0.242772109 | down |

| | | | | |
|---|---|---|---|---|
| RP11-1263C18.2 | -2.57122435 | 0.008327867 | 0.031893237 | down |
| RP11-555J4.3 | -2.570174848 | 0.378357376 | | down |
| AC006946.15 | -2.569880235 | 0.005650207 | 0.02379518 | down |
| RP11-151H2.1 | -2.569800489 | 0.082428682 | 0.176099503 | down |
| RP11-124B13.1 | -2.569782136 | 0.132001209 | 0.245989599 | down |
| PAICSP6 | -2.569778034 | 0.086855079 | 0.182818521 | down |
| DOCK9-AS1 | -2.56901353 | 0.073816191 | | down |
| RP4-686C3.7 | -2.568587682 | 0.028551238 | 0.081000319 | down |
| RBMS3-AS2 | -2.568585346 | 8.67E-06 | 0.000125578 | down |
| RP11-354E23.4 | -2.568090405 | 0.092367443 | | down |
| RP11-332H18.3 | -2.567773916 | 0.008830615 | 0.033350973 | down |
| PLB1 | -2.56776015 | 2.46E-12 | 2.45E-10 | down |
| AC106706.1 | -2.567650157 | 0.33001376 | | down |
| OR52N1 | -2.567630842 | 0.37877392 | | down |
| SLC10A1 | -2.567240117 | 0.000391117 | 0.002862535 | down |
| BNIP3P37 | -2.566206622 | 0.13582168 | 0.25085305 | down |
| LINC00599 | -2.566128322 | 0.000870144 | 0.005420386 | down |
| CTD-3187F8.14 | -2.565681846 | 0.012698082 | 0.044017673 | down |
| CTD-2281E23.1 | -2.565210679 | 0.19035129 | 0.317823395 | down |
| NA | -2.564758514 | 0.330226633 | | down |
| KCTD4 | -2.564727571 | 0.000759219 | 0.004869883 | down |
| RP11-146N23.1 | -2.564418692 | 0.000132799 | 0.001196349 | down |
| BCAS2P1 | -2.564296398 | 0.33086004 | | down |
| RP11-339F13.2 | -2.564148785 | 0.193327425 | 0.321434929 | down |
| RP11-509E10.1 | -2.563976628 | 0.06583634 | 0.149394789 | down |
| NA | -2.56387212 | 0.225195024 | | down |
| LEMD1-AS1 | -2.563784133 | 0.092523304 | 0.191180889 | down |
| RP11-478B9.1 | -2.563711068 | 0.12646215 | 0.23865759 | down |
| EMX2 | -2.56329291 | 7.14E-05 | 0.000713501 | down |
| AC019109.1 | -2.56189479 | 0.005876781 | 0.024565979 | down |
| RP11-21L19.1 | -2.561661457 | 0.039263463 | 0.10252316 | down |
| RP11-29P20.1 | -2.561612935 | 0.009164205 | 0.034308955 | down |
| AC000077.2 | -2.561360369 | 0.380062964 | | down |
| SPAG11A | -2.560595726 | 0.232237967 | 0.36489887 | down |
| CFAP74 | -2.560186772 | 1.09E-05 | 0.000150968 | down |
| ADCY8 | -2.56011644 | 0.017190142 | 0.055296504 | down |
| RP11-703H8.7 | -2.559641226 | 0.098844133 | | down |
| TTC39A-AS1 | -2.559641112 | 6.57E-06 | 9.98E-05 | down |
| CTC-525D6.3 | -2.559292294 | 0.380388619 | | down |
| RP11-454F8.4 | -2.558284221 | 0.028178379 | 0.080196357 | down |
| TCERG1L | -2.557835127 | 3.64E-05 | 0.000411745 | down |
| RP11-567L7.3 | -2.557812761 | 0.037779595 | 0.099530879 | down |
| SLC25A5P7 | -2.557575066 | 0.275697103 | | down |
| MIR3680-2 | -2.557259564 | 0.097647897 | | down |
| AC108676.1 | -2.557161893 | 0.003076851 | 0.014785813 | down |
| S100G | -2.557158194 | 0.332625157 | | down |
| NA | -2.556999542 | 0.155023542 | 0.275104978 | down |
| SOCS5P3 | -2.556952605 | 0.380887226 | | down |
| FBN3 | -2.556906482 | 0.000431513 | 0.003091173 | down |
| RP11-431K24.1 | -2.555505134 | 1.90E-05 | 0.000240031 | down |
| CYP2C9 | -2.55543476 | 2.88E-07 | 7.05E-06 | down |

| | | | | |
|---|---|---|---|---|
| POTEC | -2.554848499 | 0.098013864 | | down |
| RP11-76C10.6 | -2.554838299 | 0.05470096 | | down |
| RFPL4AP6 | -2.554084228 | 0.097064784 | 0.197875421 | down |
| RP11-348M3.2 | -2.553347703 | 0.049628588 | 0.121645398 | down |
| BRDT | -2.55320245 | 0.028263396 | 0.080364794 | down |
| PRUNEP1 | -2.552545504 | 0.33328954 | | down |
| EDNRB-AS1 | -2.552484591 | 0.003047109 | 0.0146734 | down |
| OR4D11 | -2.55212897 | 0.381790512 | | down |
| RPL10AP1 | -2.551882752 | 0.034019801 | 0.092146673 | down |
| LINC00355 | -2.551822351 | 0.06087218 | 0.14128892 | down |
| NA | -2.551755693 | 0.38179925 | | down |
| RP11-756D7.2 | -2.551496627 | 0.158752398 | 0.279892107 | down |
| MIR8078 | -2.551310385 | 0.132753931 | 0.246825862 | down |
| RP11-331H2.3 | -2.550888058 | 0.38190357 | | down |
| DDX53 | -2.550756821 | 0.382047704 | | down |
| ACADS | -2.550563995 | 8.08E-16 | 1.97E-13 | down |
| CTD-2562J15.6 | -2.550426431 | 0.002025558 | 0.01060761 | down |
| NA | -2.550293645 | 0.187142794 | | down |
| AC016716.2 | -2.55016344 | 0.015553906 | 0.051363106 | down |
| NEUROG2 | -2.550117675 | 0.15929869 | | down |
| RP11-378J18.9 | -2.550037864 | 0.000398223 | 0.002907466 | down |
| CYP4F60P | -2.55003717 | 0.007964885 | 0.030869068 | down |
| RP11-12907.2 | -2.549812526 | 0.067405499 | 0.152048262 | down |
| LINC01299 | -2.549641999 | 0.187044567 | | down |
| RN7SKP261 | -2.548779634 | 0.027642224 | 0.079032195 | down |
| RPS2P39 | -2.548283845 | 0.3825115 | | down |
| NA | -2.548236832 | 0.234174323 | 0.366943651 | down |
| LINC01360 | -2.548133965 | 0.38253962 | | down |
| RP11-321M21.3 | -2.548098939 | 0.041039634 | 0.105877256 | down |
| SLC22A13 | -2.547135584 | 0.000552683 | 0.003775534 | down |
| RP11-206P5.2 | -2.546826176 | 0.030058654 | 0.083882798 | down |
| RP11-165M1.1 | -2.546645838 | 0.002416068 | 0.012247063 | down |
| CYP2A13 | -2.546531319 | 0.382840386 | | down |
| OR2A15P | -2.546157741 | 0.333929949 | | down |
| RP11-807E13.3 | -2.545832716 | 0.229576202 | | down |
| RP5-1057I20.2 | -2.545772063 | 0.000906336 | 0.005600567 | down |
| NA | -2.545753076 | 0.031753714 | 0.087451514 | down |
| U51561.1 | -2.545591852 | 9.30E-05 | 0.000889575 | down |
| RP11-445J14.1 | -2.545350673 | 0.333301241 | | down |
| FAM153C | -2.545271549 | 7.79E-06 | 0.000114736 | down |
| SNORD88 | -2.54499878 | 0.003536904 | 0.016515812 | down |
| PBLD | -2.544600706 | 2.04E-16 | 5.58E-14 | down |
| RP11-479J7.2 | -2.544377864 | 0.012690591 | 0.043996783 | down |
| RNY1P9 | -2.54389691 | 0.103258553 | | down |
| RPS26P2 | -2.543438659 | 0.10328167 | 0.206597709 | down |
| NA | -2.543429644 | 0.000595988 | 0.004010273 | down |
| CEP57L1P1 | -2.543279173 | 0.201436493 | 0.330545525 | down |
| NA | -2.54321938 | 0.047388032 | 0.117595277 | down |
| HMGB3P17 | -2.542982462 | 0.127631441 | 0.240110144 | down |
| MCOLN3 | -2.542528133 | 7.09E-08 | 2.14E-06 | down |
| LDHAL6CP | -2.541662293 | 0.383755037 | | down |

| | | | | |
|---|---|---|---|---|
| RP11-697M17.2 | -2.540879887 | 0.002298253 | 0.011758979 | down |
| XXyac-YM21GA2.4 | -2.540878883 | 0.000308971 | 0.002388147 | down |
| RP11-756K15.2 | -2.540636603 | 0.196448754 | 0.324934285 | down |
| RP11-173E2.1 | -2.540386177 | 0.022692693 | 0.068157478 | down |
| CDH18 | -2.540327846 | 0.021820724 | 0.066200327 | down |
| MUC22 | -2.539648176 | 0.107937011 | 0.213584901 | down |
| AC008079.10 | -2.539525305 | 4.95E-09 | 2.11E-07 | down |
| CYCSP27 | -2.539455403 | 0.053777469 | 0.128914886 | down |
| RNF151 | -2.538232572 | 0.000249713 | 0.002009741 | down |
| BHLHB9P1 | -2.538212652 | 0.384403863 | | down |
| RP11-480C16.1 | -2.53794892 | 0.082110048 | | down |
| RP11-725G5.3 | -2.537525982 | 0.010085293 | 0.036913155 | down |
| G6PC2 | -2.536979883 | 0.021707307 | 0.065949473 | down |
| AC006227.1 | -2.536347033 | 0.278919427 | 0.415173169 | down |
| FSTL4 | -2.536306415 | 1.56E-07 | 4.18E-06 | down |
| AC099754.1 | -2.536040005 | 0.049584019 | 0.121568651 | down |
| BGLT3 | -2.535874766 | 0.332790227 | | down |
| NA | -2.535667448 | 0.047774446 | 0.11830988 | down |
| ADGRL3-AS1 | -2.535098248 | 0.384869553 | | down |
| NA | -2.534999648 | 0.004842455 | 0.021177105 | down |
| CHL1-AS1 | -2.534676663 | 0.047980176 | 0.118682398 | down |
| uc_338 | -2.534372572 | 0.385126912 | | down |
| AQP12B | -2.534215729 | 1.28E-05 | 0.000173766 | down |
| RP11-561B11.3 | -2.534152218 | 0.108111723 | 0.213814059 | down |
| LINC00917 | -2.533533168 | 0.195908217 | 0.324457787 | down |
| TEX33 | -2.533300905 | 0.231913676 | | down |
| RP11-844P9.6 | -2.533244692 | 5.70E-05 | 0.000594159 | down |
| NA | -2.533029323 | 0.082243186 | 0.175865785 | down |
| SLC25A10 | -2.533020828 | 1.77E-11 | 1.47E-09 | down |
| RP11-528A10.1 | -2.532949684 | 0.089219109 | 0.186430583 | down |
| NA | -2.532890554 | 0.133044024 | 0.247227528 | down |
| RP11-76908.3 | -2.532860989 | 0.010428698 | 0.037890893 | down |
| SLC8A1-AS1 | -2.532257187 | 7.13E-05 | 0.000713448 | down |
| RP11-143G3.1 | -2.532238637 | 0.385529069 | | down |
| AC092162.1 | -2.531908692 | 0.000777813 | 0.004960941 | down |
| LINC01471 | -2.530812141 | 0.031680387 | 0.087305592 | down |
| NA | -2.530472517 | 0.15867496 | | down |
| UGT1A12P | -2.530345167 | 0.280000958 | 0.416308645 | down |
| WDR49 | -2.530048464 | 0.000852419 | 0.005338768 | down |
| ANKRD20A8P | -2.529977703 | 0.154573596 | | down |
| NA | -2.529934736 | 0.017973124 | 0.057329285 | down |
| RP11-331G2.6 | -2.529097163 | 0.2044659 | 0.33409886 | down |
| RP11-549L6.3 | -2.529083068 | 0.072372738 | 0.160209736 | down |
| RP11-328P23.4 | -2.528960569 | 0.140055593 | 0.256371794 | down |
| RPL7AP2 | -2.528706281 | 0.03406963 | 0.09223175 | down |
| CNGB1 | -2.528189932 | 5.44E-06 | 8.50E-05 | down |
| AC104777.2 | -2.526936343 | 0.140717579 | 0.257243633 | down |
| OR10A2 | -2.526871557 | 0.000271417 | 0.002152141 | down |
| AC131097.4 | -2.526674125 | 1.07E-05 | 0.000149539 | down |
| RP11-175D17.3 | -2.526543515 | 0.002161149 | 0.011169319 | down |
| RP11-665C16.6 | -2.526538867 | 0.001539172 | 0.00851305 | down |

| | | | | |
|---|---|---|---|---|
| RP11-435O11.5 | -2.526530221 | 0.052774942 | 0.127089642 | down |
| AP000437.3 | -2.526350732 | 3.46E-06 | 5.85E-05 | down |
| RP11-420K14.2 | -2.526345302 | 0.004138576 | 0.018735089 | down |
| PRAP1 | -2.525797263 | 2.79E-08 | 9.38E-07 | down |
| PDCL3P6 | -2.525477484 | 0.236254319 | | down |
| THAP12P9 | -2.525331885 | 0.204601299 | 0.334233968 | down |
| WASF3-AS1 | -2.524652481 | 0.050162692 | 0.122607217 | down |
| NA | -2.524441992 | 0.233131015 | | down |
| RP11-552I14.1 | -2.524166031 | 0.387052733 | | down |
| RP11-185P18.2 | -2.524129485 | 0.387059639 | | down |
| RP11-168J19.2 | -2.523818323 | 0.241801455 | 0.375785377 | down |
| AC008074.3 | -2.523358579 | 1.95E-07 | 5.08E-06 | down |
| CASC1 | -2.522543284 | 1.63E-06 | 3.11E-05 | down |
| RN7SL51P | -2.522451459 | 0.245035715 | 0.378949555 | down |
| LINC00299 | -2.522390476 | 6.06E-09 | 2.53E-07 | down |
| RN7SL547P | -2.521989472 | 0.198170239 | 0.326899833 | down |
| RP11-63P12.7 | -2.521757898 | 3.24E-05 | 0.000372859 | down |
| C9orf57 | -2.521635794 | 0.059707585 | 0.139187979 | down |
| DNMT3L | -2.521116963 | 0.073183315 | 0.161504299 | down |
| RP11-311F12.1 | -2.520877257 | 0.012840251 | 0.044351106 | down |
| NENFP2 | -2.520475286 | 0.387750562 | | down |
| NA | -2.520370359 | 0.282202889 | 0.418753574 | down |
| RP11-501C14.9 | -2.520337344 | 9.06E-07 | 1.86E-05 | down |
| AC106801.1 | -2.520175616 | 1.95E-05 | 0.000244955 | down |
| ITPK1-AS1 | -2.519983492 | 0.000982153 | 0.005970825 | down |
| FAM19A4 | -2.519534451 | 0.026378371 | 0.076283741 | down |
| RPL19P20 | -2.519404329 | 0.19132053 | | down |
| SNX18P9 | -2.519046605 | 0.083883242 | | down |
| AC017074.1 | -2.518197229 | 0.236879885 | | down |
| RP11-65J3.2 | -2.517760691 | 0.015440246 | 0.051088848 | down |
| LINC01486 | -2.517526765 | 0.388025103 | | down |
| LRRC37A14P | -2.517421778 | 0.005034258 | 0.021812699 | down |
| RP11-47J17.2 | -2.517201524 | 0.003896609 | 0.017867784 | down |
| WDR87 | -2.517176175 | 0.012895108 | 0.044495307 | down |
| FAM71C | -2.517128621 | 0.388383996 | | down |
| RP11-810P12.1 | -2.516772273 | 0.037782095 | | down |
| RP11-255P5.3 | -2.516270125 | 0.008544565 | 0.032499419 | down |
| RPS4XP7 | -2.516119735 | 1.91E-05 | 0.000241645 | down |
| RP11-315L6.1 | -2.515994097 | 0.388598873 | | down |
| RN7SKP90 | -2.515202624 | 0.038852693 | 0.101636073 | down |
| NA | -2.515138211 | 5.36E-09 | 2.27E-07 | down |
| RP11-715J22.2 | -2.515124413 | 0.00156304 | 0.008619673 | down |
| RP11-93K22.7 | -2.514318672 | 0.15463894 | 0.274535976 | down |
| CTC-537E7.2 | -2.514205389 | 0.033957356 | 0.092035616 | down |
| RP11-136K14.3 | -2.513945253 | 0.159764645 | | down |
| NA | -2.513349589 | 0.159480995 | | down |
| WFIKKN1 | -2.513257432 | 6.95E-13 | 8.09E-11 | down |
| FDPSP3 | -2.512889489 | 0.036554881 | | down |
| CTB-49A3.4 | -2.512838396 | 0.339877157 | | down |
| SAMMSON | -2.512306487 | 0.005734638 | 0.024093337 | down |
| RP13-820C6.4 | -2.512129077 | 0.035480613 | 0.094939397 | down |

| | | | | |
|---|---|---|---|---|
| RP5-872K7.8 | -2.512002374 | 0.389292802 | | down |
| HSPB3 | -2.51172802 | 0.000928682 | 0.005717482 | down |
| SLC9A4 | -2.511516484 | 0.005019097 | 0.021762699 | down |
| NAALADL1 | -2.511374674 | 4.50E-16 | 1.16E-13 | down |
| C4orf48 | -2.511013148 | 1.21E-09 | 6.03E-08 | down |
| RNU7-19P | -2.510782822 | 0.077820764 | 0.168738961 | down |
| OR56A1 | -2.510397855 | 0.389659853 | | down |
| RP11-81A1.4 | -2.510194112 | 0.014523886 | 0.04871128 | down |
| RP11-680F8.1 | -2.510184618 | 3.51E-13 | 4.36E-11 | down |
| BRWD1-IT1 | -2.509788872 | 0.027143118 | 0.077969961 | down |
| RP11-117L5.1 | -2.509665531 | 0.012030238 | 0.042273273 | down |
| AC084149.2 | -2.509643459 | 0.063694067 | 0.145955651 | down |
| TRIM64C | -2.509424459 | 0.389844576 | | down |
| ACTN3 | -2.508761279 | 1.38E-05 | 0.000184403 | down |
| RP5-1107A17.4 | -2.508486487 | 0.004890236 | 0.021323986 | down |
| ACCSL | -2.508284261 | 0.132447853 | | down |
| OTX1 | -2.50771568 | 0.004018699 | 0.01831303 | down |
| MNX1-AS2 | -2.5074146 | 8.42E-05 | 0.000819183 | down |
| GATM-AS1 | -2.506561975 | 0.000143038 | 0.001271433 | down |
| NA | -2.506442783 | 0.006888942 | 0.027658875 | down |
| ANKRD20A3 | -2.506283524 | 0.040635667 | 0.105134639 | down |
| MARK2P13 | -2.506145178 | 0.336390803 | | down |
| RNU6-31P | -2.506092613 | 0.089443022 | 0.186763679 | down |
| AC007560.1 | -2.505786156 | 0.092462802 | 0.191121598 | down |
| ZFRP1 | -2.505407765 | 0.390607394 | | down |
| Y_RNA | -2.505058528 | 0.287786604 | | down |
| NA | -2.504849235 | 0.116474635 | 0.22521328 | down |
| RP11-112L6.2 | -2.503571492 | 0.23937522 | | down |
| AMBP | -2.50352227 | 3.59E-06 | 6.02E-05 | down |
| RP11-1E11.2 | -2.503390191 | 0.390990897 | | down |
| LYZL4 | -2.503322346 | 0.158250911 | | down |
| RP11-386E5.1 | -2.502876243 | 0.01684952 | 0.05446229 | down |
| AC018693.5 | -2.502764956 | 0.158382769 | 0.279399443 | down |
| RP11-15K2.2 | -2.502457514 | 0.391105234 | | down |
| AC092431.3 | -2.502386094 | 0.112878379 | 0.220172564 | down |
| RP11-805L22.1 | -2.502076915 | 0.338162964 | | down |
| RP11-305A4.5 | -2.502067336 | 0.100378079 | | down |
| RP11-543F8.2 | -2.501888475 | 0.070556992 | 0.15727713 | down |
| FRG2JP | -2.501861036 | 0.118152825 | 0.227579399 | down |
| AP000469.2 | -2.501641102 | 0.048884419 | 0.120396166 | down |
| NA | -2.501267264 | 1.42E-08 | 5.45E-07 | down |
| AJ006998.2 | -2.501172671 | 1.47E-06 | 2.86E-05 | down |
| RP5-1174N9.2 | -2.500927427 | 1.72E-06 | 3.25E-05 | down |
| RP11-781P14.3 | -2.500752352 | 0.288938807 | 0.426159917 | down |
| RP11-562L8.1 | -2.500714354 | 0.040275887 | 0.104467647 | down |
| AKR7L | -2.500358463 | 9.18E-18 | 3.28E-15 | down |
| GRXCR2 | -2.500229952 | 0.29015343 | | down |
| CCDC33 | -2.500094326 | 0.000146077 | 0.001290422 | down |
| Y_RNA | -2.500071536 | 0.145666009 | 0.263479203 | down |
| CECR2 | -2.499930322 | 2.49E-07 | 6.21E-06 | down |
| C17orf98 | -2.499161384 | 0.011599786 | 0.041121412 | down |

| | | | | |
|---|---|---|---|---|
| CXADRP2 | -2.499135783 | 0.391800328 | | down |
| CRIP1P4 | -2.498564197 | 0.160421878 | | down |
| KRT81 | -2.498251633 | 5.78E-10 | 3.19E-08 | down |
| LINC00885 | -2.497535263 | 0.001927346 | 0.010178776 | down |
| CTB-31N19.2 | -2.497297633 | 0.01308477 | 0.044958695 | down |
| LINC01562 | -2.497158833 | 0.000304529 | 0.002359883 | down |
| NA | -2.497127265 | 0.139814116 | 0.256051153 | down |
| SPAM1 | -2.495772834 | 0.051628015 | 0.12520039 | down |
| RP11-6N13.1 | -2.495158933 | 0.093418937 | 0.192567997 | down |
| NA | -2.495143971 | 0.036881841 | 0.097748591 | down |
| Metazoa_SRP | -2.495040045 | 0.244234819 | | down |
| Metazoa_SRP | -2.494710038 | 0.115428238 | 0.223838248 | down |
| RP11-179G8.1 | -2.494293715 | 0.392722799 | | down |
| CA10 | -2.494235998 | 1.85E-05 | 0.000234649 | down |
| RP3-399L15.1 | -2.494194296 | 0.290134818 | | down |
| FDPSP8 | -2.494115784 | 0.160846139 | | down |
| RPL17P11 | -2.494088273 | 0.102266362 | | down |
| (Xbac-B476C20.1: | -2.493993277 | 3.95E-05 | 0.000439786 | down |
| VN1R7P | -2.493857781 | 0.074289726 | 0.163189575 | down |
| RP11-1094M14.14 | -2.493830448 | 0.054102138 | 0.129476234 | down |
| RP11-862P13.1 | -2.493738167 | 0.008598169 | 0.03266022 | down |
| RP11-147G16.1 | -2.493044512 | 0.070920622 | 0.157777579 | down |
| RP11-621H8.1 | -2.493043838 | 0.287001971 | 0.424177845 | down |
| SNORA7 | -2.492851499 | 0.066391626 | 0.150302917 | down |
| MIR1302-9 | -2.492842891 | 0.003397854 | 0.015983852 | down |
| LINC00448 | -2.49197753 | 0.393164524 | | down |
| MAP3K19 | -2.491802314 | 2.70E-05 | 0.000320934 | down |
| Y_RNA | -2.491791978 | 0.19853002 | | down |
| SLITRK1 | -2.491656011 | 0.005602722 | 0.023658214 | down |
| RP11-386J22.3 | -2.491618515 | 0.000859855 | 0.005368553 | down |
| ASS1P7 | -2.491496947 | 0.067724975 | | down |
| NA | -2.491441877 | 0.242404223 | | down |
| LINC00923 | -2.491349042 | 8.02E-08 | 2.37E-06 | down |
| GNA14-AS1 | -2.491287887 | 0.000229191 | 0.001873178 | down |
| CYP2C18 | -2.490804421 | 6.08E-08 | 1.87E-06 | down |
| CHRNA7 | -2.490771413 | 1.46E-10 | 9.47E-09 | down |
| BLZF2P | -2.4902901 | 0.044051763 | 0.11153642 | down |
| SNORA38 | -2.490180022 | 0.051813293 | 0.12542733 | down |
| RP11-718G2.5 | -2.489921756 | 0.001132474 | 0.00670557 | down |
| RNU4-40P | -2.489868794 | 0.018096042 | 0.057629582 | down |
| RP11-417B4.2 | -2.489716483 | 0.290965045 | | down |
| AC012456.3 | -2.489614904 | 0.393615415 | | down |
| OPRK1 | -2.489532417 | 0.151555944 | 0.270825685 | down |
| RP11-346I3.2 | -2.489009243 | 0.207635474 | 0.337802574 | down |
| GRAMD4P7 | -2.48895031 | 0.29145588 | | down |
| RMDN2-AS1 | -2.488947307 | 0.018001406 | 0.057389032 | down |
| RP11-90P5.5 | -2.488696786 | 0.040310944 | | down |
| LINC00707 | -2.488116102 | 0.000189218 | 0.001599115 | down |
| RNU6ATAC14P | -2.48809881 | 0.096770217 | 0.197503906 | down |
| VWA3A | -2.487841026 | 3.78E-06 | 6.29E-05 | down |
| RP6-114E22.1 | -2.487645251 | 0.007127733 | 0.028367414 | down |

| | | | | |
|---|---|---|---|---|
| NA | -2.486724101 | 1.87E-06 | 3.49E-05 | down |
| RN7SL621P | -2.486349071 | 0.294013557 | 0.43144199 | down |
| TUBA3FP | -2.485927569 | 6.15E-08 | 1.88E-06 | down |
| Y_RNA | -2.485536588 | 0.10331473 | 0.206643216 | down |
| CTD-3138B18.6 | -2.485011935 | 5.00E-06 | 7.93E-05 | down |
| RP11-109P6.2 | -2.484412224 | 0.394609412 | | down |
| RP11-625I7.1 | -2.483889117 | 0.080071391 | 0.172362605 | down |
| GLRA1 | -2.483762571 | 0.059125694 | 0.138249853 | down |
| CTA-249B10.1 | -2.483761212 | 0.394347787 | | down |
| CTA-481E9.2 | -2.48367813 | 0.394749786 | | down |
| RP5-1111A8.3 | -2.48353196 | 0.39477774 | | down |
| LINC01411 | -2.483304786 | 0.004918269 | 0.021418247 | down |
| NBPF21P | -2.483101842 | 0.291805126 | 0.429018508 | down |
| RP11-1212A22.8 | -2.482966378 | 0.061506604 | 0.142410087 | down |
| NA | -2.482850432 | 0.027325019 | 0.078357629 | down |
| AQP12A | -2.482227283 | 1.93E-05 | 0.000243746 | down |
| RP11-74M13.4 | -2.482086532 | 0.0058209 | 0.024373954 | down |
| MUC17 | -2.481739616 | 2.03E-07 | 5.23E-06 | down |
| LINC01342 | -2.481438588 | 1.05E-05 | 0.00014745 | down |
| NA | -2.481284644 | 0.175820133 | 0.3004915 | down |
| RP11-80N2.2 | -2.480653542 | 0.212481499 | 0.343270106 | down |
| DEFB123 | -2.479332389 | 0.215305152 | 0.346355479 | down |
| DUXAP2 | -2.478980992 | 0.293114664 | | down |
| RP11-174G6.1 | -2.478899933 | 0.017871427 | 0.057077619 | down |
| RNU6ATAC20P | -2.478649371 | 0.094292648 | | down |
| RP11-724N1.1 | -2.478422999 | 0.019613242 | 0.061123869 | down |
| SLC9A3P2 | -2.478167987 | 0.395804402 | | down |
| SRP72P1 | -2.47802609 | 0.247817828 | 0.382111307 | down |
| MIR149 | -2.477709235 | 0.014733211 | 0.049259359 | down |
| RP11-219H23.1 | -2.477661541 | 0.251585311 | 0.386403707 | down |
| AC090505.6 | -2.477648845 | 0.024322758 | 0.071810794 | down |
| NLRP9P1 | -2.47757969 | 0.020656983 | 0.06358732 | down |
| UBE2V2P1 | -2.477353828 | 0.017920975 | 0.057211535 | down |
| AC111200.2 | -2.477061867 | 0.194573157 | 0.322898581 | down |
| RPL21P129 | -2.476920647 | 0.056789542 | 0.134232301 | down |
| RP11-30J20.1 | -2.476904246 | 0.000143589 | 0.001275192 | down |
| Y_RNA | -2.476496661 | 0.163847529 | 0.286149809 | down |
| CTD-2385L22.2 | -2.476352903 | 2.29E-06 | 4.15E-05 | down |
| RP11-366H4.3 | -2.476262589 | 0.396105494 | | down |
| CTC-55802.1 | -2.475715451 | 1.71E-06 | 3.24E-05 | down |
| KCNG4 | -2.47569824 | 0.173717041 | 0.298269098 | down |
| OR5M2P | -2.475472324 | 0.396320958 | | down |
| RP11-147L13.7 | -2.475458588 | 0.007870058 | 0.030606154 | down |
| LINC00851 | -2.475318792 | 0.396350391 | | down |
| C8orf37-AS1 | -2.474386489 | 1.38E-06 | 2.68E-05 | down |
| RP11-325K19.1 | -2.474256968 | 0.240706221 | | down |
| FAM60BP | -2.473757597 | 0.135132854 | | down |
| RP11-262I2.2 | -2.473475725 | 0.01796886 | 0.057321769 | down |
| AC002044.1 | -2.473448815 | 3.37E-05 | 0.000384446 | down |
| NA | -2.473340294 | 0.348458197 | | down |
| FGFR3 | -2.472374401 | 1.36E-13 | 1.96E-11 | down |

| | | | | |
|---|---|---|---|---|
| RP11-390B4.3 | -2.472260823 | 0.021429331 | 0.06530968 | down |
| RP11-406A9.2 | -2.471968659 | 0.071130239 | 0.158123338 | down |
| RN7SL311P | -2.471911649 | 0.397003894 | | down |
| TREHP1 | -2.471885648 | 0.009021698 | 0.033897934 | down |
| CSMD1 | -2.471615855 | 0.000159808 | 0.00138869 | down |
| MT-TC | -2.471385572 | 6.36E-05 | 0.000650704 | down |
| RP11-1338A24.1 | -2.470741634 | 0.397228457 | | down |
| FAM167A-AS1 | -2.470248851 | 0.006371262 | 0.026114353 | down |
| RAB17 | -2.470072596 | 1.05E-13 | 1.54E-11 | down |
| RP11-32K4.1 | -2.469948033 | 0.349789956 | | down |
| CTC-360P9.4 | -2.469801671 | 0.349856647 | | down |
| RP11-526P5.2 | -2.469571443 | 0.246064092 | | down |
| MIR200A | -2.469106853 | 0.003430106 | 0.016119835 | down |
| MMD2 | -2.469072661 | 0.081280561 | 0.174228964 | down |
| UGT2B7 | -2.469056874 | 5.89E-07 | 1.30E-05 | down |
| NA | -2.46893196 | 0.000403255 | 0.002931381 | down |
| RP11-9M16.3 | -2.46882461 | 0.206689478 | | down |
| GRIN2B | -2.468707814 | 1.07E-05 | 0.00014923 | down |
| LINC00639 | -2.468669611 | 2.53E-08 | 8.64E-07 | down |
| RP11-589F5.4 | -2.468658814 | 0.001158885 | 0.006817431 | down |
| NA | -2.468596909 | 0.005069769 | 0.021934928 | down |
| RP11-354K4.1 | -2.468222791 | 0.060628651 | 0.140866496 | down |
| RPS3P7 | -2.468146204 | 0.295242766 | | down |
| POTEG | -2.468121743 | 0.019597856 | 0.061095111 | down |
| NA | -2.46748865 | 0.242932618 | | down |
| SERPINA4 | -2.467080797 | 0.015157359 | 0.050330206 | down |
| RP11-544I20.2 | -2.467065242 | 3.26E-05 | 0.00037494 | down |
| RPSAP48 | -2.467035197 | 0.047567293 | 0.117894057 | down |
| SLC35G5 | -2.467013187 | 1.65E-05 | 0.000214604 | down |
| UOX | -2.466685901 | 0.001193027 | 0.006982913 | down |
| RP11-720L2.2 | -2.46667713 | 0.398009158 | | down |
| TRPV5 | -2.466237146 | 0.025427037 | 0.074204433 | down |
| ZFHX4-AS1 | -2.465491697 | 0.021064227 | 0.064521926 | down |
| ABHD15-AS1 | -2.465386799 | 0.016647432 | 0.053983317 | down |
| LINC01102 | -2.465146616 | 0.000723508 | 0.004686736 | down |
| RP11-617D20.2 | -2.464587976 | 0.245924786 | | down |
| RP11-148I19.1 | -2.464022593 | 0.398455095 | | down |
| RP11-278H7.4 | -2.462844819 | 0.003124277 | 0.014956306 | down |
| LINC00461 | -2.462474483 | 5.02E-05 | 0.000537559 | down |
| PEBP1P3 | -2.462362306 | 0.000299722 | 0.00232864 | down |
| RP11-298J23.8 | -2.46229716 | 0.001671052 | 0.009091832 | down |
| LA16c-380H5.1 | -2.46226049 | 1.86E-06 | 3.47E-05 | down |
| GUSBP2 | -2.46215787 | 4.33E-09 | 1.87E-07 | down |
| C2orf78 | -2.461704506 | 0.354872856 | | down |
| PSG4 | -2.461490243 | 0.081311903 | 0.174283713 | down |
| RP11-545M8.4 | -2.461399986 | 0.108816981 | | down |
| OR7E122P | -2.461297389 | 0.011218462 | 0.040047702 | down |
| RP11-504I13.2 | -2.46128133 | 0.019174233 | 0.060164036 | down |
| LINC01476 | -2.461196056 | 0.001011201 | 0.006118939 | down |
| CTD-2280E9.1 | -2.460574305 | 0.026717487 | 0.077012395 | down |
| VN1R110P | -2.460550263 | 0.250501528 | | down |

| | | | | |
|---|---|---|---|---|
| AC073987.1 | -2.46043574 | 0.013773074 | 0.046761149 | down |
| XXyac-YX155B6.5 | -2.460008215 | 4.83E-06 | 7.72E-05 | down |
| RP11-650L12.1 | -2.459370959 | 0.104821063 | 0.20883285 | down |
| RP11-340E6.1 | -2.458961989 | 0.187366202 | 0.31393844 | down |
| SPAG11B | -2.458310625 | 0.247921869 | | down |
| ALX4 | -2.458138271 | 0.01727797 | 0.055530522 | down |
| RP11-136K7.2 | -2.458091083 | 0.006082121 | 0.025211264 | down |
| NA | -2.457895003 | 0.063689389 | 0.145955651 | down |
| CTD-2047H16.5 | -2.457768729 | 0.074248463 | | down |
| RP11-390E23.3 | -2.457655497 | 0.295764086 | 0.433227839 | down |
| AP003774.4 | -2.457141139 | 3.00E-07 | 7.26E-06 | down |
| RP11-31H15.2 | -2.456755964 | 0.399918665 | | down |
| MIR7-3HG | -2.456613854 | 0.000135362 | 0.001214334 | down |
| RP11-352D13.5 | -2.456297662 | 0.032693174 | 0.089350875 | down |
| NA | -2.456289753 | 0.082309064 | 0.175944224 | down |
| RP11-99J16__A.2 | -2.456213074 | 0.008685913 | 0.032895445 | down |
| ALG1L11P | -2.45608447 | 0.018734845 | 0.059100136 | down |
| NA | -2.456032913 | 0.17237962 | 0.296687289 | down |
| SLC25A39P1 | -2.455965789 | 0.205082072 | | down |
| RP1-281H8.3 | -2.45590121 | 0.010389588 | 0.037776204 | down |
| RP11-67704.4 | -2.455345005 | 0.065625675 | 0.149029311 | down |
| CTC-420A11.2 | -2.455017313 | 0.400253865 | | down |
| LRRTM4 | -2.454586458 | 0.000248062 | 0.001999331 | down |
| RPL7L1P3 | -2.454581669 | 0.051766355 | | down |
| AMTN | -2.454387261 | 0.400375376 | | down |
| CENPVL3 | -2.454198536 | 0.026217432 | 0.075949911 | down |
| RP11-277J6.2 | -2.454170846 | 0.353477133 | | down |
| RP11-303E16.3 | -2.453521498 | 0.041573992 | 0.10699409 | down |
| RP11-617J18.1 | -2.453500581 | 0.106009269 | 0.210700085 | down |
| AC130464.1 | -2.45315832 | 0.252346062 | 0.387304474 | down |
| RPS26P11 | -2.452880985 | 0.005953586 | 0.024808535 | down |
| CEACAMP2 | -2.452625935 | 0.400715181 | | down |
| OR2T32P | -2.452269169 | 0.400784031 | | down |
| NA | -2.452025166 | 0.352460086 | | down |
| BLACE | -2.451392662 | 0.182241566 | 0.307806701 | down |
| ZSCAN4 | -2.451026971 | 0.003279607 | 0.015526576 | down |
| FAM60DP | -2.450785622 | 0.023412073 | 0.069749935 | down |
| RP11-599B13.9 | -2.450139338 | 0.125161305 | 0.236843758 | down |
| NA | -2.449234467 | 0.117779156 | | down |
| RP11-175P13.2 | -2.449203697 | 0.002328633 | 0.011880022 | down |
| RP5-894D12.5 | -2.449152215 | 0.000638046 | 0.00423447 | down |
| NOX5 | -2.448988222 | 9.01E-07 | 1.85E-05 | down |
| IRS4 | -2.448758692 | 0.354959894 | | down |
| IYD | -2.448664764 | 2.20E-09 | 1.03E-07 | down |
| RP11-678G14.3 | -2.448664661 | 1.98E-07 | 5.12E-06 | down |
| YIPF7 | -2.448494209 | 0.091069842 | 0.189087928 | down |
| BPIFA4P | -2.448420644 | 0.401527189 | | down |
| RSPH10B2 | -2.44820075 | 0.044047004 | 0.11153377 | down |
| TDRD1 | -2.44743838 | 8.70E-05 | 0.000840258 | down |
| POU6F2 | -2.447394483 | 0.000547646 | 0.003746235 | down |
| RPL23AP96 | -2.447271089 | 0.296490524 | 0.433909321 | down |

| | | | | |
|---|---|---|---|---|
| NA | -2.446851479 | 0.009219187 | 0.034467552 | down |
| AC011747.6 | -2.446709647 | 0.208063026 | | down |
| RBPJL | -2.446457039 | 0.012143249 | 0.042555906 | down |
| SPACA3 | -2.445931354 | 8.70E-05 | 0.000840319 | down |
| RP11-43P8.2 | -2.445174837 | 0.006565404 | 0.026702448 | down |
| RP11-284F21.9 | -2.445025482 | 1.26E-12 | 1.37E-10 | down |
| RP11-813B8.1 | -2.445020335 | 0.402184481 | | down |
| TUBA3GP | -2.444774584 | 0.40223201 | | down |
| LINC01141 | -2.444672795 | 0.000401362 | 0.002921155 | down |
| RP11-474D1.3 | -2.444266313 | 0.03295626 | 0.089881797 | down |
| RP11-28001.2 | -2.444069183 | 0.005679843 | 0.023896543 | down |
| LINC00929 | -2.444065867 | 0.251209153 | | down |
| HOXB1 | -2.443915009 | 0.032922449 | 0.089822199 | down |
| AC003664.1 | -2.44359775 | 0.171811761 | | down |
| PHBP11 | -2.443593775 | 0.402460431 | | down |
| RNU6-338P | -2.443346522 | 0.357600591 | | down |
| RP4-718J7.4 | -2.443278413 | 0.01426943 | 0.048103492 | down |
| GH2 | -2.443244515 | 0.402528009 | | down |
| AC091801.1 | -2.443199271 | 0.018925175 | 0.059538007 | down |
| CTD-2256P15.5 | -2.442526378 | 0.001974137 | 0.010372677 | down |
| Y_RNA | -2.442206098 | 0.068300808 | 0.15353756 | down |
| KRBOX1-AS1 | -2.442077133 | 0.301911923 | | down |
| HMGN2P22 | -2.441934488 | 0.357201926 | | down |
| Y_RNA | -2.441240296 | 0.094616307 | 0.194422566 | down |
| RNU6-1247P | -2.441030757 | 0.043439419 | 0.110516886 | down |
| TAT-AS1 | -2.440967364 | 0.000895509 | 0.005545066 | down |
| RN7SKP224 | -2.440958321 | 0.402970526 | | down |
| RP11-266L9.1 | -2.440899767 | 0.023886793 | 0.070837509 | down |
| NA | -2.440773272 | 2.97E-06 | 5.15E-05 | down |
| NA | -2.440725673 | 0.403015574 | | down |
| RP11-610P16.1 | -2.43970181 | 0.000780742 | 0.004977005 | down |
| ROS1 | -2.439643651 | 0.001146736 | 0.006763366 | down |
| AP000692.9 | -2.439457912 | 0.000148692 | 0.00131085 | down |
| GREB1L | -2.439228844 | 8.50E-06 | 0.000123355 | down |
| DNAH11 | -2.43914382 | 7.14E-05 | 0.000714045 | down |
| PDCD6IPP1 | -2.439099198 | 0.001886778 | 0.010019448 | down |
| RP11-748L13.2 | -2.43909592 | 0.21165133 | | down |
| CFAP52 | -2.439044223 | 0.000850792 | 0.005331813 | down |
| RNF6P1 | -2.438980065 | 0.012704469 | 0.044029654 | down |
| CTD-2062F14.3 | -2.438780181 | 0.03516588 | | down |
| RNU6-874P | -2.438529359 | 0.215850128 | 0.34697706 | down |
| NA | -2.438377256 | 0.255850732 | 0.39089853 | down |
| GGTLC3 | -2.43834024 | 0.11188898 | | down |
| RPL9P28 | -2.438032171 | 0.000138823 | 0.001240566 | down |
| AC092620.2 | -2.437817798 | 0.00138546 | 0.007835986 | down |
| RP11-95I16.2 | -2.437793705 | 0.357299507 | | down |
| RP11-58K22.4 | -2.437590504 | 0.036371189 | 0.096787954 | down |
| TPM4P1 | -2.437473826 | 0.135906107 | 0.250934399 | down |
| NA | -2.437430337 | 0.089022574 | | down |
| RP11-173D3.4 | -2.43688021 | 0.359019799 | | down |
| DRGX | -2.43627037 | 0.000270879 | 0.002149572 | down |

| | | | | |
|---|---|---|---|---|
| RN7SL764P | -2.436032087 | 0.403797269 | | down |
| APOC2 | -2.435671235 | 0.016833131 | | down |
| ATP11AUN | -2.435119713 | 0.024478379 | 0.072178665 | down |
| UBTFL9 | -2.434859046 | 0.02441416 | 0.072038797 | down |
| SLC4A1 | -2.434404286 | 0.000652258 | 0.004309751 | down |
| NA | -2.434278503 | 0.170683255 | | down |
| RP11-754I20.1 | -2.43421278 | 0.404277892 | | down |
| NA | -2.434113844 | 4.05E-07 | 9.33E-06 | down |
| RP11-25I9.2 | -2.433901129 | 0.404338355 | | down |
| RNU6-919P | -2.433812168 | 0.251838966 | | down |
| RPL7P46 | -2.433378556 | 0.166163995 | | down |
| RP11-606D9.1 | -2.433306673 | 0.356998452 | | down |
| OR51D1 | -2.433099527 | 0.080566065 | 0.173080218 | down |
| COL2A1 | -2.432856573 | 4.22E-06 | 6.91E-05 | down |
| RN7SL380P | -2.432679934 | 0.261189051 | 0.396361443 | down |
| RP11-117L5.3 | -2.432671274 | 0.208350182 | | down |
| NA | -2.432261631 | 0.000576092 | 0.003902617 | down |
| RP11-586D19.2 | -2.432115646 | 0.013209416 | 0.045267901 | down |
| ADAM6 | -2.432086645 | 0.358087793 | | down |
| NA | -2.431993419 | 0.404708583 | | down |
| POU5F1P6 | -2.431337756 | 0.000101774 | 0.000959732 | down |
| RP1-74B13.2 | -2.431064977 | 0.250727706 | | down |
| UGT2B10 | -2.430749222 | 0.01100146 | 0.039469289 | down |
| KCNQ1OT1 | -2.430588594 | 2.40E-10 | 1.47E-08 | down |
| HAO1 | -2.43050258 | 0.133164905 | | down |
| SPTA1 | -2.430402663 | 0.008911297 | 0.033583919 | down |
| TTBK1 | -2.430182682 | 2.86E-13 | 3.68E-11 | down |
| PLPPR3 | -2.430069473 | 1.20E-05 | 0.000163837 | down |
| RPL12P14 | -2.429809556 | 7.00E-10 | 3.80E-08 | down |
| LRRC71 | -2.42935958 | 8.53E-07 | 1.77E-05 | down |
| MTND6P3 | -2.429232037 | 7.98E-07 | 1.67E-05 | down |
| RP11-354K1.2 | -2.428971369 | 0.037617099 | | down |
| RP4-683M8.2 | -2.428753177 | 0.126547614 | 0.238733618 | down |
| RP11-194N12.2 | -2.42866389 | 0.00215735 | 0.011159681 | down |
| SLC25A41 | -2.428202126 | 9.22E-07 | 1.89E-05 | down |
| CACNG1 | -2.428096515 | 0.178265484 | | down |
| RP11-337C18.8 | -2.427878133 | 0.010689551 | 0.038609866 | down |
| LINC00608 | -2.427472835 | 0.218185583 | 0.349571654 | down |
| NLRP10 | -2.427408227 | 0.405599257 | | down |
| NA | -2.426747923 | 0.254022993 | | down |
| GACAT3 | -2.426669493 | 0.008473137 | 0.032293152 | down |
| RAD51AP1P1 | -2.426655963 | 0.153694468 | 0.273359968 | down |
| RP13-614K11.2 | -2.42607177 | 0.206912743 | | down |
| RPL7P15 | -2.426068656 | 0.000378931 | 0.002798143 | down |
| FOXL2NB | -2.425974119 | 0.044748121 | 0.112748453 | down |
| KCTD8 | -2.425441647 | 0.013448724 | 0.045930826 | down |
| RBBP8NL | -2.424792289 | 1.32E-10 | 8.75E-09 | down |
| UBQLN1P1 | -2.424676802 | 0.212936793 | | down |
| RP1-79C4.1 | -2.424485089 | 0.193101928 | 0.321131093 | down |
| RP11-138I17.1 | -2.423930689 | 0.005881755 | 0.024577393 | down |
| RN7SL849P | -2.423858867 | 0.058795843 | 0.137692908 | down |

| | | | | |
|---|---|---|---|---|
| NA | -2.423534819 | 0.406286643 | | down |
| RP3-340B19.3 | -2.42347127 | 0.02264056 | 0.068041712 | down |
| RP11-189G24.2 | -2.423407875 | 0.2555967 | | down |
| RP11-157P23.2 | -2.423256979 | 0.069186748 | 0.154983226 | down |
| FAM92A1P2 | -2.423059053 | 0.304412268 | | down |
| UBTFL11 | -2.422179537 | 0.056322322 | 0.133418579 | down |
| SPATA31D2P | -2.421841565 | 0.406616155 | | down |
| RP11-1038A11.1 | -2.42182985 | 0.090573471 | | down |
| NA | -2.421630469 | 0.000684315 | 0.004478229 | down |
| RP11-318K12.1 | -2.421629328 | 0.304738146 | 0.442789227 | down |
| AMD1P1 | -2.421627261 | 0.209762255 | | down |
| LINC01213 | -2.421548988 | 0.022873715 | 0.068482101 | down |
| LRFN2 | -2.421432422 | 4.94E-05 | 0.000530583 | down |
| NALCN | -2.4213487 | 1.13E-05 | 0.000155875 | down |
| RP11-323I14__A.1 | -2.421339871 | 0.209516188 | | down |
| NA | -2.421225041 | 0.072309467 | 0.160116866 | down |
| RP11-547D23.1 | -2.420722083 | 0.037777444 | 0.099530879 | down |
| NA | -2.420683566 | 0.027633868 | 0.07901703 | down |
| RP4-635A23.6 | -2.420504348 | 2.95E-06 | 5.13E-05 | down |
| TTC6 | -2.420012193 | 4.59E-05 | 0.000497997 | down |
| Metazoa_SRP | -2.41986954 | 0.143957267 | | down |
| PWRN1 | -2.419730391 | 0.000561295 | 0.003819604 | down |
| SLC17A2 | -2.419186642 | 0.407199222 | | down |
| EAF1-AS1 | -2.419084697 | 2.70E-05 | 0.000320934 | down |
| AC079612.2 | -2.418976557 | 0.057435433 | 0.135283047 | down |
| SORCS3 | -2.418917197 | 3.06E-05 | 0.000355482 | down |
| DNAJB5P1 | -2.41861868 | 0.180625378 | 0.305995334 | down |
| CTC-436K13.5 | -2.418164666 | 0.36309047 | | down |
| MYL6BP1 | -2.417862711 | 0.069237562 | 0.155074796 | down |
| CTD-2340E1.2 | -2.417118219 | 0.1525873 | 0.272053238 | down |
| GPR6 | -2.417103951 | 0.407605121 | | down |
| RAET1L | -2.416993641 | 0.026508314 | 0.076571079 | down |
| LINC01231 | -2.416926987 | 0.226244456 | 0.358317788 | down |
| RP11-215P8.3 | -2.416710099 | 1.22E-06 | 2.42E-05 | down |
| NA | -2.416366589 | 0.020277047 | 0.062756228 | down |
| LINC01121 | -2.41565393 | 0.004563064 | 0.020219573 | down |
| RP4-734C18.1 | -2.415649293 | 0.057927908 | 0.136255074 | down |
| RP11-72I2.2 | -2.415039529 | 0.40794145 | | down |
| CTC-573M9.1 | -2.413964867 | 0.1409064 | | down |
| RP1-35C21.2 | -2.413793505 | 0.213654454 | | down |
| CTC-325J23.2 | -2.413655281 | 0.001258066 | 0.007272916 | down |
| RP11-2H8.4 | -2.413469089 | 6.14E-05 | 0.000633048 | down |
| RNU1-14P | -2.413392763 | 0.359899267 | | down |
| RP11-6O2.2 | -2.41326628 | 0.005890707 | 0.024607953 | down |
| HOXC5 | -2.412875453 | 0.048075351 | | down |
| NA | -2.412587841 | 1.63E-05 | 0.000212688 | down |
| TEX37 | -2.412144723 | 0.257624918 | | down |
| ABCC6P1 | -2.411566369 | 4.04E-10 | 2.34E-08 | down |
| RP11-445N18.7 | -2.411405977 | 0.074125562 | 0.162925481 | down |
| QRFPR | -2.411288118 | 0.019848487 | 0.061693352 | down |
| NA | -2.410473141 | 0.408769397 | | down |

| | | | | |
|---|---|---|---|---|
| AP002381.2 | -2.410203172 | 0.066207484 | | down |
| NA | -2.410143673 | 0.010956066 | 0.039359565 | down |
| NA | -2.410023793 | 2.06E-05 | 0.000257665 | down |
| GBX1 | -2.409864463 | 0.262926885 | | down |
| RP11-349A8.3 | -2.409558031 | 0.05616125 | | down |
| RP1-92C8.3 | -2.409503294 | 0.105518053 | | down |
| PARP1P1 | -2.40942544 | 0.087249211 | 0.183460594 | down |
| AC006273.5 | -2.409224726 | 8.42E-08 | 2.47E-06 | down |
| RNU6ATAC39P | -2.409212827 | 0.000652737 | 0.004311971 | down |
| RNU6-28P | -2.409101932 | 0.136816669 | 0.252082853 | down |
| RNU1-7P | -2.409032101 | 0.409180526 | | down |
| RP11-368M16.7 | -2.408828082 | 0.192092485 | 0.319930518 | down |
| MIR1268A | -2.408367043 | 0.113967789 | 0.221750421 | down |
| 1-Dec | -2.40832542 | 1.51E-06 | 2.91E-05 | down |
| FXYD4 | -2.408316204 | 0.001272088 | 0.007329998 | down |
| RP11-436K8.1 | -2.408125941 | 0.000465509 | 0.003287335 | down |
| CRISP2 | -2.407990922 | 0.010175455 | 0.037173074 | down |
| RP11-307A17.2 | -2.407889606 | 0.067239115 | 0.151775505 | down |
| NA | -2.407673606 | 0.058549698 | 0.137287686 | down |
| RP11-667M19.4 | -2.407515173 | 0.014276495 | 0.048112571 | down |
| NA | -2.407503822 | 0.364296756 | | down |
| TOPORSLP1 | -2.40689337 | 0.182208292 | 0.307806701 | down |
| RP11-610J23.1 | -2.40685744 | 0.183282462 | 0.309043567 | down |
| LINC01451 | -2.406580275 | 1.05E-07 | 2.96E-06 | down |
| PKD1L3 | -2.406504099 | 1.13E-05 | 0.000156436 | down |
| RP11-252M24.1 | -2.406499121 | 0.409675639 | | down |
| RP11-354P17.15 | -2.406397069 | 0.362526598 | | down |
| RP11-30K9.1 | -2.406302177 | 0.115137009 | | down |
| CAMKV | -2.406024394 | 0.000977658 | 0.005944699 | down |
| CTB-113D17.1 | -2.405609059 | 2.50E-06 | 4.46E-05 | down |
| SLC47A1P1 | -2.405525066 | 0.061632624 | 0.142602275 | down |
| RP11-59D5__B.2 | -2.404546968 | 0.018695298 | 0.059006361 | down |
| FUT6 | -2.404335898 | 3.95E-08 | 1.28E-06 | down |
| MTRNR2L3 | -2.40432957 | 0.00852526 | 0.03245064 | down |
| RP11-719K4.3 | -2.40427034 | 0.010892894 | 0.039165398 | down |
| ACTBP8 | -2.404169741 | 0.025523969 | 0.074400129 | down |
| RP11-897M7.2 | -2.403896285 | 0.410184776 | | down |
| AC012493.1 | -2.403598958 | 0.304971379 | | down |
| AC021021.2 | -2.403437075 | 0.365801251 | | down |
| MTND2P40 | -2.403057549 | 0.009978597 | 0.036609716 | down |
| NA | -2.402954896 | 0.002637961 | 0.013110821 | down |
| RP11-467L19.6 | -2.40289304 | 0.41038112 | | down |
| CEP164P1 | -2.402598275 | 0.019504042 | 0.06088098 | down |
| RP11-672L10.1 | -2.402021307 | 0.41055177 | | down |
| NA | -2.401765922 | 0.003624339 | 0.016835135 | down |
| NA | -2.401616864 | 0.002054076 | 0.010727042 | down |
| RP11-521C20.5 | -2.401414881 | 0.020823279 | 0.063953787 | down |
| NA | -2.401141721 | 0.009432122 | 0.035061215 | down |
| CDCA4P3 | -2.400906763 | 0.410703247 | | down |
| NA | -2.400685279 | 0.084715238 | 0.179554469 | down |
| TMEM114 | -2.4001248 | 0.314969922 | | down |

| | | | | |
|---|---|---|---|---|
| RP11-135M8__A.1 | -2.400096489 | 0.365283927 | | down |
| SLC22A10 | -2.400027995 | 0.016239034 | 0.053007988 | down |
| AKR1C7P | -2.399797195 | 0.009383681 | 0.034930405 | down |
| LINC01448 | -2.399504423 | 0.361762675 | | down |
| TEKT4 | -2.398140711 | 0.21752958 | | down |
| RNA5SP367 | -2.398054674 | 0.214783386 | 0.345798835 | down |
| AWAT2 | -2.397898731 | 0.071158899 | 0.158175346 | down |
| AATK-AS1 | -2.396431799 | 0.265839424 | | down |
| EPHA8 | -2.396424225 | 0.013139084 | 0.045083445 | down |
| RP11-363I22.3 | -2.395910086 | 0.221275657 | | down |
| NA | -2.395257787 | 0.079851413 | 0.171963002 | down |
| RP11-256L6.5 | -2.395257114 | 4.40E-06 | 7.13E-05 | down |
| RP11-231P20.2 | -2.39491962 | 1.11E-06 | 2.23E-05 | down |
| GUCY2D | -2.394472226 | 5.00E-06 | 7.93E-05 | down |
| RNU6-252P | -2.394371385 | 0.221167715 | 0.352788045 | down |
| RP11-788H18.1 | -2.394041377 | 0.367064612 | | down |
| NACAP5 | -2.393429327 | 0.412105146 | | down |
| RP11-115D7.3 | -2.393056005 | 0.12210355 | 0.232889732 | down |
| AC020951.1 | -2.392972273 | 0.00285621 | 0.013953166 | down |
| RBM22P5 | -2.392767481 | 0.038911855 | | down |
| TMEM89 | -2.392564546 | 1.02E-05 | 0.000143972 | down |
| OR8B7P | -2.392549834 | 0.04369403 | 0.110929815 | down |
| 5S_rRNA | -2.392284251 | 0.412460756 | | down |
| AIPL1 | -2.392166985 | 0.04658014 | 0.116074692 | down |
| KLK13 | -2.392149194 | 3.02E-06 | 5.22E-05 | down |
| SALRNA1 | -2.3920856 | 0.412499757 | | down |
| AMER2 | -2.391740884 | 0.001294224 | 0.007429054 | down |
| MIR4300HG | -2.391659948 | 0.04547975 | 0.114065749 | down |
| RN7SKP17 | -2.391334964 | 0.306461572 | | down |
| CTD-2351A16.1 | -2.39097509 | 0.412717822 | | down |
| CEACAM22P | -2.39078043 | 1.76E-06 | 3.30E-05 | down |
| RPS27AP18 | -2.3905872 | 0.264905502 | | down |
| RP11-431M3.1 | -2.390411858 | 0.055796013 | | down |
| RP11-178F10.3 | -2.389586354 | 0.412990616 | | down |
| AC002044.4 | -2.389449258 | 0.001280465 | 0.007362734 | down |
| CTD-2329K10.1 | -2.389030729 | 0.00958011 | 0.035455141 | down |
| OAT | -2.38896103 | 8.65E-10 | 4.47E-08 | down |
| PSMA2P3 | -2.388895656 | 0.41305912 | | down |
| RP11-17A1.3 | -2.388834489 | 0.00036273 | 0.002711586 | down |
| MTND3P25 | -2.388664851 | 0.000257152 | 0.002058792 | down |
| RP11-51L5.5 | -2.388030219 | 0.309804741 | | down |
| LINC01363 | -2.387589741 | 0.114854741 | | down |
| PIN1P1 | -2.387251168 | 0.413449565 | | down |
| NA | -2.387065971 | 3.36E-06 | 5.71E-05 | down |
| CTD-3020H12.4 | -2.386958367 | 0.000180868 | 0.001540477 | down |
| IL23R | -2.38689192 | 2.97E-09 | 1.36E-07 | down |
| FKBP4P1 | -2.386888799 | 0.371206819 | | down |
| RP11-366M4.6 | -2.386620793 | 0.095699746 | 0.195858526 | down |
| RP11-723C11.2 | -2.386118506 | 0.413672282 | | down |
| USP12PX | -2.386087255 | 0.219242225 | 0.350628841 | down |
| DNAJB3 | -2.385789627 | 0.119992889 | | down |

| | | | | |
|---|---|---|---|---|
| RP5-1057J7.7 | -2.385680744 | 2.20E-08 | 7.70E-07 | down |
| RP11-700H6.2 | -2.385542789 | 0.074356455 | | down |
| AC093074.1 | -2.385256847 | 0.41384176 | | down |
| APOA2 | -2.38487983 | 0.101824415 | | down |
| RFX6 | -2.384873947 | 0.000296442 | 0.002310323 | down |
| OR10A3 | -2.384716237 | 0.413948111 | | down |
| RP11-78H18.2 | -2.384364285 | 0.414017358 | | down |
| HTN3 | -2.384059654 | 0.4140773 | | down |
| RNY3P16 | -2.383928391 | 3.12E-07 | 7.48E-06 | down |
| DHRS11 | -2.383918815 | 7.22E-16 | 1.79E-13 | down |
| NA | -2.383319215 | 0.227134877 | 0.359311302 | down |
| RP11-24B13.2 | -2.383237873 | 0.047807485 | 0.118362428 | down |
| RP11-5P15.1 | -2.383236477 | 0.368469471 | | down |
| OR1X5P | -2.383162626 | 0.093748337 | 0.193114505 | down |
| TMC4 | -2.383094003 | 1.30E-10 | 8.65E-09 | down |
| RP5-928E24.2 | -2.383030264 | 0.13387113 | 0.248104635 | down |
| NA | -2.382872584 | 0.079779842 | | down |
| RP11-368J21.4 | -2.382816869 | 0.010017658 | 0.036726114 | down |
| AC016903.2 | -2.382639688 | 0.19607782 | 0.324588794 | down |
| CYP2D6 | -2.382575231 | 1.35E-11 | 1.16E-09 | down |
| MIR760 | -2.382521108 | 0.187589528 | | down |
| DISP2 | -2.382445294 | 1.38E-21 | 1.12E-18 | down |
| NA | -2.382321119 | 0.084872839 | | down |
| AC010761.6 | -2.382080469 | 0.191336215 | 0.319080584 | down |
| NA | -2.381976106 | 0.174508387 | | down |
| RP11-626G11.4 | -2.381664493 | 1.64E-05 | 0.000213087 | down |
| RP11-536N17.1 | -2.381587281 | 0.06731482 | 0.151934756 | down |
| LA16c-380H5.2 | -2.381573235 | 0.000194569 | 0.001633287 | down |
| FAM181A | -2.381548543 | 0.313688416 | | down |
| GABRQ | -2.380787843 | 0.000397885 | 0.002905701 | down |
| LINC00348 | -2.380136248 | 0.061297089 | 0.14206663 | down |
| MIR3976HG | -2.380125917 | 0.073120642 | 0.161425276 | down |
| NA | -2.38001995 | 0.009150433 | 0.034274479 | down |
| NA | -2.379770828 | 0.4148542 | | down |
| RP11-352D13.6 | -2.379651507 | 0.002928942 | 0.014232111 | down |
| C11orf16 | -2.379499527 | 0.001766462 | 0.009505925 | down |
| RP11-560I19.2 | -2.379308021 | 0.216463326 | | down |
| MYADML2 | -2.379085993 | 0.001381133 | 0.007814454 | down |
| RP11-164H5.1 | -2.378877201 | 0.06711742 | 0.15160332 | down |
| SERPINA12 | -2.378785293 | 0.071425561 | 0.158650713 | down |
| TRPM8 | -2.377869304 | 0.004880581 | 0.021288062 | down |
| RP11-738O11.13 | -2.377451469 | 0.011113599 | 0.039759106 | down |
| AC092684.1 | -2.377322875 | 0.415404194 | | down |
| NA | -2.37713061 | 0.003763738 | 0.017371607 | down |
| RP11-76E16.2 | -2.377089765 | 0.265875692 | | down |
| RP13-786C16.1 | -2.376853132 | 0.222887081 | | down |
| RP11-1223D19.6 | -2.376849116 | 0.018309863 | 0.058137974 | down |
| FAM32BP | -2.376643022 | 0.008942416 | 0.033663193 | down |
| SPRY4-IT1_2 | -2.376588573 | 0.100237384 | 0.202292736 | down |
| ST18 | -2.376075233 | 5.56E-07 | 1.24E-05 | down |
| RP1-96H9.5 | -2.375443196 | 0.006237866 | 0.025695006 | down |

| | | | | |
|---|---|---|---|---|
| DGCR6 | -2.375406603 | 0.001388584 | 0.007849223 | down |
| RP11-298E9.6 | -2.375304481 | 3.43E-06 | 5.82E-05 | down |
| RP11-383M4.6 | -2.375125599 | 0.060698014 | 0.140939108 | down |
| JTBP1 | -2.374566691 | 0.372685274 | | down |
| NA | -2.374334894 | 0.000167598 | 0.001445458 | down |
| AC083864.3 | -2.374127624 | 0.167416762 | 0.290542352 | down |
| RP11-757F18.3 | -2.37393039 | 0.125439535 | | down |
| RP11-669M2.1 | -2.373831382 | 0.314119778 | | down |
| LINC00237 | -2.373770237 | 0.00985293 | 0.036215001 | down |
| LINC00687 | -2.373537797 | 0.37251232 | | down |
| NA | -2.373157832 | 0.043293207 | 0.110247595 | down |
| MPPED1 | -2.37311816 | 0.000106677 | 0.000998134 | down |
| CCNYL2 | -2.373035274 | 0.009472606 | 0.035165573 | down |
| RP11-384K6.8 | -2.37298248 | 3.07E-07 | 7.41E-06 | down |
| RP11-66D17.3 | -2.372493921 | 0.416356858 | | down |
| RP1-37J18.2 | -2.372414723 | 0.067968818 | 0.153020244 | down |
| RP11-15B24.5 | -2.372024466 | 0.022196615 | 0.067042839 | down |
| UST-AS1 | -2.371959405 | 0.416462387 | | down |
| TPPP2 | -2.371541607 | 0.003929113 | 0.017977287 | down |
| FAM170A | -2.371447211 | 0.104991297 | | down |
| MIR122 | -2.371325587 | 0.010596859 | 0.038358077 | down |
| AP000477.2 | -2.371279227 | 0.416402808 | | down |
| RP11-266E6.3 | -2.371182306 | 0.081394926 | 0.174392032 | down |
| DNAH9 | -2.371047047 | 7.63E-06 | 0.000112875 | down |
| CNGA2 | -2.370852814 | 0.313597248 | | down |
| ABCB11 | -2.370506163 | 1.16E-06 | 2.31E-05 | down |
| RP11-265O12.1 | -2.370450988 | 0.228874362 | | down |
| LINC00993 | -2.370420488 | 0.147174376 | 0.265552037 | down |
| FAM166A | -2.369313988 | 0.000101449 | 0.000956963 | down |
| NA | -2.369311004 | 0.125084142 | | down |
| SNORA32 | -2.369237793 | 0.319681404 | 0.458450868 | down |
| C1orf141 | -2.369016366 | 0.082442782 | 0.176117104 | down |
| RP11-731N10.1 | -2.368640849 | 0.070729536 | 0.157512281 | down |
| LINC01553 | -2.368511528 | 0.268677339 | | down |
| NA | -2.368423081 | 0.031183861 | 0.086293443 | down |
| ASB10 | -2.367975699 | 0.084412242 | 0.179025935 | down |
| KRT8P40 | -2.367951663 | 0.089861417 | 0.18733381 | down |
| NA | -2.36778171 | 0.090042492 | 0.187573762 | down |
| CASC11 | -2.367661256 | 0.000356638 | 0.002677784 | down |
| HCN4 | -2.367038421 | 0.005173316 | 0.022267492 | down |
| AC062020.1 | -2.366837753 | 0.264168241 | | down |
| LINC01100 | -2.366527307 | 0.417535735 | | down |
| RP11-646E18.4 | -2.366407742 | 0.191580154 | 0.319336578 | down |
| RP11-202A13.1 | -2.366124867 | 0.000301463 | 0.002337934 | down |
| NA | -2.366016452 | 0.26479247 | | down |
| RP5-971N18.3 | -2.365732961 | 0.051716658 | 0.125304458 | down |
| AC020743.2 | -2.365544267 | 0.008035686 | 0.031063323 | down |
| LINC01198 | -2.365301644 | 0.000762108 | 0.004885618 | down |
| PRKG1-AS1 | -2.365031489 | 0.004542586 | 0.020153197 | down |
| UBE2V1P2 | -2.365013568 | 0.002738544 | 0.013512369 | down |
| RP11-363L24.3 | -2.364841582 | 0.157835383 | | down |

| | | | | |
|---|---|---|---|---|
| USP2-AS1 | -2.3648192 | 7.43E-12 | 6.73E-10 | down |
| AC007682.1 | -2.36469411 | 0.227517109 | 0.359708412 | down |
| MDGA2 | -2.364575363 | 0.0162474 | 0.053018017 | down |
| RFX4 | -2.364126134 | 0.02658189 | 0.076761468 | down |
| SDCBPP2 | -2.363804548 | 0.058122881 | 0.13660673 | down |
| RP11-3K16.2 | -2.363614848 | 0.011240976 | 0.040100201 | down |
| CYP2J2 | -2.362872296 | 7.53E-09 | 3.08E-07 | down |
| OR5V1 | -2.362752762 | 0.418282517 | | down |
| LINC00974 | -2.362005775 | 3.38E-07 | 8.02E-06 | down |
| NA | -2.361999567 | 0.418363422 | | down |
| MED28P8 | -2.361794309 | 0.0040707 | 0.018490593 | down |
| LINC00410 | -2.36170772 | 0.418356283 | | down |
| NA | -2.361665966 | 0.003582986 | 0.016681734 | down |
| DPRX | -2.361434751 | 0.275942378 | 0.412252818 | down |
| RP11-380G5.2 | -2.361124651 | 0.00092043 | 0.005679485 | down |
| RP11-285E18.2 | -2.361099522 | 0.418609852 | | down |
| RP11-569A2.2 | -2.361050234 | 0.005162207 | 0.022229233 | down |
| RP11-1348G14.7 | -2.360901571 | 0.229234382 | 0.361661386 | down |
| RBFOX1 | -2.360662843 | 1.81E-07 | 4.75E-06 | down |
| MIR3648-1 | -2.36058138 | 8.98E-06 | 0.000129499 | down |
| RN7SKP180 | -2.360328368 | 0.266601476 | | down |
| RP11-20B24.7 | -2.360323166 | 2.86E-05 | 0.000336822 | down |
| RP4-555L14.4 | -2.360246739 | 0.074015416 | 0.162719096 | down |
| NA | -2.360066592 | 0.370230029 | | down |
| RP4-550H1.5 | -2.359911281 | 0.118384532 | 0.227865005 | down |
| NA | -2.359725485 | 0.022462276 | 0.067620903 | down |
| KRTAP12-3 | -2.359490561 | 0.372036799 | | down |
| RP11-439E19.7 | -2.359345498 | 0.000264478 | 0.002105493 | down |
| KRTAP9-12P | -2.359224236 | 0.200587154 | 0.329673986 | down |
| RP11-532L16.1 | -2.35869652 | 0.103024615 | 0.20624144 | down |
| NA | -2.358612861 | 0.025994711 | 0.075454966 | down |
| RP4-760C5.5 | -2.358490042 | 0.368505942 | 0.507020275 | down |
| NA | -2.358472926 | 0.223494107 | | down |
| RP1-135L22.1 | -2.358455376 | 0.419065359 | | down |
| GLTPD2 | -2.358346703 | 1.52E-10 | 9.78E-09 | down |
| NA | -2.358077728 | 0.318636226 | | down |
| AC007795.1 | -2.358020335 | 0.419086524 | | down |
| CTD-204904.1 | -2.357961139 | 0.0027909 | 0.013703186 | down |
| NA | -2.357845064 | 0.270112967 | | down |
| CTC-436P18.4 | -2.35776174 | 0.189377314 | | down |
| RRM2P2 | -2.356984441 | 0.056023871 | 0.132910707 | down |
| RP11-351I21.6 | -2.356336013 | 0.192848328 | 0.320833666 | down |
| KRT18P49 | -2.356223627 | 0.419576136 | | down |
| SMCR5 | -2.356129196 | 0.033058014 | 0.090085712 | down |
| RP5-1031J8.1 | -2.356071893 | 0.018542579 | 0.058702419 | down |
| LILRP2 | -2.35569445 | 0.002634596 | 0.013098428 | down |
| HEPACAM | -2.355442762 | 0.000159882 | 0.00138869 | down |
| SRGAP3-AS4 | -2.355409149 | 0.025632849 | | down |
| RP11-584P21.2 | -2.355364375 | 2.42E-05 | 0.000292545 | down |
| HSPD1P7 | -2.354789733 | 0.103641073 | 0.207158034 | down |
| RP11-344J7.4 | -2.354711241 | 0.319149527 | | down |

| | | | | |
|---|---|---|---|---|
| OR5C1 | -2.354531229 | 0.419911834 | | down |
| IGHVII-22-1 | -2.35433321 | 0.109006335 | 0.215172007 | down |
| RP11-463I20.2 | -2.354152741 | 0.376051273 | | down |
| RP11-94B19.3 | -2.353836804 | 0.130696233 | 0.244239449 | down |
| NA | -2.353700491 | 0.012649839 | 0.043901093 | down |
| PTP4A1 | -2.352976054 | 6.41E-05 | 0.00065351 | down |
| RP11-217C7.1 | -2.352972006 | 0.268484097 | | down |
| RP11-407N17.2 | -2.352394934 | 0.316974836 | | down |
| KRT8P21 | -2.352129882 | 0.232125491 | 0.364787135 | down |
| CA4 | -2.35209736 | 9.01E-06 | 0.000129835 | down |
| CTC-207P7.1 | -2.35193737 | 0.232990201 | 0.365704906 | down |
| CTD-2047H16.3 | -2.351894508 | 7.93E-05 | 0.000779709 | down |
| AC007389.2 | -2.351892596 | 0.231108334 | 0.363644014 | down |
| FAM47B | -2.351747548 | 0.420464337 | | down |
| AC016738.3 | -2.351449002 | 0.116604296 | 0.22539146 | down |
| NA | -2.351262495 | 0.319110726 | 0.45784922 | down |
| RP11-403I13.7 | -2.350316603 | 0.420748515 | | down |
| HSD17B2 | -2.349900979 | 1.84E-10 | 1.15E-08 | down |
| AC011899.10 | -2.349868417 | 0.225472309 | | down |
| NA | -2.349771309 | 0.420856837 | | down |
| RP11-100L22.3 | -2.349561356 | 0.056479296 | 0.133727163 | down |
| MFRP | -2.34934313 | 0.089369036 | | down |
| C8orf74 | -2.349071092 | 0.007893267 | 0.030658734 | down |
| NA | -2.348910252 | 0.019347623 | 0.060568927 | down |
| RP11-700P18.1 | -2.348782001 | 0.022767081 | 0.068280891 | down |
| RNU6-353P | -2.348182019 | 0.001371604 | 0.007775182 | down |
| RP11-216P16.5 | -2.348126721 | 0.421183632 | | down |
| Y_RNA | -2.348005429 | 0.000385429 | 0.002838324 | down |
| RP11-258B16.1 | -2.347399187 | 0.420911582 | | down |
| RP11-5024.1 | -2.347186991 | 0.002654466 | 0.013171061 | down |
| RPSAP49 | -2.347148538 | 0.06731978 | 0.151934756 | down |
| RP11-344A7.1 | -2.346971394 | 0.233461683 | 0.366119783 | down |
| RP1-315G1.1 | -2.346672717 | 0.189827727 | | down |
| RNU6-86P | -2.346216173 | 0.125208155 | 0.236871344 | down |
| RP11-192B18.1 | -2.346187053 | 0.421569252 | | down |
| CFHR5 | -2.346062988 | 0.421593925 | | down |
| ANKRD62 | -2.345909684 | 0.111741054 | 0.21886918 | down |
| LHX1 | -2.345873909 | 0.274032037 | 0.410358298 | down |
| KRT18P18 | -2.345526328 | 0.100338365 | | down |
| CRIP1P2 | -2.345314459 | 0.271236071 | | down |
| PSKH2 | -2.345155 | 0.421705688 | | down |
| LINC01482 | -2.345043094 | 0.010992964 | 0.039454378 | down |
| HOXC8 | -2.345013205 | 4.70E-05 | 0.000507264 | down |
| OR6K5P | -2.344804952 | 0.375497103 | | down |
| RP11-544A12.5 | -2.344285825 | 0.057428498 | | down |
| AL022237.3 | -2.343771464 | 0.154111411 | 0.273825964 | down |
| RP11-298A8.2 | -2.34351095 | 0.076197546 | 0.166198556 | down |
| CARM1P1 | -2.343109902 | 0.043136637 | 0.109942071 | down |
| RP11-373N24.2 | -2.342756196 | 0.377076835 | | down |
| RP11-5023.1 | -2.342524331 | 0.273404238 | | down |
| AVPR1B | -2.341801504 | 0.002508732 | 0.012614621 | down |

| | | | | |
|---|---|---|---|---|
| AC067959.1 | -2.341753299 | 0.000750418 | 0.004825679 | down |
| RNU4-74P | -2.341508465 | 0.3795118 | | down |
| RP4-564F22.7 | -2.340912405 | 0.012525498 | 0.04352991 | down |
| RNU6-1077P | -2.340730788 | 0.061365782 | 0.142181965 | down |
| AP000345.1 | -2.34057863 | 0.002933708 | 0.014250658 | down |
| CTD-2378E12.1 | -2.340562083 | 7.81E-07 | 1.65E-05 | down |
| RP11-23D24.2 | -2.340414461 | 0.017565944 | 0.056310714 | down |
| NUP210P1 | -2.339801442 | 0.422840218 | | down |
| Y_RNA | -2.339799919 | 0.024283419 | 0.0717428 | down |
| TRIM50 | -2.339765107 | 4.12E-05 | 0.000454739 | down |
| CTC-448D22.1 | -2.339099827 | 0.09992488 | 0.20178405 | down |
| CTB-111H14.1 | -2.339045683 | 0.00314727 | 0.015023289 | down |
| AC005757.6 | -2.338947709 | 0.192172215 | | down |
| RP11-160H12.2 | -2.338847469 | 0.242508612 | 0.376396463 | down |
| GLRA3 | -2.338761492 | 0.000289302 | 0.002265847 | down |
| RP11-579024.1 | -2.338709946 | 0.133578211 | 0.247747842 | down |
| RP11-407G23.1 | -2.338559632 | 0.05730267 | | down |
| LINC01162 | -2.338493251 | 0.423100871 | | down |
| RP11-680F8.3 | -2.33844021 | 3.24E-07 | 7.75E-06 | down |
| AC073055.2 | -2.338437088 | 0.123735766 | 0.2350266 | down |
| FOXB2 | -2.338238173 | 0.423151705 | | down |
| MUC7 | -2.338155676 | 0.423168146 | | down |
| RP1-140K8.5 | -2.338056354 | 0.01041152 | 0.037837632 | down |
| FDPSP5 | -2.338052636 | 0.03874649 | 0.101428489 | down |
| RBM22P4 | -2.337549594 | 0.42321984 | | down |
| RP11-297A16.2 | -2.33754782 | 0.002034844 | 0.010648814 | down |
| NA | -2.337435441 | 0.325014893 | | down |
| NA | -2.337260561 | 0.051609033 | 0.125171621 | down |
| RP11-354K1.1 | -2.33694053 | 0.062687408 | 0.144343025 | down |
| PKD1L2 | -2.336927332 | 3.66E-06 | 6.12E-05 | down |
| LINC01496 | -2.336796806 | 0.4230713 | | down |
| USP2 | -2.336707267 | 1.04E-17 | 3.62E-15 | down |
| RP11-126F18.2 | -2.336700891 | 0.117607118 | 0.226848244 | down |
| AC018359.1 | -2.33640343 | 0.000129874 | 0.001174575 | down |
| CLCA2 | -2.336314791 | 2.34E-06 | 4.21E-05 | down |
| PMCHL1 | -2.336048557 | 0.324580863 | | down |
| CTC-384G19.1 | -2.33588658 | 0.322801974 | | down |
| CLUHP5 | -2.335763414 | 0.423510082 | | down |
| KRT33B | -2.33572586 | 0.423652573 | | down |
| RP11-113K21.6 | -2.33568197 | 0.001668241 | 0.009078182 | down |
| RP11-425M5.8 | -2.335417209 | 0.423714131 | | down |
| CYCSP4 | -2.335329851 | 0.423731555 | | down |
| RP11-714M23.2 | -2.335007496 | 0.138197203 | 0.254053526 | down |
| RP11-521C20.1 | -2.334795229 | 0.423838196 | | down |
| RP11-728L1.1 | -2.3345091 | 0.423895277 | | down |
| NA | -2.33445295 | 0.023710345 | | down |
| CLVS1 | -2.334304239 | 0.000252466 | 0.002026477 | down |
| AC002076.10 | -2.334105288 | 0.161552583 | | down |
| CTB-73N10.1 | -2.333891157 | 0.020555242 | 0.063365657 | down |
| YBX1P6 | -2.33379112 | 0.000270576 | 0.00214774 | down |
| RP11-168016.1 | -2.333660522 | 0.000173829 | 0.001488749 | down |

| | | | | |
|---|---|---|---|---|
| ZSCAN5D | -2.333547399 | 0.008166733 | 0.031424349 | down |
| LHX4 | -2.333531636 | 2.36E-08 | 8.16E-07 | down |
| RP11-775C24.4 | -2.333519117 | 0.045593792 | 0.114275456 | down |
| RP11-364P2.2 | -2.333168288 | 0.424162819 | | down |
| NA | -2.333074145 | 0.205630251 | 0.335521384 | down |
| RP11-526D8.7 | -2.332554657 | 0.424285294 | | down |
| RP1-253P7.4 | -2.332485345 | 6.92E-05 | 0.000694854 | down |
| OLFM5P | -2.332436646 | 0.001438113 | 0.008066968 | down |
| AC090286.4 | -2.332301086 | 0.424335911 | | down |
| NMRAL1P1 | -2.331967153 | 9.55E-10 | 4.86E-08 | down |
| LINC00570 | -2.331708598 | 1.28E-07 | 3.52E-06 | down |
| PLPPR1 | -2.331355275 | 0.001287128 | 0.007392558 | down |
| AC025750.7 | -2.330648964 | 0.324862936 | 0.463322815 | down |
| RP11-83B20.3 | -2.33050649 | 0.013541238 | 0.046183783 | down |
| TMEM63C | -2.33007634 | 2.71E-12 | 2.69E-10 | down |
| LINC01508 | -2.329903194 | 0.222545917 | | down |
| AC012531.25 | -2.329659892 | 0.13377768 | | down |
| MYH1 | -2.329594807 | 0.042374213 | 0.108495897 | down |
| NA | -2.329410901 | 0.190449776 | | down |
| RP11-15I11.3 | -2.328884003 | 0.002790047 | 0.013701238 | down |
| NA | -2.328743167 | 6.72E-05 | 0.000679354 | down |
| NA | -2.328656322 | 0.277278121 | | down |
| C2orf71 | -2.327962214 | 0.020332813 | 0.062867041 | down |
| NA | -2.327883477 | 0.001926859 | 0.010178776 | down |
| RP11-188C12.3 | -2.327778113 | 0.275419994 | | down |
| RP11-1084I9.1 | -2.327598309 | 0.048201235 | 0.119140923 | down |
| OR10H1 | -2.32759454 | 0.083706343 | 0.177945402 | down |
| RP11-288K12.1 | -2.327479095 | 0.035242141 | 0.094496852 | down |
| BTBD17 | -2.327475526 | 8.63E-05 | 0.000834886 | down |
| RP11-411B10.7 | -2.327290772 | 0.002636824 | 0.013107336 | down |
| RP11-175P13.3 | -2.327121815 | 0.000218358 | 0.001796866 | down |
| LCN1 | -2.327102729 | 0.01947731 | 0.06082484 | down |
| RPS7P15 | -2.326920676 | 0.033482932 | 0.090938457 | down |
| LINC01587 | -2.326909998 | 0.059811988 | 0.139366478 | down |
| RP11-171L9.1 | -2.326727817 | 0.425449306 | | down |
| SLC26A3 | -2.326449765 | 1.01E-06 | 2.05E-05 | down |
| MTCO2P27 | -2.326294579 | 0.157462789 | | down |
| KRT8P31 | -2.326264802 | 0.2341863 | | down |
| OR2T34 | -2.326211161 | 0.425552606 | | down |
| NA | -2.326080467 | 0.425578739 | | down |
| RP11-556H2.1 | -2.325969703 | 1.12E-05 | 0.000154391 | down |
| RP1-34B20.19 | -2.32592892 | 0.233754832 | | down |
| NA | -2.325257062 | 0.000101405 | 0.000956963 | down |
| RP11-114M5.3 | -2.324879335 | 0.061476411 | 0.142372412 | down |
| BPIFC | -2.3248764 | 0.019690519 | 0.061322894 | down |
| HMGB1P9 | -2.324804779 | 0.425833873 | | down |
| NA | -2.32473426 | 0.03412001 | 0.092341312 | down |
| MARK2P6 | -2.324233079 | 0.277137605 | 0.413299028 | down |
| PTCHD3 | -2.324228819 | 0.036655059 | 0.097332533 | down |
| RP11-365O16.5 | -2.324050612 | 0.240932622 | 0.374725874 | down |
| HRCT1 | -2.323147302 | 4.59E-09 | 1.99E-07 | down |

| | | | | |
|---|---|---|---|---|
| RP11-760D2.5 | -2.322731315 | 0.052709187 | 0.126972003 | down |
| SNORD59 | -2.322610501 | 0.199521653 | 0.328533846 | down |
| NA | -2.322580787 | 0.002586145 | 0.012919547 | down |
| RP11-216P16.4 | -2.322502688 | 0.376003416 | 0.514221359 | down |
| RP11-553P9.3 | -2.322134292 | 0.061212124 | 0.141913501 | down |
| CIR1P3 | -2.321908285 | 0.20076756 | 0.329840047 | down |
| RP11-2L8.2 | -2.32182873 | 0.120440542 | 0.230757965 | down |
| Y_RNA | -2.32167532 | 0.084747788 | 0.179572786 | down |
| RP11-2P2.1 | -2.321584907 | 0.239571968 | 0.373189206 | down |
| CTD-2189E23.2 | -2.321492561 | 0.199445462 | | down |
| ANKRD31 | -2.321433175 | 0.00043282 | 0.003098322 | down |
| GSTM3P2 | -2.321370972 | 0.143183596 | 0.260336962 | down |
| RHOXF1P2 | -2.321258124 | 0.105763368 | | down |
| RP11-93G5.1 | -2.320869579 | 0.012893617 | 0.044495307 | down |
| BPIFB1 | -2.320380354 | 0.007036491 | 0.028104803 | down |
| OTX2-AS1 | -2.320166193 | 0.131935066 | | down |
| DIS3L2P1 | -2.319821887 | 0.38527393 | | down |
| MARK2P10 | -2.319651501 | 0.142724285 | 0.259753357 | down |
| MTND5P21 | -2.319488536 | 0.379432535 | | down |
| RPSAP43 | -2.319107672 | 0.192065089 | 0.319920361 | down |
| SPATA8 | -2.31906944 | 0.141087637 | 0.257739286 | down |
| RFKP2 | -2.318482127 | 0.427099696 | | down |
| FAM90A24P | -2.318482127 | 0.427099696 | | down |
| RP11-12505.2 | -2.318482127 | 0.427099696 | | down |
| RP11-368P15.1 | -2.318482127 | 0.427099696 | | down |
| AC108868.5 | -2.318207831 | 0.38227505 | | down |
| AC017104.2 | -2.318160602 | 0.016881895 | 0.054537975 | down |
| RP11-274H24.1 | -2.317851529 | 0.081602461 | 0.174694623 | down |
| AC006116.15 | -2.317834076 | 0.158873519 | | down |
| NA | -2.31782298 | 0.093565826 | 0.192817867 | down |
| RP11-104D21.2 | -2.317658419 | 0.325768433 | | down |
| NA | -2.31763786 | 0.326667773 | | down |
| AC007292.6 | -2.317468201 | 6.53E-09 | 2.70E-07 | down |
| OR2H1 | -2.316716459 | 0.177028824 | 0.301941316 | down |
| STARD10 | -2.316713886 | 8.64E-10 | 4.47E-08 | down |
| AC114814.3 | -2.31662792 | 0.27808314 | | down |
| RP11-747I9.1 | -2.316012909 | 0.427594637 | | down |
| RP5-1042K10.10 | -2.315701652 | 0.02276288 | 0.068280891 | down |
| AC092675.3 | -2.315107512 | 0.05314247 | 0.127718772 | down |
| RNU7-14P | -2.314279338 | 0.109395232 | 0.215661182 | down |
| AC007040.11 | -2.314030204 | 8.91E-05 | 0.000858998 | down |
| NA | -2.313482762 | 0.247670149 | 0.382040456 | down |
| NA | -2.313311765 | 0.000888884 | 0.005508498 | down |
| ACOT11 | -2.31329779 | 7.58E-15 | 1.46E-12 | down |
| GRAMD4P4 | -2.313218012 | 0.231688182 | | down |
| RP11-492A10.1 | -2.312151543 | 0.428369293 | | down |
| NA | -2.311793979 | 0.000896015 | 0.005545915 | down |
| RP11-338L22.3 | -2.311689018 | 0.428462138 | | down |
| SMG1P4 | -2.311416645 | 0.000341344 | 0.002587121 | down |
| RP11-182L7.1 | -2.311231313 | 0.328049686 | | down |
| NA | -2.311061263 | 0.234786523 | | down |

| | | | | |
|---|---|---|---|---|
| AGGF1P3 | -2.310932512 | 0.075830597 | 0.165589588 | down |
| RP11-20B24.3 | -2.310900661 | 0.428483623 | | down |
| NA | -2.310859942 | 0.32707469 | | down |
| CCER1 | -2.310801156 | 0.428379723 | | down |
| RP11-711C17.2 | -2.310786817 | 0.162943034 | 0.285017885 | down |
| RP11-589F4.2 | -2.310487711 | 0.428703337 | | down |
| RP11-566K19.5 | -2.310189403 | 0.138924148 | | down |
| RPL23AP86 | -2.310170063 | 0.232589428 | | down |
| RP11-319E16.1 | -2.310137412 | 0.329843894 | | down |
| AC007362.3 | -2.310023794 | 0.331664862 | | down |
| RP3-434P1.6 | -2.309814768 | 0.007573672 | 0.029717045 | down |
| NA | -2.309783153 | 0.073331423 | | down |
| RNU6-450P | -2.308992768 | 0.02507109 | 0.073472085 | down |
| LINC00511 | -2.308814801 | 1.79E-14 | 3.16E-12 | down |
| TINCR | -2.308667889 | 3.77E-12 | 3.66E-10 | down |
| PTPRJ-AS1 | -2.308393791 | 0.000853289 | 0.005343105 | down |
| BRINP3 | -2.308272565 | 1.22E-08 | 4.76E-07 | down |
| MMP20 | -2.308259026 | 0.028370334 | 0.080625497 | down |
| RP11-90E9.1 | -2.308193156 | 0.165000278 | 0.287678061 | down |
| LINC01203 | -2.308017874 | 0.000753736 | 0.004842868 | down |
| RPL5P35 | -2.307959783 | 0.109052787 | | down |
| NA | -2.307932602 | 0.331442061 | | down |
| RP11-304C12.5 | -2.307904781 | 5.35E-06 | 8.38E-05 | down |
| ANKS1B | -2.307649222 | 5.59E-07 | 1.24E-05 | down |
| RP11-113I24.1 | -2.307616466 | 0.044729194 | 0.112719671 | down |
| RP11-766N7.3 | -2.306998802 | 0.01224701 | 0.042814657 | down |
| RPS27P23 | -2.306921348 | 0.174386687 | 0.29885766 | down |
| Y_RNA | -2.306898948 | 0.128796231 | 0.24174136 | down |
| KCTD16 | -2.306828374 | 4.23E-05 | 0.000464975 | down |
| POU2F3 | -2.306569186 | 2.47E-08 | 8.48E-07 | down |
| RP11-543A18.1 | -2.306446377 | 0.429515336 | | down |
| C9orf135 | -2.306260625 | 0.062146503 | 0.143481868 | down |
| NA | -2.306251131 | 0.036423415 | 0.096888155 | down |
| RNU4-1 | -2.30586992 | 0.004793355 | 0.021020518 | down |
| NA | -2.305218572 | 0.232852233 | | down |
| ST13P19 | -2.304962055 | 0.001936407 | 0.010211196 | down |
| C3orf56 | -2.304864627 | 0.429833389 | | down |
| LRP1B | -2.304863977 | 0.000790076 | 0.005025461 | down |
| PDE4C | -2.304828299 | 0.001280034 | 0.007361668 | down |
| AC068492.1 | -2.304351583 | 0.23439726 | | down |
| AC015922.6 | -2.304253924 | 0.132208866 | 0.246192959 | down |
| AP001464.4 | -2.304250798 | 0.42963761 | | down |
| LINC01333 | -2.304247175 | 0.237141807 | | down |
| RP11-14J7.6 | -2.30404506 | 0.378742442 | 0.516848562 | down |
| RSU1P2 | -2.304024452 | 0.108979091 | 0.215136856 | down |
| SLC22A7 | -2.303992451 | 0.001615095 | 0.008854729 | down |
| NA | -2.303730113 | 0.003019983 | 0.014582507 | down |
| NKX6-2 | -2.303458376 | 0.121124985 | 0.231665616 | down |
| RP5-872K7.7 | -2.303397245 | 0.17020545 | | down |
| LINC00112 | -2.303390393 | 0.23504584 | | down |
| PYY | -2.303247416 | 4.50E-06 | 7.28E-05 | down |

| | | | | |
|---|---|---|---|---|
| RP11-447H19.1 | -2.303223979 | 0.43016343 | | down |
| RP11-502F1.1 | -2.302411167 | 0.052546146 | 0.126701157 | down |
| WBP2P1 | -2.302167719 | 0.430375989 | | down |
| KRT18P47 | -2.301911405 | 0.203309201 | 0.332764309 | down |
| NA | -2.301706475 | 0.224905857 | 0.357025867 | down |
| NPY6R | -2.301559499 | 6.35E-06 | 9.71E-05 | down |
| RNU6-1093P | -2.301380069 | 0.327878574 | | down |
| PRDM14 | -2.301375432 | 0.331376881 | | down |
| RP11-231E6.3 | -2.300779081 | 0.095240016 | 0.195210195 | down |
| NA | -2.300520105 | 0.009815358 | | down |
| NA | -2.300481553 | 0.213265252 | 0.344147664 | down |
| IL31 | -2.300359783 | 0.430669474 | | down |
| AC091814.3 | -2.300226599 | 0.005345284 | 0.022831063 | down |
| ACTL8 | -2.300059871 | 0.000669647 | 0.004399489 | down |
| GGT8P | -2.299889464 | 0.034815396 | 0.093694534 | down |
| LA16c-60D12.2 | -2.299650608 | 0.430812264 | | down |
| NA | -2.299628662 | 0.430749588 | | down |
| HIST1H2BPS2 | -2.299539536 | 0.11460017 | | down |
| RP11-38023.4 | -2.299448807 | 0.103032307 | 0.206243095 | down |
| TMEM238 | -2.299085539 | 2.02E-08 | 7.16E-07 | down |
| RP11-628018.1 | -2.29841989 | 0.431130685 | | down |
| CTD-3076017.2 | -2.29803082 | 0.056591275 | 0.133913105 | down |
| FUNDC2P2 | -2.298011917 | 0.042730421 | | down |
| RP5-991023.1 | -2.297855285 | 0.38341163 | | down |
| IFIT6P | -2.297515875 | 0.431312839 | | down |
| RP11-697E22.3 | -2.297511901 | 0.113222195 | 0.220671271 | down |
| RP11-867G2.4 | -2.297473645 | 0.072847793 | | down |
| NLRP13 | -2.297465757 | 0.153490905 | 0.273173768 | down |
| SCRT1 | -2.297440491 | 0.001276704 | 0.007346732 | down |
| KCNV1 | -2.297395937 | 0.000113645 | 0.001054459 | down |
| MIR4479 | -2.297333193 | 0.004827646 | 0.021127719 | down |
| RP11-344N17.8 | -2.296677607 | 0.384034467 | | down |
| SPDYE10P | -2.29664088 | 0.200564392 | | down |
| OR52W1 | -2.296586968 | 0.38904691 | | down |
| FAM95B1 | -2.296383037 | 0.00356132 | | down |
| GEMIN2P2 | -2.29632664 | 0.431481902 | | down |
| BCL2L12P1 | -2.296131707 | 0.141332113 | | down |
| NA | -2.296118517 | 0.01403736 | 0.047482373 | down |
| LGALS8-AS1 | -2.296097482 | 0.003758509 | 0.017356383 | down |
| ONECUT1 | -2.295891195 | 0.033380789 | 0.090708447 | down |
| POM121L1P | -2.295876803 | 0.38832212 | | down |
| LINC01607 | -2.29575926 | 5.70E-05 | 0.000594159 | down |
| RP4-758J24.6 | -2.295030428 | 7.19E-07 | 1.54E-05 | down |
| NPC1L1 | -2.294727397 | 5.94E-06 | 9.17E-05 | down |
| LINC00337 | -2.294198282 | 7.92E-10 | 4.18E-08 | down |
| SEZ6L | -2.293576293 | 2.90E-05 | 0.000341054 | down |
| SLC22A11 | -2.293520532 | 0.009958751 | 0.036541369 | down |
| RP1-213J1P__B.1 | -2.293201281 | 0.432182822 | | down |
| ANKRD30BP2 | -2.292953579 | 0.031780846 | 0.087494157 | down |
| KCNB2 | -2.292946835 | 0.001839373 | 0.009819926 | down |
| RP11-162A23.5 | -2.292915494 | 0.000533141 | 0.003664539 | down |

| | | | | |
|---|---|---|---|---|
| CTD-2235C13.3 | -2.292603126 | 4.68E-05 | 0.000505897 | down |
| RP11-1060G2.1 | -2.29208185 | 0.029112445 | 0.082132722 | down |
| RP11-484D2.5 | -2.292048467 | 0.28399899 | | down |
| C9orf24 | -2.292029946 | 2.98E-15 | 6.09E-13 | down |
| KCNJ4 | -2.291398183 | 0.001441288 | 0.008078741 | down |
| RP11-308D16.1 | -2.291323879 | 0.328779835 | | down |
| SNTG1 | -2.291128095 | 0.01021574 | 0.037283949 | down |
| RP5-951N9.2 | -2.291096517 | 0.113464159 | 0.221013823 | down |
| RP11-497G19.2 | -2.290973499 | 0.103853687 | | down |
| NA | -2.290681392 | 0.432691394 | | down |
| TRY2P | -2.290657715 | 0.334129322 | | down |
| LINC01378 | -2.290114603 | 0.334595354 | | down |
| MRPL12 | -2.289756376 | 2.00E-07 | 5.18E-06 | down |
| SSX8 | -2.289704172 | 0.282093391 | | down |
| PLA2G2F | -2.289471433 | 0.028981841 | 0.081850951 | down |
| RP11-344N17.15 | -2.288894274 | 0.43298138 | | down |
| RP11-171G2.1 | -2.288892198 | 0.282826073 | | down |
| SSTR5-AS1 | -2.288867031 | 0.000605859 | 0.00406667 | down |
| NA | -2.288789016 | 0.149826265 | 0.268747529 | down |
| RP11-473P24.2 | -2.288426185 | 0.05992868 | 0.139573432 | down |
| CTC-239J10.1 | -2.288404711 | 0.19755968 | | down |
| AP000593.5 | -2.287450762 | 0.240826767 | | down |
| SGK2 | -2.287208257 | 1.03E-11 | 8.97E-10 | down |
| FO538757.2 | -2.287160709 | 0.012044282 | 0.042307775 | down |
| ARL2BPP1 | -2.287008351 | 0.333326008 | 0.471600969 | down |
| FABP1 | -2.286987415 | 2.97E-08 | 9.95E-07 | down |
| RP11-119J18.1 | -2.286937039 | 2.75E-05 | 0.000325922 | down |
| PHGR1 | -2.286735833 | 1.06E-08 | 4.18E-07 | down |
| PDCL3P3 | -2.28668525 | 0.331202478 | | down |
| RP11-347D21.1 | -2.286433664 | 0.333650067 | | down |
| RPL18P10 | -2.286372118 | 0.038666095 | 0.101271394 | down |
| RN7SL610P | -2.286216433 | 0.036031618 | 0.096105639 | down |
| CTA-714B7.4 | -2.286136214 | 0.125820907 | 0.237746121 | down |
| RP11-371F15.3 | -2.286063953 | 0.092684524 | 0.191448185 | down |
| FOXG1 | -2.285585452 | 0.005469875 | 0.023231117 | down |
| RP11-157I4.4 | -2.285520162 | 0.283946269 | | down |
| RP11-685B24.1 | -2.28470237 | 0.433899478 | | down |
| RN7SL285P | -2.284415437 | 0.134830765 | | down |
| RP11-395C17.1 | -2.284152135 | 0.389241023 | | down |
| IGFL4 | -2.284017871 | 0.001888942 | 0.01002352 | down |
| OR6D1P | -2.283828637 | 0.068190715 | 0.153381886 | down |
| RP11-568J23.2 | -2.283709107 | 0.017928876 | 0.057230679 | down |
| TSSK1B | -2.283517398 | 0.242796823 | | down |
| ABCC5-AS1 | -2.283413377 | 0.069036729 | 0.154728795 | down |
| AC023669.1 | -2.282798037 | 0.288911334 | 0.42615592 | down |
| RP11-510J16.5 | -2.282231167 | 9.99E-05 | 0.000943999 | down |
| NA | -2.282142292 | 0.010871681 | 0.039103171 | down |
| TRIML1 | -2.282117918 | 0.392266303 | | down |
| RP11-247A12.1 | -2.282117818 | 0.286825121 | | down |
| KRT17P4 | -2.282108963 | 0.044887248 | | down |
| FBP1 | -2.281744291 | 1.44E-08 | 5.50E-07 | down |

| | | | | |
|---|---|---|---|---|
| RP11-747D18.1 | -2.281587794 | 9.89E-05 | 0.000935922 | down |
| NA | -2.281337645 | 0.001160067 | 0.006821893 | down |
| RP1-144C9.2 | -2.281331694 | 0.434581392 | | down |
| AC009228.1 | -2.281223369 | 6.11E-06 | 9.38E-05 | down |
| ASB5 | -2.281078139 | 0.001959093 | 0.010300837 | down |
| RP1-29C18.10 | -2.281066204 | 0.008108181 | 0.031232373 | down |
| NR0B2 | -2.280915033 | 0.000169835 | 0.001460784 | down |
| FOXN3P1 | -2.280901638 | 0.193473081 | 0.321588119 | down |
| LINC01169 | -2.280674986 | 0.007006728 | 0.028026911 | down |
| DLX2-AS1 | -2.280614701 | 0.434587553 | | down |
| NA | -2.280478668 | 0.211881662 | | down |
| CHCHD2P11 | -2.280374663 | 0.165895763 | 0.288837059 | down |
| NA | -2.280075493 | 0.00128907 | 0.007401144 | down |
| NA | -2.279640746 | 0.205326234 | | down |
| LINC00934 | -2.279501427 | 0.079388641 | 0.171205233 | down |
| TMEM220 | -2.279458125 | 1.14E-21 | 9.51E-19 | down |
| GABRG1 | -2.278838057 | 0.022182287 | 0.067025563 | down |
| AC005550.3 | -2.27875232 | 1.34E-09 | 6.64E-08 | down |
| ANHX | -2.278165514 | 0.393452587 | | down |
| RP11-1152H15.1 | -2.277311876 | 0.031308353 | 0.08654229 | down |
| CTC-499B15.6 | -2.277254587 | 0.010242728 | 0.037341648 | down |
| NA | -2.277246809 | 0.435337289 | | down |
| LINC00571 | -2.277155748 | 0.008226838 | 0.03159089 | down |
| CTD-3252C9.2 | -2.276929752 | 0.014635563 | 0.049003773 | down |
| TGM4 | -2.27683181 | 6.83E-05 | 0.000688204 | down |
| CTD-3064H18.2 | -2.276514233 | 0.073196622 | 0.161510029 | down |
| COX6CP7 | -2.276473817 | 0.206066862 | | down |
| GGT2 | -2.276380001 | 0.020447083 | 0.063135885 | down |
| RP11-339A11.2 | -2.276334476 | 0.295178932 | 0.432588718 | down |
| PGPEP1L | -2.276134598 | 0.001154929 | 0.006802332 | down |
| NA | -2.275803252 | 0.110654874 | 0.217274363 | down |
| OR4K14 | -2.275151232 | 0.386316401 | | down |
| RN7SKP38 | -2.275147582 | 0.20032836 | | down |
| LINC01115 | -2.275105907 | 0.051261121 | 0.124574174 | down |
| MT1H | -2.274883486 | 4.41E-07 | 1.01E-05 | down |
| LINC01193 | -2.274308116 | 0.042224742 | | down |
| CTC-265N9.1 | -2.274200861 | 0.336959733 | | down |
| RP11-385M4.1 | -2.274150459 | 0.253010521 | 0.388005793 | down |
| LEAP2 | -2.273892047 | 3.10E-13 | 3.98E-11 | down |
| NA | -2.273852744 | 0.071923498 | 0.159462 | down |
| MT3 | -2.273824097 | 4.45E-08 | 1.42E-06 | down |
| LINC00612 | -2.27353712 | 0.000256368 | 0.002053949 | down |
| AC000124.1 | -2.273488363 | 0.06889713 | | down |
| NA | -2.27278406 | 0.095348413 | | down |
| RPL21P121 | -2.272703691 | 0.172386278 | | down |
| RP11-28G8.1 | -2.272660041 | 0.019821569 | 0.061635191 | down |
| COX7B2 | -2.272558664 | 0.251012503 | 0.385730635 | down |
| RP11-570L14.1 | -2.272358368 | 0.436399767 | | down |
| P2RX6P | -2.27216153 | 0.088305333 | 0.185010142 | down |
| Y_RNA | -2.27183567 | 0.105773073 | 0.2103698 | down |
| NA | -2.271218632 | 0.394819976 | | down |

| | | | | |
|---|---|---|---|---|
| NA | -2.271136275 | 0.000312517 | 0.002412452 | down |
| RP11-544A12.8 | -2.270943192 | 0.001547757 | 0.008549514 | down |
| RP11-331K21.1 | -2.270917034 | 0.284953102 | | down |
| NA | -2.270640966 | 0.000131606 | 0.001186663 | down |
| RP11-291L22.4 | -2.270499728 | 0.436776952 | | down |
| PPEF2 | -2.270334901 | 0.016700294 | 0.054102181 | down |
| RP11-47304.3 | -2.270178596 | 0.000664119 | 0.00437093 | down |
| RNU1-22P | -2.270163757 | 0.106530088 | | down |
| RP11-158K1.3 | -2.26996749 | 4.39E-10 | 2.51E-08 | down |
| NA | -2.269811276 | 0.208905328 | | down |
| NUCB1-AS1 | -2.269680576 | 0.15678184 | 0.27734065 | down |
| RP11-789C2.1 | -2.268842759 | 0.038296744 | 0.10063612 | down |
| RP1-266L20.4 | -2.268697456 | 0.391301007 | | down |
| VSX2 | -2.268493171 | 0.016824394 | 0.054398633 | down |
| CRYBA2 | -2.26847888 | 2.22E-06 | 4.05E-05 | down |
| SMC1B | -2.268315884 | 1.84E-06 | 3.45E-05 | down |
| RP4-566D2.1 | -2.267993226 | 0.232922242 | 0.365636441 | down |
| RTBDN | -2.267879514 | 0.000267783 | 0.002128379 | down |
| RUVBL1-AS1 | -2.267112082 | 0.251775977 | | down |
| RXFP4 | -2.267076592 | 6.24E-07 | 1.37E-05 | down |
| CYP3A51P | -2.266871292 | 0.43751383 | | down |
| MFSD13B | -2.266786407 | 0.000371055 | 0.002753944 | down |
| BRINP1 | -2.266673768 | 9.73E-05 | 0.000923866 | down |
| Y_RNA | -2.266305284 | 0.139121025 | | down |
| CTB-51A17.1 | -2.266272056 | 0.437635594 | | down |
| RP11-48B3.3 | -2.265932787 | 0.004912942 | 0.021405303 | down |
| GDI2P1 | -2.265596624 | 0.251091406 | 0.385832151 | down |
| RP11-240E2.2 | -2.265410273 | 0.091262192 | 0.189330292 | down |
| AC108142.1 | -2.265196651 | 0.000159327 | 0.001385881 | down |
| NA | -2.26507728 | 0.286638098 | | down |
| RP11-1099M24.8 | -2.264744925 | 0.0791282 | 0.1708464 | down |
| CYCSP43 | -2.264356253 | 0.437589766 | | down |
| LINC00661 | -2.26392581 | 0.021008992 | 0.064387857 | down |
| RPL36AP45 | -2.263878218 | 0.003589345 | 0.016706163 | down |
| RPL21P23 | -2.263798943 | 0.065011002 | 0.147980192 | down |
| ASLP1 | -2.263408146 | 0.00082877 | 0.005227704 | down |
| RP11-525G13.2 | -2.263140358 | 0.003774671 | 0.017401597 | down |
| PRNT | -2.262827327 | 0.438335935 | | down |
| HOXB-AS4 | -2.262630876 | 7.56E-09 | 3.08E-07 | down |
| LINC01019 | -2.262523225 | 0.438397793 | | down |
| RP11-440J4.2 | -2.262523225 | 0.438397793 | | down |
| AMD1P3 | -2.262380988 | 0.174446768 | | down |
| LINC01105 | -2.261235441 | 0.002669812 | 0.013229725 | down |
| RP11-284G10.1 | -2.261038004 | 0.099719556 | 0.201531975 | down |
| GRM7 | -2.260772636 | 2.53E-05 | 0.000304363 | down |
| AC092839.3 | -2.260331168 | 0.397899966 | | down |
| NKX1-2 | -2.259548187 | 0.08514378 | | down |
| EEF1A1P27 | -2.259139734 | 0.439086364 | | down |
| MIR1-1HG | -2.258667096 | 0.014562199 | 0.048807086 | down |
| RP11-849F2.4 | -2.258089559 | 0.174906147 | | down |
| KRT8P9 | -2.257865851 | 0.002439218 | 0.012320757 | down |

| | | | | |
|---|---|---|---|---|
| RP11-626H12.3 | -2.257666289 | 0.289566686 | | down |
| RP11-366L20.2 | -2.257451071 | 0.000516092 | 0.003568578 | down |
| KCTD9P4 | -2.257337219 | 0.041347657 | 0.106482973 | down |
| SFRP5 | -2.257058782 | 5.15E-06 | 8.13E-05 | down |
| NA | -2.256960103 | 0.29183611 | | down |
| CTD-2313N18.8 | -2.256942377 | 0.396331525 | | down |
| RP11-369C8.1 | -2.256891902 | 0.139368131 | | down |
| NA | -2.256597224 | 0.393441114 | | down |
| RP3-333B15.4 | -2.256522513 | 0.439619416 | | down |
| RP11-449L13.2 | -2.256184468 | 0.341707082 | | down |
| RN7SKP106 | -2.256065633 | 0.235267919 | 0.368106934 | down |
| RP11-325L7.1 | -2.255710104 | 0.05662489 | 0.133954898 | down |
| CCDC148 | -2.25545092 | 0.000335349 | 0.002553924 | down |
| FER1L6-AS2 | -2.255308239 | 0.124111941 | | down |
| NA | -2.255126907 | 0.029613133 | 0.083040598 | down |
| NAP1L4P3 | -2.254982798 | 0.07062021 | | down |
| RP11-644F5.15 | -2.254857397 | 0.016441204 | 0.053487766 | down |
| NA | -2.254047502 | 0.110340957 | 0.21688477 | down |
| RP11-419J16.1 | -2.253103778 | 0.062592336 | | down |
| CTD-2301A4.1 | -2.252944367 | 0.043317093 | | down |
| KRT79 | -2.252926085 | 0.00370426 | 0.017137477 | down |
| HTR1D | -2.252593228 | 1.53E-05 | 0.000202171 | down |
| AC147651.1 | -2.252423842 | 0.440248526 | | down |
| ABCB1 | -2.252305091 | 3.87E-11 | 2.98E-09 | down |
| TEX101 | -2.25221793 | 0.004404718 | 0.019660579 | down |
| SLC28A2 | -2.252001798 | 6.23E-06 | 9.56E-05 | down |
| NUTF2P6 | -2.25195121 | 4.52E-07 | 1.03E-05 | down |
| RP4-655J12.5 | -2.251813657 | 0.440579405 | | down |
| TDGF1P3 | -2.251672993 | 0.202901901 | 0.33231491 | down |
| STAP2 | -2.251614326 | 6.42E-11 | 4.59E-09 | down |
| NA | -2.251530572 | 0.000695409 | 0.004540942 | down |
| MT1CP | -2.251487387 | 0.070382086 | | down |
| MRPL37P1 | -2.251383271 | 0.000664281 | 0.00437093 | down |
| GPAT2P2 | -2.251241228 | 0.000599918 | 0.004031703 | down |
| GS1-174L6.4 | -2.250891268 | 0.187484694 | 0.314070323 | down |
| AP000439.3 | -2.250383129 | 1.84E-10 | 1.15E-08 | down |
| CUBNP3 | -2.249913743 | 0.025706372 | 0.074838685 | down |
| STAU2-AS1 | -2.249452386 | 5.83E-06 | 9.02E-05 | down |
| RP11-357P18.3 | -2.248518327 | 0.021209662 | 0.064857311 | down |
| RN7SL34P | -2.248127153 | 0.007048923 | 0.028135757 | down |
| TBX5-AS1 | -2.248114043 | 0.247324059 | | down |
| TPST2P1 | -2.247817325 | 0.441253749 | | down |
| ERC2 | -2.247605676 | 3.67E-05 | 0.000415051 | down |
| RP11-15A1.4 | -2.24754415 | 4.55E-06 | 7.35E-05 | down |
| NA | -2.247486218 | 4.62E-07 | 1.05E-05 | down |
| RP3-47704.5 | -2.247305937 | 0.29081319 | | down |
| RP1-232L24.3 | -2.246915851 | 0.076696576 | | down |
| LINC00856 | -2.24681645 | 1.87E-07 | 4.88E-06 | down |
| ALLC | -2.24679234 | 0.056375211 | | down |
| KRT8P5 | -2.246419022 | 0.246438961 | | down |
| AC105393.1 | -2.24634799 | 0.441622718 | | down |

| | | | | |
|---|---|---|---|---|
| AC012485.2 | -2.246124515 | 5.31E-05 | 0.000560533 | down |
| CBR3-AS1 | -2.245946927 | 9.40E-08 | 2.69E-06 | down |
| HMGN2P16 | -2.245822941 | 0.393264606 | 0.530641503 | down |
| RP11-619A14.3 | -2.245643404 | 4.15E-06 | 6.82E-05 | down |
| NAV2-AS3 | -2.245613715 | 0.210272139 | | down |
| AF064858.7 | -2.245501825 | 0.015756371 | 0.051849334 | down |
| NOVA1-AS1 | -2.245299194 | 4.69E-05 | 0.000507264 | down |
| PDZD3 | -2.244782655 | 5.79E-11 | 4.24E-09 | down |
| Xbac-BPG308K3.5 | -2.24469698 | 0.022511163 | 0.067738219 | down |
| RN7SKP284 | -2.244693529 | 0.44203325 | | down |
| RAB28P1 | -2.244681731 | 0.442035661 | | down |
| CCDC62 | -2.244651511 | 2.91E-05 | 0.000341164 | down |
| RP11-496D1.2 | -2.244275683 | 0.068861508 | 0.154482473 | down |
| MTND2P41 | -2.244234785 | 0.442127013 | | down |
| AC004063.1 | -2.244184704 | 0.210867634 | 0.341415563 | down |
| HTR3E | -2.243869078 | 2.76E-05 | 0.000326393 | down |
| RP11-1055B8.2 | -2.243153187 | 0.072483262 | 0.160407124 | down |
| LINC01564 | -2.242893455 | 0.002142871 | 0.011094335 | down |
| BTBD8 | -2.24266959 | 7.85E-11 | 5.51E-09 | down |
| Y_RNA | -2.24259813 | 0.442389021 | | down |
| AC016712.1 | -2.242539621 | 0.08255279 | 0.176314497 | down |
| RP11-542P2.2 | -2.242268596 | 0.442456399 | | down |
| NA | -2.242231497 | 0.44239488 | | down |
| GAS2L1P1 | -2.242231497 | 0.44239488 | | down |
| FGF16 | -2.241901696 | 0.018249633 | 0.057989633 | down |
| CTRB1 | -2.241869839 | 0.064734305 | 0.147573977 | down |
| RPSAP52 | -2.241738372 | 0.001883641 | 0.010007448 | down |
| RP11-275H4.1 | -2.241686194 | 0.068954415 | 0.154636431 | down |
| RN7SL155P | -2.241660387 | 0.442213108 | | down |
| RP5-978I12.1 | -2.241568461 | 0.012206199 | 0.042726674 | down |
| HIPK1-AS1 | -2.2412808 | 6.93E-07 | 1.49E-05 | down |
| AC012362.3 | -2.240925582 | 0.442803724 | | down |
| TUBB4BP7 | -2.240925582 | 0.442803724 | | down |
| RP11-285A18.2 | -2.240925582 | 0.442803724 | | down |
| NA | -2.240925582 | 0.442803724 | | down |
| OR8J3 | -2.240764296 | 0.44276405 | | down |
| LINC00470 | -2.240469316 | 0.002835829 | 0.013871645 | down |
| ARHGEF26-AS1 | -2.240311563 | 0.000150675 | 0.001325199 | down |
| NA | -2.24026718 | 0.442938434 | | down |
| AC006369.3 | -2.240211528 | 0.215313583 | 0.346355479 | down |
| RNU6-319P | -2.240178998 | 0.117789465 | 0.227083335 | down |
| RP13-488H8.1 | -2.239931074 | 0.155619621 | 0.275820654 | down |
| RP11-360K13.1 | -2.239877441 | 0.1223273 | 0.23314908 | down |
| RP11-349A22.3 | -2.239815596 | 0.442823106 | | down |
| RP11-521M14.1 | -2.239565053 | 0.146287503 | | down |
| RP11-38014.5 | -2.239515801 | 0.106001706 | 0.210698992 | down |
| ACTN2 | -2.239330815 | 2.30E-06 | 4.17E-05 | down |
| LCE1A | -2.239283537 | 0.203965584 | | down |
| RPSAP23 | -2.239180356 | 0.397997406 | | down |
| RP1-102H19.7 | -2.239138563 | 0.261364299 | 0.396507221 | down |
| C5orf64 | -2.238398414 | 0.001880313 | 0.009994946 | down |

| | | | | |
|---|---|---|---|---|
| PRDM7 | -2.238187353 | 0.14991555 | | down |
| PCAT14 | -2.237624863 | 0.019476956 | 0.06082484 | down |
| LINC00311 | -2.23752783 | 0.001065753 | 0.006378385 | down |
| RP11-739P1.3 | -2.237103544 | 0.022341283 | 0.067324123 | down |
| NA | -2.236994632 | 0.011896664 | 0.041916662 | down |
| TUBB8P9 | -2.236986997 | 0.443609908 | | down |
| SMIM2-AS1 | -2.236838522 | 2.25E-09 | 1.05E-07 | down |
| SLC25A5P4 | -2.236619129 | 0.443414426 | | down |
| SNTN | -2.236492525 | 0.000388921 | 0.002851331 | down |
| OR5P4P | -2.236459733 | 0.249639039 | | down |
| TPRN | -2.236136062 | 4.38E-11 | 3.31E-09 | down |
| NLRP9 | -2.236101742 | 0.000881518 | 0.005473288 | down |
| AC073071.1 | -2.235929855 | 0.443826435 | | down |
| RP11-81A1.3 | -2.23547806 | 0.259934548 | 0.395096305 | down |
| RP11-706C16.7 | -2.235357946 | 0.028599731 | 0.081088308 | down |
| NA | -2.235328801 | 2.57E-05 | 0.000307619 | down |
| SLC5A4 | -2.235133466 | 0.000190651 | 0.001607158 | down |
| OR51A7 | -2.234702927 | 0.227338986 | 0.359501685 | down |
| EFCAB8 | -2.234342766 | 9.31E-07 | 1.91E-05 | down |
| SNORA20 | -2.234233706 | 0.444173971 | | down |
| HMGB1P32 | -2.234233706 | 0.444173971 | | down |
| RP11-474P2.5 | -2.234041923 | 0.03291778 | 0.089817618 | down |
| AP001187.11 | -2.234032154 | 0.211531363 | | down |
| NA | -2.233923671 | 0.059105228 | 0.138233266 | down |
| NA | -2.233614028 | 0.076939909 | 0.167421064 | down |
| FGF23 | -2.233609163 | 0.056773617 | 0.134223051 | down |
| OLIG3 | -2.233371835 | 0.444350625 | | down |
| LINC01429 | -2.232964734 | 0.124141856 | 0.235569736 | down |
| RP11-477N12.5 | -2.232814431 | 0.400464148 | | down |
| C2orf83 | -2.232688202 | 0.053350871 | 0.128127385 | down |
| LACTBL1 | -2.232684686 | 0.444491496 | | down |
| FLJ40194 | -2.232572503 | 8.31E-08 | 2.44E-06 | down |
| RNU6-703P | -2.232543697 | 0.144595761 | | down |
| RNF212B | -2.231419909 | 0.000969728 | 0.005906407 | down |
| RP11-14D22.1 | -2.231384702 | 0.145207732 | 0.262887684 | down |
| NA | -2.231277971 | 0.444706943 | | down |
| DDC | -2.230798342 | 8.30E-10 | 4.34E-08 | down |
| MATN4 | -2.230653463 | 1.30E-07 | 3.56E-06 | down |
| SOX2-OT | -2.230534351 | 6.85E-05 | 0.000689021 | down |
| Y_RNA | -2.230489963 | 0.050393637 | 0.123011571 | down |
| NA | -2.230193408 | 0.298664618 | | down |
| CDKN2B | -2.230060413 | 1.65E-10 | 1.05E-08 | down |
| RP11-512C24.3 | -2.229628626 | 0.403877976 | | down |
| PTPRD-AS2 | -2.229559754 | 0.347320975 | | down |
| RP11-524O1.4 | -2.228883043 | 0.00017304 | 0.001483254 | down |
| RP11-96D1.6 | -2.228840979 | 7.72E-06 | 0.000114053 | down |
| IFNWP18 | -2.22871947 | 0.445162203 | | down |
| BCRP2 | -2.228663428 | 0.090446931 | | down |
| RP1-200K18.1 | -2.22842436 | 0.002727888 | 0.013468637 | down |
| U6atac | -2.228285324 | 0.445122042 | | down |
| TM4SF4 | -2.227865464 | 0.002483273 | 0.012501246 | down |

| | | | | |
|---|---|---|---|---|
| RP11-50D16.4 | -2.227565752 | 6.29E-05 | 0.000645092 | down |
| STK31 | -2.226712025 | 6.91E-06 | 0.00010405 | down |
| NA | -2.226338077 | 0.445720598 | | down |
| ERBB3 | -2.226124219 | 4.44E-11 | 3.34E-09 | down |
| LINCMD1 | -2.225951697 | 0.151161666 | | down |
| SPATA32 | -2.225926477 | 3.34E-06 | 5.68E-05 | down |
| CTD-2651B20.4 | -2.225766699 | 0.020237053 | 0.062674159 | down |
| RP11-227H15.5 | -2.225234091 | 0.04788051 | 0.118504163 | down |
| LINC01251 | -2.224800806 | 0.015694847 | 0.051703848 | down |
| MEGF11 | -2.224670222 | 2.26E-06 | 4.11E-05 | down |
| RPL21P4 | -2.224586801 | 0.031010064 | 0.085891617 | down |
| RP11-432I5.2 | -2.224519802 | 0.000123648 | 0.001130513 | down |
| S100A7A | -2.224479336 | 0.40506884 | | down |
| RP11-4M23.3 | -2.224303491 | 0.051668407 | 0.125237123 | down |
| UGT1A9 | -2.224252739 | 0.026041396 | 0.075556513 | down |
| KB-1125A3.12 | -2.223775164 | 0.000542818 | 0.003719985 | down |
| AC105339.1 | -2.223714189 | 0.005610259 | 0.023686708 | down |
| AC009963.6 | -2.223669664 | 0.238411268 | 0.371882463 | down |
| CTD-2022H16.3 | -2.22344251 | 0.003597282 | 0.016735328 | down |
| CTC-431G16.2 | -2.223379214 | 0.040896334 | 0.105593801 | down |
| RNU6-364P | -2.223329054 | 0.348716205 | | down |
| RP11-10J18.3 | -2.223206794 | 0.446363809 | | down |
| MIR5689HG | -2.223170698 | 0.446235066 | | down |
| PSG7 | -2.222835236 | 0.347369189 | | down |
| AC093166.3 | -2.222707272 | 0.136229758 | 0.251359508 | down |
| OR55B1P | -2.222121814 | 0.119051112 | 0.228840154 | down |
| HELLPAR | -2.221859236 | 7.75E-06 | 0.000114358 | down |
| GOLGA6L7P | -2.221379983 | 0.09969957 | 0.201518695 | down |
| CABS1 | -2.221361915 | 0.446673188 | | down |
| CYP2G1P | -2.221090785 | 0.079086554 | | down |
| RP11-413H22.2 | -2.220703221 | 0.268100171 | 0.403830419 | down |
| RP11-154P18.2 | -2.220673031 | 0.00077253 | 0.004932704 | down |
| TRIM64DP | -2.220521481 | 0.401833558 | | down |
| NA | -2.220114541 | 0.005329176 | 0.022791425 | down |
| AC005014.5 | -2.219724479 | 0.118495142 | | down |
| CTD-2005D20.1 | -2.21970537 | 0.088246134 | 0.184928672 | down |
| RP11-286N22.8 | -2.21940286 | 1.08E-06 | 2.17E-05 | down |
| TUBBP8 | -2.219331684 | 0.447233995 | | down |
| RP11-461K13.1 | -2.219008107 | 0.406419583 | | down |
| GLTSCR1-AS1 | -2.21842149 | 0.04515839 | 0.113515617 | down |
| RPL23AP75 | -2.218176005 | 0.181521333 | | down |
| RP11-60L3.3 | -2.218037285 | 0.347513627 | | down |
| NA | -2.217753432 | 0.000390287 | 0.002858554 | down |
| RP1-102G20.5 | -2.217457027 | 0.164791589 | | down |
| CTB-129P6.7 | -2.217333402 | 0.000112276 | 0.001042401 | down |
| RP5-1057B20.3 | -2.217183722 | 0.054940762 | 0.131055201 | down |
| MCHR2 | -2.217046627 | 0.008952518 | 0.033697 | down |
| RP11-734K21.3 | -2.217029321 | 0.06942858 | | down |
| RP11-486A14.1 | -2.216919448 | 4.20E-09 | 1.82E-07 | down |
| NANOGP6 | -2.216903429 | 0.347639201 | | down |
| AC007277.3 | -2.216709246 | 0.019512231 | 0.060890548 | down |

| | | | | |
|---|---|---|---|---|
| RP11-1379J22.5 | -2.216076632 | 5.79E-09 | 2.43E-07 | down |
| NUFIP1P | -2.215947294 | 0.447857037 | | down |
| OR9Q1 | -2.21585873 | 0.033631451 | 0.091300562 | down |
| LINC01247 | -2.215851778 | 0.25912034 | | down |
| RP11-205M3.3 | -2.215846238 | 0.404234128 | | down |
| RP11-77403.2 | -2.215530793 | 0.447942794 | | down |
| OR9K1P | -2.215530793 | 0.447942794 | | down |
| GHRH | -2.215491236 | 0.112396552 | 0.219560598 | down |
| RP11-118E18.2 | -2.215353856 | 0.222751192 | | down |
| RN7SL549P | -2.215339247 | 0.346989617 | | down |
| NA | -2.214920141 | 0.145398953 | 0.263122885 | down |
| ST13P20 | -2.214880169 | 0.173445552 | | down |
| AC010745.2 | -2.214428278 | 0.195170196 | 0.323603387 | down |
| CLEC18C | -2.214411315 | 0.000457989 | 0.003245243 | down |
| RP4-671014.7 | -2.214346298 | 1.94E-06 | 3.62E-05 | down |
| LGSN | -2.214145463 | 0.003796356 | 0.017480111 | down |
| NPY4R | -2.213794197 | 0.000154498 | 0.001351701 | down |
| BC7-42418200C9. | -2.2136496 | 0.084827377 | 0.179716079 | down |
| LINC00700 | -2.213368549 | 0.300934474 | | down |
| RPS15AP17 | -2.21308982 | 0.063445721 | | down |
| LHX5-AS1 | -2.212903403 | 0.448557674 | | down |
| ACSM6 | -2.212888575 | 0.026866959 | 0.077369014 | down |
| RP11-327J17.7 | -2.212880091 | 8.03E-05 | 0.000788189 | down |
| Y_RNA | -2.212869202 | 0.164113572 | 0.286481212 | down |
| RP3-335N17.2 | -2.212614632 | 0.448617188 | | down |
| KRT8P4 | -2.212614632 | 0.448617188 | | down |
| RP1-136J15.5 | -2.212614632 | 0.448617188 | | down |
| RP11-195C7.2 | -2.212304962 | 0.178268411 | 0.303364267 | down |
| LMX1A | -2.212104203 | 0.000406359 | 0.002944685 | down |
| RP11-119D9.1 | -2.212045497 | 1.00E-12 | 1.13E-10 | down |
| PIWIL2 | -2.211803847 | 4.51E-12 | 4.29E-10 | down |
| AC092071.1 | -2.211745252 | 0.234212303 | 0.366971287 | down |
| CASC22 | -2.211695192 | 0.402728641 | | down |
| RN7SL67P | -2.211469554 | 0.104212353 | 0.207912606 | down |
| NA | -2.211413053 | 0.00801286 | 0.031007003 | down |
| BOLA3P1 | -2.211408511 | 0.19234522 | 0.32025613 | down |
| WTAPP1 | -2.211330356 | 0.001257504 | 0.007272466 | down |
| RPL35AP4 | -2.211261327 | 0.069558499 | 0.155645072 | down |
| Metazoa_SRP | -2.211159525 | 0.001128351 | 0.006682471 | down |
| MT-ND5 | -2.211096659 | 0.001877519 | 0.00998375 | down |
| FTLP5 | -2.210917065 | 0.142405588 | 0.259330433 | down |
| SPACA4 | -2.210605434 | 9.19E-06 | 0.00013201 | down |
| ZDHHC20P3 | -2.210264321 | 0.044046554 | 0.11153377 | down |
| RP11-407P15.2 | -2.210226001 | 0.161351279 | 0.28302416 | down |
| CABP7 | -2.210199076 | 0.000279302 | 0.002201878 | down |
| RP11-396020.1 | -2.210090072 | 0.031612194 | 0.087181636 | down |
| T | -2.209986473 | 0.001892085 | 0.010036337 | down |
| HSPA8P3 | -2.209914331 | 0.076564992 | 0.166854468 | down |
| RP3-394A18.1 | -2.209798445 | 1.93E-08 | 6.88E-07 | down |
| RP11-651L5.2 | -2.209789174 | 0.00499777 | 0.021685875 | down |
| CTD-2272G21.3 | -2.20944913 | 0.150974613 | 0.270193102 | down |

| | | | | |
|---|---|---|---|---|
| AC069277.2 | -2.20912157 | 3.11E-05 | 0.000360498 | down |
| PIWIL3 | -2.209075001 | 0.1388661 | 0.254846565 | down |
| YBX2P1 | -2.209002914 | 0.157467252 | 0.278209123 | down |
| CTD-2515A14.1 | -2.208901431 | 0.000744123 | 0.004790325 | down |
| SPATA31B1P | -2.208728132 | 0.449418609 | | down |
| RP11-429A20.4 | -2.208645384 | 0.018066923 | 0.057549051 | down |
| AC010731.3 | -2.208413146 | 0.449483597 | | down |
| NA | -2.208413146 | 0.449483597 | | down |
| RP11-542B15.1 | -2.20819753 | 0.050496024 | 0.123181435 | down |
| RP11-467M13.3 | -2.208045972 | 0.178237711 | 0.303347397 | down |
| RP11-767C4.1 | -2.207712575 | 0.449628157 | | down |
| AC011343.1 | -2.207557291 | 0.192089078 | | down |
| RP11-524O24.2 | -2.207442455 | 0.014680449 | | down |
| Y_RNA | -2.20734516 | 0.300105337 | | down |
| OR56B3P | -2.207292567 | 0.449714836 | | down |
| CTD-2049O17.1 | -2.207281227 | 0.199702084 | 0.328673954 | down |
| Y_RNA | -2.207122802 | 0.449675974 | | down |
| AC005392.13 | -2.206926117 | 0.449790471 | | down |
| NA | -2.206907947 | 0.449794221 | | down |
| RP11-503N18.5 | -2.206721328 | 0.000275417 | 0.002176385 | down |
| TERB2 | -2.206558349 | 0.228509687 | 0.360821203 | down |
| AC018832.1 | -2.206300757 | 0.056928321 | 0.134485002 | down |
| ASS1P10 | -2.205152716 | 0.151193026 | | down |
| DIAPH3-AS2 | -2.205028594 | 0.450108252 | | down |
| NA | -2.204903321 | 6.84E-05 | 0.000688836 | down |
| SNORD37 | -2.20459637 | 0.352201813 | | down |
| DLX6-AS1 | -2.204591927 | 0.03448383 | 0.093116558 | down |
| TPTE2P1 | -2.204466453 | 1.24E-08 | 4.82E-07 | down |
| RPL7AP61 | -2.204249525 | 0.450343129 | | down |
| RP11-1C1.4 | -2.203365975 | 0.406041475 | | down |
| RP11-219G17.6 | -2.20318386 | 0.181373 | | down |
| KRT25 | -2.203046209 | 0.034521935 | 0.093190597 | down |
| TBC1D3P7 | -2.202895523 | 0.450622846 | | down |
| MKRN7P | -2.202816279 | 0.136524868 | | down |
| AC026188.1 | -2.202803639 | 0.186587144 | | down |
| RP11-863K10.2 | -2.202209622 | 0.450764581 | | down |
| GCM2 | -2.202091695 | 0.450714869 | | down |
| PCDHA10 | -2.202088242 | 0.000173189 | 0.001484106 | down |
| PRAMEF18 | -2.201750263 | 0.223673298 | | down |
| NPM1P11 | -2.201114566 | 0.111783215 | 0.218927505 | down |
| RP11-90K18.1 | -2.200910909 | 0.091849046 | 0.190232507 | down |
| RP11-268P4.4 | -2.200868099 | 0.039767091 | | down |
| RP11-358M3.1 | -2.200831726 | 0.212741329 | 0.343560552 | down |
| Y_RNA | -2.20017558 | 0.072849055 | 0.160961035 | down |
| BRS3 | -2.199809573 | 0.023265957 | 0.069414341 | down |
| LINC00433 | -2.199551727 | 0.051075697 | 0.12423237 | down |
| RLIMP1 | -2.199223602 | 0.006133589 | 0.025382584 | down |
| RP11-417L19.2 | -2.199087649 | 0.058972742 | 0.138021115 | down |
| RP11-274M17.1 | -2.19907546 | 0.208236513 | 0.338414597 | down |
| RP1-302D9.2 | -2.198726038 | 0.154303916 | 0.274038355 | down |
| CITED1 | -2.198591405 | 0.110708284 | | down |

| | | | | |
|---|---|---|---|---|
| NA | -2.198409651 | 0.223748251 | | down |
| CRCT1 | -2.198159956 | 0.353519212 | | down |
| AC005086.1 | -2.197999676 | 0.013970999 | 0.047316503 | down |
| RP11-711K1.7 | -2.197824985 | 0.16729056 | 0.290440774 | down |
| SULT1A1 | -2.197750535 | 1.57E-18 | 6.83E-16 | down |
| MYT1L | -2.197400267 | 0.001582585 | 0.008709871 | down |
| AC004791.2 | -2.197265141 | 1.55E-05 | 0.000204098 | down |
| SDCBPP1 | -2.1971917 | 0.103067706 | 0.206272725 | down |
| NA | -2.196459248 | 0.056866586 | 0.134379401 | down |
| SETP8 | -2.196399192 | 0.263892237 | | down |
| RPS10P21 | -2.196212503 | 0.153690233 | 0.273359968 | down |
| NEGR1 | -2.196035188 | 3.92E-09 | 1.72E-07 | down |
| RP11-435F17.1 | -2.195863638 | 0.00108792 | 0.006490364 | down |
| RP11-361H10.7 | -2.19570133 | 0.452110708 | | down |
| RP11-217O12.1 | -2.195663743 | 0.075017945 | 0.164321898 | down |
| RP11-204E9.3 | -2.195661192 | 0.17819025 | | down |
| C12orf56 | -2.195591017 | 0.00433281 | 0.019394362 | down |
| NA | -2.195520841 | 0.000166189 | 0.001435179 | down |
| NA | -2.195455125 | 0.088411031 | | down |
| CTC-321K16.1 | -2.195365599 | 0.413734396 | | down |
| CTC-527H23.3 | -2.195176154 | 0.009234587 | 0.034500294 | down |
| RP11-276H7.3 | -2.195083817 | 0.000398467 | 0.002908536 | down |
| RP11-61O11.1 | -2.194960805 | 0.168518864 | 0.291949069 | down |
| CTC-444N24.9 | -2.194838476 | 0.355931259 | | down |
| RP11-96D24.1 | -2.194660538 | 0.005877775 | 0.024565979 | down |
| CTC-260E6.6 | -2.19465993 | 1.47E-06 | 2.85E-05 | down |
| FABP12 | -2.194517815 | 0.356600644 | | down |
| RP11-458I7.1 | -2.194228542 | 0.191542853 | 0.319336578 | down |
| RP4-673D20.6 | -2.193818416 | 0.411402289 | | down |
| ASIC4 | -2.193035269 | 2.98E-05 | 0.00034792 | down |
| LINC01478 | -2.193003809 | 0.452594891 | | down |
| RP11-1060J15.9 | -2.192871065 | 5.00E-06 | 7.93E-05 | down |
| LRP2 | -2.192696789 | 0.030190342 | 0.084187309 | down |
| NKAIN1 | -2.192661924 | 9.13E-05 | 0.000876955 | down |
| GRIA2 | -2.192542549 | 0.004056161 | 0.018455748 | down |
| RP11-483I13.6 | -2.192119591 | 6.29E-06 | 9.63E-05 | down |
| FAM184B | -2.19192119 | 2.04E-07 | 5.26E-06 | down |
| PPIAL4G | -2.191488758 | 0.001337034 | 0.007620918 | down |
| RP11-34A14.3 | -2.191455598 | 0.268787537 | | down |
| RP11-384F7.1 | -2.191195854 | 0.007476961 | 0.029418259 | down |
| GDPD1 | -2.190868355 | 6.29E-14 | 9.64E-12 | down |
| MIR194-2HG | -2.190737555 | 1.35E-07 | 3.69E-06 | down |
| NA | -2.190439439 | 0.303371796 | | down |
| PPFIA3 | -2.190402509 | 2.99E-12 | 2.93E-10 | down |
| ELSPBP1 | -2.19035109 | 0.453219003 | | down |
| RP11-215P8.4 | -2.190345654 | 0.030358018 | 0.084537611 | down |
| GLRA4 | -2.190282885 | 0.000296218 | 0.002309176 | down |
| ZNF804B | -2.190097826 | 0.109540168 | | down |
| H3F3AP5 | -2.189800419 | 0.260756492 | | down |
| RP1-170O19.24 | -2.189612648 | 0.000199345 | 0.001666315 | down |
| ESRRAP1 | -2.189567136 | 0.057394835 | 0.135223526 | down |

| | | | | |
|---|---|---|---|---|
| RP13-467H17.1 | -2.189330089 | 0.000178104 | 0.001521019 | down |
| FRG2IP | -2.189258736 | 0.121184698 | 0.231671822 | down |
| RN7SKP280 | -2.189077873 | 0.411738144 | | down |
| RP11-173A6.3 | -2.188966628 | 0.00540193 | 0.023027184 | down |
| NA | -2.188885825 | 2.90E-09 | 1.33E-07 | down |
| OTOGL | -2.188870758 | 9.79E-05 | 0.00092777 | down |
| AC100830.3 | -2.188270661 | 0.000146242 | 0.00129137 | down |
| CTD-2256P15.4 | -2.188120096 | 0.000235408 | 0.001913945 | down |
| RP11-83J21.3 | -2.188059764 | 0.218697626 | | down |
| RP11-9E13.4 | -2.18803626 | 0.05020148 | 0.122682061 | down |
| RP11-300M6.1 | -2.187992741 | 0.453203122 | | down |
| KIF6 | -2.18774525 | 1.63E-07 | 4.33E-06 | down |
| NA | -2.1874467 | 0.2198134 | | down |
| RP11-115H18.1 | -2.187348817 | 0.002403711 | 0.012188538 | down |
| RIMS2 | -2.187216351 | 0.000141403 | 0.001259508 | down |
| SLC45A2 | -2.186976418 | 0.002178523 | 0.011238245 | down |
| NA | -2.186622033 | 0.257926036 | | down |
| NA | -2.186485992 | 0.175862865 | 0.300547435 | down |
| SIAH1P1 | -2.186469629 | 0.355867881 | 0.494413624 | down |
| RP11-697E2.9 | -2.186159551 | 0.013228742 | 0.045303125 | down |
| KLHL34 | -2.186025443 | 4.50E-08 | 1.44E-06 | down |
| OR7E11P | -2.185342723 | 0.197175919 | | down |
| SRSF10P1 | -2.185322311 | 0.043819061 | 0.11111579 | down |
| URAD | -2.185031726 | 7.26E-07 | 1.55E-05 | down |
| HMGB1P25 | -2.184906929 | 0.45434832 | | down |
| SLC8A2 | -2.184853907 | 3.53E-06 | 5.94E-05 | down |
| RASA2-IT1 | -2.184789891 | 0.098462461 | 0.199811291 | down |
| HSD3BP2 | -2.184530232 | 0.170997146 | | down |
| RP11-473M20.11 | -2.18440591 | 1.94E-05 | 0.000244702 | down |
| ONECUT3 | -2.184353148 | 0.005929021 | 0.02472675 | down |
| NA | -2.184121841 | 0.148973757 | 0.267689448 | down |
| LINC00208 | -2.18404061 | 0.454528171 | | down |
| NA | -2.183733505 | 0.027007236 | 0.077665366 | down |
| NA | -2.183687992 | 0.307275764 | | down |
| RP11-461F11.2 | -2.18281901 | 0.000143565 | 0.001275192 | down |
| RP11-818F20.5 | -2.18248293 | 0.047427468 | 0.117670522 | down |
| RP11-76I14.1 | -2.182057358 | 0.146068977 | 0.264049119 | down |
| CTD-2377O17.1 | -2.181943528 | 0.020657635 | 0.06358732 | down |
| NA | -2.18187527 | 0.45497788 | | down |
| LINC01585 | -2.181865082 | 0.036784671 | 0.097568112 | down |
| RP11-669N7.2 | -2.181767616 | 0.455000244 | | down |
| IGDCC3 | -2.181663405 | 0.000444714 | 0.003169114 | down |
| RPL10L | -2.181164245 | 2.07E-05 | 0.000258206 | down |
| ART1 | -2.180938398 | 0.084980836 | 0.18001581 | down |
| MIR138-1 | -2.180756369 | 0.219306368 | | down |
| TMBIM7P | -2.18063266 | 0.075003728 | 0.164302734 | down |
| NA | -2.180188003 | 1.33E-05 | 0.000178614 | down |
| RP11-438L7.3 | -2.180186098 | 0.0011092 | 0.006589837 | down |
| RP11-540N6.1 | -2.180110271 | 0.067043902 | 0.151448646 | down |
| PROX1-AS1 | -2.180043735 | 6.57E-06 | 9.98E-05 | down |
| RP11-138P22.1 | -2.179975 | 0.049047029 | 0.120655771 | down |

| | | | | |
|---|---|---|---|---|
| RP4-794I6.4 | -2.179606638 | 9.60E-05 | 0.000913577 | down |
| RP11-57A19.4 | -2.179576004 | 0.028565897 | 0.081018957 | down |
| RP11-152O14.1 | -2.179322534 | 0.455508365 | | down |
| RP11-157E16.1 | -2.179311101 | 0.029301041 | 0.082479564 | down |
| KCNJ18 | -2.178743019 | 0.120663074 | 0.231036989 | down |
| LLNLR-271C9.1 | -2.178713703 | 0.000886589 | 0.005496631 | down |
| RP1-34L19.1 | -2.178136057 | 0.002397714 | 0.012162234 | down |
| ZNF33BP1 | -2.17804731 | 0.099309507 | 0.201054907 | down |
| LINC00906 | -2.177970023 | 0.074008813 | 0.162717182 | down |
| snoU13 | -2.177958049 | 0.152886811 | 0.272423424 | down |
| MKI67P1 | -2.177726662 | 0.267897598 | | down |
| RN7SL392P | -2.177609408 | 0.357765734 | | down |
| MFSD4B | -2.177597652 | 9.51E-12 | 8.38E-10 | down |
| NA | -2.177470616 | 0.213265992 | | down |
| BX255923.3 | -2.176948175 | 0.000127593 | 0.001157078 | down |
| CTD-3006G17.2 | -2.176769576 | 0.269662237 | | down |
| NA | -2.176737139 | 0.166847728 | | down |
| KRT8P30 | -2.176396982 | 0.124230393 | 0.235659675 | down |
| UNC93B6 | -2.176136844 | 0.039886979 | | down |
| AP000962.2 | -2.176054496 | 0.236006178 | 0.368993007 | down |
| MYO15A | -2.176036145 | 1.23E-08 | 4.77E-07 | down |
| RNU6-491P | -2.175937794 | 0.41402098 | | down |
| CTD-2552K11.2 | -2.175668094 | 0.414360068 | | down |
| RP1-80N2.4 | -2.174541211 | 0.000309363 | 0.002389949 | down |
| CTD-2066L21.3 | -2.17438921 | 0.092881087 | | down |
| FAM215B | -2.174342068 | 1.00E-05 | 0.000141501 | down |
| ALS2CR12 | -2.173918264 | 4.18E-06 | 6.86E-05 | down |
| Y_RNA | -2.173554591 | 0.177074649 | 0.301965647 | down |
| RP11-804H8.7 | -2.173449057 | 0.26070457 | | down |
| MISP | -2.173358619 | 1.12E-08 | 4.42E-07 | down |
| AC005235.1 | -2.172852221 | 0.183959544 | | down |
| RP11-384P7.5 | -2.172832417 | 0.418793354 | | down |
| NA | -2.172811144 | 0.042041503 | 0.10786464 | down |
| RP5-837M10.1 | -2.172738579 | 0.087984932 | 0.184506129 | down |
| C15orf32 | -2.171397946 | 0.457082184 | | down |
| KRT8P43 | -2.171393109 | 0.076924839 | 0.167412522 | down |
| AC005775.2 | -2.171360138 | 3.24E-05 | 0.000372439 | down |
| NA | -2.171302111 | 0.457030578 | | down |
| RP11-48020.5 | -2.171015593 | 0.09395328 | | down |
| CTD-2619J13.19 | -2.170786306 | 1.85E-05 | 0.000235186 | down |
| BMP10 | -2.17061108 | 0.042166861 | 0.108112407 | down |
| RP11-40E6.1 | -2.170536085 | 0.457336926 | | down |
| HSP90AB7P | -2.170438672 | 0.116256184 | 0.224964626 | down |
| CTC-490G23.2 | -2.17028259 | 5.06E-05 | 0.000540776 | down |
| RP11-465B22.8 | -2.169892712 | 9.66E-06 | 0.000137154 | down |
| RP11-252M21.6 | -2.169629877 | 0.457245852 | | down |
| TMEM37 | -2.16913913 | 2.93E-14 | 4.82E-12 | down |
| Y_RNA | -2.168791445 | 0.457625201 | | down |
| NA | -2.16867005 | 0.418086977 | | down |
| GTSCR1 | -2.168622742 | 0.003339 | 0.015748339 | down |
| RP11-439H9.1 | -2.168572906 | 0.218164717 | | down |

| | | | | |
|---|---|---|---|---|
| NA | -2.168261319 | 0.220713238 | | down |
| RP11-753N8.1 | -2.168070516 | 0.198093173 | 0.326819613 | down |
| RP11-357F12.1 | -2.167784615 | 0.274831664 | 0.411107241 | down |
| CFAP161 | -2.167672604 | 0.000814434 | 0.005149171 | down |
| LINC01533 | -2.167550678 | 0.069692613 | 0.155838408 | down |
| LINC00864 | -2.167068174 | 0.055423026 | 0.131755767 | down |
| RP11-645C24.6 | -2.166557005 | 0.306264094 | | down |
| NA | -2.166384225 | 0.360928363 | | down |
| AC139712.2 | -2.166222844 | 0.45823606 | | down |
| NA | -2.166046399 | 0.002207053 | 0.011356172 | down |
| ANKRD30BL | -2.165852024 | 0.022982496 | 0.068724179 | down |
| RP11-113I22.1 | -2.164730659 | 0.198219384 | 0.326944999 | down |
| MTATP6P2 | -2.164703771 | 0.001944749 | 0.010236164 | down |
| LINC01022 | -2.16465566 | 0.097352532 | | down |
| ANKRD22 | -2.1646079 | 1.29E-06 | 2.54E-05 | down |
| AP000350.8 | -2.164497852 | 0.458448681 | | down |
| KHDRBS2 | -2.164306227 | 4.37E-06 | 7.10E-05 | down |
| CSTF3-AS1 | -2.164236476 | 0.001391295 | 0.00786159 | down |
| AMD1P4 | -2.16419665 | 0.312789275 | | down |
| ISPD-AS1 | -2.164129875 | 0.109326287 | 0.215589336 | down |
| PNLDC1 | -2.164108695 | 7.40E-06 | 0.000109973 | down |
| RP11-62C7.2 | -2.163829401 | 0.007442614 | 0.029321518 | down |
| SNORA25 | -2.163808316 | 0.157998154 | | down |
| AF277315.13 | -2.163803781 | 0.174503046 | 0.298937707 | down |
| RP11-534P6.1 | -2.163475197 | 0.458809346 | | down |
| NA | -2.163422684 | 0.006617614 | 0.026842123 | down |
| RP11-95C14.1 | -2.163278481 | 0.359040611 | | down |
| SIX3 | -2.163035704 | 0.014680731 | 0.049125515 | down |
| RP1-32I10.11 | -2.162938891 | 0.458921291 | | down |
| RP11-472K22.2 | -2.162929591 | 0.231273701 | | down |
| CTB-176F20.3 | -2.16286333 | 6.17E-10 | 3.37E-08 | down |
| CTC-265F19.3 | -2.162308758 | 0.011889165 | 0.041895154 | down |
| CRYBB3 | -2.162236753 | 7.25E-07 | 1.55E-05 | down |
| ACTL6B | -2.162141782 | 0.00038571 | 0.002839566 | down |
| ANKS4B | -2.162016506 | 1.65E-10 | 1.05E-08 | down |
| OR7A15P | -2.161823991 | 0.163595416 | 0.285842436 | down |
| AP000235.3 | -2.161810251 | 0.357852001 | | down |
| RP1-272E8.2 | -2.161758998 | 0.459167627 | | down |
| MIR574 | -2.161704167 | 0.030954906 | 0.085749988 | down |
| CTC-490G23.4 | -2.161622821 | 0.075783027 | | down |
| RP11-2017.2 | -2.161247078 | 0.052994724 | 0.12745545 | down |
| MYOM3 | -2.160957959 | 1.40E-07 | 3.80E-06 | down |
| RP11-49I11.2 | -2.160645215 | 0.418135717 | | down |
| DSCAS | -2.160242514 | 0.001212605 | 0.00707545 | down |
| KCNMB3P1 | -2.159962016 | 0.267195615 | | down |
| CTB-160022.1 | -2.159599188 | 0.029819895 | 0.083394669 | down |
| LINC00351 | -2.159451998 | 0.459649492 | | down |
| RP3-399L15.3 | -2.158976282 | 4.52E-07 | 1.03E-05 | down |
| SNORA77 | -2.158972667 | 0.001506837 | 0.008367809 | down |
| MOGAT3 | -2.158812801 | 1.74E-08 | 6.36E-07 | down |
| LINC00471 | -2.158679251 | 3.22E-05 | 0.000371361 | down |

| | | | | |
|---|---|---|---|---|
| ANKRD30A | -2.15862432 | 0.059984078 | 0.13966997 | down |
| CTD-2083E4.4 | -2.158378196 | 1.51E-05 | 0.000199735 | down |
| RP11-21L1.1 | -2.15817007 | 0.223533451 | | down |
| NTSR1 | -2.158088422 | 3.16E-05 | 0.000365751 | down |
| CELA3A | -2.15808419 | 0.207141302 | 0.337236007 | down |
| HERC2P10 | -2.157951119 | 0.003056315 | 0.014704934 | down |
| NA | -2.157783943 | 0.106861176 | 0.212000605 | down |
| RP3-510L9.1 | -2.157613913 | 0.013111263 | 0.04502398 | down |
| TMEM212-AS1 | -2.157595542 | 0.459961761 | | down |
| RP4-591L5.1 | -2.157554474 | 0.460046041 | | down |
| NA | -2.157521999 | 0.000131186 | 0.001183943 | down |
| CYP4F3 | -2.157423199 | 1.81E-05 | 0.000230505 | down |
| Y_RNA | -2.157347473 | 0.30972049 | | down |
| SLC5A7 | -2.15702992 | 0.000451613 | 0.003207626 | down |
| GS1-393G12.14 | -2.156933318 | 0.000556365 | 0.003794649 | down |
| CTC-559E9.13 | -2.156900123 | 0.235497022 | | down |
| RP11-537P22.2 | -2.156770496 | 0.032995773 | 0.089981393 | down |
| RP11-245G13.2 | -2.156642418 | 0.000283593 | 0.002228103 | down |
| GSTA6P | -2.156458338 | 0.45993142 | | down |
| RP11-338L22.2 | -2.155965939 | 0.096777821 | 0.197503906 | down |
| RP11-22P4.2 | -2.155601155 | 0.363194327 | | down |
| Metazoa_SRP | -2.155249551 | 0.312174212 | | down |
| RP11-332J15.2 | -2.155217878 | 0.361666641 | | down |
| NA | -2.155032147 | 0.363195074 | | down |
| CYP4F23P | -2.154764317 | 0.002083926 | 0.010837712 | down |
| AC116035.1 | -2.154563729 | 0.004982794 | 0.021633392 | down |
| CTD-2383M3.1 | -2.154522609 | 0.002663398 | 0.013206655 | down |
| RPL18AP15 | -2.154494244 | 0.418135715 | | down |
| AC112220.2 | -2.154356069 | 0.274405868 | 0.410780291 | down |
| SGO1P2 | -2.153486197 | 0.460748859 | | down |
| AADACL2-AS1 | -2.153152987 | 0.009073926 | 0.034043088 | down |
| CTD-2561F5.1 | -2.152936642 | 0.461011881 | | down |
| RPL21P71 | -2.152858697 | 0.249744547 | 0.384306637 | down |
| NA | -2.152806288 | 0.024154484 | 0.07146833 | down |
| PRR30 | -2.152644406 | 0.461073041 | | down |
| RP11-420J11.2 | -2.152001688 | 0.277437591 | 0.413602689 | down |
| ATP8B5P | -2.151400704 | 3.02E-05 | 0.000352614 | down |
| ZNF101P1 | -2.151185958 | 0.079396968 | 0.171205233 | down |
| FXYD3 | -2.151176429 | 1.76E-07 | 4.64E-06 | down |
| DHRS7C | -2.150952654 | 0.000408076 | 0.002953564 | down |
| NA | -2.150454277 | 0.08667496 | 0.182613078 | down |
| SUN3 | -2.150311084 | 0.006465529 | 0.026396298 | down |
| KRTAP4-9 | -2.150164955 | 0.267085897 | | down |
| RP11-9802.1 | -2.150150347 | 0.46113469 | | down |
| RP11-94C24.8 | -2.150146819 | 0.000494046 | 0.003442284 | down |
| PCAT18 | -2.150093956 | 0.013222868 | 0.045288171 | down |
| SLC25A1P3 | -2.150068613 | 0.461612309 | | down |
| ACSF2 | -2.149816555 | 3.08E-16 | 8.05E-14 | down |
| RP11-241I20.4 | -2.149736802 | 0.461681803 | | down |
| NA | -2.149425882 | 0.191557851 | 0.319336578 | down |
| AC008697.1 | -2.149387908 | 2.34E-05 | 0.000284864 | down |

| | | | | |
|---|---|---|---|---|
| RP11-700H6.1 | -2.14906252 | 0.000232785 | 0.001895336 | down |
| ETF1P2 | -2.148820531 | 0.018405501 | 0.058361465 | down |
| PNPLA1 | -2.148509042 | 3.17E-05 | 0.000366908 | down |
| CYP2B7P | -2.147887606 | 1.17E-07 | 3.25E-06 | down |
| RP11-214O1.1 | -2.147706457 | 0.114910745 | 0.223122758 | down |
| RP11-718O11.1 | -2.147671261 | 0.110038264 | 0.216445567 | down |
| VTA1P1 | -2.147578605 | 0.461985514 | | down |
| TMEM72 | -2.147554585 | 5.56E-05 | 0.000583034 | down |
| MED15P5 | -2.147363925 | 0.068591661 | 0.154030037 | down |
| CES2 | -2.14714662 | 5.30E-14 | 8.24E-12 | down |
| RP11-143E21.3 | -2.146968178 | 0.462185802 | | down |
| RP11-81M19.3 | -2.146782594 | 0.313591173 | | down |
| NA | -2.146771169 | 0.015737728 | 0.051805141 | down |
| ABCC6 | -2.14636151 | 2.11E-14 | 3.55E-12 | down |
| RP11-739N10.1 | -2.146346321 | 0.029106079 | 0.082124629 | down |
| ZMYND19P1 | -2.146226319 | 0.17319102 | 0.297636052 | down |
| MESP1 | -2.145508955 | 3.49E-08 | 1.15E-06 | down |
| LINC00870 | -2.145421875 | 0.000105795 | 0.000991733 | down |
| RP11-820K3.2 | -2.145409087 | 0.065559452 | 0.148912691 | down |
| CTD-2620I22.2 | -2.145376421 | 0.153384999 | 0.273048251 | down |
| PKIB | -2.145307386 | 5.93E-09 | 2.47E-07 | down |
| MIR1268B | -2.145269073 | 0.41756473 | | down |
| RP11-600G3.1 | -2.145113595 | 0.275023772 | | down |
| NA | -2.145095751 | 0.15832068 | 0.279322714 | down |
| KRTAP4-12 | -2.144958173 | 0.367237567 | | down |
| RP11-328C8.5 | -2.14469131 | 0.074877975 | 0.164146945 | down |
| SUSD2 | -2.143601348 | 3.60E-09 | 1.59E-07 | down |
| HS3ST6 | -2.143598358 | 0.000525702 | 0.003622524 | down |
| TMEM150B | -2.143536745 | 2.63E-11 | 2.12E-09 | down |
| U52111.14 | -2.143336021 | 0.000123532 | 0.001129798 | down |
| RP4-604K5.3 | -2.142879016 | 0.00892296 | 0.033611009 | down |
| Y_RNA | -2.14270743 | 0.462871863 | | down |
| RP11-293E1.1 | -2.142420961 | 0.365713454 | | down |
| RP11-708H21.1 | -2.142070104 | 0.189822117 | | down |
| OVCH2 | -2.141733848 | 0.042258707 | 0.108301681 | down |
| RP11-152L7.2 | -2.141422536 | 0.322401987 | 0.4611166 | down |
| RN7SL385P | -2.141386952 | 0.223663604 | | down |
| TENM2 | -2.141370555 | 1.77E-05 | 0.000226713 | down |
| CCDC190 | -2.141315108 | 0.011821808 | 0.041716514 | down |
| MTRNR2L6 | -2.1412721 | 0.00033881 | 0.002573105 | down |
| NA | -2.141100233 | 0.463343769 | | down |
| MPRIP-AS1 | -2.140538467 | 0.074900255 | 0.164165826 | down |
| RP11-641C17.4 | -2.140494721 | 0.425400053 | | down |
| NA | -2.140434095 | 0.463483557 | | down |
| NA | -2.140216582 | 0.017195957 | 0.055296515 | down |
| GLB1L2 | -2.140203533 | 9.02E-09 | 3.61E-07 | down |
| LINC00864 | -2.140051093 | 0.038554073 | 0.101074918 | down |
| NA | -2.139811973 | 0.04227046 | 0.108322561 | down |
| DCST1 | -2.139794404 | 2.39E-06 | 4.29E-05 | down |
| SOX6 | -2.139760651 | 6.59E-09 | 2.72E-07 | down |
| NA | -2.139662968 | 0.152088339 | | down |

| | | | | |
|---|---|---|---|---|
| AP000275.65 | -2.13954484 | 5.21E-05 | 0.000553534 | down |
| RP11-336N8.2 | -2.139455677 | 0.234086214 | | down |
| CLEC18A | -2.139437639 | 3.80E-07 | 8.86E-06 | down |
| PRRX2-AS1 | -2.139404306 | 0.236445602 | | down |
| SPATA31C2 | -2.139379567 | 0.000576545 | 0.003903928 | down |
| RP1-267L14.3 | -2.139350723 | 0.154939667 | 0.275004866 | down |
| RP5-1069C8.2 | -2.139192162 | 0.209307512 | 0.33954832 | down |
| PSG3 | -2.138743529 | 0.237028758 | | down |
| SVOPL | -2.138550003 | 4.68E-05 | 0.000506637 | down |
| LINC00501 | -2.138502491 | 2.22E-06 | 4.05E-05 | down |
| LA16c-444G7.1 | -2.13832761 | 0.165829666 | | down |
| RP11-494M8.4 | -2.138064493 | 0.464130108 | | down |
| ERAS | -2.138012542 | 0.018970517 | 0.059643179 | down |
| NA | -2.137982405 | 0.006664237 | 0.027005709 | down |
| GAL3ST1 | -2.137667494 | 7.47E-09 | 3.06E-07 | down |
| RNA5SP186 | -2.137323148 | 0.364129956 | | down |
| MIR365B | -2.13660564 | 0.280192889 | 0.416490954 | down |
| DIO2-AS1 | -2.136307167 | 0.464280861 | | down |
| TRPM3 | -2.13617606 | 0.000140559 | 0.001253476 | down |
| NA | -2.135905616 | 0.027991483 | | down |
| RP11-329J18.5 | -2.135870194 | 0.272153186 | | down |
| CCNB3 | -2.135579768 | 0.000631275 | 0.004203465 | down |
| RNU6-593P | -2.13542003 | 0.195558465 | 0.324068313 | down |
| GOLGA6C | -2.135358019 | 0.101063488 | 0.203536094 | down |
| NEU4 | -2.135052478 | 5.18E-10 | 2.90E-08 | down |
| MTRNR2L5 | -2.134589819 | 0.464512964 | | down |
| SHBG | -2.133407868 | 8.24E-07 | 1.72E-05 | down |
| ATP11A-AS1 | -2.133319203 | 0.071746287 | 0.159238399 | down |
| PCDH8P1 | -2.133306805 | 0.279015802 | | down |
| NTNG1 | -2.133118392 | 2.03E-06 | 3.75E-05 | down |
| RP11-715F10.6 | -2.133098295 | 0.425115311 | | down |
| MIRLET7DHG | -2.13299009 | 0.001119486 | 0.006641754 | down |
| C2orf73 | -2.132884168 | 0.08055167 | 0.173074045 | down |
| CTAGE10P | -2.132809973 | 0.426826272 | | down |
| RP11-89M22.3 | -2.13267261 | 0.420373232 | | down |
| LINC00504 | -2.13264158 | 0.005337045 | 0.022805601 | down |
| AP000619.5 | -2.132491382 | 0.051762725 | 0.125355419 | down |
| RP11-397E7.4 | -2.132465668 | 1.45E-08 | 5.51E-07 | down |
| CASC20 | -2.132207115 | 0.193573447 | | down |
| RP11-270M14.1 | -2.132186307 | 0.117750687 | | down |
| TCEB3B | -2.131972476 | 0.465410779 | | down |
| GAPDHP57 | -2.131760565 | 0.465455362 | | down |
| RP11-346C16.1 | -2.131070116 | 0.276113068 | | down |
| RP11-507B12.2 | -2.131057421 | 0.274888142 | | down |
| LINC00403 | -2.131028962 | 0.002280314 | 0.011684128 | down |
| RP11-1110F20.1 | -2.131021671 | 0.368581551 | | down |
| RP11-5023.2 | -2.130902831 | 0.074242872 | | down |
| RBM20 | -2.130016208 | 3.27E-07 | 7.82E-06 | down |
| DAPK1-IT1 | -2.129982797 | 0.046233112 | 0.115377324 | down |
| MS4A13 | -2.129886521 | 0.23800932 | | down |
| CRIP1P3 | -2.129710506 | 0.317029592 | | down |

| | | | | |
|---|---|---|---|---|
| MFSD2B | -2.129691762 | 2.17E-05 | 0.000268155 | down |
| DLGAP2 | -2.129590776 | 5.34E-05 | 0.000563391 | down |
| LINC00111 | -2.129248922 | 0.112580983 | 0.219777977 | down |
| PTPRT | -2.129193144 | 8.86E-07 | 1.83E-05 | down |
| PRAMEF14 | -2.129085166 | 0.465799221 | | down |
| RP11-64B16.5 | -2.129058329 | 0.46587435 | | down |
| RP11-722P11.4 | -2.128932913 | 0.371290048 | | down |
| C11orf40 | -2.128897416 | 0.365465398 | | down |
| CTD-2139B15.5 | -2.12860572 | 0.171670689 | 0.295806044 | down |
| VIL1 | -2.128223111 | 1.68E-09 | 8.09E-08 | down |
| AC000120.7 | -2.12782404 | 0.00155525 | 0.008583014 | down |
| RP11-35J6.1 | -2.127803526 | 0.466288305 | | down |
| RP11-744K17.8 | -2.127803526 | 0.466288305 | | down |
| ALS2CR11 | -2.127382151 | 0.000707206 | 0.004600973 | down |
| NA | -2.127287613 | 0.370241867 | | down |
| RN7SKP109 | -2.127130999 | 0.292986812 | 0.43027157 | down |
| MRPL35P4 | -2.126953899 | 0.368789073 | 0.507182043 | down |
| SLC34A1 | -2.12678836 | 0.001631424 | 0.008923083 | down |
| SEC13P1 | -2.126493352 | 0.273399203 | | down |
| AC005786.6 | -2.125969436 | 0.319419875 | | down |
| LINC01099 | -2.125449398 | 0.269188538 | | down |
| RP11-886D15.1 | -2.125229877 | 0.05119892 | 0.124468063 | down |
| RP11-421P23.1 | -2.125084187 | 0.46678433 | | down |
| HNRNPA1P25 | -2.125036434 | 0.373399626 | | down |
| SSX6 | -2.124951174 | 0.32004452 | | down |
| RP11-354M1.2 | -2.124791995 | 0.194641369 | | down |
| CTSLP2 | -2.123956231 | 0.003478585 | 0.01629668 | down |
| RN7SL864P | -2.12389165 | 0.46711255 | | down |
| NA | -2.12375902 | 0.3693734 | | down |
| RN7SL607P | -2.123721145 | 0.182627928 | 0.308355258 | down |
| C12orf40 | -2.122982144 | 0.139171353 | | down |
| MST1L | -2.122979175 | 4.06E-08 | 1.31E-06 | down |
| RNA5SP86 | -2.12292968 | 0.235606552 | 0.368502434 | down |
| RHOXF2 | -2.122674776 | 0.052988217 | 0.127450004 | down |
| RP13-259N13.2 | -2.122540773 | 0.42841172 | | down |
| A1CF | -2.122328659 | 3.83E-10 | 2.23E-08 | down |
| FAM153B | -2.12226382 | 0.013061125 | 0.04490313 | down |
| RP11-616K22.2 | -2.122188056 | 0.127668146 | 0.240164159 | down |
| NA | -2.122084638 | 0.040294771 | | down |
| SCN1A | -2.121482761 | 0.02631523 | 0.076130455 | down |
| UNC80 | -2.121468609 | 0.000271845 | 0.002153831 | down |
| RP11-883G10.1 | -2.121423585 | 0.182539116 | 0.308222628 | down |
| DUSP21 | -2.121205212 | 0.146852107 | | down |
| A2ML1-AS2 | -2.12082743 | 0.273596818 | | down |
| CBX3P5 | -2.12070428 | 0.467784733 | | down |
| Y_RNA | -2.120595862 | 0.325090123 | 0.463550435 | down |
| RNASE10 | -2.120498235 | 0.020564554 | 0.063381357 | down |
| NA | -2.120378353 | 0.280589531 | | down |
| CTD-3253I12.1 | -2.120292112 | 0.430115693 | | down |
| CBX3P4 | -2.119730831 | 0.009724476 | 0.035856917 | down |
| GXYLT1P4 | -2.119701723 | 0.320162226 | | down |

| | | | | |
|---|---|---|---|---|
| C13-47656900H15 | -2.119276346 | 0.323198602 | | down |
| RPS6P20 | -2.119230414 | 0.14460314 | 0.262173486 | down |
| AC007163.6 | -2.119219533 | 0.181898361 | 0.307417131 | down |
| RP11-344B5.3 | -2.118974605 | 0.031540535 | 0.087015188 | down |
| RP11-109N23.5 | -2.118677771 | 0.149652725 | 0.268614675 | down |
| RP11-44F14.5 | -2.118619619 | 0.089473663 | 0.186780623 | down |
| CTB-43E15.1 | -2.118206935 | 0.243860666 | 0.377871465 | down |
| NA | -2.117985702 | 0.468358474 | | down |
| Metazoa_SRP | -2.117895338 | 0.325751041 | | down |
| LINC00824 | -2.117869891 | 0.004870806 | 0.021257772 | down |
| RP11-297C4.3 | -2.117858034 | 0.004258646 | 0.019153708 | down |
| BTG4 | -2.117590722 | 0.137693922 | | down |
| NA | -2.11698461 | 0.468492693 | | down |
| RP11-389G6.3 | -2.116691747 | 0.064864602 | 0.147767602 | down |
| SERPINB11 | -2.116492695 | 0.468523118 | | down |
| Y_RNA | -2.116491653 | 0.468206118 | | down |
| RPSAP21 | -2.116473502 | 0.281386411 | | down |
| RP11-648O15.2 | -2.116414404 | 0.155018972 | 0.275104978 | down |
| RP11-395E19.6 | -2.116367976 | 0.132576781 | | down |
| EDN3 | -2.116331754 | 1.95E-09 | 9.26E-08 | down |
| NA | -2.116272482 | 0.208148453 | | down |
| RP11-36D19.9 | -2.115727339 | 0.427427339 | | down |
| RP11-725G5.2 | -2.115230691 | 8.56E-05 | 0.000830689 | down |
| NA | -2.115159466 | 0.00356398 | 0.016622143 | down |
| CTD-2363C16.1 | -2.11497569 | 0.009055988 | 0.03398852 | down |
| NA | -2.114732719 | 0.000201708 | 0.001681964 | down |
| RP11-405F3.5 | -2.114575268 | 0.08449192 | 0.179169625 | down |
| RP11-192H23.4 | -2.114440513 | 0.043783778 | 0.111082574 | down |
| CNBD2 | -2.114172508 | 0.000153046 | 0.001341693 | down |
| RP11-504A18.1 | -2.114080388 | 0.001900275 | 0.010061771 | down |
| DNAH5 | -2.113880701 | 5.63E-05 | 0.000589478 | down |
| RP11-472I20.3 | -2.113876566 | 0.183103255 | 0.3089025 | down |
| RP11-15M15.2 | -2.113740249 | 0.009280153 | 0.03463945 | down |
| RP11-222A5.1 | -2.113627798 | 0.46920172 | | down |
| NA | -2.113546329 | 0.025474303 | 0.074291394 | down |
| CSNK1G2P1 | -2.113029751 | 0.031689452 | 0.087306546 | down |
| RP11-807H22.6 | -2.112895804 | 0.00572321 | 0.024058784 | down |
| EEF1A1P31 | -2.112755926 | 0.155277662 | 0.275393275 | down |
| PRDX3P2 | -2.112335382 | 0.117224587 | 0.226299896 | down |
| RARRES2P2 | -2.112127439 | 0.13078724 | | down |
| PSMC1P4 | -2.111788001 | 0.113111696 | 0.220527435 | down |
| RPS18P1 | -2.111719932 | 0.279262954 | | down |
| RHEBP3 | -2.111716033 | 0.135020837 | 0.249727039 | down |
| CACFD1 | -2.11141105 | 5.67E-11 | 4.17E-09 | down |
| NA | -2.111011382 | 0.469832136 | | down |
| CTD-2270L9.2 | -2.110270693 | 0.009156536 | 0.034286786 | down |
| SLC27A4 | -2.110065716 | 1.87E-12 | 1.93E-10 | down |
| RP11-391H12.8 | -2.110045781 | 0.017667322 | 0.056582137 | down |
| RP11-69C17.3 | -2.10967889 | 0.470113979 | | down |
| MESTIT1 | -2.109551558 | 0.075176493 | 0.164585189 | down |
| MIR5707 | -2.109549485 | 0.170192079 | | down |

| | | | | |
|---|---|---|---|---|
| RP11-619A14.2 | -2.10946026 | 0.115766021 | | down |
| CCDC166 | -2.109088657 | 0.470238852 | | down |
| ZAN | -2.109048093 | 0.021814852 | 0.066195325 | down |
| IGFL3 | -2.108942749 | 0.433587785 | | down |
| FNDC7 | -2.108832176 | 0.063990567 | | down |
| RP11-543H23.1 | -2.108785464 | 0.208996762 | | down |
| RP11-793H4.1 | -2.108578913 | 0.323857987 | | down |
| CTD-2535P7.1 | -2.10849793 | 0.156157546 | 0.276496778 | down |
| AC012065.4 | -2.108181784 | 0.470279561 | | down |
| TSGA13 | -2.108075767 | 0.43138788 | | down |
| MED28P4 | -2.107699551 | 0.078905821 | 0.170452006 | down |
| CES5AP1 | -2.107521999 | 0.174688911 | 0.299085573 | down |
| RN7SL118P | -2.107461699 | 0.037096637 | 0.098170647 | down |
| RN7SL660P | -2.107101123 | 0.431535658 | | down |
| CTB-46B19.2 | -2.107056268 | 0.006448712 | 0.026352706 | down |
| RNU2-33P | -2.106653409 | 0.15924469 | 0.280417253 | down |
| RP11-259G18.2 | -2.106166021 | 0.007260119 | 0.028745784 | down |
| NA | -2.106039248 | 0.157292587 | 0.278014972 | down |
| CTC-278L1.1 | -2.106019532 | 0.277309774 | | down |
| LINC00159 | -2.105990569 | 0.029536727 | 0.082911468 | down |
| FEM1AP4 | -2.105970425 | 0.434121666 | | down |
| NA | -2.105950753 | 0.373655522 | | down |
| KRT18P50 | -2.105777681 | 0.470939679 | | down |
| RP11-713N11.4 | -2.105665434 | 0.07283675 | | down |
| RP11-181B11.2 | -2.105417016 | 0.37292399 | | down |
| PCK2 | -2.105390637 | 7.74E-15 | 1.48E-12 | down |
| RP11-146E13.4 | -2.105250425 | 0.013660806 | 0.046475513 | down |
| RP11-681B3.4 | -2.104905977 | 0.003929856 | 0.017977287 | down |
| AC005514.2 | -2.104714485 | 0.118263051 | 0.227704121 | down |
| NA | -2.104672066 | 0.470648609 | | down |
| AC008937.2 | -2.104406251 | 0.118219514 | | down |
| FAAH | -2.104188937 | 2.69E-10 | 1.62E-08 | down |
| RP11-386G11.5 | -2.103962398 | 0.005577672 | 0.023588904 | down |
| TBX18-AS1 | -2.103850954 | 0.106793771 | 0.211906155 | down |
| NA | -2.10383996 | 0.471350098 | | down |
| NA | -2.103670935 | 0.285915416 | | down |
| RNU2-58P | -2.103557733 | 0.435812396 | | down |
| RN7SL246P | -2.103030706 | 0.43445468 | | down |
| OFD1P17 | -2.102911243 | 0.175256155 | | down |
| ENPP7P10 | -2.102908669 | 0.063169203 | | down |
| CTA-212A2.2 | -2.102679566 | 0.054892153 | 0.130991251 | down |
| POU4F2 | -2.102596406 | 0.377693918 | | down |
| RP11-148B3.2 | -2.102591487 | 0.065888708 | 0.149468465 | down |
| FAM136BP | -2.102515654 | 0.471630706 | | down |
| U73166.2 | -2.102394494 | 3.28E-07 | 7.82E-06 | down |
| RP11-749H20.3 | -2.102160037 | 0.370883864 | | down |
| CTA-992D9.7 | -2.102079715 | 0.471501129 | | down |
| TFAP2D | -2.101771066 | 0.032465453 | 0.088894179 | down |
| LINC00221 | -2.101730738 | 0.006878496 | 0.027628014 | down |
| RP11-61A14.1 | -2.101440767 | 2.22E-05 | 0.000271795 | down |
| NAA11 | -2.101375715 | 0.326653824 | | down |

| | | | | |
|---|---|---|---|---|
| RP11-692C24.1 | -2.101363226 | 0.120312144 | 0.230562004 | down |
| NA | -2.101021667 | 0.127311004 | 0.239657381 | down |
| RP11-35G9.5 | -2.101013097 | 1.81E-08 | 6.53E-07 | down |
| NA | -2.100766175 | 0.001759058 | 0.009474567 | down |
| AP000705.7 | -2.100727214 | 0.153498853 | 0.273173768 | down |
| SCGB3A2 | -2.100619097 | 0.244165112 | | down |
| ADAM3B | -2.099848234 | 0.328884025 | | down |
| RP11-208K4.1 | -2.099543597 | 0.472260789 | | down |
| PCDHA11 | -2.099455484 | 0.049982426 | 0.122285995 | down |
| CTD-2015G9.2 | -2.099376202 | 0.053496836 | 0.128365062 | down |
| CYP3A5 | -2.099251137 | 1.10E-09 | 5.53E-08 | down |
| FAM169B | -2.099126597 | 0.000331519 | 0.002529245 | down |
| AP000439.2 | -2.099056555 | 0.046879149 | | down |
| RP11-489P6.1 | -2.098869008 | 0.270813126 | 0.406900461 | down |
| FAM9C | -2.098399474 | 0.040077406 | 0.104105618 | down |
| RP11-715G15.1 | -2.098372039 | 0.472509289 | | down |
| RNU2-59P | -2.098344775 | 0.052623077 | 0.126825583 | down |
| C12orf77 | -2.098231442 | 0.002421118 | 0.012260249 | down |
| uc_338 | -2.098128929 | 0.375590665 | | down |
| RP11-881M11.4 | -2.098060781 | 0.063360293 | 0.145490419 | down |
| NA | -2.097848258 | 0.472542577 | | down |
| FCF1P1 | -2.097616557 | 0.1289929 | 0.24194423 | down |
| RP11-70F11.11 | -2.09756735 | 0.427440376 | | down |
| NA | -2.097380095 | 0.374709603 | | down |
| RNU6-389P | -2.097324093 | 0.373747591 | | down |
| CTD-2218G20.2 | -2.097295163 | 0.472737768 | | down |
| CTNNAP1 | -2.097103698 | 6.05E-05 | 0.000623651 | down |
| RP11-361H10.5 | -2.097047965 | 0.178373058 | | down |
| AF067845.2 | -2.097039741 | 0.107321793 | 0.212591544 | down |
| ZIM2 | -2.096816115 | 0.281949031 | | down |
| LINC00598 | -2.096417849 | 6.81E-07 | 1.47E-05 | down |
| NA | -2.096349921 | 0.47271582 | | down |
| RP11-391L3.3 | -2.096229202 | 0.00244258 | 0.01233359 | down |
| EIF3FP1 | -2.096125265 | 0.472834027 | | down |
| Y_RNA | -2.095720035 | 0.232208536 | | down |
| RP1-191L6.2 | -2.095690998 | 0.160434263 | 0.281843468 | down |
| RP11-20024.1 | -2.095473731 | 0.000313333 | 0.002416268 | down |
| CDH10 | -2.095397693 | 0.008850932 | 0.033410906 | down |
| NA | -2.094981971 | 0.281150744 | | down |
| GFRA4 | -2.094799533 | 0.144838571 | 0.262393272 | down |
| RP11-3L10.3 | -2.094452057 | 0.016173735 | | down |
| RNU6-644P | -2.09437818 | 0.133353155 | | down |
| NA | -2.094074611 | 0.042886342 | 0.109462006 | down |
| SNORA8 | -2.094022985 | 0.008032412 | 0.031058658 | down |
| NR6A1 | -2.093877088 | 1.53E-15 | 3.33E-13 | down |
| RP11-353N14.4 | -2.093610295 | 4.63E-05 | 0.000501159 | down |
| KLHL31 | -2.093565598 | 4.12E-05 | 0.000454645 | down |
| ITPKA | -2.093456051 | 1.68E-12 | 1.75E-10 | down |
| NA | -2.093018786 | 0.108448512 | 0.214314746 | down |
| CUX2 | -2.092643229 | 5.30E-05 | 0.000560533 | down |
| SEMA3E | -2.092373221 | 8.99E-07 | 1.85E-05 | down |

| | | | | |
|---|---|---|---|---|
| RP11-205K6.3 | -2.092349518 | 0.058421823 | 0.137116261 | down |
| KB-1410C5.2 | -2.092284624 | 0.199570293 | | down |
| LINC00366 | -2.09224652 | 0.268742743 | 0.404554914 | down |
| CTA-31J9.2 | -2.092217773 | 0.376936029 | | down |
| CTD-2036A2.1 | -2.092060338 | 0.122801341 | 0.233730978 | down |
| RP11-548K12.12 | -2.091817466 | 0.23930822 | | down |
| ITGB5-AS1 | -2.091748187 | 0.473915623 | | down |
| NA | -2.091630155 | 0.473940704 | | down |
| C8orf86 | -2.091405064 | 0.114305875 | 0.222206775 | down |
| RNU6-577P | -2.091397033 | 0.168210694 | | down |
| BHMG1 | -2.09126761 | 0.08948883 | 0.186799306 | down |
| RP11-361M10.3 | -2.090948198 | 2.08E-05 | 0.000259091 | down |
| C11orf97 | -2.090903269 | 0.2404613 | | down |
| PRSS2 | -2.09076833 | 0.001533589 | 0.008485294 | down |
| YTHDF1P1 | -2.090751254 | 0.474127483 | | down |
| BMP5 | -2.09048253 | 1.57E-08 | 5.87E-07 | down |
| NA | -2.090302297 | 0.038749707 | 0.101428489 | down |
| NA | -2.089933026 | 0.057386183 | 0.135213747 | down |
| XXbac-B444P24.8 | -2.089910206 | 0.001539687 | 0.008514331 | down |
| CTB-1202.1 | -2.089606384 | 0.003216669 | 0.015291393 | down |
| OR5BD1P | -2.089222696 | 0.110554656 | 0.217162738 | down |
| CTD-2593A12.3 | -2.089187422 | 0.280057974 | 0.416372812 | down |
| RPS6KA6 | -2.089064997 | 2.35E-13 | 3.09E-11 | down |
| NA | -2.088983329 | 0.013937084 | 0.047238915 | down |
| RP11-872D17.8 | -2.088851865 | 0.238054937 | | down |
| ABHD11-AS1 | -2.088706646 | 4.57E-07 | 1.04E-05 | down |
| RP11-331F4.5 | -2.08863879 | 0.000102928 | 0.000969695 | down |
| LINC01298 | -2.088596286 | 0.379133901 | | down |
| LINC00559 | -2.088483353 | 0.214277728 | 0.3451697 | down |
| C2orf27AP2 | -2.088262462 | 0.474578434 | | down |
| ELFN2 | -2.087986351 | 0.088320021 | | down |
| RN7SKP281 | -2.087985021 | 0.474715612 | | down |
| RP11-351I21.7 | -2.087664244 | 0.474783838 | | down |
| ARPP21 | -2.087285704 | 0.006487575 | 0.026454381 | down |
| RP11-417O18.1 | -2.087261249 | 0.381760747 | | down |
| RP11-312J18.7 | -2.086988633 | 0.070927439 | 0.157777579 | down |
| CTD-2547L16.3 | -2.086894099 | 0.183348912 | | down |
| NA | -2.086884688 | 0.006147991 | 0.025424672 | down |
| NA | -2.086697928 | 8.31E-09 | 3.36E-07 | down |
| RP11-169E6.3 | -2.086619404 | 0.475006103 | | down |
| KRT8P51 | -2.086456288 | 0.072062605 | 0.159676148 | down |
| RP11-212E8.1 | -2.086258539 | 0.330848746 | | down |
| RP11-1085N6.1 | -2.086250501 | 0.43199571 | | down |
| BCAR4 | -2.08624024 | 0.061474706 | 0.142372412 | down |
| MIR6129 | -2.086142034 | 0.210884619 | | down |
| HTR4 | -2.085918664 | 7.69E-05 | 0.000759655 | down |
| NA | -2.085808398 | 0.001692877 | 0.009187284 | down |
| RP11-94C24.13 | -2.085341141 | 4.53E-05 | 0.000492512 | down |
| ST8SIA2 | -2.08528211 | 0.000349063 | 0.002633661 | down |
| CD46P1 | -2.085176932 | 0.064648628 | 0.147456983 | down |
| PACRG-AS2 | -2.085018331 | 0.379809475 | | down |

| | | | | |
|---|---|---|---|---|
| SFN | -2.085015613 | 3.39E-06 | 5.75E-05 | down |
| FAM83C | -2.08487872 | 0.150421863 | | down |
| RNA5SP279 | -2.084735504 | 0.475407001 | | down |
| RP11-555G19.2 | -2.084566016 | 0.199621473 | | down |
| MTND4LP13 | -2.084179675 | 0.120218572 | | down |
| AC074391.1 | -2.084127516 | 0.000716881 | 0.004651831 | down |
| CRYGA | -2.083949855 | 0.248650634 | 0.383120142 | down |
| RP11-77K12.5 | -2.08372392 | 0.00045785 | 0.003245019 | down |
| RP11-184C23.1 | -2.083661537 | 0.21864933 | 0.350127944 | down |
| RP11-561023.5 | -2.083623122 | 0.00800768 | 0.030994941 | down |
| NA | -2.083602508 | 0.045838008 | 0.114705745 | down |
| SYT8 | -2.083340517 | 7.10E-06 | 0.000106206 | down |
| RP11-717D12.1 | -2.083295135 | 0.010619587 | 0.038417203 | down |
| NA | -2.083265653 | 5.47E-06 | 8.52E-05 | down |
| COL20A1 | -2.083105744 | 0.00259659 | 0.012952343 | down |
| RP4-697K14.3 | -2.082537545 | 0.24612636 | | down |
| RP11-191L9.4 | -2.082316844 | 0.01161148 | 0.041158013 | down |
| OR6L2P | -2.082309609 | 0.174897357 | | down |
| RP4-533D7.3 | -2.08209115 | 0.059820791 | 0.13937618 | down |
| RP11-1E4.1 | -2.081525825 | 0.215946796 | | down |
| AC102953.6 | -2.081374251 | 0.085104011 | 0.180222071 | down |
| RP11-317024.1 | -2.081258779 | 0.38054699 | | down |
| NOX3 | -2.081136351 | 0.290332005 | | down |
| RN7SKP28 | -2.08082843 | 0.476239022 | | down |
| SYCE1L | -2.080733294 | 3.60E-08 | 1.18E-06 | down |
| CH17-53B9.4 | -2.08053044 | 0.476302512 | | down |
| SF3A3P2 | -2.080201353 | 0.065723348 | | down |
| ADAM29 | -2.080044289 | 0.012905913 | 0.044517937 | down |
| RP11-408E1.1 | -2.079844152 | 0.438554155 | | down |
| Y_RNA | -2.0796713 | 0.08330747 | 0.17741799 | down |
| BARX1-AS1 | -2.079594854 | 0.189514787 | | down |
| RP11-71H17.8 | -2.079549381 | 0.068019658 | | down |
| HCP5B | -2.079288349 | 3.29E-05 | 0.00037761 | down |
| AP000345.2 | -2.0792622 | 0.429386914 | | down |
| WSPAR | -2.079233879 | 0.442411218 | | down |
| RP11-325024.2 | -2.079228375 | 0.476579987 | | down |
| AC131056.5 | -2.079116773 | 0.142099098 | 0.25892924 | down |
| RP11-739P1.2 | -2.079080418 | 0.119558563 | 0.229580562 | down |
| RP11-293D9.2 | -2.078808046 | 0.327681986 | | down |
| AP001610.9 | -2.078469866 | 0.000197228 | 0.001651482 | down |
| MT1HL1 | -2.078345906 | 0.085054452 | | down |
| RP11-532F6.2 | -2.078334578 | 0.38224529 | | down |
| RP11-538P18.2 | -2.078077236 | 0.38259879 | | down |
| RP11-369K16.1 | -2.078042687 | 1.34E-05 | 0.000180449 | down |
| CES3 | -2.077935426 | 4.92E-09 | 2.10E-07 | down |
| OR13D1 | -2.077931674 | 0.384559016 | | down |
| SNX29P2 | -2.077240769 | 0.139054528 | 0.25511445 | down |
| RP1-253B10.1 | -2.077203645 | 0.477011638 | | down |
| RIPPLY3 | -2.076930925 | 0.000282284 | 0.002221886 | down |
| LA16c-380H5.5 | -2.07681566 | 8.63E-10 | 4.47E-08 | down |
| IL31RA | -2.076409704 | 0.001896908 | 0.010052252 | down |

| | | | | |
|---|---|---|---|---|
| RP11-407A16.7 | -2.075860478 | 0.217713355 | | down |
| DPRXP2 | -2.075644285 | 0.018279125 | 0.058064923 | down |
| GOLGA8CP | -2.07556046 | 0.477362104 | | down |
| AC104667.3 | -2.075335224 | 3.23E-06 | 5.53E-05 | down |
| MBL2 | -2.074796134 | 0.380787105 | | down |
| APOH | -2.074432409 | 0.002590427 | 0.012932336 | down |
| NANOG | -2.074407287 | 0.060172728 | 0.140000732 | down |
| RP11-475O23.2 | -2.074304592 | 0.006199274 | 0.025590956 | down |
| MOBP | -2.074134439 | 0.000449101 | 0.003195827 | down |
| RP11-826F13.1 | -2.074041294 | 0.086149539 | 0.181841811 | down |
| RFPL2 | -2.074027374 | 0.009570462 | 0.035436896 | down |
| RP11-159H22.2 | -2.073974877 | 0.003436835 | 0.016141359 | down |
| PRDM12 | -2.073806151 | 0.005495318 | 0.023293078 | down |
| AFDN-AS1 | -2.073692046 | 2.44E-07 | 6.13E-06 | down |
| RP11-255G12.2 | -2.073652112 | 0.068995845 | 0.15466078 | down |
| IGLVIV-59 | -2.073084594 | 0.477890433 | | down |
| MIR604 | -2.073049803 | 0.331349809 | | down |
| OR2V2 | -2.072836968 | 0.47778966 | | down |
| NA | -2.072508399 | 0.070641188 | 0.157420551 | down |
| MIR200B | -2.072507787 | 0.00388954 | 0.01784354 | down |
| RP11-216L13.19 | -2.072246868 | 1.49E-08 | 5.63E-07 | down |
| B4GALT4-AS1 | -2.072186968 | 0.443473264 | | down |
| RP11-267M23.3 | -2.072042954 | 0.054948198 | 0.131058773 | down |
| NA | -2.071761754 | 0.209698357 | | down |
| RP1-81P11.1 | -2.071726443 | 0.478101629 | | down |
| NDST2 | -2.071661179 | 3.31E-05 | 0.000379303 | down |
| RP11-432J24.3 | -2.07154435 | 0.003311573 | 0.01565092 | down |
| HSD3BP5 | -2.070997123 | 0.053314543 | 0.128062927 | down |
| AC007389.3 | -2.070809137 | 0.436850107 | | down |
| AC093590.1 | -2.070702694 | 0.08909743 | 0.186215178 | down |
| GJA6P | -2.070527108 | 0.382748993 | | down |
| TBC1D3J | -2.070452256 | 0.478094786 | | down |
| RP11-197K6.1 | -2.070280778 | 0.187711571 | | down |
| GAPDHP72 | -2.069759445 | 0.024587486 | 0.072400999 | down |
| RP11-466G12.2 | -2.069732014 | 0.04299139 | 0.109664912 | down |
| RP11-467L24.1 | -2.069688422 | 0.176381936 | 0.301228904 | down |
| NMRK2 | -2.06950582 | 0.000350014 | 0.002639512 | down |
| NA | -2.069046678 | 0.058835699 | 0.137764768 | down |
| RP11-567J20.2 | -2.068910091 | 0.001045119 | 0.006281165 | down |
| DGAT1 | -2.068547996 | 5.45E-12 | 5.08E-10 | down |
| TGFBR3L | -2.068463514 | 3.19E-09 | 1.45E-07 | down |
| LRRC38 | -2.068306631 | 0.028750028 | 0.081364528 | down |
| NEFHP1 | -2.068281043 | 0.478837498 | | down |
| RNA5SP469 | -2.06768157 | 0.247514059 | 0.381838891 | down |
| RP3-508I15.9 | -2.06756775 | 7.85E-06 | 0.000115347 | down |
| KLKP1 | -2.067443058 | 0.065229064 | 0.148336813 | down |
| AC079354.3 | -2.067400086 | 0.005563167 | 0.023540816 | down |
| RP11-200A13.1 | -2.067387051 | 0.231874949 | 0.364582949 | down |
| RP11-983G14.1 | -2.067137083 | 0.479081961 | | down |
| MTND2P28 | -2.066784232 | 6.94E-05 | 0.00069664 | down |
| LINC00930 | -2.066676639 | 2.10E-05 | 0.000261546 | down |

| | | | | |
|---|---|---|---|---|
| RP11-180C1.1 | -2.066544783 | 0.125161799 | 0.236843758 | down |
| CTB-152G17.6 | -2.066409068 | 3.49E-06 | 5.90E-05 | down |
| NA | -2.066312135 | 0.096072464 | 0.196420627 | down |
| VN1R6P | -2.066306611 | 0.479338422 | | down |
| LCE1C | -2.066273133 | 0.167665009 | | down |
| ANKRD20A9P | -2.066226975 | 0.386012455 | | down |
| RP11-481K9.4 | -2.065619057 | 0.386956415 | | down |
| FAM186B | -2.065198858 | 1.77E-05 | 0.000225956 | down |
| RP11-274J16.5 | -2.065145737 | 0.030875664 | 0.085637782 | down |
| LRRC3C | -2.065120106 | 0.050581151 | 0.123319005 | down |
| CDH22 | -2.064930442 | 3.19E-06 | 5.47E-05 | down |
| APOBEC1 | -2.064916016 | 5.63E-06 | 8.75E-05 | down |
| AC034220.3 | -2.064679267 | 1.10E-15 | 2.56E-13 | down |
| Y_RNA | -2.06461837 | 0.218858955 | | down |
| TRIM51EP | -2.064600166 | 0.479624339 | | down |
| RP11-556N4.1 | -2.064446326 | 0.187742252 | | down |
| ASAH2 | -2.064415649 | 6.43E-05 | 0.000655079 | down |
| RP11-1079K10.5 | -2.06375793 | 0.439387921 | | down |
| NA | -2.063648967 | 0.174796826 | 0.299202133 | down |
| CACNA1B | -2.06354504 | 0.000193574 | 0.001627234 | down |
| RP11-150O12.5 | -2.063294575 | 0.001818869 | 0.009740837 | down |
| RP11-671J11.4 | -2.063209068 | 0.444742669 | | down |
| LINC01234 | -2.063174773 | 0.014822478 | 0.049496562 | down |
| NA | -2.062719714 | 0.303871327 | 0.441871649 | down |
| NA | -2.062685963 | 0.011813578 | 0.041692367 | down |
| MIR5695 | -2.062523695 | 0.254649682 | | down |
| RP11-862L9.3 | -2.061933038 | 0.003475176 | 0.01628325 | down |
| RP11-523J2.1 | -2.061812792 | 0.335274105 | | down |
| GPR158-AS1 | -2.061226945 | 0.215735959 | 0.346863705 | down |
| RP11-800A18.3 | -2.061137484 | 0.002487282 | 0.012518188 | down |
| RP11-143E21.2 | -2.061067551 | 0.440962883 | | down |
| RP11-572P18.2 | -2.060982136 | 0.153054957 | 0.272690691 | down |
| COX6B2 | -2.060814894 | 4.05E-06 | 6.68E-05 | down |
| SERPINI2 | -2.060387739 | 0.004758053 | 0.020893154 | down |
| BOLA2P2 | -2.060361639 | 0.01113383 | 0.039821999 | down |
| RP11-609N14.1 | -2.060265128 | 0.094863359 | 0.194698296 | down |
| MCRIP2 | -2.059992886 | 1.38E-11 | 1.17E-09 | down |
| CTD-2161F6.3 | -2.059902427 | 0.383668724 | | down |
| RP11-605F22.2 | -2.059162204 | 0.110737841 | 0.217380445 | down |
| RP11-478C6.5 | -2.059043466 | 0.08015194 | | down |
| RP11-667M19.10 | -2.058993325 | 0.068849888 | 0.154471364 | down |
| SDAD1P4 | -2.058500781 | 0.331003464 | | down |
| HHLA1 | -2.058458826 | 0.480863304 | | down |
| NA | -2.058167128 | 0.209335481 | | down |
| CTB-76P12.1 | -2.057748205 | 0.287303378 | | down |
| RP4-813D12.3 | -2.057694116 | 0.002494605 | 0.01254663 | down |
| VENTXP6 | -2.057209779 | 0.254399903 | | down |
| GTF2IRD2P1 | -2.057155068 | 0.000356163 | 0.002675813 | down |
| RP11-540O11.7 | -2.057050292 | 0.481319698 | | down |
| SNORA48 | -2.056974381 | 0.384728165 | | down |
| SLC6A10P | -2.056758002 | 0.339442883 | | down |

| | | | | |
|---|---|---|---|---|
| RNA5SP329 | -2.056452973 | 0.481447704 | | down |
| TTC39B | -2.056277541 | 2.49E-10 | 1.52E-08 | down |
| SLC1A1 | -2.056064023 | 2.73E-09 | 1.26E-07 | down |
| RP11-298I3.6 | -2.055019921 | 0.000118165 | 0.001089668 | down |
| GPR35 | -2.054845416 | 4.95E-09 | 2.11E-07 | down |
| RP11-459F6.1 | -2.054696652 | 0.48182419 | | down |
| RP11-80B9.1 | -2.05437625 | 0.481892889 | | down |
| RNU6-976P | -2.05406336 | 0.481318475 | | down |
| RP11-843F11.1 | -2.053926634 | 0.111623405 | 0.218743122 | down |
| POTEJ | -2.053684247 | 0.292721593 | | down |
| RP11-30P6.6 | -2.053658557 | 4.21E-06 | 6.90E-05 | down |
| NA | -2.053205415 | 0.337199175 | | down |
| RNU5E-4P | -2.05312035 | 0.048891357 | 0.120397868 | down |
| LINC00628 | -2.05311408 | 0.000870645 | 0.005420386 | down |
| OR6N1 | -2.053066057 | 0.387015152 | | down |
| GLT6D1 | -2.052898316 | 0.104159432 | 0.207834627 | down |
| RP4-778K6.1 | -2.052587056 | 0.253216453 | | down |
| AL590762.6 | -2.052512863 | 0.039587112 | 0.103160846 | down |
| LL22NC03-13G6.2 | -2.052487612 | 0.337356144 | | down |
| NA | -2.052418443 | 0.059475509 | 0.138819312 | down |
| CNOT6LP1 | -2.052139238 | 0.090076861 | 0.187596646 | down |
| FLVCR1 | -2.05204917 | 1.34E-16 | 3.84E-14 | down |
| SNHG23 | -2.052034754 | 6.37E-05 | 0.000650932 | down |
| LINC01531 | -2.051867075 | 0.002297433 | 0.011757395 | down |
| AC009014.3 | -2.05175732 | 2.48E-09 | 1.14E-07 | down |
| KRT8P28 | -2.051380601 | 0.441495307 | | down |
| TSPAN16 | -2.050878478 | 0.018612349 | 0.05883727 | down |
| CTD-2015H6.3 | -2.050427575 | 5.80E-07 | 1.28E-05 | down |
| RAP1BP3 | -2.050337906 | 0.482679717 | | down |
| RN7SKP185 | -2.050323018 | 0.014528619 | 0.048719372 | down |
| GNAT2 | -2.050025561 | 0.000961205 | 0.005869605 | down |
| RP11-77H9.1 | -2.049720137 | 0.15714335 | | down |
| RP11-129B9.2 | -2.04933589 | 0.482894771 | | down |
| CCDC74BP1 | -2.048878262 | 0.250556485 | | down |
| RP11-177N22.3 | -2.048733413 | 0.338496608 | | down |
| NA | -2.048670487 | 0.149943089 | | down |
| C3orf79 | -2.048543368 | 0.111021063 | 0.217836784 | down |
| LYPD4 | -2.048282906 | 0.076952446 | 0.167436218 | down |
| RP11-569G9.7 | -2.048217492 | 0.0401962 | | down |
| CASC17 | -2.047993083 | 0.34052684 | | down |
| SOX21-AS1 | -2.047988063 | 0.122332649 | 0.23314908 | down |
| RP5-881L22.5 | -2.047715643 | 6.80E-05 | 0.000685871 | down |
| RP5-88207.1 | -2.047647806 | 1.44E-05 | 0.000191536 | down |
| RP11-467I20.6 | -2.047609933 | 0.059872461 | 0.139474934 | down |
| LINC01574 | -2.047340661 | 0.013943989 | 0.047249959 | down |
| RN7SL104P | -2.047131258 | 0.483447733 | | down |
| RP4-614C10.5 | -2.047131258 | 0.483447733 | | down |
| RP11-567N4.3 | -2.047131258 | 0.483447733 | | down |
| AC073094.4 | -2.047131258 | 0.483447733 | | down |
| RP11-1082L8.2 | -2.047131258 | 0.483447733 | | down |
| HOXC13 | -2.047131258 | 0.483447733 | | down |

| | | | | |
|---|---|---|---|---|
| RP11-552E10.1 | -2.047131258 | 0.483447733 | | down |
| CGB1 | -2.047131258 | 0.483447733 | | down |
| PRSS41 | -2.047113467 | 0.002650522 | 0.013158009 | down |
| FAM135B | -2.046964145 | 6.87E-06 | 0.000103518 | down |
| RN7SL833P | -2.046783209 | 0.293948729 | | down |
| BPIFA1 | -2.046755249 | 0.483528502 | | down |
| CTC-457E21.1 | -2.046520833 | 0.02588084 | 0.075210423 | down |
| NA | -2.046485042 | 0.000505382 | 0.003505012 | down |
| Y_RNA | -2.046319256 | 0.483622165 | | down |
| RN7SKP22 | -2.046127241 | 0.206779872 | | down |
| NA | -2.045574858 | 2.00E-05 | 0.000250623 | down |
| RP4-543J13.1 | -2.045559315 | 0.483629947 | | down |
| RP11-433C9.2 | -2.045260893 | 0.025713349 | 0.074847578 | down |
| KB-1410C5.3 | -2.044682008 | 0.483973977 | | down |
| MPST | -2.044653503 | 4.29E-11 | 3.26E-09 | down |
| ANKRD18CP | -2.044641611 | 0.035609298 | 0.09521589 | down |
| RNA5SP126 | -2.044556563 | 0.484000938 | | down |
| SLC5A8 | -2.044308457 | 0.000774819 | 0.00494554 | down |
| RP11-439C15.4 | -2.044085713 | 2.12E-08 | 7.47E-07 | down |
| CD207 | -2.043953624 | 2.17E-08 | 7.62E-07 | down |
| GPR37L1 | -2.043544802 | 6.58E-08 | 2.00E-06 | down |
| KCNMB2-AS1 | -2.043464973 | 0.00280401 | 0.013760807 | down |
| NA | -2.043449476 | 6.06E-07 | 1.33E-05 | down |
| C8B | -2.043395379 | 0.394296085 | | down |
| RP11-6N17.1 | -2.043386253 | 0.10391615 | 0.207555964 | down |
| RP11-464C19.3 | -2.043239409 | 0.167436918 | | down |
| AC016768.1 | -2.04323587 | 0.021938859 | 0.066471469 | down |
| NA | -2.042827085 | 0.484372729 | | down |
| HHATL | -2.042495206 | 0.006238163 | 0.025695006 | down |
| CTD-2090I13.1 | -2.042103722 | 0.443480403 | | down |
| CIDEC | -2.042045889 | 9.88E-08 | 2.81E-06 | down |
| RPS26P8 | -2.042029002 | 0.0013959 | 0.007881785 | down |
| SEPT12 | -2.04188875 | 0.216305595 | | down |
| RP11-398G24.2 | -2.041668858 | 0.389869371 | | down |
| AC007078.4 | -2.041623773 | 0.19043084 | | down |
| RNU6-628P | -2.041542031 | 0.076201075 | 0.166198556 | down |
| RP11-161D15.2 | -2.04150266 | 0.445945838 | | down |
| LINC00907 | -2.041428996 | 0.00032715 | 0.002500992 | down |
| VIPR1 | -2.041408273 | 1.33E-15 | 2.99E-13 | down |
| FAM230B | -2.041395234 | 0.484680655 | | down |
| MAEL | -2.041229679 | 0.004636626 | 0.020467643 | down |
| RP11-119H12.1 | -2.041123077 | 0.292447508 | | down |
| RP11-293A10.3 | -2.040974813 | 0.484771088 | | down |
| RP11-168L22.2 | -2.040950615 | 0.099620929 | | down |
| FAM47DP | -2.040757216 | 0.25303657 | | down |
| NA | -2.040744157 | 0.001313474 | 0.007516571 | down |
| SNORD67 | -2.040703285 | 0.24768919 | | down |
| OPN1SW | -2.040668139 | 0.065006242 | 0.147980192 | down |
| CTD-2313J23.1 | -2.04029178 | 0.484689849 | | down |
| RP11-763F8.1 | -2.04020104 | 0.294929744 | | down |
| RP11-795H16.3 | -2.039949554 | 0.484835784 | | down |

| | | | | |
|---|---|---|---|---|
| SYT14 | -2.039929425 | 0.002517886 | 0.012645834 | down |
| RP4-744I24.4 | -2.039684226 | 0.393494972 | | down |
| NA | -2.039381262 | 0.014889147 | 0.049663277 | down |
| RP11-83A16.1 | -2.039275709 | 0.296600873 | | down |
| PSMC1P5 | -2.039017511 | 0.01412944 | 0.047734723 | down |
| CTD-2194D22.1 | -2.038950395 | 0.485206672 | | down |
| CTB-78H18.1 | -2.038950395 | 0.485206672 | | down |
| AC008060.8 | -2.038950395 | 0.485206672 | | down |
| OR8K5 | -2.038950395 | 0.485206672 | | down |
| RP11-158L12.4 | -2.038883352 | 1.66E-06 | 3.15E-05 | down |
| LYPLAL1-AS1 | -2.0388742 | 6.77E-05 | 0.000683675 | down |
| RP11-473M20.16 | -2.038871425 | 5.09E-06 | 8.05E-05 | down |
| FTMT | -2.038623812 | 0.44935829 | | down |
| CFL1P1 | -2.038575273 | 1.75E-07 | 4.61E-06 | down |
| NT5C1B | -2.038467072 | 0.039113972 | 0.102204022 | down |
| RP11-434H6.2 | -2.037971409 | 0.047023931 | | down |
| ABCC12 | -2.037906858 | 0.442500646 | | down |
| PNPLA5 | -2.03785725 | 0.449133642 | | down |
| RP11-146E13.5 | -2.037694133 | 0.118713415 | 0.228351741 | down |
| RP11-464D20.6 | -2.037669225 | 0.003356294 | 0.015815011 | down |
| RARRES2P1 | -2.037502018 | 0.057025075 | 0.134637434 | down |
| RP11-353N14.1 | -2.037330747 | 0.19066742 | | down |
| RP11-340L3.1 | -2.037263297 | 0.122939514 | 0.233949503 | down |
| RP11-223I10.1 | -2.037205492 | 3.21E-05 | 0.000369855 | down |
| MT-ND4L | -2.037060191 | 0.000168937 | 0.001454626 | down |
| KRT18P61 | -2.036959266 | 0.297772593 | | down |
| RP11-95P2.4 | -2.036921851 | 0.443582859 | | down |
| LRRC66 | -2.036907228 | 4.74E-07 | 1.07E-05 | down |
| PSG2 | -2.036880732 | 0.06667215 | 0.150790281 | down |
| XRCC6P1 | -2.036872841 | 0.024092891 | 0.071329013 | down |
| RP11-152C15.1 | -2.036829422 | 0.163415563 | | down |
| RP11-497J7.1 | -2.036732332 | 0.485455628 | | down |
| CTD-2283N19.1 | -2.036712807 | 0.00114171 | 0.006741184 | down |
| SERTM1 | -2.036510808 | 0.145424207 | | down |
| TMEM141 | -2.036414536 | 3.28E-08 | 1.09E-06 | down |
| CTD-2330K9.3 | -2.036376234 | 0.001436772 | 0.008063378 | down |
| PIN4P1 | -2.036313002 | 0.000428402 | 0.003074018 | down |
| AC127904.2 | -2.035973333 | 4.31E-06 | 7.03E-05 | down |
| RP11-770J1.4 | -2.035571647 | 0.008117026 | 0.031261109 | down |
| ZNF788 | -2.035488264 | 0.005597288 | 0.023645235 | down |
| RP11-864J10.4 | -2.035461039 | 0.000213867 | 0.001766684 | down |
| XRCC6P4 | -2.035402949 | 0.019313197 | 0.060511594 | down |
| RPL31P61 | -2.035392946 | 0.056296544 | 0.133378549 | down |
| MAFA | -2.0352799 | 0.000104324 | 0.000980697 | down |
| RP11-573D15.2 | -2.035003977 | 0.000665315 | 0.004374855 | down |
| MAOA | -2.034855186 | 4.75E-11 | 3.55E-09 | down |
| RP11-100K18.1 | -2.034833061 | 0.486093226 | | down |
| RGSL1 | -2.034757908 | 0.030008181 | 0.083788641 | down |
| RP11-569D9.5 | -2.034636799 | 0.017059813 | 0.05497051 | down |
| FBXW10 | -2.034456154 | 0.004601858 | 0.020344677 | down |
| CDK2AP2P3 | -2.034136483 | 0.4862433 | | down |

| | | | | |
|---|---|---|---|---|
| RPL7AP69 | -2.03405364 | 0.345422635 | 0.484020225 | down |
| MIR378A | -2.033766625 | 0.037838336 | 0.099668155 | down |
| NA | -2.033286966 | 0.214983316 | | down |
| FAM83A-AS1 | -2.0331972 | 0.083702052 | 0.177945402 | down |
| NA | -2.033037342 | 0.449627688 | | down |
| RP11-166B2.5 | -2.033021211 | 0.000570112 | 0.003869087 | down |
| AC147651.5 | -2.032961092 | 0.07381623 | | down |
| AGPAT2 | -2.032756376 | 2.76E-10 | 1.66E-08 | down |
| CTC-244M17.1 | -2.032722296 | 0.18382351 | | down |
| RBMX2P3 | -2.032678119 | 0.15235927 | | down |
| ABCC13 | -2.032554466 | 1.69E-07 | 4.49E-06 | down |
| RP11-530C5.2 | -2.032453972 | 0.136518614 | | down |
| GS1-24F4.2 | -2.032412939 | 0.004942618 | 0.021499223 | down |
| RP1-212G6.4 | -2.032398989 | 0.4452675 | | down |
| RP11-154D6.2 | -2.032389854 | 0.098722843 | 0.200231492 | down |
| RP11-196G11.3 | -2.032333772 | 0.000442477 | 0.003155419 | down |
| LINC00574 | -2.032045792 | 5.72E-07 | 1.27E-05 | down |
| NA | -2.031998071 | 0.392266537 | | down |
| XXbac-BPG34I8.3 | -2.031346581 | 0.102412545 | 0.205454227 | down |
| RP11-497H16.5 | -2.031124077 | 0.486892597 | | down |
| CYCSP2 | -2.031022785 | 0.200560773 | 0.329666696 | down |
| LINC00879 | -2.030950071 | 0.341698143 | | down |
| PRR7-AS1 | -2.030883812 | 1.65E-06 | 3.14E-05 | down |
| RP11-399D15.1 | -2.030842786 | 0.390191696 | | down |
| MTRNR2L10 | -2.030791687 | 0.000190123 | 0.00160451 | down |
| ATP5D | -2.0307912 | 3.12E-08 | 1.04E-06 | down |
| CRAT8 | -2.030726988 | 1.61E-05 | 0.000210986 | down |
| RP11-264J4.10 | -2.030381517 | 0.486972521 | | down |
| MIR4653 | -2.030355736 | 0.00042317 | 0.003040832 | down |
| LEUTX | -2.030193733 | 0.349292546 | 0.48792419 | down |
| RP11-325D8.1 | -2.029928466 | 0.11316024 | 0.220606717 | down |
| Y_RNA | -2.029655132 | 0.392468692 | 0.529971206 | down |
| CYP4B1 | -2.02939647 | 2.12E-05 | 0.000263155 | down |
| RP11-73B8.2 | -2.029053795 | 0.487258852 | | down |
| RP11-123J14.1 | -2.028774211 | 0.341554548 | | down |
| AKT3-IT1 | -2.028513676 | 0.037090231 | 0.098162341 | down |
| NA | -2.028429743 | 0.254506488 | | down |
| OR7E102P | -2.028359562 | 0.06386249 | 0.146163225 | down |
| RPS26P54 | -2.028213335 | 0.451674817 | | down |
| HHLA2 | -2.027951803 | 7.12E-08 | 2.15E-06 | down |
| NA | -2.027725734 | 0.393653023 | | down |
| CFAP65 | -2.027253906 | 7.76E-06 | 0.000114456 | down |
| C18orf63 | -2.027157398 | 0.390539893 | | down |
| NA | -2.027062948 | 0.446270292 | | down |
| RP11-284F21.11 | -2.027039197 | 0.034201027 | 0.092483502 | down |
| HLA-W | -2.027027289 | 0.04246247 | 0.108657053 | down |
| RP11-71B7.1 | -2.026916116 | 0.487720044 | | down |
| MIR202HG | -2.026865157 | 0.006217941 | 0.025653918 | down |
| RNA5SP325 | -2.026853011 | 0.343946259 | | down |
| RNU6-536P | -2.026843237 | 0.339222347 | | down |
| VN1R4 | -2.026594276 | 0.48786983 | | down |

| | | | | |
|---|---|---|---|---|
| MIR1273D | -2.026520929 | 0.487885662 | | down |
| EVX1 | -2.026490129 | 0.014360506 | 0.04831975 | down |
| CTD-2349P21.10 | -2.026297997 | 3.75E-05 | 0.000421944 | down |
| MIR6085 | -2.026121645 | 0.165604547 | 0.288413601 | down |
| BMS1P7 | -2.025935734 | 0.298083182 | | down |
| ADAMTS7P3 | -2.02569783 | 0.000530286 | 0.003652435 | down |
| MRPS17P1 | -2.025650801 | 0.176461894 | | down |
| HES5 | -2.025614321 | 3.85E-05 | 0.000431337 | down |
| SYT16 | -2.025458991 | 0.001933249 | 0.010202449 | down |
| CTC-276P9.2 | -2.025403937 | 0.488126801 | | down |
| KPNA7 | -2.02535525 | 0.000438442 | 0.00313219 | down |
| RP5-1065J22.4 | -2.02414648 | 0.029114415 | 0.082132722 | down |
| MYL6P1 | -2.023508177 | 0.488455771 | | down |
| LINC01090 | -2.023438463 | 0.048977079 | 0.120546472 | down |
| FRY-AS1 | -2.023416052 | 0.001353344 | 0.007693449 | down |
| RP11-470C13.4 | -2.022692037 | 0.005965453 | 0.024845502 | down |
| RP11-295H24.3 | -2.02268942 | 0.102955562 | | down |
| NA | -2.022613689 | 0.005262759 | 0.022568415 | down |
| OR7E24 | -2.022355368 | 0.488785259 | | down |
| CTB-134H23.3 | -2.022304837 | 3.09E-06 | 5.33E-05 | down |
| RP11-139K4.2 | -2.021902978 | 0.396169025 | | down |
| RNU6-45P | -2.02184529 | 0.04728585 | | down |
| HSPE1P27 | -2.021636292 | 0.011557269 | 0.041009399 | down |
| UBE2D3P2 | -2.021613268 | 0.453150917 | | down |
| RNU6-1036P | -2.021602819 | 0.488947874 | | down |
| RP11-884K10.6 | -2.021545819 | 0.060321831 | | down |
| RP11-800A3.2 | -2.021544828 | 0.007143071 | 0.028411215 | down |
| RP11-34P13.7 | -2.021409368 | 0.256190995 | | down |
| KRTAP5-AS1 | -2.020455104 | 1.94E-06 | 3.62E-05 | down |
| PSAT1P1 | -2.019921497 | 0.316285295 | 0.454903318 | down |
| NA | -2.019707016 | 0.00283661 | 0.013873205 | down |
| KRTAP9-10P | -2.019407497 | 0.489422418 | | down |
| FAM90A25P | -2.018633959 | 0.021591829 | 0.065680754 | down |
| GUSBP8 | -2.018588877 | 0.159029158 | 0.280195633 | down |
| CTB-47B11.3 | -2.018472464 | 0.000275068 | 0.002174201 | down |
| CTD-255008.7 | -2.018141824 | 0.042338007 | 0.108430916 | down |
| FAT2 | -2.018129362 | 2.76E-05 | 0.00032613 | down |
| CTD-2236F14.1 | -2.018043344 | 0.299706952 | | down |
| C16orf46 | -2.017993553 | 0.123823321 | | down |
| CLCN3P1 | -2.017923142 | 5.67E-05 | 0.000592555 | down |
| CA5AP1 | -2.017262476 | 0.34936235 | | down |
| GABRB1 | -2.017163706 | 0.006026375 | 0.025038918 | down |
| RP11-12D5.5 | -2.017059968 | 0.394687267 | 0.532148953 | down |
| RP11-473M20.5 | -2.016995696 | 3.72E-05 | 0.000419831 | down |
| Y_RNA | -2.016776936 | 0.396256399 | | down |
| NA | -2.01650318 | 0.03687044 | 0.097726996 | down |
| CH17-13I23.1 | -2.016494079 | 0.219624213 | | down |
| RP1-71H24.1 | -2.016465879 | 0.128882076 | 0.241826948 | down |
| AHRR | -2.016295371 | 0.002242523 | 0.011515014 | down |
| NA | -2.016282275 | 0.489867841 | | down |
| RP11-20G6.2 | -2.016262611 | 0.125093899 | 0.236789385 | down |

| | | | | |
|---|---|---|---|---|
| CTD-3066C23.1 | -2.016177725 | 0.490121018 | | down |
| AP000487.6 | -2.016028433 | 0.000318603 | 0.002446858 | down |
| ACOT6 | -2.016024364 | 0.010435585 | 0.037902165 | down |
| FAR2P3 | -2.016003629 | 0.004300201 | 0.019280638 | down |
| NA | -2.015978371 | 0.016854209 | 0.054467538 | down |
| VTRNA1-1 | -2.015724111 | 0.154585021 | | down |
| SLC25A52 | -2.015723115 | 0.078047313 | 0.169144736 | down |
| NA | -2.015640833 | 3.62E-06 | 6.06E-05 | down |
| RNU2-57P | -2.015480612 | 0.218720644 | | down |
| MIR646 | -2.015321573 | 0.490306293 | | down |
| CCDC180 | -2.015017026 | 2.19E-05 | 0.000269977 | down |
| LINC00484 | -2.015000661 | 0.000256994 | 0.00205842 | down |
| NA | -2.014861128 | 0.490405951 | | down |
| LINC01287 | -2.014806625 | 0.133651505 | 0.247850493 | down |
| PRAMEF6 | -2.014699432 | 0.490283368 | | down |
| RP1-111J24.1 | -2.013972727 | 0.191837121 | | down |
| UMODL1 | -2.013947016 | 3.58E-05 | 0.000405886 | down |
| RP11-5407.2 | -2.013845404 | 0.067722258 | 0.15256805 | down |
| TMEM132D | -2.013775188 | 0.000115345 | 0.001067925 | down |
| SIGLEC15 | -2.013699837 | 4.64E-07 | 1.05E-05 | down |
| RP11-407P15.1 | -2.01362582 | 0.299552142 | | down |
| SAMD12-AS1 | -2.013506656 | 0.000557412 | 0.00380093 | down |
| RP1-90J20.8 | -2.013480622 | 0.008816947 | | down |
| IFNA5 | -2.013344709 | 0.347479199 | | down |
| AL132640.1 | -2.013308983 | 0.264768705 | | down |
| OR6R2P | -2.013139259 | 0.119245708 | 0.229140288 | down |
| RPL12P30 | -2.012584436 | 0.2531661 | | down |
| MIR429 | -2.012234838 | 0.001614841 | 0.008854729 | down |
| AKR1B10 | -2.012165771 | 7.18E-08 | 2.16E-06 | down |
| RP3-355L5.3 | -2.012002076 | 0.490944167 | | down |
| RP11-167N4.2 | -2.01193502 | 0.251597414 | | down |
| RP11-158D2.2 | -2.011711034 | 0.104995908 | 0.209128684 | down |
| NA | -2.011680953 | 0.25390498 | 0.38894209 | down |
| SNRPCP1 | -2.011671038 | 0.454110627 | | down |
| OR56A3 | -2.011651368 | 0.175206823 | 0.299664909 | down |
| PCYT1B-AS1 | -2.01141654 | 0.395749443 | | down |
| LA16c-306E5.1 | -2.011319861 | 0.225570912 | | down |
| RP5-942I16.1 | -2.011281433 | 0.015287681 | 0.050673334 | down |
| RP11-416H1.2 | -2.011251761 | 0.178311226 | | down |
| PRAMEF4 | -2.011172171 | 0.491204772 | | down |
| CFAP77 | -2.011123393 | 0.019821123 | 0.061635191 | down |
| DNAH2 | -2.010955057 | 1.19E-05 | 0.000163224 | down |
| AC016831.5 | -2.0107509 | 0.491296039 | | down |
| TMEM256-PLSCR3 | -2.010711667 | 0.219263256 | | down |
| KRT18P9 | -2.010444461 | 0.491362434 | | down |
| LINC00466 | -2.010409318 | 0.033277912 | 0.090537135 | down |
| RP11-1348G14.5 | -2.01030989 | 0.004085963 | 0.018552012 | down |
| CTC-546K23.1 | -2.010241125 | 0.259403469 | | down |
| RP11-196B3.1 | -2.010179504 | 0.02948865 | 0.082830684 | down |
| AADACL2 | -2.010152749 | 0.012838363 | 0.04435044 | down |
| GYG1P2 | -2.009958548 | 0.491467726 | | down |

| | | | | |
|---|---|---|---|---|
| NA | -2.009445254 | 0.011692077 | 0.041375356 | down |
| IL12A-AS1 | -2.009207807 | 0.000534896 | 0.003674919 | down |
| LINC01016 | -2.00910268 | 0.000640414 | 0.004244563 | down |
| RP11-1085N6.3 | -2.008997376 | 0.189635625 | | down |
| NA | -2.008951863 | 0.000195362 | 0.001638141 | down |
| RP11-184E9.1 | -2.008858178 | 0.052770717 | 0.127089642 | down |
| NDUFB9P2 | -2.008768846 | 0.021473128 | 0.065416614 | down |
| RP11-554I8.2 | -2.008436258 | 0.200538383 | | down |
| RPL19P11 | -2.008379743 | 0.491809916 | | down |
| RP11-334E6.2 | -2.008351603 | 0.074662166 | 0.163781405 | down |
| CICP5 | -2.008143886 | 0.149136867 | 0.267918442 | down |
| AP000350.6 | -2.007709467 | 0.012870338 | 0.044435352 | down |
| RP13-395E19.4 | -2.00721083 | 0.165208624 | 0.287974466 | down |
| RP11-110J1.2 | -2.007123417 | 0.008046404 | 0.031089872 | down |
| RP11-66602.5 | -2.006760251 | 0.019394215 | 0.060680452 | down |
| KRT18P13 | -2.006738508 | 0.063107371 | | down |
| UGT2B29P | -2.006719427 | 0.040603776 | 0.105073198 | down |
| RP4-535B20.2 | -2.006715816 | 0.492170692 | | down |
| RN7SL827P | -2.0066739 | 0.037711333 | 0.099429513 | down |
| TMEFF2 | -2.006544836 | 4.51E-05 | 0.000491558 | down |
| CTD-2189E23.1 | -2.00646467 | 0.076403091 | 0.166532633 | down |
| RP11-575F12.2 | -2.006344691 | 0.000667068 | 0.004385424 | down |
| LINC01020 | -2.006253453 | 0.352175442 | | down |
| LINC01361 | -2.006042978 | 0.34943049 | | down |
| EYS | -2.005938669 | 1.15E-05 | 0.000158502 | down |
| KRT85 | -2.005453798 | 0.310658122 | | down |
| RP11-156K13.3 | -2.005260634 | 0.019325108 | 0.06051196 | down |
| RP4-590F24.1 | -2.004881082 | 0.066566071 | 0.150618393 | down |
| TMCC1P1 | -2.004680734 | 0.492612077 | | down |
| MIR4480 | -2.004232527 | 0.258090582 | | down |
| RP11-351J23.1 | -2.004215967 | 0.000121107 | 0.001111 | down |
| RP11-103H7.2 | -2.004143996 | 0.398801875 | | down |
| RP11-55204.1 | -2.004052173 | 0.457387079 | | down |
| AC093843.1 | -2.003430734 | 0.492883284 | | down |
| Y_RNA | -2.003327312 | 0.128039858 | 0.240667511 | down |
| RP4-728D4.3 | -2.003274292 | 0.395756759 | | down |
| RP11-420C9.1 | -2.002902089 | 3.68E-05 | 0.000416362 | down |
| AC091814.2 | -2.002884412 | 0.112250678 | 0.219363629 | down |
| AC096669.3 | -2.00198381 | 0.173002852 | | down |
| ZNF733P | -2.001687439 | 0.493261653 | | down |
| WI2-85898F10.1 | -2.001549788 | 0.224580081 | | down |
| RP11-378G13.2 | -2.001107682 | 0.272273292 | | down |
| MAGEB1 | -2.001004005 | 0.493410022 | | down |
| ANO3 | -2.000788843 | 0.001268218 | 0.007313309 | down |
| NA | -2.000766696 | 0.099435826 | 0.201208977 | down |
| RP11-344N17.12 | -2.000507058 | 0.493517909 | | down |
| AC026954.6 | -2.000382925 | 0.025327112 | 0.07402021 | down |
| SPATA31D5P | -2.000307783 | 0.350150656 | | down |
| OSBPL6 | -2.000137927 | 1.19E-10 | 7.97E-09 | down |
| KCNA10 | -2.000053349 | 0.493535432 | | down |
| OR1A1 | -1.999975201 | 0.093869441 | 0.193284454 | down |

| | | | | |
|---|---|---|---|---|
| CTD-2142D14.1 | -1.999111532 | 0.459559701 | | down |
| BMP2KL | -1.998865631 | 0.493793404 | | down |
| RP13-379L11.1 | -1.998726787 | 0.493904508 | | down |
| FAM90A20P | -1.998591931 | 0.210000255 | 0.340414787 | down |
| OVOL1-AS1 | -1.998264472 | 0.003352466 | 0.01579945 | down |
| OR13C1P | -1.998168452 | 0.493793842 | | down |
| SNORA68 | -1.998084349 | 0.248019673 | 0.382304811 | down |
| RP11-907D1.3 | -1.997886973 | 0.494086936 | | down |
| RP11-634B22.4 | -1.997795171 | 0.493874922 | | down |
| RN7SL138P | -1.9975987 | 6.31E-05 | 0.000646591 | down |
| NA | -1.997595762 | 0.164661697 | | down |
| AC008060.7 | -1.996792209 | 0.451354649 | | down |
| RPL7AP25 | -1.996706365 | 0.494262579 | | down |
| RP11-324H7.1 | -1.996413812 | 0.072817284 | 0.160932947 | down |
| AC079117.1 | -1.996376838 | 0.399371103 | | down |
| SOX9-AS1 | -1.996257046 | 7.32E-07 | 1.56E-05 | down |
| KDM4E | -1.996113666 | 0.107037544 | | down |
| GS1-259H13.7 | -1.996073387 | 0.020025273 | | down |
| RNU6ATAC30P | -1.995586673 | 0.185165634 | 0.31131037 | down |
| RP11-356K23.1 | -1.995585348 | 0.016496348 | 0.053614914 | down |
| RP1-69M21.2 | -1.995485988 | 0.494527853 | | down |
| DBX1 | -1.99456275 | 0.459546779 | | down |
| LINC01249 | -1.994539437 | 0.494814457 | | down |
| KB-1980E6.2 | -1.994531354 | 0.107699999 | 0.213256361 | down |
| NA | -1.99443442 | 0.014886717 | 0.049663277 | down |
| AC005562.1 | -1.994249869 | 0.000362652 | 0.002711586 | down |
| RP11-963H4.3 | -1.993799473 | 0.000377733 | 0.002790413 | down |
| OR7D4 | -1.993517629 | 0.245438568 | 0.379357771 | down |
| NA | -1.993342668 | 0.012889127 | 0.044489997 | down |
| NA | -1.993145083 | 0.451584355 | | down |
| EPPIN | -1.993023388 | 0.495063379 | | down |
| AC092669.6 | -1.992848444 | 0.495182177 | | down |
| GUCA1C | -1.992848444 | 0.495182177 | | down |
| RP11-58B2.1 | -1.992848444 | 0.495182177 | | down |
| ACTG1P18 | -1.992848444 | 0.495182177 | | down |
| RP11-114H21.2 | -1.992802697 | 0.268915273 | | down |
| RP11-114H7.2 | -1.992613696 | 0.403217963 | | down |
| RP11-423H2.5 | -1.992253583 | 0.232656695 | | down |
| RPL3P6 | -1.992209764 | 0.002523279 | 0.012670799 | down |
| EIF4BP5 | -1.991915567 | 0.187005069 | 0.313473206 | down |
| BARHL1 | -1.991870244 | 0.197705161 | | down |
| RP4-575N6.2 | -1.991714792 | 0.001151882 | 0.006788375 | down |
| RP11-736N17.11 | -1.991669595 | 0.000211888 | 0.001753712 | down |
| EIF3KP2 | -1.991607591 | 0.495452102 | | down |
| RP11-19P22.7 | -1.991339501 | 0.495510431 | | down |
| ASB18 | -1.991213464 | 0.194604306 | 0.322913842 | down |
| GM2AP2 | -1.991201429 | 0.402849338 | | down |
| AC018866.1 | -1.990737581 | 0.325521863 | 0.464033918 | down |
| CYP4A11 | -1.990704808 | 0.005826709 | 0.02439488 | down |
| RP11-362A1.1 | -1.990655511 | 0.334015612 | 0.472265285 | down |
| CTC-436K13.6 | -1.99059643 | 0.142842956 | 0.259922099 | down |

| | | | | |
|---|---|---|---|---|
| SLC9A3 | -1.990533825 | 1.97E-06 | 3.66E-05 | down |
| RP11-216M21.9 | -1.990497912 | 0.196007754 | | down |
| CXXC4 | -1.990186597 | 1.50E-07 | 4.02E-06 | down |
| KRT18P68 | -1.990126915 | 0.206409816 | | down |
| FOXE1 | -1.989932705 | 0.202785273 | | down |
| CATSPER2 | -1.989879611 | 2.47E-10 | 1.51E-08 | down |
| NA | -1.989742887 | 0.061705187 | 0.142726203 | down |
| LINC01567 | -1.989639279 | 0.495880433 | | down |
| SIGLECL1 | -1.989410357 | 0.176093203 | | down |
| TRPM5 | -1.989279115 | 1.14E-05 | 0.000156588 | down |
| NA | -1.988778795 | 0.401856235 | | down |
| RP11-221N13.4 | -1.988146633 | 0.320541477 | 0.459309779 | down |
| HSPD1P5 | -1.987610583 | 0.22561688 | | down |
| OR1Q1 | -1.986967268 | 0.353093588 | | down |
| RP11-46B11.2 | -1.986611902 | 4.15E-06 | 6.82E-05 | down |
| NA | -1.986479598 | 0.010232248 | 0.037326086 | down |
| ZNF730 | -1.986344967 | 0.001440189 | 0.008074085 | down |
| RP11-630A13.4 | -1.985989029 | 0.004633398 | 0.020459407 | down |
| AC002127.4 | -1.985974957 | 0.124432494 | 0.235921309 | down |
| RP11-537H15.4 | -1.985625668 | 0.240781617 | | down |
| RP5-1103G7.10 | -1.98550187 | 0.496781429 | | down |
| BPESC1 | -1.985380428 | 0.071602925 | 0.158985904 | down |
| NA | -1.985247775 | 0.496756311 | | down |
| AC097495.3 | -1.985246704 | 0.015076278 | 0.050133006 | down |
| RP11-438C19.2 | -1.98503467 | 0.306784402 | | down |
| NA | -1.984827963 | 0.004656606 | 0.020540753 | down |
| RP11-629N8.4 | -1.984784696 | 0.015842391 | 0.052036506 | down |
| HIST1H2APS4 | -1.984663774 | 0.007885265 | 0.030643501 | down |
| POT1-AS1 | -1.984502369 | 8.95E-05 | 0.000862553 | down |
| RNU6-194P | -1.984454978 | 0.067643154 | 0.15242912 | down |
| LINC01242 | -1.984266761 | 0.496970119 | | down |
| MRPS36P2 | -1.983535253 | 0.497051724 | | down |
| RP11-64C12.6 | -1.983535253 | 0.497051724 | | down |
| DLX2 | -1.983508772 | 0.019462733 | 0.06082484 | down |
| BTNL8 | -1.983188329 | 6.17E-06 | 9.47E-05 | down |
| RP11-401P9.1 | -1.983033464 | 0.462063998 | | down |
| KRT17P7 | -1.982733043 | 0.153957596 | | down |
| RN7SL491P | -1.982528957 | 0.219855508 | | down |
| RP11-141M1.4 | -1.982482561 | 0.015060075 | 0.050090211 | down |
| AC008984.6 | -1.982258912 | 0.035159494 | 0.094365896 | down |
| GABRA2 | -1.982105147 | 4.42E-07 | 1.01E-05 | down |
| RP11-511B7.1 | -1.982091678 | 0.497524711 | | down |
| RP3-521E19.3 | -1.981921262 | 0.13957897 | 0.255790946 | down |
| RP11-345K9.2 | -1.981840986 | 0.053315507 | 0.128062927 | down |
| RP11-521D12.4 | -1.98129722 | 0.404286202 | | down |
| RAB6C-AS1 | -1.980936742 | 0.00074312 | 0.004787967 | down |
| NA | -1.980770016 | 0.404466555 | | down |
| TGIF2P1 | -1.980749745 | 0.270111476 | | down |
| CTC-479C5.11 | -1.980549232 | 0.056134714 | | down |
| RP11-305B6.3 | -1.980529281 | 0.497865446 | | down |
| RP11-579O24.3 | -1.979734274 | 0.083344942 | 0.177463085 | down |

| | | | | |
|---|---|---|---|---|
| SLC6A13 | -1.979641714 | 0.001396999 | 0.007886405 | down |
| MIR5689 | -1.979637003 | 0.497689775 | | down |
| RP11-676F20.2 | -1.97957692 | 0.454479323 | | down |
| RP11-87N24.3 | -1.97947473 | 0.092831473 | 0.191606813 | down |
| RP11-407G23.2 | -1.97925875 | 0.001057947 | 0.006342474 | down |
| NA | -1.979230539 | 0.126735634 | 0.238948565 | down |
| CARSP1 | -1.979171738 | 0.259187209 | 0.394450694 | down |
| RP11-684B21.1 | -1.978988879 | 0.208400952 | | down |
| CTSV | -1.97888211 | 4.77E-06 | 7.63E-05 | down |
| MIR4673 | -1.9788748 | 0.408724564 | | down |
| RP11-446J8.1 | -1.978396882 | 0.147000121 | 0.265333232 | down |
| AC004231.2 | -1.978308357 | 0.091959079 | 0.190381654 | down |
| LA16c-358B7.4 | -1.978214744 | 7.06E-05 | 0.000706757 | down |
| RP11-945F5.1 | -1.977984951 | 0.083117168 | 0.177116973 | down |
| NA | -1.977809006 | 0.000926631 | 0.005711815 | down |
| NA | -1.977733443 | 0.206439718 | | down |
| NA | -1.977657808 | 0.094417889 | 0.194107777 | down |
| RP11-577B7.1 | -1.977409082 | 0.404574619 | | down |
| SHD | -1.977270115 | 1.15E-07 | 3.19E-06 | down |
| Metazoa_SRP | -1.97719808 | 0.002459437 | 0.012408285 | down |
| KHSRPP1 | -1.976832383 | 0.145392398 | 0.263122885 | down |
| MYOG | -1.976472142 | 0.038977167 | 0.101926191 | down |
| GPR83 | -1.976301434 | 0.000158776 | 0.001382042 | down |
| SDR16C6P | -1.975918302 | 0.199959188 | 0.32900746 | down |
| RP11-286B14.1 | -1.975915112 | 0.100601277 | | down |
| RP11-384C4.6 | -1.975878297 | 0.247655526 | 0.382037514 | down |
| Y_RNA | -1.97585941 | 0.209826139 | 0.340169249 | down |
| CTD-2345M20.2 | -1.975797306 | 0.308379042 | 0.446890794 | down |
| RP11-557C18.3 | -1.975764458 | 0.235514293 | | down |
| PAICSP7 | -1.975692562 | 0.498921041 | | down |
| LINC00502 | -1.975621902 | 0.498703879 | | down |
| DDX4 | -1.97522238 | 0.02480419 | 0.072861748 | down |
| AC013470.6 | -1.975014742 | 0.13208114 | 0.246068627 | down |
| ARGFX | -1.974951633 | 0.003765242 | 0.017373698 | down |
| RP11-423P10.2 | -1.974629191 | 0.001002084 | 0.006073553 | down |
| RP11-291L19.1 | -1.974482922 | 0.307814192 | | down |
| NA | -1.973687923 | 0.063487286 | 0.145664313 | down |
| LSM6P2 | -1.973581767 | 0.4056729 | | down |
| AC074019.2 | -1.973575392 | 0.248837884 | 0.383330021 | down |
| RP11-1102P22.3 | -1.973569096 | 0.075421895 | 0.165026247 | down |
| TMC1 | -1.973512944 | 0.000402718 | 0.002928465 | down |
| KRT127P | -1.9734276 | 0.499415766 | | down |
| RP11-2E17.2 | -1.97340385 | 0.357929888 | | down |
| RP11-300I6.5 | -1.973326184 | 0.464275838 | | down |
| RP11-611E13.3 | -1.97290599 | 0.257655239 | | down |
| LINC01593 | -1.972689035 | 0.230351061 | | down |
| AC005915.1 | -1.972545379 | 0.356379683 | | down |
| RP11-261C10.1 | -1.972261074 | 0.499590637 | | down |
| RNU6-1213P | -1.972009408 | 0.356084192 | | down |
| RP11-362L22.1 | -1.971949588 | 0.009359108 | 0.034864855 | down |
| MT-TP | -1.971936228 | 7.55E-06 | 0.000111887 | down |

| | | | | |
|---|---|---|---|---|
| RP11-319G6.3 | -1.971851122 | 0.262999936 | | down |
| RP11-598F7.4 | -1.971720743 | 0.005229058 | 0.022446323 | down |
| CEACAM8 | -1.971366235 | 0.009175067 | 0.034336787 | down |
| COX6B1P4 | -1.971348965 | 0.312145222 | | down |
| PRDX2P3 | -1.971181269 | 0.356490991 | | down |
| AF127577.8 | -1.971163241 | 0.216333467 | 0.347482162 | down |
| GJB7 | -1.97109027 | 0.034700564 | 0.093468149 | down |
| RP11-351O1.4 | -1.970927972 | 0.49935227 | | down |
| YWHAEP7 | -1.97086612 | 0.006708803 | 0.027135068 | down |
| RP11-157L3.9 | -1.970700635 | 0.000119662 | 0.001100773 | down |
| GRM5 | -1.970662817 | 0.499861773 | | down |
| ROR1-AS1 | -1.97065892 | 0.000403409 | 0.002931451 | down |
| PRAMEF2 | -1.970581509 | 0.500037791 | | down |
| NA | -1.970570549 | 0.500040187 | | down |
| ZBTB8B | -1.970496426 | 0.021879014 | 0.066343614 | down |
| MGAM2 | -1.970452128 | 0.000418267 | 0.003015696 | down |
| MATR3 | -1.970049059 | 0.214168742 | 0.345068144 | down |
| Y_RNA | -1.969514573 | 0.229983827 | 0.362349053 | down |
| RP11-767C1.1 | -1.969445231 | 0.167804506 | | down |
| RP11-286E11.1 | -1.969394334 | 8.55E-06 | 0.000124058 | down |
| PCBP2-OT1 | -1.969321818 | 0.038484027 | 0.100935322 | down |
| U2 | -1.969150189 | 0.29710526 | 0.434514454 | down |
| CTD-2541M15.3 | -1.968925256 | 0.001316333 | 0.007530066 | down |
| RP11-42F12.1 | -1.968863258 | 0.076075943 | 0.165961779 | down |
| RP11-476B13.2 | -1.968732932 | 0.229893417 | | down |
| AP000344.4 | -1.968687295 | 0.000291087 | 0.00227627 | down |
| NDUFB9P1 | -1.968683229 | 0.500452894 | | down |
| RP11-391M7.3 | -1.968595864 | 0.225819289 | | down |
| SERPINA6 | -1.968303123 | 0.005980062 | 0.024891242 | down |
| NOP2 | -1.968302926 | 0.000110673 | 0.001030067 | down |
| RP11-560A15.3 | -1.96823738 | 0.463977711 | | down |
| AC004076.7 | -1.967974591 | 0.136461808 | 0.251699686 | down |
| LRTM1 | -1.967885308 | 0.016405468 | 0.05340042 | down |
| TXNDC8 | -1.967618115 | 0.358238326 | | down |
| SLC38A3 | -1.967413128 | 0.000236265 | 0.001920027 | down |
| RN7SKP127 | -1.966974344 | 0.000230338 | 0.001879648 | down |
| AC096649.3 | -1.966816258 | 0.50078149 | | down |
| PCSK6-AS1 | -1.966816258 | 0.50078149 | | down |
| XXyac-YR12DB5.1 | -1.966568562 | 0.50091553 | | down |
| CACNA1G-AS1 | -1.966544949 | 0.008491739 | 0.032351738 | down |
| RP11-17403.1 | -1.966525108 | 0.27901313 | | down |
| VTI1BP4 | -1.966367981 | 0.181273015 | 0.306724656 | down |
| DGAT2L7P | -1.966353328 | 5.13E-05 | 0.000546197 | down |
| PTGR1 | -1.966036214 | 3.12E-11 | 2.49E-09 | down |
| CTD-2201E9.1 | -1.965890862 | 0.103657685 | | down |
| AF001548.3 | -1.965727874 | 0.000120969 | 0.001110757 | down |
| KIAA2012 | -1.965396994 | 9.48E-05 | 0.00090386 | down |
| RP11-15A1.2 | -1.965336927 | 0.006161835 | 0.025467901 | down |
| CCDC83 | -1.96519346 | 0.147080914 | 0.265447166 | down |
| GLIS3-AS1 | -1.96501654 | 0.000919706 | 0.005676698 | down |
| RP11-453A12.1 | -1.964979205 | 0.103997409 | 0.20760785 | down |

| | | | | |
|---|---|---|---|---|
| RP11-731J8.2 | -1.964728605 | 0.000555984 | 0.003792912 | down |
| RP13-638C3.5 | -1.964448405 | 0.025648124 | 0.074718532 | down |
| RP3-446N13.5 | -1.96432028 | 0.000206412 | 0.001715473 | down |
| RP11-746P2.6 | -1.964317034 | 0.210076355 | 0.340475956 | down |
| RP11-355N15.3 | -1.964278453 | 0.104809758 | | down |
| WBSCR27 | -1.964073153 | 2.47E-09 | 1.14E-07 | down |
| RP11-643G5.2 | -1.963999389 | 0.319523737 | 0.45829286 | down |
| ANKRD26P3 | -1.963842598 | 0.04931273 | 0.121160587 | down |
| RP11-363G15.2 | -1.963786687 | 0.09679327 | 0.19750455 | down |
| RP11-306G20.1 | -1.963652735 | 0.032027797 | 0.088020777 | down |
| PHLPP2 | -1.963601936 | 1.81E-11 | 1.50E-09 | down |
| CTB-167G5.3 | -1.962656736 | 0.50177193 | | down |
| LINC01075 | -1.962260255 | 0.501858773 | | down |
| EDA | -1.962166322 | 9.33E-06 | 0.000133534 | down |
| RNU6-1122P | -1.960787025 | 0.50218153 | | down |
| PCGEM1 | -1.960696306 | 0.458934694 | | down |
| SOX21 | -1.960670861 | 0.410787063 | | down |
| POLR3DP1 | -1.960490896 | 0.362832721 | | down |
| C1orf94 | -1.960330496 | 0.045327861 | 0.113808312 | down |
| RPL21P116 | -1.96020475 | 0.001398465 | 0.00789023 | down |
| NA | -1.960172879 | 0.46901686 | | down |
| RP11-486M23.3 | -1.960020047 | 0.315631411 | | down |
| MTCO1P3 | -1.959432342 | 0.46731642 | | down |
| TST | -1.959361693 | 1.92E-08 | 6.87E-07 | down |
| CYP4F11 | -1.959274561 | 6.16E-05 | 0.00063469 | down |
| KRT8P22 | -1.959226385 | 0.464212891 | | down |
| RP11-1113L8.1 | -1.958975519 | 0.277548205 | | down |
| CTD-2308L22.1 | -1.958799286 | 0.000318025 | 0.002443668 | down |
| NA | -1.958696906 | 0.130414682 | | down |
| U3 | -1.958498051 | 0.16131461 | 0.282992884 | down |
| PRICKLE2-AS3 | -1.958439025 | 0.326713287 | | down |
| RPL9P2 | -1.958007793 | 0.019395366 | 0.060680452 | down |
| C12orf50 | -1.958005052 | 0.068542535 | 0.153947782 | down |
| AC007395.4 | -1.957549905 | 0.118197189 | 0.227635663 | down |
| RP11-422J15.1 | -1.957504694 | 0.307371286 | 0.445688364 | down |
| MIR378H | -1.957198871 | 0.206172542 | | down |
| SLC26A9 | -1.956915429 | 0.001901794 | 0.010066509 | down |
| HINT2P1 | -1.956869504 | 0.469138416 | | down |
| SMPD4P1 | -1.956561329 | 0.144958409 | 0.262531223 | down |
| RPL32P26 | -1.956385399 | 0.146784682 | 0.265024477 | down |
| RP4-777L9.2 | -1.956360352 | 0.001179518 | 0.006918674 | down |
| AGAP1-IT1 | -1.956342805 | 9.20E-06 | 0.00013201 | down |
| RP11-138E9.2 | -1.955729346 | 0.243303274 | | down |
| IMPA1P1 | -1.955537142 | 0.024541595 | 0.072294116 | down |
| RP11-90C4.3 | -1.955389803 | 0.503285454 | | down |
| SMIM18 | -1.955340244 | 0.008225757 | 0.031590777 | down |
| AC107083.1 | -1.955055606 | 0.503438213 | | down |
| BICD1P1 | -1.955055606 | 0.503438213 | | down |
| CTD-2272G21.2 | -1.955055606 | 0.503438213 | | down |
| ARAFP1 | -1.955055606 | 0.503438213 | | down |
| VN1R46P | -1.955055606 | 0.503438213 | | down |

| | | | | |
|---|---|---|---|---|
| ARL6IP1P3 | -1.955055606 | 0.503438213 | | down |
| CTD-2382E5.2 | -1.955055606 | 0.503438213 | | down |
| GPRIN2 | -1.955040955 | 7.95E-11 | 5.55E-09 | down |
| RP11-407G23.4 | -1.95477466 | 0.000227477 | 0.001860757 | down |
| CFAP57 | -1.954492763 | 0.000301147 | 0.002336693 | down |
| AOC1 | -1.954361878 | 2.79E-07 | 6.86E-06 | down |
| RP11-201E8.1 | -1.954220037 | 0.074869592 | 0.164146945 | down |
| FCGBP | -1.953766788 | 2.11E-05 | 0.000262181 | down |
| RP11-642D21.1 | -1.953684685 | 0.278053012 | | down |
| RP11-43P5.1 | -1.953435776 | 0.141604436 | 0.258325562 | down |
| RP11-47A17.2 | -1.953308862 | 0.0364749 | 0.096969464 | down |
| AC010744.1 | -1.953158972 | 0.503854435 | | down |
| OR1F2P | -1.95244547 | 0.467019244 | | down |
| RP11-416N2.4 | -1.952142019 | 3.91E-07 | 9.07E-06 | down |
| RP11-941H19.1 | -1.951835197 | 0.020537689 | 0.063337541 | down |
| RP11-321C24.1 | -1.951637867 | 0.504188377 | | down |
| CTA-992D9.9 | -1.951637867 | 0.504188377 | | down |
| RPE65 | -1.951540843 | 0.012062812 | 0.04234811 | down |
| CTD-2246P4.1 | -1.951104168 | 0.000726503 | 0.004701067 | down |
| LPAR4 | -1.951091653 | 0.000187708 | 0.001589041 | down |
| MPP4 | -1.950654108 | 0.011414436 | 0.04060809 | down |
| RP11-622C24.1 | -1.95064447 | 0.122309972 | 0.23314908 | down |
| NA | -1.950614396 | 0.097936421 | | down |
| CTD-2135D7.2 | -1.950541875 | 0.094671805 | | down |
| NEURL3 | -1.950341314 | 1.68E-07 | 4.46E-06 | down |
| CTBP2P2 | -1.950326271 | 0.103754957 | 0.207343392 | down |
| RPSAP18 | -1.95010818 | 6.94E-06 | 0.000104333 | down |
| RN7SKP152 | -1.949663232 | 0.504622059 | | down |
| SIAH3 | -1.949247533 | 0.000790189 | 0.005025461 | down |
| HRASLS2 | -1.949154847 | 8.51E-07 | 1.77E-05 | down |
| CTB-147C22.8 | -1.949083457 | 0.50474943 | | down |
| RP11-5407.16 | -1.948754621 | 0.024142432 | 0.071440483 | down |
| NDUFB1P2 | -1.9487533 | 0.316035843 | | down |
| TM4SF20 | -1.948415186 | 0.002616786 | 0.013035767 | down |
| RP11-241J12.1 | -1.94830409 | 0.276472137 | 0.412798374 | down |
| RP11-4104.1 | -1.94824374 | 6.38E-05 | 0.000651953 | down |
| LINC00710 | -1.947764631 | 0.052544925 | 0.126701157 | down |
| TRBV1 | -1.947660304 | 0.10925104 | 0.215489644 | down |
| NA | -1.94732153 | 0.02272373 | 0.068214755 | down |
| LINC01122 | -1.946588405 | 0.000985878 | 0.005989832 | down |
| RP11-459016.8 | -1.946411765 | 0.098247605 | | down |
| PRDX2P1 | -1.946195442 | 0.173674412 | 0.298232399 | down |
| FRG2DP | -1.946165468 | 0.505311629 | | down |
| RP11-162D9.3 | -1.945918424 | 0.182836761 | | down |
| RP11-631F7.1 | -1.945864626 | 0.362312421 | | down |
| ABHD12B | -1.945529427 | 2.36E-05 | 0.000287185 | down |
| NA | -1.945305976 | 0.009539195 | 0.035342898 | down |
| RP11-65J3.15 | -1.945153788 | 0.163264397 | | down |
| CTD-2244C20.2 | -1.944987348 | 0.505649769 | | down |
| CNTFR | -1.944972823 | 2.46E-06 | 4.39E-05 | down |
| FHAD1 | -1.94491406 | 6.50E-07 | 1.41E-05 | down |

| | | | | |
|---|---|---|---|---|
| LELP1 | -1.944787834 | 0.505614599 | | down |
| EMX1 | -1.944473935 | 0.003496854 | 0.016361856 | down |
| NA | -1.943752293 | 0.264121877 | | down |
| RP11-624J6.2 | -1.943494322 | 0.414763579 | | down |
| CTB-50L17.2 | -1.943170417 | 0.325355489 | | down |
| RP11-613G13.1 | -1.943118862 | 0.473540236 | | down |
| SLC25A5P9 | -1.942904803 | 0.506107779 | | down |
| LINC01322 | -1.942701834 | 0.000109737 | 0.00102231 | down |
| Xbac-BPG249D20. | -1.942642047 | 0.003991866 | 0.018210086 | down |
| RP11-776A13.1 | -1.942633275 | 0.414731865 | | down |
| NIFKP4 | -1.942541167 | 0.318966193 | | down |
| DUX4L17 | -1.942443274 | 0.506130435 | | down |
| RP11-293P20.2 | -1.942246618 | 0.00189182 | 0.010036337 | down |
| NA | -1.941069127 | 0.083956139 | 0.178310352 | down |
| CTB-174D11.1 | -1.940815365 | 0.315242276 | | down |
| LINC00626 | -1.940799788 | 0.231982038 | 0.364674987 | down |
| TAS2R12 | -1.940725254 | 0.116100953 | | down |
| RP11-1041F24.1 | -1.940665809 | 0.506521684 | | down |
| NA | -1.940626414 | 0.414422105 | | down |
| RNU6-552P | -1.940495886 | 0.506637837 | | down |
| RNU6-437P | -1.940357333 | 0.022768562 | 0.068280891 | down |
| PLSCR5 | -1.940349774 | 0.416064994 | | down |
| RPS7P3 | -1.940271163 | 0.000116384 | 0.001075232 | down |
| RNASE8 | -1.940010137 | 0.506744757 | | down |
| RP11-329A14.2 | -1.939915226 | 0.506765649 | | down |
| RP11-567M21.3 | -1.939326523 | 0.003693441 | 0.017097959 | down |
| RP11-709A23.2 | -1.939283654 | 0.013067319 | 0.044919282 | down |
| AC024082.3 | -1.93926262 | 0.263368733 | 0.398762798 | down |
| LDHD | -1.939258518 | 5.38E-08 | 1.68E-06 | down |
| NA | -1.939003032 | 0.111875942 | 0.219002269 | down |
| PPIAP30 | -1.939002587 | 0.057604745 | 0.135643622 | down |
| RP11-23J9.3 | -1.938875706 | 0.271532815 | | down |
| TEX15 | -1.938466175 | 0.074406258 | 0.163375108 | down |
| SKINT1L | -1.93844179 | 0.016037207 | 0.052528736 | down |
| NA | -1.937932472 | 0.507202209 | | down |
| FLJ40288 | -1.937813178 | 0.164962032 | 0.287628071 | down |
| AC087491.2 | -1.937572251 | 1.47E-06 | 2.85E-05 | down |
| ARHGAP19-SLIT1 | -1.937518653 | 0.2500508 | | down |
| ENAM | -1.937263733 | 0.002648857 | 0.013151919 | down |
| GAPDHP32 | -1.937236389 | 0.226964011 | | down |
| LINC01584 | -1.937122808 | 0.368985543 | | down |
| RN7SL8P | -1.937092818 | 0.051185035 | | down |
| NA | -1.937004572 | 0.020941196 | 0.064239053 | down |
| OR10K1 | -1.936971077 | 0.122239086 | 0.233089052 | down |
| CTD-2009A10.1 | -1.936728305 | 0.210477427 | 0.341041087 | down |
| HS6ST3 | -1.936539975 | 0.004332688 | 0.019394362 | down |
| GAPDHP44 | -1.936411226 | 0.167796358 | 0.291100231 | down |
| RP11-47506.1 | -1.936372205 | 7.37E-05 | 0.000730878 | down |
| CTC-453G23.7 | -1.93609152 | 0.001413272 | 0.007958825 | down |
| LINC01152 | -1.935930817 | 0.001819513 | 0.009741485 | down |
| FAAHP1 | -1.93583967 | 8.02E-07 | 1.68E-05 | down |

| | | | | |
|---|---|---|---|---|
| CICP23 | -1.935705981 | 0.06487826 | 0.147767602 | down |
| LINC00558 | -1.935703079 | 0.419080907 | 0.555037354 | down |
| BCL2L10 | -1.935488091 | 3.76E-06 | 6.25E-05 | down |
| OSTN | -1.935485681 | 0.020304625 | 0.06280574 | down |
| Y_RNA | -1.935326351 | 0.021956802 | 0.066505711 | down |
| C11orf86 | -1.935164527 | 4.01E-05 | 0.000444861 | down |
| RP11-502F1.2 | -1.934589746 | 0.241634543 | 0.375545404 | down |
| ELMO3 | -1.934556244 | 4.15E-10 | 2.39E-08 | down |
| Xbac-BPG299F13.1 | -1.934279762 | 0.008895187 | 0.033535826 | down |
| NA | -1.934199035 | 0.001263619 | 0.007297995 | down |
| AC099684.1 | -1.934066048 | 0.366416434 | | down |
| IL36A | -1.933878322 | 0.279665793 | | down |
| DLX4 | -1.933715643 | 3.32E-05 | 0.00038029 | down |
| RP11-574M7.2 | -1.933527859 | 0.508172711 | | down |
| RP4-724E16.2 | -1.933452635 | 0.000215397 | 0.001775417 | down |
| C4orf19 | -1.933443429 | 1.56E-10 | 1.00E-08 | down |
| MIR24-2 | -1.933392897 | 9.70E-08 | 2.76E-06 | down |
| LINC01365 | -1.933275434 | 0.50822835 | | down |
| RP11-1028N23.3 | -1.932954136 | 0.475019675 | | down |
| GAPDHP20 | -1.932884808 | 0.508314459 | | down |
| NA | -1.932863593 | 0.364025721 | | down |
| RN7SKP146 | -1.932644857 | 0.374794095 | 0.513115233 | down |
| RPL34P3 | -1.932280911 | 0.508447594 | | down |
| RP11-133F8.2 | -1.932078882 | 0.508334537 | | down |
| RP4-591N18.2 | -1.932024379 | 0.009647609 | 0.035646999 | down |
| DNAH3 | -1.931867354 | 1.17E-06 | 2.32E-05 | down |
| RN7SKP203 | -1.931487251 | 0.08156176 | 0.174634523 | down |
| RP11-496N12.6 | -1.931478684 | 0.020748126 | 0.063809672 | down |
| SLC4A10 | -1.931253636 | 1.13E-05 | 0.000155792 | down |
| PPIAP10 | -1.931127345 | 0.160514104 | 0.281934273 | down |
| NA | -1.930928403 | 0.508745836 | | down |
| FAM96AP1 | -1.930928403 | 0.508745836 | | down |
| RP11-312A15.1 | -1.930928403 | 0.508745836 | | down |
| MROH4P | -1.930928403 | 0.508745836 | | down |
| KIR2DP1 | -1.93087251 | 0.508680066 | | down |
| SLC25A31 | -1.930700076 | 0.369100623 | | down |
| RP11-31I22.3 | -1.930584991 | 0.177182556 | 0.302115364 | down |
| OR8B9P | -1.930554195 | 0.470735507 | | down |
| RP11-772C9.1 | -1.930510609 | 0.149104857 | 0.267892974 | down |
| ANKRD34C | -1.930156279 | 0.123582753 | 0.234831461 | down |
| RN7SL376P | -1.93008165 | 0.508627371 | | down |
| RPS7P14 | -1.930046011 | 0.005471215 | 0.023233523 | down |
| RP11-157L3.12 | -1.929963163 | 0.132813175 | | down |
| Y_RNA | -1.929420985 | 0.509078343 | | down |
| SP3P | -1.929420985 | 0.509078343 | | down |
| RNU6ATAC29P | -1.92930517 | 0.371487514 | 0.509796497 | down |
| NA | -1.929283981 | 0.509030584 | | down |
| pRNA | -1.928778724 | 0.058444056 | | down |
| RP11-480I12.5 | -1.928760934 | 4.15E-09 | 1.81E-07 | down |
| XAGE2 | -1.92875933 | 0.509224327 | | down |
| LINC00951 | -1.928739547 | 0.282278502 | | down |

| | | | | |
|---|---|---|---|---|
| DEFB108P4 | -1.928690323 | 0.508865732 | | down |
| MIR140 | -1.928566005 | 0.509266985 | | down |
| DDX3P3 | -1.92854197 | 0.466926152 | | down |
| RP11-70D24.1 | -1.928525086 | 0.009111593 | 0.034150289 | down |
| PPIAP17 | -1.928502622 | 0.146751927 | 0.265012616 | down |
| DMBX1 | -1.928385793 | 0.094844915 | 0.194693954 | down |
| GNAT1 | -1.928370308 | 0.151219677 | 0.270350728 | down |
| RN7SKP237 | -1.928216111 | 0.418762235 | | down |
| RP11-456019.2 | -1.92819663 | 0.269919233 | 0.405939502 | down |
| OR2A5 | -1.927900021 | 0.118801109 | 0.228476541 | down |
| RIPPLY1 | -1.927849427 | 0.032145308 | | down |
| TTR | -1.927817645 | 5.98E-05 | 0.000618148 | down |
| MTRNR2L4 | -1.927808411 | 0.101726633 | | down |
| AC003958.2 | -1.927315636 | 0.418124765 | | down |
| RP11-831F12.2 | -1.927028812 | 0.121369621 | 0.231902118 | down |
| CTC-344H19.6 | -1.926895664 | 0.509402596 | | down |
| P2RX3 | -1.926863265 | 0.007711442 | 0.03013169 | down |
| NA | -1.926558159 | 0.369467707 | | down |
| RN7SL341P | -1.926125316 | 0.509805634 | | down |
| PHKBP2 | -1.926125316 | 0.509805634 | | down |
| MYOC | -1.926031373 | 0.002180243 | 0.01124519 | down |
| GOLGA2P8 | -1.925986662 | 0.029728125 | 0.083253677 | down |
| CTA-440B3.1 | -1.92598503 | 0.083972267 | | down |
| LRRC4C | -1.925928994 | 9.68E-05 | 0.000920021 | down |
| SNORD74 | -1.92546319 | 0.333225702 | 0.471525668 | down |
| C1QTNF1-AS1 | -1.925321261 | 0.000277371 | 0.002189905 | down |
| CTD-2076M15.1 | -1.925283285 | 0.509991529 | | down |
| SKIDA1 | -1.925211233 | 4.20E-05 | 0.000462282 | down |
| RP3-393E18.1 | -1.92482356 | 0.000514491 | 0.003559966 | down |
| RP11-523L20.2 | -1.924500142 | 0.090577895 | | down |
| RPL32P28 | -1.924344356 | 0.112763303 | 0.220005239 | down |
| ASCL1 | -1.92415553 | 0.000258368 | 0.002065569 | down |
| RP11-248E9.1 | -1.92406641 | 0.291044636 | 0.428193157 | down |
| RPS3AP50 | -1.923709145 | 0.335570584 | 0.473750418 | down |
| CFL1P4 | -1.923136079 | 0.226020381 | | down |
| RP11-556I14.3 | -1.922889849 | 0.107693504 | 0.213256361 | down |
| DMRTC2 | -1.922824436 | 0.229132456 | 0.361557891 | down |
| RP11-3P17.4 | -1.922675031 | 0.26376446 | 0.39914406 | down |
| NA | -1.922591043 | 0.231831113 | 0.36454199 | down |
| NA | -1.922328611 | 0.209700266 | | down |
| NLRP2B | -1.922297487 | 0.050653631 | 0.12339558 | down |
| AC013429.4 | -1.922058301 | 0.510703833 | | down |
| RP11-744I24.2 | -1.922046349 | 0.031531073 | 0.086997847 | down |
| RPL17P17 | -1.921963258 | 0.478060648 | | down |
| CTD-2566J3.1 | -1.921550708 | 2.01E-07 | 5.19E-06 | down |
| RP4-639J15.3 | -1.921199959 | 0.367543605 | | down |
| ECEL1P2 | -1.921047739 | 0.000854363 | 0.005345374 | down |
| RNU6-1205P | -1.92076825 | 0.220530841 | | down |
| RN7SL845P | -1.920714296 | 0.510923415 | | down |
| KLF17P1 | -1.920469325 | 0.477663087 | | down |
| RP11-560F18.1 | -1.920331892 | 0.510852856 | | down |

| | | | | |
|---|---|---|---|---|
| NA | -1.920331892 | 0.510852856 | | down |
| RP11-254F19.4 | -1.920322594 | 0.001653366 | 0.009020105 | down |
| RNU6-1043P | -1.920282582 | 0.245105 | | down |
| SOHLH1 | -1.9202226 | 0.163105697 | 0.285268807 | down |
| ALMS1P1 | -1.920085117 | 0.000386359 | 0.002841556 | down |
| RP4-675G8.3 | -1.919802169 | 0.511202452 | | down |
| HAPLN1 | -1.919653647 | 1.07E-05 | 0.000149539 | down |
| RP11-806L2.2 | -1.919252309 | 0.216403646 | | down |
| ONECUT2 | -1.919165781 | 0.00049552 | 0.003450949 | down |
| CH17-26202.1 | -1.91916108 | 0.511344183 | | down |
| RP3-465N24.6 | -1.919127879 | 4.16E-06 | 6.83E-05 | down |
| UPF3AP1 | -1.918954192 | 0.008021123 | 0.031027772 | down |
| PCDHB1 | -1.918621195 | 0.035683469 | 0.095363294 | down |
| FRRS1L | -1.918348637 | 0.000312703 | 0.002413084 | down |
| Y_RNA | -1.917967883 | 0.511608027 | | down |
| CTD-2649C14.1 | -1.917318553 | 0.091873458 | 0.190269951 | down |
| RNA5SP384 | -1.917266814 | 0.369011528 | | down |
| PRKCA-AS1 | -1.917264249 | 0.035158943 | 0.094365896 | down |
| PDILT | -1.917093165 | 0.369239589 | | down |
| RP11-391J13.1 | -1.916487339 | 0.511317925 | | down |
| CCL15 | -1.915905503 | 1.88E-05 | 0.00023809 | down |
| SULT2B1 | -1.91583398 | 5.00E-07 | 1.12E-05 | down |
| MUPP | -1.915812697 | 0.512084769 | | down |
| RP11-316M1.3 | -1.915784378 | 0.007082404 | 0.028239383 | down |
| AC106869.2 | -1.915781244 | 0.000131043 | 0.001183006 | down |
| RNU6-731P | -1.915707395 | 0.253913044 | | down |
| CYP51A1P3 | -1.915559717 | 0.123882394 | 0.235222313 | down |
| KRT18P35 | -1.915516487 | 0.119713481 | 0.229775239 | down |
| PCYT1B | -1.914887658 | 2.81E-05 | 0.000331406 | down |
| TIMM17BP1 | -1.914871929 | 0.479203549 | | down |
| RPL22P19 | -1.91472864 | 0.213894171 | | down |
| GOLGA2P11 | -1.914599345 | 0.183955585 | 0.309796054 | down |
| RP11-864N7.1 | -1.914056945 | 0.511855559 | | down |
| NA | -1.914033947 | 0.261143155 | 0.396331832 | down |
| HMGN2P31 | -1.913901639 | 0.074993682 | 0.164297528 | down |
| CTC-305H11.1 | -1.913892547 | 0.512509712 | | down |
| OR9A4 | -1.913872753 | 0.084066159 | 0.178493546 | down |
| RP11-1090M7.3 | -1.913795693 | 0.094547452 | 0.194307657 | down |
| AC005540.3 | -1.913783812 | 0.0163346 | 0.053227409 | down |
| ZBED3-AS1 | -1.913575324 | 8.22E-06 | 0.000120169 | down |
| RPL21P67 | -1.913474418 | 0.472285499 | | down |
| RP4-799D16.1 | -1.913469437 | 0.250705566 | | down |
| PI16 | -1.913351367 | 1.04E-05 | 0.00014637 | down |
| BRWD1P1 | -1.913235308 | 0.279477625 | 0.415756871 | down |
| AC005281.2 | -1.912838539 | 0.145140019 | | down |
| C2orf27B | -1.912227454 | 0.140591959 | | down |
| RP11-373N22.3 | -1.91176027 | 1.67E-05 | 0.000216398 | down |
| AC003986.5 | -1.911401116 | 0.512829537 | | down |
| CA14 | -1.911022156 | 8.61E-08 | 2.51E-06 | down |
| RP11-202G11.2 | -1.910602942 | 0.225376995 | 0.357452698 | down |
| RP11-274B18.3 | -1.910427344 | 0.479530221 | | down |

| | | | | |
|---|---|---|---|---|
| FOXO1B | -1.909989839 | 0.513373973 | | down |
| RP1-241P17.7 | -1.909776337 | 0.195092786 | 0.323510744 | down |
| MPL | -1.909533349 | 7.28E-07 | 1.55E-05 | down |
| TLX1NB | -1.90943649 | 0.135104884 | | down |
| RP11-96K19.4 | -1.90932687 | 0.000970181 | 0.005906407 | down |
| ZNRF2P3 | -1.909207706 | 0.513547268 | | down |
| RP11-469N6.2 | -1.909207706 | 0.513547268 | | down |
| RP11-138H10.2 | -1.909207706 | 0.513547268 | | down |
| RP11-266I3.8 | -1.909207706 | 0.513547268 | | down |
| RP11-214K3.22 | -1.909065179 | 0.121630458 | | down |
| PIPSL | -1.908597687 | 2.44E-08 | 8.38E-07 | down |
| NA | -1.908492112 | 2.15E-07 | 5.47E-06 | down |
| PPBPP2 | -1.908470433 | 0.32381502 | | down |
| POM121L6P | -1.908148834 | 0.243671958 | | down |
| RN7SKP206 | -1.908103889 | 0.513791889 | | down |
| MIR4508 | -1.907965402 | 0.513822583 | | down |
| AC004840.8 | -1.907947608 | 0.253457431 | | down |
| PPIL6 | -1.907903627 | 1.66E-08 | 6.14E-07 | down |
| ISCA2P1 | -1.907861599 | 0.291070351 | | down |
| RP11-229M1.2 | -1.907840387 | 0.482730398 | | down |
| RP11-267A15.3 | -1.907637978 | 0.513895159 | | down |
| TRPC2 | -1.907527323 | 0.000275616 | 0.002177392 | down |
| TCEA1P3 | -1.907400035 | 0.478127516 | | down |
| RP11-25I9.3 | -1.907366372 | 0.513955366 | | down |
| RP5-1039K5.16 | -1.907259005 | 0.092788606 | 0.191584143 | down |
| LINC01483 | -1.906796872 | 0.019792997 | 0.061584589 | down |
| RP11-536C10.7 | -1.906380428 | 0.483297164 | | down |
| RP11-1109M24.5 | -1.906376453 | 0.390113223 | 0.527858119 | down |
| LINC01487 | -1.905779002 | 0.372038825 | | down |
| RN7SL581P | -1.905128697 | 0.480624874 | | down |
| CTD-3051D23.4 | -1.905100024 | 0.001819638 | 0.009741485 | down |
| MRPL2 | -1.904955845 | 1.11E-05 | 0.000153194 | down |
| ZNF705G | -1.904730586 | 0.484311331 | | down |
| C9orf147 | -1.904554513 | 2.59E-05 | 0.000309824 | down |
| C1orf115 | -1.904245777 | 5.73E-14 | 8.87E-12 | down |
| DUSP18 | -1.903909678 | 5.76E-08 | 1.79E-06 | down |
| CTD-2210P24.3 | -1.903904115 | 0.329766025 | | down |
| CACNG4 | -1.903730194 | 0.000368807 | 0.002741716 | down |
| CCR9 | -1.903685094 | 0.000309225 | 0.002389491 | down |
| KHDC3L | -1.903595282 | 0.329325457 | | down |
| RP11-653B10.1 | -1.903558414 | 0.514723646 | | down |
| C9orf173-AS1 | -1.903414797 | 2.84E-05 | 0.000334595 | down |
| RTEL1P1 | -1.903354784 | 0.067184075 | 0.151708255 | down |
| LINC01277 | -1.903289907 | 0.000829465 | 0.005229893 | down |
| RNU6-523P | -1.90316379 | 0.472563182 | | down |
| KIAA1024L | -1.903037393 | 0.119940078 | 0.230092687 | down |
| ODF3 | -1.902876437 | 0.04104087 | 0.105877256 | down |
| ENTPD8 | -1.902832318 | 6.28E-07 | 1.37E-05 | down |
| CYCSP28 | -1.902827491 | 0.196784116 | 0.325399415 | down |
| HTR3B | -1.902748263 | 0.010766482 | 0.038813077 | down |
| C1QL2 | -1.902701642 | 0.149484569 | | down |

| | | | | |
|---|---|---|---|---|
| RP11-567018.1 | -1.902682057 | 0.016734736 | 0.054191753 | down |
| RPL12P32 | -1.902678362 | 0.108043411 | 0.21374586 | down |
| RP11-426C22.7 | -1.902505747 | 0.012662847 | 0.043936088 | down |
| ATP6V0CP1 | -1.902381644 | 0.051632034 | 0.12520039 | down |
| RP11-179H18.8 | -1.902365999 | 0.231503981 | 0.364113999 | down |
| UPK1A-AS1 | -1.902354786 | 0.327344664 | | down |
| TNN | -1.902309745 | 6.76E-06 | 0.000102042 | down |
| INGX | -1.90196927 | 0.251302667 | | down |
| INTS6P1 | -1.90192715 | 0.286559895 | | down |
| RPL7AP34 | -1.901865129 | 0.05644051 | | down |
| LRRC19 | -1.901814904 | 5.01E-08 | 1.58E-06 | down |
| RP11-499F3.2 | -1.901297923 | 0.001685756 | 0.009153597 | down |
| U2 | -1.901207164 | 0.036587654 | 0.097204339 | down |
| CRPP1 | -1.901048534 | 0.515356873 | | down |
| RP11-7G12.2 | -1.901048534 | 0.515356873 | | down |
| RN7SL582P | -1.901048534 | 0.515356873 | | down |
| RP11-69L16.6 | -1.901048534 | 0.515356873 | | down |
| RN7SL39P | -1.901048534 | 0.515356873 | | down |
| CARS-AS1 | -1.90057187 | 0.125684048 | | down |
| MTND5P10 | -1.900439338 | 0.076726277 | 0.167125663 | down |
| CNNM1 | -1.900316271 | 4.24E-06 | 6.94E-05 | down |
| NR1I2 | -1.900043696 | 7.56E-09 | 3.08E-07 | down |
| RNU7-97P | -1.899855178 | 0.198377022 | 0.327133172 | down |
| RP11-193P11.3 | -1.899826354 | 0.478635415 | | down |
| RP11-90J7.3 | -1.899729848 | 0.157206383 | | down |
| PAQR9-AS1 | -1.899607905 | 0.296917864 | | down |
| MIR320A | -1.899401082 | 0.073863874 | 0.162552463 | down |
| RPL23AP92 | -1.899035061 | 0.515728045 | | down |
| NA | -1.898896073 | 0.068165359 | 0.153337538 | down |
| RP11-510M2.4 | -1.898820227 | 0.515775773 | | down |
| RP11-638D14.1 | -1.898435775 | 0.515937045 | | down |
| NA | -1.898311522 | 0.515964644 | | down |
| NA | -1.898289864 | 0.000749109 | 0.004819318 | down |
| NA | -1.898036266 | 0.471998584 | | down |
| MNX1-AS1 | -1.897625661 | 1.55E-06 | 2.98E-05 | down |
| AC018495.3 | -1.897622468 | 0.515886957 | | down |
| NA | -1.897596825 | 0.02709798 | 0.077870082 | down |
| MT1G | -1.897532328 | 2.68E-07 | 6.62E-06 | down |
| DIRAS2 | -1.897505147 | 0.000923112 | 0.005692525 | down |
| WNT9B | -1.897300046 | 0.00833123 | 0.031902048 | down |
| LEXM | -1.897247592 | 0.000144742 | 0.001281646 | down |
| RP11-879F14.3 | -1.897037269 | 0.214799657 | | down |
| NA | -1.896848292 | 0.116145608 | | down |
| NA | -1.896391021 | 0.062034723 | 0.143278846 | down |
| RP11-379B18.6 | -1.896353595 | 0.010655775 | 0.038497128 | down |
| RP11-21J7.1 | -1.896332259 | 0.383440565 | 0.521153057 | down |
| RP11-17G12.3 | -1.896286092 | 0.093383565 | | down |
| RP11-649A18.4 | -1.895556059 | 0.001658411 | 0.009031228 | down |
| LINC00581 | -1.895336163 | 0.516322149 | | down |
| RP11-748H22.1 | -1.894732423 | 0.382149099 | | down |
| NEK4P2 | -1.894682122 | 0.516617379 | | down |

| | | | | |
|---|---|---|---|---|
| FMO5 | -1.894206052 | 4.94E-13 | 5.98E-11 | down |
| RP11-730B22.1 | -1.894158774 | 0.022671904 | 0.068108657 | down |
| CSMD2-AS1 | -1.89406831 | 0.516753906 | | down |
| RP11-428C19.4 | -1.894064005 | 0.027644433 | 0.079032195 | down |
| FGF8 | -1.893817719 | 0.006413894 | 0.026265264 | down |
| Y_RNA | -1.893712802 | 0.195798078 | | down |
| AC004893.10 | -1.893662389 | 0.075692204 | | down |
| AMT | -1.893492554 | 9.33E-06 | 0.000133534 | down |
| FMN2 | -1.893491974 | 0.000179226 | 0.001529301 | down |
| CPNE6 | -1.89305392 | 5.06E-05 | 0.000540868 | down |
| RP11-1023L17.2 | -1.892996326 | 0.000238967 | 0.001937791 | down |
| RP11-13J8.1 | -1.89297224 | 0.015818886 | 0.051998097 | down |
| RP11-637O11.2 | -1.892897751 | 0.067367657 | 0.151985725 | down |
| PIH1D2 | -1.892605714 | 8.99E-06 | 0.000129631 | down |
| PATE1 | -1.892464894 | 0.480416163 | | down |
| PTBP1P | -1.892347443 | 0.000756803 | 0.004857802 | down |
| EXOC3L4 | -1.892274485 | 4.90E-10 | 2.76E-08 | down |
| RP11-963H1.1 | -1.89202337 | 0.517362365 | | down |
| Y_RNA | -1.891985564 | 0.388378234 | 0.526031722 | down |
| CTD-2286N8.1 | -1.891458063 | 0.007657938 | 0.029983925 | down |
| RP11-420K10.1 | -1.89143385 | 0.11416651 | 0.22206517 | down |
| FAM189A1 | -1.891100907 | 7.10E-07 | 1.52E-05 | down |
| C7orf77 | -1.891053866 | 0.485310531 | | down |
| EFCAB6 | -1.891039808 | 3.25E-05 | 0.000373481 | down |
| RP11-689C9.1 | -1.890753899 | 0.170484769 | 0.294361381 | down |
| TECRP1 | -1.890707819 | 3.56E-05 | 0.00040367 | down |
| RP4-603I14.3 | -1.890593933 | 0.201744692 | 0.330906672 | down |
| FAM217A | -1.890577776 | 0.164866925 | 0.28752899 | down |
| RP11-586K12.4 | -1.890472029 | 0.424999294 | | down |
| CLDN3 | -1.89044629 | 6.74E-06 | 0.000101896 | down |
| NA | -1.889870915 | 0.012110786 | 0.042469842 | down |
| NA | -1.889856905 | 0.000326852 | 0.002499349 | down |
| NA | -1.889685539 | 0.181281716 | | down |
| KRT8P11 | -1.889675218 | 0.151783246 | | down |
| RP11-531H8.2 | -1.889656093 | 0.146341239 | | down |
| RDH8 | -1.889653062 | 0.062905283 | 0.144688894 | down |
| SLC19A3 | -1.889491646 | 8.02E-10 | 4.23E-08 | down |
| SNORA62 | -1.888759443 | 0.518088632 | | down |
| RP11-68I3.7 | -1.88874192 | 0.253854725 | | down |
| RP11-330M19.1 | -1.888570029 | 0.016915853 | 0.054606199 | down |
| RP11-678G14.4 | -1.888508476 | 0.326950458 | | down |
| NA | -1.887598845 | 0.488514399 | | down |
| LINC00484 | -1.887354488 | 3.60E-05 | 0.000407341 | down |
| PAX1 | -1.887289671 | 0.215593202 | 0.346693901 | down |
| RPL7P48 | -1.887193941 | 0.292445962 | | down |
| RP11-627G23.1 | -1.886778336 | 2.95E-07 | 7.16E-06 | down |
| FAM196B | -1.886571808 | 0.003928483 | 0.017977287 | down |
| EPS15P1 | -1.88640367 | 0.152325106 | 0.271793295 | down |
| RP11-44N21.1 | -1.886389921 | 1.22E-09 | 6.07E-08 | down |
| RPS14P4 | -1.886307589 | 0.036770335 | 0.097568112 | down |
| SNCA-AS1 | -1.886298607 | 0.518406677 | | down |

| | | | | |
|---|---|---|---|---|
| YBX1P5 | -1.886259963 | 0.518492291 | | down |
| NOC2LP2 | -1.885804879 | 0.019068561 | 0.059882496 | down |
| RP3-399J4.4 | -1.885731165 | 0.279271145 | 0.415532006 | down |
| COL24A1 | -1.885519131 | 0.000133003 | 0.001197825 | down |
| LINC00459 | -1.88513688 | 0.478791101 | | down |
| NA | -1.884674426 | 0.097585118 | 0.198527038 | down |
| NA | -1.884663843 | 0.258395789 | | down |
| AC000370.2 | -1.884305839 | 0.377698552 | | down |
| RP11-871F6.3 | -1.884198178 | 0.172862333 | 0.297194617 | down |
| RP11-295K3.1 | -1.884136109 | 0.00031391 | 0.002418966 | down |
| RP11-351N6.1 | -1.88391181 | 0.519168258 | | down |
| RP11-463I20.3 | -1.883792138 | 0.488099093 | | down |
| RP11-501C14.6 | -1.883760113 | 0.519202062 | | down |
| SHOX2 | -1.883530246 | 0.002256343 | 0.011580042 | down |
| RP11-560I19.1 | -1.883300426 | 0.200264227 | 0.329341423 | down |
| SLC1A7 | -1.883279243 | 5.61E-07 | 1.24E-05 | down |
| SMIM21 | -1.882971449 | 0.519303062 | | down |
| C11orf52 | -1.882921458 | 0.519314207 | | down |
| RP11-521O16.2 | -1.882786926 | 0.184843433 | 0.310890449 | down |
| RP11-950K24.2 | -1.882602127 | 0.078505731 | 0.169856415 | down |
| RP11-498M14.1 | -1.882450555 | 0.519493924 | | down |
| GUSBP10 | -1.882363317 | 0.37791434 | | down |
| MIR219A1 | -1.88232386 | 0.004503669 | 0.020007133 | down |
| RPS20P32 | -1.882079182 | 0.304313956 | | down |
| RP11-140C18.2 | -1.882056939 | 0.519581665 | | down |
| LINC00967 | -1.882047191 | 0.32684172 | | down |
| DDX11L1 | -1.881470573 | 0.140640994 | | down |
| GCNT4 | -1.881087365 | 1.20E-07 | 3.31E-06 | down |
| KRT8P35 | -1.881016724 | 0.057846066 | | down |
| FBXO40 | -1.881004415 | 0.000927384 | 0.005711834 | down |
| RP4-580O19.2 | -1.880831657 | 0.170551947 | 0.294413702 | down |
| HCN1 | -1.880823987 | 0.014328104 | 0.048237738 | down |
| LL22NC03-63E9.3 | -1.880671366 | 0.431790331 | | down |
| LUZP2 | -1.88031596 | 2.47E-05 | 0.000297635 | down |
| RP11-141C7.5 | -1.880283103 | 0.013618368 | 0.046388918 | down |
| NA | -1.88017413 | 0.096808679 | | down |
| MIR5087 | -1.880108866 | 0.336706336 | | down |
| RP11-481G8.2 | -1.880073986 | 0.298823276 | 0.436368548 | down |
| RP1-283K11.3 | -1.880005666 | 0.022110995 | 0.066851438 | down |
| ACADL | -1.879916054 | 0.005927105 | 0.02472219 | down |
| ENPP3 | -1.879864931 | 1.41E-08 | 5.42E-07 | down |
| RP11-118G23.2 | -1.87975229 | 0.485426725 | | down |
| DNAJC19P7 | -1.879677288 | 0.489843061 | | down |
| KCNQ3 | -1.879615047 | 2.70E-05 | 0.00032129 | down |
| RPL12P11 | -1.879128638 | 0.294656097 | | down |
| CTB-139P11.2 | -1.878952031 | 0.236281448 | | down |
| RP11-486A14.2 | -1.878942185 | 0.001491095 | 0.008297546 | down |
| RP6-24A23.7 | -1.8787293 | 0.481942395 | | down |
| RP11-63D14.1 | -1.878369878 | 0.520403924 | | down |
| AC093162.3 | -1.8782797 | 0.338753628 | | down |
| RP11-575B7.3 | -1.8782595 | 0.520354125 | | down |

| | | | | |
|---|---|---|---|---|
| KIR3DX1 | -1.877944214 | 0.00155946 | 0.00860309 | down |
| RP11-436F23.1 | -1.877816024 | 0.382739846 | | down |
| NA | -1.877647535 | 0.379421124 | | down |
| RP11-163O19.16 | -1.877108546 | 0.520685369 | | down |
| RP11-404O13.4 | -1.877094002 | 0.198954212 | | down |
| RP11-14C22.6 | -1.876853091 | 0.125439943 | | down |
| MTCO1P20 | -1.876553622 | 0.430452728 | | down |
| VWA3B | -1.876068538 | 2.99E-07 | 7.25E-06 | down |
| DNASE1 | -1.876038895 | 3.14E-13 | 4.00E-11 | down |
| RN7SL174P | -1.876008461 | 0.145443403 | 0.263171621 | down |
| RP11-336A10.5 | -1.875579709 | 1.60E-05 | 0.000210086 | down |
| ACSM5P1 | -1.875575078 | 0.208202861 | | down |
| BTNL2 | -1.875510314 | 0.005483111 | 0.023264312 | down |
| AC067956.1 | -1.875504513 | 0.162793332 | 0.284846547 | down |
| RP11-403.1 | -1.875380137 | 0.52107116 | | down |
| AC126365.1 | -1.87536063 | 0.118237726 | 0.227669951 | down |
| RP11-4B14.3 | -1.874897107 | 0.082835206 | 0.17675259 | down |
| CELF6 | -1.874622632 | 0.485058396 | | down |
| RP11-963H4.2 | -1.874213039 | 0.293351009 | | down |
| MYL10 | -1.874197968 | 0.338385308 | | down |
| CTB-147C22.3 | -1.87399349 | 0.521380774 | | down |
| CTB-22K14.1 | -1.873936604 | 0.148727034 | 0.267422048 | down |
| NCRNA00250 | -1.87381005 | 0.290854226 | | down |
| RNU6-1165P | -1.873434401 | 0.243513551 | 0.377547792 | down |
| CTB-1144G6.5 | -1.873088931 | 0.225604206 | 0.357682215 | down |
| RP13-653N12.1 | -1.872977065 | 0.521607783 | | down |
| SRP72P2 | -1.872952263 | 0.007303685 | 0.028884023 | down |
| OR6K1P | -1.87294023 | 0.521616011 | | down |
| AC079834.1 | -1.872820764 | 0.179695596 | 0.304915027 | down |
| NOL4 | -1.872777304 | 0.00152457 | 0.008439182 | down |
| RP5-1158E12.3 | -1.872663122 | 0.00049074 | 0.003427612 | down |
| NA | -1.872476145 | 0.242660647 | 0.376555781 | down |
| FKBP4P6 | -1.872338465 | 0.433952058 | | down |
| RP11-263C24.1 | -1.872088466 | 0.381325969 | | down |
| CTD-2024F21.1 | -1.871835831 | 0.152811043 | | down |
| RP11-121C6.5 | -1.871581815 | 0.005569528 | 0.023564415 | down |
| EFCAB5 | -1.871512637 | 3.43E-06 | 5.82E-05 | down |
| UNC93B3 | -1.871434522 | 0.02666428 | 0.076918063 | down |
| SEMA3B-AS1 | -1.871403939 | 2.35E-06 | 4.22E-05 | down |
| CACNG5 | -1.870869156 | 0.00804643 | 0.031089872 | down |
| RP11-96C23.13 | -1.870855812 | 0.061506132 | 0.142410087 | down |
| RP11-1072C15.6 | -1.870516752 | 0.032519701 | 0.089019814 | down |
| SAXO1 | -1.870482114 | 0.00019462 | 0.001633287 | down |
| NA | -1.870339861 | 0.090463703 | | down |
| RP11-343K8.3 | -1.870307641 | 0.382329839 | | down |
| NA | -1.870304809 | 0.029727111 | 0.083253677 | down |
| NA | -1.870244812 | 0.000148694 | 0.00131085 | down |
| AF146191.4 | -1.870238541 | 9.47E-05 | 0.000903086 | down |
| KRT12 | -1.870144732 | 0.000323402 | 0.002477384 | down |
| BTF3P4 | -1.870123876 | 0.12329176 | | down |
| S100A7L2 | -1.870018004 | 0.491314375 | | down |

| | | | | |
|---|---|---|---|---|
| NA | -1.869509758 | 0.522382553 | | down |
| MRPL49P1 | -1.869355959 | 0.490830975 | | down |
| CTB-131B5.2 | -1.86918274 | 0.008872696 | 0.033480443 | down |
| RPL23AP48 | -1.868995886 | 0.110637707 | 0.217269055 | down |
| NA | -1.868693541 | 0.26656639 | | down |
| NA | -1.868584174 | 0.14427966 | 0.261756051 | down |
| EPHA6 | -1.868510002 | 0.025271285 | 0.073892992 | down |
| RP11-63E16.1 | -1.868421792 | 0.334066019 | | down |
| FOXH1 | -1.868417825 | 0.000723296 | 0.004686369 | down |
| EIF2S2P3 | -1.868391986 | 0.000682154 | 0.004466032 | down |
| CTC-55802.2 | -1.868351172 | 0.04274316 | | down |
| MLIP | -1.867491107 | 0.001522766 | 0.008434793 | down |
| RP11-379P15.1 | -1.86748179 | 0.522835975 | | down |
| RP11-472F14.4 | -1.867260474 | 0.52288547 | | down |
| RP11-362F19.1 | -1.866859803 | 0.015050499 | 0.050069448 | down |
| RP11-16C1.3 | -1.866849594 | 0.342096621 | | down |
| EPN3 | -1.866844111 | 2.81E-07 | 6.89E-06 | down |
| RP11-787D11.1 | -1.866684598 | 0.221200949 | 0.352822325 | down |
| RP11-437J2.4 | -1.866558606 | 0.333578386 | | down |
| RP11-364L4.3 | -1.86647074 | 0.384127661 | | down |
| RP11-350D17.2 | -1.866341366 | 0.523091044 | | down |
| RP11-123M21.1 | -1.8662964 | 0.523101102 | | down |
| RP11-146F11.2 | -1.866179181 | 0.523053526 | | down |
| LINC00853 | -1.866153726 | 3.03E-05 | 0.000353144 | down |
| RP11-109D20.1 | -1.866142699 | 0.523061689 | | down |
| RP11-259F16.3 | -1.866047941 | 0.522854579 | | down |
| RP11-297J22.1 | -1.865952786 | 0.086691014 | 0.182621289 | down |
| NA | -1.865632594 | 0.311258526 | | down |
| RNU6-262P | -1.865578934 | 0.523033381 | | down |
| EPO | -1.865349505 | 0.002293156 | 0.01174182 | down |
| RP11-103J8.1 | -1.865217769 | 0.017555975 | 0.056291539 | down |
| RNU6-1307P | -1.865131968 | 0.43280888 | | down |
| CHD5 | -1.865120035 | 4.09E-05 | 0.000452725 | down |
| MIR4521 | -1.864908759 | 0.295699016 | | down |
| MIR5588 | -1.864867455 | 0.493417732 | | down |
| NA | -1.86477081 | 0.096023307 | | down |
| Metazoa_SRP | -1.864636397 | 0.43656225 | | down |
| AC013472.3 | -1.864607601 | 0.035389132 | 0.094770575 | down |
| MYLK3 | -1.864386835 | 7.82E-05 | 0.000769943 | down |
| RP11-12704.2 | -1.864301331 | 0.523319357 | | down |
| KCNJ16 | -1.864053906 | 0.001845832 | 0.009846623 | down |
| RPL38P1 | -1.863836379 | 0.523651535 | | down |
| DLK1 | -1.863816461 | 0.162071788 | 0.283972986 | down |
| RP11-28H5.2 | -1.863582102 | 0.000870785 | 0.005420386 | down |
| FER1L6-AS1 | -1.863509409 | 0.235243811 | 0.368088384 | down |
| RFPL1S | -1.86314891 | 3.24E-05 | 0.000372443 | down |
| RP3-400B16.3 | -1.862735527 | 0.301824681 | | down |
| RP11-887P2.6 | -1.86243354 | 0.007150706 | 0.028436241 | down |
| RP11-38J22.3 | -1.86241816 | 0.227432432 | | down |
| C1orf234 | -1.862323822 | 0.110497749 | 0.217079341 | down |
| RP11-294J22.7 | -1.862238901 | 0.013473484 | 0.045978799 | down |

| | | | | |
|---|---|---|---|---|
| RP11-5P22.1 | -1.86198957 | 0.179681932 | | down |
| LINC01224 | -1.861621873 | 8.79E-09 | 3.53E-07 | down |
| NA | -1.861311161 | 0.117704176 | 0.226970115 | down |
| HERC2P5 | -1.86098592 | 0.141142073 | 0.257771511 | down |
| RP4-784A16.4 | -1.86087955 | 0.439915321 | | down |
| RP11-9502.1 | -1.860742606 | 0.064493531 | 0.147216704 | down |
| MYPN | -1.860723969 | 0.002722315 | 0.013445542 | down |
| MAPK4 | -1.860571019 | 0.00313228 | 0.014978766 | down |
| RP11-1396013.15 | -1.860498295 | 0.524171089 | | down |
| RP11-118A1.2 | -1.860302204 | 0.181230039 | 0.306723828 | down |
| ACTN4P2 | -1.859965445 | 0.38973101 | | down |
| NA | -1.859884789 | 0.180010831 | 0.305255532 | down |
| RP11-128D14.1 | -1.859550989 | 0.524537497 | | down |
| AC015849.16 | -1.859548331 | 0.004709579 | 0.020716629 | down |
| RP11-699L21.1 | -1.859527406 | 0.00151496 | 0.008402335 | down |
| OR2A25 | -1.859363739 | 0.349519158 | | down |
| NCBP2L | -1.858908894 | 0.25839051 | | down |
| RP5-855F14.2 | -1.858794245 | 0.524707041 | | down |
| SCGB2A1 | -1.858618985 | 0.000258726 | 0.002067878 | down |
| TPH2 | -1.858583307 | 0.0874343 | 0.183722722 | down |
| AL360294.1 | -1.858543932 | 0.073630171 | 0.162219055 | down |
| AC083862.6 | -1.858274053 | 0.050579803 | 0.123319005 | down |
| CTD-2561J22.1 | -1.858065481 | 0.149749427 | 0.268681504 | down |
| RP11-29A19.2 | -1.858020404 | 0.072636304 | | down |
| RP11-349E4.1 | -1.857971722 | 0.487719711 | | down |
| RP11-115M14.1 | -1.857965562 | 0.342722334 | | down |
| RP11-632K5.2 | -1.857962081 | 0.149378756 | | down |
| RP1-292B18.4 | -1.857870382 | 0.387920493 | | down |
| COTL1P2 | -1.857846187 | 0.524993079 | | down |
| NA | -1.857707425 | 1.63E-05 | 0.000212671 | down |
| GJB1 | -1.857682292 | 2.05E-07 | 5.26E-06 | down |
| GK-IT1 | -1.857562469 | 0.003672144 | 0.017016555 | down |
| RP11-753H16.5 | -1.857548495 | 0.049844861 | 0.122028929 | down |
| RP11-679I18.4 | -1.857241343 | 0.525128634 | | down |
| RP11-118D22.3 | -1.85700805 | 0.525180924 | | down |
| RP11-157L10.1 | -1.85700805 | 0.525180924 | | down |
| CTD-2526A2.4 | -1.856985057 | 0.23269288 | | down |
| Y_RNA | -1.856725916 | 7.41E-05 | 0.000734698 | down |
| NA | -1.856572561 | 0.002524895 | 0.012674588 | down |
| DLGAP1-AS3 | -1.856528609 | 0.061617932 | 0.142598529 | down |
| KRT8P44 | -1.85649482 | 0.440831376 | | down |
| BAIAP2L2 | -1.856278522 | 5.04E-11 | 3.73E-09 | down |
| MIR5787 | -1.856168143 | 0.525295618 | | down |
| NA | -1.856038306 | 0.195387419 | 0.32390272 | down |
| KCNH5 | -1.855968236 | 0.094715399 | 0.194506457 | down |
| CTD-3088G3.6 | -1.855413651 | 0.131511373 | 0.245289774 | down |
| HSD11B2 | -1.855166592 | 9.09E-07 | 1.87E-05 | down |
| OR9G1 | -1.855078413 | 0.525613529 | | down |
| RP11-424I19.1 | -1.855042569 | 0.525547963 | | down |
| RP11-159H20.3 | -1.855009518 | 0.490266585 | | down |
| NA | -1.85490829 | 0.042403487 | 0.108552349 | down |

| | | | | |
|---|---|---|---|---|
| AF121897.4 | -1.854897531 | 0.496306844 | | down |
| CTD-3032J10.3 | -1.854786654 | 0.084523649 | 0.179224257 | down |
| C16orf95 | -1.854723066 | 2.18E-06 | 4.00E-05 | down |
| FOXD1 | -1.854343416 | 0.000858401 | 0.005361706 | down |
| DARS-AS1 | -1.854288859 | 4.11E-05 | 0.000454145 | down |
| RNU7-197P | -1.854230093 | 0.09388536 | 0.193303711 | down |
| AC008155.1 | -1.854211153 | 0.026907376 | 0.077440866 | down |
| RP11-252K23.1 | -1.854040381 | 0.222318466 | 0.354209895 | down |
| AOX2P | -1.853869513 | 0.351337207 | | down |
| CLPSL2 | -1.853770785 | 0.182853915 | | down |
| CLDN9 | -1.853591801 | 0.001282942 | 0.007374155 | down |
| RP11-486M23.1 | -1.853446869 | 0.0015245 | 0.008439182 | down |
| RP11-93N20.1 | -1.853036238 | 0.351090284 | 0.489769072 | down |
| LANCL3 | -1.852738105 | 1.07E-07 | 2.99E-06 | down |
| NA | -1.852701285 | 0.030522805 | 0.084878484 | down |
| RNU5A-1 | -1.852675245 | 0.005752536 | 0.024141526 | down |
| CTC-512J14.5 | -1.852663013 | 0.096971604 | | down |
| NAP1L6 | -1.852625892 | 0.054630996 | 0.130492419 | down |
| DNM1P51 | -1.852587014 | 0.007171685 | 0.028500132 | down |
| NAGS | -1.852226615 | 8.26E-09 | 3.34E-07 | down |
| HSPA8P11 | -1.852071199 | 0.083660574 | 0.177940385 | down |
| TAF1L | -1.851995342 | 0.219082947 | 0.350542049 | down |
| KNG1 | -1.851964482 | 0.043752151 | 0.111032494 | down |
| RP11-83C7.1 | -1.851893502 | 0.060866312 | | down |
| CXorf58 | -1.851733416 | 0.038306306 | 0.10063612 | down |
| RP11-1147P1.1 | -1.851627498 | 0.49537178 | | down |
| RP5-1021I20.5 | -1.851548553 | 0.012072516 | 0.042367325 | down |
| CTB-30L5.1 | -1.851488682 | 0.002721808 | 0.013445542 | down |
| WARSP1 | -1.850599755 | 0.028669497 | 0.081228544 | down |
| LINC01250 | -1.8504229 | 0.253822235 | 0.388854992 | down |
| GNL2P1 | -1.850187949 | 0.52671073 | | down |
| LMOD2 | -1.850174328 | 0.302369373 | | down |
| SPIRE2 | -1.849591386 | 4.29E-11 | 3.26E-09 | down |
| NPBWR2 | -1.849458028 | 0.497574447 | | down |
| RP11-47304.4 | -1.849360259 | 0.151265877 | | down |
| RP11-396J6.1 | -1.849340957 | 0.224312935 | | down |
| SRGAP3-AS2 | -1.849246049 | 0.38753784 | | down |
| NA | -1.849134182 | 1.30E-05 | 0.000175064 | down |
| WNT7A | -1.849052936 | 0.00727143 | 0.028779191 | down |
| TF | -1.849045504 | 1.41E-06 | 2.74E-05 | down |
| PRKACG | -1.849022267 | 0.388176686 | | down |
| LINC01492 | -1.848804784 | 0.126445421 | 0.23864101 | down |
| OR2S1P | -1.848346396 | 3.90E-05 | 0.000435247 | down |
| CDHR3 | -1.84827014 | 5.96E-13 | 7.05E-11 | down |
| NA | -1.848266235 | 0.045885406 | 0.114805225 | down |
| AC078883.4 | -1.847938472 | 0.019836891 | 0.061668103 | down |
| RP11-6E9.4 | -1.84780597 | 0.527245559 | | down |
| OR6C6 | -1.84779418 | 0.527248207 | | down |
| CNTN2 | -1.847065039 | 7.35E-06 | 0.000109466 | down |
| RP11-324P9.1 | -1.847021101 | 0.52734803 | | down |
| ABCC8 | -1.846655585 | 5.28E-06 | 8.29E-05 | down |

| | | | | |
|---|---|---|---|---|
| NA | -1.846176775 | 0.014335826 | 0.048258325 | down |
| NA | -1.846117565 | 0.218661664 | | down |
| EPHA7 | -1.845893351 | 4.72E-06 | 7.58E-05 | down |
| HNRNPA1P3 | -1.84554892 | 0.527525985 | | down |
| BOLA3P4 | -1.845529525 | 0.198647381 | 0.327484448 | down |
| MARK2P4 | -1.84548805 | 0.527692411 | | down |
| EEF1DP5 | -1.844561235 | 0.135000295 | 0.249704418 | down |
| PRELID1P3 | -1.844450249 | 0.299577098 | | down |
| AP000560.3 | -1.844438244 | 0.03821256 | 0.100468903 | down |
| FAM183A | -1.843832327 | 0.003527946 | 0.016476544 | down |
| GPR156 | -1.843796626 | 0.003718536 | 0.017192934 | down |
| XXbac-BPG55C20.7 | -1.843792134 | 0.005228339 | 0.022446323 | down |
| ZC3H11A | -1.843716097 | 0.015367596 | 0.050893303 | down |
| RP11-227H15.4 | -1.843273776 | 0.012034682 | 0.042283942 | down |
| AC007040.8 | -1.843132441 | 0.135886942 | | down |
| PARP4P2 | -1.843110401 | 0.028140214 | 0.080121739 | down |
| AC009502.1 | -1.842986282 | 0.398198161 | 0.535158378 | down |
| RP11-1G11.2 | -1.842972587 | 0.528331648 | | down |
| AC226118.1 | -1.842862013 | 0.010113289 | 0.03699095 | down |
| NA | -1.84271356 | 0.000725566 | 0.004697029 | down |
| NA | -1.842605306 | 0.163456375 | | down |
| TESC-AS1 | -1.842243684 | 0.34477445 | | down |
| ENOX1-AS1 | -1.842169663 | 0.011585263 | 0.04109511 | down |
| IGSF11-AS1 | -1.841853748 | 0.528509266 | | down |
| RP5-842K16.1 | -1.841685806 | 0.528620985 | | down |
| MAGEB18 | -1.841685806 | 0.528620985 | | down |
| HSD52 | -1.841618153 | 2.20E-05 | 0.000270056 | down |
| Metazoa_SRP | -1.841407972 | 0.006840588 | 0.027512535 | down |
| RP11-257K9.8 | -1.84137877 | 0.386757063 | | down |
| RP11-220I1.2 | -1.841264057 | 0.122133144 | | down |
| CTB-23I7.3 | -1.84110136 | 0.528380754 | | down |
| TRIM53CP | -1.840855747 | 0.52880767 | | down |
| SMIM6 | -1.840786393 | 6.13E-07 | 1.34E-05 | down |
| RP11-456O19.3 | -1.84077381 | 0.5288261 | | down |
| RP1-130G2.1 | -1.84058773 | 0.004833077 | 0.021145326 | down |
| RP11-452G18.1 | -1.840580564 | 0.112152726 | 0.219298177 | down |
| FSTL5 | -1.840456884 | 0.114983634 | 0.223206584 | down |
| BTNL3 | -1.840220255 | 1.54E-06 | 2.97E-05 | down |
| FSIP2-AS1 | -1.84012939 | 0.005250468 | 0.022525356 | down |
| FANCD2OS | -1.839773806 | 0.098564299 | 0.199976884 | down |
| ABCC6P2 | -1.839664029 | 1.47E-07 | 3.94E-06 | down |
| CICP18 | -1.83955103 | 0.34557522 | | down |
| PPP1R14D | -1.83947321 | 9.84E-07 | 2.00E-05 | down |
| AC010886.2 | -1.839309299 | 0.495269509 | | down |
| SRMP2 | -1.83924171 | 0.44249113 | | down |
| KRT16P4 | -1.838848863 | 0.529259165 | | down |
| NAV2-AS4 | -1.838771241 | 0.442835645 | | down |
| RP11-158L12.5 | -1.838726862 | 0.120966197 | 0.231455127 | down |
| TRMT2B-AS1 | -1.838635329 | 0.163849623 | | down |
| LAMA1 | -1.838493195 | 6.05E-08 | 1.86E-06 | down |
| NA | -1.838486099 | 0.033116452 | 0.090195875 | down |

| | | | | |
|---|---|---|---|---|
| THEGL | -1.838214334 | 0.395836077 | 0.532981434 | down |
| TMPRSS9 | -1.838188277 | 1.12E-06 | 2.24E-05 | down |
| NA | -1.838011103 | 0.529447696 | | down |
| AQP11 | -1.837959183 | 2.90E-07 | 7.06E-06 | down |
| KRT18P20 | -1.837507908 | 0.005751201 | 0.024140555 | down |
| RP11-609L3.1 | -1.837379771 | 0.202802297 | 0.332187995 | down |
| DENND5B-AS1 | -1.837294452 | 0.000369982 | 0.002747459 | down |
| IRF5P1 | -1.837073543 | 0.529584648 | | down |
| RP6-102.1 | -1.836950499 | 0.529537457 | | down |
| NPTX1 | -1.836622892 | 2.15E-05 | 0.000265295 | down |
| C2orf80 | -1.836349988 | 0.050172892 | 0.122622172 | down |
| PRRG2 | -1.836084402 | 7.69E-09 | 3.13E-07 | down |
| UCN3 | -1.835855988 | 0.000621591 | 0.004149352 | down |
| RP11-554D14.5 | -1.835755149 | 0.39055411 | | down |
| OR10H5 | -1.835483325 | 0.105116848 | 0.209327937 | down |
| CTD-2562J17.4 | -1.835313367 | 0.006230709 | 0.025686126 | down |
| IGLCOR22-1 | -1.835156723 | 0.530090305 | | down |
| RP11-83M16.4 | -1.835115096 | 0.004828534 | 0.021128526 | down |
| GXYLT1P6 | -1.835074168 | 0.241138268 | | down |
| NA | -1.835067032 | 0.493787845 | | down |
| CTB-118N6.2 | -1.835033293 | 9.30E-05 | 0.000889131 | down |
| NA | -1.834894167 | 0.443775548 | | down |
| AC104297.1 | -1.834771668 | 0.105201765 | | down |
| OR2T2 | -1.834738337 | 0.22164722 | | down |
| RP11-394I13.3 | -1.834701592 | 0.060104569 | | down |
| RP11-317B3.2 | -1.834481643 | 0.228321255 | | down |
| AC012513.6 | -1.834376427 | 0.530266042 | | down |
| NPHP3-AS1 | -1.834366556 | 0.205433161 | | down |
| RP3-428A13.1 | -1.834099644 | 0.314554188 | | down |
| RP11-297D21.2 | -1.833948326 | 0.021989488 | 0.066577869 | down |
| GTF2I | -1.833926265 | 4.73E-09 | 2.03E-07 | down |
| PTCHD4 | -1.833901129 | 0.004503036 | 0.020007133 | down |
| RP11-740N7.3 | -1.833878292 | 0.341483118 | | down |
| AC008985.1 | -1.833815229 | 0.061621512 | 0.142598529 | down |
| RP5-1119A7.14 | -1.833614573 | 0.000441549 | 0.00314955 | down |
| VLDLR-AS1 | -1.833569419 | 0.001103076 | 0.006563836 | down |
| RPL32P33 | -1.833403596 | 0.136809473 | | down |
| RP11-1086I4.2 | -1.833350813 | 0.530497074 | | down |
| RP11-447M4.1 | -1.833106086 | 0.530552209 | | down |
| CTD-2139B15.4 | -1.833003392 | 0.444326991 | | down |
| TDGF1P4 | -1.832994972 | 0.298355508 | | down |
| RP11-1C8.7 | -1.832641007 | 0.074038223 | | down |
| LINC00382 | -1.83244804 | 0.530700476 | | down |
| GPX6 | -1.832412459 | 0.530482966 | | down |
| AC092839.4 | -1.83208369 | 0.392530762 | | down |
| DPP4 | -1.832042354 | 5.69E-06 | 8.84E-05 | down |
| KLF18 | -1.831404466 | 0.530935651 | | down |
| RNA5SP224 | -1.831404466 | 0.530935651 | | down |
| NA | -1.83139045 | 0.258476936 | | down |
| HIPK4 | -1.831385582 | 0.000500334 | 0.003477223 | down |
| RP11-178L8.5 | -1.831371236 | 0.238082219 | | down |

| | | | | |
|---|---|---|---|---|
| CTD-2354A18.1 | -1.831099027 | 0.442742982 | | down |
| RP11-253I19.3 | -1.831078979 | 0.012366041 | 0.043130409 | down |
| RP11-1260E13.2 | -1.830576488 | 0.001503653 | 0.00835196 | down |
| RP11-302F12.3 | -1.830367011 | 0.443955278 | | down |
| LGALS9C | -1.830319278 | 3.92E-06 | 6.48E-05 | down |
| RP11-89B16.2 | -1.830265631 | 0.009321654 | 0.034755495 | down |
| NA | -1.830039983 | 0.531243224 | | down |
| MCF2 | -1.829879477 | 0.001657722 | 0.009030751 | down |
| RP11-26608.1 | -1.829000721 | 0.000358784 | 0.00268744 | down |
| WBP11P1 | -1.828959333 | 0.395617143 | | down |
| ZNF429 | -1.828953283 | 1.65E-10 | 1.05E-08 | down |
| CTD-2542L18.1 | -1.828946499 | 0.021461102 | 0.065386608 | down |
| NA | -1.828914372 | 0.531422726 | | down |
| RP11-3L21.2 | -1.828914372 | 0.531422726 | | down |
| RP1-305B16.3 | -1.828914372 | 0.531422726 | | down |
| GABRR1 | -1.828871258 | 0.044517723 | 0.112328849 | down |
| HNRNPA1P2 | -1.828772462 | 0.197188534 | | down |
| SETP6 | -1.828632022 | 0.253250314 | 0.388196553 | down |
| RP11-13K12.1 | -1.828569394 | 0.001801258 | 0.009660297 | down |
| CPNE4 | -1.828468574 | 0.001524936 | 0.008439182 | down |
| NA | -1.828458036 | 0.003452011 | 0.016194911 | down |
| ANXA8 | -1.828258716 | 0.016192868 | 0.052914784 | down |
| RP11-1137G4.4 | -1.828184366 | 0.209475833 | 0.33971132 | down |
| NA | -1.828180653 | 0.01301097 | 0.044776815 | down |
| GKN3P | -1.82816888 | 0.395567635 | | down |
| RP11-304C12.4 | -1.828036324 | 0.206720806 | | down |
| OR52B4 | -1.82799259 | 0.531704901 | | down |
| RAPGEFL1 | -1.827926267 | 2.86E-11 | 2.29E-09 | down |
| NA | -1.827492698 | 0.531817655 | | down |
| MEIOC | -1.827275379 | 0.001053052 | 0.006319995 | down |
| RP11-519G16.2 | -1.82715155 | 0.070786673 | 0.1575928 | down |
| RHOXF2B | -1.826977281 | 0.063843515 | 0.146157353 | down |
| CDH8 | -1.826853005 | 0.005855575 | 0.02449866 | down |
| ARHGAP26-AS1 | -1.826839589 | 0.029746606 | 0.083274974 | down |
| SPDYE17 | -1.826713447 | 0.000790619 | 0.005026103 | down |
| RP11-5L12.1 | -1.826708961 | 0.177259146 | 0.302217233 | down |
| RP11-111F5.4 | -1.826373598 | 0.001470113 | 0.008210583 | down |
| AC097533.1 | -1.826301416 | 0.244862094 | 0.378778537 | down |
| RP11-326A19.3 | -1.826255529 | 0.025369653 | 0.074094086 | down |
| RP11-529K1.2 | -1.82624863 | 2.47E-05 | 0.000297639 | down |
| KRT19P2 | -1.826227942 | 0.270316464 | | down |
| LINC01153 | -1.826191961 | 0.5321111 | | down |
| TDGF1P1 | -1.825705907 | 0.531781713 | | down |
| UGT1A10 | -1.825532823 | 0.002828691 | 0.013845745 | down |
| RP11-867G23.8 | -1.825456607 | 1.05E-16 | 3.07E-14 | down |
| CD177 | -1.825394232 | 0.000941357 | 0.005773038 | down |
| NXPH2 | -1.825322546 | 0.176430259 | 0.30126737 | down |
| PCDHA6 | -1.825311553 | 0.033105857 | 0.09017666 | down |
| ALG1L7P | -1.825236503 | 0.022825754 | 0.068393035 | down |
| AC002128.5 | -1.82504506 | 0.001758761 | 0.009474567 | down |
| Metazoa_SRP | -1.825013926 | 0.030499139 | 0.08483623 | down |

| | | | | |
|---|---|---|---|---|
| GATA6-AS1 | -1.825006182 | 3.99E-07 | 9.23E-06 | down |
| CHODL-AS1 | -1.824667026 | 0.283066791 | | down |
| RP11-437J2.3 | -1.824387633 | 0.28538913 | | down |
| SLC35D3 | -1.824374991 | 0.009594078 | 0.035486788 | down |
| RP11-223A3.1 | -1.823886831 | 3.78E-05 | 0.000425254 | down |
| RP11-293B20.2 | -1.823857969 | 0.395228135 | | down |
| LINC00483 | -1.82385647 | 7.27E-08 | 2.18E-06 | down |
| RP11-713P14.1 | -1.823628334 | 0.532689691 | | down |
| OPRD1 | -1.823472236 | 0.000143429 | 0.001274525 | down |
| RP13-235E23.1 | -1.823121158 | 0.496091332 | | down |
| SLC39A4 | -1.822907028 | 1.51E-10 | 9.71E-09 | down |
| NA | -1.822880879 | 0.000588012 | 0.00396905 | down |
| RP11-80H5.7 | -1.822857994 | 0.16565207 | | down |
| NA | -1.822692857 | 0.077440352 | 0.168132528 | down |
| RP11-244M2.1 | -1.822687185 | 3.84E-05 | 0.0004301 | down |
| RP11-489E7.1 | -1.822554963 | 0.497661141 | | down |
| AC017002.4 | -1.822246851 | 0.532853306 | | down |
| RPL23AP91 | -1.822031945 | 0.136170119 | 0.251280329 | down |
| RGR | -1.821808729 | 0.171792234 | 0.295981527 | down |
| AP006216.5 | -1.821609791 | 0.294173815 | | down |
| PPP1R14BP5 | -1.821546433 | 0.533159793 | | down |
| RP5-1198O20.5 | -1.821222757 | 0.533084639 | | down |
| AJ009632.3 | -1.821095803 | 0.53311332 | | down |
| RP11-507F16.1 | -1.821006121 | 0.203009919 | 0.332455576 | down |
| SUMO2P16 | -1.820761971 | 0.444637547 | | down |
| NA | -1.820695032 | 0.504166265 | | down |
| PNLIPRP3 | -1.82048738 | 0.39658353 | | down |
| RP11-690C23.4 | -1.820105962 | 0.533485178 | | down |
| SLC36A3 | -1.819885694 | 0.131611646 | | down |
| TCP11X3P | -1.819420906 | 0.533639959 | | down |
| NGEF | -1.819397772 | 1.08E-10 | 7.31E-09 | down |
| LINC00521 | -1.819243115 | 0.055011747 | 0.131120423 | down |
| SLC22A16 | -1.819055252 | 0.025147149 | 0.073637519 | down |
| FOXP1-AS1 | -1.818952226 | 0.005727604 | 0.024067152 | down |
| MIR3680-1 | -1.818910301 | 0.234313264 | | down |
| PLS1 | -1.818750813 | 8.79E-07 | 1.82E-05 | down |
| NSRP1P1 | -1.818717594 | 0.008056532 | 0.031103886 | down |
| AP000343.1 | -1.818583256 | 0.504957785 | | down |
| AC004870.4 | -1.818531566 | 0.103919164 | | down |
| AC139143.2 | -1.818517755 | 0.346516869 | | down |
| SNRPEP7 | -1.818276468 | 0.398075776 | | down |
| RP11-1134I14.4 | -1.81824004 | 0.0085988 | 0.03266022 | down |
| AL590762.11 | -1.818230084 | 0.030005029 | 0.083787629 | down |
| RP11-172F10.1 | -1.818228731 | 0.146445205 | 0.264617032 | down |
| RNU6-113P | -1.818059833 | 0.533947546 | | down |
| MYH8 | -1.8180171 | 0.017947926 | 0.057273232 | down |
| NA | -1.818002107 | 0.001439713 | 0.008072927 | down |
| NHP2P2 | -1.817991371 | 0.533888444 | | down |
| NA | -1.817958962 | 0.533970345 | | down |
| NA | -1.817860599 | 0.006867192 | 0.027597366 | down |
| MESP2 | -1.817848487 | 7.17E-05 | 0.000715992 | down |

| | | | | |
|---|---|---|---|---|
| RP11-135N5.3 | -1.81781395 | 0.000708926 | 0.004608542 | down |
| RP11-65L3.3 | -1.817728189 | 0.236338573 | | down |
| HHIPL2 | -1.817227507 | 0.000165039 | 0.001427708 | down |
| CTB-102L5.7 | -1.817219936 | 0.012792573 | 0.044217677 | down |
| RP11-683L23.7 | -1.817180331 | 0.148557609 | 0.267277371 | down |
| RP11-2I17.4 | -1.817140356 | 0.078415899 | 0.169723167 | down |
| GRIA4 | -1.816997781 | 4.14E-06 | 6.81E-05 | down |
| RP11-85G21.3 | -1.816862268 | 0.144878053 | 0.262436309 | down |
| NA | -1.816417609 | 0.492581885 | 0.620716809 | down |
| OR8B5P | -1.816349359 | 0.236266636 | 0.369246504 | down |
| RP11-251P6.1 | -1.816225686 | 0.400424617 | | down |
| RNU4-2 | -1.815908328 | 0.004582197 | 0.020273006 | down |
| CTD-3105H18.18 | -1.815668913 | 0.097201472 | 0.19808187 | down |
| NA | -1.815336664 | 0.00905915 | 0.033996141 | down |
| CTA-407F11.9 | -1.815013917 | 0.501651754 | | down |
| CELF5 | -1.814962164 | 5.44E-05 | 0.000572852 | down |
| RP11-844P9.2 | -1.814911958 | 0.534659268 | | down |
| RP11-293N14.1 | -1.814911958 | 0.534659268 | | down |
| RBMX2P2 | -1.814911958 | 0.534659268 | | down |
| PRB2 | -1.814571981 | 0.048039099 | | down |
| SCARNA20 | -1.814187169 | 0.162826714 | | down |
| LDHAL6EP | -1.814030369 | 0.503022069 | | down |
| RP5-901A4.4 | -1.813754456 | 0.069627974 | 0.155750851 | down |
| CDC42-IT1 | -1.81374031 | 0.019522443 | 0.060906779 | down |
| LLNLR-284B4.2 | -1.813679773 | 0.446755185 | | down |
| RP11-397P13.6 | -1.813670491 | 0.186051171 | | down |
| RP11-661C3.2 | -1.813597652 | 0.003400112 | 0.015988881 | down |
| RP11-218E20.2 | -1.81353227 | 0.53497136 | | down |
| TMEM253 | -1.813526089 | 0.003227376 | 0.015332587 | down |
| FAM132A | -1.813444582 | 2.46E-07 | 6.17E-06 | down |
| RP11-654E17.2 | -1.81322736 | 0.045265979 | 0.113690944 | down |
| PSAPL1 | -1.812933292 | 0.292178236 | 0.429435754 | down |
| CTB-147C13.1 | -1.812879616 | 0.535119025 | | down |
| NA | -1.812744391 | 0.284755541 | | down |
| AKNAD1 | -1.812522453 | 0.002379521 | 0.012084236 | down |
| CCT4P1 | -1.811500296 | 0.535431167 | | down |
| RP1-111D6.5 | -1.811249226 | 0.535487994 | | down |
| TCF23 | -1.81095125 | 0.008222775 | 0.031587399 | down |
| EIF5AP2 | -1.810851201 | 0.007876396 | 0.030619789 | down |
| RP11-139E19.2 | -1.810787311 | 0.535444768 | | down |
| ZAR1 | -1.810604317 | 0.165765099 | | down |
| CTD-2503O16.4 | -1.810567719 | 0.012493407 | 0.043443509 | down |
| CTD-2501B8.1 | -1.81056329 | 0.197318217 | 0.32590591 | down |
| AC016629.3 | -1.810392236 | 0.234861004 | | down |
| TRIM51CP | -1.810385214 | 0.535683578 | | down |
| DEPDC7 | -1.810357518 | 1.41E-07 | 3.83E-06 | down |
| CTA-397C4.2 | -1.810202102 | 0.535100577 | | down |
| PKMP5 | -1.810076608 | 0.449187054 | | down |
| CTA-415G2.2 | -1.809892744 | 0.495920994 | | down |
| CTD-2296D1.5 | -1.809731584 | 0.535831562 | | down |
| POM121L7 | -1.809677058 | 0.504042953 | | down |

| | | | | |
|---|---|---|---|---|
| RPL23AP57 | -1.809503559 | 0.006951682 | 0.027854939 | down |
| WDR93 | -1.809308674 | 0.001137736 | 0.006727445 | down |
| FAM25C | -1.809289358 | 0.116127773 | 0.224759571 | down |
| HCRTR1 | -1.809218072 | 0.006592452 | 0.026758122 | down |
| RP11-642A1.1 | -1.809131154 | 0.10000578 | | down |
| CT62 | -1.809113036 | 0.079905037 | 0.172053819 | down |
| CTD-2168K21.1 | -1.808458273 | 0.34971456 | | down |
| NA | -1.808422017 | 0.104121369 | 0.207786278 | down |
| RP11-492D6.3 | -1.808387414 | 0.083780651 | 0.178038329 | down |
| NA | -1.808148823 | 0.130859088 | 0.24442216 | down |
| RP11-770E5.3 | -1.808086227 | 0.086710213 | 0.182648926 | down |
| IGHVIII-44 | -1.808080977 | 0.249131594 | | down |
| SPDYE1 | -1.808060858 | 0.000124051 | 0.001132351 | down |
| SARNP | -1.807895423 | 0.001859419 | 0.00990679 | down |
| MIR4530 | -1.807876329 | 0.003854662 | 0.017699081 | down |
| MTCO2P3 | -1.80783803 | 0.536260384 | | down |
| ZFP64P1 | -1.807569894 | 0.399485117 | | down |
| RP11-1078H9.6 | -1.807534718 | 0.536329089 | | down |
| AC093690.1 | -1.807446714 | 0.001225697 | 0.007131174 | down |
| bP-218909.3 | -1.807106819 | 0.256862793 | | down |
| RP11-432M24.4 | -1.807100039 | 0.407574406 | 0.544229576 | down |
| CHST13 | -1.807041143 | 5.01E-05 | 0.000536372 | down |
| LINC00633 | -1.806911164 | 0.536470348 | | down |
| BX255923.2 | -1.806752046 | 0.105527057 | 0.210005624 | down |
| RP11-159D12.11 | -1.806555204 | 0.049100838 | | down |
| PFN1P4 | -1.80650387 | 0.065231859 | 0.148336813 | down |
| RP11-446F3.2 | -1.806480504 | 0.536493049 | | down |
| MIR96 | -1.806157577 | 0.536641087 | | down |
| BRIX1P1 | -1.806157577 | 0.536641087 | | down |
| OR52B2 | -1.806157577 | 0.536641087 | | down |
| MRGPRX1 | -1.806157577 | 0.536641087 | | down |
| IGHV3-75 | -1.806157577 | 0.536641087 | | down |
| KRTAP4-8 | -1.806157577 | 0.536641087 | | down |
| AQP7 | -1.806141038 | 1.40E-07 | 3.80E-06 | down |
| NA | -1.805947585 | 0.160661688 | 0.282134646 | down |
| RN7SL487P | -1.805933699 | 0.308645874 | | down |
| OGFOD1P1 | -1.805780503 | 0.106370313 | | down |
| HMGB3P5 | -1.805650517 | 0.107253641 | 0.212484562 | down |
| RP1L1 | -1.805440305 | 0.002059181 | 0.010738764 | down |
| AWAT1 | -1.805329814 | 0.499300096 | | down |
| AC092675.4 | -1.805204709 | 0.536856988 | | down |
| AC002401.1 | -1.805144419 | 0.179114068 | | down |
| NTM-AS1 | -1.805142548 | 0.313127562 | | down |
| NA | -1.805128988 | 0.003042931 | 0.014665033 | down |
| CTD-2651B20.6 | -1.804855571 | 0.241199678 | | down |
| RP11-52J3.2 | -1.80431067 | 0.407734078 | | down |
| LRRC34P1 | -1.804285914 | 0.246274249 | 0.38039502 | down |
| LRRC69 | -1.803934501 | 0.000196558 | 0.001647245 | down |
| APOBEC4 | -1.803794492 | 0.06774948 | | down |
| NA | -1.803598434 | 0.536922499 | | down |
| RP11-82O19.1 | -1.803006408 | 0.042499314 | 0.108714296 | down |

| | | | | |
|---|---|---|---|---|
| CH17-373J23.1 | -1.802989566 | 0.000752413 | 0.004836436 | down |
| PCLO | -1.802732558 | 1.39E-10 | 9.12E-09 | down |
| SLC25A15P4 | -1.80271364 | 0.537421619 | | down |
| ARHGAP42P3 | -1.80271364 | 0.537421619 | | down |
| RP11-441A11.1 | -1.80271364 | 0.537421619 | | down |
| RP11-485M7.3 | -1.80269419 | 0.450467308 | | down |
| MAGIX | -1.802654995 | 5.72E-07 | 1.27E-05 | down |
| SMCO1 | -1.802549291 | 0.10942386 | | down |
| C20orf78 | -1.802145133 | 0.503545073 | | down |
| MAST1 | -1.801908679 | 9.08E-07 | 1.86E-05 | down |
| AC005152.3 | -1.801882989 | 0.037514988 | 0.099033504 | down |
| CTD-2184C24.2 | -1.801857541 | 1.78E-08 | 6.43E-07 | down |
| PHF21B | -1.801804849 | 0.000731492 | 0.004730297 | down |
| RN7SL91P | -1.801753852 | 0.537639245 | | down |
| NA | -1.801713258 | 0.167330459 | 0.290442908 | down |
| MT1F | -1.801577213 | 2.12E-10 | 1.32E-08 | down |
| MOXD2P | -1.801566565 | 0.105166266 | | down |
| WNT3 | -1.801554497 | 4.73E-05 | 0.000510177 | down |
| LINC01474 | -1.801509026 | 0.004688407 | 0.020639869 | down |
| TMEM54 | -1.801324674 | 1.73E-06 | 3.27E-05 | down |
| ADARB2 | -1.80122585 | 2.43E-06 | 4.34E-05 | down |
| RP11-159N11.3 | -1.80121169 | 0.268544649 | | down |
| NA | -1.801123152 | 0.084282149 | 0.178800514 | down |
| ANKRD44-IT1 | -1.800860938 | 0.003142167 | 0.015008467 | down |
| RNA5SP207 | -1.80082108 | 0.065571917 | 0.148929745 | down |
| AC005786.5 | -1.800730789 | 0.355921065 | | down |
| RNU4-86P | -1.80069687 | 0.131161193 | 0.244848762 | down |
| NA | -1.800670864 | 0.279920479 | | down |
| RP13-487K5.1 | -1.800613331 | 0.054625483 | 0.130489625 | down |
| IFNA6 | -1.800552004 | 0.537911819 | | down |
| PRAMEF12 | -1.800496521 | 0.537924404 | | down |
| RP11-68E19.2 | -1.800391686 | 0.151013662 | 0.270194745 | down |
| CTD-2521M24.13 | -1.800368971 | 0.002967901 | 0.014381872 | down |
| RPS12P20 | -1.800342359 | 0.075682975 | 0.165368676 | down |
| AC105053.3 | -1.80018518 | 0.128609282 | 0.24151062 | down |
| SLC52A3 | -1.799668732 | 3.92E-09 | 1.72E-07 | down |
| BCL9P1 | -1.799574012 | 0.083408871 | 0.17757484 | down |
| AF131215.4 | -1.799523572 | 0.109449826 | | down |
| EFTUD1P2 | -1.799333046 | 0.538188343 | | down |
| CTD-3032J10.2 | -1.798983191 | 0.156592212 | | down |
| AL591704.9 | -1.798945495 | 0.36358222 | | down |
| NA | -1.798879302 | 0.241988302 | | down |
| AF003529.2 | -1.798876325 | 0.53829197 | | down |
| NA | -1.798608544 | 1.75E-06 | 3.30E-05 | down |
| CTD-2026K11.2 | -1.79848671 | 0.091749668 | 0.190095503 | down |
| AC104076.3 | -1.798352297 | 0.538263835 | | down |
| RP11-393K12.4 | -1.798265286 | 0.223147346 | 0.355204818 | down |
| RP4-553F17.1 | -1.798198493 | 0.538445784 | | down |
| NA | -1.797803734 | 0.538460552 | | down |
| OR7E129P | -1.79748757 | 0.53860713 | | down |
| RNASEH2B-AS1 | -1.797486478 | 0.000381229 | 0.002810712 | down |

| | | | | |
|---|---|---|---|---|
| TRIM10 | -1.796991128 | 1.53E-06 | 2.96E-05 | down |
| RP11-813F20.2 | -1.796897346 | 0.052295538 | 0.126218439 | down |
| NPM1P12 | -1.796878343 | 0.45396497 | | down |
| RP11-545G3.1 | -1.796783807 | 0.00502932 | 0.021797652 | down |
| NA | -1.796650491 | 0.538797138 | | down |
| RP11-61J19.2 | -1.796609513 | 0.538731628 | | down |
| KRT13 | -1.796506969 | 0.056067762 | 0.132966698 | down |
| RP11-296L22.8 | -1.796383994 | 0.538857637 | | down |
| NA | -1.796379566 | 0.501397626 | | down |
| PPP1R16A | -1.796362264 | 5.55E-12 | 5.14E-10 | down |
| NA | -1.796041432 | 0.511066808 | | down |
| HNRNPCP8 | -1.795826089 | 0.0919747 | 0.190400875 | down |
| RP11-513G19.1 | -1.79580126 | 0.000430692 | 0.003087502 | down |
| SLC25A48 | -1.795771981 | 5.41E-06 | 8.45E-05 | down |
| RP11-252C24.3 | -1.795642781 | 0.539025922 | | down |
| PRSS54 | -1.795591581 | 0.217972489 | 0.349318438 | down |
| SNORA26 | -1.7954879 | 0.147273422 | 0.265652318 | down |
| CTD-3126B10.5 | -1.795432085 | 0.009452454 | 0.035121138 | down |
| TMEM184A | -1.795262039 | 6.79E-08 | 2.06E-06 | down |
| CYP1D1P | -1.7951741 | 0.509940195 | | down |
| NA | -1.795090628 | 0.005486911 | 0.023267289 | down |
| TJP2 | -1.794968417 | 1.45E-05 | 0.000193115 | down |
| ALG1L5P | -1.794663009 | 0.004663931 | 0.020564007 | down |
| MYCBP2-AS1 | -1.794537277 | 0.149571001 | | down |
| RP11-777B9.1 | -1.794487974 | 0.539213364 | | down |
| RNU6-1160P | -1.794414259 | 0.057786254 | | down |
| IGHV1OR21-1 | -1.794267131 | 0.539263522 | | down |
| NA | -1.794207238 | 0.539277126 | | down |
| RP11-575F12.1 | -1.794079192 | 0.000321708 | 0.002466919 | down |
| AC091770.3 | -1.793970866 | 0.539405609 | | down |
| RP11-470L19.2 | -1.793936823 | 0.318072753 | | down |
| NA | -1.793932039 | 0.119855658 | 0.229989359 | down |
| MTND2P9 | -1.793930592 | 0.248508122 | | down |
| RP11-521H3.3 | -1.793918889 | 0.539417415 | | down |
| EML6 | -1.793773899 | 8.05E-07 | 1.68E-05 | down |
| SHANK2-AS2 | -1.793664167 | 0.539328448 | | down |
| PLEKHG6 | -1.793522037 | 2.20E-08 | 7.70E-07 | down |
| RP11-252O18.3 | -1.793187273 | 0.539583607 | | down |
| KRT18P12 | -1.792775717 | 0.539455339 | | down |
| NA | -1.792373508 | 0.539621723 | | down |
| RP11-15G8.1 | -1.792219424 | 0.319500056 | 0.45829286 | down |
| POU3F2 | -1.792214317 | 0.161873677 | | down |
| RBMXP1 | -1.79212474 | 0.539825013 | | down |
| RP1-310O13.13 | -1.791973175 | 0.539859453 | | down |
| NA | -1.791910894 | 0.015619515 | 0.051511823 | down |
| TGM6 | -1.791879266 | 0.507507263 | | down |
| RP11-435I3.1 | -1.791821171 | 0.539819215 | | down |
| RP11-57C19.6 | -1.791774207 | 0.030113792 | 0.08402106 | down |
| RP11-472N13.2 | -1.79134297 | 0.003096822 | 0.014860391 | down |
| AC006509.4 | -1.79112209 | 0.12462601 | | down |
| NA | -1.790810206 | 0.183599475 | | down |

| | | | | |
|---|---|---|---|---|
| RP11-67L3.4 | -1.7904773 | 0.025541703 | 0.0744446 | down |
| RP11-867G23.3 | -1.790385108 | 0.000180895 | 0.001540477 | down |
| OR2F1 | -1.790167717 | 0.213335841 | 0.344233587 | down |
| TBCAP2 | -1.790069118 | 0.540217435 | | down |
| RP11-368I23.2 | -1.790059168 | 0.064876419 | 0.147767602 | down |
| NA | -1.78946215 | 0.512435386 | | down |
| RP11-298H24.1 | -1.789303797 | 0.184914915 | 0.310961072 | down |
| DEFB118 | -1.78924578 | 0.123183011 | 0.234258542 | down |
| OR2A12 | -1.788906074 | 9.21E-05 | 0.000882191 | down |
| AC074286.1 | -1.788512289 | 1.08E-05 | 0.000149762 | down |
| RP11-358H18.2 | -1.787965025 | 0.540624011 | | down |
| CTC-264010.2 | -1.787943831 | 0.540775432 | | down |
| RP11-336N8.1 | -1.787943831 | 0.540775432 | | down |
| LINC00887 | -1.787904627 | 0.020783252 | 0.063864899 | down |
| MZT1P1 | -1.787635478 | 0.511684196 | | down |
| AC097381.1 | -1.787507496 | 0.007484636 | 0.029436889 | down |
| RP1-117B12.4 | -1.7873352 | 0.00529956 | 0.022691447 | down |
| NA | -1.78731176 | 0.021941647 | 0.066473211 | down |
| AC009498.1 | -1.786969857 | 0.368980167 | | down |
| LNX1-AS2 | -1.786733044 | 0.070177087 | 0.15664365 | down |
| NA | -1.78669904 | 0.54098382 | | down |
| NA | -1.786698472 | 0.513139053 | | down |
| RP11-253M7.3 | -1.786660087 | 0.540920842 | | down |
| RP11-108M12.3 | -1.786633986 | 0.131107867 | | down |
| RP11-46I8.3 | -1.786577109 | 0.079036796 | 0.170673578 | down |
| KIF12 | -1.786560729 | 5.95E-08 | 1.83E-06 | down |
| BRI3BPP1 | -1.786384326 | 0.244069042 | | down |
| GPR149 | -1.7863458 | 0.228342607 | 0.360690078 | down |
| SPTB | -1.786246423 | 2.25E-05 | 0.000275465 | down |
| FMR1-IT1 | -1.786194469 | 0.001169624 | 0.006867343 | down |
| IBSP | -1.786065631 | 0.356366062 | | down |
| AC008265.2 | -1.786040591 | 0.512516259 | | down |
| RP11-372H2.1 | -1.786040399 | 0.3199507 | | down |
| CCK | -1.785667966 | 0.022212151 | 0.067062793 | down |
| CDON | -1.785500203 | 0.000133684 | 0.001203236 | down |
| NA | -1.785430198 | 0.03167372 | 0.087295225 | down |
| RP11-727F15.14 | -1.785402043 | 0.0588965 | 0.137885641 | down |
| RP11-586K12.11 | -1.785359195 | 0.541363395 | | down |
| NA | -1.785340881 | 0.052555436 | 0.126713388 | down |
| USP8P1 | -1.784942578 | 0.360899367 | | down |
| YY1P1 | -1.784839476 | 0.455474198 | | down |
| ASTN2-AS1 | -1.784222682 | 0.406207304 | | down |
| CTD-2639E6.4 | -1.784194374 | 0.004874659 | 0.021270751 | down |
| PLA2G10 | -1.784190083 | 0.000272875 | 0.002160851 | down |
| ERVV-2 | -1.783996977 | 0.101694682 | 0.204505691 | down |
| IL9RP4 | -1.783733025 | 0.541658763 | | down |
| FBP2 | -1.783484049 | 0.0206262 | 0.063512714 | down |
| RP11-514F8.2 | -1.783421531 | 0.173014506 | 0.297439221 | down |
| RP11-217B7.3 | -1.782968561 | 0.03658651 | 0.097204339 | down |
| EFCAB6-AS1 | -1.782930825 | 0.254870323 | | down |
| NA | -1.782718551 | 0.059262384 | 0.138472524 | down |

| | | | | |
|---|---|---|---|---|
| SLC39A2 | -1.782451153 | 0.005225454 | 0.022440473 | down |
| RP11-475E11.2 | -1.782434087 | 0.271117213 | | down |
| RP11-168F9.2 | -1.78229288 | 0.005585925 | 0.023610515 | down |
| NA | -1.782236338 | 0.304612697 | 0.442649764 | down |
| RP11-107I14.5 | -1.782234225 | 0.506837644 | | down |
| RP11-498C9.17 | -1.782123696 | 0.542099864 | | down |
| RP11-730K11.1 | -1.782122556 | 0.009187089 | 0.034373215 | down |
| RP11-927P21.7 | -1.782004402 | 0.54198046 | | down |
| RP11-121M22.1 | -1.78168406 | 2.44E-05 | 0.000295143 | down |
| OPRM1 | -1.781662534 | 0.026359113 | 0.076235386 | down |
| RP11-443B7.1 | -1.781556443 | 1.75E-06 | 3.29E-05 | down |
| ADH6 | -1.78155588 | 8.67E-09 | 3.49E-07 | down |
| HYAL4 | -1.781509925 | 0.062161171 | 0.143504705 | down |
| CTD-230905.3 | -1.781483008 | 0.542171052 | | down |
| RP11-8P11.4 | -1.781205442 | 0.542234265 | | down |
| RP11-235C23.6 | -1.781172574 | 0.542316453 | | down |
| RP11-15E1.6 | -1.78091131 | 0.542301255 | | down |
| RP13-16H11.9 | -1.780792902 | 0.412379438 | | down |
| MMEL1 | -1.780745905 | 2.64E-06 | 4.67E-05 | down |
| CTB-96E2.6 | -1.780558368 | 0.005417262 | 0.023076169 | down |
| C1orf143 | -1.780499328 | 0.113355961 | 0.22085695 | down |
| RP11-629N8.3 | -1.780425813 | 0.226267417 | | down |
| GP6 | -1.78039016 | 0.000125368 | 0.00114173 | down |
| GRAMD2 | -1.780243484 | 1.89E-08 | 6.76E-07 | down |
| RAX | -1.779607102 | 0.068243287 | 0.153446217 | down |
| KLF4P1 | -1.779562479 | 0.514393658 | | down |
| GAS2L2 | -1.779509655 | 0.007754797 | 0.03026174 | down |
| RP1-20208.2 | -1.779508279 | 0.366878788 | | down |
| SLC22A8 | -1.779483539 | 0.307473369 | 0.445814869 | down |
| UNC45B | -1.779438853 | 0.002419252 | 0.012252907 | down |
| NA | -1.779407801 | 0.542643752 | | down |
| RP11-306K13.1 | -1.779378071 | 0.510430841 | | down |
| LCN12 | -1.779255784 | 1.23E-05 | 0.000168105 | down |
| NA | -1.779215661 | 0.08317345 | | down |
| NA | -1.77915449 | 0.542776154 | | down |
| CTD-2320J21.1 | -1.77915449 | 0.542776154 | | down |
| AC005229.7 | -1.77915449 | 0.542776154 | | down |
| MIR4291 | -1.77915449 | 0.542776154 | | down |
| KRTAP1-3 | -1.77915449 | 0.542776154 | | down |
| RP11-755E23.2 | -1.77905422 | 0.031127658 | 0.086177603 | down |
| SAMD7 | -1.779011048 | 0.027581921 | 0.078906041 | down |
| NA | -1.778952222 | 0.542747553 | | down |
| THSD7B | -1.778934552 | 0.015054686 | 0.050077834 | down |
| GAPDHP56 | -1.778926392 | 0.542828124 | | down |
| HNRNPRP2 | -1.778769129 | 0.542863957 | | down |
| RP11-386I8.4 | -1.778571078 | 0.542909085 | | down |
| RP11-930O11.1 | -1.77841379 | 0.002217051 | 0.011401761 | down |
| RP11-647P12.1 | -1.778396627 | 0.457345222 | | down |
| NA | -1.778376633 | 0.361533194 | | down |
| LIFR-AS1 | -1.778337379 | 2.26E-05 | 0.000276785 | down |
| AIFM3 | -1.778318093 | 2.36E-06 | 4.24E-05 | down |

| | | | | |
|---|---|---|---|---|
| RP11-927P21.2 | -1.778092962 | 0.010700567 | 0.038634078 | down |
| NA | -1.778077096 | 0.118514481 | | down |
| LINC00596 | -1.777974563 | 0.543045019 | | down |
| RP11-177H2.2 | -1.777837098 | 0.142373871 | 0.259288391 | down |
| KSR2 | -1.777746358 | 4.32E-06 | 7.04E-05 | down |
| EIF4BP1 | -1.77743023 | 0.543169076 | | down |
| RP11-111I12.1 | -1.777091484 | 0.008250224 | 0.031660457 | down |
| GM140 | -1.776892689 | 0.543291599 | | down |
| RP11-889L3.4 | -1.776649539 | 0.359751457 | | down |
| NA | -1.776253318 | 0.222275808 | | down |
| CTD-2267D19.4 | -1.77601975 | 0.101964785 | | down |
| AC006372.4 | -1.775867987 | 0.208501421 | 0.338679536 | down |
| RP13-766D20.2 | -1.775744714 | 6.18E-05 | 0.00063644 | down |
| LA16c-395F10.2 | -1.775695758 | 0.031874434 | 0.087690094 | down |
| RP11-690C23.2 | -1.775624968 | 0.409487804 | | down |
| CKB | -1.775621154 | 4.56E-06 | 7.36E-05 | down |
| HEATR4 | -1.775460191 | 6.69E-06 | 0.000101262 | down |
| RP11-122G18.9 | -1.775378282 | 0.543416377 | | down |
| CGB8 | -1.775227594 | 0.457502244 | | down |
| UGT1A13P | -1.775225318 | 0.197201294 | | down |
| AADACP1 | -1.775003152 | 2.02E-05 | 0.000252745 | down |
| AC099342.1 | -1.774821083 | 0.133988081 | | down |
| RP11-62904.1 | -1.774786183 | 0.379016451 | 0.51703461 | down |
| IL20RB-AS1 | -1.774597038 | 0.407472482 | | down |
| NA | -1.774234801 | 0.00017887 | 0.0015267 | down |
| RP11-120I21.2 | -1.77394538 | 0.543963617 | | down |
| RP11-216B9.8 | -1.773712136 | 0.090697948 | 0.188542345 | down |
| FEZF1 | -1.773493201 | 0.290814293 | | down |
| RP4-680D5.8 | -1.773401675 | 0.031171783 | | down |
| RP11-1C1.5 | -1.772999457 | 0.516147959 | | down |
| COL6A4P1 | -1.772903886 | 0.036022007 | 0.096097067 | down |
| C3orf30 | -1.772899616 | 0.544127533 | | down |
| GFI1B | -1.772574533 | 9.30E-08 | 2.67E-06 | down |
| NA | -1.772141337 | 0.186706319 | | down |
| RP11-310P5.1 | -1.771632792 | 0.099786269 | 0.201618341 | down |
| RP4-800O15.3 | -1.771575802 | 0.544504135 | | down |
| IFNL4 | -1.771443658 | 0.544459746 | | down |
| NA | -1.771230234 | 0.086006702 | 0.181689309 | down |
| SNORA25 | -1.770838474 | 0.544597863 | | down |
| RP11-881L2.1 | -1.770773781 | 0.017329686 | 0.055678867 | down |
| NA | -1.770594871 | 0.195120798 | 0.323539337 | down |
| AE000660.4 | -1.770427457 | 0.062048856 | 0.143290987 | down |
| FAM86MP | -1.770074275 | 0.147032736 | 0.265376157 | down |
| RP11-66N24.7 | -1.770022164 | 0.199266791 | | down |
| RP11-167H9.5 | -1.769928333 | 0.544880091 | | down |
| NA | -1.769551645 | 0.544891604 | | down |
| MTCYBP10 | -1.769160402 | 0.545055378 | | down |
| GNL3LP1 | -1.768989549 | 0.202243381 | | down |
| RP11-77K12.10 | -1.76891749 | 0.004328185 | 0.019388099 | down |
| CTD-3032J10.4 | -1.768609808 | 0.026423607 | 0.076392499 | down |
| STX18-IT1 | -1.768559028 | 0.410318387 | | down |

| | | | | |
|---|---|---|---|---|
| RP11-20D14.3 | -1.768313948 | 0.519848268 | | down |
| RP11-57A1.1 | -1.768214276 | 0.092744295 | | down |
| RP11-253M7.4 | -1.768020399 | 0.042031874 | 0.107849145 | down |
| LINC00551 | -1.767935516 | 0.005681728 | 0.023901124 | down |
| NA | -1.767698422 | 0.392948612 | 0.530333233 | down |
| AC104634.3 | -1.767641929 | 0.059480307 | | down |
| RP11-407N17.5 | -1.767511622 | 0.000769781 | 0.004920704 | down |
| NA | -1.767388596 | 0.025109082 | 0.073540385 | down |
| RP11-402N8.1 | -1.76627195 | 0.296438308 | 0.433855832 | down |
| RGS8 | -1.766157801 | 0.005059276 | 0.021895837 | down |
| RP11-707P20.1 | -1.766082946 | 0.545758113 | | down |
| CHRM1 | -1.765948604 | 1.64E-06 | 3.12E-05 | down |
| RP11-624J12.1 | -1.765658288 | 0.410720777 | | down |
| HBZ | -1.765373367 | 0.112297801 | 0.219433066 | down |
| NANOGP8 | -1.765327789 | 0.305650967 | 0.443934681 | down |
| FOXN1 | -1.765286732 | 0.007539566 | 0.029594814 | down |
| RP1-92C4.1 | -1.764939381 | 0.545872814 | | down |
| MTCYBP27 | -1.764939381 | 0.545872814 | | down |
| RP11-778J16.3 | -1.764924319 | 0.514317485 | | down |
| RP11-438O11.1 | -1.764852826 | 0.546039134 | | down |
| RP5-1049G16.4 | -1.764762161 | 0.035888556 | 0.09577693 | down |
| LINC01017 | -1.764720701 | 0.545995048 | | down |
| OR7G15P | -1.764666315 | 0.248208498 | 0.382555579 | down |
| RPL17P26 | -1.76457343 | 0.065993952 | 0.149650713 | down |
| RP11-1060G2.2 | -1.764540301 | 0.025991738 | 0.075454966 | down |
| RP11-760L24.1 | -1.764329192 | 0.513519898 | | down |
| SLC25A5P8 | -1.764125029 | 0.367466968 | | down |
| CTD-2576D5.1 | -1.763995391 | 0.130715322 | 0.244259929 | down |
| RP11-90D11.1 | -1.763859788 | 0.516143879 | | down |
| RP11-357D18.1 | -1.763752145 | 0.044429113 | 0.112209544 | down |
| ADGRG7 | -1.763676037 | 0.000125407 | 0.001141736 | down |
| NA | -1.763432535 | 0.186565151 | | down |
| RP11-275I14.2 | -1.763410349 | 0.517210739 | | down |
| IMPG1 | -1.763131079 | 0.009505866 | 0.035254197 | down |
| AC093166.4 | -1.762937174 | 0.254277188 | 0.389313757 | down |
| ABHD17AP1 | -1.762873008 | 0.0001313 | 0.001184622 | down |
| RP11-297P16.4 | -1.762611283 | 0.152531124 | 0.27198321 | down |
| AKAP8P1 | -1.762604514 | 0.546552946 | | down |
| NA | -1.762554431 | 0.004641721 | 0.02048111 | down |
| AC106870.2 | -1.762302552 | 0.225473491 | 0.357549117 | down |
| AGXT | -1.762268762 | 0.000296582 | 0.002310817 | down |
| AC068489.1 | -1.762197135 | 0.356998335 | 0.495420802 | down |
| GP2 | -1.761937316 | 0.000404841 | 0.002937197 | down |
| IGBP1P4 | -1.761874644 | 0.319028375 | | down |
| RP11-316M21.7 | -1.761677835 | 0.000581688 | 0.00393343 | down |
| RP11-104J23.2 | -1.761605918 | 0.072846555 | | down |
| NA | -1.761572093 | 0.002969882 | 0.014389151 | down |
| RP11-55L3.1 | -1.761273813 | 0.546857165 | | down |
| RP11-364B6.2 | -1.761191051 | 0.361430852 | | down |
| RP11-687M24.7 | -1.761186059 | 0.318943721 | | down |
| CTB-13F3.1 | -1.761077151 | 0.000476927 | 0.003348634 | down |

| | | | | |
|---|---|---|---|---|
| RP11-736K20.6 | -1.761024211 | 0.000659513 | 0.004347236 | down |
| RP11-746B8.1 | -1.76094298 | 0.463424876 | | down |
| ZSWIM5P2 | -1.760884418 | 0.223226442 | | down |
| GPR139 | -1.760827096 | 0.54695931 | | down |
| AC079145.4 | -1.76082569 | 0.009390552 | 0.034947324 | down |
| CCDC27 | -1.760798108 | 0.096510099 | 0.197100769 | down |
| CHST5 | -1.760719063 | 9.44E-06 | 0.000134712 | down |
| KLK5 | -1.760567581 | 0.365891065 | | down |
| AC016995.3 | -1.760480969 | 0.000496136 | 0.003453639 | down |
| ANKRD60 | -1.760382549 | 0.546986865 | | down |
| NA | -1.760354703 | 0.004064504 | 0.018474124 | down |
| UBTFL8 | -1.760316464 | 0.143916995 | 0.261354098 | down |
| C7orf69 | -1.760247283 | 0.173321057 | | down |
| RP11-136K14.2 | -1.760175744 | 0.092276102 | | down |
| RP11-230B22.1 | -1.760118732 | 0.001141678 | 0.006741184 | down |
| LINC00592 | -1.760064862 | 0.15912265 | 0.280293205 | down |
| RP11-944C7.1 | -1.75984634 | 0.000511735 | 0.00354416 | down |
| Y_RNA | -1.759691402 | 0.086933439 | 0.182952385 | down |
| NA | -1.759351039 | 0.150496941 | 0.269636238 | down |
| DISP3 | -1.75933625 | 1.15E-06 | 2.29E-05 | down |
| RP11-510N19.3 | -1.759270305 | 0.547241296 | | down |
| RP11-15E18.4 | -1.759270305 | 0.547241296 | | down |
| FAM90A27P | -1.759270305 | 0.547241296 | | down |
| OR2AM1P | -1.759238167 | 0.547248649 | | down |
| TCTE1 | -1.759125875 | 0.002418045 | 0.012250884 | down |
| LRRC8E | -1.75907946 | 0.048356875 | 0.119466652 | down |
| CTA-286B10.8 | -1.758091014 | 0.460170345 | | down |
| RP11-46107.1 | -1.758076408 | 0.000261013 | 0.002084497 | down |
| RP11-20I23.5 | -1.758019618 | 0.011472072 | 0.040774509 | down |
| NA | -1.757990951 | 0.000428644 | 0.00307502 | down |
| RPL34P27 | -1.757830687 | 0.001003635 | 0.006081176 | down |
| SEC14L5 | -1.757795113 | 2.42E-05 | 0.000293062 | down |
| RN7SL368P | -1.757562594 | 0.092355443 | 0.190952234 | down |
| ADAMTS17 | -1.757541432 | 6.62E-11 | 4.72E-09 | down |
| RP11-235G24.1 | -1.757457169 | 0.221466346 | 0.353151897 | down |
| CTC-459M5.1 | -1.757318983 | 0.155310759 | 0.275426902 | down |
| SPDYE5 | -1.756946907 | 1.27E-05 | 0.000172239 | down |
| MIR4466 | -1.756905445 | 0.520303388 | | down |
| CTC-498M16.2 | -1.75666172 | 0.520022761 | | down |
| TFF3 | -1.756643941 | 1.47E-05 | 0.000194441 | down |
| PGGT1BP2 | -1.756609553 | 0.547924144 | | down |
| AC005307.1 | -1.756116458 | 0.052758728 | | down |
| RP1-122K4.2 | -1.756095863 | 0.413809391 | | down |
| OR8H2 | -1.756020017 | 0.547765557 | | down |
| CTC-529L17.1 | -1.755806658 | 0.548033993 | | down |
| NA | -1.75572491 | 0.227512672 | | down |
| CHIAP3 | -1.755697195 | 0.150388906 | 0.269506966 | down |
| CTD-3193013.1 | -1.755295539 | 0.112177984 | | down |
| OR5D14 | -1.755212411 | 0.548243948 | | down |
| PEBP1P2 | -1.755186883 | 0.013665355 | 0.046485724 | down |
| RP5-874C20.6 | -1.754822008 | 0.322414768 | | down |

| | | | | |
|---|---|---|---|---|
| RP11-1396O13.22 | -1.754692814 | 0.548289029 | | down |
| ANKRD1 | -1.754688278 | 0.00502654 | 0.02179183 | down |
| PLA2G4D | -1.754594363 | 0.003231124 | 0.015340692 | down |
| Metazoa_SRP | -1.754558208 | 0.108310108 | | down |
| MED15P6 | -1.754506462 | 0.548405573 | | down |
| TCF3P1 | -1.754506087 | 0.121892042 | | down |
| NDUFS5P3 | -1.754399229 | 0.06347937 | 0.145663652 | down |
| RP11-254F19.3 | -1.75396657 | 0.378046755 | | down |
| MIR3939 | -1.753934526 | 0.276942318 | | down |
| RP11-214K3.21 | -1.753707374 | 0.008066358 | 0.031133822 | down |
| RP4-706G24.1 | -1.753526598 | 0.106731007 | 0.211826291 | down |
| NA | -1.752715616 | 0.025078079 | 0.073477116 | down |
| TLCD2 | -1.752661893 | 4.02E-12 | 3.85E-10 | down |
| GRIK2 | -1.752346794 | 0.000471168 | 0.003318278 | down |
| CTC-525D6.1 | -1.752342783 | 0.325998857 | | down |
| RP11-400F19.12 | -1.75223153 | 0.125475317 | | down |
| CTB-49A3.5 | -1.751894251 | 0.549003834 | | down |
| NA | -1.751815796 | 0.302547636 | 0.440437114 | down |
| RP11-488C13.4 | -1.751733193 | 0.414158979 | | down |
| KRT74 | -1.751422769 | 0.34087965 | | down |
| EN1 | -1.751384917 | 0.281872387 | | down |
| RP11-476F14.1 | -1.751176087 | 0.510562182 | | down |
| PPP1R36 | -1.751046133 | 1.82E-08 | 6.56E-07 | down |
| RP11-21G15.1 | -1.750952003 | 0.155835508 | 0.27613813 | down |
| NA | -1.750756343 | 2.70E-05 | 0.00032129 | down |
| CTD-2251F13.1 | -1.75073404 | 0.549269654 | | down |
| CDKN2A-AS1 | -1.750609088 | 0.465889745 | | down |
| RP1-142F18.2 | -1.750475519 | 0.549328893 | | down |
| RP11-148K1.10 | -1.750417584 | 0.023964502 | 0.071018888 | down |
| RP11-345I18.4 | -1.74995469 | 0.335085805 | | down |
| SHISA6 | -1.749854177 | 0.001745904 | 0.009422301 | down |
| MUC16 | -1.749759024 | 0.003005312 | 0.014516331 | down |
| GOLGA6L2 | -1.749721335 | 0.205038117 | 0.334846181 | down |
| LINC00940 | -1.749695612 | 0.010283193 | 0.037452812 | down |
| RP11-102M11.2 | -1.749447026 | 0.25751828 | | down |
| RORA-AS2 | -1.74902004 | 0.549588818 | | down |
| LINC01053 | -1.748882963 | 0.078957516 | 0.170526893 | down |
| AC002310.10 | -1.748814893 | 0.099095801 | 0.2008117 | down |
| RP11-526K17.2 | -1.748704223 | 0.549734862 | | down |
| CLCN1 | -1.748448184 | 0.000121354 | 0.001112598 | down |
| NEK4P3 | -1.748399222 | 0.549804781 | | down |
| PKDREJ | -1.748384266 | 4.40E-06 | 7.13E-05 | down |
| KIAA1549L | -1.748275373 | 0.001219955 | 0.007105918 | down |
| RP11-1166P10.6 | -1.748228047 | 0.235995392 | | down |
| RP11-288G3.3 | -1.747910279 | 0.549916877 | | down |
| NA | -1.747698192 | 0.057321142 | | down |
| CYP4F62P | -1.747501557 | 0.526339005 | | down |
| AC013401.1 | -1.747215327 | 0.412635088 | | down |
| SLITRK2 | -1.74715741 | 0.001085663 | 0.006479057 | down |
| AKR1D1P1 | -1.747146562 | 0.463971403 | | down |
| RP11-173D14.3 | -1.747138611 | 0.51715254 | | down |

| | | | | |
|---|---|---|---|---|
| NA | -1.747061605 | 0.003266497 | 0.015476841 | down |
| RN7SL364P | -1.747005225 | 0.001140264 | 0.006738412 | down |
| CTA-221G9.7 | -1.746867048 | 0.014658781 | 0.049065108 | down |
| CTC-459F4.7 | -1.746802507 | 0.550170886 | | down |
| RPSAP11 | -1.746575584 | 0.001479213 | 0.008240587 | down |
| RP11-886P16.10 | -1.746552795 | 0.106358634 | | down |
| RP11-337N6.2 | -1.746407465 | 0.004251263 | 0.019131958 | down |
| AADACL3 | -1.746127921 | 0.550325595 | | down |
| OR10H4 | -1.745515533 | 0.550319837 | | down |
| AC004019.13 | -1.745408674 | 0.015043459 | 0.050057113 | down |
| AC034110.1 | -1.745225294 | 0.182205751 | 0.307806701 | down |
| NA | -1.745000155 | 0.550584284 | | down |
| MIR4313 | -1.744988326 | 0.519389191 | | down |
| LINC01182 | -1.744569164 | 0.097251389 | 0.198129498 | down |
| RP11-1003J3.1 | -1.744457974 | 0.461727751 | | down |
| RP11-697H9.3 | -1.74440179 | 0.300429094 | | down |
| NA | -1.744269177 | 0.468255327 | | down |
| RP11-353N14.2 | -1.743818444 | 0.002025046 | 0.010606781 | down |
| LINC00354 | -1.743421261 | 0.307324211 | 0.445641613 | down |
| RNU6-151P | -1.743334648 | 0.418816566 | | down |
| RP11-93O17.2 | -1.74324452 | 0.227192261 | 0.359345317 | down |
| AC007394.3 | -1.742945497 | 0.463517658 | | down |
| SERPINA10 | -1.742894542 | 0.033002259 | 0.089983456 | down |
| MAGEA10 | -1.742802812 | 0.156271859 | 0.276682877 | down |
| CDHR1 | -1.742695374 | 3.76E-06 | 6.25E-05 | down |
| RP11-484L7.2 | -1.742578667 | 0.551139927 | | down |
| RP11-321G12.1 | -1.742476211 | 0.000612265 | 0.004104168 | down |
| UPK1A | -1.742034956 | 0.028502303 | 0.080907332 | down |
| RPL18P13 | -1.742029954 | 0.011638708 | 0.041244792 | down |
| RP11-313F23.4 | -1.74187632 | 0.023569727 | 0.070097888 | down |
| RP11-387H17.6 | -1.741874533 | 0.141764301 | 0.258460262 | down |
| RPL6P9 | -1.741813613 | 0.416033205 | | down |
| SH2D6 | -1.741716169 | 3.13E-05 | 0.000362446 | down |
| GRM7-AS3 | -1.741601048 | 0.280456743 | | down |
| SPDYE9P | -1.741578029 | 0.28094499 | 0.41736112 | down |
| LLNLR-304A6.2 | -1.741445607 | 0.007632605 | 0.02990997 | down |
| WDR88 | -1.741289938 | 2.24E-05 | 0.000273591 | down |
| SYT3 | -1.741076602 | 0.000148148 | 0.001306796 | down |
| CTC-453G23.8 | -1.740662895 | 0.000491707 | 0.003429965 | down |
| DPY19L2P5 | -1.740380744 | 0.551644507 | | down |
| TEX35 | -1.740306624 | 0.017411947 | 0.055883404 | down |
| AC073283.4 | -1.740235502 | 6.36E-06 | 9.73E-05 | down |
| RP11-510D21.1 | -1.74015608 | 0.468119485 | | down |
| Metazoa_SRP | -1.740152193 | 0.416609695 | | down |
| PNCK | -1.740121405 | 0.000265277 | 0.002111251 | down |
| RP11-385F7.1 | -1.739989841 | 4.26E-06 | 6.96E-05 | down |
| ANO9 | -1.739903347 | 5.75E-08 | 1.78E-06 | down |
| RPL5P24 | -1.739850541 | 0.20055757 | | down |
| RP11-362K14.5 | -1.739792797 | 0.008259194 | 0.031682741 | down |
| RP11-700F16.3 | -1.739661597 | 0.525401541 | | down |
| RP11-160H22.5 | -1.739653269 | 8.90E-06 | 0.000128497 | down |

| | | | | |
|---|---|---|---|---|
| RP11-815I22.1 | -1.739578914 | 0.551828641 | | down |
| RP1-300I2.3 | -1.739578914 | 0.551828641 | | down |
| AGGF1P6 | -1.73955851 | 0.551833326 | | down |
| RP11-260M19.2 | -1.739541474 | 0.001354757 | 0.007698565 | down |
| RP5-1128N12.3 | -1.739541387 | 0.551691141 | | down |
| RP11-291H24.1 | -1.739529597 | 0.330364261 | 0.468813352 | down |
| CLYBL-AS2 | -1.739487999 | 0.016448164 | 0.053504618 | down |
| KRTAP10-2 | -1.73937101 | 0.469330576 | | down |
| RP11-433J20.2 | -1.739323661 | 0.206218396 | 0.336152433 | down |
| AC097467.2 | -1.739271343 | 0.182870696 | 0.308643738 | down |
| AC079776.1 | -1.738998831 | 0.022765378 | 0.068280891 | down |
| RP11-439L8.2 | -1.738694009 | 0.552031886 | | down |
| ACTBP12 | -1.738694009 | 0.552031886 | | down |
| RP11-108M11.3 | -1.738694009 | 0.552031886 | | down |
| RP11-587P21.3 | -1.738694009 | 0.552031886 | | down |
| ZNF646P1 | -1.738694009 | 0.552031886 | | down |
| OR7E156P | -1.738694009 | 0.552031886 | | down |
| SNX33P1 | -1.738694009 | 0.552031886 | | down |
| DEFB121 | -1.738694009 | 0.552031886 | | down |
| NA | -1.738694009 | 0.552031886 | | down |
| AC091878.1 | -1.738635308 | 0.00194099 | 0.01022713 | down |
| RP4-790G17.7 | -1.738572958 | 0.418984077 | | down |
| NA | -1.738398672 | 0.109011221 | 0.215172007 | down |
| PROM1 | -1.738349766 | 8.32E-08 | 2.44E-06 | down |
| RP11-267N12.2 | -1.738213336 | 0.552142302 | | down |
| NA | -1.738208891 | 0.552143323 | | down |
| SNRPGP20 | -1.7381283 | 0.518644455 | | down |
| RP11-864G5.1 | -1.737902351 | 0.284656147 | | down |
| NA | -1.737650128 | 0.464036616 | | down |
| AC012370.2 | -1.737626075 | 0.225106009 | | down |
| MT-ND6 | -1.737408845 | 0.000131361 | 0.001184808 | down |
| RP5-859M6.1 | -1.737398233 | 0.139702666 | 0.255912864 | down |
| RP11-703G6.1 | -1.73730461 | 0.551916625 | | down |
| RP11-168L7.3 | -1.736983772 | 0.463164274 | | down |
| RP11-10A14.5 | -1.736940492 | 0.000742167 | 0.004782854 | down |
| HSP90AB4P | -1.73679843 | 0.003526255 | 0.016471205 | down |
| OR9K2 | -1.736747722 | 0.552479037 | | down |
| CTA-221G9.12 | -1.736452606 | 0.002473133 | 0.012462731 | down |
| SLC6A20 | -1.736352794 | 0.000756238 | 0.004856864 | down |
| NDUFAB1P1 | -1.735892184 | 0.052645776 | | down |
| TARID | -1.735618319 | 0.000968962 | 0.005904966 | down |
| RP11-794P6.1 | -1.735500934 | 0.020075943 | 0.062274006 | down |
| MTCO1P2 | -1.735446185 | 0.011036994 | 0.039563763 | down |
| ADGRA1-AS1 | -1.735277138 | 0.519773755 | | down |
| RP11-329N22.1 | -1.735228347 | 0.008882369 | 0.033502768 | down |
| RP11-778H2.1 | -1.735205795 | 0.468818513 | | down |
| HMGB1P36 | -1.735176842 | 0.552840069 | | down |
| RP11-525A16.4 | -1.735024766 | 0.001609614 | 0.008829512 | down |
| AC007563.5 | -1.734866692 | 0.419529758 | | down |
| RP5-1070G24.2 | -1.734547996 | 0.219012227 | | down |
| CNGA1 | -1.734457756 | 2.19E-08 | 7.68E-07 | down |

| | | | | |
|---|---|---|---|---|
| RP5-991C6.4 | -1.734455041 | 0.552932931 | | down |
| PTPRR | -1.734302493 | 8.47E-06 | 0.000123195 | down |
| RP11-829H16.2 | -1.734296721 | 0.127359066 | | down |
| BEND2 | -1.734145914 | 0.174201157 | 0.298684054 | down |
| NEUROD6 | -1.734023412 | 0.553032181 | | down |
| RP11-945A11.1 | -1.734023412 | 0.553032181 | | down |
| RP5-1056L3.1 | -1.733979311 | 0.19112811 | | down |
| RP11-399E6.2 | -1.733620836 | 0.335245346 | 0.473558286 | down |
| TAF9BP1 | -1.733439612 | 0.37800371 | | down |
| RBM47 | -1.733423443 | 1.03E-09 | 5.20E-08 | down |
| OR9I1 | -1.733405429 | 0.55317428 | | down |
| AK3P5 | -1.73339678 | 0.2344713 | | down |
| RPL7AP26 | -1.733391441 | 0.328456344 | | down |
| NDP-AS1 | -1.733341961 | 0.55326192 | | down |
| CTB-14A14.2 | -1.733288374 | 0.553274243 | | down |
| LCN9 | -1.733288374 | 0.553274243 | | down |
| RP11-169L17.3 | -1.733288374 | 0.553274243 | | down |
| NA | -1.733231527 | 0.37535967 | | down |
| NA | -1.732973744 | 0.529434482 | | down |
| MACROD1 | -1.732807366 | 2.28E-08 | 7.91E-07 | down |
| RP11-626P14.2 | -1.732770088 | 0.018778185 | 0.059193347 | down |
| PAGE1 | -1.732452197 | 0.125029412 | 0.236694349 | down |
| RP11-1069G10.2 | -1.732211107 | 0.110494602 | | down |
| RP5-1018K9.1 | -1.732112297 | 0.327371824 | | down |
| RP11-624L4.2 | -1.732059276 | 0.194625279 | | down |
| RP11-388M20.7 | -1.732008896 | 0.115131551 | 0.223411451 | down |
| Z95704.3 | -1.731827409 | 0.553537212 | | down |
| GLYCTK | -1.731736973 | 1.12E-15 | 2.59E-13 | down |
| CTB-131B5.5 | -1.731692494 | 1.25E-05 | 0.000170086 | down |
| TRIM60P17 | -1.731177302 | 0.086100202 | 0.181810125 | down |
| MIR5688 | -1.731033827 | 0.350780961 | 0.48947393 | down |
| RP11-33I11.2 | -1.730963589 | 0.001898163 | 0.010056147 | down |
| SLC9C2 | -1.730770193 | 0.073285919 | 0.161647614 | down |
| FAM170B | -1.730486947 | 0.023934638 | 0.070944383 | down |
| KCNA4 | -1.730253469 | 0.060404869 | 0.140453826 | down |
| RP11-962G15.1 | -1.730056205 | 0.214712591 | 0.345726141 | down |
| RP11-481J8.3 | -1.729819919 | 0.420038478 | | down |
| RP11-534L6.5 | -1.729789728 | 0.554079046 | | down |
| RP11-290K4.2 | -1.729434513 | 0.46706065 | | down |
| RP11-328C8.4 | -1.729330587 | 0.262860794 | | down |
| AC053503.11 | -1.729198755 | 0.059109398 | 0.138233266 | down |
| RP11-665J16.1 | -1.729135453 | 0.467854487 | | down |
| RP11-532F12.5 | -1.728826808 | 1.05E-07 | 2.96E-06 | down |
| RP11-398A8.1 | -1.728765714 | 0.420458007 | | down |
| OR6S1 | -1.728503077 | 0.163570483 | 0.285832117 | down |
| FER1L6 | -1.728494241 | 0.00129724 | 0.0074421 | down |
| IMPDH1P11 | -1.728296631 | 0.473522288 | | down |
| CTD-2501M5.1 | -1.728290325 | 0.422740748 | | down |
| YES1P1 | -1.727803365 | 0.000447918 | 0.00318892 | down |
| BNIP3P16 | -1.727744968 | 0.050901628 | 0.123909294 | down |
| HOXC10 | -1.727536762 | 0.021394901 | 0.065231218 | down |

| | | | | |
|---|---|---|---|---|
| NA | -1.727508522 | 0.083999623 | 0.17837135 | down |
| RORB-AS1 | -1.727421801 | 0.226373469 | 0.358484319 | down |
| RN7SKP299 | -1.727195687 | 0.269132429 | 0.404938637 | down |
| RN7SL587P | -1.726988316 | 0.036347913 | 0.096756984 | down |
| KLK3 | -1.726842276 | 0.000285602 | 0.002239786 | down |
| C9orf135-AS1 | -1.726777742 | 0.470584318 | 0.601210401 | down |
| OR4C13 | -1.726316853 | 0.554878376 | | down |
| C10orf53 | -1.726282324 | 0.421549429 | | down |
| RPL23AP32 | -1.725860504 | 0.008009994 | 0.030999906 | down |
| PLD5 | -1.7255301 | 0.002329511 | 0.011882483 | down |
| NA | -1.725423297 | 0.000276994 | 0.002187702 | down |
| NA | -1.725241775 | 0.523550578 | | down |
| RP11-319G9.3 | -1.724994502 | 6.23E-05 | 0.000639708 | down |
| RP11-10J21.6 | -1.724831186 | 0.468362487 | | down |
| NA | -1.724688 | 0.068578163 | 0.154011238 | down |
| LATS2-AS1 | -1.724503312 | 0.46847912 | | down |
| TNPO1P1 | -1.724475275 | 0.050996032 | | down |
| RP11-832N8.1 | -1.724326326 | 0.019288104 | | down |
| ERBB4 | -1.724208051 | 0.006417544 | 0.026265264 | down |
| RP11-411B10.5 | -1.724140971 | 0.555306678 | | down |
| BNIP3P8 | -1.724086284 | 0.290185095 | 0.42739934 | down |
| Y_RNA | -1.723823546 | 0.007181377 | 0.028521011 | down |
| ADH1A | -1.723751398 | 0.117647603 | | down |
| RP11-622C24.2 | -1.723729616 | 0.006886914 | 0.027654432 | down |
| NA | -1.72358859 | 0.555506711 | | down |
| AC005522.6 | -1.723512768 | 0.555308286 | | down |
| FAM183CP | -1.723512768 | 0.555308286 | | down |
| RP11-217E13.1 | -1.723372967 | 0.299038513 | | down |
| GOLGA6L17P | -1.723317638 | 0.055054649 | 0.131191456 | down |
| RP11-798K23.3 | -1.723286464 | 0.42423669 | | down |
| MTND2P18 | -1.723175386 | 0.555601905 | | down |
| TRAPPC3L | -1.723133078 | 0.042346889 | 0.108444421 | down |
| C2orf57 | -1.723126732 | 0.47336409 | | down |
| ARSE | -1.722705424 | 3.48E-06 | 5.89E-05 | down |
| LY6L | -1.722039477 | 0.342197404 | | down |
| CTD-2269F5.1 | -1.721992875 | 3.82E-06 | 6.34E-05 | down |
| RP11-16L21.7 | -1.72197915 | 0.252402604 | | down |
| NA | -1.721735215 | 0.045244416 | 0.113653416 | down |
| RP11-756J15.3 | -1.72173104 | 0.525308283 | | down |
| RP11-40H20.5 | -1.721712403 | 0.528549378 | | down |
| UGT2B28 | -1.721629996 | 0.24058314 | | down |
| ASCL4 | -1.721507587 | 0.004247067 | 0.019121665 | down |
| RPS5P2 | -1.721269195 | 0.130650527 | 0.244229992 | down |
| CTC-429C10.7 | -1.721177733 | 0.555916709 | | down |
| CYP4F25P | -1.721115529 | 0.001069309 | 0.006393296 | down |
| MMP24 | -1.720816038 | 8.78E-07 | 1.82E-05 | down |
| RP11-428P16.3 | -1.72076361 | 0.215295303 | 0.346355479 | down |
| AF196972.3 | -1.720647223 | 0.246015308 | | down |
| CLEC4M | -1.720605339 | 0.000525307 | 0.003621462 | down |
| HMGN2P8 | -1.720490015 | 0.530719239 | | down |
| RNU6ATAC42P | -1.720382401 | 0.52892172 | | down |

| | | | | |
|---|---|---|---|---|
| RP11-95M5.1 | -1.720301833 | 0.474841949 | | down |
| RP1-308E4.1 | -1.719960529 | 0.374549229 | | down |
| RP11-107E5.3 | -1.719927271 | 0.036183376 | 0.09645048 | down |
| SPATA31D1 | -1.719658868 | 0.556412354 | | down |
| CTC-529P8.1 | -1.719487052 | 0.051904514 | 0.125557104 | down |
| RP11-260A9.6 | -1.719338679 | 0.556486176 | | down |
| RHCE | -1.719079403 | 0.002808948 | 0.013773795 | down |
| CTD-2022H16.1 | -1.719012213 | 0.520035809 | | down |
| RP11-419I17.2 | -1.719009458 | 0.032561586 | 0.089088453 | down |
| RP13-492C18.2 | -1.718930144 | 0.556080931 | | down |
| WFDC12 | -1.718757021 | 0.01888626 | 0.059440477 | down |
| NA | -1.718728418 | 0.159867907 | | down |
| RP11-380O24.1 | -1.718482592 | 0.002341505 | 0.01192544 | down |
| LINC01305 | -1.718243997 | 0.015113453 | 0.05023994 | down |
| AC073257.1 | -1.717765234 | 0.026965223 | 0.0775702 | down |
| RP11-1069G10.1 | -1.717745103 | 4.88E-05 | 0.000524611 | down |
| RP11-97E7.2 | -1.717733193 | 0.357397907 | 0.495805639 | down |
| RP11-111A21.1 | -1.717712073 | 0.000403817 | 0.002933338 | down |
| LL22NC03-86D4.1 | -1.717583516 | 0.217651298 | 0.349050688 | down |
| AC002069.5 | -1.717559571 | 0.474798592 | | down |
| ZNF676 | -1.717407373 | 0.005153037 | 0.022196106 | down |
| ANO1-AS2 | -1.717348784 | 0.376738976 | | down |
| SLC29A2 | -1.717331971 | 4.49E-11 | 3.37E-09 | down |
| NA | -1.717289217 | 0.003060236 | 0.014720098 | down |
| KCNK16 | -1.717235458 | 0.292393304 | | down |
| LYRM7 | -1.717165147 | 1.56E-10 | 9.97E-09 | down |
| PABPC1L2B | -1.716879157 | 0.296053282 | | down |
| RP11-340I6.11 | -1.716609745 | 0.557115544 | | down |
| BMPR1B | -1.71659973 | 0.002621067 | 0.013046365 | down |
| RP11-1336O20.2 | -1.716578338 | 0.061967208 | 0.143155082 | down |
| LINC01555 | -1.716562514 | 0.035262049 | 0.094520092 | down |
| CTB-187L3.1 | -1.71652552 | 0.179935155 | 0.305227611 | down |
| GS1-122H1.1 | -1.716486231 | 0.557144038 | | down |
| NA | -1.716486231 | 0.557144038 | | down |
| NA | -1.716486231 | 0.557144038 | | down |
| LINC00620 | -1.716486231 | 0.557144038 | | down |
| RPSAP33 | -1.716486231 | 0.557144038 | | down |
| RP11-366M4.8 | -1.716486231 | 0.557144038 | | down |
| GS1-111G14.1 | -1.716486231 | 0.557144038 | | down |
| MYO5BP1 | -1.716486231 | 0.557144038 | | down |
| RP11-65B23.5 | -1.716486231 | 0.557144038 | | down |
| OR9I2P | -1.716486231 | 0.557144038 | | down |
| SFR1P2 | -1.716486231 | 0.557144038 | | down |
| MIR6087 | -1.71630157 | 0.029226072 | 0.082339469 | down |
| DRICH1 | -1.716139006 | 3.46E-05 | 0.000393473 | down |
| RP11-666A20.3 | -1.716043312 | 0.557246223 | | down |
| TCEB1P29 | -1.715907407 | 0.47750785 | | down |
| RP11-323P17.2 | -1.715877401 | 0.000546002 | 0.003736693 | down |
| XAGE3 | -1.715611065 | 0.343800181 | | down |
| NA | -1.715555533 | 0.557358767 | | down |
| RP11-806O11.1 | -1.715526665 | 0.000620824 | 0.004148619 | down |

| | | | | |
|---|---|---|---|---|
| TRHDE | -1.715348583 | 2.77E-06 | 4.88E-05 | down |
| RP4-814D15.1 | -1.715124271 | 0.052898311 | | down |
| RP11-432J24.5 | -1.715076166 | 0.031318918 | 0.08656353 | down |
| NA | -1.714932902 | 0.289990512 | 0.427186808 | down |
| RP11-573G6.10 | -1.714880091 | 0.006962684 | 0.027884148 | down |
| DNAI2 | -1.71438378 | 0.000322869 | 0.002473928 | down |
| KCNJ14 | -1.714274616 | 5.21E-05 | 0.000553375 | down |
| LINC00895 | -1.713822228 | 0.372981756 | | down |
| XX-CR54.1 | -1.713754673 | 0.157436185 | 0.278170592 | down |
| RP5-968D22.3 | -1.713694651 | 0.006654355 | 0.02697294 | down |
| ACSBG2 | -1.713444806 | 0.022580859 | 0.067909843 | down |
| DACH2 | -1.713249556 | 0.003127476 | 0.014969235 | down |
| HMX1 | -1.713191233 | 0.097477501 | | down |
| NA | -1.713036236 | 0.016057263 | 0.052574585 | down |
| ZNF578 | -1.713015991 | 1.27E-05 | 0.000172121 | down |
| NA | -1.712991771 | 0.557950473 | | down |
| AC096570.1 | -1.712991771 | 0.557950473 | | down |
| AC016561.1 | -1.712991771 | 0.557950473 | | down |
| RP11-675M1.2 | -1.712991771 | 0.557950473 | | down |
| RP11-563K23.1 | -1.712940417 | 0.115310675 | | down |
| AC011406.2 | -1.712922885 | 0.529997753 | | down |
| RP5-856G1.1 | -1.712861203 | 0.297497833 | | down |
| KRT8P2 | -1.712622362 | 0.558035746 | | down |
| DEFB105B | -1.712449379 | 0.38022003 | | down |
| HRAT92 | -1.712019658 | 4.29E-05 | 0.000469732 | down |
| GAB4 | -1.711869921 | 0.242420336 | | down |
| RNU6-910P | -1.711560521 | 0.479246062 | 0.608711791 | down |
| FGF14 | -1.71104304 | 0.000677872 | 0.004442238 | down |
| EEF1A1P17 | -1.710998068 | 0.116765614 | | down |
| OR4D7P | -1.710972647 | 0.557918041 | | down |
| FEV | -1.710494629 | 2.84E-05 | 0.000334476 | down |
| PRKX-AS1 | -1.710427182 | 0.216036926 | 0.347110188 | down |
| LHCGR | -1.710380679 | 0.249631192 | | down |
| LINC00482 | -1.710180267 | 3.28E-07 | 7.82E-06 | down |
| CTD-3193013.8 | -1.710079664 | 0.298086428 | | down |
| RFC3P1 | -1.710054544 | 0.55862866 | | down |
| NA | -1.709610588 | 0.558731201 | | down |
| MIR425 | -1.709489467 | 0.297269267 | | down |
| NA | -1.709460052 | 0.342901645 | | down |
| ADAMTS7P1 | -1.709390236 | 0.000243506 | 0.001968224 | down |
| NA | -1.709370073 | 0.333785057 | | down |
| RP11-475C16.2 | -1.709349183 | 0.558168586 | | down |
| NA | -1.709208746 | 0.473409475 | | down |
| AF038458.5 | -1.709120898 | 0.121911012 | 0.232611105 | down |
| RP11-143J24.1 | -1.70877783 | 0.008813985 | 0.033304913 | down |
| FAM3D-AS1 | -1.708758914 | 0.013978373 | 0.04732547 | down |
| NA | -1.708597648 | 0.115589909 | | down |
| HIRAP1 | -1.708157716 | 0.558994853 | | down |
| RP5-905G11.3 | -1.708065228 | 0.097350409 | 0.19818539 | down |
| KY | -1.70804045 | 0.000120581 | 0.001107875 | down |
| CSPG4P11 | -1.70790165 | 0.125374791 | | down |

| | | | | |
|---|---|---|---|---|
| MAP1LC3BP1 | -1.70782741 | 0.231742385 | | down |
| RP11-96A1.4 | -1.707665473 | 0.559180573 | | down |
| FAM182A | -1.707661595 | 0.032890177 | 0.089758605 | down |
| AC079776.2 | -1.70741369 | 0.002588812 | 0.012929112 | down |
| CH507-528H12.1 | -1.707057237 | 0.011417387 | 0.040610473 | down |
| NA | -1.707052025 | 0.559322332 | | down |
| TNP2 | -1.707020325 | 0.559257757 | | down |
| SALL1P1 | -1.706923019 | 0.559280252 | | down |
| RP11-567M16.1 | -1.706640108 | 0.000279001 | 0.002200083 | down |
| PIGZ | -1.706605725 | 3.18E-09 | 1.44E-07 | down |
| CTD-2532D12.4 | -1.706466214 | 0.339137728 | | down |
| RNU4ATAC5P | -1.706418424 | 0.559396908 | | down |
| AC090505.4 | -1.706342013 | 0.267251311 | 0.40297608 | down |
| C5orf67 | -1.706145589 | 0.004482827 | 0.019944034 | down |
| RP11-56P9.10 | -1.705677655 | 0.55963999 | | down |
| TULP2 | -1.705490257 | 0.000125878 | 0.001144635 | down |
| C16orf82 | -1.705455906 | 0.168218788 | 0.291580527 | down |
| RP11-360N9.2 | -1.70536546 | 0.00094219 | 0.00577697 | down |
| AIM1L | -1.704951232 | 1.47E-06 | 2.86E-05 | down |
| LINC01168 | -1.704854369 | 0.246884746 | | down |
| NA | -1.704843498 | 0.004407718 | 0.019668122 | down |
| RP1-240B8.3 | -1.704652444 | 0.046582098 | 0.116074692 | down |
| SPINT1 | -1.704647363 | 2.74E-07 | 6.76E-06 | down |
| CTD-2356P16.6 | -1.704553338 | 0.521203925 | | down |
| AC006960.7 | -1.70446297 | 0.478284631 | | down |
| FAM157B | -1.704292796 | 0.085481885 | 0.18084767 | down |
| AP006621.9 | -1.704205645 | 0.053795362 | 0.128947492 | down |
| TOMM22P6 | -1.704054761 | 0.560015202 | | down |
| MARVELD3 | -1.70386768 | 3.01E-07 | 7.28E-06 | down |
| RP11-261P13.5 | -1.703683108 | 0.043727306 | 0.111004916 | down |
| RP11-87E22.2 | -1.7035343 | 0.530172443 | | down |
| ZNF57 | -1.70349547 | 0.005643839 | 0.023785034 | down |
| AC003991.3 | -1.703279093 | 0.000686279 | 0.004487177 | down |
| AC006262.6 | -1.703223815 | 0.034945439 | 0.093942444 | down |
| RP11-1E11.1 | -1.70315776 | 0.191586576 | | down |
| ALG1L8P | -1.703114563 | 0.000900357 | 0.005567072 | down |
| TCEB3-AS1 | -1.703039092 | 9.33E-06 | 0.000133534 | down |
| NA | -1.703024728 | 0.244844442 | 0.378770734 | down |
| MTRNR2L1 | -1.702822928 | 0.006113476 | 0.025313297 | down |
| PCDHA12 | -1.702353704 | 0.13569327 | | down |
| RP4-56404.1 | -1.702309992 | 0.560418725 | | down |
| RP5-875H18.4 | -1.702188611 | 0.071817264 | | down |
| RP11-654A16.3 | -1.702129923 | 0.158804995 | | down |
| NA | -1.702122915 | 6.42E-06 | 9.81E-05 | down |
| RP11-118B18.2 | -1.70171767 | 0.302968857 | | down |
| PHBP21 | -1.701416274 | 0.260658305 | | down |
| AC005539.2 | -1.701296153 | 0.47306745 | | down |
| SLC35G1 | -1.7009226 | 7.53E-08 | 2.25E-06 | down |
| GLOD5 | -1.700757614 | 1.34E-06 | 2.63E-05 | down |
| RP11-1074012.1 | -1.700738048 | 0.045044053 | 0.113313534 | down |
| ZFR2 | -1.700601247 | 0.000118032 | 0.001088941 | down |

| | | | | |
|---|---|---|---|---|
| KCNIP4 | -1.700510156 | 0.000307234 | 0.002377781 | down |
| HYI-AS1 | -1.700444637 | 0.141634084 | | down |
| HORMAD1 | -1.700199792 | 0.003348447 | 0.015782982 | down |
| NOG | -1.700193045 | 0.000274603 | 0.00217167 | down |
| RP11-382A20.6 | -1.7001897 | 0.185705723 | | down |
| CTD-2207A17.1 | -1.699985696 | 0.212244602 | 0.342979606 | down |
| CRB2 | -1.699926851 | 3.13E-06 | 5.38E-05 | down |
| KRT18P21 | -1.699701275 | 0.560878295 | | down |
| RP11-297L17.2 | -1.699475055 | 0.000246898 | 0.001990825 | down |
| ILDR2 | -1.699252682 | 6.69E-05 | 0.000677332 | down |
| RP4-616B8.5 | -1.699076562 | 0.000290502 | 0.002272873 | down |
| RP11-285C1.2 | -1.699048914 | 0.070550497 | 0.157276777 | down |
| NA | -1.698941105 | 0.561126847 | | down |
| BEND3P2 | -1.698763756 | 0.349805742 | | down |
| RP11-496D24.2 | -1.698662347 | 0.025787645 | 0.074994097 | down |
| CH17-353B19.1 | -1.698421287 | 0.044435746 | 0.112209544 | down |
| RPS27P15 | -1.698164829 | 0.535350596 | | down |
| RP1-197J16.1 | -1.698115224 | 0.379438994 | | down |
| RNF182 | -1.698050018 | 0.015728636 | 0.051792101 | down |
| CUX2P1 | -1.69792943 | 0.561432344 | | down |
| RP11-442N1.2 | -1.697682568 | 0.56148949 | | down |
| RN7SL543P | -1.697290682 | 0.338282592 | 0.476705544 | down |
| RP11-321A17.3 | -1.697266268 | 0.007811679 | 0.030424433 | down |
| RP11-903H12.3 | -1.696717064 | 0.247565869 | | down |
| RP11-764K9.1 | -1.696576944 | 0.127609058 | 0.24008307 | down |
| PCSK5 | -1.6964138 | 3.24E-11 | 2.57E-09 | down |
| LINC01208 | -1.696296449 | 0.207741321 | 0.337885096 | down |
| TINAG | -1.696035125 | 4.50E-07 | 1.02E-05 | down |
| RNU6-37P | -1.696009787 | 0.046408939 | 0.115730183 | down |
| AC022201.4 | -1.695920316 | 0.379519124 | | down |
| RN7SL472P | -1.695911467 | 0.234376482 | 0.367151938 | down |
| SPDYC | -1.695883057 | 0.00702903 | 0.028089943 | down |
| RNU6-379P | -1.695836297 | 0.260678891 | 0.395867196 | down |
| HAS2-AS1 | -1.69581319 | 9.07E-05 | 0.000872397 | down |
| KRT18P32 | -1.695438685 | 0.562009057 | | down |
| RP11-526H11.1 | -1.695188426 | 0.158473897 | 0.279527374 | down |
| AC079779.6 | -1.695139642 | 0.480255479 | | down |
| LINC00347 | -1.69511161 | 0.396047201 | 0.533105837 | down |
| RP11-45P15.2 | -1.695028721 | 0.385085134 | | down |
| MRPS10P2 | -1.694998706 | 0.561682055 | | down |
| RP11-1C8.5 | -1.694932464 | 0.075752892 | 0.165485341 | down |
| NA | -1.694580541 | 0.562064434 | | down |
| OPN4 | -1.694228175 | 0.005596234 | 0.023644108 | down |
| SERPINB12 | -1.694165372 | 0.561946057 | | down |
| AC068831.10 | -1.694019915 | 0.000646878 | 0.004280793 | down |
| AC016727.3 | -1.693970772 | 0.334387595 | 0.472642701 | down |
| SYCP1 | -1.693968337 | 0.184536043 | 0.310564692 | down |
| TARDBPP1 | -1.693929416 | 0.562358654 | | down |
| AC087163.2 | -1.69376199 | 0.028714154 | 0.08128619 | down |
| CTD-3046C4.1 | -1.69375454 | 0.562399168 | | down |
| KB-1460A1.2 | -1.693689797 | 0.294045658 | | down |

| | | | | |
|---|---|---|---|---|
| B3GALT5 | -1.693636157 | 4.61E-06 | 7.42E-05 | down |
| NA | -1.693571099 | 0.382272683 | | down |
| AZU1 | -1.693464358 | 0.000386341 | 0.002841556 | down |
| RP11-221H10.2 | -1.693270917 | 0.56236798 | | down |
| SSTR1 | -1.692948851 | 0.000212908 | 0.001760698 | down |
| RP4-738P15.4 | -1.692805218 | 0.562405613 | | down |
| HTR3D | -1.69274467 | 0.47561126 | | down |
| ENO4 | -1.692727548 | 0.01575099 | 0.051837303 | down |
| SERPINC1 | -1.692703118 | 0.008645718 | 0.032772473 | down |
| RN7SKP232 | -1.69257253 | 0.562673043 | | down |
| Y_RNA | -1.69248995 | 0.562692179 | | down |
| PRSS29P | -1.692242402 | 0.486225786 | | down |
| USP24P1 | -1.692134956 | 0.562774446 | | down |
| MIR148B | -1.691820019 | 0.42950712 | | down |
| RP4-536B24.3 | -1.69168246 | 0.020254261 | 0.062720989 | down |
| EXPH5 | -1.691606036 | 1.22E-08 | 4.76E-07 | down |
| RP3-468B3.2 | -1.691432014 | 0.008892446 | 0.033529699 | down |
| CHRNA1 | -1.691237917 | 0.00072382 | 0.004687744 | down |
| RPL36AP29 | -1.690944061 | 0.068522436 | 0.153947782 | down |
| RP11-494O16.4 | -1.690826252 | 0.057346606 | 0.135162895 | down |
| NA | -1.690639863 | 0.563120985 | | down |
| RNASE7 | -1.690631443 | 0.10092331 | | down |
| CGN | -1.69048678 | 5.81E-08 | 1.80E-06 | down |
| CATSPERG | -1.690484279 | 1.12E-07 | 3.12E-06 | down |
| RP11-103H7.1 | -1.690434523 | 0.563168587 | | down |
| NA | -1.690285256 | 0.562918994 | | down |
| CALR4P | -1.690187051 | 0.0001582 | 0.00137807 | down |
| RNU7-77P | -1.690182254 | 0.152786236 | 0.27227651 | down |
| SDCBPP3 | -1.690086075 | 0.279246713 | | down |
| LINC00345 | -1.690069875 | 0.112002388 | | down |
| RP4-565E6.1 | -1.689957518 | 0.007527516 | 0.029563999 | down |
| CTB-50L17.16 | -1.689679906 | 0.002297552 | 0.011757395 | down |
| RPL7AP64 | -1.689590616 | 0.008047381 | 0.031089872 | down |
| AC002069.6 | -1.68921789 | 0.56345067 | | down |
| RP11-78A19.2 | -1.68921789 | 0.56345067 | | down |
| AC005740.3 | -1.689182609 | 0.257086687 | | down |
| NA | -1.689006936 | 0.000811983 | 0.00513908 | down |
| DLG1-AS1 | -1.688943801 | 0.003833407 | 0.017623701 | down |
| MTATP6P27 | -1.68892411 | 0.068763238 | 0.154334583 | down |
| SNORA26 | -1.688847471 | 0.194265507 | 0.322477092 | down |
| RP11-171I2.1 | -1.688785962 | 0.386581172 | | down |
| RP11-733C7.1 | -1.688737739 | 0.293510834 | 0.43083054 | down |
| HPX | -1.688643343 | 1.77E-08 | 6.42E-07 | down |
| HSPE1P7 | -1.688528607 | 0.124213585 | 0.235659675 | down |
| RN7SL354P | -1.688464508 | 0.406805258 | 0.543311198 | down |
| OR5H1 | -1.68828796 | 0.563666324 | | down |
| MIF | -1.688229182 | 0.045106097 | 0.113403144 | down |
| ACTBP9 | -1.68812989 | 0.014823927 | 0.049496562 | down |
| PODXL2 | -1.687783953 | 5.25E-07 | 1.17E-05 | down |
| ARR3 | -1.687731316 | 5.01E-05 | 0.000536549 | down |
| AC004237.1 | -1.68765825 | 0.048994254 | 0.120575309 | down |

| | | | | |
|---|---|---|---|---|
| SSX5 | -1.687644752 | 0.480724719 | | down |
| RNU1-148P | -1.687375788 | 0.563877898 | | down |
| RP11-417L19.5 | -1.68715569 | 0.011042825 | 0.039567135 | down |
| RP11-267J23.1 | -1.68713384 | 0.191737327 | | down |
| NA | -1.68687 | 0.008657072 | 0.032804148 | down |
| MCCD1 | -1.68682927 | 0.538904163 | | down |
| MIR543 | -1.686604399 | 0.268906985 | 0.404680498 | down |
| RP11-468N14.09 | -1.686460576 | 0.563733863 | | down |
| RP11-284L19.1 | -1.686171279 | 0.563730061 | | down |
| RP11-410N8.1 | -1.685915671 | 0.38444798 | | down |
| KLF17 | -1.685832727 | 0.082865518 | 0.176789856 | down |
| AC010524.2 | -1.685752115 | 0.002074033 | 0.01080377 | down |
| RP11-571L19.7 | -1.685746456 | 0.277186973 | | down |
| TRIM34 | -1.685689912 | 0.003155939 | 0.015056012 | down |
| RP11-363H12.1 | -1.685681361 | 0.242766972 | | down |
| EIF2B5-AS1 | -1.685530232 | 0.222536427 | | down |
| TCF4-AS1 | -1.685446569 | 0.275647589 | | down |
| AC019129.1 | -1.685341019 | 0.217992657 | 0.349318438 | down |
| OR2A9P | -1.68494507 | 1.72E-05 | 0.000221198 | down |
| RP11-245J24.1 | -1.684881256 | 0.539310486 | | down |
| RP4-671014.5 | -1.684852809 | 0.015548316 | 0.051350293 | down |
| RP11-432M8.9 | -1.684681404 | 0.564503071 | | down |
| PKD1P1 | -1.684321244 | 0.04497906 | 0.113178465 | down |
| AL672294.1 | -1.684318907 | 0.03222119 | 0.088390562 | down |
| DMRTB1 | -1.68386476 | 0.56469262 | | down |
| CNN2P8 | -1.68386476 | 0.56469262 | | down |
| CTC-436K13.4 | -1.68386476 | 0.56469262 | | down |
| KLHL1 | -1.68386476 | 0.56469262 | | down |
| RP11-640I15.2 | -1.68386476 | 0.56469262 | | down |
| RP11-846F4.11 | -1.68386476 | 0.56469262 | | down |
| TEX26-AS1 | -1.683732836 | 0.011234084 | 0.040085132 | down |
| LINC01220 | -1.683667449 | 0.000106787 | 0.00099885 | down |
| KLHL10 | -1.683596802 | 0.01014529 | 0.037080911 | down |
| RP11-374A4.1 | -1.683396388 | 0.119951028 | 0.230098881 | down |
| RP4-697P8.3 | -1.683356582 | 0.164691587 | 0.287273226 | down |
| PPIAP28 | -1.683309682 | 0.183699067 | | down |
| NCLP1 | -1.683294097 | 0.018046811 | 0.057497179 | down |
| TMEM105 | -1.683243024 | 0.000310165 | 0.002395524 | down |
| AC007204.3 | -1.683015958 | 0.357358972 | 0.495774518 | down |
| CHRM2 | -1.682559647 | 0.025332822 | 0.074029696 | down |
| RP11-59E19.3 | -1.682516267 | 0.261676703 | 0.396794863 | down |
| NA | -1.682205715 | 0.078646496 | | down |
| PDYN | -1.682204769 | 0.390630836 | | down |
| AC012594.1 | -1.681897998 | 0.176743983 | 0.301689691 | down |
| RP5-1086L22.1 | -1.681696066 | 0.243254781 | | down |
| CTD-2529021.2 | -1.681673535 | 0.118165097 | | down |
| CAPN14 | -1.681595496 | 0.001455765 | 0.008141662 | down |
| RNU4-52P | -1.681534877 | 0.286861853 | | down |
| CRYGN | -1.681034254 | 0.003686141 | 0.017069424 | down |
| RP11-546K22.1 | -1.680879261 | 0.004645608 | 0.020495248 | down |
| RPS23P6 | -1.680874239 | 0.000832298 | 0.005240579 | down |

| | | | | |
|---|---|---|---|---|
| RNY4P36 | -1.680723774 | 0.565351936 | | down |
| RP1-167A14.2 | -1.680663436 | 0.002632341 | 0.013093336 | down |
| HNRNPA1P33 | -1.680615502 | 0.002056526 | 0.010730688 | down |
| AC008753.6 | -1.680570995 | 0.001045098 | 0.006281165 | down |
| RP4-736I12.1 | -1.680422346 | 0.565491963 | | down |
| RP11-674I16.1 | -1.680422346 | 0.565491963 | | down |
| RP4-594A5.1 | -1.680422346 | 0.565491963 | | down |
| RP11-583F24.5 | -1.680422346 | 0.565491963 | | down |
| LINC00564 | -1.680422346 | 0.565491963 | | down |
| SIGLEC31P | -1.680422346 | 0.565491963 | | down |
| RP3-461P17.9 | -1.680422346 | 0.565491963 | | down |
| AP000959.2 | -1.680422346 | 0.565491963 | | down |
| GS1-590J6.2 | -1.680422346 | 0.565491963 | | down |
| RP4-655L22.5 | -1.680422346 | 0.565491963 | | down |
| CTD-2396E7.11 | -1.680406877 | 8.82E-10 | 4.55E-08 | down |
| RP1-7G5.5 | -1.680248603 | 0.43412392 | | down |
| RP11-5N11.2 | -1.680203749 | 0.565400987 | | down |
| RP11-814E24.3 | -1.680203749 | 0.565400987 | | down |
| NA | -1.680056508 | 0.018082666 | 0.057593089 | down |
| AC009963.4 | -1.67982367 | 0.220636665 | 0.352306392 | down |
| RP11-193H5.1 | -1.67963839 | 0.565461794 | | down |
| RSL24D1P9 | -1.679187431 | 0.224150296 | 0.356391379 | down |
| SMARCA5-AS1 | -1.67885164 | 0.025080143 | 0.073477116 | down |
| RPF2P1 | -1.678764699 | 0.135306384 | | down |
| CTD-3080F16.3 | -1.678699725 | 0.39583994 | 0.532981434 | down |
| RP11-123J14.4 | -1.678533746 | 0.221694627 | | down |
| HNF1A | -1.678183386 | 7.15E-05 | 0.000714563 | down |
| RP11-33A14.1 | -1.677774259 | 0.144612075 | 0.262173486 | down |
| U1 | -1.677552677 | 0.0243621 | 0.071913435 | down |
| RPL23AP83 | -1.677533403 | 0.197071378 | 0.325659341 | down |
| SERPINA11 | -1.677381892 | 0.010726944 | 0.038703051 | down |
| RP11-248G5.9 | -1.677359966 | 0.0125743 | 0.043679299 | down |
| RP11-489G11.3 | -1.6772985 | 0.252368605 | | down |
| MIR3181 | -1.677282283 | 0.323501085 | | down |
| LINC01375 | -1.67699539 | 0.566288251 | | down |
| RP5-1029F21.3 | -1.676941446 | 0.087837111 | 0.184263958 | down |
| RP11-96L7.2 | -1.676831136 | 0.280707684 | | down |
| SHROOM1 | -1.676726497 | 5.50E-12 | 5.12E-10 | down |
| AKR7A3 | -1.676430559 | 2.32E-07 | 5.87E-06 | down |
| CHTF8P1 | -1.676042214 | 0.348683443 | | down |
| AC009227.2 | -1.675814008 | 0.109539328 | 0.21587403 | down |
| CTBP2P1 | -1.675764627 | 0.566574361 | | down |
| VDAC1P4 | -1.675763555 | 0.307325802 | | down |
| NBEAP1 | -1.675732694 | 0.048543705 | 0.119829682 | down |
| RP11-40F6.1 | -1.675633975 | 0.035411627 | 0.094822364 | down |
| ZNF98 | -1.675275735 | 0.050279525 | 0.122804797 | down |
| Y_RNA | -1.675260479 | 0.063489355 | 0.145664313 | down |
| COL4A6 | -1.675219667 | 0.000185073 | 0.001570714 | down |
| RP11-313C15.1 | -1.675115677 | 0.566725247 | | down |
| uc_338 | -1.674826078 | 0.300745693 | 0.438472539 | down |
| RP11-321M21.4 | -1.674802646 | 0.435212765 | | down |

| | | | | |
|---|---|---|---|---|
| ATP8A2P3 | -1.674457043 | 0.481393981 | | down |
| ACP7 | -1.674369761 | 0.103058247 | 0.206267535 | down |
| RP13-554M15.5 | -1.674243811 | 0.014177433 | 0.047834026 | down |
| CFHR2 | -1.67410282 | 0.566960783 | | down |
| YBX1P9 | -1.674073878 | 0.566967514 | | down |
| ZNF341-AS1 | -1.674010136 | 0.015196145 | 0.050419986 | down |
| LRRC43 | -1.673995966 | 0.000335685 | 0.002555183 | down |
| NA | -1.673842053 | 0.001623137 | 0.008890709 | down |
| RP11-150D5.2 | -1.673781698 | 0.567035468 | | down |
| BOLA2P1 | -1.673781698 | 0.567035468 | | down |
| OR2AI1P | -1.673717583 | 0.566909366 | | down |
| RP11-1084A12.1 | -1.673513059 | 0.032684805 | 0.08934576 | down |
| RP11-215P8.2 | -1.673341619 | 0.002819691 | 0.013809328 | down |
| MYO7B | -1.673336243 | 2.71E-09 | 1.25E-07 | down |
| SMARCE1P5 | -1.673269501 | 0.028554733 | 0.081002587 | down |
| AC017053.1 | -1.673133741 | 0.388232405 | | down |
| RP4-739H11.3 | -1.67306681 | 0.004827341 | 0.021127719 | down |
| RP11-435F13.2 | -1.672990116 | 0.14706398 | | down |
| SCEL | -1.672426625 | 0.006782774 | 0.027345901 | down |
| TULP3P1 | -1.672265729 | 0.282883878 | | down |
| RP11-304L19.4 | -1.672093411 | 0.436299288 | | down |
| RN7SL774P | -1.671940122 | 0.31734707 | | down |
| RPL23AP19 | -1.671745817 | 0.567439714 | | down |
| CPEB2-AS1 | -1.671624381 | 0.034909682 | 0.093871519 | down |
| RP11-467I20.3 | -1.671607049 | 0.539404989 | | down |
| AC009961.2 | -1.671548943 | 0.163871806 | | down |
| RP11-229P13.25 | -1.671545676 | 0.002736892 | 0.013506437 | down |
| RP11-849N15.4 | -1.671446028 | 0.150334133 | 0.269440952 | down |
| AC093381.2 | -1.671339659 | 0.56760358 | | down |
| NA | -1.671286313 | 2.22E-05 | 0.000271747 | down |
| LINC01210 | -1.671127976 | 0.321285173 | 0.460046312 | down |
| ADAP1 | -1.671035138 | 3.46E-07 | 8.15E-06 | down |
| GGT1 | -1.671028284 | 0.00183952 | 0.009819926 | down |
| NA | -1.670975576 | 0.190198122 | | down |
| RP5-996D20.3 | -1.670678136 | 0.567757522 | | down |
| RP11-409I10.2 | -1.67065302 | 0.045637899 | 0.114361978 | down |
| CHCHD2P8 | -1.67064029 | 0.56776633 | | down |
| TP53AIP1 | -1.670581874 | 0.033337957 | 0.090651243 | down |
| LINC01630 | -1.670449995 | 0.044473287 | 0.112263101 | down |
| RP11-670E13.6 | -1.670381266 | 4.59E-05 | 0.00049785 | down |
| RP11-331N16.1 | -1.67021422 | 0.024165934 | 0.071474829 | down |
| Xbac-BPG299F13.1 | -1.670157648 | 0.567878658 | | down |
| NA | -1.670046458 | 0.000359013 | 0.002688478 | down |
| LARGE-AS1 | -1.670040452 | 0.129619021 | 0.242679246 | down |
| RP11-556H2.2 | -1.670031952 | 0.113368922 | 0.22085695 | down |
| RP3-525N10.2 | -1.670008762 | 0.000172211 | 0.001477841 | down |
| RPS26P43 | -1.669922694 | 0.388070199 | | down |
| DCAF8L1 | -1.669787724 | 0.56796476 | | down |
| RP11-597D13.7 | -1.66955934 | 0.02541292 | 0.074177186 | down |
| KCNK10 | -1.669371127 | 3.37E-05 | 0.000384507 | down |
| RP11-325L7.2 | -1.66931208 | 0.48356856 | | down |

| | | | | |
|---|---|---|---|---|
| LINC01310 | -1.668883478 | 0.080137588 | 0.172455677 | down |
| CTA-929C8.6 | -1.668735403 | 0.208123984 | | down |
| NA | -1.668584464 | 0.390204123 | | down |
| RP11-379C10.1 | -1.668575858 | 0.542413613 | | down |
| TMEM30CP | -1.668535631 | 0.354678862 | | down |
| RP11-6D1.3 | -1.668465155 | 0.349838523 | | down |
| RP11-29O13.1 | -1.668326214 | 0.296037199 | 0.433519163 | down |
| RP11-886P16.6 | -1.668156189 | 0.024262599 | 0.071690158 | down |
| PGAM1P2 | -1.668155263 | 0.485001394 | | down |
| PEX5L | -1.668102311 | 0.006679071 | 0.027051226 | down |
| EBLN2 | -1.668077873 | 0.006269264 | 0.025787755 | down |
| RP11-617F23.2 | -1.667932026 | 0.044641017 | 0.112563552 | down |
| NA | -1.667809858 | 0.034976285 | | down |
| RP11-10106.2 | -1.667695074 | 0.006548405 | 0.026647742 | down |
| NA | -1.667686106 | 0.077415038 | 0.168116527 | down |
| NA | -1.667582663 | 0.006691439 | 0.027094017 | down |
| LDHC | -1.667477081 | 0.059391038 | 0.13864379 | down |
| NA | -1.667424342 | 0.568445951 | | down |
| NA | -1.667424342 | 0.568445951 | | down |
| AC053503.4 | -1.667199349 | 0.004404282 | 0.019660579 | down |
| RP11-1018N14.1 | -1.667162598 | 0.365650891 | 0.504112306 | down |
| SCARNA2 | -1.667094194 | 0.56859188 | | down |
| RN7SKP83 | -1.667046887 | 0.488555701 | | down |
| LINC01389 | -1.666819673 | 6.35E-05 | 0.000650003 | down |
| RP3-522D1.2 | -1.666586713 | 0.351227031 | | down |
| CTA-125H2.1 | -1.666566075 | 0.249723061 | 0.384299526 | down |
| NA | -1.666556243 | 5.34E-06 | 8.37E-05 | down |
| RP11-220D10.1 | -1.666274052 | 0.000119091 | 0.001096528 | down |
| CNFN | -1.666134501 | 3.91E-07 | 9.07E-06 | down |
| SUMO2P7 | -1.665992356 | 0.245408101 | 0.379330194 | down |
| FOXD4L6 | -1.665824933 | 0.149011748 | | down |
| RP11-26L20.4 | -1.665820581 | 0.568819594 | | down |
| CDX2 | -1.665668064 | 6.89E-07 | 1.49E-05 | down |
| CH17-125A10.2 | -1.665619988 | 0.568866336 | | down |
| ZSCAN10 | -1.66552726 | 0.114633414 | 0.222685005 | down |
| RP3-463P15.1 | -1.665293589 | 0.232116703 | 0.364787135 | down |
| RPL34P31 | -1.665167658 | 0.001604705 | 0.008804192 | down |
| AC011380.8 | -1.665150606 | 0.028119298 | 0.080077365 | down |
| RP11-408N14.1 | -1.664786827 | 0.288103077 | 0.425407619 | down |
| EEF1GP8 | -1.664775444 | 0.569132004 | | down |
| RP11-348J24.2 | -1.664746178 | 0.176605937 | 0.301474362 | down |
| NA | -1.664583297 | 0.008206073 | 0.031538473 | down |
| ZNF863P | -1.664441013 | 0.25540496 | 0.390537455 | down |
| ETFDH | -1.664397528 | 7.26E-14 | 1.09E-11 | down |
| PRMT8 | -1.664127119 | 0.047268084 | 0.117394588 | down |
| RPL6P5 | -1.664050346 | 0.542129057 | | down |
| NA | -1.663975116 | 0.322047275 | | down |
| ASPDH | -1.663752214 | 3.87E-05 | 0.000433073 | down |
| GPR33 | -1.663702305 | 0.349596828 | | down |
| RP11-419I17.1 | -1.663673784 | 3.48E-07 | 8.18E-06 | down |
| TRPV3 | -1.663464328 | 1.02E-06 | 2.07E-05 | down |

| | | | | |
|---|---|---|---|---|
| RP11-338N10.2 | -1.663333183 | 0.003665834 | 0.016996345 | down |
| SLC9A3R1 | -1.663201819 | 1.86E-14 | 3.24E-12 | down |
| RP11-139K1.2 | -1.663099597 | 0.00203672 | 0.010654922 | down |
| RP11-245A18.1 | -1.662909672 | 0.389488295 | | down |
| PPP1R2P6 | -1.662532459 | 0.486982142 | | down |
| LRRC74A | -1.662374481 | 0.013643881 | 0.046433705 | down |
| RN7SL107P | -1.662267494 | 0.569436927 | | down |
| RP11-229P13.15 | -1.662144728 | 0.103036435 | | down |
| NA | -1.661953448 | 0.486407096 | | down |
| CTD-2350C19.5 | -1.661852037 | 0.332824954 | 0.471158314 | down |
| C1orf106 | -1.661491407 | 1.06E-08 | 4.20E-07 | down |
| RP11-183G22.1 | -1.661460928 | 0.206148793 | | down |
| AC010971.1 | -1.661290349 | 0.569944269 | | down |
| RP11-65F13.3 | -1.661154206 | 0.569907423 | | down |
| BANF1P2 | -1.661136195 | 0.004153846 | 0.018789244 | down |
| RN7SL612P | -1.660993086 | 0.569873987 | | down |
| SHANK1 | -1.660687353 | 0.000208147 | 0.001727505 | down |
| RP11-31F19.1 | -1.660661676 | 0.040876925 | 0.105553169 | down |
| KCNK5 | -1.660400091 | 3.55E-11 | 2.76E-09 | down |
| RP11-524F11.3 | -1.660233757 | 0.109638351 | 0.215984173 | down |
| SLC12A3 | -1.659997502 | 0.000762179 | 0.004885618 | down |
| RP11-187E13.1 | -1.659893324 | 0.000284936 | 0.002235729 | down |
| IPO7P2 | -1.659892172 | 0.166299071 | | down |
| RP1-144F13.3 | -1.659891154 | 0.241452646 | | down |
| AC083822.2 | -1.659776665 | 0.539070547 | | down |
| B3GALNT1P1 | -1.659669914 | 0.115264211 | 0.223585303 | down |
| ZBTB46-AS1 | -1.65961608 | 0.57012429 | | down |
| RP11-536C5.2 | -1.659546341 | 0.001419877 | 0.007985544 | down |
| AC004070.1 | -1.65936787 | 0.570392524 | | down |
| ZNF439 | -1.659304418 | 8.74E-08 | 2.54E-06 | down |
| CTD-2555A7.2 | -1.659061952 | 0.032361272 | 0.088674694 | down |
| ACSM2A | -1.658981697 | 0.006796592 | 0.027390584 | down |
| CTC-510F12.3 | -1.658908593 | 0.095429494 | | down |
| MIR646HG | -1.65882958 | 0.000123821 | 0.001131756 | down |
| LINC01591 | -1.658673808 | 0.484610864 | | down |
| RP11-415J8.7 | -1.658582145 | 0.0503641 | 0.122959452 | down |
| IL1F10 | -1.658445309 | 0.570468387 | | down |
| RP11-1180F24.1 | -1.658415864 | 0.037344042 | 0.09866893 | down |
| SYT9 | -1.658304116 | 0.008051407 | 0.031096089 | down |
| LINC00334 | -1.658173383 | 0.000839463 | 0.005276323 | down |
| RP11-662B19.2 | -1.658161954 | 0.130901282 | 0.244485771 | down |
| ENPP7P9 | -1.65797207 | 0.488513438 | | down |
| RN7SL546P | -1.657911586 | 0.326934426 | | down |
| RP11-325O24.5 | -1.657628767 | 0.115128181 | 0.223411451 | down |
| DAP3P2 | -1.65762719 | 0.005446196 | 0.023166162 | down |
| RP11-531F16.3 | -1.657473745 | 0.038363436 | 0.100759775 | down |
| GOLGA8T | -1.657331276 | 0.216969159 | | down |
| CYP2S1 | -1.65732427 | 1.44E-08 | 5.51E-07 | down |
| RP11-384C12.1 | -1.657197973 | 0.486816532 | | down |
| SUMO2P18 | -1.657108731 | 0.333272439 | 0.47156199 | down |
| PHBP19 | -1.656941635 | 0.060749586 | 0.14102616 | down |

| | | | | |
|---|---|---|---|---|
| ACTG1P9 | -1.65690316 | 0.05613138 | 0.13306069 | down |
| AC093702.1 | -1.656729699 | 0.029545196 | 0.08292749 | down |
| PCDHGC4 | -1.656712355 | 0.102168268 | 0.205146961 | down |
| RARSP1 | -1.656602271 | 0.544922251 | | down |
| AGGF1P5 | -1.656572623 | 0.571044558 | | down |
| RP11-525K10.1 | -1.656541371 | 0.54849347 | | down |
| RP11-342M21.2 | -1.656319383 | 0.347074827 | | down |
| NDUFB4P9 | -1.65607664 | 0.54180428 | | down |
| RP11-545A16.3 | -1.656018659 | 0.036349889 | 0.096756984 | down |
| NA | -1.656001927 | 0.53895482 | | down |
| DCAF12L1 | -1.655926597 | 0.488994742 | | down |
| CCDC175 | -1.655426893 | 0.188814146 | 0.3158361 | down |
| RP11-417O11.5 | -1.655321348 | 0.012051957 | 0.042321332 | down |
| RPL31P50 | -1.655295836 | 0.571342474 | | down |
| AC092168.3 | -1.655173703 | 0.571370974 | | down |
| RPL9P5 | -1.654516753 | 0.128639357 | | down |
| RP11-94B19.1 | -1.654496598 | 0.548576951 | | down |
| RNA5SP255 | -1.654253005 | 0.570970028 | | down |
| AC092687.3 | -1.654199612 | 0.339868583 | 0.478197505 | down |
| NAALADL2 | -1.654024339 | 8.45E-09 | 3.41E-07 | down |
| AC007278.2 | -1.653936131 | 0.251914828 | | down |
| HMGN1P17 | -1.653774211 | 0.537994619 | | down |
| LEKR1 | -1.653769863 | 1.33E-05 | 0.000178933 | down |
| GGT6 | -1.653739251 | 1.27E-05 | 0.000172267 | down |
| TMEM125 | -1.653541775 | 2.82E-07 | 6.91E-06 | down |
| CTD-2224J9.8 | -1.653538669 | 0.054722484 | 0.130659008 | down |
| THAP12P6 | -1.653532754 | 0.571686126 | | down |
| SCGB2A2 | -1.653483062 | 0.197039086 | 0.325659341 | down |
| NA | -1.653019721 | 0.571805944 | | down |
| EPB41L4B | -1.652977138 | 1.54E-08 | 5.80E-07 | down |
| NA | -1.652964512 | 0.571677209 | | down |
| ACOX1 | -1.652938769 | 1.49E-21 | 1.15E-18 | down |
| FOXI1 | -1.65280385 | 0.023183793 | 0.069203566 | down |
| SEPT7P3 | -1.65271428 | 0.286076663 | | down |
| RP11-438L7.1 | -1.652667754 | 0.492660931 | | down |
| RP11-860B13.1 | -1.652621034 | 0.173714311 | | down |
| MYH14 | -1.652295377 | 5.42E-08 | 1.69E-06 | down |
| RP11-720L8.1 | -1.652209439 | 0.346225265 | 0.484896133 | down |
| RP11-20P5.2 | -1.652169246 | 0.572072226 | | down |
| RP11-165H23.1 | -1.652169246 | 0.572072226 | | down |
| RP3-473L9.4 | -1.651786407 | 0.000194772 | 0.001634103 | down |
| AL355480.4 | -1.651601008 | 0.439667636 | | down |
| PCSK4 | -1.65119603 | 2.50E-07 | 6.24E-06 | down |
| AC006076.1 | -1.651138332 | 0.325332235 | | down |
| RP11-662M24.1 | -1.651041284 | 0.572268117 | | down |
| UGT2B4 | -1.65091432 | 0.145949401 | 0.263864714 | down |
| YBX1P3 | -1.650904195 | 0.490695131 | | down |
| DUX4L34 | -1.650887745 | 0.572233294 | | down |
| RP11-673E11.2 | -1.650747419 | 0.396995848 | | down |
| OR52Z1 | -1.650634293 | 0.572430636 | | down |
| MIR6784 | -1.650585402 | 0.081111944 | 0.173954404 | down |

| | | | | |
|---|---|---|---|---|
| RP11-605P22.1 | -1.650079763 | 0.35177787 | 0.490523267 | down |
| RP11-21I4.3 | -1.64999522 | 0.439384876 | | down |
| LYG2 | -1.649970233 | 0.024692539 | 0.072628395 | down |
| RP5-875H3.2 | -1.6498269 | 0.443006444 | | down |
| SCUBE1 | -1.649755387 | 4.10E-08 | 1.32E-06 | down |
| PKMP4 | -1.649609219 | 0.278210908 | | down |
| RP11-193H5.8 | -1.649511304 | 0.57269289 | | down |
| AC113610.1 | -1.649511304 | 0.57269289 | | down |
| OR12D2 | -1.649511304 | 0.57269289 | | down |
| RPS3AP42 | -1.649511304 | 0.57269289 | | down |
| ACY3 | -1.649448193 | 1.61E-08 | 6.03E-07 | down |
| NA | -1.649135609 | 2.04E-05 | 0.000255 | down |
| NA | -1.649028821 | 0.272763456 | 0.409001846 | down |
| NA | -1.648882762 | 0.006393575 | 0.02619866 | down |
| RP11-356M20.1 | -1.648823325 | 1.04E-05 | 0.000145873 | down |
| LINC01266 | -1.648795665 | 0.003440627 | 0.016156643 | down |
| RNF113B | -1.64870514 | 0.572881187 | | down |
| ASTN1 | -1.648580796 | 0.000640018 | 0.004244525 | down |
| SBK2 | -1.648456101 | 0.322980672 | | down |
| TERF1P4 | -1.648416833 | 0.245833144 | | down |
| RNU6-234P | -1.647992388 | 0.57304769 | | down |
| RPL10P1 | -1.647937887 | 0.012820245 | 0.044303133 | down |
| NPM1P35 | -1.647913271 | 0.093613478 | 0.192889604 | down |
| AC102953.4 | -1.647902747 | 0.004011072 | 0.018286593 | down |
| SMAD1-AS2 | -1.64781419 | 0.573089321 | | down |
| FLJ26850 | -1.647797636 | 0.075958267 | 0.165753209 | down |
| CTD-2196P11.2 | -1.647779594 | 0.164675832 | | down |
| RP11-945A11.2 | -1.647704933 | 0.203696602 | 0.333102189 | down |
| NA | -1.647527165 | 0.237589669 | | down |
| PCDHA5 | -1.647507825 | 0.040696912 | 0.105250756 | down |
| SPRY4-IT1_1 | -1.647420183 | 0.354482922 | | down |
| MIR5585 | -1.647389177 | 0.57318862 | | down |
| ANGPT4 | -1.647218003 | 0.011738987 | 0.041492487 | down |
| RP11-480G7.2 | -1.646815822 | 0.073161104 | 0.161479003 | down |
| RP11-406A20.1 | -1.646675679 | 0.016214245 | 0.052955839 | down |
| RP11-10F11.2 | -1.646551484 | 0.572897311 | | down |
| RP11-88I21.1 | -1.646551461 | 0.5457738 | | down |
| CTB-25B13.9 | -1.646329067 | 0.151298222 | | down |
| RP11-474P2.2 | -1.646058888 | 0.076932055 | 0.167416099 | down |
| RP11-654C22.2 | -1.646045846 | 0.051690173 | 0.125260491 | down |
| PNKDP1 | -1.645977159 | 0.099984299 | 0.201890469 | down |
| CTD-2062F14.2 | -1.64595417 | 0.019460365 | 0.06082484 | down |
| RP11-370I10.12 | -1.645951282 | 0.000798215 | 0.005067954 | down |
| AMHR2 | -1.645917053 | 0.000809331 | 0.005126615 | down |
| HIGD2B | -1.645899282 | 0.170386215 | 0.294250931 | down |
| RP11-5N11.4 | -1.645655156 | 0.247811623 | 0.382111307 | down |
| RNU4-89P | -1.645047498 | 0.167114651 | 0.290336701 | down |
| RP11-13N12.1 | -1.644981243 | 0.102599406 | 0.205727391 | down |
| RN7SL798P | -1.644797989 | 0.226482095 | | down |
| RNU4-42P | -1.644557724 | 0.573850363 | | down |
| RP11-649G15.2 | -1.644536007 | 0.000112019 | 0.001040846 | down |

| | | | | |
|---|---|---|---|---|
| HAPLN2 | -1.644376938 | 0.000146971 | 0.001296797 | down |
| BCO1 | -1.644312013 | 1.25E-05 | 0.000170465 | down |
| NA | -1.644222483 | 0.036525996 | 0.09708812 | down |
| CYP2F2P | -1.644178746 | 0.00534336 | 0.022829339 | down |
| SIDT1-AS1 | -1.643916516 | 0.083859926 | 0.178168985 | down |
| PRSS36 | -1.643836328 | 1.31E-10 | 8.68E-09 | down |
| BRD7P1 | -1.643304237 | 0.574143432 | | down |
| RP11-253A20.1 | -1.643131534 | 0.00247448 | 0.012467429 | down |
| RP11-440I14.3 | -1.643119332 | 0.000582155 | 0.003935703 | down |
| RNU2-32P | -1.642946414 | 0.544131487 | | down |
| RNU7-40P | -1.642928872 | 0.001272579 | 0.007330075 | down |
| CTD-2297D10.2 | -1.642923367 | 0.052568147 | 0.126733864 | down |
| TEX11 | -1.642842259 | 2.12E-05 | 0.000262438 | down |
| RP11-318K12.3 | -1.642783549 | 0.161336842 | | down |
| TBATA | -1.642718964 | 0.08683886 | | down |
| ARHGDIG | -1.642465444 | 2.01E-05 | 0.000251601 | down |
| CTD-2516K3.3 | -1.642391771 | 0.574081697 | | down |
| AC068858.1 | -1.642391771 | 0.574081697 | | down |
| RP11-56L13.1 | -1.642327307 | 0.574371889 | | down |
| AC007392.4 | -1.642312881 | 0.061816866 | 0.142904404 | down |
| NFIA-AS2 | -1.642231751 | 0.000235227 | 0.001913146 | down |
| C1GALT1P3 | -1.641962678 | 0.57445717 | | down |
| SLC6A1-AS1 | -1.64192145 | 0.351415953 | | down |
| RP11-381N20.1 | -1.641795378 | 0.200641319 | 0.329726933 | down |
| SLC22A5 | -1.641745679 | 3.37E-13 | 4.20E-11 | down |
| TBX10 | -1.641355625 | 0.000144494 | 0.001280209 | down |
| CTD-2023N9.1 | -1.641354947 | 0.024471162 | 0.072164468 | down |
| RP11-693J15.3 | -1.641192687 | 0.542932085 | | down |
| DSG2-AS1 | -1.64104175 | 0.000416645 | 0.003005445 | down |
| RP11-244F12.1 | -1.640866895 | 0.116846384 | | down |
| AC011648.1 | -1.640857594 | 0.574649844 | | down |
| RP1-101G11.2 | -1.640857594 | 0.574649844 | | down |
| NA | -1.640818509 | 0.574658993 | | down |
| NEURL1-AS1 | -1.640524136 | 0.352000343 | | down |
| LINC01385 | -1.640493899 | 0.574800751 | | down |
| OR7E85P | -1.640244587 | 0.44920343 | | down |
| HMGN1P36 | -1.640182982 | 0.0073738 | 0.02910002 | down |
| NA | -1.64014067 | 0.032130847 | 0.088223283 | down |
| RP11-117D22.2 | -1.640087255 | 0.002911025 | 0.014158788 | down |
| Y_RNA | -1.639603858 | 0.358252492 | 0.496697871 | down |
| RP11-866E20.3 | -1.639468894 | 0.207641614 | 0.337802574 | down |
| LINC-PINT | -1.6394143 | 6.47E-06 | 9.86E-05 | down |
| RP13-463N16.6 | -1.639268293 | 0.009045231 | 0.033964428 | down |
| SPDYE21P | -1.639096013 | 5.90E-05 | 0.000612057 | down |
| WIPF2 | -1.63883015 | 1.12E-11 | 9.69E-10 | down |
| AP001476.2 | -1.638653261 | 0.001828759 | 0.009772898 | down |
| NLGN4Y-AS1 | -1.638611325 | 0.232092999 | 0.364787135 | down |
| PLA2G1B | -1.638566208 | 0.001227539 | 0.007136176 | down |
| LTK | -1.638284092 | 3.10E-10 | 1.83E-08 | down |
| CYP4F32P | -1.638233803 | 0.575329628 | | down |
| KRT8P37 | -1.63821685 | 0.006291033 | 0.025848987 | down |

| | | | | |
|---|---|---|---|---|
| RP11-148B18.1 | -1.638088365 | 0.075301177 | 0.16482303 | down |
| NRBF2P2 | -1.638074309 | 0.244926276 | | down |
| RP11-823P9.3 | -1.63785076 | 0.089176132 | 0.186366703 | down |
| RPL19P18 | -1.637640575 | 0.575194408 | | down |
| RP11-219B4.7 | -1.637588776 | 0.07867266 | 0.170135121 | down |
| CYP2AB1P | -1.637415622 | 0.286782793 | 0.423922592 | down |
| CTCFL | -1.637368633 | 0.015779361 | 0.051896566 | down |
| RP11-1080G15.2 | -1.637135932 | 0.105953202 | 0.210658327 | down |
| LINC01217 | -1.637030652 | 0.545810629 | | down |
| TOMM22P4 | -1.636914867 | 0.395740106 | | down |
| RP1-207H1.3 | -1.636828426 | 0.043370202 | 0.110396877 | down |
| OR7E62P | -1.63678012 | 0.4955121 | | down |
| RP11-345F18.1 | -1.636772777 | 0.152630621 | | down |
| PUDPP3 | -1.636526785 | 0.575729232 | | down |
| RP3-495K2.4 | -1.636447331 | 0.079162132 | 0.170884802 | down |
| RBBP4P5 | -1.636428789 | 0.445714073 | | down |
| AC009963.5 | -1.636307788 | 0.315940514 | | down |
| RN7SL170P | -1.636166467 | 0.307759369 | 0.446121899 | down |
| LINC01216 | -1.636131054 | 0.57582189 | | down |
| RP6-91H8.5 | -1.635954343 | 0.14224869 | 0.259123242 | down |
| KB-1552D7.2 | -1.635881847 | 0.131783607 | 0.245675521 | down |
| RP11-816B4.1 | -1.635165296 | 0.550060657 | | down |
| CTD-2515C13.2 | -1.635013535 | 0.491824605 | | down |
| RNU7-18P | -1.635009191 | 0.227155571 | | down |
| RP11-418J17.3 | -1.635007025 | 0.043486373 | 0.11058016 | down |
| DNAJB8-AS1 | -1.634886161 | 0.575978557 | | down |
| RP11-326L9.1 | -1.634886161 | 0.575978557 | | down |
| RP11-451L19.1 | -1.634886161 | 0.575978557 | | down |
| PYCARD-AS1 | -1.63479989 | 0.150042305 | | down |
| RP11-544M22.3 | -1.634733263 | 0.1170159 | | down |
| AC005351.1 | -1.634630362 | 0.576173328 | | down |
| CH17-360D5.2 | -1.634608105 | 0.015909932 | 0.052211688 | down |
| SNORA62 | -1.634590445 | 0.165574687 | 0.288413601 | down |
| MTND6P4 | -1.6342213 | 0.000135215 | 0.001213804 | down |
| RP11-118E18.4 | -1.634140457 | 0.158072971 | 0.278967596 | down |
| TMEM51-AS1 | -1.634086136 | 1.16E-05 | 0.000159403 | down |
| CTD-2302M15.1 | -1.633997436 | 0.330910541 | | down |
| SLC24A2 | -1.633964034 | 0.037730113 | 0.099461764 | down |
| RP11-347C12.12 | -1.633907855 | 0.000946517 | 0.005796407 | down |
| AC010987.5 | -1.633813371 | 0.161637017 | 0.283409544 | down |
| RP11-24M17.4 | -1.633801492 | 0.015184651 | 0.05039298 | down |
| FAM131C | -1.633735496 | 0.000482641 | 0.003378488 | down |
| RP5-1092A3.5 | -1.633650005 | 0.013471633 | 0.045977707 | down |
| RP11-71E19.1 | -1.633561849 | 0.045814897 | 0.114667009 | down |
| NA | -1.633438302 | 0.281210146 | 0.417589839 | down |
| LINC00629 | -1.633265651 | 0.141137348 | 0.257771511 | down |
| RP11-103C16.2 | -1.633008431 | 0.145501941 | | down |
| DSG1 | -1.63269453 | 0.160797091 | | down |
| SIX1 | -1.632674071 | 0.012975573 | 0.044680587 | down |
| RP11-267L5.1 | -1.632582815 | 0.556105127 | | down |
| NA | -1.632475961 | 0.034486351 | 0.093116558 | down |

| | | | | |
|---|---|---|---|---|
| RP11-100F15.1 | -1.63223287 | 0.576734989 | | down |
| RN7SL819P | -1.63223287 | 0.576734989 | | down |
| EIF4A3P1 | -1.63223287 | 0.576734989 | | down |
| CTB-186H2.2 | -1.632217551 | 0.266017813 | 0.401700803 | down |
| RP11-707P17.1 | -1.632098192 | 0.406019873 | | down |
| CTA-941F9.10 | -1.631711412 | 8.15E-05 | 0.000798965 | down |
| RP1-168P16.1 | -1.631643426 | 0.349795307 | | down |
| SDSL | -1.63147806 | 9.09E-08 | 2.62E-06 | down |
| PHF2P2 | -1.631418199 | 0.197055016 | 0.325659341 | down |
| CH17-78J1.1 | -1.631324336 | 0.013791565 | 0.046809059 | down |
| ZNF114 | -1.631250925 | 0.000554902 | 0.003789832 | down |
| MTND1P32 | -1.631113925 | 0.576997212 | | down |
| RP11-350D17.3 | -1.630650027 | 0.499642477 | | down |
| RP11-81K13.1 | -1.630355193 | 0.577175052 | | down |
| NRG4 | -1.6295545 | 4.05E-06 | 6.68E-05 | down |
| LINC00637 | -1.629020167 | 0.02624765 | 0.075990925 | down |
| OSGIN1 | -1.628998915 | 3.47E-08 | 1.14E-06 | down |
| RP11-78J21.4 | -1.628880767 | 0.021955886 | 0.066505711 | down |
| AC094019.4 | -1.62878746 | 0.203202451 | | down |
| DEFB132 | -1.628651154 | 0.360142452 | | down |
| IL17RE | -1.62849195 | 1.23E-07 | 3.38E-06 | down |
| SOSTDC1 | -1.6283866 | 0.001759891 | 0.009477356 | down |
| RP5-1100H13.3 | -1.628311971 | 0.57765409 | | down |
| SLC38A4 | -1.628159088 | 0.001500694 | 0.008340148 | down |
| RP11-329B9.5 | -1.628141725 | 0.081386923 | 0.174392032 | down |
| RN7SL477P | -1.627973638 | 0.224985058 | | down |
| RPSAP51 | -1.627951294 | 0.499994902 | | down |
| RP1-170O19.17 | -1.627949224 | 0.192353567 | | down |
| NA | -1.627883059 | 0.016499634 | 0.053619795 | down |
| NBPF4 | -1.62770996 | 0.027534127 | 0.078791819 | down |
| NA | -1.627390933 | 0.577736704 | | down |
| RPL7P10 | -1.627366702 | 0.062038082 | | down |
| RP11-158I3.1 | -1.627344158 | 0.260929821 | | down |
| AP000697.6 | -1.62728373 | 0.57783117 | | down |
| RP11-483F11.7 | -1.627274324 | 0.555053811 | | down |
| CA3 | -1.627242181 | 1.96E-05 | 0.000246055 | down |
| EGLN3-AS1 | -1.627207386 | 0.399274544 | | down |
| CTC-548K16.1 | -1.627143894 | 0.000959644 | 0.00586126 | down |
| RP11-264J4.8 | -1.626947692 | 0.090603147 | 0.188405382 | down |
| RP11-856M7.1 | -1.626895465 | 0.577714383 | | down |
| ATF1P1 | -1.626694005 | 0.578033558 | | down |
| RP11-497D6.3 | -1.626479106 | 0.086607734 | 0.182509839 | down |
| AC063976.3 | -1.626389173 | 0.578105064 | | down |
| YJEFN3 | -1.626376324 | 1.06E-05 | 0.00014828 | down |
| RPL18AP7 | -1.62636307 | 0.014465841 | 0.048559969 | down |
| NA | -1.626347468 | 0.541034431 | | down |
| RP11-841C19.4 | -1.626291876 | 0.578063928 | | down |
| PFKFB4 | -1.626015605 | 2.09E-07 | 5.35E-06 | down |
| IGLON5 | -1.625935852 | 0.000130637 | 0.001179706 | down |
| RP11-352M15.1 | -1.625440861 | 0.451611772 | | down |
| RP3-399L15.2 | -1.625405736 | 0.578335783 | | down |

| | | | | |
|---|---|---|---|---|
| AC010524.4 | -1.625400209 | 0.203943892 | 0.333403542 | down |
| GPR101 | -1.625110707 | 0.577924291 | | down |
| RNU1-55P | -1.625088536 | 0.578410209 | | down |
| MKRN2OS | -1.625065691 | 1.85E-08 | 6.65E-07 | down |
| AC015849.19 | -1.625046598 | 0.000328482 | 0.002509897 | down |
| RNF128 | -1.624977127 | 8.48E-05 | 0.000824046 | down |
| RP11-95G17.2 | -1.624865232 | 0.019808589 | 0.061613962 | down |
| BOLA3P3 | -1.624625381 | 0.005324233 | 0.022773526 | down |
| Metazoa_SRP | -1.6244721 | 0.548905634 | | down |
| RP11-536G4.2 | -1.624109758 | 0.451529238 | | down |
| RP4-562J12.1 | -1.624089146 | 0.578304096 | | down |
| RP11-51L5.3 | -1.624061856 | 0.409951746 | | down |
| RP11-84C10.1 | -1.62369587 | 0.042899491 | 0.109483486 | down |
| IFNL3P1 | -1.623578947 | 0.100486996 | 0.202646751 | down |
| GMPSP1 | -1.623249464 | 0.0059153 | 0.024683234 | down |
| TRAV33 | -1.623059442 | 0.548675062 | | down |
| HNRNPA1P9 | -1.623049509 | 0.254944138 | | down |
| RP11-136B18.2 | -1.622800764 | 0.237902903 | 0.371205132 | down |
| CLDN15 | -1.62277943 | 4.30E-10 | 2.47E-08 | down |
| RP11-288A5.2 | -1.622616638 | 0.030543439 | 0.084912286 | down |
| EPS8L2 | -1.622511019 | 9.57E-11 | 6.55E-09 | down |
| NA | -1.622372383 | 0.040592573 | | down |
| NA | -1.622367915 | 0.000137775 | 0.001233406 | down |
| PTK6 | -1.622253862 | 4.06E-08 | 1.31E-06 | down |
| OIT3 | -1.622188798 | 0.007228968 | 0.028648874 | down |
| NA | -1.622106803 | 0.005748176 | 0.024136717 | down |
| ADI1P1 | -1.621869077 | 0.579165854 | | down |
| CRAT37 | -1.62165472 | 0.104275014 | 0.207982375 | down |
| DNER | -1.621260837 | 0.000152115 | 0.001335131 | down |
| AC022816.2 | -1.621210718 | 0.121524991 | 0.232169455 | down |
| CTC-425023.2 | -1.621097747 | 0.019853387 | 0.061702198 | down |
| AC068137.13 | -1.621037992 | 0.04436008 | | down |
| CYP2C8 | -1.621029225 | 0.001565286 | 0.008627306 | down |
| TBC1D28 | -1.62082786 | 0.579278114 | | down |
| CTAGE3P | -1.620706153 | 0.226446623 | | down |
| RP11-470L19.6 | -1.620671056 | 0.579176496 | | down |
| AC109815.2 | -1.620671056 | 0.579176496 | | down |
| RP11-344N17.9 | -1.620633751 | 0.388712225 | 0.52634172 | down |
| OR2M4 | -1.620471485 | 0.448096733 | | down |
| SNORA31 | -1.6204413 | 0.369206491 | 0.50757023 | down |
| MTCO3P45 | -1.620438349 | 0.579438461 | | down |
| CTD-2653B5.1 | -1.620255136 | 0.555398097 | | down |
| RP3-357I16.1 | -1.620238245 | 0.579485477 | | down |
| CST9L | -1.620185867 | 0.579561106 | | down |
| RP11-66B24.7 | -1.620139901 | 0.000644647 | 0.004266967 | down |
| DCX | -1.620087461 | 0.002538398 | 0.012719083 | down |
| RP11-438P9.2 | -1.620077002 | 0.352516112 | | down |
| RP11-279O17.2 | -1.619559759 | 0.579708161 | | down |
| RP11-789C1.2 | -1.619550247 | 0.17219294 | 0.296484881 | down |
| ZNF536 | -1.619305739 | 2.00E-06 | 3.70E-05 | down |
| BPIFB3 | -1.619173604 | 0.579798867 | | down |

| | | | | |
|---|---|---|---|---|
| RP11-308N19.3 | -1.618933051 | 0.579792182 | | down |
| KRT18P10 | -1.618750785 | 0.00135412 | 0.007696402 | down |
| AC104772.1 | -1.61868539 | 0.579913554 | | down |
| CTC-537E7.1 | -1.61868539 | 0.579913554 | | down |
| CTB-88F18.2 | -1.61868539 | 0.579913554 | | down |
| SERPINB8P1 | -1.61868539 | 0.579913554 | | down |
| KRTAP1-4 | -1.61868539 | 0.579913554 | | down |
| MIR526A1 | -1.61868539 | 0.579913554 | | down |
| ELAVL3 | -1.618573155 | 0.002026886 | 0.010612718 | down |
| MME-AS1 | -1.618255747 | 0.357056934 | | down |
| BOD1P1 | -1.618206451 | 0.580026074 | | down |
| RP11-699L21.2 | -1.618161021 | 0.169698201 | | down |
| AC005498.4 | -1.617743505 | 0.407871792 | | down |
| CTD-2243E23.1 | -1.617699236 | 0.580145247 | | down |
| NUPR2 | -1.617393211 | 0.005639225 | 0.023773565 | down |
| OR8A1 | -1.617200084 | 0.132973973 | 0.247112639 | down |
| RP11-15B24.3 | -1.617017822 | 0.580305367 | | down |
| RP11-457D20.2 | -1.617017822 | 0.580305367 | | down |
| PPP1R2P9 | -1.616945672 | 0.155485689 | 0.275599531 | down |
| RNF19BPX | -1.616907991 | 0.580331177 | | down |
| RP1-274L7.4 | -1.616631246 | 0.156904103 | 0.277540587 | down |
| CTD-2296D1.1 | -1.616542804 | 0.580285534 | | down |
| KRT86 | -1.616472424 | 3.21E-05 | 0.000369855 | down |
| CTD-2249K22.1 | -1.616422571 | 0.259580614 | 0.394807517 | down |
| RP11-310E22.5 | -1.616312874 | 0.067684285 | 0.152493937 | down |
| RP1-80N2.2 | -1.616265169 | 8.31E-06 | 0.000121131 | down |
| NA | -1.615886157 | 0.452156274 | | down |
| TTLL10 | -1.61585119 | 0.000305763 | 0.002367008 | down |
| RP11-179A10.2 | -1.61583322 | 0.215503601 | 0.34660547 | down |
| PABPC1P6 | -1.615688658 | 0.580617756 | | down |
| KANK4 | -1.615541282 | 1.42E-05 | 0.000189062 | down |
| RP5-903G2.2 | -1.6155101 | 2.10E-05 | 0.00026116 | down |
| AC004951.5 | -1.615061983 | 1.53E-05 | 0.000201404 | down |
| RP11-46A10.6 | -1.614481955 | 0.176812199 | | down |
| RP1-153P14.8 | -1.614447809 | 0.024539024 | 0.072294116 | down |
| NA | -1.614428785 | 3.84E-06 | 6.36E-05 | down |
| RPEL1 | -1.614258984 | 0.208557505 | | down |
| GIPC2 | -1.614132427 | 8.29E-11 | 5.76E-09 | down |
| RP11-314B1.2 | -1.614082422 | 0.056758053 | 0.134214322 | down |
| VRTN | -1.61406796 | 0.001276371 | 0.007346225 | down |
| RP4-791K14.2 | -1.613960748 | 0.100937569 | 0.203350655 | down |
| GALR1 | -1.613942027 | 0.014730388 | 0.049255401 | down |
| TRBV6-8 | -1.613881456 | 0.160940942 | | down |
| ZNF503-AS1 | -1.613728189 | 5.02E-06 | 7.96E-05 | down |
| ASIC5 | -1.613658544 | 0.404178174 | | down |
| RP11-263F14.3 | -1.613653489 | 0.581033586 | | down |
| RP5-1033E15.3 | -1.613653489 | 0.581033586 | | down |
| DYX1C1 | -1.613248597 | 0.005524956 | 0.023405496 | down |
| KLRG2 | -1.613183732 | 0.132144253 | 0.246118773 | down |
| CTB-167B5.1 | -1.613079474 | 0.035690886 | 0.095374631 | down |
| SLC6A7 | -1.612995527 | 2.21E-06 | 4.04E-05 | down |

| | | | | |
|---|---|---|---|---|
| RPA4 | -1.612951016 | 0.009789069 | 0.036033177 | down |
| RP11-287F9.2 | -1.612902223 | 0.082180171 | 0.175749043 | down |
| VN1R53P | -1.612778784 | 0.410498832 | | down |
| GEMIN2P1 | -1.612511885 | 0.581095682 | | down |
| RP5-1065P14.2 | -1.612105929 | 0.04167406 | 0.107187352 | down |
| MIATNB | -1.612038375 | 7.97E-06 | 0.000116831 | down |
| RP11-483P21.6 | -1.61194521 | 0.12592289 | | down |
| Y_RNA | -1.611776459 | 0.27226472 | 0.408526257 | down |
| NFE2L3P1 | -1.61173058 | 0.581486032 | | down |
| CTC-422A18.1 | -1.611463367 | 0.581069063 | | down |
| MT-CO1 | -1.611456232 | 0.000600518 | 0.004034425 | down |
| INTS6L-AS1 | -1.611382262 | 0.083090739 | 0.177116973 | down |
| RP11-260A9.1 | -1.611261919 | 0.58165872 | | down |
| KRT18P65 | -1.611200529 | 0.106311956 | 0.211120127 | down |
| MIR553 | -1.61100697 | 0.408467251 | | down |
| CTD-2331H12.7 | -1.610881365 | 0.006494785 | 0.026476164 | down |
| RP11-5P18.10 | -1.610872898 | 0.557897295 | | down |
| DPP3P2 | -1.610731256 | 0.581783565 | | down |
| KRT18P54 | -1.610630423 | 0.581399848 | | down |
| NA | -1.610598529 | 0.011440683 | 0.040677381 | down |
| RP11-277A4.4 | -1.610566272 | 0.013860633 | 0.04702754 | down |
| LINC01079 | -1.610037368 | 0.096866992 | 0.197567631 | down |
| NA | -1.609991466 | 0.581957631 | | down |
| RP11-685B13.2 | -1.609991466 | 0.581957631 | | down |
| FSD2 | -1.609965013 | 0.054998246 | 0.131098644 | down |
| PLA2G2E | -1.609845782 | 0.272371225 | | down |
| RP11-756P10.2 | -1.609542204 | 0.043681199 | 0.110917235 | down |
| RNU6-918P | -1.6094609 | 0.36539127 | | down |
| CLDN23 | -1.609458076 | 4.12E-06 | 6.79E-05 | down |
| NA | -1.609449688 | 0.019223858 | 0.060300856 | down |
| RP11-68D16.1 | -1.609105508 | 0.498846125 | | down |
| CLDN7 | -1.609036944 | 3.56E-05 | 0.00040367 | down |
| RP11-1114I9.1 | -1.608924702 | 0.327527575 | | down |
| RP11-588F10.1 | -1.608833302 | 0.392634624 | 0.53007615 | down |
| AC005480.1 | -1.608675398 | 0.37382565 | | down |
| NTRK3-AS1 | -1.608552359 | 0.295380076 | | down |
| RP11-369G6.2 | -1.608288165 | 0.582296413 | | down |
| CTAGE9 | -1.608156615 | 0.023039346 | 0.068847635 | down |
| RP5-994D16.11 | -1.607898302 | 0.00045063 | 0.003202162 | down |
| RP13-192B19.2 | -1.607843553 | 0.559619273 | | down |
| RP11-326K13.5 | -1.607799545 | 0.015483637 | 0.051187322 | down |
| RP11-647P12.2 | -1.607209081 | 0.582482704 | | down |
| RNU1-47P | -1.607191541 | 0.290405906 | 0.42756812 | down |
| AC068831.6 | -1.607111882 | 0.001858669 | 0.009905118 | down |
| RP11-749I16.3 | -1.606933716 | 0.004331535 | 0.019394362 | down |
| AC005019.3 | -1.606912108 | 0.55613884 | | down |
| HOGA1 | -1.606708139 | 0.016411333 | 0.053410517 | down |
| NA | -1.606663252 | 0.209578745 | | down |
| RP11-681L4.2 | -1.606612976 | 0.196559003 | 0.325080849 | down |
| RP11-145H9.3 | -1.606582992 | 0.411209348 | | down |
| AC007365.1 | -1.606573052 | 0.3070868 | | down |

| | | | | |
|---|---|---|---|---|
| RP11-503C24.1 | -1.60628528 | 0.582768153 | | down |
| MAP7D2 | -1.6062477 | 5.13E-06 | 8.10E-05 | down |
| RP11-46A10.2 | -1.606086745 | 0.411483626 | | down |
| NA | -1.60601521 | 0.001114594 | 0.006617958 | down |
| RP11-386M24.9 | -1.605814463 | 0.068912528 | | down |
| RP11-455F5.4 | -1.605802931 | 0.032341593 | 0.088650023 | down |
| RP11-337N6.1 | -1.60531023 | 0.236067884 | | down |
| AC231657.1 | -1.605302102 | 0.504137788 | | down |
| ZNF728 | -1.605105158 | 0.120227778 | 0.230467897 | down |
| RP11-493L12.5 | -1.604772694 | 0.00719705 | 0.028571376 | down |
| TNR | -1.604553328 | 0.000664242 | 0.00437093 | down |
| NBPF6 | -1.604476549 | 0.06476702 | 0.147611084 | down |
| RPS17P2 | -1.604355377 | 0.030250931 | | down |
| RP11-802F5.1 | -1.604302206 | 0.341813838 | 0.480172766 | down |
| CTA-972D3.2 | -1.604108312 | 0.370548942 | | down |
| SPDYE2B | -1.604071788 | 0.032621973 | 0.089213049 | down |
| RP11-69M1.8 | -1.604035851 | 0.501892921 | | down |
| CLCA1 | -1.603823871 | 0.001625868 | 0.00890078 | down |
| NA | -1.603793433 | 0.000347179 | 0.002622745 | down |
| PAX3 | -1.603750878 | 0.315948412 | 0.454549351 | down |
| RP13-93L13.2 | -1.603471004 | 0.021116244 | 0.06464327 | down |
| RP11-426L16.3 | -1.603460953 | 0.023143163 | 0.069109751 | down |
| RP11-307P5.1 | -1.603458183 | 0.583297288 | | down |
| RBPMSLP | -1.603140757 | 0.36932501 | | down |
| C10orf62 | -1.603063704 | 0.583588696 | | down |
| RP11-334N17.1 | -1.602975804 | 0.583609404 | | down |
| RP11-178L8.7 | -1.602960635 | 2.45E-05 | 0.000296067 | down |
| AC096669.2 | -1.602894532 | 0.559305845 | | down |
| RP11-941H19.2 | -1.602862748 | 0.079862916 | | down |
| RP5-1022P6.5 | -1.602757827 | 0.408883451 | | down |
| RPS4XP22 | -1.602663094 | 0.045589767 | 0.1142749 | down |
| CERS6-AS1 | -1.602192271 | 0.085619587 | 0.181062496 | down |
| SUCLG2P2 | -1.602070333 | 0.011242804 | 0.04010196 | down |
| CTD-2347I17.1 | -1.601880634 | 0.315196113 | 0.453879985 | down |
| BRWD1P3 | -1.601739275 | 0.337355946 | | down |
| CTB-70D19.1 | -1.601720496 | 0.583707125 | | down |
| ZNF735 | -1.601694357 | 0.454724576 | | down |
| CTBP2P6 | -1.601647559 | 0.583861153 | | down |
| Y_RNA | -1.601414091 | 0.454448781 | | down |
| GRIK1-AS1 | -1.601327626 | 0.003401845 | 0.01599453 | down |
| OR4D1 | -1.601192521 | 0.011290854 | 0.040249456 | down |
| NA | -1.601077768 | 0.420507812 | | down |
| AC007556.3 | -1.601047019 | 0.111703096 | 0.218851528 | down |
| RP11-16604.6 | -1.600904914 | 0.00031276 | 0.002413084 | down |
| AC074363.1 | -1.600754821 | 0.062727707 | 0.144421252 | down |
| EPHA5-AS1 | -1.600565675 | 0.325683232 | | down |
| RP11-651J20.1 | -1.600477369 | 0.584198135 | | down |
| DNAJC27-AS1 | -1.600453339 | 2.06E-07 | 5.29E-06 | down |
| MTCYBP44 | -1.600435865 | 0.584207917 | | down |
| CYLC2 | -1.600415735 | 0.247793478 | 0.382111307 | down |
| GC | -1.60040453 | 0.457353916 | | down |

| | | | | |
|---|---|---|---|---|
| ATP5HP2 | -1.600296317 | 0.084472591 | | down |
| MTCO3P38 | -1.600295245 | 0.584241061 | | down |
| AC093698.4 | -1.600292606 | 0.560007278 | | down |
| PNLIPP1 | -1.600067657 | 0.584294705 | | down |
| RNU2-70P | -1.600053524 | 0.584237046 | | down |
| PACRG-AS1 | -1.599800214 | 0.141477942 | 0.258217171 | down |
| WEE2-AS1 | -1.599776768 | 1.83E-05 | 0.000233249 | down |
| AC091729.7 | -1.599765697 | 0.184608443 | | down |
| NA | -1.599690905 | 0.372327278 | | down |
| LINC00421 | -1.599384802 | 0.248896551 | | down |
| BICDL2 | -1.599184565 | 1.08E-05 | 0.00014983 | down |
| AC145343.2 | -1.599038449 | 0.000280077 | 0.002207406 | down |
| FLJ12825 | -1.599033509 | 0.00182475 | 0.009753211 | down |
| C4orf22 | -1.598832065 | 0.076280746 | 0.166337451 | down |
| AC007405.8 | -1.598704844 | 0.584487946 | | down |
| NA | -1.59862298 | 0.170968799 | 0.294867198 | down |
| AQP7P1 | -1.598606775 | 0.011251287 | 0.040127453 | down |
| RP11-567C20.2 | -1.598509476 | 0.584464799 | | down |
| CTD-2001J20.1 | -1.598340202 | 0.03514517 | 0.094352739 | down |
| MIR215 | -1.598281817 | 0.076900669 | 0.167372043 | down |
| PADI2 | -1.59793052 | 1.97E-06 | 3.66E-05 | down |
| RP11-1275H24.1 | -1.597899043 | 3.05E-06 | 5.26E-05 | down |
| KRT26 | -1.59785028 | 0.559936165 | | down |
| L22NC03-23C6.1 | -1.597667607 | 0.460619155 | | down |
| NA | -1.597489161 | 0.001929569 | 0.010186597 | down |
| RP11-756A22.7 | -1.597261357 | 0.001113655 | 0.006614995 | down |
| SUGT1P2 | -1.597242212 | 0.250701276 | | down |
| RRN3P3 | -1.597209696 | 1.07E-06 | 2.15E-05 | down |
| RP11-340A13.1 | -1.59678635 | 0.550595869 | | down |
| OR5A1 | -1.596478862 | 0.244809115 | 0.378770734 | down |
| AC004449.6 | -1.596237162 | 0.037562026 | 0.099105429 | down |
| RP11-137O10.1 | -1.596208144 | 0.332028398 | 0.470407488 | down |
| RP11-270B14.1 | -1.596047184 | 0.585046108 | | down |
| RP11-181B11.1 | -1.595873456 | 0.506431893 | | down |
| RP11-982M15.8 | -1.595869717 | 0.008285422 | 0.031775239 | down |
| LINC01134 | -1.595837685 | 4.24E-09 | 1.84E-07 | down |
| RP11-517I3.1 | -1.595519217 | 9.65E-05 | 0.000917879 | down |
| AC007327.5 | -1.595361836 | 0.585404411 | | down |
| CTB-16J4.1 | -1.595238848 | 0.053827549 | 0.129004058 | down |
| NA | -1.595193836 | 0.585444045 | | down |
| STPG2-AS1 | -1.595171534 | 0.508348815 | | down |
| RPL37AP8 | -1.595107817 | 0.056065101 | 0.132966698 | down |
| CTD-2034I4.2 | -1.595083438 | 0.026242852 | 0.07598747 | down |
| SLC1A2 | -1.594818352 | 0.000304786 | 0.002361264 | down |
| RP11-268I9.1 | -1.594640451 | 0.324593154 | 0.463056723 | down |
| GRIA1 | -1.594605929 | 0.004598584 | 0.02033557 | down |
| CTB-140J7.2 | -1.594552398 | 0.240458507 | | down |
| LINC01592 | -1.594426481 | 0.4123814 | | down |
| RP11-463O12.5 | -1.594083708 | 4.94E-07 | 1.11E-05 | down |
| RP1-168L15.5 | -1.593962657 | 0.000706809 | 0.00459939 | down |
| uc_338 | -1.593749414 | 0.585657863 | | down |

| | | | | |
|---|---|---|---|---|
| LINC01456 | -1.593712135 | 0.585597623 | | down |
| IGF2BP2-AS1 | -1.593637116 | 0.040177705 | 0.104275999 | down |
| NA | -1.593548098 | 0.585772261 | | down |
| FLJ42102 | -1.593464784 | 0.362337488 | | down |
| SEMA6D | -1.59330767 | 4.74E-08 | 1.50E-06 | down |
| AC090587.5 | -1.593152753 | 0.089265239 | | down |
| LINC01254 | -1.59309949 | 0.585878196 | | down |
| NA | -1.592924236 | 0.505601255 | | down |
| RP11-736N17.9 | -1.592614892 | 0.074001573 | 0.162717182 | down |
| RP11-864I4.4 | -1.592441535 | 0.000337082 | 0.002562671 | down |
| RP4-801G22.3 | -1.592298614 | 0.412416265 | | down |
| FGF6 | -1.592051873 | 0.55775793 | | down |
| AC079305.11 | -1.592004242 | 0.057850322 | 0.13613099 | down |
| RP11-379B8.1 | -1.591993617 | 0.000595514 | 0.004008605 | down |
| RP11-293K19.1 | -1.591986024 | 0.586201075 | | down |
| VPS35P1 | -1.59188522 | 0.561983071 | | down |
| SNORA74 | -1.591654415 | 0.586279359 | | down |
| NIPA2P2 | -1.591654415 | 0.586279359 | | down |
| PIGCP2 | -1.591654415 | 0.586279359 | | down |
| RP11-279L11.1 | -1.591654415 | 0.586279359 | | down |
| MIR1185-2 | -1.591654415 | 0.586279359 | | down |
| RP11-43P5.3 | -1.591654415 | 0.586279359 | | down |
| RPS10P27 | -1.591654415 | 0.586279359 | | down |
| bac-BPGBPG24018 | -1.591631721 | 0.586284717 | | down |
| AP000358.5 | -1.591443399 | 0.559649779 | | down |
| HDHD3 | -1.590863285 | 3.38E-09 | 1.52E-07 | down |
| GNG12-AS1 | -1.590740481 | 0.001378066 | 0.007801511 | down |
| RP4-800O15.2 | -1.590710461 | 0.562735908 | | down |
| OR1D3P | -1.590566393 | 0.58647653 | | down |
| RP5-837I24.6 | -1.590264314 | 0.508381353 | | down |
| RP11-111E14.2 | -1.590214923 | 0.408892097 | | down |
| RPL23AP67 | -1.590177373 | 0.170577343 | | down |
| RP11-245C17.2 | -1.590152516 | 0.031196059 | 0.086315024 | down |
| RP11-551L14.7 | -1.590102611 | 0.236284163 | | down |
| OR3A2 | -1.589979598 | 0.059539402 | 0.138903696 | down |
| NA | -1.589910967 | 0.50987002 | | down |
| AC159540.14 | -1.589703611 | 0.586613824 | | down |
| CTD-2382H12.2 | -1.589613482 | 0.508990516 | 0.635212722 | down |
| RP4-625H18.2 | -1.589436379 | 0.000532632 | 0.003663551 | down |
| AC018735.1 | -1.589187497 | 0.586802353 | | down |
| NA | -1.589065826 | 0.168319911 | | down |
| SLC7A4 | -1.588816584 | 0.000244769 | 0.001976841 | down |
| MSL3P1 | -1.588802128 | 0.12562513 | | down |
| RP11-336K24.5 | -1.588363375 | 0.190611291 | 0.318111551 | down |
| FAM66E | -1.588328071 | 0.417647318 | | down |
| CDK15 | -1.587883413 | 7.69E-05 | 0.000759655 | down |
| RP11-461A8.5 | -1.58767489 | 0.025452648 | 0.074250787 | down |
| RP3-395M20.8 | -1.587543902 | 5.67E-09 | 2.38E-07 | down |
| CTD-260009.1 | -1.587458459 | 0.20881189 | 0.339018158 | down |
| TRBV5-3 | -1.587334386 | 0.375421692 | | down |
| RP11-809H16.5 | -1.587296968 | 0.587308448 | | down |

| | | | | |
|---|---|---|---|---|
| LINC00866 | -1.587195604 | 0.069324746 | 0.15524692 | down |
| OR10D1P | -1.586801757 | 0.587366221 | | down |
| RPL30P5 | -1.586775431 | 0.551789717 | | down |
| ODF3L2 | -1.586572845 | 0.000832055 | 0.005240579 | down |
| AC009950.2 | -1.586554389 | 5.24E-05 | 0.000555769 | down |
| WNT8B | -1.58654216 | 0.173912753 | 0.298403401 | down |
| NA | -1.586506034 | 0.10759886 | 0.213084183 | down |
| FOSL1P1 | -1.58647978 | 0.174283562 | 0.298766139 | down |
| LA16c-312E8.2 | -1.586365746 | 0.335817097 | | down |
| RP1-296G17.3 | -1.58634597 | 0.350340659 | | down |
| CTD-2583P5.3 | -1.586146726 | 0.001690407 | 0.009175534 | down |
| RPL12P29 | -1.586133036 | 0.509745472 | | down |
| VN1R9P | -1.585840417 | 0.510354186 | | down |
| TMEM266 | -1.585786182 | 2.45E-07 | 6.17E-06 | down |
| AF196970.3 | -1.585766903 | 0.225266831 | | down |
| CEBPA-AS1 | -1.585524226 | 1.28E-10 | 8.52E-09 | down |
| AC073636.1 | -1.585155434 | 0.17677337 | | down |
| RP11-620J15.1 | -1.585080556 | 0.033430502 | 0.090812477 | down |
| RP11-144I2.1 | -1.585071038 | 0.209089249 | 0.339285844 | down |
| RNU1-136P | -1.585039695 | 0.276553818 | 0.412807444 | down |
| RP11-398B16.2 | -1.585000312 | 0.587726027 | | down |
| RP1-63G5.7 | -1.584824266 | 0.427201937 | | down |
| HHIP | -1.584791204 | 5.33E-05 | 0.000561766 | down |
| RNU6-720P | -1.58478916 | 0.371652174 | | down |
| RP11-533E19.2 | -1.584769598 | 0.587905512 | | down |
| RP11-593P24.4 | -1.584769598 | 0.587905512 | | down |
| RNA5SP415 | -1.584769598 | 0.587905512 | | down |
| WI2-8325B5.1 | -1.584769598 | 0.587905512 | | down |
| RP11-467N20.7 | -1.584769178 | 0.587905612 | | down |
| FUCA1P1 | -1.58464233 | 0.462165166 | | down |
| TMEM232 | -1.584383271 | 0.000104254 | 0.000980652 | down |
| COL6A6 | -1.584267131 | 0.023332288 | 0.069556983 | down |
| NOS2P2 | -1.584201505 | 0.183933687 | | down |
| AC007405.4 | -1.584109579 | 0.007527779 | 0.029563999 | down |
| NA | -1.584007538 | 0.264983228 | | down |
| NA | -1.583759562 | 0.359875792 | 0.498116383 | down |
| AC073465.2 | -1.583603697 | 0.269784173 | | down |
| KCNG3 | -1.583597641 | 0.005477544 | 0.023253825 | down |
| COBL | -1.583550439 | 1.18E-08 | 4.64E-07 | down |
| NA | -1.582926637 | 0.048701823 | 0.120111441 | down |
| SLC12A1 | -1.582693973 | 0.029377189 | 0.082620324 | down |
| IL22RA1 | -1.582519174 | 3.34E-05 | 0.000382267 | down |
| FGFR2 | -1.582474843 | 1.51E-10 | 9.71E-09 | down |
| RP11-132N15.1 | -1.58236831 | 0.588348772 | | down |
| NA | -1.582317696 | 0.365697613 | | down |
| ESX1 | -1.582103969 | 0.512198013 | | down |
| RP11-285E9.6 | -1.582098339 | 0.010216973 | 0.037283949 | down |
| SIAH2-AS1 | -1.582069183 | 0.004621684 | 0.020419689 | down |
| RP11-161I6.2 | -1.582067929 | 0.068342847 | 0.153611248 | down |
| CTB-162E12.1 | -1.582049005 | 0.588424342 | | down |
| RP11-794G24.1 | -1.582048876 | 0.025973966 | 0.075419139 | down |

| | | | | |
|---|---|---|---|---|
| METTL11B | -1.581694297 | 0.512791439 | | down |
| NDUFA5P2 | -1.58167262 | 0.588637509 | | down |
| MYO3B | -1.581439131 | 0.000973235 | 0.005922598 | down |
| RP11-231P20.6 | -1.581295823 | 0.417120929 | | down |
| CTC-329H14.1 | -1.581213548 | 0.413723527 | | down |
| RP11-96C21.2 | -1.581087971 | 0.30760611 | | down |
| RNU7-176P | -1.581045116 | 0.588785875 | | down |
| AC010731.4 | -1.581045116 | 0.588785875 | | down |
| NA | -1.581045116 | 0.588785875 | | down |
| MRPL9P1 | -1.581045116 | 0.588785875 | | down |
| RP11-67H2.1 | -1.581045116 | 0.588785875 | | down |
| RP11-536C10.10 | -1.581045116 | 0.588785875 | | down |
| RP11-350A1.2 | -1.581045116 | 0.588785875 | | down |
| CTB-147N14.4 | -1.581045116 | 0.588785875 | | down |
| MT-ND1 | -1.58092173 | 0.000532862 | 0.003664294 | down |
| RN7SL153P | -1.580721229 | 0.46416367 | | down |
| ZSCAN5C | -1.580308741 | 0.122168421 | 0.232983879 | down |
| RP11-148B3.1 | -1.580287202 | 0.059793538 | 0.139334293 | down |
| MIR4477A | -1.580262968 | 0.357244364 | | down |
| RP11-448G15.1 | -1.580224944 | 0.00835085 | 0.031956814 | down |
| MT-RNR2 | -1.580198846 | 0.012774227 | 0.044193731 | down |
| RP11-526P6.1 | -1.580157152 | 0.557102108 | | down |
| POM121L2 | -1.580070951 | 0.56900384 | | down |
| ACTN1-AS1 | -1.579963045 | 0.002530071 | 0.0126888 | down |
| RPL18AP8 | -1.579922595 | 0.589051326 | | down |
| GK2 | -1.579922595 | 0.589051326 | | down |
| RP11-513017.3 | -1.579922595 | 0.589051326 | | down |
| RP11-347H15.6 | -1.579922595 | 0.589051326 | | down |
| OR5W1P | -1.579922595 | 0.589051326 | | down |
| KRTAP1-1 | -1.579922595 | 0.589051326 | | down |
| RP11-92G19.2 | -1.579922595 | 0.589051326 | | down |
| RP11-321M21.1 | -1.579922595 | 0.589051326 | | down |
| AP000946.2 | -1.579922595 | 0.589051326 | | down |
| CTC-367F4.1 | -1.579810276 | 0.249803079 | 0.384363603 | down |
| IDI2 | -1.579758938 | 0.512815733 | | down |
| CEACAM7 | -1.579676924 | 0.005662103 | 0.023835257 | down |
| HSD3BP4 | -1.579342501 | 0.418061431 | | down |
| OR10AB1P | -1.578734914 | 0.159175108 | 0.2803363 | down |
| SPRR4 | -1.578500148 | 0.589329904 | | down |
| ST8SIA3 | -1.578452991 | 0.024461343 | 0.072142591 | down |
| RN7SL419P | -1.578298427 | 0.377035268 | | down |
| DOCK3 | -1.578218393 | 2.62E-06 | 4.64E-05 | down |
| TKFC | -1.578029441 | 2.08E-11 | 1.70E-09 | down |
| RP11-87C12.5 | -1.57778375 | 0.306777337 | | down |
| SLC26A2 | -1.577742821 | 5.47E-06 | 8.52E-05 | down |
| KLF1 | -1.577725653 | 0.00349288 | 0.016350903 | down |
| RP11-158I9.8 | -1.577592638 | 2.77E-06 | 4.88E-05 | down |
| AC012462.2 | -1.577501921 | 0.303480333 | | down |
| CLRN3 | -1.577271894 | 2.33E-05 | 0.000283414 | down |
| RP4-60503.4 | -1.577232007 | 8.91E-07 | 1.83E-05 | down |
| RP4-781K5.7 | -1.576870362 | 0.589715686 | | down |

| | | | | |
|---|---|---|---|---|
| NBPF25P | -1.576837418 | 0.00023087 | 0.001882809 | down |
| OR8J1 | -1.576669774 | 0.589698105 | | down |
| MRPL2P1 | -1.576669774 | 0.589698105 | | down |
| LMX1B | -1.576491426 | 0.043918495 | 0.111302174 | down |
| TMEM19 | -1.575891973 | 6.74E-07 | 1.46E-05 | down |
| CCDC13 | -1.575736345 | 0.001893046 | 0.010039662 | down |
| LZTS1-AS1 | -1.57567549 | 0.33481783 | 0.473043353 | down |
| KB-1269D1.8 | -1.575397784 | 0.022750297 | 0.068269071 | down |
| RP11-930P14.2 | -1.575316012 | 0.000193694 | 0.001627789 | down |
| SLC27A2 | -1.575248811 | 8.87E-07 | 1.83E-05 | down |
| AC139099.7 | -1.575183735 | 0.566141662 | | down |
| CTB-47B8.5 | -1.575146363 | 0.376612484 | | down |
| AC009505.4 | -1.575019847 | 0.376498642 | | down |
| RP11-575L7.4 | -1.574823853 | 0.090792253 | 0.188668035 | down |
| AATK | -1.574697157 | 3.45E-07 | 8.15E-06 | down |
| NA | -1.574649866 | 0.322085485 | 0.460840856 | down |
| RPL21P7 | -1.57446122 | 0.001625687 | 0.00890078 | down |
| OR14K1 | -1.574351917 | 0.569799125 | | down |
| PDCD6IPP2 | -1.574197339 | 0.001451136 | 0.008126363 | down |
| C13-47488600E17 | -1.573387795 | 0.000363421 | 0.002712713 | down |
| RP11-14K2.1 | -1.573256822 | 0.519299496 | | down |
| EQTN | -1.573250398 | 0.0126408 | 0.043874792 | down |
| LINC01397 | -1.573069457 | 0.049492485 | 0.121443338 | down |
| NA | -1.573053303 | 0.56700331 | | down |
| TCEB2P2 | -1.572755273 | 0.34529934 | | down |
| ARL2BPP3 | -1.572682857 | 0.59070741 | | down |
| RP11-1151B14.1 | -1.572625614 | 0.56829872 | | down |
| NA | -1.572437705 | 0.06511629 | | down |
| FXNP2 | -1.57238995 | 0.570120329 | | down |
| RP11-57A19.7 | -1.572283014 | 0.241205071 | 0.375013722 | down |
| AC004019.10 | -1.572217886 | 0.590753229 | | down |
| C2orf88 | -1.572182264 | 2.59E-10 | 1.56E-08 | down |
| RP11-121C2.3 | -1.572093908 | 0.51244416 | | down |
| SHANK2 | -1.571991095 | 4.38E-06 | 7.12E-05 | down |
| RP5-1139I1.2 | -1.571791476 | 0.372258888 | | down |
| MIR2116 | -1.571667421 | 0.157801114 | | down |
| MAOB | -1.571548252 | 1.98E-06 | 3.67E-05 | down |
| RP11-707M1.1 | -1.571242033 | 0.153933426 | 0.273642225 | down |
| LINC00547 | -1.571070572 | 0.514556891 | | down |
| AZGP1P1 | -1.570386101 | 0.030573535 | 0.084968441 | down |
| AC004490.1 | -1.570261212 | 0.021592624 | 0.065680754 | down |
| IRX1 | -1.569890237 | 0.419521205 | | down |
| RP11-855A2.3 | -1.569794203 | 0.036126508 | 0.096307437 | down |
| AC010091.1 | -1.569524349 | 0.571138596 | | down |
| AC139712.4 | -1.56942164 | 0.591416394 | | down |
| PRLR | -1.56901501 | 6.63E-06 | 0.00010058 | down |
| RP11-131N11.4 | -1.568987185 | 0.094255251 | | down |
| RP11-1260E13.3 | -1.568955579 | 0.029910199 | 0.083588104 | down |
| RSPH10B | -1.568729354 | 0.012361634 | 0.043125048 | down |
| DPPA5P1 | -1.568539617 | 0.510222916 | | down |
| GDF5OS | -1.568324315 | 0.213962299 | 0.344827986 | down |

| | | | | |
|---|---|---|---|---|
| RP11-465N4.4 | -1.567716761 | 2.04E-07 | 5.26E-06 | down |
| PPARGC1A | -1.567673091 | 4.73E-09 | 2.03E-07 | down |
| DISC1-IT1 | -1.567440137 | 0.516569396 | | down |
| NDST3 | -1.567231832 | 0.002906245 | 0.014140118 | down |
| NA | -1.567091646 | 0.592089056 | | down |
| TRPM4 | -1.567005527 | 1.48E-08 | 5.60E-07 | down |
| RNU4-14P | -1.566750319 | 0.592113708 | | down |
| IGHV3OR16-15 | -1.566562379 | 0.463635171 | | down |
| LINC01405 | -1.565993456 | 0.095220812 | 0.195193524 | down |
| RPL21P119 | -1.565977668 | 3.84E-05 | 0.000430034 | down |
| CTD-2278I10.1 | -1.565952485 | 0.141624529 | 0.2583356 | down |
| HTN1 | -1.565934086 | 0.564724161 | | down |
| RP11-182J1.14 | -1.565808173 | 0.004332779 | 0.019394362 | down |
| CTA-392C11.2 | -1.565700626 | 0.183198364 | 0.309006685 | down |
| NA | -1.565681501 | 0.018020744 | 0.057444585 | down |
| PRR31 | -1.565604457 | 0.33647379 | | down |
| RP11-123C5.5 | -1.565504293 | 0.565238283 | | down |
| CTD-2028E8.2 | -1.565440347 | 0.20666845 | 0.336721633 | down |
| HSP90AA2P | -1.565433237 | 1.72E-05 | 0.000221448 | down |
| RP4-753M9.1 | -1.565408795 | 0.518699581 | | down |
| RPS2P24 | -1.565150427 | 0.592549259 | | down |
| RPL5P28 | -1.565150427 | 0.592549259 | | down |
| AC007115.3 | -1.565149954 | 0.592549371 | | down |
| CAMK2B | -1.564874658 | 4.36E-05 | 0.000476196 | down |
| LINC00083 | -1.564815462 | 0.15773869 | 0.27855845 | down |
| CTD-2061E19.6 | -1.564426273 | 0.592665033 | | down |
| SLC6A8 | -1.564263371 | 9.83E-07 | 2.00E-05 | down |
| SH3GL2 | -1.564197969 | 0.007054183 | 0.028145535 | down |
| RP11-173M1.4 | -1.564011985 | 0.015821136 | 0.051999543 | down |
| NA | -1.563965297 | 0.59250916 | | down |
| LINC00431 | -1.563852169 | 0.094672503 | 0.194471536 | down |
| RP4-650F12.2 | -1.563830429 | 0.280607512 | | down |
| NA | -1.563813675 | 6.70E-05 | 0.000678137 | down |
| CTD-2530H12.1 | -1.563800462 | 0.464079197 | | down |
| EIF4EBP2P1 | -1.563718439 | 0.567992185 | | down |
| RP3-455J7.3 | -1.563625913 | 0.517283789 | | down |
| ASB9P1 | -1.563590664 | 0.282596299 | 0.419109646 | down |
| AK6P1 | -1.56339712 | 0.59284617 | | down |
| TMEM249 | -1.563300169 | 0.006827547 | 0.027482158 | down |
| AL356289.1 | -1.563282437 | 0.316618891 | 0.455252355 | down |
| NPIPA2 | -1.563281291 | 0.060676334 | 0.140921442 | down |
| R3HDML | -1.563272673 | 0.05413152 | 0.129530976 | down |
| LINC00704 | -1.563256378 | 0.002577372 | 0.012884289 | down |
| NA | -1.563214279 | 0.593008413 | | down |
| IFNA14 | -1.563214279 | 0.593008413 | | down |
| AP000662.9 | -1.563116189 | 0.138946718 | 0.254963366 | down |
| ACSL5 | -1.563105318 | 2.45E-08 | 8.42E-07 | down |
| RBMY2EP | -1.562899407 | 0.199231855 | | down |
| MLNR | -1.56285201 | 0.315374736 | | down |
| RP11-169L17.5 | -1.562729968 | 0.593123279 | | down |
| RP11-393K12.2 | -1.56265469 | 0.592954816 | | down |

| | | | | |
|---|---|---|---|---|
| TEX13B | -1.562605957 | 0.592832044 | | down |
| AP001626.1 | -1.562601816 | 0.028264492 | 0.080364794 | down |
| MARK2P9 | -1.562581552 | 0.217991375 | 0.349318438 | down |
| RP11-91P24.1 | -1.562496757 | 0.008169383 | 0.031430523 | down |
| SFTPC | -1.562268361 | 0.298375315 | | down |
| CKS1BP3 | -1.562267789 | 0.082929766 | | down |
| CTC-1337H24.4 | -1.562209238 | 0.000227069 | 0.001859382 | down |
| RP11-383B4.4 | -1.562035211 | 0.019045614 | 0.059829195 | down |
| OR7D2 | -1.561960069 | 0.061971608 | 0.143155082 | down |
| RP11-433M22.2 | -1.561785178 | 0.4214856 | | down |
| LYZL6 | -1.561651833 | 0.375099299 | | down |
| MOGAT1 | -1.561384485 | 0.318702807 | | down |
| SLC26A5 | -1.560706825 | 0.027481939 | 0.078687447 | down |
| DPF1 | -1.560506511 | 4.99E-05 | 0.000534964 | down |
| OR1E2 | -1.56050125 | 0.51210762 | | down |
| AC007422.1 | -1.560498351 | 0.173198345 | 0.297636052 | down |
| RPL7P38 | -1.560383909 | 0.192331767 | | down |
| NA | -1.560181394 | 5.95E-08 | 1.83E-06 | down |
| MTUS2 | -1.559976414 | 0.000454399 | 0.003225125 | down |
| RP11-43D4.3 | -1.559572635 | 0.097337131 | | down |
| PRR15 | -1.559338172 | 1.28E-06 | 2.52E-05 | down |
| USP41 | -1.559279669 | 0.218062058 | | down |
| PTCRA | -1.55923026 | 0.000164284 | 0.00142241 | down |
| SPA17P1 | -1.559197816 | 0.152849297 | | down |
| RP11-298D21.1 | -1.558959399 | 0.174005668 | 0.298460049 | down |
| NA | -1.558758184 | 0.594065669 | | down |
| RP11-883A18.3 | -1.558723676 | 0.192839063 | 0.320833666 | down |
| RP11-57106.2 | -1.558628786 | 0.233187541 | 0.365915319 | down |
| AC093620.5 | -1.558413018 | 6.39E-06 | 9.77E-05 | down |
| NA | -1.558086409 | 0.346084241 | 0.48474382 | down |
| OR7E126P | -1.558082092 | 0.033225206 | 0.090401927 | down |
| RAG1 | -1.558041519 | 0.00023672 | 0.00192269 | down |
| ASB12 | -1.557947946 | 0.000876102 | 0.005447351 | down |
| RNU7-41P | -1.557876088 | 0.38396716 | | down |
| MYH15 | -1.557713279 | 1.19E-07 | 3.30E-06 | down |
| BRD7P4 | -1.55769778 | 0.424551313 | | down |
| VSTM2A-OT1 | -1.557579756 | 0.187247206 | | down |
| PTCHD3P2 | -1.557537685 | 0.175951448 | | down |
| C20orf187 | -1.55733252 | 0.096796954 | | down |
| RP11-737024.1 | -1.557187901 | 0.19614081 | | down |
| CTD-2653D5.1 | -1.557136918 | 0.174585003 | 0.299009909 | down |
| NDUFA6-AS1 | -1.557132304 | 1.42E-10 | 9.24E-09 | down |
| NA | -1.556992055 | 0.198557684 | | down |
| LINC01515 | -1.556975168 | 3.11E-06 | 5.35E-05 | down |
| FCF1P7 | -1.556970992 | 0.007110638 | 0.028318135 | down |
| Y_RNA | -1.556858329 | 0.594516689 | | down |
| RP11-6L6.7 | -1.55677657 | 0.594481386 | | down |
| CRYBB2 | -1.556607004 | 0.000715344 | 0.004644866 | down |
| RP11-486M3.2 | -1.556530285 | 0.517736895 | | down |
| SLC7A14 | -1.556498382 | 0.00354587 | 0.016552538 | down |
| TBL1XR1-AS1 | -1.556319089 | 0.126772197 | | down |

| | | | | |
|---|---|---|---|---|
| AC006160.5 | -1.556193103 | 0.003962134 | 0.018104693 | down |
| CTC-455F18.1 | -1.555927656 | 0.046209146 | 0.115346259 | down |
| RP11-8L2.1 | -1.555778537 | 0.171112354 | 0.295027746 | down |
| RNU6-1315P | -1.555156556 | 0.404844481 | 0.541281989 | down |
| LINC00882 | -1.555053697 | 0.000700105 | 0.004566643 | down |
| RP11-359D14.2 | -1.554897766 | 0.013489008 | 0.046021321 | down |
| RP13-516M14.4 | -1.554382637 | 5.59E-05 | 0.000585683 | down |
| RP11-1008C21.1 | -1.554185857 | 0.009758129 | 0.035954527 | down |
| RBM17P4 | -1.554130998 | 0.373489701 | | down |
| RP11-415J8.3 | -1.554073049 | 0.000450608 | 0.003202162 | down |
| ZNF358 | -1.554050055 | 6.30E-06 | 9.64E-05 | down |
| EPPK1 | -1.553935127 | 1.02E-05 | 0.000143972 | down |
| NA | -1.553715324 | 0.008540574 | 0.032488353 | down |
| HPGD | -1.553618657 | 4.03E-07 | 9.30E-06 | down |
| ITIH2 | -1.553385493 | 0.000315272 | 0.002425619 | down |
| RP5-1198020.4 | -1.55328399 | 4.07E-07 | 9.38E-06 | down |
| CTD-2600H12.2 | -1.553184872 | 0.001591782 | 0.00874607 | down |
| MIR940 | -1.553071946 | 1.55E-05 | 0.000204017 | down |
| AC010642.2 | -1.553024184 | 0.318787303 | | down |
| HNRNPA1P43 | -1.552850034 | 0.566754375 | | down |
| NRXN3 | -1.552725269 | 3.96E-05 | 0.000440288 | down |
| ETHE1 | -1.552682912 | 3.86E-06 | 6.39E-05 | down |
| DCTN1-AS1 | -1.552454044 | 0.014491492 | 0.048618915 | down |
| RP11-536K17.1 | -1.552421516 | 0.431719243 | | down |
| Y_RNA | -1.552289866 | 0.236317373 | | down |
| RN7SL248P | -1.551902717 | 0.595693729 | | down |
| RP11-332E4.1 | -1.551861418 | 0.025867364 | 0.075189539 | down |
| CHGA | -1.551659718 | 0.001296332 | 0.007438309 | down |
| Y_RNA | -1.551471853 | 0.242425382 | | down |
| CTB-187M2.1 | -1.551417875 | 0.595808943 | | down |
| CPB2-AS1 | -1.551249818 | 0.002639353 | 0.013115566 | down |
| COMTD1 | -1.551194899 | 6.70E-07 | 1.45E-05 | down |
| HMGB1P45 | -1.551171069 | 0.595752316 | | down |
| YAP1P1 | -1.550798143 | 0.257326897 | | down |
| RP11-245P10.6 | -1.550774279 | 0.040535465 | 0.104946154 | down |
| RP11-595B24.1 | -1.550609613 | 0.178675959 | 0.30374812 | down |
| RAD51AP2 | -1.550574129 | 0.174140799 | 0.298640685 | down |
| RP11-252E2.2 | -1.550418068 | 0.438675715 | 0.572850575 | down |
| ABHD17AP3 | -1.550408387 | 0.008620736 | 0.032710457 | down |
| CLLU1 | -1.55015367 | 0.048507077 | 0.119750291 | down |
| RP11-203H19.2 | -1.550132655 | 0.098044493 | | down |
| CHDH | -1.549756299 | 4.41E-10 | 2.52E-08 | down |
| MMP15 | -1.549723713 | 9.29E-07 | 1.90E-05 | down |
| AC009945.4 | -1.549426755 | 0.428043987 | | down |
| HMGCS2 | -1.549227967 | 0.000764118 | 0.004897008 | down |
| RPL23AP66 | -1.549083441 | 0.430575982 | | down |
| RP11-21A7A.4 | -1.548907432 | 0.019798768 | 0.061596168 | down |
| CTC-228N24.2 | -1.548890537 | 0.075636674 | 0.165297115 | down |
| AC009410.1 | -1.548683813 | 0.316184558 | | down |
| NDUFA9P1 | -1.548638836 | 0.058627206 | 0.137394365 | down |
| RNU6-1183P | -1.548609628 | 0.243388274 | 0.377431471 | down |

| | | | | |
|---|---|---|---|---|
| IGHV3-47 | -1.548488774 | 0.299979129 | | down |
| STK25P1 | -1.548123005 | 0.389153838 | | down |
| MFSD3 | -1.548067122 | 1.57E-06 | 3.01E-05 | down |
| CR1L | -1.547961051 | 0.003033674 | 0.014636856 | down |
| AC003986.7 | -1.547602028 | 0.145894464 | 0.263813017 | down |
| AC104451.2 | -1.547530184 | 0.473426865 | | down |
| AC010729.1 | -1.547485937 | 0.521791102 | | down |
| PP14571 | -1.547448995 | 0.00164045 | 0.008963476 | down |
| RP11-379J13.2 | -1.547043371 | 0.522659606 | | down |
| RP11-314A15.3 | -1.547037701 | 0.559786106 | | down |
| RP11-51P8.1 | -1.54700944 | 0.596856988 | | down |
| RPS27P14 | -1.547006978 | 0.379696935 | | down |
| GABRB2 | -1.546768068 | 0.012041115 | 0.042301597 | down |
| HMGA1P1 | -1.546729715 | 0.351434334 | | down |
| LINC00278 | -1.546709449 | 0.025871392 | 0.075193974 | down |
| VSIG10 | -1.546530855 | 3.51E-14 | 5.64E-12 | down |
| AC013402.5 | -1.546398441 | 0.596949591 | | down |
| RP11-138A23.2 | -1.546199376 | 0.59704966 | | down |
| Y_RNA | -1.546199376 | 0.59704966 | | down |
| RP11-244B22.6 | -1.546199376 | 0.59704966 | | down |
| RP11-165N19.2 | -1.546157528 | 0.390159721 | | down |
| EIF4E1B | -1.546121254 | 0.174802816 | | down |
| RP11-216P6.2 | -1.546008109 | 0.303781541 | | down |
| AC007050.17 | -1.545951536 | 0.189244961 | | down |
| C22orf24 | -1.545595794 | 0.000922132 | 0.005688814 | down |
| CFAP61 | -1.545485926 | 0.019252766 | 0.060379283 | down |
| C8A | -1.545465242 | 0.597044286 | | down |
| KLHL33 | -1.545404098 | 9.07E-05 | 0.000872084 | down |
| IGFN1 | -1.545066657 | 0.004497964 | 0.019993615 | down |
| TRAJ2 | -1.544963492 | 0.030408219 | 0.084653868 | down |
| RNU6-991P | -1.544949529 | 0.597233552 | | down |
| RP11-424I3.1 | -1.544949529 | 0.597233552 | | down |
| NA | -1.544898988 | 0.293977068 | 0.431409516 | down |
| MTCO1P40 | -1.544800225 | 0.000258092 | 0.00206391 | down |
| IHH | -1.544761778 | 1.84E-05 | 0.000233508 | down |
| PACERR | -1.544637336 | 0.070788856 | 0.1575928 | down |
| CTD-3010D24.3 | -1.544548004 | 0.00021819 | 0.001795974 | down |
| RP1-261D10.2 | -1.544320502 | 8.40E-05 | 0.000818115 | down |
| C14orf105 | -1.544157693 | 0.139876817 | 0.256104794 | down |
| RP3-337H4.8 | -1.544136978 | 6.98E-05 | 0.000700814 | down |
| C7orf65 | -1.543957479 | 0.518704455 | | down |
| AC010890.1 | -1.543936324 | 0.007791572 | 0.030375115 | down |
| RP11-38L15.2 | -1.543884019 | 0.201785309 | 0.330955226 | down |
| OR9A2 | -1.543497937 | 0.597692394 | | down |
| NA | -1.543075473 | 0.393053342 | | down |
| NA | -1.542962539 | 0.364483321 | | down |
| NA | -1.542946523 | 0.000848538 | 0.005321115 | down |
| DSG4 | -1.542744416 | 0.120307787 | 0.230562004 | down |
| RP11-323J4.1 | -1.542711771 | 0.070247772 | 0.156753124 | down |
| CTD-2547H18.1 | -1.54271147 | 0.000205836 | 0.001712112 | down |
| RPL36AP13 | -1.542408166 | 0.02577791 | 0.074973044 | down |

| | | | | |
|---|---|---|---|---|
| AC106876.2 | -1.542264789 | 0.001923532 | 0.010167242 | down |
| RP11-382A18.3 | -1.542204644 | 0.598000208 | | down |
| RPS3AP49 | -1.542165008 | 0.000849253 | 0.005323378 | down |
| RAD17P2 | -1.542014137 | 0.167927642 | | down |
| RNA5SP179 | -1.541653333 | 0.598131447 | | down |
| SNORA42 | -1.541641278 | 0.5761336 | | down |
| AC012314.19 | -1.541595764 | 0.218309023 | 0.349732126 | down |
| FAM188B2 | -1.541508285 | 0.384513598 | 0.52220986 | down |
| ATOH7 | -1.541439832 | 0.011791214 | 0.041627898 | down |
| ENPP1 | -1.541380121 | 3.46E-11 | 2.70E-09 | down |
| C22orf31 | -1.541357145 | 0.001295781 | 0.007436569 | down |
| MIR6070 | -1.541244745 | 0.598228718 | | down |
| RP11-434D9.1 | -1.541189687 | 0.063709039 | 0.145967694 | down |
| RP5-964H19.2 | -1.54078917 | 0.52045146 | | down |
| RPSAP46 | -1.54075191 | 0.147693359 | | down |
| EIF4A1P9 | -1.540498153 | 0.522162235 | | down |
| CTD-2540F13.2 | -1.540491927 | 9.09E-05 | 0.000873633 | down |
| NA | -1.540447214 | 0.598418605 | | down |
| NA | -1.540426725 | 0.00030814 | 0.002382953 | down |
| AC016907.3 | -1.540166109 | 0.024432753 | 0.072079499 | down |
| TUBBP6 | -1.540118321 | 0.59849692 | | down |
| NA | -1.540118321 | 0.59849692 | | down |
| Y_RNA | -1.539846438 | 0.567226054 | | down |
| RP11-365F18.3 | -1.53982085 | 0.574796043 | | down |
| RP11-326J18.1 | -1.539764554 | 0.476137875 | | down |
| RNFT1P3 | -1.539694415 | 0.440033708 | | down |
| RP11-630D6.5 | -1.539681693 | 0.386995677 | | down |
| RNMTL1P2 | -1.539659719 | 0.598606129 | | down |
| RP11-210M15.1 | -1.5396529 | 0.307304123 | | down |
| RP11-730N24.1 | -1.5395964 | 0.598509357 | | down |
| RP11-666E17.1 | -1.5395964 | 0.598509357 | | down |
| TMEM160 | -1.539281636 | 1.05E-05 | 0.000147032 | down |
| CTA-331P3.1 | -1.539070174 | 0.476885037 | | down |
| KRT18P1 | -1.538959693 | 0.052369795 | 0.126371748 | down |
| RPL7P17 | -1.538830159 | 0.30852535 | | down |
| NA | -1.538812552 | 0.522257591 | | down |
| RP11-567C2.1 | -1.538618387 | 0.004935904 | 0.021473256 | down |
| AC097468.4 | -1.538361074 | 0.027736285 | 0.079219406 | down |
| AC019070.1 | -1.538344758 | 0.52256751 | | down |
| RNF222 | -1.538067345 | 0.021850755 | 0.06627805 | down |
| CADM2-AS1 | -1.538011055 | 0.407299349 | 0.543910643 | down |
| RPL36P2 | -1.537997898 | 0.431916455 | | down |
| RNU6-795P | -1.537900092 | 0.449834328 | 0.582811788 | down |
| MIR376C | -1.537890391 | 0.599027551 | | down |
| NEB | -1.537838115 | 0.000229933 | 0.001877716 | down |
| AC006380.3 | -1.537730203 | 0.388625606 | | down |
| RP5-1070A16.1 | -1.537552568 | 0.599057015 | | down |
| RP11-472N19.3 | -1.537418688 | 0.294119441 | | down |
| RP11-21M24.3 | -1.537398296 | 0.162731699 | 0.284813653 | down |
| RP11-22L13.1 | -1.537182905 | 0.007630483 | 0.029908707 | down |
| OLA1P3 | -1.537099695 | 0.114358863 | | down |

| | | | | |
|---|---|---|---|---|
| ZSCAN1 | -1.537044756 | 0.001097847 | 0.006539196 | down |
| LINC01091 | -1.536970069 | 0.000166427 | 0.001436776 | down |
| CFAP47 | -1.536958749 | 0.131306521 | 0.245015355 | down |
| EHMT2-AS1 | -1.536926536 | 0.000445359 | 0.003171456 | down |
| CTD-3105H18.16 | -1.536805988 | 0.599235031 | | down |
| KB-1460A1.3 | -1.536777052 | 0.065340075 | 0.148549165 | down |
| RP11-381K20.2 | -1.536452633 | 2.33E-08 | 8.09E-07 | down |
| NA | -1.536382563 | 0.382130415 | 0.519947165 | down |
| TOP1P1 | -1.536326426 | 0.000241044 | 0.001953052 | down |
| RN7SL620P | -1.536320151 | 0.229952604 | 0.362318861 | down |
| RP11-353M9.1 | -1.536317492 | 0.119902962 | | down |
| LLNLR-307A6.1 | -1.535841479 | 0.002677868 | 0.013263083 | down |
| RP6-186E3.1 | -1.535815477 | 0.577756626 | | down |
| AC006994.3 | -1.535655954 | 0.363725936 | | down |
| LINC01070 | -1.535541352 | 0.599536626 | | down |
| U47924.27 | -1.535202506 | 8.47E-05 | 0.00082382 | down |
| CTD-2062A1.2 | -1.534921515 | 0.575265954 | | down |
| GS1-542M4.4 | -1.534831938 | 0.474963698 | | down |
| RP11-541F9.2 | -1.534619589 | 0.599431417 | | down |
| CLCA4 | -1.534437217 | 0.006975114 | 0.027926484 | down |
| SERINC2 | -1.53441017 | 1.26E-06 | 2.48E-05 | down |
| ZRANB2-AS2 | -1.534142596 | 0.000366775 | 0.00273163 | down |
| CYCSP41 | -1.534136692 | 0.599922035 | | down |
| EVPL | -1.533875504 | 2.55E-07 | 6.33E-06 | down |
| MTND1P23 | -1.533723834 | 0.000214998 | 0.001773588 | down |
| GPS2P2 | -1.53349855 | 0.600074145 | | down |
| ARHGAP44 | -1.533432936 | 4.78E-10 | 2.70E-08 | down |
| CCNJL | -1.533377876 | 1.90E-10 | 1.19E-08 | down |
| OTOF | -1.533357869 | 0.000331056 | 0.002527636 | down |
| ANKRD20A1 | -1.533283978 | 0.037390196 | 0.098773504 | down |
| SPATA8-AS1 | -1.533171172 | 0.600152186 | | down |
| CTA-363E6.2 | -1.533157296 | 0.014478289 | 0.048590898 | down |
| Y_RNA | -1.533061292 | 0.278332938 | | down |
| RP11-540B6.2 | -1.532912139 | 0.095339156 | | down |
| MIR3180-5 | -1.532902902 | 0.600216141 | | down |
| CTC-338M12.2 | -1.532727606 | 0.000659535 | 0.004347236 | down |
| MINOS1P2 | -1.532675866 | 0.073022645 | | down |
| NWD2 | -1.532623675 | 0.097134825 | 0.197986342 | down |
| AC079466.1 | -1.532216857 | 0.600270113 | | down |
| NA | -1.532096126 | 3.41E-07 | 8.08E-06 | down |
| AC092933.4 | -1.532095162 | 0.254759968 | 0.389794688 | down |
| NXNL2 | -1.532055557 | 0.005591465 | 0.023627283 | down |
| RNU7-49P | -1.531936974 | 0.037452524 | 0.098912063 | down |
| NA | -1.531922973 | 0.314241139 | | down |
| CDC42BPG | -1.531858299 | 1.67E-12 | 1.74E-10 | down |
| RP11-613H2.1 | -1.53173075 | 0.600495614 | | down |
| ULK4P3 | -1.531640968 | 0.112490067 | | down |
| C11orf72 | -1.531611921 | 0.102756936 | 0.20593822 | down |
| Y_RNA | -1.531592297 | 0.600528629 | | down |
| RP11-414H23.2 | -1.531592297 | 0.600528629 | | down |
| IVL | -1.531579222 | 0.600356953 | | down |

| | | | | |
|---|---|---|---|---|
| RN7SKP133 | -1.531407356 | 0.600000391 | | down |
| RGS6 | -1.531230481 | 6.08E-06 | 9.34E-05 | down |
| LAMTOR4 | -1.531191281 | 1.18E-06 | 2.35E-05 | down |
| SCHLAP1 | -1.530969589 | 0.401997656 | | down |
| CFAP44-AS1 | -1.530935312 | 0.208025411 | | down |
| LLNLR-246C6.1 | -1.530910047 | 0.000697039 | 0.004550594 | down |
| RP1-45C12.1 | -1.530869291 | 0.001064582 | 0.006376073 | down |
| RP11-344F13.1 | -1.530752427 | 0.051415174 | 0.124835978 | down |
| CDH4 | -1.530698034 | 0.001301001 | 0.007456557 | down |
| KRT8 | -1.530669308 | 8.70E-06 | 0.000125903 | down |
| NHSL1 | -1.530626211 | 1.78E-08 | 6.43E-07 | down |
| CELSR3-AS1 | -1.530592137 | 0.000694566 | 0.004536421 | down |
| NA | -1.530220344 | 0.159033819 | 0.280195633 | down |
| SLC22A18 | -1.530200531 | 1.01E-05 | 0.000141963 | down |
| RP11-1144P22.1 | -1.530078066 | 0.384738674 | 0.52249192 | down |
| AFP | -1.529884177 | 0.193449015 | | down |
| PPIHP1 | -1.529686494 | 0.180224622 | | down |
| LRRC26 | -1.529683717 | 0.001105142 | 0.006572224 | down |
| RP11-855O10.3 | -1.529563827 | 0.60101242 | | down |
| RP11-449H3.3 | -1.529508072 | 0.601025721 | | down |
| PTPRH | -1.52939476 | 7.02E-07 | 1.51E-05 | down |
| Metazoa_SRP | -1.529377391 | 0.077454026 | 0.168139719 | down |
| DGKI | -1.529278937 | 0.000504952 | 0.003503911 | down |
| RN7SL300P | -1.529239047 | 0.287169432 | 0.424362718 | down |
| RNU6-1069P | -1.529128807 | 0.102422318 | 0.20546 | down |
| RN7SL149P | -1.529077786 | 0.479680234 | | down |
| TMEM220-AS1 | -1.528900137 | 7.84E-06 | 0.000115309 | down |
| ZNF205 | -1.528766067 | 5.45E-07 | 1.22E-05 | down |
| MIR3134 | -1.528732321 | 0.432226034 | | down |
| RNU6-806P | -1.528636748 | 0.601233588 | | down |
| WEE2 | -1.528525738 | 0.116464046 | 0.225207298 | down |
| RP6-74O6.2 | -1.528499617 | 0.001496636 | 0.008320677 | down |
| EPX | -1.528381982 | 0.003697901 | 0.017115965 | down |
| RPS14P7 | -1.527941846 | 0.60139939 | | down |
| L22NC03-121E8.[illegible] | -1.527941846 | 0.60139939 | | down |
| PPP1R1A | -1.527864384 | 0.000331406 | 0.002529022 | down |
| SMPD3 | -1.527793959 | 1.69E-08 | 6.21E-07 | down |
| C5orf46 | -1.527681509 | 0.350426922 | 0.489138935 | down |
| WT1-AS | -1.527681188 | 0.118589739 | 0.228172277 | down |
| NA | -1.527465525 | 0.02772747 | 0.079212508 | down |
| AC144831.3 | -1.527341884 | 1.26E-05 | 0.00017085 | down |
| RNA5SP425 | -1.52730906 | 0.157085456 | | down |
| Metazoa_SRP | -1.527247866 | 0.004211208 | 0.018991523 | down |
| NA | -1.526987249 | 0.081579277 | 0.174657435 | down |
| PGM5P2 | -1.526971798 | 0.006268331 | 0.02578745 | down |
| RP11-367F23.1 | -1.526852243 | 0.209595235 | | down |
| RP11-145M4.1 | -1.526778119 | 0.580329171 | | down |
| CTA-407F11.7 | -1.52675714 | 0.434367184 | | down |
| SOWAHB | -1.526674598 | 1.97E-05 | 0.000247259 | down |
| RP11-831A10.2 | -1.52640903 | 0.601716296 | | down |
| RP11-96L14.8 | -1.526377752 | 0.524603984 | | down |

| | | | | |
|---|---|---|---|---|
| RAB28P2 | -1.526348489 | 0.57343013 | | down |
| OR2B2 | -1.525958182 | 0.410393492 | 0.54716704 | down |
| AC010761.14 | -1.525858471 | 0.208621509 | | down |
| BNIP3P30 | -1.525743245 | 0.206393305 | | down |
| HMGN5 | -1.52573654 | 4.49E-10 | 2.55E-08 | down |
| CTD-2523D13.1 | -1.52559212 | 0.601852739 | | down |
| KB-173C10.1 | -1.525444091 | 0.041712321 | 0.107267394 | down |
| RP3-522P13.2 | -1.525284818 | 0.528550278 | | down |
| RP11-108E14.1 | -1.525195517 | 0.386493105 | | down |
| AC007792.1 | -1.52511449 | 0.529438962 | | down |
| RP11-561B11.6 | -1.524879221 | 0.602081771 | | down |
| CTD-2562J17.2 | -1.524585491 | 0.249656656 | 0.384236697 | down |
| NA | -1.524503161 | 0.027736117 | 0.079219406 | down |
| TMC3 | -1.524405985 | 0.007462266 | 0.029379679 | down |
| RP11-963H4.5 | -1.524135496 | 0.158344211 | | down |
| RP11-382A20.7 | -1.524005366 | 0.077635467 | | down |
| AC007365.3 | -1.523956039 | 0.065463615 | 0.148751238 | down |
| RP11-10L7.1 | -1.523947554 | 0.021656669 | 0.065848899 | down |
| SNRPEP5 | -1.523790306 | 0.293058941 | | down |
| LINC01339 | -1.523607333 | 0.577513362 | | down |
| CTD-2515H24.2 | -1.523545041 | 0.186696039 | | down |
| GOLGA8F | -1.523513729 | 0.136671785 | 0.251973927 | down |
| NA | -1.523457739 | 0.040421674 | 0.10475535 | down |
| RP11-795H16.2 | -1.523225351 | 0.602525275 | | down |
| LINC00656 | -1.52311062 | 0.213852792 | 0.344725468 | down |
| RP11-68L1.1 | -1.523059811 | 0.60251656 | | down |
| CTD-2527I21.15 | -1.523053003 | 0.098739062 | 0.200247493 | down |
| ARHGAP23P1 | -1.523031094 | 0.578013214 | | down |
| MTND6P12 | -1.522855533 | 0.602613595 | | down |
| NA | -1.522821051 | 8.62E-05 | 0.000834725 | down |
| LRRC75A | -1.522702962 | 1.51E-08 | 5.71E-07 | down |
| RNU6-950P | -1.522478271 | 0.602597267 | | down |
| SOST | -1.522193601 | 0.28054277 | | down |
| TRIM64B | -1.522138909 | 0.479927923 | | down |
| AC009274.6 | -1.522069698 | 0.230769837 | 0.363206505 | down |
| GRIN2A | -1.522037708 | 0.000777892 | 0.004960941 | down |
| MYO15B | -1.521834121 | 2.47E-09 | 1.14E-07 | down |
| 3C11-4932300016. | -1.521345908 | 0.602974184 | | down |
| CPA1 | -1.521149147 | 0.032401493 | 0.088763605 | down |
| NA | -1.521068557 | 0.571271992 | | down |
| RP11-43F13.4 | -1.521058286 | 0.00040093 | 0.00291942 | down |
| TDP2 | -1.52093528 | 9.00E-12 | 7.98E-10 | down |
| GS1-124K5.2 | -1.520904983 | 0.206451719 | | down |
| ATCAY | -1.520817685 | 0.001867975 | 0.009943556 | down |
| RP11-22C8.1 | -1.520773876 | 0.476142095 | | down |
| RP1-167G20.2 | -1.520443275 | 0.532268685 | | down |
| ENTPD2 | -1.520337343 | 4.43E-07 | 1.01E-05 | down |
| LMOD3 | -1.520303914 | 0.00015375 | 0.001346331 | down |
| MTND2P7 | -1.520185048 | 0.603251532 | | down |
| RP11-23B7.4 | -1.520185048 | 0.603251532 | | down |
| CTB-159G17.4 | -1.520185048 | 0.603251532 | | down |

| | | | | |
|---|---|---|---|---|
| AC016910.1 | -1.52012999 | 0.19690635 | 0.32551196 | down |
| LINC01497 | -1.520019148 | 0.481531333 | | down |
| AP000435.3 | -1.519987109 | 0.603298828 | | down |
| RSPH4A | -1.519752206 | 0.000218996 | 0.001801125 | down |
| RP11-259P15.4 | -1.519614646 | 0.435876589 | | down |
| RP4-671014.6 | -1.519569139 | 0.001402279 | 0.007907296 | down |
| RPSAP24 | -1.519520218 | 0.603362827 | | down |
| RP11-604N13.1 | -1.519520218 | 0.603362827 | | down |
| RFC5P1 | -1.519468518 | 0.391537993 | | down |
| CSMD3 | -1.5192088 | 0.113802164 | 0.221542941 | down |
| AC012507.3 | -1.519155274 | 0.098329478 | 0.199625781 | down |
| PPIAP15 | -1.519140687 | 0.525576899 | | down |
| NPAP1 | -1.519042408 | 0.316665114 | | down |
| C7orf57 | -1.519034222 | 0.055206655 | 0.131407761 | down |
| RP3-495K2.3 | -1.518939588 | 0.289928634 | 0.427158487 | down |
| ZNF101P2 | -1.518830367 | 0.281344034 | | down |
| PDX1-AS1 | -1.518746033 | 0.110303586 | 0.216865547 | down |
| CTB-181F24.1 | -1.518722302 | 0.233782975 | 0.366504696 | down |
| RPSAP9 | -1.518626506 | 2.68E-07 | 6.62E-06 | down |
| SELENBP1 | -1.518457248 | 1.32E-06 | 2.59E-05 | down |
| FAM197Y5P | -1.518389485 | 0.603680632 | | down |
| VAC14-AS1 | -1.518089326 | 0.000662684 | 0.004363331 | down |
| OASL | -1.517735339 | 2.72E-05 | 0.000322463 | down |
| TTTY14 | -1.517624662 | 0.036944806 | 0.097846417 | down |
| GRPEL2P2 | -1.517620916 | 0.60362848 | | down |
| NA | -1.51740995 | 3.94E-05 | 0.000438918 | down |
| CTD-3118D11.3 | -1.517397772 | 0.351944587 | 0.490641881 | down |
| RP11-348B17.1 | -1.517313181 | 0.212922425 | | down |
| RP11-27G14.4 | -1.517262847 | 6.20E-05 | 0.000637654 | down |
| C2orf81 | -1.517205341 | 0.029757462 | 0.083297604 | down |
| RP11-563H6.1 | -1.517026313 | 0.001223316 | 0.007122733 | down |
| CTD-2020K17.4 | -1.516995801 | 1.92E-05 | 0.000242292 | down |
| RP11-5A11.1 | -1.516865411 | 0.265224899 | 0.400840284 | down |
| RP11-114G11.5 | -1.516735279 | 0.077895834 | 0.16887736 | down |
| FAM187B2P | -1.516674689 | 0.060599885 | 0.14082009 | down |
| FUOM | -1.516518944 | 1.01E-07 | 2.85E-06 | down |
| RP11-66D17.5 | -1.516481022 | 0.027418705 | 0.078566291 | down |
| MTRNR2L12 | -1.516352633 | 0.000400818 | 0.002919316 | down |
| PIPOX | -1.516321262 | 6.95E-06 | 0.000104426 | down |
| MTND5P23 | -1.516176198 | 0.604162818 | | down |
| RP11-810P12.7 | -1.516104448 | 4.58E-06 | 7.38E-05 | down |
| RPL7AP15 | -1.516075261 | 0.482515646 | | down |
| RP11-169F17.1 | -1.516043137 | 0.135184235 | 0.249932935 | down |
| CTD-2292P10.4 | -1.51602219 | 0.010776828 | 0.038841053 | down |
| RP11-185N2.1 | -1.515980511 | 0.582555176 | | down |
| ANKRD20A11P | -1.515912557 | 0.017386968 | 0.055821123 | down |
| SCUBE3 | -1.515869062 | 0.000152775 | 0.001339744 | down |
| PTGES3L | -1.515546988 | 1.42E-05 | 0.000189062 | down |
| CISD3 | -1.515499084 | 5.47E-09 | 2.31E-07 | down |
| CMBL | -1.515464227 | 2.55E-06 | 4.53E-05 | down |
| AC019330.1 | -1.515390833 | 0.039669412 | 0.103313785 | down |

| | | | | |
|---|---|---|---|---|
| SNRPFP1 | -1.515066496 | 0.038784002 | | down |
| RYR1 | -1.514967647 | 1.75E-07 | 4.61E-06 | down |
| GRK7 | -1.514813951 | 0.062949801 | 0.144769123 | down |
| RP11-895K13.2 | -1.514738234 | 0.58627536 | | down |
| MIR4285 | -1.514720175 | 0.272783875 | 0.409001846 | down |
| RP11-531A24.3 | -1.514588736 | 0.01845048 | 0.058485568 | down |
| CTB-133G6.2 | -1.514517982 | 0.002614934 | 0.013028703 | down |
| RP11-142L1.1 | -1.514497798 | 0.604507221 | | down |
| RP11-840I19.3 | -1.514417357 | 0.02959703 | 0.083015281 | down |
| RN7SL448P | -1.514230079 | 0.029783852 | 0.083343634 | down |
| RP11-276H7.2 | -1.514217481 | 0.000300814 | 0.002334707 | down |
| GAPDHP34 | -1.514176124 | 0.604688066 | | down |
| SCRT2 | -1.514102997 | 0.604537713 | | down |
| NAT2 | -1.514100975 | 8.57E-06 | 0.000124301 | down |
| MTRNR2L13 | -1.513945103 | 0.604743326 | | down |
| CTB-105N12.2 | -1.513942795 | 0.26360319 | 0.399035823 | down |
| AP004290.1 | -1.513681366 | 0.281934285 | | down |
| LINC00632 | -1.513576108 | 0.001598402 | 0.008777623 | down |
| RP5-912I13.2 | -1.513428111 | 0.023853862 | 0.07075382 | down |
| MIR181A1HG | -1.513342026 | 0.046164798 | 0.115292985 | down |
| RNU6-674P | -1.513284464 | 0.527915463 | | down |
| ST3GAL6-AS1 | -1.513273053 | 0.000390609 | 0.002860163 | down |
| CHST9 | -1.513242442 | 0.002590151 | 0.012932336 | down |
| RP11-425A23.1 | -1.513193529 | 0.484117953 | | down |
| FAM186A | -1.512984161 | 0.000564253 | 0.003837127 | down |
| F12 | -1.512867297 | 6.37E-05 | 0.000650932 | down |
| RP11-247C2.2 | -1.512707561 | 0.004431847 | 0.019764052 | down |
| OR5M6P | -1.512648655 | 0.533723497 | | down |
| AC068057.1 | -1.512635468 | 0.605010144 | | down |
| TECRP2 | -1.512635468 | 0.605010144 | | down |
| LINC01098 | -1.512635468 | 0.605010144 | | down |
| CPXCR1 | -1.512635468 | 0.605010144 | | down |
| PEX11G | -1.512481772 | 6.10E-11 | 4.43E-09 | down |
| RP3-509I19.1 | -1.51236721 | 0.535528465 | | down |
| GDPD3 | -1.51233927 | 1.75E-05 | 0.000224499 | down |
| SNORA74B | -1.512335249 | 0.285364456 | | down |
| LINC00475 | -1.512293914 | 0.000856299 | 0.005351911 | down |
| KCNQ2 | -1.512196493 | 0.001888706 | 0.01002352 | down |
| AC098826.5 | -1.512064154 | 0.139457211 | 0.255634682 | down |
| AC015849.13 | -1.511979853 | 0.133527323 | 0.247696647 | down |
| CRYGC | -1.511916713 | 0.60522861 | | down |
| RP11-526I2.1 | -1.511914706 | 0.323928704 | | down |
| NA | -1.511874353 | 0.000362916 | 0.002711629 | down |
| AC009878.2 | -1.511787369 | 0.576943211 | | down |
| RP11-98J9.1 | -1.511663836 | 0.605289122 | | down |
| ARG2 | -1.511483356 | 2.87E-05 | 0.000337129 | down |
| MROH3P | -1.511452923 | 0.002055294 | 0.010728803 | down |
| FAM95B1 | -1.511268074 | 0.018331738 | 0.058182836 | down |
| SCNN1B | -1.510996251 | 0.000416567 | 0.003005445 | down |
| LINGO2 | -1.510942517 | 0.144722026 | 0.262277028 | down |
| RP11-84A14.4 | -1.510923026 | 0.000205353 | 0.001709351 | down |

| | | | | |
|---|---|---|---|---|
| RP11-57106.1 | -1.510800834 | 0.570818159 | | down |
| MIR193A | -1.510787183 | 0.107148244 | 0.212383273 | down |
| RP11-345J4.5 | -1.510690495 | 0.060062823 | 0.139809984 | down |
| RP11-507K2.6 | -1.51057488 | 0.001791134 | 0.009611151 | down |
| RP5-947P14.2 | -1.510487048 | 0.605524495 | | down |
| BAK1P2 | -1.510231251 | 0.605529282 | | down |
| RP11-165N12.2 | -1.510190262 | 0.481499794 | | down |
| RP1-40E16.2 | -1.510134978 | 0.120315241 | 0.230562004 | down |
| NA | -1.510012458 | 0.064553683 | 0.147296336 | down |
| AC007106.1 | -1.509830798 | 0.605561591 | | down |
| SULT1C3 | -1.509754591 | 0.18489311 | 0.310956594 | down |
| RP11-312B8.2 | -1.509581784 | 0.212619208 | 0.343466943 | down |
| KDM5C-IT1 | -1.509574544 | 0.395828634 | | down |
| RPL23AP71 | -1.509509106 | 0.533391299 | | down |
| MAFTRR | -1.508987948 | 5.92E-05 | 0.000613633 | down |
| MTCYBP15 | -1.508911024 | 0.605901932 | | down |
| RP11-544H14.1 | -1.508911024 | 0.605901932 | | down |
| HNRNPA1P20 | -1.508911024 | 0.605901932 | | down |
| CTC-327F10.4 | -1.508911024 | 0.605901932 | | down |
| RP11-76H14.2 | -1.508911024 | 0.605901932 | | down |
| CTB-60B18.17 | -1.508911024 | 0.605901932 | | down |
| PLEKHJ1 | -1.508697406 | 1.68E-08 | 6.19E-07 | down |
| RP11-820I16.3 | -1.508676813 | 0.139603765 | | down |
| AC073343.13 | -1.508676071 | 0.05155396 | 0.125096672 | down |
| ANKRD63 | -1.50851283 | 0.442961817 | | down |
| RP11-73M18.11 | -1.508349637 | 0.151975215 | | down |
| RP11-65J3.14 | -1.508266181 | 0.277009908 | | down |
| CCAT2 | -1.507788388 | 0.606170853 | | down |
| LINC01067 | -1.507788388 | 0.606170853 | | down |
| LINC01300 | -1.507248422 | 0.019563112 | 0.061014652 | down |
| SNORA68 | -1.507183241 | 0.528396833 | | down |
| SATB2-AS1 | -1.506950404 | 7.35E-05 | 0.0007307 | down |
| TUBB8P6 | -1.506926263 | 0.606377387 | | down |
| NA | -1.506847445 | 0.606442165 | | down |
| RP1-65J11.5 | -1.506847445 | 0.606442165 | | down |
| MIR4265 | -1.506847445 | 0.606442165 | | down |
| MTND5P22 | -1.506847445 | 0.606442165 | | down |
| RN7SL863P | -1.506847445 | 0.606442165 | | down |
| RP11-232A1.2 | -1.506847445 | 0.606442165 | | down |
| RP11-271F18.1 | -1.506847445 | 0.606442165 | | down |
| RP11-1081L13.3 | -1.506847445 | 0.606442165 | | down |
| HNRNPA1P18 | -1.506847445 | 0.606442165 | | down |
| RN7SKP107 | -1.506847445 | 0.606442165 | | down |
| RP11-403B2.10 | -1.506847445 | 0.606442165 | | down |
| U3 | -1.506847445 | 0.606442165 | | down |
| RPL12P36 | -1.506847445 | 0.606442165 | | down |
| MTND2P13 | -1.506847445 | 0.606442165 | | down |
| KRTAP3-2 | -1.506847445 | 0.606442165 | | down |
| RNU6ATAC3P | -1.506847445 | 0.606442165 | | down |
| CTD-3187F8.11 | -1.506847445 | 0.606442165 | | down |
| RP11-144M17.1 | -1.506847445 | 0.606442165 | | down |

| | | | | |
|---|---|---|---|---|
| ACER3 | -1.506426632 | 1.11E-09 | 5.58E-08 | down |
| PRAMEF27 | -1.506354102 | 0.606560326 | | down |
| AC073321.4 | -1.506354102 | 0.606560326 | | down |
| NA | -1.506198895 | 0.32307861 | | down |
| RP11-809N8.5 | -1.506144313 | 0.006255702 | 0.025753125 | down |
| HS3ST2 | -1.505979478 | 0.001218971 | 0.007102056 | down |
| KB-1572G7.3 | -1.505816454 | 0.175203056 | 0.299664909 | down |
| RP11-524H19.2 | -1.505315305 | 0.022240786 | 0.06712901 | down |
| SEMA3B | -1.505154536 | 2.99E-08 | 1.00E-06 | down |
| RP11-173P15.7 | -1.505109097 | 0.586495925 | | down |
| NOS1AP | -1.505053274 | 2.32E-05 | 0.000282823 | down |
| MIR6124 | -1.504995474 | 0.390205181 | | down |
| DSP | -1.504632651 | 1.94E-08 | 6.91E-07 | down |
| RP11-734C14.2 | -1.504516562 | 0.207433771 | | down |
| AC097713.5 | -1.50445256 | 0.315822271 | | down |
| MKNK1-AS1 | -1.504235869 | 0.022386128 | 0.067445721 | down |
| NIFKP7 | -1.504190848 | 0.583707755 | | down |
| CTD-2245F17.9 | -1.503994766 | 0.485189123 | | down |
| CTC-471J1.10 | -1.503669098 | 0.164743187 | | down |
| EDRF1-AS1 | -1.503469191 | 0.025689084 | 0.074816095 | down |
| RNA5SP319 | -1.503401658 | 0.607222053 | | down |
| RP11-96801.5 | -1.503398723 | 0.008295793 | 0.03179472 | down |
| TEKT5 | -1.503106725 | 0.001141539 | 0.006741184 | down |
| RNA5SP503 | -1.503024635 | 0.579665007 | | down |
| NA | -1.502980674 | 0.484276617 | | down |
| PCYT2 | -1.502916968 | 5.28E-09 | 2.24E-07 | down |
| Metazoa_SRP | -1.502850988 | 0.086371492 | 0.182126973 | down |
| NA | -1.502830076 | 0.019327311 | 0.06051196 | down |
| CDC42EP3P1 | -1.502555198 | 0.397410997 | | down |
| RPSAP2 | -1.502478606 | 0.210523709 | 0.341097681 | down |
| RP11-806K15.1 | -1.502343601 | 0.350555887 | | down |
| Y_RNA | -1.502140957 | 0.220882478 | 0.352547358 | down |
| SEZ6 | -1.502036287 | 0.005632748 | 0.023758285 | down |
| RP5-1065J22.8 | -1.502005171 | 2.48E-07 | 6.21E-06 | down |
| Metazoa_SRP | -1.501674676 | 0.037690532 | 0.099392114 | down |
| KRT20 | -1.501623664 | 9.84E-05 | 0.000931283 | down |
| RP11-350E12.4 | -1.501609977 | 0.111846292 | 0.218998305 | down |
| AC090617.1 | -1.50159467 | 0.484982222 | | down |
| RASSF7 | -1.501455156 | 2.99E-06 | 5.18E-05 | down |
| RP11-14807.2 | -1.501377363 | 0.270742918 | | down |
| CALML3-AS1 | -1.501136094 | 0.02051627 | 0.063303976 | down |
| PPP1R17 | -1.501081129 | 0.167003841 | 0.290177746 | down |
| RP11-155I9.1 | -1.501058963 | 0.607828976 | | down |
| RN7SL116P | -1.500977755 | 0.196463391 | 0.324940606 | down |
| RP11-122D10.1 | -1.500879787 | 0.587378154 | | down |
| RP11-691N7.6 | -1.500809425 | 0.095654979 | | down |
| CTC-498J12.3 | -1.500736149 | 0.010591693 | 0.038343997 | down |
| LINC01348 | -1.500697343 | 0.003374339 | 0.015882599 | down |
| AC012613.2 | -1.500665373 | 0.220912616 | 0.352568321 | down |
| NA | -1.500000066 | 0.006101622 | 0.025281636 | down |
| NDUFB7 | -1.499578562 | 1.35E-06 | 2.63E-05 | down |

| | | | | |
|---|---|---|---|---|
| RP11-576I22.2 | -1.499381573 | 0.001245624 | 0.007221832 | down |
| TEKT2 | -1.498778843 | 0.000589808 | 0.003979385 | down |
| Y_RNA | -1.498754728 | 0.608336477 | | down |
| KCNN1 | -1.498677214 | 8.70E-06 | 0.000125903 | down |
| RP11-481J2.1 | -1.498673146 | 0.60835605 | | down |
| EREG | -1.498495535 | 0.000464289 | 0.003280053 | down |
| NA | -1.498214686 | 0.00237521 | 0.012066418 | down |
| ARSEP1 | -1.49806219 | 0.115883116 | 0.224429442 | down |
| CTD-2117L12.1 | -1.497954625 | 0.608573244 | | down |
| RN7SL804P | -1.497954625 | 0.608573244 | | down |
| CTC-303L1.1 | -1.49750649 | 0.170740145 | 0.294591221 | down |
| CTD-2350J17.1 | -1.497301839 | 0.488995825 | | down |
| PDIA2 | -1.497301184 | 1.12E-06 | 2.24E-05 | down |
| LINC01088 | -1.497091585 | 0.000109271 | 0.001018627 | down |
| RPL31P58 | -1.497062012 | 0.400399998 | | down |
| RNU6-464P | -1.496929422 | 0.6087198 | | down |
| NA | -1.49677494 | 0.222997895 | | down |
| RP11-638I8.1 | -1.49658924 | 0.608739843 | | down |
| RP11-1000B6.2 | -1.496576109 | 0.288383867 | | down |
| CRYM-AS1 | -1.496162206 | 0.004316183 | 0.019340104 | down |
| NA | -1.495955558 | 0.300692735 | | down |
| RP13-643D4.1 | -1.495931094 | 0.243492712 | 0.377534966 | down |
| CELA2B | -1.495839709 | 0.024324939 | 0.071810794 | down |
| RP11-309J13.1 | -1.49576649 | 0.398966734 | | down |
| MBL3P | -1.49573411 | 0.60910586 | | down |
| RP11-3M1.3 | -1.495701701 | 0.530614069 | | down |
| RNASE9 | -1.495656162 | 0.609080074 | | down |
| SNORA63 | -1.495567375 | 0.609046899 | | down |
| LINC00605 | -1.495542371 | 0.000375832 | 0.002780471 | down |
| RP13-895J2.3 | -1.495193661 | 0.090156299 | | down |
| C1orf210 | -1.495163298 | 2.94E-06 | 5.12E-05 | down |
| PRAMEF35P | -1.49513666 | 0.609249201 | | down |
| RP3-340N1.6 | -1.49513666 | 0.609249201 | | down |
| RP11-19E11.1 | -1.49513666 | 0.609249201 | | down |
| FAM47E-STBD1 | -1.49513666 | 0.609249201 | | down |
| RP11-529H2.1 | -1.49513666 | 0.609249201 | | down |
| RP11-310A13.2 | -1.49513666 | 0.609249201 | | down |
| NA | -1.49513666 | 0.609249201 | | down |
| SNX19P2 | -1.49513666 | 0.609249201 | | down |
| RP11-80F22.14 | -1.49513666 | 0.609249201 | | down |
| BAGE5 | -1.49513666 | 0.609249201 | | down |
| AC007064.29 | -1.49513666 | 0.609249201 | | down |
| NPR3 | -1.495101609 | 0.001119381 | 0.006641754 | down |
| RP11-502N13.2 | -1.494218134 | 0.397577723 | | down |
| LHX2 | -1.493911778 | 0.01416154 | 0.047810912 | down |
| SIM2 | -1.493758319 | 0.000460606 | 0.003258128 | down |
| H2AFZP4 | -1.493667447 | 0.398647022 | | down |
| NA | -1.493319456 | 0.583030125 | | down |
| AC002511.2 | -1.493228249 | 0.006667001 | 0.027013268 | down |
| RP1-167G20.1 | -1.493055278 | 0.48989657 | | down |
| NA | -1.4930549 | 0.085171182 | 0.180304607 | down |

| | | | | |
|---|---|---|---|---|
| CTC-232P5.4 | -1.493033435 | 0.201118786 | | down |
| ISCA1P2 | -1.493016288 | 0.609713908 | | down |
| AC018878.3 | -1.493016288 | 0.609713908 | | down |
| Y_RNA | -1.493016288 | 0.609713908 | | down |
| AC108448.2 | -1.492949409 | 0.002754704 | 0.013567593 | down |
| RP11-428L9.1 | -1.492790519 | 0.609812232 | | down |
| SETP9 | -1.492747552 | 0.085865668 | 0.181480698 | down |
| MRPL23 | -1.492617667 | 7.42E-06 | 0.00011024 | down |
| ALKBH7 | -1.492595295 | 1.62E-07 | 4.31E-06 | down |
| RP1-78B3.1 | -1.492522335 | 0.068988369 | 0.15466078 | down |
| CTD-2587H19.3 | -1.492373241 | 0.256431692 | 0.391495185 | down |
| TEX41 | -1.49194017 | 0.004968264 | 0.021589027 | down |
| RP11-24M17.3 | -1.491889094 | 0.483606617 | | down |
| OR2A14 | -1.491714792 | 0.490550985 | | down |
| RP11-353H3.1 | -1.491700216 | 0.014595425 | 0.04890208 | down |
| AC004951.6 | -1.491669101 | 0.001423079 | 0.008002055 | down |
| RP11-386G11.10 | -1.491666771 | 0.000239817 | 0.001944162 | down |
| SPANXN4 | -1.491471788 | 0.609904513 | | down |
| GS1-72M22.1 | -1.49141869 | 0.014928923 | 0.049765111 | down |
| CTD-2357A8.3 | -1.491400026 | 0.000532134 | 0.003662642 | down |
| RP11-702L15.4 | -1.491313769 | 0.087768512 | 0.184191918 | down |
| RP11-766F14.2 | -1.491244025 | 0.409334699 | | down |
| AC004775.5 | -1.491165658 | 0.487895574 | | down |
| SLITRK5 | -1.491133499 | 0.003162909 | 0.015078775 | down |
| CTB-3204.2 | -1.491007568 | 0.016809181 | 0.05436662 | down |
| PCAT7 | -1.49099372 | 6.03E-05 | 0.000622105 | down |
| CECR3 | -1.490807545 | 0.322045902 | | down |
| RP11-14C10.4 | -1.490796003 | 0.238102761 | | down |
| RPS5P7 | -1.490684196 | 0.274292257 | 0.410666097 | down |
| PSCA | -1.490665346 | 0.004825733 | 0.021127719 | down |
| RP11-379C10.4 | -1.490555804 | 0.257843871 | | down |
| SLC6A1 | -1.490421194 | 0.001323721 | 0.007559373 | down |
| RP11-344P13.1 | -1.49033853 | 0.357850186 | | down |
| TMPRSS11CP | -1.490236683 | 0.110309707 | 0.216865547 | down |
| RP11-702H23.2 | -1.490117146 | 0.252824176 | | down |
| AC004221.2 | -1.490114855 | 0.270076634 | | down |
| RPL23AP26 | -1.489479859 | 0.212101769 | 0.342840994 | down |
| CTA-246H3.12 | -1.489330952 | 0.142209728 | | down |
| RP11-187C18.4 | -1.489282162 | 0.000153131 | 0.001341693 | down |
| RP11-542G1.1 | -1.489254813 | 0.197417015 | | down |
| CICP27 | -1.489181414 | 0.016689719 | 0.054079584 | down |
| CTC-344H19.4 | -1.489061775 | 0.004495494 | 0.019985589 | down |
| CICP13 | -1.489027277 | 0.445339902 | | down |
| LINC00689 | -1.48894292 | 0.001272752 | 0.007330075 | down |
| LINC01340 | -1.488840036 | 0.234244037 | | down |
| PRKAG3 | -1.488627687 | 0.049452798 | 0.121395528 | down |
| AC104088.1 | -1.488605516 | 0.19756519 | 0.32624209 | down |
| SPX | -1.488421336 | 0.0018587 | 0.009905118 | down |
| RP13-329D4.3 | -1.488396308 | 0.610867378 | | down |
| TMPRSS11E | -1.488341505 | 0.342631605 | | down |
| RP11-296E7.1 | -1.488268682 | 0.109079109 | 0.215207556 | down |

| | | | | |
|---|---|---|---|---|
| RBMXL3 | -1.488002929 | 0.610739214 | | down |
| RP11-130L8.2 | -1.487971145 | 0.000527357 | 0.003633093 | down |
| PTPN5 | -1.487920093 | 0.005297383 | 0.022689856 | down |
| NA | -1.487909409 | 0.610566537 | | down |
| RP11-677I18.4 | -1.487909409 | 0.610566537 | | down |
| COX8C | -1.48781692 | 0.446263382 | | down |
| RP11-73M7.6 | -1.487804146 | 0.003760054 | 0.017360851 | down |
| RC3H1-IT1 | -1.487754349 | 0.288773717 | | down |
| snoU13 | -1.487706145 | 0.489902344 | | down |
| NA | -1.48760207 | 0.380267014 | | down |
| SLC22A18AS | -1.487540833 | 7.61E-06 | 0.000112765 | down |
| SCAND1 | -1.487227913 | 1.72E-05 | 0.000221198 | down |
| ZNF560 | -1.487216391 | 0.221955809 | | down |
| PRNCR1 | -1.487047756 | 0.02548558 | 0.07431707 | down |
| RP11-310E22.4 | -1.486702414 | 0.168119909 | | down |
| RP11-74E22.8 | -1.486628854 | 0.000123931 | 0.001132245 | down |
| KARSP2 | -1.486547607 | 0.10118008 | | down |
| FAM53B-AS1 | -1.486509926 | 0.037957543 | 0.09989457 | down |
| AF038458.3 | -1.486367555 | 0.334827511 | | down |
| ANO7 | -1.485955059 | 0.000149431 | 0.001316572 | down |
| RP11-434I12.3 | -1.485905856 | 0.536257405 | | down |
| AC009312.1 | -1.485717212 | 0.071248944 | 0.158316935 | down |
| CTD-2616J11.16 | -1.485334626 | 0.047433485 | 0.117670522 | down |
| HCG24 | -1.48520542 | 0.337595341 | | down |
| CTD-2036J7.1 | -1.485186541 | 0.538898428 | | down |
| CHEK2P2 | -1.485172819 | 0.364632524 | | down |
| NA | -1.484714675 | 0.611655962 | | down |
| PDE9A | -1.484704466 | 1.67E-08 | 6.17E-07 | down |
| RP11-244B22.10 | -1.484663809 | 0.611348229 | | down |
| NA | -1.484630395 | 0.536648089 | | down |
| RN7SKP117 | -1.484599444 | 0.611779734 | | down |
| SLC6A11 | -1.484501064 | 0.07563922 | 0.165297115 | down |
| NA | -1.484448935 | 0.013904903 | 0.047140477 | down |
| RP11-844P9.5 | -1.484410767 | 0.409253868 | | down |
| RP11-593F23.1 | -1.484183669 | 0.039474029 | 0.102920916 | down |
| HAP1 | -1.483617014 | 2.93E-05 | 0.000343382 | down |
| RP11-1017G21.6 | -1.483387029 | 4.50E-05 | 0.00048987 | down |
| RP11-758M2.1 | -1.483345654 | 0.204469303 | 0.33409886 | down |
| Y_RNA | -1.48322548 | 0.58849992 | | down |
| MFSD2A | -1.483042474 | 1.13E-06 | 2.26E-05 | down |
| NA | -1.482890414 | 0.181870239 | 0.307404189 | down |
| DNAH10 | -1.48258844 | 0.000224828 | 0.001843037 | down |
| NA | -1.482583241 | 0.001026455 | 0.006196264 | down |
| FOXR1 | -1.482513798 | 0.489359009 | | down |
| RP11-1081M5.2 | -1.482499853 | 0.380599522 | | down |
| RP11-381P6.1 | -1.482073332 | 0.095282408 | 0.195223549 | down |
| RP11-540K16.1 | -1.482021255 | 0.215600538 | | down |
| CTB-186G2.4 | -1.482009727 | 0.179024753 | | down |
| RP11-89K22.1 | -1.481983073 | 0.581672308 | | down |
| AC005307.5 | -1.481933639 | 0.013981295 | 0.047330032 | down |
| RP11-255M2.3 | -1.481862395 | 0.000188228 | 0.001591504 | down |

| | | | | |
|---|---|---|---|---|
| RP1-45N11.1 | -1.481837592 | 0.021582784 | 0.065670767 | down |
| NA | -1.481790989 | 0.209766008 | | down |
| LINC00499 | -1.48171249 | 0.276177701 | 0.412461244 | down |
| DAPK2 | -1.481590539 | 4.15E-14 | 6.63E-12 | down |
| RP13-923023.6 | -1.48141758 | 0.301638006 | | down |
| SMC2-AS1 | -1.481292668 | 0.003516629 | 0.016444139 | down |
| RP11-557N21.1 | -1.481070877 | 0.006695967 | 0.027103857 | down |
| LINC00854 | -1.481041908 | 0.000359664 | 0.002692016 | down |
| RP11-114M5.1 | -1.48103515 | 0.189818783 | 0.317075254 | down |
| MED28P7 | -1.481020812 | 0.44749009 | | down |
| Metazoa_SRP | -1.480741946 | 0.612664377 | | down |
| AC110926.4 | -1.480671628 | 0.354408883 | 0.493021787 | down |
| RP11-706D8.3 | -1.480523104 | 0.282210448 | | down |
| AP000797.4 | -1.480316573 | 0.41650404 | | down |
| SCGB1A1 | -1.480314294 | 0.30533694 | 0.443531585 | down |
| KRT18P22 | -1.480243315 | 0.405126603 | | down |
| UGT2B17 | -1.4802291 | 0.007473561 | 0.029412583 | down |
| LINC01337 | -1.48000517 | 0.014045159 | 0.047503404 | down |
| RNU6-407P | -1.479992378 | 0.336256472 | | down |
| CCDC183 | -1.479932478 | 3.53E-08 | 1.16E-06 | down |
| AC008088.4 | -1.479878786 | 0.051885296 | 0.125551051 | down |
| LINC01284 | -1.479842792 | 0.022314751 | 0.067271159 | down |
| NA | -1.479805532 | 0.612932526 | | down |
| RP11-787P24.4 | -1.479763483 | 0.578715641 | | down |
| AC005776.1 | -1.479641549 | 0.240506844 | 0.374276714 | down |
| HIST1H2AB | -1.479603129 | 0.009295204 | 0.034682701 | down |
| LINC00602 | -1.479543592 | 0.453417034 | | down |
| MTND6P22 | -1.479304891 | 0.016900966 | 0.054587468 | down |
| ASB11 | -1.479016228 | 0.090588464 | 0.188387888 | down |
| HOXB5 | -1.478838644 | 5.95E-07 | 1.31E-05 | down |
| NA | -1.478489492 | 0.000840177 | 0.005278601 | down |
| GCOM1 | -1.478429203 | 2.05E-07 | 5.26E-06 | down |
| AC019080.1 | -1.478092536 | 0.491995003 | | down |
| RP11-158M2.5 | -1.47802683 | 0.034122395 | 0.092341312 | down |
| NCR1 | -1.477835343 | 0.000249452 | 0.002008723 | down |
| CTC-499J9.1 | -1.477820322 | 0.448330753 | | down |
| RP1-148H17.1 | -1.477420196 | 0.002536745 | 0.012712924 | down |
| RP11-330C7.4 | -1.477288001 | 0.460055433 | 0.591944558 | down |
| RP11-363N22.3 | -1.47711613 | 0.012409696 | 0.043235889 | down |
| RPL7P22 | -1.476750919 | 0.541769603 | | down |
| SLC16A9 | -1.476663911 | 1.34E-05 | 0.000180099 | down |
| RP5-1039K5.12 | -1.476651845 | 0.000274538 | 0.00217167 | down |
| AHSG | -1.47661854 | 0.26150778 | | down |
| POLR2LP1 | -1.476438922 | 0.400239082 | | down |
| RP11-314N13.9 | -1.476334129 | 0.004137203 | 0.018733703 | down |
| UQCRHP2 | -1.476261428 | 0.155601314 | | down |
| NA | -1.476204577 | 0.163483565 | 0.285730089 | down |
| AC133785.1 | -1.475547348 | 0.238823724 | 0.372274565 | down |
| RP11-19P22.5 | -1.475220558 | 0.613818999 | | down |
| LINC00269 | -1.475154013 | 0.242229138 | | down |
| UBE2CP2 | -1.475140273 | 0.158170467 | | down |

| | | | | |
|---|---|---|---|---|
| RP11-1258F18.1 | -1.47513024 | 0.429275689 | 0.564575328 | down |
| HMGB2P1 | -1.47479652 | 0.080355688 | | down |
| RP11-146I2.1 | -1.474696153 | 0.012356661 | 0.043117716 | down |
| LINC01097 | -1.474655115 | 0.448906639 | | down |
| AC099552.3 | -1.474417002 | 0.6141361 | | down |
| RP13-585F24.1 | -1.474328482 | 0.001729634 | 0.009359712 | down |
| HNF4GP1 | -1.474238348 | 0.614179166 | | down |
| RP11-274B21.5 | -1.474233678 | 0.42338054 | | down |
| CTD-2616J11.10 | -1.474166558 | 0.015016888 | 0.049989026 | down |
| RP11-349G13.2 | -1.474027941 | 0.587610127 | | down |
| AC005808.3 | -1.474004513 | 0.614328765 | | down |
| ST14 | -1.473623322 | 1.04E-05 | 0.000145927 | down |
| RP11-586K2.1 | -1.473603072 | 0.110586346 | 0.217196587 | down |
| RP11-47909.3 | -1.473505716 | 0.0567954 | 0.134232301 | down |
| RP11-893F2.5 | -1.473357239 | 0.212342258 | 0.343100506 | down |
| RP3-325F22.5 | -1.473190746 | 1.97E-05 | 0.000247405 | down |
| HOOK2 | -1.473021419 | 2.95E-14 | 4.82E-12 | down |
| POTED | -1.472913695 | 0.614591468 | | down |
| RP3-400B16.1 | -1.472754875 | 0.013969732 | 0.047316503 | down |
| GNAQP1 | -1.472684393 | 0.542395052 | | down |
| LNX1-AS1 | -1.472631356 | 0.449065204 | | down |
| RP11-296I10.5 | -1.472479299 | 0.207646716 | 0.337802574 | down |
| SCN8A | -1.472447084 | 6.74E-05 | 0.000681355 | down |
| MCIDAS | -1.472249888 | 0.120696504 | 0.231071545 | down |
| NA | -1.472154168 | 0.506350629 | 0.632675943 | down |
| CTD-2522E6.4 | -1.472093303 | 0.223136791 | 0.355204818 | down |
| RP11-571E6.3 | -1.4720038 | 0.614768417 | | down |
| RP11-671J11.5 | -1.471985643 | 0.042349117 | | down |
| RP11-1080G15.1 | -1.471803553 | 0.098138239 | 0.199314896 | down |
| ATP5A1P2 | -1.471769502 | 0.051415271 | 0.124835978 | down |
| RP11-380D15.2 | -1.471760939 | 0.61482694 | | down |
| RP11-240G22.5 | -1.471757363 | 0.167813686 | 0.291113482 | down |
| GPR32P1 | -1.471735775 | 0.496226668 | | down |
| MEMO1 | -1.471725772 | 2.54E-07 | 6.31E-06 | down |
| AP001627.1 | -1.47166446 | 0.004780892 | 0.020975047 | down |
| SNRPFP4 | -1.471626847 | 0.338316732 | 0.476731293 | down |
| SUPT20HL2 | -1.47159204 | 0.328463868 | | down |
| RNU6-645P | -1.471549162 | 0.543053953 | | down |
| USH1C | -1.471458646 | 2.77E-07 | 6.82E-06 | down |
| Metazoa_SRP | -1.47120304 | 0.614659041 | | down |
| AC093326.3 | -1.471072942 | 0.203109483 | 0.33254612 | down |
| CTB-180A7.6 | -1.470705651 | 0.103009869 | 0.206225662 | down |
| C6orf201 | -1.470603224 | 0.048920803 | 0.120443876 | down |
| RP5-1172N10.2 | -1.470599824 | 0.035889252 | 0.09577693 | down |
| SLC29A4 | -1.47056794 | 1.23E-08 | 4.79E-07 | down |
| RP11-311H10.7 | -1.470322796 | 0.537010968 | | down |
| RP11-132J14.3 | -1.470284626 | 0.332676822 | 0.471037393 | down |
| KRTAP4-6 | -1.470202132 | 0.494377579 | | down |
| MTND4P26 | -1.470177016 | 0.302036041 | | down |
| KIAA1211 | -1.470154692 | 0.000560224 | 0.003814843 | down |
| EHHADH | -1.47009539 | 6.30E-09 | 2.61E-07 | down |

| | | | | |
|---|---|---|---|---|
| BDH2P1 | -1.470010172 | 0.173583823 | | down |
| OR2A7 | -1.469995682 | 0.000387377 | 0.002844865 | down |
| NA | -1.469947004 | 0.20142801 | 0.330545525 | down |
| NLGN1 | -1.469753927 | 0.005897718 | 0.024630393 | down |
| RAB28P4 | -1.469519342 | 0.615367219 | | down |
| ZSWIM5P1 | -1.469518602 | 0.615367397 | | down |
| WDR63 | -1.469513849 | 0.015981867 | 0.052395347 | down |
| MIR4307HG | -1.46946288 | 0.19724817 | 0.325826041 | down |
| NA | -1.46945451 | 0.175673492 | 0.300292124 | down |
| RP11-405O10.2 | -1.469447539 | 0.242855302 | | down |
| WSCD1 | -1.469149902 | 3.57E-09 | 1.59E-07 | down |
| PRAMEF19 | -1.469132033 | 0.537768187 | | down |
| CTB-31O20.8 | -1.46910167 | 0.030180119 | 0.084182666 | down |
| DDX11L10 | -1.468929425 | 0.000998993 | 0.006057267 | down |
| RP11-318I4.2 | -1.46889589 | 0.319682504 | 0.458450868 | down |
| CLEC18B | -1.468848315 | 0.000545773 | 0.00373598 | down |
| OR51N1P | -1.468828354 | 0.327196991 | 0.465670602 | down |
| CABP1 | -1.468795267 | 2.13E-06 | 3.91E-05 | down |
| TTLL2 | -1.468582514 | 0.005907081 | 0.024652791 | down |
| TRPC5 | -1.468225979 | 0.541713365 | | down |
| RP11-794M8.1 | -1.467946222 | 0.418714268 | | down |
| MGAT4D | -1.467925138 | 0.59735373 | | down |
| NECAB2 | -1.467615417 | 0.000965702 | 0.005888688 | down |
| DCAF8L2 | -1.46746222 | 0.615755245 | | down |
| RP11-737O24.3 | -1.467363506 | 0.200566548 | | down |
| GRTP1-AS1 | -1.467339098 | 0.024861791 | 0.072994004 | down |
| CNTN5 | -1.467271084 | 0.232398382 | 0.365062062 | down |
| RP11-831A10.1 | -1.467238921 | 0.270602803 | 0.406621517 | down |
| SRD5A3P1 | -1.467168654 | 0.615934013 | | down |
| ITGA9-AS1 | -1.467161245 | 4.27E-08 | 1.37E-06 | down |
| STXBP5L | -1.466723062 | 0.03304361 | 0.090062799 | down |
| Y_RNA | -1.466406126 | 0.410421775 | | down |
| CTRC | -1.466212696 | 0.00069944 | 0.004563295 | down |
| CTD-2013N17.1 | -1.466028652 | 0.026443324 | 0.076430717 | down |
| MTND5P12 | -1.465869653 | 0.407375927 | | down |
| KB-1732A1.1 | -1.465807773 | 1.10E-05 | 0.00015199 | down |
| RP11-392E22.3 | -1.465723079 | 0.616233382 | | down |
| RP4-668J24.2 | -1.465614976 | 0.000491214 | 0.003428116 | down |
| THUMPD1P1 | -1.465604297 | 0.397085538 | 0.534069205 | down |
| RIIAD1 | -1.465541216 | 0.003785287 | 0.017439835 | down |
| RP5-828H9.1 | -1.465361724 | 0.00599288 | 0.024930778 | down |
| ISX | -1.465222448 | 0.000154888 | 0.001353927 | down |
| SPDYE2 | -1.465174352 | 0.006284707 | 0.025826526 | down |
| RP11-66N24.4 | -1.465150245 | 0.00054017 | 0.003705484 | down |
| HYKK | -1.465067092 | 2.35E-10 | 1.44E-08 | down |
| IGHV3-16 | -1.464858675 | 0.543835191 | | down |
| RP11-612B6.1 | -1.464840821 | 0.046751835 | 0.116362531 | down |
| RP11-744D14.2 | -1.464818737 | 0.248220558 | 0.382555579 | down |
| ANKRD10-IT1 | -1.464726593 | 0.000472053 | 0.003321758 | down |
| RP13-204A15.5 | -1.464334774 | 0.54376487 | | down |
| RP11-204E9.1 | -1.464180481 | 0.414312633 | | down |

| | | | | |
|---|---|---|---|---|
| NA | -1.464071284 | 0.499406826 | | down |
| RP11-393J16.4 | -1.464032905 | 0.61639095 | | down |
| AC005355.1 | -1.463906939 | 0.544639087 | | down |
| AGFG2 | -1.46368901 | 8.41E-10 | 4.39E-08 | down |
| RP11-519G16.3 | -1.463674617 | 0.000220349 | 0.001811267 | down |
| GLRA2 | -1.463632892 | 0.031798178 | 0.087517813 | down |
| SRGAP3 | -1.463483659 | 1.13E-08 | 4.45E-07 | down |
| FLJ46066 | -1.463407155 | 0.616792587 | | down |
| RP11-321L2.2 | -1.463340895 | 0.540249726 | | down |
| TNNT2 | -1.463071979 | 0.040728849 | 0.105304484 | down |
| CHRFAM7A | -1.463029736 | 0.007348577 | 0.02902717 | down |
| ANKRD18A | -1.462955049 | 0.001048615 | 0.006300916 | down |
| TBX20 | -1.462635876 | 0.381748573 | | down |
| RP11-514F3.5 | -1.462570582 | 0.050227029 | 0.122734516 | down |
| LARP4P | -1.462194613 | 0.538854327 | | down |
| KRTCAP3 | -1.462038193 | 2.13E-05 | 0.000263762 | down |
| RNU7-128P | -1.461841364 | 0.242825396 | 0.376733601 | down |
| AC141930.2 | -1.461766044 | 0.070365607 | 0.156946124 | down |
| KRT17P2 | -1.461539026 | 0.124380281 | 0.235856777 | down |
| GRIN1 | -1.461502893 | 2.37E-05 | 0.000287616 | down |
| RP11-196H14.4 | -1.461287539 | 0.617304525 | | down |
| Y_RNA | -1.461266697 | 0.373620454 | | down |
| RN7SKP247 | -1.461249921 | 0.227601318 | 0.359783963 | down |
| C9orf106 | -1.461172275 | 0.023131231 | 0.069087851 | down |
| AF131216.5 | -1.461164027 | 0.215869129 | | down |
| ADCY2 | -1.460701644 | 7.29E-05 | 0.000725219 | down |
| TH | -1.460686357 | 0.01685477 | 0.054467538 | down |
| Metazoa_SRP | -1.46068215 | 0.458365653 | | down |
| MIRLET7BHG | -1.460650319 | 4.91E-05 | 0.000527728 | down |
| RP11-57C19.2 | -1.460238174 | 0.61760633 | | down |
| RN7SL326P | -1.460095147 | 0.617592597 | | down |
| RP11-846F4.6 | -1.46008305 | 0.597253248 | | down |
| HYDIN2 | -1.459906793 | 0.001169392 | 0.006867318 | down |
| CLDN4 | -1.459807679 | 6.83E-05 | 0.000688204 | down |
| SSMEM1 | -1.459459605 | 0.324611441 | 0.463056723 | down |
| ID2-AS1 | -1.459458826 | 1.21E-05 | 0.000164862 | down |
| RP11-510I21.1 | -1.459445865 | 0.59569508 | | down |
| AC068286.1 | -1.459238721 | 0.598726874 | | down |
| RP11-204N11.1 | -1.459221636 | 0.499916521 | | down |
| BMPR1APS1 | -1.459014592 | 0.617853701 | | down |
| RP11-757G1.6 | -1.458617714 | 0.000555375 | 0.003791338 | down |
| NA | -1.458215915 | 0.085418157 | 0.1807383 | down |
| NA | -1.458101387 | 0.592494575 | | down |
| RP5-1148A21.3 | -1.458056749 | 7.04E-05 | 0.000705534 | down |
| RP1-15D23.2 | -1.458012601 | 0.104775449 | 0.208772621 | down |
| RP11-754B17.1 | -1.457790015 | 0.018389975 | 0.058330702 | down |
| SUMO1P1 | -1.45766848 | 0.597916003 | | down |
| RP11-10N23.5 | -1.45761311 | 0.091093171 | | down |
| RP11-225B17.1 | -1.457582394 | 0.421051924 | 0.556683858 | down |
| AC010980.2 | -1.457532657 | 0.006757287 | 0.027276983 | down |
| CATIP | -1.457399592 | 0.014681811 | 0.049125515 | down |

| | | | | |
|---|---|---|---|---|
| AC003080.4 | -1.457136634 | 0.595247856 | | down |
| RP11-174G17.3 | -1.456838353 | 0.047117828 | 0.117089165 | down |
| TISP43 | -1.456686324 | 0.145830823 | 0.263729685 | down |
| GCNT1P5 | -1.456606813 | 0.618524191 | | down |
| RP11-14I17.2 | -1.456606813 | 0.618524191 | | down |
| NA | -1.456606813 | 0.618524191 | | down |
| GAPDHP46 | -1.456606464 | 0.618524276 | | down |
| IGKV2D-19 | -1.456606464 | 0.618524276 | | down |
| RPL35P3 | -1.456606464 | 0.618524276 | | down |
| RP11-3L10.2 | -1.456606464 | 0.618524276 | | down |
| RCC2P3 | -1.456606464 | 0.618524276 | | down |
| RP11-17A4.4 | -1.456606464 | 0.618524276 | | down |
| RP11-738O11.12 | -1.456606464 | 0.618524276 | | down |
| RP11-430C1.1 | -1.456606464 | 0.618524276 | | down |
| KBTBD11-OT1 | -1.456460281 | 0.000733169 | 0.004740121 | down |
| RP11-798K23.4 | -1.456433459 | 0.498338461 | | down |
| NA | -1.456376441 | 0.551374596 | | down |
| CTD-2385L22.1 | -1.456269083 | 0.055422789 | 0.131755767 | down |
| C17orf64 | -1.456055385 | 0.037892332 | 0.099766647 | down |
| KRT8P3 | -1.455935764 | 4.89E-06 | 7.79E-05 | down |
| HNRNPA1P32 | -1.455693376 | 0.618744772 | | down |
| RN7SL305P | -1.455693376 | 0.618744772 | | down |
| CITF22-62D4.1 | -1.455433369 | 0.293223382 | 0.43053481 | down |
| ZNF157 | -1.455414639 | 0.034863429 | 0.09378882 | down |
| RP11-304F15.4 | -1.455324762 | 0.549419041 | | down |
| RP11-84C10.4 | -1.455118287 | 0.040572339 | 0.105000892 | down |
| RP13-616I3.1 | -1.455068965 | 1.96E-05 | 0.000246104 | down |
| ZSCAN5B | -1.455066152 | 0.29261384 | | down |
| RP11-342A1.1 | -1.455026653 | 0.406631687 | | down |
| TMSB4XP1 | -1.455023528 | 0.013175159 | | down |
| RNU6-1004P | -1.45470492 | 0.326239855 | | down |
| NA | -1.454692247 | 0.618986568 | | down |
| RP11-93K22.1 | -1.454692247 | 0.618986568 | | down |
| RNU6-84P | -1.454692247 | 0.618986568 | | down |
| RPL5P19 | -1.454692247 | 0.618986568 | | down |
| C7orf66 | -1.454412701 | 0.414340266 | 0.550886993 | down |
| NA | -1.454400499 | 0.023569523 | 0.070097888 | down |
| BECN2 | -1.454274868 | 0.618999562 | | down |
| SCN5A | -1.454246924 | 0.001741683 | 0.009404589 | down |
| RP11-72K17.1 | -1.454241155 | 0.618823592 | | down |
| MIR206 | -1.454187583 | 0.619108472 | | down |
| RNU6-957P | -1.454187583 | 0.619108472 | | down |
| NA | -1.454187583 | 0.619108472 | | down |
| RP11-383B24.3 | -1.454187583 | 0.619108472 | | down |
| RN7SL865P | -1.454187583 | 0.619108472 | | down |
| RP11-240G22.1 | -1.454187583 | 0.619108472 | | down |
| RP11-536C10.4 | -1.454187583 | 0.619108472 | | down |
| POTEH-AS1 | -1.454187583 | 0.619108472 | | down |
| OLAH | -1.454088037 | 0.027281338 | 0.078269281 | down |
| RP11-49P4.7 | -1.453931447 | 0.457399482 | | down |
| SULT1B1 | -1.453698238 | 9.51E-06 | 0.000135404 | down |

| | | | | |
|---|---|---|---|---|
| U6 | -1.453590951 | 0.619252605 | | down |
| NA | -1.453590951 | 0.619252605 | | down |
| NA | -1.453590951 | 0.619252605 | | down |
| FTH1P6 | -1.453590951 | 0.619252605 | | down |
| MTND1P27 | -1.453590951 | 0.619252605 | | down |
| RN7SKP42 | -1.453590951 | 0.619252605 | | down |
| NA | -1.453590951 | 0.619252605 | | down |
| RP11-638O6.1 | -1.453590951 | 0.619252605 | | down |
| USP17L4 | -1.453590951 | 0.619252605 | | down |
| SPATA31A3 | -1.453590951 | 0.619252605 | | down |
| OR56A5 | -1.453590951 | 0.619252605 | | down |
| RP11-56P9.8 | -1.453590951 | 0.619252605 | | down |
| NA | -1.453590951 | 0.619252605 | | down |
| RP11-314D7.3 | -1.453590951 | 0.619252605 | | down |
| RP11-168P6.2 | -1.453590951 | 0.619252605 | | down |
| NA | -1.453590951 | 0.619252605 | | down |
| Y_RNA | -1.453590951 | 0.619252605 | | down |
| MIR485 | -1.453590951 | 0.619252605 | | down |
| KRTAP9-6 | -1.453590951 | 0.619252605 | | down |
| RP11-15E18.3 | -1.453590951 | 0.619252605 | | down |
| RPS4XP21 | -1.453590951 | 0.619252605 | | down |
| U3 | -1.453590951 | 0.619252605 | | down |
| RP11-151E14.1 | -1.453590951 | 0.619252605 | | down |
| CTD-2311M21.3 | -1.453571755 | 0.077546982 | 0.168266758 | down |
| RP11-74C13.3 | -1.45348844 | 0.548057634 | | down |
| TAS2R6P | -1.453353405 | 0.45713422 | | down |
| AC105393.2 | -1.453271081 | 0.619329883 | | down |
| AC007557.4 | -1.453271081 | 0.619329883 | | down |
| RP11-19O2.1 | -1.453271081 | 0.619329883 | | down |
| RP11-9G10.1 | -1.453271081 | 0.619329883 | | down |
| R3HDM2P2 | -1.453271081 | 0.619329883 | | down |
| TPM3P4 | -1.453271081 | 0.619329883 | | down |
| NDUFB9P3 | -1.453271081 | 0.619329883 | | down |
| RP11-983C2.1 | -1.453271081 | 0.619329883 | | down |
| snoU109 | -1.453271081 | 0.619329883 | | down |
| CTD-2008P7.6 | -1.453271081 | 0.619329883 | | down |
| RP11-805L22.3 | -1.453271081 | 0.619329883 | | down |
| RNA5SP448 | -1.453271081 | 0.619329883 | | down |
| CTC-457E21.7 | -1.453271081 | 0.619329883 | | down |
| SDC4P | -1.453271081 | 0.619329883 | | down |
| NA | -1.453262388 | 0.149701554 | 0.268614675 | down |
| CTD-2410N18.4 | -1.453137536 | 0.114694434 | 0.222774734 | down |
| RP11-1016B18.1 | -1.453111384 | 0.456517888 | | down |
| SLCO1A2 | -1.452982826 | 0.044291155 | 0.111949472 | down |
| LINC01014 | -1.452949323 | 0.130559514 | | down |
| HOXA-AS3 | -1.452932227 | 0.000114695 | 0.001063223 | down |
| RP11-109L13.5 | -1.452875203 | 0.619425532 | | down |
| RP11-799D4.3 | -1.452875203 | 0.619425532 | | down |
| ABHD14A-ACY1 | -1.452861218 | 0.211769276 | | down |
| HLA-V | -1.452779928 | 0.001419835 | 0.007985544 | down |
| AP005135.2 | -1.45265467 | 0.619478817 | | down |

| | | | | |
|---|---|---|---|---|
| EPHX2 | -1.452564733 | 3.16E-11 | 2.51E-09 | down |
| WNK3 | -1.4524644 | 0.00049702 | 0.003458191 | down |
| RP3-352A20.1 | -1.45245438 | 0.619527214 | | down |
| RP11-262A16.1 | -1.452437268 | 0.001658331 | 0.009031228 | down |
| UGT1A2P | -1.452429127 | 0.605692316 | | down |
| FEZF2 | -1.452373453 | 0.619546769 | | down |
| RPL10P8 | -1.452361884 | 0.601617325 | | down |
| RUNDC3B | -1.452076881 | 6.99E-06 | 0.000104889 | down |
| ACTL9 | -1.45202725 | 0.619630427 | | down |
| AC012513.4 | -1.451993119 | 0.250582229 | | down |
| MSANTD2P1 | -1.451968268 | 0.498234135 | | down |
| AC005752.10 | -1.45173267 | 0.103166816 | 0.206429824 | down |
| POM121L4P | -1.45152537 | 0.054482361 | | down |
| ALMS1-IT1 | -1.451276774 | 0.000269582 | 0.002140978 | down |
| KRT8P36 | -1.451237595 | 0.004917988 | 0.021418247 | down |
| RNU2-37P | -1.45103756 | 0.598112829 | | down |
| AP000479.1 | -1.450925319 | 0.496459047 | | down |
| WNT11 | -1.450888972 | 0.000428371 | 0.003074018 | down |
| CYP2W1 | -1.450880126 | 0.00125915 | 0.007274981 | down |
| MT1DP | -1.450831398 | 0.002867753 | 0.01399975 | down |
| SLCO4C1 | -1.45062495 | 0.008483863 | 0.032327833 | down |
| NA | -1.450577134 | 0.45360945 | | down |
| B3GNTL1P1 | -1.450524572 | 0.552266051 | | down |
| RP11-141J13.3 | -1.450305692 | 0.501474669 | | down |
| RP11-395C3.1 | -1.450209902 | 0.008362579 | 0.031985363 | down |
| AC004980.10 | -1.45020673 | 0.055115974 | 0.131295933 | down |
| NA | -1.450153118 | 0.018595214 | 0.058795475 | down |
| RP11-286N22.16 | -1.450133794 | 0.228486505 | | down |
| MRPL15P1 | -1.450060563 | 0.376482147 | 0.5146992 | down |
| RP11-76C10.3 | -1.449682826 | 0.361523986 | | down |
| SLFNL1 | -1.449237722 | 0.024183365 | 0.071519345 | down |
| GDAP1L1 | -1.44923448 | 0.008531792 | 0.032467279 | down |
| GRID2 | -1.449227991 | 0.095113516 | 0.19506509 | down |
| GLUD1P7 | -1.449140474 | 0.006161088 | 0.025467901 | down |
| RP11-708L7.7 | -1.449138652 | 0.45692395 | | down |
| MYBPC1 | -1.448874348 | 0.004101197 | 0.018604313 | down |
| RP11-158G18.1 | -1.448795527 | 0.620411446 | | down |
| RP11-166B2.3 | -1.448781788 | 0.002722229 | 0.013445542 | down |
| RP3-380B8.4 | -1.44860253 | 0.216574752 | | down |
| CTC-470E21.1 | -1.448535121 | 0.499784491 | | down |
| FAM83B | -1.448481657 | 1.85E-05 | 0.000234649 | down |
| NA | -1.448307693 | 0.37886845 | | down |
| AC073043.1 | -1.448139414 | 0.016972056 | 0.05476409 | down |
| KB-68A7.2 | -1.448050947 | 0.620551633 | | down |
| LRRIQ4 | -1.447988921 | 0.000842017 | 0.005286848 | down |
| SKOR2 | -1.44790702 | 0.194899417 | 0.323328104 | down |
| RP11-418I22.3 | -1.447805287 | 0.352767347 | | down |
| CAMSAP3 | -1.447564693 | 1.00E-07 | 2.83E-06 | down |
| NA | -1.44743735 | 0.601126525 | | down |
| MT-CYB | -1.447432627 | 0.001513893 | 0.008399522 | down |
| RP11-68L1.2 | -1.447386021 | 0.620666619 | | down |

| | | | | |
|---|---|---|---|---|
| RP11-44N11.1 | -1.447132503 | 0.022707701 | 0.068188923 | down |
| MIR4514 | -1.447092492 | 0.620555344 | | down |
| FLJ42969 | -1.447010917 | 0.121271616 | 0.231788564 | down |
| RP11-973N13.2 | -1.446866066 | 0.550705265 | | down |
| MEI4 | -1.446800569 | 0.27762817 | | down |
| PRSS48 | -1.446755046 | 0.257558444 | 0.392640671 | down |
| RNY1P10 | -1.446559697 | 0.237936194 | 0.371237797 | down |
| CCDC87 | -1.446524023 | 0.012426576 | 0.04327623 | down |
| CLUHP6 | -1.446408715 | 0.620988456 | | down |
| RNA5SP379 | -1.446347839 | 0.366571589 | | down |
| AP2S1 | -1.44613683 | 6.15E-06 | 9.45E-05 | down |
| TCP10L | -1.446113742 | 0.099622627 | 0.201417376 | down |
| RPL23P11 | -1.445915625 | 0.621107689 | | down |
| APOA1-AS | -1.445694827 | 0.062357902 | 0.143804176 | down |
| RP11-375O18.2 | -1.445462299 | 0.135108866 | 0.24985909 | down |
| RP11-141A19.2 | -1.445246532 | 0.621269497 | | down |
| DPPA4 | -1.445133246 | 0.01477711 | 0.049384149 | down |
| RP11-395B7.2 | -1.444930583 | 0.000300467 | 0.002333218 | down |
| NA | -1.444614649 | 0.064197006 | 0.14671648 | down |
| PLCB3 | -1.444530815 | 9.15E-11 | 6.29E-09 | down |
| RP11-566K19.12 | -1.44441476 | 0.621330774 | | down |
| FAM205A | -1.444398965 | 0.309744516 | | down |
| RP11-14C10.6 | -1.444364258 | 0.135163005 | 0.249932935 | down |
| RP11-44M6.1 | -1.44414607 | 0.262629543 | 0.397924353 | down |
| JAKMIP3 | -1.444123293 | 0.004124872 | 0.018697593 | down |
| FREM1 | -1.444082523 | 0.000407626 | 0.00295165 | down |
| OR4F17 | -1.444041997 | 0.621476545 | | down |
| RP11-1166P10.7 | -1.443936411 | 0.621586378 | | down |
| CES1P1 | -1.443864049 | 0.008774674 | 0.03318141 | down |
| RP11-304C12.3 | -1.443806724 | 0.621578758 | | down |
| SLC25A30-AS1 | -1.443740613 | 0.011976327 | 0.042147917 | down |
| NXF2 | -1.443544519 | 0.335735047 | 0.473917459 | down |
| RPS26P15 | -1.443211115 | 0.028495063 | 0.080894423 | down |
| MYCBPAP | -1.443030699 | 0.000171107 | 0.001470045 | down |
| HDAC1P2 | -1.442909107 | 0.391076977 | | down |
| RP11-489O18.1 | -1.442753309 | 0.002753536 | 0.013564065 | down |
| TMEM31 | -1.442634774 | 0.084580024 | | down |
| PARD6B | -1.442568134 | 9.24E-08 | 2.65E-06 | down |
| YBX1P4 | -1.442508041 | 0.107855401 | 0.213507786 | down |
| ZNF713 | -1.442490857 | 3.74E-06 | 6.22E-05 | down |
| CNDP1 | -1.442188285 | 0.011065199 | 0.039633148 | down |
| C17orf74 | -1.44205585 | 0.507773121 | | down |
| NA | -1.442042649 | 0.622005889 | | down |
| NA | -1.442041997 | 0.622006047 | | down |
| Y_RNA | -1.441869112 | 0.500148682 | | down |
| LRRC52 | -1.441768518 | 0.240329452 | | down |
| CELA1 | -1.441712008 | 0.012925159 | 0.044547834 | down |
| RP11-673E1.1 | -1.441680854 | 0.002689941 | 0.013309713 | down |
| RP11-465C12.1 | -1.441642194 | 0.135179234 | 0.249932935 | down |
| GJA3 | -1.44143928 | 0.035461361 | 0.094904787 | down |
| RP11-879F14.2 | -1.441424491 | 0.008577633 | 0.032600432 | down |

| | | | | |
|---|---|---|---|---|
| RP11-212P7.1 | -1.441264534 | 0.001766269 | 0.009505925 | down |
| RP11-505P4.7 | -1.44114254 | 0.167132328 | | down |
| RP11-901H12.1 | -1.441129089 | 0.593637971 | | down |
| STPG2 | -1.441044155 | 0.045767133 | 0.114604746 | down |
| NA | -1.44103617 | 0.256021943 | 0.391052 | down |
| RP3-340N1.2 | -1.441028906 | 0.033975802 | 0.092069 | down |
| RNU6-649P | -1.440979187 | 0.622163645 | | down |
| RNU6-1064P | -1.44094313 | 0.216033263 | 0.347110188 | down |
| LINC01362 | -1.440914247 | 0.459716675 | | down |
| RN7SL16P | -1.440787403 | 0.460641516 | | down |
| SH2D7 | -1.440605337 | 0.001344809 | 0.007657969 | down |
| CFAP221 | -1.440565236 | 0.006073495 | 0.025182457 | down |
| NA | -1.440501758 | 0.622334259 | | down |
| NA | -1.440501758 | 0.622334259 | | down |
| CTC-281F24.5 | -1.440405034 | 0.000394555 | 0.002883488 | down |
| CTD-2200P10.1 | -1.440375645 | 0.622447977 | | down |
| RP11-382E9.1 | -1.440375028 | 0.622448126 | | down |
| EIF3LP1 | -1.440201888 | 0.547584929 | | down |
| PTPRF | -1.439941948 | 2.63E-08 | 8.89E-07 | down |
| RP11-383G6.3 | -1.439915673 | 0.23381741 | | down |
| MB | -1.43975251 | 3.02E-05 | 0.000352167 | down |
| RP11-50D9.1 | -1.439707153 | 0.002873905 | 0.014015304 | down |
| DEFB127 | -1.439530154 | 0.62261445 | | down |
| LINC01400 | -1.439447699 | 0.376485462 | | down |
| KRT90P | -1.439378413 | 0.473123453 | 0.603537748 | down |
| MARVELD2 | -1.439245238 | 1.36E-06 | 2.66E-05 | down |
| MIR4510 | -1.439239952 | 0.622640064 | | down |
| THRSP | -1.439201972 | 0.048552117 | 0.119830757 | down |
| USP12-AS2 | -1.439125711 | 0.035975001 | 0.095988714 | down |
| RP11-403N16.3 | -1.439100642 | 0.421856154 | | down |
| RP11-243M5.5 | -1.438892578 | 0.358414527 | | down |
| SYNDIG1L | -1.438731768 | 0.000822437 | 0.005195396 | down |
| CYP4F22 | -1.438715628 | 0.006861247 | 0.027580855 | down |
| HSP90AA5P | -1.438213734 | 0.108689634 | 0.214706514 | down |
| MAGEB2 | -1.438026742 | 0.320167004 | | down |
| RP11-659E9.2 | -1.437806383 | 0.623032119 | | down |
| RN7SL70P | -1.437697783 | 0.591538713 | | down |
| KIAA0319 | -1.437651229 | 0.000157033 | 0.001369496 | down |
| POU5F1B | -1.43756597 | 0.001270683 | 0.00732471 | down |
| EIF1P2 | -1.437517795 | 0.596113754 | | down |
| SLIT1-AS1 | -1.437479364 | 0.602098036 | | down |
| RP11-762E8.1 | -1.437385243 | 0.307762395 | | down |
| TUBB8P11 | -1.437372216 | 0.45986608 | | down |
| CDV3P1 | -1.436776932 | 0.623237174 | | down |
| AC124997.1 | -1.436702087 | 0.305427661 | | down |
| SCARNA16 | -1.436581358 | 0.623366542 | | down |
| RP11-781M16.2 | -1.436354611 | 0.50357083 | | down |
| AC004691.5 | -1.436330176 | 0.348261703 | | down |
| RP13-392I16.1 | -1.436302623 | 0.451247069 | 0.584163519 | down |
| SMPX | -1.436266326 | 0.331446513 | 0.469782471 | down |
| RNU6-877P | -1.436129035 | 0.54979914 | | down |

| | | | | |
|---|---|---|---|---|
| TPM3P7 | -1.435807135 | 0.462440427 | | down |
| CRYL1 | -1.435720008 | 1.13E-12 | 1.25E-10 | down |
| AC093642.6 | -1.435654181 | 0.623509444 | | down |
| C4orf50 | -1.435549436 | 0.00533084 | 0.022795297 | down |
| AGAP13P | -1.434982262 | 0.033090742 | 0.090166004 | down |
| CTC-308K20.4 | -1.434885737 | 0.168771184 | 0.292217696 | down |
| PHKG1P1 | -1.434713284 | 0.623781877 | | down |
| LINC00254 | -1.434713284 | 0.623781877 | | down |
| CTD-2579N5.3 | -1.434713284 | 0.623781877 | | down |
| NACAP1 | -1.43439825 | 0.00507963 | 0.021958619 | down |
| RP11-266N13.2 | -1.434352118 | 0.086276051 | 0.182015145 | down |
| NKAIN2 | -1.434295407 | 0.013721986 | 0.046625578 | down |
| NA | -1.434166368 | 0.167488699 | | down |
| MIR548I2 | -1.433934646 | 0.232030547 | | down |
| LY6K | -1.43388914 | 0.012920093 | 0.044543078 | down |
| RPL36AP41 | -1.433795255 | 0.028544949 | 0.080997693 | down |
| FER1L5 | -1.433708953 | 0.005060827 | 0.021899394 | down |
| TSSK3 | -1.433543653 | 0.133254245 | 0.247373365 | down |
| RP11-848P1.7 | -1.433144715 | 0.007188059 | 0.028543226 | down |
| ABHD1 | -1.433053537 | 0.000307916 | 0.002381834 | down |
| HCRT | -1.43298096 | 0.14317941 | 0.260336962 | down |
| RP11-377K22.2 | -1.432720316 | 0.09009705 | 0.187612681 | down |
| RP11-847H18.2 | -1.432599311 | 0.282924794 | 0.419451888 | down |
| NA | -1.432593145 | 7.86E-05 | 0.000773894 | down |
| AC020983.5 | -1.432304434 | 0.508053027 | | down |
| DAP3P1 | -1.43225014 | 0.312059395 | | down |
| RP11-21A7A.2 | -1.43215818 | 0.079488239 | | down |
| CLDN8 | -1.43214651 | 0.133052302 | 0.247227618 | down |
| NBEAL1 | -1.43205542 | 8.22E-10 | 4.32E-08 | down |
| GTF2A1L | -1.431932714 | 0.466707131 | | down |
| MAG | -1.431890669 | 0.014892666 | 0.049665362 | down |
| DQX1 | -1.431712136 | 7.40E-07 | 1.58E-05 | down |
| ZDHHC22 | -1.431641691 | 0.080122213 | 0.172447293 | down |
| RP11-27P7.1 | -1.431597333 | 0.273686556 | 0.40998399 | down |
| RP11-2N1.2 | -1.431490614 | 0.296965061 | | down |
| PZP | -1.431483256 | 0.010285053 | 0.037455046 | down |
| PCSK7 | -1.431463908 | 5.38E-07 | 1.20E-05 | down |
| RP1-35C21.1 | -1.431441287 | 0.002048668 | 0.010702516 | down |
| AC002044.3 | -1.431407048 | 0.337652783 | 0.476018968 | down |
| LINC01065 | -1.431294155 | 0.50351442 | | down |
| NA | -1.431185634 | 0.279789467 | | down |
| EFR3B | -1.431180967 | 5.36E-06 | 8.39E-05 | down |
| RP11-57C13.3 | -1.431123659 | 0.598102769 | | down |
| DKK4 | -1.431115136 | 0.230290164 | 0.362679545 | down |
| AF186192.6 | -1.431060909 | 0.170230193 | 0.294066002 | down |
| RP11-170M17.1 | -1.430842933 | 0.238425611 | | down |
| LINC01381 | -1.430681472 | 0.605807615 | | down |
| HLA-DQA2 | -1.43041728 | 0.010426879 | 0.037888868 | down |
| LINC00705 | -1.430410266 | 0.089506331 | 0.186822857 | down |
| AC079154.1 | -1.430206481 | 0.489381958 | 0.617980478 | down |
| OR6M1 | -1.430163748 | 0.551225421 | | down |

| | | | | |
|---|---|---|---|---|
| RP11-320N7.1 | -1.430141839 | 0.037292986 | 0.098568704 | down |
| RP11-306I1.2 | -1.430064407 | 0.138712978 | 0.254671477 | down |
| HSPA8P18 | -1.429699418 | 0.457284242 | | down |
| STRCP1 | -1.42965974 | 5.50E-05 | 0.000577804 | down |
| L22NC03-75H12.2 | -1.429545763 | 0.016596568 | 0.053866754 | down |
| PPM1E | -1.429544543 | 0.010011764 | 0.036708988 | down |
| Y_RNA | -1.429472002 | 0.597119714 | | down |
| MICD | -1.429352123 | 0.188513832 | | down |
| THAP12P1 | -1.429191869 | 0.306400102 | 0.444787624 | down |
| AC011290.4 | -1.42897078 | 0.056302558 | 0.133382277 | down |
| TOP3B | -1.42862167 | 0.072897427 | 0.161038993 | down |
| RP5-930J4.2 | -1.428423705 | 0.625263876 | | down |
| ZNF321P | -1.427980095 | 1.66E-05 | 0.000215022 | down |
| NA | -1.427910039 | 0.605206125 | | down |
| RP11-100L22.1 | -1.427884171 | 0.014808118 | 0.049471267 | down |
| RHOXF1P1 | -1.42786415 | 0.285554332 | | down |
| AC010127.3 | -1.427834826 | 0.095037662 | 0.194968353 | down |
| TTC21B-AS1 | -1.427706243 | 0.154510791 | 0.274348796 | down |
| CTD-3131K8.3 | -1.427659483 | 0.000814303 | 0.005149171 | down |
| MT-TT | -1.427287143 | 0.508218441 | | down |
| PRELID1P1 | -1.426801141 | 0.001415408 | 0.007964887 | down |
| ANGPTL3 | -1.426742358 | 0.260943689 | | down |
| GPR78 | -1.426728688 | 0.104243978 | 0.207961891 | down |
| CTA-246H3.8 | -1.426549416 | 0.083471797 | 0.177658449 | down |
| ZNF564 | -1.426419357 | 0.001325463 | 0.007566449 | down |
| RP11-561B11.1 | -1.426151457 | 0.258712006 | | down |
| RP11-29G8.3 | -1.426144527 | 0.086955129 | 0.182985214 | down |
| RP11-626P14.1 | -1.426087368 | 0.556477986 | | down |
| YBX2 | -1.426073612 | 5.40E-06 | 8.45E-05 | down |
| SPINK14 | -1.426043791 | 0.559182026 | | down |
| RN7SL72P | -1.426035599 | 0.552732849 | | down |
| LINC01290 | -1.425954716 | 0.050610852 | 0.123341373 | down |
| RP11-368M16.3 | -1.425850348 | 0.320907142 | 0.459623699 | down |
| RP4-656G21.1 | -1.425820927 | 0.625939104 | | down |
| RP11-90C4.2 | -1.425695683 | 0.626004857 | | down |
| MIR3648-2 | -1.425485103 | 0.270992517 | | down |
| RP11-388P9.2 | -1.425310576 | 0.271293516 | | down |
| RP11-317F20.3 | -1.425153295 | 0.625945879 | | down |
| DUX4L9 | -1.425075293 | 0.400689083 | | down |
| AP000640.10 | -1.424860023 | 0.0074466 | 0.029333374 | down |
| DSCAM-AS1 | -1.424745248 | 0.596873597 | | down |
| MIR514B | -1.424509396 | 0.462475157 | | down |
| CTD-2192J16.11 | -1.424476484 | 0.02161769 | 0.065750341 | down |
| RP11-547D13.1 | -1.424457912 | 0.003116254 | 0.014927413 | down |
| RP11-104H15.12 | -1.424455063 | 0.626115698 | | down |
| CTC-503J8.4 | -1.424243708 | 0.26465739 | 0.400149295 | down |
| Z82214.3 | -1.424091037 | 0.599801392 | | down |
| LINC01609 | -1.423878748 | 0.604134633 | | down |
| CTD-3064H18.4 | -1.423778343 | 0.081762788 | | down |
| NRG3 | -1.423379582 | 0.052371676 | 0.126371748 | down |
| CTD-2006K23.2 | -1.423348567 | 0.042461131 | 0.108657053 | down |

| | | | | |
|---|---|---|---|---|
| C10orf120 | -1.423331256 | 0.552094246 | | down |
| AFG3L2P1 | -1.423295833 | 0.465498998 | | down |
| C4orf36 | -1.423243732 | 0.136831552 | 0.252082853 | down |
| NA | -1.423185915 | 0.068165895 | 0.153337538 | down |
| SCN2A | -1.423170617 | 0.008072591 | 0.031153701 | down |
| RP11-174G17.2 | -1.423002438 | 0.626623486 | | down |
| RP11-43A1.1 | -1.423002438 | 0.626623486 | | down |
| RP1-293L8.2 | -1.423002438 | 0.626623486 | | down |
| AC104389.16 | -1.423002438 | 0.626623486 | | down |
| HMGN1P5 | -1.422814034 | 0.31161737 | 0.450237197 | down |
| MTCO3P22 | -1.422690292 | 0.463642975 | | down |
| CACNA2D3 | -1.422580591 | 3.04E-05 | 0.000354517 | down |
| PRR26 | -1.422517349 | 5.24E-11 | 3.86E-09 | down |
| CTD-3080P12.3 | -1.422388648 | 0.007335235 | 0.028986713 | down |
| MIR1915 | -1.422208948 | 0.293793942 | | down |
| RP11-383F6.1 | -1.422182239 | 0.226394477 | | down |
| P2RX6 | -1.4221242 | 2.10E-05 | 0.000260796 | down |
| MFAP1P1 | -1.42209668 | 0.137379751 | | down |
| NA | -1.422081005 | 0.01394506 | 0.047249959 | down |
| TMEM45B | -1.421969522 | 5.66E-05 | 0.000592293 | down |
| RP11-112N23.1 | -1.42187148 | 0.506503978 | | down |
| LRRC37A11P | -1.421841763 | 0.046491802 | 0.115888136 | down |
| RP11-420H19.3 | -1.421768876 | 0.626880482 | | down |
| RP11-296E3.2 | -1.421546441 | 0.003447183 | 0.016177154 | down |
| RP11-1275H24.3 | -1.421417693 | 0.000846507 | 0.005310602 | down |
| RP11-549K20.1 | -1.421265099 | 0.229410873 | 0.361844827 | down |
| SLC2A1-AS1 | -1.421114616 | 0.00046203 | 0.003266939 | down |
| RIPK4 | -1.420929092 | 0.000435087 | 0.003111581 | down |
| AFM | -1.420904809 | 0.627167487 | | down |
| NA | -1.42082932 | 0.220471674 | | down |
| NEK5 | -1.420784464 | 0.000422922 | 0.003040832 | down |
| ROPN1 | -1.420558607 | 0.263655147 | | down |
| NA | -1.420486148 | 1.61E-05 | 0.000210986 | down |
| RN7SKP165 | -1.420467406 | 0.509499091 | | down |
| PEPD | -1.419821298 | 2.67E-07 | 6.61E-06 | down |
| HNF4A | -1.419711672 | 1.03E-06 | 2.08E-05 | down |
| RP3-405J10.4 | -1.419452414 | 0.162263844 | | down |
| RNU6-615P | -1.41934068 | 0.627547263 | | down |
| RP11-58A17.4 | -1.41934068 | 0.627547263 | | down |
| RP4-797C5.2 | -1.41929732 | 0.561116661 | | down |
| FAM71D | -1.419076272 | 0.005116729 | 0.02208404 | down |
| LINC00677 | -1.418964039 | 0.219705054 | 0.351219465 | down |
| NA | -1.418849253 | 0.553642807 | | down |
| RP11-500M8.6 | -1.418664997 | 0.562220363 | | down |
| HNRNPA1P59 | -1.418572363 | 0.196296954 | | down |
| CTD-2200A16.1 | -1.41823973 | 0.612468079 | | down |
| RP11-196G11.6 | -1.418224017 | 0.000130638 | 0.001179706 | down |
| PLEKHS1 | -1.417932548 | 0.00014087 | 0.001255132 | down |
| RP11-116N8.1 | -1.417919815 | 0.4312442 | | down |
| RP11-637N19.1 | -1.417869524 | 0.323079122 | | down |
| NA | -1.417857702 | 0.560563752 | | down |

| | | | | |
|---|---|---|---|---|
| RP5-1119A7.17 | -1.417852512 | 0.177473477 | 0.302491243 | down |
| CXADR | -1.417628246 | 3.41E-05 | 0.000387868 | down |
| TRIM72 | -1.417365898 | 0.001884881 | 0.010012265 | down |
| SEC63P2 | -1.417263562 | 0.37487534 | 0.513132932 | down |
| RNY4P25 | -1.417156496 | 0.424520466 | | down |
| CYCSP10 | -1.417124784 | 0.142927863 | | down |
| PRSS38 | -1.417061303 | 0.455969467 | 0.588324958 | down |
| LINC01505 | -1.41683767 | 0.025761719 | 0.07494046 | down |
| CCT5P2 | -1.416675503 | 0.628194603 | | down |
| ZDHHC11B | -1.416501695 | 0.000213733 | 0.001766062 | down |
| EEF1A1P24 | -1.416260448 | 0.020203958 | 0.06259102 | down |
| ANO4 | -1.416234549 | 0.017066675 | 0.054980826 | down |
| RP11-529H20.3 | -1.416160162 | 0.140935886 | | down |
| RP5-1016A21.1 | -1.416086569 | 0.223390255 | 0.355446288 | down |
| RN7SL574P | -1.415990383 | 0.080178079 | 0.172504468 | down |
| LGALS4 | -1.415941577 | 1.81E-05 | 0.000230569 | down |
| SLITRK4 | -1.41585245 | 0.000324894 | 0.002486909 | down |
| RP11-1099M24.6 | -1.41574347 | 0.000114415 | 0.001060953 | down |
| RP11-735A19.3 | -1.415661798 | 0.28702289 | | down |
| DEPDC4 | -1.415606516 | 0.0019139 | 0.010125098 | down |
| RP11-672A2.3 | -1.415545775 | 0.554567084 | | down |
| RP11-401I19.2 | -1.415268726 | 0.62822516 | | down |
| E2F3P2 | -1.415195528 | 0.628554194 | | down |
| SNORD38 | -1.41506666 | 0.609403236 | | down |
| CACNA1A | -1.414922203 | 1.36E-05 | 0.000182011 | down |
| BTBD16 | -1.414797224 | 0.01086717 | 0.039091625 | down |
| ERVMER34-1 | -1.414739516 | 0.004731003 | 0.020783483 | down |
| GRIK4 | -1.414651316 | 0.001045107 | 0.006281165 | down |
| CTD-2047H16.4 | -1.414588918 | 0.000115413 | 0.001068229 | down |
| ZBTB20-AS3 | -1.414542615 | 0.468826478 | | down |
| ACSS2 | -1.414374362 | 1.52E-09 | 7.36E-08 | down |
| RP11-14C10.3 | -1.414359468 | 0.064662215 | 0.147476777 | down |
| ANKRD9 | -1.41435879 | 3.63E-09 | 1.60E-07 | down |
| NA | -1.41423436 | 0.028695459 | 0.081271341 | down |
| LINC01470 | -1.414212847 | 0.556998801 | 0.676620148 | down |
| NA | -1.414162518 | 0.555772524 | | down |
| RN7SKP181 | -1.413886423 | 0.628839202 | | down |
| AC003682.17 | -1.413863425 | 0.350631196 | | down |
| NMU | -1.413851177 | 0.000213687 | 0.001766062 | down |
| STOX2 | -1.413755037 | 3.80E-05 | 0.000426718 | down |
| AC114765.2 | -1.413686268 | 0.615048568 | | down |
| CTA-268H5.9 | -1.413536805 | 0.613444922 | | down |
| ANKRD34C-AS1 | -1.413357872 | 0.355332783 | 0.493898595 | down |
| RP11-157J24.1 | -1.413114587 | 0.426818952 | | down |
| EIF1AXP2 | -1.412853681 | 0.508040067 | | down |
| NA | -1.412755717 | 0.068790822 | 0.15438496 | down |
| RP11-374P20.4 | -1.412738042 | 0.166550624 | 0.289660362 | down |
| NA | -1.412683092 | 0.009906733 | 0.036381601 | down |
| LRRC7 | -1.412600132 | 0.000853777 | 0.005343931 | down |
| KIAA1456 | -1.412309671 | 5.30E-10 | 2.95E-08 | down |
| OR10G4 | -1.4121427 | 0.555806042 | | down |

| | | | | |
|---|---|---|---|---|
| TRIM36 | -1.412112764 | 6.07E-07 | 1.34E-05 | down |
| RP13-580B18.2 | -1.412047896 | 0.62919408 | | down |
| RP11-800A3.4 | -1.411976457 | 8.45E-08 | 2.48E-06 | down |
| NA | -1.411944327 | 0.59960915 | | down |
| RP11-162G10.1 | -1.411844096 | 0.332525237 | | down |
| MTCO2P12 | -1.41169476 | 0.000984394 | 0.005982023 | down |
| AC018442.1 | -1.411551686 | 0.051555951 | 0.125096672 | down |
| NA | -1.411302862 | 0.053245104 | 0.127934734 | down |
| NA | -1.411156935 | 0.000357887 | 0.002683824 | down |
| OR10Z1 | -1.410861812 | 0.556862124 | | down |
| FLJ45079 | -1.410836586 | 0.042908267 | 0.10949006 | down |
| MT-ATP8 | -1.410654461 | 0.008982781 | 0.033785512 | down |
| HIST1H2AJ | -1.410642492 | 0.003902514 | 0.017889401 | down |
| AF131215.6 | -1.410481611 | 0.469859474 | | down |
| 5S_rRNA | -1.410412746 | 0.340172748 | | down |
| CLSTN2-AS1 | -1.41037313 | 0.613356068 | | down |
| DNAH6 | -1.410317724 | 6.41E-05 | 0.00065351 | down |
| RP11-60A8.1 | -1.410177279 | 0.103974686 | 0.207590076 | down |
| AL133245.2 | -1.409823409 | 0.020339927 | 0.062882564 | down |
| OR2T7 | -1.409486241 | 0.604575328 | | down |
| HMGB1P44 | -1.409440214 | 0.561402083 | | down |
| RNA5SP80 | -1.409421218 | 0.629834005 | | down |
| TRIM36-IT1 | -1.409150415 | 0.212044803 | 0.342767355 | down |
| RP3-414A15.2 | -1.408926525 | 0.215036993 | | down |
| RP13-46H24.1 | -1.408748475 | 0.078736176 | 0.170216377 | down |
| LMO7DN | -1.408732501 | 0.088144795 | 0.184763986 | down |
| RP13-753N3.1 | -1.408539759 | 0.003626879 | 0.016841722 | down |
| RP11-556G22.2 | -1.408480334 | 0.598675105 | | down |
| NA | -1.408240229 | 0.195955484 | 0.324457787 | down |
| AL592528.1 | -1.408140876 | 0.016038014 | 0.052528736 | down |
| AC104135.2 | -1.40803011 | 0.044904128 | 0.113065673 | down |
| LINC00051 | -1.407962248 | 0.469143004 | | down |
| RP11-338K13.1 | -1.407584796 | 0.176777347 | 0.301712069 | down |
| KRT18P11 | -1.40698632 | 0.009700538 | 0.035795009 | down |
| SLFN14 | -1.406939778 | 0.080691603 | 0.173263187 | down |
| CTD-2231E14.8 | -1.40691867 | 0.01554386 | 0.051350293 | down |
| CLNS1AP1 | -1.406893566 | 0.488828726 | 0.617437579 | down |
| RP11-379F4.9 | -1.406817899 | 0.011588263 | 0.04109511 | down |
| RNU6-456P | -1.406801791 | 0.630563465 | | down |
| RPL26P35 | -1.406502612 | 0.220619633 | 0.352306392 | down |
| RP11-43N5.1 | -1.406481583 | 0.058479957 | 0.1371884 | down |
| RP11-379B18.5 | -1.406411743 | 1.96E-06 | 3.64E-05 | down |
| RNU4-58P | -1.406045636 | 0.35859908 | 0.496918171 | down |
| RP11-678G14.5 | -1.405838765 | 0.230573779 | 0.362954973 | down |
| OTOR | -1.40578025 | 0.47294265 | | down |
| RP11-753C18.12 | -1.405582737 | 0.36345467 | 0.501890523 | down |
| RP11-20I20.1 | -1.405509302 | 0.21775308 | 0.34913937 | down |
| SHCBP1L | -1.405479668 | 0.217685697 | 0.349068589 | down |
| CYP27A1 | -1.405423156 | 1.59E-16 | 4.45E-14 | down |
| CTD-2527I21.7 | -1.405394395 | 0.086302273 | 0.182044898 | down |
| PAX2 | -1.40537213 | 0.185464399 | 0.311638277 | down |

| | | | | |
|---|---|---|---|---|
| RP3-388N13.3 | -1.405347301 | 0.315667875 | | down |
| RP11-504G3.4 | -1.405303803 | 0.306339122 | | down |
| RP11-229D13.3 | -1.40480888 | 0.015391302 | 0.05094494 | down |
| RP1-121G13.2 | -1.40460102 | 0.630951951 | | down |
| RP11-400F19.18 | -1.404466964 | 0.021531428 | 0.065554338 | down |
| RP11-226M10.3 | -1.404369017 | 0.047544177 | 0.117865933 | down |
| PTPN2P2 | -1.404359674 | 0.611398975 | | down |
| RNF223 | -1.404337479 | 0.000480477 | 0.003365693 | down |
| TCTEX1D4 | -1.404206006 | 0.000929494 | 0.00572131 | down |
| WDR72 | -1.403743034 | 0.015940256 | 0.052288355 | down |
| CDCA4P4 | -1.403608986 | 0.196771521 | 0.325396499 | down |
| KLRF2 | -1.403590684 | 0.222203045 | | down |
| NA | -1.403397251 | 0.059581813 | 0.138963703 | down |
| RP3-333H23.9 | -1.40320305 | 0.01006769 | 0.036877619 | down |
| CTD-2201I18.1 | -1.403174498 | 0.004725328 | 0.020770124 | down |
| AC098795.1 | -1.403129927 | 0.562967351 | | down |
| CTB-174D11.2 | -1.403094155 | 0.202404388 | | down |
| EIF1P6 | -1.403054364 | 0.028240016 | 0.080322166 | down |
| NA | -1.402965117 | 0.214640546 | | down |
| RPL21P75 | -1.402925103 | 5.45E-06 | 8.51E-05 | down |
| ATP5F1P1 | -1.402912056 | 0.241648377 | | down |
| RP11-104E19.1 | -1.402895597 | 0.362178936 | | down |
| VWA5B2 | -1.402806591 | 0.00047892 | 0.003357916 | down |
| HMX3 | -1.402800186 | 0.002042241 | 0.010672656 | down |
| LINC00457 | -1.402530566 | 0.631457164 | | down |
| RP11-16P20.3 | -1.402363898 | 0.006493172 | 0.02647318 | down |
| FAM83F | -1.402201871 | 1.98E-05 | 0.00024852 | down |
| RP11-269G24.6 | -1.402163294 | 0.020604874 | 0.063460055 | down |
| AC079305.8 | -1.40200768 | 0.002593211 | 0.01293978 | down |
| AL022344.7 | -1.40195969 | 0.002939572 | 0.014272221 | down |
| RP11-722M1.1 | -1.401930987 | 0.631709285 | | down |
| SNORD39 | -1.401821809 | 0.631807359 | | down |
| PSMC1P11 | -1.401738461 | 0.512626096 | | down |
| RP13-16H11.5 | -1.401605919 | 0.240516984 | 0.374276714 | down |
| RPL3L | -1.4013915 | 0.012174061 | 0.042645938 | down |
| CTD-2561B21.8 | -1.401356958 | 5.65E-05 | 0.000591506 | down |
| MIR1273E | -1.401252749 | 0.476614476 | | down |
| BSG | -1.401098991 | 1.28E-07 | 3.51E-06 | down |
| MEGF10 | -1.401026031 | 0.005384464 | 0.022969024 | down |
| CATSPER2P1 | -1.400872361 | 4.04E-05 | 0.000447617 | down |
| NA | -1.400767789 | 0.034148854 | 0.0923838 | down |
| RP4-811H24.9 | -1.400592622 | 0.22074919 | 0.352419825 | down |
| AC004899.3 | -1.40040526 | 0.631975937 | | down |
| RP11-436D10.3 | -1.400322303 | 0.178684024 | | down |
| FAM215A | -1.400301071 | 0.058408508 | 0.137110406 | down |
| MPND | -1.40029091 | 1.72E-08 | 6.30E-07 | down |
| RP11-109A6.2 | -1.400089632 | 0.351946693 | | down |
| RP11-867G2.8 | -1.39986991 | 0.632211841 | | down |
| RP11-122K13.7 | -1.399748315 | 0.02944948 | 0.082743868 | down |
| AC073065.3 | -1.399668332 | 0.433662105 | | down |
| AC092198.1 | -1.399598194 | 0.064083234 | 0.146545645 | down |

| | | | | |
|---|---|---|---|---|
| RP11-167P11.2 | -1.399580872 | 0.045487243 | 0.114075019 | down |
| NA | -1.399511284 | 0.400416434 | | down |
| RP11-403F21.4 | -1.399469776 | 0.322956431 | | down |
| RP11-627G18.1 | -1.399428458 | 0.047549373 | 0.117869089 | down |
| RNA5SP483 | -1.399408223 | 0.328438816 | | down |
| LINC01521 | -1.399403485 | 0.001150753 | 0.006783053 | down |
| CTD-2102P23.1 | -1.399261516 | 0.188458605 | 0.315364282 | down |
| KRT8P32 | -1.399018876 | 0.049575882 | 0.121568544 | down |
| PSG5 | -1.398600135 | 0.632292214 | | down |
| RP11-423C15.3 | -1.398466802 | 0.152069437 | 0.271463681 | down |
| RNA5SP151 | -1.398280728 | 0.237061013 | 0.37016462 | down |
| TCAM1P | -1.39819204 | 0.061715186 | 0.142727353 | down |
| RNU6-698P | -1.398097565 | 0.558284918 | | down |
| RP11-731D1.4 | -1.398036917 | 0.052840772 | 0.127197192 | down |
| ZBTB8OSP2 | -1.39795266 | 0.140351343 | 0.256800134 | down |
| RP11-281P11.1 | -1.397892287 | 0.328801242 | 0.467223408 | down |
| LINC00664 | -1.397858696 | 0.015418075 | 0.051026728 | down |
| TMEM52 | -1.39779852 | 5.97E-05 | 0.00061746 | down |
| RP11-393B14.2 | -1.397712948 | 0.303612553 | | down |
| RP11-25C19.3 | -1.397589465 | 0.476236752 | | down |
| DPRXP4 | -1.397554659 | 0.016412125 | 0.053410517 | down |
| IGSF11 | -1.397498394 | 0.007124136 | 0.028356854 | down |
| PROC | -1.397492839 | 3.12E-06 | 5.37E-05 | down |
| PROM2 | -1.397340911 | 2.63E-05 | 0.000314603 | down |
| RP11-694I15.7 | -1.397119947 | 4.12E-05 | 0.000454979 | down |
| CTD-2537I9.19 | -1.396872429 | 0.567524817 | | down |
| RP11-174J11.1 | -1.396762104 | 0.138482598 | 0.254422502 | down |
| RP5-915N17.11 | -1.396510681 | 0.059373082 | 0.138634093 | down |
| ASCL5 | -1.396463528 | 0.006757509 | 0.027276983 | down |
| RP11-893F2.14 | -1.395889966 | 0.12371044 | 0.234999776 | down |
| RP11-1029J19.2 | -1.395783702 | 0.252945491 | 0.388005793 | down |
| CTD-2561B21.4 | -1.395557753 | 0.274560561 | | down |
| PRH1 | -1.395494597 | 0.002136544 | 0.011069206 | down |
| RP11-449J21.5 | -1.395317031 | 0.015616663 | 0.051511396 | down |
| METTL16 | -1.395302768 | 3.64E-07 | 8.51E-06 | down |
| RPL7P60 | -1.395278121 | 0.214896925 | 0.345963092 | down |
| GNA11 | -1.395269644 | 6.71E-09 | 2.76E-07 | down |
| RP11-115A14.1 | -1.395237827 | 0.226944087 | 0.359078572 | down |
| RP11-109P14.8 | -1.395161532 | 0.607639005 | | down |
| NA | -1.395144839 | 0.273099688 | 0.409308941 | down |
| RP5-837I24.4 | -1.395034765 | 0.633391462 | | down |
| RP3-414A15.11 | -1.394945025 | 0.049399909 | 0.121305799 | down |
| UBE2CP1 | -1.394900907 | 0.519923168 | | down |
| DMP1 | -1.394722667 | 0.401585627 | | down |
| NA | -1.394711724 | 0.565141392 | | down |
| NA | -1.394614065 | 0.122229546 | 0.233085653 | down |
| LINC01346 | -1.394352436 | 0.264725162 | | down |
| CTB-1048E9.7 | -1.394331228 | 0.268903518 | 0.404680498 | down |
| CTD-2227I18.1 | -1.394245707 | 0.072935891 | 0.161088429 | down |
| XIRP2 | -1.393955771 | 0.614228832 | | down |
| DLGAP1 | -1.393776519 | 0.001349088 | 0.007675062 | down |

| | | | | |
|---|---|---|---|---|
| RP11-1055B8.3 | -1.393668282 | 0.004932062 | 0.021465883 | down |
| PAX4 | -1.393198702 | 0.01880699 | 0.059271709 | down |
| AC005355.3 | -1.393142526 | 0.00533452 | 0.022801296 | down |
| OR7E29P | -1.39305892 | 0.384922946 | | down |
| BPIFA3 | -1.392968353 | 0.633482591 | | down |
| NA | -1.392824541 | 0.52092313 | | down |
| HRASLS | -1.392570759 | 0.064184874 | 0.14671648 | down |
| NA | -1.392463329 | 0.105685743 | 0.210238961 | down |
| RP1-138B7.7 | -1.39221518 | 0.020440759 | 0.063129257 | down |
| FAM189A2 | -1.392206084 | 5.50E-05 | 0.000577804 | down |
| MYO5B | -1.39219368 | 7.29E-06 | 0.000108822 | down |
| RP3-509L4.3 | -1.392053476 | 0.285401573 | | down |
| RP11-118A3.1 | -1.391945388 | 0.61294396 | | down |
| RP1-66H9.1 | -1.391895846 | 0.634110622 | | down |
| RP11-12M5.1 | -1.391885625 | 0.634057313 | | down |
| LINC01511 | -1.391817552 | 0.091635784 | 0.18988244 | down |
| RP11-302M6.5 | -1.391760052 | 0.240691485 | | down |
| PIGU | -1.39174362 | 2.62E-06 | 4.64E-05 | down |
| RP11-259P15.1 | -1.391607117 | 0.516327556 | | down |
| LINC01388 | -1.391062378 | 0.438500651 | | down |
| RP11-479J7.1 | -1.391013135 | 0.347378195 | 0.486156115 | down |
| CTD-2125J1.1 | -1.390781355 | 0.466212049 | 0.597522878 | down |
| RP11-434P11.1 | -1.390718541 | 0.112345294 | 0.219474739 | down |
| MUC1 | -1.390620548 | 0.009542385 | 0.035350361 | down |
| RP11-644A7.2 | -1.390571075 | 0.070333566 | 0.156897955 | down |
| CTC-471C19.1 | -1.390537503 | 0.475991562 | | down |
| AC092171.2 | -1.390391797 | 0.007205991 | 0.028591758 | down |
| WWC3-AS1 | -1.390150469 | 0.479849196 | | down |
| RP11-22C11.2 | -1.390039769 | 0.001241525 | 0.007200846 | down |
| IGLJ5 | -1.389408196 | 0.324368245 | | down |
| SYT10 | -1.389244969 | 0.038303592 | 0.10063612 | down |
| TSFM | -1.389084372 | 0.018198251 | 0.057851993 | down |
| CTB-161M19.4 | -1.389001563 | 0.00210511 | 0.010925191 | down |
| HCG14 | -1.388943114 | 0.076191706 | 0.166198556 | down |
| PHEX | -1.388895097 | 0.000594943 | 0.004005931 | down |
| RP1-79C4.4 | -1.388829857 | 0.005074604 | 0.021949524 | down |
| IFITM10 | -1.38865356 | 0.000656017 | 0.00433173 | down |
| CPA4 | -1.388559854 | 0.117284745 | 0.226372058 | down |
| AC005932.1 | -1.388468854 | 0.010407629 | 0.037832644 | down |
| AC009262.2 | -1.388257708 | 0.566823264 | | down |
| RP11-661C8.2 | -1.388154625 | 0.635111233 | | down |
| RN7SL390P | -1.387781402 | 0.389047495 | 0.526597427 | down |
| BCRP3 | -1.387688913 | 0.036244278 | 0.096577709 | down |
| RP11-982M15.5 | -1.387611982 | 0.072107516 | 0.15974032 | down |
| RP11-983C2.2 | -1.387462885 | 0.635240528 | | down |
| RP11-894P9.2 | -1.38735368 | 0.135684385 | | down |
| RP5-1077I2.3 | -1.387025518 | 0.280258668 | | down |
| RP11-1007J8.1 | -1.386915375 | 0.617809255 | | down |
| MPDU1 | -1.386815506 | 5.96E-06 | 9.20E-05 | down |
| DNMBP-AS1 | -1.386756342 | 0.001138077 | 0.006727467 | down |
| RPS3AP46 | -1.386491331 | 0.192657554 | | down |

| | | | | |
|---|---|---|---|---|
| TIGD3 | -1.386478007 | 1.35E-05 | 0.000181541 | down |
| bP-2171C21.5 | -1.386407954 | 0.617869997 | | down |
| AC011516.2 | -1.386403226 | 0.172858511 | 0.297194617 | down |
| FRG2C | -1.386297606 | 0.567111785 | | down |
| SYNGR4 | -1.38626021 | 0.035377079 | 0.094746742 | down |
| BEST2 | -1.386254214 | 0.007436334 | 0.029312152 | down |
| RP11-365O16.3 | -1.38616925 | 0.01628934 | 0.053111232 | down |
| RP11-432I13.6 | -1.386074466 | 0.400019578 | | down |
| RP11-665C16.8 | -1.385753493 | 0.295331898 | | down |
| GALNT13 | -1.385728506 | 0.029492027 | 0.082832426 | down |
| SCUBE2 | -1.385610114 | 2.79E-09 | 1.29E-07 | down |
| MTCO1P12 | -1.385270246 | 0.004148148 | 0.018769119 | down |
| AC010677.5 | -1.385185705 | 0.521443513 | | down |
| BEST4 | -1.384688073 | 0.00073577 | 0.004751823 | down |
| RP11-210H10__A.[illegible] | -1.384619939 | 0.523103237 | | down |
| ZC2HC1B | -1.384619304 | 0.375850066 | 0.514097294 | down |
| RP11-428J1.4 | -1.38452338 | 0.008537121 | 0.032483442 | down |
| RP11-73G16.3 | -1.384472542 | 0.636010691 | | down |
| CTB-158E9.1 | -1.384472542 | 0.636010691 | | down |
| RP1-290I10.2 | -1.384472542 | 0.636010691 | | down |
| RN7SKP192 | -1.384472542 | 0.636010691 | | down |
| RNU6-131P | -1.384472542 | 0.636010691 | | down |
| SCN11A | -1.384410412 | 0.000843615 | 0.005295775 | down |
| RNU6-1203P | -1.384378274 | 0.563513275 | | down |
| NOVA1 | -1.383950914 | 7.63E-07 | 1.61E-05 | down |
| GP5 | -1.383844082 | 0.020593484 | 0.063431478 | down |
| U1 | -1.383703448 | 0.065720319 | 0.149176584 | down |
| RP11-442J17.3 | -1.383544396 | 0.521700276 | | down |
| NA | -1.383325124 | 0.635781606 | | down |
| RP11-779O18.3 | -1.38314244 | 0.028937391 | 0.081771537 | down |
| HEPACAM2 | -1.383107692 | 0.001654448 | 0.009022661 | down |
| ATP5EP2 | -1.383002058 | 0.001782715 | 0.009572814 | down |
| RP11-164P12.3 | -1.382860471 | 0.078753366 | | down |
| PLA2G4E | -1.382784311 | 0.021257686 | 0.064957885 | down |
| HRK | -1.382651464 | 0.002322004 | 0.011856267 | down |
| RP11-164C12.1 | -1.382602755 | 0.288062956 | 0.425371029 | down |
| BRD7P5 | -1.382558191 | 0.636478532 | | down |
| RP11-585F1.6 | -1.382370244 | 0.176928407 | | down |
| FAM222A-AS1 | -1.38236014 | 0.081015386 | 0.173815232 | down |
| AC009229.6 | -1.382334464 | 0.404055105 | | down |
| RP11-163F15.1 | -1.382093014 | 0.566388392 | | down |
| RP11-321C24.4 | -1.382074952 | 0.396647381 | 0.533551553 | down |
| C16orf96 | -1.382071478 | 0.013701025 | 0.046570154 | down |
| RN7SL744P | -1.382053491 | 0.636601898 | | down |
| HS3ST5 | -1.381947599 | 0.029414368 | 0.08267614 | down |
| NA | -1.381930444 | 0.098103214 | 0.199270702 | down |
| AC005740.5 | -1.381892454 | 0.055626539 | 0.132175531 | down |
| SNRPGP5 | -1.381819366 | 0.566369233 | | down |
| HSPE1P10 | -1.381711326 | 0.443885772 | 0.577470518 | down |
| RP11-307C19.1 | -1.381634553 | 0.317964361 | | down |
| TMPOP2 | -1.381633107 | 0.064681677 | 0.147491252 | down |

| | | | | |
|---|---|---|---|---|
| RN7SL37P | -1.381456817 | 0.636747759 | | down |
| FKBP4P2 | -1.381456817 | 0.636747759 | | down |
| NA | -1.381096662 | 0.301448455 | | down |
| BMS1P8 | -1.381088155 | 0.078898578 | 0.170448614 | down |
| RP5-1114G22.2 | -1.380686138 | 0.636936176 | | down |
| SNORA25 | -1.380320187 | 0.637025653 | | down |
| ZP4 | -1.380239233 | 0.637045447 | | down |
| CALML5 | -1.380190046 | 0.618693155 | | down |
| RP11-69J7.1 | -1.379854674 | 0.263340358 | | down |
| RP11-390K5.3 | -1.379747654 | 0.233250755 | | down |
| PRSS3 | -1.379501408 | 7.30E-06 | 0.000108911 | down |
| MMAB | -1.379149259 | 1.54E-06 | 2.96E-05 | down |
| LAD1 | -1.378816526 | 1.33E-05 | 0.000178533 | down |
| RP11-447H19.3 | -1.378779533 | 0.312778538 | 0.451458967 | down |
| HMGN1P4 | -1.378657678 | 0.43893862 | | down |
| RP1-145M24.1 | -1.378649313 | 0.29019784 | | down |
| RPS7P5 | -1.378506631 | 0.615429497 | | down |
| LA16c-429E7.1 | -1.378475597 | 0.008034477 | 0.031062646 | down |
| LRRD1 | -1.378235585 | 0.342865724 | | down |
| CKMT1B | -1.378032975 | 8.87E-05 | 0.000855851 | down |
| NA | -1.378028272 | 0.004875196 | 0.021270751 | down |
| AC005481.5 | -1.378011691 | 0.034514027 | 0.09318291 | down |
| NA | -1.377860783 | 0.388519297 | | down |
| TEC | -1.377732138 | 2.24E-08 | 7.82E-07 | down |
| AC009313.1 | -1.377546855 | 0.477546527 | | down |
| P3H2-AS1 | -1.377542842 | 0.092736458 | 0.191489633 | down |
| RP11-39E3.4 | -1.377319464 | 0.520633891 | | down |
| RPL35AP32 | -1.377309609 | 0.299795397 | | down |
| RP1-266L20.9 | -1.377208522 | 0.001524071 | 0.008439182 | down |
| RP11-573D15.8 | -1.377164894 | 0.030302288 | 0.084421545 | down |
| NA | -1.377110969 | 0.266624236 | | down |
| RNU6-660P | -1.376872334 | 0.524043418 | | down |
| WASIR2 | -1.376740929 | 0.018180943 | 0.057820288 | down |
| SLC9A3P3 | -1.376367044 | 0.480224161 | | down |
| RP11-499E14.1 | -1.376166782 | 0.237167415 | 0.370249822 | down |
| NA | -1.376114342 | 0.003513747 | 0.016433223 | down |
| LINC00238 | -1.375876749 | 0.009865494 | 0.036252313 | down |
| GER4P2-CDK2AP2I | -1.375812287 | 0.059079876 | 0.138190207 | down |
| OR7A11P | -1.375764818 | 0.620471561 | | down |
| GUCY1B2 | -1.375589273 | 0.007200026 | 0.028579413 | down |
| AC004053.1 | -1.375511649 | 0.148124399 | 0.266801526 | down |
| RN7SL589P | -1.375348865 | 0.395491421 | | down |
| FAM234A | -1.375327495 | 2.85E-09 | 1.31E-07 | down |
| RP11-883G14.3 | -1.375251825 | 0.638182919 | | down |
| Y_RNA | -1.375251825 | 0.638182919 | | down |
| RP11-344B23.2 | -1.375239963 | 0.292965072 | | down |
| ETFB | -1.375201781 | 2.84E-06 | 4.99E-05 | down |
| PLA2G2C | -1.37511279 | 0.029019875 | 0.081927563 | down |
| VSIG2 | -1.375026452 | 0.000439984 | 0.003139874 | down |
| Metazoa_SRP | -1.374974792 | 0.248485545 | 0.382924688 | down |
| HMGB1P21 | -1.374939147 | 0.359183409 | | down |

| | | | | |
|---|---|---|---|---|
| CTD-2185K10.1 | -1.374896864 | 0.207697909 | 0.337867561 | down |
| RP11-1H8.5 | -1.374821671 | 0.013970768 | 0.047316503 | down |
| RP1-249I4.2 | -1.374368677 | 0.006081444 | 0.025211264 | down |
| CACNB2 | -1.374326992 | 3.44E-07 | 8.14E-06 | down |
| KIF5C | -1.374278059 | 6.87E-06 | 0.000103518 | down |
| RNU6-88P | -1.374171121 | 0.278043258 | | down |
| ALOX12P2 | -1.374146434 | 0.009425941 | 0.035048219 | down |
| YWHAZP5 | -1.374127847 | 0.523414113 | | down |
| RP11-483I13.2 | -1.374113076 | 0.390491227 | | down |
| CTD-2140G10.2 | -1.374031816 | 0.367174601 | | down |
| PRDX2P4 | -1.373868296 | 0.623220005 | | down |
| LL22NC03-86G7.1 | -1.373432633 | 9.90E-07 | 2.01E-05 | down |
| RP11-100G15.12 | -1.37341964 | 0.316082486 | | down |
| LINC01572 | -1.373141182 | 0.003761467 | 0.017364709 | down |
| OR7E1P | -1.373082618 | 0.6211431 | | down |
| SPATA2L | -1.373056552 | 3.40E-07 | 8.07E-06 | down |
| CTD-2296D1.3 | -1.372753632 | 0.638741701 | | down |
| KB-1562D12.1 | -1.372407556 | 0.524440289 | | down |
| XX-FW83563B9.5 | -1.372194772 | 0.005465353 | 0.023218476 | down |
| NA | -1.372045037 | 0.092459013 | 0.191121598 | down |
| KLHL32 | -1.371852065 | 0.000368616 | 0.002741716 | down |
| TKTL2 | -1.371766006 | 0.216156331 | 0.347261428 | down |
| RP11-160H22.3 | -1.371753803 | 0.042128968 | 0.108042912 | down |
| HK2P1 | -1.371614782 | 0.336797552 | | down |
| MAVS | -1.37157643 | 7.29E-09 | 2.99E-07 | down |
| RP11-298D21.3 | -1.371411877 | 0.049310833 | 0.121160587 | down |
| NA | -1.371276177 | 0.010964788 | 0.039376773 | down |
| uc_338 | -1.371092156 | 0.565156945 | | down |
| KRT18P63 | -1.370630528 | 0.034113982 | 0.092335179 | down |
| CTD-2023M8.1 | -1.370625175 | 0.004249279 | 0.019125933 | down |
| LRRC6P1 | -1.370442297 | 0.639467878 | | down |
| LINC01494 | -1.370339267 | 0.525360751 | | down |
| NLRP12 | -1.370207061 | 0.0089785 | 0.033773641 | down |
| RP11-184M15.2 | -1.370174926 | 0.639470426 | | down |
| MNX1 | -1.369954884 | 5.99E-05 | 0.000618832 | down |
| TBC1D3G | -1.369928067 | 0.572674881 | | down |
| RP3-336K20__B.2 | -1.369793242 | 0.049594942 | 0.121585511 | down |
| CTD-2587M23.1 | -1.369508475 | 0.576796148 | | down |
| CHST6 | -1.369419565 | 3.70E-05 | 0.000417686 | down |
| KRTDAP | -1.369300027 | 0.324868807 | 0.463322815 | down |
| RP11-63N3.1 | -1.369244536 | 0.354242699 | 0.492885086 | down |
| GMCL1P2 | -1.369145709 | 0.483834033 | | down |
| PPP1R1AP1 | -1.368989914 | 0.617017613 | | down |
| ZNF887P | -1.368956393 | 0.080392103 | | down |
| RP11-596D21.1 | -1.368937392 | 0.044353107 | 0.112063305 | down |
| RP11-114H23.1 | -1.36889639 | 0.489231741 | | down |
| LINC00266-1 | -1.368880163 | 0.226133181 | | down |
| LINC00550 | -1.368841281 | 0.410144174 | 0.546980272 | down |
| CTC-338M12.13 | -1.368767922 | 0.172445505 | 0.296732689 | down |
| NA | -1.368744657 | 0.235959615 | | down |
| MTCO1P28 | -1.368716265 | 0.292709393 | | down |

| | | | | |
|---|---|---|---|---|
| VDAC3P1 | -1.368607528 | 0.384217131 | | down |
| RP11-166B2.7 | -1.36844608 | 0.573737517 | | down |
| GMNC | -1.368368049 | 0.639870106 | | down |
| MT-ND3 | -1.368021363 | 0.000900483 | 0.005567072 | down |
| HAGLR | -1.367981854 | 1.71E-08 | 6.28E-07 | down |
| SVOP | -1.367977619 | 0.006267865 | 0.02578745 | down |
| RN7SL648P | -1.367663917 | 0.244828437 | 0.378770734 | down |
| CDIPT-AS1 | -1.367257647 | 0.063527939 | 0.14570833 | down |
| HSPA8P7 | -1.367232989 | 0.527548083 | | down |
| MTCO1P53 | -1.367199041 | 0.011413437 | 0.04060809 | down |
| DRD5P1 | -1.366922647 | 0.484277669 | | down |
| NA | -1.366891044 | 0.037613925 | 0.099224933 | down |
| RP4-569D19.8 | -1.366888881 | 0.077535059 | 0.16825928 | down |
| WI2-85898F10.2 | -1.36677913 | 0.255710265 | | down |
| NA | -1.366654951 | 0.322550777 | | down |
| HPN-AS1 | -1.366456886 | 0.12345197 | 0.234657155 | down |
| RP11-90C4.1 | -1.366299966 | 0.067980958 | 0.153036108 | down |
| RP11-736N17.8 | -1.365813582 | 0.012433741 | 0.043291149 | down |
| UCKL1-AS1 | -1.365811754 | 0.000150463 | 0.001323725 | down |
| RP11-563D10.1 | -1.365590806 | 0.029912547 | 0.083588104 | down |
| RP1-120G22.11 | -1.365517536 | 0.000809302 | 0.005126615 | down |
| RP1-178F15.4 | -1.365497062 | 0.067421255 | 0.152070235 | down |
| MTRF1LP1 | -1.365475213 | 0.574133473 | | down |
| NA | -1.365316112 | 0.009166123 | 0.034311862 | down |
| RP1-60019.1 | -1.365224551 | 0.001063834 | 0.006373247 | down |
| AC008694.3 | -1.36514185 | 0.618040827 | | down |
| NA | -1.365037909 | 0.244358736 | 0.378331041 | down |
| MT-TL2 | -1.364813793 | 0.640846014 | | down |
| FAM177A1P1 | -1.36464284 | 0.64078373 | | down |
| FBP2P1 | -1.36464284 | 0.64078373 | | down |
| AC019181.3 | -1.364578267 | 0.165236718 | 0.288006727 | down |
| NEDD4L | -1.364543311 | 1.76E-12 | 1.83E-10 | down |
| ZBTB7A | -1.364516949 | 5.82E-10 | 3.21E-08 | down |
| RHAG | -1.364448529 | 0.114327617 | 0.222220283 | down |
| VDAC1P3 | -1.364434677 | 0.197189812 | | down |
| TMEM171 | -1.364238269 | 6.02E-05 | 0.000621719 | down |
| NA | -1.3641412 | 0.611985234 | | down |
| RP5-936J12.1 | -1.364126573 | 0.641014364 | | down |
| OR11I1P | -1.364126573 | 0.641014364 | | down |
| OR6N2 | -1.364126573 | 0.641014364 | | down |
| RP4-560B9.5 | -1.364126573 | 0.641014364 | | down |
| OR14A2 | -1.364126573 | 0.641014364 | | down |
| AC079112.1 | -1.364126573 | 0.641014364 | | down |
| AC068042.1 | -1.364126573 | 0.641014364 | | down |
| AC006499.7 | -1.364126573 | 0.641014364 | | down |
| MIR7978 | -1.364126573 | 0.641014364 | | down |
| MTCO3P44 | -1.364126573 | 0.641014364 | | down |
| RP11-769N22.1 | -1.364126573 | 0.641014364 | | down |
| RP11-731J8.1 | -1.364126573 | 0.641014364 | | down |
| RP11-317024.2 | -1.364126573 | 0.641014364 | | down |
| CTB-85P21.1 | -1.364126573 | 0.641014364 | | down |

| | | | |
|---|---|---|---|
| VGLL2 | -1.364126573 | 0.641014364 | down |
| RPL7AP38 | -1.364126573 | 0.641014364 | down |
| HTR5A-AS1 | -1.364126573 | 0.641014364 | down |
| RP5-912I13.1 | -1.364126573 | 0.641014364 | down |
| RP11-98H4.1 | -1.364126573 | 0.641014364 | down |
| RNU6-325P | -1.364126573 | 0.641014364 | down |
| KRT8P38 | -1.364126573 | 0.641014364 | down |
| RP11-63C8.1 | -1.364126573 | 0.641014364 | down |
| RPL7AP57 | -1.364126573 | 0.641014364 | down |
| OR8S1 | -1.364126573 | 0.641014364 | down |
| RPL35AP30 | -1.364126573 | 0.641014364 | down |
| GSX1 | -1.364126573 | 0.641014364 | down |
| SQSTM1P1 | -1.364126573 | 0.641014364 | down |
| SPACA7 | -1.364126573 | 0.641014364 | down |
| ZNF720P1 | -1.364126573 | 0.641014364 | down |
| RP11-19N8.2 | -1.364126573 | 0.641014364 | down |
| RP11-640N20.9 | -1.364126573 | 0.641014364 | down |
| SPPL2C | -1.364126573 | 0.641014364 | down |
| MIR181D | -1.364126573 | 0.641014364 | down |
| NTF6B | -1.364126573 | 0.641014364 | down |
| BIRC8 | -1.364126573 | 0.641014364 | down |
| RP5-1128N12.2 | -1.364126573 | 0.641014364 | down |
| AC006548.19 | -1.364126573 | 0.641014364 | down |
| ZNF280A | -1.364126573 | 0.641014364 | down |
| RNA5SP45 | -1.364126199 | 0.641014456 | down |
| NA | -1.364126199 | 0.641014456 | down |
| LCE3A | -1.364126199 | 0.641014456 | down |
| OR6K6 | -1.364126199 | 0.641014456 | down |
| APCS | -1.364126199 | 0.641014456 | down |
| AC007253.1 | -1.364126199 | 0.641014456 | down |
| RNU4-84P | -1.364126199 | 0.641014456 | down |
| NA | -1.364126199 | 0.641014456 | down |
| GAPDHP50 | -1.364126199 | 0.641014456 | down |
| RP11-646E18.3 | -1.364126199 | 0.641014456 | down |
| RP11-480D4.1 | -1.364126199 | 0.641014456 | down |
| RNU4-43P | -1.364126199 | 0.641014456 | down |
| RNU2-49P | -1.364126199 | 0.641014456 | down |
| CTA-242H14.1 | -1.364126199 | 0.641014456 | down |
| RP11-577N1.2 | -1.364126199 | 0.641014456 | down |
| MIR3663HG | -1.364126199 | 0.641014456 | down |
| MIR3663 | -1.364126199 | 0.641014456 | down |
| RP11-702F3.2 | -1.364126199 | 0.641014456 | down |
| UBTFL2 | -1.364126199 | 0.641014456 | down |
| RP11-136I14.5 | -1.364126199 | 0.641014456 | down |
| OR10N1P | -1.364126199 | 0.641014456 | down |
| RP11-861E23.2 | -1.364126199 | 0.641014456 | down |
| GNAI2P1 | -1.364126199 | 0.641014456 | down |
| RNU5A-7P | -1.364126199 | 0.641014456 | down |
| Metazoa_SRP | -1.364126199 | 0.641014456 | down |
| FAM206BP | -1.364126199 | 0.641014456 | down |
| OR7E159P | -1.364126199 | 0.641014456 | down |

| | | | | |
|---|---|---|---|---|
| RN7SL108P | -1.364126199 | 0.641014456 | | down |
| RP11-305A4.3 | -1.364126199 | 0.641014456 | | down |
| RP11-23E19.1 | -1.364126199 | 0.641014456 | | down |
| NA | -1.364126199 | 0.641014456 | | down |
| RP11-874J12.3 | -1.364126199 | 0.641014456 | | down |
| OR7H1P | -1.364126199 | 0.641014456 | | down |
| CTB-60B18.12 | -1.364126199 | 0.641014456 | | down |
| MIR4752 | -1.364126199 | 0.641014456 | | down |
| RP4-796I8.1 | -1.364126199 | 0.641014456 | | down |
| RP1-138B7.4 | -1.364126199 | 0.641014456 | | down |
| SNORA33 | -1.364126199 | 0.641014456 | | down |
| KRTAP10-4 | -1.364126199 | 0.641014456 | | down |
| Metazoa_SRP | -1.364016755 | 0.621550037 | | down |
| OTOS | -1.364013108 | 0.574454698 | | down |
| ALG1L15P | -1.363951576 | 0.067037893 | 0.151446459 | down |
| RAD17P1 | -1.363942035 | 0.225392594 | | down |
| GRID2IP | -1.363839783 | 2.49E-06 | 4.44E-05 | down |
| RP11-151F5.2 | -1.36378048 | 0.035151454 | 0.094361176 | down |
| ELL2P1 | -1.36377341 | 0.008400373 | 0.032082361 | down |
| RP11-47J17.1 | -1.363692602 | 0.570662533 | | down |
| RP11-10N16.2 | -1.363672089 | 0.165708223 | 0.288560914 | down |
| ESRRB | -1.363647628 | 0.010433782 | 0.037900199 | down |
| AC073415.2 | -1.363611083 | 0.30676063 | | down |
| MAPT-AS1 | -1.363566732 | 0.16800457 | | down |
| RP11-708J19.3 | -1.363518279 | 0.529282257 | | down |
| NA | -1.363380983 | 0.573763803 | | down |
| GRB7 | -1.363065116 | 2.19E-07 | 5.56E-06 | down |
| LINC01341 | -1.362904494 | 0.001247828 | 0.007230424 | down |
| RNASE13 | -1.362770652 | 0.531434784 | | down |
| TP53TG1 | -1.362668576 | 1.41E-05 | 0.000187906 | down |
| RPL7P21 | -1.362585785 | 0.193723595 | | down |
| RPL27AP8 | -1.362579123 | 0.641332596 | | down |
| RPL7P44 | -1.362507722 | 0.14095697 | | down |
| ABCA17P | -1.362444769 | 0.001645164 | 0.008983517 | down |
| ACOX2 | -1.362302117 | 9.80E-05 | 0.000928974 | down |
| RP11-554A11.7 | -1.362212384 | 0.172854959 | 0.297194617 | down |
| LSM1P1 | -1.362172885 | 0.623603042 | | down |
| AL022476.2 | -1.362132382 | 0.091309029 | 0.189401214 | down |
| CNTNAP2 | -1.362105906 | 1.96E-07 | 5.09E-06 | down |
| MTND5P35 | -1.362085669 | 0.641453648 | | down |
| RP11-386B13.3 | -1.361904985 | 0.370736511 | 0.509130296 | down |
| DLEU1 | -1.361882785 | 3.60E-07 | 8.44E-06 | down |
| RPL15P21 | -1.361839331 | 0.044547625 | 0.112375219 | down |
| RP11-609D21.3 | -1.36163583 | 0.184221715 | 0.310171022 | down |
| RP11-327J17.1 | -1.361555383 | 0.352484378 | | down |
| RP5-1142J19.1 | -1.360972503 | 0.001517423 | 0.008414443 | down |
| RP11-100N20.1 | -1.36091002 | 0.355874682 | | down |
| RP11-322L20.1 | -1.360449442 | 0.369945317 | 0.50839983 | down |
| RNU6-975P | -1.360445168 | 0.571529206 | | down |
| AP001442.2 | -1.360380388 | 0.145904423 | | down |
| TMEM229B | -1.360123305 | 3.90E-13 | 4.80E-11 | down |

| | | | | |
|---|---|---|---|---|
| RP11-240L7.4 | -1.36010119 | 0.443663772 | | down |
| RP11-480010.1 | -1.360087028 | 0.641730649 | | down |
| NA | -1.360010254 | 0.642023132 | | down |
| RP5-892F13.2 | -1.359883647 | 0.485923305 | | down |
| PXT1 | -1.35976138 | 0.162390962 | 0.284349812 | down |
| LINC01356 | -1.359568583 | 0.012900655 | 0.044509332 | down |
| LINC01426 | -1.35943646 | 0.003803472 | 0.01750751 | down |
| AC107983.4 | -1.35939904 | 0.000840644 | 0.005280431 | down |
| GRK5-IT1 | -1.359358533 | 0.186893905 | 0.313321829 | down |
| TPH1 | -1.359355756 | 3.03E-05 | 0.000353501 | down |
| CAPN12 | -1.359300562 | 0.000100368 | 0.000948259 | down |
| CTD-2532N20.1 | -1.359220948 | 0.032368907 | 0.088682422 | down |
| STX17-AS1 | -1.358942754 | 0.000143898 | 0.001276753 | down |
| MT-ND2 | -1.358765605 | 0.0027649 | 0.013608887 | down |
| RP11-64B16.3 | -1.358597857 | 0.486396906 | | down |
| NA | -1.358195566 | 0.339643212 | | down |
| F10-AS1 | -1.358017406 | 0.338059848 | 0.476436348 | down |
| SLC16A5 | -1.357926051 | 2.19E-11 | 1.78E-09 | down |
| NA | -1.357893086 | 0.004356501 | 0.019468499 | down |
| TDRD9 | -1.357881718 | 0.002114539 | 0.010968443 | down |
| FOXCUT | -1.357795138 | 0.066773871 | 0.150973168 | down |
| RP1-102D24.5 | -1.357631775 | 0.159996112 | 0.281252171 | down |
| TMC2 | -1.3573002 | 0.003236808 | 0.015362819 | down |
| RP11-93H10.1 | -1.35718843 | 0.626204137 | | down |
| Y_RNA | -1.357160409 | 0.629437367 | | down |
| RP11-452H21.2 | -1.357050118 | 0.389870866 | | down |
| SPHKAP | -1.356910511 | 0.142025923 | 0.25884645 | down |
| ANXA2P3 | -1.356746076 | 0.526620396 | | down |
| TRIM54 | -1.356714019 | 0.000242455 | 0.001961316 | down |
| RPL32P31 | -1.356482949 | 0.629058998 | | down |
| RP1-288H2.2 | -1.356306192 | 0.289013362 | | down |
| RP11-745L13.2 | -1.356295302 | 0.315323394 | | down |
| RP11-659P15.1 | -1.356240897 | 0.452312189 | | down |
| GSTO2 | -1.356161663 | 3.16E-06 | 5.42E-05 | down |
| RPS3AP24 | -1.356118427 | 0.61930018 | | down |
| RP11-392017.1 | -1.355974186 | 0.026669492 | 0.076918326 | down |
| ANKRD7 | -1.355861152 | 0.032245144 | 0.088448199 | down |
| RP11-55605.6 | -1.355767565 | 0.443381199 | | down |
| RP11-186F10.2 | -1.355719343 | 0.40817095 | | down |
| AP001619.2 | -1.355644731 | 0.108704384 | 0.21470946 | down |
| TSEN2P1 | -1.355467251 | 0.643137227 | | down |
| NA | -1.355467251 | 0.643137227 | | down |
| MIR139 | -1.355467251 | 0.643137227 | | down |
| RP11-483E23.3 | -1.355467251 | 0.643137227 | | down |
| ITPKB-IT1 | -1.355289776 | 0.014357314 | 0.04831442 | down |
| RP11-162012.1 | -1.355280288 | 0.615609985 | | down |
| RP11-437L7.1 | -1.355276263 | 0.643159022 | | down |
| RP13-401N8.1 | -1.35526199 | 0.002324705 | 0.011868045 | down |
| CA7 | -1.354870099 | 0.002656953 | 0.013181226 | down |
| RP11-91I20.3 | -1.354801821 | 0.008741451 | 0.03307243 | down |
| TCERG1L-AS1 | -1.354667464 | 0.643333443 | | down |

| | | | | |
|---|---|---|---|---|
| LINC00377 | -1.354657349 | 0.221948048 | 0.35378858 | down |
| CDRT1 | -1.354572951 | 0.025273868 | 0.073893351 | down |
| RPL7AP14 | -1.354558101 | 0.293451391 | | down |
| RP11-451G4.2 | -1.354436364 | 0.103221341 | 0.206499062 | down |
| RP11-278J6.4 | -1.354229361 | 0.068492416 | 0.153910706 | down |
| RP13-870H17.3 | -1.354224522 | 0.000380538 | 0.002806992 | down |
| SNORD2 | -1.354029168 | 0.643038912 | | down |
| WI2-87327B8.1 | -1.353993212 | 0.616720348 | | down |
| CCL23 | -1.353992503 | 0.001548099 | 0.00854983 | down |
| CLIP2 | -1.353869588 | 7.44E-10 | 3.97E-08 | down |
| RP11-561C5.4 | -1.353827907 | 0.134310718 | | down |
| RP11-99E15.3 | -1.353790628 | 0.643397349 | | down |
| ZNF556 | -1.353696706 | 0.098762254 | 0.200257351 | down |
| RP11-26M5.3 | -1.353641084 | 0.6157741 | | down |
| PLEKHG7 | -1.353626246 | 0.000640126 | 0.004244525 | down |
| ZNF385C | -1.353552702 | 2.04E-08 | 7.22E-07 | down |
| ALPPL2 | -1.353486459 | 0.041256136 | 0.106312664 | down |
| MT-ND4 | -1.35322783 | 0.00343073 | 0.016120244 | down |
| RP11-226L15.1 | -1.353209143 | 0.489011626 | | down |
| COL22A1 | -1.353148546 | 0.000448523 | 0.003192472 | down |
| LINC01312 | -1.353096056 | 0.624143845 | | down |
| RP11-770E5.1 | -1.353053345 | 0.072049559 | 0.159662395 | down |
| MTUS2-AS2 | -1.353019123 | 0.643529872 | | down |
| DRD2 | -1.352933439 | 0.008977676 | 0.033773641 | down |
| MED15P8 | -1.352320886 | 0.643701663 | | down |
| RP11-1029M24.1 | -1.35227622 | 0.096394485 | | down |
| RP11-529E10.7 | -1.352098912 | 0.448373993 | | down |
| RP11-64C12.3 | -1.351880466 | 0.028191432 | 0.080214355 | down |
| ZNF410 | -1.351841981 | 0.004280437 | 0.01921145 | down |
| SMIM9 | -1.351779013 | 0.445535348 | | down |
| MVD | -1.351769006 | 2.67E-05 | 0.000318368 | down |
| NA | -1.351505353 | 0.020576981 | 0.063400481 | down |
| KAZALD1 | -1.351380581 | 4.29E-08 | 1.37E-06 | down |
| ADH1C | -1.351224214 | 0.000631485 | 0.004203933 | down |
| RP11-91N2.3 | -1.351215086 | 0.630219592 | | down |
| RP11-134K13.2 | -1.351206648 | 0.131248633 | 0.24495179 | down |
| RP1-163G9.2 | -1.351193285 | 0.334642961 | | down |
| GPR12 | -1.35103997 | 0.159413627 | 0.280608332 | down |
| RP4-635A23.3 | -1.350993126 | 0.579430786 | | down |
| HOXC13-AS | -1.35086777 | 0.644207565 | | down |
| NA | -1.35086777 | 0.644207565 | | down |
| HMGN2P9 | -1.35086777 | 0.644207565 | | down |
| RP11-126H7.4 | -1.350836233 | 0.401701367 | | down |
| RP11-280H21.1 | -1.350786274 | 0.644285991 | | down |
| AP000302.58 | -1.350732416 | 0.142555648 | | down |
| RP11-350G24.2 | -1.35070939 | 0.238911636 | 0.372353645 | down |
| ZNF483 | -1.350620439 | 1.06E-05 | 0.00014828 | down |
| GCOM2 | -1.350170815 | 0.098732145 | | down |
| REG1CP | -1.350117432 | 0.63000036 | | down |
| RP11-80I15.4 | -1.350059155 | 0.408243309 | | down |
| RP1-7G5.6 | -1.3499347 | 0.491309807 | | down |

| | | | | |
|---|---|---|---|---|
| RP11-874J12.4 | -1.349905309 | 0.488843397 | | down |
| RP1-228H13.2 | -1.349863708 | 0.643733076 | | down |
| CACNG8 | -1.349794983 | 0.005319454 | 0.022762809 | down |
| LRRC45 | -1.349753576 | 4.53E-06 | 7.32E-05 | down |
| RP11-479O16.1 | -1.349566566 | 0.446755323 | | down |
| RHCG | -1.349539773 | 0.005448637 | 0.023170396 | down |
| AC090616.2 | -1.349425421 | 0.006869887 | 0.027601595 | down |
| RAC1P5 | -1.349266932 | 0.147112709 | | down |
| AC007193.6 | -1.348953968 | 0.57949552 | | down |
| GPR26 | -1.348947662 | 0.167517989 | 0.290667657 | down |
| RP3-470B24.5 | -1.348886128 | 0.066158866 | 0.149979398 | down |
| DYNC1I1 | -1.348735405 | 0.002933327 | 0.014250658 | down |
| GUCY2C | -1.348649045 | 0.000616464 | 0.004124295 | down |
| RP11-187A9.3 | -1.348424755 | 0.066424281 | 0.150365514 | down |
| EIF4EBP1P1 | -1.348405895 | 0.628998848 | | down |
| TEX14 | -1.348278496 | 0.006129884 | 0.025370744 | down |
| RPL3P1 | -1.348276727 | 0.062831904 | 0.144597604 | down |
| CTD-2373J6.1 | -1.348216009 | 0.013633714 | 0.046408781 | down |
| NA | -1.348023688 | 0.58606929 | | down |
| BTBD18 | -1.348020066 | 0.115236172 | 0.22355237 | down |
| HTATSF1P1 | -1.347967643 | 0.574604123 | | down |
| RP11-554D15.3 | -1.347957362 | 0.539452697 | | down |
| DHRS2 | -1.347779283 | 0.00653348 | 0.026608327 | down |
| TUBB8P8 | -1.347712386 | 0.408893858 | | down |
| FAM74A4 | -1.347621323 | 0.421204921 | | down |
| NA | -1.347578674 | 0.032490283 | 0.08895007 | down |
| RN7SL851P | -1.347564102 | 0.48975988 | | down |
| TMEM25 | -1.347319823 | 8.48E-06 | 0.000123237 | down |
| MMACHC | -1.34718407 | 1.72E-07 | 4.54E-06 | down |
| RP11-550A5.2 | -1.34704851 | 0.097327006 | | down |
| RP11-48B14.1 | -1.346799296 | 0.002554194 | 0.012785441 | down |
| RDH12 | -1.346790246 | 0.049247202 | 0.121062612 | down |
| RP11-350J20.5 | -1.346784314 | 0.04228739 | 0.108347034 | down |
| RP11-53I6.3 | -1.346686815 | 0.018570661 | 0.058747631 | down |
| RP11-110G21.2 | -1.346555931 | 0.079155159 | 0.170884802 | down |
| CPSF4L | -1.346409053 | 0.08584582 | 0.181451515 | down |
| RP5-902P8.12 | -1.3464089 | 0.00127891 | 0.007356612 | down |
| AC090954.5 | -1.346362981 | 0.188069375 | 0.314888336 | down |
| NA | -1.346353411 | 0.224867073 | 0.357002026 | down |
| CTC-470C15.1 | -1.346349502 | 0.645110994 | | down |
| RP11-210L7.3 | -1.346123492 | 0.285262606 | 0.422292669 | down |
| KB-1507C5.3 | -1.345957645 | 0.20640693 | 0.336441504 | down |
| RP11-61N20.3 | -1.345878003 | 0.178204926 | 0.303342141 | down |
| Metazoa_SRP | -1.345582636 | 0.421543538 | 0.557162204 | down |
| ARHGEF16 | -1.345423545 | 2.28E-06 | 4.14E-05 | down |
| RP11-374N7.2 | -1.345262486 | 0.645585409 | | down |
| RP5-881L22.6 | -1.345208897 | 0.280957014 | | down |
| SPOCK3 | -1.345183934 | 0.031987865 | 0.087935165 | down |
| KIF4B | -1.345072581 | 0.528872634 | | down |
| CHMP4BP1 | -1.345007811 | 0.000287757 | 0.002255513 | down |
| RP11-495P10.4 | -1.34482814 | 0.629510046 | | down |

| | | | | |
|---|---|---|---|---|
| RP11-295K2.3 | -1.344783628 | 0.003182034 | 0.015160327 | down |
| RP11-227D13.1 | -1.344748067 | 0.03316195 | 0.090254337 | down |
| SDCBP2 | -1.344646542 | 4.70E-05 | 0.000507264 | down |
| RP11-671M22.4 | -1.344118913 | 0.111871355 | 0.219002269 | down |
| GABRA3 | -1.344085711 | 0.045345397 | 0.113833314 | down |
| ANXA13 | -1.344076246 | 0.000878165 | 0.005456794 | down |
| CBR1 | -1.344005812 | 1.34E-06 | 2.62E-05 | down |
| CTA-407F11.8 | -1.344005331 | 0.151203259 | 0.270343459 | down |
| LINC00469 | -1.343778863 | 0.533322997 | | down |
| RP11-46H11.11 | -1.343426555 | 0.012418436 | 0.043252898 | down |
| CTNNA3 | -1.343409682 | 0.002896532 | 0.014111139 | down |
| CCER2 | -1.343283437 | 0.000201241 | 0.001678537 | down |
| IDSP1 | -1.343261127 | 0.036486478 | 0.09699166 | down |
| RP11-40A7.2 | -1.343221808 | 0.027314485 | 0.078342375 | down |
| AC004012.1 | -1.34305138 | 0.071322017 | 0.158455866 | down |
| RN7SL316P | -1.342893501 | 0.450457134 | | down |
| MCM8-AS1 | -1.342853429 | 0.142110776 | | down |
| NDUFA3P4 | -1.342602909 | 0.094494496 | 0.194238686 | down |
| CTD-2033C11.1 | -1.3425391 | 0.00213386 | 0.011057209 | down |
| GCM1 | -1.342482904 | 0.099194128 | 0.200943142 | down |
| RP11-219B4.3 | -1.342481906 | 0.158140611 | | down |
| RP11-457I16.2 | -1.34234796 | 0.583911383 | | down |
| HNRNPA1P34 | -1.342326365 | 0.627139285 | | down |
| LINC00363 | -1.342281447 | 0.64637533 | | down |
| RP11-529H22.1 | -1.342267099 | 0.255454066 | 0.390537553 | down |
| NA | -1.342224062 | 0.342813464 | | down |
| MIR3622A | -1.341823753 | 0.013264933 | 0.045411537 | down |
| MAB21L3 | -1.341697468 | 0.018745134 | 0.059126385 | down |
| NDUFV1 | -1.341672729 | 2.46E-07 | 6.17E-06 | down |
| NA | -1.341636363 | 0.400540688 | | down |
| SPATA9 | -1.341611717 | 0.008108525 | 0.031232373 | down |
| RPS26P13 | -1.341420022 | 0.002077732 | 0.010814861 | down |
| AC005794.1 | -1.341184523 | 0.646322723 | | down |
| RYKP1 | -1.341117195 | 0.141726776 | 0.258438897 | down |
| FOXN4 | -1.341046714 | 0.078298345 | 0.169505367 | down |
| ST6GAL2 | -1.341043403 | 0.013213758 | 0.045272455 | down |
| CTB-91J4.1 | -1.340975799 | 0.410159557 | | down |
| HTR1DP1 | -1.340672307 | 0.39299174 | | down |
| RP11-632K20.6 | -1.340595279 | 0.024136 | 0.071428485 | down |
| LINC00668 | -1.340278879 | 0.000480647 | 0.003366095 | down |
| CCDC170 | -1.340233056 | 1.82E-06 | 3.41E-05 | down |
| POLRMTP1 | -1.339714987 | 3.93E-05 | 0.000438367 | down |
| RPL7P20 | -1.339624244 | 0.354988421 | | down |
| FGFR4 | -1.33944407 | 4.30E-06 | 7.01E-05 | down |
| ATP6V1G3 | -1.33939024 | 0.180260699 | | down |
| TOM1L1 | -1.339263742 | 5.26E-06 | 8.27E-05 | down |
| ADCK5 | -1.339082129 | 8.59E-08 | 2.51E-06 | down |
| NA | -1.338830578 | 2.76E-05 | 0.000326513 | down |
| TMEM145 | -1.338781976 | 0.000174312 | 0.001492035 | down |
| OR8G5 | -1.338660603 | 0.405676336 | | down |
| CTA-929C8.5 | -1.338626807 | 0.622775337 | | down |

| | | | | |
|---|---|---|---|---|
| AC006499.3 | -1.338559144 | 0.647290642 | | down |
| RP11-572M11.1 | -1.338507382 | 0.294521645 | | down |
| AC135050.5 | -1.338373788 | 0.19203767 | | down |
| OR7G3 | -1.338363542 | 0.647338755 | | down |
| NA | -1.338283011 | 0.218916453 | 0.350368949 | down |
| CLDND2 | -1.338138296 | 1.83E-05 | 0.000232693 | down |
| GCAT | -1.338099808 | 4.72E-06 | 7.58E-05 | down |
| CTC-340D7.1 | -1.337705514 | 0.021104262 | 0.064627097 | down |
| TLDC2 | -1.337633766 | 7.73E-07 | 1.63E-05 | down |
| RP11-558F24.2 | -1.337254361 | 0.355504349 | 0.494114206 | down |
| FAR2P1 | -1.337132774 | 0.420941507 | 0.556674791 | down |
| RP11-595B24.2 | -1.337051565 | 0.01139904 | 0.040586959 | down |
| LINC01359 | -1.337019649 | 0.005532305 | 0.023430022 | down |
| CYP4Z1 | -1.336883393 | 0.204430573 | | down |
| RP3-405J10.3 | -1.336804954 | 0.004816197 | 0.021092978 | down |
| FNDC5 | -1.33667605 | 0.000235453 | 0.001913945 | down |
| RP11-462G12.1 | -1.336254737 | 0.009116733 | 0.03416103 | down |
| PHBP20 | -1.336225504 | 0.57675367 | | down |
| MT-RNR1 | -1.336205727 | 0.000997175 | 0.006048682 | down |
| RP11-249M12.2 | -1.336100657 | 0.479829143 | 0.609218984 | down |
| AC068134.6 | -1.335876785 | 0.490529234 | | down |
| RP11-191L9.5 | -1.33555941 | 0.648028661 | | down |
| RP11-981G7.3 | -1.335535838 | 0.175908186 | | down |
| TCEA3 | -1.335404384 | 1.48E-06 | 2.86E-05 | down |
| CICP17 | -1.335078647 | 0.537470703 | | down |
| NA | -1.33500091 | 0.366197681 | | down |
| FIRRE | -1.334741136 | 0.002100725 | 0.010908086 | down |
| PPIAP3 | -1.334592051 | 0.153200465 | | down |
| OR5B2 | -1.334430781 | 0.393455245 | | down |
| VN1R74P | -1.334326078 | 0.533802929 | | down |
| KRT18P39 | -1.333990526 | 0.025739971 | 0.07489457 | down |
| IFITM8P | -1.333794059 | 0.631571193 | | down |
| KCCAT333 | -1.333586103 | 0.350147326 | | down |
| CTD-2187J20.1 | -1.333466817 | 0.341127414 | | down |
| SST | -1.333373909 | 0.000241706 | 0.001956838 | down |
| FLJ42393 | -1.333366916 | 0.043896235 | 0.111255145 | down |
| CTD-2089N3.2 | -1.333347117 | 0.178349191 | 0.303432986 | down |
| U1 | -1.333315194 | 0.166219832 | | down |
| AGAP11 | -1.333137233 | 0.236171343 | 0.369135981 | down |
| MT-CO3 | -1.33311044 | 0.001230801 | 0.00715107 | down |
| RP11-239A17.1 | -1.332373149 | 0.454714466 | | down |
| RORC | -1.332304794 | 5.29E-07 | 1.18E-05 | down |
| RPL35AP | -1.332248733 | 0.448440293 | 0.581406817 | down |
| NA | -1.332178193 | 0.083350473 | 0.177463085 | down |
| ARL17B | -1.331880897 | 0.000400502 | 0.00291914 | down |
| EEF1GP2 | -1.331877877 | 0.296304837 | 0.433781078 | down |
| AC092652.1 | -1.331806676 | 0.021298539 | 0.065029808 | down |
| ZSWIM1 | -1.33174228 | 1.71E-08 | 6.28E-07 | down |
| RP11-168K11.5 | -1.331614938 | 0.374127057 | | down |
| KLF2P3 | -1.331533933 | 0.63216746 | | down |
| RP11-661A12.9 | -1.331465498 | 0.03453409 | 0.093204652 | down |

| | | | | |
|---|---|---|---|---|
| CTSD | -1.331371898 | 4.04E-05 | 0.000448123 | down |
| RP11-343C2.10 | -1.331338983 | 0.455842776 | | down |
| RP11-758M4.4 | -1.331279101 | 0.296280092 | | down |
| CTC-573N18.1 | -1.331117604 | 0.142985899 | 0.260134936 | down |
| NA | -1.331090585 | 0.11247303 | 0.219635969 | down |
| ZFAND2B | -1.331009653 | 8.46E-08 | 2.48E-06 | down |
| RP11-561N12.5 | -1.330975383 | 0.631198423 | | down |
| RP11-141J13.5 | -1.330971281 | 0.234494564 | 0.367279464 | down |
| AC064834.1 | -1.330624489 | 0.498917539 | | down |
| AC005033.6 | -1.330282116 | 0.621322426 | | down |
| PLSCR2 | -1.330031872 | 0.068662607 | 0.154131752 | down |
| RP11-325E14.5 | -1.330003513 | 0.306445058 | | down |
| ABCF2P1 | -1.329921732 | 0.500292634 | | down |
| TERB1 | -1.32976043 | 0.299189403 | 0.436775822 | down |
| CD302 | -1.329556956 | 0.000410467 | 0.002967291 | down |
| MIR606 | -1.329318285 | 0.649511248 | | down |
| RP11-404K5.3 | -1.329217082 | 0.649590181 | | down |
| RP3-400B16.2 | -1.329217082 | 0.649590181 | | down |
| RP11-521D12.1 | -1.329200541 | 0.059218301 | 0.138423296 | down |
| NA | -1.329131674 | 0.375814054 | | down |
| NA | -1.329002488 | 0.623818543 | | down |
| KLKB1 | -1.329001764 | 2.00E-06 | 3.71E-05 | down |
| CLIC5 | -1.328662041 | 2.07E-07 | 5.31E-06 | down |
| SKOR1 | -1.328635787 | 2.15E-05 | 0.000265473 | down |
| RP11-243J18.2 | -1.328389646 | 0.586585747 | | down |
| NEK10 | -1.328373374 | 0.007989639 | 0.030949036 | down |
| NXF2B | -1.328311926 | 0.374292921 | 0.512686082 | down |
| ABO | -1.328263165 | 7.60E-07 | 1.61E-05 | down |
| KIF25-AS1 | -1.328124847 | 0.125814036 | 0.237746121 | down |
| CRISP3 | -1.327992407 | 0.256785883 | 0.391836816 | down |
| RP11-1086F11.1 | -1.327802779 | 0.206838384 | 0.336888888 | down |
| RP11-144G16.1 | -1.32772957 | 0.516053836 | 0.641127699 | down |
| GAPDHP58 | -1.327542971 | 0.649977958 | | down |
| ZNF99 | -1.327502284 | 0.110355351 | 0.216898873 | down |
| RP11-53I6.1 | -1.327306515 | 0.297230384 | | down |
| RP1-140A9.1 | -1.32724353 | 0.000209087 | 0.001733388 | down |
| RP11-95O2.5 | -1.327186182 | 0.079563722 | 0.171454057 | down |
| B3GALT1 | -1.327083237 | 0.002055485 | 0.010728803 | down |
| TUBBP9 | -1.326840416 | 0.380092635 | | down |
| RP11-203M5.6 | -1.326760859 | 0.09286635 | 0.19162614 | down |
| RP11-210K20.2 | -1.326680607 | 0.260003955 | 0.395150778 | down |
| XXbac-B476C20.9 | -1.326562729 | 7.40E-06 | 0.000109973 | down |
| PRB3 | -1.326430624 | 0.096231646 | 0.196652398 | down |
| NA | -1.326308368 | 0.650306836 | | down |
| KRT17P5 | -1.326223898 | 0.584455457 | | down |
| NOXA1 | -1.325843584 | 8.28E-07 | 1.73E-05 | down |
| RP11-617B3.2 | -1.325766868 | 0.639520372 | | down |
| NA | -1.325687204 | 0.102298989 | 0.205328429 | down |
| ADAMDEC1 | -1.32543985 | 7.57E-05 | 0.000750002 | down |
| RP11-346L1.2 | -1.324895062 | 0.451499813 | | down |
| GAPDHP47 | -1.324281012 | 0.418346716 | | down |

| | | | | |
|---|---|---|---|---|
| SLC22A4 | -1.324252679 | 0.001044785 | 0.006281165 | down |
| RP11-386D6.1 | -1.324225046 | 0.585137602 | | down |
| HAS3 | -1.324013611 | 0.000416851 | 0.003006207 | down |
| RPS3AP25 | -1.323914563 | 0.001343242 | 0.007650495 | down |
| MTCO3P5 | -1.323172948 | 0.150731806 | 0.269944347 | down |
| OVOL2 | -1.322646937 | 5.98E-06 | 9.22E-05 | down |
| AC012360.4 | -1.322637271 | 0.091127011 | 0.18916587 | down |
| RP11-476I15.5 | -1.322413519 | 0.627298484 | | down |
| ZNF603P | -1.322346418 | 0.248686427 | 0.383155642 | down |
| NA | -1.32189562 | 0.002417782 | 0.012250884 | down |
| RPL36AP26 | -1.321885146 | 0.100833721 | 0.203168689 | down |
| RPL36AP33 | -1.321873172 | 0.651209858 | | down |
| RP11-67L14.1 | -1.321856503 | 0.197805289 | 0.326512944 | down |
| DECR2 | -1.321812846 | 1.47E-08 | 5.57E-07 | down |
| NDUFB8P2 | -1.321805863 | 0.040534244 | 0.104946154 | down |
| NA | -1.321779186 | 0.582308189 | | down |
| LO9NC01-251B2.[illegible] | -1.32176608 | 0.000808897 | 0.005126615 | down |
| DNAH10OS | -1.321509137 | 0.000966056 | 0.005889646 | down |
| LINC01143 | -1.321389838 | 0.58844324 | | down |
| RP11-309M7.1 | -1.321273227 | 0.440452949 | 0.574198056 | down |
| NPAP1P4 | -1.32096426 | 0.540874497 | | down |
| AIRN | -1.320797685 | 0.41892197 | | down |
| C12orf42 | -1.320717335 | 0.003433966 | 0.016132929 | down |
| AC007256.5 | -1.3206996 | 0.139585027 | 0.255790946 | down |
| CEACAM19 | -1.320409513 | 1.46E-07 | 3.94E-06 | down |
| RP11-566K19.6 | -1.320110439 | 0.005484543 | 0.023266325 | down |
| RP5-933K21.2 | -1.320005168 | 0.501531347 | | down |
| RP11-402J6.3 | -1.319714306 | 0.011830792 | 0.041729708 | down |
| RP1-228P16.5 | -1.319266653 | 0.229217154 | 0.361653197 | down |
| RP11-524F11.2 | -1.319209012 | 0.500179811 | | down |
| STAC2 | -1.318786374 | 0.002841959 | 0.013892583 | down |
| NDUFA9 | -1.318617178 | 0.000117949 | 0.001088681 | down |
| DAZAP2P1 | -1.31861063 | 0.087272468 | 0.183460594 | down |
| AC009961.3 | -1.318609501 | 0.00021048 | 0.001743979 | down |
| RP11-491F9.3 | -1.318312877 | 0.003815166 | 0.017550581 | down |
| LINC00636 | -1.318299165 | 0.15054885 | 0.269697075 | down |
| CTAGE4 | -1.318237907 | 0.00313312 | 0.014978766 | down |
| RNU6-1099P | -1.318165671 | 0.210316172 | | down |
| CTD-2293H3.2 | -1.318140338 | 0.271355211 | | down |
| ERBB2 | -1.318120952 | 1.86E-08 | 6.68E-07 | down |
| RNA5SP69 | -1.318085098 | 0.300865866 | | down |
| AP001476.3 | -1.318069157 | 0.181967965 | | down |
| AC073342.12 | -1.317986553 | 0.318555856 | | down |
| RP11-168O16.2 | -1.317586046 | 0.366483948 | | down |
| KIAA2022 | -1.317545146 | 0.000542417 | 0.003718084 | down |
| RP11-542C10.1 | -1.317528678 | 0.397274313 | 0.534275267 | down |
| IGLVIVOR22-1 | -1.317253285 | 0.652539875 | | down |
| KB-1958F4.1 | -1.317010948 | 0.3595664 | | down |
| RP1-41C23.1 | -1.316743708 | 0.385812203 | | down |
| MIR3131 | -1.316689758 | 0.094424365 | 0.19410781 | down |
| MTCO1P39 | -1.316559523 | 0.652405471 | | down |

| | | | | |
|---|---|---|---|---|
| RP11-431M7.3 | -1.31640397 | 0.017332555 | 0.055682131 | down |
| RPLP1P13 | -1.316369842 | 0.428886063 | | down |
| CTC-398G3.1 | -1.31636291 | 0.03057916 | 0.08497228 | down |
| CFC1 | -1.316275935 | 0.413976171 | | down |
| PNPLA6 | -1.316173376 | 1.32E-05 | 0.000177539 | down |
| NA | -1.316054112 | 0.231498412 | | down |
| SFTA1P | -1.315924487 | 0.000507855 | 0.00352135 | down |
| NA | -1.315658645 | 0.652933407 | | down |
| SECTM1 | -1.315558175 | 2.53E-05 | 0.000304109 | down |
| RP4-809F18.1 | -1.31529123 | 0.02868834 | 0.081258837 | down |
| FLT3LG | -1.315070102 | 0.000442727 | 0.003156248 | down |
| RPTN | -1.314998574 | 0.624514362 | | down |
| FTLP14 | -1.314970925 | 0.004132167 | 0.018719367 | down |
| HMGB3P3 | -1.314938599 | 0.504747565 | | down |
| MIR210 | -1.31488132 | 0.052582584 | 0.126758498 | down |
| RP11-824M15.3 | -1.314821626 | 0.135571633 | 0.250468217 | down |
| LINC01301 | -1.314701965 | 0.007708123 | 0.030122638 | down |
| PPIAP27 | -1.314658076 | 0.470265161 | 0.600912151 | down |
| NUTM2G | -1.314371038 | 0.006363922 | 0.026098509 | down |
| Y_RNA | -1.314311842 | 0.373970069 | | down |
| FRMPD1 | -1.314034685 | 0.004402161 | 0.019655004 | down |
| POTEI | -1.313905291 | 0.188305459 | | down |
| RP11-276H1.3 | -1.31243102 | 0.14994086 | 0.268896508 | down |
| RP11-641A6.5 | -1.311820201 | 0.351385554 | | down |
| RP11-715J22.6 | -1.311750712 | 0.003576525 | 0.01666224 | down |
| VILL | -1.311433807 | 3.10E-07 | 7.45E-06 | down |
| CEL | -1.311276466 | 0.000226605 | 0.001856086 | down |
| RP4-597J3.1 | -1.311268548 | 0.584741755 | | down |
| RPL3P9 | -1.311115691 | 0.078499474 | | down |
| YWHABP2 | -1.310885825 | 0.102943483 | 0.206188933 | down |
| RP11-307I2.1 | -1.310881784 | 0.653633713 | | down |
| GPR39 | -1.310799857 | 0.004094479 | 0.018579449 | down |
| RP3-343K2.4 | -1.310755322 | 0.504038608 | | down |
| DNAJC22 | -1.310727336 | 2.22E-06 | 4.05E-05 | down |
| NOSIP | -1.31067644 | 2.56E-06 | 4.55E-05 | down |
| AC008937.3 | -1.310559295 | 0.588552234 | | down |
| SNORA44 | -1.310501325 | 0.381433199 | 0.519270426 | down |
| RP11-6L6.6 | -1.310344293 | 0.351124575 | | down |
| NA | -1.310301905 | 0.223155612 | 0.355204818 | down |
| RP11-170N16.3 | -1.310127916 | 0.001023583 | 0.006182652 | down |
| RP4-800G7.1 | -1.310050443 | 0.004482049 | 0.019944034 | down |
| CTD-2639E6.9 | -1.310031926 | 2.47E-05 | 0.000297732 | down |
| OR52K1 | -1.31000655 | 0.654241281 | | down |
| LINC00686 | -1.309972834 | 0.654036359 | | down |
| FTOP1 | -1.309599251 | 0.001822386 | 0.009742634 | down |
| RP11-377D9.3 | -1.309574065 | 0.072851423 | 0.160961035 | down |
| U8 | -1.309322683 | 0.654447829 | | down |
| RP1-97G4.1 | -1.309322683 | 0.654447829 | | down |
| ZNF645 | -1.309322683 | 0.654447829 | | down |
| CA13 | -1.309270807 | 4.95E-06 | 7.87E-05 | down |
| ANO5 | -1.309239421 | 1.90E-06 | 3.55E-05 | down |

| | | | | |
|---|---|---|---|---|
| SNORA31 | -1.309002768 | 0.654526923 | | down |
| RP11-144O23.22 | -1.309002768 | 0.654526923 | | down |
| DSCR10 | -1.309002768 | 0.654526923 | | down |
| L3MBTL4-AS1 | -1.308879656 | 0.008613533 | 0.032703777 | down |
| HNRNPCP7 | -1.308532852 | 0.055433823 | 0.131771008 | down |
| RP11-269F21.3 | -1.308515721 | 0.640018477 | | down |
| KRT18P8 | -1.308488665 | 0.092154533 | 0.190654923 | down |
| RBM22P2 | -1.30835479 | 0.020526181 | 0.06331505 | down |
| AC010492.2 | -1.308194577 | 0.097950807 | 0.199095735 | down |
| AC099344.3 | -1.308103709 | 0.640953424 | | down |
| TNRC18P3 | -1.307959377 | 0.406093153 | 0.542661686 | down |
| CTC-325H20.7 | -1.30781628 | 0.389858829 | | down |
| POU5F1P3 | -1.30776625 | 0.040848451 | 0.105506437 | down |
| RPH3A | -1.307637096 | 0.003579841 | 0.016672258 | down |
| ARF4-AS1 | -1.307440349 | 0.022527695 | 0.067777096 | down |
| DDTP1 | -1.307385753 | 0.641770943 | | down |
| CASKIN1 | -1.307343874 | 0.043656711 | 0.11088192 | down |
| CCDC151 | -1.307223824 | 8.61E-05 | 0.000834335 | down |
| CIDEA | -1.307205329 | 0.022281094 | 0.067216907 | down |
| RNA5SP466 | -1.307093756 | 0.0842706 | 0.178788637 | down |
| RP11-124O11.1 | -1.30699716 | 0.424065568 | | down |
| GRIP1 | -1.306953351 | 0.000616286 | 0.004124295 | down |
| MIR4526 | -1.30688622 | 0.639983513 | | down |
| CTD-3099C6.9 | -1.306781658 | 0.000304019 | 0.002356536 | down |
| C7orf34 | -1.306632423 | 0.262044945 | | down |
| SGO1-AS1 | -1.306603779 | 0.01334 | 0.045632127 | down |
| TRMT112P1 | -1.306526217 | 0.65513933 | | down |
| NA | -1.306478635 | 0.654902195 | | down |
| RPL19P13 | -1.306448096 | 0.636818358 | | down |
| CH17-258A22.4 | -1.305635701 | 0.029405273 | 0.082664117 | down |
| CNTN6 | -1.305330684 | 0.087879943 | 0.18433743 | down |
| SLC35E1P1 | -1.305278274 | 0.399216842 | | down |
| RP11-90O23.1 | -1.305271047 | 0.13732497 | | down |
| CTD-3064M3.4 | -1.305246108 | 0.233549614 | 0.366219447 | down |
| RPL19P21 | -1.305223153 | 0.074335672 | 0.163243982 | down |
| CTD-3096M3.1 | -1.305220249 | 0.504741402 | | down |
| KRTAP5-11 | -1.305153451 | 0.591063993 | | down |
| HMGB1P29 | -1.305090711 | 0.548039493 | | down |
| RGPD3 | -1.305023039 | 0.021356519 | 0.065142169 | down |
| SLC37A4 | -1.304830972 | 1.38E-09 | 6.83E-08 | down |
| RP11-113O24.3 | -1.304723369 | 0.216546919 | | down |
| RP11-80P20.3 | -1.304423763 | 0.017879272 | 0.057090535 | down |
| MTATP8P2 | -1.304273774 | 0.018162602 | 0.057774189 | down |
| RPL21P10 | -1.304096349 | 0.222962287 | 0.35498934 | down |
| DMRTA1 | -1.303858406 | 0.001051224 | 0.00631407 | down |
| C5orf66-AS2 | -1.303778094 | 0.505075679 | | down |
| Y_RNA | -1.302729032 | 0.656042035 | | down |
| CDC42EP5 | -1.302558055 | 4.02E-05 | 0.000446204 | down |
| CTC-255N20.1 | -1.30255409 | 0.144102445 | 0.261596003 | down |
| GRAMD1C | -1.302306702 | 8.27E-05 | 0.000807893 | down |
| CTB-55O6.13 | -1.302062986 | 0.062335455 | 0.143774482 | down |

| | | | | |
|---|---|---|---|---|
| TMEM56 | -1.30204417 | 2.31E-06 | 4.17E-05 | down |
| AC093375.1 | -1.302033699 | 0.019520127 | 0.060905877 | down |
| CTD-2568P8.1 | -1.301772813 | 0.548310846 | | down |
| ATP6V0A1 | -1.30171158 | 1.15E-05 | 0.000158502 | down |
| DIP2A-IT1 | -1.301660837 | 0.105030645 | | down |
| MAJIN | -1.301520772 | 0.020915423 | 0.064199322 | down |
| NA | -1.301455965 | 0.46478633 | 0.596320643 | down |
| AC005102.1 | -1.301419515 | 0.208239679 | | down |
| LTC4S | -1.301379542 | 6.66E-05 | 0.000674606 | down |
| CTD-2574D22.2 | -1.301274457 | 0.016586864 | 0.053845029 | down |
| FAM196A | -1.301267335 | 0.004935357 | 0.021473256 | down |
| IGFBP2 | -1.301236133 | 3.77E-05 | 0.000423893 | down |
| RP11-320N21.2 | -1.301182363 | 0.070691467 | 0.157481252 | down |
| RP11-459I19.1 | -1.301107542 | 0.008497794 | 0.032366597 | down |
| RP11-864I4.3 | -1.300988889 | 0.049981611 | 0.122285995 | down |
| RP11-329J18.3 | -1.300895567 | 0.400173434 | | down |
| RP11-120J1.1 | -1.300873298 | 0.075575541 | 0.165206021 | down |
| LDLRAD1 | -1.300856448 | 0.186827174 | | down |
| RP11-119H12.4 | -1.300837537 | 0.642101082 | | down |
| AC079584.2 | -1.300680219 | 0.641228613 | | down |
| RP11-86708.5 | -1.30061759 | 0.006531936 | 0.026605951 | down |
| CTC-529I10.1 | -1.300589787 | 0.003598076 | 0.01673643 | down |
| ZNF20 | -1.300505076 | 0.001225658 | 0.007131174 | down |
| Y_RNA | -1.300483365 | 0.656447999 | | down |
| KRT8P7 | -1.300328143 | 0.067605055 | 0.152372564 | down |
| RP11-545M17.2 | -1.300226737 | 0.305544633 | | down |
| RP11-83A24.2 | -1.300216127 | 1.59E-07 | 4.25E-06 | down |
| AC097359.2 | -1.299879305 | 0.17906026 | | down |
| RP11-1277A3.2 | -1.299870126 | 0.003399051 | 0.015986397 | down |
| CCDC63 | -1.299834147 | 0.326609574 | 0.465010807 | down |
| MYT1 | -1.299813979 | 0.004675084 | 0.020599057 | down |
| UQCRC1 | -1.299669753 | 1.39E-07 | 3.79E-06 | down |
| RPL23AP38 | -1.299585205 | 0.269536989 | | down |
| TMEM120A | -1.299247576 | 1.63E-08 | 6.06E-07 | down |
| RP11-542M13.3 | -1.299040468 | 0.502687294 | | down |
| NA | -1.298962739 | 0.055764725 | 0.132463267 | down |
| NA | -1.298892576 | 0.422532096 | | down |
| NA | -1.298876923 | 0.261479858 | 0.39662245 | down |
| TJP3 | -1.298815262 | 9.12E-06 | 0.000131106 | down |
| IGBP1P1 | -1.298649735 | 0.293324553 | | down |
| PHACTR3 | -1.298493257 | 0.000335802 | 0.002555431 | down |
| GCG | -1.298176991 | 0.022078447 | 0.066779911 | down |
| RP13-714J12.1 | -1.298088507 | 0.002777104 | 0.013648104 | down |
| SOHLH2 | -1.298070531 | 0.202567351 | 0.331875531 | down |
| NA | -1.297941618 | 0.012757326 | 0.044146637 | down |
| RP11-753E22.2 | -1.297774394 | 0.657269145 | | down |
| RP11-795J1.1 | -1.297680515 | 0.028470674 | 0.080840463 | down |
| LINC01395 | -1.297331263 | 0.387565554 | | down |
| RNU6-375P | -1.297324093 | 0.549266418 | | down |
| RP3-476K8.4 | -1.296421044 | 0.111447766 | | down |
| RP11-61102.6 | -1.296233635 | 0.657609652 | | down |

| | | | | |
|---|---|---|---|---|
| DCAF12L2 | -1.295876823 | 0.059535117 | 0.138903696 | down |
| RP5-1116H23.4 | -1.295870347 | 0.044712734 | 0.112687643 | down |
| NA | -1.295518416 | 0.004994379 | 0.021677422 | down |
| MROH9 | -1.295425692 | 0.638596068 | | down |
| XYLB | -1.295060179 | 4.90E-09 | 2.10E-07 | down |
| C22orf23 | -1.294939004 | 0.00577654 | 0.024218583 | down |
| RP11-452D12.1 | -1.294769787 | 0.070855296 | 0.15769883 | down |
| EPS8L3 | -1.294671658 | 4.10E-05 | 0.00045332 | down |
| RGPD1 | -1.294580127 | 0.054285303 | 0.129790519 | down |
| NA | -1.294561208 | 0.347704412 | 0.486446163 | down |
| KB-1991G8.1 | -1.294401476 | 0.237018285 | | down |
| CHGB | -1.293948951 | 0.000407714 | 0.00295165 | down |
| CBSL | -1.29373089 | 0.022208807 | 0.067059436 | down |
| PXMP2 | -1.29368823 | 2.56E-05 | 0.000307338 | down |
| CTB-161C1.1 | -1.293588298 | 0.061673604 | 0.142664134 | down |
| NA | -1.293498785 | 0.161360696 | | down |
| ALG1L3P | -1.293469002 | 0.090861207 | 0.188759099 | down |
| AC072052.7 | -1.293041987 | 0.007107705 | 0.028314269 | down |
| CHRNB4 | -1.29272659 | 0.004401085 | 0.019653924 | down |
| RP4-555D20.3 | -1.292709307 | 0.27924815 | | down |
| RPL7AP71 | -1.292508748 | 0.658533376 | | down |
| CTC-325H20.8 | -1.292402055 | 0.093209987 | 0.192203215 | down |
| EBP | -1.292294895 | 2.98E-05 | 0.00034792 | down |
| RP11-217E22.5 | -1.292221914 | 0.548489145 | | down |
| RP11-160C18.5 | -1.29209721 | 0.589655339 | | down |
| PRAMEF25 | -1.291992186 | 0.658761103 | | down |
| NA | -1.291992186 | 0.658761103 | | down |
| OR2T35 | -1.291992186 | 0.658761103 | | down |
| Metazoa_SRP | -1.291992186 | 0.658761103 | | down |
| RP11-767L7.1 | -1.291992186 | 0.658761103 | | down |
| RP11-432M8.17 | -1.291992186 | 0.658761103 | | down |
| HMGB1P47 | -1.291992186 | 0.658761103 | | down |
| UQCRFS1P2 | -1.291992186 | 0.658761103 | | down |
| FAM90A12P | -1.291992186 | 0.658761103 | | down |
| RP13-461N9.2 | -1.291992186 | 0.658761103 | | down |
| RP11-328K15.1 | -1.291992186 | 0.658761103 | | down |
| FAM99B | -1.291992186 | 0.658761103 | | down |
| AP000997.3 | -1.291992186 | 0.658761103 | | down |
| TRAJ29 | -1.291992186 | 0.658761103 | | down |
| RP11-471L13.2 | -1.291992186 | 0.658761103 | | down |
| RP11-118B18.3 | -1.291992186 | 0.658761103 | | down |
| AF241725.6 | -1.291992186 | 0.658761103 | | down |
| SMARCE1P3 | -1.291897719 | 0.642597294 | | down |
| RP11-394B2.7 | -1.291893711 | 0.641932407 | | down |
| RNU5E-8P | -1.291772585 | 0.592478375 | | down |
| RP11-131H24.5 | -1.291712604 | 0.591897417 | | down |
| GRM3 | -1.291654496 | 0.122789954 | 0.233729653 | down |
| RP11-601I15.1 | -1.291603092 | 0.354090959 | | down |
| RP11-835E18.5 | -1.291601198 | 0.597215372 | | down |
| RP11-671E7.1 | -1.291545798 | 0.267389791 | | down |
| RP11-297C4.1 | -1.29142131 | 0.382967338 | | down |

| | | | | |
|---|---|---|---|---|
| RP11-89H19.2 | -1.291394793 | 0.000357944 | 0.002683824 | down |
| GSTA10P | -1.291385766 | 0.658811962 | | down |
| RP5-879K22.1 | -1.291251761 | 0.55310275 | | down |
| PCSK1N | -1.291190415 | 5.91E-05 | 0.000613426 | down |
| SNORA77 | -1.291061702 | 0.081419052 | 0.17442627 | down |
| DDX25 | -1.290965911 | 0.006740524 | 0.027230375 | down |
| MFSD4BP1 | -1.290421779 | 0.243724351 | 0.377774013 | down |
| TEPP | -1.290361947 | 0.001402816 | 0.007908842 | down |
| RP11-366L20.3 | -1.290355628 | 0.643215869 | | down |
| RP11-120E13.1 | -1.290330103 | 0.638854551 | | down |
| RP11-474D1.2 | -1.290326632 | 0.638267176 | | down |
| NA | -1.290184936 | 0.371760599 | | down |
| RP11-484D2.2 | -1.290104803 | 0.210253082 | | down |
| OSGEPL1 | -1.29006018 | 3.94E-07 | 9.12E-06 | down |
| RPGRIP1 | -1.290032669 | 0.000428321 | 0.003074018 | down |
| FAM101A | -1.289835837 | 9.25E-06 | 0.000132683 | down |
| CELF4 | -1.289615186 | 0.00205656 | 0.010730688 | down |
| LA16c-306A4.2 | -1.289438463 | 0.018882167 | 0.059433821 | down |
| NA | -1.289424459 | 0.115103831 | 0.223396609 | down |
| CALR3 | -1.289407316 | 0.500768487 | 0.627846614 | down |
| KRT18P55 | -1.289383646 | 0.322574222 | 0.461193812 | down |
| MTCO3P12 | -1.289197572 | 0.005636738 | 0.023768443 | down |
| AL163953.2 | -1.289044185 | 0.152378125 | 0.271855605 | down |
| NKX2-2 | -1.289027584 | 0.010798038 | 0.03889883 | down |
| RP11-388M20.1 | -1.288788336 | 0.001042145 | 0.006269636 | down |
| PTX4 | -1.288774654 | 0.128315497 | 0.241080033 | down |
| STK33P1 | -1.288744263 | 0.332953289 | 0.471295577 | down |
| HOXB-AS2 | -1.288590849 | 0.095560382 | 0.19563994 | down |
| CCDC181 | -1.288404663 | 0.008179296 | 0.031460605 | down |
| MLC1 | -1.288353494 | 0.014703188 | 0.049170127 | down |
| RP11-259G18.3 | -1.288235443 | 0.078239229 | 0.169462864 | down |
| RP11-288G3.4 | -1.287876219 | 0.659781043 | | down |
| AC011742.3 | -1.287709964 | 0.140152605 | | down |
| AC018804.6 | -1.287559018 | 0.008131967 | 0.031310627 | down |
| MTRNR2L8 | -1.286961491 | 0.002766944 | 0.013614825 | down |
| NA | -1.286949036 | 0.506588497 | | down |
| NA | -1.28685658 | 0.108214468 | 0.213964822 | down |
| CTD-3234P18.6 | -1.28683662 | 0.049346512 | 0.121228136 | down |
| RNU6-1300P | -1.286812687 | 0.017059298 | 0.05497051 | down |
| LINC01057 | -1.286751922 | 4.08E-05 | 0.000451786 | down |
| LINC01166 | -1.286329177 | 0.475409848 | | down |
| NRXN1 | -1.286078391 | 0.001390085 | 0.007856227 | down |
| NPM1P9 | -1.285994006 | 0.120795924 | | down |
| RP11-13K12.6 | -1.285944706 | 0.649827658 | | down |
| CTC-575I10.1 | -1.285771091 | 0.474429744 | | down |
| NA | -1.28570592 | 0.001019882 | 0.00616278 | down |
| RN7SKP255 | -1.285565231 | 0.2024682 | 0.331782718 | down |
| RP11-387A1.5 | -1.285350724 | 0.040675904 | 0.105205481 | down |
| RPS6P16 | -1.28512142 | 0.32673296 | | down |
| KBTBD13 | -1.284759368 | 0.053414379 | 0.128238901 | down |
| AC008268.1 | -1.284578159 | 0.646237292 | | down |

| | | | | |
|---|---|---|---|---|
| RP11-1020A11.2 | -1.284437125 | 4.18E-05 | 0.000460065 | down |
| HNRNPA3P2 | -1.284182377 | 0.313039123 | | down |
| CHMP1B2P | -1.284167046 | 0.010551486 | 0.038231622 | down |
| NDUFB10P1 | -1.284144035 | 0.387840811 | | down |
| CCDC155 | -1.283896967 | 0.071820316 | 0.15929201 | down |
| RP11-296I10.3 | -1.283379508 | 0.37792597 | | down |
| Cyp2c44 | -1.283333196 | 0.660907537 | | down |
| RP11-359K18.3 | -1.283305908 | 0.646002524 | | down |
| RP11-335F8.2 | -1.283273736 | 0.644760161 | | down |
| NA | -1.283080072 | 0.08934732 | 0.186629857 | down |
| KB-51A8.1 | -1.283044607 | 0.031890823 | 0.087708522 | down |
| LINC00535 | -1.282739767 | 0.007999076 | 0.030975212 | down |
| RP4-803A2.1 | -1.28273345 | 0.033511329 | 0.091007354 | down |
| RP11-50C13.2 | -1.282620502 | 0.594417347 | | down |
| RPS28P7 | -1.282436411 | 4.44E-06 | 7.20E-05 | down |
| GDPGP1 | -1.282393897 | 1.28E-07 | 3.51E-06 | down |
| ATP7B | -1.282366302 | 0.000136105 | 0.001220634 | down |
| CDH17 | -1.282307708 | 0.000250329 | 0.002012544 | down |
| KCND3-AS1 | -1.282295827 | 0.643762992 | | down |
| FBXL15 | -1.282259881 | 5.21E-06 | 8.20E-05 | down |
| NA | -1.282160972 | 0.276799142 | | down |
| KB-1615E4.3 | -1.28214576 | 0.139615596 | 0.255831359 | down |
| NA | -1.281936867 | 0.000835338 | 0.005255896 | down |
| STAR | -1.281852326 | 0.000219058 | 0.001801147 | down |
| LINC01023 | -1.281716993 | 5.76E-06 | 8.93E-05 | down |
| NA | -1.281566319 | 0.046314298 | 0.115551134 | down |
| AC016735.1 | -1.281483956 | 0.005040121 | 0.021831802 | down |
| RP1-80N2.3 | -1.281439134 | 0.000918303 | 0.005669854 | down |
| SNHG25 | -1.281380137 | 0.015451287 | 0.051119753 | down |
| RP1-97D16.1 | -1.28136116 | 0.177050459 | | down |
| KIR2DL4 | -1.281283658 | 0.01814033 | 0.057732598 | down |
| SPAG17 | -1.28126346 | 0.169832199 | 0.293533941 | down |
| RNA5SP209 | -1.28109179 | 0.505141351 | 0.631735102 | down |
| RP11-297M9.1 | -1.281053346 | 0.156427239 | | down |
| RLN3 | -1.28102103 | 0.325771901 | | down |
| PNPLA3 | -1.280718221 | 0.007181515 | 0.028521011 | down |
| LRRC37A15P | -1.280702383 | 0.515428747 | | down |
| NA | -1.28047601 | 0.01135575 | 0.040461591 | down |
| RP1-179N16.3 | -1.280424468 | 0.229938833 | 0.362316163 | down |
| RP11-61D1.3 | -1.280186709 | 0.661489447 | | down |
| C7orf72 | -1.280080356 | 0.092501732 | 0.191175181 | down |
| HMGB3P22 | -1.280055754 | 0.000182395 | 0.001550616 | down |
| AC018755.16 | -1.280014878 | 0.000386875 | 0.002843139 | down |
| RP11-235E17.4 | -1.279890345 | 0.185077559 | | down |
| HIST1H3E | -1.279767235 | 2.47E-06 | 4.41E-05 | down |
| RN7SL636P | -1.279639355 | 0.461859227 | 0.593629887 | down |
| KIR2DL1 | -1.279495329 | 0.176141888 | 0.300938691 | down |
| ST3GAL3 | -1.279377041 | 0.000492365 | 0.003433756 | down |
| BNIP3P13 | -1.279285006 | 0.596039123 | | down |
| RP11-739G5.1 | -1.279093564 | 0.469273309 | | down |
| FARSA-AS1 | -1.279081914 | 0.219715522 | | down |

| | | | | |
|---|---|---|---|---|
| ADAMTS19-AS1 | -1.279015952 | 0.330333605 | | down |
| RP11-247I13.6 | -1.278944197 | 0.599438857 | | down |
| MSANTD1 | -1.278940586 | 0.000969966 | 0.005906407 | down |
| LINC01343 | -1.278894157 | 0.662030271 | | down |
| RP11-342M1.6 | -1.278894157 | 0.662030271 | | down |
| RNA5SP51 | -1.278894157 | 0.662030271 | | down |
| RP4-681L3.2 | -1.278894157 | 0.662030271 | | down |
| NA | -1.278894157 | 0.662030271 | | down |
| RPSAP28 | -1.278894157 | 0.662030271 | | down |
| RN7SL126P | -1.278894157 | 0.662030271 | | down |
| NA | -1.278894157 | 0.662030271 | | down |
| AC079248.1 | -1.278894157 | 0.662030271 | | down |
| RPEP5 | -1.278894157 | 0.662030271 | | down |
| NA | -1.278894157 | 0.662030271 | | down |
| FECHP1 | -1.278894157 | 0.662030271 | | down |
| RP11-441M10.1 | -1.278894157 | 0.662030271 | | down |
| HPRT1P1 | -1.278894157 | 0.662030271 | | down |
| RP11-11N6.1 | -1.278894157 | 0.662030271 | | down |
| RP11-27G13.4 | -1.278894157 | 0.662030271 | | down |
| RP11-654F3.1 | -1.278894157 | 0.662030271 | | down |
| CTD-2194D22.4 | -1.278894157 | 0.662030271 | | down |
| HTR1A | -1.278894157 | 0.662030271 | | down |
| RNU4-90P | -1.278894157 | 0.662030271 | | down |
| RPL7P4 | -1.278894157 | 0.662030271 | | down |
| RNU4-72P | -1.278894157 | 0.662030271 | | down |
| RN7SL408P | -1.278894157 | 0.662030271 | | down |
| RP1-28C20.1 | -1.278894157 | 0.662030271 | | down |
| AC073325.1 | -1.278894157 | 0.662030271 | | down |
| SLC29A4P2 | -1.278894157 | 0.662030271 | | down |
| NDUFAF4P2 | -1.278894157 | 0.662030271 | | down |
| ARF1P1 | -1.278894157 | 0.662030271 | | down |
| EIF3IP1 | -1.278894157 | 0.662030271 | | down |
| RP11-533K11.1 | -1.278894157 | 0.662030271 | | down |
| RNU6-295P | -1.278894157 | 0.662030271 | | down |
| KB-1000E4.2 | -1.278894157 | 0.662030271 | | down |
| CTD-2517M22.9 | -1.278894157 | 0.662030271 | | down |
| HMGB3P23 | -1.278894157 | 0.662030271 | | down |
| RN7SL787P | -1.278894157 | 0.662030271 | | down |
| RN7SL754P | -1.278894157 | 0.662030271 | | down |
| RP11-733D4.2 | -1.278894157 | 0.662030271 | | down |
| RP11-564C4.4 | -1.278894157 | 0.662030271 | | down |
| ZNF519P1 | -1.278894157 | 0.662030271 | | down |
| CTA-109P11.4 | -1.278894157 | 0.662030271 | | down |
| OR4C5 | -1.278894157 | 0.662030271 | | down |
| OR4A42P | -1.278894157 | 0.662030271 | | down |
| OR5D18 | -1.278894157 | 0.662030271 | | down |
| OR8L1P | -1.278894157 | 0.662030271 | | down |
| OR5AK3P | -1.278894157 | 0.662030271 | | down |
| RP11-678K21.1 | -1.278894157 | 0.662030271 | | down |
| AP000770.2 | -1.278894157 | 0.662030271 | | down |
| OR10G9 | -1.278894157 | 0.662030271 | | down |

| | | | | |
|---|---|---|---|---|
| NA | -1.278894157 | 0.662030271 | | down |
| RNU6-1188P | -1.278894157 | 0.662030271 | | down |
| RP11-1042B17.3 | -1.278894157 | 0.662030271 | | down |
| RP11-986E7.2 | -1.278894157 | 0.662030271 | | down |
| AC011196.3 | -1.278894157 | 0.662030271 | | down |
| MRPS15P1 | -1.278894157 | 0.662030271 | | down |
| LINC01195 | -1.278894157 | 0.662030271 | | down |
| RP11-170L3.2 | -1.278894157 | 0.662030271 | | down |
| RP11-1109M24.16 | -1.278894157 | 0.662030271 | | down |
| NA | -1.278894157 | 0.662030271 | | down |
| CTC-304I17.5 | -1.278894157 | 0.662030271 | | down |
| MIR152 | -1.278894157 | 0.662030271 | | down |
| CSHL1 | -1.278894157 | 0.662030271 | | down |
| CH17-351M24.1 | -1.278894157 | 0.662030271 | | down |
| NA | -1.278894157 | 0.662030271 | | down |
| NA | -1.278894157 | 0.662030271 | | down |
| CTD-2265O21.3 | -1.278894157 | 0.662030271 | | down |
| ADIG | -1.278894157 | 0.662030271 | | down |
| bP-21201H5.2 | -1.278894157 | 0.662030271 | | down |
| CNN2P7 | -1.278894157 | 0.662030271 | | down |
| Metazoa_SRP | -1.278894157 | 0.662030271 | | down |
| CKS1BP6 | -1.278894157 | 0.662030271 | | down |
| CYCSP44 | -1.278894157 | 0.662030271 | | down |
| AC002523.1 | -1.278894157 | 0.662030271 | | down |
| NA | -1.278886052 | 0.514226814 | | down |
| MAD1L1 | -1.278885884 | 0.003280276 | 0.015526576 | down |
| PRR13P4 | -1.278882872 | 0.650488765 | | down |
| RP11-13K12.5 | -1.278880642 | 0.009769569 | 0.035974621 | down |
| PIWIL1 | -1.278867423 | 0.198656408 | 0.327484448 | down |
| RPSAP75 | -1.278828318 | 0.601130571 | | down |
| LINC01173 | -1.27879885 | 0.662053908 | | down |
| RP11-108B14.5 | -1.27879885 | 0.662053908 | | down |
| RP11-640A1.3 | -1.278788623 | 0.510133993 | | down |
| RP11-125D12.2 | -1.278733548 | 0.661992933 | | down |
| AP000221.1 | -1.278686379 | 0.662081802 | | down |
| SHISA9 | -1.278659568 | 0.001475199 | 0.008224373 | down |
| SAGE2P | -1.278652169 | 0.662069048 | | down |
| RP11-20I23.7 | -1.278626428 | 0.601665447 | | down |
| ATP13A4 | -1.278610773 | 0.000590818 | 0.003982617 | down |
| RP11-573D15.3 | -1.278488802 | 0.652307427 | | down |
| AC016909.1 | -1.278373278 | 0.662159458 | | down |
| RP11-114F10.3 | -1.278340754 | 0.210937495 | 0.341479281 | down |
| SCARNA14 | -1.278324091 | 0.662171658 | | down |
| NA | -1.278156514 | 0.468491765 | | down |
| RP11-79N23.1 | -1.277959214 | 0.005297875 | 0.022689856 | down |
| RP11-550H2.1 | -1.277718557 | 0.555866737 | | down |
| GKN1 | -1.277559067 | 0.639192591 | | down |
| RP5-875H18.10 | -1.277350273 | 0.019584118 | 0.061067488 | down |
| ZNF337-AS1 | -1.277215033 | 0.00011555 | 0.001068834 | down |
| ZC2HC1C | -1.277052525 | 2.33E-07 | 5.88E-06 | down |
| CHRNA4 | -1.277021763 | 0.045578106 | 0.114255205 | down |

| | | | | |
|---|---|---|---|---|
| LGI3 | -1.276972821 | 0.007106933 | 0.028314269 | down |
| CACNA1F | -1.276775397 | 1.20E-05 | 0.000163513 | down |
| RP11-101C21.4 | -1.276705623 | 0.161151548 | | down |
| TPRX2P | -1.276663846 | 0.662583493 | | down |
| CFTRP1 | -1.276656206 | 0.649110964 | | down |
| AC004593.3 | -1.276627684 | 0.604711945 | | down |
| RP1-67K17.4 | -1.276599422 | 0.019715225 | 0.061376094 | down |
| MMP28 | -1.276500473 | 1.71E-06 | 3.25E-05 | down |
| RP11-84A19.4 | -1.27642614 | 0.080423341 | 0.172897235 | down |
| RP11-96D1.5 | -1.276389828 | 0.022889634 | 0.068514677 | down |
| RABL6 | -1.276073328 | 7.71E-08 | 2.30E-06 | down |
| RP11-77H9.8 | -1.275572044 | 0.006891343 | 0.027663967 | down |
| FAM71E1 | -1.275200654 | 0.000555742 | 0.003792912 | down |
| ZNF839P1 | -1.274978848 | 0.550367333 | | down |
| U3 | -1.274852444 | 0.365941083 | | down |
| RP11-791G16.5 | -1.274678016 | 0.662961839 | | down |
| RHBDL1 | -1.274634307 | 1.28E-05 | 0.000173225 | down |
| NA | -1.274611547 | 0.274831276 | | down |
| RP11-181C3.1 | -1.274590632 | 0.00045044 | 0.003202162 | down |
| AP000487.5 | -1.274389649 | 0.004189082 | 0.018914456 | down |
| UGT1A6 | -1.274109985 | 0.089724043 | 0.187147261 | down |
| RP11-435J9.2 | -1.274030424 | 0.183409647 | | down |
| PRG3 | -1.273997671 | 0.352092967 | 0.490803188 | down |
| RP11-26L20.3 | -1.273928144 | 0.475217475 | | down |
| KRT8P33 | -1.273914317 | 3.68E-05 | 0.000415588 | down |
| LINC00933 | -1.27378265 | 0.006679031 | 0.027051226 | down |
| RP11-247L20.3 | -1.273614166 | 0.097971878 | 0.199113123 | down |
| NA | -1.273563689 | 0.412864769 | 0.549495058 | down |
| PADI3 | -1.273372627 | 0.150185501 | 0.269206681 | down |
| SPDYE4 | -1.273351954 | 0.434834255 | | down |
| CTA-503F6.2 | -1.273246118 | 0.476881576 | | down |
| RP11-539E19.2 | -1.27322922 | 0.604894938 | | down |
| NA | -1.273202586 | 0.300259004 | | down |
| RP4-710M16.2 | -1.27315468 | 0.033734353 | 0.09152645 | down |
| RP11-367H1.1 | -1.273119004 | 0.158181289 | | down |
| OSTN-AS1 | -1.273015974 | 0.004335877 | 0.019396527 | down |
| TPT1-AS1 | -1.272388676 | 1.95E-07 | 5.08E-06 | down |
| RP11-862G15.1 | -1.272321413 | 0.600884569 | | down |
| RP11-122M14.1 | -1.272305736 | 0.139448248 | 0.255633849 | down |
| GRIK1 | -1.272058002 | 0.000358218 | 0.002685206 | down |
| CTC-350I8.1 | -1.271625506 | 0.106305774 | 0.211120127 | down |
| AL121578.7 | -1.271571644 | 0.152750795 | 0.272258224 | down |
| RP11-63L7.5 | -1.271286058 | 0.211659546 | | down |
| C7orf71 | -1.271161938 | 0.441231489 | | down |
| AL109767.1 | -1.271095053 | 0.096144782 | 0.19652836 | down |
| ARL14 | -1.270943153 | 3.16E-05 | 0.000365751 | down |
| CTD-3065B20.3 | -1.270923794 | 0.321934931 | | down |
| RP11-255A11.2 | -1.270907502 | 0.66393816 | | down |
| NETO1 | -1.270867753 | 0.055281184 | 0.131512224 | down |
| LRRC75B | -1.270867187 | 1.77E-08 | 6.42E-07 | down |
| RP11-414J4.2 | -1.270347102 | 0.157846984 | 0.278716121 | down |

| | | | | |
|---|---|---|---|---|
| RP11-582E3.5 | -1.270087167 | 0.552261547 | | down |
| CKMT1A | -1.269847959 | 0.000583186 | 0.003939128 | down |
| RP11-463D19.1 | -1.269712675 | 0.601584644 | | down |
| NA | -1.269609014 | 0.564546978 | | down |
| AC008278.2 | -1.269501729 | 0.463381879 | | down |
| STSP1 | -1.269395189 | 0.518929366 | | down |
| MIR615 | -1.269390934 | 0.604832256 | | down |
| NA | -1.269374217 | 0.556055782 | | down |
| RP11-124N19.3 | -1.269311598 | 0.34871956 | | down |
| RP11-90P13.1 | -1.269166645 | 0.062219681 | 0.143602818 | down |
| RP11-367J11.3 | -1.269142494 | 0.000563593 | 0.00383437 | down |
| RP11-93B14.6 | -1.269116143 | 0.005967146 | 0.024847806 | down |
| NA | -1.268978799 | 0.641891925 | | down |
| NA | -1.268910456 | 0.071785595 | 0.15928147 | down |
| RP11-680F8.4 | -1.26888473 | 0.310092606 | | down |
| NA | -1.268726061 | 0.280593714 | 0.416983605 | down |
| NA | -1.268713998 | 0.000189736 | 0.001602147 | down |
| SLC26A4 | -1.268563133 | 0.004269885 | 0.019178041 | down |
| AC018647.3 | -1.268288892 | 0.005353918 | 0.022858194 | down |
| RP11-576C12.1 | -1.268271214 | 0.189752931 | 0.316982877 | down |
| RP11-114H24.2 | -1.268253871 | 0.134740212 | 0.249335574 | down |
| RP4-697K14.15 | -1.26822724 | 0.241722731 | | down |
| AP006621.5 | -1.267989871 | 3.15E-09 | 1.43E-07 | down |
| PTH2R | -1.267869148 | 0.101427814 | 0.204102004 | down |
| RP11-496N12.9 | -1.267575912 | 0.649963084 | | down |
| CTA-38K21.2 | -1.267544017 | 0.045639732 | 0.114361978 | down |
| TRAV39 | -1.26747545 | 0.039270881 | 0.10252471 | down |
| NA | -1.267385271 | 0.068133841 | 0.15332283 | down |
| RP11-80H5.9 | -1.267344969 | 0.040425972 | 0.104757454 | down |
| TMEM139 | -1.267205119 | 0.00119943 | 0.007009953 | down |
| NA | -1.267203915 | 0.27603306 | 0.41232379 | down |
| RP11-76C10.2 | -1.267091376 | 0.51217439 | | down |
| TRAJ38 | -1.267031966 | 0.476479205 | | down |
| RP11-400D2.2 | -1.267008551 | 0.356412598 | | down |
| RP6-74O6.6 | -1.266971931 | 0.02720633 | 0.078114199 | down |
| HTR5BP | -1.26660559 | 0.09127411 | 0.189341943 | down |
| GBP6 | -1.266324788 | 0.145103536 | 0.262740746 | down |
| RP11-116D17.3 | -1.266277999 | 0.040216598 | 0.104349898 | down |
| CTD-2319I12.2 | -1.266160716 | 0.004907324 | 0.021389182 | down |
| RP11-485M7.2 | -1.266080721 | 0.016936294 | 0.054660439 | down |
| RNF224 | -1.265939723 | 0.023318652 | 0.069530128 | down |
| RP11-475D10.4 | -1.265831168 | 0.003924616 | 0.017966045 | down |
| RP11-481C4.2 | -1.265470164 | 3.72E-05 | 0.000419831 | down |
| BNIPL | -1.265274794 | 0.00078965 | 0.005024195 | down |
| CXXC1P1 | -1.265234567 | 0.251853172 | 0.386725784 | down |
| RP11-216L13.21 | -1.265178052 | 0.095208813 | 0.195193524 | down |
| RP11-680G24.4 | -1.265153706 | 0.001434036 | 0.008054621 | down |
| PSORS1C3 | -1.265093803 | 0.015623526 | 0.051519397 | down |
| RNF216-IT1 | -1.264888145 | 0.151046618 | 0.270194745 | down |
| DIRC3 | -1.264791953 | 0.001575366 | 0.008676498 | down |
| B4GALT1-AS1 | -1.264629251 | 2.36E-06 | 4.24E-05 | down |

| | | | | |
|---|---|---|---|---|
| Y_RNA | -1.264517705 | 0.665598993 | | down |
| NA | -1.264517705 | 0.665598993 | | down |
| L22NC03-N95F10. | -1.264333202 | 0.030516104 | 0.084875563 | down |
| BBOX1-AS1 | -1.264252176 | 0.071113138 | 0.15810872 | down |
| RP11-370I10.10 | -1.264205585 | 0.115484842 | 0.22391911 | down |
| MIR635 | -1.26393812 | 0.001029511 | 0.00620474 | down |
| RP11-1000B6.3 | -1.263752664 | 0.022285661 | 0.067217188 | down |
| AC073621.2 | -1.263128694 | 0.517741604 | | down |
| RP11-11M20.4 | -1.263120481 | 1.38E-05 | 0.000184403 | down |
| RP11-69G7.1 | -1.262990234 | 0.143104197 | 0.2602556 | down |
| HCG20 | -1.262909252 | 0.045288054 | 0.113727374 | down |
| RNU6-554P | -1.262898676 | 0.217140387 | 0.348398707 | down |
| RP11-7F17.3 | -1.262667442 | 0.00041015 | 0.002965718 | down |
| SUMO2P19 | -1.262663954 | 0.140103102 | 0.256443134 | down |
| PA2G4P1 | -1.26263791 | 0.244699172 | 0.378707478 | down |
| RP11-103J8.2 | -1.262371435 | 0.258666162 | | down |
| PRCAT47 | -1.262222722 | 0.221061572 | 0.352712367 | down |
| RP11-104N10.1 | -1.262116377 | 0.0006476 | 0.004283687 | down |
| NAP1L1P3 | -1.261984071 | 0.181709957 | | down |
| NA | -1.261937842 | 0.519390959 | | down |
| RRP7A | -1.261825876 | 4.58E-05 | 0.000496967 | down |
| NA | -1.261509342 | 0.167278301 | 0.290436273 | down |
| Metazoa_SRP | -1.261460471 | 0.076581273 | 0.166870305 | down |
| RP11-613M5.2 | -1.26114927 | 0.004120321 | 0.018679785 | down |
| RP11-681N23.1 | -1.2611284 | 0.096847918 | 0.197565013 | down |
| MT-CO2 | -1.260973761 | 0.001629584 | 0.008914641 | down |
| FAM185BP | -1.260733485 | 0.000768968 | 0.004916551 | down |
| RP5-836N17.4 | -1.260709787 | 0.029131267 | 0.082164833 | down |
| RP11-875O11.2 | -1.260608686 | 0.247343425 | 0.381699218 | down |
| REG3A | -1.260431955 | 0.082725463 | 0.17659541 | down |
| CTD-2073O6.1 | -1.25995081 | 0.142237158 | 0.259117947 | down |
| RNU5D-1 | -1.259872617 | 0.042585022 | 0.108877918 | down |
| SLC4A4 | -1.259830137 | 0.00028482 | 0.002235729 | down |
| CTD-2206N4.4 | -1.259340996 | 0.063119981 | 0.145071658 | down |
| H2AFJ | -1.259226697 | 6.85E-06 | 0.000103387 | down |
| RNU6-370P | -1.259086871 | 0.482371409 | 0.611543869 | down |
| RP11-507K2.2 | -1.258913464 | 0.278712548 | | down |
| RP11-461A8.4 | -1.258901252 | 0.002201944 | 0.011340846 | down |
| RP11-849H4.4 | -1.258826983 | 0.00075482 | 0.004848792 | down |
| LINC00682 | -1.258315356 | 0.001324689 | 0.007563466 | down |
| RP11-490D19.8 | -1.258273481 | 0.563321808 | | down |
| WASH4P | -1.258110339 | 6.57E-05 | 0.000666657 | down |
| CTB-164L20.1 | -1.258014419 | 0.667065279 | | down |
| AC108868.6 | -1.257764224 | 0.604811031 | | down |
| PLAC1 | -1.257739665 | 0.366958783 | | down |
| GOLGA6L22 | -1.257621974 | 0.65026811 | | down |
| MGAT4B | -1.257470421 | 2.26E-08 | 7.86E-07 | down |
| AK3P2 | -1.257421321 | 0.296207633 | | down |
| COL26A1 | -1.257312108 | 0.019283646 | 0.060439348 | down |
| SLC10A5 | -1.257306887 | 0.00234117 | 0.01192544 | down |
| SNORA46 | -1.257237461 | 0.653258627 | | down |

| | | | | |
|---|---|---|---|---|
| RPL5P22 | -1.256944717 | 0.473214568 | | down |
| NA | -1.256447983 | 0.560859997 | | down |
| LINC01479 | -1.255956787 | 0.140995724 | | down |
| AGBL4 | -1.255877847 | 0.023781197 | 0.070587066 | down |
| CTD-2525I3.5 | -1.255854111 | 0.058160453 | 0.136648305 | down |
| AC107399.2 | -1.255809933 | 0.5160905 | 0.641143855 | down |
| DHRS4L2 | -1.255675442 | 6.92E-06 | 0.000104123 | down |
| AP3S2 | -1.255606924 | 1.08E-10 | 7.31E-09 | down |
| EEF1B2P5 | -1.255408809 | 0.667824795 | | down |
| NA | -1.255180225 | 0.557645107 | | down |
| RP3-428L16.1 | -1.255012142 | 0.068244853 | 0.153446217 | down |
| SLC25A25-AS1 | -1.254478605 | 1.65E-13 | 2.28E-11 | down |
| RP11-580I16.2 | -1.254454782 | 0.00592317 | 0.024709209 | down |
| LINC01490 | -1.254330187 | 0.450907304 | | down |
| RP11-359E3.4 | -1.254200944 | 0.000142469 | 0.001267872 | down |
| RP11-108O10.2 | -1.253947844 | 0.15422626 | 0.273962516 | down |
| SALL3 | -1.253843845 | 0.286021279 | 0.423123901 | down |
| SSXP10 | -1.253554797 | 0.23204646 | | down |
| RP11-779O18.2 | -1.253445713 | 0.318260271 | | down |
| HMGN2P17 | -1.253419014 | 0.022269276 | 0.067188004 | down |
| INTS1 | -1.253397807 | 1.30E-06 | 2.55E-05 | down |
| NA | -1.253374895 | 0.283785171 | | down |
| ARSD | -1.253265402 | 2.47E-07 | 6.18E-06 | down |
| CTC-806A22.1 | -1.25316425 | 0.341174355 | | down |
| HCRTR2 | -1.252821872 | 0.4051826 | | down |
| CCDC30 | -1.252758763 | 0.000115633 | 0.001069277 | down |
| AP006621.8 | -1.252733203 | 0.099618946 | 0.201417376 | down |
| RP11-158H5.7 | -1.25229358 | 1.76E-05 | 0.000225555 | down |
| GYG2P1 | -1.25219857 | 0.079473226 | 0.171308194 | down |
| RP11-562A8.1 | -1.2521175 | 0.162888634 | | down |
| NA | -1.251776041 | 0.030541977 | 0.084912286 | down |
| CTD-2583A14.1 | -1.251632949 | 0.416383282 | | down |
| SNORA63 | -1.251447571 | 0.183839947 | | down |
| IL37 | -1.251322688 | 0.065435738 | 0.148710387 | down |
| RNU1-67P | -1.251272567 | 0.035722708 | 0.095434203 | down |
| TCEB1P31 | -1.251083616 | 0.561720639 | | down |
| TPSG1 | -1.250921719 | 0.000352959 | 0.002655059 | down |
| ZNF859P | -1.250497956 | 0.532021793 | | down |
| RP11-310H4.1 | -1.250491886 | 0.486905494 | | down |
| AKAP6 | -1.250391369 | 0.002325333 | 0.011869235 | down |
| RP11-788M5.4 | -1.250341455 | 0.135654779 | 0.25059101 | down |
| NPAS4 | -1.250270867 | 0.020009882 | 0.062111467 | down |
| AC138430.4 | -1.250259681 | 0.522034213 | | down |
| GALR2 | -1.250087032 | 0.007517484 | 0.029542875 | down |
| RP11-246A10.1 | -1.250081876 | 0.378052346 | | down |
| CTC-535M15.1 | -1.249961109 | 0.443414195 | 0.576931968 | down |
| RP11-91A18.1 | -1.249743747 | 0.600171252 | | down |
| MTRNR2L9 | -1.249699438 | 0.352827406 | | down |
| RP11-439H8.4 | -1.249293526 | 0.274747803 | 0.411047459 | down |
| C1orf61 | -1.249050956 | 0.001176293 | 0.006902448 | down |
| POU5F1 | -1.248994767 | 3.56E-06 | 5.97E-05 | down |

| | | | | |
|---|---|---|---|---|
| RP3-453C12.14 | -1.248814528 | 0.016791504 | 0.054317962 | down |
| RP11-714G18.1 | -1.248721014 | 0.084543986 | 0.17925473 | down |
| KBTBD11 | -1.248621348 | 3.89E-08 | 1.27E-06 | down |
| HERC2P4 | -1.248587766 | 0.028536297 | 0.080980873 | down |
| RP11-515E23.1 | -1.248325116 | 0.607187934 | | down |
| HCFC2P1 | -1.24826359 | 0.649105929 | | down |
| ENPP7P4 | -1.24821478 | 0.027301554 | 0.078312762 | down |
| B4GALNT3 | -1.247909965 | 0.001653891 | 0.009021331 | down |
| GKN2 | -1.24728356 | 0.205282592 | | down |
| UGT2B15 | -1.24697084 | 0.01692302 | 0.054623464 | down |
| CTD-2192J16.21 | -1.246923083 | 0.061339812 | 0.142143719 | down |
| NDUFB4P6 | -1.246895046 | 0.522752982 | | down |
| Metazoa_SRP | -1.246555376 | 0.562590918 | | down |
| CTD-2083E4.6 | -1.246442247 | 0.129034933 | 0.241996286 | down |
| SFTPB | -1.246424309 | 0.099858581 | 0.201690838 | down |
| RP11-505K9.3 | -1.24602025 | 0.173372396 | 0.297832939 | down |
| RP11-10017.3 | -1.245920417 | 0.066636816 | 0.15073306 | down |
| RP11-392017.2 | -1.24582433 | 0.014028464 | 0.047468316 | down |
| RNU1-85P | -1.245803559 | 0.284037803 | | down |
| RP11-252I13.2 | -1.245798793 | 0.604779023 | | down |
| TSGA10IP | -1.245783312 | 0.054780104 | 0.13076541 | down |
| RP4-630C24.3 | -1.245727033 | 0.444776071 | | down |
| NA | -1.245558587 | 0.062448532 | 0.14395793 | down |
| RP11-498J9.2 | -1.245300654 | 0.028272998 | 0.080377929 | down |
| RP5-1125N11.2 | -1.245120849 | 0.088765057 | 0.18568844 | down |
| ARHGEF26 | -1.245083012 | 3.03E-05 | 0.000353241 | down |
| LCA5L | -1.245007116 | 0.000282384 | 0.002222088 | down |
| MFSD6L | -1.244965134 | 0.009311281 | 0.034721128 | down |
| CBLN2 | -1.244479238 | 0.005013039 | 0.021743305 | down |
| FUT2 | -1.244431782 | 0.000101443 | 0.000956963 | down |
| RP11-167N5.5 | -1.244397136 | 0.002346725 | 0.011947977 | down |
| ADD3-AS1 | -1.244302248 | 0.000142871 | 0.001270696 | down |
| RP11-513G11.2 | -1.244279015 | 0.331213246 | | down |
| PIGR | -1.244274467 | 0.001858776 | 0.009905118 | down |
| MEIG1 | -1.243959365 | 0.023294858 | 0.069486767 | down |
| PAPPA2 | -1.243874983 | 0.023569414 | 0.070097888 | down |
| LINC01252 | -1.243584333 | 0.000912766 | 0.005637983 | down |
| SEC16B | -1.243501139 | 0.018113615 | 0.057676359 | down |
| RP11-567G11.1 | -1.2433216 | 0.107228682 | 0.212477148 | down |
| FTH1P10 | -1.243290527 | 0.004982608 | 0.021633392 | down |
| MMP23A | -1.243015272 | 0.005741414 | 0.024115065 | down |
| LLNLR-304A6.1 | -1.242839138 | 0.14345826 | 0.260736209 | down |
| RP11-118F19.1 | -1.242720537 | 0.088757144 | 0.18568844 | down |
| TTC29 | -1.242687178 | 0.489158403 | | down |
| CYP51A1-AS1 | -1.242588985 | 0.092326733 | 0.190919153 | down |
| RP11-108K14.8 | -1.242552573 | 0.008496453 | 0.032365592 | down |
| GCHFR | -1.242459631 | 0.004344063 | 0.019424474 | down |
| RP11-182J23.1 | -1.242151243 | 0.099331447 | 0.201062669 | down |
| GSTM4 | -1.242015787 | 1.19E-10 | 7.97E-09 | down |
| RP11-401N16.2 | -1.241294814 | 0.48113078 | 0.610357351 | down |
| ERVV-1 | -1.241038701 | 0.249320563 | 0.383896423 | down |

| | | | | |
|---|---|---|---|---|
| FNDC10 | -1.240943725 | 2.08E-05 | 0.000259465 | down |
| HKDC1 | -1.240910337 | 0.000268749 | 0.002135491 | down |
| CYP2AC1P | -1.240905904 | 0.570046451 | | down |
| PANK1 | -1.240777239 | 1.50E-09 | 7.31E-08 | down |
| FPGT-TNNI3K | -1.240723136 | 0.052276655 | 0.126213252 | down |
| NA | -1.24069983 | 0.213364328 | 0.344233587 | down |
| ARHGAP8 | -1.240310944 | 0.007218884 | 0.028624014 | down |
| RP11-44D5.2 | -1.240289027 | 0.320730119 | 0.459492427 | down |
| RP11-491H9.3 | -1.240204699 | 0.671590843 | | down |
| MIR8086 | -1.240204699 | 0.671590843 | | down |
| RPL22P18 | -1.240204699 | 0.671590843 | | down |
| RNU6-826P | -1.240204699 | 0.671590843 | | down |
| GPA33 | -1.240104463 | 0.000599636 | 0.004031703 | down |
| ECT2L | -1.239937444 | 0.007131337 | 0.028377295 | down |
| SMIM5 | -1.239712823 | 9.40E-05 | 0.000897821 | down |
| TTC23L | -1.239596401 | 0.022925179 | 0.068601998 | down |
| DSC1 | -1.239453071 | 0.277781278 | 0.413971266 | down |
| NA | -1.239233714 | 0.216681441 | | down |
| GAPDHP61 | -1.239030184 | 0.078047178 | 0.169144736 | down |
| RP1-206D15.6 | -1.238888932 | 9.21E-05 | 0.000882191 | down |
| ADPRHL1 | -1.238833829 | 2.60E-08 | 8.83E-07 | down |
| SUB1P2 | -1.238283307 | 0.464313132 | | down |
| RP11-325P15.1 | -1.238212328 | 0.427054648 | | down |
| RNU6-549P | -1.238197874 | 0.171539997 | 0.295614757 | down |
| CASQ1 | -1.238195378 | 0.005487859 | 0.023268026 | down |
| CAMK2N1 | -1.238142276 | 1.54E-07 | 4.13E-06 | down |
| GPR152 | -1.237935133 | 0.145929097 | 0.263848013 | down |
| ZNF317P1 | -1.237838674 | 0.671942396 | | down |
| LINC00928 | -1.237649717 | 0.452784201 | | down |
| HRH3 | -1.237520179 | 0.041172009 | 0.106141398 | down |
| NA | -1.237091513 | 0.323921333 | | down |
| AC005593.2 | -1.237082968 | 0.146451884 | | down |
| CTD-2193G5.1 | -1.236970289 | 0.528299371 | | down |
| RP11-547C5.1 | -1.236868611 | 0.672424275 | | down |
| AC005616.2 | -1.236734378 | 0.654327819 | | down |
| CT45A11P | -1.236697985 | 0.082389816 | 0.17606655 | down |
| SEPT7P8 | -1.236383456 | 0.612207149 | | down |
| DFNA5 | -1.236252726 | 0.001258034 | 0.007272916 | down |
| RP11-445L13__B.[illegible] | -1.23625207 | 0.672578345 | | down |
| PPIAP6 | -1.236224709 | 0.087635964 | | down |
| RP11-345N11.1 | -1.235769026 | 0.194767694 | | down |
| CARMIL3 | -1.235385719 | 0.000105997 | 0.000993003 | down |
| PTRH1 | -1.235302938 | 0.179983411 | 0.305228133 | down |
| RPS10P16 | -1.235286507 | 0.2034793 | 0.332919 | down |
| RP5-901A4.1 | -1.235285614 | 0.017759338 | 0.056834422 | down |
| CNTFR-AS1 | -1.235270433 | 0.222973101 | 0.35498934 | down |
| FEM1AP2 | -1.235141116 | 0.425958775 | | down |
| OCA2 | -1.235077033 | 0.016070337 | 0.052588739 | down |
| NF1P9 | -1.235021694 | 0.411159343 | 0.547872058 | down |
| AC007679.4 | -1.234774878 | 0.524810339 | | down |
| RPS27P16 | -1.234721244 | 0.224164442 | 0.35639501 | down |

| | | | | |
|---|---|---|---|---|
| ORAOV1P1 | -1.234611722 | 0.228414479 | | down |
| KCNK6 | -1.234360333 | 0.00040632 | 0.002944685 | down |
| NRTN | -1.234186962 | 0.001192641 | 0.006982913 | down |
| NA | -1.234131601 | 0.003230009 | 0.015340245 | down |
| P2RX4 | -1.233705725 | 1.03E-07 | 2.89E-06 | down |
| RP11-158I9.5 | -1.233692873 | 0.019292979 | 0.060460859 | down |
| AC005616.1 | -1.233638166 | 0.606106782 | | down |
| Metazoa_SRP | -1.233624909 | 0.655610867 | | down |
| ACER1 | -1.233473872 | 0.021799837 | 0.066163683 | down |
| NA | -1.233173332 | 0.404519587 | | down |
| IGLJ7 | -1.233148062 | 0.488689782 | | down |
| RP11-296K13.4 | -1.233023199 | 0.309495487 | | down |
| RP11-274G22.1 | -1.232572333 | 0.335977141 | 0.474212968 | down |
| TLX2 | -1.232376235 | 0.001316785 | 0.00753122 | down |
| SLITRK6 | -1.232145437 | 0.00149505 | 0.008316361 | down |
| CTD-2306A12.1 | -1.232070059 | 0.003848561 | 0.017685249 | down |
| VWC2L | -1.231951252 | 0.330979698 | | down |
| CDS1 | -1.231905841 | 7.23E-05 | 0.000720399 | down |
| RPS17P14 | -1.231670345 | 0.564150375 | | down |
| HIST2H2AB | -1.231475123 | 0.025158715 | 0.07365703 | down |
| NCKAP5 | -1.231464895 | 0.000735349 | 0.004751823 | down |
| RP11-567M16.2 | -1.231400027 | 0.377877092 | | down |
| FRMD6-AS2 | -1.231322097 | 0.080183228 | 0.172504468 | down |
| RP11-460N20.4 | -1.231272313 | 0.165871873 | | down |
| RP11-3J10.7 | -1.231008471 | 0.452966855 | | down |
| C10orf99 | -1.23085829 | 0.003871008 | 0.017763948 | down |
| Y_RNA | -1.230460458 | 0.565244258 | | down |
| GAPDHP25 | -1.230326505 | 0.607050392 | | down |
| CRAT | -1.230241107 | 4.66E-11 | 3.49E-09 | down |
| NA | -1.230238752 | 0.314512272 | | down |
| MIR519B | -1.230238302 | 0.647589842 | | down |
| ECHS1 | -1.230204099 | 3.31E-09 | 1.49E-07 | down |
| SFTA3 | -1.230154241 | 0.521935945 | 0.646237771 | down |
| NA | -1.230116067 | 0.673765543 | | down |
| PGM5-AS1 | -1.230081127 | 0.101862288 | 0.204746811 | down |
| FAM83G | -1.229969692 | 7.37E-09 | 3.02E-07 | down |
| AC005237.4 | -1.22995805 | 0.264438821 | 0.399919457 | down |
| RP4-800G7.3 | -1.229889822 | 0.036318309 | 0.096715766 | down |
| TMEM210 | -1.229790731 | 0.107922812 | 0.213570871 | down |
| AP000892.4 | -1.229746552 | 0.674204537 | | down |
| RNU6-23P | -1.229737123 | 0.607153207 | | down |
| RP11-1124B17.1 | -1.229518181 | 0.486612656 | | down |
| RP11-91K11.2 | -1.229370675 | 0.203926008 | | down |
| FZD5 | -1.229333541 | 1.54E-06 | 2.96E-05 | down |
| RP11-45506.5 | -1.229286809 | 0.018627481 | 0.058860332 | down |
| NSUN7 | -1.229207705 | 0.000292248 | 0.002282377 | down |
| AC130469.2 | -1.229158905 | 0.030749619 | 0.085382768 | down |
| RNU6-61P | -1.229092585 | 0.653703078 | | down |
| CTD-2026K11.5 | -1.229077494 | 0.003137253 | 0.014989764 | down |
| AP001331.1 | -1.229055362 | 0.613403602 | | down |
| CTD-3028N15.3 | -1.228952372 | 0.231036402 | | down |

| | | | | |
|---|---|---|---|---|
| RP11-39M21.1 | -1.228600902 | 0.362237095 | 0.500600335 | down |
| NEURL1 | -1.228551232 | 8.22E-07 | 1.72E-05 | down |
| RP11-1252I4.2 | -1.228453472 | 0.613679009 | | down |
| B3GNT4 | -1.227907991 | 0.019491849 | 0.060852143 | down |
| OPLAH | -1.227736836 | 3.49E-07 | 8.21E-06 | down |
| RP11-476C8.2 | -1.227699705 | 0.452417735 | | down |
| HPCA | -1.227597478 | 0.000316805 | 0.002435965 | down |
| MALL | -1.227464721 | 0.003256912 | 0.01544848 | down |
| HAO2 | -1.227392871 | 0.040770456 | 0.105351496 | down |
| LINC00969 | -1.226862723 | 4.11E-07 | 9.45E-06 | down |
| SLC2A5 | -1.226859187 | 2.82E-05 | 0.000332642 | down |
| RUNDC3A-AS1 | -1.226849729 | 0.012625762 | 0.043832726 | down |
| RP11-209M4.1 | -1.226610209 | 0.193857809 | 0.321963091 | down |
| NA | -1.226581029 | 0.032803607 | 0.08957931 | down |
| CTD-2589H19.6 | -1.22651701 | 0.000376614 | 0.002783515 | down |
| RP11-126D17.1 | -1.226325795 | 0.674986436 | | down |
| RP1-63M2.7 | -1.22603221 | 0.002861917 | 0.013975563 | down |
| RP4-569D19.5 | -1.225999144 | 0.205794156 | 0.335697667 | down |
| BCYRN1 | -1.225971883 | 0.000743735 | 0.004788891 | down |
| PLK5 | -1.225646544 | 0.046229234 | 0.115377231 | down |
| RN7SL167P | -1.225640266 | 0.675197684 | | down |
| NA | -1.225594789 | 0.299694673 | 0.437258487 | down |
| RP11-318G21.4 | -1.225501917 | 0.657427957 | | down |
| SMARCE1P1 | -1.225328082 | 0.486672275 | | down |
| PGAP3 | -1.225256964 | 1.68E-09 | 8.09E-08 | down |
| LINC00871 | -1.225156375 | 0.179547278 | 0.304712106 | down |
| AGBL2 | -1.22509843 | 0.00080231 | 0.005091802 | down |
| RP11-250H24.2 | -1.224901618 | 0.222418342 | | down |
| RP11-6N17.2 | -1.224880642 | 0.136013582 | 0.251083998 | down |
| RP11-563N12.2 | -1.224583492 | 0.398098663 | 0.535083672 | down |
| NA | -1.224471702 | 0.232055675 | 0.364752868 | down |
| SRI | -1.224326172 | 3.82E-07 | 8.88E-06 | down |
| F13B | -1.224243884 | 0.499192238 | | down |
| IMPDH1P6 | -1.223850155 | 0.573801052 | | down |
| CRHR2 | -1.22375947 | 0.010347629 | 0.037655545 | down |
| IRX3 | -1.223640629 | 0.028711728 | 0.08128619 | down |
| SLC35G6 | -1.223510079 | 0.224150032 | 0.356391379 | down |
| RPL3P4 | -1.223448893 | 1.58E-06 | 3.03E-05 | down |
| RP11-894P9.1 | -1.223124683 | 0.004045682 | 0.018413644 | down |
| KRTAP10-11 | -1.223098707 | 0.518422056 | 0.643162747 | down |
| GAPDHS | -1.223060716 | 0.359642301 | | down |
| MACC1 | -1.223001943 | 2.44E-06 | 4.35E-05 | down |
| RN7SKP23 | -1.222604714 | 0.04305125 | 0.109780322 | down |
| PPP6R2P1 | -1.222540839 | 0.65252639 | | down |
| RPRML | -1.222507631 | 0.061531176 | 0.142449873 | down |
| CXCL14 | -1.222453401 | 5.97E-06 | 9.21E-05 | down |
| COX5BP6 | -1.222413197 | 0.025401319 | 0.074150527 | down |
| IGFBPL1 | -1.222140938 | 0.002137548 | 0.01107059 | down |
| RP11-84A14.6 | -1.221727925 | 0.492525593 | | down |
| SLC36A1 | -1.221666585 | 6.96E-09 | 2.86E-07 | down |
| PIGQ | -1.221631441 | 3.32E-05 | 0.000380024 | down |

| | | | | |
|---|---|---|---|---|
| AC009237.8 | -1.221599908 | 0.000764738 | 0.004899932 | down |
| NA | -1.221421194 | 0.190419117 | | down |
| GALNT12 | -1.221241026 | 1.70E-05 | 0.000219839 | down |
| RP11-382A20.4 | -1.221178594 | 0.492301062 | | down |
| CTD-2647L4.4 | -1.22100098 | 0.011195743 | 0.039981564 | down |
| KLK1 | -1.220757046 | 0.0001251 | 0.001139977 | down |
| RP5-979D14.1 | -1.220590117 | 0.533518609 | | down |
| LINC00350 | -1.220439227 | 0.259242829 | 0.394463993 | down |
| C17orf102 | -1.219964848 | 0.616703999 | | down |
| RN7SL538P | -1.219858173 | 0.676702038 | | down |
| EGFLAM-AS2 | -1.219858173 | 0.676702038 | | down |
| KB-1410C5.1 | -1.219858173 | 0.676702038 | | down |
| RP11-445P17.3 | -1.219858173 | 0.676702038 | | down |
| DNM1P31 | -1.219858173 | 0.676702038 | | down |
| LINGO1-AS2 | -1.219858173 | 0.676702038 | | down |
| RNU6ATAC7P | -1.219858173 | 0.676702038 | | down |
| KRT34 | -1.219858173 | 0.676702038 | | down |
| KRT43P | -1.219858173 | 0.676702038 | | down |
| CTD-2619J13.1 | -1.219858173 | 0.676702038 | | down |
| RP11-356I18.1 | -1.219784353 | 0.664576667 | | down |
| RP1-140C12.2 | -1.219652796 | 0.06487453 | 0.147767602 | down |
| BHLHB9 | -1.219469221 | 0.618157662 | | down |
| CTC-338M12.6 | -1.219391179 | 0.027711045 | 0.079184958 | down |
| MGAT4C | -1.219262084 | 0.013755655 | 0.046711994 | down |
| RP11-737O24.2 | -1.219241768 | 0.171294248 | 0.295242063 | down |
| RP11-114F3.4 | -1.219146739 | 0.170728099 | | down |
| RP11-382M14.1 | -1.219141348 | 0.1426244 | | down |
| RP11-442O18.2 | -1.219077741 | 0.569335503 | | down |
| DYNLL1P4 | -1.219012633 | 0.041915861 | 0.1076526 | down |
| SLC22A23 | -1.218741698 | 5.05E-11 | 3.73E-09 | down |
| MIR5684 | -1.218686672 | 0.527407761 | | down |
| PRELID2 | -1.218533025 | 2.08E-07 | 5.32E-06 | down |
| SYT5 | -1.21842311 | 0.001867207 | 0.009942994 | down |
| RP11-621L6.2 | -1.2182749 | 0.4108227 | | down |
| RP5-1014D13.2 | -1.21817972 | 0.000409012 | 0.002959626 | down |
| CFAP70 | -1.217931548 | 0.006371235 | 0.026114353 | down |
| RNU6-857P | -1.217761662 | 0.572137341 | | down |
| MIR99AHG | -1.217749515 | 0.001493661 | 0.008310286 | down |
| CTA-520D8.2 | -1.217585169 | 0.573768068 | | down |
| RP11-361H10.3 | -1.217486432 | 0.082903434 | 0.176849671 | down |
| SSTR2 | -1.217397287 | 6.70E-05 | 0.000678427 | down |
| MST1P2 | -1.217369778 | 0.000107997 | 0.001008915 | down |
| AC104820.2 | -1.217223979 | 0.000725386 | 0.004696873 | down |
| RP11-735G4.1 | -1.217214251 | 0.125190858 | 0.236853536 | down |
| GPR143 | -1.217160374 | 0.006586167 | 0.026736224 | down |
| ZNF442 | -1.217083897 | 5.29E-05 | 0.000560308 | down |
| TUB-AS1 | -1.216877167 | 0.614401509 | | down |
| RPS29P20 | -1.216736111 | 0.53210675 | | down |
| CTD-261308.1 | -1.216693628 | 0.49706245 | 0.624548592 | down |
| RP11-568A7.2 | -1.216535272 | 0.244947891 | 0.378852625 | down |
| RP11-55J15.2 | -1.216524619 | 0.415687122 | | down |

| | | | | |
|---|---|---|---|---|
| SPTY2D1-AS1 | -1.216481219 | 0.000881586 | 0.005473288 | down |
| NA | -1.216306063 | 0.023379365 | 0.069676583 | down |
| RP11-35P15.1 | -1.216209449 | 0.012326701 | 0.043038165 | down |
| CTD-2017D15.1 | -1.215987524 | 0.205164404 | 0.33499779 | down |
| CAPN9 | -1.215941811 | 0.000352829 | 0.002654891 | down |
| RP11-370I10.11 | -1.215855908 | 0.083291367 | 0.177412678 | down |
| WNK2 | -1.215426824 | 4.51E-07 | 1.02E-05 | down |
| CC2D1A | -1.214997036 | 4.31E-09 | 1.87E-07 | down |
| DLEC1 | -1.21479797 | 2.92E-05 | 0.000342338 | down |
| CASC16 | -1.214732154 | 0.017026958 | 0.054905864 | down |
| AP000347.2 | -1.214555764 | 0.000720696 | 0.00467154 | down |
| AP001462.6 | -1.214527244 | 0.002779017 | 0.013653767 | down |
| LRRN3 | -1.21447979 | 0.001470901 | 0.008210583 | down |
| RP11-204K16.1 | -1.214420377 | 0.616083629 | | down |
| RP11-930O11.2 | -1.214418179 | 0.469354763 | | down |
| C9orf78 | -1.214316284 | 9.71E-08 | 2.76E-06 | down |
| HCAR1 | -1.21404749 | 0.080941895 | 0.173713715 | down |
| RP4-633I8.3 | -1.213967632 | 0.359465745 | 0.497713075 | down |
| MINOS1P3 | -1.213940881 | 0.370372069 | | down |
| AC002451.3 | -1.213922879 | 0.001310466 | 0.007503648 | down |
| RP11-417N10.3 | -1.213717754 | 0.330446874 | | down |
| RP11-307C12.13 | -1.213684134 | 0.06861239 | 0.154055038 | down |
| LINC01068 | -1.213459515 | 0.25462764 | | down |
| GTF2IRD2 | -1.213333867 | 8.20E-08 | 2.42E-06 | down |
| RP11-631M6.2 | -1.21331835 | 0.004662286 | 0.020559773 | down |
| NA | -1.213204555 | 0.190854343 | 0.318450953 | down |
| LINC01028 | -1.213113571 | 0.438532887 | | down |
| RP11-439K3.3 | -1.21285921 | 0.112003963 | 0.219121381 | down |
| AC007403.2 | -1.212617842 | 0.354539505 | 0.49316102 | down |
| GRM6 | -1.212424216 | 0.01388837 | 0.047095056 | down |
| HIST1H4A | -1.212395493 | 0.060387692 | 0.140424754 | down |
| MORC2-AS1 | -1.21202041 | 0.055186368 | 0.131369878 | down |
| RP11-69E9.1 | -1.212003 | 0.678696215 | | down |
| RP4-591B8.3 | -1.212003 | 0.678696215 | | down |
| PFN1P12 | -1.212003 | 0.678696215 | | down |
| RP5-940F7.2 | -1.212003 | 0.678696215 | | down |
| RFKP1 | -1.212003 | 0.678696215 | | down |
| RPL10AP5 | -1.212003 | 0.678696215 | | down |
| RP11-107G24.3 | -1.212003 | 0.678696215 | | down |
| AC013403.13 | -1.212003 | 0.678696215 | | down |
| RN7SKP119 | -1.212003 | 0.678696215 | | down |
| AC092669.3 | -1.212003 | 0.678696215 | | down |
| AC092570.1 | -1.212003 | 0.678696215 | | down |
| AC023347.1 | -1.212003 | 0.678696215 | | down |
| MTCO1P18 | -1.212003 | 0.678696215 | | down |
| AC016710.1 | -1.212003 | 0.678696215 | | down |
| AC009969.1 | -1.212003 | 0.678696215 | | down |
| AC012501.3 | -1.212003 | 0.678696215 | | down |
| MIR1246 | -1.212003 | 0.678696215 | | down |
| LIN28AP1 | -1.212003 | 0.678696215 | | down |
| AC008072.1 | -1.212003 | 0.678696215 | | down |

| | | | |
|---|---|---|---|
| RNU2-64P | -1.212003 | 0.678696215 | down |
| MTND1P16 | -1.212003 | 0.678696215 | down |
| RP11-517B11.6 | -1.212003 | 0.678696215 | down |
| RP11-489M13.2 | -1.212003 | 0.678696215 | down |
| RP11-315A17.1 | -1.212003 | 0.678696215 | down |
| RP13-644M16.3 | -1.212003 | 0.678696215 | down |
| RP11-364P22.1 | -1.212003 | 0.678696215 | down |
| RP11-248N22.2 | -1.212003 | 0.678696215 | down |
| RP11-756P10.1 | -1.212003 | 0.678696215 | down |
| NA | -1.212003 | 0.678696215 | down |
| CTC-480C2.1 | -1.212003 | 0.678696215 | down |
| RP1-21P18.1 | -1.212003 | 0.678696215 | down |
| [illegible]bac-BPG258E24.[illegible] | -1.212003 | 0.678696215 | down |
| MIR3135B | -1.212003 | 0.678696215 | down |
| RP11-524K22.1 | -1.212003 | 0.678696215 | down |
| RP3-431P23.2 | -1.212003 | 0.678696215 | down |
| VN1R25P | -1.212003 | 0.678696215 | down |
| RP13-580B18.1 | -1.212003 | 0.678696215 | down |
| SAPCD2P2 | -1.212003 | 0.678696215 | down |
| RP5-1186P10.1 | -1.212003 | 0.678696215 | down |
| TAS2R39 | -1.212003 | 0.678696215 | down |
| RP11-175E9.1 | -1.212003 | 0.678696215 | down |
| RP13-225O21.5 | -1.212003 | 0.678696215 | down |
| Y_RNA | -1.212003 | 0.678696215 | down |
| NA | -1.212003 | 0.678696215 | down |
| CTSLP4 | -1.212003 | 0.678696215 | down |
| Y_RNA | -1.212003 | 0.678696215 | down |
| OR51F5P | -1.212003 | 0.678696215 | down |
| RP11-646J21.7 | -1.212003 | 0.678696215 | down |
| VN2R9P | -1.212003 | 0.678696215 | down |
| AP006288.1 | -1.212003 | 0.678696215 | down |
| RP11-170L9.1 | -1.212003 | 0.678696215 | down |
| RP11-643G5.7 | -1.212003 | 0.678696215 | down |
| RP11-27G22.1 | -1.212003 | 0.678696215 | down |
| RP11-687M24.4 | -1.212003 | 0.678696215 | down |
| NA | -1.212003 | 0.678696215 | down |
| RP11-776A13.2 | -1.212003 | 0.678696215 | down |
| RPL30P13 | -1.212003 | 0.678696215 | down |
| RP11-70F11.7 | -1.212003 | 0.678696215 | down |
| SNORA70 | -1.212003 | 0.678696215 | down |
| CENPIP1 | -1.212003 | 0.678696215 | down |
| KRT18P27 | -1.212003 | 0.678696215 | down |
| LINC01049 | -1.212003 | 0.678696215 | down |
| CTD-2014B16.2 | -1.212003 | 0.678696215 | down |
| CTD-2644I21.1 | -1.212003 | 0.678696215 | down |
| RP11-566K19.9 | -1.212003 | 0.678696215 | down |
| Y_RNA | -1.212003 | 0.678696215 | down |
| CTD-2311M21.4 | -1.212003 | 0.678696215 | down |
| RP11-545E8.1 | -1.212003 | 0.678696215 | down |
| RP11-276H1.2 | -1.212003 | 0.678696215 | down |
| RARRES2P9 | -1.212003 | 0.678696215 | down |

| | | | | |
|---|---|---|---|---|
| RARRES2P7 | -1.212003 | 0.678696215 | | down |
| RP11-53L24.1 | -1.212003 | 0.678696215 | | down |
| AC005703.3 | -1.212003 | 0.678696215 | | down |
| KRT31 | -1.212003 | 0.678696215 | | down |
| RN7SL258P | -1.212003 | 0.678696215 | | down |
| CTD-2532D12.5 | -1.212003 | 0.678696215 | | down |
| RP11-384E22.1 | -1.212003 | 0.678696215 | | down |
| LINC01233 | -1.212003 | 0.678696215 | | down |
| CTC-461H2.2 | -1.212003 | 0.678696215 | | down |
| Y_RNA | -1.212003 | 0.678696215 | | down |
| RP1-298M8.1 | -1.212003 | 0.678696215 | | down |
| GAPDHP54 | -1.212003 | 0.678696215 | | down |
| RP1-66N13.4 | -1.212003 | 0.678696215 | | down |
| RP5-827A12.2 | -1.212003 | 0.678696215 | | down |
| LINC00307 | -1.212003 | 0.678696215 | | down |
| KRTAP15-1 | -1.212003 | 0.678696215 | | down |
| KRTAP10-5 | -1.212003 | 0.678696215 | | down |
| CECR9 | -1.212003 | 0.678696215 | | down |
| POM121L8P | -1.212003 | 0.678696215 | | down |
| AC000041.8 | -1.212003 | 0.678696215 | | down |
| RP11-126O22.6 | -1.212003 | 0.678696215 | | down |
| RP11-524P6.1 | -1.212003 | 0.678696215 | | down |
| AF238380.8 | -1.212003 | 0.678696215 | | down |
| PUDPP1 | -1.212003 | 0.678696215 | | down |
| AC008703.1 | -1.212001354 | 0.672492175 | | down |
| AC104978.1 | -1.211999594 | 0.666082169 | | down |
| RP11-120M18.5 | -1.211666026 | 0.192719293 | 0.320707789 | down |
| RP11-76E17.4 | -1.21135621 | 0.294903062 | | down |
| C19orf35 | -1.211277103 | 6.50E-05 | 0.000661083 | down |
| RP11-147K16.3 | -1.210967597 | 0.617554558 | | down |
| KB-173C10.2 | -1.210809178 | 0.108255422 | 0.214017627 | down |
| KRT18P29 | -1.210728803 | 0.225241158 | | down |
| AKAP14 | -1.210624967 | 0.240160359 | | down |
| RPL7P6 | -1.210421737 | 0.012172065 | 0.042645938 | down |
| RP11-98D18.9 | -1.210379485 | 0.006246905 | 0.02572396 | down |
| BARX2 | -1.210213849 | 1.81E-05 | 0.000230666 | down |
| OR5B21 | -1.210166526 | 0.621371239 | | down |
| NA | -1.210029334 | 0.047235796 | 0.117324094 | down |
| RN7SL444P | -1.209969719 | 0.450392906 | | down |
| C8orf34 | -1.209689744 | 0.035094603 | 0.094250672 | down |
| RP13-582O9.7 | -1.209561874 | 0.002137356 | 0.01107059 | down |
| FAM227A | -1.209536909 | 0.002984658 | 0.014439787 | down |
| CRB1 | -1.209490541 | 0.13459124 | 0.249142316 | down |
| NA | -1.209355954 | 0.663027259 | | down |
| RP3-508D13.1 | -1.209324881 | 0.614189361 | | down |
| RP11-417B4.3 | -1.209293023 | 0.679326069 | | down |
| TRIM40 | -1.209015194 | 0.010763013 | 0.038805229 | down |
| FGF12-AS2 | -1.208996692 | 0.577333103 | | down |
| DCXR | -1.208958008 | 2.29E-05 | 0.000279277 | down |
| ADRA1B | -1.208750386 | 0.00253029 | 0.0126888 | down |
| SPATA3-AS1 | -1.208659861 | 0.013499033 | 0.046045068 | down |

| | | | | |
|---|---|---|---|---|
| GCNT2 | -1.208106528 | 1.75E-08 | 6.39E-07 | down |
| HNRNPA1P40 | -1.207947393 | 0.217099195 | 0.348354324 | down |
| RP11-705C15.4 | -1.207653633 | 0.202263221 | 0.331548111 | down |
| CTC-458I2.2 | -1.207623508 | 0.150943891 | 0.270192941 | down |
| TTC3-AS1 | -1.207466827 | 0.277868621 | | down |
| uc_338 | -1.207453204 | 0.352804844 | | down |
| AC002064.4 | -1.207355752 | 0.312425608 | | down |
| NPAP1P3 | -1.207290513 | 0.582100444 | | down |
| RN7SL559P | -1.206942557 | 0.621843664 | | down |
| NA | -1.206789325 | 0.660657398 | | down |
| GALM | -1.206786757 | 2.28E-12 | 2.31E-10 | down |
| U91324.1 | -1.206760065 | 0.680004368 | | down |
| Six3os1_5 | -1.206760065 | 0.680004368 | | down |
| RP11-180K7.1 | -1.206760065 | 0.680004368 | | down |
| CTD-219904.1 | -1.206760065 | 0.680004368 | | down |
| RP11-409011.3 | -1.206760065 | 0.680004368 | | down |
| PRPF38AP1 | -1.206760065 | 0.680004368 | | down |
| RP11-266E8.2 | -1.206760065 | 0.680004368 | | down |
| RP11-481E4.2 | -1.206760065 | 0.680004368 | | down |
| RP11-678G15.1 | -1.206760065 | 0.680004368 | | down |
| RP11-157B13.3 | -1.206760065 | 0.680004368 | | down |
| ATG3P1 | -1.206760065 | 0.680004368 | | down |
| TUBB4AP1 | -1.206760065 | 0.680004368 | | down |
| GAPDHP73 | -1.206748426 | 0.66870311 | | down |
| RP11-815J4.5 | -1.206748203 | 0.668491677 | | down |
| CTD-3187F8.7 | -1.206746499 | 0.666883166 | | down |
| SLIT1 | -1.206743304 | 0.001714547 | 0.009293133 | down |
| 5S_rRNA | -1.206701107 | 0.487689656 | | down |
| IL36B | -1.206664742 | 0.680028258 | | down |
| RP11-802023.4 | -1.20665157 | 0.666435326 | | down |
| KIF3B | -1.206592061 | 1.79E-10 | 1.14E-08 | down |
| NA | -1.206552271 | 0.680056444 | | down |
| RP1-127H14.3 | -1.206541388 | 0.668548032 | | down |
| CTB-23I7.1 | -1.206537871 | 0.664946137 | | down |
| RPL5P27 | -1.206462211 | 0.671103076 | | down |
| AC104794.4 | -1.206354752 | 0.670859237 | | down |
| Vault | -1.206351575 | 0.667295443 | | down |
| RP11-449D8.5 | -1.206297466 | 0.011682233 | 0.041350259 | down |
| RP11-410D17.2 | -1.206245366 | 0.169874644 | | down |
| DIO3OS | -1.206232203 | 4.44E-05 | 0.000483901 | down |
| CTBP2P8 | -1.20609431 | 0.315883701 | | down |
| CLUL1 | -1.205891108 | 0.375980486 | | down |
| RP11-398A8.5 | -1.205692701 | 0.337243599 | | down |
| RP11-93K22.6 | -1.205672037 | 0.667166953 | | down |
| MED6P1 | -1.205652871 | 0.679641515 | | down |
| TNNI1 | -1.205513809 | 7.07E-06 | 0.000105822 | down |
| LGALS2 | -1.205360427 | 4.43E-06 | 7.17E-05 | down |
| MIR23B | -1.205359493 | 0.498244078 | | down |
| HMGN2P46 | -1.204958312 | 9.79E-06 | 0.00013867 | down |
| NA | -1.204858116 | 0.253168473 | 0.388090903 | down |
| RP11-35609.1 | -1.204573628 | 0.495906602 | | down |

| | | | | |
|---|---|---|---|---|
| MIR3145 | -1.20449242 | 0.623966504 | | down |
| B3GALT2 | -1.204447318 | 0.003618139 | 0.016809478 | down |
| U3 | -1.204414721 | 0.535302612 | | down |
| MYCNUT | -1.204364434 | 0.660999125 | | down |
| RBMS3-AS3 | -1.204231402 | 0.00392699 | 0.017974177 | down |
| RP11-545D22.1 | -1.204063275 | 0.202824167 | | down |
| ALKBH3-AS1 | -1.203998828 | 0.016781583 | 0.054295269 | down |
| RN7SL452P | -1.203970812 | 0.194283202 | 0.322488647 | down |
| BEND7 | -1.203956878 | 3.88E-05 | 0.000433632 | down |
| LINC01123 | -1.203666765 | 2.74E-05 | 0.000324131 | down |
| MIR101-1 | -1.203487475 | 0.621242136 | | down |
| MTCO2P2 | -1.20338258 | 0.00595574 | 0.024814069 | down |
| ULK4P2 | -1.203332377 | 0.211790839 | | down |
| ERVW-1 | -1.203023262 | 0.137109042 | 0.252454745 | down |
| PSMD10P2 | -1.202798536 | 0.113013222 | 0.220378349 | down |
| RP11-363J20.2 | -1.202731662 | 0.288627022 | | down |
| RLBP1 | -1.202682836 | 0.056593486 | 0.133913105 | down |
| KDM8 | -1.202621757 | 1.81E-08 | 6.53E-07 | down |
| RP11-640L9.2 | -1.202430192 | 0.005689351 | 0.023929843 | down |
| NA | -1.202333868 | 0.189089539 | 0.316214803 | down |
| NA | -1.202297125 | 0.681051253 | | down |
| RP11-449J1.1 | -1.202158785 | 0.38658433 | | down |
| RAB19 | -1.201876167 | 3.44E-05 | 0.000391301 | down |
| TPSB2 | -1.201846046 | 0.002280513 | 0.011684128 | down |
| SMG1P6 | -1.20175771 | 0.018768815 | 0.059176228 | down |
| B3GALT5-AS1 | -1.201609011 | 0.00642954 | 0.026295819 | down |
| SLC30A4 | -1.201298543 | 9.53E-11 | 6.54E-09 | down |
| RP11-346D6.4 | -1.201169407 | 0.61621779 | | down |
| RFPL4AL1 | -1.201076676 | 0.346296998 | | down |
| OLIG2 | -1.200884398 | 0.133123718 | 0.24728385 | down |
| RP11-417L14.1 | -1.200878486 | 0.270982094 | | down |
| PARD6G-AS1 | -1.200872751 | 0.00014039 | 0.00125234 | down |
| TSSC4 | -1.200796998 | 4.32E-05 | 0.000472421 | down |
| RP13-317D12.3 | -1.200666757 | 0.001255653 | 0.007264554 | down |
| GACAT2 | -1.200551642 | 0.176780385 | | down |
| TMEM63B | -1.20047405 | 3.71E-06 | 6.19E-05 | down |
| AC011747.4 | -1.200424102 | 0.000491603 | 0.003429965 | down |
| MFSD11 | -1.200206059 | 2.18E-06 | 4.00E-05 | down |
| RP11-351C8.1 | -1.199862476 | 0.56106466 | 0.680369116 | down |
| ILVBL | -1.199635158 | 7.75E-07 | 1.63E-05 | down |
| WDR38 | -1.199492447 | 0.09638598 | 0.196911474 | down |
| PON1 | -1.19929104 | 0.075559876 | 0.165206021 | down |
| CTD-2568A17.8 | -1.199158409 | 0.476660437 | | down |
| RP11-703M24.5 | -1.19893911 | 0.447280185 | 0.580566399 | down |
| KAAG1 | -1.198904597 | 0.577793564 | | down |
| XK | -1.198734414 | 8.92E-05 | 0.000859762 | down |
| RP3-414A15.10 | -1.19863165 | 0.273022882 | | down |
| ERN2 | -1.198426707 | 0.001117289 | 0.00663265 | down |
| ANKRD20A5P | -1.198412933 | 0.012359486 | 0.043122563 | down |
| NA | -1.198397231 | 0.146534008 | 0.264730471 | down |
| TTC38 | -1.198284845 | 3.98E-10 | 2.31E-08 | down |

| | | | | |
|---|---|---|---|---|
| C19orf44 | -1.198180054 | 0.000175057 | 0.001497555 | down |
| ASS1P1 | -1.198158393 | 0.036669901 | 0.097358652 | down |
| MIR6835 | -1.198132075 | 0.19843075 | | down |
| RAB3B | -1.197914065 | 0.000261608 | 0.002087585 | down |
| FGF9 | -1.197911814 | 0.003286889 | 0.015548647 | down |
| RP11-53B2.1 | -1.197904602 | 0.186314356 | | down |
| LA16c-360A4.1 | -1.197776888 | 0.074118851 | 0.162922647 | down |
| MUC12 | -1.197653755 | 0.013995342 | 0.047372246 | down |
| C19orf70 | -1.197607803 | 1.65E-05 | 0.000214544 | down |
| TMEM132E | -1.197362255 | 0.004582412 | 0.020273006 | down |
| STKLD1 | -1.197325867 | 0.000395786 | 0.002891779 | down |
| TSPEAR-AS1 | -1.197306785 | 0.017291877 | 0.055569277 | down |
| LHFPL4 | -1.197045888 | 0.007774027 | 0.030321025 | down |
| MMP8 | -1.196747594 | 0.151851321 | 0.271221751 | down |
| ADTRP | -1.196598741 | 2.06E-05 | 0.000257502 | down |
| MYL12AP1 | -1.196285712 | 0.628021224 | | down |
| RP11-235E17.6 | -1.196151748 | 0.001076795 | 0.006434216 | down |
| ZSWIM5 | -1.195739834 | 2.99E-06 | 5.18E-05 | down |
| NA | -1.1955637 | 0.332515653 | 0.470875767 | down |
| OSMR-AS1 | -1.195405684 | 0.002696564 | 0.013340287 | down |
| ZFHX2 | -1.195401451 | 0.000832207 | 0.005240579 | down |
| TRIM49B | -1.195107886 | 0.472534646 | 0.60286343 | down |
| RP11-26F2.2 | -1.194976099 | 0.577102601 | | down |
| NA | -1.19479552 | 0.459102701 | | down |
| AC007969.4 | -1.194704808 | 0.399139824 | | down |
| RP11-761I4.5 | -1.19446017 | 0.119654703 | 0.229704309 | down |
| RP11-53L24.2 | -1.194349339 | 0.617623335 | | down |
| RP11-757F18.5 | -1.19431515 | 0.001939578 | 0.010223511 | down |
| HNF1B | -1.194279636 | 4.19E-05 | 0.000461225 | down |
| OR2A4 | -1.194128123 | 0.4951378 | | down |
| NA | -1.193650943 | 0.52876006 | 0.652110638 | down |
| RP11-390N6.1 | -1.19364467 | 0.423698742 | | down |
| CSMD2 | -1.19363331 | 0.009750478 | 0.035935151 | down |
| CIB4 | -1.193574596 | 0.308594424 | | down |
| NA | -1.193529314 | 0.003275952 | 0.015514299 | down |
| STMND1 | -1.19347036 | 0.052629947 | 0.126831966 | down |
| RP11-380M21.4 | -1.193429791 | 0.049089308 | 0.120749892 | down |
| AC090945.1 | -1.193308296 | 0.08400267 | 0.17837135 | down |
| PPFIA4 | -1.193303029 | 0.002299204 | 0.011761841 | down |
| NA | -1.193109669 | 0.002173783 | 0.011219574 | down |
| S100A14 | -1.193020441 | 0.000337095 | 0.002562671 | down |
| LINC01170 | -1.192885534 | 0.126961619 | 0.239194512 | down |
| DNAH17 | -1.192747923 | 0.001123625 | 0.006660396 | down |
| AC137932.5 | -1.192709373 | 0.004396194 | 0.019637116 | down |
| PCDH10 | -1.192513705 | 0.051277219 | 0.124577292 | down |
| RP3-406A7.7 | -1.192426675 | 0.002995091 | 0.014476272 | down |
| CTD-2144E22.9 | -1.192353882 | 0.379802418 | | down |
| EEF1A1P36 | -1.192262813 | 0.543473067 | | down |
| RP11-826N14.4 | -1.192169419 | 0.373195399 | | down |
| TRIM15 | -1.192097722 | 0.000606257 | 0.004068434 | down |
| RP5-1184F4.5 | -1.191972462 | 0.183948259 | 0.309796054 | down |

| | | | | |
|---|---|---|---|---|
| RP11-429E11.3 | -1.191726037 | 0.284847328 | | down |
| RP11-118H4.3 | -1.191663725 | 0.683483833 | | down |
| RP11-738B7.1 | -1.191570844 | 0.543026099 | | down |
| RP11-572C15.5 | -1.191368396 | 0.621006757 | | down |
| AC001226.7 | -1.191302638 | 0.077328524 | 0.168011148 | down |
| RP11-60C6.3 | -1.191268786 | 0.683823166 | | down |
| RP11-830F9.7 | -1.191262857 | 0.00698087 | 0.027945804 | down |
| NA | -1.191189616 | 0.68384308 | | down |
| TAC4 | -1.191096354 | 0.015138448 | 0.050290192 | down |
| RP11-383G10.3 | -1.19101609 | 0.367759717 | | down |
| AC016691.2 | -1.19095606 | 0.301991341 | | down |
| RP11-571F15.3 | -1.190881249 | 0.000411073 | 0.002970956 | down |
| RP11-520A21.1 | -1.190777504 | 0.625346474 | | down |
| GGN | -1.190578628 | 0.004104077 | 0.018614568 | down |
| NA | -1.19056488 | 0.366075153 | | down |
| RP5-1025A1.2 | -1.19045518 | 0.657545391 | | down |
| PLAC9 | -1.190367729 | 0.00043282 | 0.003098322 | down |
| TTYH1 | -1.190278665 | 0.000674715 | 0.00442891 | down |
| RGS7 | -1.190238458 | 0.204565109 | 0.334230368 | down |
| TTC39C-AS1 | -1.190109611 | 0.001226083 | 0.007131174 | down |
| OR2A20P | -1.189896347 | 0.002953538 | 0.014323937 | down |
| RP3-527G5.1 | -1.189740899 | 0.462798277 | | down |
| AC006547.13 | -1.189713853 | 0.001757838 | 0.009471393 | down |
| TUSC8 | -1.189307389 | 0.000785164 | 0.004999888 | down |
| DANT2 | -1.189266371 | 0.113247395 | 0.220677438 | down |
| AC097662.2 | -1.189102411 | 0.000405003 | 0.002937197 | down |
| CTD-2506P8.6 | -1.188876719 | 0.004636 | 0.020467643 | down |
| WFIKKN2 | -1.188806091 | 0.047705265 | 0.118158037 | down |
| NUP210L | -1.188754805 | 0.018652965 | 0.058909878 | down |
| FAM71F2 | -1.188717891 | 0.000706523 | 0.004598519 | down |
| AC114730.3 | -1.188715446 | 0.008928176 | 0.033626442 | down |
| RP11-72I8.1 | -1.18855742 | 0.027100647 | 0.077870299 | down |
| DHRS4 | -1.188524977 | 2.01E-08 | 7.15E-07 | down |
| RP11-438B23.2 | -1.188504083 | 0.288541243 | | down |
| NTHL1 | -1.188251318 | 0.000109015 | 0.00101684 | down |
| NA | -1.188237886 | 0.005128922 | 0.022123951 | down |
| TRDV2 | -1.188067557 | 0.204917233 | | down |
| RP11-499E18.1 | -1.187959337 | 0.002423522 | 0.012266222 | down |
| RP11-736I10.1 | -1.187885005 | 0.657922064 | | down |
| CICP26 | -1.187879136 | 0.50453946 | 0.631204811 | down |
| RP11-248M19.1 | -1.187810825 | 0.032438956 | 0.088841932 | down |
| MTND5P11 | -1.187676703 | 0.005858168 | 0.0245061 | down |
| MARC1 | -1.187385375 | 4.58E-06 | 7.39E-05 | down |
| RP11-399K21.10 | -1.187163926 | 0.541129312 | | down |
| HMGB3P6 | -1.186877962 | 0.166549559 | 0.289660362 | down |
| SLC26A4-AS1 | -1.186653851 | 0.06744501 | 0.152091712 | down |
| MYZAP | -1.186544103 | 3.26E-05 | 0.000374244 | down |
| TNFAIP8L1 | -1.186452282 | 2.38E-05 | 0.000289153 | down |
| RP11-1023L17.1 | -1.186419832 | 0.000291358 | 0.002277598 | down |
| TRAF6P1 | -1.186368437 | 0.68497756 | | down |
| NA | -1.186353543 | 0.000558876 | 0.003808536 | down |

| | | | | |
|---|---|---|---|---|
| RPS12P31 | -1.186216423 | 0.30928789 | 0.447711172 | down |
| CAB39P1 | -1.185981614 | 0.5893734 | | down |
| TAS2R64P | -1.185713981 | 0.367532547 | | down |
| CTD-3126B10.2 | -1.185659862 | 0.128908327 | 0.241861099 | down |
| PON3 | -1.185560076 | 0.000157398 | 0.001371877 | down |
| SCAPER | -1.185443409 | 8.82E-07 | 1.82E-05 | down |
| AC005009.2 | -1.185347948 | 0.234858251 | 0.367657425 | down |
| Y_RNA | -1.185245519 | 0.52461376 | 0.64872834 | down |
| EFCAB9 | -1.185241786 | 0.582435153 | | down |
| ADAM32 | -1.185079329 | 0.006870082 | 0.027601595 | down |
| TNNT1 | -1.18501987 | 0.007028993 | 0.028089943 | down |
| CTC-241F20.4 | -1.184979778 | 0.416337205 | | down |
| MTCO1P42 | -1.184948673 | 0.685462747 | | down |
| ALDOC | -1.184895847 | 0.000156561 | 0.001366961 | down |
| NPFFR1 | -1.184695222 | 0.008427207 | 0.032158899 | down |
| CTD-2509G16.2 | -1.184257154 | 0.042852905 | 0.109385954 | down |
| NA | -1.184104682 | 0.255389499 | | down |
| LINC01364 | -1.184075911 | 0.46480039 | | down |
| FAM207BP | -1.184016323 | 0.040100722 | 0.104142991 | down |
| GDPD4 | -1.183890135 | 0.086563338 | 0.182441875 | down |
| RP11-45M22.5 | -1.183855553 | 0.583167353 | | down |
| RP11-357N13.3 | -1.183764257 | 0.289733301 | 0.426996336 | down |
| NA | -1.183662796 | 0.160866235 | 0.282387681 | down |
| CROCC2 | -1.183643323 | 0.008104209 | 0.031225757 | down |
| AC010967.2 | -1.18361572 | 0.475525356 | 0.60549624 | down |
| CASC9 | -1.183543479 | 0.003600089 | 0.016743199 | down |
| RBBP4P1 | -1.183443954 | 0.128965845 | 0.241923692 | down |
| EXD3 | -1.183161538 | 1.87E-07 | 4.89E-06 | down |
| ANO7L1 | -1.18308136 | 0.000121673 | 0.001115175 | down |
| RGS20 | -1.183014235 | 0.046586908 | 0.11607705 | down |
| PPP1R1C | -1.182994055 | 0.00603188 | 0.025058323 | down |
| SNORD114-18 | -1.182854942 | 0.453009453 | 0.585588441 | down |
| ZKSCAN1 | -1.182685998 | 1.82E-10 | 1.15E-08 | down |
| RP1-212P9.3 | -1.182617299 | 0.586259946 | | down |
| NA | -1.182537147 | 0.686047052 | | down |
| ACTN4P1 | -1.182513016 | 0.128845302 | 0.241803251 | down |
| FTCD | -1.182364263 | 0.00892182 | 0.033610929 | down |
| ZNF714 | -1.182176631 | 1.62E-05 | 0.000211535 | down |
| LINC01447 | -1.182129221 | 0.032012363 | 0.087986408 | down |
| RPL10P9 | -1.182012304 | 3.82E-05 | 0.000428706 | down |
| EDARADD | -1.18176569 | 0.001209426 | 0.007060659 | down |
| CD160 | -1.18173825 | 0.007784411 | 0.030353648 | down |
| AC018462.2 | -1.181703733 | 0.097408978 | 0.198272667 | down |
| NA | -1.181553188 | 0.661584606 | | down |
| LLNLF-187D8.1 | -1.181423679 | 0.07947107 | 0.171308194 | down |
| TEX26 | -1.181389626 | 0.252334239 | 0.387304474 | down |
| NBL1 | -1.181161055 | 2.11E-05 | 0.000262181 | down |
| RP11-351J23.2 | -1.181068902 | 0.082271817 | 0.175889453 | down |
| CTD-2616J11.14 | -1.180873633 | 0.54809962 | | down |
| AP001469.9 | -1.180581742 | 0.000121068 | 0.001111 | down |
| ATP6V1B1 | -1.180536002 | 0.179899202 | 0.305188685 | down |

| | | | | |
|---|---|---|---|---|
| ADAMTS9-AS2 | -1.180523502 | 0.004848023 | 0.0211922 | down |
| HP09025 | -1.180406069 | 0.047518049 | 0.117820605 | down |
| RP11-182J1.13 | -1.180109044 | 0.510800351 | | down |
| RP11-354K4.2 | -1.180077103 | 0.442231896 | | down |
| RP11-403A3.3 | -1.179966731 | 0.059256105 | 0.138472524 | down |
| AP000344.3 | -1.17986493 | 0.029817271 | 0.083394669 | down |
| NA | -1.179702827 | 0.126236746 | 0.2383754 | down |
| TMIGD2 | -1.179505518 | 0.000371424 | 0.002754644 | down |
| RP5-965G21.4 | -1.179483136 | 0.001127985 | 0.006681619 | down |
| LINC00498 | -1.179241422 | 0.664440296 | | down |
| RETNLB | -1.179220429 | 0.006724333 | 0.02717593 | down |
| FRK | -1.178982073 | 6.35E-05 | 0.000650003 | down |
| BMPR1B-AS1 | -1.178836646 | 0.623035731 | | down |
| HR | -1.178736643 | 1.98E-07 | 5.13E-06 | down |
| RNU6-1190P | -1.178654474 | 0.412121136 | 0.548861536 | down |
| PLEKHH1 | -1.178570022 | 0.000246878 | 0.001990825 | down |
| PPP2R2C | -1.178263744 | 0.003559672 | 0.016606642 | down |
| MUC3A | -1.178230317 | 9.44E-06 | 0.000134712 | down |
| RP11-180M15.4 | -1.178209507 | 0.081562762 | 0.174634523 | down |
| NA | -1.178087333 | 0.353037356 | | down |
| ?P1-UPK3BP1-PMS? | -1.17791049 | 0.000252082 | 0.00202447 | down |
| RP1-59D14.9 | -1.177615637 | 0.540087989 | | down |
| FAM160A1 | -1.177607882 | 3.17E-05 | 0.000366481 | down |
| SPATA1 | -1.177488671 | 0.000768577 | 0.004916144 | down |
| HCCAT5 | -1.177262406 | 0.377321006 | | down |
| MIR3150B | -1.177248062 | 0.02255169 | 0.067835701 | down |
| MCPH1-AS1 | -1.177216853 | 1.40E-05 | 0.000187209 | down |
| RP11-272G22.3 | -1.177083621 | 0.587220657 | | down |
| MIR7845 | -1.17697072 | 0.135901656 | 0.250934399 | down |
| TMEM219 | -1.176952341 | 1.29E-05 | 0.000175055 | down |
| MSI1 | -1.176841306 | 3.05E-05 | 0.000354809 | down |
| RP5-1039K5.18 | -1.17654293 | 0.509632729 | | down |
| ABHD14B | -1.176539365 | 3.85E-08 | 1.26E-06 | down |
| AC084117.3 | -1.176430795 | 0.029923667 | 0.083607053 | down |
| RP11-285E23.2 | -1.176393062 | 0.195552898 | | down |
| OR4E2 | -1.176115461 | 0.421882279 | 0.557413747 | down |
| SSUH2 | -1.175596829 | 0.000191631 | 0.001613613 | down |
| RP11-181G12.4 | -1.175535513 | 0.112507337 | 0.219662751 | down |
| CTD-2349P21.3 | -1.175399585 | 0.061373138 | 0.142188045 | down |
| NA | -1.17539666 | 0.667219144 | | down |
| GPR137C | -1.175226474 | 0.006694484 | 0.02710269 | down |
| NA | -1.175218455 | 0.040963089 | 0.105743751 | down |
| LINC01095 | -1.174964746 | 0.244960428 | 0.378852625 | down |
| FUT3 | -1.17476756 | 0.002429641 | 0.012288913 | down |
| ZNF843 | -1.174714229 | 0.002983225 | 0.014435176 | down |
| ZNF280B | -1.174645015 | 0.001599954 | 0.008784545 | down |
| NOCT | -1.174610812 | 0.000237755 | 0.001930047 | down |
| ZAR1L | -1.17459634 | 0.134602973 | 0.249142316 | down |
| RP11-131L23.1 | -1.174445724 | 0.005035384 | 0.021814429 | down |
| AL022344.2 | -1.174376687 | 0.688077025 | | down |
| TBC1D21 | -1.174180944 | 0.350395377 | 0.489117629 | down |

| | | | | |
|---|---|---|---|---|
| TDRD5 | -1.174109924 | 0.012842024 | 0.044351106 | down |
| MBOAT4 | -1.17366453 | 0.059305221 | 0.138524785 | down |
| RP11-14I17.3 | -1.173523721 | 0.479480131 | | down |
| ZFP57 | -1.173443382 | 0.142177748 | 0.259041132 | down |
| CTB-50L17.8 | -1.173378259 | 0.000518289 | 0.003580469 | down |
| PHKG1 | -1.173246599 | 0.000222057 | 0.001822806 | down |
| RP11-613C6.2 | -1.173218923 | 0.473040032 | | down |
| NA | -1.172915176 | 0.355426712 | | down |
| KRT18P33 | -1.17273208 | 0.370855061 | | down |
| AKR1B15 | -1.172319467 | 0.126731791 | 0.238948565 | down |
| RAB11FIP1 | -1.172051255 | 1.76E-05 | 0.000225636 | down |
| TECR | -1.172037961 | 7.18E-05 | 0.000716306 | down |
| RP11-613M10.6 | -1.171521267 | 0.000245692 | 0.001983099 | down |
| NA | -1.171499919 | 0.484187441 | | down |
| KRT18 | -1.17149552 | 0.000736083 | 0.004752821 | down |
| DUXAP8 | -1.171320573 | 0.015516532 | 0.051279142 | down |
| C9orf129 | -1.171180715 | 0.392882303 | | down |
| NA | -1.17117517 | 0.002841362 | 0.013891928 | down |
| NA | -1.17057476 | 0.479375988 | | down |
| TMEM151B | -1.170510705 | 0.04035387 | 0.104615728 | down |
| JOSD2 | -1.1705001 | 7.68E-05 | 0.000759199 | down |
| BPHL | -1.170317122 | 3.88E-05 | 0.000433989 | down |
| RP11-566H8.3 | -1.170305729 | 0.625177812 | | down |
| PCCA-AS1 | -1.170165293 | 0.337722462 | | down |
| C2orf27AP3 | -1.170070124 | 0.287165997 | | down |
| RPL21P120 | -1.169878836 | 0.029523169 | 0.082888898 | down |
| RP11-616M22.3 | -1.169853001 | 0.15182968 | 0.271199221 | down |
| KCTD19 | -1.169679246 | 0.051611837 | 0.125171621 | down |
| AARSD1 | -1.169674603 | 0.001364449 | 0.00774339 | down |
| ZNF385C | -1.169555353 | 0.000473325 | 0.003327227 | down |
| ENTPD5 | -1.169533095 | 2.18E-05 | 0.000268155 | down |
| NA | -1.16940875 | 0.628367516 | 0.736814989 | down |
| NA | -1.169402885 | 0.548599574 | | down |
| RP11-481J2.2 | -1.169129083 | 0.151960384 | 0.271344445 | down |
| LINC01257 | -1.169105445 | 0.0526487 | 0.126856808 | down |
| RP11-72L22.1 | -1.16901164 | 0.350014119 | 0.488767101 | down |
| CYP1B1-AS1 | -1.168916465 | 0.001474504 | 0.008224373 | down |
| MT-ATP6 | -1.168905072 | 0.007729682 | 0.030186551 | down |
| RP11-281N10.1 | -1.168832514 | 0.348077847 | 0.48687792 | down |
| RP11-38107.3 | -1.168800847 | 0.387172231 | | down |
| CERS3 | -1.168752428 | 0.129082826 | 0.242067562 | down |
| C6orf99 | -1.168596117 | 0.001042157 | 0.006269636 | down |
| RP3-431A14.4 | -1.168594116 | 0.445444693 | | down |
| RP11-252A24.5 | -1.168575242 | 0.006595189 | 0.026765615 | down |
| RP11-80H5.2 | -1.168338851 | 0.176107317 | | down |
| CTD-2074D8.1 | -1.168070777 | 0.689667305 | | down |
| RP5-1139I1.1 | -1.168070777 | 0.689667305 | | down |
| PLA2G4E-AS1 | -1.167845846 | 0.346523603 | | down |
| RP4-803J11.2 | -1.167722 | 0.210844345 | | down |
| LENEP | -1.167097504 | 0.412861041 | | down |
| RP11-426J5.1 | -1.167075769 | 0.630732106 | | down |

| | | | | |
|---|---|---|---|---|
| MEIOB | -1.167024908 | 0.109341383 | 0.215589336 | down |
| RP11-160E2.19 | -1.166894653 | 0.408867731 | | down |
| RP11-579D7.4 | -1.166779158 | 0.003205416 | 0.015252379 | down |
| CTC-451P13.1 | -1.166776833 | 0.001755889 | 0.00946938 | down |
| C14orf180 | -1.166757801 | 0.061863701 | 0.142971771 | down |
| PLCH1 | -1.166657468 | 0.004676017 | 0.020599158 | down |
| PHBP13 | -1.166538739 | 0.323721955 | | down |
| RPL5P32 | -1.166499185 | 0.445653043 | | down |
| RP11-413N10.3 | -1.166436228 | 0.297901488 | 0.43538222 | down |
| NA | -1.166344303 | 0.588093026 | | down |
| RP5-1029K10.4 | -1.166231531 | 0.516334455 | | down |
| CTHRC1P1 | -1.165971997 | 0.673454444 | | down |
| PLA2R1 | -1.165958539 | 8.44E-05 | 0.000820946 | down |
| UQCC3 | -1.165953916 | 4.64E-05 | 0.000502517 | down |
| RNU6-820P | -1.165651215 | 0.690277824 | | down |
| ELANE | -1.16548769 | 0.001267533 | 0.007312168 | down |
| RP11-53B2.3 | -1.165204121 | 0.166052347 | 0.28907618 | down |
| NCRUPAR_1 | -1.16511088 | 0.690293308 | | down |
| LINC01514 | -1.165013718 | 0.673673662 | | down |
| NA | -1.164959923 | 0.044127197 | 0.111652139 | down |
| DNAJB7 | -1.164941585 | 0.096935907 | 0.197674771 | down |
| NRBF2P3 | -1.164892351 | 0.324976194 | 0.463431972 | down |
| RP11-182J1.10 | -1.16480229 | 0.589825668 | | down |
| ZNF695 | -1.164672691 | 0.055150234 | 0.1313352 | down |
| RP3-337H4.9 | -1.164650491 | 0.212661496 | | down |
| RP11-81A22.4 | -1.164643841 | 2.39E-08 | 8.23E-07 | down |
| RN7SL395P | -1.164575686 | 0.404664627 | | down |
| NR2F6 | -1.164563428 | 1.30E-05 | 0.000176091 | down |
| RP11-430C1.2 | -1.164331373 | 0.270088491 | 0.406083978 | down |
| KRTAP10-12 | -1.164251929 | 0.625357823 | | down |
| GAPDHP76 | -1.164244207 | 0.210748786 | 0.341333068 | down |
| RP11-687F6.4 | -1.16415861 | 0.400621299 | | down |
| GNG3 | -1.164015273 | 0.120395782 | 0.230686918 | down |
| EFNA1 | -1.163228838 | 1.51E-06 | 2.91E-05 | down |
| ITGAD | -1.163116591 | 0.018644977 | 0.058897035 | down |
| ITGBL1 | -1.163109759 | 0.046701756 | 0.116279783 | down |
| NA | -1.162923677 | 0.551026329 | | down |
| CNTD1 | -1.162899271 | 0.000179757 | 0.00153296 | down |
| HIST3H2BA | -1.162686826 | 0.294155491 | | down |
| WFDC3 | -1.162406936 | 0.003155057 | 0.015055675 | down |
| RP3-467N11.2 | -1.162390571 | 0.290766671 | | down |
| ERVFRD-1 | -1.162337786 | 0.01034703 | 0.037655545 | down |
| AC003088.1 | -1.162330599 | 0.628327318 | | down |
| RP11-473I1.6 | -1.162000245 | 0.557133311 | | down |
| RBKS | -1.161993952 | 9.80E-08 | 2.79E-06 | down |
| TCEB2 | -1.161910098 | 4.06E-05 | 0.000449718 | down |
| RP11-273G15.2 | -1.161794684 | 0.00944475 | 0.035096853 | down |
| NA | -1.16177865 | 0.001889002 | 0.01002352 | down |
| CTD-2017D11.2 | -1.161700184 | 0.032611647 | 0.08919293 | down |
| EIF1B-AS1 | -1.161697264 | 6.86E-05 | 0.000690031 | down |
| NA | -1.161499866 | 0.411065206 | 0.547843812 | down |

| | | | | |
|---|---|---|---|---|
| AC105052.1 | -1.161425887 | 0.000301694 | 0.002339121 | down |
| RP11-320M2.1 | -1.161349467 | 5.23E-06 | 8.22E-05 | down |
| RP11-283G6.4 | -1.16134206 | 0.038405123 | 0.100816426 | down |
| TBX6 | -1.161191435 | 0.00013872 | 0.001240019 | down |
| TIMM13 | -1.160820436 | 1.31E-05 | 0.000177206 | down |
| CEBPE | -1.160738716 | 0.001894125 | 0.010043609 | down |
| GSTZ1 | -1.160428981 | 1.47E-06 | 2.85E-05 | down |
| RP11-269C23.5 | -1.160372818 | 0.134527061 | 0.249059097 | down |
| RP4-800M22.1 | -1.1602902 | 0.0076545 | 0.029975468 | down |
| NYX | -1.160187979 | 0.435453483 | | down |
| Y_RNA | -1.160179199 | 0.55112499 | | down |
| AP001619.3 | -1.160077859 | 0.221094157 | | down |
| NAT8L | -1.159927416 | 0.000741328 | 0.004779497 | down |
| RP5-888M10.2 | -1.159800179 | 0.264227257 | | down |
| RNU1-1 | -1.159673227 | 0.188175199 | 0.315035041 | down |
| RNU6-964P | -1.159570307 | 0.664641678 | | down |
| RP11-524N5.1 | -1.159319204 | 0.03834666 | 0.100724519 | down |
| RP11-716O23.2 | -1.159313794 | 0.548812751 | | down |
| TRDV3 | -1.159178938 | 0.16480227 | | down |
| CLK3P2 | -1.159113731 | 0.519516083 | | down |
| RP5-1021I20.6 | -1.159100267 | 0.595002767 | | down |
| RHOV | -1.159033999 | 0.002899767 | 0.014121268 | down |
| RP11-6B4.1 | -1.15899759 | 0.377751831 | | down |
| SFTPD-AS1 | -1.158950048 | 0.211506738 | 0.34210005 | down |
| ALG1L | -1.15892083 | 0.009536802 | 0.035342751 | down |
| ABAT | -1.158904814 | 1.64E-05 | 0.000213087 | down |
| NA | -1.158662385 | 0.288956113 | | down |
| MIR339 | -1.158312949 | 0.045935691 | 0.114870341 | down |
| RAB26 | -1.158235629 | 0.00011805 | 0.001088941 | down |
| AC015987.1 | -1.158044673 | 0.148674382 | 0.267375382 | down |
| TPSAB1 | -1.157896877 | 0.001240509 | 0.007197732 | down |
| RP4-621N11.2 | -1.157632561 | 0.238901278 | 0.372353645 | down |
| RP11-657O9.1 | -1.157480797 | 0.108472395 | 0.214347845 | down |
| AC009505.2 | -1.157452564 | 0.157493376 | 0.278235226 | down |
| RP11-213G21.2 | -1.15718765 | 0.547114113 | | down |
| USP43 | -1.157152583 | 5.20E-06 | 8.18E-05 | down |
| WDR34 | -1.15697835 | 2.15E-06 | 3.94E-05 | down |
| NKAIN4 | -1.156952042 | 0.030899671 | 0.085656921 | down |
| RP11-141M1.3 | -1.156939228 | 0.002167203 | 0.011189458 | down |
| ZNF285B | -1.156896842 | 0.186450611 | 0.312770639 | down |
| VWCE | -1.156680876 | 0.001621885 | 0.008887092 | down |
| IFNE | -1.156518112 | 0.377491771 | | down |
| RP11-418H16.1 | -1.156450807 | 0.1170064 | 0.226007919 | down |
| AC098820.2 | -1.156356846 | 0.518174702 | | down |
| RP11-13P5.1 | -1.156120471 | 0.032840548 | 0.08965574 | down |
| RP11-1090M7.1 | -1.15610659 | 0.112478998 | 0.219635969 | down |
| NA | -1.156000893 | 0.441784362 | | down |
| PCOLCE-AS1 | -1.154739627 | 0.044326545 | 0.112015033 | down |
| TBX3 | -1.154474948 | 1.64E-05 | 0.0002139 | down |
| CTD-3116E22.7 | -1.154439424 | 0.045722701 | 0.114503027 | down |
| ST13P4 | -1.154410203 | 0.087336433 | | down |

| | | | | |
|---|---|---|---|---|
| RP13-1032I1.7 | -1.154390882 | 1.61E-05 | 0.000210986 | down |
| CUBNP2 | -1.154167295 | 0.401431381 | | down |
| SLC20A1 | -1.153944406 | 1.77E-07 | 4.65E-06 | down |
| TTC22 | -1.153937967 | 3.00E-05 | 0.000350016 | down |
| LINC00392 | -1.153661311 | 0.63490768 | | down |
| PTCD2P2 | -1.153516257 | 0.683357835 | | down |
| GPR119 | -1.153473727 | 0.513256475 | | down |
| CTD-2373J6.2 | -1.153430735 | 0.248614523 | | down |
| RP11-254I22.1 | -1.153225814 | 0.043584716 | 0.110767866 | down |
| LINC00365 | -1.153073594 | 0.056356299 | 0.133478018 | down |
| NA | -1.153008554 | 0.113246553 | 0.220677438 | down |
| SLAMF6P1 | -1.152846061 | 0.065253013 | 0.148373686 | down |
| RP11-233E12.1 | -1.152734229 | 0.632264125 | | down |
| RP11-258F1.1 | -1.152700116 | 0.029781674 | 0.083343634 | down |
| PGLS | -1.15252732 | 3.34E-05 | 0.000382267 | down |
| RP1-149A16.17 | -1.152444777 | 0.441145996 | | down |
| KCNF1 | -1.152266502 | 0.004545562 | 0.020163422 | down |
| C1orf194 | -1.152252895 | 0.088888771 | 0.185895464 | down |
| Z83844.1 | -1.152220256 | 0.012842984 | 0.044351106 | down |
| RP11-624L4.1 | -1.152041327 | 0.064688198 | 0.147491252 | down |
| NA | -1.151966919 | 0.693664262 | | down |
| NA | -1.151966216 | 0.69366444 | | down |
| LINC00703 | -1.151966216 | 0.69366444 | | down |
| AC006539.2 | -1.151774438 | 0.516360318 | | down |
| KRT8P13 | -1.151682505 | 0.138781581 | 0.254722574 | down |
| RPL21P44 | -1.151350295 | 0.14109433 | 0.257739286 | down |
| RP11-74E22.6 | -1.151080551 | 0.028090042 | 0.08002439 | down |
| SLC25A1 | -1.15091653 | 9.35E-07 | 1.91E-05 | down |
| SH3BP1 | -1.150858102 | 1.76E-05 | 0.000225823 | down |
| NA | -1.150804353 | 0.126255204 | | down |
| PAQR5 | -1.150751351 | 9.69E-05 | 0.000920349 | down |
| LHX5 | -1.150138128 | 0.298105794 | | down |
| CYP4A22-AS1 | -1.15004499 | 0.226865829 | 0.358980195 | down |
| RP3-437C15.1 | -1.149936529 | 0.19721474 | 0.325806644 | down |
| CTD-2561J22.5 | -1.149899543 | 0.011502248 | 0.040847937 | down |
| DNM1P35 | -1.149743669 | 0.002758762 | 0.013585353 | down |
| EXOSC4 | -1.149703443 | 0.000895732 | 0.005545306 | down |
| MRPL28 | -1.149077145 | 8.77E-05 | 0.000847153 | down |
| SALL4P2 | -1.149019374 | 0.635311327 | | down |
| RP11-482M8.3 | -1.148468399 | 0.317553476 | | down |
| NA | -1.147965929 | 0.505839397 | | down |
| HNRNPCL2 | -1.147914919 | 0.694809431 | | down |
| SNORA62 | -1.147914919 | 0.694809431 | | down |
| RP11-42O15.2 | -1.147914919 | 0.694809431 | | down |
| RP5-837I24.2 | -1.147914919 | 0.694809431 | | down |
| NA | -1.147914919 | 0.694809431 | | down |
| C1orf68 | -1.147914919 | 0.694809431 | | down |
| RP11-740C1.2 | -1.147914919 | 0.694809431 | | down |
| RPL29P7 | -1.147914919 | 0.694809431 | | down |
| RNU5A-8P | -1.147914919 | 0.694809431 | | down |
| MIR4427 | -1.147914919 | 0.694809431 | | down |

| | | | |
|---|---|---|---|
| AC073628.1 | -1.147914919 | 0.694809431 | down |
| NA | -1.147914919 | 0.694809431 | down |
| AC009227.3 | -1.147914919 | 0.694809431 | down |
| API5P2 | -1.147914919 | 0.694809431 | down |
| RNA5SP115 | -1.147914919 | 0.694809431 | down |
| SNAI1P1 | -1.147914919 | 0.694809431 | down |
| FBXO36-IT1 | -1.147914919 | 0.694809431 | down |
| AC092421.1 | -1.147914919 | 0.694809431 | down |
| PRR23C | -1.147914919 | 0.694809431 | down |
| RN7SKP177 | -1.147914919 | 0.694809431 | down |
| RN7SKP265 | -1.147914919 | 0.694809431 | down |
| NMNAT1P3 | -1.147914919 | 0.694809431 | down |
| FAM166AP10 | -1.147914919 | 0.694809431 | down |
| MESTP3 | -1.147914919 | 0.694809431 | down |
| U6 | -1.147914919 | 0.694809431 | down |
| SCGB1D5P | -1.147914919 | 0.694809431 | down |
| LINC00290 | -1.147914919 | 0.694809431 | down |
| RP11-308K2.1 | -1.147914919 | 0.694809431 | down |
| CTD-2313F11.3 | -1.147914919 | 0.694809431 | down |
| FTH1P9 | -1.147914919 | 0.694809431 | down |
| CTC-493L21.2 | -1.147914919 | 0.694809431 | down |
| LECT2 | -1.147914919 | 0.694809431 | down |
| NA | -1.147914919 | 0.694809431 | down |
| RNU6-556P | -1.147914919 | 0.694809431 | down |
| RP11-401E14.3 | -1.147914919 | 0.694809431 | down |
| OR2W1 | -1.147914919 | 0.694809431 | down |
| RP1-137F1.3 | -1.147914919 | 0.694809431 | down |
| RP11-228O6.2 | -1.147914919 | 0.694809431 | down |
| NA | -1.147914919 | 0.694809431 | down |
| PHBP1 | -1.147914919 | 0.694809431 | down |
| RP11-486M3.3 | -1.147914919 | 0.694809431 | down |
| RP4-655C5.7 | -1.147914919 | 0.694809431 | down |
| OR7E59P | -1.147914919 | 0.694809431 | down |
| GS1-165B14.2 | -1.147914919 | 0.694809431 | down |
| SLC29A4P1 | -1.147914919 | 0.694809431 | down |
| RP11-561N12.2 | -1.147914919 | 0.694809431 | down |
| TAS2R62P | -1.147914919 | 0.694809431 | down |
| RP11-555E9.1 | -1.147914919 | 0.694809431 | down |
| CTD-3023L14.1 | -1.147914919 | 0.694809431 | down |
| RP11-447G11.1 | -1.147914919 | 0.694809431 | down |
| RP11-521M14.2 | -1.147914919 | 0.694809431 | down |
| RP11-959I15.4 | -1.147914919 | 0.694809431 | down |
| NA | -1.147914919 | 0.694809431 | down |
| RN7SL198P | -1.147914919 | 0.694809431 | down |
| RP11-435M3.2 | -1.147914919 | 0.694809431 | down |
| NA | -1.147914919 | 0.694809431 | down |
| Y_RNA | -1.147914919 | 0.694809431 | down |
| OR51S1 | -1.147914919 | 0.694809431 | down |
| OR5E1P | -1.147914919 | 0.694809431 | down |
| THAP12P4 | -1.147914919 | 0.694809431 | down |
| OR4C14P | -1.147914919 | 0.694809431 | down |

| | | | |
|---|---|---|---|
| OR5D3P | -1.147914919 | 0.694809431 | down |
| OR9G4 | -1.147914919 | 0.694809431 | down |
| OR5G3 | -1.147914919 | 0.694809431 | down |
| OR1S2 | -1.147914919 | 0.694809431 | down |
| RP11-160H12.3 | -1.147914919 | 0.694809431 | down |
| RP11-851M3.1 | -1.147914919 | 0.694809431 | down |
| MIR4301 | -1.147914919 | 0.694809431 | down |
| Y_RNA | -1.147914919 | 0.694809431 | down |
| OPCML-IT1 | -1.147914919 | 0.694809431 | down |
| RP11-776A13.4 | -1.147914919 | 0.694809431 | down |
| RP11-845M18.7 | -1.147914919 | 0.694809431 | down |
| OR6C4 | -1.147914919 | 0.694809431 | down |
| RP11-74K11.1 | -1.147914919 | 0.694809431 | down |
| RNA5SP375 | -1.147914919 | 0.694809431 | down |
| RP11-572C21.2 | -1.147914919 | 0.694809431 | down |
| LINC01066 | -1.147914919 | 0.694809431 | down |
| LINC00358 | -1.147914919 | 0.694809431 | down |
| NA | -1.147914919 | 0.694809431 | down |
| NA | -1.147914919 | 0.694809431 | down |
| RP11-569D9.6 | -1.147914919 | 0.694809431 | down |
| RP11-557C9.1 | -1.147914919 | 0.694809431 | down |
| OR7E106P | -1.147914919 | 0.694809431 | down |
| RP11-173D3.1 | -1.147914919 | 0.694809431 | down |
| MIR4311 | -1.147914919 | 0.694809431 | down |
| RP11-64K10.1 | -1.147914919 | 0.694809431 | down |
| RP11-164C12.2 | -1.147914919 | 0.694809431 | down |
| CTD-2522B17.8 | -1.147914919 | 0.694809431 | down |
| RP11-256I9.2 | -1.147914919 | 0.694809431 | down |
| NA | -1.147914919 | 0.694809431 | down |
| HMGB3P27 | -1.147914919 | 0.694809431 | down |
| CTD-2534I21.9 | -1.147914919 | 0.694809431 | down |
| CRHR1 | -1.147914919 | 0.694809431 | down |
| RP11-110H1.8 | -1.147914919 | 0.694809431 | down |
| LINC01538 | -1.147914919 | 0.694809431 | down |
| RP11-231E4.3 | -1.147914919 | 0.694809431 | down |
| OR7C2 | -1.147914919 | 0.694809431 | down |
| CTD-2231E14.4 | -1.147914919 | 0.694809431 | down |
| CTD-2542C24.14 | -1.147914919 | 0.694809431 | down |
| CEACAMP4 | -1.147914919 | 0.694809431 | down |
| NTF6A | -1.147914919 | 0.694809431 | down |
| VN1R104P | -1.147914919 | 0.694809431 | down |
| RNU6-228P | -1.147914919 | 0.694809431 | down |
| CST9LP1 | -1.147914919 | 0.694809431 | down |
| GAPDHP53 | -1.147914919 | 0.694809431 | down |
| RP11-49G10.3 | -1.147914919 | 0.694809431 | down |
| RP4-614C15.2 | -1.147914919 | 0.694809431 | down |
| FRG2MP | -1.147914919 | 0.694809431 | down |
| NA | -1.147914919 | 0.694809431 | down |
| RP4-539M6.18 | -1.147914919 | 0.694809431 | down |
| EIF5P2 | -1.147914919 | 0.694809431 | down |
| RP11-149B9.2 | -1.147914919 | 0.694809431 | down |

| | | | | |
|---|---|---|---|---|
| Y_RNA | -1.147914919 | 0.694809431 | | down |
| SNORD45 | -1.147914919 | 0.694809431 | | down |
| RNU4-44P | -1.147914919 | 0.694809431 | | down |
| HMGB3P31 | -1.147914919 | 0.694809431 | | down |
| RP11-805F19.1 | -1.147914767 | 0.692441336 | | down |
| RP11-534I8.1 | -1.147914393 | 0.686840218 | | down |
| NA | -1.147914322 | 0.685814756 | | down |
| TCEB1P33 | -1.147914266 | 0.685004098 | | down |
| NA | -1.147914177 | 0.683737025 | | down |
| TLK1P1 | -1.147911191 | 0.647236067 | | down |
| RPL17P33 | -1.147910866 | 0.643766433 | | down |
| LINC01248 | -1.147724161 | 0.694828366 | | down |
| AC016912.3 | -1.147724161 | 0.694828366 | | down |
| RP3-503A6.2 | -1.147724161 | 0.694828366 | | down |
| NEUROD4 | -1.147724161 | 0.694828366 | | down |
| RP11-274J7.2 | -1.147724161 | 0.694828366 | | down |
| IGHV3-37 | -1.147724161 | 0.694828366 | | down |
| RP11-325K19.2 | -1.147724161 | 0.694828366 | | down |
| RP11-227D2.3 | -1.147724161 | 0.694828366 | | down |
| RP11-15M15.1 | -1.147724161 | 0.694828366 | | down |
| NA | -1.147724161 | 0.694828366 | | down |
| RP11-252C15.1 | -1.147618639 | 0.67851858 | | down |
| RP11-90B9.3 | -1.14756354 | 0.07400913 | 0.162717182 | down |
| RP11-52I18.1 | -1.147396951 | 0.678271479 | | down |
| CAMK2A | -1.147351677 | 0.001120061 | 0.006643851 | down |
| RP11-388E23.2 | -1.147302199 | 0.634871924 | | down |
| CTB-113I20.2 | -1.147240307 | 6.66E-06 | 0.000100878 | down |
| RP11-452D21.1 | -1.147051324 | 0.592511771 | | down |
| NA | -1.146995994 | 0.418886235 | | down |
| PLS3-AS1 | -1.146812086 | 0.119304278 | 0.22922413 | down |
| RP11-627K11.3 | -1.146739779 | 0.637990456 | | down |
| RP3-401D24.1 | -1.146739591 | 0.637971107 | | down |
| DCLRE1C | -1.14667197 | 8.98E-06 | 0.000129499 | down |
| RP11-629G13.1 | -1.146590553 | 0.227329253 | 0.359501685 | down |
| MIR3687-2 | -1.146548568 | 0.551782863 | | down |
| 5S_rRNA | -1.146489961 | 0.222056418 | | down |
| BCL2L15 | -1.146246817 | 1.17E-05 | 0.000160914 | down |
| C3orf20 | -1.145976068 | 0.060692237 | 0.140936586 | down |
| NA | -1.145846914 | 0.634245233 | | down |
| RP11-45M22.4 | -1.145809398 | 0.069771282 | 0.155944681 | down |
| 4-Mar | -1.145589022 | 0.010957877 | 0.039361364 | down |
| TMED6 | -1.145580571 | 0.00034441 | 0.002607723 | down |
| ATP8A2 | -1.145443942 | 0.000431302 | 0.003090399 | down |
| RP11-336A10.4 | -1.145431399 | 0.233709911 | | down |
| AC112229.1 | -1.145393626 | 0.179242888 | 0.304439712 | down |
| RP11-383I23.2 | -1.145370057 | 0.064063111 | 0.14653617 | down |
| HEPH | -1.145250001 | 5.00E-05 | 0.00053561 | down |
| AC010745.1 | -1.145093987 | 0.058276689 | 0.136850464 | down |
| PFKL | -1.144952044 | 3.13E-09 | 1.43E-07 | down |
| CTB-102L5.8 | -1.144945838 | 0.234414047 | 0.367172496 | down |
| CEACAMP10 | -1.14477594 | 0.50363043 | 0.63033003 | down |

| | | | | |
|---|---|---|---|---|
| NA | -1.144631663 | 0.331959843 | | down |
| RP11-214O1.3 | -1.144357499 | 0.011184605 | 0.039960789 | down |
| CSTP1 | -1.144346067 | 0.513556397 | | down |
| XXbac-B135H6.15 | -1.144148614 | 2.20E-05 | 0.000270056 | down |
| CNTF | -1.143922972 | 0.044557334 | 0.112390273 | down |
| NUDT8 | -1.143668747 | 0.000211125 | 0.001748358 | down |
| MAS1LP1 | -1.143444369 | 0.18338749 | 0.309133914 | down |
| CTD-2293H3.1 | -1.143032886 | 0.445811618 | | down |
| KRT18P17 | -1.142858683 | 0.039015941 | 0.102009827 | down |
| NA | -1.142833907 | 0.017705349 | 0.056685795 | down |
| FOXD4L5 | -1.14278812 | 0.680049857 | | down |
| RP11-174N3.4 | -1.142511954 | 0.069226694 | | down |
| NA | -1.142358369 | 0.640989521 | | down |
| CEACAM1 | -1.142280999 | 0.004133989 | 0.018724793 | down |
| RPS23P5 | -1.142243428 | 0.441291134 | | down |
| RP11-525J21.1 | -1.142019251 | 0.405739768 | 0.542329959 | down |
| RP11-253M7.6 | -1.142011464 | 0.158789042 | 0.279892107 | down |
| Metazoa_SRP | -1.141969591 | 0.453690432 | | down |
| DRC1 | -1.1419686 | 0.040046335 | 0.104042899 | down |
| WSCD2 | -1.141898371 | 0.010491236 | 0.038053688 | down |
| SLC2A8 | -1.141897514 | 2.43E-08 | 8.35E-07 | down |
| RP11-287D1.2 | -1.141827885 | 0.554995665 | | down |
| FETUB | -1.141722103 | 0.696346488 | | down |
| RP11-438D14.3 | -1.14143704 | 0.445486469 | | down |
| SH3TC2 | -1.141218148 | 0.006276132 | 0.025805406 | down |
| RP5-908D6.1 | -1.141169861 | 0.319514065 | | down |
| SLC44A4 | -1.141140263 | 0.000715758 | 0.004646549 | down |
| EPHB3 | -1.140830363 | 0.002892296 | 0.014095076 | down |
| KRTAP11-1 | -1.140788491 | 0.696242507 | | down |
| RP11-445K13.2 | -1.140691536 | 0.317940414 | | down |
| PRTG | -1.140542605 | 0.012501169 | 0.04346547 | down |
| CDH1 | -1.140541033 | 0.000191166 | 0.001610145 | down |
| CTD-2001E22.1 | -1.140394659 | 0.597744729 | | down |
| RHOU | -1.140131021 | 4.59E-05 | 0.000498182 | down |
| NUTM2A | -1.140048978 | 0.002159701 | 0.011166073 | down |
| NA | -1.139868872 | 0.696840751 | | down |
| RP11-296O14.1 | -1.139868872 | 0.696840751 | | down |
| NA | -1.139868872 | 0.696840751 | | down |
| OR2T6 | -1.139868872 | 0.696840751 | | down |
| RPL21P41 | -1.139868872 | 0.696840751 | | down |
| TMEM212 | -1.139868872 | 0.696840751 | | down |
| RP11-5P22.3 | -1.139868872 | 0.696840751 | | down |
| NA | -1.139868872 | 0.696840751 | | down |
| RP4-655C5.9 | -1.139868872 | 0.696840751 | | down |
| AC004744.3 | -1.139868872 | 0.696840751 | | down |
| RP11-595O22.1 | -1.139868872 | 0.696840751 | | down |
| RP11-21C4.5 | -1.139868872 | 0.696840751 | | down |
| SNORD116 | -1.139868872 | 0.696840751 | | down |
| RP11-473C19.1 | -1.139868872 | 0.696840751 | | down |
| RP11-753D20.3 | -1.139868872 | 0.696840751 | | down |
| RP11-1180F24.3 | -1.139868872 | 0.696840751 | | down |

| | | | | |
|---|---|---|---|---|
| NPM1P42 | -1.139868872 | 0.696840751 | | down |
| RP11-24M17.5 | -1.139868872 | 0.696840751 | | down |
| RP11-15F12.6 | -1.139868872 | 0.696840751 | | down |
| Y_RNA | -1.139868872 | 0.696840751 | | down |
| FAM47C | -1.139868872 | 0.696840751 | | down |
| SNORA11B | -1.139866744 | 0.689366989 | | down |
| ACTRT1 | -1.139865874 | 0.686397276 | | down |
| CTAGE13P | -1.139865714 | 0.685855957 | | down |
| RP11-208N14.4 | -1.139865352 | 0.684634566 | | down |
| RNA5SP100 | -1.139865018 | 0.683517587 | | down |
| CTD-233602.2 | -1.139864903 | 0.68313351 | | down |
| LINC01164 | -1.139864604 | 0.682141807 | | down |
| KRT35 | -1.139864082 | 0.680418481 | | down |
| RP11-150012.4 | -1.139852997 | 0.646853788 | | down |
| RP11-66B24.5 | -1.139852898 | 0.646574761 | | down |
| MKRN4P | -1.139851968 | 0.64398214 | | down |
| Metazoa_SRP | -1.139633601 | 0.558599477 | | down |
| NA | -1.139587051 | 0.276520762 | 0.412807444 | down |
| ADAM22 | -1.139312628 | 7.47E-07 | 1.59E-05 | down |
| MTCO2P22 | -1.139262822 | 0.027982428 | 0.079785904 | down |
| PCP4L1 | -1.139218856 | 0.077128963 | 0.167681277 | down |
| DMRT2 | -1.139168129 | 0.016120101 | 0.052725778 | down |
| HESX1 | -1.138978405 | 0.005619418 | 0.023715381 | down |
| RP11-140L24.3 | -1.138950134 | 0.248334364 | 0.382711343 | down |
| RP11-480010.2 | -1.138841835 | 0.285299497 | 0.422326473 | down |
| BRD7P2 | -1.138834734 | 0.028698934 | 0.081273519 | down |
| RN7SL470P | -1.138630911 | 0.637477491 | | down |
| LINC00961 | -1.138626701 | 0.003262957 | 0.015464946 | down |
| LLNLR-260G6.1 | -1.138382608 | 0.113861296 | 0.22160062 | down |
| ZNF524 | -1.137835188 | 0.000119627 | 0.001100773 | down |
| RP11-728K20.2 | -1.137783552 | 0.188485378 | | down |
| SLCO1B1 | -1.137700586 | 0.339183824 | 0.477696522 | down |
| EVX2 | -1.137475245 | 0.479993837 | | down |
| CTD-3075F15.1 | -1.13733948 | 0.076847199 | 0.167292025 | down |
| RP11-356C4.3 | -1.137324354 | 0.636343246 | | down |
| RP11-268G13.1 | -1.13730806 | 0.638334127 | | down |
| RP11-349H17.2 | -1.136964088 | 0.465779101 | 0.597145487 | down |
| ATP13A5 | -1.1368881 | 0.315267977 | 0.453961711 | down |
| SLC17A3 | -1.136871581 | 0.341678508 | | down |
| ANK3 | -1.136770476 | 1.58E-07 | 4.22E-06 | down |
| MTND5P2 | -1.136630572 | 0.493406116 | | down |
| MAP2K2 | -1.136476538 | 2.34E-07 | 5.91E-06 | down |
| ELMOD1 | -1.136373118 | 0.103008952 | 0.206225662 | down |
| RP3-342P20.2 | -1.136186972 | 0.020111633 | 0.062362875 | down |
| RN7SL541P | -1.136179914 | 0.41256784 | | down |
| UNC13A | -1.136047116 | 0.003233703 | 0.015350508 | down |
| AC009005.2 | -1.135940988 | 0.015005391 | 0.049969184 | down |
| RP11-14N9.2 | -1.135518114 | 0.475426275 | 0.605421397 | down |
| RP11-318K15.2 | -1.13540808 | 0.399662476 | | down |
| C15orf48 | -1.135207987 | 0.000705553 | 0.004593205 | down |
| EXTL1 | -1.134866131 | 0.000612601 | 0.004105506 | down |

| | | | | |
|---|---|---|---|---|
| RP11-114B7.6 | -1.134625972 | 0.698162167 | | down |
| NA | -1.134625972 | 0.698162167 | | down |
| AC009411.2 | -1.134625972 | 0.698162167 | | down |
| MIR217HG | -1.134625972 | 0.698162167 | | down |
| RPL7P41 | -1.134625972 | 0.698162167 | | down |
| RP11-91I8.3 | -1.134625972 | 0.698162167 | | down |
| CTD-2537I9.15 | -1.134625972 | 0.698162167 | | down |
| RN7SL665P | -1.134615082 | 0.688142489 | | down |
| CYP11B1 | -1.134562007 | 0.644501149 | | down |
| RNU7-113P | -1.134561036 | 0.643768991 | | down |
| NA | -1.134559089 | 0.642306647 | | down |
| RP4-781K5.4 | -1.134558109 | 0.64157339 | | down |
| RP11-797A18.5 | -1.134505932 | 0.029184887 | 0.08225429 | down |
| RP11-365P13.6 | -1.134465104 | 0.69813301 | | down |
| TTC39DP | -1.134430565 | 0.559799965 | | down |
| RP11-239H6.2 | -1.134425767 | 0.557139714 | | down |
| RP11-354P11.2 | -1.134418164 | 0.698214762 | | down |
| NA | -1.134359508 | 0.644663662 | | down |
| RP11-430L16.1 | -1.13435938 | 0.644557945 | | down |
| L22NC03-102D1.1 | -1.134353826 | 0.519902965 | | down |
| CTB-58E17.5 | -1.134305708 | 0.015548286 | 0.051350293 | down |
| RSF1-IT1 | -1.134162648 | 0.18315453 | 0.308966574 | down |
| RPL23AP62 | -1.134011501 | 0.698119576 | | down |
| CWH43 | -1.134011211 | 0.065799149 | 0.149338073 | down |
| RP11-713C19.2 | -1.133955506 | 0.600629084 | | down |
| RN7SL569P | -1.133742541 | 0.688443461 | | down |
| TATDN2P3 | -1.133590815 | 0.675347784 | | down |
| RP11-101O21.1 | -1.133565681 | 0.641891277 | | down |
| RP11-15E18.1 | -1.133478246 | 0.007269228 | 0.028774266 | down |
| NA | -1.133147548 | 0.136147398 | 0.251280329 | down |
| C3orf35 | -1.133031025 | 0.000743741 | 0.004788891 | down |
| AC139099.4 | -1.132940081 | 0.10783687 | 0.213499238 | down |
| ACPT | -1.132939528 | 0.015955417 | 0.052326662 | down |
| GLRX3P2 | -1.132923889 | 0.12941279 | 0.242450593 | down |
| CDHR4 | -1.1324112 | 0.046634287 | 0.116146939 | down |
| RP11-48G14.1 | -1.132390349 | 0.690347441 | | down |
| RNU6-930P | -1.132356426 | 0.642721951 | | down |
| RP11-175B9.3 | -1.132331591 | 0.000934389 | 0.005744382 | down |
| RP11-442O1.3 | -1.132234337 | 0.013874525 | 0.047064044 | down |
| RP11-79L9.2 | -1.132232885 | 0.518741942 | | down |
| FRMPD4 | -1.13218795 | 0.20507402 | 0.33488661 | down |
| AC073115.6 | -1.13209655 | 0.196103253 | 0.32461301 | down |
| RP11-478C6.1 | -1.132075098 | 0.102309209 | 0.205328429 | down |
| RP11-134K1.2 | -1.131921189 | 0.050440743 | 0.123082505 | down |
| RP11-260E18.1 | -1.13178363 | 0.067125145 | 0.151609372 | down |
| OR6Y1 | -1.13177138 | 0.565110745 | | down |
| RN7SKP26 | -1.131456157 | 0.645712429 | | down |
| SIGLEC11 | -1.13139373 | 0.01370314 | 0.046572076 | down |
| BNIP3P39 | -1.13119818 | 0.600663398 | | down |
| HLA-DRB6 | -1.131105302 | 0.038849722 | 0.101636073 | down |
| PATJ | -1.131050245 | 7.76E-08 | 2.31E-06 | down |

| | | | | |
|---|---|---|---|---|
| HAAO | -1.13104705 | 4.51E-07 | 1.02E-05 | down |
| RP11-742D12.2 | -1.130885064 | 0.083777903 | 0.178038329 | down |
| CPEB1-AS1 | -1.13069989 | 0.271047983 | | down |
| FOLH1 | -1.130480232 | 0.009612647 | 0.035531785 | down |
| ARHGEF5 | -1.130329967 | 5.81E-07 | 1.28E-05 | down |
| RNA5SP369 | -1.130321331 | 0.548015938 | 0.668805374 | down |
| SPTBN4 | -1.13013758 | 0.000129161 | 0.001169184 | down |
| MYH13 | -1.130008937 | 0.011723471 | 0.041457151 | down |
| FAM157A | -1.129966995 | 0.018730211 | 0.059097928 | down |
| AVIL | -1.129830935 | 4.96E-06 | 7.87E-05 | down |
| 11-Mar | -1.129742443 | 0.556945699 | | down |
| RP11-520D19.2 | -1.129625883 | 0.420047181 | | down |
| ATP5G2P4 | -1.129527904 | 0.110472105 | 0.217057348 | down |
| RP11-986G18.2 | -1.129263674 | 0.011380993 | 0.040537114 | down |
| CNGA3 | -1.129262238 | 0.08778605 | 0.184191918 | down |
| NA | -1.129195903 | 0.638656792 | | down |
| MROH2A | -1.129180721 | 0.150673708 | 0.269856387 | down |
| RP11-439C15.5 | -1.12915323 | 0.078463863 | 0.169790283 | down |
| RP11-632F7.3 | -1.129074822 | 0.526903348 | | down |
| WIPF3 | -1.128616476 | 0.000345575 | 0.002614571 | down |
| RP11-466I1.1 | -1.128576548 | 0.520885527 | 0.645179946 | down |
| Y_RNA | -1.128183363 | 0.63749985 | | down |
| RP11-10G12.2 | -1.127997904 | 0.699646596 | | down |
| C17orf99 | -1.127904692 | 0.041509302 | 0.106864222 | down |
| HNRNPA1P41 | -1.127895901 | 0.233991062 | 0.366720264 | down |
| SCNN1A | -1.127724193 | 0.001573856 | 0.00866977 | down |
| RP11-534L20.5 | -1.127502409 | 0.046184276 | 0.115322506 | down |
| APOF | -1.127490216 | 0.074347011 | 0.163256951 | down |
| RP11-478J18.2 | -1.126956019 | 0.116877766 | 0.225832889 | down |
| RP11-730A19.5 | -1.126923968 | 0.04014038 | 0.104215139 | down |
| RP11-663N22.1 | -1.126869569 | 0.60047372 | | down |
| RP11-466A19.1 | -1.12675726 | 0.032260387 | 0.088465778 | down |
| RP11-180P8.5 | -1.126745109 | 0.452234814 | 0.585076015 | down |
| AC079807.4 | -1.12660431 | 0.015391666 | 0.05094494 | down |
| DET1 | -1.126499894 | 0.000257731 | 0.002061571 | down |
| NA | -1.126291541 | 0.001351259 | 0.007685956 | down |
| RP5-859D4.3 | -1.125937487 | 0.643820734 | | down |
| TEX12 | -1.125933193 | 0.082619186 | 0.176418679 | down |
| RP11-511H23.2 | -1.125864041 | 0.034681744 | 0.093434211 | down |
| RP11-203F10.1 | -1.125767406 | 0.640347521 | | down |
| NA | -1.125648899 | 0.055860854 | 0.13264966 | down |
| CAP2P1 | -1.125632432 | 0.178521208 | 0.303588112 | down |
| RP3-422G23.4 | -1.125586253 | 0.114275992 | 0.222191814 | down |
| RP11-506H20.1 | -1.12558063 | 0.176916432 | 0.301841572 | down |
| ANP32BP1 | -1.125490345 | 0.0182126 | 0.057890326 | down |
| LINC-ROR | -1.125248356 | 0.490125131 | | down |
| CCDC105 | -1.124966106 | 0.640473683 | | down |
| RP11-62H7.3 | -1.124819197 | 0.323115983 | | down |
| RP11-33B1.3 | -1.12470621 | 0.25417513 | 0.389245421 | down |
| RP11-36N20.1 | -1.124597261 | 0.600609027 | | down |
| bac-BPGBPG55C20 | -1.124558649 | 0.256815716 | | down |

| | | | | |
|---|---|---|---|---|
| RP11-57H12.5 | -1.12452486 | 0.070191743 | 0.156663006 | down |
| Z69890.1 | -1.124503912 | 0.334956312 | | down |
| TXNRD2 | -1.124498597 | 3.75E-06 | 6.25E-05 | down |
| NA | -1.124387182 | 0.172377276 | 0.296687289 | down |
| AC087501.1 | -1.124323543 | 0.422622664 | | down |
| U91319.1 | -1.124270922 | 0.203426558 | 0.332883851 | down |
| LCTL | -1.124077525 | 0.080121636 | 0.172447293 | down |
| ASPSCR1 | -1.123939051 | 7.98E-06 | 0.000116854 | down |
| TMEM92 | -1.123887657 | 0.005817497 | 0.024363099 | down |
| Y_RNA | -1.123617417 | 0.700911694 | | down |
| PRC1-AS1 | -1.12330441 | 0.01140358 | 0.040593501 | down |
| LINC01440 | -1.123170807 | 0.681981221 | | down |
| FTH1P15 | -1.122996832 | 0.222079522 | 0.353904267 | down |
| AC003986.6 | -1.12293969 | 0.418419472 | 0.554521157 | down |
| STPG3 | -1.122436921 | 0.016569571 | 0.053806332 | down |
| EPHA10 | -1.122307874 | 0.000281051 | 0.002213921 | down |
| DEUP1 | -1.122036292 | 0.126803174 | 0.238982317 | down |
| SERF1B | -1.122008303 | 0.01064631 | 0.038469135 | down |
| C9orf163 | -1.121910319 | 0.02607042 | 0.075633421 | down |
| LRRC37A7P | -1.121806283 | 0.099427083 | 0.201208977 | down |
| RP11-224P11.1 | -1.12177472 | 0.492399769 | 0.620591436 | down |
| RP11-707P17.2 | -1.121699006 | 0.073709239 | 0.162342964 | down |
| RP11-60I3.4 | -1.12166146 | 0.547134275 | | down |
| LINC00240 | -1.121650797 | 0.006056559 | 0.025122484 | down |
| RP11-157B13.7 | -1.121591051 | 0.640521051 | | down |
| RP11-295D4.5 | -1.121458118 | 0.031820924 | 0.087564373 | down |
| CNN2P1 | -1.12142771 | 0.03643371 | 0.096893383 | down |
| PTPRD | -1.121357085 | 0.000168521 | 0.001451975 | down |
| RP11-394B2.5 | -1.121339618 | 0.026990694 | 0.077628605 | down |
| MIR3161 | -1.120917733 | 0.566708408 | | down |
| NECTIN3 | -1.120814472 | 1.72E-06 | 3.25E-05 | down |
| DRD4 | -1.120648812 | 0.000465654 | 0.003287335 | down |
| HCG9 | -1.120334635 | 0.049829441 | 0.122001118 | down |
| RALGPS1 | -1.120297958 | 2.05E-11 | 1.67E-09 | down |
| GRIA3 | -1.120243049 | 0.002398908 | 0.01216624 | down |
| AP1M2 | -1.120090834 | 0.000160837 | 0.001396183 | down |
| RP11-68I3.5 | -1.120006711 | 0.200913866 | | down |
| CTAGE6 | -1.119812945 | 0.491108249 | | down |
| RP11-569G13.2 | -1.119620034 | 0.162704658 | 0.284782916 | down |
| FDX1P2 | -1.119584383 | 0.701934942 | | down |
| PKD2L1 | -1.119545077 | 0.00310732 | 0.014894116 | down |
| RBM5-AS1 | -1.119175861 | 0.109086536 | 0.215207556 | down |
| ATP5I | -1.119159451 | 0.00167951 | 0.009126279 | down |
| PCNX2 | -1.119127596 | 2.05E-05 | 0.000256551 | down |
| KCNN2 | -1.118963388 | 0.045377531 | 0.113856903 | down |
| ABLIM2 | -1.118900024 | 4.28E-07 | 9.81E-06 | down |
| RP11-820I16.4 | -1.118785199 | 0.000565234 | 0.003841189 | down |
| RP11-292E2.1 | -1.118774748 | 0.317754781 | | down |
| TPGS1 | -1.118584127 | 0.00052555 | 0.003622307 | down |
| Y_RNA | -1.118500716 | 0.493623128 | | down |
| CTD-3023L14.2 | -1.118271027 | 0.469502326 | 0.600321504 | down |

| | | | | |
|---|---|---|---|---|
| AHSP | -1.118243049 | 0.14510908 | 0.262740746 | down |
| INTS6-AS1 | -1.118090746 | 0.000454789 | 0.003227131 | down |
| RP11-510N19.5 | -1.118068362 | 0.053382383 | 0.12818257 | down |
| COLEC10 | -1.117759809 | 0.0325464 | 0.089079352 | down |
| LINC01108 | -1.117633218 | 0.394554411 | | down |
| NKX2-8 | -1.117602011 | 0.607149744 | | down |
| LRP1-AS | -1.117564834 | 0.244157047 | | down |
| RP11-543D5.1 | -1.117336467 | 0.175598573 | 0.300215304 | down |
| NA | -1.117092611 | 0.036981417 | 0.097917483 | down |
| AC092535.3 | -1.116951117 | 0.128239801 | 0.240973758 | down |
| AL357140.1 | -1.11683545 | 0.279941705 | | down |
| KCNB1 | -1.116819253 | 0.014931291 | 0.049765111 | down |
| NA | -1.116788657 | 0.702341071 | | down |
| EMX2OS | -1.116764917 | 0.188064845 | 0.314888336 | down |
| NPSR1-AS1 | -1.116533558 | 0.270465811 | 0.40646058 | down |
| NA | -1.116310361 | 0.128864197 | 0.241823606 | down |
| COL17A1 | -1.116293015 | 0.001841073 | 0.00982647 | down |
| RP11-589M4.3 | -1.116224466 | 0.284266827 | 0.42112981 | down |
| RNU2-5P | -1.115836144 | 0.212637636 | 0.343466943 | down |
| RAB3IP | -1.115788996 | 6.25E-10 | 3.41E-08 | down |
| RN7SL471P | -1.115754956 | 0.678599825 | | down |
| THRB-IT1 | -1.11569202 | 0.144512804 | 0.262055932 | down |
| CLYBL | -1.115222075 | 1.60E-08 | 5.99E-07 | down |
| RP11-384P7.7 | -1.115129094 | 0.030560444 | 0.084943841 | down |
| RNU1-120P | -1.115127274 | 0.227385373 | | down |
| SLC5A2 | -1.11500149 | 0.019120437 | 0.060032857 | down |
| RP11-214K3.24 | -1.11493924 | 0.144686999 | 0.262245186 | down |
| KRT39 | -1.114665936 | 0.176448457 | | down |
| RP11-405K6.1 | -1.114586652 | 0.387742234 | 0.525288708 | down |
| TCHH | -1.114125995 | 0.028166168 | 0.080180438 | down |
| DCST2 | -1.113788229 | 0.00023042 | 0.001879648 | down |
| RP11-278A23.2 | -1.113714515 | 0.033138193 | 0.090230549 | down |
| RP11-13N13.6 | -1.11365615 | 0.126583455 | | down |
| NA | -1.113219318 | 0.183506416 | 0.309230286 | down |
| RPL13AP20 | -1.113011039 | 0.000228489 | 0.001867953 | down |
| SRGAP1 | -1.113004264 | 8.69E-06 | 0.000125792 | down |
| NA | -1.112994184 | 0.094795732 | 0.194618215 | down |
| RP4-666F24.3 | -1.112985557 | 0.067730327 | 0.152574787 | down |
| RP11-382N13.3 | -1.11298284 | 0.686490688 | | down |
| LLNLR-249E10.1 | -1.112801934 | 0.292640419 | | down |
| PLEKHG4B | -1.112267713 | 0.00182176 | 0.009742634 | down |
| SEMA6A | -1.11220614 | 3.45E-07 | 8.15E-06 | down |
| LINC01268 | -1.112053781 | 0.000439134 | 0.003134557 | down |
| MAP1LC3B2 | -1.112049464 | 0.001858213 | 0.009905118 | down |
| OR7M1P | -1.111966893 | 0.26292648 | | down |
| RP11-488C13.1 | -1.1118887 | 0.003293083 | 0.015568433 | down |
| AC100830.5 | -1.111662416 | 0.326752783 | | down |
| NA | -1.111533021 | 0.703954113 | | down |
| CTD-2245E15.3 | -1.11145099 | 0.046781135 | 0.116425814 | down |
| RP11-972P1.11 | -1.111450111 | 0.050088489 | 0.122445775 | down |
| FBXW5 | -1.111043248 | 2.11E-07 | 5.37E-06 | down |

| | | | | |
|---|---|---|---|---|
| PC | -1.110913251 | 2.43E-06 | 4.34E-05 | down |
| RP11-390P2.4 | -1.110904609 | 0.000335521 | 0.002554589 | down |
| SH3D21 | -1.110875306 | 2.87E-06 | 5.02E-05 | down |
| CTD-2021H9.3 | -1.110750745 | 0.061388197 | 0.142211965 | down |
| SUMO1P4 | -1.110692314 | 0.495497161 | 0.623344641 | down |
| CELF3 | -1.110546185 | 0.012295756 | 0.042955079 | down |
| SQRDL | -1.109984651 | 0.000294079 | 0.002293695 | down |
| LINC00552 | -1.109929044 | 0.157810413 | | down |
| OTOA | -1.109796877 | 0.007027111 | 0.028089943 | down |
| NA | -1.109601079 | 0.198416958 | 0.327181069 | down |
| RP11-479A21.1 | -1.1095069 | 0.129789796 | 0.242893026 | down |
| NA | -1.109456116 | 0.028116487 | 0.080076948 | down |
| TFR2 | -1.109234472 | 0.001069279 | 0.006393296 | down |
| NA | -1.10921491 | 0.266657071 | | down |
| MIR33B | -1.109116125 | 0.285682942 | | down |
| MYH7B | -1.10866201 | 0.000139561 | 0.001246426 | down |
| RP11-66602.2 | -1.108595183 | 0.49741512 | | down |
| RNU6-529P | -1.108488885 | 0.051133958 | 0.124362701 | down |
| HIST1H3A | -1.108445198 | 0.039313647 | 0.102582874 | down |
| RP11-548L20.1 | -1.10829521 | 0.680842785 | | down |
| RP11-95P2.3 | -1.108215636 | 0.036742805 | 0.097526356 | down |
| RP11-478B9.2 | -1.108020901 | 0.684057309 | | down |
| FAM109A | -1.107740308 | 5.13E-08 | 1.61E-06 | down |
| RP11-138B4.1 | -1.107473162 | 0.121825352 | 0.232521496 | down |
| Y_RNA | -1.107359013 | 0.705076744 | | down |
| EIF4EP2 | -1.107325484 | 0.089351746 | 0.186629857 | down |
| TRIM67 | -1.10730052 | 0.027485138 | 0.078689105 | down |
| IGSF3 | -1.106561986 | 4.52E-05 | 0.000492512 | down |
| SCOC-AS1 | -1.106124731 | 0.001633661 | 0.008933691 | down |
| DSG2 | -1.106057122 | 0.00122598 | 0.007131174 | down |
| Y_RNA | -1.106030223 | 0.607702582 | | down |
| RP11-123K19.1 | -1.105972208 | 0.447943198 | | down |
| BAIAP3 | -1.105963475 | 4.72E-08 | 1.50E-06 | down |
| RP11-863P13.2 | -1.105431025 | 0.574331508 | | down |
| RNF17 | -1.105368407 | 0.242312497 | 0.376238334 | down |
| AE000662.92 | -1.105317925 | 0.318973924 | | down |
| RP11-26H16.4 | -1.10507328 | 0.222460946 | 0.354358724 | down |
| PLA2G4F | -1.104723302 | 0.001837501 | 0.009812636 | down |
| RNA5SP203 | -1.104539293 | 0.39293173 | | down |
| RP11-81K2.1 | -1.104332019 | 0.061506899 | 0.142410087 | down |
| AP000692.10 | -1.104280312 | 0.014177939 | 0.047834026 | down |
| OGDHL | -1.104131452 | 0.000208546 | 0.001730335 | down |
| RP11-51J9.5 | -1.104115083 | 0.000594935 | 0.004005931 | down |
| NA | -1.104101697 | 0.377992037 | | down |
| CTC-232P5.3 | -1.10400753 | 0.20797434 | 0.33818906 | down |
| TMEM52B | -1.103895414 | 0.03868862 | 0.101296699 | down |
| RP11-115L11.1 | -1.103732445 | 0.141593859 | 0.258321952 | down |
| AC011523.2 | -1.103642165 | 0.022683375 | 0.068136304 | down |
| MPV17L | -1.103622396 | 3.29E-06 | 5.63E-05 | down |
| RP11-235E17.3 | -1.103580829 | 0.224436174 | | down |
| NA | -1.103560384 | 0.000469226 | 0.003307703 | down |

| | | | | |
|---|---|---|---|---|
| LMAN1L | -1.103542779 | 0.649026011 | | down |
| COX4I1P1 | -1.103348904 | 0.70591754 | | down |
| MRPS31P2 | -1.10321196 | 0.150885753 | 0.270139537 | down |
| AC003973.4 | -1.103117703 | 0.605405042 | | down |
| RP11-66B24.2 | -1.102931924 | 0.091132589 | 0.18916587 | down |
| 5S_rRNA | -1.10292375 | 0.337135218 | | down |
| TENM1 | -1.102625475 | 0.005573428 | 0.023576207 | down |
| LINC01222 | -1.102594665 | 0.337290776 | | down |
| AC013268.3 | -1.10258737 | 0.401301475 | | down |
| SDR42E1 | -1.10251291 | 1.60E-06 | 3.07E-05 | down |
| ZNF883 | -1.102174664 | 0.118131498 | 0.227552909 | down |
| GS1-124K5.7 | -1.101931893 | 0.643489883 | | down |
| RP4-798A10.2 | -1.101794487 | 0.005490424 | 0.023275619 | down |
| RP11-521B24.3 | -1.101597705 | 0.006167662 | 0.025484972 | down |
| MRPL27 | -1.101506276 | 3.21E-05 | 0.000369855 | down |
| RP11-318C24.2 | -1.10138067 | 0.015604733 | 0.051491335 | down |
| GEMIN8P4 | -1.100991692 | 0.000359805 | 0.002692397 | down |
| CTA-221G9.10 | -1.100967204 | 0.498739367 | | down |
| RETSAT | -1.100962812 | 9.69E-05 | 0.000920349 | down |
| AC010096.2 | -1.100366509 | 0.706761929 | | down |
| RNU6-79P | -1.100284928 | 0.706596518 | | down |
| ADRB1 | -1.100174262 | 0.000804673 | 0.005104646 | down |
| PYCARD | -1.099977898 | 1.58E-05 | 0.000206858 | down |
| RPL29P2 | -1.09997385 | 0.57193002 | | down |
| INHA | -1.099877909 | 0.021448116 | 0.065360299 | down |
| BCL2L14 | -1.099751315 | 2.18E-07 | 5.52E-06 | down |
| RP11-304L19.3 | -1.099677678 | 0.002716389 | 0.0134251 | down |
| RANP6 | -1.099663377 | 0.170094769 | 0.293882756 | down |
| ALOX12P1 | -1.099621517 | 0.540007784 | | down |
| RP11-81K2.2 | -1.099474539 | 0.222578928 | 0.354474493 | down |
| CASZ1 | -1.099462469 | 6.12E-07 | 1.34E-05 | down |
| GLI4 | -1.099449501 | 0.000805571 | 0.005109265 | down |
| Y_RNA | -1.099386354 | 0.416392998 | 0.552591158 | down |
| NA | -1.09935324 | 0.084121366 | 0.178582663 | down |
| DMRTA2 | -1.09933011 | 0.216337833 | 0.347482162 | down |
| SIRPAP1 | -1.099311414 | 0.258380789 | 0.393471156 | down |
| RN7SL391P | -1.099190152 | 0.707117208 | | down |
| NA | -1.099027721 | 0.120885184 | 0.231329584 | down |
| RP11-540O11.1 | -1.098969802 | 0.005256826 | 0.022546194 | down |
| RP11-457M11.7 | -1.098810684 | 0.322033211 | | down |
| STXBP5-AS1 | -1.098347219 | 1.14E-05 | 0.000156996 | down |
| SYP | -1.098327985 | 0.000346987 | 0.002622613 | down |
| VN1R20P | -1.09811526 | 0.353889657 | | down |
| RP5-984P4.6 | -1.097877605 | 0.149204423 | 0.26797571 | down |
| CRK | -1.097582881 | 4.51E-06 | 7.30E-05 | down |
| IL17RC | -1.097462602 | 6.90E-06 | 0.000103937 | down |
| MYO16 | -1.097439027 | 0.010459791 | 0.037971719 | down |
| GRTP1 | -1.097349535 | 5.46E-06 | 8.51E-05 | down |
| RP11-20I20.2 | -1.097310284 | 0.169108245 | | down |
| NA | -1.097299111 | 0.377417451 | 0.515625933 | down |
| CCHCR1 | -1.097216492 | 3.00E-07 | 7.27E-06 | down |

| | | | | |
|---|---|---|---|---|
| RP11-315A16.1 | -1.097169326 | 0.024721479 | 0.072681487 | down |
| RP11-135A24.2 | -1.097139363 | 0.389147087 | | down |
| AP3B2 | -1.096903467 | 0.000177976 | 0.001520363 | down |
| ZP3 | -1.096627372 | 0.003590423 | 0.016708594 | down |
| RNF208 | -1.096431552 | 8.58E-05 | 0.000831921 | down |
| RP11-129M6.1 | -1.096418777 | 0.212025346 | 0.342754345 | down |
| CORO2A | -1.095870099 | 7.82E-07 | 1.65E-05 | down |
| RAB6C | -1.095754145 | 0.070694636 | 0.157481252 | down |
| YWHAQP5 | -1.095689871 | 0.421177852 | | down |
| NXPH1 | -1.095641487 | 0.610844024 | | down |
| RP4-580N22.2 | -1.095575363 | 0.40071517 | | down |
| RP11-185E8.1 | -1.095342636 | 0.129717715 | 0.242818632 | down |
| NA | -1.095325561 | 0.242410636 | 0.376302067 | down |
| NUDT14 | -1.095222669 | 6.17E-05 | 0.000634778 | down |
| HFM1 | -1.095195569 | 0.01963149 | 0.061164418 | down |
| TMEM144 | -1.095171678 | 1.07E-05 | 0.000148824 | down |
| ROMO1 | -1.095094952 | 0.000190756 | 0.001607492 | down |
| FGF22 | -1.094987739 | 0.012234649 | 0.042796753 | down |
| ZNF784 | -1.094984845 | 3.00E-08 | 1.00E-06 | down |
| SEPT14P12 | -1.094721395 | 0.33774636 | | down |
| RP11-20L24.1 | -1.094333207 | 0.433901294 | 0.568803833 | down |
| AC004381.6 | -1.094174893 | 4.26E-06 | 6.96E-05 | down |
| LDHAL6FP | -1.094145384 | 0.499040079 | | down |
| RP11-124N2.1 | -1.094112917 | 0.006703999 | 0.027126591 | down |
| AP000476.1 | -1.093520856 | 0.040421492 | 0.10475535 | down |
| RP3-399J4.2 | -1.093514371 | 0.203230793 | | down |
| COX7CP1 | -1.093319696 | 0.003214066 | 0.015281724 | down |
| UBE2SP2 | -1.093208458 | 0.052037216 | 0.125756606 | down |
| ISM2 | -1.093195788 | 0.296674511 | 0.434074657 | down |
| NA | -1.093194647 | 0.56254222 | 0.681573254 | down |
| CEACAMP11 | -1.092600298 | 0.708772552 | | down |
| PAH | -1.092302266 | 0.272792394 | 0.409001846 | down |
| RDH13 | -1.092228892 | 4.94E-07 | 1.11E-05 | down |
| ACSBG1 | -1.092156051 | 0.022589635 | 0.067912183 | down |
| KRT33A | -1.092134717 | 0.647228959 | | down |
| RP11-810P8.1 | -1.092095504 | 0.652386322 | | down |
| KIAA1522 | -1.092061187 | 8.07E-06 | 0.00011811 | down |
| PDLIM1P1 | -1.091878782 | 0.574031285 | | down |
| DGKB | -1.091314771 | 0.055932733 | 0.132778369 | down |
| LINC01518 | -1.091076184 | 0.491655342 | | down |
| RP11-348H3.2 | -1.090918449 | 0.184381263 | 0.310391139 | down |
| RPS7P4 | -1.090913777 | 0.036828259 | 0.097641035 | down |
| RP11-646I6.3 | -1.090551298 | 0.133499467 | 0.247660265 | down |
| NAALAD2 | -1.09044481 | 0.006766074 | 0.027300556 | down |
| LRRC56 | -1.090304031 | 6.97E-07 | 1.50E-05 | down |
| RP11-448P19.1 | -1.090123837 | 0.511886331 | | down |
| NA | -1.090087678 | 0.542817588 | | down |
| RP11-795F19.1 | -1.090068909 | 0.028771961 | 0.081411265 | down |
| KRT73 | -1.090018267 | 0.114672597 | 0.222746719 | down |
| NA | -1.089985023 | 0.007712499 | 0.030131901 | down |
| KIAA1683 | -1.089810773 | 9.92E-06 | 0.000140249 | down |

| | | | | |
|---|---|---|---|---|
| FAM27D1 | -1.089764527 | 0.037428604 | 0.098866272 | down |
| HMGCL | -1.089671583 | 2.10E-07 | 5.36E-06 | down |
| CTD-3073N11.5 | -1.089459415 | 0.539119236 | | down |
| RP11-417L19.4 | -1.089331153 | 0.050974493 | 0.124026375 | down |
| SPDYE6 | -1.089328134 | 0.001642217 | 0.008970688 | down |
| NA | -1.089132598 | 0.001298819 | 0.007446894 | down |
| CTB-54I1.1 | -1.088960078 | 0.459110304 | | down |
| SIRT6 | -1.088939214 | 1.48E-05 | 0.000196116 | down |
| GAS8-AS1 | -1.088815746 | 0.182867917 | 0.308643738 | down |
| RP11-556I14.1 | -1.088587839 | 0.404611113 | 0.541080258 | down |
| TRIM63 | -1.088409947 | 0.025187151 | 0.073704369 | down |
| AC008073.9 | -1.088120815 | 0.416934031 | | down |
| POU1F1 | -1.088083805 | 0.224253508 | 0.356513257 | down |
| AC092641.2 | -1.087826111 | 0.326356428 | | down |
| RP11-367J7.3 | -1.08748169 | 0.372588016 | | down |
| RP11-429J17.7 | -1.087217112 | 0.231101644 | | down |
| RP11-114H24.4 | -1.087187463 | 0.338244511 | | down |
| ADGB | -1.087031923 | 0.189510828 | 0.31668757 | down |
| RP4-564F22.5 | -1.086926843 | 0.013158793 | 0.045135607 | down |
| RP11-407B7.3 | -1.086788917 | 0.533133233 | 0.656103504 | down |
| CAV3 | -1.086542951 | 0.096238318 | 0.196652657 | down |
| RP3-395M20.9 | -1.086390253 | 9.78E-05 | 0.00092777 | down |
| MSMB | -1.086280483 | 0.302923115 | | down |
| LINC01545 | -1.086259064 | 0.156049265 | 0.276389884 | down |
| RPS20P31 | -1.086222856 | 0.578772703 | | down |
| RP11-151G14.2 | -1.086169465 | 0.098570686 | 0.199976884 | down |
| RN7SL270P | -1.086069436 | 0.406068193 | | down |
| RP4-539M6.20 | -1.0860439 | 0.104548816 | | down |
| RP4-781K5.6 | -1.086042268 | 0.16279599 | 0.284846547 | down |
| RP11-50102.5 | -1.086006073 | 0.545629197 | | down |
| RP5-1074L1.1 | -1.085962752 | 0.001099912 | 0.006548902 | down |
| SLC25A18 | -1.085962262 | 0.000383632 | 0.002827737 | down |
| NA | -1.085723806 | 0.004167588 | 0.018837619 | down |
| RP11-175K6.2 | -1.085671769 | 0.002935723 | 0.014258142 | down |
| TRAV18 | -1.085492324 | 0.178405348 | 0.303476971 | down |
| RP11-28601.1 | -1.085032602 | 0.577573229 | | down |
| WDR66 | -1.084944183 | 0.007059039 | 0.028157431 | down |
| RP11-527H14.4 | -1.084867863 | 0.710743284 | | down |
| RNU6-137P | -1.084835087 | 0.106089231 | 0.21078929 | down |
| WASH7P | -1.084825047 | 1.67E-05 | 0.000216119 | down |
| RP11-65J21.4 | -1.084799301 | 0.448323238 | | down |
| RP5-1077B9.5 | -1.084736824 | 0.596707653 | 0.710731237 | down |
| RPL7P52 | -1.084697982 | 0.334640492 | 0.472871938 | down |
| RP11-448N3.1 | -1.084614714 | 0.621716837 | | down |
| NA | -1.084613344 | 0.702695726 | | down |
| NA | -1.08461318 | 0.709307127 | | down |
| RNU6-1199P | -1.084613139 | 0.710860604 | | down |
| MIR551A | -1.084613139 | 0.710860604 | | down |
| NA | -1.084613139 | 0.710860604 | | down |
| RP5-1044H5.1 | -1.084613139 | 0.710860604 | | down |
| HSD3BP3 | -1.084613139 | 0.710860604 | | down |

| | | | |
|---|---|---|---|
| CH17-13I23.2 | -1.084613139 | 0.710860604 | down |
| TCHHL1 | -1.084613139 | 0.710860604 | down |
| SUMO1P2 | -1.084613139 | 0.710860604 | down |
| MORF4L1P7 | -1.084613139 | 0.710860604 | down |
| RP11-505O17.1 | -1.084613139 | 0.710860604 | down |
| GAPDHP24 | -1.084613139 | 0.710860604 | down |
| RP11-66A2.1 | -1.084613139 | 0.710860604 | down |
| MTND5P18 | -1.084613139 | 0.710860604 | down |
| AC018463.5 | -1.084613139 | 0.710860604 | down |
| AC092635.1 | -1.084613139 | 0.710860604 | down |
| AC018892.8 | -1.084613139 | 0.710860604 | down |
| NA | -1.084613139 | 0.710860604 | down |
| AC114813.1 | -1.084613139 | 0.710860604 | down |
| GPR148 | -1.084613139 | 0.710860604 | down |
| AC011901.2 | -1.084613139 | 0.710860604 | down |
| RPS6P2 | -1.084613139 | 0.710860604 | down |
| KRT18P19 | -1.084613139 | 0.710860604 | down |
| AC006196.1 | -1.084613139 | 0.710860604 | down |
| AC009965.2 | -1.084613139 | 0.710860604 | down |
| AC010731.6 | -1.084613139 | 0.710860604 | down |
| Y_RNA | -1.084613139 | 0.710860604 | down |
| FABP5P14 | -1.084613139 | 0.710860604 | down |
| AC097468.7 | -1.084613139 | 0.710860604 | down |
| NA | -1.084613139 | 0.710860604 | down |
| RP11-354H21.2 | -1.084613139 | 0.710860604 | down |
| RN7SL397P | -1.084613139 | 0.710860604 | down |
| RNU7-82P | -1.084613139 | 0.710860604 | down |
| MIR4798 | -1.084613139 | 0.710860604 | down |
| RP11-395F4.1 | -1.084613139 | 0.710860604 | down |
| RP11-231C18.2 | -1.084613139 | 0.710860604 | down |
| RP11-1J11.1 | -1.084613139 | 0.710860604 | down |
| RP11-398J16.1 | -1.084613139 | 0.710860604 | down |
| ZACNP1 | -1.084613139 | 0.710860604 | down |
| NA | -1.084613139 | 0.710860604 | down |
| RP11-6C14.1 | -1.084613139 | 0.710860604 | down |
| CTD-2046I8.1 | -1.084613139 | 0.710860604 | down |
| LINC00603 | -1.084613139 | 0.710860604 | down |
| CTD-2160D9.1 | -1.084613139 | 0.710860604 | down |
| CTB-3M24.3 | -1.084613139 | 0.710860604 | down |
| CH17-140K24.1 | -1.084613139 | 0.710860604 | down |
| HIST1H2AA | -1.084613139 | 0.710860604 | down |
| RN7SL26P | -1.084613139 | 0.710860604 | down |
| PGK2 | -1.084613139 | 0.710860604 | down |
| GSTA11P | -1.084613139 | 0.710860604 | down |
| NA | -1.084613139 | 0.710860604 | down |
| RN7SL415P | -1.084613139 | 0.710860604 | down |
| RP3-495O10.1 | -1.084613139 | 0.710860604 | down |
| RNA5SP230 | -1.084613139 | 0.710860604 | down |
| NA | -1.084613139 | 0.710860604 | down |
| BSNDP4 | -1.084613139 | 0.710860604 | down |
| RNA5SP231 | -1.084613139 | 0.710860604 | down |

| | | | |
|---|---|---|---|
| NA | -1.084613139 | 0.710860604 | down |
| RP11-645N11.3 | -1.084613139 | 0.710860604 | down |
| MIR182 | -1.084613139 | 0.710860604 | down |
| NA | -1.084613139 | 0.710860604 | down |
| RP11-219J21.2 | -1.084613139 | 0.710860604 | down |
| RPL10AP3 | -1.084613139 | 0.710860604 | down |
| RP11-589C21.1 | -1.084613139 | 0.710860604 | down |
| PDCL3P1 | -1.084613139 | 0.710860604 | down |
| RP11-449M6.3 | -1.084613139 | 0.710860604 | down |
| RP11-326E22.1 | -1.084613139 | 0.710860604 | down |
| CTD-2588J1.1 | -1.084613139 | 0.710860604 | down |
| RP11-398H6.1 | -1.084613139 | 0.710860604 | down |
| RNU6-765P | -1.084613139 | 0.710860604 | down |
| RP11-15B24.4 | -1.084613139 | 0.710860604 | down |
| RP11-65B23.3 | -1.084613139 | 0.710860604 | down |
| RP11-402G3.6 | -1.084613139 | 0.710860604 | down |
| RN7SL159P | -1.084613139 | 0.710860604 | down |
| GOLGA2P6 | -1.084613139 | 0.710860604 | down |
| Y_RNA | -1.084613139 | 0.710860604 | down |
| OR51T1 | -1.084613139 | 0.710860604 | down |
| OR52X1P | -1.084613139 | 0.710860604 | down |
| CTC-497E21.3 | -1.084613139 | 0.710860604 | down |
| RP11-277K23.1 | -1.084613139 | 0.710860604 | down |
| OR4A43P | -1.084613139 | 0.710860604 | down |
| FAM8A2P | -1.084613139 | 0.710860604 | down |
| RP11-573M3.5 | -1.084613139 | 0.710860604 | down |
| MTATP6P15 | -1.084613139 | 0.710860604 | down |
| NA | -1.084613139 | 0.710860604 | down |
| RP11-434C1.5 | -1.084613139 | 0.710860604 | down |
| KNOP1P1 | -1.084613139 | 0.710860604 | down |
| RP11-843B15.3 | -1.084613139 | 0.710860604 | down |
| RP11-361A23.2 | -1.084613139 | 0.710860604 | down |
| RP11-541G9.1 | -1.084613139 | 0.710860604 | down |
| RP11-554D14.1 | -1.084613139 | 0.710860604 | down |
| ZNF962P | -1.084613139 | 0.710860604 | down |
| RN7SL597P | -1.084613139 | 0.710860604 | down |
| NA | -1.084613139 | 0.710860604 | down |
| RP11-65D24.2 | -1.084613139 | 0.710860604 | down |
| RP11-30G8.1 | -1.084613139 | 0.710860604 | down |
| RN7SKP95 | -1.084613139 | 0.710860604 | down |
| RP11-299H22.6 | -1.084613139 | 0.710860604 | down |
| NA | -1.084613139 | 0.710860604 | down |
| LA16c-407A10.3 | -1.084613139 | 0.710860604 | down |
| NA | -1.084613139 | 0.710860604 | down |
| RP11-463O9.2 | -1.084613139 | 0.710860604 | down |
| RP11-28B23.1 | -1.084613139 | 0.710860604 | down |
| RP11-501C14.5 | -1.084613139 | 0.710860604 | down |
| CALM2P1 | -1.084613139 | 0.710860604 | down |
| RP11-78A19.4 | -1.084613139 | 0.710860604 | down |
| RN7SL97P | -1.084613139 | 0.710860604 | down |
| RP11-736G13.1 | -1.084613139 | 0.710860604 | down |

| | | | | |
|---|---|---|---|---|
| NA | -1.084613139 | 0.710860604 | | down |
| OR7D1P | -1.084613139 | 0.710860604 | | down |
| ZNF725P | -1.084613139 | 0.710860604 | | down |
| RNA5-8SP4 | -1.084613139 | 0.710860604 | | down |
| CTC-400I9.3 | -1.084613139 | 0.710860604 | | down |
| RN7SL525P | -1.084613139 | 0.710860604 | | down |
| RNU1-94P | -1.084613139 | 0.710860604 | | down |
| BPIFB6 | -1.084613139 | 0.710860604 | | down |
| RP5-991B18.1 | -1.084613139 | 0.710860604 | | down |
| DSCR4-IT1 | -1.084613139 | 0.710860604 | | down |
| GSC2 | -1.084613139 | 0.710860604 | | down |
| MIR6086 | -1.084613139 | 0.710860604 | | down |
| RP3-339A18.3 | -1.084613139 | 0.710860604 | | down |
| RP6-22P16.1 | -1.084613139 | 0.710860604 | | down |
| RP6-43L17.1 | -1.084613139 | 0.710860604 | | down |
| RNU2-26P | -1.084613139 | 0.710860604 | | down |
| CENPV | -1.084501181 | 9.31E-07 | 1.91E-05 | down |
| RP11-483L5.1 | -1.084385643 | 0.034994559 | 0.094049247 | down |
| PGC | -1.084350964 | 0.194935295 | 0.32335667 | down |
| CSAG2 | -1.084180185 | 0.538536062 | | down |
| RP11-442O1.4 | -1.083783941 | 0.01282273 | 0.044306625 | down |
| RP11-205A8.3 | -1.083775094 | 0.443417197 | | down |
| LINC01616 | -1.083724341 | 0.580367143 | | down |
| AC005062.2 | -1.083195927 | 0.010488932 | 0.038049926 | down |
| OR2T33 | -1.083153629 | 0.452493076 | | down |
| LFNG | -1.083148996 | 1.88E-05 | 0.000238822 | down |
| GK5 | -1.083137555 | 3.67E-05 | 0.000415114 | down |
| RP11-426D19.1 | -1.082840903 | 0.128182459 | 0.240890335 | down |
| NA | -1.082765193 | 0.311390871 | 0.449996504 | down |
| ARL2 | -1.082685324 | 0.000464321 | 0.003280053 | down |
| ATP5G1 | -1.082581969 | 3.72E-05 | 0.000419632 | down |
| RP11-161H23.8 | -1.082511929 | 0.035272784 | 0.094534836 | down |
| BTBD7P1 | -1.082345219 | 0.082143755 | 0.175690679 | down |
| MROH1 | -1.08225217 | 8.84E-08 | 2.57E-06 | down |
| RP11-151A6.4 | -1.082026516 | 0.205750119 | 0.335662282 | down |
| RP11-506K6.4 | -1.081903961 | 0.09511728 | 0.19506509 | down |
| UCMA | -1.081851955 | 0.689051244 | | down |
| CERKL | -1.081694401 | 0.036226818 | 0.096549146 | down |
| LA16c-380A1.1 | -1.081582892 | 0.021638074 | 0.065805677 | down |
| RP11-333E1.1 | -1.080924209 | 0.000716788 | 0.004651831 | down |
| PPP1R14BP2 | -1.080410408 | 0.136278116 | 0.251386979 | down |
| VAMP8 | -1.080068295 | 8.63E-05 | 0.000835028 | down |
| TPPP | -1.07987976 | 0.000165485 | 0.001430335 | down |
| CRB3 | -1.079781727 | 0.000360346 | 0.002695102 | down |
| NA | -1.079390866 | 0.012781815 | 0.044193731 | down |
| TCEB2P1 | -1.079248818 | 0.211324832 | | down |
| CTD-3131K8.2 | -1.079062484 | 0.000322556 | 0.002472792 | down |
| ARPC3P1 | -1.078855031 | 0.050860628 | 0.123829554 | down |
| SLC24A4 | -1.078742715 | 0.000810196 | 0.005129932 | down |
| RP11-160E2.6 | -1.078739946 | 0.016650903 | 0.053988748 | down |
| RP11-114F3.5 | -1.078628954 | 0.254564591 | 0.389594956 | down |

| | | | | |
|---|---|---|---|---|
| DPF3 | -1.078558393 | 1.19E-05 | 0.000162306 | down |
| RP11-129B9.1 | -1.078462055 | 0.339562449 | | down |
| MTMR11 | -1.078219217 | 9.93E-06 | 0.000140305 | down |
| RP11-125M16.1 | -1.07768194 | 0.712511043 | | down |
| RP11-21L23.2 | -1.077626191 | 0.011874492 | 0.041858504 | down |
| RP11-703I16.1 | -1.07750418 | 0.000601234 | 0.004038334 | down |
| CTD-2017C7.3 | -1.077380623 | 0.383317273 | 0.521009054 | down |
| AC004471.9 | -1.077348783 | 0.053343336 | 0.128119529 | down |
| EPYC | -1.077256932 | 0.239765278 | 0.373470968 | down |
| RP11-815J21.1 | -1.077012746 | 0.190905804 | 0.318489318 | down |
| AC195454.1 | -1.076715082 | 0.132099952 | 0.246068627 | down |
| RP11-881M11.8 | -1.076610952 | 0.362467309 | | down |
| AL157902.3 | -1.076231701 | 0.454898999 | | down |
| AC002064.5 | -1.076216187 | 0.544167236 | | down |
| CLUH | -1.076198828 | 1.02E-06 | 2.07E-05 | down |
| NA | -1.076139689 | 0.079451701 | 0.171286373 | down |
| CASP14 | -1.076106472 | 0.712913526 | | down |
| RP11-460I13.2 | -1.075780791 | 0.713109935 | | down |
| RN7SL788P | -1.075780791 | 0.713109935 | | down |
| U3 | -1.075780791 | 0.713109935 | | down |
| RP11-533N14.3 | -1.075780791 | 0.713109935 | | down |
| MIR4259 | -1.075780791 | 0.713109935 | | down |
| OR13G1 | -1.075780791 | 0.713109935 | | down |
| VEZF1P1 | -1.075780791 | 0.713109935 | | down |
| RNA5SP154 | -1.075780791 | 0.713109935 | | down |
| RP11-777B9.5 | -1.075780791 | 0.713109935 | | down |
| RP4-529N6.2 | -1.075780791 | 0.713109935 | | down |
| RP11-354I10.1 | -1.075780791 | 0.713109935 | | down |
| SPACA1 | -1.075780791 | 0.713109935 | | down |
| RP11-244K5.6 | -1.075780791 | 0.713109935 | | down |
| KCNMA1-AS2 | -1.075780791 | 0.713109935 | | down |
| RN7SL846P | -1.075780791 | 0.713109935 | | down |
| OR52K2 | -1.075780791 | 0.713109935 | | down |
| LDHAL6DP | -1.075780791 | 0.713109935 | | down |
| RP11-740D6.3 | -1.075780791 | 0.713109935 | | down |
| RP11-1060J15.3 | -1.075780791 | 0.713109935 | | down |
| SALL4P7 | -1.075780791 | 0.713109935 | | down |
| RP11-1078H9.2 | -1.075780791 | 0.713109935 | | down |
| RP11-719K4.7 | -1.075780791 | 0.713109935 | | down |
| FLJ26245 | -1.075780791 | 0.713109935 | | down |
| Y_RNA | -1.075780791 | 0.713109935 | | down |
| RP11-510M2.8 | -1.075780791 | 0.713109935 | | down |
| TBC1D3P3 | -1.075780791 | 0.713109935 | | down |
| RP11-680N20.1 | -1.075780791 | 0.713109935 | | down |
| LINC01282 | -1.075780791 | 0.713109935 | | down |
| AC007312.3 | -1.07578017 | 0.704765149 | | down |
| RPS7P6 | -1.075780151 | 0.704520031 | | down |
| NA | -1.075780135 | 0.704311143 | | down |
| AC114814.4 | -1.07578007 | 0.703474271 | | down |
| SPATA31D4 | -1.075780053 | 0.703261142 | | down |
| RNU6-871P | -1.075776393 | 0.663091149 | | down |

| | | | | |
|---|---|---|---|---|
| NA | -1.075771778 | 0.623136026 | | down |
| SMIM2 | -1.075771514 | 0.621072572 | | down |
| SNX18P3 | -1.075728869 | 0.099459277 | 0.201208977 | down |
| NEURL2 | -1.075675229 | 0.015180594 | 0.050385083 | down |
| LINC01005 | -1.075590148 | 0.713130873 | | down |
| RP11-399H11.3 | -1.075590148 | 0.713130873 | | down |
| OR52T1P | -1.075590148 | 0.713130873 | | down |
| SLC22A25 | -1.075590148 | 0.713130873 | | down |
| RP11-626I20.3 | -1.075590148 | 0.713130873 | | down |
| RP11-394B2.6 | -1.075588629 | 0.293606776 | | down |
| CTD-2195M15.3 | -1.075472533 | 0.695998544 | | down |
| RP4-669H2.1 | -1.075210706 | 0.077454857 | 0.168139719 | down |
| NAMA | -1.075146272 | 0.219846987 | 0.351352884 | down |
| RP11-506O24.1 | -1.074811423 | 0.61613741 | | down |
| MIR659 | -1.074555398 | 0.207496362 | | down |
| AC083843.2 | -1.074515555 | 0.546037584 | | down |
| RP11-92K15.1 | -1.074441308 | 0.579597407 | | down |
| CACNB4 | -1.074325175 | 0.001251886 | 0.00724974 | down |
| RP11-674P19.2 | -1.074305849 | 0.202183894 | 0.331448961 | down |
| RP11-895M11.2 | -1.07426465 | 0.418104074 | | down |
| GATA6 | -1.074134387 | 3.89E-05 | 0.000434287 | down |
| RP11-420O4.2 | -1.073996208 | 0.540813388 | | down |
| LLGL2 | -1.073837753 | 4.75E-06 | 7.61E-05 | down |
| BEGAIN | -1.07374952 | 2.68E-05 | 0.000319462 | down |
| KIAA1161 | -1.073558728 | 0.000538843 | 0.003698654 | down |
| CTD-2017D11.1 | -1.073496831 | 0.000848386 | 0.005321115 | down |
| CDK20 | -1.073466938 | 4.51E-07 | 1.02E-05 | down |
| MORF4L1P5 | -1.073125601 | 0.692794093 | | down |
| YWHAEP5 | -1.073125325 | 0.248501869 | 0.382930202 | down |
| MIR8055 | -1.072911591 | 0.50574154 | | down |
| RP11-93G23.2 | -1.072879394 | 0.48570444 | 0.61449885 | down |
| SRSF9P1 | -1.072855716 | 0.012244077 | 0.042809385 | down |
| NRIR | -1.072741389 | 0.022223744 | 0.067091055 | down |
| RP11-57A19.2 | -1.072648257 | 0.032275392 | 0.088498849 | down |
| MIER3 | -1.072608603 | 2.51E-06 | 4.47E-05 | down |
| NA | -1.072558729 | 0.082945557 | 0.176914406 | down |
| RP11-102G14.1 | -1.072465858 | 0.129103311 | 0.242072082 | down |
| RP11-44N22.3 | -1.072211765 | 0.034429809 | 0.093009303 | down |
| CTD-2008P7.10 | -1.072156887 | 0.43299075 | | down |
| NDUFS6 | -1.072102241 | 4.99E-05 | 0.000535177 | down |
| RP11-234B24.4 | -1.072096316 | 0.019999215 | 0.062093604 | down |
| NA | -1.072068 | 0.000739972 | 0.004774455 | down |
| TG | -1.072033412 | 0.003758026 | 0.017356383 | down |
| SLC16A6P1 | -1.071930014 | 0.145303135 | 0.263004281 | down |
| FCGRT | -1.07189882 | 8.30E-08 | 2.44E-06 | down |
| RP11-500G9.1 | -1.071803331 | 0.587300695 | | down |
| FSIP2 | -1.071760053 | 0.00115767 | 0.006811797 | down |
| CMTM1 | -1.071713215 | 0.010785572 | 0.038867381 | down |
| RP1-149A16.16 | -1.071397109 | 0.098626455 | 0.200063009 | down |
| AF064860.7 | -1.071389931 | 0.268121108 | | down |
| ME2P1 | -1.07113714 | 0.658327119 | | down |

| | | | | |
|---|---|---|---|---|
| ASH2LP1 | -1.070964458 | 0.2520564 | | down |
| RP11-1070N10.5 | -1.070895243 | 0.33170757 | 0.470085954 | down |
| RP11-46H11.3 | -1.070865621 | 0.010278261 | 0.037443929 | down |
| RPL5P3 | -1.07077818 | 0.087849304 | | down |
| FOXD4L4 | -1.070748847 | 0.437014586 | | down |
| RP11-631M6.3 | -1.070687059 | 0.252618806 | | down |
| ZNF727 | -1.070671671 | 0.011212465 | 0.040031763 | down |
| AC064850.4 | -1.07048243 | 0.436729835 | | down |
| SLC22A1 | -1.070445836 | 0.005947819 | 0.024791381 | down |
| NA | -1.070147199 | 0.454515229 | | down |
| RP11-441F2.5 | -1.069988591 | 0.293140409 | | down |
| PDE4DIPP1 | -1.069844612 | 0.547459687 | | down |
| RP11-314N14.1 | -1.06982675 | 0.656232891 | | down |
| RP11-359J14.2 | -1.069778805 | 0.177650612 | 0.302604319 | down |
| CIB1 | -1.069749124 | 0.000138478 | 0.001238595 | down |
| CTD-3193K9.4 | -1.069651056 | 0.035729517 | 0.095440279 | down |
| RNA5SP108 | -1.069639325 | 0.514152441 | | down |
| RP11-401P9.5 | -1.069592588 | 0.029668496 | 0.083164797 | down |
| NA | -1.069467139 | 0.009596189 | 0.035486788 | down |
| NA | -1.069299137 | 0.095967704 | 0.196286589 | down |
| RP11-556N21.1 | -1.069281715 | 0.368741161 | 0.507162582 | down |
| TSPEAR | -1.069216048 | 0.008607698 | 0.032689885 | down |
| PPARGC1B | -1.06889808 | 5.03E-06 | 7.96E-05 | down |
| RNU1-42P | -1.068835854 | 0.653984839 | | down |
| ARID3C | -1.068753188 | 0.151067056 | 0.270194745 | down |
| PLXNB1 | -1.068619078 | 2.94E-06 | 5.11E-05 | down |
| PLPP2 | -1.068569251 | 0.000123988 | 0.001132245 | down |
| AC093609.1 | -1.068542057 | 0.001065641 | 0.006378385 | down |
| TOX4P1 | -1.068498861 | 0.503812071 | | down |
| HMGA2 | -1.068338209 | 0.001285467 | 0.007385843 | down |
| COX6CP14 | -1.068300212 | 0.4914839 | 0.619697091 | down |
| TMEM196 | -1.068084468 | 0.241022079 | 0.374806798 | down |
| CNKSR2 | -1.067850333 | 0.001380444 | 0.007813176 | down |
| RPS23P9 | -1.067734743 | 0.715160396 | | down |
| OR14J1 | -1.067734743 | 0.715160396 | | down |
| MTCO1P25 | -1.067734743 | 0.715160396 | | down |
| AL022345.11 | -1.067734743 | 0.715160396 | | down |
| RP11-168L7.1 | -1.067734743 | 0.715160396 | | down |
| RP11-272D12.2 | -1.067734743 | 0.715160396 | | down |
| CTD-2588J6.2 | -1.067734743 | 0.715160396 | | down |
| NA | -1.067734743 | 0.715160396 | | down |
| AC144838.2 | -1.067734743 | 0.715160396 | | down |
| AP000235.2 | -1.067734743 | 0.715160396 | | down |
| MTATP6P20 | -1.067734743 | 0.715160396 | | down |
| RP11-30B1.1 | -1.06773241 | 0.707534036 | | down |
| RP11-157E14.1 | -1.067731028 | 0.70315906 | | down |
| Y_RNA | -1.06772978 | 0.699293592 | | down |
| MIR3909 | -1.067717034 | 0.663466717 | | down |
| GGTLC1 | -1.067715443 | 0.659377783 | | down |
| CTD-2583P5.1 | -1.06771522 | 0.658810112 | | down |
| MT-TE | -1.067698747 | 0.620207243 | | down |

| | | | | |
|---|---|---|---|---|
| MIR648 | -1.067676912 | 0.576603095 | | down |
| NA | -1.067659103 | 0.545565222 | | down |
| RP11-39704.1 | -1.067657512 | 0.542952747 | | down |
| KNOP1 | -1.067400362 | 2.04E-06 | 3.75E-05 | down |
| CYP8B1 | -1.067350855 | 0.148193361 | 0.266893738 | down |
| EPN1 | -1.067192935 | 1.37E-06 | 2.68E-05 | down |
| RN7SL184P | -1.067168552 | 0.715260578 | | down |
| NA | -1.067132326 | 0.060743187 | 0.141022201 | down |
| CTD-2619J13.3 | -1.066971637 | 0.004058875 | 0.018462502 | down |
| ZNF541 | -1.066849546 | 0.006225854 | 0.02567246 | down |
| CTD-2023N9.3 | -1.066815633 | 0.583102075 | | down |
| ZNF653 | -1.066739007 | 4.71E-05 | 0.000508437 | down |
| RPL21P40 | -1.066719767 | 0.139662252 | 0.255885638 | down |
| LA16c-380A1.2 | -1.06667902 | 0.199336755 | 0.328374487 | down |
| RP11-527L4.6 | -1.066452477 | 0.157352198 | 0.278082121 | down |
| RP11-337C18.4 | -1.066438011 | 0.618465385 | | down |
| PMFBP1 | -1.066270604 | 0.006234058 | 0.025690742 | down |
| GPX3 | -1.066236587 | 0.000613221 | 0.004107832 | down |
| FLJ27354 | -1.066088393 | 0.00015235 | 0.001336796 | down |
| GABRG2 | -1.066078327 | 0.069968885 | 0.156291352 | down |
| RP11-1378G18.1 | -1.066021915 | 0.715598991 | | down |
| CTB-78021.1 | -1.065981517 | 0.323590044 | | down |
| USP6 | -1.064926043 | 0.019682379 | 0.061303897 | down |
| SRGAP2-AS1 | -1.064772575 | 0.5395086 | | down |
| RP11-403I13.5 | -1.064602014 | 0.062873835 | 0.144642301 | down |
| MAGEC2 | -1.064600946 | 0.657563453 | | down |
| RP11-181K12.2 | -1.064256879 | 0.255445165 | | down |
| RP11-374M1.9 | -1.064178391 | 0.157120676 | 0.277841883 | down |
| RP11-154J22.1 | -1.0641278 | 0.029699465 | 0.083215363 | down |
| RP6-191P20.4 | -1.064093323 | 0.660194525 | | down |
| FAM13A-AS1 | -1.063780604 | 0.000124212 | 0.001133257 | down |
| CFAP45 | -1.063731483 | 0.008182214 | 0.031463771 | down |
| RP1-90G24.11 | -1.063464943 | 0.239717269 | | down |
| SND1-IT1 | -1.063355557 | 0.339703144 | 0.478116597 | down |
| EEF1A1P3 | -1.06334885 | 0.311878969 | | down |
| PWRN4 | -1.063276911 | 0.31417832 | 0.452826743 | down |
| RHO | -1.063124082 | 0.716191494 | | down |
| EVPLL | -1.063124082 | 0.716191494 | | down |
| THOP1 | -1.062917915 | 3.55E-06 | 5.97E-05 | down |
| CLCN2 | -1.062890041 | 0.000421524 | 0.003032631 | down |
| RHOQP1 | -1.062811983 | 0.07732294 | 0.168011148 | down |
| RP11-88G17.6 | -1.062680042 | 0.393900198 | 0.531331099 | down |
| NR0B1 | -1.062669632 | 0.142369182 | 0.259288391 | down |
| LINC01513 | -1.062651602 | 0.519959525 | | down |
| RN7SKP55 | -1.06249188 | 0.716494399 | | down |
| NA | -1.06249188 | 0.716494399 | | down |
| GTF3AP6 | -1.06249188 | 0.716494399 | | down |
| RP11-544P1.3 | -1.06249188 | 0.716494399 | | down |
| RNU6-1169P | -1.06249188 | 0.716494399 | | down |
| HMGN1P26 | -1.06249188 | 0.716494399 | | down |
| RP11-616M22.10 | -1.06249188 | 0.716494399 | | down |

| | | | | |
|---|---|---|---|---|
| TECTB | -1.062490446 | 0.262531008 | | down |
| LINC00379 | -1.062479167 | 0.7055177 | | down |
| L3MBTL1 | -1.062478944 | 0.000419217 | 0.003018922 | down |
| AC010974.3 | -1.06247711 | 0.703791121 | | down |
| AC018693.6 | -1.06247364 | 0.700906107 | | down |
| MIR5095 | -1.062472134 | 0.699665657 | | down |
| MIR3944 | -1.062426436 | 0.664767663 | | down |
| RN7SL225P | -1.062424057 | 0.663078471 | | down |
| RHOT1P2 | -1.062423441 | 0.662642543 | | down |
| RP11-160C18.2 | -1.062364088 | 0.623726323 | | down |
| MIR4451 | -1.06236317 | 0.623166384 | | down |
| RNA5SP399 | -1.062283156 | 0.578215884 | | down |
| NUTF2P7 | -1.062260912 | 0.273990065 | | down |
| CTD-2231E14.5 | -1.062222871 | 0.548457321 | | down |
| NA | -1.062077934 | 0.002232869 | 0.011475252 | down |
| ABCB9 | -1.062017083 | 9.29E-05 | 0.000889128 | down |
| UROC1 | -1.061969666 | 0.180019888 | 0.305255532 | down |
| SALL4 | -1.061938341 | 0.026289287 | 0.076062725 | down |
| PHACTR2-AS1 | -1.061785091 | 0.143272122 | 0.260450635 | down |
| RP11-486013.3 | -1.061594087 | 0.29824115 | | down |
| AC138744.2 | -1.061536653 | 0.172746584 | 0.297063613 | down |
| RN7SL583P | -1.061055671 | 0.471607534 | | down |
| CHRNA2 | -1.06086365 | 0.205543958 | 0.335398798 | down |
| RP11-25010.2 | -1.060401642 | 0.449158995 | | down |
| RP11-79E3.3 | -1.060291559 | 0.416676346 | 0.552886112 | down |
| RNU7-12P | -1.060146952 | 0.697407085 | | down |
| RP11-489D6.2 | -1.06014638 | 0.236408035 | 0.369429054 | down |
| RP11-89K21.1 | -1.060051663 | 0.183252145 | 0.309036104 | down |
| FGF17 | -1.059923275 | 0.028462228 | 0.0808394 | down |
| MIR4767 | -1.059794735 | 0.629109269 | | down |
| AC005546.2 | -1.059682755 | 0.250578646 | 0.385251668 | down |
| U1 | -1.059637311 | 0.242270332 | | down |
| RP11-23I7.1 | -1.059330073 | 0.71728452 | | down |
| C10orf82 | -1.059326231 | 0.039061195 | 0.102110376 | down |
| LINC00690 | -1.059290541 | 0.49391751 | 0.622020569 | down |
| SLC25A23 | -1.059175146 | 1.48E-08 | 5.60E-07 | down |
| RP11-280024.3 | -1.05917071 | 0.661203918 | | down |
| MTND1P8 | -1.058889953 | 0.550199776 | | down |
| CLLU1OS | -1.058790814 | 0.164848659 | 0.287513822 | down |
| AC006947.1 | -1.058732548 | 0.51792059 | 0.642741118 | down |
| KCNJ6-AS1 | -1.058681618 | 0.355177547 | | down |
| SPINK2 | -1.058648915 | 0.00176567 | 0.009505925 | down |
| RP11-797J4.1 | -1.05826016 | 0.511934751 | | down |
| MIR3174 | -1.058231185 | 0.627153117 | | down |
| SYT13 | -1.05815644 | 0.001477938 | 0.008236537 | down |
| NA | -1.058137434 | 0.717606912 | | down |
| NA | -1.057953133 | 0.089440757 | 0.186763679 | down |
| RPL29P12 | -1.057947607 | 0.086604959 | 0.182509839 | down |
| CTRB2 | -1.057941954 | 0.349276787 | 0.48792419 | down |
| CTD-2213F21.2 | -1.057760859 | 0.011237926 | 0.040094082 | down |
| LINC01489 | -1.057750632 | 0.62577435 | | down |

| | | | | |
|---|---|---|---|---|
| CAMP | -1.057701821 | 0.026288526 | 0.076062725 | down |
| RP11-77P6.2 | -1.057665764 | 0.001197699 | 0.007004805 | down |
| NA | -1.057592296 | 0.59068069 | | down |
| CTD-2339F6.1 | -1.057493575 | 0.123075086 | 0.234133344 | down |
| RP11-151N17.1 | -1.057465143 | 0.073700772 | 0.162342919 | down |
| NA | -1.057354848 | 0.120458975 | 0.230778564 | down |
| WASH3P | -1.0573305 | 9.00E-06 | 0.000129712 | down |
| RPS23P1 | -1.056928746 | 0.114299058 | 0.222206775 | down |
| VDR | -1.056880093 | 2.90E-07 | 7.07E-06 | down |
| CA1 | -1.056782121 | 0.102380603 | 0.205428807 | down |
| PDCL2P2 | -1.056671495 | 0.372763065 | | down |
| INSL6 | -1.056658859 | 0.157924449 | 0.278787364 | down |
| CTB-129P6.11 | -1.056498085 | 0.020765147 | 0.063829859 | down |
| MRPS18CP6 | -1.056436048 | 0.62306073 | | down |
| BLOC1S1 | -1.056388582 | 0.002560272 | 0.012807328 | down |
| DOCK5 | -1.056138357 | 8.48E-08 | 2.48E-06 | down |
| ZER1 | -1.055780965 | 1.09E-06 | 2.19E-05 | down |
| CST4 | -1.055580414 | 0.516965173 | | down |
| MCF2L2 | -1.055303113 | 0.001014616 | 0.006135897 | down |
| ASS1P11 | -1.055099963 | 0.341519345 | | down |
| SEC14L2 | -1.055059295 | 4.30E-05 | 0.000470769 | down |
| RP11-207C16.4 | -1.055023199 | 0.204586781 | | down |
| APPL2 | -1.054965406 | 9.15E-06 | 0.000131603 | down |
| CH17-340M24.3 | -1.054851715 | 0.000112039 | 0.001040846 | down |
| OR1F12 | -1.054840469 | 0.323154164 | | down |
| RIMKLA | -1.054839586 | 0.000188168 | 0.001591504 | down |
| RP11-21K12.2 | -1.054628385 | 0.193961592 | 0.322043772 | down |
| FTH1P3 | -1.054460331 | 0.015171874 | 0.050364716 | down |
| RP5-843L14.1 | -1.054357382 | 0.69541353 | | down |
| ZNF969P | -1.054282971 | 0.479875362 | 0.609227779 | down |
| MKRNP2 | -1.054068705 | 0.283528986 | | down |
| RNU6-1065P | -1.053848045 | 0.443742829 | | down |
| RP4-665N4.4 | -1.053839257 | 0.339079099 | 0.477581568 | down |
| RN7SL262P | -1.053202355 | 0.586171415 | | down |
| RPL19P14 | -1.053171273 | 0.703157962 | | down |
| KIAA1549 | -1.053166333 | 0.002187977 | 0.011276911 | down |
| SCAANT1 | -1.052879145 | 0.051787413 | 0.125394991 | down |
| EEF1DP7 | -1.052789889 | 0.344557676 | 0.483074748 | down |
| AC007386.2 | -1.052489339 | 0.001192945 | 0.006982913 | down |
| PACRG | -1.052330882 | 0.015997099 | 0.052423304 | down |
| RP11-441F2.2 | -1.052325169 | 0.625217541 | | down |
| RP11-796E10.1 | -1.052124431 | 0.178071688 | 0.303184053 | down |
| SPTLC3 | -1.052061642 | 4.35E-05 | 0.000474759 | down |
| RNU6-658P | -1.051862412 | 0.591595723 | | down |
| AC004538.3 | -1.051517411 | 0.035419026 | 0.094833724 | down |
| CICP14 | -1.051366075 | 0.000460675 | 0.003258128 | down |
| RP11-761I4.4 | -1.051303929 | 0.112678113 | 0.219891635 | down |
| GPRC5C | -1.051262838 | 0.000130477 | 0.001178963 | down |
| RP11-342K6.4 | -1.05124795 | 0.227621383 | | down |
| KCNRG | -1.051220107 | 0.032770204 | 0.089504366 | down |
| RPSAP19 | -1.051016776 | 0.006774551 | 0.027331087 | down |

| | | | | |
|---|---|---|---|---|
| AC018755.17 | -1.050877564 | 0.256882027 | 0.391903906 | down |
| SACS-AS1 | -1.050695297 | 0.42017323 | | down |
| RP11-91H12.1 | -1.050663914 | 0.154659395 | | down |
| TMEM41A | -1.050637476 | 3.60E-09 | 1.59E-07 | down |
| CHCHD4P2 | -1.050424116 | 0.498352416 | 0.62560029 | down |
| NA | -1.050285643 | 0.230562088 | 0.362954973 | down |
| RP11-434D11.4 | -1.050284908 | 0.265839775 | 0.401553051 | down |
| SRRM2-AS1 | -1.050248159 | 0.002028165 | 0.010617562 | down |
| FAM213A | -1.050166936 | 3.75E-11 | 2.90E-09 | down |
| TEX36 | -1.050161816 | 0.507668023 | 0.63390458 | down |
| PAGE5 | -1.050085193 | 0.084660352 | 0.179476124 | down |
| RP11-146D12.2 | -1.050041438 | 0.147499315 | 0.265930714 | down |
| C19orf24 | -1.049969127 | 0.000512332 | 0.003547477 | down |
| CTC-246B18.8 | -1.049833156 | 0.252469705 | | down |
| RP11-725D20.1 | -1.049767535 | 0.465228028 | 0.596715864 | down |
| CTD-3035D6.2 | -1.049570296 | 0.100055718 | 0.202007526 | down |
| ELAVL4 | -1.049402542 | 0.008907909 | 0.033575364 | down |
| CTC-459F4.3 | -1.049307361 | 9.94E-05 | 0.000939676 | down |
| LINC00167 | -1.0491578 | 0.044443271 | 0.112209544 | down |
| RNF165 | -1.049144736 | 0.010630638 | 0.038434043 | down |
| TTC25 | -1.0485265 | 0.003651168 | 0.016938803 | down |
| CNNM2 | -1.048466699 | 2.54E-07 | 6.31E-06 | down |
| CTD-2555K7.2 | -1.048438643 | 0.021689792 | 0.065929595 | down |
| CTD-2547L16.1 | -1.048426274 | 0.002515425 | 0.012636561 | down |
| UPB1 | -1.048387853 | 0.004850205 | 0.021195573 | down |
| PHOX2B | -1.048158128 | 0.045554683 | 0.114210449 | down |
| NA | -1.048038019 | 0.554580497 | | down |
| SPATA45 | -1.048029899 | 0.146194831 | 0.264213035 | down |
| CTD-2587H24.1 | -1.047908299 | 0.287201078 | 0.424388609 | down |
| CTD-2574D22.3 | -1.047908244 | 0.005117949 | 0.022086132 | down |
| RPL34P26 | -1.047896427 | 0.222924417 | | down |
| RP11-191G24.1 | -1.047875393 | 0.003115953 | 0.014927413 | down |
| RPL23P10 | -1.047825239 | 0.720017705 | | down |
| CCDC58P3 | -1.047574491 | 0.556267016 | | down |
| SYNPR | -1.04748691 | 0.106127849 | 0.210852075 | down |
| HSPA8P15 | -1.047471027 | 0.176634408 | | down |
| RPS15AP10 | -1.047353359 | 0.247050389 | | down |
| GLYATL1P3 | -1.047310579 | 0.285738085 | 0.422812439 | down |
| LINC01291 | -1.047178148 | 0.016668147 | 0.054032996 | down |
| WDR17 | -1.047170471 | 0.019327412 | 0.06051196 | down |
| KB-68A7.1 | -1.047133979 | 0.004449998 | 0.019824397 | down |
| CLEC4GP1 | -1.046764176 | 0.018950133 | 0.059591563 | down |
| SLC31A1P1 | -1.046736631 | 0.519904005 | | down |
| C20orf144 | -1.046386606 | 0.054582799 | 0.130408398 | down |
| SPNS3 | -1.046346401 | 0.00146379 | 0.008180452 | down |
| USP26 | -1.046233009 | 0.663537009 | | down |
| FBXO24 | -1.046040517 | 0.018114569 | 0.057676359 | down |
| ABCG4 | -1.045665874 | 0.05346273 | 0.12832422 | down |
| AP003774.6 | -1.045596304 | 0.056040641 | 0.132929498 | down |
| RP11-5G9.5 | -1.045392033 | 0.168013582 | 0.29135931 | down |
| CTC-338M12.1 | -1.045012862 | 0.047378269 | 0.117580763 | down |

| | | | | |
|---|---|---|---|---|
| DNM1P47 | -1.044969012 | 0.148521356 | 0.267228149 | down |
| PROX2 | -1.044953236 | 0.059312436 | 0.138524785 | down |
| RP11-466A19.6 | -1.044731883 | 0.396747366 | | down |
| RNU6-1 | -1.044629421 | 0.591423233 | | down |
| MKLN1-AS | -1.044472357 | 4.01E-07 | 9.27E-06 | down |
| GATM | -1.044378305 | 0.000574559 | 0.003895743 | down |
| KCNIP2-AS1 | -1.044239009 | 0.259983178 | | down |
| PRSS8 | -1.044216355 | 0.010263545 | 0.037403925 | down |
| RP4-616B8.4 | -1.043584226 | 0.128949467 | 0.241908073 | down |
| CTB-26E19.1 | -1.043555682 | 0.463667563 | | down |
| GPAA1 | -1.043456697 | 5.57E-05 | 0.000583958 | down |
| RP11-640N20.4 | -1.043285507 | 0.00464064 | 0.020479345 | down |
| C19orf67 | -1.043208523 | 0.058094363 | 0.136564733 | down |
| TMEM53 | -1.043102394 | 0.000289687 | 0.00226768 | down |
| AP000255.6 | -1.04285282 | 0.011220214 | 0.040047702 | down |
| KLLN | -1.04277982 | 0.000394239 | 0.002881876 | down |
| IGHVII-31-1 | -1.042533931 | 0.526151824 | | down |
| TAF3 | -1.04240347 | 4.80E-06 | 7.67E-05 | down |
| DUSP19 | -1.04238204 | 0.001800251 | 0.009656621 | down |
| MIGA1 | -1.042348513 | 1.73E-06 | 3.26E-05 | down |
| TSPAN12 | -1.0423033 | 1.95E-06 | 3.63E-05 | down |
| RP1-45I4.3 | -1.042089435 | 0.139567478 | 0.255789989 | down |
| NA | -1.041655509 | 0.108236654 | 0.213994607 | down |
| AC003092.1 | -1.041571468 | 0.265325322 | 0.400927968 | down |
| CD2AP | -1.041543065 | 4.56E-08 | 1.45E-06 | down |
| KRT8P39 | -1.041334012 | 0.220228681 | 0.351888023 | down |
| NA | -1.041324765 | 0.630391958 | | down |
| AC016999.2 | -1.041133785 | 0.118212807 | 0.227636557 | down |
| NA | -1.040902455 | 0.460618664 | 0.592491635 | down |
| SEL1L2 | -1.040870096 | 0.167198854 | 0.290356823 | down |
| HOXB-AS3 | -1.040849324 | 0.000458353 | 0.003247052 | down |
| ARF4P2 | -1.040827073 | 0.435405782 | | down |
| RP11-544M22.13 | -1.04074978 | 0.129036763 | 0.241996286 | down |
| RP11-20B24.5 | -1.040717202 | 0.388016542 | | down |
| MIF-AS1 | -1.040585293 | 0.022256956 | 0.067169758 | down |
| TMEM38A | -1.040115056 | 2.38E-06 | 4.28E-05 | down |
| RP11-650P15.1 | -1.0399239 | 0.24538519 | | down |
| SFXN5 | -1.039624262 | 2.92E-10 | 1.74E-08 | down |
| OR7E94P | -1.03951962 | 0.204605884 | 0.334233968 | down |
| Y_RNA | -1.039397877 | 0.631854888 | | down |
| NA | -1.039363093 | 0.23703866 | 0.370164224 | down |
| RNU6-218P | -1.039293488 | 0.440876572 | 0.574501029 | down |
| AMDHD2 | -1.039151603 | 4.26E-05 | 0.000467512 | down |
| MYOZ3 | -1.038859825 | 0.010704252 | 0.038638391 | down |
| ARSDP1 | -1.038458123 | 0.405554258 | | down |
| TUBA8 | -1.03844321 | 0.083043777 | 0.177071751 | down |
| RP13-895J2.6 | -1.038075715 | 0.422306772 | | down |
| WWC1 | -1.037990169 | 9.03E-05 | 0.000868782 | down |
| RP11-776H12.1 | -1.037812995 | 0.03466713 | 0.093419971 | down |
| EPCAM | -1.037781852 | 0.003328203 | 0.015704812 | down |
| MED16 | -1.037731794 | 5.24E-05 | 0.000555769 | down |

| | | | | |
|---|---|---|---|---|
| HNRNPA1P52 | -1.037586604 | 0.139861047 | 0.256093692 | down |
| RP11-111M22.3 | -1.037366747 | 6.44E-06 | 9.83E-05 | down |
| RP11-54C4.2 | -1.037316363 | 0.632637562 | | down |
| RP11-956A19.1 | -1.037307132 | 0.522159855 | | down |
| VN1R12P | -1.037299989 | 0.70234399 | | down |
| RP11-322E11.2 | -1.037297498 | 0.229851047 | | down |
| CTD-233602.3 | -1.037295465 | 0.170823938 | 0.294685025 | down |
| GSN-AS1 | -1.037251512 | 0.015523158 | 0.051290933 | down |
| ZNF221 | -1.037200196 | 0.009557337 | 0.035392657 | down |
| LIM2 | -1.03715725 | 0.447717698 | 0.581021577 | down |
| CTD-2562G15.2 | -1.036718936 | 0.371295731 | 0.509649531 | down |
| MIR3173 | -1.036671865 | 0.632239337 | | down |
| RPL7P11 | -1.036650565 | 0.507462093 | | down |
| RP11-234B24.2 | -1.036488734 | 0.002237132 | 0.011491265 | down |
| PRAMEF20 | -1.03646656 | 0.59951283 | | down |
| TRGV8 | -1.036465904 | 0.266882768 | | down |
| RPL17P43 | -1.036292654 | 0.002470193 | 0.012450006 | down |
| GS1-124K5.10 | -1.036133138 | 0.001934031 | 0.010202989 | down |
| SOX4 | -1.036116384 | 2.51E-05 | 0.000302057 | down |
| PSMC1P3 | -1.03602631 | 0.601299545 | | down |
| SUSD4 | -1.035992319 | 0.009555782 | 0.03539126 | down |
| TLE6 | -1.035938875 | 0.000479933 | 0.003362959 | down |
| TUBB8P1 | -1.035922136 | 0.390089663 | | down |
| FAM138B | -1.035796497 | 0.197855483 | 0.326546208 | down |
| RP11-543P15.3 | -1.03572931 | 0.508843186 | | down |
| RP11-74E22.3 | -1.035657268 | 0.033149961 | 0.090251294 | down |
| CTC-459M5.2 | -1.035487854 | 0.59990476 | | down |
| RP11-535A5.1 | -1.035287751 | 0.041121432 | 0.106047408 | down |
| TRHDE-AS1 | -1.035272555 | 0.019481017 | 0.060828069 | down |
| HOXA11-AS | -1.035082955 | 0.012467751 | 0.043382149 | down |
| AGRP | -1.034872364 | 0.082604371 | 0.176399581 | down |
| GCNT7 | -1.034770635 | 0.129109408 | 0.242072082 | down |
| WFDC13 | -1.034760917 | 0.523834739 | | down |
| C14orf80 | -1.0344603 | 0.000420677 | 0.003027989 | down |
| RP11-21K12.3 | -1.034327789 | 0.118641915 | 0.228243433 | down |
| TMEM102 | -1.034080477 | 0.000115775 | 0.001070056 | down |
| RP5-1099C19.2 | -1.03407082 | 0.667517869 | | down |
| TRABD2A | -1.033968423 | 0.00016594 | 0.001433441 | down |
| RP11-109A6.5 | -1.03377349 | 0.315472721 | 0.454125937 | down |
| LINC01219 | -1.033755284 | 0.479660728 | 0.609032496 | down |
| EEF1DP1 | -1.033651362 | 0.004152137 | 0.018784339 | down |
| VCX3A | -1.033408781 | 0.431417579 | | down |
| RP4-535B20.4 | -1.033299442 | 0.205345061 | 0.335147057 | down |
| ABCA9 | -1.033293245 | 0.000840032 | 0.005278601 | down |
| HCG16 | -1.033141263 | 0.312645898 | | down |
| CAPS | -1.033065564 | 0.000543166 | 0.003721525 | down |
| RP11-685M7.3 | -1.032844022 | 0.002592766 | 0.012939713 | down |
| RP11-643M14.1 | -1.032743752 | 0.153319437 | 0.272956568 | down |
| ERICH6B | -1.03259415 | 0.238547782 | 0.371979528 | down |
| AF064858.6 | -1.032564445 | 0.00141472 | 0.007963697 | down |
| RP11-15I11.2 | -1.032422486 | 0.101575211 | 0.204306463 | down |

| | | | | |
|---|---|---|---|---|
| PPP5D1 | -1.032210414 | 0.021297486 | 0.065029808 | down |
| GSTA7P | -1.032138027 | 0.480984287 | | down |
| MAMDC2-AS1 | -1.032136213 | 0.063797513 | 0.146114475 | down |
| RP11-69M1.4 | -1.031997176 | 0.442116907 | | down |
| SOCS5P4 | -1.031867712 | 0.120579975 | 0.230922027 | down |
| NA | -1.031836607 | 0.30486345 | | down |
| NA | -1.031812234 | 0.075118338 | 0.164482583 | down |
| SLC26A8 | -1.031790129 | 0.025140484 | 0.073625177 | down |
| CABYR | -1.031569618 | 0.008186414 | 0.031475894 | down |
| IL20RA | -1.031504064 | 0.001168906 | 0.006865982 | down |
| MRPS24 | -1.031387066 | 0.043626696 | 0.110843167 | down |
| RP11-115N4.1 | -1.03138049 | 0.110300903 | 0.216865547 | down |
| GATA3 | -1.031056786 | 0.000420866 | 0.003028619 | down |
| MTND4P15 | -1.031031839 | 0.597781288 | | down |
| KCNK17 | -1.030934974 | 0.014425695 | 0.048479371 | down |
| RP1-142L7.8 | -1.030894507 | 0.599741719 | | down |
| FAM157C | -1.030852083 | 0.055095467 | 0.1312679 | down |
| CFAP73 | -1.030390356 | 0.015983336 | 0.052395347 | down |
| RP4-570O12.3 | -1.030308327 | 0.121686391 | 0.232382943 | down |
| RP11-848P1.5 | -1.029975464 | 0.002239289 | 0.011500374 | down |
| EGFL8 | -1.029891536 | 0.040735212 | 0.105304484 | down |
| GOLGA8K | -1.029867346 | 0.372640138 | | down |
| SLC13A4 | -1.029731816 | 0.01513206 | 0.050279541 | down |
| RP11-631M21.7 | -1.029133347 | 0.565211434 | | down |
| CAPN5 | -1.028997221 | 1.42E-05 | 0.000189062 | down |
| RP11-592H6.3 | -1.028970483 | 0.447394655 | 0.580627431 | down |
| LINC01532 | -1.028734868 | 0.331302174 | 0.469644358 | down |
| ZPLD1 | -1.028709367 | 0.298716596 | 0.43627638 | down |
| BDNF-AS | -1.028666895 | 0.000572947 | 0.003887448 | down |
| RP5-952N6.1 | -1.028581179 | 0.43042777 | | down |
| NA | -1.028505575 | 0.307714676 | 0.446078635 | down |
| AL928654.7 | -1.028036051 | 0.200884442 | 0.329945997 | down |
| RP11-434D2.9 | -1.027966473 | 0.214458957 | 0.345406074 | down |
| NA | -1.027391168 | 0.019284103 | 0.060439348 | down |
| ATP2B3 | -1.027074631 | 0.095379161 | 0.195386768 | down |
| NPIPB1P | -1.026736546 | 0.256100885 | 0.391129269 | down |
| SPRN | -1.026376307 | 0.057179205 | 0.134905924 | down |
| LAMB3 | -1.026167189 | 0.001084495 | 0.006473784 | down |
| HAVCR1 | -1.026073171 | 0.17351039 | 0.297967771 | down |
| CFD | -1.02604892 | 0.003526185 | 0.016471205 | down |
| KB-1440D3.14 | -1.026005177 | 0.199688926 | 0.328673954 | down |
| RP11-216M21.1 | -1.025959349 | 0.238088922 | 0.371456798 | down |
| P2RY1 | -1.02586571 | 8.56E-05 | 0.000830933 | down |
| CTD-2012K14.2 | -1.025692609 | 0.261319826 | 0.396459771 | down |
| PCDHGA1 | -1.025598523 | 0.031527408 | 0.086995724 | down |
| SUGCT | -1.025418859 | 0.000144092 | 0.001277775 | down |
| METTL24 | -1.025372504 | 0.087464831 | 0.183745752 | down |
| NA | -1.025218589 | 0.222608975 | 0.354496672 | down |
| RP5-1142J19.2 | -1.025179838 | 0.419104719 | | down |
| AL035610.1 | -1.024883421 | 0.178951911 | 0.304062392 | down |
| RP11-456K23.1 | -1.024738898 | 0.001908948 | 0.010102597 | down |

| | | | | |
|---|---|---|---|---|
| RP11-418J17.1 | -1.024708165 | 4.89E-06 | 7.80E-05 | down |
| RP11-498M5.2 | -1.024535017 | 0.599979835 | | down |
| RP11-4B16.1 | -1.024337713 | 0.080410279 | 0.172881525 | down |
| RP11-589M4.4 | -1.024213296 | 0.569284344 | | down |
| SHISA7 | -1.024155778 | 0.204401395 | 0.334078747 | down |
| AC007568.1 | -1.023769008 | 0.420674908 | 0.556454759 | down |
| NA | -1.023722237 | 0.604753217 | | down |
| MARK2P11 | -1.023685811 | 0.601452303 | | down |
| AC069513.4 | -1.023543424 | 0.032051556 | 0.088055868 | down |
| RPL23AP18 | -1.023543248 | 0.044594709 | 0.112456222 | down |
| RP5-884C9.2 | -1.023439494 | 0.233033532 | 0.365734704 | down |
| GPR85 | -1.023248986 | 0.002367288 | 0.012028207 | down |
| LINC01563 | -1.023213 | 0.162020119 | 0.283948694 | down |
| PLAC8 | -1.023205974 | 0.000486281 | 0.003401589 | down |
| RP11-286N22.6 | -1.023160368 | 0.361681011 | 0.500022833 | down |
| TSPAN1 | -1.02308929 | 0.01330525 | 0.045539179 | down |
| FAM155B | -1.022947164 | 0.01349307 | 0.046029953 | down |
| DBF4P1 | -1.022634258 | 0.407926234 | | down |
| RP11-583F2.5 | -1.022570756 | 0.378282356 | 0.516384893 | down |
| NA | -1.022298624 | 0.122281995 | 0.233126484 | down |
| PDE6C | -1.022236948 | 0.021738208 | 0.066016649 | down |
| PEX11A | -1.022233886 | 1.17E-07 | 3.26E-06 | down |
| SEC1P | -1.02211932 | 0.005811892 | 0.024343021 | down |
| MAGEE2 | -1.022021386 | 0.151107698 | 0.270223865 | down |
| ST13P13 | -1.021888836 | 0.681423936 | | down |
| RP11-108A14.1 | -1.021888816 | 0.682886065 | | down |
| UNCX | -1.021888144 | 0.722659425 | | down |
| RP1-37J18.1 | -1.021888055 | 0.726892431 | | down |
| RP11-422P22.1 | -1.021888055 | 0.726892431 | | down |
| RN7SL371P | -1.021888055 | 0.726892431 | | down |
| RP5-1066H13.4 | -1.021888055 | 0.726892431 | | down |
| RNU6-723P | -1.021888055 | 0.726892431 | | down |
| RP11-567C20.3 | -1.021888055 | 0.726892431 | | down |
| RP11-181B18.1 | -1.021888055 | 0.726892431 | | down |
| RNU6-1102P | -1.021888055 | 0.726892431 | | down |
| LPCAT2BP | -1.021888055 | 0.726892431 | | down |
| LCE6A | -1.021888055 | 0.726892431 | | down |
| RN7SL44P | -1.021888055 | 0.726892431 | | down |
| OR10J2P | -1.021888055 | 0.726892431 | | down |
| FMO8P | -1.021888055 | 0.726892431 | | down |
| RP11-357J22.1 | -1.021888055 | 0.726892431 | | down |
| RP11-540K16.2 | -1.021888055 | 0.726892431 | | down |
| FTH1P25 | -1.021888055 | 0.726892431 | | down |
| RP11-463J7.2 | -1.021888055 | 0.726892431 | | down |
| MRPS21P3 | -1.021888055 | 0.726892431 | | down |
| RP11-385M4.2 | -1.021888055 | 0.726892431 | | down |
| YWHAQP9 | -1.021888055 | 0.726892431 | | down |
| Y_RNA | -1.021888055 | 0.726892431 | | down |
| NA | -1.021888055 | 0.726892431 | | down |
| Y_RNA | -1.021888055 | 0.726892431 | | down |
| KRT18P26 | -1.021888055 | 0.726892431 | | down |

| | | | |
|---|---|---|---|
| AC010105.1 | -1.021888055 | 0.726892431 | down |
| AC127391.3 | -1.021888055 | 0.726892431 | down |
| MIR4267 | -1.021888055 | 0.726892431 | down |
| RN7SL468P | -1.021888055 | 0.726892431 | down |
| RNU2-21P | -1.021888055 | 0.726892431 | down |
| AC012462.3 | -1.021888055 | 0.726892431 | down |
| OR9S24P | -1.021888055 | 0.726892431 | down |
| RPEP2 | -1.021888055 | 0.726892431 | down |
| RNU6-873P | -1.021888055 | 0.726892431 | down |
| ICE2P2 | -1.021888055 | 0.726892431 | down |
| RP11-702L6.1 | -1.021888055 | 0.726892431 | down |
| LINC00901 | -1.021888055 | 0.726892431 | down |
| HMGN1P9 | -1.021888055 | 0.726892431 | down |
| FLYWCH1P1 | -1.021888055 | 0.726892431 | down |
| RNU6-420P | -1.021888055 | 0.726892431 | down |
| RP11-240A16.1 | -1.021888055 | 0.726892431 | down |
| RN7SL822P | -1.021888055 | 0.726892431 | down |
| RP11-340A13.2 | -1.021888055 | 0.726892431 | down |
| MTND5P13 | -1.021888055 | 0.726892431 | down |
| RP11-468N14.10 | -1.021888055 | 0.726892431 | down |
| MEPE | -1.021888055 | 0.726892431 | down |
| RP11-650J17.2 | -1.021888055 | 0.726892431 | down |
| RBM48P1 | -1.021888055 | 0.726892431 | down |
| RP11-361D14.2 | -1.021888055 | 0.726892431 | down |
| RP11-580J4.1 | -1.021888055 | 0.726892431 | down |
| RP11-294O2.1 | -1.021888055 | 0.726892431 | down |
| NA | -1.021888055 | 0.726892431 | down |
| HELT | -1.021888055 | 0.726892431 | down |
| RP11-560A7.1 | -1.021888055 | 0.726892431 | down |
| CTD-2061E9.1 | -1.021888055 | 0.726892431 | down |
| CTD-2122P11.1 | -1.021888055 | 0.726892431 | down |
| LINC01386 | -1.021888055 | 0.726892431 | down |
| RP3-486D24.1 | -1.021888055 | 0.726892431 | down |
| RP4-724P12.1 | -1.021888055 | 0.726892431 | down |
| EEF1A1P42 | -1.021888055 | 0.726892431 | down |
| RP11-346C16.6 | -1.021888055 | 0.726892431 | down |
| RFPL4B | -1.021888055 | 0.726892431 | down |
| SOCS5P5 | -1.021888055 | 0.726892431 | down |
| HMGB1P13 | -1.021888055 | 0.726892431 | down |
| RP1-45I4.2 | -1.021888055 | 0.726892431 | down |
| NA | -1.021888055 | 0.726892431 | down |
| RP11-472G23.10 | -1.021888055 | 0.726892431 | down |
| NANOGP11 | -1.021888055 | 0.726892431 | down |
| AC006000.5 | -1.021888055 | 0.726892431 | down |
| THUMPD3P1 | -1.021888055 | 0.726892431 | down |
| RP11-419M24.5 | -1.021888055 | 0.726892431 | down |
| ZNF479 | -1.021888055 | 0.726892431 | down |
| RP11-196D18.1 | -1.021888055 | 0.726892431 | down |
| SAPCD2P4 | -1.021888055 | 0.726892431 | down |
| RNA5SP233 | -1.021888055 | 0.726892431 | down |
| RN7SKP129 | -1.021888055 | 0.726892431 | down |

| | | | |
|---|---|---|---|
| AC000111.4 | -1.021888055 | 0.726892431 | down |
| CYCSP20 | -1.021888055 | 0.726892431 | down |
| AC004911.2 | -1.021888055 | 0.726892431 | down |
| USP17L1 | -1.021888055 | 0.726892431 | down |
| RP11-98P2.1 | -1.021888055 | 0.726892431 | down |
| KCTD9P6 | -1.021888055 | 0.726892431 | down |
| MIR548AO | -1.021888055 | 0.726892431 | down |
| 5S_rRNA | -1.021888055 | 0.726892431 | down |
| RP11-509P12.1 | -1.021888055 | 0.726892431 | down |
| CTC-788C1.2 | -1.021888055 | 0.726892431 | down |
| MTCO1P4 | -1.021888055 | 0.726892431 | down |
| RN7SKP153 | -1.021888055 | 0.726892431 | down |
| RP11-706C16.13 | -1.021888055 | 0.726892431 | down |
| RP11-296L22.7 | -1.021888055 | 0.726892431 | down |
| NA | -1.021888055 | 0.726892431 | down |
| RP11-791O21.5 | -1.021888055 | 0.726892431 | down |
| ZYG11AP1 | -1.021888055 | 0.726892431 | down |
| RN7SL187P | -1.021888055 | 0.726892431 | down |
| CHCHD3P1 | -1.021888055 | 0.726892431 | down |
| RP11-108B14.1 | -1.021888055 | 0.726892431 | down |
| RNA5SP304 | -1.021888055 | 0.726892431 | down |
| FAM210CP | -1.021888055 | 0.726892431 | down |
| RN7SL847P | -1.021888055 | 0.726892431 | down |
| RP11-324I22.2 | -1.021888055 | 0.726892431 | down |
| RP11-92J19.3 | -1.021888055 | 0.726892431 | down |
| RP11-272J7.6 | -1.021888055 | 0.726892431 | down |
| RP11-257I14.1 | -1.021888055 | 0.726892431 | down |
| H2AFZP5 | -1.021888055 | 0.726892431 | down |
| RP11-442O18.1 | -1.021888055 | 0.726892431 | down |
| OR51P1P | -1.021888055 | 0.726892431 | down |
| Y_RNA | -1.021888055 | 0.726892431 | down |
| NA | -1.021888055 | 0.726892431 | down |
| RP11-460B17.1 | -1.021888055 | 0.726892431 | down |
| NA | -1.021888055 | 0.726892431 | down |
| OR5T1 | -1.021888055 | 0.726892431 | down |
| RN7SL309P | -1.021888055 | 0.726892431 | down |
| RP11-665E10.1 | -1.021888055 | 0.726892431 | down |
| RP11-313I2.5 | -1.021888055 | 0.726892431 | down |
| OR10G6 | -1.021888055 | 0.726892431 | down |
| OR8X1P | -1.021888055 | 0.726892431 | down |
| ZNF123P | -1.021888055 | 0.726892431 | down |
| RP11-651K21.1 | -1.021888055 | 0.726892431 | down |
| RP11-609L23.1 | -1.021888055 | 0.726892431 | down |
| RP11-848D3.4 | -1.021888055 | 0.726892431 | down |
| LRRC10 | -1.021888055 | 0.726892431 | down |
| RP11-167N24.5 | -1.021888055 | 0.726892431 | down |
| RP11-541G9.2 | -1.021888055 | 0.726892431 | down |
| RP1-267D11.1 | -1.021888055 | 0.726892431 | down |
| RP11-417O18.2 | -1.021888055 | 0.726892431 | down |
| RP11-495K9.2 | -1.021888055 | 0.726892431 | down |
| FREM2-AS1 | -1.021888055 | 0.726892431 | down |

| | | | | |
|---|---|---|---|---|
| NA | -1.021888055 | 0.726892431 | | down |
| AMMECR1LP1 | -1.021888055 | 0.726892431 | | down |
| LDHBP1 | -1.021888055 | 0.726892431 | | down |
| EDDM3B | -1.021888055 | 0.726892431 | | down |
| RP11-226P1.2 | -1.021888055 | 0.726892431 | | down |
| RN7SKP108 | -1.021888055 | 0.726892431 | | down |
| SNORD51 | -1.021888055 | 0.726892431 | | down |
| CEND1P1 | -1.021888055 | 0.726892431 | | down |
| SPATA31E2P | -1.021888055 | 0.726892431 | | down |
| COX6CP4 | -1.021888055 | 0.726892431 | | down |
| RP11-37C7.2 | -1.021888055 | 0.726892431 | | down |
| CTD-2014N11.3 | -1.021888055 | 0.726892431 | | down |
| AGBL1-AS1 | -1.021888055 | 0.726892431 | | down |
| OR4F14P | -1.021888055 | 0.726892431 | | down |
| OR4G2P | -1.021888055 | 0.726892431 | | down |
| NA | -1.021888055 | 0.726892431 | | down |
| CTF2P | -1.021888055 | 0.726892431 | | down |
| LINC00273 | -1.021888055 | 0.726892431 | | down |
| AC009110.1 | -1.021888055 | 0.726892431 | | down |
| RP11-2C15.1 | -1.021888055 | 0.726892431 | | down |
| NPM1P45 | -1.021888055 | 0.726892431 | | down |
| TNPO1P3 | -1.021888055 | 0.726892431 | | down |
| KRTAP9-3 | -1.021888055 | 0.726892431 | | down |
| NA | -1.021888055 | 0.726892431 | | down |
| AC118653.2 | -1.021888055 | 0.726892431 | | down |
| Y_RNA | -1.021888055 | 0.726892431 | | down |
| RP11-26N15.2 | -1.021888055 | 0.726892431 | | down |
| PA2G4P3 | -1.021888055 | 0.726892431 | | down |
| NRBF2P1 | -1.021888055 | 0.726892431 | | down |
| TCEB3CL | -1.021888055 | 0.726892431 | | down |
| RPL17P46 | -1.021888055 | 0.726892431 | | down |
| LINC01029 | -1.021888055 | 0.726892431 | | down |
| AC005394.1 | -1.021888055 | 0.726892431 | | down |
| MIR517A | -1.021888055 | 0.726892431 | | down |
| RNU6-751P | -1.021888055 | 0.726892431 | | down |
| AC006116.21 | -1.021888055 | 0.726892431 | | down |
| NA | -1.021888055 | 0.726892431 | | down |
| RP13-30A9.2 | -1.021888055 | 0.726892431 | | down |
| CH507-152C13.3 | -1.021888055 | 0.726892431 | | down |
| LINC00113 | -1.021888055 | 0.726892431 | | down |
| USF1P1 | -1.021888055 | 0.726892431 | | down |
| KRTAP10-13P | -1.021888055 | 0.726892431 | | down |
| LA16c-60D12.1 | -1.021888055 | 0.726892431 | | down |
| AP000361.2 | -1.021888055 | 0.726892431 | | down |
| RP1-127L4.7 | -1.021888055 | 0.726892431 | | down |
| GS1-600G8.4 | -1.021888055 | 0.726892431 | | down |
| RNU1-142P | -1.021888055 | 0.726892431 | | down |
| OR4E1 | -1.021719634 | 0.572077234 | | down |
| CTD-2651B20.7 | -1.021279351 | 0.383081481 | | down |
| KTN1-AS1 | -1.021202714 | 0.000227424 | 0.001860757 | down |
| IGF2 | -1.021126366 | 0.110316741 | 0.216865547 | down |

| | | | | |
|---|---|---|---|---|
| MRVI1-AS1 | -1.021108534 | 0.10981192 | 0.216185878 | down |
| RP11-900F13.3 | -1.021072187 | 0.046707998 | 0.116282324 | down |
| SH2D3A | -1.021024172 | 1.41E-10 | 9.22E-09 | down |
| HOXA10-AS | -1.020965653 | 0.064545096 | 0.147287929 | down |
| RPS4XP13 | -1.020876819 | 0.073703815 | 0.162342919 | down |
| CDK11A | -1.020741809 | 0.000772246 | 0.004932262 | down |
| AP000146.2 | -1.020466141 | 0.727204884 | | down |
| AP000696.2 | -1.020298133 | 0.70813441 | | down |
| SOX3 | -1.020055131 | 0.341347477 | 0.479697068 | down |
| RP11-72M17.1 | -1.020047179 | 0.009103949 | 0.034138671 | down |
| RP11-87C7.3 | -1.020016848 | 0.563366295 | | down |
| RP11-10K16.1 | -1.019827628 | 0.000854943 | 0.005347887 | down |
| RP11-40109.3 | -1.019797628 | 0.295222548 | 0.432624438 | down |
| RPL13P12 | -1.019609954 | 0.003667115 | 0.016999663 | down |
| RP5-907D15.3 | -1.019223597 | 0.461567853 | | down |
| VGLL1 | -1.019019798 | 0.675149564 | | down |
| PRR5 | -1.018983656 | 0.000375316 | 0.002778022 | down |
| RP11-343J18.2 | -1.018868469 | 0.727615584 | | down |
| DENND2C | -1.018601094 | 0.001007597 | 0.006100815 | down |
| ABCC3 | -1.01857604 | 0.000486448 | 0.003401964 | down |
| ADGRL1 | -1.018450797 | 3.51E-06 | 5.93E-05 | down |
| STXBP2 | -1.018364991 | 0.000171696 | 0.001473836 | down |
| NVL | -1.018359944 | 2.36E-05 | 0.000287185 | down |
| LSAMP-AS1 | -1.018284353 | 0.422282537 | | down |
| KCCAT211 | -1.018277897 | 0.263633019 | 0.39904229 | down |
| HMGA1P3 | -1.017762584 | 0.140357975 | 0.256800134 | down |
| SLC25A15 | -1.017701417 | 0.000712672 | 0.004629516 | down |
| TIAM2 | -1.017515987 | 0.000123955 | 0.001132245 | down |
| GAPDHP70 | -1.017358656 | 0.472760118 | | down |
| RP11-343B5.1 | -1.01728288 | 0.003657596 | 0.016966005 | down |
| RP3-388M5.9 | -1.01722308 | 0.114052445 | 0.221857666 | down |
| PEG13 | -1.017215084 | 0.01030109 | 0.037504356 | down |
| CACNA1E | -1.017117854 | 0.029428702 | 0.082700957 | down |
| RNU7-48P | -1.016694233 | 0.492878885 | | down |
| NA | -1.016669639 | 0.037519217 | 0.099035964 | down |
| THSD4 | -1.016378527 | 6.27E-05 | 0.000643382 | down |
| ACVR1B | -1.016195201 | 1.11E-05 | 0.000153484 | down |
| SLC26A10 | -1.016151059 | 0.293907912 | | down |
| MIR130B | -1.016148573 | 0.062928428 | 0.144731049 | down |
| CTD-2012K14.5 | -1.01597754 | 0.060598557 | 0.14082009 | down |
| RP11-235E17.2 | -1.015904572 | 0.404172348 | | down |
| IL32 | -1.015861576 | 0.000168738 | 0.001453434 | down |
| CCDC177 | -1.015802652 | 0.469235883 | 0.600132939 | down |
| FEM1A | -1.015775146 | 0.000593524 | 0.003998171 | down |
| RP11-484D2.4 | -1.015713298 | 0.042333008 | 0.108427356 | down |
| RP11-50D9.3 | -1.015393288 | 0.215966245 | 0.347110188 | down |
| LOXNC01-237H1.[illegible] | -1.01527893 | 0.16858877 | 0.292036497 | down |
| SC22CB-1E7.1 | -1.015207103 | 0.001027584 | 0.006199344 | down |
| RP1-131F15.2 | -1.015168398 | 0.106061846 | 0.210752339 | down |
| CTD-3222D19.4 | -1.015025885 | 0.567780414 | | down |
| RP13-39P12.3 | -1.015009847 | 0.177388614 | 0.302415226 | down |

| | | | | |
|---|---|---|---|---|
| C16orf71 | -1.014986598 | 0.011077197 | 0.039661937 | down |
| RP11-759L5.2 | -1.014979753 | 0.424646281 | | down |
| FAM25E | -1.014910143 | 0.358869022 | | down |
| RPSAP5 | -1.014777369 | 0.395095238 | | down |
| PRRG3 | -1.014766187 | 0.0121569 | 0.042598776 | down |
| GLIPR1L1 | -1.014490272 | 0.049036437 | 0.120639594 | down |
| TMUB1 | -1.014239353 | 5.12E-06 | 8.10E-05 | down |
| AC004076.5 | -1.01410979 | 0.021967255 | 0.066517255 | down |
| CATIP-AS2 | -1.013788558 | 0.151982073 | 0.271358496 | down |
| TPMTP1 | -1.013661427 | 0.604223856 | | down |
| RP11-176N18.2 | -1.013581899 | 0.538531715 | | down |
| ERVH48-1 | -1.013494188 | 0.189967313 | 0.317288078 | down |
| RP11-219G10.1 | -1.013360696 | 0.536275052 | | down |
| GALNT9 | -1.013272848 | 0.00832418 | 0.03188318 | down |
| RP11-396C23.2 | -1.013224881 | 0.012779919 | 0.044193731 | down |
| RP11-909N17.2 | -1.012985863 | 0.49459773 | | down |
| OXER1 | -1.01289547 | 3.32E-06 | 5.66E-05 | down |
| RP11-888D10.3 | -1.012649537 | 0.05143459 | 0.124862704 | down |
| DSG1-AS1 | -1.012493306 | 0.708869892 | | down |
| RP11-302F12.1 | -1.012480674 | 0.603204084 | | down |
| RP11-380D23.1 | -1.01248033 | 0.643136086 | | down |
| RP11-1I2.1 | -1.012479874 | 0.681277661 | | down |
| LINC01054 | -1.012479852 | 0.682842044 | | down |
| AC024619.2 | -1.012479836 | 0.683946927 | | down |
| RP11-501C14.7 | -1.012479819 | 0.685146434 | | down |
| CTA-714B7.7 | -1.012479812 | 0.685624537 | | down |
| SPINK8 | -1.012479797 | 0.686589862 | | down |
| LARGE-IT1 | -1.012479226 | 0.719159823 | | down |
| RP5-1186N24.3 | -1.012479198 | 0.720533075 | | down |
| RPS6P22 | -1.012479188 | 0.720978404 | | down |
| RP5-1033H2.1 | -1.012479005 | 0.72930783 | | down |
| RP11-451O13.1 | -1.012479005 | 0.72930783 | | down |
| MIR5008 | -1.012479005 | 0.72930783 | | down |
| AC016696.1 | -1.012479005 | 0.72930783 | | down |
| AC140481.8 | -1.012479005 | 0.72930783 | | down |
| MTND4P22 | -1.012479005 | 0.72930783 | | down |
| MRPL50P2 | -1.012479005 | 0.72930783 | | down |
| RP11-785G17.1 | -1.012479005 | 0.72930783 | | down |
| RNU6-547P | -1.012479005 | 0.72930783 | | down |
| RP11-539L10.4 | -1.012479005 | 0.72930783 | | down |
| PDCD5P2 | -1.012479005 | 0.72930783 | | down |
| CCT7P1 | -1.012479005 | 0.72930783 | | down |
| NPM1P14 | -1.012479005 | 0.72930783 | | down |
| RN7SL250P | -1.012479005 | 0.72930783 | | down |
| KCNK4 | -1.012479005 | 0.72930783 | | down |
| SNX18P26 | -1.012479005 | 0.72930783 | | down |
| LINC01076 | -1.012479005 | 0.72930783 | | down |
| OR4N2 | -1.012479005 | 0.72930783 | | down |
| NA | -1.012479005 | 0.72930783 | | down |
| RP11-39M21.2 | -1.012479005 | 0.72930783 | | down |
| CRHR1 | -1.012479005 | 0.72930783 | | down |

| | | | | |
|---|---|---|---|---|
| RN7SL606P | -1.012479005 | 0.72930783 | | down |
| NA | -1.012479005 | 0.72930783 | | down |
| SIGLEC19P | -1.012479005 | 0.72930783 | | down |
| IGKV1OR22-5 | -1.012479005 | 0.72930783 | | down |
| TTTY18 | -1.012479005 | 0.72930783 | | down |
| RPS24P1 | -1.012479005 | 0.72930783 | | down |
| NA | -1.012469542 | 0.439521011 | | down |
| LINC01139 | -1.012439736 | 0.332242989 | 0.470622405 | down |
| RP11-725M22.1 | -1.012250739 | 0.587093651 | 0.702622755 | down |
| RP11-445F6.2 | -1.012034254 | 0.029160692 | 0.082201522 | down |
| RP11-849N15.3 | -1.011988437 | 0.073515161 | 0.162046184 | down |
| HID1-AS1 | -1.011871674 | 0.075692882 | 0.165375281 | down |
| DIRC3-AS1 | -1.01179611 | 0.153270614 | 0.272929251 | down |
| RP11-483G21.3 | -1.011576233 | 0.639093228 | | down |
| IGBP1-AS2 | -1.011563266 | 0.425940843 | | down |
| RPS3AP2 | -1.011440155 | 0.307240301 | | down |
| RP1-92014.3 | -1.011429154 | 0.002769839 | 0.013622041 | down |
| GOLGA8O | -1.011333985 | 0.140720247 | 0.257243633 | down |
| EVX1-AS | -1.011289669 | 0.64119004 | | down |
| AC139100.3 | -1.011276072 | 0.25747586 | 0.392601225 | down |
| CHMP4C | -1.011238476 | 0.000220983 | 0.001815981 | down |
| NA | -1.010914465 | 0.009107659 | 0.034144062 | down |
| RP11-110H1.4 | -1.010822087 | 0.141548126 | 0.258269883 | down |
| MDS2 | -1.010766191 | 0.020725667 | 0.063766713 | down |
| RP11-153F1.1 | -1.010381889 | 0.389058817 | 0.526597427 | down |
| RP11-407N8.4 | -1.010316544 | 0.534612102 | 0.657305011 | down |
| Y_RNA | -1.010128335 | 0.138005543 | 0.253774895 | down |
| SYDE2 | -1.010043466 | 0.004071205 | 0.018490593 | down |
| RPL7P26 | -1.010013804 | 0.010836183 | 0.039003507 | down |
| NA | -1.009913599 | 0.284431434 | | down |
| Y_RNA | -1.009886467 | 0.025569964 | 0.074512512 | down |
| RP3-391022.1 | -1.009761831 | 0.450858924 | | down |
| LINC01550 | -1.009759527 | 1.73E-05 | 0.000222546 | down |
| RP11-210M15.2 | -1.009683409 | 0.009248501 | 0.034534174 | down |
| CIR1P2 | -1.009634618 | 0.530953878 | | down |
| PTPN3 | -1.009600252 | 2.58E-05 | 0.000308811 | down |
| DNAH14 | -1.009578797 | 0.000676517 | 0.004436867 | down |
| HNRNPMP1 | -1.009361766 | 0.535425408 | | down |
| CTC-490E21.14 | -1.009326292 | 0.013631451 | 0.046408781 | down |
| FMO4 | -1.009262177 | 0.000125214 | 0.001140672 | down |
| RP11-84G21.1 | -1.009045775 | 0.002054863 | 0.010728803 | down |
| HSD17B11 | -1.008987866 | 2.18E-06 | 4.00E-05 | down |
| AC009495.2 | -1.008904976 | 0.022725385 | 0.068214755 | down |
| ARL4AP2 | -1.008800392 | 0.097351218 | 0.19818539 | down |
| KB-226F1.2 | -1.008665522 | 0.039665696 | 0.103313785 | down |
| AC087650.1 | -1.008564713 | 0.442882206 | | down |
| CTD-2201E9.4 | -1.008537445 | 0.476390486 | | down |
| PMM1 | -1.008363972 | 7.91E-11 | 5.54E-09 | down |
| RP11-135D11.2 | -1.008291278 | 0.023142353 | 0.069109751 | down |
| Y_RNA | -1.008153188 | 0.421906589 | 0.557421353 | down |
| LINC01498 | -1.008138583 | 0.251532488 | 0.386351843 | down |

| | | | | |
|---|---|---|---|---|
| RP11-64P14.7 | -1.007897952 | 0.039402352 | 0.10277863 | down |
| VWA7 | -1.007884121 | 0.000186612 | 0.001581094 | down |
| FAM83E | -1.00774221 | 0.000343398 | 0.002600718 | down |
| TONSL-AS1 | -1.007557866 | 0.082329723 | 0.175963165 | down |
| FRRS1 | -1.007443764 | 0.025016112 | 0.073339586 | down |
| RP11-214K3.23 | -1.007426852 | 0.158771502 | 0.279892107 | down |
| MIR3124 | -1.007374098 | 0.119799024 | 0.229910052 | down |
| UNGP1 | -1.007274802 | 0.544225476 | | down |
| RN7SKP150 | -1.006954486 | 0.279936925 | | down |
| RP11-390D11.1 | -1.00687761 | 0.485538587 | | down |
| RP11-563J2.2 | -1.006866859 | 0.00402093 | 0.018317636 | down |
| PQLC2 | -1.006835928 | 1.06E-05 | 0.000147891 | down |
| MIR4754 | -1.006817873 | 0.342043642 | | down |
| NA | -1.006738199 | 0.168965953 | 0.292403265 | down |
| TSPO | -1.006708013 | 0.000587746 | 0.003968143 | down |
| METRN | -1.006594663 | 9.33E-05 | 0.00089159 | down |
| NA | -1.006496507 | 0.057366586 | 0.135184024 | down |
| RP3-448I9.2 | -1.006402346 | 0.280470442 | 0.41684165 | down |
| RP4-737E23.2 | -1.006383919 | 0.036885988 | 0.097750959 | down |
| RAB11FIP4 | -1.006367039 | 1.54E-12 | 1.63E-10 | down |
| FRG1DP | -1.005977673 | 0.030757758 | 0.085389584 | down |
| RP3-407E4.4 | -1.005946562 | 0.455719579 | | down |
| GNG13 | -1.00591876 | 0.010793741 | 0.038888015 | down |
| INF2 | -1.005884629 | 1.27E-07 | 3.48E-06 | down |
| HIST1H4J | -1.005854253 | 0.014030869 | 0.047471106 | down |
| RP11-574K11.8 | -1.005799703 | 0.382526577 | | down |
| FAM19A3 | -1.005739509 | 0.200574806 | 0.329671725 | down |
| GGTLC4P | -1.005732865 | 0.08052499 | 0.173029095 | down |
| RP11-7807.3 | -1.005492855 | 0.481295368 | | down |
| XDH | -1.005444143 | 0.013865563 | 0.047038954 | down |
| OMP | -1.005444097 | 0.087149862 | 0.183307949 | down |
| RP11-977B10.2 | -1.005337355 | 0.052168348 | 0.126006047 | down |
| NA | -1.005134981 | 0.110433458 | 0.217023993 | down |
| RP3-522J7.6 | -1.005101286 | 0.198011488 | 0.326741008 | down |
| LRRC31 | -1.005052532 | 0.007483928 | 0.029436889 | down |
| RPS12P26 | -1.004811846 | 0.053934452 | 0.129194648 | down |
| GRIN3A | -1.004807044 | 0.002739588 | 0.013515299 | down |
| LINC01126 | -1.004638559 | 0.028563634 | 0.081018957 | down |
| UNC5CL | -1.004383723 | 0.007305373 | 0.028886894 | down |
| LDHAP7 | -1.004287706 | 0.286609328 | | down |
| RP13-685P2.8 | -1.003936994 | 0.458141925 | | down |
| SAMD13 | -1.00393438 | 0.000559035 | 0.003808536 | down |
| AC096570.2 | -1.003646662 | 0.731577077 | | down |
| IGKV1D-42 | -1.003646662 | 0.731577077 | | down |
| AC026202.5 | -1.003646662 | 0.731577077 | | down |
| PROS2P | -1.003646662 | 0.731577077 | | down |
| AC004051.2 | -1.003646662 | 0.731577077 | | down |
| RP11-760D2.1 | -1.003646662 | 0.731577077 | | down |
| RNU1-58P | -1.003646662 | 0.731577077 | | down |
| NIFKP1 | -1.003646662 | 0.731577077 | | down |
| RP11-554A11.8 | -1.003646662 | 0.731577077 | | down |

| | | | | |
|---|---|---|---|---|
| RP11-684B2.5 | -1.003646662 | 0.731577077 | | down |
| RP11-178H8.1 | -1.003646662 | 0.731577077 | | down |
| C11orf44 | -1.003646662 | 0.731577077 | | down |
| RP11-155N3.4 | -1.003646662 | 0.731577077 | | down |
| CTD-2311B13.5 | -1.003646662 | 0.731577077 | | down |
| CTD-2576F9.2 | -1.003646662 | 0.731577077 | | down |
| SNORA31 | -1.003646662 | 0.731577077 | | down |
| RP5-1125A11.4 | -1.003646662 | 0.731577077 | | down |
| RP5-1119A7.11 | -1.003646662 | 0.731577077 | | down |
| RP11-486P11.1 | -1.003646195 | 0.725939464 | | down |
| PRR13P3 | -1.003646011 | 0.723783758 | | down |
| RP11-24I21.1 | -1.003645891 | 0.722403392 | | down |
| RP5-988G15.1 | -1.00364579 | 0.721259595 | | down |
| RP11-558A11.1 | -1.003645756 | 0.720866763 | | down |
| SPATA16 | -1.003645657 | 0.719761432 | | down |
| AC011753.5 | -1.003645495 | 0.717957712 | | down |
| RP11-1012A1.7 | -1.003642224 | 0.685658139 | | down |
| RP11-98J23.1 | -1.003641991 | 0.68358982 | | down |
| HTR1E | -1.003641634 | 0.680457894 | | down |
| NA | -1.003641575 | 0.679947169 | | down |
| CTD-2333K2.1 | -1.0036415 | 0.67930087 | | down |
| RP4-587D13.2 | -1.003456135 | 0.73160004 | | down |
| OTUD6A | -1.003456135 | 0.73160004 | | down |
| IZUMO1 | -1.003436946 | 0.059535725 | 0.138903696 | down |
| CECR5-AS1 | -1.003435978 | 0.296380994 | 0.433793082 | down |
| RPSAP67 | -1.003341949 | 0.715935381 | | down |
| RP11-1191J2.4 | -1.003304887 | 0.430203931 | | down |
| PHBP12 | -1.003144877 | 0.516088803 | | down |
| CH17-12M21.1 | -1.003067132 | 0.681872307 | | down |
| SCDP1 | -1.003051976 | 0.680118843 | | down |
| AC011294.3 | -1.003016102 | 0.676014307 | | down |
| ATP5F1P6 | -1.002808048 | 0.642860593 | | down |
| SNORD45 | -1.002806657 | 0.674823714 | | down |
| RPL5P31 | -1.001908316 | 0.731951434 | | down |
| KLF2P2 | -1.001614628 | 0.350363282 | 0.489112913 | down |
| TTLL6 | -1.001593907 | 0.003705817 | 0.017142044 | down |
| CYB5A | -1.001371723 | 0.000120692 | 0.001108546 | down |
| NR3C2 | -1.001263455 | 0.000292559 | 0.002284208 | down |
| SETP5 | -1.00113811 | 0.158735764 | 0.279892107 | down |
| DDX55P1 | -1.001034153 | 0.377877881 | | down |
| ABHD18 | -1.00100401 | 2.82E-06 | 4.95E-05 | down |
| CMA1 | -1.000938145 | 0.016210299 | 0.05294871 | down |
| RNF217-AS1 | -1.000886635 | 0.039816222 | 0.103597349 | down |
| SOD1P1 | -1.000836156 | 0.610387858 | | down |
| RP11-203J24.9 | -1.00072826 | 0.078948173 | 0.170518973 | down |
| HOXA4 | -1.000694078 | 0.071762778 | 0.159257332 | down |
| TAAR3 | -1.000593062 | 0.147822385 | 0.266393873 | down |
| PEMT | -1.000489394 | 4.27E-05 | 0.000468573 | down |
| RP11-506M13.3 | -1.00040991 | 0.004511172 | 0.020037499 | down |
| CTD-2203K17.1 | -1.000135969 | 0.014447397 | 0.048514318 | down |
| LRRC3 | -0.999659943 | 6.27E-05 | 0.000643382 | down |

| | | | | |
|---|---|---|---|---|
| RP11-34F20.7 | -0.999565761 | 0.028466822 | 0.080840463 | down |
| U91328.2 | -0.999551618 | 0.21702897 | 0.348312948 | down |
| CTD-2337A12.1 | -0.999150149 | 0.369521169 | | down |
| LINC01627 | -0.999149218 | 0.282821171 | 0.419339647 | down |
| AC005780.1 | -0.999093342 | 0.551864649 | | down |
| NDUFA10 | -0.998899497 | 1.82E-06 | 3.41E-05 | down |
| SLC25A6P2 | -0.998886291 | 0.081397266 | 0.174392032 | down |
| MED28P1 | -0.998863557 | 0.645659745 | | down |
| RP11-439K3.1 | -0.998661009 | 0.168140771 | 0.291512914 | down |
| CTD-3179P9.1 | -0.998385258 | 0.019353458 | 0.060580881 | down |
| RP11-96D1.7 | -0.998111255 | 0.257444147 | | down |
| LHFPL3-AS2 | -0.998081809 | 0.004446062 | 0.01981568 | down |
| RP11-91P24.7 | -0.998051644 | 0.208671658 | | down |
| STRADB | -0.997914608 | 1.26E-05 | 0.000170786 | down |
| AC005355.2 | -0.997787765 | 0.002428428 | 0.012284844 | down |
| SLC18A1 | -0.997598858 | 0.017885857 | 0.057105491 | down |
| NA | -0.997504911 | 0.253299666 | 0.388252394 | down |
| AC092170.1 | -0.99750318 | 0.681137246 | 0.779046946 | down |
| SUMO2P20 | -0.997391137 | 0.572131034 | | down |
| PCDH9 | -0.997357797 | 0.01561119 | 0.051501327 | down |
| RAPSN | -0.997119283 | 0.001222579 | 0.007119822 | down |
| RP11-946L16.1 | -0.997044507 | 0.246447485 | | down |
| KRT18P38 | -0.996636024 | 0.377610006 | | down |
| MCAT | -0.996606717 | 0.000133915 | 0.001204776 | down |
| SLC2A4 | -0.996484345 | 0.001340297 | 0.007638066 | down |
| IFITM5 | -0.996230556 | 0.217062656 | 0.348329796 | down |
| RP11-507K2.3 | -0.996168896 | 0.064770235 | 0.147611084 | down |
| SLC25A51P3 | -0.996142682 | 0.71285331 | | down |
| TAL2 | -0.995842794 | 0.308194658 | | down |
| RNF43 | -0.9957471 | 0.000163069 | 0.001413517 | down |
| RNU7-174P | -0.995738349 | 0.540101914 | 0.66195242 | down |
| NEGR1-IT1 | -0.995600615 | 0.733645602 | | down |
| AC026185.1 | -0.995600615 | 0.733645602 | | down |
| RP11-112L7.1 | -0.995600615 | 0.733645602 | | down |
| RNU1-23P | -0.995600615 | 0.733645602 | | down |
| AC011718.2 | -0.995600615 | 0.733645602 | | down |
| Metazoa_SRP | -0.995600615 | 0.733645602 | | down |
| KRTAP5-6 | -0.995596816 | 0.722258837 | | down |
| NA | -0.995596447 | 0.721190232 | | down |
| RP11-321L2.1 | -0.995596173 | 0.720399547 | | down |
| RN7SL356P | -0.995596173 | 0.720400944 | | down |
| CTA-280A3__B.2 | -0.995596089 | 0.720157728 | | down |
| RP11-711M9.2 | -0.995582979 | 0.685566432 | | down |
| COX6B1 | -0.995581521 | 0.000119917 | 0.001102441 | down |
| RP11-468N14.13 | -0.995580933 | 0.680635058 | | down |
| SAMHD1 | -0.995575574 | 0.000177305 | 0.001515058 | down |
| RPS5P3 | -0.995564793 | 0.644970405 | | down |
| NA | -0.995564471 | 0.644308023 | | down |
| RNU6-782P | -0.995563029 | 0.641367052 | | down |
| FAM110C | -0.995397414 | 0.00018133 | 0.001542658 | down |
| AC002059.10 | -0.995371092 | 0.036943716 | 0.097846417 | down |

| | | | | |
|---|---|---|---|---|
| RP11-755B10.3 | -0.995290617 | 0.552368744 | | down |
| AC064871.3 | -0.995199985 | 0.73360896 | | down |
| RP11-332L8.1 | -0.994866845 | 0.645579719 | | down |
| ZNF165 | -0.99481525 | 0.000149881 | 0.001319373 | down |
| KIAA1257 | -0.994757575 | 0.001027196 | 0.006199072 | down |
| UPK3B | -0.994688183 | 0.009111215 | 0.034150289 | down |
| JPX | -0.994304851 | 0.009913198 | 0.036391999 | down |
| CCKAR | -0.994286172 | 0.733660636 | | down |
| Metazoa_SRP | -0.994248205 | 0.5414815 | | down |
| RP11-1007O24.3 | -0.994142655 | 0.002619403 | 0.013042318 | down |
| FSCN3 | -0.993827477 | 0.155422087 | 0.275551822 | down |
| RP11-382D12.2 | -0.993810021 | 0.036472658 | 0.096969464 | down |
| CES5A | -0.993795069 | 0.29008898 | 0.427310909 | down |
| MTRNR2L2 | -0.993678286 | 0.034195986 | 0.092483502 | down |
| NA | -0.993674156 | 0.02078734 | 0.063864899 | down |
| AC004895.4 | -0.993486445 | 0.072788313 | 0.160904428 | down |
| RBMXP2 | -0.993427387 | 0.092712337 | 0.191466143 | down |
| RP11-504I13.3 | -0.993153127 | 0.246417376 | 0.380576962 | down |
| CTB-63M22.1 | -0.992807188 | 0.001246317 | 0.007223063 | down |
| HADH | -0.992651509 | 7.46E-10 | 3.98E-08 | down |
| TMEM132B | -0.992611056 | 0.013250342 | 0.045366754 | down |
| RP11-264L1.3 | -0.992583768 | 0.472860233 | | down |
| ANKRD30B | -0.992551965 | 0.200345875 | 0.32943963 | down |
| NKX2-1 | -0.992439834 | 0.714680178 | | down |
| RP11-166B2.1 | -0.992155137 | 0.017567578 | 0.056310714 | down |
| RP11-425D17.2 | -0.992026398 | 0.164596702 | 0.287124387 | down |
| PDXP | -0.991562959 | 0.019272088 | 0.060426906 | down |
| CTD-3094K11.1 | -0.991304356 | 0.000255899 | 0.002051286 | down |
| ABCD1 | -0.991298092 | 4.78E-07 | 1.08E-05 | down |
| NA | -0.991278426 | 0.26573893 | | down |
| NA | -0.991156241 | 0.311811617 | | down |
| OVOL1 | -0.99113082 | 0.005400166 | 0.02302293 | down |
| ATP1A2 | -0.99097938 | 0.018036748 | 0.057477308 | down |
| ACOT4 | -0.990909886 | 1.44E-05 | 0.000191565 | down |
| VCX3B | -0.990586616 | 0.373046297 | | down |
| COX4I1 | -0.990581633 | 5.18E-06 | 8.17E-05 | down |
| NA | -0.990447581 | 0.040569631 | 0.105000892 | down |
| RP11-292E2.2 | -0.990439745 | 0.544050334 | | down |
| TIMM9P2 | -0.99039979 | 0.250014888 | 0.384610716 | down |
| NA | -0.990370012 | 0.448382604 | | down |
| ERICH3-AS1 | -0.990357787 | 0.734991493 | | down |
| RNU6-716P | -0.990357787 | 0.734991493 | | down |
| CTD-2210P24.6 | -0.990343982 | 0.7238461 | | down |
| BIN2P1 | -0.990342347 | 0.722563335 | | down |
| GLDN | -0.990292678 | 0.02006296 | 0.062256926 | down |
| RP11-45F15.2 | -0.990291128 | 0.685590347 | | down |
| HNRNPA1P22 | -0.990291055 | 0.685541752 | | down |
| RP11-196I18.3 | -0.990287391 | 0.683102512 | | down |
| TRGV2 | -0.990268317 | 0.057767385 | 0.135962657 | down |
| NA | -0.99023803 | 0.652334893 | | down |
| EIF4BP8 | -0.990230947 | 0.648202586 | | down |

| | | | | |
|---|---|---|---|---|
| RP11-25I15.2 | -0.990196659 | 0.734970918 | | down |
| RP11-61708.1 | -0.990196659 | 0.734970918 | | down |
| ARF4P1 | -0.990158486 | 0.60917571 | | down |
| AC006116.20 | -0.990089694 | 0.008722208 | 0.033020414 | down |
| RP11-344N10.2 | -0.990085112 | 0.574491231 | | down |
| RP11-370B11.3 | -0.989952959 | 0.727932491 | | down |
| RP11-31E13.2 | -0.98985978 | 0.570288143 | | down |
| ZBTB8A | -0.989826722 | 8.66E-05 | 0.000836904 | down |
| ANKRD66 | -0.989813817 | 0.53630307 | | down |
| RP11-372E1.4 | -0.989795297 | 0.612009779 | | down |
| CTAGE12P | -0.989789001 | 0.465395811 | | down |
| BNIP3P25 | -0.989729811 | 0.575620302 | | down |
| RP11-653J6.1 | -0.989722442 | 0.57180324 | | down |
| RP11-553A21.3 | -0.98968629 | 0.10211749 | 0.205077823 | down |
| BRAFP1 | -0.989588604 | 0.466386607 | | down |
| RP11-677M14.3 | -0.989355392 | 0.00014474 | 0.001281646 | down |
| RPL17P22 | -0.989279943 | 0.577261971 | | down |
| NA | -0.989075791 | 0.549070602 | | down |
| RP11-572M11.4 | -0.989032657 | 0.011008731 | 0.039482667 | down |
| RP11-386M24.5 | -0.988990344 | 0.463377452 | | down |
| LINC01588 | -0.988979277 | 0.000144924 | 0.001282504 | down |
| PKP3 | -0.988951735 | 0.002079515 | 0.010822268 | down |
| DYNLL1P1 | -0.988792629 | 0.050080258 | 0.122435615 | down |
| RP11-77M5.1 | -0.988667041 | 0.433422286 | | down |
| ECHDC2 | -0.98864884 | 2.11E-12 | 2.16E-10 | down |
| ENPP6 | -0.988635683 | 0.00091979 | 0.005676698 | down |
| RP11-923I11.4 | -0.988627099 | 0.07490298 | 0.164165826 | down |
| SGMS1-AS1 | -0.988089216 | 8.69E-07 | 1.80E-05 | down |
| LINC01103 | -0.98807975 | 0.612125058 | | down |
| RNU6-558P | -0.988009205 | 0.225918051 | 0.357970673 | down |
| ACVR2B | -0.98789816 | 1.05E-05 | 0.000147155 | down |
| MYOZ2 | -0.987885739 | 0.086342712 | 0.182091846 | down |
| CERS3-AS1 | -0.987684776 | 0.087268505 | 0.183460594 | down |
| KLHL7-AS1 | -0.987596884 | 0.042722077 | 0.109126175 | down |
| PRTN3 | -0.987267815 | 0.22913268 | 0.361557891 | down |
| RP11-61K12.2 | -0.986985968 | 0.487451501 | | down |
| POLRMT | -0.986853392 | 2.22E-06 | 4.05E-05 | down |
| RP11-759F5.1 | -0.986820334 | 0.446146302 | 0.579482923 | down |
| MIGA2 | -0.986685658 | 3.33E-07 | 7.94E-06 | down |
| RP11-346E8.1 | -0.9865448 | 0.484273462 | | down |
| RPL23AP95 | -0.986456487 | 0.230587232 | | down |
| RP11-691H4.4 | -0.98622387 | 0.394842383 | | down |
| AC004112.4 | -0.986199887 | 0.289757293 | 0.427010748 | down |
| TRBV29OR9-2 | -0.986009604 | 0.508993279 | | down |
| FRG1KP | -0.985924124 | 0.380170281 | | down |
| RP11-14N9.1 | -0.985909878 | 0.535672947 | | down |
| TRIM45 | -0.985901974 | 9.91E-05 | 0.000937666 | down |
| RP11-486B10.4 | -0.98586324 | 0.037317358 | 0.098607098 | down |
| RP11-823P9.4 | -0.985817391 | 0.345761364 | 0.484336741 | down |
| RP11-462G2.2 | -0.985801203 | 0.434294287 | 0.56908311 | down |
| TESK2 | -0.985558865 | 0.000590189 | 0.003981061 | down |

| | | | | |
|---|---|---|---|---|
| ZNF837 | -0.985272935 | 3.81E-05 | 0.000427264 | down |
| C5orf60 | -0.98511949 | 0.40756788 | | down |
| HGFAC | -0.985110246 | 0.051651103 | 0.125214903 | down |
| RP11-167B3.3 | -0.985032615 | 0.202477571 | 0.331782718 | down |
| CTC-459F4.1 | -0.984819884 | 0.018861241 | 0.059392294 | down |
| OR2L2 | -0.984738485 | 0.364970198 | | down |
| SEPP1 | -0.984708149 | 0.001239238 | 0.007193058 | down |
| RP11-819M15.1 | -0.984472584 | 0.514769598 | | down |
| AC005498.3 | -0.984405728 | 0.085033957 | 0.180115637 | down |
| AC004549.6 | -0.984385049 | 0.553850907 | | down |
| CTD-2396E7.7 | -0.984059839 | 0.736442446 | | down |
| TRBV16 | -0.983969287 | 0.486733719 | | down |
| RP11-30K9.6 | -0.983920282 | 0.010281 | 0.037449366 | down |
| TRPV6 | -0.983876587 | 0.026243043 | 0.07598747 | down |
| DGCR5 | -0.983828651 | 0.092584444 | 0.191294067 | down |
| RNU7-50P | -0.983825274 | 0.660413485 | 0.762224376 | down |
| RP4-550H1.7 | -0.983785874 | 0.346170045 | | down |
| C19orf45 | -0.983694068 | 0.031093134 | 0.086105826 | down |
| RPSAP17 | -0.983578703 | 0.020933669 | 0.06422908 | down |
| ZBTB20 | -0.983573647 | 0.001768553 | 0.009513772 | down |
| CTB-131K11.1 | -0.983217536 | 0.327708301 | | down |
| RP11-305L7.1 | -0.983072549 | 0.006312883 | 0.025917497 | down |
| CYP2D7 | -0.98298477 | 0.002809702 | 0.013775244 | down |
| OCLN | -0.982400352 | 0.008675019 | 0.032858758 | down |
| RMDN2 | -0.982146928 | 0.000845658 | 0.005306382 | down |
| AC006373.1 | -0.981847643 | 0.616381824 | | down |
| POU6F2-AS2 | -0.981707504 | 0.581152216 | | down |
| UQCR10 | -0.98169208 | 0.001133132 | 0.006706521 | down |
| NR5A2 | -0.981643285 | 0.000111036 | 0.001032803 | down |
| PPARA | -0.981627903 | 3.37E-09 | 1.51E-07 | down |
| NA | -0.981543078 | 2.77E-06 | 4.88E-05 | down |
| NA | -0.981515987 | 0.391357099 | | down |
| AC006077.3 | -0.981466227 | 0.003842446 | 0.017659849 | down |
| TSTD1 | -0.981315301 | 5.10E-05 | 0.00054282 | down |
| MAMDC4 | -0.981186344 | 5.31E-05 | 0.000560533 | down |
| FASTK | -0.981039161 | 8.60E-05 | 0.000833558 | down |
| PEX26 | -0.980158644 | 8.14E-10 | 4.28E-08 | down |
| RP11-539L10.3 | -0.979914589 | 0.007547897 | 0.029619779 | down |
| RP5-908M14.10 | -0.979760084 | 0.003030579 | 0.014624273 | down |
| SPATC1 | -0.979611326 | 0.009813031 | 0.036090427 | down |
| PRSS30P | -0.979591794 | 0.00851333 | 0.032413444 | down |
| FAM47E | -0.979436724 | 0.004973635 | 0.021609239 | down |
| NA | -0.979371703 | 0.650929771 | | down |
| RP5-854E16.2 | -0.979367539 | 0.03255394 | 0.089081709 | down |
| RP11-1376P16.1 | -0.979216144 | 0.59247802 | | down |
| NA | -0.979050069 | 0.613257931 | | down |
| NA | -0.978866805 | 0.215591846 | 0.346693901 | down |
| NA | -0.97874382 | 0.492991458 | | down |
| NANOGNBP3 | -0.978600735 | 0.201742319 | 0.330906672 | down |
| GPM6A | -0.978554753 | 0.011084018 | 0.039672175 | down |
| RP11-80H18.4 | -0.978287506 | 0.168183708 | 0.291536541 | down |

| | | | | |
|---|---|---|---|---|
| NA | -0.978158882 | 0.25443914 | 0.389482321 | down |
| UROS | -0.977981033 | 1.50E-05 | 0.000199015 | down |
| LRIT2 | -0.977964771 | 0.12553365 | 0.237322739 | down |
| HILS1 | -0.977825407 | 0.170640582 | 0.294487085 | down |
| RP11-541E12.1 | -0.977744922 | 0.721308429 | | down |
| RP11-764D10.2 | -0.977645765 | 0.171812127 | 0.295995427 | down |
| AKR1C3 | -0.977587521 | 0.001029273 | 0.00620474 | down |
| NA | -0.977556323 | 0.294014472 | | down |
| RDH5 | -0.977538085 | 0.001152111 | 0.006788394 | down |
| RP11-544D21.2 | -0.977462451 | 0.594887251 | 0.709040844 | down |
| NA | -0.977361909 | 0.242345179 | 0.376241141 | down |
| CST3 | -0.977288403 | 9.14E-05 | 0.000876956 | down |
| ETV2 | -0.977229414 | 0.000672376 | 0.004415486 | down |
| RP11-210N13.1 | -0.977198655 | 0.352260392 | 0.491013789 | down |
| RNU6-1024P | -0.977193955 | 0.414948428 | | down |
| NA | -0.977171277 | 0.291832685 | 0.429038031 | down |
| Metazoa_SRP | -0.977156225 | 0.3856753 | 0.523409012 | down |
| RP11-1134I14.8 | -0.977050349 | 0.043312101 | 0.110269037 | down |
| NA | -0.976566345 | 0.095671101 | 0.195813241 | down |
| LGALS9B | -0.976342702 | 0.0120224 | 0.042255614 | down |
| RP11-420A23.1 | -0.97618628 | 0.004958673 | 0.021556701 | down |
| PAQR8 | -0.97586786 | 2.93E-06 | 5.10E-05 | down |
| FAM19A2 | -0.975763791 | 0.006907352 | 0.02772168 | down |
| DDX50P2 | -0.975342428 | 0.454337355 | 0.586900546 | down |
| RP11-693J15.6 | -0.97533264 | 0.4780314 | | down |
| TM4SF19-AS1 | -0.975291886 | 0.119729391 | 0.229791097 | down |
| DLEU7 | -0.975291016 | 0.051786849 | 0.125394991 | down |
| RP11-428P16.2 | -0.975290647 | 0.318237805 | 0.457142559 | down |
| SNORA68 | -0.975201704 | 0.342147659 | | down |
| PGM5-AS1 | -0.975169367 | 0.024046521 | 0.071209677 | down |
| MYO3A | -0.974913148 | 0.027618643 | 0.078981013 | down |
| RPL36AP39 | -0.974839225 | 0.618214186 | | down |
| AC015922.7 | -0.974746064 | 0.425751313 | | down |
| RP11-469N6.1 | -0.974638669 | 0.250604803 | 0.385261766 | down |
| PRRG4 | -0.974596594 | 0.000184271 | 0.001564353 | down |
| NA | -0.974506357 | 0.522175064 | | down |
| DYNLRB2 | -0.974273887 | 0.020451601 | 0.063139241 | down |
| RP11-166B2.9 | -0.974210671 | 0.546550182 | | down |
| AC016722.4 | -0.974001261 | 0.135559507 | 0.250461216 | down |
| LINC00543 | -0.973984915 | 0.001507342 | 0.00836781 | down |
| CTD-2373H9.5 | -0.973719762 | 0.400239101 | | down |
| PWWP2B | -0.973479063 | 5.90E-06 | 9.12E-05 | down |
| CYYR1-AS1 | -0.973408258 | 0.435998098 | | down |
| RP11-36I17.2 | -0.973334573 | 0.097010391 | 0.197786387 | down |
| ACVR1C | -0.973245239 | 0.004730186 | 0.020782935 | down |
| SPATA25 | -0.972957756 | 0.024904919 | 0.073084918 | down |
| SMPD1 | -0.972868186 | 0.000280441 | 0.002209696 | down |
| RP11-548H18.2 | -0.972690194 | 0.400068216 | | down |
| RP11-314C9.2 | -0.972602102 | 0.457552711 | | down |
| LINC00384 | -0.972278591 | 0.264099148 | 0.399566664 | down |
| PRL | -0.972190079 | 0.330348532 | | down |

| | | | | |
|---|---|---|---|---|
| CTD-2152M20.2 | -0.972163457 | 0.002621084 | 0.013046365 | down |
| CTD-2026K11.4 | -0.971903726 | 0.01302036 | 0.044793737 | down |
| HIST1H4L | -0.971862548 | 0.198672141 | 0.327484448 | down |
| ID1 | -0.971765534 | 9.20E-06 | 0.00013201 | down |
| ANO10 | -0.971561936 | 6.92E-07 | 1.49E-05 | down |
| NA | -0.971363746 | 0.652575761 | | down |
| RP11-21B21.4 | -0.971326206 | 0.080932115 | 0.173709862 | down |
| RP11-452C13.1 | -0.971304637 | 0.061631398 | 0.142602275 | down |
| CTC-453G23.5 | -0.971294175 | 0.199537625 | 0.328542139 | down |
| MAST2 | -0.971042622 | 1.97E-11 | 1.61E-09 | down |
| FOXD2-AS1 | -0.97104252 | 0.000768222 | 0.00491492 | down |
| KLK15 | -0.970944254 | 0.056238754 | 0.133283672 | down |
| P4HA2-AS1 | -0.970881848 | 0.515867513 | | down |
| LA16c-360H6.1 | -0.970630184 | 0.024838356 | 0.072946586 | down |
| PICK1 | -0.970615139 | 1.70E-05 | 0.000218902 | down |
| ATP1A1 | -0.970401418 | 2.18E-05 | 0.000268155 | down |
| AC000068.5 | -0.970223647 | 0.064194758 | 0.14671648 | down |
| UNC5B-AS1 | -0.970204347 | 0.03670372 | 0.097439831 | down |
| CTD-3076O17.1 | -0.970134208 | 0.318478498 | 0.457335246 | down |
| GVQW2 | -0.970051766 | 0.008465356 | 0.032275781 | down |
| GPATCH3 | -0.96979766 | 1.18E-05 | 0.000161643 | down |
| ASXL3 | -0.969706686 | 0.002647908 | 0.01314938 | down |
| RPL38P4 | -0.969432433 | 0.61932692 | | down |
| RP11-330L19.1 | -0.969354118 | 0.075627012 | 0.165294481 | down |
| RP11-967K21.1 | -0.969301021 | 0.026156074 | 0.075801399 | down |
| OR5G5P | -0.969256463 | 0.691552055 | | down |
| CCDC153 | -0.969058823 | 2.31E-06 | 4.17E-05 | down |
| ZNF711 | -0.96898654 | 1.33E-05 | 0.000178877 | down |
| RP11-502H18.2 | -0.968955153 | 0.6879838 | | down |
| IGF2BP2 | -0.968947043 | 2.93E-06 | 5.11E-05 | down |
| LINC01558 | -0.968577846 | 0.012939881 | 0.04457812 | down |
| RP11-793I11.1 | -0.968474429 | 0.067207387 | 0.151726686 | down |
| RSPH14 | -0.96833 | 0.037135102 | 0.098246477 | down |
| FGF18 | -0.968327817 | 0.028710562 | 0.08128619 | down |
| RP11-397G5.2 | -0.968291451 | 0.654457499 | | down |
| LINC01185 | -0.96825889 | 0.242093943 | 0.376123199 | down |
| AC108004.3 | -0.968085552 | 0.507659583 | | down |
| RP11-481A20.10 | -0.968002372 | 0.585887238 | | down |
| RN7SL679P | -0.967950584 | 0.655907953 | | down |
| CTD-2583A14.8 | -0.967653375 | 3.35E-05 | 0.0003828 | down |
| NA | -0.967312003 | 0.02965866 | 0.083144982 | down |
| CROT | -0.967305065 | 4.45E-07 | 1.01E-05 | down |
| RP11-431K24.3 | -0.967291388 | 0.589351755 | | down |
| ESRG | -0.967290856 | 0.740822438 | | down |
| RNU5A-3P | -0.967179791 | 0.611724609 | 0.723279115 | down |
| IRF6 | -0.967135552 | 3.90E-05 | 0.000435663 | down |
| CFP | -0.966817759 | 0.009488913 | 0.035204358 | down |
| RP4-646N3.1 | -0.966808394 | 0.468782041 | | down |
| RP11-613M10.9 | -0.966762305 | 0.420093798 | | down |
| MOCS1 | -0.966757293 | 7.63E-06 | 0.000112875 | down |
| TMEM95 | -0.966724507 | 0.064529445 | 0.147263399 | down |

| | | | | |
|---|---|---|---|---|
| DUSP13 | -0.966422847 | 0.391694951 | 0.529283219 | down |
| SERBP1P1 | -0.966383593 | 0.014324468 | 0.048234953 | down |
| FRMD6-AS1 | -0.966113178 | 0.057313215 | 0.135114214 | down |
| RP11-252K23.2 | -0.966083887 | 0.04768929 | 0.118137948 | down |
| FBXL19-AS1 | -0.966079467 | 0.001029076 | 0.00620474 | down |
| GPAT3 | -0.965999623 | 0.00662217 | 0.026856974 | down |
| RP11-96K19.5 | -0.965681999 | 0.105671841 | 0.210238961 | down |
| NA | -0.965597598 | 0.127352031 | 0.239719593 | down |
| CD320 | -0.965310517 | 0.001646935 | 0.00899155 | down |
| AC005578.3 | -0.965285063 | 0.242797902 | 0.376710398 | down |
| ATP1A4 | -0.965282441 | 0.079545236 | 0.171426517 | down |
| RP5-997D24.3 | -0.965258495 | 0.339629021 | | down |
| RP5-827C21.1 | -0.965194607 | 0.147661218 | 0.266206639 | down |
| RP11-320G24.1 | -0.965016679 | 0.172536024 | 0.296805239 | down |
| NOC2LP1 | -0.964963865 | 0.020927227 | 0.064222433 | down |
| CCT8P1 | -0.964786009 | 0.00127099 | 0.007325076 | down |
| CTA-331F8.1 | -0.964657732 | 0.554981518 | | down |
| RNA5SP378 | -0.964599616 | 0.422414635 | 0.557945366 | down |
| MTCO2P4 | -0.964580663 | 0.74155939 | | down |
| RP11-462B18.2 | -0.964418185 | 0.425287666 | | down |
| ZNF575 | -0.964019817 | 6.24E-07 | 1.37E-05 | down |
| ISLR2 | -0.963981876 | 0.007429846 | 0.029290419 | down |
| KB-1572G7.4 | -0.963949158 | 0.506108603 | | down |
| PRR22 | -0.96391246 | 0.001637004 | 0.008948785 | down |
| ADAD2 | -0.96365065 | 0.048795174 | 0.120252826 | down |
| RP1-178F10.1 | -0.963367728 | 0.025882058 | 0.075210423 | down |
| CTD-2310F14.1 | -0.963331864 | 0.040102192 | 0.104142991 | down |
| RN7SL182P | -0.963294012 | 0.534723277 | | down |
| RP11-354E11.2 | -0.962977417 | 0.004930262 | 0.02146425 | down |
| NA | -0.962967279 | 6.34E-05 | 0.000649337 | down |
| CTC-484M2.1 | -0.962952007 | 0.30292077 | 0.440852142 | down |
| RP11-91J3.3 | -0.962845728 | 0.543763511 | | down |
| STYK1 | -0.962827892 | 0.001366633 | 0.007751393 | down |
| AC006159.5 | -0.962793644 | 0.589256241 | | down |
| RP11-366M4.1 | -0.962320765 | 0.037005493 | 0.097963964 | down |
| PGAM1P5 | -0.962259844 | 0.396566939 | | down |
| NA | -0.961968404 | 0.272672712 | 0.408893385 | down |
| RP11-60E8.2 | -0.961815776 | 0.368779603 | | down |
| BDH1 | -0.9614971 | 9.47E-06 | 0.000134962 | down |
| CRACR2B | -0.961479963 | 0.001365291 | 0.007746704 | down |
| MRPL41 | -0.961373047 | 0.00036886 | 0.002741716 | down |
| NA | -0.96115536 | 0.227980737 | 0.360194218 | down |
| SRRM3 | -0.96115334 | 0.000859536 | 0.005367681 | down |
| HOXA6 | -0.9611178 | 0.133073941 | 0.247237245 | down |
| ZDHHC19 | -0.960997025 | 0.011958063 | 0.042103369 | down |
| U4 | -0.960941456 | 0.285312641 | | down |
| AF131217.1 | -0.960813437 | 0.01722171 | 0.055373402 | down |
| RP11-203J24.8 | -0.960533071 | 0.030725739 | 0.085324346 | down |
| AC009120.3 | -0.960059208 | 0.059384419 | 0.13864379 | down |
| CTD-3037G24.4 | -0.960054007 | 0.446225891 | | down |
| NA | -0.959957307 | 0.53002587 | | down |

| | | | | |
|---|---|---|---|---|
| RP3-453I5.2 | -0.959877636 | 0.622324261 | | down |
| BMPER | -0.959857821 | 0.00539689 | 0.02301223 | down |
| HEBP1 | -0.959711608 | 0.000179624 | 0.001532262 | down |
| HECW1 | -0.959693844 | 0.066378754 | 0.150302917 | down |
| CTD-3105H18.7 | -0.959628237 | 0.479593327 | | down |
| ALDH7A1P3 | -0.959486019 | 0.560675829 | | down |
| MTND4P29 | -0.959163081 | 0.736008129 | | down |
| RP11-432I5.4 | -0.959163063 | 0.737227439 | | down |
| KRT8P17 | -0.959163041 | 0.738638051 | | down |
| NA | -0.959163026 | 0.739602509 | | down |
| RP11-184I16.4 | -0.95916297 | 0.743044745 | | down |
| ST13P21 | -0.95916297 | 0.743044745 | | down |
| RP11-350G8.4 | -0.95916297 | 0.743044745 | | down |
| RP11-331H2.4 | -0.95916297 | 0.743044745 | | down |
| RP11-22M7.2 | -0.95916297 | 0.743044745 | | down |
| AL672296.1 | -0.95916297 | 0.743044745 | | down |
| AC113607.3 | -0.95916297 | 0.743044745 | | down |
| NA | -0.95916297 | 0.743044745 | | down |
| AC017083.2 | -0.95916297 | 0.743044745 | | down |
| AC092568.1 | -0.95916297 | 0.743044745 | | down |
| NA | -0.95916297 | 0.743044745 | | down |
| RPL30P2 | -0.95916297 | 0.743044745 | | down |
| RP11-572N21.1 | -0.95916297 | 0.743044745 | | down |
| AC114812.9 | -0.95916297 | 0.743044745 | | down |
| RP11-424N24.2 | -0.95916297 | 0.743044745 | | down |
| RPS3AP15 | -0.95916297 | 0.743044745 | | down |
| RP11-469L4.1 | -0.95916297 | 0.743044745 | | down |
| CLRN1-AS1 | -0.95916297 | 0.743044745 | | down |
| DYNLL1P5 | -0.95916297 | 0.743044745 | | down |
| TMEM207 | -0.95916297 | 0.743044745 | | down |
| RNU6-836P | -0.95916297 | 0.743044745 | | down |
| RP11-1267H10.2 | -0.95916297 | 0.743044745 | | down |
| RP11-767N15.1 | -0.95916297 | 0.743044745 | | down |
| RP11-501E14.1 | -0.95916297 | 0.743044745 | | down |
| RP11-63B13.1 | -0.95916297 | 0.743044745 | | down |
| NA | -0.95916297 | 0.743044745 | | down |
| RP11-24P24.1 | -0.95916297 | 0.743044745 | | down |
| CTD-2244C20.1 | -0.95916297 | 0.743044745 | | down |
| RP11-1174L13.2 | -0.95916297 | 0.743044745 | | down |
| RN7SL378P | -0.95916297 | 0.743044745 | | down |
| NA | -0.95916297 | 0.743044745 | | down |
| RP11-619L12.4 | -0.95916297 | 0.743044745 | | down |
| MRPL48P1 | -0.95916297 | 0.743044745 | | down |
| RP11-191A15.1 | -0.95916297 | 0.743044745 | | down |
| OR2W4P | -0.95916297 | 0.743044745 | | down |
| XXbac-BPG13B8.1 | -0.95916297 | 0.743044745 | | down |
| HCG19P | -0.95916297 | 0.743044745 | | down |
| NA | -0.95916297 | 0.743044745 | | down |
| RP11-252P19.2 | -0.95916297 | 0.743044745 | | down |
| AC013436.6 | -0.95916297 | 0.743044745 | | down |
| CDC14C | -0.95916297 | 0.743044745 | | down |

| | | | |
|---|---|---|---|
| NA | -0.95916297 | 0.743044745 | down |
| NA | -0.95916297 | 0.743044745 | down |
| MTCYBP29 | -0.95916297 | 0.743044745 | down |
| RP11-548K12.11 | -0.95916297 | 0.743044745 | down |
| CTD-2526L21.2 | -0.95916297 | 0.743044745 | down |
| RP5-1106H14.1 | -0.95916297 | 0.743044745 | down |
| RP11-115J16.3 | -0.95916297 | 0.743044745 | down |
| RPL12P48 | -0.95916297 | 0.743044745 | down |
| KB-1582A10.2 | -0.95916297 | 0.743044745 | down |
| RNU6-656P | -0.95916297 | 0.743044745 | down |
| RP11-628E19.2 | -0.95916297 | 0.743044745 | down |
| RP11-324F11.1 | -0.95916297 | 0.743044745 | down |
| RNU6-694P | -0.95916297 | 0.743044745 | down |
| TUBB4BP2 | -0.95916297 | 0.743044745 | down |
| RBM17P1 | -0.95916297 | 0.743044745 | down |
| RBPJP5 | -0.95916297 | 0.743044745 | down |
| OR13I1P | -0.95916297 | 0.743044745 | down |
| LCN1P2 | -0.95916297 | 0.743044745 | down |
| RP11-98L5.5 | -0.95916297 | 0.743044745 | down |
| ATP6V1G1P3 | -0.95916297 | 0.743044745 | down |
| RP11-402L1.11 | -0.95916297 | 0.743044745 | down |
| RP11-186O14.7 | -0.95916297 | 0.743044745 | down |
| PHB2P1 | -0.95916297 | 0.743044745 | down |
| OR10A6 | -0.95916297 | 0.743044745 | down |
| MRGPRX4 | -0.95916297 | 0.743044745 | down |
| OR2BH1P | -0.95916297 | 0.743044745 | down |
| Y_RNA | -0.95916297 | 0.743044745 | down |
| RP11-698N11.2 | -0.95916297 | 0.743044745 | down |
| RP11-40H19.1 | -0.95916297 | 0.743044745 | down |
| RP11-58K22.2 | -0.95916297 | 0.743044745 | down |
| NA | -0.95916297 | 0.743044745 | down |
| OR5G4P | -0.95916297 | 0.743044745 | down |
| OR6Q1 | -0.95916297 | 0.743044745 | down |
| RP11-697H9.5 | -0.95916297 | 0.743044745 | down |
| RP11-748H22.2 | -0.95916297 | 0.743044745 | down |
| RP11-136I14.3 | -0.95916297 | 0.743044745 | down |
| AP000711.1 | -0.95916297 | 0.743044745 | down |
| OR8B8 | -0.95916297 | 0.743044745 | down |
| RP11-688I9.2 | -0.95916297 | 0.743044745 | down |
| OPCML-IT2 | -0.95916297 | 0.743044745 | down |
| SNORA74 | -0.95916297 | 0.743044745 | down |
| Y_RNA | -0.95916297 | 0.743044745 | down |
| RP11-528M18.2 | -0.95916297 | 0.743044745 | down |
| RP11-753B7.2 | -0.95916297 | 0.743044745 | down |
| LINC00415 | -0.95916297 | 0.743044745 | down |
| NA | -0.95916297 | 0.743044745 | down |
| TOMM22P3 | -0.95916297 | 0.743044745 | down |
| RP11-189B4.7 | -0.95916297 | 0.743044745 | down |
| TXNL1P1 | -0.95916297 | 0.743044745 | down |
| RPS6P23 | -0.95916297 | 0.743044745 | down |
| RP11-65D24.1 | -0.95916297 | 0.743044745 | down |

| | | | | |
|---|---|---|---|---|
| FOXG1-AS1 | -0.95916297 | 0.743044745 | | down |
| AC090696.2 | -0.95916297 | 0.743044745 | | down |
| RP11-402F9.3 | -0.95916297 | 0.743044745 | | down |
| RP11-507J18.5 | -0.95916297 | 0.743044745 | | down |
| LINGO1-AS1 | -0.95916297 | 0.743044745 | | down |
| VN1R66P | -0.95916297 | 0.743044745 | | down |
| RP11-586K12.2 | -0.95916297 | 0.743044745 | | down |
| RP11-22D3.2 | -0.95916297 | 0.743044745 | | down |
| RP11-351A20.2 | -0.95916297 | 0.743044745 | | down |
| RP11-558A11.3 | -0.95916297 | 0.743044745 | | down |
| NA | -0.95916297 | 0.743044745 | | down |
| RP11-434D2.3 | -0.95916297 | 0.743044745 | | down |
| VN1R71P | -0.95916297 | 0.743044745 | | down |
| RP11-28A22.2 | -0.95916297 | 0.743044745 | | down |
| NA | -0.95916297 | 0.743044745 | | down |
| CTD-2319I12.3 | -0.95916297 | 0.743044745 | | down |
| RP11-180P8.4 | -0.95916297 | 0.743044745 | | down |
| RP11-14P20.1 | -0.95916297 | 0.743044745 | | down |
| RNU6-340P | -0.95916297 | 0.743044745 | | down |
| RP11-128P17.1 | -0.95916297 | 0.743044745 | | down |
| RP11-64C12.5 | -0.95916297 | 0.743044745 | | down |
| RP11-636O21.1 | -0.95916297 | 0.743044745 | | down |
| RP11-529J17.1 | -0.95916297 | 0.743044745 | | down |
| CCND3P2 | -0.95916297 | 0.743044745 | | down |
| MIR7-3 | -0.95916297 | 0.743044745 | | down |
| OR7A1P | -0.95916297 | 0.743044745 | | down |
| CTC-461H2.3 | -0.95916297 | 0.743044745 | | down |
| AC006133.7 | -0.95916297 | 0.743044745 | | down |
| AC008984.5 | -0.95916297 | 0.743044745 | | down |
| RP5-1177M21.1 | -0.95916297 | 0.743044745 | | down |
| LINC01432 | -0.95916297 | 0.743044745 | | down |
| XXyac-YX60D10.1 | -0.95916297 | 0.743044745 | | down |
| RPL31P3 | -0.95916297 | 0.743044745 | | down |
| SNORA40 | -0.95916297 | 0.743044745 | | down |
| NA | -0.95916297 | 0.743044745 | | down |
| Y_RNA | -0.95916297 | 0.743044745 | | down |
| RP1-231P7P.1 | -0.95916297 | 0.743044745 | | down |
| RP1-302D9.5 | -0.95916297 | 0.743044745 | | down |
| RP1-288L1.5 | -0.95916297 | 0.743044745 | | down |
| Y_RNA | -0.95916297 | 0.743044745 | | down |
| DRAXINP1 | -0.95916297 | 0.743044745 | | down |
| RN7SL658P | -0.95916297 | 0.743044745 | | down |
| SNORA11C | -0.95916297 | 0.743044745 | | down |
| RP3-393P12.1 | -0.95916297 | 0.743044745 | | down |
| RP11-552J9.9 | -0.95916297 | 0.743044745 | | down |
| MEMO1P4 | -0.95916297 | 0.743044745 | | down |
| OR5AW1P | -0.95916297 | 0.743044745 | | down |
| RNU6-616P | -0.95916297 | 0.743044745 | | down |
| RP11-635O16.1 | -0.95916297 | 0.743044745 | | down |
| NA | -0.958965509 | 0.126618959 | 0.238848497 | down |
| PCDHB17P | -0.958934416 | 0.531084129 | | down |

| | | | | |
|---|---|---|---|---|
| RP11-950C14.7 | -0.958498722 | 0.25707143 | | down |
| SCN3A | -0.958048673 | 0.004277486 | 0.019203942 | down |
| CAGE1 | -0.957786477 | 0.182671264 | 0.308411097 | down |
| RP11-67M9.1 | -0.957694434 | 0.548192092 | 0.668898504 | down |
| NDUFA7 | -0.957427915 | 0.018399241 | 0.058353931 | down |
| LIPH | -0.957400704 | 0.004775868 | 0.020959125 | down |
| RNF144A-AS1 | -0.957394919 | 0.01341712 | 0.04583852 | down |
| RPL7P32 | -0.957295388 | 0.016810874 | 0.05436662 | down |
| RPL18AP16 | -0.957245592 | 0.287963893 | | down |
| RNU6ATAC18P | -0.957234568 | 0.256446298 | | down |
| RP5-958B11.1 | -0.957021698 | 0.598264791 | | down |
| RNF152 | -0.956924936 | 0.000889053 | 0.005508498 | down |
| MAP3K9 | -0.956814239 | 0.000100406 | 0.00094832 | down |
| CNPY1 | -0.956256338 | 0.599494661 | | down |
| RP11-613M5.1 | -0.95623112 | 0.39796407 | | down |
| NA | -0.955996419 | 0.277917909 | | down |
| CYCSP55 | -0.95596301 | 0.231679167 | 0.364351386 | down |
| MAN1B1 | -0.955756418 | 0.000177 | 0.001512883 | down |
| SLC16A1-AS1 | -0.955674838 | 0.001405044 | 0.007919916 | down |
| BTNL10 | -0.955666754 | 0.517684023 | | down |
| KRBOX1 | -0.955570074 | 0.204868541 | 0.334587435 | down |
| DOK7 | -0.955438288 | 0.020955752 | 0.064270582 | down |
| RP11-295G20.2 | -0.955182347 | 0.003262807 | 0.015464946 | down |
| RP11-108L7.14 | -0.954970046 | 0.075812328 | 0.165567026 | down |
| APOPT1 | -0.954963242 | 0.000290798 | 0.002274601 | down |
| PLCE1 | -0.954896576 | 2.81E-07 | 6.89E-06 | down |
| RP11-315I20.1 | -0.954822549 | 0.006154555 | 0.025444812 | down |
| AC093901.1 | -0.954732843 | 0.061124794 | 0.14173291 | down |
| FAR1-IT1 | -0.954732671 | 0.31913655 | | down |
| SLC16A1 | -0.954718159 | 5.02E-05 | 0.000537559 | down |
| RP11-334J6.6 | -0.954699277 | 0.032941257 | 0.089865353 | down |
| RP11-15F12.1 | -0.954515336 | 0.083553043 | 0.177768403 | down |
| ARMC3 | -0.954362832 | 0.296357988 | 0.433787612 | down |
| GAPDHP66 | -0.954158963 | 0.541354658 | 0.662962514 | down |
| GRAMD3 | -0.954137506 | 1.02E-06 | 2.07E-05 | down |
| RP5-890E16.5 | -0.95406456 | 0.027268989 | 0.078241764 | down |
| RP11-872J21.3 | -0.953987879 | 0.007112735 | 0.028322731 | down |
| CHMP1A | -0.953813689 | 3.70E-07 | 8.65E-06 | down |
| CARD14 | -0.953780016 | 0.002039848 | 0.010665717 | down |
| CTC-471J1.9 | -0.953761988 | 0.317553893 | | down |
| NA | -0.953699384 | 0.190514685 | 0.318025513 | down |
| RNU6-761P | -0.953672305 | 0.483616014 | | down |
| RP11-640L9.1 | -0.953601351 | 0.021032296 | 0.064446129 | down |
| RP11-195C7.3 | -0.953478931 | 0.342614372 | | down |
| SEMA3D | -0.953285592 | 0.010109101 | 0.036980132 | down |
| NA | -0.953115348 | 0.24648301 | 0.380633783 | down |
| PECR | -0.953040043 | 6.27E-06 | 9.61E-05 | down |
| FOLR1 | -0.953015885 | 0.080994496 | 0.17381159 | down |
| CTD-2619J13.8 | -0.952997425 | 0.013414295 | 0.045834081 | down |
| RNU6-610P | -0.952965696 | 0.085108199 | 0.180222071 | down |
| RN7SL79P | -0.952964841 | 0.695470989 | 0.790239793 | down |

| | | | | |
|---|---|---|---|---|
| RP11-745C15.2 | -0.952800247 | 0.375390388 | 0.513713803 | down |
| PSPC1P1 | -0.9527679 | 0.147399971 | 0.265799455 | down |
| NA | -0.952666868 | 0.354059816 | | down |
| CRHBP | -0.952558709 | 0.006819417 | 0.02745679 | down |
| RP11-161H23.9 | -0.952427378 | 0.239795885 | 0.373499278 | down |
| RHPN1-AS1 | -0.952406364 | 0.082677171 | 0.176529949 | down |
| GTF2IRD2B | -0.95212275 | 2.50E-08 | 8.56E-07 | down |
| CCDC68 | -0.952075786 | 0.001227036 | 0.007134715 | down |
| RHD | -0.951969065 | 0.038989007 | 0.101948278 | down |
| OR13J1 | -0.95191361 | 0.477778188 | | down |
| AC079305.10 | -0.95168803 | 0.008771855 | 0.033174925 | down |
| KLC4 | -0.951661732 | 1.14E-08 | 4.45E-07 | down |
| LPIN3 | -0.951578392 | 0.000493508 | 0.003439912 | down |
| CCDC144B | -0.951472076 | 0.056533541 | 0.133813417 | down |
| AP000568.2 | -0.95144101 | 0.152487029 | 0.271953004 | down |
| ARHGAP32 | -0.951154052 | 0.000299961 | 0.002329892 | down |
| ZDHHC8P1 | -0.950996745 | 0.009712714 | 0.035826737 | down |
| RP1-102E24.1 | -0.950988314 | 0.155729115 | 0.275982158 | down |
| TTN | -0.950982255 | 0.006349691 | 0.026050816 | down |
| XKR4 | -0.950860232 | 0.003850456 | 0.01768855 | down |
| RP11-104F15.9 | -0.950847778 | 0.065526534 | 0.148877597 | down |
| TMPRSS2 | -0.950833322 | 0.009648562 | 0.035646999 | down |
| MOV10L1 | -0.95076149 | 0.014440267 | 0.048495795 | down |
| CTD-2526A2.2 | -0.950741038 | 0.091169891 | 0.189207274 | down |
| RP11-411B10.8 | -0.950699291 | 0.322217005 | | down |
| SNX6P1 | -0.950362276 | 0.538240712 | 0.660360338 | down |
| AC003956.1 | -0.950298676 | 0.149665718 | 0.268614675 | down |
| LINC00526 | -0.950278972 | 6.45E-05 | 0.000656601 | down |
| ABHD17C | -0.950167025 | 0.00012231 | 0.001119991 | down |
| SERPINB6 | -0.950110879 | 0.000140821 | 0.001255065 | down |
| SLC19A1 | -0.950063409 | 0.000126067 | 0.00114566 | down |
| STX18-AS1 | -0.949998588 | 2.78E-05 | 0.000328765 | down |
| CTC-463A16.1 | -0.949877741 | 0.014783755 | 0.049395365 | down |
| KLB | -0.949862341 | 0.016111872 | 0.052707434 | down |
| Y_RNA | -0.949755145 | 0.626953356 | | down |
| CTD-2651B20.2 | -0.94975486 | 0.668510677 | | down |
| RP11-973N13.3 | -0.949754498 | 0.705624192 | | down |
| RNA5SP92 | -0.949754158 | 0.731127209 | | down |
| RP5-1073F15.1 | -0.949754056 | 0.737576755 | | down |
| RPS15P9 | -0.949754033 | 0.738951393 | | down |
| RP11-643A5.3 | -0.949754015 | 0.74003012 | | down |
| CTD-2114J12.1 | -0.949753994 | 0.741265623 | | down |
| AL450226.2 | -0.949753992 | 0.741379376 | | down |
| CTD-2196E14.5 | -0.949753989 | 0.741563591 | | down |
| PMCHL2 | -0.949753974 | 0.74242024 | | down |
| RP11-331F4.1 | -0.949753962 | 0.74309162 | | down |
| NPPB | -0.94975392 | 0.745477654 | | down |
| RP5-1092L12.2 | -0.94975392 | 0.745477654 | | down |
| AC113607.2 | -0.94975392 | 0.745477654 | | down |
| AC010904.1 | -0.94975392 | 0.745477654 | | down |
| AC012506.4 | -0.94975392 | 0.745477654 | | down |

| | | | | |
|---|---|---|---|---|
| RP11-575M22.1 | -0.94975392 | 0.745477654 | | down |
| AC017048.2 | -0.94975392 | 0.745477654 | | down |
| RPSAP27 | -0.94975392 | 0.745477654 | | down |
| RP11-572C15.3 | -0.94975392 | 0.745477654 | | down |
| RPL23AP93 | -0.94975392 | 0.745477654 | | down |
| RP11-472K22.1 | -0.94975392 | 0.745477654 | | down |
| RNU1-138P | -0.94975392 | 0.745477654 | | down |
| RP11-93I21.2 | -0.94975392 | 0.745477654 | | down |
| MIR4637 | -0.94975392 | 0.745477654 | | down |
| RP11-451H23.3 | -0.94975392 | 0.745477654 | | down |
| RP11-404H14.1 | -0.94975392 | 0.745477654 | | down |
| NA | -0.94975392 | 0.745477654 | | down |
| AC004901.1 | -0.94975392 | 0.745477654 | | down |
| RP11-535E8.2 | -0.94975392 | 0.745477654 | | down |
| TRBV12-1 | -0.94975392 | 0.745477654 | | down |
| L09NC01-254D11. | -0.94975392 | 0.745477654 | | down |
| RNU6-946P | -0.94975392 | 0.745477654 | | down |
| RNA5SP309 | -0.94975392 | 0.745477654 | | down |
| RP11-219F10.1 | -0.94975392 | 0.745477654 | | down |
| RP11-400G3.3 | -0.94975392 | 0.745477654 | | down |
| RPS3AP39 | -0.94975392 | 0.745477654 | | down |
| UBQLN3 | -0.94975392 | 0.745477654 | | down |
| CACNA1C-AS3 | -0.94975392 | 0.745477654 | | down |
| NA | -0.94975392 | 0.745477654 | | down |
| LINC00554 | -0.94975392 | 0.745477654 | | down |
| RP11-84C10.3 | -0.94975392 | 0.745477654 | | down |
| SNORA79 | -0.94975392 | 0.745477654 | | down |
| RP11-109M19.1 | -0.94975392 | 0.745477654 | | down |
| KRT16P3 | -0.94975392 | 0.745477654 | | down |
| STH | -0.94975392 | 0.745477654 | | down |
| U8 | -0.94975392 | 0.745477654 | | down |
| MAN1A2P1 | -0.94975392 | 0.745477654 | | down |
| AP001607.1 | -0.94975392 | 0.745477654 | | down |
| OR7E23P | -0.94975392 | 0.745477654 | | down |
| IGLVIV-64 | -0.94975392 | 0.745477654 | | down |
| CITF22-24E5.1 | -0.94975392 | 0.745477654 | | down |
| HTR7 | -0.94973367 | 0.012249914 | 0.042819826 | down |
| FAM188B | -0.949500664 | 0.019923682 | 0.061901459 | down |
| CTC-251D13.1 | -0.949452329 | 0.074279842 | 0.163181016 | down |
| AC015849.2 | -0.949389448 | 0.140899666 | 0.25747762 | down |
| ADAMTS16 | -0.949318098 | 0.046481165 | 0.115871235 | down |
| RP11-68L18.2 | -0.949237948 | 0.619226187 | 0.729569865 | down |
| HIST3H2BB | -0.949180801 | 0.048885396 | 0.120396166 | down |
| BIRC7 | -0.949151361 | 0.021732414 | 0.066005726 | down |
| RP11-757A13.1 | -0.948982 | 0.454010102 | | down |
| SPR | -0.948847829 | 2.28E-05 | 0.000278227 | down |
| AC008522.1 | -0.948751977 | 0.364400727 | 0.502757873 | down |
| OR5AC2 | -0.948652016 | 0.745713516 | | down |
| CTA-113A6.1 | -0.948644822 | 0.454755915 | | down |
| NA | -0.94848177 | 0.052493801 | 0.126595261 | down |
| LINC00308 | -0.94817336 | 0.728887339 | | down |

| | | | | |
|---|---|---|---|---|
| C6orf15 | -0.948124184 | 0.629447338 | | down |
| RP5-862P8.2 | -0.948037257 | 0.000922764 | 0.005691544 | down |
| C2orf50 | -0.948004349 | 0.037748902 | 0.099502371 | down |
| RP4-536B24.4 | -0.947970812 | 0.112327571 | 0.219464516 | down |
| RP11-834C11.11 | -0.947963754 | 0.354974496 | | down |
| ZNF827 | -0.947941852 | 6.59E-08 | 2.01E-06 | down |
| AQP6 | -0.947704177 | 0.167050018 | 0.290241194 | down |
| AC073130.1 | -0.947695651 | 0.474457574 | | down |
| RP11-151A10.3 | -0.947678323 | 0.232143761 | 0.364795604 | down |
| OR10T1P | -0.947598542 | 0.730759582 | | down |
| PCDH8 | -0.947194554 | 0.180711362 | 0.306065288 | down |
| AP006216.10 | -0.947141216 | 0.564885547 | | down |
| RNU5F-1 | -0.94695158 | 0.095979037 | 0.196296404 | down |
| RPL14P3 | -0.946897456 | 0.058318991 | 0.136939099 | down |
| MZF1-AS1 | -0.946634305 | 5.20E-05 | 0.000553174 | down |
| BX470102.3 | -0.946519685 | 0.012779257 | 0.044193731 | down |
| FOXA3 | -0.946205261 | 0.001155724 | 0.006805677 | down |
| RHOA-IT1 | -0.946179664 | 0.100717944 | 0.203003483 | down |
| XX-C2158C6.1 | -0.945915752 | 0.386208499 | | down |
| PAK3 | -0.945745318 | 0.006369904 | 0.026114353 | down |
| NA | -0.945724035 | 0.279727385 | 0.416025422 | down |
| SEPHS2 | -0.945451621 | 2.67E-07 | 6.61E-06 | down |
| ARHGEF37 | -0.945430853 | 1.46E-07 | 3.94E-06 | down |
| SLC25A21-AS1 | -0.945413918 | 0.022457421 | 0.06761338 | down |
| RP11-379K17.4 | -0.945381535 | 0.120053627 | 0.23023847 | down |
| CIB2 | -0.944970047 | 0.000208985 | 0.001733067 | down |
| NA | -0.944894483 | 0.251309725 | 0.386068894 | down |
| SLC13A3 | -0.944729135 | 0.025737584 | 0.07489457 | down |
| RP3-375P9.2 | -0.944711941 | 0.00735678 | 0.029051933 | down |
| RP11-285E9.5 | -0.944662602 | 0.413549885 | 0.550130297 | down |
| GOLGA6L10 | -0.944246643 | 0.031104189 | 0.08612397 | down |
| RP11-73M7.1 | -0.944164717 | 0.011390928 | 0.04056769 | down |
| ROGDI | -0.944093046 | 3.03E-06 | 5.23E-05 | down |
| LIPE-AS1 | -0.94402383 | 0.020884221 | 0.064116649 | down |
| CTB-25B13.5 | -0.944016443 | 0.071986367 | 0.159566062 | down |
| KCNH4 | -0.943979935 | 0.009329274 | 0.034779589 | down |
| BRWD1-AS2 | -0.943910906 | 0.002528975 | 0.012686681 | down |
| RP11-392O18.2 | -0.943666306 | 0.391883829 | | down |
| LINC01191 | -0.943391673 | 0.037507654 | 0.099031544 | down |
| NA | -0.943389368 | 0.02250985 | 0.067738219 | down |
| NA | -0.943341459 | 0.049123152 | 0.120813358 | down |
| RP11-578F21.2 | -0.943272273 | 0.411056089 | | down |
| ADAMTS18 | -0.943235435 | 0.159348973 | 0.28051096 | down |
| KRTAP5-10 | -0.943210155 | 0.069268642 | 0.155132843 | down |
| SPG20-AS1 | -0.943152391 | 0.149499667 | 0.268441788 | down |
| ST8SIA6-AS1 | -0.943017391 | 0.124743961 | 0.236393861 | down |
| AC025165.8 | -0.942802645 | 0.18535403 | 0.311522515 | down |
| BCDIN3D-AS1 | -0.942638148 | 0.038968628 | 0.101912732 | down |
| IGF2BP3 | -0.942404454 | 0.098744062 | 0.200247493 | down |
| MED18 | -0.942309575 | 4.75E-05 | 0.000512432 | down |
| RP11-92C4.3 | -0.942236529 | 0.276799701 | 0.41300011 | down |

| | | | | |
|---|---|---|---|---|
| GBP7 | -0.941962113 | 0.305833218 | | down |
| WWTR1-AS1 | -0.941889405 | 0.075833682 | 0.165589588 | down |
| RP4-61404.11 | -0.941779206 | 0.156613814 | 0.277135088 | down |
| NA | -0.941735347 | 0.343128545 | 0.481501822 | down |
| CCDC42 | -0.941702621 | 0.138857503 | 0.254846354 | down |
| RP11-206L10.9 | -0.941603777 | 0.001766104 | 0.009505925 | down |
| CASC8 | -0.94151825 | 0.077137629 | 0.167681277 | down |
| RP11-83B20.1 | -0.941382703 | 0.127593605 | 0.240069031 | down |
| LINC01106 | -0.941063775 | 0.001654945 | 0.009022661 | down |
| REV3L-IT1 | -0.941023938 | 0.278751657 | 0.415015575 | down |
| AC136289.1 | -0.941005224 | 0.168964854 | 0.292403265 | down |
| SPINK1 | -0.940955247 | 0.015734973 | 0.051805141 | down |
| CTB-35F21.2 | -0.940917667 | 0.69476266 | | down |
| LGMNP1 | -0.940888274 | 0.449049791 | | down |
| SLC16A12 | -0.940704209 | 0.061543049 | 0.142449873 | down |
| FDXR | -0.940509608 | 0.000291409 | 0.002277598 | down |
| RP11-781P6.1 | -0.940345973 | 0.660802205 | | down |
| RPL35AP21 | -0.940345927 | 0.666484941 | | down |
| NA | -0.940345611 | 0.698522835 | | down |
| RP11-211G23.1 | -0.940345583 | 0.700933888 | | down |
| STK24P1 | -0.940345554 | 0.703405019 | | down |
| CTC-499B15.7 | -0.940345533 | 0.705136552 | | down |
| RP11-295P9.2 | -0.940345106 | 0.73491219 | | down |
| NA | -0.940345051 | 0.738098421 | | down |
| RP13-577H12.2 | -0.940345041 | 0.738712136 | | down |
| RNU7-125P | -0.940345016 | 0.740095019 | | down |
| RNA5SP440 | -0.940344995 | 0.741272995 | | down |
| RP11-597A11.4 | -0.940344985 | 0.7418379 | | down |
| RP11-222G7.2 | -0.940344974 | 0.742447511 | | down |
| NA | -0.940344973 | 0.742469052 | | down |
| RP11-415A20.1 | -0.94034487 | 0.747913103 | | down |
| RP11-202K23.1 | -0.94034487 | 0.747913103 | | down |
| AC108059.2 | -0.94034487 | 0.747913103 | | down |
| AC016772.4 | -0.94034487 | 0.747913103 | | down |
| AC097713.3 | -0.94034487 | 0.747913103 | | down |
| RP11-285B24.1 | -0.94034487 | 0.747913103 | | down |
| NA | -0.94034487 | 0.747913103 | | down |
| RP11-692C23.1 | -0.94034487 | 0.747913103 | | down |
| MIR4655 | -0.94034487 | 0.747913103 | | down |
| AC002383.2 | -0.94034487 | 0.747913103 | | down |
| RN7SL306P | -0.94034487 | 0.747913103 | | down |
| TRIM51 | -0.94034487 | 0.747913103 | | down |
| RP11-467D10.2 | -0.94034487 | 0.747913103 | | down |
| RNU6ATAC25P | -0.94034487 | 0.747913103 | | down |
| KRTAP10-6 | -0.94034487 | 0.747913103 | | down |
| RP11-403E24.1 | -0.94034487 | 0.747913103 | | down |
| RN7SL684P | -0.940217132 | 0.15465462 | 0.27454759 | down |
| LIPC | -0.940098023 | 0.001442404 | 0.008083492 | down |
| SPIC | -0.94003132 | 0.114042235 | 0.221852169 | down |
| JAML | -0.939855558 | 0.000456745 | 0.003237952 | down |
| LINC01571 | -0.939780262 | 0.073975466 | 0.162706504 | down |

| | | | | |
|---|---|---|---|---|
| NA | -0.939774258 | 0.172209353 | 0.296496148 | down |
| GOLGA7B | -0.939710944 | 0.000389342 | 0.002853715 | down |
| STK33 | -0.939594684 | 0.001844792 | 0.009842822 | down |
| RP11-192M23.1 | -0.939592962 | 0.40931525 | | down |
| NA | -0.939304316 | 0.210738247 | 0.341333068 | down |
| TTLL8 | -0.939261716 | 0.254261109 | 0.389308977 | down |
| GATA1 | -0.939223279 | 0.018136173 | 0.057726793 | down |
| KCCAT198 | -0.939155933 | 0.515184292 | | down |
| RPSAP69 | -0.939055068 | 0.195955329 | 0.324457787 | down |
| RP11-30K9.5 | -0.939050073 | 0.221779066 | | down |
| PPP1R42 | -0.939014204 | 0.547101165 | | down |
| RP11-136B18.1 | -0.93879282 | 0.726927462 | | down |
| LINC00867 | -0.938638478 | 0.31098825 | 0.449457907 | down |
| TLN2 | -0.938382906 | 2.02E-06 | 3.74E-05 | down |
| RP11-47G11.2 | -0.938280695 | 0.550471331 | | down |
| EPHA5 | -0.938116043 | 0.216740951 | 0.348018017 | down |
| RSL24D1P6 | -0.938005523 | 0.168607801 | 0.292052625 | down |
| RP13-122B23.8 | -0.93771424 | 0.001522011 | 0.008433707 | down |
| PRKCG | -0.937655025 | 0.034146351 | 0.0923838 | down |
| EZR | -0.937509641 | 1.99E-07 | 5.16E-06 | down |
| COX5B | -0.937443187 | 7.34E-06 | 0.000109352 | down |
| JARID2-AS1 | -0.937437699 | 0.041854845 | 0.107560258 | down |
| RP11-1060J15.5 | -0.937378489 | 0.078075171 | 0.169192904 | down |
| RP11-363E6.3 | -0.937201868 | 0.075470816 | 0.165086275 | down |
| KB-1683C8.1 | -0.937166096 | 0.385697874 | | down |
| IFNL1 | -0.937022028 | 0.22319723 | 0.355214407 | down |
| RPL12P42 | -0.936901792 | 0.012068953 | 0.042359768 | down |
| SENP8 | -0.936900903 | 0.00127891 | 0.007356612 | down |
| VEPH1 | -0.936838903 | 0.070400098 | 0.156999744 | down |
| CTD-2655K5.1 | -0.936734156 | 0.183318945 | 0.309087737 | down |
| AC084809.3 | -0.936733219 | 0.086907117 | 0.182909803 | down |
| SLC30A3 | -0.936701673 | 0.039308445 | 0.102578209 | down |
| TRBV12-5 | -0.936031226 | 0.147387642 | 0.265793176 | down |
| FLJ31356 | -0.936018815 | 0.033739031 | 0.09152645 | down |
| AC006277.2 | -0.935712992 | 0.007105993 | 0.028314269 | down |
| RP3-425P12.4 | -0.935441867 | 0.153722999 | 0.273375791 | down |
| ENHO | -0.935385362 | 0.013457932 | 0.045951825 | down |
| Metazoa_SRP | -0.935323666 | 0.49666677 | | down |
| LGALS9 | -0.935307961 | 0.000746059 | 0.004801754 | down |
| CTC-276P9.4 | -0.935268392 | 0.417197159 | | down |
| PTPN2P1 | -0.935204299 | 0.361898379 | | down |
| CPN2 | -0.935204073 | 0.557118716 | | down |
| AC061992.2 | -0.935108904 | 0.04598658 | 0.114962625 | down |
| AC073072.7 | -0.935018568 | 0.011490103 | 0.040816438 | down |
| AL022393.9 | -0.934936688 | 0.075581399 | 0.165206806 | down |
| HMGA1P2 | -0.934667042 | 0.228715347 | | down |
| UCA1 | -0.934638221 | 0.146678498 | 0.264895937 | down |
| ZBTB48 | -0.934508298 | 1.80E-05 | 0.000230411 | down |
| OR52B5P | -0.934363383 | 0.568719933 | | down |
| TP53TG3D | -0.934263727 | 0.266616126 | 0.402300983 | down |
| PQLC1 | -0.933879147 | 6.77E-05 | 0.000683574 | down |

| | | | | |
|---|---|---|---|---|
| SV2C | -0.933757611 | 0.068270602 | 0.153492626 | down |
| RP11-177B4.1 | -0.933388544 | 0.176789486 | 0.301712069 | down |
| AF131216.1 | -0.933240189 | 0.039138611 | 0.102250471 | down |
| AP001439.2 | -0.933191495 | 0.409721509 | | down |
| EFHC2 | -0.933009715 | 0.005916337 | 0.024684132 | down |
| CTD-2529P6.4 | -0.93297332 | 0.54751343 | 0.668352571 | down |
| KCNQ1-AS1 | -0.932953836 | 0.537958728 | | down |
| FAM201B | -0.932776177 | 0.584799051 | | down |
| TMEM191A | -0.932768332 | 0.000213945 | 0.001766836 | down |
| MRPS21P8 | -0.932389997 | 0.696879134 | | down |
| RP11-454F8.2 | -0.932253229 | 0.575239255 | | down |
| IQCD | -0.932183115 | 0.003272793 | 0.015504227 | down |
| RP11-92K15.3 | -0.932116218 | 0.004265004 | 0.019167956 | down |
| AD000091.2 | -0.931948131 | 0.727988878 | | down |
| AC007041.2 | -0.931887825 | 0.325336799 | | down |
| NA | -0.931838289 | 0.750016734 | | down |
| TPRG1L | -0.931752593 | 5.35E-10 | 2.97E-08 | down |
| TSPO2 | -0.931749584 | 0.149669783 | 0.268614675 | down |
| RP1-228H13.1 | -0.931737469 | 0.195551306 | 0.324068313 | down |
| KRT18P23 | -0.931657376 | 0.408605253 | | down |
| LINC01376 | -0.931655959 | 0.037886834 | 0.099761829 | down |
| UNC93B7 | -0.931594306 | 0.377263656 | | down |
| RNU6-636P | -0.931512534 | 0.750201051 | | down |
| AC142293.3 | -0.931512534 | 0.750201051 | | down |
| PIP5K1P2 | -0.931512534 | 0.750201051 | | down |
| RP11-35J23.1 | -0.931512534 | 0.750201051 | | down |
| NA | -0.931512534 | 0.750201051 | | down |
| LALBA | -0.931512534 | 0.750201051 | | down |
| RP11-189E14.4 | -0.931512534 | 0.750201051 | | down |
| RP11-9L18.3 | -0.931511666 | 0.741067516 | | down |
| HSPD1P3 | -0.931511524 | 0.739628571 | | down |
| H1FOO | -0.931511362 | 0.738004445 | | down |
| CTA-215D11.4 | -0.931511306 | 0.737454372 | | down |
| RP11-429P3.5 | -0.931511258 | 0.736978555 | | down |
| SPATA42 | -0.931511113 | 0.735559367 | | down |
| AC114812.10 | -0.93150766 | 0.705057789 | | down |
| MTND3P23 | -0.931507522 | 0.703945574 | | down |
| RP4-682E18.1 | -0.931502431 | 0.666824343 | | down |
| NA | -0.931502376 | 0.666457109 | | down |
| CTB-33018.2 | -0.931502155 | 0.664985836 | | down |
| OR7E41P | -0.931501846 | 0.662948166 | | down |
| RP11-327F22.5 | -0.931497139 | 0.633948154 | | down |
| RP11-368P15.2 | -0.931496678 | 0.631287197 | | down |
| RP11-109N23.1 | -0.931496604 | 0.630863152 | | down |
| BTF3P6 | -0.931489933 | 0.595247972 | | down |
| AC016682.1 | -0.931322123 | 0.750226059 | | down |
| LINC00408 | -0.931322123 | 0.750226059 | | down |
| RP11-333E13.2 | -0.931094422 | 0.279907096 | | down |
| CCDC162P | -0.930987189 | 0.001690153 | 0.009175534 | down |
| RP11-846C15.2 | -0.930984441 | 0.522284276 | 0.646457822 | down |
| PRODH2 | -0.93098391 | 0.276662147 | 0.41287689 | down |

| | | | | |
|---|---|---|---|---|
| SNORD123 | -0.930872833 | 0.322435505 | | down |
| NA | -0.930577933 | 0.024596031 | 0.072409083 | down |
| CMTM4 | -0.930543229 | 0.000373754 | 0.002769193 | down |
| NUTM2B | -0.930170047 | 0.016251037 | 0.053018369 | down |
| NA | -0.930138164 | 0.628669982 | | down |
| LA16c-329F2.1 | -0.930109272 | 0.277386185 | 0.413546574 | down |
| FOXD2 | -0.929853915 | 0.005095548 | 0.022011593 | down |
| CFL1P5 | -0.929484143 | 0.25942926 | | down |
| HIST2H3D | -0.929217323 | 0.058851472 | 0.137790962 | down |
| RGS9 | -0.92915645 | 4.18E-05 | 0.000460065 | down |
| RP11-179B15.6 | -0.929135001 | 0.196445954 | 0.324934285 | down |
| RP11-729I10.3 | -0.929108165 | 0.568522984 | | down |
| LCMT1 | -0.929065239 | 2.34E-06 | 4.22E-05 | down |
| LINC00643 | -0.92906463 | 0.122142872 | 0.232949943 | down |
| RP11-69L16.4 | -0.928695879 | 0.635857069 | | down |
| NA | -0.928603604 | 0.089770671 | 0.187205528 | down |
| NA | -0.928487377 | 0.546597076 | | down |
| LETM1 | -0.928383329 | 7.41E-07 | 1.58E-05 | down |
| PARK2 | -0.928349113 | 0.000418957 | 0.003017776 | down |
| TNIK | -0.928142491 | 1.95E-05 | 0.000245038 | down |
| AC009784.3 | -0.928138352 | 0.078851727 | 0.170359649 | down |
| VLDLR | -0.928080535 | 0.009795133 | 0.036046667 | down |
| RP11-172H24.4 | -0.928026377 | 0.080502951 | 0.173006486 | down |
| RP11-420L9.2 | -0.928006008 | 0.276786737 | 0.41300011 | down |
| RP11-568J23.4 | -0.927905333 | 0.369429021 | 0.507806447 | down |
| GLYAT | -0.927748809 | 0.395800413 | 0.532981434 | down |
| SYT7 | -0.927648756 | 0.000199692 | 0.001667933 | down |
| GJB6 | -0.927242186 | 0.138749843 | 0.254695441 | down |
| RPS15AP6 | -0.927194205 | 0.382337303 | | down |
| NA | -0.92708114 | 0.285985418 | 0.423091684 | down |
| IDNK | -0.926780672 | 6.82E-06 | 0.000102915 | down |
| TMEM191C | -0.926744514 | 0.001009484 | 0.006111011 | down |
| RP11-492E3.2 | -0.92672829 | 0.021493494 | 0.065458748 | down |
| RP1-232L22__B.1 | -0.926700098 | 0.148645394 | 0.267371014 | down |
| TRAPPC6A | -0.926468194 | 2.09E-05 | 0.00026039 | down |
| RPL7AP31 | -0.926190787 | 0.234809524 | 0.367619455 | down |
| GPC5 | -0.926017403 | 0.193938281 | 0.322032924 | down |
| RP11-131M11.3 | -0.92588588 | 0.039376126 | 0.102736979 | down |
| AC093642.5 | -0.925826438 | 0.011793835 | 0.041627898 | down |
| HOMER2P2 | -0.92575014 | 0.668963579 | | down |
| FAM155A | -0.925631505 | 0.020302889 | 0.06280574 | down |
| AC073063.10 | -0.925586013 | 0.08857597 | 0.185411121 | down |
| SYTL5 | -0.92543982 | 0.033859557 | 0.091795392 | down |
| LEP | -0.925377847 | 0.133368053 | 0.247462314 | down |
| SCGB1C2 | -0.92535546 | 0.583228294 | | down |
| NME9 | -0.925340295 | 0.054102128 | 0.129476234 | down |
| TAS2R10 | -0.92529836 | 0.29417922 | | down |
| Y_RNA | -0.925171503 | 0.596317782 | 0.710333323 | down |
| ZNF395 | -0.925087539 | 7.43E-08 | 2.23E-06 | down |
| RP11-802D6.1 | -0.924878556 | 0.082182742 | 0.175749043 | down |
| RP13-672B3.5 | -0.924829063 | 0.325217304 | | down |

| | | | | |
|---|---|---|---|---|
| PRR13 | -0.924768745 | 5.99E-06 | 9.22E-05 | down |
| KIF21A | -0.924702541 | 1.90E-05 | 0.000239664 | down |
| RP11-849F2.5 | -0.924625497 | 0.004288659 | 0.019245475 | down |
| ADGRF3 | -0.924531675 | 0.021275203 | 0.064991581 | down |
| MPP5 | -0.924522636 | 4.52E-06 | 7.31E-05 | down |
| CTD-3149D2.2 | -0.924514039 | 0.75195034 | | down |
| RP4-694B14.8 | -0.924503117 | 0.014631012 | 0.048999456 | down |
| MUC6 | -0.924418613 | 0.027442514 | 0.078604527 | down |
| LCA10 | -0.924417746 | 0.469583485 | | down |
| Y_RNA | -0.924285133 | 0.201128039 | 0.330147561 | down |
| H2AFZP3 | -0.924220123 | 0.168229755 | 0.291582714 | down |
| RP11-20G13.2 | -0.924065103 | 0.497603296 | | down |
| SLC9A2 | -0.924000272 | 0.013698748 | 0.046570154 | down |
| NT5C | -0.923967318 | 0.000681402 | 0.004462082 | down |
| RP13-580B18.4 | -0.923856448 | 0.424241024 | 0.559876843 | down |
| SLC46A3 | -0.923853846 | 0.000192587 | 0.001619847 | down |
| CA12 | -0.923775023 | 0.013165148 | 0.045148257 | down |
| NA | -0.923757796 | 0.485048866 | | down |
| AC009495.3 | -0.923546977 | 0.271139418 | | down |
| RP11-175G14.2 | -0.923466487 | 0.752286531 | | down |
| RP5-865N13.1 | -0.923466487 | 0.752286531 | | down |
| RP11-163I18.2 | -0.923466487 | 0.752286531 | | down |
| CTD-2309H9.3 | -0.923466487 | 0.752286531 | | down |
| OR52P1P | -0.923466487 | 0.752286531 | | down |
| MIR4314 | -0.923466487 | 0.752286531 | | down |
| AC006326.3 | -0.923462594 | 0.741523867 | | down |
| NA | -0.923462325 | 0.740804848 | | down |
| RP11-145P16.2 | -0.923461639 | 0.738977998 | | down |
| AC108039.1 | -0.923461562 | 0.738772908 | | down |
| RP1-315G1.3 | -0.923448649 | 0.016619351 | 0.053915055 | down |
| PHBP18 | -0.923447981 | 0.705703852 | | down |
| CTD-2375G15.1 | -0.923447728 | 0.705133691 | | down |
| MTCO1P29 | -0.923445374 | 0.699903578 | | down |
| RP11-54I5.1 | -0.923432158 | 0.672553924 | | down |
| NA | -0.923431156 | 0.670602589 | | down |
| NA | -0.923430934 | 0.670173626 | | down |
| NA | -0.923429847 | 0.66807738 | | down |
| RP11-381N20.2 | -0.923429338 | 0.667101555 | | down |
| AC139103.1 | -0.923411909 | 0.63579235 | | down |
| RP11-35101.3 | -0.92340755 | 0.628518001 | | down |
| RN7SL146P | -0.92339013 | 0.601265856 | | down |
| TMCC3 | -0.923363871 | 5.73E-06 | 8.89E-05 | down |
| AC006509.7 | -0.923333347 | 0.527315021 | | down |
| RP11-621K7.1 | -0.923332178 | 0.52597612 | | down |
| NA | -0.923270211 | 0.101998217 | 0.204910359 | down |
| RSL24D1P8 | -0.923069193 | 0.482744229 | | down |
| ABHD11 | -0.923018968 | 8.31E-06 | 0.000121105 | down |
| ACAD11 | -0.922835652 | 0.600029212 | | down |
| PRADC1 | -0.922804552 | 5.98E-06 | 9.22E-05 | down |
| RP11-422P24.9 | -0.92262033 | 0.209456256 | | down |
| ATF7IP2 | -0.92260083 | 0.000380332 | 0.002806854 | down |

| | | | | |
|---|---|---|---|---|
| CTB-60E11.9 | -0.922584807 | 0.736682985 | | down |
| EEF1B2P2 | -0.92236954 | 0.04375258 | 0.111032494 | down |
| AC009413.2 | -0.922254194 | 0.127742533 | 0.240257129 | down |
| SDK2 | -0.922172232 | 0.00191428 | 0.010125098 | down |
| NA | -0.922066483 | 0.225222712 | 0.357377794 | down |
| FAM58DP | -0.921784448 | 0.50460196 | | down |
| RP11-169D4.4 | -0.921505928 | 0.572794307 | | down |
| RNA5SP477 | -0.921328586 | 0.339218473 | 0.477710687 | down |
| NA | -0.921304113 | 0.547962287 | | down |
| RPL21P18 | -0.921232283 | 0.374871514 | 0.513132932 | down |
| PGPEP1 | -0.920758186 | 1.50E-10 | 9.68E-09 | down |
| HIST1H3G | -0.920376661 | 0.005743067 | 0.024118633 | down |
| ZNF804A | -0.919822212 | 0.078693095 | 0.170163765 | down |
| MRPS34 | -0.919481178 | 0.001098124 | 0.006539551 | down |
| ISCA1P6 | -0.919415616 | 0.344332316 | | down |
| CTC-297N7.9 | -0.919251197 | 0.602641877 | | down |
| CCDC158 | -0.919155584 | 0.124902764 | 0.236556164 | down |
| OR7E47P | -0.919104942 | 0.162314506 | 0.284265638 | down |
| AC015849.14 | -0.918923826 | 0.513368528 | | down |
| RP5-1132H15.1 | -0.918921842 | 0.219154357 | 0.350601996 | down |
| ZNF573 | -0.918920298 | 0.004100412 | 0.018603559 | down |
| TMPRSS6 | -0.918878956 | 0.015015196 | 0.049989026 | down |
| ASS1P5 | -0.918859495 | 0.451604059 | | down |
| KRT8P45 | -0.918592036 | 0.007794209 | 0.030380022 | down |
| RP11-766H1.1 | -0.918445892 | 0.40952017 | | down |
| SEPT7P7 | -0.918443377 | 0.753570494 | | down |
| RP11-85G18.6 | -0.918402965 | 0.406734472 | | down |
| RN7SL627P | -0.918394042 | 0.738791268 | | down |
| RP11-686F15.2 | -0.918391226 | 0.262729201 | 0.397995969 | down |
| C1QL3 | -0.918358392 | 0.082815814 | 0.176750608 | down |
| RP11-134O21.1 | -0.918277691 | 0.014418562 | 0.04846082 | down |
| ARL16 | -0.91822934 | 3.94E-05 | 0.000439248 | down |
| MTND2P12 | -0.918223694 | 0.753643591 | | down |
| RP11-737G21.1 | -0.918223694 | 0.753643591 | | down |
| CTC-425O23.5 | -0.918223071 | 0.003894967 | 0.017862982 | down |
| GPR50-AS1 | -0.918155024 | 0.706262612 | | down |
| RP3-418A9.3 | -0.918154688 | 0.263659411 | | down |
| snoU13 | -0.918096413 | 0.672199356 | | down |
| MYOT | -0.918089737 | 0.14167832 | 0.258397586 | down |
| RNU11-3P | -0.91794431 | 0.637299398 | | down |
| RP11-561I11.3 | -0.917898087 | 0.671898105 | | down |
| RP5-1050E16.2 | -0.91789675 | 0.671111556 | | down |
| PPIAP14 | -0.917794392 | 0.570040005 | | down |
| HORMAD2 | -0.917789377 | 0.424136417 | 0.559801191 | down |
| RP11-154D17.1 | -0.917776862 | 0.199635346 | 0.328649004 | down |
| FAM86KP | -0.917748544 | 0.527865908 | | down |
| YTHDF3-AS1 | -0.91770984 | 0.001597187 | 0.008772555 | down |
| LINC00992 | -0.917680107 | 0.026630904 | 0.076851299 | down |
| AC110769.3 | -0.917593903 | 0.603080973 | | down |
| CHRM3 | -0.917454259 | 0.021380313 | 0.065193354 | down |
| RP11-318A15.8 | -0.917367621 | 0.276498892 | 0.412807444 | down |

| | | | | |
|---|---|---|---|---|
| RILP | -0.91733811 | 2.12E-05 | 0.000262548 | down |
| CTD-2525I3.2 | -0.917211526 | 0.329467328 | 0.467837999 | down |
| RP4-680D5.2 | -0.916998963 | 0.158656699 | 0.279800532 | down |
| RPL21P126 | -0.916818314 | 0.585695047 | 0.701256206 | down |
| MUC20P1 | -0.916694761 | 0.02123561 | 0.06491023 | down |
| UGT1A8 | -0.916631349 | 0.192183815 | 0.32003469 | down |
| KDELC1P1 | -0.916583618 | 0.370434371 | | down |
| HOXB7 | -0.916491107 | 2.01E-06 | 3.72E-05 | down |
| CRYBA1 | -0.916433037 | 0.176795399 | 0.301712069 | down |
| FSCN2 | -0.916178845 | 0.010033766 | 0.036767328 | down |
| NA | -0.916140449 | 0.610506386 | | down |
| SAC3D1 | -0.916077037 | 0.001818823 | 0.009740837 | down |
| RP11-731D1.1 | -0.9158359 | 0.60637552 | | down |
| ADGRB2 | -0.915783053 | 0.000190488 | 0.001606481 | down |
| RNU1-56P | -0.915677717 | 0.410529625 | | down |
| AC009237.11 | -0.915655707 | 0.467779334 | | down |
| NA | -0.915642045 | 0.00138241 | 0.007820211 | down |
| Y_RNA | -0.9153595 | 0.550572952 | 0.67086076 | down |
| FOXR2 | -0.915195857 | 0.754372979 | | down |
| RP11-61J19.3 | -0.915080091 | 0.636276913 | | down |
| POR | -0.914931768 | 6.50E-07 | 1.41E-05 | down |
| UPF3AP2 | -0.914924816 | 0.021200278 | 0.064841816 | down |
| TTC34 | -0.914638521 | 0.024698809 | 0.072636161 | down |
| NME2 | -0.914559518 | 0.023456108 | 0.069836024 | down |
| RP11-325E5.4 | -0.914471584 | 0.566750655 | | down |
| AC098617.1 | -0.914135458 | 0.421774178 | | down |
| RP5-891H21.5 | -0.913782264 | 0.486927458 | | down |
| RP11-1018N14.5 | -0.913736658 | 0.197118312 | 0.32570107 | down |
| SCN9A | -0.913709593 | 0.010249798 | 0.037362889 | down |
| NDUFS8 | -0.913652931 | 0.00020899 | 0.001733067 | down |
| SLC44A5 | -0.913620563 | 0.094669056 | 0.194471536 | down |
| RP11-77E14.2 | -0.913539912 | 0.171120806 | 0.295027746 | down |
| RP11-497E19.1 | -0.913475526 | 0.091473796 | 0.189638333 | down |
| ZNF774 | -0.913201465 | 0.001745829 | 0.009422301 | down |
| MS4A2 | -0.913022724 | 0.002876572 | 0.014025273 | down |
| RP11-44F14.1 | -0.912971696 | 0.15806852 | 0.278967596 | down |
| RAVER2 | -0.912900003 | 3.23E-05 | 0.000372439 | down |
| CTNND2 | -0.912845074 | 0.017770501 | 0.05685198 | down |
| CITF22-92A6.2 | -0.912793245 | 0.267270963 | | down |
| CH17-302M23.1 | -0.912712251 | 0.230329995 | | down |
| UBE2NL | -0.912686699 | 0.608067891 | | down |
| RP11-150012.3 | -0.912420782 | 0.003056594 | 0.014704934 | down |
| CTA-212A2.4 | -0.91241834 | 0.61533314 | | down |
| RP11-170K4.2 | -0.912176155 | 0.370504635 | 0.508939649 | down |
| SLC44A3 | -0.912162461 | 0.000954054 | 0.005833991 | down |
| PYGB | -0.91210578 | 0.000153874 | 0.001347021 | down |
| SNORA2A | -0.912068027 | 0.331884896 | | down |
| SMCO3 | -0.911901314 | 0.606766334 | | down |
| CTD-237103.2 | -0.911896661 | 0.35658995 | 0.495050527 | down |
| ARMC12 | -0.911761289 | 0.027728596 | 0.079212508 | down |
| SALRNA2 | -0.911732666 | 0.654698349 | 0.757665622 | down |

| | | | | |
|---|---|---|---|---|
| CFAP100 | -0.911565325 | 0.125314803 | 0.236998486 | down |
| LINC00562 | -0.91152853 | 0.044006193 | 0.111486809 | down |
| RP3-522P13.1 | -0.911476766 | 0.678474065 | 0.777039409 | down |
| ZNF320 | -0.911446402 | 3.28E-05 | 0.000376205 | down |
| HMGN2P6 | -0.911415789 | 0.283102517 | 0.419673954 | down |
| RP11-520H14.7 | -0.911392105 | 0.077615528 | 0.168403331 | down |
| bP-2171C21.6 | -0.911228587 | 0.605918527 | | down |
| LGR5 | -0.911112318 | 0.140574968 | 0.257103204 | down |
| KRTAP4-1 | -0.910975883 | 0.230461007 | 0.362888669 | down |
| AXDND1 | -0.910841044 | 0.027555014 | 0.078836571 | down |
| CTA-363E6.6 | -0.910838556 | 0.313612806 | | down |
| TCP1P1 | -0.910486873 | 0.489124439 | | down |
| NA | -0.910452917 | 0.070896384 | 0.157755219 | down |
| SEMA4G | -0.910374593 | 0.002092949 | 0.010871468 | down |
| AP000251.3 | -0.910353188 | 0.034640088 | 0.093395356 | down |
| MATR3 | -0.909958756 | 0.023634465 | 0.070245527 | down |
| RP11-475I24.3 | -0.909848999 | 0.029730685 | 0.083253677 | down |
| AK7 | -0.909773871 | 0.005585035 | 0.023610074 | down |
| RP11-499F19.3 | -0.909455699 | 0.283760494 | 0.420441893 | down |
| Y_RNA | -0.909318405 | 0.010553017 | 0.038231622 | down |
| RP11-976B16.1 | -0.909311501 | 0.394054855 | | down |
| RP11-182J1.5 | -0.909299654 | 0.475404145 | | down |
| NA | -0.909298756 | 0.001883133 | 0.010006519 | down |
| STEAP3 | -0.909168139 | 0.002416792 | 0.012248669 | down |
| RP11-16E18.3 | -0.908984402 | 0.023180663 | 0.069201948 | down |
| CH507-42P11.7 | -0.908888524 | 0.355348563 | | down |
| KCP | -0.908602455 | 0.001193596 | 0.006984887 | down |
| C6orf136 | -0.908424091 | 8.87E-08 | 2.57E-06 | down |
| WBP2NL | -0.908347585 | 0.010641111 | 0.038458022 | down |
| NA | -0.908310425 | 0.756135959 | | down |
| UQCRBP1 | -0.908158761 | 0.014352178 | 0.048302547 | down |
| EFNA2 | -0.908003003 | 0.00414457 | 0.018755754 | down |
| AP001625.4 | -0.907981552 | 0.438637285 | 0.572850575 | down |
| RP11-94M14.3 | -0.907903114 | 0.681910689 | 0.779610481 | down |
| ARHGAP40 | -0.907898625 | 0.380916505 | 0.518851929 | down |
| PLXNA2 | -0.907764336 | 1.21E-05 | 0.000164969 | down |
| RPS26P21 | -0.907612322 | 0.382812534 | | down |
| CTD-2021J15.1 | -0.907539488 | 0.639662569 | | down |
| MATK | -0.907444817 | 0.000698017 | 0.004555991 | down |
| NDUFB11 | -0.907444282 | 0.001167623 | 0.006860956 | down |
| HOXB8 | -0.907436349 | 0.001373327 | 0.007782013 | down |
| EXOC3-AS1 | -0.907271908 | 1.44E-05 | 0.000191565 | down |
| HORMAD2-AS1 | -0.907245889 | 0.351836944 | 0.49053734 | down |
| RABAC1 | -0.907168895 | 0.007132105 | 0.028377295 | down |
| RP5-1039K5.17 | -0.906798262 | 0.141627136 | 0.2583356 | down |
| FMO1 | -0.906623743 | 0.056620178 | 0.133954898 | down |
| PLD1 | -0.906486233 | 9.66E-08 | 2.75E-06 | down |
| AC144835.1 | -0.906376564 | 0.074081747 | 0.162853004 | down |
| RP11-730G20.2 | -0.906361989 | 0.004813533 | 0.021084384 | down |
| ASAP1-IT2 | -0.906100518 | 0.120173252 | 0.230398629 | down |
| FRMD3 | -0.906098712 | 0.001417233 | 0.007973659 | down |

| | | | | |
|---|---|---|---|---|
| RP11-421E14.2 | -0.905986475 | 0.493914837 | | down |
| FGF21 | -0.905983504 | 0.577323724 | 0.693998878 | down |
| CGA | -0.905777919 | 0.511235657 | | down |
| NA | -0.905617168 | 0.306244696 | 0.444634207 | down |
| MYO1D | -0.905596405 | 0.000289522 | 0.002266979 | down |
| LA16c-390E6.3 | -0.905557573 | 0.188761963 | 0.31580156 | down |
| RPL22P11 | -0.90549282 | 0.323726418 | | down |
| SYN3 | -0.905337972 | 0.008503501 | 0.032380124 | down |
| RP11-620J15.4 | -0.90533095 | 0.095645381 | 0.195773936 | down |
| TYRO3 | -0.905246356 | 0.000677145 | 0.004440001 | down |
| RP4-564F22.6 | -0.905087228 | 0.525829511 | | down |
| RP11-470P21.2 | -0.904676417 | 0.178107652 | 0.303210919 | down |
| GFER | -0.904498083 | 4.12E-06 | 6.79E-05 | down |
| PPP1R14C | -0.904483751 | 0.007853209 | 0.030562411 | down |
| TMEM88B | -0.904470002 | 0.247244856 | 0.381599942 | down |
| FAM66C | -0.904433039 | 0.004943304 | 0.021499223 | down |
| NCCRP1 | -0.904335557 | 0.026588903 | 0.076766961 | down |
| GNRH2 | -0.904312421 | 0.070091088 | 0.156517569 | down |
| MGST3 | -0.904301631 | 5.87E-07 | 1.29E-05 | down |
| RPS10P28 | -0.90428906 | 0.706499336 | | down |
| ZNF341 | -0.904219392 | 1.87E-05 | 0.000237826 | down |
| HM13-AS1 | -0.903981086 | 0.102090933 | 0.205055488 | down |
| RP11-138J23.1 | -0.90398092 | 0.557011254 | 0.676620148 | down |
| RP11-1007O24.2 | -0.903903358 | 0.064989455 | 0.147964028 | down |
| NA | -0.903897033 | 0.486645158 | | down |
| LRP4 | -0.903607307 | 0.010894196 | 0.039165398 | down |
| RP11-847H18.3 | -0.903600921 | 0.285754609 | 0.422812439 | down |
| RP11-1127D7.1 | -0.903349496 | 0.496871465 | 0.624383832 | down |
| PEBP4 | -0.90333477 | 0.035031027 | 0.09411404 | down |
| RP11-382A20.1 | -0.903242533 | 0.249220592 | | down |
| RNU6-306P | -0.903240276 | 0.607097753 | | down |
| PGM5P4 | -0.903222161 | 0.113902197 | 0.221651506 | down |
| GS1-590J6.3 | -0.903185723 | 0.166353354 | 0.289466013 | down |
| RP13-143G15.4 | -0.902568033 | 0.196890461 | 0.325503605 | down |
| RP3-454B23.1 | -0.902278541 | 0.617173333 | | down |
| RPS4XP2 | -0.902246572 | 0.183911681 | 0.309756842 | down |
| MYL12BP2 | -0.902188082 | 0.131168979 | 0.244848762 | down |
| SLC2A3P2 | -0.901886981 | 0.613262826 | | down |
| SYCP2 | -0.901767057 | 0.023240388 | 0.069351829 | down |
| EMILIN3 | -0.901746537 | 0.024397131 | 0.071995619 | down |
| C6orf52 | -0.901736891 | 0.000996359 | 0.006046172 | down |
| HADHA | -0.90149025 | 6.38E-13 | 7.48E-11 | down |
| GPHA2 | -0.901477644 | 0.104034597 | 0.207654493 | down |
| RP5-1009E24.8 | -0.90130088 | 0.038679901 | 0.101296699 | down |
| CTC-444N24.13 | -0.901275502 | 0.292188979 | 0.429435754 | down |
| OSGEPL1-AS1 | -0.901104606 | 0.036284071 | 0.096658861 | down |
| C5orf38 | -0.900829037 | 0.105417304 | 0.209856711 | down |
| CTD-2643K12.1 | -0.900763241 | 0.24388624 | 0.377891604 | down |
| RPSAP54 | -0.900479874 | 0.014683368 | 0.049125515 | down |
| PRLHR | -0.900398664 | 0.608569116 | 0.720659435 | down |
| D2HGDH | -0.899939451 | 0.000418705 | 0.003017574 | down |

| | | | | |
|---|---|---|---|---|
| MTCYBP45 | -0.89987354 | 0.654985473 | | down |
| RP11-558A11.2 | -0.899816928 | 0.434905478 | | down |
| CFDP1 | -0.899807601 | 1.30E-07 | 3.56E-06 | down |
| CYP2D8P | -0.899737267 | 0.020577715 | 0.063400481 | down |
| HFE2 | -0.899663168 | 0.310035445 | 0.448598773 | down |
| ACBD4 | -0.899367685 | 9.67E-10 | 4.91E-08 | down |
| IL36G | -0.899270719 | 0.482190195 | | down |
| FLJ16779 | -0.89901831 | 0.115006846 | 0.22323722 | down |
| RBPMS-AS1 | -0.898997551 | 0.027211857 | 0.078115139 | down |
| RP11-322D14.2 | -0.898949431 | 0.087144718 | 0.183307949 | down |
| PLAC8L1 | -0.898903892 | 0.004863676 | 0.021229739 | down |
| RP11-93O14.2 | -0.898838467 | 0.045247254 | 0.113653416 | down |
| NA | -0.898831394 | 0.497415848 | | down |
| NPPC | -0.898762089 | 0.086486584 | 0.182307579 | down |
| AP000679.2 | -0.898688479 | 0.375906713 | | down |
| Xbac-B444P24.1( | -0.898610246 | 0.067650681 | 0.152429657 | down |
| RP11-474N24.6 | -0.898605543 | 0.096468721 | 0.197029657 | down |
| RPS6KA5 | -0.898545099 | 2.84E-06 | 4.98E-05 | down |
| NA | -0.898332699 | 0.529005218 | | down |
| AC005606.14 | -0.898260368 | 0.02647799 | 0.076512909 | down |
| FN3K | -0.898069915 | 0.000299027 | 0.002323844 | down |
| LRRC36 | -0.8980123 | 0.014280219 | 0.048119661 | down |
| RP11-885B4.2 | -0.897837554 | 0.326049275 | | down |
| JMJD4 | -0.897824839 | 0.000565038 | 0.003840724 | down |
| HMGB1P39 | -0.897641431 | 0.524446046 | | down |
| NA | -0.897613571 | 0.577572361 | | down |
| RP11-305P14.1 | -0.897561349 | 0.495456913 | 0.623344641 | down |
| ACKR2 | -0.897271486 | 0.004911961 | 0.021405303 | down |
| STRC | -0.897219643 | 0.033992405 | 0.092089074 | down |
| CA2 | -0.897123121 | 0.051234987 | 0.124539155 | down |
| C1orf100 | -0.897088266 | 0.083672954 | 0.177940385 | down |
| CBX1P3 | -0.896675611 | 0.562598333 | 0.681588141 | down |
| RP11-734E19.1 | -0.896516803 | 0.421156063 | | down |
| RANBP17 | -0.896495691 | 0.004067568 | 0.01848246 | down |
| IQGAP2 | -0.896483593 | 9.66E-05 | 0.000919052 | down |
| MLLT10P1 | -0.896477873 | 0.31520844 | | down |
| RP11-749H17.2 | -0.896441473 | 0.474816172 | 0.604849569 | down |
| Y_RNA | -0.896437885 | 0.759310971 | | down |
| HSPE1P25 | -0.896437885 | 0.759310971 | | down |
| TCEB1P20 | -0.896437885 | 0.759310971 | | down |
| PUDPP2 | -0.896437885 | 0.759310971 | | down |
| PRR9 | -0.896437885 | 0.759310971 | | down |
| RP11-316I3.1 | -0.896437885 | 0.759310971 | | down |
| LINC01221 | -0.896437885 | 0.759310971 | | down |
| RP11-453O22.1 | -0.896437885 | 0.759310971 | | down |
| RP5-865N13.2 | -0.896437885 | 0.759310971 | | down |
| RP4-799P18.2 | -0.896437885 | 0.759310971 | | down |
| AC114808.3 | -0.896437885 | 0.759310971 | | down |
| BRD7P6 | -0.896437885 | 0.759310971 | | down |
| CNN2P11 | -0.896437885 | 0.759310971 | | down |
| RNU6-1320P | -0.896437885 | 0.759310971 | | down |

| | | | |
|---|---|---|---|
| AC097499.2 | -0.896437885 | 0.759310971 | down |
| AC093787.1 | -0.896437885 | 0.759310971 | down |
| AC069394.1 | -0.896437885 | 0.759310971 | down |
| AC023469.1 | -0.896437885 | 0.759310971 | down |
| MTCO1P17 | -0.896437885 | 0.759310971 | down |
| CRYGFP | -0.896437885 | 0.759310971 | down |
| AC098973.1 | -0.896437885 | 0.759310971 | down |
| SALL4P6 | -0.896437885 | 0.759310971 | down |
| RP11-10022.1 | -0.896437885 | 0.759310971 | down |
| RP11-208P4.1 | -0.896437885 | 0.759310971 | down |
| RNU1-70P | -0.896437885 | 0.759310971 | down |
| EEF1B2P8 | -0.896437885 | 0.759310971 | down |
| RP11-440L14.3 | -0.896437885 | 0.759310971 | down |
| RP11-357G3.1 | -0.896437885 | 0.759310971 | down |
| MIR4274 | -0.896437885 | 0.759310971 | down |
| ZEB2P1 | -0.896437885 | 0.759310971 | down |
| RP11-600L4.1 | -0.896437885 | 0.759310971 | down |
| RP11-241F15.7 | -0.896437885 | 0.759310971 | down |
| RP11-593F5.2 | -0.896437885 | 0.759310971 | down |
| RP11-580P21.1 | -0.896437885 | 0.759310971 | down |
| RP11-542G1.2 | -0.896437885 | 0.759310971 | down |
| NPM1P41 | -0.896437885 | 0.759310971 | down |
| RP11-51M24.1 | -0.896437885 | 0.759310971 | down |
| NACAP6 | -0.896437885 | 0.759310971 | down |
| RP11-5N11.6 | -0.896437885 | 0.759310971 | down |
| CTD-2197M16.1 | -0.896437885 | 0.759310971 | down |
| EGFLAM-AS3 | -0.896437885 | 0.759310971 | down |
| AC008834.2 | -0.896437885 | 0.759310971 | down |
| Y_RNA | -0.896437885 | 0.759310971 | down |
| CTC-546K23.2 | -0.896437885 | 0.759310971 | down |
| RP11-461L18.1 | -0.896437885 | 0.759310971 | down |
| RP11-375B1.2 | -0.896437885 | 0.759310971 | down |
| OR2V1 | -0.896437885 | 0.759310971 | down |
| RNU2-45P | -0.896437885 | 0.759310971 | down |
| OR2G1P | -0.896437885 | 0.759310971 | down |
| KHDC1P1 | -0.896437885 | 0.759310971 | down |
| RP1-177I10.1 | -0.896437885 | 0.759310971 | down |
| EIF4EBP2P3 | -0.896437885 | 0.759310971 | down |
| RP11-809N15.2 | -0.896437885 | 0.759310971 | down |
| RP11-428F8.2 | -0.896437885 | 0.759310971 | down |
| RN7SKP245 | -0.896437885 | 0.759310971 | down |
| RNA5SP218 | -0.896437885 | 0.759310971 | down |
| GNG5P1 | -0.896437885 | 0.759310971 | down |
| RP4-815D20.1 | -0.896437885 | 0.759310971 | down |
| RP11-760D2.4 | -0.896437885 | 0.759310971 | down |
| AC005276.1 | -0.896437885 | 0.759310971 | down |
| uc_338 | -0.896437885 | 0.759310971 | down |
| CTD-2023J5.1 | -0.896437885 | 0.759310971 | down |
| RP11-561E1.1 | -0.896437885 | 0.759310971 | down |
| MTND2P32 | -0.896437885 | 0.759310971 | down |
| RP11-359P18.8 | -0.896437885 | 0.759310971 | down |

| | | | |
|---|---|---|---|
| RNU6-519P | -0.896437885 | 0.759310971 | down |
| RP11-300E4.2 | -0.896437885 | 0.759310971 | down |
| RPS20P23 | -0.896437885 | 0.759310971 | down |
| NA | -0.896437885 | 0.759310971 | down |
| RN7SL228P | -0.896437885 | 0.759310971 | down |
| PDSS1P1 | -0.896437885 | 0.759310971 | down |
| IFNA12P | -0.896437885 | 0.759310971 | down |
| NA | -0.896437885 | 0.759310971 | down |
| SPATA31A1 | -0.896437885 | 0.759310971 | down |
| RPL5P25 | -0.896437885 | 0.759310971 | down |
| RNU4ATAC6P | -0.896437885 | 0.759310971 | down |
| FXYD6P1 | -0.896437885 | 0.759310971 | down |
| MRPS35P3 | -0.896437885 | 0.759310971 | down |
| MIR4676 | -0.896437885 | 0.759310971 | down |
| RPL39P26 | -0.896437885 | 0.759310971 | down |
| RPL7AP58 | -0.896437885 | 0.759310971 | down |
| RP11-707M1.7 | -0.896437885 | 0.759310971 | down |
| OR4A19P | -0.896437885 | 0.759310971 | down |
| OR5F1 | -0.896437885 | 0.759310971 | down |
| RP11-643G5.4 | -0.896437885 | 0.759310971 | down |
| TCEB1P22 | -0.896437885 | 0.759310971 | down |
| OR6M3P | -0.896437885 | 0.759310971 | down |
| OR8B4 | -0.896437885 | 0.759310971 | down |
| RP11-69C13.1 | -0.896437885 | 0.759310971 | down |
| RP11-59N23.3 | -0.896437885 | 0.759310971 | down |
| RP11-560G2.2 | -0.896437885 | 0.759310971 | down |
| RN7SL734P | -0.896437885 | 0.759310971 | down |
| NA | -0.896437885 | 0.759310971 | down |
| RP11-406H4.1 | -0.896437885 | 0.759310971 | down |
| ATXN8OS | -0.896437885 | 0.759310971 | down |
| RP11-272M24.2 | -0.896437885 | 0.759310971 | down |
| LINC01043 | -0.896437885 | 0.759310971 | down |
| KRT8P1 | -0.896437885 | 0.759310971 | down |
| RP11-88E18.1 | -0.896437885 | 0.759310971 | down |
| RPA2P1 | -0.896437885 | 0.759310971 | down |
| MIR4504 | -0.896437885 | 0.759310971 | down |
| RP11-26L16.1 | -0.896437885 | 0.759310971 | down |
| RP11-8L8.1 | -0.896437885 | 0.759310971 | down |
| IGHVIII-67-2 | -0.896437885 | 0.759310971 | down |
| RP11-173D3.3 | -0.896437885 | 0.759310971 | down |
| RP11-467L19.8 | -0.896437885 | 0.759310971 | down |
| RP11-123C21.3 | -0.896437885 | 0.759310971 | down |
| RP11-313H3.1 | -0.896437885 | 0.759310971 | down |
| RP11-379K22.2 | -0.896437885 | 0.759310971 | down |
| RARRES2P5 | -0.896437885 | 0.759310971 | down |
| CTB-55P19.1 | -0.896437885 | 0.759310971 | down |
| MOCS1P1 | -0.896437885 | 0.759310971 | down |
| HSPE1P5 | -0.896437885 | 0.759310971 | down |
| NA | -0.896437885 | 0.759310971 | down |
| FOXC2-AS1 | -0.896437885 | 0.759310971 | down |
| OR1E3 | -0.896437885 | 0.759310971 | down |

| | | | | |
|---|---|---|---|---|
| MIR1269B | -0.896437885 | 0.759310971 | | down |
| KYNUP1 | -0.896437885 | 0.759310971 | | down |
| RP11-238F2.1 | -0.896437885 | 0.759310971 | | down |
| AP002414.1 | -0.896437885 | 0.759310971 | | down |
| RPL23AP77 | -0.896437885 | 0.759310971 | | down |
| DYNAP | -0.896437885 | 0.759310971 | | down |
| RN7SL795P | -0.896437885 | 0.759310971 | | down |
| RP11-669I1.1 | -0.896437885 | 0.759310971 | | down |
| RP11-173L6.1 | -0.896437885 | 0.759310971 | | down |
| SIGLEC28P | -0.896437885 | 0.759310971 | | down |
| RN7SL14P | -0.896437885 | 0.759310971 | | down |
| EEF1A1P34 | -0.896437885 | 0.759310971 | | down |
| LINC01384 | -0.896437885 | 0.759310971 | | down |
| RP1-300I2.2 | -0.896437885 | 0.759310971 | | down |
| SPINT4 | -0.896437885 | 0.759310971 | | down |
| RNU7-6P | -0.896437885 | 0.759310971 | | down |
| KRTAP27-1 | -0.896437885 | 0.759310971 | | down |
| KRTAP19-2 | -0.896437885 | 0.759310971 | | down |
| ABCD1P4 | -0.896437885 | 0.759310971 | | down |
| ZNF402P | -0.896437885 | 0.759310971 | | down |
| IGLVVII-41-1 | -0.896437885 | 0.759310971 | | down |
| RN7SL20P | -0.896437885 | 0.759310971 | | down |
| RP11-501H19.2 | -0.896437885 | 0.759310971 | | down |
| FTHL17 | -0.896437885 | 0.759310971 | | down |
| RP11-192I24.1 | -0.896437885 | 0.759310971 | | down |
| UBE2DNL | -0.896437885 | 0.759310971 | | down |
| RP13-75G22.1 | -0.896437885 | 0.759310971 | | down |
| BEND7P1 | -0.896437885 | 0.759310971 | | down |
| RNU6-382P | -0.896437885 | 0.759310971 | | down |
| RPARP-AS1 | -0.896297759 | 0.000263027 | 0.002096682 | down |
| CH17-260O16.1 | -0.896156497 | 0.104900448 | 0.2089524 | down |
| GALNT6 | -0.896070569 | 0.001156041 | 0.006806209 | down |
| ATP6V0D1 | -0.896042792 | 4.52E-05 | 0.000491873 | down |
| ABHD6 | -0.89591163 | 0.000684892 | 0.004480054 | down |
| NCLN | -0.895853424 | 0.000661029 | 0.004355252 | down |
| SLC25A2 | -0.895709986 | 0.22773891 | 0.359912048 | down |
| RP11-368I7.6 | -0.895682317 | 0.00433793 | 0.019399936 | down |
| GLIDR | -0.895373587 | 0.000856014 | 0.005351911 | down |
| ENTPD1-AS1 | -0.895365156 | 0.007201495 | 0.02858147 | down |
| RP11-259O2.1 | -0.895242148 | 0.041754341 | 0.107366264 | down |
| VPS13A-AS1 | -0.895205954 | 0.480851586 | | down |
| NA | -0.895065715 | 0.200921647 | 0.32995299 | down |
| RP11-777J24.1 | -0.895055785 | 0.161066147 | 0.282639526 | down |
| HOMER2 | -0.894969154 | 0.00283734 | 0.013874519 | down |
| RP11-103B5.4 | -0.894885488 | 0.147450392 | 0.265858464 | down |
| RPS20P10 | -0.894592261 | 0.064507387 | 0.147224244 | down |
| RP11-876N24.2 | -0.894586044 | 0.194960363 | 0.323380398 | down |
| DDO | -0.894528283 | 0.00657211 | 0.026708515 | down |
| GABRB3 | -0.89448388 | 0.037887181 | 0.099761829 | down |
| AC012358.7 | -0.894369249 | 0.34001529 | 0.478339547 | down |
| LRRC1 | -0.894248582 | 5.59E-06 | 8.69E-05 | down |

| | | | | |
|---|---|---|---|---|
| Metazoa_SRP | -0.894195362 | 0.294986312 | 0.432425897 | down |
| CCS | -0.894165339 | 0.000169549 | 0.001459157 | down |
| FABP5P2 | -0.893981944 | 0.138137021 | 0.253958429 | down |
| TAC3 | -0.893597463 | 0.005423599 | 0.023096614 | down |
| RP11-374M1.2 | -0.893569782 | 0.00046579 | 0.003287335 | down |
| ELF3 | -0.893427484 | 0.003332519 | 0.015720239 | down |
| Y_RNA | -0.893416829 | 0.125903664 | 0.237842662 | down |
| DTX2 | -0.893066213 | 0.000217207 | 0.001789355 | down |
| IGHD1-14 | -0.893056693 | 0.481161761 | | down |
| RP11-1D12.2 | -0.892976054 | 0.487810494 | | down |
| NANOS3 | -0.892853855 | 0.021521123 | 0.065529605 | down |
| AP006748.1 | -0.892696288 | 0.242251424 | 0.376219144 | down |
| RP11-255H23.2 | -0.892469969 | 0.006134617 | 0.025383339 | down |
| SDHA | -0.892253501 | 0.0008679 | 0.005409791 | down |
| MEIS1-AS3 | -0.892106531 | 0.420363311 | 0.556074137 | down |
| RP11-757G1.5 | -0.892025661 | 0.06430121 | 0.146876421 | down |
| RP11-656D10.3 | -0.89166491 | 0.007791939 | 0.030375115 | down |
| RP13-890H12.2 | -0.891604251 | 0.043208582 | 0.11006827 | down |
| RP11-116D17.5 | -0.891393486 | 0.468157463 | | down |
| EPGN | -0.891266879 | 0.280868237 | 0.417299727 | down |
| ULK4 | -0.891183684 | 0.001896894 | 0.010052252 | down |
| RP11-305L7.7 | -0.891025206 | 0.17573186 | 0.300374807 | down |
| CTD-2357A8.2 | -0.890997706 | 0.332305408 | 0.470622405 | down |
| RP1-273G13.2 | -0.890976126 | 0.11108199 | 0.217913482 | down |
| DTX4 | -0.890953182 | 6.05E-10 | 3.32E-08 | down |
| OR13A1 | -0.890939681 | 0.124568154 | 0.236116944 | down |
| GAREM1 | -0.890606123 | 8.36E-05 | 0.000815712 | down |
| RNU6-133P | -0.890406962 | 0.600168145 | | down |
| APOBR | -0.8902952 | 0.002227569 | 0.011451929 | down |
| CTD-2509G16.5 | -0.890083911 | 0.111981816 | 0.219092968 | down |
| ZNF233 | -0.890043086 | 0.002594383 | 0.012943481 | down |
| RP11-309L24.4 | -0.889979109 | 0.002366804 | 0.012027783 | down |
| CYP2A6 | -0.889884077 | 0.146583767 | 0.264788522 | down |
| FAM126B | -0.889808772 | 1.63E-06 | 3.11E-05 | down |
| LINC00336 | -0.889486456 | 0.159455551 | 0.280649243 | down |
| ART5 | -0.889468865 | 0.077050115 | 0.167568504 | down |
| FLVCR2 | -0.889437017 | 0.000765513 | 0.004902807 | down |
| TPRG1-AS1 | -0.889018064 | 0.354890602 | | down |
| SNHG18 | -0.889004524 | 0.0001009 | 0.000952688 | down |
| LINC00884 | -0.888903668 | 0.005186661 | 0.022300061 | down |
| ZNF814 | -0.888857666 | 0.000121091 | 0.001111 | down |
| RP11-110I1.11 | -0.888580439 | 0.090016433 | 0.187548802 | down |
| PSORS1C1 | -0.888518216 | 0.002409228 | 0.012214455 | down |
| GPR160 | -0.888269373 | 0.000108547 | 0.001013421 | down |
| SCRG1 | -0.888047136 | 0.071651784 | 0.159070878 | down |
| TEF | -0.888014873 | 3.50E-07 | 8.22E-06 | down |
| YBEY | -0.887873801 | 8.18E-05 | 0.000801332 | down |
| ANAPC1P1 | -0.887733023 | 0.177557193 | 0.302530953 | down |
| EEF1A1P16 | -0.887593524 | 0.142018179 | 0.25884645 | down |
| CCNI2 | -0.88756681 | 0.000856176 | 0.005351911 | down |
| MPPED2 | -0.887526682 | 0.045359849 | 0.113850569 | down |

| | | | | |
|---|---|---|---|---|
| RNU7-171P | -0.887502946 | 0.229413794 | | down |
| RP11-462B18.3 | -0.887446884 | 0.411713333 | | down |
| NA | -0.887134367 | 0.065683092 | 0.149103347 | down |
| UQCR11 | -0.887039542 | 4.30E-05 | 0.000470769 | down |
| CTD-2046J7.1 | -0.887029466 | 0.684673647 | | down |
| Y_RNA | -0.887029464 | 0.684996706 | | down |
| MIR6512 | -0.887029188 | 0.726751707 | | down |
| RP11-277B15.1 | -0.887028901 | 0.756235573 | | down |
| RP11-75L1.6 | -0.887028852 | 0.760412597 | | down |
| RP11-177A2.5 | -0.887028835 | 0.761760392 | | down |
| AC125238.3 | -0.887028835 | 0.761760392 | | down |
| RP11-24C3.2 | -0.887028835 | 0.761760392 | | down |
| RP11-641C17.3 | -0.887028835 | 0.761760392 | | down |
| RP11-735B13.2 | -0.887028835 | 0.761760392 | | down |
| KLF7P1 | -0.887028835 | 0.761760392 | | down |
| RP11-431M7.2 | -0.887028835 | 0.761760392 | | down |
| ATG10-IT1 | -0.887028835 | 0.761760392 | | down |
| NA | -0.887028835 | 0.761760392 | | down |
| RP5-1173I20.1 | -0.887028835 | 0.761760392 | | down |
| MTND2P6 | -0.887028835 | 0.761760392 | | down |
| RN7SL750P | -0.887028835 | 0.761760392 | | down |
| RP5-1166A24.1 | -0.887028835 | 0.761760392 | | down |
| LINC01181 | -0.887028835 | 0.761760392 | | down |
| RNA5SP285 | -0.887028835 | 0.761760392 | | down |
| RP11-29B11.5 | -0.887028835 | 0.761760392 | | down |
| RP5-933E2.1 | -0.887028835 | 0.761760392 | | down |
| ATP5G1P8 | -0.887028835 | 0.761760392 | | down |
| RCBTB2P1 | -0.887028835 | 0.761760392 | | down |
| OR51B8P | -0.887028835 | 0.761760392 | | down |
| CTC-830E23.1 | -0.887028835 | 0.761760392 | | down |
| RP11-21903.2 | -0.887028835 | 0.761760392 | | down |
| FTLP6 | -0.887028835 | 0.761760392 | | down |
| EGLN3P1 | -0.887028835 | 0.761760392 | | down |
| RP11-972L6.2 | -0.887028835 | 0.761760392 | | down |
| RP11-512N21.1 | -0.887028835 | 0.761760392 | | down |
| KARSP1 | -0.887028835 | 0.761760392 | | down |
| RP11-460G11.2 | -0.887028835 | 0.761760392 | | down |
| LINC00440 | -0.887028835 | 0.761760392 | | down |
| IGHV3-19 | -0.887028835 | 0.761760392 | | down |
| Y_RNA | -0.887028835 | 0.761760392 | | down |
| MIR6504 | -0.887028835 | 0.761760392 | | down |
| AC015922.5 | -0.887028835 | 0.761760392 | | down |
| ABCA9-AS1 | -0.887028835 | 0.761760392 | | down |
| RP11-243E13.2 | -0.887028835 | 0.761760392 | | down |
| AC114273.3 | -0.887028835 | 0.761760392 | | down |
| RNU6-192P | -0.887028835 | 0.761760392 | | down |
| RP11-571E6.1 | -0.887028835 | 0.761760392 | | down |
| RP11-432N13.2 | -0.887028835 | 0.761760392 | | down |
| C20orf166-AS1 | -0.886778284 | 0.036428096 | 0.096888155 | down |
| RP4-657E11.10 | -0.886751708 | 0.060127626 | 0.139917466 | down |
| SNHG19 | -0.886591543 | 0.003726669 | 0.017227887 | down |

| | | | | |
|---|---|---|---|---|
| UBE2SP1 | -0.886432937 | 0.05814454 | 0.136625571 | down |
| SF3B5 | -0.886431123 | 0.000466319 | 0.003290295 | down |
| RP11-25I15.3 | -0.88639006 | 0.552091056 | | down |
| RP11-383C5.5 | -0.886346143 | 0.227478778 | 0.35968488 | down |
| BMS1P1 | -0.886135521 | 0.0120117 | 0.042231308 | down |
| RNPEPL1 | -0.886111943 | 0.000178596 | 0.001524794 | down |
| CLTB | -0.885941657 | 0.000352848 | 0.002654891 | down |
| MIR4768 | -0.885871528 | 0.159839839 | 0.281029268 | down |
| C15orf56 | -0.885766896 | 0.258040476 | | down |
| ABCA10 | -0.885609599 | 0.003260654 | 0.015463373 | down |
| CTB-113P19.3 | -0.88544032 | 0.164927742 | 0.287601665 | down |
| RP11-45901.2 | -0.885240661 | 0.12173724 | 0.232412381 | down |
| RP11-667K14.8 | -0.885151006 | 0.325452187 | 0.463978623 | down |
| KCNS2 | -0.885050435 | 0.069884384 | 0.156127784 | down |
| RP11-135A1.3 | -0.884828457 | 0.410303366 | | down |
| RP11-497G19.7 | -0.884812986 | 0.48097134 | | down |
| NA | -0.884762162 | 0.033370934 | 0.090699868 | down |
| RP11-462L8.2 | -0.884459418 | 0.390202122 | | down |
| HMGB1P37 | -0.884454661 | 0.152318461 | 0.271793295 | down |
| CHCHD5 | -0.884452288 | 0.000825821 | 0.005211292 | down |
| RP11-190A12.7 | -0.884408347 | 0.163509203 | 0.285756064 | down |
| SEPT7P9 | -0.884185792 | 0.070833011 | 0.15766091 | down |
| CA3-AS1 | -0.884160898 | 0.021665894 | 0.065870283 | down |
| TRPC3 | -0.883897941 | 0.050546734 | 0.12327511 | down |
| TRDC | -0.883891165 | 0.00381999 | 0.017570083 | down |
| IL2 | -0.883811667 | 0.158050207 | 0.278967596 | down |
| HES2 | -0.883684688 | 0.006846036 | 0.027530758 | down |
| RP11-104L21.2 | -0.883671638 | 0.264128334 | 0.39958897 | down |
| RN7SKP80 | -0.883505465 | 0.043859343 | 0.111189782 | down |
| RP11-468E2.5 | -0.883469325 | 0.036916294 | 0.097796766 | down |
| CYP24A1 | -0.883428634 | 0.179876947 | 0.305168162 | down |
| AP001625.6 | -0.883161115 | 0.136753102 | 0.252035984 | down |
| ERP29P1 | -0.883128212 | 0.089957025 | 0.18747703 | down |
| PLEKHA7 | -0.883087007 | 0.001540641 | 0.008516472 | down |
| NA | -0.883040835 | 0.094363737 | 0.194009724 | down |
| DSC3 | -0.882963114 | 0.097288406 | 0.198149848 | down |
| SLC10A4 | -0.88295766 | 0.142197487 | 0.259061385 | down |
| RP11-575A19.2 | -0.882873662 | 0.103959698 | 0.207587742 | down |
| MASP2 | -0.882733666 | 0.085162713 | 0.180299382 | down |
| NA | -0.882515126 | 0.074822953 | 0.164075507 | down |
| ACSL6 | -0.882511529 | 0.007295621 | 0.028863527 | down |
| HOOK1 | -0.882364821 | 0.001052423 | 0.006317479 | down |
| ZNF793 | -0.882350087 | 0.00426894 | 0.019177039 | down |
| RP11-401F2.3 | -0.882267952 | 0.029625989 | 0.083068896 | down |
| UGGT1 | -0.882201315 | 0.000129858 | 0.001174575 | down |
| LRRC14B | -0.882149177 | 0.277636668 | 0.413829262 | down |
| C11orf53 | -0.882101497 | 0.017130022 | 0.05515533 | down |
| RP11-553N16.1 | -0.882025234 | 0.379480188 | | down |
| RP11-549B18.1 | -0.881943121 | 0.010352653 | 0.037664 | down |
| RP11-510I5.4 | -0.881920073 | 0.712582167 | | down |
| STAB2 | -0.881894812 | 0.036291207 | 0.09666073 | down |

| | | | | |
|---|---|---|---|---|
| RHOXF1P3 | -0.881722805 | 0.116000565 | 0.224585705 | down |
| CTD-2666L21.3 | -0.881209711 | 0.075353167 | 0.164899887 | down |
| RP11-386I14.2 | -0.881109659 | 0.206889858 | 0.336936193 | down |
| GLRB | -0.881053325 | 0.122466417 | 0.233320285 | down |
| RP11-849F2.9 | -0.880792088 | 0.029790598 | 0.083351146 | down |
| IGLVI-63 | -0.880715213 | 0.65132128 | | down |
| BORCS6 | -0.880466076 | 0.000935029 | 0.005744787 | down |
| CCDC144NL | -0.880226754 | 0.510678928 | 0.636500216 | down |
| OSBPL7 | -0.880013564 | 6.00E-06 | 9.23E-05 | down |
| SLC17A7 | -0.879861777 | 0.011489485 | 0.040816438 | down |
| CTD-2026K11.6 | -0.879764507 | 0.017609214 | 0.056426113 | down |
| RP1-265C24.8 | -0.879699579 | 0.237252897 | | down |
| RP5-1112D6.7 | -0.879681182 | 0.046596015 | 0.116083028 | down |
| RP11-510C10.2 | -0.879535091 | 0.763665934 | | down |
| ADGRE4P | -0.879296135 | 0.059921793 | 0.13956821 | down |
| SAMD5 | -0.879232463 | 0.000968585 | 0.005903869 | down |
| CADPS | -0.879127023 | 0.002105855 | 0.010925284 | down |
| RYR3 | -0.879015519 | 0.016471735 | 0.053568768 | down |
| NA | -0.878969042 | 0.007694819 | 0.030082386 | down |
| ECI1 | -0.878697413 | 1.01E-05 | 0.00014265 | down |
| CTD-2017C7.2 | -0.878584058 | 0.050633126 | 0.123365634 | down |
| CCND2P1 | -0.878536055 | 0.746855695 | | down |
| PRR5L | -0.878184186 | 1.09E-05 | 0.000151053 | down |
| PROKR1 | -0.878010551 | 0.28300057 | 0.419543527 | down |
| RP11-219A15.5 | -0.877922445 | 0.656101837 | | down |
| CTD-3154N5.1 | -0.877620491 | 0.687792116 | | down |
| RP11-145E17.2 | -0.87762027 | 0.719175602 | | down |
| GZMAP1 | -0.877620259 | 0.720462293 | | down |
| MIR5687 | -0.877620231 | 0.723838249 | | down |
| RP11-7C6.1 | -0.877619902 | 0.755383772 | | down |
| IGFL1P1 | -0.877619897 | 0.75581407 | | down |
| RP11-457M11.6 | -0.877619896 | 0.755852968 | | down |
| RP11-57A19.6 | -0.877619878 | 0.757262682 | | down |
| RNU4ATAC11P | -0.877619874 | 0.757625613 | | down |
| LINC00945 | -0.877619864 | 0.75834767 | | down |
| NA | -0.87761986 | 0.758695497 | | down |
| SUCLA2P1 | -0.877619856 | 0.758999007 | | down |
| RN7SL459P | -0.877619851 | 0.75935501 | | down |
| RPL7P36 | -0.877619838 | 0.760381356 | | down |
| ANKRD30BP1 | -0.877619835 | 0.760552008 | | down |
| RP13-436F16.1 | -0.877619786 | 0.764212201 | | down |
| RP4-612C19.2 | -0.877619786 | 0.764212201 | | down |
| RNU6-1116P | -0.877619786 | 0.764212201 | | down |
| TNP1 | -0.877619786 | 0.764212201 | | down |
| NA | -0.877619786 | 0.764212201 | | down |
| RP1-303A1.1 | -0.877619786 | 0.764212201 | | down |
| PRELID1P2 | -0.877619786 | 0.764212201 | | down |
| PPIAP9 | -0.877619786 | 0.764212201 | | down |
| CICP20 | -0.877619786 | 0.764212201 | | down |
| KCTD9P3 | -0.877619786 | 0.764212201 | | down |
| RP11-94M14.2 | -0.877619786 | 0.764212201 | | down |

| | | | | |
|---|---|---|---|---|
| RP11-735A19.2 | -0.877619786 | 0.764212201 | | down |
| RNU6-876P | -0.877619786 | 0.764212201 | | down |
| NA | -0.877619786 | 0.764212201 | | down |
| RP11-126O1.2 | -0.877619786 | 0.764212201 | | down |
| RP4-620E11.4 | -0.877619786 | 0.764212201 | | down |
| EDF1 | -0.877416003 | 9.13E-05 | 0.000876955 | down |
| C21orf62 | -0.877396084 | 0.092514178 | 0.191175181 | down |
| NA | -0.877363727 | 0.266767188 | 0.402488494 | down |
| RAD51B | -0.877294031 | 0.002922684 | 0.014206295 | down |
| RP3-416J7.4 | -0.87703705 | 0.308619041 | 0.447066078 | down |
| CDC14A | -0.876986937 | 0.002652123 | 0.013163786 | down |
| NA | -0.876829146 | 0.653740771 | | down |
| COA3 | -0.876646156 | 9.78E-05 | 0.00092777 | down |
| MAFA-AS1 | -0.876485105 | 0.32368486 | 0.462094937 | down |
| NA | -0.876472311 | 0.687458062 | | down |
| GS1-279B7.1 | -0.87645521 | 0.470110636 | 0.6007658 | down |
| NA | -0.876089109 | 0.203539041 | 0.332959106 | down |
| ITPK1 | -0.876048814 | 5.42E-09 | 2.29E-07 | down |
| CTD-2206G10.2 | -0.875953145 | 0.065666609 | 0.14908846 | down |
| OTX2P1 | -0.875561225 | 0.563244323 | 0.682058768 | down |
| CTD-2171N6.1 | -0.875406443 | 0.285757112 | 0.422812439 | down |
| PENK | -0.875085595 | 0.200683669 | 0.329753575 | down |
| PPP2R3A | -0.875079449 | 0.000135246 | 0.001213804 | down |
| TBC1D3B | -0.874978461 | 0.367729167 | | down |
| RAC1P2 | -0.874859039 | 0.008568107 | 0.032568345 | down |
| GRAMD1B | -0.874831326 | 0.001117647 | 0.006633465 | down |
| RP11-773H22.4 | -0.874640136 | 0.158135736 | 0.279045581 | down |
| FAM90A28P | -0.874621642 | 0.717292512 | | down |
| C11orf88 | -0.874614964 | 0.255160022 | | down |
| NA | -0.87453846 | 0.109432574 | 0.215691941 | down |
| TDRD15 | -0.87451465 | 0.488953243 | | down |
| EMID1 | -0.874464209 | 0.005255223 | 0.022542536 | down |
| RP11-131H24.4 | -0.874406373 | 0.148955072 | 0.267671881 | down |
| PRR13P5 | -0.874400604 | 0.007668411 | 0.030006477 | down |
| SFTPD | -0.874117251 | 0.05135218 | 0.124713029 | down |
| DRP2 | -0.873908313 | 0.015824518 | 0.051999543 | down |
| CTA-313A17.3 | -0.873852072 | 0.314165681 | 0.452826743 | down |
| NA | -0.873841391 | 0.363218717 | 0.501633867 | down |
| CTD-2410N18.3 | -0.873836717 | 0.041191242 | 0.106181867 | down |
| NA | -0.87383269 | 0.027209012 | 0.078114434 | down |
| RP11-440D17.4 | -0.873641432 | 0.097677521 | 0.198701567 | down |
| KCNC1 | -0.873275402 | 0.078754091 | 0.170222151 | down |
| PHLDB3 | -0.873217233 | 2.15E-07 | 5.47E-06 | down |
| REP15 | -0.873168072 | 0.030556361 | 0.084940352 | down |
| BACH1-IT1 | -0.873105653 | 0.111062665 | 0.217904179 | down |
| TMEM8B | -0.872936109 | 2.44E-07 | 6.13E-06 | down |
| BCAT2 | -0.872888203 | 1.97E-05 | 0.000246678 | down |
| CTD-2015B23.2 | -0.872814862 | 0.471752824 | | down |
| MIR27B | -0.87266497 | 0.35393193 | 0.49254393 | down |
| AKR7A2P1 | -0.872551821 | 0.406896159 | | down |
| RP1-102E24.10 | -0.872518208 | 0.012779867 | 0.044193731 | down |

| | | | | |
|---|---|---|---|---|
| AC007381.3 | -0.872444404 | 0.112683118 | 0.219891635 | down |
| RP11-182L21.5 | -0.872436573 | 0.395521744 | 0.532849513 | down |
| KRTAP5-1 | -0.872257435 | 0.068996087 | 0.15466078 | down |
| TP53I13 | -0.872186419 | 0.000274088 | 0.002169311 | down |
| CTD-2623H2.7 | -0.872166564 | 0.685247082 | | down |
| RP11-523O18.7 | -0.872057933 | 0.473294829 | 0.603705097 | down |
| POF1B | -0.871903139 | 0.008965833 | 0.033738662 | down |
| RP11-690I21.1 | -0.871770204 | 0.558502493 | | down |
| RP11-110I1.5 | -0.87148367 | 0.14422942 | 0.261756051 | down |
| Y_RNA | -0.87124521 | 0.519961877 | 0.644487587 | down |
| GATSL3 | -0.871235457 | 0.096177655 | 0.196568807 | down |
| AKAP9 | -0.871192368 | 4.18E-05 | 0.000460501 | down |
| MROH8 | -0.871191326 | 0.000853977 | 0.00534407 | down |
| SAP30L-AS1 | -0.870799485 | 0.003459117 | 0.016223181 | down |
| WDR97 | -0.870764205 | 0.007581682 | 0.029740706 | down |
| RP3-514P16.1 | -0.870726773 | 0.751165885 | | down |
| ASB13 | -0.870652071 | 3.64E-06 | 6.09E-05 | down |
| RP1 | -0.870486552 | 0.221133365 | 0.352765578 | down |
| ANKRD24 | -0.870423376 | 7.73E-05 | 0.000762239 | down |
| AC010884.1 | -0.87028474 | 0.434883846 | | down |
| ANKK1 | -0.869943019 | 0.01209616 | 0.042445343 | down |
| SH3GL1P2 | -0.869933393 | 0.090582419 | 0.188387888 | down |
| RP11-552F3.9 | -0.869823856 | 0.006832817 | 0.027499686 | down |
| ALDH7A1P1 | -0.869644403 | 0.288005717 | | down |
| CEACAM16 | -0.869557332 | 0.251952618 | 0.386819176 | down |
| ST13P6 | -0.869521716 | 0.143997362 | 0.261436835 | down |
| NA | -0.869410497 | 0.272578495 | 0.408813314 | down |
| LDHAL6B | -0.869270794 | 0.275082325 | 0.411379406 | down |
| TNNT3 | -0.869238395 | 0.012677436 | 0.04396132 | down |
| NAPRT | -0.86918999 | 1.61E-05 | 0.000210761 | down |
| AC079135.1 | -0.869180353 | 0.686216153 | | down |
| NPBWR1 | -0.869170667 | 0.418904287 | 0.554894422 | down |
| RP11-567M16.3 | -0.869061425 | 0.370245152 | | down |
| RP11-16K12.1 | -0.869038636 | 0.139233394 | 0.255364634 | down |
| NA | -0.868751422 | 0.162792918 | 0.284846547 | down |
| RP3-512B11.3 | -0.868687637 | 0.00155373 | 0.008577779 | down |
| CTD-2014D20.1 | -0.868637295 | 0.150897767 | 0.270144948 | down |
| WASH1 | -0.868421876 | 0.001051753 | 0.006314765 | down |
| RPS17P5 | -0.868212069 | 0.547428941 | | down |
| RP11-91I8.1 | -0.868211867 | 0.619985633 | | down |
| RP11-811O14.1 | -0.868211865 | 0.620527688 | | down |
| RP11-424E7.3 | -0.868211537 | 0.687745475 | | down |
| AC007383.6 | -0.868211527 | 0.68928841 | | down |
| MIR1910 | -0.868211524 | 0.689777269 | | down |
| PASD1 | -0.868211522 | 0.690081867 | | down |
| AC005197.2 | -0.868211268 | 0.722446911 | | down |
| OR1E1 | -0.868211245 | 0.724921883 | | down |
| RP11-416N4.4 | -0.868211245 | 0.724905066 | | down |
| CTD-2631K10.1 | -0.86821121 | 0.728477401 | | down |
| RP11-400N13.2 | -0.868210857 | 0.758428242 | | down |
| CTB-31N19.4 | -0.86821085 | 0.758938095 | | down |

| | | | | |
|---|---|---|---|---|
| AC117490.2 | -0.868210849 | 0.758993161 | | down |
| CTD-2235C13.2 | -0.868210833 | 0.760174275 | | down |
| MIR4284 | -0.868210809 | 0.761808389 | | down |
| RP11-365D9.1 | -0.868210736 | 0.766666377 | | down |
| RP11-416L21.1 | -0.868210736 | 0.766666377 | | down |
| AC114752.3 | -0.868210736 | 0.766666377 | | down |
| DNAJB8 | -0.868210736 | 0.766666377 | | down |
| RP1-290I10.3 | -0.868210736 | 0.766666377 | | down |
| PRR13P7 | -0.868210736 | 0.766666377 | | down |
| RP11-736I10.2 | -0.868210736 | 0.766666377 | | down |
| RP11-277J24.1 | -0.868210736 | 0.766666377 | | down |
| AC069368.3 | -0.868210736 | 0.766666377 | | down |
| RP11-288C17.1 | -0.868210736 | 0.766666377 | | down |
| AC011516.1 | -0.868210736 | 0.766666377 | | down |
| IFNL2 | -0.868210736 | 0.766666377 | | down |
| RP11-266I3.1 | -0.868210736 | 0.766666377 | | down |
| RP11-231N9.1 | -0.868210736 | 0.766666377 | | down |
| CTD-3020H12.3 | -0.868084466 | 0.76584437 | | down |
| RPS26P56 | -0.868076365 | 0.541817646 | 0.663310795 | down |
| SHISA2 | -0.867937096 | 0.016370658 | 0.053331717 | down |
| RASGRF1 | -0.867919043 | 0.006175168 | 0.025505463 | down |
| AGR3 | -0.867811183 | 0.012056212 | 0.042329884 | down |
| DDTL | -0.867222376 | 0.014884142 | 0.049663277 | down |
| SERPINH1P1 | -0.867020416 | 0.688911411 | | down |
| RP11-141C7.3 | -0.867006615 | 0.100797808 | 0.203123572 | down |
| ARRB1 | -0.866916226 | 2.43E-06 | 4.35E-05 | down |
| LINC01423 | -0.866872524 | 0.543453257 | | down |
| TOB2P1 | -0.86685408 | 0.056933139 | 0.134485002 | down |
| RP4-591B8.2 | -0.866694103 | 0.631169297 | | down |
| ANG | -0.86666004 | 0.004695822 | 0.020668221 | down |
| ISOC2 | -0.866548917 | 0.000363626 | 0.002713556 | down |
| CCDC150 | -0.866492219 | 0.004173224 | 0.018854188 | down |
| SPPL2B | -0.866409878 | 6.05E-05 | 0.000623651 | down |
| MAMSTR | -0.866075434 | 0.001040294 | 0.006263444 | down |
| GOLGA2P5 | -0.865877294 | 6.51E-05 | 0.000662014 | down |
| MAP6D1 | -0.865802769 | 0.091257325 | 0.189330292 | down |
| DRAXIN | -0.865707213 | 0.032600357 | 0.089178286 | down |
| NA | -0.865693067 | 0.086062509 | 0.181768859 | down |
| MTCYBP21 | -0.865586267 | 0.65909651 | | down |
| LINC00540 | -0.864961483 | 0.0509493 | 0.123985159 | down |
| COQ4 | -0.864870062 | 1.93E-06 | 3.59E-05 | down |
| RP11-538C21.2 | -0.864608592 | 0.684293805 | | down |
| RP11-21M24.2 | -0.864456266 | 0.391050181 | 0.528649073 | down |
| Metazoa_SRP | -0.864305873 | 0.620388971 | 0.730653322 | down |
| NA | -0.86427807 | 0.325896592 | 0.464413851 | down |
| RPSAP1 | -0.864013644 | 0.59453828 | | down |
| RP5-940J5.8 | -0.864001868 | 0.300250559 | | down |
| ENTPD3-AS1 | -0.86397791 | 7.63E-06 | 0.000112888 | down |
| HEATR9 | -0.863911686 | 0.128301006 | 0.241067881 | down |
| BBOX1 | -0.863906312 | 0.291177271 | 0.42826308 | down |
| JUP | -0.863729159 | 0.000269127 | 0.002137927 | down |

| | | | | |
|---|---|---|---|---|
| RP11-1114A5.4 | -0.86364099 | 0.008821527 | 0.03332503 | down |
| HNRNPA1P26 | -0.86337408 | 0.226133186 | 0.35819821 | down |
| RP11-217B7.2 | -0.86331205 | 0.264502943 | 0.399958234 | down |
| NA | -0.863302768 | 0.767906069 | | down |
| RP11-1143G9.5 | -0.863244551 | 0.061572822 | 0.142507808 | down |
| GPLD1 | -0.863080246 | 0.023785095 | 0.070591662 | down |
| APC2 | -0.863000566 | 0.000927191 | 0.005711815 | down |
| NA | -0.862969957 | 0.441415591 | | down |
| RP11-727A23.4 | -0.862897553 | 0.078824606 | 0.170313302 | down |
| RP11-20G13.5 | -0.862859542 | 0.145984559 | 0.263912395 | down |
| RP11-425D17.1 | -0.862811022 | 0.195930043 | 0.324457787 | down |
| DUSP15 | -0.862678719 | 0.006337659 | 0.026005001 | down |
| TMEM106C | -0.862647302 | 2.68E-06 | 4.74E-05 | down |
| NA | -0.862638302 | 0.42572194 | | down |
| ADGRG2 | -0.862637616 | 0.062388591 | 0.143852864 | down |
| LINC00640 | -0.862546218 | 0.114797925 | 0.222932509 | down |
| NPAS3 | -0.86240957 | 0.004499425 | 0.01999715 | down |
| LINC00271 | -0.862302445 | 0.071789056 | 0.15928147 | down |
| RANBP20P | -0.862264224 | 0.690140313 | | down |
| NA | -0.861643243 | 0.646136609 | 0.75108777 | down |
| GPR52 | -0.861642267 | 0.360019326 | | down |
| RDM1 | -0.861630324 | 0.009303509 | 0.034696452 | down |
| PTOV1-AS1 | -0.861550648 | 0.023675973 | 0.070323375 | down |
| bP-2171C21.4 | -0.86146485 | 0.315404771 | 0.454071634 | down |
| KLHDC7A | -0.861136075 | 0.163781528 | 0.286084431 | down |
| FLRT1 | -0.86103415 | 0.088558146 | 0.185397599 | down |
| SDC4 | -0.861031231 | 0.000954208 | 0.005833991 | down |
| CTC-1337H24.3 | -0.860963194 | 0.20688794 | 0.336936193 | down |
| RP11-399H11.2 | -0.860723449 | 0.416800824 | | down |
| RP11-803B1.8 | -0.860644756 | 0.002868075 | 0.01399975 | down |
| NA | -0.86043972 | 0.626483707 | | down |
| CHMP2A | -0.860405163 | 3.32E-05 | 0.000380018 | down |
| RP11-331F9.3 | -0.860064175 | 0.29031862 | 0.427502456 | down |
| NA | -0.860043265 | 0.292282021 | 0.429551487 | down |
| RPH3AL | -0.860006395 | 3.20E-05 | 0.000369855 | down |
| KLF5 | -0.85999226 | 0.002814161 | 0.013790353 | down |
| SETP1 | -0.859958655 | 0.668365193 | | down |
| RP11-144G6.12 | -0.859936806 | 0.273870672 | | down |
| ZNF768 | -0.859932817 | 1.30E-07 | 3.55E-06 | down |
| RP11-361L15.5 | -0.859880577 | 0.144286445 | 0.261756051 | down |
| SRCIN1 | -0.859854839 | 0.000470411 | 0.003314499 | down |
| RUNDC3A | -0.859832928 | 0.001943942 | 0.010235508 | down |
| EGF | -0.859713374 | 0.082467972 | 0.176156065 | down |
| MSH5 | -0.859676385 | 0.037942338 | 0.099872052 | down |
| CTD-3018017.6 | -0.859419788 | 0.383260539 | 0.520963646 | down |
| MRPS31P1 | -0.859378406 | 0.768971777 | | down |
| C3orf84 | -0.859378406 | 0.768971777 | | down |
| RCC2P4 | -0.859378406 | 0.768971777 | | down |
| RP11-119H12.6 | -0.859378406 | 0.768971777 | | down |
| NA | -0.859378406 | 0.768971777 | | down |
| ARF1P3 | -0.859378406 | 0.768971777 | | down |

| | | | | |
|---|---|---|---|---|
| LIPK | -0.859378406 | 0.768971777 | | down |
| OR52R1 | -0.859378406 | 0.768971777 | | down |
| Y_RNA | -0.859378406 | 0.768971777 | | down |
| NA | -0.859378406 | 0.768971777 | | down |
| CTD-2579N5.4 | -0.859378406 | 0.768971777 | | down |
| CTC-454I21.4 | -0.859378406 | 0.768971777 | | down |
| RP5-1069C8.3 | -0.859378406 | 0.768971777 | | down |
| CT47B1 | -0.859378406 | 0.768971777 | | down |
| IGHV3OR16-8 | -0.859377616 | 0.7615869 | | down |
| RN7SL128P | -0.859377349 | 0.759179545 | | down |
| RP11-1103G16.1 | -0.859377349 | 0.759182548 | | down |
| AC096554.1 | -0.859377245 | 0.758252157 | | down |
| RP11-118H15.1 | -0.859377208 | 0.757928163 | | down |
| BHLHE23 | -0.859372951 | 0.724038955 | | down |
| IGKV2OR22-3 | -0.859372876 | 0.723499181 | | down |
| RP11-249L12.1 | -0.859372759 | 0.722659385 | | down |
| MIR4682 | -0.859372325 | 0.719565009 | | down |
| RNF152P1 | -0.859369195 | 0.698595481 | | down |
| HCG9P5 | -0.859368769 | 0.695897786 | | down |
| RP11-92F20.1 | -0.859368354 | 0.693301995 | | down |
| AF131215.1 | -0.859368287 | 0.692885899 | | down |
| L22NC03-30E12.1 | -0.859368267 | 0.692766857 | | down |
| RP11-65M17.1 | -0.859368208 | 0.692397744 | | down |
| CTD-2509G16.1 | -0.859362725 | 0.66076263 | | down |
| RP11-82O19.2 | -0.859362703 | 0.660643501 | | down |
| RP11-10J21.3 | -0.859355958 | 0.626656235 | | down |
| TNNI2 | -0.859158048 | 0.006941328 | 0.027828298 | down |
| GARNL3 | -0.859100135 | 0.002612894 | 0.013020699 | down |
| PGAM4P2 | -0.859033192 | 0.721042974 | | down |
| HOOK3 | -0.859024068 | 0.001355932 | 0.007702704 | down |
| GTF2IRD1P1 | -0.858964883 | 0.224002894 | 0.356251281 | down |
| TSC22D1-AS1 | -0.858877965 | 0.026124925 | 0.075764773 | down |
| MIR6772 | -0.858814896 | 0.462542745 | | down |
| ATP5O | -0.858776012 | 0.001554753 | 0.008581849 | down |
| CTC-484P3.3 | -0.858695857 | 0.66490772 | | down |
| FUNDC2P4 | -0.85866129 | 0.528048131 | | down |
| PRORY | -0.858503412 | 0.373800524 | 0.512281896 | down |
| ACTBP13 | -0.858483695 | 0.285768519 | 0.422812439 | down |
| OSTCP4 | -0.858460856 | 0.497285281 | | down |
| RPLP2 | -0.858416605 | 0.001807619 | 0.009689368 | down |
| SCAND2P | -0.858257723 | 0.00793184 | 0.030768778 | down |
| HMGB3P4 | -0.858211095 | 0.360134264 | | down |
| TMCO2 | -0.858055694 | 0.347986977 | 0.486773477 | down |
| CNIH3 | -0.857821187 | 0.001443334 | 0.008087194 | down |
| SLC27A5 | -0.85778925 | 0.001266943 | 0.007310169 | down |
| RP11-38G5.4 | -0.857624877 | 0.197244863 | 0.325826041 | down |
| MTATP6P1 | -0.857623106 | 0.077635279 | 0.168434021 | down |
| LMO7DN-IT1 | -0.857622588 | 0.6273914 | | down |
| SNORD114-3 | -0.857593447 | 0.625348693 | | down |
| SLC35D2 | -0.8575773 | 7.75E-06 | 0.000114358 | down |
| AGMAT | -0.85743966 | 8.24E-05 | 0.000806387 | down |

| | | | | |
|---|---|---|---|---|
| OR2C1 | -0.857409566 | 0.325985068 | 0.464441877 | down |
| CTA-351J1.1 | -0.857380033 | 0.169271791 | 0.292814469 | down |
| DENND2A | -0.857192091 | 0.00015075 | 0.001325469 | down |
| RP3-339A18.6 | -0.85709481 | 0.103761413 | 0.207343392 | down |
| EYA2 | -0.856955892 | 0.012741678 | 0.04410451 | down |
| NA | -0.856744453 | 0.404358352 | 0.540969066 | down |
| AC016722.3 | -0.856731517 | 0.316088371 | 0.454728932 | down |
| NA | -0.856713078 | 0.420220328 | 0.555951895 | down |
| CTC-246B18.10 | -0.856533423 | 0.133839011 | 0.248086874 | down |
| RGPD4 | -0.856508186 | 0.52198433 | | down |
| NKD2 | -0.856322247 | 0.000471655 | 0.003320153 | down |
| SLC26A1 | -0.856264569 | 0.001872135 | 0.00996041 | down |
| KLRB1 | -0.856191389 | 0.000673073 | 0.004419099 | down |
| GPR45 | -0.856040458 | 0.469554791 | | down |
| AP001628.7 | -0.855716294 | 0.40898409 | | down |
| RP11-850A17.1 | -0.855703428 | 0.130442454 | 0.243947282 | down |
| MARC2 | -0.85543989 | 0.001482711 | 0.00825701 | down |
| DUSP8 | -0.855276218 | 6.40E-05 | 0.00065341 | down |
| RP11-605B16.1 | -0.855180375 | 0.480641414 | | down |
| FAH | -0.855173598 | 5.32E-06 | 8.34E-05 | down |
| ZNF702P | -0.854790606 | 0.004631808 | 0.020455393 | down |
| RP11-452L6.6 | -0.854719612 | 0.054378123 | 0.12998141 | down |
| AC084809.2 | -0.854598195 | 0.018667575 | 0.058937434 | down |
| MAGI1 | -0.854508775 | 1.06E-06 | 2.13E-05 | down |
| KCNK7 | -0.854375332 | 0.014580072 | 0.048856086 | down |
| TRAPPC5 | -0.854368727 | 0.008817141 | 0.033312648 | down |
| RP11-467I17.1 | -0.854367257 | 0.257613264 | 0.392640671 | down |
| RP11-197K3.1 | -0.854309565 | 0.256623222 | 0.391628388 | down |
| RP3-454G6.2 | -0.854236631 | 0.208052816 | 0.338280053 | down |
| CTD-2086L14.1 | -0.85400132 | 0.441448635 | | down |
| KRT82 | -0.853968428 | 0.722466298 | | down |
| CTD-3162L10.1 | -0.853825057 | 0.056017061 | 0.132905045 | down |
| AC079610.1 | -0.853820609 | 0.696544243 | | down |
| CTB-12A17.2 | -0.853690584 | 0.133856422 | 0.248092679 | down |
| COX6CP1 | -0.853572479 | 0.292891066 | | down |
| CTB-171A8.1 | -0.853553906 | 0.000420349 | 0.003026349 | down |
| ADAM11 | -0.853535245 | 0.018581574 | 0.058764718 | down |
| ADAMTS13 | -0.853329593 | 8.61E-06 | 0.000124824 | down |
| TRBV7-7 | -0.853277349 | 0.228634749 | 0.36096661 | down |
| RP11-74J13.8 | -0.853258742 | 0.012268081 | 0.042873353 | down |
| RPL9P7 | -0.853115506 | 0.010754212 | 0.038784646 | down |
| TMEM61 | -0.853023464 | 0.063670953 | 0.145924945 | down |
| PPP1R1B | -0.852994674 | 0.003580474 | 0.016672623 | down |
| RBP1 | -0.852859109 | 5.31E-05 | 0.000560533 | down |
| PRSS33 | -0.852796444 | 0.141438596 | 0.258193996 | down |
| HNRNPLP2 | -0.85267404 | 0.050039104 | 0.122384798 | down |
| RP11-393N4.2 | -0.852672708 | 0.025165703 | 0.073663132 | down |
| NRG2 | -0.852624993 | 0.016450883 | 0.053507667 | down |
| AMACR | -0.852604054 | 0.026657578 | 0.076906113 | down |
| DRD3 | -0.852470278 | 0.48959259 | | down |
| MUC20 | -0.852376007 | 0.00294869 | 0.014304934 | down |

| | | | | |
|---|---|---|---|---|
| HIST1H1E | -0.852329592 | 0.040649256 | 0.105154661 | down |
| DOK4 | -0.852255821 | 0.000227485 | 0.001860757 | down |
| CTD-2649C14.3 | -0.852201579 | 0.311758726 | 0.450376457 | down |
| CHCHD2P6 | -0.852124108 | 0.007798722 | 0.030389729 | down |
| MT1M | -0.851949092 | 0.022303733 | 0.067251441 | down |
| AC112721.1 | -0.851921668 | 0.412601993 | 0.549234128 | down |
| KRT18P5 | -0.851891319 | 0.212261208 | 0.342987991 | down |
| ERCC2 | -0.851764557 | 0.000182771 | 0.001553373 | down |
| SDHAF2 | -0.851593832 | 0.002749167 | 0.013551859 | down |
| SLC39A3 | -0.851416405 | 0.004016234 | 0.018304574 | down |
| FZD9 | -0.851354832 | 0.054326302 | 0.129878207 | down |
| RP11-308B5.2 | -0.851332358 | 0.771073074 | | down |
| RP11-664H17.1 | -0.851327814 | 0.7596016 | | down |
| RPL13P4 | -0.851327661 | 0.759228453 | | down |
| AC138517.4 | -0.851313519 | 0.727434692 | | down |
| LINC01149 | -0.851295977 | 0.693600756 | | down |
| NA | -0.851279196 | 0.665209006 | | down |
| UBE2V1P1 | -0.851278058 | 0.663395643 | | down |
| U40455.1 | -0.851276638 | 0.661149976 | | down |
| IGLV2-33 | -0.851276586 | 0.661068529 | | down |
| CTD-3239E11.2 | -0.851275948 | 0.660065515 | | down |
| CTD-2534I21.8 | -0.851273111 | 0.655656552 | | down |
| RP11-439A17.9 | -0.851262808 | 0.481213989 | | down |
| RP11-3K24.1 | -0.851254547 | 0.628501919 | | down |
| RP11-34H11.3 | -0.851251133 | 0.623794336 | | down |
| RP11-190I17.2 | -0.851231731 | 0.598476386 | | down |
| RP11-720D4.2 | -0.851230632 | 0.597109541 | | down |
| KLRC3 | -0.851172223 | 0.532541824 | | down |
| RP11-332O19.2 | -0.851063484 | 0.661038534 | | down |
| LINC01135 | -0.850936463 | 0.000167983 | 0.001448176 | down |
| GDF3 | -0.850842582 | 0.320631605 | 0.459373197 | down |
| AC010976.2 | -0.850822405 | 0.068451479 | 0.153830221 | down |
| UBE2Q2P1 | -0.850812944 | 0.086081857 | 0.181796254 | down |
| RPP25 | -0.850620047 | 9.65E-05 | 0.000917879 | down |
| RP11-107N7.1 | -0.850258962 | 0.72429746 | | down |
| CTAGE5 | -0.850173943 | 9.29E-05 | 0.000889131 | down |
| C1orf167 | -0.85008908 | 0.154014003 | 0.273735387 | down |
| ZNF716 | -0.850055441 | 0.694238586 | | down |
| AC021106.1 | -0.85001765 | 0.211178033 | 0.341687658 | down |
| KIAA1324 | -0.849854994 | 0.006018656 | 0.025013764 | down |
| RNASEH2A | -0.849842853 | 0.044809143 | 0.112864344 | down |
| BCO2 | -0.849836945 | 0.001376106 | 0.007794821 | down |
| Y_RNA | -0.849808422 | 0.354920307 | | down |
| ZZEF1 | -0.849606384 | 1.13E-05 | 0.000156436 | down |
| AC079741.2 | -0.849491946 | 0.410633062 | | down |
| AC003005.2 | -0.849405794 | 0.418003865 | 0.554117019 | down |
| C9orf172 | -0.849333935 | 2.90E-05 | 0.000340861 | down |
| PLCD1 | -0.849072181 | 1.89E-05 | 0.000238936 | down |
| MICALL2 | -0.849067024 | 2.23E-05 | 0.000272739 | down |
| IGHEP2 | -0.848893249 | 0.342123594 | 0.480518034 | down |
| WBP1LP2 | -0.848800035 | 0.074908922 | 0.164166871 | down |

| | | | | |
|---|---|---|---|---|
| NDUFB9 | -0.848791647 | 0.000580081 | 0.003924331 | down |
| RP11-51F16.9 | -0.84877172 | 0.055269127 | 0.131504368 | down |
| FAM181A-AS1 | -0.848769654 | 0.400397166 | 0.53739256 | down |
| CHL1-AS2 | -0.848595245 | 0.066063277 | 0.14979661 | down |
| RP1-16A9.1 | -0.848434998 | 0.464153338 | | down |
| PRR15L | -0.848097837 | 0.006480025 | 0.026437515 | down |
| RP11-88L20.1 | -0.848036412 | 0.603051228 | | down |
| RP11-157F20.3 | -0.847982119 | 0.338810268 | 0.477247676 | down |
| MAGI2 | -0.847717156 | 0.003468782 | 0.016258359 | down |
| TACO1 | -0.847197817 | 1.58E-05 | 0.000207809 | down |
| TLR8-AS1 | -0.846965357 | 0.489878112 | | down |
| NRAP | -0.846945001 | 0.100396993 | 0.20253322 | down |
| NA | -0.846895582 | 0.617997753 | | down |
| CSN3 | -0.846703299 | 0.469160736 | | down |
| NOL4L | -0.846601895 | 9.44E-08 | 2.70E-06 | down |
| EFNA4 | -0.846371959 | 5.69E-05 | 0.000594159 | down |
| TMEM72-AS1 | -0.846216638 | 0.047889218 | 0.118515953 | down |
| RP11-340I6.7 | -0.846072695 | 0.760742219 | | down |
| CHKB-AS1 | -0.846040225 | 0.001736628 | 0.009385728 | down |
| MRGPRG-AS1 | -0.846014384 | 0.724741598 | | down |
| NA | -0.845994203 | 0.772465524 | | down |
| FAM103A2P | -0.8459641 | 0.110667121 | 0.21728421 | down |
| BMS1P9 | -0.84596159 | 0.696400963 | | down |
| PSG1 | -0.845953393 | 0.692281824 | | down |
| HDGFL1 | -0.845928215 | 0.77242892 | | down |
| OR7E116P | -0.845928215 | 0.77242892 | | down |
| SNRPGP13 | -0.845928215 | 0.77242892 | | down |
| RP11-144G7.2 | -0.845886754 | 0.661043157 | | down |
| ZNF660 | -0.845845804 | 0.001355998 | 0.007702704 | down |
| HYMAI | -0.845738733 | 0.160351783 | 0.281718409 | down |
| RP11-493G17.4 | -0.845631807 | 0.632500724 | | down |
| RPS27AP2 | -0.845460521 | 0.632397197 | | down |
| PLAG1 | -0.845252944 | 0.006857772 | 0.027570575 | down |
| EFCAB12 | -0.845192079 | 0.066197972 | 0.150022771 | down |
| RPL13AP3 | -0.845031498 | 0.079610818 | 0.171530939 | down |
| FOXP1 | -0.845025895 | 0.000154965 | 0.001354209 | down |
| RP3-468K18.5 | -0.845019487 | 0.446743686 | | down |
| C20orf202 | -0.844968229 | 0.041685906 | 0.107208644 | down |
| NA | -0.844955576 | 0.726763203 | | down |
| RP11-342K6.3 | -0.844758504 | 0.571182856 | | down |
| AC002456.2 | -0.844743259 | 0.127225973 | 0.239542342 | down |
| CTD-3220F14.2 | -0.84465071 | 0.417691705 | | down |
| SH3RF2 | -0.844545339 | 0.000839144 | 0.005275425 | down |
| RP11-459E5.1 | -0.844402804 | 0.191147171 | 0.318851013 | down |
| CTD-2587H24.10 | -0.844361193 | 0.116447864 | 0.225207298 | down |
| AC005597.1 | -0.844152782 | 0.577003654 | 0.693780677 | down |
| PDK2 | -0.844116351 | 0.002278776 | 0.011680994 | down |
| FAM153A | -0.843701568 | 0.075781432 | 0.16553565 | down |
| GNRHR2P1 | -0.84369806 | 0.632798282 | | down |
| CTC-265F19.1 | -0.843679103 | 0.296063979 | 0.433519163 | down |
| RASGEF1C | -0.843561969 | 0.025255321 | 0.0738535 | down |

| | | | | |
|---|---|---|---|---|
| U3 | -0.84351495 | 0.10977392 | 0.216152028 | down |
| CTD-2571L23.8 | -0.84342464 | 0.193765988 | 0.321879096 | down |
| CMB9-94B1.2 | -0.843383331 | 0.195861662 | 0.324409712 | down |
| BCL2L2-PABPN1 | -0.843087646 | 0.203702662 | 0.333102189 | down |
| PTCH1 | -0.842935534 | 0.002493146 | 0.012542546 | down |
| CTD-3193013.12 | -0.842934875 | 0.27194402 | 0.408146975 | down |
| MIR3178 | -0.842860525 | 0.317308705 | 0.456047777 | down |
| DLEU2L | -0.842805937 | 0.332730266 | 0.471046466 | down |
| RP11-861A13.3 | -0.842730125 | 0.079680329 | 0.171643781 | down |
| FAM3A | -0.842576548 | 4.13E-06 | 6.79E-05 | down |
| RNU6-100P | -0.842572333 | 0.574808824 | | down |
| RTCA-AS1 | -0.842200159 | 0.000358664 | 0.002687206 | down |
| WFDC10B | -0.842125472 | 0.220609546 | 0.352306392 | down |
| AC022431.3 | -0.842090339 | 0.341745619 | | down |
| RP1-122K4.3 | -0.841917352 | 0.072769948 | 0.160875667 | down |
| KCNA2 | -0.8416792 | 0.050480785 | 0.12315426 | down |
| OR6A2 | -0.841326717 | 0.668420958 | | down |
| EIF4HP2 | -0.841292498 | 0.03021741 | 0.084224128 | down |
| TBL3 | -0.841260852 | 0.000418887 | 0.003017776 | down |
| C6orf25 | -0.840769736 | 0.050622522 | 0.123349801 | down |
| FAM81A | -0.840611118 | 0.000863154 | 0.005385795 | down |
| LINC00293 | -0.840459243 | 0.728959762 | 0.815735515 | down |
| GSX2 | -0.840355603 | 0.314834597 | 0.453458439 | down |
| RP11-446E24.3 | -0.840348291 | 0.337587153 | | down |
| TRPV1 | -0.840233851 | 4.80E-05 | 0.000517458 | down |
| MAPK8IP2 | -0.840094865 | 0.011161153 | 0.039910221 | down |
| AC008073.7 | -0.840093628 | 0.402704238 | | down |
| SMAP1 | -0.840014344 | 0.01085292 | 0.039050813 | down |
| RPS10P2 | -0.83976874 | 0.221571349 | | down |
| NA | -0.839707237 | 0.636479559 | | down |
| RP11-819M15.2 | -0.839683819 | 0.233064201 | | down |
| RNF219-AS1 | -0.839184705 | 0.467235386 | | down |
| RP11-338N10.1 | -0.839143256 | 0.383488469 | 0.521194588 | down |
| THA1P | -0.839116377 | 0.034815796 | 0.093694534 | down |
| RP11-25D3.1 | -0.838985696 | 0.637075026 | | down |
| LHFPL3 | -0.838834157 | 0.097198966 | 0.19808187 | down |
| AC098828.3 | -0.838765041 | 0.22489546 | 0.357025867 | down |
| PUS10 | -0.838550514 | 4.79E-05 | 0.000516213 | down |
| RASA4CP | -0.838268512 | 0.002482687 | 0.012500395 | down |
| MIR2052HG | -0.838205381 | 0.444334432 | 0.577854024 | down |
| ADORA2A-AS1 | -0.838167612 | 0.109311276 | 0.215580148 | down |
| RP5-1021I20.1 | -0.837861538 | 0.000641979 | 0.004252119 | down |
| RP11-2I17.1 | -0.837671152 | 0.664198192 | | down |
| HOMER2P1 | -0.837638327 | 0.588990733 | | down |
| NPIPB6 | -0.837400839 | 0.062558837 | 0.144123741 | down |
| RP11-171I2.3 | -0.837209922 | 0.546927485 | | down |
| NA | -0.836945125 | 0.249390668 | 0.383965011 | down |
| Metazoa_SRP | -0.836818841 | 0.385808184 | 0.523542054 | down |
| DLGAP4-AS1 | -0.836812073 | 0.025507294 | 0.074358738 | down |
| HSPE1P18 | -0.836625139 | 0.40448869 | | down |
| RP3-508I15.14 | -0.836536244 | 0.176254947 | 0.301097608 | down |

| | | | | |
|---|---|---|---|---|
| C6orf132 | -0.836499822 | 0.000167095 | 0.001441756 | down |
| RP5-875O13.7 | -0.836414047 | 0.451245561 | | down |
| RP11-736N17.10 | -0.836392937 | 0.199568233 | 0.328574528 | down |
| CTD-2016O11.1 | -0.836363138 | 0.418442042 | 0.554526608 | down |
| RP11-603J24.5 | -0.836255329 | 0.234705383 | 0.36753302 | down |
| PPP1R26-AS1 | -0.836156931 | 0.007808142 | 0.030414601 | down |
| LINC00659 | -0.83604043 | 0.150349457 | 0.269452344 | down |
| RP4-794H19.1 | -0.835986459 | 0.001233993 | 0.007165458 | down |
| SNORA71B | -0.835868505 | 0.270873342 | 0.406926281 | down |
| RP11-510M2.5 | -0.835812728 | 0.668512047 | | down |
| RPL12P33 | -0.835525387 | 0.076404134 | 0.166532633 | down |
| NA | -0.835486234 | 0.256336152 | 0.391404548 | down |
| MINOS1 | -0.835421451 | 4.37E-05 | 0.00047683 | down |
| CTA-363E6.3 | -0.835368738 | 0.472886682 | | down |
| S100A5 | -0.835330149 | 0.024710085 | 0.072655098 | down |
| TMEM8C | -0.835307526 | 0.775185905 | | down |
| AP000620.1 | -0.835148868 | 0.323330969 | | down |
| Y_RNA | -0.835080011 | 0.730344616 | | down |
| RP11-165E7.1 | -0.835000097 | 0.254646593 | | down |
| KB-1027C11.4 | -0.834878216 | 0.34555177 | | down |
| CHMP4A | -0.834682902 | 0.00921576 | 0.034459028 | down |
| RP11-669C19.1 | -0.834502218 | 0.354386667 | 0.493021787 | down |
| AC004453.8 | -0.834500789 | 0.001040628 | 0.006264199 | down |
| LINC01529 | -0.834499835 | 0.064975865 | 0.147951523 | down |
| NA | -0.834435708 | 0.205519867 | 0.335377703 | down |
| DPM3 | -0.834356386 | 0.001740984 | 0.009402503 | down |
| RP11-114F10.2 | -0.834307515 | 0.592880302 | | down |
| RP11-572M11.3 | -0.834292138 | 0.050392155 | 0.123011571 | down |
| SIRT4 | -0.834208304 | 0.007033509 | 0.028100367 | down |
| SPATA31E1 | -0.834157314 | 0.486068437 | | down |
| VPREB1 | -0.834149104 | 0.221082846 | 0.352714313 | down |
| AC011933.2 | -0.834141229 | 0.043471874 | 0.110571366 | down |
| NA | -0.833967982 | 0.584964814 | | down |
| RP11-402G3.5 | -0.833896881 | 0.206061383 | 0.335969403 | down |
| RP11-56F10.3 | -0.833719569 | 0.576691588 | | down |
| RP5-845O24.8 | -0.833712801 | 0.775684388 | | down |
| RN7SL304P | -0.833712801 | 0.775684388 | | down |
| MIR6127 | -0.833712801 | 0.775684388 | | down |
| Y_RNA | -0.833712801 | 0.775684388 | | down |
| CHMP1AP1 | -0.833712801 | 0.775684388 | | down |
| Y_RNA | -0.833712801 | 0.775684388 | | down |
| MTCO2P34 | -0.833712801 | 0.775684388 | | down |
| RP11-393I23.4 | -0.833712801 | 0.775684388 | | down |
| RNU6-1177P | -0.833712801 | 0.775684388 | | down |
| RNU6-371P | -0.833712801 | 0.775684388 | | down |
| CASP3P1 | -0.833712801 | 0.775684388 | | down |
| RP11-339A11.3 | -0.833712801 | 0.775684388 | | down |
| NA | -0.833712801 | 0.775684388 | | down |
| WDR82P2 | -0.833712801 | 0.775684388 | | down |
| CDK4P1 | -0.833712801 | 0.775684388 | | down |
| TXNP3 | -0.833712801 | 0.775684388 | | down |

| | | | |
|---|---|---|---|
| RP11-710N8.3 | -0.833712801 | 0.775684388 | down |
| RP4-630J13.1 | -0.833712801 | 0.775684388 | down |
| OR13Z1P | -0.833712801 | 0.775684388 | down |
| RP1-43O17.2 | -0.833712801 | 0.775684388 | down |
| AL591704.7 | -0.833712801 | 0.775684388 | down |
| NA | -0.833712801 | 0.775684388 | down |
| OR10R1P | -0.833712801 | 0.775684388 | down |
| OR10J4 | -0.833712801 | 0.775684388 | down |
| RP11-180D21.3 | -0.833712801 | 0.775684388 | down |
| RP11-227F8.2 | -0.833712801 | 0.775684388 | down |
| ISCUP1 | -0.833712801 | 0.775684388 | down |
| RN7SL654P | -0.833712801 | 0.775684388 | down |
| GAPDHP75 | -0.833712801 | 0.775684388 | down |
| RNU6-983P | -0.833712801 | 0.775684388 | down |
| RP11-567E21.3 | -0.833712801 | 0.775684388 | down |
| RPL10P4 | -0.833712801 | 0.775684388 | down |
| C4BPAP1 | -0.833712801 | 0.775684388 | down |
| U3 | -0.833712801 | 0.775684388 | down |
| RN7SL464P | -0.833712801 | 0.775684388 | down |
| FAM166AP5 | -0.833712801 | 0.775684388 | down |
| RP5-1068B5.1 | -0.833712801 | 0.775684388 | down |
| RP11-278H7.3 | -0.833712801 | 0.775684388 | down |
| SNORA25 | -0.833712801 | 0.775684388 | down |
| Six3os1_1 | -0.833712801 | 0.775684388 | down |
| RP11-429P7.1 | -0.833712801 | 0.775684388 | down |
| RNU4-49P | -0.833712801 | 0.775684388 | down |
| TCEB2P3 | -0.833712801 | 0.775684388 | down |
| RNU6-439P | -0.833712801 | 0.775684388 | down |
| RP11-358H12.1 | -0.833712801 | 0.775684388 | down |
| SCARNA16 | -0.833712801 | 0.775684388 | down |
| NA | -0.833712801 | 0.775684388 | down |
| snoU109 | -0.833712801 | 0.775684388 | down |
| LYARP1 | -0.833712801 | 0.775684388 | down |
| AC098817.5 | -0.833712801 | 0.775684388 | down |
| RP11-241M13.2 | -0.833712801 | 0.775684388 | down |
| IGKV3-25 | -0.833712801 | 0.775684388 | down |
| SLC2AXP1 | -0.833712801 | 0.775684388 | down |
| AC018880.2 | -0.833712801 | 0.775684388 | down |
| RPL23AP27 | -0.833712801 | 0.775684388 | down |
| MTCO1P43 | -0.833712801 | 0.775684388 | down |
| AC079988.3 | -0.833712801 | 0.775684388 | down |
| RP11-329A6.1 | -0.833712801 | 0.775684388 | down |
| RN7SKP102 | -0.833712801 | 0.775684388 | down |
| NA | -0.833712801 | 0.775684388 | down |
| VDAC2P4 | -0.833712801 | 0.775684388 | down |
| AC010740.1 | -0.833712801 | 0.775684388 | down |
| AC012501.2 | -0.833712801 | 0.775684388 | down |
| AC062022.1 | -0.833712801 | 0.775684388 | down |
| AC093639.1 | -0.833712801 | 0.775684388 | down |
| AC106883.1 | -0.833712801 | 0.775684388 | down |
| MTCO2P17 | -0.833712801 | 0.775684388 | down |

| | | | |
|---|---|---|---|
| RNU6-474P | -0.833712801 | 0.775684388 | down |
| AC106901.1 | -0.833712801 | 0.775684388 | down |
| OR5S1P | -0.833712801 | 0.775684388 | down |
| PNPT1P1 | -0.833712801 | 0.775684388 | down |
| RN7SL553P | -0.833712801 | 0.775684388 | down |
| ATP2B2-IT1 | -0.833712801 | 0.775684388 | down |
| AC093142.2 | -0.833712801 | 0.775684388 | down |
| MTND4LP9 | -0.833712801 | 0.775684388 | down |
| RP11-134J21.1 | -0.833712801 | 0.775684388 | down |
| HMGN2P24 | -0.833712801 | 0.775684388 | down |
| RP11-229A12.2 | -0.833712801 | 0.775684388 | down |
| RNU2-10P | -0.833712801 | 0.775684388 | down |
| RP11-1036F1.1 | -0.833712801 | 0.775684388 | down |
| CCDC75P1 | -0.833712801 | 0.775684388 | down |
| U3 | -0.833712801 | 0.775684388 | down |
| RNU6-488P | -0.833712801 | 0.775684388 | down |
| OR5K1 | -0.833712801 | 0.775684388 | down |
| RP11-227H4.1 | -0.833712801 | 0.775684388 | down |
| RP11-779P15.1 | -0.833712801 | 0.775684388 | down |
| RNU1-43P | -0.833712801 | 0.775684388 | down |
| NA | -0.833712801 | 0.775684388 | down |
| RP11-457K10.2 | -0.833712801 | 0.775684388 | down |
| LINC00903 | -0.833712801 | 0.775684388 | down |
| NIP7P2 | -0.833712801 | 0.775684388 | down |
| RNU6-1294P | -0.833712801 | 0.775684388 | down |
| LARP7P4 | -0.833712801 | 0.775684388 | down |
| RP11-167H9.1 | -0.833712801 | 0.775684388 | down |
| GPR87 | -0.833712801 | 0.775684388 | down |
| Y_RNA | -0.833712801 | 0.775684388 | down |
| RP11-290K4.1 | -0.833712801 | 0.775684388 | down |
| RP11-12N13.1 | -0.833712801 | 0.775684388 | down |
| PSAT1P4 | -0.833712801 | 0.775684388 | down |
| RN7SKP36 | -0.833712801 | 0.775684388 | down |
| RNA5SP156 | -0.833712801 | 0.775684388 | down |
| AC006160.8 | -0.833712801 | 0.775684388 | down |
| RNA5SP157 | -0.833712801 | 0.775684388 | down |
| RNU7-126P | -0.833712801 | 0.775684388 | down |
| RP11-457P14.6 | -0.833712801 | 0.775684388 | down |
| MIR8053 | -0.833712801 | 0.775684388 | down |
| TPI1P4 | -0.833712801 | 0.775684388 | down |
| RP11-241F15.3 | -0.833712801 | 0.775684388 | down |
| Y_RNA | -0.833712801 | 0.775684388 | down |
| RP11-25L5.2 | -0.833712801 | 0.775684388 | down |
| RNU2-40P | -0.833712801 | 0.775684388 | down |
| RP13-644M16.2 | -0.833712801 | 0.775684388 | down |
| HNRNPA1P55 | -0.833712801 | 0.775684388 | down |
| HIGD1AP13 | -0.833712801 | 0.775684388 | down |
| RP11-10L12.1 | -0.833712801 | 0.775684388 | down |
| RN7SL808P | -0.833712801 | 0.775684388 | down |
| RP11-27G13.5 | -0.833712801 | 0.775684388 | down |
| MTND4P8 | -0.833712801 | 0.775684388 | down |

| | | | |
|---|---|---|---|
| MTCO2P9 | -0.833712801 | 0.775684388 | down |
| RP11-138A23.1 | -0.833712801 | 0.775684388 | down |
| RNU6ATAC13P | -0.833712801 | 0.775684388 | down |
| RP11-624A4.1 | -0.833712801 | 0.775684388 | down |
| RN7SKP67 | -0.833712801 | 0.775684388 | down |
| MIR4455 | -0.833712801 | 0.775684388 | down |
| RP11-661C8.3 | -0.833712801 | 0.775684388 | down |
| RP11-468D11.1 | -0.833712801 | 0.775684388 | down |
| CTD-2383I20.1 | -0.833712801 | 0.775684388 | down |
| CTD-2215L10.1 | -0.833712801 | 0.775684388 | down |
| RP11-42L13.3 | -0.833712801 | 0.775684388 | down |
| RNU6-363P | -0.833712801 | 0.775684388 | down |
| CTD-2233C11.3 | -0.833712801 | 0.775684388 | down |
| RP11-1325J9.1 | -0.833712801 | 0.775684388 | down |
| RP11-301A5.2 | -0.833712801 | 0.775684388 | down |
| RPSAP38 | -0.833712801 | 0.775684388 | down |
| NA | -0.833712801 | 0.775684388 | down |
| RP11-45H22.2 | -0.833712801 | 0.775684388 | down |
| CTD-2313F11.2 | -0.833712801 | 0.775684388 | down |
| CTD-2060F24.1 | -0.833712801 | 0.775684388 | down |
| CTC-422A18.2 | -0.833712801 | 0.775684388 | down |
| RPS3AP22 | -0.833712801 | 0.775684388 | down |
| AC008592.7 | -0.833712801 | 0.775684388 | down |
| RP11-274E7.1 | -0.833712801 | 0.775684388 | down |
| ARL2BPP4 | -0.833712801 | 0.775684388 | down |
| CTB-47B8.4 | -0.833712801 | 0.775684388 | down |
| RNU6-164P | -0.833712801 | 0.775684388 | down |
| RN7SL339P | -0.833712801 | 0.775684388 | down |
| RP11-619L12.3 | -0.833712801 | 0.775684388 | down |
| NA | -0.833712801 | 0.775684388 | down |
| HIST1H1PS2 | -0.833712801 | 0.775684388 | down |
| VN1R13P | -0.833712801 | 0.775684388 | down |
| Xbac-BCX196D17. | -0.833712801 | 0.775684388 | down |
| RNU7-65P | -0.833712801 | 0.775684388 | down |
| RP11-448N11.1 | -0.833712801 | 0.775684388 | down |
| NA | -0.833712801 | 0.775684388 | down |
| OOEP-AS1 | -0.833712801 | 0.775684388 | down |
| LAP3P1 | -0.833712801 | 0.775684388 | down |
| DUTP5 | -0.833712801 | 0.775684388 | down |
| RP1-263J7.2 | -0.833712801 | 0.775684388 | down |
| RNU6-1144P | -0.833712801 | 0.775684388 | down |
| RP11-249L21.2 | -0.833712801 | 0.775684388 | down |
| RN7SL617P | -0.833712801 | 0.775684388 | down |
| RN7SKP51 | -0.833712801 | 0.775684388 | down |
| RNU4-76P | -0.833712801 | 0.775684388 | down |
| TAAR4P | -0.833712801 | 0.775684388 | down |
| FTH1P26 | -0.833712801 | 0.775684388 | down |
| RP5-899B16.2 | -0.833712801 | 0.775684388 | down |
| RP11-347L18.1 | -0.833712801 | 0.775684388 | down |
| RP11-302L19.1 | -0.833712801 | 0.775684388 | down |
| Y_RNA | -0.833712801 | 0.775684388 | down |

| | | | |
|---|---|---|---|
| AC006150.1 | -0.833712801 | 0.775684388 | down |
| Metazoa_SRP | -0.833712801 | 0.775684388 | down |
| RNA5SP228 | -0.833712801 | 0.775684388 | down |
| AC004485.3 | -0.833712801 | 0.775684388 | down |
| NA | -0.833712801 | 0.775684388 | down |
| TRGV11 | -0.833712801 | 0.775684388 | down |
| ELK1P1 | -0.833712801 | 0.775684388 | down |
| RN7SKP218 | -0.833712801 | 0.775684388 | down |
| RP11-806J6.1 | -0.833712801 | 0.775684388 | down |
| MTCO3P4 | -0.833712801 | 0.775684388 | down |
| RP11-548K12.7 | -0.833712801 | 0.775684388 | down |
| CICP24 | -0.833712801 | 0.775684388 | down |
| RP13-157F18.2 | -0.833712801 | 0.775684388 | down |
| Y_RNA | -0.833712801 | 0.775684388 | down |
| RPL10P11 | -0.833712801 | 0.775684388 | down |
| MIR653 | -0.833712801 | 0.775684388 | down |
| RNU6-532P | -0.833712801 | 0.775684388 | down |
| RNU7-154P | -0.833712801 | 0.775684388 | down |
| RNU6-102P | -0.833712801 | 0.775684388 | down |
| AC000374.1 | -0.833712801 | 0.775684388 | down |
| RNA5SP246 | -0.833712801 | 0.775684388 | down |
| AC093106.6 | -0.833712801 | 0.775684388 | down |
| AC009518.3 | -0.833712801 | 0.775684388 | down |
| AC083875.2 | -0.833712801 | 0.775684388 | down |
| TRBVB | -0.833712801 | 0.775684388 | down |
| SLC16A1P1 | -0.833712801 | 0.775684388 | down |
| ATP5F1P3 | -0.833712801 | 0.775684388 | down |
| NA | -0.833712801 | 0.775684388 | down |
| MIR7160 | -0.833712801 | 0.775684388 | down |
| RP11-281H11.1 | -0.833712801 | 0.775684388 | down |
| PRR23D1 | -0.833712801 | 0.775684388 | down |
| DEFB107A | -0.833712801 | 0.775684388 | down |
| RN7SL178P | -0.833712801 | 0.775684388 | down |
| RP11-16P20.2 | -0.833712801 | 0.775684388 | down |
| TPT1P8 | -0.833712801 | 0.775684388 | down |
| RP11-15G16.1 | -0.833712801 | 0.775684388 | down |
| RP11-101E19.8 | -0.833712801 | 0.775684388 | down |
| RP11-101E19.7 | -0.833712801 | 0.775684388 | down |
| RP11-738G5.1 | -0.833712801 | 0.775684388 | down |
| NA | -0.833712801 | 0.775684388 | down |
| RN7SKP135 | -0.833712801 | 0.775684388 | down |
| NA | -0.833712801 | 0.775684388 | down |
| RN7SL675P | -0.833712801 | 0.775684388 | down |
| HAUS1P3 | -0.833712801 | 0.775684388 | down |
| RP11-363E6.1 | -0.833712801 | 0.775684388 | down |
| CTB-118P15.3 | -0.833712801 | 0.775684388 | down |
| KB-1090H4.2 | -0.833712801 | 0.775684388 | down |
| MRPL57P7 | -0.833712801 | 0.775684388 | down |
| NA | -0.833712801 | 0.775684388 | down |
| RNU11-4P | -0.833712801 | 0.775684388 | down |
| RP11-240B13.2 | -0.833712801 | 0.775684388 | down |

| | | | |
|---|---|---|---|
| CTC-369M3.1 | -0.833712801 | 0.775684388 | down |
| RP11-252M18.3 | -0.833712801 | 0.775684388 | down |
| RP11-275A14.1 | -0.833712801 | 0.775684388 | down |
| RP11-48L13.1 | -0.833712801 | 0.775684388 | down |
| RN7SKP24 | -0.833712801 | 0.775684388 | down |
| YWHABP1 | -0.833712801 | 0.775684388 | down |
| SKP1P3 | -0.833712801 | 0.775684388 | down |
| CR848007.5 | -0.833712801 | 0.775684388 | down |
| RP11-452D2.1 | -0.833712801 | 0.775684388 | down |
| LYPLA2P3 | -0.833712801 | 0.775684388 | down |
| RN7SKP87 | -0.833712801 | 0.775684388 | down |
| OR13C8 | -0.833712801 | 0.775684388 | down |
| RP11-3J11.1 | -0.833712801 | 0.775684388 | down |
| RP11-428F18.2 | -0.833712801 | 0.775684388 | down |
| SNORA31 | -0.833712801 | 0.775684388 | down |
| RP5-1119O21.2 | -0.833712801 | 0.775684388 | down |
| RNA5SP299 | -0.833712801 | 0.775684388 | down |
| RP11-265G8.3 | -0.833712801 | 0.775684388 | down |
| RP11-183E9.2 | -0.833712801 | 0.775684388 | down |
| RP11-748L13.7 | -0.833712801 | 0.775684388 | down |
| RPL36AP55 | -0.833712801 | 0.775684388 | down |
| RPSAP10 | -0.833712801 | 0.775684388 | down |
| RP11-810B23.1 | -0.833712801 | 0.775684388 | down |
| AL031601.5 | -0.833712801 | 0.775684388 | down |
| ZNF32-AS3 | -0.833712801 | 0.775684388 | down |
| NEFMP1 | -0.833712801 | 0.775684388 | down |
| LINC00845 | -0.833712801 | 0.775684388 | down |
| RNU6-697P | -0.833712801 | 0.775684388 | down |
| RP11-399N22.3 | -0.833712801 | 0.775684388 | down |
| RP11-432J9.3 | -0.833712801 | 0.775684388 | down |
| NA | -0.833712801 | 0.775684388 | down |
| RP11-344L13.1 | -0.833712801 | 0.775684388 | down |
| RP11-399L7.3 | -0.833712801 | 0.775684388 | down |
| RNU6-271P | -0.833712801 | 0.775684388 | down |
| RNU6-1274P | -0.833712801 | 0.775684388 | down |
| Y_RNA | -0.833712801 | 0.775684388 | down |
| CCDC172 | -0.833712801 | 0.775684388 | down |
| NA | -0.833712801 | 0.775684388 | down |
| LINC01167 | -0.833712801 | 0.775684388 | down |
| COX6CP18 | -0.833712801 | 0.775684388 | down |
| OR52J1P | -0.833712801 | 0.775684388 | down |
| AC113331.9 | -0.833712801 | 0.775684388 | down |
| OR51A1P | -0.833712801 | 0.775684388 | down |
| OR51B6 | -0.833712801 | 0.775684388 | down |
| OR5P1P | -0.833712801 | 0.775684388 | down |
| RP11-583F24.8 | -0.833712801 | 0.775684388 | down |
| RP4-562D20.2 | -0.833712801 | 0.775684388 | down |
| NA | -0.833712801 | 0.775684388 | down |
| RP11-111A24.2 | -0.833712801 | 0.775684388 | down |
| RP11-707M1.9 | -0.833712801 | 0.775684388 | down |
| OR4C48P | -0.833712801 | 0.775684388 | down |

| | | | |
|---|---|---|---|
| OR4C46 | -0.833712801 | 0.775684388 | down |
| OR4A6P | -0.833712801 | 0.775684388 | down |
| OR4A17P | -0.833712801 | 0.775684388 | down |
| OR5J2 | -0.833712801 | 0.775684388 | down |
| NA | -0.833712801 | 0.775684388 | down |
| OR9Q2 | -0.833712801 | 0.775684388 | down |
| OR10W1 | -0.833712801 | 0.775684388 | down |
| OR5BC1P | -0.833712801 | 0.775684388 | down |
| OR7E145P | -0.833712801 | 0.775684388 | down |
| KRTAP5-14P | -0.833712801 | 0.775684388 | down |
| RP11-809N8.6 | -0.833712801 | 0.775684388 | down |
| CTD-2028E8.1 | -0.833712801 | 0.775684388 | down |
| AP000648.6 | -0.833712801 | 0.775684388 | down |
| MED28P5 | -0.833712801 | 0.775684388 | down |
| NA | -0.833712801 | 0.775684388 | down |
| RP11-361A21.3 | -0.833712801 | 0.775684388 | down |
| RP11-708L7.9 | -0.833712801 | 0.775684388 | down |
| NXPE2P1 | -0.833712801 | 0.775684388 | down |
| RNU1-21P | -0.833712801 | 0.775684388 | down |
| OR10D5P | -0.833712801 | 0.775684388 | down |
| OR8A2P | -0.833712801 | 0.775684388 | down |
| RNU6-321P | -0.833712801 | 0.775684388 | down |
| RN7SKP279 | -0.833712801 | 0.775684388 | down |
| NA | -0.833712801 | 0.775684388 | down |
| RPL23AP14 | -0.833712801 | 0.775684388 | down |
| RP11-133L14.2 | -0.833712801 | 0.775684388 | down |
| RP13-81N3.2 | -0.833712801 | 0.775684388 | down |
| RP11-4N23.1 | -0.833712801 | 0.775684388 | down |
| RNU6-254P | -0.833712801 | 0.775684388 | down |
| RNA5SP356 | -0.833712801 | 0.775684388 | down |
| RP11-1020M18.10 | -0.833712801 | 0.775684388 | down |
| RP11-641A6.3 | -0.833712801 | 0.775684388 | down |
| VDAC1P5 | -0.833712801 | 0.775684388 | down |
| RP11-335M9.1 | -0.833712801 | 0.775684388 | down |
| CCNG2P1 | -0.833712801 | 0.775684388 | down |
| RNU1-117P | -0.833712801 | 0.775684388 | down |
| Y_RNA | -0.833712801 | 0.775684388 | down |
| RP11-554D14.3 | -0.833712801 | 0.775684388 | down |
| NA | -0.833712801 | 0.775684388 | down |
| RN7SKP197 | -0.833712801 | 0.775684388 | down |
| RP3-446N13.2 | -0.833712801 | 0.775684388 | down |
| RP11-955H22.3 | -0.833712801 | 0.775684388 | down |
| RP13-507P19.1 | -0.833712801 | 0.775684388 | down |
| RP11-897M7.3 | -0.833712801 | 0.775684388 | down |
| ZNF965P | -0.833712801 | 0.775684388 | down |
| RP11-101P17.14 | -0.833712801 | 0.775684388 | down |
| MIR2276 | -0.833712801 | 0.775684388 | down |
| RPL26P34 | -0.833712801 | 0.775684388 | down |
| RP11-676B18.1 | -0.833712801 | 0.775684388 | down |
| RNA5SP27 | -0.833712801 | 0.775684388 | down |
| RP11-78L16.1 | -0.833712801 | 0.775684388 | down |

| | | | |
|---|---|---|---|
| uc_338 | -0.833712801 | 0.775684388 | down |
| LINC00374 | -0.833712801 | 0.775684388 | down |
| RNY3P5 | -0.833712801 | 0.775684388 | down |
| RP11-432J3.4 | -0.833712801 | 0.775684388 | down |
| TRIM60P19 | -0.833712801 | 0.775684388 | down |
| LINC00446 | -0.833712801 | 0.775684388 | down |
| NIPA2P5 | -0.833712801 | 0.775684388 | down |
| RP11-464I4.1 | -0.833712801 | 0.775684388 | down |
| DDX6P2 | -0.833712801 | 0.775684388 | down |
| NA | -0.833712801 | 0.775684388 | down |
| MIR4306 | -0.833712801 | 0.775684388 | down |
| RNY1P2 | -0.833712801 | 0.775684388 | down |
| FAM155A-IT1 | -0.833712801 | 0.775684388 | down |
| OR4H12P | -0.833712801 | 0.775684388 | down |
| TRDJ2 | -0.833712801 | 0.775684388 | down |
| Y_RNA | -0.833712801 | 0.775684388 | down |
| NA | -0.833712801 | 0.775684388 | down |
| NA | -0.833712801 | 0.775684388 | down |
| AL356806.3 | -0.833712801 | 0.775684388 | down |
| RP11-506K19.2 | -0.833712801 | 0.775684388 | down |
| NA | -0.833712801 | 0.775684388 | down |
| RP11-1042B17.2 | -0.833712801 | 0.775684388 | down |
| Y_RNA | -0.833712801 | 0.775684388 | down |
| MIR5694 | -0.833712801 | 0.775684388 | down |
| RNU6-921P | -0.833712801 | 0.775684388 | down |
| RP11-688D15.2 | -0.833712801 | 0.775684388 | down |
| RP11-753D20.4 | -0.833712801 | 0.775684388 | down |
| MPPE1P1 | -0.833712801 | 0.775684388 | down |
| SNORD114-24 | -0.833712801 | 0.775684388 | down |
| MIR300 | -0.833712801 | 0.775684388 | down |
| IGHVII-40-1 | -0.833712801 | 0.775684388 | down |
| IGHVII-62-1 | -0.833712801 | 0.775684388 | down |
| SLC20A1P3 | -0.833712801 | 0.775684388 | down |
| IGHD5OR15-5B | -0.833712801 | 0.775684388 | down |
| RP11-294C11.7 | -0.833712801 | 0.775684388 | down |
| SNORD115-39 | -0.833712801 | 0.775684388 | down |
| SNORD115-41 | -0.833712801 | 0.775684388 | down |
| MIR211 | -0.833712801 | 0.775684388 | down |
| Metazoa_SRP | -0.833712801 | 0.775684388 | down |
| NA | -0.833712801 | 0.775684388 | down |
| RN7SKP101 | -0.833712801 | 0.775684388 | down |
| RP11-408J6.1 | -0.833712801 | 0.775684388 | down |
| RP11-138E16.1 | -0.833712801 | 0.775684388 | down |
| AHCYP7 | -0.833712801 | 0.775684388 | down |
| RNA5SP396 | -0.833712801 | 0.775684388 | down |
| NMNAT1P5 | -0.833712801 | 0.775684388 | down |
| RNA5SP397 | -0.833712801 | 0.775684388 | down |
| RP11-162I7.1 | -0.833712801 | 0.775684388 | down |
| RP11-138H8.3 | -0.833712801 | 0.775684388 | down |
| FKBP1AP2 | -0.833712801 | 0.775684388 | down |
| RPL18P11 | -0.833712801 | 0.775684388 | down |

| | | | |
|---|---|---|---|
| MIR1179 | -0.833712801 | 0.775684388 | down |
| DUXAP6 | -0.833712801 | 0.775684388 | down |
| NA | -0.833712801 | 0.775684388 | down |
| ENPP7P14 | -0.833712801 | 0.775684388 | down |
| AF001550.7 | -0.833712801 | 0.775684388 | down |
| RNU7-24P | -0.833712801 | 0.775684388 | down |
| RP11-170L3.6 | -0.833712801 | 0.775684388 | down |
| RP11-652G5.2 | -0.833712801 | 0.775684388 | down |
| RP11-812E19.7 | -0.833712801 | 0.775684388 | down |
| IGHV3OR16-11 | -0.833712801 | 0.775684388 | down |
| C2orf69P1 | -0.833712801 | 0.775684388 | down |
| HMGN2P41 | -0.833712801 | 0.775684388 | down |
| KLF8P1 | -0.833712801 | 0.775684388 | down |
| CTD-2034I21.1 | -0.833712801 | 0.775684388 | down |
| RP11-883G14.4 | -0.833712801 | 0.775684388 | down |
| RP11-467J12.3 | -0.833712801 | 0.775684388 | down |
| RPL31P56 | -0.833712801 | 0.775684388 | down |
| UBE2FP2 | -0.833712801 | 0.775684388 | down |
| CTD-2015G9.1 | -0.833712801 | 0.775684388 | down |
| OR1D5 | -0.833712801 | 0.775684388 | down |
| AC005324.7 | -0.833712801 | 0.775684388 | down |
| AC087499.8 | -0.833712801 | 0.775684388 | down |
| RP11-227G15.6 | -0.833712801 | 0.775684388 | down |
| RP11-1018N14.3 | -0.833712801 | 0.775684388 | down |
| CCDC182 | -0.833712801 | 0.775684388 | down |
| GS1-531I17.2 | -0.833712801 | 0.775684388 | down |
| RNU6-227P | -0.833712801 | 0.775684388 | down |
| RP11-78F17.1 | -0.833712801 | 0.775684388 | down |
| RN7SKP44 | -0.833712801 | 0.775684388 | down |
| RP11-789C7.1 | -0.833712801 | 0.775684388 | down |
| MLECP1 | -0.833712801 | 0.775684388 | down |
| RP11-638L3.2 | -0.833712801 | 0.775684388 | down |
| SNORA25 | -0.833712801 | 0.775684388 | down |
| NA | -0.833712801 | 0.775684388 | down |
| CTC-499B15.1 | -0.833712801 | 0.775684388 | down |
| BNIP3P31 | -0.833712801 | 0.775684388 | down |
| BNIP3P32 | -0.833712801 | 0.775684388 | down |
| CTC-457E21.5 | -0.833712801 | 0.775684388 | down |
| LLNLF-18A12.1 | -0.833712801 | 0.775684388 | down |
| RNA5SP472 | -0.833712801 | 0.775684388 | down |
| CTD-2329C7.2 | -0.833712801 | 0.775684388 | down |
| LGALS13 | -0.833712801 | 0.775684388 | down |
| PSG9 | -0.833712801 | 0.775684388 | down |
| L34079.4 | -0.833712801 | 0.775684388 | down |
| AC138472.6 | -0.833712801 | 0.775684388 | down |
| IGFL1 | -0.833712801 | 0.775684388 | down |
| MIR516B1 | -0.833712801 | 0.775684388 | down |
| AC010518.3 | -0.833712801 | 0.775684388 | down |
| NA | -0.833712801 | 0.775684388 | down |
| MACROD2-IT1 | -0.833712801 | 0.775684388 | down |
| RNU6-1159P | -0.833712801 | 0.775684388 | down |

| | | | |
|---|---|---|---|
| RN7SL237P | -0.833712801 | 0.775684388 | down |
| RP11-380D15.3 | -0.833712801 | 0.775684388 | down |
| C20orf181 | -0.833712801 | 0.775684388 | down |
| FGF7P2 | -0.833712801 | 0.775684388 | down |
| GTF2IP2 | -0.833712801 | 0.775684388 | down |
| AP000432.2 | -0.833712801 | 0.775684388 | down |
| AP000431.1 | -0.833712801 | 0.775684388 | down |
| AP000431.2 | -0.833712801 | 0.775684388 | down |
| AP000855.4 | -0.833712801 | 0.775684388 | down |
| KRT18P2 | -0.833712801 | 0.775684388 | down |
| ZNF299P | -0.833712801 | 0.775684388 | down |
| AP001601.2 | -0.833712801 | 0.775684388 | down |
| KRTAP20-4 | -0.833712801 | 0.775684388 | down |
| AC008079.9 | -0.833712801 | 0.775684388 | down |
| IGLL4P | -0.833712801 | 0.775684388 | down |
| IGLVIV-53 | -0.833712801 | 0.775684388 | down |
| NA | -0.833712801 | 0.775684388 | down |
| RPS27AP17 | -0.833712801 | 0.775684388 | down |
| RP11-126O22.10 | -0.833712801 | 0.775684388 | down |
| Y_RNA | -0.833712801 | 0.775684388 | down |
| RN7SKP290 | -0.833712801 | 0.775684388 | down |
| TMSB10P2 | -0.833712801 | 0.775684388 | down |
| GS1-46604.2 | -0.833712801 | 0.775684388 | down |
| PTP4A1P5 | -0.833712801 | 0.775684388 | down |
| RP11-12D5.2 | -0.833712801 | 0.775684388 | down |
| RP11-467P22.5 | -0.833712801 | 0.775684388 | down |
| RNU6-1087P | -0.833712801 | 0.775684388 | down |
| SSXP1 | -0.833712801 | 0.775684388 | down |
| RPSAP62 | -0.833712801 | 0.775684388 | down |
| RP6-29D12.2 | -0.833712801 | 0.775684388 | down |
| MTND1P30 | -0.833712801 | 0.775684388 | down |
| MIR1468 | -0.833712801 | 0.775684388 | down |
| RP11-151G12.2 | -0.833712801 | 0.775684388 | down |
| RNA5SP507 | -0.833712801 | 0.775684388 | down |
| RNA5SP509 | -0.833712801 | 0.775684388 | down |
| RNU6-332P | -0.833712801 | 0.775684388 | down |
| RP13-213K19.1 | -0.833712801 | 0.775684388 | down |
| GHc-602D8.2 | -0.833712801 | 0.775684388 | down |
| LL0XNC01-177E8.2 | -0.833712801 | 0.775684388 | down |
| RP6-191P20.3 | -0.833712801 | 0.775684388 | down |
| RPL36P18 | -0.833712801 | 0.775684388 | down |
| AKR1B1P8 | -0.833712801 | 0.775684388 | down |
| RP11-232D9.4 | -0.833712801 | 0.775684388 | down |
| RP3-428A13.2 | -0.833712801 | 0.775684388 | down |
| RP11-529O20.2 | -0.833712801 | 0.775684388 | down |
| RP11-364B14.1 | -0.833712801 | 0.775684388 | down |
| GS1-164F24.1 | -0.833712801 | 0.775684388 | down |
| GS1-54N10.1 | -0.833712801 | 0.775684388 | down |
| RP5-937E21.1 | -0.833712801 | 0.775684388 | down |
| RP5-1014O16.1 | -0.833712801 | 0.775684388 | down |
| RP11-122L9.1 | -0.833712801 | 0.775684388 | down |

| | | | | |
|---|---|---|---|---|
| RP11-644L4.1 | -0.833662961 | 0.325173118 | | down |
| RP11-212I21.2 | -0.833583836 | 0.101432799 | 0.204102004 | down |
| RGS11 | -0.833529741 | 0.000714908 | 0.004643041 | down |
| PGCP1 | -0.833342217 | 0.589650815 | | down |
| SNX32 | -0.83330944 | 0.002513664 | 0.012633072 | down |
| RP11-299H21.1 | -0.833043031 | 0.052291804 | 0.126218439 | down |
| POLR2I | -0.832966694 | 0.031216961 | 0.086344255 | down |
| RP11-403B2.7 | -0.832881243 | 0.223731858 | 0.355857903 | down |
| NA | -0.832721769 | 0.094150471 | 0.193703811 | down |
| TIGD4 | -0.832561925 | 0.153587268 | 0.273301619 | down |
| SRPK3 | -0.832519732 | 0.000491007 | 0.003427612 | down |
| TBC1D3K | -0.832493105 | 0.576257222 | | down |
| CTC-250P20.2 | -0.832348077 | 0.485193606 | | down |
| TSPAN15 | -0.832291735 | 0.000298971 | 0.002323844 | down |
| FGD6 | -0.832231946 | 0.009467793 | 0.035152048 | down |
| RP11-159F24.5 | -0.831957217 | 0.207300623 | 0.337422252 | down |
| ATP6V0E1P4 | -0.831893279 | 0.634792986 | 0.741974808 | down |
| NSMCE1 | -0.831721051 | 0.00084507 | 0.0053038 | down |
| MMP16 | -0.831713352 | 0.070541972 | 0.157276777 | down |
| PROZ | -0.831541362 | 0.113751249 | 0.221472525 | down |
| RP11-783K16.14 | -0.831466397 | 0.165788434 | 0.28866692 | down |
| RP11-343J3.2 | -0.831406596 | 0.193534783 | 0.321655089 | down |
| RP11-244N9.4 | -0.83130087 | 0.477938355 | | down |
| GABRG3 | -0.830904227 | 0.214113404 | 0.344997486 | down |
| RP4-669P10.19 | -0.830442558 | 0.091350008 | 0.189447066 | down |
| KIR2DS4 | -0.830311948 | 0.309828592 | 0.448385849 | down |
| RP11-112J3.16 | -0.829908281 | 0.027528337 | 0.078784228 | down |
| CLDN34 | -0.829906552 | 0.469509144 | | down |
| CISTR | -0.829807529 | 0.282707409 | 0.419191659 | down |
| DICER1-AS1 | -0.829775618 | 0.002082928 | 0.010834397 | down |
| RP11-715H19.2 | -0.829666844 | 0.536615965 | | down |
| SEZ6L2 | -0.829424195 | 0.00266234 | 0.013205769 | down |
| ZNF729 | -0.829065344 | 0.561184744 | 0.680439112 | down |
| CELF2-AS2 | -0.828909371 | 0.542054565 | | down |
| RAP1GAP | -0.828638102 | 0.004787651 | 0.020998566 | down |
| RP3-413H6.2 | -0.828463296 | 0.345035779 | | down |
| RP5-1185H19.2 | -0.828386131 | 0.398531134 | | down |
| ADAMTSL4-AS1 | -0.828260243 | 0.045085523 | 0.113389258 | down |
| NEFLP1 | -0.828157066 | 0.70495151 | | down |
| GHET1 | -0.828099373 | 0.136533641 | 0.251769401 | down |
| RP13-20L14.4 | -0.828078725 | 0.087481668 | 0.183745752 | down |
| PAICSP1 | -0.827982962 | 0.090053386 | 0.187573762 | down |
| LINC00654 | -0.827963638 | 0.002509757 | 0.012615549 | down |
| PRSS27 | -0.827851144 | 0.007458919 | 0.029370349 | down |
| LINC01522 | -0.827800329 | 0.405414156 | 0.541947182 | down |
| PCBD2 | -0.82778101 | 2.96E-10 | 1.76E-08 | down |
| NA | -0.827775944 | 0.550729872 | | down |
| DEFB122 | -0.827727796 | 0.589331668 | 0.704589993 | down |
| CTD-2537I9.13 | -0.827725716 | 0.155348655 | 0.275437886 | down |
| NBPF1 | -0.8276213 | 0.006057358 | 0.025122484 | down |
| AC007246.3 | -0.827420273 | 6.74E-06 | 0.000101896 | down |

| | | | | |
|---|---|---|---|---|
| AC002075.4 | -0.827408888 | 0.140661216 | 0.257182667 | down |
| WTH3DI | -0.827320989 | 0.060301162 | 0.140256106 | down |
| CCDC34P1 | -0.827310145 | 0.758138568 | | down |
| LRRC34 | -0.827257308 | 0.034309986 | 0.092732029 | down |
| NA | -0.827250749 | 0.37460498 | | down |
| CD300LB | -0.827206282 | 0.031919825 | 0.08778025 | down |
| RP11-685B14.1 | -0.827188269 | 0.328281449 | | down |
| MFAP3L | -0.827149811 | 0.011348366 | 0.04044008 | down |
| RP1-149A16.3 | -0.82708622 | 0.372751591 | 0.511087478 | down |
| CNTNAP3B | -0.827068973 | 0.034208638 | 0.092491388 | down |
| RP11-3B7.1 | -0.82695258 | 0.074447858 | 0.16344256 | down |
| MRPL35P2 | -0.826908144 | 0.36178683 | | down |
| NELL1 | -0.82688756 | 0.115915782 | 0.224464953 | down |
| UTS2B | -0.826863366 | 0.058513561 | 0.137235085 | down |
| RPL29P14 | -0.826836552 | 0.088462315 | 0.185248609 | down |
| MYLK2 | -0.826804403 | 0.059207245 | 0.138408222 | down |
| TMEM74B | -0.826495799 | 0.024496041 | 0.072218167 | down |
| HCG15 | -0.826495216 | 0.34555961 | | down |
| SETP2 | -0.826422236 | 0.592078173 | | down |
| RP11-2K6.1 | -0.826293842 | 0.055385279 | 0.131707719 | down |
| ANKDD1B | -0.826271547 | 0.010701398 | 0.038634078 | down |
| DPY19L2P3 | -0.826250009 | 0.06334891 | 0.1454754 | down |
| RP11-215A21.2 | -0.826227133 | 0.028633737 | 0.081157735 | down |
| BCAS1 | -0.826225016 | 0.002835114 | 0.013870406 | down |
| C1GALT1C1L | -0.826136845 | 0.1243814 | 0.235856777 | down |
| PPARG | -0.825942301 | 0.0002458 | 0.001983099 | down |
| LMNTD2 | -0.825917971 | 0.002128601 | 0.011035667 | down |
| AC020743.3 | -0.825835908 | 0.731444048 | | down |
| RP11-51G5.1 | -0.825689096 | 0.472872519 | 0.603243257 | down |
| CTD-2619J13.27 | -0.825631045 | 0.382528027 | | down |
| LRRC37A5P | -0.825514289 | 0.082258662 | 0.175880921 | down |
| RPS6P25 | -0.825402453 | 0.025665167 | 0.074760934 | down |
| AC110781.3 | -0.825351465 | 0.351880593 | | down |
| NA | -0.825269613 | 0.01110285 | 0.039725382 | down |
| INHBC | -0.825210436 | 0.226707263 | 0.358842686 | down |
| RP11-135J2.3 | -0.824991342 | 0.593745561 | | down |
| RPL13AP6 | -0.824983014 | 0.015629026 | 0.051531877 | down |
| ARFRP1 | -0.824523595 | 0.000589805 | 0.003979385 | down |
| RP11-490O24.2 | -0.824444005 | 0.761435435 | | down |
| RP11-138B9.1 | -0.824304011 | 0.714126472 | | down |
| RN7SL121P | -0.82430392 | 0.742257489 | | down |
| RP11-134D3.2 | -0.824303916 | 0.743322252 | | down |
| RNU6-633P | -0.824303783 | 0.772421875 | | down |
| C3orf22 | -0.824303778 | 0.773310562 | | down |
| RP11-149I2.4 | -0.824303776 | 0.773827723 | | down |
| IGLJ4 | -0.824303755 | 0.777469 | | down |
| RP3-437I16.1 | -0.824303751 | 0.778149299 | | down |
| RP11-376N17.4 | -0.824303751 | 0.778149299 | | down |
| NA | -0.824303751 | 0.778149299 | | down |
| RN7SL440P | -0.824303751 | 0.778149299 | | down |
| MIR548AC | -0.824303751 | 0.778149299 | | down |

| | | | |
|---|---|---|---|
| RP11-211A18.1 | -0.824303751 | 0.778149299 | down |
| RPL12P18 | -0.824303751 | 0.778149299 | down |
| IGKV3-31 | -0.824303751 | 0.778149299 | down |
| AC068491.3 | -0.824303751 | 0.778149299 | down |
| MIR4437 | -0.824303751 | 0.778149299 | down |
| RP11-764E7.1 | -0.824303751 | 0.778149299 | down |
| RP11-536I6.2 | -0.824303751 | 0.778149299 | down |
| RP11-588D3.1 | -0.824303751 | 0.778149299 | down |
| RP11-689P11.3 | -0.824303751 | 0.778149299 | down |
| RNU6-197P | -0.824303751 | 0.778149299 | down |
| RP11-466G12.3 | -0.824303751 | 0.778149299 | down |
| RP11-1E22.1 | -0.824303751 | 0.778149299 | down |
| CTC-507E12.1 | -0.824303751 | 0.778149299 | down |
| SNORA74A | -0.824303751 | 0.778149299 | down |
| MIR218-2 | -0.824303751 | 0.778149299 | down |
| RP11-446F17.3 | -0.824303751 | 0.778149299 | down |
| RP11-124I4.2 | -0.824303751 | 0.778149299 | down |
| MRPL30P1 | -0.824303751 | 0.778149299 | down |
| KNOP1P4 | -0.824303751 | 0.778149299 | down |
| AC005550.5 | -0.824303751 | 0.778149299 | down |
| Y_RNA | -0.824303751 | 0.778149299 | down |
| RP11-380I10.3 | -0.824303751 | 0.778149299 | down |
| RP11-25D10.2 | -0.824303751 | 0.778149299 | down |
| RP11-520B13.8 | -0.824303751 | 0.778149299 | down |
| RP11-18B3.2 | -0.824303751 | 0.778149299 | down |
| RNU6-996P | -0.824303751 | 0.778149299 | down |
| RP11-477J21.2 | -0.824303751 | 0.778149299 | down |
| PGGT1BP1 | -0.824303751 | 0.778149299 | down |
| EIF4A1P8 | -0.824303751 | 0.778149299 | down |
| NA | -0.824303751 | 0.778149299 | down |
| OR10V2P | -0.824303751 | 0.778149299 | down |
| MIR4488 | -0.824303751 | 0.778149299 | down |
| SNORA75 | -0.824303751 | 0.778149299 | down |
| PCNPP3 | -0.824303751 | 0.778149299 | down |
| RP11-453D16.1 | -0.824303751 | 0.778149299 | down |
| IMMP1LP2 | -0.824303751 | 0.778149299 | down |
| NDE1P2 | -0.824303751 | 0.778149299 | down |
| CFL1P8 | -0.824303751 | 0.778149299 | down |
| RP11-964E11.3 | -0.824303751 | 0.778149299 | down |
| IGHV3-36 | -0.824303751 | 0.778149299 | down |
| NA | -0.824303751 | 0.778149299 | down |
| NA | -0.824303751 | 0.778149299 | down |
| NA | -0.824303751 | 0.778149299 | down |
| RN7SL743P | -0.824303751 | 0.778149299 | down |
| MTND4LP25 | -0.824303751 | 0.778149299 | down |
| KRTAP17-1 | -0.824303751 | 0.778149299 | down |
| RN7SL125P | -0.824303751 | 0.778149299 | down |
| NA | -0.824303751 | 0.778149299 | down |
| RN7SL615P | -0.824303751 | 0.778149299 | down |
| RP5-1030M6.3 | -0.824303751 | 0.778149299 | down |
| MTND1P9 | -0.824303751 | 0.778149299 | down |

| | | | | |
|---|---|---|---|---|
| MIR3196 | -0.824303751 | 0.778149299 | | down |
| RNU6-331P | -0.824303751 | 0.778149299 | | down |
| PTCHD1-AS | -0.824303751 | 0.778149299 | | down |
| RP11-234P3.4 | -0.824303751 | 0.778149299 | | down |
| RPS28 | -0.824232199 | 0.001068305 | 0.006391111 | down |
| SPINK4 | -0.824178938 | 0.048845952 | 0.12035822 | down |
| NA | -0.824151213 | 0.216602574 | | down |
| RP11-656G20.1 | -0.824082461 | 0.169893001 | 0.293584799 | down |
| C12orf54 | -0.823723872 | 0.152588507 | 0.272053238 | down |
| NA | -0.823702744 | 0.174640361 | 0.299036533 | down |
| RP11-389O22.5 | -0.823635053 | 0.086719632 | 0.182655961 | down |
| RPSAP42 | -0.823607724 | 0.778189428 | | down |
| AC053503.12 | -0.823503154 | 0.108075322 | 0.213774112 | down |
| NA | -0.823418685 | 0.03845163 | 0.100903202 | down |
| RP11-739N20.2 | -0.823118138 | 0.1211901 | 0.231671822 | down |
| OCEL1 | -0.823112388 | 6.53E-05 | 0.000663904 | down |
| THNSL2 | -0.823089596 | 0.000497162 | 0.003458379 | down |
| RP11-90B9.2 | -0.823009527 | 0.316348153 | 0.454928389 | down |
| CTC-360G5.9 | -0.822989002 | 0.152483693 | 0.271953004 | down |
| CDX1 | -0.822918291 | 0.004130397 | 0.018714167 | down |
| CEP126 | -0.822830468 | 0.004637414 | 0.020468118 | down |
| TPI1P2 | -0.822650593 | 0.073492389 | 0.162007875 | down |
| TMEM14EP | -0.822623002 | 0.345990503 | | down |
| ZBTB20-AS4 | -0.82242168 | 0.426201512 | | down |
| RP11-678K21.2 | -0.822386288 | 0.676442867 | | down |
| SMG7-AS1 | -0.822381892 | 0.0476579 | 0.118094418 | down |
| NA | -0.822300233 | 0.733957833 | | down |
| FBXW12 | -0.822153868 | 0.33531435 | 0.473601279 | down |
| NA | -0.82205208 | 0.623548886 | | down |
| RP11-234K24.6 | -0.821917978 | 0.127157471 | 0.239458384 | down |
| RP11-789C17.1 | -0.821883625 | 0.256006426 | 0.391052 | down |
| CTD-2256P15.1 | -0.821814911 | 0.368519563 | 0.507020275 | down |
| RP11-1212A22.4 | -0.821814381 | 0.70653529 | | down |
| ZBTB12BP | -0.821420703 | 0.303756457 | 0.441747374 | down |
| CTB-40H15.4 | -0.82136278 | 0.115215384 | 0.223526475 | down |
| NDRG1 | -0.821258927 | 2.20E-06 | 4.03E-05 | down |
| LINC00441 | -0.821177766 | 0.106440272 | 0.211333037 | down |
| MSRA | -0.821109376 | 2.60E-08 | 8.83E-07 | down |
| RPL17P50 | -0.820638445 | 0.033013122 | 0.08999603 | down |
| CD24 | -0.82057558 | 0.011964651 | 0.042116694 | down |
| RP11-356M20.2 | -0.820535363 | 0.322307378 | 0.461048558 | down |
| RP1-278D1.2 | -0.820386792 | 0.377890869 | | down |
| AF127936.7 | -0.820342902 | 0.228999321 | 0.361404401 | down |
| HOXA2 | -0.820163598 | 0.005305029 | 0.022710783 | down |
| MTHFR | -0.820031171 | 7.95E-06 | 0.00011653 | down |
| ANOS2P | -0.819860112 | 0.229810255 | 0.362151549 | down |
| DNASE1L2 | -0.81943257 | 0.038482579 | 0.100935322 | down |
| KDF1 | -0.819368004 | 0.001471711 | 0.008212495 | down |
| AE000661.37 | -0.819342131 | 0.075215735 | 0.164659103 | down |
| NA | -0.81925722 | 0.218680507 | 0.350133937 | down |
| LINC01535 | -0.819101537 | 0.073133112 | 0.161440941 | down |

| | | | | |
|---|---|---|---|---|
| PRKAR1B | -0.818925745 | 1.13E-05 | 0.000156436 | down |
| TUNAR | -0.818912614 | 0.240895332 | 0.374718949 | down |
| AURKAIP1 | -0.818716375 | 0.000953616 | 0.005832742 | down |
| NCMAP | -0.818655612 | 0.017356561 | 0.055735411 | down |
| CTB-12904.1 | -0.818489301 | 0.040718131 | 0.105287505 | down |
| RP11-762H8.1 | -0.81806673 | 0.478627735 | | down |
| RNA5SP502 | -0.817924832 | 0.600702443 | 0.713819729 | down |
| RTL1 | -0.817903258 | 0.213601195 | 0.344467756 | down |
| RP11-30506.3 | -0.81774497 | 0.340118855 | 0.478418038 | down |
| RP11-383C5.3 | -0.817377559 | 0.132512505 | 0.24652955 | down |
| RN7SL835P | -0.817271366 | 0.691781336 | 0.787297945 | down |
| FKBP1B | -0.817259926 | 0.004022079 | 0.018318904 | down |
| RP11-33301.1 | -0.817207653 | 0.552877295 | | down |
| TM7SF2 | -0.817196815 | 0.000833903 | 0.005247966 | down |
| PLGLB2 | -0.817132891 | 0.174349001 | 0.298823934 | down |
| Y_RNA | -0.816823568 | 0.76383343 | | down |
| CNDP2 | -0.816225898 | 8.80E-06 | 0.000127124 | down |
| UPP1 | -0.815675443 | 0.00522661 | 0.02244223 | down |
| NA | -0.815612108 | 0.133774034 | 0.248001177 | down |
| CARD10 | -0.815563114 | 0.000140258 | 0.001251907 | down |
| MISP3 | -0.815526356 | 0.005965765 | 0.024845502 | down |
| SLC22A20 | -0.815362735 | 0.074192919 | 0.163049672 | down |
| ALDH1A1 | -0.815332058 | 0.006696576 | 0.027103857 | down |
| RP11-299P2.2 | -0.815309022 | 0.32225134 | 0.461034251 | down |
| PRR11 | -0.815298027 | 0.003121835 | 0.014946998 | down |
| CD101 | -0.815274641 | 0.002366196 | 0.012026731 | down |
| RP11-31F15.2 | -0.81526429 | 0.101629468 | 0.204388227 | down |
| SNCB | -0.815216409 | 0.068677983 | 0.15415475 | down |
| PHYH | -0.815159719 | 8.39E-06 | 0.000122107 | down |
| MYOM1 | -0.815011096 | 0.001675726 | 0.009113961 | down |
| RP11-627K11.1 | -0.814952044 | 0.019322079 | 0.06051196 | down |
| RP11-355F22.1 | -0.814895151 | 0.675431273 | | down |
| RP1-228P16.3 | -0.814894928 | 0.742405237 | | down |
| RP11-131K5.1 | -0.814894921 | 0.743870725 | | down |
| IGLV3-7 | -0.814894921 | 0.744031739 | | down |
| RP11-395N21.2 | -0.81489491 | 0.746192723 | | down |
| TRBV12-2 | -0.814894908 | 0.746589197 | | down |
| CTD-2326C4.1 | -0.814894785 | 0.768532873 | | down |
| NA | -0.814894753 | 0.773298807 | | down |
| RP11-1058N17.1 | -0.814894751 | 0.773643228 | | down |
| RP11-142G1.1 | -0.814894737 | 0.775686541 | | down |
| RNU6-1310P | -0.814894736 | 0.775840234 | | down |
| Y_RNA | -0.814894731 | 0.776463768 | | down |
| CTC-524C5.5 | -0.814894727 | 0.7770933 | | down |
| LINC01541 | -0.814894725 | 0.777330439 | | down |
| AC006445.7 | -0.814894715 | 0.77867239 | | down |
| AC007349.7 | -0.814894715 | 0.778695996 | | down |
| RP11-266I3.7 | -0.814894709 | 0.779501485 | | down |
| NA | -0.814894708 | 0.779719232 | | down |
| RP1-137K2.2 | -0.814894706 | 0.779944596 | | down |
| TMEM185AP1 | -0.814894705 | 0.780101234 | | down |

| | | | | |
|---|---|---|---|---|
| RP11-117D22.1 | -0.814894701 | 0.780616443 | | down |
| HMGB3P9 | -0.814894701 | 0.780616443 | | down |
| RP4-590F24.2 | -0.814894701 | 0.780616443 | | down |
| SNORD70 | -0.814894701 | 0.780616443 | | down |
| RP11-501M7.1 | -0.814894701 | 0.780616443 | | down |
| RP11-758I14.3 | -0.814894701 | 0.780616443 | | down |
| RBBP4P4 | -0.814894701 | 0.780616443 | | down |
| RP11-474L11.5 | -0.814894701 | 0.780616443 | | down |
| POU6F2-AS1 | -0.814894701 | 0.780616443 | | down |
| HSPE1P14 | -0.814894701 | 0.780616443 | | down |
| RP11-15K3.1 | -0.814894701 | 0.780616443 | | down |
| RNU6-1123P | -0.814894701 | 0.780616443 | | down |
| RP11-457D13.4 | -0.814894701 | 0.780616443 | | down |
| RP11-473M10.3 | -0.814894701 | 0.780616443 | | down |
| RP11-23A22.1 | -0.814894701 | 0.780616443 | | down |
| CTC-539A10.7 | -0.814894701 | 0.780616443 | | down |
| LLNLF-158E9.1 | -0.814894701 | 0.780616443 | | down |
| CTC-490G23.6 | -0.814894701 | 0.780616443 | | down |
| RP11-157D23.2 | -0.814894701 | 0.780616443 | | down |
| ASB17 | -0.81484694 | 0.621510489 | 0.731486705 | down |
| CPHL1P | -0.814828419 | 0.264314206 | 0.399751121 | down |
| RP11-197N18.7 | -0.814675614 | 0.252994557 | 0.388005793 | down |
| DHX57 | -0.814630955 | 9.70E-06 | 0.000137728 | down |
| GAS2 | -0.814606953 | 0.020906487 | 0.06417845 | down |
| TRAPPC12-AS1 | -0.814595706 | 0.119504576 | 0.229520902 | down |
| RP11-342A23.2 | -0.814176085 | 0.385426472 | | down |
| NPM2 | -0.813970408 | 0.004765869 | 0.020918302 | down |
| MAB21L1 | -0.81390001 | 0.109657075 | 0.216006895 | down |
| RNU6-287P | -0.813763423 | 0.526498278 | 0.649961551 | down |
| TMEM205 | -0.813706733 | 0.01995503 | 0.061992445 | down |
| RP11-62J1.4 | -0.813690588 | 0.436400503 | | down |
| RAB41 | -0.813652192 | 0.012387506 | 0.043183256 | down |
| FBLIM1 | -0.813611731 | 3.21E-05 | 0.000369855 | down |
| AKAP1 | -0.813541823 | 3.91E-05 | 0.000436473 | down |
| HMGB1P1 | -0.813532623 | 0.030281417 | 0.084373061 | down |
| RP5-1098D14.1 | -0.813464353 | 0.132373147 | 0.246407611 | down |
| RP11-609N14.4 | -0.813395898 | 0.140160878 | 0.256517631 | down |
| EYA1 | -0.813246707 | 0.127555572 | 0.240027534 | down |
| EXOC3L1 | -0.813064068 | 0.008054379 | 0.031103568 | down |
| SOX30 | -0.813028509 | 0.030574951 | 0.084968441 | down |
| RP5-940J5.3 | -0.812991215 | 0.376330928 | | down |
| AGAP14 | -0.812987001 | 0.20464551 | 0.334277698 | down |
| FO538757.1 | -0.812961185 | 0.000200021 | 0.001669752 | down |
| TUBB4A | -0.812865287 | 0.073724379 | 0.162364406 | down |
| RP11-800A18.4 | -0.812827655 | 0.17247924 | 0.29677374 | down |
| RP11-404P21.5 | -0.812764608 | 0.650343722 | | down |
| ANGPTL8 | -0.812641524 | 0.123767737 | 0.235049172 | down |
| DPEP3 | -0.812603791 | 0.01622123 | 0.052960241 | down |
| RP4-621B10.8 | -0.812603641 | 0.117200112 | 0.226295641 | down |
| ATRNL1 | -0.812597689 | 0.069033949 | 0.154728795 | down |
| RP11-366M4.17 | -0.812516458 | 0.130037538 | 0.243265741 | down |

| | | | | |
|---|---|---|---|---|
| CTD-2081C10.1 | -0.812427659 | 0.563167548 | 0.682030224 | down |
| RP11-96B5.3 | -0.812374946 | 0.328454761 | 0.466929821 | down |
| HSPB1P1 | -0.812359402 | 0.043310168 | 0.110269037 | down |
| NA | -0.812192145 | 0.262485906 | 0.3977669 | down |
| RCC2P6 | -0.812146771 | 0.238438864 | 0.371887586 | down |
| RP11-502I4.3 | -0.812067974 | 0.007191082 | 0.028551455 | down |
| CTC-429P9.1 | -0.811955377 | 0.017931105 | 0.057231714 | down |
| AGBL5-AS1 | -0.811489636 | 0.141760988 | 0.258460262 | down |
| NDUFA3 | -0.811476331 | 0.002051521 | 0.010715558 | down |
| NECAB3 | -0.811402516 | 1.73E-09 | 8.29E-08 | down |
| WDR18 | -0.811392008 | 0.000863537 | 0.005387068 | down |
| CBWD7 | -0.811377569 | 0.000104321 | 0.000980697 | down |
| RP11-725P16.2 | -0.811230934 | 0.529484772 | | down |
| MAGI3 | -0.811174351 | 0.000785162 | 0.004999888 | down |
| CACHD1 | -0.81111102 | 0.03121881 | 0.086344255 | down |
| RP11-313I2.11 | -0.81103986 | 0.6233518 | 0.732964716 | down |
| RP4-740C4.7 | -0.811029108 | 0.031259459 | 0.086423042 | down |
| FOXP4-AS1 | -0.810992247 | 0.018274216 | 0.058055465 | down |
| CNOT4P1 | -0.810864598 | 0.781423875 | | down |
| UBE3D | -0.810726087 | 0.001241162 | 0.007200128 | down |
| AC096558.1 | -0.810707031 | 0.060598714 | 0.14082009 | down |
| DNM1P17 | -0.810693496 | 0.600582495 | | down |
| AC004985.12 | -0.810606996 | 0.131186152 | 0.244853003 | down |
| RP11-1293J14.1 | -0.81059363 | 0.108649938 | 0.214656326 | down |
| SSPO | -0.81047537 | 0.004128737 | 0.01870947 | down |
| PTGDR2 | -0.810415049 | 0.049753429 | 0.121840109 | down |
| RP11-454H19.2 | -0.81040987 | 0.274959407 | 0.41123459 | down |
| RP11-93B14.9 | -0.81017531 | 0.000298968 | 0.002323844 | down |
| RNF5P1 | -0.810121851 | 0.082398711 | 0.176073034 | down |
| LINC01387 | -0.809930218 | 0.765086423 | | down |
| AC009948.7 | -0.809746528 | 0.13591373 | 0.250934399 | down |
| QRICH2 | -0.809565792 | 0.001730322 | 0.009360063 | down |
| AC005523.3 | -0.809477621 | 0.114248647 | 0.222167402 | down |
| CLIP1-AS1 | -0.809317628 | 0.368316674 | 0.506950071 | down |
| HIST1H2BM | -0.809304988 | 0.274855923 | 0.411120748 | down |
| GPR179 | -0.809304072 | 0.185055543 | 0.311160105 | down |
| RP4-569M23.5 | -0.809065392 | 0.144911331 | 0.262477605 | down |
| SUMO2P3 | -0.808785595 | 0.203030006 | 0.33247035 | down |
| RAB44 | -0.808539063 | 0.044991232 | 0.113190134 | down |
| RP11-325I22.3 | -0.808451515 | 0.071993681 | 0.159570503 | down |
| EMC3-AS1 | -0.80842025 | 0.048862095 | 0.120378251 | down |
| LINC01314 | -0.808344007 | 0.01703252 | 0.054917904 | down |
| DMAP1 | -0.808322138 | 0.000134484 | 0.001208622 | down |
| RP1-283E3.8 | -0.808221485 | 0.040781244 | 0.105369083 | down |
| FA2H | -0.80787109 | 0.007108409 | 0.028314269 | down |
| NPHP3 | -0.80778127 | 0.000287943 | 0.00225638 | down |
| RNU5B-1 | -0.807627847 | 0.379483136 | 0.517530247 | down |
| INSR | -0.807611164 | 0.000505107 | 0.003503911 | down |
| NARFL | -0.807548169 | 0.000818528 | 0.005172874 | down |
| RSG1 | -0.807297727 | 0.0001341 | 0.001205898 | down |
| OSR1 | -0.807274194 | 0.0219652 | 0.066517255 | down |

| | | | | |
|---|---|---|---|---|
| MVP | -0.807265726 | 4.37E-06 | 7.10E-05 | down |
| TUBB7P | -0.807257152 | 0.446380016 | | down |
| SLC26A6 | -0.806843631 | 0.00016762 | 0.001445458 | down |
| SAPCD1-AS1 | -0.806673757 | 0.289818482 | 0.427039914 | down |
| RP13-497K6.1 | -0.806653419 | 0.402951669 | 0.539615776 | down |
| FAM133A | -0.806564353 | 0.229662098 | 0.361956044 | down |
| MTCO3P29 | -0.806542437 | 0.317921815 | | down |
| USP32P1 | -0.80639024 | 0.367193746 | 0.50572884 | down |
| WDR78 | -0.806299073 | 0.004563622 | 0.020219573 | down |
| RP11-25L3.3 | -0.806087921 | 0.514938796 | | down |
| AC013264.2 | -0.80607563 | 0.119879059 | 0.230019572 | down |
| ZNF92P3 | -0.805812789 | 0.5878603 | | down |
| PHF7 | -0.80566994 | 2.04E-13 | 2.71E-11 | down |
| FAM173A | -0.80557047 | 0.001757738 | 0.009471393 | down |
| MAP2K4P1 | -0.80556887 | 0.15662775 | 0.277135088 | down |
| RP3-439F8.1 | -0.805542146 | 0.049716413 | 0.121793862 | down |
| AC010095.6 | -0.805486388 | 0.577455804 | | down |
| CTC-529G1.1 | -0.805486192 | 0.680156823 | | down |
| RBMY2TP | -0.805486186 | 0.682188114 | | down |
| Xbac-BPGBPG34I8. | -0.805486084 | 0.711918102 | | down |
| CD200R1L | -0.805486081 | 0.712814362 | | down |
| NA | -0.805485939 | 0.742892755 | | down |
| RP11-10L12.2 | -0.805485933 | 0.743975432 | | down |
| Y_RNA | -0.805485926 | 0.745282809 | | down |
| RP11-132E11.3 | -0.805485923 | 0.745744659 | | down |
| RP11-388N2.1 | -0.805485923 | 0.745821305 | | down |
| IGHV3OR16-9 | -0.805485919 | 0.746418001 | | down |
| CICP9 | -0.805485739 | 0.772899542 | | down |
| MYCBP2-AS2 | -0.805485723 | 0.774817744 | | down |
| RNA5SP302 | -0.805485719 | 0.775331099 | | down |
| RN7SKP211 | -0.805485718 | 0.775434766 | | down |
| ITM2BP1 | -0.805485705 | 0.776965126 | | down |
| SFR1P1 | -0.805485689 | 0.778834511 | | down |
| RP11-616M17.1 | -0.80548568 | 0.779853443 | | down |
| GAST | -0.805485679 | 0.779980068 | | down |
| RP11-403A3.1 | -0.805485663 | 0.781809753 | | down |
| RP11-444C12.1 | -0.805485651 | 0.783085799 | | down |
| RNU1-141P | -0.805485651 | 0.783085799 | | down |
| Y_RNA | -0.805485651 | 0.783085799 | | down |
| Y_RNA | -0.805485651 | 0.783085799 | | down |
| MAGI1-AS1 | -0.805485651 | 0.783085799 | | down |
| RP11-35J1.1 | -0.805485651 | 0.783085799 | | down |
| RP11-643A5.1 | -0.805485651 | 0.783085799 | | down |
| NA | -0.805485651 | 0.783085799 | | down |
| IGLV3-32 | -0.805485651 | 0.783085799 | | down |
| RN7SL687P | -0.805485651 | 0.783085799 | | down |
| HMX2 | -0.805313503 | 0.167162189 | 0.290346211 | down |
| RP11-567P19.1 | -0.805190044 | 0.16438894 | 0.286857227 | down |
| RPL37P6 | -0.8050339 | 0.054591423 | 0.130418631 | down |
| RP11-89N17.4 | -0.804910587 | 0.382482908 | 0.520204459 | down |
| RP11-93209.8 | -0.804860714 | 0.116096099 | 0.224735148 | down |

| | | | | |
|---|---|---|---|---|
| RP11-981G7.6 | -0.804821632 | 0.0327877 | 0.08954401 | down |
| RP11-646J21.2 | -0.804788679 | 0.571419759 | | down |
| NA | -0.804761866 | 0.76735835 | | down |
| RP11-26H16.1 | -0.804725527 | 0.509516661 | 0.635631714 | down |
| TMEM35A | -0.804612081 | 0.103478927 | 0.206888993 | down |
| SMG1P3 | -0.804558714 | 0.000359951 | 0.002692818 | down |
| DHRS3 | -0.804494785 | 2.91E-06 | 5.08E-05 | down |
| CLEC4F | -0.804418585 | 0.04423122 | 0.111815341 | down |
| MESTP4 | -0.804383749 | 0.78333277 | | down |
| LIN7B | -0.804343588 | 1.77E-05 | 0.000226698 | down |
| NA | -0.804215235 | 0.501369604 | 0.628390488 | down |
| AC092573.2 | -0.804160528 | 0.290936036 | 0.428054959 | down |
| CEP152 | -0.804139657 | 0.000886396 | 0.005496571 | down |
| LINC00310 | -0.804107247 | 0.081903771 | 0.175289705 | down |
| LINC01297 | -0.80385737 | 0.503105718 | 0.629856997 | down |
| OR7E7P | -0.803815978 | 0.07079192 | 0.1575928 | down |
| ZPBP | -0.803693026 | 0.651139567 | | down |
| TMEM74 | -0.803668982 | 0.109888193 | 0.216300036 | down |
| LHB | -0.803576083 | 0.062222789 | 0.143602818 | down |
| RP11-104L21.3 | -0.803566494 | 0.143477848 | 0.260745734 | down |
| MVB12A | -0.803510279 | 0.000478501 | 0.003355769 | down |
| RP4-801G22.2 | -0.803350594 | 0.597825699 | | down |
| RP5-965G21.3 | -0.803338572 | 0.02832352 | 0.080513941 | down |
| NAAA | -0.803241907 | 2.35E-05 | 0.000286055 | down |
| RP11-304L19.11 | -0.803055947 | 0.017762378 | 0.056838095 | down |
| RP11-180I4.4 | -0.802869805 | 0.050320741 | 0.122863579 | down |
| CTC-756D1.1 | -0.802664262 | 0.324613335 | | down |
| RP11-95H3.1 | -0.802543015 | 0.253394196 | 0.388318277 | down |
| NA | -0.802507982 | 0.069797597 | 0.155968687 | down |
| RP11-320H14.1 | -0.802472772 | 0.133690717 | 0.247892606 | down |
| RPL32P34 | -0.802458338 | 0.711538086 | | down |
| CYBA | -0.802407578 | 0.021093891 | 0.064601924 | down |
| RP11-862L9.2 | -0.802351782 | 0.552830156 | | down |
| RP6-65G23.5 | -0.802346427 | 0.061050839 | 0.141597477 | down |
| NA | -0.80234596 | 0.573090824 | | down |
| EEF1GP5 | -0.802293762 | 0.030499133 | 0.08483623 | down |
| DNAAF3 | -0.802292675 | 0.058441751 | 0.137141604 | down |
| RP11-696F12.1 | -0.802194991 | 0.246944893 | 0.381233622 | down |
| RP11-108L7.15 | -0.80212777 | 0.009010346 | 0.033872225 | down |
| ARPC4 | -0.802003759 | 0.006292148 | 0.025850034 | down |
| C2CD4D | -0.801855895 | 0.010510425 | 0.038104888 | down |
| RPS23P8 | -0.801747819 | 0.00040346 | 0.002931451 | down |
| CH17-296N19.1 | -0.801567405 | 0.486724635 | | down |
| NA | -0.801342747 | 0.179372229 | 0.304518237 | down |
| LINC00671 | -0.801250011 | 0.09131529 | 0.189401214 | down |
| LY6H | -0.801231501 | 0.051201597 | 0.124468063 | down |
| RP11-330M2.4 | -0.801060732 | 0.341761345 | | down |
| SCN4A | -0.801033593 | 0.034557554 | 0.093241775 | down |
| SGCG | -0.800983309 | 0.126803856 | 0.238982317 | down |
| RP11-355O1.7 | -0.800903433 | 0.270233137 | | down |
| ULBP3 | -0.800843468 | 0.014018797 | 0.047446292 | down |

| | | | | |
|---|---|---|---|---|
| RP5-1182A14.5 | -0.800650761 | 0.18749828 | 0.314072164 | down |
| SUZ12P1 | -0.800643947 | 0.000351819 | 0.002650465 | down |
| RP11-456N14.4 | -0.800625422 | 0.340912871 | 0.479265654 | down |
| FAM86JP | -0.800484666 | 0.008723317 | 0.033020455 | down |
| NA | -0.800339418 | 0.109605137 | 0.215951824 | down |
| WWTR1-IT1 | -0.799786568 | 0.209751138 | 0.340066008 | down |
| ABCD3 | -0.799749607 | 0.000781347 | 0.004979804 | down |
| MT1E | -0.799515983 | 0.004335054 | 0.019395734 | down |
| ODF3L1 | -0.799492332 | 0.017097711 | 0.055063097 | down |
| RP5-1153D9.5 | -0.799297681 | 0.298933519 | 0.43646772 | down |
| RP11-168P8.5 | -0.799275906 | 0.598035435 | | down |
| NMUR2 | -0.799118736 | 0.320863659 | 0.45959608 | down |
| UG0898H09 | -0.799069127 | 0.179331568 | 0.30448642 | down |
| RP11-582J16.5 | -0.798987562 | 0.03807648 | 0.100163714 | down |
| EPHB2 | -0.79866074 | 0.012727709 | 0.044094936 | down |
| C5orf66-AS1 | -0.798486079 | 0.033710763 | 0.091482807 | down |
| TNFSF15 | -0.798080303 | 0.027160346 | 0.078011991 | down |
| FP325317.1 | -0.797843586 | 0.327698545 | 0.466174095 | down |
| COL19A1 | -0.797669981 | 0.079216097 | 0.170937903 | down |
| SNORD66 | -0.797621163 | 0.682740858 | | down |
| VPS28 | -0.79750546 | 4.22E-05 | 0.000463458 | down |
| RP11-627G18.2 | -0.797375076 | 0.343849308 | | down |
| NA | -0.797324197 | 0.617516459 | | down |
| TMPRSS4 | -0.797029712 | 0.013719961 | 0.046623969 | down |
| TUFT1 | -0.796994935 | 7.61E-05 | 0.000752856 | down |
| RP11-474C8.8 | -0.796960806 | 0.279551071 | 0.415812597 | down |
| LINC00506 | -0.796931803 | 0.087241318 | 0.183460594 | down |
| RP13-923O23.7 | -0.796899374 | 0.024033482 | 0.071197233 | down |
| ATG4B | -0.796863718 | 5.75E-05 | 0.000598799 | down |
| RP11-455F5.6 | -0.796860612 | 0.071814578 | 0.15929201 | down |
| OR5BA1P | -0.796821868 | 0.090726926 | 0.188558368 | down |
| PDZD9 | -0.796800538 | 0.229441655 | 0.361855385 | down |
| RP11-861E21.2 | -0.796739601 | 0.068982149 | 0.15466078 | down |
| RP11-347P5.1 | -0.796571218 | 0.097442895 | 0.198312281 | down |
| AC011893.3 | -0.796458105 | 0.184790535 | 0.310836282 | down |
| IFI27 | -0.79642546 | 0.005485909 | 0.023266325 | down |
| KRT27 | -0.796411094 | 0.223314563 | 0.355363492 | down |
| IGSF22 | -0.796366132 | 0.006938879 | 0.027822193 | down |
| PPP1R9A | -0.796264983 | 0.001029406 | 0.00620474 | down |
| RP11-669B18.1 | -0.796077469 | 0.580176107 | | down |
| HMGB1P19 | -0.79607733 | 0.651572871 | | down |
| NDUFA3P2 | -0.796077252 | 0.678843133 | | down |
| RP11-344B2.3 | -0.796077135 | 0.709465103 | | down |
| BAATP1 | -0.796077123 | 0.712179473 | | down |
| IFNW1 | -0.79607711 | 0.715132103 | | down |
| SCARNA9 | -0.796077106 | 0.715976667 | | down |
| MIR150 | -0.796076941 | 0.745627816 | | down |
| RPL23AP8 | -0.796076935 | 0.746524571 | | down |
| RP6-127F18.2 | -0.796076732 | 0.772524448 | | down |
| STRA8 | -0.796076686 | 0.777370229 | | down |
| CTD-2306M5.1 | -0.796076601 | 0.785557341 | | down |

| | | | | |
|---|---|---|---|---|
| OR10C1 | -0.796076601 | 0.785557341 | | down |
| RP11-775B15.2 | -0.796076601 | 0.785557341 | | down |
| LYZL2 | -0.796076601 | 0.785557341 | | down |
| NA | -0.796076601 | 0.785557341 | | down |
| RPS8P3 | -0.796076601 | 0.785557341 | | down |
| RPS2P6 | -0.796076601 | 0.785557341 | | down |
| NA | -0.796004556 | 0.768440454 | | down |
| AC016683.5 | -0.795896322 | 0.188069096 | 0.314888336 | down |
| MDH1B | -0.795870381 | 0.05491344 | 0.131021235 | down |
| CTD-2095E4.4 | -0.795800609 | 0.159483328 | 0.280663813 | down |
| CKMT2 | -0.795702252 | 0.074159316 | 0.162987746 | down |
| TCEAL6 | -0.795305086 | 0.143434741 | 0.260714705 | down |
| CTD-2303H24.2 | -0.795245894 | 0.276842452 | 0.413043384 | down |
| CTD-2013N17.6 | -0.795243807 | 0.026275025 | 0.07604343 | down |
| TRMT61A | -0.795194991 | 0.000848907 | 0.005322323 | down |
| RP11-536K7.5 | -0.795163784 | 0.17054059 | 0.294413702 | down |
| RP1-239B22.5 | -0.79513872 | 0.005445639 | 0.023166162 | down |
| SLC7A7 | -0.795126674 | 0.000735669 | 0.004751823 | down |
| RP11-430B1.2 | -0.795003256 | 0.063250409 | 0.145304732 | down |
| OTUD7A | -0.794998686 | 0.000341045 | 0.002585945 | down |
| DRAIC | -0.794908729 | 0.010853225 | 0.039050813 | down |
| C1orf116 | -0.794905524 | 0.008737993 | 0.033065755 | down |
| NA | -0.794834192 | 0.785794043 | | down |
| RP6-109B7.5 | -0.794630369 | 0.106246496 | 0.21103198 | down |
| ZNF440 | -0.794565796 | 1.18E-05 | 0.000161093 | down |
| DDX10P1 | -0.794565639 | 0.434502231 | | down |
| VPS18 | -0.794355875 | 0.000432638 | 0.003098322 | down |
| CYP2T1P | -0.794322306 | 0.000555917 | 0.003792912 | down |
| FTH1P7 | -0.794175876 | 0.002203318 | 0.011341354 | down |
| RP11-138I1.2 | -0.794036207 | 0.419304103 | 0.555137964 | down |
| RIOK3 | -0.793787611 | 1.52E-06 | 2.94E-05 | down |
| CFAP99 | -0.793612136 | 0.048483091 | 0.119729245 | down |
| TRAJ34 | -0.793608049 | 0.511275601 | | down |
| HDAC11 | -0.793548866 | 6.57E-06 | 9.98E-05 | down |
| ALDH18A1 | -0.793385542 | 4.99E-07 | 1.12E-05 | down |
| LINC00924 | -0.793216128 | 0.112242739 | 0.219363629 | down |
| LINC01570 | -0.793176445 | 0.528638903 | 0.652014746 | down |
| KCNG1 | -0.793081447 | 0.001109086 | 0.006589837 | down |
| GLP2R | -0.793078965 | 0.064686671 | 0.147491252 | down |
| RPSA | -0.793076822 | 0.00090011 | 0.005567072 | down |
| CTD-2005H7.2 | -0.793014364 | 0.245717806 | 0.379691703 | down |
| RP11-400N9.1 | -0.792846946 | 0.147734803 | 0.266291363 | down |
| RNU4-62P | -0.792776771 | 0.081755795 | 0.174997941 | down |
| AP001469.5 | -0.792770934 | 0.201705186 | 0.330883391 | down |
| KRTAP5-9 | -0.7927541 | 0.075471308 | 0.165086275 | down |
| RP11-492M23.2 | -0.792700097 | 0.082261972 | 0.175880921 | down |
| ZBTB7B | -0.792629645 | 5.39E-05 | 0.000568217 | down |
| SPINT2 | -0.792500634 | 0.003391609 | 0.015958887 | down |
| GABRD | -0.792432118 | 0.063763655 | 0.146070549 | down |
| OCIAD2 | -0.792223637 | 1.82E-05 | 0.000232286 | down |
| PGK1P2 | -0.791702872 | 0.234254751 | 0.367018655 | down |

| | | | | |
|---|---|---|---|---|
| ZFY-AS1 | -0.791627754 | 0.359564132 | | down |
| C9orf84 | -0.791560479 | 0.153121046 | 0.272759861 | down |
| SYMPK | -0.791407788 | 0.000375021 | 0.00277721 | down |
| GADD45GIP1 | -0.791390304 | 0.005503835 | 0.023322596 | down |
| MYLPF | -0.791303754 | 0.03531819 | 0.094597459 | down |
| RP5-1166F10.1 | -0.791297667 | 0.786725512 | | down |
| AIRE | -0.79119689 | 0.086228988 | 0.181928632 | down |
| ACA64 | -0.790903924 | 0.378980639 | | down |
| ECSIT | -0.790822987 | 2.38E-06 | 4.28E-05 | down |
| TP53INP2 | -0.790429918 | 0.002102881 | 0.010915506 | down |
| XX-DJ76P10__A.2 | -0.790259876 | 0.644590849 | 0.74975174 | down |
| GK | -0.790165079 | 0.013440777 | 0.045908902 | down |
| RP11-552D4.1 | -0.790026284 | 0.633972901 | | down |
| H2AFZP1 | -0.790013367 | 0.462244957 | | down |
| PCDHA4 | -0.789780197 | 0.138415877 | 0.254342043 | down |
| TPI1 | -0.78972644 | 0.000429224 | 0.003078448 | down |
| OR7E115P | -0.789716924 | 0.371426031 | 0.509796497 | down |
| THBS4 | -0.789708117 | 0.058465404 | 0.137175681 | down |
| RP5-1180D12.1 | -0.789707233 | 0.06473231 | 0.147573977 | down |
| RP11-64C12.8 | -0.789698728 | 0.279378586 | 0.4156507 | down |
| DERA | -0.789629187 | 7.81E-05 | 0.000769728 | down |
| AC097721.2 | -0.789483464 | 0.211905491 | 0.342615896 | down |
| SPATA17 | -0.789386392 | 0.070995742 | 0.157882759 | down |
| CD151 | -0.789375485 | 0.002793691 | 0.013714649 | down |
| NA | -0.789367603 | 0.300983251 | 0.438755011 | down |
| HIF3A | -0.789180035 | 0.016602957 | 0.053873986 | down |
| RP11-1348G14.4 | -0.78904378 | 0.016525816 | 0.053687458 | down |
| CTD-2650P22.1 | -0.788894088 | 0.574820865 | | down |
| PTH1R | -0.788395636 | 0.010371829 | 0.037716197 | down |
| CALM2P3 | -0.788356705 | 0.237521063 | | down |
| RP11-395P13.4 | -0.788355216 | 0.545106389 | 0.666455312 | down |
| LINC01344 | -0.788337329 | 0.370457659 | 0.508917691 | down |
| SOX1 | -0.788171021 | 0.367318094 | 0.505853722 | down |
| OR52I2 | -0.788149188 | 0.582865221 | | down |
| CASC15 | -0.788023112 | 0.01598859 | 0.052406852 | down |
| ALG1L9P | -0.787894161 | 0.017558399 | 0.056293304 | down |
| RNU1-13P | -0.787744017 | 0.572729406 | | down |
| NIPAL1 | -0.787599995 | 0.021926184 | 0.066439765 | down |
| CPT2 | -0.787586582 | 2.37E-05 | 0.000288203 | down |
| RNU7-170P | -0.787243116 | 0.778419118 | | down |
| RP11-101E5.1 | -0.787238751 | 0.747159484 | | down |
| AC108868.3 | -0.787238598 | 0.746160339 | | down |
| AC133106.2 | -0.787233712 | 0.716562651 | | down |
| SYP-AS1 | -0.787233702 | 0.716504123 | | down |
| OR1L3 | -0.7872334 | 0.71480663 | | down |
| AC090957.2 | -0.787233299 | 0.71424132 | | down |
| CTD-2104P17.2 | -0.787227176 | 0.682371645 | | down |
| RP11-96C23.14 | -0.787214074 | 0.625485944 | | down |
| RP5-1086K13.1 | -0.787203976 | 0.027528852 | 0.078784228 | down |
| HMGN1P7 | -0.787198547 | 0.499881916 | 0.626970538 | down |
| RP11-433A10.3 | -0.787182754 | 0.523918947 | | down |

| | | | | |
|---|---|---|---|---|
| RP11-64K12.9 | -0.787145753 | 0.437062603 | | down |
| RP11-359E8.5 | -0.787054097 | 0.787908055 | | down |
| ZFYVE19 | -0.78700437 | 2.25E-06 | 4.09E-05 | down |
| RP5-828K20.1 | -0.786994213 | 0.46595307 | | down |
| RP11-384F7.2 | -0.7869841 | 0.266873847 | 0.402588766 | down |
| RP11-128B16.5 | -0.786944182 | 0.462109255 | | down |
| AP000442.4 | -0.786749079 | 0.044208892 | 0.111783534 | down |
| NA | -0.786745371 | 0.313294001 | 0.451942429 | down |
| DPYSL5 | -0.786694316 | 0.10190632 | 0.204787805 | down |
| PAGE2 | -0.786684315 | 0.386990285 | | down |
| RP11-324E6.6 | -0.786660945 | 0.747523961 | | down |
| LINC00491 | -0.7866603 | 0.747463509 | | down |
| AC073150.6 | -0.786315564 | 0.717211668 | | down |
| RP11-814P5.1 | -0.786294361 | 0.715469327 | | down |
| RP11-131L12.4 | -0.786024935 | 0.056630846 | 0.133954898 | down |
| RP13-254B10.1 | -0.785958738 | 0.108088108 | 0.213785326 | down |
| RP11-339B21.8 | -0.785910746 | 0.495597107 | | down |
| RN7SL383P | -0.785773278 | 0.684668954 | | down |
| LINC01096 | -0.785771747 | 0.229642795 | 0.361944609 | down |
| BCAN | -0.785634841 | 0.012675652 | 0.04396021 | down |
| RP6-201G10.2 | -0.78555603 | 0.112106565 | 0.219236454 | down |
| RP11-690G19.4 | -0.785541647 | 0.558486175 | 0.678110194 | down |
| PLA2G15 | -0.785498532 | 0.001970793 | 0.010358731 | down |
| ALAS2 | -0.785359719 | 0.186700646 | 0.313085187 | down |
| LGI1 | -0.7853538 | 0.097815761 | 0.198923617 | down |
| CCDC134 | -0.785155797 | 0.019325966 | 0.06051196 | down |
| LINC00264 | -0.785124996 | 0.09373175 | 0.193093574 | down |
| PCDHGA8 | -0.785098088 | 0.363261494 | 0.501669884 | down |
| BCAP29 | -0.784945825 | 0.080206587 | 0.172542368 | down |
| RP11-350J20.9 | -0.784843457 | 0.659653981 | | down |
| AC006486.10 | -0.784822721 | 0.032037543 | 0.088032064 | down |
| DDX50P1 | -0.784644512 | 0.272353146 | 0.408597719 | down |
| NA | -0.784637622 | 0.108811326 | 0.214890388 | down |
| MYBPC2 | -0.784614138 | 0.066981123 | 0.151329587 | down |
| RP11-5A1.1 | -0.784543549 | 0.69788103 | | down |
| RPL21P39 | -0.784381798 | 0.15903928 | 0.280195633 | down |
| OR7E99P | -0.784263152 | 0.632048873 | | down |
| PRDX5 | -0.78424409 | 0.000749275 | 0.004819358 | down |
| CHCHD10 | -0.784146596 | 0.00047768 | 0.003353132 | down |
| RP11-1191J2.2 | -0.784114748 | 0.265259104 | 0.400857327 | down |
| HMGCR | -0.784055632 | 0.000211657 | 0.001752284 | down |
| RP11-1260E13.4 | -0.784024812 | 0.041888689 | 0.107601212 | down |
| RP11-68I18.10 | -0.783911774 | 0.193634986 | 0.321750429 | down |
| AC010746.3 | -0.783870753 | 0.574387344 | | down |
| SLC14A1 | -0.783817423 | 0.021055012 | 0.064502577 | down |
| SNORA42 | -0.78371916 | 0.351889826 | | down |
| TXN2 | -0.783702706 | 4.78E-05 | 0.000514875 | down |
| ACSF3 | -0.783666617 | 8.83E-07 | 1.82E-05 | down |
| RP11-108P20.2 | -0.783502265 | 0.445443956 | | down |
| RP11-167J8.3 | -0.78345919 | 0.538245392 | | down |
| RP11-305L7.3 | -0.78322727 | 0.225630164 | 0.357684288 | down |

| | | | | |
|---|---|---|---|---|
| RP11-968A15.8 | -0.782985587 | 0.528146958 | | down |
| RNU6-678P | -0.782981299 | 0.493888823 | | down |
| RP11-318A15.2 | -0.782952914 | 0.229361763 | 0.361843353 | down |
| RP11-1036E20.9 | -0.78293423 | 0.169557487 | 0.293184904 | down |
| ASAH2B | -0.782814098 | 0.006428435 | 0.026294879 | down |
| COLCA1 | -0.782552465 | 0.007516738 | 0.029542875 | down |
| RP11-266L9.8 | -0.782361249 | 0.00277741 | 0.013648104 | down |
| MRRFP1 | -0.782201398 | 0.546244244 | | down |
| RP11-756P10.6 | -0.782198624 | 0.018859864 | 0.059392294 | down |
| CTC-336P14.1 | -0.782111387 | 0.110542581 | 0.217153217 | down |
| RP11-131L12.2 | -0.782107931 | 0.195643431 | 0.3241376 | down |
| HSPA8P16 | -0.78209267 | 0.771804137 | | down |
| RN7SL451P | -0.78201357 | 0.744809859 | | down |
| OXT | -0.78198768 | 0.048782423 | 0.120241128 | down |
| SERPINA5 | -0.78198458 | 0.046340614 | 0.115607191 | down |
| RP11-478C6.2 | -0.781883593 | 0.481942121 | | down |
| LRRC18 | -0.78187433 | 0.10452982 | 0.208379929 | down |
| FOXI2 | -0.781820539 | 0.198000841 | 0.326741008 | down |
| ATP6V0D2 | -0.781698007 | 0.083047577 | 0.177071751 | down |
| HN1 | -0.781280956 | 0.001478676 | 0.008239119 | down |
| SLC6A12 | -0.781258994 | 0.031397944 | 0.08673621 | down |
| RP11-64K12.1 | -0.781170286 | 0.288162231 | 0.425471431 | down |
| AP000436.4 | -0.78109709 | 0.511048844 | 0.636855592 | down |
| AL162151.4 | -0.781069695 | 0.721844337 | | down |
| ABCA5 | -0.781020723 | 1.26E-05 | 0.000170786 | down |
| ACAA2 | -0.780940329 | 1.95E-05 | 0.000245307 | down |
| RP11-1134I14.6 | -0.780819356 | 0.657084186 | | down |
| KB-1615E4.2 | -0.780743946 | 0.162169104 | 0.284093794 | down |
| AC073046.25 | -0.780679696 | 0.060141269 | 0.139938375 | down |
| RPS6KA1 | -0.780485074 | 1.53E-06 | 2.94E-05 | down |
| LINC01207 | -0.7804464 | 0.007632796 | 0.02990997 | down |
| RP11-462G2.1 | -0.780394678 | 0.182208772 | 0.307806701 | down |
| RP11-501J20.5 | -0.780255721 | 0.203516524 | 0.332951239 | down |
| RP11-752D24.2 | -0.780170496 | 0.301561192 | 0.439384309 | down |
| LGALS9DP | -0.779680704 | 0.2474126 | 0.381760781 | down |
| C1QL4 | -0.779593706 | 0.371681552 | 0.509970461 | down |
| CTD-2095E4.3 | -0.779467871 | 0.077404992 | 0.168116503 | down |
| UBE2Q2P6 | -0.779458947 | 0.514910269 | | down |
| MIR523 | -0.77919823 | 0.789994863 | | down |
| RP11-553E24.1 | -0.779192268 | 0.776418182 | | down |
| TBX15 | -0.779162783 | 0.193859417 | 0.321963091 | down |
| IFIT1B | -0.779161695 | 0.718620888 | | down |
| RP11-451O18.1 | -0.779161345 | 0.718042732 | | down |
| RP11-556I14.2 | -0.779160992 | 0.717459276 | | down |
| RP11-30P6.1 | -0.77915976 | 0.715436189 | | down |
| CTC-457E21.9 | -0.779141154 | 0.686820177 | | down |
| CTC-329D1.3 | -0.779137804 | 0.68200671 | | down |
| TRAJ36 | -0.779120535 | 0.658550761 | | down |
| BANF1P4 | -0.779120314 | 0.658264003 | | down |
| CMB9-22P13.1 | -0.779088957 | 0.002869816 | 0.014003694 | down |
| RP11-177C12.4 | -0.779069092 | 0.598897935 | | down |

| | | | | |
|---|---|---|---|---|
| BTF3P5 | -0.779006063 | 0.539773903 | | down |
| RP11-783K16.5 | -0.778924554 | 0.477808699 | | down |
| LINC00899 | -0.778894283 | 0.001386227 | 0.007837373 | down |
| CTD-2240E14.4 | -0.778857337 | 0.331688109 | | down |
| FTH1P5 | -0.778853339 | 0.193705204 | 0.321831508 | down |
| LINC00862 | -0.778757757 | 0.196306996 | 0.324771332 | down |
| MAST4-AS1 | -0.778752623 | 0.039968731 | 0.103899449 | down |
| RP11-91P24.5 | -0.778446312 | 0.12133122 | 0.231858234 | down |
| UGT8 | -0.778394828 | 0.005421291 | 0.023090056 | down |
| RP11-161M6.3 | -0.778134988 | 0.229571055 | 0.361888492 | down |
| RP11-2G1.1 | -0.778004594 | 0.676754727 | | down |
| KLHL6-AS1 | -0.777970607 | 0.374376179 | 0.512708834 | down |
| PDP2 | -0.777957872 | 4.78E-06 | 7.64E-05 | down |
| GS1-278J22.1 | -0.777907726 | 0.717320011 | | down |
| LSR | -0.777905134 | 0.00320637 | 0.015254504 | down |
| MORN1 | -0.777800065 | 3.76E-05 | 0.000422841 | down |
| ACCS | -0.777740082 | 0.00016394 | 0.001419839 | down |
| NA | -0.777728585 | 0.331711634 | | down |
| DEPDC1-AS1 | -0.777647014 | 0.303739054 | 0.441747374 | down |
| CTD-3195I5.3 | -0.777580113 | 0.055492118 | 0.131899145 | down |
| ALDH6A1 | -0.77744766 | 0.000237397 | 0.001927663 | down |
| IRS2 | -0.777405989 | 0.000490676 | 0.003427612 | down |
| RP3-406P24.4 | -0.777381327 | 0.517712473 | | down |
| MAPK3 | -0.776815916 | 1.25E-06 | 2.47E-05 | down |
| FAM83H | -0.776706912 | 0.00208983 | 0.010859019 | down |
| KIF25 | -0.776470289 | 0.044396292 | 0.112144124 | down |
| RNU6-790P | -0.776292702 | 0.594341354 | | down |
| RP11-138E2.1 | -0.776236972 | 0.271133989 | 0.407236393 | down |
| FIS1 | -0.776189158 | 0.001255653 | 0.007264554 | down |
| KRT72 | -0.776184437 | 0.189238226 | 0.316386937 | down |
| ZNHIT2 | -0.776167662 | 0.003321365 | 0.015681244 | down |
| RP4-592A1.2 | -0.776130709 | 0.065048704 | 0.148054792 | down |
| RP11-47909.4 | -0.775898863 | 0.058478422 | 0.1371884 | down |
| RP11-16F15.2 | -0.775810369 | 0.10890653 | 0.215021869 | down |
| CTD-2017F17.2 | -0.775799031 | 0.154985659 | 0.275070247 | down |
| STBD1 | -0.775787951 | 0.00079806 | 0.005067954 | down |
| NA | -0.775656466 | 0.620881598 | 0.730946973 | down |
| AC012442.6 | -0.775419338 | 0.065556243 | 0.148912691 | down |
| ALOXE3 | -0.775373125 | 0.24404782 | 0.377953156 | down |
| RP11-164O23.8 | -0.775338835 | 0.513947336 | | down |
| RPL5P29 | -0.775267325 | 0.320525067 | 0.459309624 | down |
| ADAM1B | -0.775105592 | 0.24338577 | 0.377431471 | down |
| NA | -0.774921931 | 0.100068306 | 0.202019364 | down |
| USP50 | -0.774854045 | 0.156021773 | 0.276386684 | down |
| RP11-713P17.4 | -0.774749397 | 0.311922003 | 0.450547339 | down |
| MON1B | -0.774726677 | 7.09E-06 | 0.000106077 | down |
| RP5-867C24.4 | -0.774594141 | 0.280987723 | 0.417368377 | down |
| RP11-108B14.4 | -0.774580049 | 0.31585522 | | down |
| TTC9B | -0.774571991 | 0.039473883 | 0.102920916 | down |
| NA | -0.774534534 | 0.282539448 | 0.419066705 | down |
| MZT2B | -0.774167945 | 0.02092952 | 0.064222909 | down |

| | | | | |
|---|---|---|---|---|
| ACTRT2 | -0.773955509 | 0.791372025 | | down |
| Metazoa_SRP | -0.773876423 | 0.745460746 | | down |
| FAM57B | -0.773836799 | 0.064810601 | 0.147671907 | down |
| TULP1 | -0.773785885 | 0.124912192 | 0.236556164 | down |
| SNRPCP2 | -0.77375726 | 0.690407973 | | down |
| MGAT5B | -0.773696869 | 0.063902085 | 0.146242706 | down |
| TYMS | -0.773613611 | 0.008957914 | 0.033713085 | down |
| RP11-358B23.1 | -0.773572924 | 0.294694182 | 0.432086486 | down |
| RNU6-431P | -0.773389132 | 0.543028301 | | down |
| RP11-379H8.1 | -0.773252449 | 0.631703748 | | down |
| PSPN | -0.773200002 | 0.00046333 | 0.00327536 | down |
| RP11-660M5.1 | -0.773086596 | 0.386043065 | | down |
| C1orf159 | -0.773031031 | 0.001617453 | 0.008866038 | down |
| RP11-588H23.3 | -0.772958287 | 0.038437653 | 0.100875335 | down |
| SUCLG1 | -0.772918319 | 9.44E-06 | 0.000134712 | down |
| RP11-92G12.3 | -0.772897351 | 0.294081884 | 0.431497393 | down |
| AK5 | -0.77284981 | 0.013700996 | 0.046570154 | down |
| ZMYND10 | -0.772761721 | 0.036633131 | 0.097286819 | down |
| RP11-900F13.2 | -0.772721463 | 0.492897279 | | down |
| RP1-149A16.12 | -0.772688778 | 0.563279557 | | down |
| BCAP31 | -0.772652595 | 0.001872593 | 0.009961083 | down |
| CPNE7 | -0.7726148 | 0.008353294 | 0.031962093 | down |
| ILF2P1 | -0.772596873 | 0.631834476 | | down |
| EFNA5 | -0.772332745 | 0.000926811 | 0.005711815 | down |
| NA | -0.77231743 | 0.06400214 | 0.146427071 | down |
| ABCA7 | -0.772246334 | 0.018810685 | 0.059277136 | down |
| RPL21P3 | -0.772199093 | 0.2159698 | | down |
| CACNA1I | -0.772093755 | 0.029303251 | 0.082479564 | down |
| LDB3 | -0.772078007 | 0.035164698 | 0.094371433 | down |
| PRDX6 | -0.771965321 | 0.000634177 | 0.004213447 | down |
| IQCH | -0.77194115 | 0.043486169 | 0.11058016 | down |
| P2RY2 | -0.771908726 | 0.006422786 | 0.02627893 | down |
| Y_RNA | -0.771907943 | 0.720935182 | | down |
| TCEANC | -0.771755052 | 0.000155048 | 0.001354542 | down |
| CPB1 | -0.771717757 | 0.225465416 | 0.357549117 | down |
| RP11-707A18.1 | -0.771649243 | 0.462021478 | | down |
| NA | -0.771600187 | 0.025104213 | 0.073533293 | down |
| DLG1 | -0.771559439 | 6.93E-06 | 0.000104166 | down |
| RP11-321N4.4 | -0.771464453 | 0.606777501 | | down |
| NA | -0.771417675 | 0.298678921 | 0.436243195 | down |
| RP11-336A10.2 | -0.771395578 | 0.661369971 | | down |
| PRKCZ | -0.771332918 | 0.000115789 | 0.001070056 | down |
| CDK10 | -0.771265099 | 0.00019221 | 0.001617392 | down |
| RP11-262H14.4 | -0.771165856 | 0.00483961 | 0.021170827 | down |
| RP11-740P5.2 | -0.770987716 | 0.792158129 | | down |
| RPL23AP89 | -0.770987716 | 0.792158129 | | down |
| RN7SL503P | -0.770987716 | 0.792158129 | | down |
| FTLP18 | -0.770987716 | 0.792158129 | | down |
| NA | -0.770987716 | 0.792158129 | | down |
| RNU6-880P | -0.770987716 | 0.792158129 | | down |
| RP11-141A19.1 | -0.770987716 | 0.792158129 | | down |

| | | | |
|---|---|---|---|
| RNU2-30P | -0.770987716 | 0.792158129 | down |
| RP1-158P9.1 | -0.770987716 | 0.792158129 | down |
| NA | -0.770987716 | 0.792158129 | down |
| CCNT2P1 | -0.770987716 | 0.792158129 | down |
| RNVU1-17 | -0.770987716 | 0.792158129 | down |
| OR6K2 | -0.770987716 | 0.792158129 | down |
| RNA5SP65 | -0.770987716 | 0.792158129 | down |
| RP1-69E11.3 | -0.770987716 | 0.792158129 | down |
| RN7SL230P | -0.770987716 | 0.792158129 | down |
| RN7SL837P | -0.770987716 | 0.792158129 | down |
| AC107057.1 | -0.770987716 | 0.792158129 | down |
| RP11-734K21.4 | -0.770987716 | 0.792158129 | down |
| RP11-245G13.1 | -0.770987716 | 0.792158129 | down |
| SNORD14 | -0.770987716 | 0.792158129 | down |
| Y_RNA | -0.770987716 | 0.792158129 | down |
| RP11-761B3.1 | -0.770987716 | 0.792158129 | down |
| AC008074.5 | -0.770987716 | 0.792158129 | down |
| RP11-292K15.1 | -0.770987716 | 0.792158129 | down |
| AC013402.3 | -0.770987716 | 0.792158129 | down |
| AC092664.1 | -0.770987716 | 0.792158129 | down |
| AC023469.2 | -0.770987716 | 0.792158129 | down |
| RP11-17G11.1 | -0.770987716 | 0.792158129 | down |
| AC079305.5 | -0.770987716 | 0.792158129 | down |
| MIR1258 | -0.770987716 | 0.792158129 | down |
| MTCO3P16 | -0.770987716 | 0.792158129 | down |
| AC009965.1 | -0.770987716 | 0.792158129 | down |
| RNA5SP120 | -0.770987716 | 0.792158129 | down |
| RNU6-787P | -0.770987716 | 0.792158129 | down |
| RP11-62G11.1 | -0.770987716 | 0.792158129 | down |
| RN7SL294P | -0.770987716 | 0.792158129 | down |
| HMGB1P38 | -0.770987716 | 0.792158129 | down |
| MTCO1P6 | -0.770987716 | 0.792158129 | down |
| RCC2P5 | -0.770987716 | 0.792158129 | down |
| HNRNPA1P17 | -0.770987716 | 0.792158129 | down |
| Y_RNA | -0.770987716 | 0.792158129 | down |
| RPL21P43 | -0.770987716 | 0.792158129 | down |
| RNU6-1233P | -0.770987716 | 0.792158129 | down |
| PEX5L-AS2 | -0.770987716 | 0.792158129 | down |
| RP11-259P15.3 | -0.770987716 | 0.792158129 | down |
| TPRG1-AS2 | -0.770987716 | 0.792158129 | down |
| RP11-302F12.2 | -0.770987716 | 0.792158129 | down |
| RNU6-573P | -0.770987716 | 0.792158129 | down |
| GRXCR1 | -0.770987716 | 0.792158129 | down |
| TMPRSS11BNL | -0.770987716 | 0.792158129 | down |
| RP11-468N14.6 | -0.770987716 | 0.792158129 | down |
| STATH | -0.770987716 | 0.792158129 | down |
| PCAT4 | -0.770987716 | 0.792158129 | down |
| RP11-274J2.1 | -0.770987716 | 0.792158129 | down |
| PDHA2 | -0.770987716 | 0.792158129 | down |
| MIR8066 | -0.770987716 | 0.792158129 | down |
| RP11-318I4.1 | -0.770987716 | 0.792158129 | down |

| | | | |
|---|---|---|---|
| RP11-392L5.2 | -0.770987716 | 0.792158129 | down |
| RNA5SP166 | -0.770987716 | 0.792158129 | down |
| RP11-84H6.1 | -0.770987716 | 0.792158129 | down |
| RP11-54F2.8 | -0.770987716 | 0.792158129 | down |
| RNA5SP184 | -0.770987716 | 0.792158129 | down |
| RN7SKP157 | -0.770987716 | 0.792158129 | down |
| RP11-346J10.3 | -0.770987716 | 0.792158129 | down |
| LINC00492 | -0.770987716 | 0.792158129 | down |
| CTD-2158P22.1 | -0.770987716 | 0.792158129 | down |
| CTB-77H17.1 | -0.770987716 | 0.792158129 | down |
| RN7SKP148 | -0.770987716 | 0.792158129 | down |
| RP11-1259L22.1 | -0.770987716 | 0.792158129 | down |
| RP11-451H23.2 | -0.770987716 | 0.792158129 | down |
| RNA5SP205 | -0.770987716 | 0.792158129 | down |
| RN7SKP240 | -0.770987716 | 0.792158129 | down |
| RP11-96J19.4 | -0.770987716 | 0.792158129 | down |
| GFRAL | -0.770987716 | 0.792158129 | down |
| RP1-288M22.1 | -0.770987716 | 0.792158129 | down |
| FAM8A6P | -0.770987716 | 0.792158129 | down |
| AC074389.6 | -0.770987716 | 0.792158129 | down |
| RP11-455J15.1 | -0.770987716 | 0.792158129 | down |
| GUSBP12 | -0.770987716 | 0.792158129 | down |
| ZNF734P | -0.770987716 | 0.792158129 | down |
| RP5-945F2.2 | -0.770987716 | 0.792158129 | down |
| NA | -0.770987716 | 0.792158129 | down |
| MTHFD2P5 | -0.770987716 | 0.792158129 | down |
| AC009263.2 | -0.770987716 | 0.792158129 | down |
| RP11-511P7.4 | -0.770987716 | 0.792158129 | down |
| AF238378.7 | -0.770987716 | 0.792158129 | down |
| RNU6-1151P | -0.770987716 | 0.792158129 | down |
| NDUFA5P12 | -0.770987716 | 0.792158129 | down |
| RP11-89A16.1 | -0.770987716 | 0.792158129 | down |
| RP11-333A23.3 | -0.770987716 | 0.792158129 | down |
| RP11-99H20.1 | -0.770987716 | 0.792158129 | down |
| IGJP1 | -0.770987716 | 0.792158129 | down |
| MIR548A3 | -0.770987716 | 0.792158129 | down |
| RP11-147I11.1 | -0.770987716 | 0.792158129 | down |
| RN7SL25P | -0.770987716 | 0.792158129 | down |
| MTCO3P30 | -0.770987716 | 0.792158129 | down |
| Y_RNA | -0.770987716 | 0.792158129 | down |
| VN1R48P | -0.770987716 | 0.792158129 | down |
| NA | -0.770987716 | 0.792158129 | down |
| RP11-15J10.8 | -0.770987716 | 0.792158129 | down |
| RP11-308N19.5 | -0.770987716 | 0.792158129 | down |
| U8 | -0.770987716 | 0.792158129 | down |
| RP11-445P17.6 | -0.770987716 | 0.792158129 | down |
| TCEB1P3 | -0.770987716 | 0.792158129 | down |
| FTLP19 | -0.770987716 | 0.792158129 | down |
| U3 | -0.770987716 | 0.792158129 | down |
| MKNK2P1 | -0.770987716 | 0.792158129 | down |
| UQCRHP3 | -0.770987716 | 0.792158129 | down |

| | | | |
|---|---|---|---|
| C10orf71-AS1 | -0.770987716 | 0.792158129 | down |
| SLC9A3P1 | -0.770987716 | 0.792158129 | down |
| MIR605 | -0.770987716 | 0.792158129 | down |
| ALDH7A1P4 | -0.770987716 | 0.792158129 | down |
| MRPL35P3 | -0.770987716 | 0.792158129 | down |
| RP11-96C23.10 | -0.770987716 | 0.792158129 | down |
| DDX18P6 | -0.770987716 | 0.792158129 | down |
| RP11-169K19.1 | -0.770987716 | 0.792158129 | down |
| RP11-282I1.2 | -0.770987716 | 0.792158129 | down |
| RP11-701I24.3 | -0.770987716 | 0.792158129 | down |
| CTD-2019O4.1 | -0.770987716 | 0.792158129 | down |
| OR4S1 | -0.770987716 | 0.792158129 | down |
| OR4C3 | -0.770987716 | 0.792158129 | down |
| CTD-2132H18.5 | -0.770987716 | 0.792158129 | down |
| RP11-810P12.6 | -0.770987716 | 0.792158129 | down |
| RP11-334E6.3 | -0.770987716 | 0.792158129 | down |
| OR10S1 | -0.770987716 | 0.792158129 | down |
| RPS27P20 | -0.770987716 | 0.792158129 | down |
| RP5-1154L15.1 | -0.770987716 | 0.792158129 | down |
| NA | -0.770987716 | 0.792158129 | down |
| RP11-588G21.1 | -0.770987716 | 0.792158129 | down |
| Y_RNA | -0.770987716 | 0.792158129 | down |
| RP3-446N13.4 | -0.770987716 | 0.792158129 | down |
| NA | -0.770987716 | 0.792158129 | down |
| NME1P1 | -0.770987716 | 0.792158129 | down |
| RP11-179A7.2 | -0.770987716 | 0.792158129 | down |
| FABP3P2 | -0.770987716 | 0.792158129 | down |
| NDFIP2-AS1 | -0.770987716 | 0.792158129 | down |
| RN7SL164P | -0.770987716 | 0.792158129 | down |
| RP11-573N10.1 | -0.770987716 | 0.792158129 | down |
| RP11-536C10.11 | -0.770987716 | 0.792158129 | down |
| BMS1P17 | -0.770987716 | 0.792158129 | down |
| OR4K5 | -0.770987716 | 0.792158129 | down |
| NA | -0.770987716 | 0.792158129 | down |
| RNU6-297P | -0.770987716 | 0.792158129 | down |
| RP11-242P2.2 | -0.770987716 | 0.792158129 | down |
| Y_RNA | -0.770987716 | 0.792158129 | down |
| RP11-467N20.6 | -0.770987716 | 0.792158129 | down |
| TVP23BP1 | -0.770987716 | 0.792158129 | down |
| RP11-100M12.1 | -0.770987716 | 0.792158129 | down |
| RP11-1H8.4 | -0.770987716 | 0.792158129 | down |
| RN7SL497P | -0.770987716 | 0.792158129 | down |
| RNA5SP393 | -0.770987716 | 0.792158129 | down |
| RP11-798K3.3 | -0.770987716 | 0.792158129 | down |
| SALRNA3 | -0.770987716 | 0.792158129 | down |
| Y_RNA | -0.770987716 | 0.792158129 | down |
| RNU1-77P | -0.770987716 | 0.792158129 | down |
| RP11-452K20.1 | -0.770987716 | 0.792158129 | down |
| snoMe28S-Am2634 | -0.770987716 | 0.792158129 | down |
| RP11-475D10.5 | -0.770987716 | 0.792158129 | down |
| ABHD17AP8 | -0.770987716 | 0.792158129 | down |

| | | | | |
|---|---|---|---|---|
| RNA5SP408 | -0.770987716 | 0.792158129 | | down |
| Metazoa_SRP | -0.770987716 | 0.792158129 | | down |
| RN7SKP76 | -0.770987716 | 0.792158129 | | down |
| SNORA48 | -0.770987716 | 0.792158129 | | down |
| RPL23AP73 | -0.770987716 | 0.792158129 | | down |
| RP11-477N12.6 | -0.770987716 | 0.792158129 | | down |
| GSG1L2 | -0.770987716 | 0.792158129 | | down |
| RP11-219A15.4 | -0.770987716 | 0.792158129 | | down |
| RNU6-258P | -0.770987716 | 0.792158129 | | down |
| MIR4522 | -0.770987716 | 0.792158129 | | down |
| MIR548AT | -0.770987716 | 0.792158129 | | down |
| ATP5LP7 | -0.770987716 | 0.792158129 | | down |
| RNU6-938P | -0.770987716 | 0.792158129 | | down |
| CTD-2533G20.1 | -0.770987716 | 0.792158129 | | down |
| RP11-63I8.1 | -0.770987716 | 0.792158129 | | down |
| RP11-202D1.2 | -0.770987716 | 0.792158129 | | down |
| RP11-325K19.3 | -0.770987716 | 0.792158129 | | down |
| Metazoa_SRP | -0.770987716 | 0.792158129 | | down |
| CTC-482H14.5 | -0.770987716 | 0.792158129 | | down |
| RN7SL823P | -0.770987716 | 0.792158129 | | down |
| CTD-2050I18.2 | -0.770987716 | 0.792158129 | | down |
| MIR520C | -0.770987716 | 0.792158129 | | down |
| RNU6-115P | -0.770987716 | 0.792158129 | | down |
| MRPS11P1 | -0.770987716 | 0.792158129 | | down |
| NA | -0.770987716 | 0.792158129 | | down |
| RNU6-929P | -0.770987716 | 0.792158129 | | down |
| NA | -0.770987716 | 0.792158129 | | down |
| LINC00317 | -0.770987716 | 0.792158129 | | down |
| AP000474.1 | -0.770987716 | 0.792158129 | | down |
| KRTAP13-4 | -0.770987716 | 0.792158129 | | down |
| AF015720.3 | -0.770987716 | 0.792158129 | | down |
| AP000688.11 | -0.770987716 | 0.792158129 | | down |
| MIR1286 | -0.770987716 | 0.792158129 | | down |
| L22NC03-84E4.1 | -0.770987716 | 0.792158129 | | down |
| RNA5SP495 | -0.770987716 | 0.792158129 | | down |
| CTA-992D9.6 | -0.770987716 | 0.792158129 | | down |
| RPEP4 | -0.770987716 | 0.792158129 | | down |
| CTA-250D10.19 | -0.770987716 | 0.792158129 | | down |
| RP11-459A10.1 | -0.770987716 | 0.792158129 | | down |
| Y_RNA | -0.770987716 | 0.792158129 | | down |
| RP3-389A20.2 | -0.770987716 | 0.792158129 | | down |
| AKAP4 | -0.770987716 | 0.792158129 | | down |
| RN7SL799P | -0.770987716 | 0.792158129 | | down |
| FAM46D | -0.770987716 | 0.792158129 | | down |
| RP3-326L13.2 | -0.770987716 | 0.792158129 | | down |
| FAM197Y1P | -0.770987716 | 0.792158129 | | down |
| ACTR3BP1 | -0.770987716 | 0.792158129 | | down |
| RP11-295P22.4 | -0.770987716 | 0.792158129 | | down |
| RP11-263I1.1 | -0.77098771 | 0.790173653 | | down |
| ASB4 | -0.770951136 | 0.247770956 | 0.382110941 | down |
| GPR155 | -0.770942234 | 0.002819872 | 0.013809328 | down |

| | | | | |
|---|---|---|---|---|
| RP11-44F21.3 | -0.770686474 | 0.313830833 | | down |
| RN7SL473P | -0.770619777 | 0.396608126 | | down |
| RP11-5407.3 | -0.770528386 | 0.003442277 | 0.016161865 | down |
| RP11-465L10.10 | -0.770486688 | 0.234400548 | 0.367170494 | down |
| NA | -0.770416562 | 0.03150242 | 0.086958721 | down |
| ZNF705E | -0.770348698 | 0.354028098 | | down |
| RP11-452L6.5 | -0.77032058 | 0.000351149 | 0.002646741 | down |
| ZMAT4 | -0.770244739 | 0.181756037 | 0.307314896 | down |
| TEX264 | -0.770234266 | 0.001178357 | 0.006913213 | down |
| HOXA5 | -0.769634894 | 0.000644646 | 0.004266967 | down |
| AC018738.2 | -0.769551006 | 0.015223223 | 0.050498676 | down |
| BTC | -0.76952247 | 0.062397433 | 0.143862211 | down |
| RP11-57G10.8 | -0.769390053 | 0.111894643 | 0.219007339 | down |
| GCNT3 | -0.769221794 | 0.038433886 | 0.100875335 | down |
| ANTXRL | -0.769111083 | 0.326770381 | 0.465186981 | down |
| AF001548.6 | -0.769046005 | 0.488674145 | | down |
| KYAT1 | -0.768782613 | 0.208411678 | 0.338590943 | down |
| HIST1H2BE | -0.768770867 | 0.091138862 | 0.18916587 | down |
| RP11-29H23.5 | -0.768631141 | 0.049581766 | 0.121568651 | down |
| CTB-25B13.12 | -0.768483947 | 0.00055215 | 0.00377275 | down |
| GRHL2 | -0.768364972 | 0.010216351 | 0.037283949 | down |
| EIF3LP3 | -0.768267315 | 0.422214306 | | down |
| DOCK7 | -0.76824402 | 2.46E-05 | 0.000297427 | down |
| ATP9A | -0.76817699 | 4.81E-05 | 0.000517458 | down |
| PSMA6P2 | -0.768167641 | 0.409492289 | 0.546207881 | down |
| CTB-191K22.6 | -0.768128412 | 0.27532001 | 0.411630474 | down |
| KRT8P10 | -0.768073797 | 0.095282571 | 0.195223549 | down |
| ANP32AP1 | -0.768054723 | 0.029861044 | 0.083494201 | down |
| SYNPR-AS1 | -0.768043976 | 0.148951194 | 0.267671881 | down |
| BEND3P1 | -0.768021115 | 0.083440252 | 0.177616475 | down |
| FSIP1 | -0.767958747 | 0.074591419 | 0.163697926 | down |
| BABAM1 | -0.767955251 | 0.007098521 | 0.028296133 | down |
| BOLA2B | -0.767884686 | 0.006271199 | 0.025792182 | down |
| VWA8-AS1 | -0.767703938 | 0.396356861 | | down |
| CTD-2050E21.2 | -0.767573866 | 0.661907317 | | down |
| IZUMO1R | -0.767431229 | 0.310355706 | 0.448910829 | down |
| CTC-421K24.1 | -0.767428295 | 0.42932674 | | down |
| DNAJB13 | -0.767337512 | 0.118004213 | 0.227468149 | down |
| CTNNA2 | -0.767065964 | 0.145501372 | 0.263244804 | down |
| GLP1R | -0.767027533 | 0.22193919 | 0.35378858 | down |
| C1orf229 | -0.767021114 | 0.058903585 | 0.137891484 | down |
| UQCRQ | -0.766939611 | 0.001369057 | 0.007763677 | down |
| NDUFB4P11 | -0.766924125 | 0.166252535 | 0.289340854 | down |
| RP11-401L13.4 | -0.766715613 | 0.028039003 | 0.079916879 | down |
| KLHL30 | -0.766660614 | 0.051809435 | 0.12542733 | down |
| DIO1 | -0.766478164 | 0.18513623 | 0.311278353 | down |
| RP11-71G12.1 | -0.766290753 | 0.171997881 | 0.296216929 | down |
| PCOLCE2 | -0.766289785 | 0.023633728 | 0.070245527 | down |
| C15orf54 | -0.766252649 | 0.536830903 | | down |
| ATP2C2 | -0.766147545 | 0.022713224 | 0.068193936 | down |
| NA | -0.766111444 | 0.3655436 | | down |

| | | | | |
|---|---|---|---|---|
| RP11-430L17.1 | -0.766095266 | 0.552144814 | | down |
| RP11-44N12.2 | -0.766053125 | 0.278707996 | 0.415002381 | down |
| CFAP46 | -0.765973714 | 0.059895832 | 0.13951856 | down |
| RP11-314N13.3 | -0.765634619 | 0.059062366 | 0.138166294 | down |
| AMDHD1 | -0.76556296 | 0.093483199 | 0.192674023 | down |
| ZNF703 | -0.765541167 | 0.000187116 | 0.001584629 | down |
| STK11 | -0.765383405 | 2.22E-05 | 0.000272145 | down |
| RP11-21B23.3 | -0.765369476 | 0.39817127 | 0.535146176 | down |
| RP11-132N15.3 | -0.76535311 | 0.286039986 | 0.423130741 | down |
| MGAT4A | -0.76525234 | 3.43E-07 | 8.12E-06 | down |
| HNRNPA3P15 | -0.765252145 | 0.609181535 | | down |
| LINC00630 | -0.765156193 | 0.080571831 | 0.17308023 | down |
| NMUR1 | -0.765085502 | 0.029014832 | 0.081927563 | down |
| LIPJ | -0.765011191 | 0.361637311 | 0.500001443 | down |
| LINC01347 | -0.764925184 | 0.109548993 | 0.215878915 | down |
| GAPDHP38 | -0.764682809 | 0.381784772 | | down |
| ASB14 | -0.764665832 | 0.017066465 | 0.054980826 | down |
| RP11-603J24.14 | -0.76457593 | 0.310761688 | 0.44920802 | down |
| CTD-2609K8.3 | -0.764372227 | 0.421814136 | | down |
| CCDC142 | -0.764107601 | 0.00061042 | 0.004095458 | down |
| C21orf62-AS1 | -0.764028878 | 0.018637548 | 0.058879757 | down |
| DACT2 | -0.764004622 | 0.016124499 | 0.052725778 | down |
| NA | -0.763967945 | 0.632030923 | 0.739811317 | down |
| PTPN14 | -0.763948497 | 0.008883073 | 0.033502768 | down |
| RP11-483P21.2 | -0.763917923 | 0.044383868 | 0.112122167 | down |
| TMEM30B | -0.763905706 | 0.004708518 | 0.020714999 | down |
| CFLAR-AS1 | -0.763771077 | 0.117708074 | 0.226970115 | down |
| RP11-221N13.3 | -0.763741431 | 0.386605264 | | down |
| C1QTNF9 | -0.763409608 | 0.129575569 | 0.242643256 | down |
| RP11-347I19.7 | -0.763317859 | 0.166551816 | 0.289660362 | down |
| NDUFA11 | -0.763247045 | 0.01256826 | 0.043663368 | down |
| COL4A5 | -0.763020633 | 0.038104914 | 0.100220961 | down |
| RP11-1006G14.1 | -0.76286404 | 0.372587978 | | down |
| RP13-516M14.8 | -0.76273936 | 0.147930354 | 0.266531907 | down |
| CRACR2A | -0.762561992 | 0.000124075 | 0.001132351 | down |
| NT5C3AP1 | -0.762364496 | 0.226504152 | 0.358615659 | down |
| RP11-506B6.6 | -0.761993582 | 0.112302051 | 0.219433066 | down |
| RP11-616M22.7 | -0.76172662 | 0.557945026 | | down |
| RP11-372B4.3 | -0.761672686 | 0.01925288 | 0.060379283 | down |
| NA | -0.761578666 | 0.794637488 | | down |
| EIF2S2P5 | -0.761578666 | 0.794637488 | | down |
| RPS3AP12 | -0.761578666 | 0.794637488 | | down |
| RNU1-92P | -0.761578666 | 0.794637488 | | down |
| PPIAL4D | -0.761578666 | 0.794637488 | | down |
| CYCSP52 | -0.761578666 | 0.794637488 | | down |
| OR10T2 | -0.761578666 | 0.794637488 | | down |
| RP4-560B9.4 | -0.761578666 | 0.794637488 | | down |
| U7 | -0.761578666 | 0.794637488 | | down |
| NA | -0.761578666 | 0.794637488 | | down |
| RP5-1099E6.3 | -0.761578666 | 0.794637488 | | down |
| TUBB8P10 | -0.761578666 | 0.794637488 | | down |

| | | | |
|---|---|---|---|
| RP4-614C10.4 | -0.761578666 | 0.794637488 | down |
| AC073464.4 | -0.761578666 | 0.794637488 | down |
| AC018865.5 | -0.761578666 | 0.794637488 | down |
| YY1P2 | -0.761578666 | 0.794637488 | down |
| RN7SKP179 | -0.761578666 | 0.794637488 | down |
| AC007879.6 | -0.761578666 | 0.794637488 | down |
| RN7SKP43 | -0.761578666 | 0.794637488 | down |
| AC114812.8 | -0.761578666 | 0.794637488 | down |
| RP11-40B20.1 | -0.761578666 | 0.794637488 | down |
| RP11-781E19.1 | -0.761578666 | 0.794637488 | down |
| OR5H15 | -0.761578666 | 0.794637488 | down |
| LSM3P4 | -0.761578666 | 0.794637488 | down |
| RNA5SP165 | -0.761578666 | 0.794637488 | down |
| RN7SL776P | -0.761578666 | 0.794637488 | down |
| SNORA57 | -0.761578666 | 0.794637488 | down |
| snoU13 | -0.761578666 | 0.794637488 | down |
| CTD-2582M21.1 | -0.761578666 | 0.794637488 | down |
| SUMO2P4 | -0.761578666 | 0.794637488 | down |
| RP11-466P24.5 | -0.761578666 | 0.794637488 | down |
| AC091962.3 | -0.761578666 | 0.794637488 | down |
| CTB-158E9.2 | -0.761578666 | 0.794637488 | down |
| CTB-47B11.1 | -0.761578666 | 0.794637488 | down |
| RP1-261G23.4 | -0.761578666 | 0.794637488 | down |
| GSTA8P | -0.761578666 | 0.794637488 | down |
| RP1-249F5.3 | -0.761578666 | 0.794637488 | down |
| AC002486.3 | -0.761578666 | 0.794637488 | down |
| RP11-89N17.3 | -0.761578666 | 0.794637488 | down |
| CYP3A52P | -0.761578666 | 0.794637488 | down |
| RP4-604G5.1 | -0.761578666 | 0.794637488 | down |
| RP11-362B23.1 | -0.761578666 | 0.794637488 | down |
| OR7E160P | -0.761578666 | 0.794637488 | down |
| RP11-403D15.2 | -0.761578666 | 0.794637488 | down |
| RP11-31K23.2 | -0.761578666 | 0.794637488 | down |
| RP11-527D15.1 | -0.761578666 | 0.794637488 | down |
| NA | -0.761578666 | 0.794637488 | down |
| Y_RNA | -0.761578666 | 0.794637488 | down |
| ATP5J2P3 | -0.761578666 | 0.794637488 | down |
| NA | -0.761578666 | 0.794637488 | down |
| RP11-341A22.1 | -0.761578666 | 0.794637488 | down |
| RP11-18B3.3 | -0.761578666 | 0.794637488 | down |
| MIR455 | -0.761578666 | 0.794637488 | down |
| RN7SL30P | -0.761578666 | 0.794637488 | down |
| MIR3911 | -0.761578666 | 0.794637488 | down |
| NA | -0.761578666 | 0.794637488 | down |
| ZNF33CP | -0.761578666 | 0.794637488 | down |
| NPAP1P2 | -0.761578666 | 0.794637488 | down |
| HBG1 | -0.761578666 | 0.794637488 | down |
| OR52L2P | -0.761578666 | 0.794637488 | down |
| RP5-1027O15.1 | -0.761578666 | 0.794637488 | down |
| RP1-53C18.3 | -0.761578666 | 0.794637488 | down |
| MIR670HG | -0.761578666 | 0.794637488 | down |

| | | | |
|---|---|---|---|
| CTD-2026G22.1 | -0.761578666 | 0.794637488 | down |
| RP11-179A16.2 | -0.761578666 | 0.794637488 | down |
| RP11-482L11.1 | -0.761578666 | 0.794637488 | down |
| TRIM51BP | -0.761578666 | 0.794637488 | down |
| TAS2R46 | -0.761578666 | 0.794637488 | down |
| CAPZA3 | -0.761578666 | 0.794637488 | down |
| RP11-31A23.1 | -0.761578666 | 0.794637488 | down |
| RP11-630C16.1 | -0.761578666 | 0.794637488 | down |
| RP11-60E8.3 | -0.761578666 | 0.794637488 | down |
| OR6C3 | -0.761578666 | 0.794637488 | down |
| RP11-114H23.2 | -0.761578666 | 0.794637488 | down |
| RN7SL330P | -0.761578666 | 0.794637488 | down |
| RP11-536G4.1 | -0.761578666 | 0.794637488 | down |
| RP11-818F20.4 | -0.761578666 | 0.794637488 | down |
| RP1-46F2.3 | -0.761578666 | 0.794637488 | down |
| RNU6-82P | -0.761578666 | 0.794637488 | down |
| RNU6-64P | -0.761578666 | 0.794637488 | down |
| SCAND3P1 | -0.761578666 | 0.794637488 | down |
| AE000661.36 | -0.761578666 | 0.794637488 | down |
| CTD-2560C21.1 | -0.761578666 | 0.794637488 | down |
| COX6CP11 | -0.761578666 | 0.794637488 | down |
| CTD-2050N2.1 | -0.761578666 | 0.794637488 | down |
| RP11-1123I8.1 | -0.761578666 | 0.794637488 | down |
| RP11-76E17.2 | -0.761578666 | 0.794637488 | down |
| NPIPA7 | -0.761578666 | 0.794637488 | down |
| RP11-467M13.2 | -0.761578666 | 0.794637488 | down |
| RP11-812E19.9 | -0.761578666 | 0.794637488 | down |
| OR1D4 | -0.761578666 | 0.794637488 | down |
| RP11-434D2.12 | -0.761578666 | 0.794637488 | down |
| AC087499.4 | -0.761578666 | 0.794637488 | down |
| RP11-118G23.3 | -0.761578666 | 0.794637488 | down |
| AC015849.12 | -0.761578666 | 0.794637488 | down |
| KATNBL1P3 | -0.761578666 | 0.794637488 | down |
| NA | -0.761578666 | 0.794637488 | down |
| RP11-64C12.7 | -0.761578666 | 0.794637488 | down |
| LINC01477 | -0.761578666 | 0.794637488 | down |
| AC119403.1 | -0.761578666 | 0.794637488 | down |
| MIR7974 | -0.761578666 | 0.794361201 | down |
| BNIP3P22 | -0.761578666 | 0.794637488 | down |
| CTD-2626G11.2 | -0.761578666 | 0.794637488 | down |
| BNIP3P38 | -0.761578666 | 0.794637488 | down |
| CEACAMP1 | -0.761578666 | 0.794637488 | down |
| TPRX1 | -0.761578666 | 0.794637488 | down |
| VN1R2 | -0.761578666 | 0.794637488 | down |
| RP5-859I17.3 | -0.761578666 | 0.794637488 | down |
| DYNLT3P1 | -0.761578666 | 0.794637488 | down |
| RNU6-123P | -0.761578666 | 0.794637488 | down |
| MIR3200 | -0.761578666 | 0.794637488 | down |
| Y_RNA | -0.761578666 | 0.794637488 | down |
| NHS-AS1 | -0.761578666 | 0.794637488 | down |
| RP11-265D20.1 | -0.761578666 | 0.794637488 | down |

| | | | | |
|---|---|---|---|---|
| YBX1P8 | -0.761578666 | 0.794637488 | | down |
| RP11-41L14.1 | -0.761578666 | 0.794637488 | | down |
| RN7SL644P | -0.761578663 | 0.792265606 | | down |
| RNA5SP429 | -0.761578626 | 0.760516792 | | down |
| NA | -0.761578606 | 0.73670918 | | down |
| RP11-440E5.1 | -0.761578603 | 0.73225455 | | down |
| NA | -0.761578601 | 0.729065268 | | down |
| RP4-669K10.8 | -0.761567162 | 0.109758578 | 0.216135986 | down |
| U1 | -0.761490032 | 0.117090357 | 0.226127319 | down |
| MIR1972-1 | -0.761378573 | 0.582850706 | | down |
| IGHV1-14 | -0.761371587 | 0.610123805 | | down |
| CCDC85B | -0.761283912 | 0.017145764 | 0.055194186 | down |
| MIR3180-1 | -0.761245307 | 0.334002996 | | down |
| THAP12P7 | -0.761001321 | 0.332861399 | 0.471187705 | down |
| RIN1 | -0.760945553 | 0.012742216 | 0.04410451 | down |
| PRR5-ARHGAP8 | -0.760877617 | 0.447719734 | | down |
| Y_RNA | -0.760552775 | 0.522685767 | 0.646849742 | down |
| KCTD21-AS1 | -0.760539962 | 0.021916653 | 0.066417583 | down |
| PAFAH2 | -0.760528921 | 2.18E-05 | 0.000268155 | down |
| AGAP1 | -0.760280806 | 0.0011825 | 0.00693481 | down |
| GCSAML | -0.76010878 | 0.079657415 | 0.171607298 | down |
| RN7SL842P | -0.760089655 | 0.511979645 | 0.637505742 | down |
| RP11-31I22.2 | -0.760088763 | 0.599582639 | 0.712940231 | down |
| RP11-449P15.2 | -0.759883898 | 0.014300028 | 0.048175604 | down |
| IBA57-AS1 | -0.759722153 | 0.068613995 | 0.154055038 | down |
| CTAGE8 | -0.759702999 | 0.131307157 | 0.245015355 | down |
| RP11-303E16.5 | -0.759527298 | 0.484816808 | | down |
| TBC1D3F | -0.759500579 | 0.604978171 | | down |
| MIR3143 | -0.759464927 | 0.477822606 | 0.607494493 | down |
| TACR2 | -0.759126711 | 0.07592796 | 0.165714834 | down |
| LMAN2 | -0.759126575 | 0.003644273 | 0.016911036 | down |
| ACO2 | -0.759121316 | 0.011392628 | 0.040568935 | down |
| OTUD7B | -0.758680989 | 6.86E-05 | 0.000690017 | down |
| RP11-127I20.8 | -0.758630192 | 0.063849016 | 0.146157353 | down |
| EMC9 | -0.75855217 | 0.000558951 | 0.003808536 | down |
| RP11-124G5.3 | -0.758549997 | 0.706435656 | | down |
| FAM90A2P | -0.758320874 | 0.487761762 | | down |
| LY6G6C | -0.758134875 | 0.166187918 | 0.289261909 | down |
| F11-AS1 | -0.757720351 | 0.408208395 | 0.544955033 | down |
| RN7SL698P | -0.7575588 | 0.726203472 | | down |
| NA | -0.75754855 | 0.011023081 | 0.039519981 | down |
| NA | -0.757400711 | 0.273803339 | 0.410097587 | down |
| RP11-500B12.1 | -0.757241213 | 0.230464664 | 0.362888669 | down |
| RPS26P47 | -0.757225051 | 0.110442229 | 0.217024577 | down |
| AC090602.2 | -0.757218527 | 0.110180713 | 0.216683207 | down |
| AP002954.3 | -0.757196542 | 0.289317308 | 0.42661149 | down |
| CD226 | -0.757185762 | 0.01418366 | 0.047842582 | down |
| FAM221A | -0.757055524 | 0.008800485 | 0.033266453 | down |
| SETP14 | -0.756775344 | 0.078740088 | 0.170216377 | down |
| SLC6A9 | -0.756709065 | 0.001748541 | 0.009434838 | down |
| AC068522.4 | -0.756551173 | 0.104210736 | 0.207912606 | down |

| | | | | |
|---|---|---|---|---|
| LMTK3 | -0.756514112 | 0.000277492 | 0.002189905 | down |
| AP000936.1 | -0.75650038 | 0.062898184 | 0.144683641 | down |
| RP11-867G23.1 | -0.75642571 | 0.329097939 | | down |
| RAB43P1 | -0.7561956 | 0.31043197 | 0.44899362 | down |
| JAKMIP2 | -0.75603808 | 0.027053216 | 0.077763759 | down |
| SH2D4A | -0.755859947 | 0.000386872 | 0.002843139 | down |
| RP11-18C24.8 | -0.75573327 | 0.17763107 | 0.302588187 | down |
| RP11-35609.2 | -0.75572948 | 0.158947656 | 0.280083482 | down |
| RP11-438F14.3 | -0.755702084 | 0.452346769 | 0.585076015 | down |
| COLGALT2 | -0.755681294 | 0.028804981 | 0.081481674 | down |
| SUPT4H1 | -0.755578454 | 0.557751744 | | down |
| RP11-105N14.2 | -0.755523963 | 0.479452653 | | down |
| L22NC03-30E12.1 | -0.755520532 | 0.619203144 | | down |
| SYT2 | -0.755443615 | 0.058656517 | 0.137441616 | down |
| NA | -0.755427509 | 0.31697612 | | down |
| RPS3AP26 | -0.755402956 | 0.001626839 | 0.008902868 | down |
| CTC-526N19.1 | -0.755370289 | 0.023572255 | 0.070098468 | down |
| FRMPD2 | -0.755315677 | 0.573986487 | | down |
| ATP2A1 | -0.755291607 | 0.013876793 | 0.047066423 | down |
| SLC9A7P1 | -0.755278335 | 0.228408551 | 0.360756308 | down |
| TMEM233 | -0.755253251 | 0.031648259 | 0.087243614 | down |
| ZNF497 | -0.755186874 | 0.067356509 | 0.151985408 | down |
| AC137934.1 | -0.754887248 | 0.035210923 | 0.094447583 | down |
| PTMAP3 | -0.75479358 | 0.524173874 | | down |
| NA | -0.75474764 | 0.677503844 | | down |
| RP11-313J2.1 | -0.754722801 | 0.057712133 | 0.135843257 | down |
| RP11-1008C21.2 | -0.754620687 | 0.016210215 | 0.05294871 | down |
| SHPK | -0.754496432 | 0.000377433 | 0.002788884 | down |
| NA | -0.754188251 | 0.19276676 | 0.320751247 | down |
| SORL1 | -0.754027137 | 0.000173263 | 0.00148432 | down |
| Y_RNA | -0.753976111 | 0.673974341 | | down |
| TGFA | -0.753945585 | 0.006007821 | 0.024979099 | down |
| SMPD2 | -0.753898132 | 0.000874692 | 0.005440836 | down |
| ZNF286B | -0.753768083 | 0.006107345 | 0.025301498 | down |
| RP3-424M6.4 | -0.753501889 | 0.067362458 | 0.151985408 | down |
| CTB-35F21.4 | -0.753483805 | 0.647805676 | | down |
| ZNF670-ZNF695 | -0.753394201 | 0.392020562 | 0.529555439 | down |
| ZNF414 | -0.753221838 | 0.00126814 | 0.007313309 | down |
| RP11-303E16.9 | -0.753207386 | 0.225708978 | 0.357752601 | down |
| KCNQ1 | -0.752860439 | 0.002337693 | 0.011916124 | down |
| CREG2 | -0.752821436 | 0.178481249 | 0.303571707 | down |
| FIGN | -0.752760545 | 0.033125344 | 0.090203737 | down |
| CTD-2288F12.1 | -0.752739096 | 0.06223095 | 0.143610619 | down |
| COLEC11 | -0.752599623 | 0.02769251 | 0.079139519 | down |
| LIPI | -0.752496221 | 0.517622174 | | down |
| RP11-313E19.1 | -0.752372357 | 0.577027677 | 0.693781797 | down |
| RP11-542A14.2 | -0.752169622 | 0.764508045 | | down |
| SNRPCP14 | -0.752169622 | 0.762068629 | | down |
| NA | -0.752169621 | 0.765188684 | | down |
| MIR194-1 | -0.752169621 | 0.767969477 | | down |
| RP11-449J10.1 | -0.752169621 | 0.766486395 | | down |

| | | | | |
|---|---|---|---|---|
| RPL36AP40 | -0.752169617 | 0.795345621 | | down |
| AP000936.4 | -0.752169617 | 0.79521841 | | down |
| RP1-161N10.1 | -0.752169617 | 0.795917677 | | down |
| RP11-104D3.2 | -0.752169616 | 0.797118923 | | down |
| BTF3P9 | -0.752169616 | 0.796542526 | | down |
| OR2T29 | -0.752169616 | 0.797118923 | | down |
| AC092484.1 | -0.752169616 | 0.797118923 | | down |
| RPL23AP29 | -0.752169616 | 0.797118923 | | down |
| AC079354.5 | -0.752169616 | 0.797118923 | | down |
| NA | -0.752169616 | 0.797118923 | | down |
| RBM43P1 | -0.752169616 | 0.797118923 | | down |
| GPR79 | -0.752169616 | 0.79641436 | | down |
| RP11-259O2.2 | -0.752169616 | 0.797118923 | | down |
| NA | -0.752169616 | 0.797118923 | | down |
| CTD-2045M21.1 | -0.752169616 | 0.797118923 | | down |
| RP1-149L1.1 | -0.752169616 | 0.797118923 | | down |
| RP3-466I7.1 | -0.752169616 | 0.797118923 | | down |
| RP11-438N5.2 | -0.752169616 | 0.797118923 | | down |
| RP11-347H15.2 | -0.752169616 | 0.79618131 | | down |
| RP11-49G2.4 | -0.752169616 | 0.797118923 | | down |
| NA | -0.752169616 | 0.797118923 | | down |
| RP11-797M17.1 | -0.752169616 | 0.797118923 | | down |
| RP11-1085N6.5 | -0.752169616 | 0.797118923 | | down |
| AC087163.3 | -0.752169616 | 0.797118923 | | down |
| AC008753.4 | -0.752169616 | 0.797118923 | | down |
| MIR3192 | -0.752169616 | 0.797118923 | | down |
| RP11-366F6.2 | -0.752169616 | 0.797118923 | | down |
| LARP1P1 | -0.751701886 | 0.559747214 | | down |
| RPP25L | -0.75163949 | 0.000256235 | 0.002053438 | down |
| RIMS4 | -0.751619862 | 0.118076758 | 0.227507301 | down |
| AC074141.3 | -0.751468419 | 0.063940264 | 0.146296649 | down |
| DOC2A | -0.751372332 | 0.07480299 | 0.164054386 | down |
| NCR3LG1 | -0.751300182 | 0.071112234 | 0.15810872 | down |
| ALDH3A2 | -0.751270879 | 5.51E-07 | 1.23E-05 | down |
| CORO7 | -0.751270453 | 0.003685178 | 0.017067594 | down |
| NA | -0.751201825 | 0.473916604 | | down |
| SERBP1P3 | -0.751198648 | 0.06422397 | 0.146744615 | down |
| MGLL | -0.751039859 | 3.21E-05 | 0.000369855 | down |
| RP5-1033H22.2 | -0.750921506 | 0.013597606 | 0.046336334 | down |
| AC104623.2 | -0.750918385 | 0.538615758 | | down |
| LINC00989 | -0.750789731 | 0.15363342 | 0.273313847 | down |
| SLC35F3 | -0.750341556 | 0.118978119 | 0.228729115 | down |
| RP5-919F19.5 | -0.750314838 | 0.439720351 | 0.573591448 | down |
| RP4-547N15.3 | -0.750234763 | 0.352295712 | | down |
| STPG1 | -0.74996038 | 1.98E-08 | 7.05E-07 | down |
| CTSG | -0.749656988 | 0.026444458 | 0.076430717 | down |
| ZDHHC11 | -0.749619957 | 0.012271236 | 0.042879393 | down |
| MAP1LC3A | -0.749453368 | 0.001258366 | 0.00727325 | down |
| LKAAEAR1 | -0.749335567 | 0.132966893 | 0.247112639 | down |
| FAM86FP | -0.748876137 | 0.007169288 | 0.028495049 | down |
| MYLKP1 | -0.748841514 | 0.755927179 | | down |

| | | | | |
|---|---|---|---|---|
| OLA1P1 | -0.748689621 | 0.224848689 | 0.356991704 | down |
| FOXP1-IT1 | -0.748426444 | 0.558014848 | | down |
| NA | -0.748383576 | 0.015423324 | 0.051038479 | down |
| CTD-2521M24.4 | -0.748381439 | 0.134618739 | 0.249151967 | down |
| RP3-382I10.7 | -0.748378154 | 0.291900425 | 0.42911662 | down |
| AC092933.3 | -0.748241203 | 0.233432912 | 0.366119783 | down |
| F2 | -0.748240953 | 0.353403026 | | down |
| RP11-539L10.2 | -0.748199755 | 0.033334777 | 0.090650801 | down |
| CH507-9B2.3 | -0.748148653 | 0.002165449 | 0.011183695 | down |
| SNORD3B-2 | -0.748129979 | 0.129092252 | 0.242070129 | down |
| RPS2P2 | -0.747863202 | 0.42944542 | | down |
| RXFP1 | -0.747816473 | 0.088800514 | 0.185736747 | down |
| ACVR2A | -0.747815307 | 0.001601041 | 0.008788309 | down |
| RP11-58H15.3 | -0.747764535 | 0.783008858 | | down |
| LINC00896 | -0.74768112 | 0.085743651 | 0.181261066 | down |
| QTRT1 | -0.747641694 | 0.000144923 | 0.001282504 | down |
| RP11-7F17.8 | -0.747441872 | 0.009718738 | 0.035844556 | down |
| WI2-89031B12.1 | -0.747427089 | 0.19044143 | 0.31793856 | down |
| PRC1 | -0.74737097 | 0.173730181 | 0.298269098 | down |
| DDN | -0.747188574 | 0.184734771 | 0.310777281 | down |
| DNASE1L3 | -0.74715404 | 0.002334306 | 0.011904919 | down |
| B3GNT5 | -0.747129856 | 0.00700891 | 0.028028175 | down |
| PLLP | -0.747124733 | 0.001924011 | 0.010167984 | down |
| RP11-374M1.4 | -0.747105695 | 0.501336198 | 0.628374832 | down |
| TRAF4 | -0.746789224 | 2.73E-07 | 6.73E-06 | down |
| RPL23P4 | -0.746771974 | 0.730162645 | | down |
| LINC01151 | -0.746675876 | 0.487389378 | 0.616137611 | down |
| FTCDNL1 | -0.746666445 | 0.0075265 | 0.029563999 | down |
| RNF157 | -0.746565837 | 0.000356335 | 0.00267643 | down |
| AC009120.11 | -0.746461159 | 0.230848091 | 0.363272577 | down |
| RP4-751H13.7 | -0.746350164 | 0.168806203 | 0.292252843 | down |
| TLR3 | -0.746206269 | 0.008367908 | 0.031997637 | down |
| CCDC92B | -0.74604041 | 0.381197343 | 0.519043416 | down |
| CTD-3195I5.4 | -0.745902294 | 0.34528909 | 0.483878232 | down |
| AP000289.6 | -0.745825678 | 0.518136464 | 0.642941184 | down |
| SUCLG2 | -0.745804784 | 1.37E-05 | 0.000182868 | down |
| HIST1H3F | -0.745528439 | 0.179101633 | 0.30423076 | down |
| HNRNPA3P9 | -0.745510281 | 0.494794214 | | down |
| EXOC3 | -0.745479475 | 4.61E-06 | 7.42E-05 | down |
| TMEM256 | -0.745464069 | 0.026783272 | 0.077150203 | down |
| NA | -0.745281373 | 0.177527856 | 0.302508606 | down |
| TKTL1 | -0.745274666 | 0.074209252 | 0.163073638 | down |
| SLC27A6 | -0.74524284 | 0.156468254 | 0.27696531 | down |
| RP11-177G23.2 | -0.745155249 | 0.433518942 | 0.568538264 | down |
| RP11-930P14.1 | -0.745077594 | 0.602603375 | | down |
| TOM1 | -0.744929637 | 0.001674705 | 0.009110057 | down |
| CTD-3162L10.5 | -0.744896684 | 0.587913237 | | down |
| LINC01276 | -0.744565683 | 0.301676143 | 0.439466546 | down |
| F2RL1 | -0.744483633 | 0.037982895 | 0.099943781 | down |
| TUBB8 | -0.744388225 | 0.383604527 | 0.521328738 | down |
| LRRC39 | -0.74436383 | 0.123065611 | 0.234130143 | down |

| | | | | |
|---|---|---|---|---|
| NA | -0.744291043 | 0.356843146 | 0.495333371 | down |
| LINC00942 | -0.74422907 | 0.180250109 | 0.305490736 | down |
| RP11-355B11.2 | -0.744197159 | 0.003557332 | 0.016600884 | down |
| RP11-145A3.2 | -0.744176059 | 0.645960534 | | down |
| NDUFB10 | -0.744045531 | 0.00012268 | 0.001123036 | down |
| PXMP4 | -0.743989473 | 1.45E-08 | 5.51E-07 | down |
| TRDMT1 | -0.743966364 | 0.000656872 | 0.004335468 | down |
| LURAP1L-AS1 | -0.743947813 | 0.542048648 | 0.663460832 | down |
| C2orf15 | -0.743708376 | 0.006950732 | 0.027854849 | down |
| RP11-169D4.3 | -0.74355402 | 0.583797848 | | down |
| TPTE2P5 | -0.743449342 | 0.098756366 | 0.200257351 | down |
| CHP1 | -0.743430231 | 9.12E-05 | 0.000876402 | down |
| FAAP20 | -0.743421756 | 0.007359523 | 0.029058946 | down |
| RP11-107F6.4 | -0.743297581 | 0.229566796 | 0.361888492 | down |
| AC005592.2 | -0.743183386 | 0.414154736 | 0.550739827 | down |
| GS1-57L11.1 | -0.743138548 | 0.133282524 | 0.247391774 | down |
| MLANA | -0.743043379 | 0.114403371 | 0.222321722 | down |
| Y_RNA | -0.742829077 | 0.358668036 | 0.496925178 | down |
| RP11-127I20.5 | -0.742760689 | 0.6692947 | | down |
| XXbac-B33L19.12 | -0.742760686 | 0.674190586 | | down |
| TRAJ28 | -0.742760671 | 0.704319759 | | down |
| NA | -0.74276067 | 0.705163117 | | down |
| MIR3649 | -0.74276065 | 0.733597793 | | down |
| CDSN | -0.742760622 | 0.76238081 | | down |
| SDHCP2 | -0.742760622 | 0.762702491 | | down |
| RP11-44F21.2 | -0.742760621 | 0.763463944 | | down |
| MIR4525 | -0.742760621 | 0.763781307 | | down |
| MIR4449 | -0.74276062 | 0.763904091 | | down |
| RP11-480I12.10 | -0.742760619 | 0.765094417 | | down |
| RP11-1D12.1 | -0.742760588 | 0.787204516 | | down |
| RNU6-25P | -0.742760585 | 0.789268501 | | down |
| CBX3P3 | -0.742760582 | 0.791238052 | | down |
| UGT2B27P | -0.742760581 | 0.791797859 | | down |
| RXFP3 | -0.742760579 | 0.792675947 | | down |
| LINC00443 | -0.742760578 | 0.793392402 | | down |
| TDRG1 | -0.742760574 | 0.795769416 | | down |
| WFDC8 | -0.742760574 | 0.795508559 | | down |
| RP3-408N23.4 | -0.742760574 | 0.795581476 | | down |
| NA | -0.742760573 | 0.796068235 | | down |
| NA | -0.742760567 | 0.799445421 | | down |
| RP11-184I16.3 | -0.742760566 | 0.79960241 | | down |
| HNRNPA1P63 | -0.742760566 | 0.79960241 | | down |
| HAUS4P1 | -0.742760566 | 0.79960241 | | down |
| RP11-978I15.10 | -0.742760566 | 0.79960241 | | down |
| RN7SL352P | -0.742760566 | 0.79960241 | | down |
| RP11-20I6.1 | -0.742760566 | 0.79960241 | | down |
| NA | -0.742760566 | 0.79960241 | | down |
| RP11-349G13.3 | -0.742760566 | 0.79960241 | | down |
| RP11-430E17.1 | -0.742760566 | 0.79960241 | | down |
| RNF225 | -0.742760566 | 0.79960241 | | down |
| RP1-154K9.2 | -0.742760566 | 0.79960241 | | down |

| | | | | |
|---|---|---|---|---|
| MROH6 | -0.742695565 | 0.004582325 | 0.020273006 | down |
| CD164L2 | -0.742556119 | 0.231103796 | 0.363644014 | down |
| GOLGA6L3 | -0.742468575 | 0.391704282 | | down |
| CTD-2319I12.4 | -0.742405507 | 0.043078323 | 0.109830715 | down |
| C19orf57 | -0.742398526 | 0.006529373 | 0.026599117 | down |
| ERICH5 | -0.74216846 | 0.02404194 | 0.071209677 | down |
| CNTD2 | -0.742153383 | 0.095258347 | 0.19522113 | down |
| RP11-252P19.1 | -0.742112795 | 0.599682744 | 0.712974609 | down |
| RP3-395M20.12 | -0.742006881 | 0.012363838 | 0.043127732 | down |
| NUGGC | -0.741961447 | 0.062812162 | 0.144563244 | down |
| RP11-344B5.4 | -0.741915918 | 0.589306033 | | down |
| BAALC-AS2 | -0.741808035 | 0.334475047 | | down |
| CCDC85C | -0.741581703 | 0.000658863 | 0.004345746 | down |
| MIS18A-AS1 | -0.741347922 | 0.206061038 | 0.335969403 | down |
| SLC8A3 | -0.741154198 | 0.128617025 | 0.24151062 | down |
| CTD-2308N23.2 | -0.741088717 | 0.605664182 | | down |
| SATB2 | -0.741063145 | 0.007939173 | 0.030781327 | down |
| RP11-7I15.4 | -0.741003827 | 0.2411661 | 0.374972535 | down |
| RP11-165H4.2 | -0.740964661 | 0.06638515 | 0.150302917 | down |
| AP000356.2 | -0.740714195 | 0.610228359 | 0.721999517 | down |
| TM4SF1-AS1 | -0.740524672 | 0.203352927 | 0.332801598 | down |
| C9orf50 | -0.740440545 | 0.123985682 | 0.235344064 | down |
| LGR4 | -0.740180297 | 0.013109298 | 0.045022375 | down |
| ZNF285 | -0.740053802 | 0.025452964 | 0.074250787 | down |
| DGKQ | -0.739899239 | 9.62E-06 | 0.000136754 | down |
| RASD2 | -0.739877053 | 0.000639783 | 0.004244127 | down |
| LINC01624 | -0.739724919 | 0.074966215 | 0.164256487 | down |
| RP11-196G11.4 | -0.73950038 | 0.222470851 | 0.354358724 | down |
| AP000688.15 | -0.739479904 | 0.677002917 | 0.775827731 | down |
| NA | -0.73935924 | 0.70572773 | | down |
| PDXK | -0.739353153 | 0.000765739 | 0.004903207 | down |
| XAB2 | -0.739230665 | 0.000326048 | 0.002493836 | down |
| U82671.8 | -0.739227405 | 0.671695583 | | down |
| CNKSR3 | -0.738725343 | 0.002440156 | 0.012323426 | down |
| RP11-535M15.2 | -0.738720405 | 0.107104496 | 0.21234311 | down |
| RP3-500L14.2 | -0.738703934 | 0.276640564 | 0.412867769 | down |
| PPDPF | -0.738703334 | 0.001810813 | 0.009704616 | down |
| RP5-944M2.3 | -0.738689984 | 0.638367176 | | down |
| RP11-6N17.3 | -0.738537491 | 0.123978908 | 0.235344064 | down |
| CIB3 | -0.7385359 | 0.213770678 | 0.344646965 | down |
| CSNK1G2-AS1 | -0.738487533 | 0.291795935 | 0.429018508 | down |
| AC142472.6 | -0.73846987 | 0.016501849 | 0.053621194 | down |
| ACBD7 | -0.738302121 | 0.014573539 | 0.048839643 | down |
| RP1-136B1.1 | -0.738232889 | 0.175343048 | 0.299846692 | down |
| ENPP7P7 | -0.738185618 | 0.349050287 | 0.487734012 | down |
| RP1-30E17.2 | -0.738170591 | 0.505771135 | 0.632140277 | down |
| RP11-154H23.3 | -0.737793068 | 0.118935293 | 0.228661415 | down |
| ARL4A | -0.737732346 | 1.62E-05 | 0.000211405 | down |
| RP11-1263C18.1 | -0.73764369 | 0.351718907 | 0.490484821 | down |
| ZPBP2 | -0.737568075 | 0.262999314 | 0.398299758 | down |
| RP11-398E10.1 | -0.737565577 | 0.426646153 | | down |

| | | | | |
|---|---|---|---|---|
| NPIPB8 | -0.737479038 | 0.653201254 | | down |
| RP11-462D18.4 | -0.737363486 | 0.46866095 | | down |
| NR1H3 | -0.737345014 | 0.000132723 | 0.001196015 | down |
| LINC00642 | -0.737241683 | 0.477390575 | | down |
| MYL6 | -0.737070131 | 0.006432113 | 0.026302763 | down |
| KAT6B | -0.736888202 | 0.00095263 | 0.005827902 | down |
| NA | -0.73687129 | 0.545055631 | | down |
| RP11-245J9.5 | -0.73685561 | 0.135788985 | 0.250823501 | down |
| SRMS | -0.736799736 | 0.058141221 | 0.136625571 | down |
| NA | -0.736689704 | 0.646766211 | 0.751630945 | down |
| BPNT1 | -0.736642227 | 3.77E-08 | 1.23E-06 | down |
| YIPF4 | -0.736621481 | 0.000316322 | 0.002433068 | down |
| GCNT1 | -0.736583364 | 0.005051291 | 0.021870727 | down |
| CRYBB2P1 | -0.736397477 | 0.002354205 | 0.011979968 | down |
| CT55 | -0.736393419 | 0.589605001 | | down |
| SNHG11 | -0.736144331 | 0.000436523 | 0.003121111 | down |
| RP11-507E23.1 | -0.736126356 | 0.03283003 | 0.089635172 | down |
| RP11-415I12.2 | -0.736058854 | 0.736294075 | | down |
| VPS4A | -0.736039774 | 0.001299668 | 0.007450338 | down |
| RP11-805F19.2 | -0.735950972 | 0.55788891 | 0.677494519 | down |
| RP11-289K10.1 | -0.735569496 | 0.686615601 | 0.7832585 | down |
| UCP1 | -0.735278541 | 0.54089498 | 0.662642668 | down |
| CDH15 | -0.734913599 | 0.163802756 | 0.286104877 | down |
| RP11-90L1.8 | -0.734804964 | 0.151411168 | 0.270628658 | down |
| RP11-284B18.3 | -0.734773185 | 0.229439218 | | down |
| HMGN2P20 | -0.73460897 | 0.608333014 | | down |
| GALNT8 | -0.734520986 | 0.040551721 | 0.104965615 | down |
| LUC7L3 | -0.734475164 | 0.00040483 | 0.002937197 | down |
| DNAJB5-AS1 | -0.734219985 | 0.226998544 | 0.359133452 | down |
| FREM3 | -0.734051895 | 0.432614874 | 0.567600387 | down |
| RP11-114J13.1 | -0.734042899 | 0.592150251 | | down |
| ZBTB7C | -0.73377848 | 0.003790983 | 0.017460721 | down |
| NA | -0.733757762 | 0.704438971 | | down |
| FAM178B | -0.733750198 | 0.054885177 | 0.130985007 | down |
| CELSR2 | -0.7336723 | 0.008333392 | 0.031906261 | down |
| CTC-499B15.5 | -0.733669388 | 0.17683566 | 0.301763636 | down |
| DHDDS | -0.733643312 | 7.63E-06 | 0.000112875 | down |
| RP11-173P15.10 | -0.733571763 | 0.186176868 | 0.312468455 | down |
| PNPT1P2 | -0.73336985 | 0.717977143 | 0.807294359 | down |
| RP11-315E17.1 | -0.733369088 | 0.683806153 | | down |
| ATP5F1P7 | -0.733351764 | 0.651609324 | | down |
| LINC01526 | -0.733351746 | 0.676309435 | | down |
| SNORA38B | -0.733351746 | 0.676034506 | | down |
| NA | -0.733351724 | 0.69887465 | | down |
| AC125634.1 | -0.733351717 | 0.705912 | | down |
| RP11-712P20.2 | -0.733351714 | 0.708292989 | | down |
| MIR337 | -0.733351681 | 0.732970652 | | down |
| CTC-391G2.1 | -0.733351678 | 0.734913193 | | down |
| RP11-305L7.5 | -0.733351629 | 0.762097515 | | down |
| RP11-153N17.1 | -0.733351625 | 0.764205844 | | down |
| DPPA5 | -0.733351624 | 0.764661615 | | down |

| | | | | |
|---|---|---|---|---|
| MIR6769A | -0.733351624 | 0.764626766 | | down |
| KCNE2 | -0.733351621 | 0.765822757 | | down |
| TRAJ26 | -0.733351618 | 0.767287651 | | down |
| RP11-597M17.1 | -0.733351617 | 0.767738188 | | down |
| NA | -0.73335154 | 0.795450632 | | down |
| RNA5SP74 | -0.733351516 | 0.802087926 | | down |
| AC079790.2 | -0.733351516 | 0.802087926 | | down |
| RP11-120A1.1 | -0.733351516 | 0.802087926 | | down |
| TECRL | -0.733351516 | 0.802087926 | | down |
| RP4-669B10.6 | -0.733351516 | 0.802087926 | | down |
| RP11-1141N12.1 | -0.733351516 | 0.802087926 | | down |
| OR11J2P | -0.733351516 | 0.802087926 | | down |
| RP5-1055C14.6 | -0.733351516 | 0.802087926 | | down |
| PSMB11 | -0.733331783 | 0.492440218 | 0.620601623 | down |
| B3GNT3 | -0.733266501 | 0.037194829 | 0.098361186 | down |
| RP11-738E22.3 | -0.733186243 | 0.110654419 | 0.217274363 | down |
| RP11-5407.11 | -0.733118169 | 0.439964007 | 0.573831704 | down |
| ZNF554 | -0.732929359 | 1.99E-06 | 3.69E-05 | down |
| NUDT18 | -0.732910123 | 0.001248868 | 0.007235056 | down |
| RPL21P33 | -0.732793342 | 0.631509027 | 0.739344448 | down |
| RP11-665G4.1 | -0.732601267 | 0.327685348 | 0.466174095 | down |
| RP11-539G18.3 | -0.73247795 | 0.064477664 | 0.147201134 | down |
| NSMF | -0.732397231 | 2.32E-07 | 5.88E-06 | down |
| AC016712.2 | -0.732338722 | 0.197265865 | 0.325837354 | down |
| GPR37 | -0.732334092 | 0.198001829 | 0.326741008 | down |
| TTLL10-AS1 | -0.732187617 | 0.143201336 | 0.260337707 | down |
| NA | -0.732126517 | 0.415383369 | 0.551858195 | down |
| RNF24 | -0.732033866 | 0.002805927 | 0.013767966 | down |
| BCRP1 | -0.73197353 | 0.476222347 | | down |
| OR4Q3 | -0.731533358 | 0.802529872 | | down |
| PIGM | -0.731474226 | 5.76E-05 | 0.000600072 | down |
| Y_RNA | -0.731357385 | 0.630448343 | | down |
| NA | -0.731342756 | 0.573516065 | 0.69066525 | down |
| TRIM75P | -0.731193795 | 0.648281309 | 0.752708769 | down |
| GALNT14 | -0.731130066 | 0.0835043 | 0.17770245 | down |
| VSIG8 | -0.731045005 | 0.307040463 | 0.44535913 | down |
| DNPEP | -0.730598679 | 1.54E-05 | 0.000202416 | down |
| DSEL | -0.730546846 | 0.024457045 | 0.072136998 | down |
| RP11-77H9.6 | -0.730515088 | 0.308567945 | 0.447022532 | down |
| RP11-70C1.1 | -0.730512465 | 0.186372394 | 0.312674346 | down |
| NA | -0.730442172 | 0.083116631 | 0.177116973 | down |
| CASQ2 | -0.730344638 | 0.109395434 | 0.215661182 | down |
| CTC-436P18.3 | -0.730274123 | 0.258038944 | 0.393070123 | down |
| STIP1P3 | -0.730259357 | 0.441376222 | | down |
| PLAC4 | -0.730197672 | 0.090610641 | 0.188407924 | down |
| PTPRG-AS1 | -0.729590079 | 0.217389913 | 0.348743195 | down |
| RP11-420K14.3 | -0.729200271 | 0.572420828 | | down |
| AC007191.4 | -0.729119598 | 0.080565966 | 0.173080218 | down |
| DUX4L26 | -0.729022325 | 0.474859116 | 0.604860888 | down |
| ETV3L | -0.728589157 | 0.259622879 | 0.394822321 | down |
| CTB-3204.3 | -0.728417822 | 0.502237003 | | down |

| | | | | |
|---|---|---|---|---|
| C15orf59 | -0.72840104 | 0.022063318 | 0.066757831 | down |
| CLN3 | -0.72835405 | 0.024298322 | 0.071767477 | down |
| CTA-398F10.2 | -0.728306996 | 0.266541448 | 0.402228703 | down |
| FGF12 | -0.728270834 | 0.054281492 | 0.129790519 | down |
| ABHD12 | -0.728241596 | 0.000189814 | 0.001602351 | down |
| ALOX15 | -0.727949628 | 0.09920592 | 0.200943142 | down |
| RP11-1055B8.4 | -0.727711015 | 0.038272275 | 0.100599495 | down |
| RP11-384L8.1 | -0.727609716 | 0.029367562 | 0.082606406 | down |
| RP11-158H5.8 | -0.727451277 | 0.200918786 | 0.32995299 | down |
| ENO1-AS1 | -0.727393262 | 0.204607613 | 0.334233968 | down |
| EIF3K | -0.7273683 | 0.000590471 | 0.003982066 | down |
| LINC01204 | -0.727231939 | 0.45222327 | | down |
| CCDC24 | -0.727208119 | 2.49E-05 | 0.000299908 | down |
| DSCAML1 | -0.727137632 | 0.07463805 | 0.163752414 | down |
| RP11-933H2.4 | -0.726989492 | 0.046073107 | 0.115140611 | down |
| ADORA3 | -0.726984756 | 0.019526951 | 0.060912221 | down |
| LACTB2-AS1 | -0.726980171 | 0.059832892 | 0.139393565 | down |
| ELL3 | -0.726944069 | 0.280354587 | 0.416690077 | down |
| CCDC144CP | -0.726933473 | 0.307520418 | 0.445861569 | down |
| NA | -0.726919139 | 0.330548606 | 0.46888585 | down |
| RP5-1126H10.2 | -0.72684462 | 0.029515837 | 0.082876057 | down |
| AF131216.6 | -0.726796257 | 0.259600003 | 0.394807517 | down |
| POTEKP | -0.726465233 | 0.382161409 | 0.519947165 | down |
| RP11-234N17.1 | -0.726403453 | 0.176549072 | 0.301411542 | down |
| RPSAP58 | -0.726354073 | 0.01776753 | 0.056848526 | down |
| RPS2P35 | -0.726225847 | 0.174434457 | 0.29891304 | down |
| RP1-161P9.5 | -0.726184185 | 0.268089152 | 0.403830419 | down |
| CTD-2349P21.11 | -0.726021815 | 0.536196951 | | down |
| AC004862.6 | -0.725890122 | 0.710581775 | | down |
| PFKFB2 | -0.725706632 | 5.29E-05 | 0.000559743 | down |
| SLC2A13 | -0.725637823 | 0.00287325 | 0.014015304 | down |
| RGPD8 | -0.725484823 | 0.022895798 | 0.068520905 | down |
| TXNP6 | -0.725336027 | 0.387318922 | | down |
| RP11-709D24.6 | -0.725165399 | 0.498107342 | | down |
| C6 | -0.72491493 | 0.144389107 | 0.261894852 | down |
| Y_RNA | -0.724832372 | 0.585768618 | | down |
| RP11-320P7.2 | -0.724817605 | 0.307208719 | 0.445525702 | down |
| KLRC2 | -0.724701211 | 0.129847848 | 0.242976603 | down |
| CTD-2024I7.18 | -0.724610567 | 0.562608428 | | down |
| CBS | -0.724546604 | 0.052219785 | 0.126096384 | down |
| C2orf72 | -0.724470319 | 0.010033795 | 0.036767328 | down |
| SH3GLB2 | -0.724385107 | 5.09E-05 | 0.000542531 | down |
| RP11-713D19.1 | -0.724062575 | 0.569566664 | | down |
| RP11-713M15.2 | -0.724060052 | 0.234653299 | 0.367470613 | down |
| AP001092.4 | -0.7239429 | 0.557547331 | | down |
| DPPA5P2 | -0.723942839 | 0.64791851 | | down |
| KNOP1P2 | -0.723942838 | 0.649443983 | | down |
| RPL6P7 | -0.723942836 | 0.650894861 | | down |
| RP11-278C7.2 | -0.723942803 | 0.680657766 | | down |
| LCE5A | -0.723942764 | 0.70803452 | | down |
| AP004289.2 | -0.723942762 | 0.708931365 | | down |

| | | | | |
|---|---|---|---|---|
| RP1-313L4.3 | -0.72394276 | 0.710522594 | | down |
| NA | -0.72394276 | 0.710070678 | | down |
| PHBP8 | -0.723942757 | 0.711729489 | | down |
| NA | -0.723942709 | 0.736453761 | | down |
| RP11-543D10.2 | -0.723942699 | 0.740892303 | | down |
| AC004471.10 | -0.723942645 | 0.761034706 | | down |
| VN1R5 | -0.723942616 | 0.769943945 | | down |
| CYCTP | -0.723942514 | 0.79529638 | | down |
| RP11-96B2.1 | -0.723942511 | 0.795885492 | | down |
| OR2J3 | -0.723942467 | 0.804575445 | | down |
| OR8B12 | -0.723942467 | 0.804575445 | | down |
| GPR31 | -0.723810591 | 0.244064345 | 0.377953156 | down |
| MARK2 | -0.723580646 | 3.53E-06 | 5.94E-05 | down |
| RP1-167O22.1 | -0.723414557 | 0.192418657 | 0.320331696 | down |
| AC006538.1 | -0.723373812 | 0.074477401 | 0.163495473 | down |
| TMEM211 | -0.723351847 | 0.190212999 | 0.317627797 | down |
| OR5M14P | -0.723165888 | 0.70617459 | 0.797881222 | down |
| CCR12P | -0.723150207 | 0.163145079 | 0.285321075 | down |
| LNX1 | -0.723140313 | 0.028845569 | 0.081581124 | down |
| CTD-2525I3.6 | -0.723100193 | 0.561036691 | | down |
| LINC01018 | -0.722792068 | 0.251254455 | 0.386023101 | down |
| NA | -0.722767247 | 0.570515684 | | down |
| ZDHHC12 | -0.722619347 | 0.000254011 | 0.002037521 | down |
| OR7E37P | -0.722404721 | 0.658004182 | | down |
| CAMK2G | -0.722342523 | 1.35E-05 | 0.000181515 | down |
| AC022182.2 | -0.7223171 | 0.147799253 | 0.266375571 | down |
| GHRHR | -0.72225353 | 0.35442867 | | down |
| RP11-463O9.1 | -0.721936313 | 0.37497277 | 0.513235264 | down |
| AP000350.5 | -0.721785846 | 0.211650215 | 0.342295261 | down |
| C1orf132 | -0.721746339 | 0.006703451 | 0.027126591 | down |
| RIBC2 | -0.721715218 | 0.026564023 | 0.076717246 | down |
| OPA1-AS1 | -0.721628396 | 0.739391654 | | down |
| RP11-663P9.1 | -0.721613501 | 0.41835152 | | down |
| PLEKHA5 | -0.721547332 | 0.017829454 | 0.056998098 | down |
| NA | -0.721495775 | 0.142562316 | 0.259537188 | down |
| DLG2 | -0.721476929 | 0.06346281 | 0.145646617 | down |
| SLC25A5P6 | -0.72145739 | 0.684879324 | | down |
| RP11-231D20.2 | -0.721338648 | 0.290740571 | 0.427872139 | down |
| MYO6 | -0.721189398 | 0.000496357 | 0.003454379 | down |
| IBA57 | -0.721091008 | 2.32E-05 | 0.000282403 | down |
| C3orf49 | -0.720864735 | 0.223168049 | 0.355204818 | down |
| SMG1P5 | -0.720797331 | 0.022182694 | 0.067025563 | down |
| MYO10 | -0.720653563 | 0.000249891 | 0.002010636 | down |
| RP11-20G13.1 | -0.720544506 | 0.088055151 | 0.184608111 | down |
| ANKRD2 | -0.720416453 | 0.120220822 | 0.230467897 | down |
| CTC-412M14.6 | -0.720410793 | 0.490908612 | | down |
| VSTM2A | -0.720264406 | 0.232258058 | 0.36489887 | down |
| NA | -0.720252554 | 0.094845584 | 0.194693954 | down |
| RP5-887A10.1 | -0.720087901 | 0.044191089 | 0.111766737 | down |
| RP11-356I2.4 | -0.719995047 | 0.020489516 | 0.063234415 | down |
| NUPR1 | -0.719938349 | 0.008726393 | 0.033027935 | down |

| | | | | |
|---|---|---|---|---|
| RP11-120K24.4 | -0.719857625 | 0.439085706 | 0.573286296 | down |
| ARRDC4 | -0.719848439 | 0.003979591 | 0.018170644 | down |
| FAM213B | -0.719791204 | 7.92E-06 | 0.000116283 | down |
| GSS | -0.719442159 | 0.000192242 | 0.001617392 | down |
| DRC3 | -0.719201258 | 0.027719343 | 0.079201135 | down |
| CASC2 | -0.719167321 | 0.05512257 | 0.131299904 | down |
| RP11-274H2.3 | -0.719124895 | 0.43355171 | 0.56855642 | down |
| WDR13 | -0.718982013 | 0.000416278 | 0.003004661 | down |
| SGSM3 | -0.718871994 | 0.063910421 | 0.146250642 | down |
| COMMD4 | -0.71875919 | 0.000935487 | 0.005746424 | down |
| CLSPN | -0.718705207 | 0.01224318 | 0.042809385 | down |
| RP11-438L19.1 | -0.718676144 | 0.089714296 | 0.187145478 | down |
| HSD17B13 | -0.718648283 | 0.088762277 | 0.18568844 | down |
| SLC43A1 | -0.718617722 | 0.010968385 | 0.039384985 | down |
| RP5-994D16.3 | -0.718504614 | 0.313206632 | | down |
| SNX31 | -0.718482476 | 0.310736177 | 0.44920802 | down |
| CFTR | -0.718374491 | 0.049436058 | 0.121372222 | down |
| EPHA1-AS1 | -0.71821923 | 0.051761883 | 0.125355419 | down |
| RP11-212P7.2 | -0.718190092 | 6.85E-06 | 0.000103349 | down |
| RP4-760C5.3 | -0.718184809 | 0.670152147 | | down |
| NA | -0.717688553 | 0.023662529 | 0.070290392 | down |
| RP11-778J16.2 | -0.717484917 | 0.409069192 | | down |
| RP11-299J3.8 | -0.717460664 | 0.014376129 | 0.048356069 | down |
| METTL7A | -0.717290523 | 6.34E-05 | 0.000649495 | down |
| KLHDC8A | -0.717199644 | 0.047293758 | 0.117438935 | down |
| RP11-649A18.5 | -0.716999656 | 0.212990579 | 0.343815291 | down |
| RP11-734I18.1 | -0.716854087 | 0.579200166 | | down |
| SNHG22 | -0.716566417 | 0.101810821 | 0.204657971 | down |
| NMRAL1 | -0.716514465 | 0.000795582 | 0.005053376 | down |
| AC016292.1 | -0.716346428 | 0.551024163 | | down |
| NDFIP2 | -0.71627012 | 0.000741923 | 0.004782307 | down |
| FAM66B | -0.716089592 | 0.039974275 | 0.103900606 | down |
| LOXNC01-131B10. | -0.716023033 | 0.461509723 | | down |
| IER2 | -0.715947867 | 0.002381474 | 0.012092108 | down |
| S100A2 | -0.71591325 | 0.061302235 | 0.142067599 | down |
| NA | -0.715903051 | 0.487392562 | | down |
| AC110299.5 | -0.715899182 | 0.502218472 | | down |
| RP1-182O16.2 | -0.715871528 | 0.611839068 | | down |
| TBC1D32 | -0.71580987 | 0.038546712 | 0.101064439 | down |
| C19orf48 | -0.71557936 | 0.007317059 | 0.02892549 | down |
| RP1-40E16.9 | -0.715512657 | 0.457973248 | 0.58999821 | down |
| RP11-466F5.8 | -0.715154817 | 0.191586289 | 0.319336578 | down |
| AC020595.1 | -0.715110149 | 0.806911888 | | down |
| NOL8P1 | -0.715110149 | 0.806911888 | | down |
| AK4P2 | -0.715110149 | 0.806911888 | | down |
| RP4-800O15.1 | -0.715110149 | 0.806911888 | | down |
| CTC-523E23.6 | -0.715110149 | 0.806911888 | | down |
| KRTAP12-2 | -0.715110149 | 0.806911888 | | down |
| RP11-319E12.1 | -0.715108283 | 0.793815517 | | down |
| AC090044.2 | -0.715099466 | 0.742393651 | | down |
| RBMX2P5 | -0.715099427 | 0.742195439 | | down |

| | | | | |
|---|---|---|---|---|
| AC011286.1 | -0.715099363 | 0.741868585 | | down |
| RP11-806L2.5 | -0.715098662 | 0.73830312 | | down |
| RNA5SP38 | -0.715098622 | 0.738104668 | | down |
| RP11-474B12.1 | -0.715098515 | 0.737565198 | | down |
| AC006026.9 | -0.715086029 | 0.68213226 | | down |
| RP13-977J11.9 | -0.715085687 | 0.680777229 | | down |
| NA | -0.715085679 | 0.680744577 | | down |
| CTD-2376I20.1 | -0.715069509 | 0.623657167 | | down |
| RP11-2H3.7 | -0.715060927 | 0.597599544 | | down |
| RP11-541N10.3 | -0.71504831 | 0.563152488 | | down |
| PCDHA7 | -0.714989126 | 0.324102807 | 0.462515781 | down |
| RP11-219G17.4 | -0.71498223 | 0.110047096 | 0.21644877 | down |
| ECI2 | -0.714968393 | 7.18E-05 | 0.000715992 | down |
| CTA-373H7.7 | -0.714931849 | 0.515967774 | | down |
| RPS23P4 | -0.714920085 | 0.806943107 | | down |
| RP11-296E23.2 | -0.714920085 | 0.806943107 | | down |
| TMEM8A | -0.714888174 | 1.61E-05 | 0.000210761 | down |
| TAS2R4 | -0.714787915 | 0.121690881 | 0.232382943 | down |
| TEKT3 | -0.714721889 | 0.026226927 | 0.075962768 | down |
| GPD1L | -0.714625009 | 1.74E-05 | 0.000223203 | down |
| ESRP1 | -0.714563673 | 0.014905774 | 0.049703561 | down |
| PLIN2 | -0.714518742 | 0.00125224 | 0.00725006 | down |
| RP11-546B15.1 | -0.714427759 | 0.539362778 | | down |
| LCN1P1 | -0.714219556 | 0.298417821 | 0.435945979 | down |
| NA | -0.714133987 | 0.319015765 | 0.45782229 | down |
| RPS6KA2-IT1 | -0.714123095 | 0.48212511 | | down |
| RP11-242J7.1 | -0.714119932 | 0.363686975 | 0.502068511 | down |
| SCARNA17 | -0.714112922 | 0.737070289 | | down |
| RP11-397E7.1 | -0.714041394 | 0.404508094 | 0.541040859 | down |
| NBPF13P | -0.71395034 | 0.292753335 | 0.430096879 | down |
| CTD-2666L21.1 | -0.71382424 | 0.209191179 | 0.339396251 | down |
| LL0XNC01-16G2.1 | -0.713817126 | 0.715185114 | | down |
| LRRC58 | -0.713772616 | 0.010680705 | 0.038582553 | down |
| RP11-78I14.1 | -0.713749326 | 0.71153621 | | down |
| SLC35E3 | -0.713743305 | 0.000313455 | 0.002416589 | down |
| SMIM23 | -0.713664698 | 0.224752948 | 0.356903327 | down |
| CTD-3088G3.8 | -0.713444556 | 0.03544711 | 0.094892005 | down |
| PAPSS2 | -0.71341437 | 0.003948313 | 0.01805252 | down |
| ANGPTL5 | -0.713293531 | 0.415280081 | 0.551796681 | down |
| MTND5P28 | -0.713265486 | 0.428514143 | | down |
| SNORA3 | -0.713207346 | 0.600832155 | | down |
| RNU1-108P | -0.713145919 | 0.538457479 | 0.660536089 | down |
| POU5F1P5 | -0.712917876 | 0.661196155 | | down |
| RP11-403A21.1 | -0.712877336 | 0.113634085 | 0.221273086 | down |
| IFNLR1 | -0.712866 | 0.003778024 | 0.017411712 | down |
| RP1-159A19.4 | -0.712827033 | 0.055183025 | 0.131369878 | down |
| RP11-378A12.1 | -0.712607002 | 0.626450769 | 0.735486007 | down |
| NEO1 | -0.712583106 | 0.00128365 | 0.007376815 | down |
| NA | -0.712570129 | 0.380194352 | 0.518123883 | down |
| FGFR1OP | -0.712511293 | 8.39E-05 | 0.000817629 | down |
| CH17-385C13.2 | -0.712395872 | 0.002306797 | 0.011788641 | down |

| | | | | |
|---|---|---|---|---|
| RP11-180P8.1 | -0.712058194 | 0.356328607 | 0.494733429 | down |
| NA | -0.711899797 | 0.30533763 | 0.443531585 | down |
| ZNF492 | -0.711839128 | 0.199373888 | 0.328380522 | down |
| GPR61 | -0.71180958 | 0.061362164 | 0.142181965 | down |
| AC131056.3 | -0.711794876 | 0.141059742 | 0.257723114 | down |
| RP3-337018.9 | -0.711704921 | 0.587107711 | | down |
| PSMA6 | -0.711428558 | 0.041884141 | 0.10759873 | down |
| STX1B | -0.711198944 | 0.004763994 | 0.020913128 | down |
| AATBC | -0.711161196 | 0.104328406 | 0.208052097 | down |
| NCAN | -0.710751734 | 0.247183709 | 0.381537539 | down |
| FLVCR1-AS1 | -0.710626731 | 0.009836513 | 0.036167935 | down |
| PLCD3 | -0.710545694 | 0.010176738 | 0.037173241 | down |
| RP11-428G5.1 | -0.710537705 | 0.604464407 | | down |
| ATRIP | -0.710514248 | 0.007822041 | 0.030454036 | down |
| MYRF | -0.710512441 | 0.02728379 | 0.078269281 | down |
| NSFP1 | -0.71050694 | 0.447851303 | 0.58109456 | down |
| MAL2 | -0.710219072 | 0.019469989 | 0.06082484 | down |
| SAGE1 | -0.709986747 | 0.699034823 | | down |
| RP11-235E17.5 | -0.709967666 | 0.432457549 | 0.567468318 | down |
| NA | -0.709879006 | 0.266988148 | 0.402680318 | down |
| NA | -0.709438191 | 0.343368547 | | down |
| NDUFA5P11 | -0.709413377 | 0.37575336 | 0.514030323 | down |
| CTC-428G20.1 | -0.709382442 | 0.21008332 | 0.340475956 | down |
| PDLIM2 | -0.709321663 | 1.89E-06 | 3.52E-05 | down |
| ACADVL | -0.709259178 | 0.000127069 | 0.001153021 | down |
| COL23A1 | -0.708976704 | 0.012382714 | 0.043173526 | down |
| TEN1-CDK3 | -0.708969386 | 0.130607794 | 0.244165303 | down |
| RP11-358L22.3 | -0.708938926 | 0.029598163 | 0.083015281 | down |
| RPL31P52 | -0.708936804 | 0.225950617 | 0.357984514 | down |
| GAMT | -0.708874672 | 0.015544931 | 0.051350293 | down |
| NPAS1 | -0.708860702 | 0.065098787 | 0.148123899 | down |
| AF127577.12 | -0.708826278 | 0.425776513 | | down |
| POLR2J3 | -0.708805832 | 0.002767343 | 0.013614825 | down |
| DPY19L1P2 | -0.708657427 | 0.167439684 | 0.290565347 | down |
| AHCYL2 | -0.708564946 | 0.008390327 | 0.032054805 | down |
| ANKRD34B | -0.708540658 | 0.301912932 | 0.439726204 | down |
| RP11-172F4.2 | -0.708514726 | 0.328388956 | 0.466880456 | down |
| GTF3C2-AS1 | -0.708402716 | 0.302170644 | 0.440032148 | down |
| EIF2S3L | -0.708380107 | 0.174530798 | 0.298951157 | down |
| RP11-534L20.4 | -0.708340592 | 0.368304631 | | down |
| WBP1LP6 | -0.708262632 | 0.808725193 | | down |
| HNRNPCL4 | -0.708262632 | 0.808725193 | | down |
| RN7SL122P | -0.708262632 | 0.808725193 | | down |
| RP1-144F13.4 | -0.708262632 | 0.808725193 | | down |
| RP11-335E6.2 | -0.708262632 | 0.808725193 | | down |
| NA | -0.708262632 | 0.808725193 | | down |
| RN7SL488P | -0.708262632 | 0.808725193 | | down |
| RNU2-15P | -0.708262632 | 0.808725193 | | down |
| RP11-74K19.1 | -0.708262632 | 0.808725193 | | down |
| HNRNPA1P64 | -0.708262632 | 0.808725193 | | down |
| RNU6-210P | -0.708262632 | 0.808725193 | | down |

| | | | |
|---|---|---|---|
| RP5-837O21.8 | -0.708262632 | 0.808725193 | down |
| GAPDHP29 | -0.708262632 | 0.808725193 | down |
| RP5-964H19.4 | -0.708262632 | 0.808725193 | down |
| HAO2-IT1 | -0.708262632 | 0.808725193 | down |
| RP4-704D21.2 | -0.708262632 | 0.808725193 | down |
| RP11-433J22.3 | -0.708262632 | 0.808725193 | down |
| RN7SL480P | -0.708262632 | 0.808725193 | down |
| RP11-98D18.15 | -0.708262632 | 0.808725193 | down |
| RP1-43O17.1 | -0.708262632 | 0.808725193 | down |
| OR6P1 | -0.708262632 | 0.808725193 | down |
| RNA5SP60 | -0.708262632 | 0.808725193 | down |
| OR10J8P | -0.708262632 | 0.808725193 | down |
| OR10J9P | -0.708262632 | 0.808725193 | down |
| OR10J6P | -0.708262632 | 0.808725193 | down |
| MIR1255B2 | -0.708262632 | 0.808725193 | down |
| RP1-117P20.3 | -0.708262632 | 0.808725193 | down |
| RP11-548N1.1 | -0.708262632 | 0.808725193 | down |
| Y_RNA | -0.708262632 | 0.808725193 | down |
| RP5-925F19.1 | -0.708262632 | 0.808725193 | down |
| RN7SKP126 | -0.708262632 | 0.808725193 | down |
| RP4-608O15.4 | -0.708262632 | 0.808725193 | down |
| CDKN2AIPNLP1 | -0.708262632 | 0.808725193 | down |
| RNU6-180P | -0.708262632 | 0.808725193 | down |
| RNU4-77P | -0.708262632 | 0.808725193 | down |
| MTND5P19 | -0.708262632 | 0.808725193 | down |
| DNAJC19P8 | -0.708262632 | 0.808725193 | down |
| OR6R1P | -0.708262632 | 0.808725193 | down |
| OR2T4 | -0.708262632 | 0.808725193 | down |
| NPM1P48 | -0.708262632 | 0.808725193 | down |
| AC073143.1 | -0.708262632 | 0.808725193 | down |
| Metazoa_SRP | -0.708262632 | 0.808725193 | down |
| AC010745.3 | -0.708262632 | 0.808725193 | down |
| NUTF2P8 | -0.708262632 | 0.808725193 | down |
| SIX3-AS1 | -0.708262632 | 0.808725193 | down |
| RNU6-634P | -0.708262632 | 0.808725193 | down |
| PSAT1P2 | -0.708262632 | 0.808725193 | down |
| AC009474.2 | -0.708262632 | 0.808725193 | down |
| RP11-85D18.1 | -0.708262632 | 0.808725193 | down |
| NA | -0.708262632 | 0.808725193 | down |
| AC073091.2 | -0.708262632 | 0.808725193 | down |
| RPL37P10 | -0.708262632 | 0.808725193 | down |
| AC078974.2 | -0.708262632 | 0.808725193 | down |
| TCF7L1-IT1 | -0.708262632 | 0.808725193 | down |
| IGKV2-36 | -0.708262632 | 0.808725193 | down |
| IGKV1D-32 | -0.708262632 | 0.808725193 | down |
| AC103563.2 | -0.708262632 | 0.808725193 | down |
| AC009238.8 | -0.708262632 | 0.808725193 | down |
| AC009237.6 | -0.708262632 | 0.808725193 | down |
| RN7SL210P | -0.708262632 | 0.808725193 | down |
| NANOGNBP1 | -0.708262632 | 0.808725193 | down |
| AHCYP3 | -0.708262632 | 0.808725193 | down |

| | | | |
|---|---|---|---|
| AC009963.3 | -0.708262632 | 0.808725193 | down |
| AC093166.1 | -0.708262632 | 0.808725193 | down |
| NA | -0.708262632 | 0.808725193 | down |
| RNU6-1147P | -0.708262632 | 0.808725193 | down |
| RNA5SP103 | -0.708262632 | 0.808725193 | down |
| AC068483.1 | -0.708262632 | 0.808725193 | down |
| AC134915.1 | -0.708262632 | 0.808725193 | down |
| MTCO2P7 | -0.708262632 | 0.808725193 | down |
| NA | -0.708262632 | 0.808725193 | down |
| RNA5-8SP5 | -0.708262632 | 0.808725193 | down |
| YBX1P7 | -0.708262632 | 0.808725193 | down |
| RNU6-692P | -0.708262632 | 0.808725193 | down |
| Y_RNA | -0.708262632 | 0.808725193 | down |
| RP11-311B14.1 | -0.708262632 | 0.808725193 | down |
| RN7SL813P | -0.708262632 | 0.808725193 | down |
| AC010894.4 | -0.708262632 | 0.808725193 | down |
| MIR561 | -0.708262632 | 0.808725193 | down |
| AC068135.1 | -0.708262632 | 0.808725193 | down |
| NA | -0.708262632 | 0.808725193 | down |
| RN7SKP200 | -0.708262632 | 0.808725193 | down |
| CRYGEP | -0.708262632 | 0.808725193 | down |
| MTCO1P46 | -0.708262632 | 0.808725193 | down |
| ENSAP3 | -0.708262632 | 0.808725193 | down |
| AC074019.1 | -0.708262632 | 0.808725193 | down |
| NEU2 | -0.708262632 | 0.808725193 | down |
| HSPE1P9 | -0.708262632 | 0.808725193 | down |
| SRGAP3-AS3 | -0.708262632 | 0.808725193 | down |
| AC090960.1 | -0.708262632 | 0.808725193 | down |
| RPL31P20 | -0.708262632 | 0.808725193 | down |
| PRSS45 | -0.708262632 | 0.808725193 | down |
| BSN-AS1 | -0.708262632 | 0.808725193 | down |
| CACNA2D3-AS1 | -0.708262632 | 0.808725193 | down |
| RN7SL271P | -0.708262632 | 0.808725193 | down |
| Metazoa_SRP | -0.708262632 | 0.808725193 | down |
| RP11-331K15.1 | -0.708262632 | 0.808725193 | down |
| Y_RNA | -0.708262632 | 0.808725193 | down |
| OR5H8 | -0.708262632 | 0.808725193 | down |
| MTND4P16 | -0.708262632 | 0.808725193 | down |
| U3 | -0.708262632 | 0.808725193 | down |
| MIR8076 | -0.708262632 | 0.808725193 | down |
| NA | -0.708262632 | 0.808725193 | down |
| MARK2P17 | -0.708262632 | 0.808725193 | down |
| HMGB3P13 | -0.708262632 | 0.808725193 | down |
| RN7SKP124 | -0.708262632 | 0.808725193 | down |
| Y_RNA | -0.708262632 | 0.808725193 | down |
| RNA5SP145 | -0.708262632 | 0.808725193 | down |
| RP11-656A15.1 | -0.708262632 | 0.808725193 | down |
| PABPC1P10 | -0.708262632 | 0.808725193 | down |
| RP11-113A11.1 | -0.708262632 | 0.808725193 | down |
| RNU4-91P | -0.708262632 | 0.808725193 | down |
| RP11-809F4.4 | -0.708262632 | 0.808725193 | down |

| | | | |
|---|---|---|---|
| RN7SL703P | -0.708262632 | 0.808725193 | down |
| RNU1-20P | -0.708262632 | 0.808725193 | down |
| RNU6-860P | -0.708262632 | 0.808725193 | down |
| AC006499.6 | -0.708262632 | 0.808725193 | down |
| SNORA63 | -0.708262632 | 0.808725193 | down |
| MIR4275 | -0.708262632 | 0.808725193 | down |
| EEF1A1P21 | -0.708262632 | 0.808725193 | down |
| RP11-174E22.2 | -0.708262632 | 0.808725193 | down |
| MTCYBP43 | -0.708262632 | 0.808725193 | down |
| RNU6-887P | -0.708262632 | 0.808725193 | down |
| RNU6-1195P | -0.708262632 | 0.808725193 | down |
| SNX18P24 | -0.708262632 | 0.808725193 | down |
| HMGN1P11 | -0.708262632 | 0.808725193 | down |
| NA | -0.708262632 | 0.808725193 | down |
| RP11-642E20.3 | -0.708262632 | 0.808725193 | down |
| SMR3B | -0.708262632 | 0.808725193 | down |
| RP11-545L5.1 | -0.708262632 | 0.808725193 | down |
| RP11-630D6.1 | -0.708262632 | 0.808725193 | down |
| CTB-179I1.3 | -0.708262632 | 0.808725193 | down |
| RPSAP39 | -0.708262632 | 0.808725193 | down |
| Y_RNA | -0.708262632 | 0.808725193 | down |
| RNU6-1298P | -0.708262632 | 0.808725193 | down |
| RNU6-462P | -0.708262632 | 0.808725193 | down |
| Y_RNA | -0.708262632 | 0.808725193 | down |
| SNORA11 | -0.708262632 | 0.808725193 | down |
| RP11-780O17.1 | -0.708262632 | 0.808725193 | down |
| RP11-208N20.1 | -0.708262632 | 0.808725193 | down |
| MIR4799 | -0.708262632 | 0.808725193 | down |
| RN7SKP35 | -0.708262632 | 0.808725193 | down |
| MTND6P17 | -0.708262632 | 0.808725193 | down |
| MTHFD2P4 | -0.708262632 | 0.808725193 | down |
| RPL35AP12 | -0.708262632 | 0.808725193 | down |
| RP11-18D7.2 | -0.708262632 | 0.808725193 | down |
| SNORA31 | -0.708262632 | 0.808725193 | down |
| RP11-445O3.1 | -0.708262632 | 0.808725193 | down |
| CTD-2161F6.2 | -0.708262632 | 0.808725193 | down |
| TAS2R1 | -0.708262632 | 0.808725193 | down |
| RPS23P5 | -0.708262632 | 0.808725193 | down |
| SEPHS2P1 | -0.708262632 | 0.808725193 | down |
| RN7SL58P | -0.708262632 | 0.808725193 | down |
| NA | -0.708262632 | 0.808725193 | down |
| CTD-2130F23.1 | -0.708262632 | 0.808725193 | down |
| KATNBL1P4 | -0.708262632 | 0.808725193 | down |
| RNA5SP183 | -0.708262632 | 0.808725193 | down |
| RNU6ATAC2P | -0.708262632 | 0.808725193 | down |
| RNU6-913P | -0.708262632 | 0.808725193 | down |
| MAST4-IT1 | -0.708262632 | 0.808725193 | down |
| RP11-1315F8.2 | -0.708262632 | 0.808725193 | down |
| RP11-229C3.4 | -0.708262632 | 0.808725193 | down |
| LINC01455 | -0.708262632 | 0.808725193 | down |
| CTD-2218K11.3 | -0.708262632 | 0.808725193 | down |

| | | | |
|---|---|---|---|
| RP11-456A14.1 | -0.708262632 | 0.808725193 | down |
| CTD-2331D11.3 | -0.708262632 | 0.808725193 | down |
| CTD-2331D11.4 | -0.708262632 | 0.808725193 | down |
| RNU6-1119P | -0.708262632 | 0.808725193 | down |
| RP11-6N13.4 | -0.708262632 | 0.808725193 | down |
| RP11-231G15.2 | -0.708262632 | 0.808725193 | down |
| CTD-2089O24.2 | -0.708262632 | 0.808725193 | down |
| CTC-505O3.1 | -0.708262632 | 0.808725193 | down |
| CTD-2544H17.1 | -0.708262632 | 0.808725193 | down |
| ARGFXP1 | -0.708262632 | 0.808725193 | down |
| CTB-1I21.1 | -0.708262632 | 0.808725193 | down |
| CTB-1I21.2 | -0.708262632 | 0.808725193 | down |
| HMGN1P16 | -0.708262632 | 0.808725193 | down |
| RNU6-588P | -0.708262632 | 0.808725193 | down |
| RP11-443C10.3 | -0.708262632 | 0.808725193 | down |
| RP1-290I10.5 | -0.708262632 | 0.808725193 | down |
| RNU6-1060P | -0.708262632 | 0.808725193 | down |
| NA | -0.708262632 | 0.808725193 | down |
| DDX6P1 | -0.708262632 | 0.808725193 | down |
| OR12D3 | -0.708262632 | 0.808725193 | down |
| RP1-149M18.3 | -0.708262632 | 0.808725193 | down |
| RP11-256G5.1 | -0.708262632 | 0.808725193 | down |
| RP3-449H6.2 | -0.708262632 | 0.808725193 | down |
| NUDT19P4 | -0.708262632 | 0.808725193 | down |
| MIR586 | -0.708262632 | 0.808725193 | down |
| RP11-812I20.2 | -0.708262632 | 0.808725193 | down |
| RP11-719J20.1 | -0.708262632 | 0.808725193 | down |
| GSTA12P | -0.708262632 | 0.808725193 | down |
| GAPDHP15 | -0.708262632 | 0.808725193 | down |
| NA | -0.708262632 | 0.808725193 | down |
| HNRNPDP2 | -0.708262632 | 0.808725193 | down |
| RP11-554D15.4 | -0.708262632 | 0.808725193 | down |
| RP11-415D17.1 | -0.708262632 | 0.808725193 | down |
| RP3-455E7.1 | -0.708262632 | 0.808725193 | down |
| NDUFA5P9 | -0.708262632 | 0.808725193 | down |
| RP3-382I10.2 | -0.708262632 | 0.808725193 | down |
| ST13P16 | -0.708262632 | 0.808725193 | down |
| RP4-570O12.2 | -0.708262632 | 0.808725193 | down |
| MTCO1P56 | -0.708262632 | 0.808725193 | down |
| RP11-524K14.1 | -0.708262632 | 0.808725193 | down |
| CYCSP17 | -0.708262632 | 0.808725193 | down |
| RNU6-1106P | -0.708262632 | 0.808725193 | down |
| SNORA40 | -0.708262632 | 0.808725193 | down |
| RP11-506B6.7 | -0.708262632 | 0.808725193 | down |
| TAAR7P | -0.708262632 | 0.808725193 | down |
| RPL23AP46 | -0.708262632 | 0.808725193 | down |
| FUNDC2P3 | -0.708262632 | 0.808725193 | down |
| RP11-361F15.5 | -0.708262632 | 0.808725193 | down |
| FABP12P1 | -0.708262632 | 0.808725193 | down |
| MTCO2P31 | -0.708262632 | 0.808725193 | down |
| HMGB3P19 | -0.708262632 | 0.808725193 | down |

| | | | |
|---|---|---|---|
| RNU6-1103P | -0.708262632 | 0.808725193 | down |
| RNU6-575P | -0.708262632 | 0.808725193 | down |
| POM121L12 | -0.708262632 | 0.808725193 | down |
| CICP12 | -0.708262632 | 0.808725193 | down |
| RP11-760D2.11 | -0.708262632 | 0.808725193 | down |
| NA | -0.708262632 | 0.808725193 | down |
| RP11-548K12.8 | -0.708262632 | 0.808725193 | down |
| MTND3P2 | -0.708262632 | 0.808725193 | down |
| VN1R38P | -0.708262632 | 0.808725193 | down |
| AC006355.3 | -0.708262632 | 0.808725193 | down |
| RP5-1093G23.1 | -0.708262632 | 0.808725193 | down |
| RP5-1136A10.1 | -0.708262632 | 0.808725193 | down |
| AC002127.2 | -0.708262632 | 0.808725193 | down |
| RN7SL7P | -0.708262632 | 0.808725193 | down |
| GS1-345D13.1 | -0.708262632 | 0.808725193 | down |
| RP4-604G5.3 | -0.708262632 | 0.808725193 | down |
| AC073626.2 | -0.708262632 | 0.808725193 | down |
| RP11-458K10.1 | -0.708262632 | 0.808725193 | down |
| NA | -0.708262632 | 0.808725193 | down |
| AC000111.3 | -0.708262632 | 0.808725193 | down |
| TAS2R16 | -0.708262632 | 0.808725193 | down |
| RP11-61D3.1 | -0.708262632 | 0.808725193 | down |
| AC008154.4 | -0.708262632 | 0.808725193 | down |
| MYL6P4 | -0.708262632 | 0.808725193 | down |
| RNU6-1184P | -0.708262632 | 0.808725193 | down |
| RP11-745K9.2 | -0.708262632 | 0.808725193 | down |
| DEFA10P | -0.708262632 | 0.808725193 | down |
| RP11-52B19.1 | -0.708262632 | 0.808725193 | down |
| DEFB135 | -0.708262632 | 0.808725193 | down |
| ZNF705CP | -0.708262632 | 0.808725193 | down |
| RPS3AP35 | -0.708262632 | 0.808725193 | down |
| RP11-369E15.4 | -0.708262632 | 0.808725193 | down |
| CTD-3107M8.1 | -0.708262632 | 0.808725193 | down |
| RNA5SP264 | -0.708262632 | 0.808725193 | down |
| AC090453.1 | -0.708262632 | 0.808725193 | down |
| RP11-359E19.1 | -0.708262632 | 0.808725193 | down |
| RP11-726G23.12 | -0.708262632 | 0.808725193 | down |
| RP11-359P18.5 | -0.708262632 | 0.808725193 | down |
| CTD-2210A23.1 | -0.708262632 | 0.808725193 | down |
| RFPL4AP7 | -0.708262632 | 0.808725193 | down |
| RP11-26M5.2 | -0.708262632 | 0.808725193 | down |
| NARSP2 | -0.708262632 | 0.808725193 | down |
| RNU7-102P | -0.708262632 | 0.808725193 | down |
| LINC01109 | -0.708262632 | 0.808725193 | down |
| MIR5708 | -0.708262632 | 0.808725193 | down |
| RNU6-1172P | -0.708262632 | 0.808725193 | down |
| KB-1930G5.3 | -0.708262632 | 0.808725193 | down |
| RP11-85M22.1 | -0.708262632 | 0.808725193 | down |
| RFPL4AP5 | -0.708262632 | 0.808725193 | down |
| MIR1208 | -0.708262632 | 0.808725193 | down |
| CTD-2534J5.1 | -0.708262632 | 0.808725193 | down |

| | | | |
|---|---|---|---|
| Metazoa_SRP | -0.708262632 | 0.808725193 | down |
| RP11-341G2.3 | -0.708262632 | 0.808725193 | down |
| RP11-284P20.3 | -0.708262632 | 0.808725193 | down |
| NA | -0.708262632 | 0.808725193 | down |
| MIR873 | -0.708262632 | 0.808725193 | down |
| MIR4476 | -0.708262632 | 0.808725193 | down |
| ARMC8P1 | -0.708262632 | 0.808725193 | down |
| RP13-198D9.4 | -0.708262632 | 0.808725193 | down |
| RP11-160N1.11 | -0.708262632 | 0.808725193 | down |
| RBPJP7 | -0.708262632 | 0.808725193 | down |
| FKBP4P7 | -0.708262632 | 0.808725193 | down |
| RP11-470F18.1 | -0.708262632 | 0.808725193 | down |
| RP11-403N16.2 | -0.708262632 | 0.808725193 | down |
| RP11-333B6.1 | -0.708262632 | 0.808725193 | down |
| MIR4290 | -0.708262632 | 0.808725193 | down |
| MIR2278 | -0.708262632 | 0.808725193 | down |
| NA | -0.708262632 | 0.808725193 | down |
| TUBB4BP6 | -0.708262632 | 0.808725193 | down |
| RP11-555H7.2 | -0.708262632 | 0.808725193 | down |
| RP11-414H17.2 | -0.708262632 | 0.808725193 | down |
| RN7SKP220 | -0.708262632 | 0.808725193 | down |
| RP11-241I20.5 | -0.708262632 | 0.808725193 | down |
| MTND4LP11 | -0.708262632 | 0.808725193 | down |
| RP11-550A9.1 | -0.708262632 | 0.808725193 | down |
| RN7SKP196 | -0.708262632 | 0.808725193 | down |
| RP11-367H5.8 | -0.708262632 | 0.808725193 | down |
| HMGN2P34 | -0.708262632 | 0.808725193 | down |
| RP11-369J21.11 | -0.708262632 | 0.808725193 | down |
| FARSBP1 | -0.708262632 | 0.808725193 | down |
| RNU6-441P | -0.708262632 | 0.808725193 | down |
| NA | -0.708262632 | 0.808725193 | down |
| RP11-475D12.1 | -0.708262632 | 0.808725193 | down |
| Y_RNA | -0.708262632 | 0.808725193 | down |
| CYP2C59P | -0.708262632 | 0.808725193 | down |
| RP11-375N9.2 | -0.708262632 | 0.808725193 | down |
| Hammerhead_HH10 | -0.708262632 | 0.808725193 | down |
| NA | -0.708262632 | 0.808725193 | down |
| NA | -0.708262632 | 0.808725193 | down |
| OR51C4P | -0.708262632 | 0.808725193 | down |
| Metazoa_SRP | -0.708262632 | 0.808725193 | down |
| PTH | -0.708262632 | 0.808725193 | down |
| MIR8054 | -0.708262632 | 0.808725193 | down |
| RNU6-783P | -0.708262632 | 0.808725193 | down |
| RP11-747E23.1 | -0.708262632 | 0.808725193 | down |
| OR4B1 | -0.708262632 | 0.808725193 | down |
| RP11-107P7.2 | -0.708262632 | 0.808725193 | down |
| OR4C12 | -0.708262632 | 0.808725193 | down |
| OR4C49P | -0.708262632 | 0.808725193 | down |
| RP11-347H15.4 | -0.708262632 | 0.808725193 | down |
| TRIM48 | -0.708262632 | 0.808725193 | down |
| OR5J7P | -0.708262632 | 0.808725193 | down |

| | | | |
|---|---|---|---|
| OR8K2P | -0.708262632 | 0.808725193 | down |
| OR5AK1P | -0.708262632 | 0.808725193 | down |
| MIR130A | -0.708262632 | 0.808725193 | down |
| OR9L1P | -0.708262632 | 0.808725193 | down |
| OR5AN2P | -0.708262632 | 0.808725193 | down |
| RP11-959F10.5 | -0.708262632 | 0.808725193 | down |
| NA | -0.708262632 | 0.808725193 | down |
| CMB9-52H4.1 | -0.708262632 | 0.808725193 | down |
| RN7SKP297 | -0.708262632 | 0.808725193 | down |
| RP11-529A4.9 | -0.708262632 | 0.808725193 | down |
| RP11-178H8.3 | -0.708262632 | 0.808725193 | down |
| RP11-684B20.1 | -0.708262632 | 0.808725193 | down |
| MTCO3P15 | -0.708262632 | 0.808725193 | down |
| MIR4693 | -0.708262632 | 0.808725193 | down |
| RP11-94P11.3 | -0.708262632 | 0.808725193 | down |
| RNU6-44P | -0.708262632 | 0.808725193 | down |
| RPL12P46 | -0.708262632 | 0.808725193 | down |
| snoU13 | -0.708262632 | 0.808725193 | down |
| RP11-775A1.2 | -0.708262632 | 0.808725193 | down |
| OR8F1P | -0.708262632 | 0.808725193 | down |
| OR8B10P | -0.708262632 | 0.808725193 | down |
| RP11-535N6.2 | -0.708262632 | 0.808725193 | down |
| RP11-986G18.3 | -0.708262632 | 0.808725193 | down |
| RP11-1038A11.2 | -0.708262632 | 0.808725193 | down |
| RP11-59N23.1 | -0.708262632 | 0.808725193 | down |
| RN7SL38P | -0.708262632 | 0.808725193 | down |
| RP11-115F18.1 | -0.708262632 | 0.808725193 | down |
| RP11-547C5.2 | -0.708262632 | 0.808725193 | down |
| Y_RNA | -0.708262632 | 0.808725193 | down |
| RP11-498M15.1 | -0.708262632 | 0.808725193 | down |
| RP11-314D7.1 | -0.708262632 | 0.808725193 | down |
| MIR617 | -0.708262632 | 0.808725193 | down |
| RP11-384P14.1 | -0.708262632 | 0.808725193 | down |
| RP11-248E9.5 | -0.708262632 | 0.808725193 | down |
| Y_RNA | -0.708262632 | 0.808725193 | down |
| RN7SL630P | -0.708262632 | 0.808725193 | down |
| RNA5SP366 | -0.708262632 | 0.808725193 | down |
| RPL11P5 | -0.708262632 | 0.808725193 | down |
| RP11-200K12.2 | -0.708262632 | 0.808725193 | down |
| LINC00388 | -0.708262632 | 0.808725193 | down |
| RNU6-76P | -0.708262632 | 0.808725193 | down |
| RNA5SP24 | -0.708262632 | 0.808725193 | down |
| IPPKP1 | -0.708262632 | 0.808725193 | down |
| LINC00362 | -0.708262632 | 0.808725193 | down |
| LINC00352 | -0.708262632 | 0.808725193 | down |
| NPM1P4 | -0.708262632 | 0.808725193 | down |
| LINC00572 | -0.708262632 | 0.808725193 | down |
| SNORA25 | -0.708262632 | 0.808725193 | down |
| TCEAL4P1 | -0.708262632 | 0.808725193 | down |
| RPS12P24 | -0.708262632 | 0.808725193 | down |
| RP11-24H2.3 | -0.708262632 | 0.808725193 | down |

| | | | |
|---|---|---|---|
| RP11-384G23.1 | -0.708262632 | 0.808725193 | down |
| LINC01442 | -0.708262632 | 0.808725193 | down |
| RAC1P8 | -0.708262632 | 0.808725193 | down |
| RP11-429G17.2 | -0.708262632 | 0.808725193 | down |
| LINC00395 | -0.708262632 | 0.808725193 | down |
| PCDH9-AS3 | -0.708262632 | 0.808725193 | down |
| RP11-411G9.2 | -0.708262632 | 0.808725193 | down |
| PSMC1P13 | -0.708262632 | 0.808725193 | down |
| SCEL-AS1 | -0.708262632 | 0.808725193 | down |
| UBE2D3P4 | -0.708262632 | 0.808725193 | down |
| NA | -0.708262632 | 0.808725193 | down |
| LINC00430 | -0.708262632 | 0.808725193 | down |
| RP11-545P6.2 | -0.708262632 | 0.808725193 | down |
| PPIAP23 | -0.708262632 | 0.808725193 | down |
| FABP5P4 | -0.708262632 | 0.808725193 | down |
| MIR3170 | -0.708262632 | 0.808725193 | down |
| RP11-536C10.3 | -0.708262632 | 0.808725193 | down |
| TRAV7 | -0.708262632 | 0.808725193 | down |
| RP11-388G3.1 | -0.708262632 | 0.808725193 | down |
| RP11-116N8.2 | -0.708262632 | 0.808725193 | down |
| CTD-2298J14.1 | -0.708262632 | 0.808725193 | down |
| RP11-794A8.1 | -0.708262632 | 0.808725193 | down |
| MIR625 | -0.708262632 | 0.808725193 | down |
| NA | -0.708262632 | 0.808725193 | down |
| MTND4P33 | -0.708262632 | 0.808725193 | down |
| MIR299 | -0.708262632 | 0.808725193 | down |
| IGHV3-63 | -0.708262632 | 0.808725193 | down |
| RN7SL759P | -0.708262632 | 0.808725193 | down |
| OR11J5P | -0.708262632 | 0.808725193 | down |
| SNORD115-28 | -0.708262632 | 0.808725193 | down |
| RP11-844G16.1 | -0.708262632 | 0.808725193 | down |
| SNORA25 | -0.708262632 | 0.808725193 | down |
| RNU6-339P | -0.708262632 | 0.808725193 | down |
| Z84721.4 | -0.708262632 | 0.808725193 | down |
| RP11-468I15.1 | -0.708262632 | 0.808725193 | down |
| RNU7-99P | -0.708262632 | 0.808725193 | down |
| RP11-279O17.3 | -0.708262632 | 0.808725193 | down |
| RP11-568A19.1 | -0.708262632 | 0.808725193 | down |
| MTND3P13 | -0.708262632 | 0.808725193 | down |
| AC009134.1 | -0.708262632 | 0.808725193 | down |
| SNORA42 | -0.708262632 | 0.808725193 | down |
| RP11-2C24.4 | -0.708262632 | 0.808725193 | down |
| VN1R65P | -0.708262632 | 0.808725193 | down |
| CTD-2358C21.2 | -0.708262632 | 0.808725193 | down |
| CTD-2595P9.2 | -0.708262632 | 0.808725193 | down |
| RP11-106J23.1 | -0.708262632 | 0.808725193 | down |
| MIR4719 | -0.708262632 | 0.808725193 | down |
| CTD-2300H10.2 | -0.708262632 | 0.808725193 | down |
| RP11-543N12.1 | -0.708262632 | 0.808725193 | down |
| RNU6-1264P | -0.708262632 | 0.808725193 | down |
| RP11-488L1.1 | -0.708262632 | 0.808725193 | down |

| | | | |
|---|---|---|---|
| CDRT15L2 | -0.708262632 | 0.808725193 | down |
| PDLIM1P3 | -0.708262632 | 0.808725193 | down |
| RPS16P8 | -0.708262632 | 0.808725193 | down |
| CTC-542B22.1 | -0.708262632 | 0.808725193 | down |
| Y_RNA | -0.708262632 | 0.808725193 | down |
| KRTAP4-5 | -0.708262632 | 0.808725193 | down |
| RN7SL507P | -0.708262632 | 0.808725193 | down |
| SMCO4P1 | -0.708262632 | 0.808725193 | down |
| RNU6-518P | -0.708262632 | 0.808725193 | down |
| RPSAP66 | -0.708262632 | 0.808725193 | down |
| AC037445.1 | -0.708262632 | 0.808725193 | down |
| NA | -0.708262632 | 0.808725193 | down |
| CTD-2526A2.6 | -0.708262632 | 0.808725193 | down |
| CTD-2124B20.2 | -0.708262632 | 0.808725193 | down |
| RP11-703I16.2 | -0.708262632 | 0.808725193 | down |
| FGF7P1 | -0.708262632 | 0.808725193 | down |
| RP11-296E23.1 | -0.708262632 | 0.808725193 | down |
| RNU6-435P | -0.708262632 | 0.808725193 | down |
| RPS24P18 | -0.708262632 | 0.808725193 | down |
| NPM1P2 | -0.708262632 | 0.808725193 | down |
| RP11-675P14.2 | -0.708262632 | 0.808725193 | down |
| RP11-25I11.1 | -0.708262632 | 0.808725193 | down |
| MIR187 | -0.708262632 | 0.808725193 | down |
| RNA5SP454 | -0.708262632 | 0.808725193 | down |
| RN7SL705P | -0.708262632 | 0.808725193 | down |
| LARP7P3 | -0.708262632 | 0.808725193 | down |
| RNA5SP461 | -0.708262632 | 0.808725193 | down |
| AC092192.1 | -0.708262632 | 0.808725193 | down |
| ZNRF4 | -0.708262632 | 0.808725193 | down |
| BNIP3P36 | -0.708262632 | 0.808725193 | down |
| VN1R91P | -0.708262632 | 0.808725193 | down |
| CTB-60E11.12 | -0.708262632 | 0.808725193 | down |
| RNU6-841P | -0.708262632 | 0.808725193 | down |
| MIR512-2 | -0.708262632 | 0.808725193 | down |
| MIR520B | -0.708262632 | 0.808725193 | down |
| MIR520G | -0.708262632 | 0.808725193 | down |
| MIR526A2 | -0.708262632 | 0.808725193 | down |
| MIR516A1 | -0.708262632 | 0.808725193 | down |
| DEFB129 | -0.708262632 | 0.808725193 | down |
| MIR8062 | -0.708262632 | 0.808725193 | down |
| RP11-102J14.1 | -0.708262632 | 0.808725193 | down |
| RP5-1097P24.1 | -0.708262632 | 0.808725193 | down |
| Y_RNA | -0.708262632 | 0.808725193 | down |
| FRG2EP | -0.708262632 | 0.808725193 | down |
| LINC00489 | -0.708262632 | 0.808725193 | down |
| RN7SL666P | -0.708262632 | 0.808725193 | down |
| RP4-678D15.1 | -0.708262632 | 0.808725193 | down |
| NA | -0.708262632 | 0.808725193 | down |
| RNA5SP487 | -0.708262632 | 0.808725193 | down |
| RP5-827L5.1 | -0.708262632 | 0.808725193 | down |
| U1 | -0.708262632 | 0.808725193 | down |

| | | | |
|---|---|---|---|
| CH507-216K13.1 | -0.708262632 | 0.808725193 | down |
| AJ239318.1 | -0.708262632 | 0.808725193 | down |
| OR4K12P | -0.708262632 | 0.808725193 | down |
| RNU6-954P | -0.708262632 | 0.808725193 | down |
| RPS26P5 | -0.708262632 | 0.808725193 | down |
| AP000403.3 | -0.708262632 | 0.808725193 | down |
| RPS3AP1 | -0.708262632 | 0.808725193 | down |
| AP000233.2 | -0.708262632 | 0.808725193 | down |
| EIF4A1P1 | -0.708262632 | 0.808725193 | down |
| KRTAP20-2 | -0.708262632 | 0.808725193 | down |
| NA | -0.708262632 | 0.808725193 | down |
| MIR649 | -0.708262632 | 0.808725193 | down |
| IGLVI-38 | -0.708262632 | 0.808725193 | down |
| RP5-1119A7.10 | -0.708262632 | 0.808725193 | down |
| MIR33A | -0.708262632 | 0.808725193 | down |
| Z82214.2 | -0.708262632 | 0.808725193 | down |
| SNORA20 | -0.708262632 | 0.808725193 | down |
| GS1-542M4.3 | -0.708262632 | 0.808725193 | down |
| FRMPD4-AS1 | -0.708262632 | 0.808725193 | down |
| MAGEB6P1 | -0.708262632 | 0.808725193 | down |
| PLCE1P1 | -0.708262632 | 0.808725193 | down |
| RNA5SP501 | -0.708262632 | 0.808725193 | down |
| RP11-32A1.2 | -0.708262632 | 0.808725193 | down |
| FTH1P18 | -0.708262632 | 0.808725193 | down |
| MIR548AJ2 | -0.708262632 | 0.808725193 | down |
| RP11-552J9.6 | -0.708262632 | 0.808725193 | down |
| RP11-30M20.1 | -0.708262632 | 0.808725193 | down |
| PKMP2 | -0.708262632 | 0.808725193 | down |
| RP13-26D14.2 | -0.708262632 | 0.808725193 | down |
| AC004386.3 | -0.708262632 | 0.808725193 | down |
| RN7SL460P | -0.708262632 | 0.808725193 | down |
| PPATP2 | -0.708262632 | 0.808725193 | down |
| SETP4 | -0.708262632 | 0.808725193 | down |
| RNA5SP510 | -0.708262632 | 0.808725193 | down |
| BRDTP1 | -0.708262632 | 0.808725193 | down |
| RPL6P29 | -0.708262632 | 0.808725193 | down |
| RNU6-934P | -0.708262632 | 0.808725193 | down |
| MTND2P2 | -0.708262632 | 0.808725193 | down |
| RP1-26905.3 | -0.708262632 | 0.808725193 | down |
| RNU6-1323P | -0.708262632 | 0.808725193 | down |
| SNORD112 | -0.708262632 | 0.808725193 | down |
| HS6ST2-AS1 | -0.708262632 | 0.808725193 | down |
| RP13-210D15.4 | -0.708262632 | 0.808725193 | down |
| RNU6ATAC23P | -0.708262632 | 0.808725193 | down |
| RPS17P17 | -0.708262632 | 0.808725193 | down |
| GS1-256022.5 | -0.708262632 | 0.808725193 | down |
| GS1-115M3.4 | -0.708262632 | 0.808725193 | down |
| RP11-183K14.1 | -0.708262632 | 0.808725193 | down |
| RP11-329E24.6 | -0.708262632 | 0.808725193 | down |
| OR3B1P | -0.708262632 | 0.808725193 | down |
| CTD-3162L10.3 | -0.708262426 | 0.788487743 | down |

| | | | | |
|---|---|---|---|---|
| CTD-3131K8.1 | -0.708262423 | 0.788179397 | | down |
| DSC2 | -0.708136768 | 0.012854618 | 0.044386179 | down |
| CTB-147N14.6 | -0.707971633 | 0.121139554 | 0.231665616 | down |
| CTD-2535I10.1 | -0.707854227 | 0.584107962 | | down |
| FITM1 | -0.70778067 | 0.024587518 | 0.072400999 | down |
| LINC00412 | -0.707759449 | 0.193588986 | 0.321709629 | down |
| MYHAS | -0.707631592 | 0.647464749 | | down |
| RXRA | -0.707620308 | 9.55E-08 | 2.73E-06 | down |
| FGL1 | -0.707588761 | 0.425020914 | | down |
| RP11-813P10.2 | -0.707296053 | 0.455798149 | | down |
| FAM105A | -0.707281813 | 0.002161244 | 0.011169319 | down |
| AC009474.1 | -0.707064102 | 0.809041292 | | down |
| RP11-524C21.1 | -0.707064102 | 0.809041292 | | down |
| RSL24D1P3 | -0.707064102 | 0.809041292 | | down |
| RP11-642E20.2 | -0.70705817 | 0.796847441 | | down |
| RP11-142I2.1 | -0.707028562 | 0.745654035 | | down |
| NA | -0.707027538 | 0.744094705 | | down |
| RP11-453E17.4 | -0.707026952 | 0.74320779 | | down |
| DYTN | -0.707026278 | 0.74219127 | | down |
| RP11-728K20.1 | -0.707008499 | 0.716921685 | | down |
| RP11-1100L3.7 | -0.706986945 | 0.689541428 | | down |
| RP11-203M5.2 | -0.706985432 | 0.68773019 | | down |
| NA | -0.706985323 | 0.687599803 | | down |
| OR7E21P | -0.706983296 | 0.685194212 | | down |
| RP11-328P23.3 | -0.706963028 | 0.662315238 | | down |
| RP5-961K14.1 | -0.70696275 | 0.662015218 | | down |
| RNY4P6 | -0.706962429 | 0.661669076 | | down |
| AC003973.3 | -0.706932673 | 0.631396022 | | down |
| RP11-959F10.4 | -0.706905179 | 0.606134061 | | down |
| RN7SKP271 | -0.706897736 | 0.599672989 | | down |
| MRPL54 | -0.706259146 | 0.000891212 | 0.005519601 | down |
| RNU2-36P | -0.705840535 | 0.688450016 | | down |
| RP11-146F11.5 | -0.705810784 | 0.155676388 | 0.275904991 | down |
| ASPA | -0.705495716 | 0.022982559 | 0.068724179 | down |
| PHKG2 | -0.705478816 | 0.002684747 | 0.013292774 | down |
| RPL35P5 | -0.70547495 | 0.04404309 | 0.11153377 | down |
| TUBBP5 | -0.705469897 | 0.041997667 | 0.107798197 | down |
| RP11-430G17.3 | -0.705425753 | 0.091410367 | 0.189546074 | down |
| SHROOM3 | -0.705354338 | 0.003855105 | 0.017699081 | down |
| NA | -0.705254909 | 0.173873112 | 0.298386004 | down |
| GPR142 | -0.705190844 | 0.234826001 | 0.367626095 | down |
| CDC25C | -0.705040546 | 0.003508835 | 0.016412803 | down |
| EEF1A1P38 | -0.704976857 | 0.1665484 | 0.289660362 | down |
| RP11-173A16.2 | -0.704873445 | 0.554661046 | | down |
| ERI3 | -0.704751986 | 0.00035547 | 0.002671273 | down |
| AP4M1 | -0.704502087 | 5.78E-08 | 1.79E-06 | down |
| FAM174B | -0.704483221 | 0.000363224 | 0.002712713 | down |
| KIAA2013 | -0.704381425 | 1.73E-05 | 0.00022214 | down |
| GAS2L3 | -0.70431738 | 0.001060567 | 0.006356206 | down |
| RN7SL277P | -0.704287528 | 0.63799187 | | down |
| MFSD5 | -0.704242191 | 0.001136024 | 0.006721286 | down |

| | | | | |
|---|---|---|---|---|
| GPR158 | -0.703904307 | 0.189454614 | 0.316633742 | down |
| XKRX | -0.703850875 | 0.08553418 | 0.180932824 | down |
| CBLN4 | -0.703718122 | 0.373412461 | 0.511888873 | down |
| UBE2V2P3 | -0.703706754 | 0.600205274 | | down |
| CDK18 | -0.703634578 | 0.000105928 | 0.000992673 | down |
| PLXNB3 | -0.703452924 | 0.188377024 | 0.315262884 | down |
| LINC00959 | -0.703349462 | 0.004897859 | 0.021354127 | down |
| NTN3 | -0.703073311 | 0.2347655 | 0.367588841 | down |
| CTD-2619J13.16 | -0.702507721 | 0.169597522 | 0.293225986 | down |
| SLC16A8 | -0.702396913 | 0.071973743 | 0.159549851 | down |
| MPP6 | -0.702369687 | 0.014950596 | 0.049819517 | down |
| RP11-379L18.2 | -0.702347627 | 0.768669273 | | down |
| LPAL2 | -0.702172328 | 0.044109472 | 0.111635497 | down |
| LA16c-13E4.3 | -0.702132197 | 0.54340886 | 0.664855554 | down |
| KRR1P1 | -0.702068746 | 0.327801829 | 0.466222219 | down |
| SNORA70 | -0.70204106 | 0.810356617 | | down |
| DNAJC17 | -0.701860698 | 0.001991041 | 0.01045053 | down |
| SRIP2 | -0.701821417 | 0.810427352 | | down |
| NA | -0.701798671 | 0.797410264 | | down |
| RP11-359N11.1 | -0.70154755 | 0.690924095 | | down |
| LINC01120 | -0.701541283 | 0.797658649 | | down |
| RP11-415J8.5 | -0.701459395 | 0.662494948 | | down |
| AP001630.5 | -0.701459245 | 0.662449111 | | down |
| RP11-434D2.11 | -0.701452755 | 0.660471941 | | down |
| SIGIRR | -0.701434462 | 0.002023568 | 0.010600888 | down |
| RP4-791M13.3 | -0.701430368 | 0.690820805 | | down |
| CTC-429L19.3 | -0.701351462 | 0.250300829 | 0.384971748 | down |
| CCNL2P1 | -0.701250752 | 0.605074165 | | down |
| AC008171.1 | -0.701100441 | 0.721341585 | | down |
| HSPBP1 | -0.701063699 | 0.005189269 | 0.022301015 | down |
| COX14 | -0.70102838 | 0.001900564 | 0.010061771 | down |
| RP11-266L9.4 | -0.700936653 | 0.023908172 | 0.07089082 | down |
| ETFBKMT | -0.70092546 | 0.000169727 | 0.001460272 | down |
| LIPC-AS1 | -0.700752737 | 0.692273884 | | down |
| NA | -0.700665053 | 0.466943216 | 0.598065204 | down |
| RP11-504P24.3 | -0.700611048 | 0.002355295 | 0.011983486 | down |
| RP11-118H4.1 | -0.700610179 | 0.308868431 | 0.447342761 | down |
| Y_RNA | -0.700526961 | 0.690456361 | | down |
| RP11-352E6.2 | -0.700522373 | 0.525738916 | 0.649504503 | down |
| NA | -0.700518203 | 0.241853072 | 0.375846154 | down |
| AL133243.4 | -0.700469488 | 0.322997932 | 0.461552907 | down |
| C7orf50 | -0.700420801 | 0.00194347 | 0.010235252 | down |
| TMEM14DP | -0.700212611 | 0.664599463 | | down |
| PNPLA7 | -0.700154986 | 0.004863646 | 0.021229739 | down |
| CTD-2311M21.2 | -0.700127063 | 0.474093443 | | down |
| NA | -0.699888966 | 0.317085824 | 0.455836476 | down |
| CAPN8 | -0.699857418 | 0.065128156 | 0.148168282 | down |
| ANKMY1 | -0.699700541 | 0.000333656 | 0.002543103 | down |
| RP11-613E4.4 | -0.699534421 | 0.674150498 | | down |
| RP3-514A23.2 | -0.699272315 | 0.560978061 | | down |
| MIR4730 | -0.699272256 | 0.615013219 | | down |

| | | | | |
|---|---|---|---|---|
| AC008592.8 | -0.699270383 | 0.614213686 | | down |
| NA | -0.699069111 | 0.745079919 | | down |
| RP11-486012.2 | -0.698983719 | 0.00807408 | 0.031153701 | down |
| RP11-7F17.4 | -0.698928656 | 0.669664444 | 0.769550227 | down |
| Y_RNA | -0.698853582 | 0.811217939 | | down |
| NA | -0.698853582 | 0.811217939 | | down |
| RP11-287D1.5 | -0.698853582 | 0.811217939 | | down |
| SMIM12P1 | -0.698853582 | 0.811217939 | | down |
| AC011999.1 | -0.698853582 | 0.811217939 | | down |
| UBBP3 | -0.698853582 | 0.811217939 | | down |
| CHCHD4P4 | -0.698853582 | 0.811217939 | | down |
| AC116035.2 | -0.698853582 | 0.811217939 | | down |
| Metazoa_SRP | -0.698853582 | 0.811217939 | | down |
| UQCRC2P1 | -0.698853582 | 0.811217939 | | down |
| CCDC54 | -0.698853582 | 0.811217939 | | down |
| CCDC37-AS1 | -0.698853582 | 0.811217939 | | down |
| RP11-278L15.2 | -0.698853582 | 0.811217939 | | down |
| MTND5P5 | -0.698853582 | 0.811217939 | | down |
| RP11-364M6.1 | -0.698853582 | 0.811217939 | | down |
| NUDT19P5 | -0.698853582 | 0.811217939 | | down |
| CTD-2194D22.5 | -0.698853582 | 0.811217939 | | down |
| RP11-269M20.1 | -0.698853582 | 0.811217939 | | down |
| AC016644.1 | -0.698853582 | 0.811217939 | | down |
| RNU6-540P | -0.698853582 | 0.811217939 | | down |
| RP11-79P5.3 | -0.698853582 | 0.811217939 | | down |
| RNU6-260P | -0.698853582 | 0.811217939 | | down |
| HLA-P | -0.698853582 | 0.811217939 | | down |
| WASF5P | -0.698853582 | 0.811217939 | | down |
| RP3-403A15.1 | -0.698853582 | 0.811217939 | | down |
| RP11-548K12.9 | -0.698853582 | 0.811217939 | | down |
| Y_RNA | -0.698853582 | 0.811217939 | | down |
| SNRPBP1 | -0.698853582 | 0.811217939 | | down |
| RP11-539E17.5 | -0.698853582 | 0.811217939 | | down |
| FAM25A | -0.698853582 | 0.811217939 | | down |
| OR51V1 | -0.698853582 | 0.811217939 | | down |
| RP11-72M10.4 | -0.698853582 | 0.811217939 | | down |
| GLYATL1P2 | -0.698853582 | 0.811217939 | | down |
| OR10V3P | -0.698853582 | 0.811217939 | | down |
| OOSP2 | -0.698853582 | 0.811217939 | | down |
| RPA2P3 | -0.698853582 | 0.811217939 | | down |
| CTD-2355J17.2 | -0.698853582 | 0.811217939 | | down |
| RP13-653N12.2 | -0.698853582 | 0.811217939 | | down |
| RP11-351N4.4 | -0.698853582 | 0.811217939 | | down |
| RNA5SP28 | -0.698853582 | 0.811217939 | | down |
| RP11-99L13.2 | -0.698853582 | 0.811217939 | | down |
| RP11-857B24.2 | -0.698853582 | 0.811217939 | | down |
| RP11-300A12.2 | -0.698853582 | 0.811217939 | | down |
| RP11-123C21.1 | -0.698853582 | 0.811217939 | | down |
| RP11-19J5.1 | -0.698853582 | 0.811217939 | | down |
| CTA-363E6.5 | -0.698853582 | 0.811217939 | | down |
| CTD-2267D19.7 | -0.698853582 | 0.811217939 | | down |

| | | | | |
|---|---|---|---|---|
| KRTAP4-16 | -0.698853582 | 0.811217939 | | down |
| CTC-264K15.6 | -0.698853582 | 0.811217939 | | down |
| FAM166AP6 | -0.698853582 | 0.811217939 | | down |
| CTC-360G5.8 | -0.698853582 | 0.811217939 | | down |
| LINC01056 | -0.698853582 | 0.811217939 | | down |
| AL049758.2 | -0.698853582 | 0.811217939 | | down |
| LUZP4 | -0.698853582 | 0.811217939 | | down |
| ZIC3 | -0.698853582 | 0.811217939 | | down |
| ZNF886P | -0.698853582 | 0.811217939 | | down |
| YWHAZP6 | -0.698853385 | 0.788154431 | | down |
| CTD-3216D2.4 | -0.69885336 | 0.784514424 | | down |
| RP4-597N16.1 | -0.698853349 | 0.782933353 | | down |
| HMGN2P28 | -0.698853211 | 0.758515797 | | down |
| AF228730.8 | -0.698853194 | 0.755103042 | | down |
| KCNMA1-AS3 | -0.698787252 | 0.714611701 | | down |
| TMEM151A | -0.698584104 | 0.17858223 | 0.303623141 | down |
| CTD-3035D6.1 | -0.69856192 | 0.074601812 | 0.163708775 | down |
| LENG8-AS1 | -0.698513451 | 0.01811754 | 0.057679705 | down |
| RP11-489M13.3 | -0.698483904 | 0.584506105 | | down |
| PMPCA | -0.698370064 | 0.000248086 | 0.001999331 | down |
| ZNF726 | -0.698301572 | 0.074006156 | 0.162717182 | down |
| ARSH | -0.698118701 | 0.496173547 | | down |
| CTU1 | -0.698020202 | 0.0091935 | 0.034384354 | down |
| RP11-474I11.7 | -0.697976209 | 0.639071209 | | down |
| RP11-634H22.1 | -0.697903598 | 0.102237771 | 0.205240652 | down |
| NA | -0.697825442 | 0.591269425 | | down |
| CCDC171 | -0.697764056 | 0.006357605 | 0.02607616 | down |
| HOXB9 | -0.697641467 | 0.021120301 | 0.06464327 | down |
| TMEM67 | -0.697597269 | 0.032052289 | 0.088055868 | down |
| CYP46A1 | -0.697507692 | 0.02967434 | 0.083167322 | down |
| RP11-2C24.3 | -0.697481498 | 0.019475349 | 0.06082484 | down |
| AC021218.2 | -0.697383919 | 0.151206613 | 0.270343459 | down |
| UBOX5-AS1 | -0.697292414 | 0.172404134 | 0.296695487 | down |
| NPW | -0.697083589 | 0.080501428 | 0.173006486 | down |
| RP11-408A13.3 | -0.697057941 | 0.165202102 | 0.287974466 | down |
| LYPD6 | -0.697037881 | 0.028955734 | 0.081800289 | down |
| CTC-756D1.2 | -0.696840845 | 0.311835464 | 0.450449373 | down |
| AC144449.1 | -0.696797274 | 0.137168032 | 0.252532407 | down |
| CDKL2 | -0.696790471 | 0.051685661 | 0.125259659 | down |
| RPL13P2 | -0.696681646 | 0.428058051 | | down |
| CPTP | -0.696581526 | 0.002090337 | 0.010859778 | down |
| RP4-568F9.6 | -0.696539241 | 0.646643677 | | down |
| NA | -0.696470071 | 0.133144651 | 0.247289376 | down |
| TLCD1 | -0.696350942 | 0.01732824 | 0.055678867 | down |
| PRICKLE2-AS1 | -0.696348622 | 0.669566623 | | down |
| RP5-1184F4.7 | -0.696136187 | 0.325302528 | 0.46380928 | down |
| LSM7 | -0.695837352 | 0.005902836 | 0.02464496 | down |
| CTD-2194A8.2 | -0.695721382 | 0.647932105 | | down |
| RP11-5809.2 | -0.694753014 | 0.15533043 | 0.275426902 | down |
| EXTL3-AS1 | -0.69462824 | 0.140829955 | 0.257397201 | down |
| RP11-66N11.8 | -0.69460986 | 0.02468106 | 0.072612394 | down |

| | | | | |
|---|---|---|---|---|
| ZFHX4 | -0.694595962 | 0.156085888 | 0.276402477 | down |
| SIPA1L3 | -0.694453319 | 4.91E-05 | 0.000527319 | down |
| RP3-460G2.2 | -0.69433761 | 0.223435911 | 0.355500107 | down |
| NA | -0.694311617 | 0.114256793 | 0.222168862 | down |
| RP11-120K18.3 | -0.694055189 | 0.603274773 | | down |
| ZCWPW2 | -0.694038744 | 0.079970683 | 0.172173998 | down |
| DHCR24 | -0.693950091 | 0.006763077 | 0.027295635 | down |
| AC092168.2 | -0.693853139 | 0.660904679 | | down |
| NENF | -0.693765728 | 0.006470128 | 0.026411486 | down |
| RP11-212F11.1 | -0.693651329 | 0.72003221 | | down |
| RP11-7K24.3 | -0.693575582 | 0.072928172 | 0.161088429 | down |
| NA | -0.693464074 | 0.348457865 | 0.487159986 | down |
| RPL13AP5 | -0.693332416 | 0.000242283 | 0.001960977 | down |
| RP11-159D12.6 | -0.693086662 | 0.089716959 | 0.187145478 | down |
| CCNDBP1 | -0.692936073 | 1.89E-05 | 0.00023904 | down |
| RP11-714L20.1 | -0.692861252 | 0.362828272 | 0.501232877 | down |
| MFSD13A | -0.692762794 | 2.71E-05 | 0.000321359 | down |
| RPL21P111 | -0.692638243 | 0.722536036 | | down |
| RP11-386P4.1 | -0.692596799 | 0.696126069 | 0.79065521 | down |
| PDC | -0.692573687 | 0.638828074 | | down |
| CTD-2369P2.5 | -0.692488289 | 0.558999432 | | down |
| TMEM98 | -0.692211318 | 0.006448249 | 0.026352706 | down |
| RP11-544A12.4 | -0.692190999 | 0.416509751 | | down |
| ZNF620 | -0.692118141 | 0.00193391 | 0.010202989 | down |
| NA | -0.69189213 | 0.598515812 | 0.712179059 | down |
| LGALSL | -0.691862899 | 0.000386942 | 0.002843139 | down |
| RNF186 | -0.691715366 | 0.033354316 | 0.090679313 | down |
| RP11-10N23.2 | -0.691606752 | 0.184459476 | 0.310470617 | down |
| BRMS1 | -0.691568559 | 0.001717713 | 0.009306935 | down |
| CDK5R2 | -0.691567874 | 0.126443972 | 0.23864101 | down |
| CALHM1 | -0.691540075 | 0.186667456 | 0.313047002 | down |
| RP11-130F10.1 | -0.691525893 | 0.150439532 | 0.269549457 | down |
| RP11-159H10.3 | -0.691447258 | 0.258690247 | 0.393822639 | down |
| RP11-330H6.6 | -0.691312474 | 0.11994014 | 0.230092687 | down |
| MIR137HG | -0.691205332 | 0.377726061 | 0.515789555 | down |
| RP11-248J23.5 | -0.691149995 | 0.566627644 | 0.684760331 | down |
| FLNB | -0.691077691 | 0.000340619 | 0.002583581 | down |
| BMS1P4 | -0.690875411 | 0.094336912 | 0.193990351 | down |
| RP11-378E13.3 | -0.69083572 | 0.427337208 | | down |
| DAZL | -0.69083 | 0.207556101 | 0.337746602 | down |
| OR7E22P | -0.690754451 | 0.473416607 | 0.603783527 | down |
| NA | -0.690620299 | 0.194427411 | 0.322710189 | down |
| NPIPB15 | -0.690592825 | 0.208537488 | 0.338714357 | down |
| LYPLA2 | -0.690535866 | 0.001497501 | 0.008323946 | down |
| RBM34 | -0.690479158 | 0.071816411 | 0.15929201 | down |
| IL17RB | -0.690400077 | 0.008630906 | 0.032732509 | down |
| NA | -0.690319411 | 0.452519372 | 0.585205407 | down |
| C15orf52 | -0.690309652 | 0.000424262 | 0.00304795 | down |
| AC098820.3 | -0.690217258 | 0.101149983 | 0.203669332 | down |
| LINC01137 | -0.690207115 | 0.00233939 | 0.011920731 | down |
| TMEM91 | -0.690164943 | 0.019513187 | 0.060890548 | down |

| | | | | |
|---|---|---|---|---|
| ATP8B1 | -0.690144303 | 0.005361564 | 0.022887587 | down |
| RP11-368N21.5 | -0.690129851 | 0.055126381 | 0.131299904 | down |
| CTD-2542C24.3 | -0.69008077 | 0.383833008 | 0.521544882 | down |
| CTC-441N14.1 | -0.689977991 | 0.555845476 | | down |
| GMPPA | -0.689928727 | 0.011694431 | 0.04137881 | down |
| PTENP1-AS | -0.689754788 | 0.443258626 | | down |
| KALRN | -0.689681345 | 0.00271369 | 0.013416175 | down |
| ARHGEF34P | -0.689643586 | 0.046958104 | 0.11675985 | down |
| CITF22-92A6.1 | -0.689623227 | 0.002170958 | 0.011206919 | down |
| SCLY | -0.689621471 | 0.013731104 | 0.046646043 | down |
| DGCR6L | -0.689559621 | 0.000708802 | 0.004608542 | down |
| RN7SL810P | -0.689457555 | 0.714444287 | 0.804074327 | down |
| GAPDHP23 | -0.689444532 | 0.8137126 | | down |
| LCE2C | -0.689444532 | 0.8137126 | | down |
| RPS7P2 | -0.689444532 | 0.8137126 | | down |
| Metazoa_SRP | -0.689444532 | 0.8137126 | | down |
| RP11-95P13.2 | -0.689444532 | 0.8137126 | | down |
| RP11-478H16.1 | -0.689444532 | 0.8137126 | | down |
| AC104781.1 | -0.689444532 | 0.8137126 | | down |
| RP11-127H13.1 | -0.689444532 | 0.8137126 | | down |
| AC108051.2 | -0.689444532 | 0.8137126 | | down |
| AC013727.2 | -0.689444532 | 0.8137126 | | down |
| OR7E28P | -0.689444532 | 0.8137126 | | down |
| OR5H7P | -0.689444532 | 0.8137126 | | down |
| WWP1P1 | -0.689444532 | 0.8137126 | | down |
| NA | -0.689444532 | 0.8137126 | | down |
| NA | -0.689444532 | 0.8137126 | | down |
| RN7SL275P | -0.689444532 | 0.8137126 | | down |
| MIR7849 | -0.689444532 | 0.8137126 | | down |
| RP11-18H21.2 | -0.689444532 | 0.8137126 | | down |
| RP11-116B13.1 | -0.689444532 | 0.8137126 | | down |
| RP11-252I13.1 | -0.689444532 | 0.8137126 | | down |
| MIR1271 | -0.689444532 | 0.8137126 | | down |
| RPL12P1 | -0.689444532 | 0.8137126 | | down |
| MESTP1 | -0.689444532 | 0.8137126 | | down |
| HAUS6P1 | -0.689444532 | 0.8137126 | | down |
| RP11-10F11.4 | -0.689444532 | 0.8137126 | | down |
| RP11-792A8.1 | -0.689444532 | 0.8137126 | | down |
| KB-1205A7.2 | -0.689444532 | 0.8137126 | | down |
| MIR3686 | -0.689444532 | 0.8137126 | | down |
| RNU4-29P | -0.689444532 | 0.8137126 | | down |
| RP11-276H19.4 | -0.689444532 | 0.8137126 | | down |
| RN7SL398P | -0.689444532 | 0.8137126 | | down |
| RP11-508N22.9 | -0.689444532 | 0.8137126 | | down |
| CHEK2P5 | -0.689444532 | 0.8137126 | | down |
| RP11-119F7.3 | -0.689444532 | 0.8137126 | | down |
| RPL31P47 | -0.689444532 | 0.8137126 | | down |
| ZNF970P | -0.689444532 | 0.8137126 | | down |
| OR5BJ1P | -0.689444532 | 0.8137126 | | down |
| RPL21P103 | -0.689444532 | 0.8137126 | | down |
| RN7SL515P | -0.689444532 | 0.8137126 | | down |

| | | | | |
|---|---|---|---|---|
| RP11-279N8.1 | -0.689444532 | 0.8137126 | | down |
| CTD-2515H24.3 | -0.689444532 | 0.8137126 | | down |
| DDX11L9 | -0.689444532 | 0.8137126 | | down |
| RPL7L1P5 | -0.689444532 | 0.8137126 | | down |
| RP11-861L17.3 | -0.689444532 | 0.8137126 | | down |
| RP11-63N3.2 | -0.689444532 | 0.8137126 | | down |
| CTD-2291D10.1 | -0.689444532 | 0.8137126 | | down |
| CTD-3187F8.6 | -0.689444532 | 0.8137126 | | down |
| NEK2P2 | -0.689444532 | 0.8137126 | | down |
| RP1-27C22.1 | -0.689444532 | 0.8137126 | | down |
| BX842568.1 | -0.689444532 | 0.8137126 | | down |
| FTLP16 | -0.689444532 | 0.8137126 | | down |
| AC004074.4 | -0.689444532 | 0.8137126 | | down |
| RP11-775J23.2 | -0.689444531 | 0.81359414 | | down |
| HMGB1P23 | -0.689444531 | 0.81365145 | | down |
| RP11-357N13.1 | -0.689444528 | 0.813329488 | | down |
| RP11-398A8.2 | -0.689444524 | 0.812821223 | | down |
| GS1-115G20.2 | -0.689444503 | 0.810214601 | | down |
| RP11-849N15.1 | -0.689444334 | 0.784411419 | | down |
| NA | -0.689444331 | 0.783936039 | | down |
| MIR191 | -0.689444311 | 0.780003172 | | down |
| MIR4483 | -0.689444221 | 0.759958348 | | down |
| TRAJ24 | -0.689444109 | 0.725090173 | | down |
| MAS1L | -0.689421862 | 0.306610104 | 0.444949715 | down |
| RP11-216N14.7 | -0.689312589 | 0.43408255 | | down |
| ITPKB-AS1 | -0.689252337 | 0.47655117 | | down |
| HADHB | -0.689147609 | 8.51E-05 | 0.000826532 | down |
| ZNF460 | -0.688969851 | 0.097221713 | 0.198109682 | down |
| NA | -0.688831513 | 0.03294821 | 0.08987616 | down |
| PLD4 | -0.688818972 | 0.041568481 | 0.106989073 | down |
| RP11-703I16.3 | -0.688681582 | 0.459150442 | 0.591083893 | down |
| CTD-3105H18.14 | -0.68836056 | 0.23212623 | 0.364787135 | down |
| REN | -0.688213705 | 0.416029039 | 0.552352134 | down |
| CTD-2325M2.1 | -0.688133017 | 0.06717887 | 0.151707904 | down |
| AC022182.1 | -0.688090411 | 0.062614108 | 0.144206842 | down |
| ZRANB2-AS1 | -0.687937315 | 0.215917057 | | down |
| HPYR1 | -0.687735061 | 0.752063449 | | down |
| AC016831.7 | -0.687479407 | 0.095271558 | 0.195223549 | down |
| AC011551.3 | -0.687362041 | 0.316442862 | 0.455042805 | down |
| RHOC | -0.687358015 | 0.012352612 | 0.04310879 | down |
| NA | -0.687261536 | 0.616857883 | | down |
| PEX16 | -0.68720078 | 0.000612039 | 0.004103572 | down |
| RP11-150012.6 | -0.686968787 | 0.094338213 | 0.193990351 | down |
| BAD | -0.686953927 | 0.005478487 | 0.023254546 | down |
| SMC5-AS1 | -0.686799414 | 0.092922117 | 0.19171488 | down |
| RPS16P5 | -0.686797766 | 0.288064138 | 0.425371029 | down |
| RP11-863P13.5 | -0.686776943 | 0.634952058 | | down |
| KCNQ5-IT1 | -0.686592231 | 0.620376281 | | down |
| CNGA4 | -0.686558594 | 0.26302314 | 0.398299758 | down |
| NA | -0.686529184 | 0.349183234 | 0.487878951 | down |
| RP11-635L1.3 | -0.686477882 | 0.297320139 | 0.434744062 | down |

| | | | | |
|---|---|---|---|---|
| PNPO | -0.686468558 | 0.000363422 | 0.002712713 | down |
| AC008269.2 | -0.686117346 | 0.149830952 | 0.268747529 | down |
| CFAP43 | -0.68592259 | 0.128015448 | 0.240651741 | down |
| CFAP58 | -0.685892383 | 0.239479883 | 0.373084449 | down |
| PAIP1P1 | -0.685775279 | 0.163258622 | 0.285386749 | down |
| PGA5 | -0.68572687 | 0.538471904 | 0.660536089 | down |
| AC026150.6 | -0.685633619 | 0.176463241 | 0.301299251 | down |
| CRTC3-AS1 | -0.685514166 | 0.170559926 | 0.294413702 | down |
| NA | -0.685476035 | 0.563481524 | | down |
| HERC2P3 | -0.685386581 | 0.075898008 | 0.165681883 | down |
| GS1-184P14.2 | -0.685187941 | 0.100418424 | 0.202562853 | down |
| TONSL | -0.68491948 | 0.000369998 | 0.002747459 | down |
| KRT18P28 | -0.684587938 | 0.389937665 | | down |
| CLIC3 | -0.684484227 | 0.020963906 | 0.064282466 | down |
| CCT6B | -0.684328569 | 0.004455826 | 0.019847417 | down |
| RN7SL521P | -0.683934128 | 0.208549841 | 0.338714357 | down |
| RP6-218J18.2 | -0.68387229 | 0.197834822 | 0.326543753 | down |
| OCLM | -0.683845558 | 0.565095625 | | down |
| RP1-269M15.3 | -0.683839225 | 0.451306498 | | down |
| HSPB9 | -0.683830493 | 0.183677386 | 0.309466319 | down |
| DCP1A | -0.683733286 | 0.000156963 | 0.001369276 | down |
| FAM161B | -0.683720483 | 0.012953207 | 0.044609645 | down |
| ZNF91 | -0.683689937 | 0.000127348 | 0.001155205 | down |
| RP11-127B20.2 | -0.683679652 | 0.030023467 | 0.083802854 | down |
| PTCHD3P3 | -0.683654559 | 0.683985194 | | down |
| EFCAB11 | -0.683509123 | 5.56E-05 | 0.000583248 | down |
| ZNF629 | -0.683500564 | 9.50E-06 | 0.000135288 | down |
| SLC4A8 | -0.683436045 | 0.032576519 | 0.089121192 | down |
| RP11-358B23.5 | -0.683411651 | 0.488637208 | | down |
| PXN-AS1 | -0.683390294 | 0.002992235 | 0.014467122 | down |
| NA | -0.683323099 | 0.658343911 | 0.760419994 | down |
| MAPT | -0.683207595 | 0.090909055 | 0.188832388 | down |
| RP11-797A18.6 | -0.682963232 | 0.045635847 | 0.114361978 | down |
| GTF3C5 | -0.682868803 | 0.000267395 | 0.002125858 | down |
| HIST1H1B | -0.682830291 | 0.160489167 | 0.281921585 | down |
| RPL5P18 | -0.682768626 | 0.435407201 | | down |
| HTR6 | -0.682678881 | 0.155975914 | 0.276321739 | down |
| ITIH1 | -0.682571961 | 0.13013428 | 0.243431562 | down |
| KIAA1958 | -0.682527205 | 0.009941783 | 0.036488018 | down |
| RELN | -0.682511205 | 0.07282324 | 0.160934272 | down |
| NCOR1 | -0.682302251 | 6.48E-05 | 0.000659223 | down |
| RP11-554D14.6 | -0.681704942 | 0.320356389 | 0.459175962 | down |
| LINC00472 | -0.681701074 | 0.127276034 | 0.23962158 | down |
| ACER2 | -0.681422301 | 0.225994613 | 0.358035337 | down |
| SPG7 | -0.681203024 | 1.39E-05 | 0.000185504 | down |
| NEIL3 | -0.681026738 | 0.027616515 | 0.078981013 | down |
| CDH23 | -0.680971401 | 0.000765161 | 0.004901596 | down |
| NA | -0.680831273 | 0.448515208 | 0.581478851 | down |
| RP11-1079K10.3 | -0.680730785 | 0.218715985 | 0.35015942 | down |
| DHFR2 | -0.6806598 | 0.00658528 | 0.026736224 | down |
| ZNF812P | -0.680614808 | 0.290878793 | 0.428012658 | down |

| | | | | |
|---|---|---|---|---|
| RP11-883G14.1 | -0.680341808 | 0.650057691 | | down |
| RP11-478P10.1 | -0.680312915 | 0.681291172 | | down |
| INTU | -0.680269353 | 0.038774304 | 0.101484031 | down |
| RFNG | -0.680261336 | 0.002988845 | 0.014455385 | down |
| AC000068.10 | -0.680083354 | 0.441544214 | | down |
| RP11-763B22.6 | -0.680035482 | 0.816209153 | | down |
| CDC27P1 | -0.680035482 | 0.816209153 | | down |
| RP11-289A15.1 | -0.680035482 | 0.816209153 | | down |
| RPS12P5 | -0.680035482 | 0.816209153 | | down |
| RNU6-1256P | -0.680035482 | 0.816209153 | | down |
| RNU6-1105P | -0.680035482 | 0.816209153 | | down |
| AC005609.16 | -0.680035482 | 0.816209153 | | down |
| AC138647.1 | -0.680035482 | 0.816209153 | | down |
| DDX10P2 | -0.680035482 | 0.816209153 | | down |
| MIR129-2 | -0.680035482 | 0.816209153 | | down |
| OR6C75 | -0.680035482 | 0.816209153 | | down |
| RP11-307O13.1 | -0.680035482 | 0.816209153 | | down |
| OR4M1 | -0.680035482 | 0.816209153 | | down |
| CTD-2055G21.1 | -0.680035482 | 0.816209153 | | down |
| CTAGE1 | -0.680035482 | 0.816209153 | | down |
| DPRXP6 | -0.680035474 | 0.814962752 | | down |
| PFN3 | -0.680035473 | 0.814888091 | | down |
| IGLVIV-66-1 | -0.680035472 | 0.814674078 | | down |
| MIR6069 | -0.680035471 | 0.814561664 | | down |
| CTC-339D2.1 | -0.680035468 | 0.814153202 | | down |
| NA | -0.680035465 | 0.813689191 | | down |
| NA | -0.680035462 | 0.813213041 | | down |
| NA | -0.680035456 | 0.812184082 | | down |
| CTD-2555K7.3 | -0.680035453 | 0.811744739 | | down |
| GCNT1P3 | -0.680035438 | 0.809162771 | | down |
| RP11-218E20.5 | -0.680035435 | 0.808644248 | | down |
| OR7A5 | -0.680035427 | 0.807380123 | | down |
| RP3-412A9.15 | -0.680035321 | 0.785855633 | | down |
| AC073957.15 | -0.680035319 | 0.785194591 | | down |
| C1GALT1P2 | -0.680035316 | 0.784583481 | | down |
| RP4-777L9.3 | -0.680035316 | 0.784649322 | | down |
| RP11-693L9.2 | -0.680035314 | 0.784045561 | | down |
| RP11-628M2.1 | -0.680035226 | 0.758802207 | | down |
| PTGES3P3 | -0.680035225 | 0.758411626 | | down |
| RP11-218C14.5 | -0.68003522 | 0.756704588 | | down |
| RP11-321F6.2 | -0.680035217 | 0.755324738 | | down |
| RP11-15E1.5 | -0.680035212 | 0.753675872 | | down |
| RNU6-977P | -0.680035201 | 0.749707443 | | down |
| AC009166.7 | -0.680035067 | 0.6747113 | | down |
| RP11-34P1.1 | -0.679997757 | 0.72975971 | | down |
| NA | -0.679888506 | 0.502927708 | 0.629739104 | down |
| RHOXF1 | -0.679876489 | 0.102959502 | 0.206207272 | down |
| OR10AH1P | -0.679838953 | 0.500695156 | 0.627825843 | down |
| NA | -0.679498585 | 0.072422688 | 0.160296688 | down |
| RP11-314N13.10 | -0.679056925 | 0.220887747 | 0.352547358 | down |
| SNORD3C | -0.678935683 | 0.445382232 | 0.578667394 | down |

| | | | | |
|---|---|---|---|---|
| RP11-699C17.1 | -0.678893028 | 0.307573854 | 0.445896008 | down |
| RPL34-AS1 | -0.678890776 | 0.224746885 | 0.356903327 | down |
| MGMT | -0.678877559 | 0.009418943 | 0.035031284 | down |
| NA | -0.678810006 | 0.250542445 | 0.385244752 | down |
| RPL12P35 | -0.678806545 | 0.300693963 | 0.438456836 | down |
| OR14L1P | -0.678766748 | 0.651012313 | | down |
| PLEKHA6 | -0.678564937 | 0.002487471 | 0.012518188 | down |
| CSPG5 | -0.678488205 | 0.107020372 | 0.212232346 | down |
| LINC01052 | -0.67842661 | 0.752585199 | | down |
| MYH3 | -0.678379982 | 0.003340553 | 0.01575072 | down |
| TWF1P1 | -0.678324448 | 0.448438115 | | down |
| IQCC | -0.678058717 | 0.015069088 | 0.050114643 | down |
| RP11-312B8.1 | -0.678024138 | 0.024099567 | 0.071334717 | down |
| SAT2 | -0.677969858 | 0.000575432 | 0.003900782 | down |
| RP6-99M1.3 | -0.677893083 | 0.247779435 | 0.382110941 | down |
| CCDC36 | -0.677852712 | 0.117299967 | 0.226372058 | down |
| SERF2 | -0.677688017 | 0.030657253 | 0.085173526 | down |
| RP11-107N15.1 | -0.677627224 | 0.169330038 | 0.292885759 | down |
| RP11-1094H24.4 | -0.677464882 | 0.193390761 | 0.321509138 | down |
| ORM2 | -0.677434161 | 0.107914084 | 0.213567666 | down |
| C9 | -0.677430974 | 0.557343302 | | down |
| KB-1742H10.3 | -0.677167601 | 0.267531493 | 0.403277115 | down |
| RN7SKP78 | -0.677096615 | 0.471359958 | 0.601957763 | down |
| MIR210HG | -0.677051371 | 0.01319593 | 0.045232004 | down |
| RP5-1056H1.2 | -0.677039866 | 0.104436858 | 0.208214097 | down |
| RARA-AS1 | -0.676916115 | 0.011777449 | 0.04159907 | down |
| HIST1H3J | -0.676789794 | 0.22041049 | 0.352085258 | down |
| RP11-402G3.4 | -0.676726843 | 0.712787505 | | down |
| UBOX5 | -0.676692164 | 4.17E-05 | 0.000460065 | down |
| SYT17 | -0.676484965 | 0.005150387 | 0.022190828 | down |
| NTAN1P2 | -0.676378587 | 0.140644759 | 0.257168227 | down |
| FAAP24 | -0.676362814 | 0.001897093 | 0.010052252 | down |
| TMEM27 | -0.676248648 | 0.166289252 | 0.289387992 | down |
| DDX11-AS1 | -0.676026872 | 0.072322087 | 0.160133008 | down |
| RP11-803B1.2 | -0.675849361 | 0.509748874 | | down |
| SPHK2 | -0.675815906 | 5.09E-05 | 0.00054282 | down |
| RP5-963E22.4 | -0.675730016 | 0.450866733 | | down |
| CHD1L | -0.675677028 | 0.289819726 | 0.427039914 | down |
| CTD-2002J20.1 | -0.675396583 | 0.419978943 | 0.5557299 | down |
| RP11-45M22.2 | -0.675391434 | 0.139312057 | 0.255463149 | down |
| TBC1D22A | -0.675256704 | 7.06E-07 | 1.51E-05 | down |
| ATP1A1-AS1 | -0.675125307 | 0.018793516 | 0.059235459 | down |
| ARL5C | -0.675082472 | 0.651664952 | | down |
| ACAD10 | -0.675005702 | 8.40E-07 | 1.75E-05 | down |
| LHPP | -0.674973296 | 6.83E-07 | 1.47E-05 | down |
| LINC01446 | -0.674896107 | 0.500293787 | | down |
| NA | -0.674867618 | 0.128627206 | 0.241514641 | down |
| TMEM161B-AS1 | -0.674800609 | 0.000364639 | 0.002719091 | down |
| NKD1 | -0.674712055 | 0.064994051 | 0.147964028 | down |
| RP11-79C23.1 | -0.674654079 | 0.625633868 | 0.734814071 | down |
| AC010894.3 | -0.674637062 | 0.052145869 | 0.125968521 | down |

| | | | | |
|---|---|---|---|---|
| OR2A42 | -0.674578991 | 0.229856663 | 0.362205685 | down |
| CARMIL1 | -0.674573915 | 0.005141509 | 0.02216234 | down |
| EEF1B2P6 | -0.67457115 | 0.008379846 | 0.032024953 | down |
| RNF126P1 | -0.674544498 | 0.382525827 | 0.520239281 | down |
| MIR3677 | -0.674448167 | 0.32096514 | 0.459675692 | down |
| SEPT10P1 | -0.674349549 | 0.550731799 | | down |
| RP13-15M17.1 | -0.674257757 | 0.348530647 | | down |
| GRIK3 | -0.674235978 | 0.111001281 | 0.217812193 | down |
| RP11-727F15.9 | -0.674231782 | 0.136960324 | 0.252258213 | down |
| DHRS1 | -0.674149272 | 9.09E-06 | 0.000130724 | down |
| RP4-734G22.3 | -0.674028766 | 0.033988484 | 0.092089074 | down |
| C5orf42 | -0.67402715 | 0.029834735 | 0.083428405 | down |
| CANT1 | -0.673997981 | 0.002041401 | 0.010671977 | down |
| GOLGA8Q | -0.67393853 | 0.470394273 | | down |
| MROH7-TTC4 | -0.673871796 | 0.496687517 | | down |
| PNKD | -0.673625312 | 0.000392122 | 0.002868497 | down |
| NA | -0.67356747 | 0.696256787 | 0.790714001 | down |
| TTLL12 | -0.673542189 | 0.000250233 | 0.00201231 | down |
| ANKH | -0.673529082 | 3.62E-06 | 6.06E-05 | down |
| LINC00520 | -0.67350467 | 0.301361646 | 0.439136157 | down |
| PLEC | -0.673480302 | 0.002598131 | 0.012957874 | down |
| NA | -0.673400796 | 0.101878842 | 0.204766386 | down |
| RP11-448A19.1 | -0.67333449 | 0.008001731 | 0.030979898 | down |
| COX6C | -0.673202211 | 6.26E-05 | 0.000642838 | down |
| ATP8B3 | -0.673199445 | 0.0489431 | 0.120479024 | down |
| CATSPER1 | -0.673196482 | 0.08746254 | 0.183745752 | down |
| MPI | -0.673183056 | 8.79E-07 | 1.82E-05 | down |
| TSKU | -0.672717704 | 0.002434472 | 0.012302992 | down |
| EPN2-AS1 | -0.672658144 | 0.461508466 | | down |
| RP11-290F24.6 | -0.672493969 | 0.116122303 | 0.224759571 | down |
| SNAP25-AS1 | -0.672410728 | 0.395864205 | 0.532981434 | down |
| LINC01597 | -0.672118241 | 0.057885164 | 0.136175856 | down |
| COL9A1 | -0.672095767 | 0.11758679 | 0.226823597 | down |
| RP11-76C10.4 | -0.671773815 | 0.715715552 | | down |
| RP13-554M15.2 | -0.671747481 | 0.20785361 | 0.338047629 | down |
| RN7SL181P | -0.671744823 | 0.39617663 | 0.533199715 | down |
| EIF6 | -0.671639763 | 0.000551563 | 0.003769601 | down |
| FAM221B | -0.671573939 | 0.366696744 | 0.505191425 | down |
| LINC00265 | -0.671213718 | 0.039163065 | 0.102296568 | down |
| UGDH-AS1 | -0.671060994 | 0.011537362 | 0.040958114 | down |
| NDUFB2 | -0.671052665 | 0.003566603 | 0.016628649 | down |
| ZNF200 | -0.670975709 | 0.000633094 | 0.004209176 | down |
| DLX1 | -0.670962761 | 0.217379152 | 0.348743195 | down |
| BTBD9 | -0.670941662 | 5.09E-10 | 2.85E-08 | down |
| NFIA | -0.67063714 | 0.00266689 | 0.013218366 | down |
| RP11-335E14.1 | -0.670626432 | 0.818707572 | | down |
| MIOXP1 | -0.670626432 | 0.818707572 | | down |
| RP11-20J1.1 | -0.670626432 | 0.818707572 | | down |
| RP11-244H18.3 | -0.670626432 | 0.818707572 | | down |
| RP11-133L19.1 | -0.670626432 | 0.818707572 | | down |
| RP11-702C7.2 | -0.670626432 | 0.818707572 | | down |

| | | | | |
|---|---|---|---|---|
| RNU6-780P | -0.670626418 | 0.815809051 | | down |
| POM121L14P | -0.670626416 | 0.815320181 | | down |
| NA | -0.670626415 | 0.815141729 | | down |
| RPS24P16 | -0.670626414 | 0.814913745 | | down |
| RP11-289H16.1 | -0.67062641 | 0.813938693 | | down |
| PAQR9 | -0.670626408 | 0.813529498 | | down |
| MIR3139 | -0.670626407 | 0.813410514 | | down |
| RNU6-313P | -0.670626407 | 0.813237166 | | down |
| OR11H4 | -0.670626406 | 0.813031609 | | down |
| RP11-168P13.1 | -0.670626405 | 0.812809465 | | down |
| EFCAB3 | -0.670626403 | 0.812384242 | | down |
| NA | -0.670626401 | 0.811824488 | | down |
| LINC01350 | -0.670626398 | 0.811283026 | | down |
| IGKV2-10 | -0.670626312 | 0.786996269 | | down |
| RP11-63E5.6 | -0.670626312 | 0.787206453 | | down |
| RP11-14I17.1 | -0.670626312 | 0.787092218 | | down |
| RP11-225N10.3 | -0.670626308 | 0.785799812 | | down |
| Y_RNA | -0.670626308 | 0.785763466 | | down |
| Y_RNA | -0.670626253 | 0.764098451 | | down |
| RP11-578B16.1 | -0.670626247 | 0.760963734 | | down |
| RP11-12K11.1 | -0.670626233 | 0.754017014 | | down |
| TAS2R50 | -0.670626198 | 0.733785755 | | down |
| RP11-641C17.2 | -0.670626195 | 0.731707972 | | down |
| RP11-152H18.4 | -0.670626194 | 0.731244003 | | down |
| RP11-507M3.1 | -0.670626192 | 0.729935922 | | down |
| RP11-459D22.1 | -0.670626132 | 0.676180706 | | down |
| RP11-665C16.5 | -0.670626109 | 0.64462073 | | down |
| SPATA21 | -0.670530066 | 0.294415402 | 0.431820754 | down |
| RP1-278C19.8 | -0.670525945 | 0.20227922 | 0.331548111 | down |
| AL133493.2 | -0.670393621 | 0.169556151 | 0.293184904 | down |
| 9-Mar | -0.670381937 | 5.51E-05 | 0.000578638 | down |
| CTD-2033A16.3 | -0.670320995 | 0.261029802 | 0.396242369 | down |
| DNAJC5G | -0.670147169 | 0.535703105 | 0.65829885 | down |
| ZGLP1 | -0.670059226 | 0.06188386 | 0.14300736 | down |
| SFXN1 | -0.670009827 | 4.62E-06 | 7.43E-05 | down |
| NA | -0.669919761 | 0.382909141 | 0.520666315 | down |
| SSC4D | -0.669472791 | 0.110712725 | 0.217348449 | down |
| RTP1 | -0.669461191 | 0.733156961 | | down |
| HIST1H3I | -0.669345754 | 0.257985019 | 0.393026251 | down |
| HAND2-AS1 | -0.669228493 | 0.048716628 | 0.120138092 | down |
| RP11-30506.4 | -0.669171609 | 0.069607675 | 0.155729614 | down |
| ITGA3 | -0.669068809 | 0.002276938 | 0.011675767 | down |
| POLD2 | -0.668797736 | 0.002037083 | 0.010654968 | down |
| ATP2A3 | -0.668755392 | 0.015588076 | 0.051458978 | down |
| RP11-498C9.15 | -0.668737786 | 0.010696818 | 0.038629963 | down |
| RP11-511B23.1 | -0.668691252 | 0.200908917 | 0.32995299 | down |
| FAM179A | -0.668686733 | 0.056958957 | 0.134523614 | down |
| RECQL4 | -0.668560399 | 0.018556408 | 0.058729169 | down |
| UXT-AS1 | -0.668414373 | 0.120987409 | 0.231478183 | down |
| RP11-533E19.5 | -0.668395458 | 0.106608338 | 0.211610788 | down |
| RP11-288L9.4 | -0.66824384 | 0.348430898 | 0.487144953 | down |

| | | | | |
|---|---|---|---|---|
| RAC3 | -0.668243201 | 0.000861218 | 0.005374832 | down |
| TDRD6 | -0.668240234 | 0.041664763 | 0.107180723 | down |
| ASL | -0.668142054 | 0.000599764 | 0.004031703 | down |
| CTC-435M10.12 | -0.668126854 | 0.103950335 | 0.207587742 | down |
| MIR559 | -0.667842764 | 0.470621668 | 0.601214278 | down |
| MAPK13 | -0.667786529 | 0.001776728 | 0.009547491 | down |
| NA | -0.667368568 | 0.614450161 | | down |
| FAM229A | -0.667305798 | 0.015453237 | 0.051120573 | down |
| RP11-543C4.1 | -0.66715579 | 0.102807419 | 0.206012545 | down |
| ZNF257 | -0.666940316 | 0.087370749 | 0.183625772 | down |
| DNAJC6 | -0.666819873 | 0.118212127 | 0.227636557 | down |
| ZBTB3 | -0.666686894 | 0.000659667 | 0.004347236 | down |
| CAPNS1 | -0.666546031 | 0.000576359 | 0.003903544 | down |
| C10orf105 | -0.666491525 | 0.164449284 | 0.286913568 | down |
| SESN2 | -0.666375137 | 0.000438721 | 0.003133093 | down |
| RP11-705O1.8 | -0.666164558 | 0.184839314 | 0.310890449 | down |
| ZNHIT1 | -0.665987731 | 0.001998534 | 0.010482533 | down |
| RP11-266L9.5 | -0.665876409 | 0.0103679 | 0.037706476 | down |
| RP11-387H17.4 | -0.665828092 | 0.160213858 | 0.281522117 | down |
| RP1-111D6.3 | -0.665765047 | 0.372607279 | | down |
| ECH1 | -0.665527289 | 0.001319787 | 0.007544077 | down |
| RPL5P30 | -0.66547714 | 0.281745645 | 0.418207057 | down |
| AC013275.2 | -0.665367464 | 0.390162633 | | down |
| AP004782.1 | -0.665251419 | 0.671693069 | | down |
| C9orf131 | -0.665191875 | 0.456274261 | | down |
| PIGN | -0.665060733 | 0.000238566 | 0.001935063 | down |
| LINC00858 | -0.664884906 | 0.641616294 | | down |
| ACOD1 | -0.664883589 | 0.477785638 | 0.607476308 | down |
| FXYD2 | -0.664848666 | 0.201099632 | 0.330137006 | down |
| EPHX1 | -0.664783959 | 0.008139257 | 0.03133468 | down |
| BAG1 | -0.664386545 | 3.77E-05 | 0.000424344 | down |
| RP11-33I11.3 | -0.664230481 | 0.386142069 | 0.523805866 | down |
| RP11-301L8.2 | -0.664183812 | 0.388317146 | 0.525972694 | down |
| LPCAT3 | -0.664124895 | 0.164156631 | 0.286539729 | down |
| CNTN3 | -0.66403486 | 0.183471926 | 0.309210881 | down |
| ACTG1P15 | -0.663883002 | 0.452360896 | 0.585076015 | down |
| C10orf91 | -0.663811216 | 0.100790425 | 0.203123572 | down |
| MYL5 | -0.663804667 | 0.000142828 | 0.001270693 | down |
| Y_RNA | -0.663804107 | 0.661824361 | 0.763383541 | down |
| ALDH16A1 | -0.663705935 | 0.005201683 | 0.022351168 | down |
| LNX2 | -0.663582602 | 0.000970156 | 0.005906407 | down |
| PPP2R1A | -0.663459711 | 0.000352537 | 0.002653878 | down |
| LINC01089 | -0.663421084 | 0.026696728 | 0.076961199 | down |
| B3GALT4 | -0.663383142 | 0.001437378 | 0.008064346 | down |
| SGSM1 | -0.663287995 | 0.036959813 | 0.097868907 | down |
| STON1-GTF2A1L | -0.663200892 | 0.469589154 | 0.600321504 | down |
| RP11-220H4.2 | -0.662929835 | 0.71899488 | | down |
| COQ6 | -0.662908073 | 0.004665072 | 0.020566021 | down |
| ACTG1P24 | -0.662732781 | 0.410916147 | 0.547742346 | down |
| ZNF610 | -0.662662704 | 0.041830848 | 0.107516983 | down |
| RP11-760H22.2 | -0.662632631 | 0.018161083 | 0.057774189 | down |

| | | | | |
|---|---|---|---|---|
| RP11-75A9.3 | -0.662462984 | 0.428573028 | 0.563922746 | down |
| CTC-467M3.1 | -0.662454985 | 0.256170808 | 0.391196287 | down |
| RP11-617F23.1 | -0.662123815 | 0.003984532 | 0.018182153 | down |
| NUS1P1 | -0.662059472 | 0.282332975 | 0.418863854 | down |
| NPM1P49 | -0.661917935 | 0.735509677 | | down |
| RP11-950C14.10 | -0.661866033 | 0.299236628 | 0.436806431 | down |
| TRIM58 | -0.661817757 | 0.029876292 | 0.083529064 | down |
| CTD-2175A23.1 | -0.661697894 | 0.538918968 | | down |
| METRNL | -0.661668777 | 0.007941663 | 0.030787008 | down |
| PIP5K1B | -0.661640578 | 0.013349137 | 0.045652986 | down |
| HHAT | -0.661500966 | 0.00715914 | 0.02845848 | down |
| RP11-348N5.9 | -0.661420093 | 0.141725437 | 0.258438897 | down |
| MIR199A1 | -0.661270501 | 0.49916428 | | down |
| KRT19 | -0.66126763 | 0.061542953 | 0.142449873 | down |
| PIDD1 | -0.661234864 | 0.001414932 | 0.007963697 | down |
| AC010729.2 | -0.661217382 | 0.821207833 | | down |
| RP11-366M4.18 | -0.661217382 | 0.821207833 | | down |
| RP11-631F7.2 | -0.661217382 | 0.821207833 | | down |
| OR51Q1 | -0.661217382 | 0.821207833 | | down |
| RP3-333B15.5 | -0.661217382 | 0.821207833 | | down |
| RP1-257I9.2 | -0.661217364 | 0.814835644 | | down |
| AC092295.4 | -0.661217364 | 0.814686558 | | down |
| RNU1-129P | -0.661217361 | 0.813681106 | | down |
| IAPP | -0.661217358 | 0.812478967 | | down |
| RP11-287D1.3 | -0.661217309 | 0.789984373 | | down |
| MTCO1P15 | -0.661217305 | 0.787793625 | | down |
| NA | -0.661217268 | 0.762514516 | | down |
| RP11-468N14.5 | -0.661217268 | 0.762778543 | | down |
| RP11-512N21.3 | -0.661217265 | 0.760762667 | | down |
| POLR3GP1 | -0.66121724 | 0.736861356 | | down |
| Metazoa_SRP | -0.661217238 | 0.734734948 | | down |
| RP1-102E24.9 | -0.661217238 | 0.734231742 | | down |
| MIR203A | -0.661217232 | 0.727567777 | | down |
| uc_338 | -0.661217219 | 0.710820663 | | down |
| FDPSP4 | -0.661217214 | 0.703397833 | | down |
| RP4-545L17.12 | -0.661217202 | 0.68356345 | | down |
| AC007000.10 | -0.661217201 | 0.680824966 | | down |
| RP4-704D23.1 | -0.6612172 | 0.678193898 | | down |
| Metazoa_SRP | -0.661217188 | 0.652245155 | | down |
| PABPN1L | -0.661084585 | 0.278077525 | 0.414314973 | down |
| ZNF214 | -0.660898582 | 0.080509111 | 0.173007348 | down |
| AC093627.12 | -0.660868961 | 0.642540176 | | down |
| RP11-511B23.2 | -0.660646164 | 0.214679426 | 0.345705563 | down |
| RP11-416N2.3 | -0.660518987 | 0.583714456 | 0.699581823 | down |
| RP11-331F4.4 | -0.660293805 | 0.391931963 | 0.529484415 | down |
| NA | -0.660058136 | 0.085886414 | 0.18149901 | down |
| HIGD1A | -0.659964744 | 0.004559825 | 0.020208787 | down |
| FUK | -0.659828106 | 8.03E-05 | 0.000788228 | down |
| RP1-137D17.2 | -0.659688577 | 0.058006885 | 0.136387451 | down |
| ANGPTL7 | -0.659665396 | 0.134953613 | 0.249633441 | down |
| PAAF1 | -0.659097114 | 0.000119736 | 0.001101116 | down |

| | | | | |
|---|---|---|---|---|
| ARHGEF38 | -0.659060743 | 0.056995954 | 0.134589832 | down |
| RP11-211N11.5 | -0.659057342 | 0.154053889 | 0.273773322 | down |
| CTC-548K16.6 | -0.659040211 | 0.253138346 | 0.388090903 | down |
| TLL2 | -0.658901871 | 0.02796638 | 0.079755284 | down |
| RPL12P10 | -0.658869854 | 0.364342295 | 0.50270034 | down |
| KRT17P8 | -0.658850449 | 0.535267059 | | down |
| MAP2K5 | -0.6588398 | 0.000642662 | 0.004255704 | down |
| HIST1H2BI | -0.658560179 | 0.159491854 | 0.280663813 | down |
| LINC00638 | -0.658511906 | 0.065368239 | 0.148579466 | down |
| GPR150 | -0.658485419 | 0.114906028 | 0.223122758 | down |
| DBIL5P2 | -0.658441688 | 0.657487314 | | down |
| C1QTNF4 | -0.658388064 | 0.049033736 | 0.120639594 | down |
| L22NC03-N14H11. | -0.658378869 | 0.257551003 | 0.392640671 | down |
| RP11-242C19.2 | -0.658276082 | 0.255438092 | 0.390537455 | down |
| NA | -0.658152729 | 0.353809937 | 0.49254393 | down |
| VAMP2 | -0.658149337 | 0.000459089 | 0.003249966 | down |
| CATSPERB | -0.658006094 | 0.038586181 | 0.101114979 | down |
| RPL3P7 | -0.657848316 | 0.037152222 | 0.098283116 | down |
| ADIPOQ | -0.657750748 | 0.240519255 | 0.374276714 | down |
| TOX3 | -0.657742632 | 0.015205681 | 0.050446056 | down |
| GPX4 | -0.657618615 | 0.001091498 | 0.006509124 | down |
| NRK | -0.657349505 | 0.201412254 | 0.330545525 | down |
| DHRS7 | -0.657326514 | 0.02135919 | 0.065142169 | down |
| HIF1A-AS2 | -0.657323891 | 0.144172956 | 0.261692381 | down |
| RP5-965G21.6 | -0.657118522 | 0.102667428 | 0.20582812 | down |
| RP11-271K11.5 | -0.656800812 | 0.271157613 | 0.407251515 | down |
| AC005532.5 | -0.656768889 | 0.220654686 | 0.352306392 | down |
| RAB25 | -0.656744268 | 0.028209993 | 0.080251966 | down |
| RP11-1109F11.3 | -0.656667454 | 0.10715303 | 0.212383273 | down |
| LRRTM1 | -0.65664598 | 0.181373953 | 0.306858826 | down |
| SORT1 | -0.656499594 | 0.002279124 | 0.011680994 | down |
| NBR2 | -0.656464282 | 0.000321029 | 0.002462479 | down |
| ACTG1P17 | -0.656445906 | 0.160665745 | 0.282134646 | down |
| DOLPP1 | -0.656215664 | 0.002250303 | 0.011551018 | down |
| RP11-12J10.3 | -0.656008636 | 0.331765515 | 0.470133003 | down |
| AGBL5-IT1 | -0.65598769 | 0.020368575 | 0.062958176 | down |
| REEP1 | -0.655920502 | 0.094730337 | 0.194510542 | down |
| CENPE | -0.655798421 | 0.062687651 | 0.144343025 | down |
| RP11-43303.1 | -0.655796227 | 0.236209516 | 0.369176439 | down |
| NA | -0.655726378 | 0.322871002 | 0.46143737 | down |
| NPIPB7 | -0.655498971 | 0.385906936 | 0.523620573 | down |
| RP11-96L14.7 | -0.65540776 | 0.316316735 | 0.454907178 | down |
| KRT42P | -0.655390665 | 0.257997062 | 0.393026251 | down |
| Y_RNA | -0.655383186 | 0.482409192 | | down |
| IQCH-AS1 | -0.655365 | 0.005415253 | 0.023074155 | down |
| RP11-437B10.1 | -0.655171773 | 0.108893818 | 0.2150109 | down |
| HEXA | -0.655109077 | 0.009576631 | 0.035451447 | down |
| RP3-467D16.3 | -0.655087725 | 0.569117312 | | down |
| AC012358.8 | -0.655087515 | 0.077052286 | 0.167568504 | down |
| ZNF350-AS1 | -0.65505141 | 0.129529107 | 0.242601559 | down |
| DNLZ | -0.654825345 | 0.122265273 | 0.233109397 | down |

| | | | | |
|---|---|---|---|---|
| CALY | -0.654817189 | 0.076968732 | 0.167459526 | down |
| RP11-445N20.2 | -0.654633711 | 0.229380276 | 0.361844827 | down |
| Metazoa_SRP | -0.654380419 | 0.084306911 | 0.178840418 | down |
| CTD-2184D3.1 | -0.654039894 | 0.157155845 | 0.277847251 | down |
| REEP2 | -0.653878034 | 0.01515306 | 0.050321922 | down |
| PSMA6P1 | -0.653615777 | 0.144629988 | 0.262173486 | down |
| bP-21264C1.2 | -0.653601444 | 0.130460784 | 0.243960603 | down |
| CHADL | -0.653433363 | 0.018257405 | 0.058008192 | down |
| SPANXB1 | -0.653356208 | 0.785364465 | | down |
| CTC-492K19.4 | -0.653193583 | 0.206522277 | 0.336574738 | down |
| RPLP1P6 | -0.653163402 | 0.04281381 | 0.109324226 | down |
| NA | -0.653068903 | 0.22848451 | 0.360821203 | down |
| NA | -0.65293683 | 0.365215381 | 0.503627435 | down |
| RPP21 | -0.652900193 | 0.044578411 | 0.112433997 | down |
| ETNK1 | -0.652826218 | 6.22E-05 | 0.000639618 | down |
| MTND4P12 | -0.652793753 | 0.143501541 | 0.260773018 | down |
| C19orf25 | -0.652341948 | 0.000625873 | 0.00417394 | down |
| RP11-227G15.11 | -0.652308414 | 0.162976471 | 0.285059387 | down |
| RP11-68I3.10 | -0.65224333 | 0.550445931 | | down |
| GCK | -0.652043355 | 0.110572773 | 0.217184127 | down |
| RPS10P3 | -0.652039629 | 0.050411211 | 0.123044474 | down |
| AC074117.13 | -0.652021415 | 0.448862414 | | down |
| RP11-214K3.19 | -0.652013808 | 0.280132551 | 0.416463079 | down |
| UPK3BP1 | -0.651837436 | 0.522654123 | | down |
| RNA5SP35 | -0.651808291 | 0.761648891 | | down |
| RP11-317B17.4 | -0.651808282 | 0.737300635 | | down |
| CTC-312O10.3 | -0.651808282 | 0.738003276 | | down |
| HSFY3P | -0.651808282 | 0.737473311 | | down |
| FAM224B | -0.651808282 | 0.738149833 | | down |
| AL121656.5 | -0.651808281 | 0.734863236 | | down |
| HLA-T | -0.651808275 | 0.713268737 | | down |
| CTD-2313J17.1 | -0.651808275 | 0.711238001 | | down |
| RP11-1396O13.2 | -0.651808273 | 0.705098266 | | down |
| RP5-1029F21.4 | -0.651808273 | 0.706615446 | | down |
| RP13-88F20.1 | -0.651808269 | 0.683628053 | | down |
| RP11-642C5.1 | -0.651808265 | 0.658857859 | | down |
| RP11-90H3.1 | -0.651808262 | 0.633275037 | | down |
| RPS3AP43 | -0.651808261 | 0.626429941 | | down |
| ZNF66 | -0.651754512 | 0.081489825 | 0.174515669 | down |
| RP11-293M10.2 | -0.651603009 | 0.395504091 | 0.532849513 | down |
| NA | -0.651549979 | 0.556991436 | | down |
| BCL7C | -0.651544875 | 0.007047594 | 0.028134192 | down |
| INAFM1 | -0.651359996 | 0.012706456 | 0.044031461 | down |
| SETD9 | -0.651345503 | 0.005971138 | 0.024860985 | down |
| RASL10B | -0.651308581 | 0.070875971 | 0.157721478 | down |
| BSN-AS2 | -0.651284786 | 0.565203936 | | down |
| SNAP91 | -0.651213475 | 0.146615655 | 0.264819693 | down |
| NA | -0.650827688 | 0.56087525 | | down |
| SCARNA5 | -0.650822939 | 0.472087022 | 0.602522621 | down |
| PDDC1 | -0.650770627 | 0.000205831 | 0.001712112 | down |
| ARMC5 | -0.650633557 | 0.003605898 | 0.01676762 | down |

| | | | | |
|---|---|---|---|---|
| RP11-430K21.2 | -0.650628174 | 0.21308615 | 0.343932539 | down |
| ACVR2B-AS1 | -0.650591058 | 0.079540807 | 0.171426517 | down |
| NA | -0.650580986 | 0.616663728 | | down |
| AC128709.3 | -0.650579995 | 0.698081118 | | down |
| TMIE | -0.650527181 | 0.026439343 | 0.076430638 | down |
| RPS12P27 | -0.650506359 | 0.49190205 | | down |
| ICOSLG | -0.650187818 | 0.117290833 | 0.226372058 | down |
| ADAMTSL5 | -0.650167704 | 0.005281462 | 0.022635594 | down |
| KB-1125A3.11 | -0.649920575 | 0.479057056 | | down |
| RP11-247A12.2 | -0.649697796 | 0.138012999 | 0.253774895 | down |
| AC006273.4 | -0.649625545 | 0.326149778 | 0.464569556 | down |
| LGALS3BP | -0.649517663 | 0.006548009 | 0.026647742 | down |
| AC005618.6 | -0.649354448 | 0.175166599 | 0.299664909 | down |
| FAM78B | -0.649261797 | 0.009683321 | 0.035740258 | down |
| PSMC1P9 | -0.649115427 | 0.342230139 | | down |
| MED12L | -0.648722131 | 0.115017511 | 0.223243497 | down |
| DBP | -0.648597134 | 0.003809076 | 0.017525251 | down |
| FABP5P11 | -0.648452615 | 0.448319262 | 0.581400436 | down |
| KRT36 | -0.648385406 | 0.211032918 | 0.341574463 | down |
| HMGB1P8 | -0.64835948 | 0.335736227 | 0.473917459 | down |
| RP11-57H12.3 | -0.648354866 | 0.208375518 | 0.338579107 | down |
| AQP4 | -0.648269704 | 0.440067553 | | down |
| SEPT14P4 | -0.648121838 | 0.571931037 | | down |
| RP3-370M22.8 | -0.648032274 | 0.104012763 | 0.207624705 | down |
| KLF3-AS1 | -0.648022956 | 0.006246286 | 0.02572396 | down |
| KRTAP5-8 | -0.647911533 | 0.189083383 | 0.316214803 | down |
| DCTPP1 | -0.647911171 | 0.005055529 | 0.021882771 | down |
| DUSP23 | -0.647875712 | 0.004049363 | 0.018427607 | down |
| RP11-220I1.5 | -0.647730996 | 0.416823408 | 0.553015786 | down |
| CTC-205M6.1 | -0.647657056 | 0.087609053 | 0.18394901 | down |
| TRPM2 | -0.647552992 | 0.005526541 | 0.023408911 | down |
| RP1-90G24.10 | -0.64723356 | 0.244761081 | 0.378770734 | down |
| RP11-650L12.4 | -0.647165415 | 0.475489677 | | down |
| SGF29 | -0.646791289 | 0.001943553 | 0.010235252 | down |
| RP11-425L10.1 | -0.646726583 | 0.007669722 | 0.030007701 | down |
| THRB-AS1 | -0.646705708 | 0.326424791 | 0.464791774 | down |
| NABP1 | -0.64662671 | 0.004782863 | 0.02098063 | down |
| RP11-388C12.8 | -0.646599116 | 0.130916893 | 0.244496043 | down |
| Xbac-BPG248L24.1 | -0.646540221 | 0.664438013 | 0.765516572 | down |
| THRB | -0.646457478 | 0.015246519 | 0.050548049 | down |
| TRAPPC9 | -0.646455634 | 4.60E-05 | 0.0004989 | down |
| WDFY3-AS2 | -0.64633277 | 0.076643524 | 0.166981729 | down |
| VAV3 | -0.646021917 | 0.000948592 | 0.005806742 | down |
| GABPB2 | -0.646017126 | 2.39E-05 | 0.000289765 | down |
| RP5-884G6.2 | -0.645822924 | 0.226488889 | 0.358610392 | down |
| RP11-71E19.2 | -0.645798833 | 0.609457205 | | down |
| RP11-211N8.2 | -0.645651014 | 0.174330919 | 0.298823934 | down |
| PRAMEF11 | -0.645537547 | 0.825378453 | | down |
| RP4-597A16.2 | -0.645537547 | 0.825378453 | | down |
| RP11-380J14.4 | -0.645537547 | 0.825378453 | | down |
| DYNLL1P3 | -0.645537547 | 0.825378453 | | down |

| | | | |
|---|---|---|---|
| RNU4-28P | -0.645537547 | 0.825378453 | down |
| RPS4XP4 | -0.645537547 | 0.825378453 | down |
| RP5-891H21.1 | -0.645537547 | 0.825378453 | down |
| HMGB1P18 | -0.645537547 | 0.825378453 | down |
| RP11-14O19.2 | -0.645537547 | 0.825378453 | down |
| CH17-333M13.1 | -0.645537547 | 0.825378453 | down |
| RP11-666A1.4 | -0.645537547 | 0.825378453 | down |
| RP1-20N18.10 | -0.645537547 | 0.825378453 | down |
| MIR921 | -0.645537547 | 0.825378453 | down |
| MIR557 | -0.645537547 | 0.825378453 | down |
| SLC4A1APP2 | -0.645537547 | 0.825378453 | down |
| RP11-435P24.2 | -0.645537547 | 0.825378453 | down |
| AC011995.1 | -0.645537547 | 0.825378453 | down |
| RN7SL66P | -0.645537547 | 0.825378453 | down |
| AC104698.2 | -0.645537547 | 0.825378453 | down |
| KRT18P52 | -0.645537547 | 0.825378453 | down |
| RNU4-63P | -0.645537547 | 0.825378453 | down |
| CTBP2P5 | -0.645537547 | 0.825378453 | down |
| AC011306.1 | -0.645537547 | 0.825378453 | down |
| Metazoa_SRP | -0.645537547 | 0.825378453 | down |
| AC005034.2 | -0.645537547 | 0.825378453 | down |
| AC010975.1 | -0.645537547 | 0.825378453 | down |
| Y_RNA | -0.645537547 | 0.825378453 | down |
| NDUFB4P7 | -0.645537547 | 0.825378453 | down |
| MTCO1P7 | -0.645537547 | 0.825378453 | down |
| AC097532.2 | -0.645537547 | 0.825378453 | down |
| NA | -0.645537547 | 0.825378453 | down |
| AC019178.2 | -0.645537547 | 0.825378453 | down |
| PRLH | -0.645537547 | 0.825378453 | down |
| CRB3P1 | -0.645537547 | 0.825378453 | down |
| DLEC1P1 | -0.645537547 | 0.825378453 | down |
| RP11-272D20.2 | -0.645537547 | 0.825378453 | down |
| RNA5SP136 | -0.645537547 | 0.825378453 | down |
| Metazoa_SRP | -0.645537547 | 0.825378453 | down |
| RP11-413E6.2 | -0.645537547 | 0.825378453 | down |
| EIF4E2P2 | -0.645537547 | 0.825378453 | down |
| OR7E93P | -0.645537547 | 0.825378453 | down |
| RPS27P12 | -0.645537547 | 0.825378453 | down |
| RPL23AP41 | -0.645537547 | 0.825378453 | down |
| NAALADL2-AS3 | -0.645537547 | 0.825378453 | down |
| RP11-332P22.2 | -0.645537547 | 0.825378453 | down |
| SNRPCP16 | -0.645537547 | 0.825378453 | down |
| RNU1-49P | -0.645537547 | 0.825378453 | down |
| RP11-545H22.1 | -0.645537547 | 0.825378453 | down |
| RP11-468N14.12 | -0.645537547 | 0.825378453 | down |
| UGT2A3P7 | -0.645537547 | 0.825378453 | down |
| RP11-119H12.3 | -0.645537547 | 0.825378453 | down |
| RP11-521E5.1 | -0.645537547 | 0.825378453 | down |
| MTATP6P9 | -0.645537547 | 0.825378453 | down |
| NA | -0.645537547 | 0.825378453 | down |
| MIR4457 | -0.645537547 | 0.825378453 | down |

| | | | |
|---|---|---|---|
| RP11-315A16.2 | -0.645537547 | 0.825378453 | down |
| MIR6131 | -0.645537547 | 0.825378453 | down |
| NA | -0.645537547 | 0.825378453 | down |
| AC063979.1 | -0.645537547 | 0.825378453 | down |
| RPS27AP9 | -0.645537547 | 0.825378453 | down |
| RN7SKP34 | -0.645537547 | 0.825378453 | down |
| RP11-155G15.3 | -0.645537547 | 0.825378453 | down |
| CTC-276P9.3 | -0.645537547 | 0.825378453 | down |
| NA | -0.645537547 | 0.825378453 | down |
| CTB-7E3.1 | -0.645537547 | 0.825378453 | down |
| RP5-1077H22.2 | -0.645537547 | 0.825378453 | down |
| RP11-556O15.1 | -0.645537547 | 0.825378453 | down |
| ZDHHC20P2 | -0.645537547 | 0.825378453 | down |
| RP11-121P10.1 | -0.645537547 | 0.825378453 | down |
| RPS6P8 | -0.645537547 | 0.825378453 | down |
| MTHFD2P2 | -0.645537547 | 0.825378453 | down |
| NA | -0.645537547 | 0.825378453 | down |
| RP11-346D19.1 | -0.645537547 | 0.825378453 | down |
| RP11-129H15.4 | -0.645537547 | 0.825378453 | down |
| RP11-753G20.2 | -0.645537547 | 0.825378453 | down |
| TAAR1 | -0.645537547 | 0.825378453 | down |
| RNA5SP223 | -0.645537547 | 0.825378453 | down |
| RP11-196O2.1 | -0.645537547 | 0.825378453 | down |
| Y_RNA | -0.645537547 | 0.825378453 | down |
| NMD3P2 | -0.645537547 | 0.825378453 | down |
| MTND5P6 | -0.645537547 | 0.825378453 | down |
| MTND1P4 | -0.645537547 | 0.825378453 | down |
| RP11-368M16.9 | -0.645537547 | 0.825378453 | down |
| RP11-548K12.2 | -0.645537547 | 0.825378453 | down |
| VN1R31P | -0.645537547 | 0.825378453 | down |
| NA | -0.645537547 | 0.825378453 | down |
| AC004878.8 | -0.645537547 | 0.825378453 | down |
| Y_RNA | -0.645537547 | 0.825378453 | down |
| AC004006.2 | -0.645537547 | 0.825378453 | down |
| RP4-742N3.1 | -0.645537547 | 0.825378453 | down |
| NA | -0.645537547 | 0.825378453 | down |
| RP11-29A2.1 | -0.645537547 | 0.825378453 | down |
| DEFB134 | -0.645537547 | 0.825378453 | down |
| RP11-618M23.3 | -0.645537547 | 0.825378453 | down |
| RP11-24P4.1 | -0.645537547 | 0.825378453 | down |
| KB-1582A10.1 | -0.645537547 | 0.825378453 | down |
| RP11-546K22.2 | -0.645537547 | 0.825378453 | down |
| RNA5SP267 | -0.645537547 | 0.825378453 | down |
| Metazoa_SRP | -0.645537547 | 0.825378453 | down |
| RN7SL350P | -0.645537547 | 0.825378453 | down |
| RPL12P24 | -0.645537547 | 0.825378453 | down |
| RP11-3G20.1 | -0.645537547 | 0.825378453 | down |
| ZYXP1 | -0.645537547 | 0.825378453 | down |
| CTD-3145H4.1 | -0.645537547 | 0.825378453 | down |
| RP11-59O6.3 | -0.645537547 | 0.825378453 | down |
| RP11-132E11.4 | -0.645537547 | 0.825378453 | down |

| | | | |
|---|---|---|---|
| MIR876 | -0.645537547 | 0.825378453 | down |
| CNTNAP3P5 | -0.645537547 | 0.825378453 | down |
| NA | -0.645537547 | 0.825378453 | down |
| NA | -0.645537547 | 0.825378453 | down |
| PCNPP2 | -0.645537547 | 0.825378453 | down |
| RNU6-669P | -0.645537547 | 0.825378453 | down |
| FYTTD1P1 | -0.645537547 | 0.825378453 | down |
| ARL2BPP7 | -0.645537547 | 0.825378453 | down |
| MIR3154 | -0.645537547 | 0.825378453 | down |
| FXYD6P2 | -0.645537547 | 0.825378453 | down |
| RP11-733D4.1 | -0.645537547 | 0.825378453 | down |
| RPL23AP61 | -0.645537547 | 0.825378453 | down |
| CH17-335B8.4 | -0.645537547 | 0.825378453 | down |
| RPL17P35 | -0.645537547 | 0.825378453 | down |
| RPA2P2 | -0.645537547 | 0.825378453 | down |
| HBG2 | -0.645537547 | 0.825378453 | down |
| OR51A10P | -0.645537547 | 0.825378453 | down |
| OR52H2P | -0.645537547 | 0.825378453 | down |
| MIR4299 | -0.645537547 | 0.825378453 | down |
| NA | -0.645537547 | 0.825378453 | down |
| RP11-646J21.6 | -0.645537547 | 0.825378453 | down |
| RP11-958J22.3 | -0.645537547 | 0.825378453 | down |
| OR4C9P | -0.645537547 | 0.825378453 | down |
| OR4A11P | -0.645537547 | 0.825378453 | down |
| OR4A15 | -0.645537547 | 0.825378453 | down |
| RP11-674C21.9 | -0.645537547 | 0.825378453 | down |
| OR5M10 | -0.645537547 | 0.825378453 | down |
| OR9G3P | -0.645537547 | 0.825378453 | down |
| EEF1G | -0.645537547 | 0.825378453 | down |
| RNU2-23P | -0.645537547 | 0.825378453 | down |
| Metazoa_SRP | -0.645537547 | 0.825378453 | down |
| CTD-2564P9.3 | -0.645537547 | 0.825378453 | down |
| NA | -0.645537547 | 0.825378453 | down |
| Y_RNA | -0.645537547 | 0.825378453 | down |
| OR8D2 | -0.645537547 | 0.825378453 | down |
| Y_RNA | -0.645537547 | 0.825378453 | down |
| AP002856.4 | -0.645537547 | 0.825378453 | down |
| RNU6-485P | -0.645537547 | 0.825378453 | down |
| NDFIP1P1 | -0.645537547 | 0.825378453 | down |
| KRT126P | -0.645537547 | 0.825378453 | down |
| OR9R1P | -0.645537547 | 0.825378453 | down |
| KRT8P19 | -0.645537547 | 0.825378453 | down |
| HNRNPA1P69 | -0.645537547 | 0.825378453 | down |
| RP11-397H6.1 | -0.645537547 | 0.825378453 | down |
| RP11-100F15.2 | -0.645537547 | 0.825378453 | down |
| RP11-121J20.1 | -0.645537547 | 0.825378453 | down |
| RP11-983C2.3 | -0.645537547 | 0.825378453 | down |
| RP13-895J2.8 | -0.645537547 | 0.825378453 | down |
| RP11-165I9.10 | -0.645537547 | 0.825378453 | down |
| RNA5SP29 | -0.645537547 | 0.825378453 | down |
| OR7E33P | -0.645537547 | 0.825378453 | down |

| | | | |
|---|---|---|---|
| RPL35AP31 | -0.645537547 | 0.825378453 | down |
| PSMD10P3 | -0.645537547 | 0.825378453 | down |
| RN7SL571P | -0.645537547 | 0.825378453 | down |
| LINC00560 | -0.645537547 | 0.825378453 | down |
| PSMA6P4 | -0.645537547 | 0.825378453 | down |
| ARHGEF7-AS1 | -0.645537547 | 0.825378453 | down |
| 5_8S_rRNA | -0.645537547 | 0.825378453 | down |
| OR4K17 | -0.645537547 | 0.825378453 | down |
| Y_RNA | -0.645537547 | 0.825378453 | down |
| RP4-687K1.2 | -0.645537547 | 0.825378453 | down |
| NDUFB8P1 | -0.645537547 | 0.825378453 | down |
| NA | -0.645537547 | 0.825378453 | down |
| RP11-594C13.1 | -0.645537547 | 0.825378453 | down |
| RP11-1082A3.1 | -0.645537547 | 0.825378453 | down |
| MIR487B | -0.645537547 | 0.825378453 | down |
| Y_RNA | -0.645537547 | 0.825378453 | down |
| RP11-417P24.1 | -0.645537547 | 0.825378453 | down |
| NA | -0.645537547 | 0.825378453 | down |
| RNU4-80P | -0.645537547 | 0.825378453 | down |
| CTD-2071N1.1 | -0.645537547 | 0.825378453 | down |
| PGAM1P12 | -0.645537547 | 0.825378453 | down |
| MIR193B | -0.645537547 | 0.825378453 | down |
| SNORA75 | -0.645537547 | 0.825378453 | down |
| AGGF1P9 | -0.645537547 | 0.825378453 | down |
| RARRES2P8 | -0.645537547 | 0.825378453 | down |
| RPL34P29 | -0.645537547 | 0.825378453 | down |
| NA | -0.645537547 | 0.825378453 | down |
| CTD-3032H12.2 | -0.645537547 | 0.825378453 | down |
| RP11-439I14.3 | -0.645537547 | 0.825378453 | down |
| RP11-319G9.4 | -0.645537547 | 0.825378453 | down |
| Y_RNA | -0.645537547 | 0.825378453 | down |
| AC005722.4 | -0.645537547 | 0.825378453 | down |
| RP11-744K17.1 | -0.645537547 | 0.825378453 | down |
| MTATP6P3 | -0.645537547 | 0.825378453 | down |
| CTD-2008P7.5 | -0.645537547 | 0.825378453 | down |
| OOSP1P2 | -0.645537547 | 0.825378453 | down |
| TBC1D3H | -0.645537547 | 0.825378453 | down |
| MIR454 | -0.645537547 | 0.825378453 | down |
| RP11-3K24.3 | -0.645537547 | 0.825378453 | down |
| RP11-556O9.4 | -0.645537547 | 0.825378453 | down |
| Y_RNA | -0.645537547 | 0.825378453 | down |
| RP11-64C12.4 | -0.645537547 | 0.825378453 | down |
| CTD-2515C13.1 | -0.645537547 | 0.825378453 | down |
| OR7A10 | -0.645537547 | 0.825378453 | down |
| CTC-400I9.2 | -0.645537547 | 0.825378453 | down |
| AC011525.2 | -0.645537547 | 0.825378453 | down |
| RPL31P60 | -0.645537547 | 0.825378453 | down |
| CTC-339O9.2 | -0.645537547 | 0.825378453 | down |
| MIR498 | -0.645537547 | 0.825378453 | down |
| RNU6-27P | -0.645537547 | 0.825378453 | down |
| NA | -0.645537547 | 0.825378453 | down |

| | | | | |
|---|---|---|---|---|
| RP11-410N8.4 | -0.645537547 | 0.825378453 | | down |
| MIR548O2 | -0.645537547 | 0.825378453 | | down |
| RP11-101E14.3 | -0.645537547 | 0.825378453 | | down |
| MIR4325 | -0.645537547 | 0.825378453 | | down |
| RNU6-994P | -0.645537547 | 0.825378453 | | down |
| AJ239321.3 | -0.645537547 | 0.825378453 | | down |
| KRTAP23-1 | -0.645537547 | 0.825378453 | | down |
| NA | -0.645537547 | 0.825378453 | | down |
| PABPC1P9 | -0.645537547 | 0.825378453 | | down |
| AC006548.26 | -0.645537547 | 0.825378453 | | down |
| L22NC03-123E1.! | -0.645537547 | 0.825378453 | | down |
| RP1-288L1.4 | -0.645537547 | 0.825378453 | | down |
| RP11-483M24.2 | -0.645537547 | 0.825378453 | | down |
| RNU7-56P | -0.645537547 | 0.825378453 | | down |
| FTH1P14 | -0.645537547 | 0.825378453 | | down |
| SSX4B | -0.645537547 | 0.825378453 | | down |
| RP11-167P23.2 | -0.645537547 | 0.825378453 | | down |
| SERBP1P4 | -0.645537547 | 0.825378453 | | down |
| EEF1A1P15 | -0.645537547 | 0.825378453 | | down |
| MIR652 | -0.645537547 | 0.825378453 | | down |
| HTR2C | -0.645537547 | 0.825378453 | | down |
| RP5-878I13.1 | -0.645537547 | 0.825378453 | | down |
| SPANXC | -0.645537547 | 0.825378453 | | down |
| MIR890 | -0.645537547 | 0.825378453 | | down |
| RN7SKP267 | -0.645537547 | 0.825378453 | | down |
| MAGEA9 | -0.645537547 | 0.825378453 | | down |
| TEX28 | -0.645537547 | 0.825378453 | | down |
| SERBP1P2 | -0.645537547 | 0.825378453 | | down |
| FAM197Y9 | -0.645537547 | 0.825378453 | | down |
| AC007274.5 | -0.645537547 | 0.825378453 | | down |
| FAM197Y8 | -0.645537547 | 0.825378453 | | down |
| FAM197Y3 | -0.645537547 | 0.825378453 | | down |
| TSPY15P | -0.645537547 | 0.825378453 | | down |
| DUX4L19 | -0.645537547 | 0.825378453 | | down |
| MT-TL1 | -0.645537547 | 0.825378453 | | down |
| NA | -0.645521461 | 0.501321386 | 0.628374832 | down |
| OBSCN | -0.645413547 | 0.001361348 | 0.007728712 | down |
| RP11-10A14.6 | -0.645370241 | 0.645561531 | 0.750532105 | down |
| NIM1K | -0.645258469 | 0.051987977 | 0.125667937 | down |
| RPL7AP8 | -0.645187806 | 0.647806441 | 0.752355975 | down |
| RP11-44H4.1 | -0.645149085 | 0.537411085 | 0.659557951 | down |
| RP11-416H1.1 | -0.64499254 | 0.65077507 | 0.754715377 | down |
| FMC1 | -0.64498764 | 0.075131489 | 0.164498648 | down |
| NA | -0.644974255 | 0.13220151 | 0.246192959 | down |
| HSPB1P2 | -0.644924451 | 0.127908633 | 0.240465989 | down |
| RN7SKP230 | -0.64473552 | 0.444822375 | 0.578288335 | down |
| DDR1 | -0.64467236 | 0.009465731 | 0.035150529 | down |
| TMEM187 | -0.644641265 | 0.001471095 | 0.008210583 | down |
| PACS2 | -0.644635277 | 4.22E-05 | 0.00046396 | down |
| SRXN1 | -0.644562888 | 0.306268752 | 0.444647646 | down |
| LINC01569 | -0.644407627 | 0.005148324 | 0.022185346 | down |

| | | | | |
|---|---|---|---|---|
| KRT8P12 | -0.644347259 | 0.007970261 | 0.030885919 | down |
| AC005487.2 | -0.644182724 | 0.629806396 | 0.737954969 | down |
| LINC01475 | -0.644172188 | 0.14093661 | 0.257529468 | down |
| RP3-455J7.4 | -0.644169627 | 0.200444961 | 0.329548453 | down |
| Y_RNA | -0.644094534 | 0.359383809 | 0.497677266 | down |
| CIRBP | -0.643894301 | 0.000739368 | 0.004771983 | down |
| SPDEF | -0.643637418 | 0.055041257 | 0.131176778 | down |
| NA | -0.6435383 | 0.199237509 | 0.328326295 | down |
| RPS3AP6 | -0.643393231 | 0.000183278 | 0.001557241 | down |
| ZNF688 | -0.643300782 | 0.000487651 | 0.003408396 | down |
| RP11-561O23.7 | -0.642976021 | 0.826059889 | | down |
| RP1-107N3.2 | -0.642976021 | 0.826059889 | | down |
| RP11-164A6.1 | -0.642974184 | 0.814849291 | | down |
| RP11-955H22.1 | -0.642969693 | 0.790125776 | | down |
| SPRR2G | -0.642969579 | 0.78953498 | | down |
| MIR4753 | -0.642964781 | 0.766318245 | | down |
| LAMTOR3P2 | -0.642958994 | 0.741371817 | | down |
| RP11-358D17.1 | -0.642952653 | 0.716887262 | | down |
| CTC-268N12.3 | -0.642952011 | 0.714545215 | | down |
| MIR4755 | -0.642951235 | 0.711744393 | | down |
| CTA-292E10.7 | -0.642951074 | 0.711164279 | | down |
| RP11-67L3.2 | -0.642945456 | 0.691831723 | | down |
| CTD-2072I24.1 | -0.642944043 | 0.687196607 | | down |
| PCDHA2 | -0.642936743 | 0.664488325 | | down |
| PYY2 | -0.642935899 | 0.124915575 | 0.236556164 | down |
| CFL1P3 | -0.642935496 | 0.660797908 | | down |
| NA | -0.642926547 | 0.63570445 | | down |
| PLA2G4C-AS1 | -0.642904587 | 0.582286425 | | down |
| CADM2 | -0.642848261 | 0.124305789 | 0.235772925 | down |
| USP5 | -0.642832989 | 0.001259143 | 0.007274981 | down |
| RP11-157E21.1 | -0.642768486 | 0.520811314 | 0.645179946 | down |
| NA | -0.642664827 | 0.815109945 | | down |
| RP11-390G14.1 | -0.642557612 | 0.575121233 | | down |
| RP3-462D8.2 | -0.642549238 | 0.44618224 | 0.579504548 | down |
| MBD3 | -0.642316787 | 0.004805135 | 0.021056809 | down |
| RP11-333J10.3 | -0.642086746 | 0.30948466 | 0.447974423 | down |
| CHIT1 | -0.642078865 | 0.212962994 | 0.343815291 | down |
| ZNF296 | -0.641923841 | 0.008616599 | 0.032704098 | down |
| RP11-324O2.3 | -0.641911715 | 0.095285519 | 0.195223549 | down |
| ALG1L2 | -0.641663942 | 0.03069963 | 0.085267605 | down |
| DNASE2B | -0.64158136 | 0.388151146 | 0.525771552 | down |
| TFDP2 | -0.641568211 | 9.12E-10 | 4.67E-08 | down |
| ENPP7P11 | -0.641564242 | 0.243568773 | 0.377613924 | down |
| RP11-467D18.2 | -0.641504402 | 0.5434905 | | down |
| RP11-460I13.6 | -0.641345761 | 0.313538092 | 0.452142577 | down |
| KBTBD6 | -0.641254544 | 0.00497909 | 0.021626683 | down |
| RLN1 | -0.641212098 | 0.258548272 | 0.393666343 | down |
| FBF1 | -0.641076332 | 0.006163407 | 0.025470892 | down |
| NA | -0.640983775 | 0.21682768 | 0.348120067 | down |
| LRRC37A6P | -0.640896959 | 0.312326155 | 0.451001009 | down |
| ZYG11A | -0.640858377 | 0.208035425 | 0.338270083 | down |

| | | | | |
|---|---|---|---|---|
| DLG3 | -0.640753847 | 7.77E-05 | 0.000766836 | down |
| FHDC1 | -0.640616095 | 0.010004461 | 0.036686686 | down |
| RP11-75C10.9 | -0.640546722 | 0.440758778 | | down |
| PAK6 | -0.640544274 | 0.031197405 | 0.086315024 | down |
| SOBP | -0.640401347 | 0.014817531 | 0.049491705 | down |
| RP11-480D4.2 | -0.640294253 | 0.633733018 | | down |
| ATOH1 | -0.640233411 | 0.136770083 | 0.252035984 | down |
| TTC21A | -0.640125402 | 0.012008941 | 0.042228071 | down |
| RP11-109N23.6 | -0.640055879 | 0.103825298 | 0.207429662 | down |
| OR7E14P | -0.639997467 | 0.027875758 | 0.079557261 | down |
| DUOX1 | -0.639993004 | 0.019912256 | 0.061872358 | down |
| DLG5 | -0.639824809 | 0.030403363 | 0.084648193 | down |
| CEP83-AS1 | -0.639746892 | 0.219411404 | 0.350824706 | down |
| EZR-AS1 | -0.63960388 | 0.533841073 | | down |
| ASPHD1 | -0.63950696 | 0.015692447 | 0.051703848 | down |
| NA | -0.639421282 | 0.308451489 | 0.446952664 | down |
| RP11-295G12.1 | -0.639362325 | 0.751626336 | | down |
| C5orf30 | -0.639229244 | 0.012046071 | 0.042309113 | down |
| RP1-182D15.2 | -0.639169252 | 0.216000708 | 0.347110188 | down |
| PCDHGA7 | -0.63912456 | 0.231150803 | 0.363687744 | down |
| SPATA13-AS1 | -0.639099658 | 0.465675535 | 0.597053547 | down |
| PAFAH1B3 | -0.638983838 | 0.009551483 | 0.0353797 | down |
| AVPI1 | -0.638926027 | 0.003807261 | 0.017519583 | down |
| RNFT2 | -0.638832711 | 0.041158566 | 0.106115847 | down |
| AP003774.1 | -0.638797946 | 0.028532353 | 0.08097733 | down |
| RIPK3 | -0.638795018 | 0.00059962 | 0.004031703 | down |
| MORN3 | -0.638786546 | 0.073220349 | 0.161538565 | down |
| ZNF708 | -0.638722187 | 0.028620858 | 0.081128885 | down |
| ECE2 | -0.638677828 | 0.563422525 | | down |
| LIPE | -0.638583141 | 0.001726908 | 0.009350012 | down |
| RAB40C | -0.638536338 | 0.000115477 | 0.001068492 | down |
| FAUP1 | -0.638416445 | 0.047556063 | 0.117875948 | down |
| RP11-283G6.6 | -0.638406151 | 0.593309082 | | down |
| IGHVII-74-1 | -0.638369211 | 0.752565913 | 0.832425048 | down |
| RP11-462G22.2 | -0.638286079 | 0.501432951 | | down |
| RP11-834C11.6 | -0.638211372 | 0.195404633 | 0.32390272 | down |
| RP11-94C24.6 | -0.638003216 | 0.649809945 | | down |
| ERI2 | -0.637927647 | 0.030130172 | 0.084058955 | down |
| RP11-222K16.1 | -0.637855163 | 0.012894854 | 0.044495307 | down |
| TIMM8A | -0.637623011 | 0.00237698 | 0.012073369 | down |
| RP11-477I4.4 | -0.637594212 | 0.066346782 | 0.150279479 | down |
| RP1-278O22.2 | -0.637446738 | 0.678696112 | | down |
| HCG25 | -0.637345371 | 0.098338289 | 0.199625781 | down |
| MTATP8P1 | -0.63719978 | 0.303196352 | 0.441210462 | down |
| RP11-62H7.2 | -0.637196513 | 0.113850923 | 0.221594787 | down |
| RN7SL403P | -0.63697375 | 0.440656207 | 0.574338428 | down |
| MAGOH2P | -0.636912194 | 0.329425478 | 0.467831187 | down |
| IGFBP6 | -0.636892406 | 0.037673464 | 0.099355825 | down |
| Metazoa_SRP | -0.636768854 | 0.395868749 | 0.532981434 | down |
| TRBV7-4 | -0.636749405 | 0.310575789 | 0.449056211 | down |
| RP11-496I2.5 | -0.636735152 | 0.38767248 | 0.525217893 | down |

| | | | | |
|---|---|---|---|---|
| LINC00381 | -0.636720024 | 0.328323652 | 0.466809701 | down |
| RP11-588L15.2 | -0.636678351 | 0.518404391 | | down |
| BVES-AS1 | -0.636564033 | 0.232624811 | 0.365327269 | down |
| KIF16B | -0.636473041 | 0.011831103 | 0.041729708 | down |
| RP11-442N24__B.[illegible] | -0.636367821 | 0.126212738 | 0.238351593 | down |
| MCCD1P2 | -0.636176135 | 0.585876372 | | down |
| RPEP3 | -0.636128497 | 0.827883507 | | down |
| RP11-319C21.1 | -0.636128497 | 0.827883507 | | down |
| MIR7156 | -0.636128497 | 0.827883507 | | down |
| CCDC185 | -0.636128497 | 0.827883507 | | down |
| RP11-125A15.3 | -0.636128497 | 0.827883507 | | down |
| MIR4429 | -0.636128497 | 0.827883507 | | down |
| RNA5SP91 | -0.636128497 | 0.827883507 | | down |
| AC008074.4 | -0.636128497 | 0.827883507 | | down |
| AC007881.4 | -0.636128497 | 0.827883507 | | down |
| SUCLA2P2 | -0.636128497 | 0.827883507 | | down |
| RP11-223J6.2 | -0.636128497 | 0.827883507 | | down |
| RPL10P5 | -0.636128497 | 0.827883507 | | down |
| VIS1 | -0.636128497 | 0.827883507 | | down |
| NA | -0.636128497 | 0.827883507 | | down |
| RP11-646I19.1 | -0.636128497 | 0.827883507 | | down |
| AC092162.2 | -0.636128497 | 0.827883507 | | down |
| DPRXP1 | -0.636128497 | 0.827883507 | | down |
| RNU6-959P | -0.636128497 | 0.827883507 | | down |
| MTATP6P16 | -0.636128497 | 0.827883507 | | down |
| RP11-1029M24.4 | -0.636128497 | 0.827883507 | | down |
| RP11-875H7.1 | -0.636128497 | 0.827883507 | | down |
| RP11-1E6.1 | -0.636128497 | 0.827883507 | | down |
| U6 | -0.636128497 | 0.827883507 | | down |
| MRPL22P1 | -0.636128497 | 0.827883507 | | down |
| CXCL1P1 | -0.636128497 | 0.827883507 | | down |
| RN7SL127P | -0.636128497 | 0.827883507 | | down |
| RP11-75N20.2 | -0.636128497 | 0.827883507 | | down |
| RNU6-1054P | -0.636128497 | 0.827883507 | | down |
| RP11-775H9.1 | -0.636128497 | 0.827883507 | | down |
| RNU7-194P | -0.636128497 | 0.827883507 | | down |
| DEAR | -0.636128497 | 0.827883507 | | down |
| RP11-158C21.2 | -0.636128497 | 0.827883507 | | down |
| RP11-616K6.1 | -0.636128497 | 0.827883507 | | down |
| SNORD79 | -0.636128497 | 0.827883507 | | down |
| DUX4L8 | -0.636128497 | 0.827883507 | | down |
| RP11-3507.1 | -0.636128497 | 0.827883507 | | down |
| LINC01335 | -0.636128497 | 0.827883507 | | down |
| BIN2P2 | -0.636128497 | 0.827883507 | | down |
| CTD-2197I11.1 | -0.636128497 | 0.827883507 | | down |
| SNORA74 | -0.636128497 | 0.827883507 | | down |
| RNU7-180P | -0.636128497 | 0.827883507 | | down |
| CTD-2593A12.2 | -0.636128497 | 0.827883507 | | down |
| RP3-430A16.1 | -0.636128497 | 0.827883507 | | down |
| NA | -0.636128497 | 0.827883507 | | down |
| RNU6-411P | -0.636128497 | 0.827883507 | | down |

| | | | |
|---|---|---|---|
| RP11-204P2.3 | -0.636128497 | 0.827883507 | down |
| CACYBPP3 | -0.636128497 | 0.827883507 | down |
| AC010082.2 | -0.636128497 | 0.827883507 | down |
| NPM1P13 | -0.636128497 | 0.827883507 | down |
| GNGT1 | -0.636128497 | 0.827883507 | down |
| RNA5SP237 | -0.636128497 | 0.827883507 | down |
| RNA5SP242 | -0.636128497 | 0.827883507 | down |
| OR7E158P | -0.636128497 | 0.827883507 | down |
| AC100802.3 | -0.636128497 | 0.827883507 | down |
| ASNSP4 | -0.636128497 | 0.827883507 | down |
| CTD-2182N23.1 | -0.636128497 | 0.827883507 | down |
| RNU4-26P | -0.636128497 | 0.827883507 | down |
| AL137067.2 | -0.636128497 | 0.827883507 | down |
| OR13D3P | -0.636128497 | 0.827883507 | down |
| MIR1296 | -0.636128497 | 0.827883507 | down |
| RP11-344L13.2 | -0.636128497 | 0.827883507 | down |
| HMGB3P8 | -0.636128497 | 0.827883507 | down |
| OR51J1 | -0.636128497 | 0.827883507 | down |
| RP11-748C4.1 | -0.636128497 | 0.827883507 | down |
| RN7SL786P | -0.636128497 | 0.827883507 | down |
| BLID | -0.636128497 | 0.827883507 | down |
| RERG-AS1 | -0.636128497 | 0.827883507 | down |
| NPM1P22 | -0.636128497 | 0.827883507 | down |
| BTF3P11 | -0.636128497 | 0.827883507 | down |
| RP11-16O13.1 | -0.636128497 | 0.827883507 | down |
| RP11-757H14.2 | -0.636128497 | 0.827883507 | down |
| AL132796.1 | -0.636128497 | 0.827883507 | down |
| IGHV1OR15-6 | -0.636128497 | 0.827883507 | down |
| POTEB3 | -0.636128497 | 0.827883507 | down |
| RP11-536P16.4 | -0.636128497 | 0.827883507 | down |
| RP11-261B23.1 | -0.636128497 | 0.827883507 | down |
| MTCYBP23 | -0.636128497 | 0.827883507 | down |
| CTD-3229J4.1 | -0.636128497 | 0.827883507 | down |
| RP11-510J16.6 | -0.636128497 | 0.827883507 | down |
| RN7SL792P | -0.636128497 | 0.827883507 | down |
| TSEN15P1 | -0.636128497 | 0.827883507 | down |
| CTD-2206N4.5 | -0.636128497 | 0.827883507 | down |
| RN7SL716P | -0.636128497 | 0.827883507 | down |
| RN7SL862P | -0.636128497 | 0.827883507 | down |
| NA | -0.636128497 | 0.827883507 | down |
| AC010641.1 | -0.636128497 | 0.827883507 | down |
| CTD-2538G9.6 | -0.636128497 | 0.827883507 | down |
| SNORA70 | -0.636128497 | 0.827883507 | down |
| CTB-60B18.23 | -0.636128497 | 0.827883507 | down |
| Y_RNA | -0.636128497 | 0.827883507 | down |
| RN7SL48P | -0.636128497 | 0.827883507 | down |
| RP11-22B10.3 | -0.636128497 | 0.827883507 | down |
| ACA64 | -0.636128497 | 0.827883507 | down |
| SNORA25 | -0.636128497 | 0.827883507 | down |
| MTCO1P19 | -0.636128497 | 0.827883507 | down |
| RPS29P12 | -0.636128183 | 0.810006315 | down |

| | | | | |
|---|---|---|---|---|
| SNORD92 | -0.636128162 | 0.808594325 | | down |
| MTCO1P24 | -0.636128139 | 0.807028861 | | down |
| RN7SKP241 | -0.636128133 | 0.806614794 | | down |
| ASIC3 | -0.63612598 | 0.01443885 | 0.048495795 | down |
| RP11-832A4.7 | -0.635978044 | 0.194980332 | 0.323395666 | down |
| PTPRVP | -0.635770055 | 0.07636147 | 0.166486951 | down |
| RP11-521I2.3 | -0.635753629 | 0.099688503 | 0.201514918 | down |
| CTC-297N7.1 | -0.635745728 | 0.318454987 | 0.457323342 | down |
| DYNLRB1 | -0.635121609 | 0.061104008 | 0.141695647 | down |
| ILF3-AS1 | -0.634931012 | 1.91E-05 | 0.000241309 | down |
| AF067845.3 | -0.634924526 | 0.818180298 | | down |
| RPS3P2 | -0.634894182 | 0.770873427 | | down |
| IGLV5-52 | -0.634829658 | 0.696119002 | | down |
| RNA5SP450 | -0.634828365 | 0.694840382 | | down |
| LAMTOR2 | -0.634814317 | 0.003934863 | 0.017993764 | down |
| AC064834.3 | -0.634798481 | 0.666962563 | | down |
| AC093826.1 | -0.634798146 | 0.6666661 | | down |
| RP11-578F21.10 | -0.634798101 | 0.666626597 | | down |
| RP11-380M21.3 | -0.634796312 | 0.665051846 | | down |
| AK9 | -0.634768175 | 0.006612023 | 0.026823064 | down |
| IGHVII-78-1 | -0.634729693 | 0.612185228 | | down |
| RPL21P5 | -0.634702296 | 0.593088687 | | down |
| RP11-390F4.3 | -0.634660449 | 0.0163822 | 0.053347801 | down |
| EPHA1 | -0.634585968 | 0.005588295 | 0.023617209 | down |
| DCAF11 | -0.634564198 | 6.26E-05 | 0.000642288 | down |
| Z97634.3 | -0.634540619 | 0.035865683 | 0.095756561 | down |
| RP11-334A14.8 | -0.634397097 | 0.378334076 | 0.51643203 | down |
| CTDSPL | -0.634384198 | 0.00033128 | 0.002528703 | down |
| HECTD3 | -0.634336634 | 9.70E-05 | 0.000921795 | down |
| ALG1L10P | -0.63429782 | 0.181854728 | 0.307395266 | down |
| LIPT2 | -0.634266625 | 0.004631757 | 0.020455393 | down |
| CTD-2591A1.1 | -0.634236714 | 0.418684107 | | down |
| NUDT16 | -0.63422161 | 2.59E-05 | 0.000310211 | down |
| TSPAN10 | -0.63409402 | 0.138932885 | 0.254953554 | down |
| C1orf146 | -0.633940091 | 0.358126882 | 0.496564705 | down |
| RN7SKP60 | -0.633842572 | 0.65131535 | 0.755117635 | down |
| MSTN | -0.633840878 | 0.378244858 | 0.516380629 | down |
| POM121B | -0.633608746 | 0.313527618 | 0.452142577 | down |
| RP11-849H4.2 | -0.633285378 | 0.077487138 | 0.168187819 | down |
| CTD-2186M15.3 | -0.633214158 | 0.002088536 | 0.010856053 | down |
| RP5-1024G6.7 | -0.633147097 | 0.660623726 | | down |
| GNRH1 | -0.632968654 | 0.034622246 | 0.093361532 | down |
| RPS15AP1 | -0.632838081 | 0.052753349 | 0.12705801 | down |
| C2CD2L | -0.632810208 | 0.000152467 | 0.001337437 | down |
| AC016734.2 | -0.63274792 | 0.101415846 | 0.204095228 | down |
| FBXL18 | -0.632690489 | 0.002161444 | 0.011169319 | down |
| MPP7 | -0.632657745 | 0.004092963 | 0.01857818 | down |
| EEFSEC | -0.632488078 | 0.00516404 | 0.022233938 | down |
| RP11-560J1.2 | -0.632366114 | 0.141262212 | 0.257928101 | down |
| CDK13 | -0.632306893 | 0.000471545 | 0.003320153 | down |
| OR2A1-AS1 | -0.632167592 | 0.123972188 | 0.235344064 | down |

| | | | | |
|---|---|---|---|---|
| RPL23AP64 | -0.632157843 | 0.278046232 | 0.414314973 | down |
| BTN3A2 | -0.632123584 | 0.002345197 | 0.01194222 | down |
| RP4-635E18.6 | -0.632082295 | 0.210655758 | 0.341242587 | down |
| RP11-542A14.1 | -0.631900658 | 0.698474932 | | down |
| CDKL1 | -0.631732401 | 0.000477874 | 0.003353716 | down |
| SCGB2B2 | -0.631694659 | 0.2294899 | 0.361888492 | down |
| RP11-7306.3 | -0.631386297 | 0.387301139 | 0.524975205 | down |
| SCNN1D | -0.631364509 | 0.005033607 | 0.021812699 | down |
| KREMEN2 | -0.63123163 | 0.083947721 | 0.178305079 | down |
| CLMN | -0.631023977 | 0.000200171 | 0.001670538 | down |
| NA | -0.630773291 | 0.401035723 | | down |
| IGSF5 | -0.630728401 | 0.352675862 | | down |
| RNU6-1154P | -0.630717918 | 0.771057619 | | down |
| RP11-392P7.6 | -0.630643696 | 0.006234563 | 0.025690742 | down |
| BOP1 | -0.630478197 | 0.011001908 | 0.039469289 | down |
| BCRP9 | -0.630436087 | 0.829384774 | | down |
| KRT17P6 | -0.630387669 | 0.622450669 | | down |
| RP1-13D10.2 | -0.630342624 | 0.454732372 | 0.587223367 | down |
| COASY | -0.630038153 | 0.001327697 | 0.007577763 | down |
| ADARB2-AS1 | -0.630007116 | 0.611206576 | 0.722885646 | down |
| TMEM115 | -0.629973749 | 0.00311174 | 0.014912924 | down |
| AMH | -0.629939292 | 0.073295163 | 0.161656135 | down |
| RP11-519G16.5 | -0.629794783 | 0.072530122 | 0.160499005 | down |
| RP5-1061H20.4 | -0.629721766 | 0.006087443 | 0.025229842 | down |
| ENSAP1 | -0.629687324 | 0.829595702 | | down |
| LINC00216 | -0.629683364 | 0.506899391 | | down |
| GBX2 | -0.62966554 | 0.818367042 | | down |
| Metazoa_SRP | -0.629549344 | 0.76803543 | | down |
| FBXO36 | -0.629496018 | 0.054737043 | 0.130683386 | down |
| CTD-2021A8.2 | -0.629485979 | 0.745098241 | | down |
| RP11-351M16.4 | -0.629484995 | 0.744760256 | | down |
| ADAMTSL3 | -0.629452495 | 0.152113203 | 0.271511946 | down |
| MOCOS | -0.629425886 | 0.036231871 | 0.096554047 | down |
| C12orf43 | -0.629294986 | 0.000633946 | 0.004213238 | down |
| CTD-2024I7.13 | -0.629262476 | 0.747340077 | | down |
| MZT2A | -0.629224239 | 0.00518828 | 0.022301015 | down |
| PINK1-AS | -0.629145342 | 0.043952345 | 0.11136917 | down |
| NRN1L | -0.629119357 | 0.745714769 | | down |
| RP11-685G9.4 | -0.629031008 | 0.6238249 | | down |
| POC1A | -0.629029247 | 0.004058349 | 0.018462502 | down |
| FAM45A | -0.629022377 | 0.000184178 | 0.001564 | down |
| NA | -0.628780942 | 0.447029292 | | down |
| HAL | -0.628544328 | 0.101736097 | 0.204561592 | down |
| SLC25A5P2 | -0.628384021 | 0.656488764 | | down |
| RPS15AP11 | -0.62824473 | 0.136156097 | 0.251280329 | down |
| STX19 | -0.627955047 | 0.039924851 | 0.103817052 | down |
| AP1S1 | -0.627901358 | 0.00181497 | 0.009725157 | down |
| RP11-3304.2 | -0.627887477 | 0.343626021 | 0.482019805 | down |
| RP11-227G15.8 | -0.627627972 | 0.561434304 | | down |
| AF186192.5 | -0.627529887 | 0.208209202 | 0.338414597 | down |
| NA | -0.627519894 | 0.409253939 | 0.546035409 | down |

| | | | | |
|---|---|---|---|---|
| JHDM1D-AS1 | -0.627477482 | 4.18E-05 | 0.000460065 | down |
| RP11-17E13.2 | -0.627436825 | 0.165374161 | 0.288179415 | down |
| CCDC17 | -0.627328554 | 0.005282191 | 0.022635594 | down |
| NA | -0.62716927 | 0.130493029 | 0.243988323 | down |
| RP11-101E7.2 | -0.627036043 | 0.43531015 | 0.570115874 | down |
| AC005785.2 | -0.626993747 | 0.093227916 | 0.192226992 | down |
| RP4-593H12.1 | -0.626939903 | 0.605697175 | | down |
| UBE2V2P4 | -0.626896041 | 0.691153402 | | down |
| CTD-2358C21.5 | -0.62685874 | 0.390230235 | 0.527945115 | down |
| ARHGAP35 | -0.626850202 | 3.00E-05 | 0.000349869 | down |
| NA | -0.626719447 | 0.830390312 | | down |
| RP4-665J23.2 | -0.626719447 | 0.830390312 | | down |
| VPS25P1 | -0.626719447 | 0.830390312 | | down |
| AC116609.2 | -0.626719447 | 0.830390312 | | down |
| SNORA64 | -0.626719447 | 0.830390312 | | down |
| NA | -0.626719447 | 0.830390312 | | down |
| CYCSP8 | -0.626719447 | 0.830390312 | | down |
| RP11-91K8.2 | -0.626719447 | 0.830390312 | | down |
| LARP1BP1 | -0.626719447 | 0.830390312 | | down |
| ACTR3BP4 | -0.626719447 | 0.830390312 | | down |
| RP11-402J6.1 | -0.626719447 | 0.830390312 | | down |
| CTB-51E13.1 | -0.626719447 | 0.830390312 | | down |
| RP11-76G10.1 | -0.626719447 | 0.830390312 | | down |
| CTD-2290C23.2 | -0.626719447 | 0.830390312 | | down |
| SCARNA18 | -0.626719447 | 0.830390312 | | down |
| COQ10BP2 | -0.626719447 | 0.830390312 | | down |
| RPL17P25 | -0.626719447 | 0.830390312 | | down |
| RNU6-979P | -0.626719447 | 0.830390312 | | down |
| NA | -0.626719447 | 0.830390312 | | down |
| AC073900.4 | -0.626719447 | 0.830390312 | | down |
| RP11-359P18.7 | -0.626719447 | 0.830390312 | | down |
| CRH | -0.626719447 | 0.830390312 | | down |
| RBM17P3 | -0.626719447 | 0.830390312 | | down |
| RP11-410K21.2 | -0.626719447 | 0.830390312 | | down |
| RP11-45A16.4 | -0.626719447 | 0.830390312 | | down |
| NA | -0.626719447 | 0.830390312 | | down |
| RP11-91P24.3 | -0.626719447 | 0.830390312 | | down |
| NA | -0.626719447 | 0.830390312 | | down |
| RP11-611O2.1 | -0.626719447 | 0.830390312 | | down |
| YWHAQP7 | -0.626719447 | 0.830390312 | | down |
| RP11-128P10.1 | -0.626719447 | 0.830390312 | | down |
| RP11-117L5.4 | -0.626719447 | 0.830390312 | | down |
| RP11-991C1.2 | -0.626719447 | 0.830390312 | | down |
| NA | -0.626719447 | 0.830390312 | | down |
| RP11-271K11.6 | -0.626719447 | 0.830390312 | | down |
| CSH1 | -0.626719447 | 0.830390312 | | down |
| RFWD2P1 | -0.626719447 | 0.830390312 | | down |
| Y_RNA | -0.626719447 | 0.830390312 | | down |
| RNA5SP462 | -0.626719447 | 0.830390312 | | down |
| AC010649.1 | -0.626719447 | 0.830390312 | | down |
| U6 | -0.626719447 | 0.830390312 | | down |

| | | | | |
|---|---|---|---|---|
| MRPS16P2 | -0.626719447 | 0.830390312 | | down |
| SNORA80A | -0.626719447 | 0.830390312 | | down |
| AP000351.4 | -0.626719447 | 0.830390312 | | down |
| MTCYBP34 | -0.626719447 | 0.830390312 | | down |
| NA | -0.626719447 | 0.830390312 | | down |
| CKBP1 | -0.626719422 | 0.829021988 | | down |
| PAGE4 | -0.626719406 | 0.828129347 | | down |
| PLS1-AS1 | -0.626719113 | 0.808962977 | | down |
| RP11-451F14.1 | -0.626719012 | 0.800674449 | | down |
| PLCXD2-AS1 | -0.62671884 | 0.783766044 | | down |
| CTC-559E9.4 | -0.626718806 | 0.779859998 | | down |
| GPC6-AS2 | -0.626698275 | 0.676757092 | | down |
| NUDT1 | -0.626666786 | 0.020424167 | 0.063091063 | down |
| RP11-707023.5 | -0.626522492 | 0.541666702 | 0.663263547 | down |
| AF240627.2 | -0.626364643 | 0.83030301 | | down |
| SGK223 | -0.62616524 | 0.006505965 | 0.026510946 | down |
| AC011330.5 | -0.626098329 | 0.516855704 | | down |
| LRRC28 | -0.62591966 | 3.59E-07 | 8.41E-06 | down |
| AIFM2 | -0.625913749 | 0.000185693 | 0.001575084 | down |
| NA | -0.625912688 | 0.663502385 | 0.76473187 | down |
| RNU6-50P | -0.625891751 | 0.51685643 | | down |
| RSPH3 | -0.625503377 | 0.002283702 | 0.011696478 | down |
| NCAM1 | -0.625420589 | 0.035903109 | 0.095805402 | down |
| IPPK | -0.62537185 | 0.169057832 | 0.292528566 | down |
| FOCAD-AS1 | -0.625170482 | 0.496453821 | 0.624077766 | down |
| LINC00954 | -0.625156417 | 0.116555771 | 0.225355658 | down |
| RP11-284F21.10 | -0.624934128 | 0.027424021 | 0.07857403 | down |
| RP3-369A17.4 | -0.624883311 | 0.620095953 | 0.730422755 | down |
| AC019048.1 | -0.624797941 | 0.18835816 | 0.315248874 | down |
| TMEM86B | -0.624773014 | 0.091031762 | 0.189021927 | down |
| DYRK2 | -0.624762709 | 0.002813141 | 0.013789853 | down |
| RPL7P18 | -0.624705301 | 0.330329283 | 0.468813352 | down |
| MT1P1 | -0.624487408 | 0.533270573 | | down |
| ME2 | -0.624385607 | 0.000880939 | 0.005471763 | down |
| TPSP2 | -0.624329348 | 0.599729833 | | down |
| RN7SKP71 | -0.624278568 | 0.576716082 | | down |
| NA | -0.624264845 | 0.422022558 | 0.557525536 | down |
| CTC-303L1.2 | -0.624140083 | 0.612882098 | | down |
| RP11-115H15.2 | -0.623998567 | 0.649351612 | | down |
| MIR92A2 | -0.623959211 | 0.630369941 | | down |
| KRT1 | -0.623939925 | 0.091751261 | 0.190095503 | down |
| DAGLB | -0.623674624 | 0.001322695 | 0.007554954 | down |
| MAGEA11 | -0.623639549 | 0.773755507 | | down |
| HNRNPA3P3 | -0.623612137 | 0.320280045 | 0.459127252 | down |
| SIL1 | -0.623593787 | 0.076977451 | 0.167466367 | down |
| CTB-113P19.1 | -0.623453785 | 0.275411099 | 0.411705197 | down |
| RP11-380N8.7 | -0.623420365 | 0.330439073 | 0.468813352 | down |
| SLC25A26 | -0.623064033 | 0.000124583 | 0.001135959 | down |
| PDK3 | -0.622969336 | 4.36E-08 | 1.39E-06 | down |
| HNRNPKP3 | -0.622966362 | 0.773204541 | | down |
| ZNF559-ZNF177 | -0.622881249 | 0.602868871 | | down |

| | | | | |
|---|---|---|---|---|
| RP11-531A24.7 | -0.622797348 | 0.530435324 | | down |
| RP11-85E16.1 | -0.622788651 | 0.630648762 | | down |
| MFSD9 | -0.622658564 | 0.000409694 | 0.002963128 | down |
| NA | -0.62259527 | 0.099889157 | 0.201725471 | down |
| WASH2P | -0.622588296 | 0.000262131 | 0.00209065 | down |
| KCNQ4 | -0.622535761 | 0.029392195 | 0.082645407 | down |
| NA | -0.622527431 | 0.685016129 | | down |
| KIF5A | -0.622309509 | 0.155085983 | 0.275166462 | down |
| RPS15 | -0.622106419 | 0.010064404 | 0.036870499 | down |
| C1orf50 | -0.622062804 | 0.002971548 | 0.014393639 | down |
| NA | -0.62206133 | 0.17109861 | 0.295023338 | down |
| AC027612.6 | -0.621970477 | 0.02164412 | 0.065817404 | down |
| SLC9A1 | -0.621902965 | 0.000353716 | 0.002659419 | down |
| AC016582.2 | -0.621876948 | 0.389022146 | 0.52659524 | down |
| ACOT8 | -0.621827914 | 0.000104639 | 0.000983048 | down |
| RECQL5 | -0.621825659 | 0.000675838 | 0.004434345 | down |
| RP11-770J1.3 | -0.621714059 | 0.223926435 | 0.356148534 | down |
| SLC7A10 | -0.621689392 | 0.214374002 | 0.345287756 | down |
| RP3-522J7.5 | -0.621670836 | 0.607423157 | | down |
| RP11-83N9.6 | -0.621490746 | 0.292614798 | 0.429956423 | down |
| SH3RF1 | -0.62144049 | 0.000813258 | 0.005143899 | down |
| CLC | -0.621365181 | 0.155453128 | 0.275574331 | down |
| RP11-386M24.6 | -0.621246111 | 0.490246539 | | down |
| P3H4 | -0.621151301 | 0.009660461 | 0.035677798 | down |
| RP11-504P24.9 | -0.62094951 | 0.355863207 | 0.494413624 | down |
| RP11-434H14.1 | -0.620939327 | 0.219224521 | 0.350628841 | down |
| GPATCH1 | -0.62093156 | 1.09E-06 | 2.18E-05 | down |
| RP11-317B17.3 | -0.620917932 | 0.657702699 | | down |
| RP11-1391J7.1 | -0.620831397 | 0.053485897 | 0.128359318 | down |
| SGK494 | -0.620830242 | 0.020257997 | 0.062725863 | down |
| ZNF503-AS2 | -0.620775273 | 0.031208039 | 0.086336495 | down |
| ELFN2 | -0.620752724 | 0.218387108 | 0.349829037 | down |
| CCDC144NL-AS1 | -0.620691506 | 0.13419219 | 0.248561662 | down |
| SMPDL3A | -0.620625456 | 0.016578844 | 0.05382481 | down |
| RPL10P3 | -0.620372266 | 0.11619422 | 0.22487369 | down |
| RHPN2 | -0.620287066 | 0.012294371 | 0.042955079 | down |
| PMVK | -0.620212114 | 0.000680841 | 0.00445938 | down |
| ZMYND8 | -0.620097099 | 1.15E-05 | 0.000157849 | down |
| RNPEP | -0.620011717 | 0.000717854 | 0.004656132 | down |
| CCDC141 | -0.619975769 | 0.088510619 | 0.185336844 | down |
| SLCO1C1 | -0.619956982 | 0.199347176 | 0.328374487 | down |
| PNMT | -0.619815668 | 0.109041248 | 0.215207556 | down |
| CTC-21504.4 | -0.619720499 | 0.628720511 | | down |
| RP11-613H2.2 | -0.619642668 | 0.483746426 | | down |
| PARL | -0.619617615 | 0.034958141 | 0.093959778 | down |
| RP11-664D1.1 | -0.619597264 | 0.194466013 | 0.322738602 | down |
| RP11-505K9.1 | -0.619577612 | 0.036336181 | 0.096749097 | down |
| LINC01393 | -0.61939395 | 0.400924313 | 0.537835777 | down |
| RP11-5407.1 | -0.619364042 | 0.368642808 | 0.507116301 | down |
| RAB40B | -0.619251442 | 0.000122063 | 0.001118065 | down |
| NA | -0.619047638 | 0.058602205 | 0.137357204 | down |

| | | | | |
|---|---|---|---|---|
| PRADC1P1 | -0.618988738 | 0.439270793 | | down |
| MIR4677 | -0.618922195 | 0.681226197 | | down |
| HSPE1P2 | -0.618793945 | 0.283268293 | 0.41983684 | down |
| RP11-495P10.6 | -0.618773187 | 0.695772528 | | down |
| LINC01163 | -0.61875231 | 0.404740562 | 0.541191216 | down |
| NCAM1-AS1 | -0.618668122 | 0.474150049 | 0.604334129 | down |
| RP4-740C4.5 | -0.618541307 | 0.018761304 | 0.059158756 | down |
| MGAT3-AS1 | -0.618530426 | 0.427590978 | 0.562999007 | down |
| CDC34 | -0.618338996 | 0.005691411 | 0.023935156 | down |
| AC125421.1 | -0.618297487 | 0.711086748 | 0.801624299 | down |
| HLA-DPA2 | -0.618275575 | 0.673338188 | | down |
| NA | -0.61820108 | 0.097273195 | 0.198147392 | down |
| FLJ31104 | -0.617916285 | 0.091780071 | 0.190142081 | down |
| MMP24-AS1 | -0.617875199 | 0.000770565 | 0.004922573 | down |
| FIGLA | -0.617857188 | 0.626952647 | | down |
| MS4A4E | -0.61783301 | 0.063723999 | 0.145990837 | down |
| AC008065.1 | -0.617773449 | 0.509782145 | | down |
| NADSYN1 | -0.617764664 | 2.93E-05 | 0.00034342 | down |
| MICAL3 | -0.617549781 | 0.006376155 | 0.02613084 | down |
| AC074212.6 | -0.617392052 | 0.092730907 | 0.191489633 | down |
| RN7SL632P | -0.617310397 | 0.832898846 | | down |
| RNU2-39P | -0.617310397 | 0.832898846 | | down |
| CCDC148-AS1 | -0.617310397 | 0.832898846 | | down |
| IGBP1P3 | -0.617310397 | 0.832898846 | | down |
| RCC2P8 | -0.617310397 | 0.832898846 | | down |
| RNU6-335P | -0.617310397 | 0.832898846 | | down |
| RP11-505P4.5 | -0.617310397 | 0.832898846 | | down |
| TAS2R60 | -0.617310397 | 0.832898846 | | down |
| RP11-796I2.1 | -0.617310397 | 0.832898846 | | down |
| AC019084.7 | -0.617310397 | 0.832898846 | | down |
| RP11-770E5.2 | -0.617310397 | 0.832898846 | | down |
| RP11-246K15.1 | -0.617310397 | 0.832898846 | | down |
| CER1 | -0.617310397 | 0.832898846 | | down |
| RPL7P33 | -0.617310397 | 0.832898846 | | down |
| KRT18P24 | -0.617310397 | 0.832898846 | | down |
| OR7E13P | -0.617310397 | 0.832898846 | | down |
| RN7SKP3 | -0.617310397 | 0.832898846 | | down |
| CLCP2 | -0.617310397 | 0.832898846 | | down |
| RP11-232C2.1 | -0.617310397 | 0.832898846 | | down |
| NA | -0.617310397 | 0.832898846 | | down |
| AC087499.10 | -0.617310397 | 0.832898846 | | down |
| Metazoa_SRP | -0.617310397 | 0.832898846 | | down |
| RPL17P45 | -0.617310397 | 0.832898846 | | down |
| BDP1P | -0.617310397 | 0.832898846 | | down |
| SLC25A6P4 | -0.617310397 | 0.832898846 | | down |
| AC007326.9 | -0.617310397 | 0.832898846 | | down |
| RP11-337A23.5 | -0.617310361 | 0.830718314 | | down |
| RP11-454C18.1 | -0.617310347 | 0.829863479 | | down |
| HIST1H4PS1 | -0.617310341 | 0.829476058 | | down |
| SLC24A5 | -0.617310341 | 0.82948221 | | down |
| NA | -0.617310068 | 0.809076813 | | down |

| | | | | |
|---|---|---|---|---|
| RP11-489M13.1 | -0.617310051 | 0.807515649 | | down |
| RP11-804A23.2 | -0.617310046 | 0.807021169 | | down |
| CYP51A1P1 | -0.617310045 | 0.807002754 | | down |
| USP12-AS1 | -0.617310028 | 0.805386822 | | down |
| NA | -0.617309649 | 0.755599538 | | down |
| RP11-805F19.4 | -0.617309646 | 0.755170765 | | down |
| RNU6-1128P | -0.617309635 | 0.752986668 | | down |
| CTD-2377D24.2 | -0.617309503 | 0.722892438 | | down |
| RNA5SP473 | -0.6173095 | 0.722202096 | | down |
| RP1-76B20.11 | -0.617309352 | 0.669879798 | | down |
| ZNF552 | -0.617305376 | 6.46E-05 | 0.000657529 | down |
| EI24P2 | -0.617185201 | 0.728650398 | | down |
| RP11-1609.2 | -0.617158148 | 0.591446733 | 0.706425446 | down |
| NUDT16P1 | -0.617128146 | 0.025439549 | 0.07423269 | down |
| TLK2P1 | -0.617087766 | 0.262899193 | 0.398192345 | down |
| NDUFAF4P3 | -0.616893166 | 0.416481609 | 0.55265993 | down |
| RP11-64C1.1 | -0.616857758 | 0.751272742 | | down |
| RP11-369E15.3 | -0.616729012 | 0.521421654 | 0.645737517 | down |
| GRP | -0.616646372 | 0.511636273 | 0.637270436 | down |
| SBK1 | -0.616609782 | 0.043204818 | 0.11006827 | down |
| ZMIZ1-AS1 | -0.616530047 | 0.083641778 | 0.177931995 | down |
| CTD-2286N8.2 | -0.616372711 | 0.139884159 | 0.256104794 | down |
| NAA38 | -0.616316929 | 0.018616129 | 0.058843028 | down |
| CH17-264L24.1 | -0.616303927 | 0.122676668 | 0.233635692 | down |
| FLNB-AS1 | -0.616124916 | 0.152677779 | 0.272196249 | down |
| SLC25A20 | -0.616111995 | 0.005624698 | 0.023728968 | down |
| MELTF | -0.616071314 | 0.006709852 | 0.027135658 | down |
| RP11-514P8.2 | -0.615754701 | 0.341567904 | 0.4798946 | down |
| ESRRA | -0.615710519 | 0.027835211 | 0.079464186 | down |
| RHBDD2 | -0.615685692 | 0.001107603 | 0.006584255 | down |
| C15orf40 | -0.615608692 | 3.27E-06 | 5.60E-05 | down |
| RNU12 | -0.615401462 | 0.361225716 | 0.499639308 | down |
| RP11-213G2.1 | -0.6153198 | 0.549971477 | | down |
| PSD | -0.615262033 | 0.037177105 | 0.098331628 | down |
| NMT2 | -0.615251625 | 3.92E-12 | 3.79E-10 | down |
| HYPK | -0.615193832 | 0.123403268 | 0.234579423 | down |
| PRR36 | -0.615103129 | 0.051842442 | 0.125467563 | down |
| AC019186.1 | -0.614742823 | 0.462731568 | 0.594496781 | down |
| CROCCP3 | -0.614326588 | 0.032220734 | 0.088390562 | down |
| Y_RNA | -0.614246095 | 0.754544941 | | down |
| CHPT1 | -0.61416801 | 0.020554139 | 0.063365657 | down |
| CTA-268H5.14 | -0.613999599 | 0.256656577 | 0.391659397 | down |
| RAB11B-AS1 | -0.613981478 | 0.004564942 | 0.020219573 | down |
| TNFRSF11A | -0.613914136 | 0.027013425 | 0.077671676 | down |
| MAN1B1-AS1 | -0.613826234 | 0.000703183 | 0.004582449 | down |
| RP1-151B14.6 | -0.613767101 | 0.403656263 | 0.540318501 | down |
| COX17P1 | -0.613723947 | 0.183492071 | 0.309223457 | down |
| FXYD1 | -0.613673146 | 0.215414353 | 0.346480476 | down |
| RP4-798P15.3 | -0.613383813 | 0.47876963 | 0.608363846 | down |
| C17orf97 | -0.613172919 | 0.027779477 | 0.079327688 | down |
| ZNF562 | -0.613160707 | 0.000509901 | 0.00353472 | down |

| | | | | |
|---|---|---|---|---|
| S100Z | -0.613057394 | 0.210845096 | 0.341415563 | down |
| PCDHGC5 | -0.612994996 | 0.42783512 | 0.563272496 | down |
| CLCN5 | -0.612993599 | 0.015357557 | 0.050871274 | down |
| STK38 | -0.612903571 | 6.92E-08 | 2.09E-06 | down |
| RP11-329B9.4 | -0.612843407 | 0.112606256 | 0.219798749 | down |
| PLCD4 | -0.612699626 | 0.023915094 | 0.070900442 | down |
| MIR657 | -0.612662958 | 0.634840214 | | down |
| SH3BGRL2 | -0.612658181 | 0.004470812 | 0.019905316 | down |
| MIR320E | -0.612603385 | 0.268307571 | 0.404082042 | down |
| CTD-2278I10.4 | -0.612559886 | 0.252416652 | 0.387393028 | down |
| IDH3G | -0.612500358 | 0.00074096 | 0.004778151 | down |
| ABCB8 | -0.61244853 | 0.0046691 | 0.020577737 | down |
| TPMT | -0.612444632 | 0.000748204 | 0.004814531 | down |
| PLIN5 | -0.612334713 | 0.032342328 | 0.088650023 | down |
| C11orf54 | -0.612297274 | 0.000214071 | 0.001767226 | down |
| NME3 | -0.612220919 | 0.006516498 | 0.026550266 | down |
| RP11-17M24.2 | -0.612190067 | 0.750825934 | | down |
| NAXE | -0.612090835 | 0.002527878 | 0.012683297 | down |
| WDR24 | -0.612061811 | 0.00306978 | 0.014754193 | down |
| RP11-471B22.3 | -0.612031875 | 0.128509497 | 0.24138414 | down |
| TSEN54 | -0.612021835 | 0.000163241 | 0.001414194 | down |
| LMO7 | -0.611952631 | 0.017777721 | 0.056857591 | down |
| RP11-188P17.2 | -0.611923988 | 0.426633388 | 0.562183048 | down |
| LRCOL1 | -0.611883517 | 0.132682226 | 0.24670781 | down |
| RP1-257I20.14 | -0.611858404 | 0.211178776 | 0.341687658 | down |
| TMEM63A | -0.611855184 | 0.001273152 | 0.007330506 | down |
| RP11-431N15.2 | -0.611677545 | 0.574584126 | | down |
| MBLAC1 | -0.611627418 | 0.00277429 | 0.013639462 | down |
| UBAC2 | -0.611581726 | 3.76E-06 | 6.25E-05 | down |
| YIF1A | -0.611423742 | 0.015807344 | 0.051965842 | down |
| RP11-44B19.1 | -0.611411217 | 0.501739076 | | down |
| MUC13 | -0.611324523 | 0.093952732 | 0.193363192 | down |
| RP11-181G12.2 | -0.611281954 | 0.076018127 | 0.165851834 | down |
| RP11-16D22.2 | -0.611164764 | 0.54892392 | 0.669475938 | down |
| RP11-326A19.4 | -0.610831251 | 0.404017438 | 0.540609268 | down |
| POLR2L | -0.610786934 | 0.027390849 | 0.078493958 | down |
| ADCK1 | -0.610774511 | 0.000156639 | 0.001367248 | down |
| MYOM2 | -0.610620225 | 0.007262727 | 0.02875232 | down |
| RP11-1060J15.4 | -0.610512621 | 0.612462656 | 0.723786651 | down |
| RP11-34P1.2 | -0.610387295 | 0.106196068 | 0.21097366 | down |
| RP11-452G18.2 | -0.610214927 | 0.169879903 | 0.293579052 | down |
| RP13-608F4.5 | -0.610019666 | 0.473781564 | 0.604069484 | down |
| ATG4D | -0.60999784 | 0.000523335 | 0.00361035 | down |
| RP11-61K9.2 | -0.609829687 | 0.469005176 | 0.59994003 | down |
| GOLPH3L | -0.609793248 | 7.22E-05 | 0.000719193 | down |
| WASH7P | -0.609769715 | 0.005184403 | 0.022296048 | down |
| PRUNE2 | -0.609683037 | 0.046613287 | 0.116104261 | down |
| DHRS7B | -0.609589896 | 2.72E-05 | 0.000322463 | down |
| RP11-324I22.4 | -0.609578431 | 0.053480189 | 0.128355872 | down |
| RP11-678G14.2 | -0.609572283 | 0.354429744 | | down |
| TCL6 | -0.609567849 | 0.253160549 | 0.388090903 | down |

| | | | | |
|---|---|---|---|---|
| NPY5R | -0.609558216 | 0.374612134 | 0.512936239 | down |
| RP11-702B10.2 | -0.609485229 | 0.421631555 | | down |
| LSMEM1 | -0.60939093 | 0.024740655 | 0.072716517 | down |
| AC007952.5 | -0.609203347 | 0.202863638 | 0.332270356 | down |
| NA | -0.60920288 | 0.024983949 | 0.073259591 | down |
| UBXN10 | -0.609152788 | 0.011039243 | 0.039563763 | down |
| FOXB1 | -0.608946622 | 0.361852536 | 0.500137903 | down |
| GCGR | -0.608922188 | 0.126510786 | 0.238704393 | down |
| PTP4A2P1 | -0.608820556 | 0.142268098 | 0.259141592 | down |
| USP44 | -0.608716785 | 0.145154702 | 0.262807513 | down |
| RP11-134N1.2 | -0.608640443 | 0.479848158 | 0.609218984 | down |
| RP11-650L12.2 | -0.60859614 | 0.143049981 | 0.26017382 | down |
| ZNF408 | -0.608550307 | 0.000708983 | 0.004608542 | down |
| ARRDC1 | -0.608443921 | 0.006024833 | 0.025035975 | down |
| ING2 | -0.608375787 | 0.003230888 | 0.015340692 | down |
| TRBV10-1 | -0.60832599 | 0.483466633 | | down |
| RTEL1 | -0.608310765 | 0.181940438 | 0.307453655 | down |
| AC010761.13 | -0.608292163 | 0.36231495 | 0.500684898 | down |
| RP11-710F7.3 | -0.607981227 | 0.458005095 | | down |
| RPS3AP17 | -0.607901347 | 0.835409081 | | down |
| RP11-191A15.2 | -0.607901347 | 0.835409081 | | down |
| RCN1P1 | -0.607901347 | 0.835409081 | | down |
| RSU1P1 | -0.607901347 | 0.835409081 | | down |
| OR5D16 | -0.607901347 | 0.835409081 | | down |
| NA | -0.607901347 | 0.835409081 | | down |
| RN7SL483P | -0.607901347 | 0.835409081 | | down |
| EIF4EBP2P2 | -0.607901347 | 0.835409081 | | down |
| RP11-42H13.1 | -0.607901347 | 0.835409081 | | down |
| MIR4316 | -0.607901347 | 0.835409081 | | down |
| TAF9P2 | -0.607901347 | 0.835409081 | | down |
| RNY1P12 | -0.607901346 | 0.835318767 | | down |
| NA | -0.607901325 | 0.833914947 | | down |
| SNORD62A | -0.607901311 | 0.832922484 | | down |
| SNORA36 | -0.607901291 | 0.831549715 | | down |
| RP11-151H2.3 | -0.607901276 | 0.830447749 | | down |
| RN7SL492P | -0.607901272 | 0.830171839 | | down |
| RNU6-557P | -0.607901269 | 0.829888525 | | down |
| RP11-626E13.1 | -0.607901028 | 0.808821315 | | down |
| IGHVIII-2-1 | -0.607901026 | 0.80859532 | | down |
| GGTA2P | -0.607901022 | 0.808216786 | | down |
| GXYLT1P3 | -0.607901014 | 0.807347882 | | down |
| RP4-631H13.4 | -0.607900977 | 0.803274 | | down |
| AC004543.2 | -0.607900824 | 0.783009435 | | down |
| RP11-362K2.2 | -0.607900824 | 0.783025159 | | down |
| PTTG4P | -0.607900819 | 0.782264836 | | down |
| SUMO2P15 | -0.607900818 | 0.782162445 | | down |
| RP11-383G10.5 | -0.607900814 | 0.781583694 | | down |
| AC073869.2 | -0.607900814 | 0.781590235 | | down |
| NA | -0.607900804 | 0.779995399 | | down |
| RP11-311F12.2 | -0.607900788 | 0.777304515 | | down |
| RP11-259P20.1 | -0.607900678 | 0.756656551 | | down |

| | | | | |
|---|---|---|---|---|
| RNU6-212P | -0.607900673 | 0.755486901 | | down |
| Metazoa_SRP | -0.607900668 | 0.7544038 | | down |
| CH17-140K24.6 | -0.60790058 | 0.732146997 | | down |
| CCT5P1 | -0.607900575 | 0.73063614 | | down |
| RN7SL738P | -0.607900571 | 0.729676681 | | down |
| AC091492.2 | -0.607900497 | 0.704592001 | | down |
| RNA5SP301 | -0.607900487 | 0.700767217 | | down |
| RP4-738P11.3 | -0.607900438 | 0.678834204 | | down |
| RP11-648015.1 | -0.607900436 | 0.677927394 | | down |
| HINT1P1 | -0.607900426 | 0.672732308 | | down |
| MCCD1P1 | -0.607900385 | 0.648981305 | | down |
| CTB-179K24.3 | -0.607476418 | 0.149683149 | 0.268614675 | down |
| RP11-130L8.3 | -0.607269513 | 0.114976341 | 0.223206584 | down |
| RP11-480A16.1 | -0.607119914 | 0.035264136 | 0.094520092 | down |
| NA | -0.60668738 | 0.607715479 | | down |
| SMIM17 | -0.60654583 | 0.229431143 | 0.361855385 | down |
| NLRP4 | -0.60618268 | 0.412084346 | 0.548853257 | down |
| NA | -0.606178674 | 0.43697663 | | down |
| TUBB3 | -0.606009978 | 0.333026022 | 0.471354116 | down |
| AF131215.8 | -0.605798116 | 0.273882251 | 0.410195332 | down |
| RP11-294N21.3 | -0.605729316 | 0.452119673 | | down |
| RP1-256G22.2 | -0.605664361 | 0.488855204 | 0.617445065 | down |
| LINC01331 | -0.605617855 | 0.516824252 | 0.64179837 | down |
| ZNF850 | -0.605580221 | 0.017351471 | 0.055725024 | down |
| GSDMD | -0.605513153 | 0.007877125 | 0.030619789 | down |
| CTB-180A7.3 | -0.605386526 | 0.5903777 | | down |
| SGK1 | -0.60533116 | 0.032363135 | 0.088674694 | down |
| SNORD73B | -0.605280989 | 0.593583352 | | down |
| RPL13 | -0.605017616 | 0.00702402 | 0.028084863 | down |
| RP1-317E23.3 | -0.604981929 | 0.132306209 | 0.246313529 | down |
| RPL7AP60 | -0.604806896 | 0.669913442 | | down |
| IFT122 | -0.604697785 | 0.000357073 | 0.002679968 | down |
| CTC-490E21.10 | -0.604542242 | 0.099833299 | 0.201680449 | down |
| NA | -0.604517593 | 0.692956386 | | down |
| CYP51A1 | -0.604467227 | 0.276151599 | 0.412442764 | down |
| DUXAP3 | -0.60436613 | 0.767992443 | 0.844031588 | down |
| C9orf142 | -0.604123567 | 0.013459629 | 0.045952398 | down |
| TOR1AIP2 | -0.604068185 | 1.11E-07 | 3.09E-06 | down |
| TP53TG5 | -0.604054743 | 0.168159886 | 0.291528886 | down |
| NA | -0.604053929 | 0.133290011 | 0.247391774 | down |
| ISPD | -0.604040903 | 0.045369667 | 0.113856903 | down |
| MT1XP1 | -0.603731238 | 0.182215643 | 0.307806701 | down |
| MYO1C | -0.603676717 | 4.18E-05 | 0.000460065 | down |
| CEP170B | -0.60363488 | 0.0057104 | 0.024008293 | down |
| OGFRP1 | -0.60336915 | 0.012378512 | 0.043164604 | down |
| VIPR2 | -0.603366439 | 0.038794342 | 0.101518789 | down |
| GPS1 | -0.603352334 | 0.002899553 | 0.014121268 | down |
| PAX8 | -0.602998791 | 0.008104728 | 0.031225757 | down |
| AC006946.17 | -0.602875943 | 0.388929135 | 0.526540506 | down |
| GUCA1B | -0.602854809 | 0.028784677 | 0.081439574 | down |
| PARP12 | -0.60254586 | 0.003247195 | 0.015404818 | down |

| | | | | |
|---|---|---|---|---|
| KLRC1 | -0.602462677 | 0.107056951 | 0.212288683 | down |
| RP11-535A19.2 | -0.602413606 | 0.344840272 | 0.483384553 | down |
| RP11-195C7.1 | -0.602319658 | 0.259881461 | 0.395075576 | down |
| RP4-745E8.2 | -0.601993195 | 0.463151091 | 0.594844473 | down |
| TNRC18P1 | -0.601948199 | 0.307225081 | 0.445525702 | down |
| AKAP5 | -0.601867961 | 0.012028078 | 0.042270627 | down |
| TVP23A | -0.601662529 | 0.069047515 | 0.154741424 | down |
| RP1-153P14.5 | -0.601426757 | 0.406136031 | 0.542682074 | down |
| ZNF844 | -0.6013429 | 0.001321159 | 0.007549048 | down |
| THAP7 | -0.601277545 | 0.008591281 | 0.032644041 | down |
| AC012360.6 | -0.601222961 | 0.27415332 | 0.410519455 | down |
| PLCG1-AS1 | -0.601222433 | 0.123517889 | 0.2347379 | down |
| FKBPL | -0.601066362 | 0.005510319 | 0.023346782 | down |
| RP11-33E12.2 | -0.601013388 | 0.105518423 | 0.210002352 | down |
| RP11-426J5.3 | -0.601010079 | 0.281187312 | 0.41757657 | down |
| RP11-428O18.6 | -0.6010021 | 0.211059764 | 0.341574463 | down |
| NA | -0.600973111 | 0.399595861 | | down |
| RBAKDN | -0.600873199 | 0.392258095 | | down |
| CEP76 | -0.600829009 | 0.030921392 | 0.085693415 | down |
| CTD-2240J17.4 | -0.600811958 | 0.246003225 | 0.380074098 | down |
| TRAJ31 | -0.600775564 | 0.29503362 | 0.432474146 | down |
| B4GALT4 | -0.600449838 | 0.000602369 | 0.004045053 | down |
| RP11-439L18.1 | -0.600332919 | 0.18335726 | 0.309133914 | down |
| DOPEY2 | -0.600100921 | 0.003094527 | 0.014856498 | down |
| GIPC1 | -0.59981433 | 0.02276893 | 0.068280891 | down |
| RAD21-AS1 | -0.599798828 | 0.330785373 | 0.469155254 | down |
| LPAR3 | -0.599776667 | 0.200776021 | 0.329840047 | down |
| SLC2A9 | -0.599605217 | 0.009858072 | 0.036229469 | down |
| CLEC4C | -0.599600568 | 0.302679521 | 0.440607758 | down |
| SCP2 | -0.599533818 | 0.048436214 | 0.119633147 | down |
| CLEC2L | -0.599467569 | 0.111159696 | 0.218019115 | down |
| RP6-109B7.2 | -0.599382791 | 0.193514339 | 0.321638903 | down |
| RP11-936I5.1 | -0.599352176 | 0.379308618 | 0.51733921 | down |
| C5orf17 | -0.599008386 | 0.510319396 | 0.636184063 | down |
| DNMBP | -0.59899973 | 0.001137155 | 0.006725331 | down |
| OCM | -0.598971188 | 0.177262418 | 0.302217233 | down |
| TRIM31-AS1 | -0.598937311 | 0.296564891 | 0.433956535 | down |
| ATP6V0CP3 | -0.598914367 | 0.309976401 | 0.448534942 | down |
| NFKBIL1 | -0.598737729 | 0.006281139 | 0.025815397 | down |
| HCN3 | -0.598673255 | 0.117690387 | 0.226970115 | down |
| NA | -0.598658691 | 0.625341082 | | down |
| EPM2A | -0.598574503 | 0.019492778 | 0.060852143 | down |
| NA | -0.598492297 | 0.837920995 | | down |
| RP11-10I6.2 | -0.598492297 | 0.837920995 | | down |
| BX322559.3 | -0.598492297 | 0.837920995 | | down |
| IGHV3-30-2 | -0.598492023 | 0.811811873 | | down |
| MRPL53P1 | -0.598491993 | 0.808117889 | | down |
| CTD-2201E9.2 | -0.598491992 | 0.807892716 | | down |
| RP11-17M15.2 | -0.59849198 | 0.806449498 | | down |
| DDX39BP1 | -0.598491844 | 0.78581634 | | down |
| RP1-300G12.2 | -0.598491844 | 0.785849253 | | down |

| | | | | |
|---|---|---|---|---|
| NA | -0.598491843 | 0.785566311 | | down |
| RP11-479F13.1 | -0.598491835 | 0.784196901 | | down |
| RP11-172E10.1 | -0.598491834 | 0.784077693 | | down |
| RP11-650J17.1 | -0.598491828 | 0.782923326 | | down |
| SNORA51 | -0.598491809 | 0.779300755 | | down |
| OR5AK4P | -0.598491724 | 0.760939492 | | down |
| RP11-37606.2 | -0.598491716 | 0.759020924 | | down |
| RPL7P7 | -0.598491708 | 0.756821618 | | down |
| SLC6A21P | -0.5984917 | 0.754720902 | | down |
| ARF1P2 | -0.598491698 | 0.754297926 | | down |
| CTD-2339M3.1 | -0.598491692 | 0.752589579 | | down |
| VN1R51P | -0.598491631 | 0.734293577 | | down |
| MC2R | -0.598491562 | 0.707381737 | | down |
| CYB561D1 | -0.598467724 | 1.06E-07 | 2.97E-06 | down |
| POLR2J | -0.598347299 | 0.006974578 | 0.027926484 | down |
| RP11-641D5.1 | -0.5981943 | 0.002540524 | 0.012723702 | down |
| AC007036.6 | -0.59819008 | 0.206663708 | 0.336721633 | down |
| ADGRF2 | -0.598038337 | 0.631647616 | 0.739421032 | down |
| PRMT7 | -0.597947579 | 0.002036429 | 0.010654922 | down |
| BNIP3P1 | -0.597880894 | 0.521653476 | | down |
| STEAP3-AS1 | -0.597852402 | 0.431483844 | | down |
| MGARP | -0.59751742 | 0.266518048 | 0.402213594 | down |
| KLHL35 | -0.597329976 | 0.040295376 | 0.104500154 | down |
| MAPRE3 | -0.597004028 | 0.011490661 | 0.040816438 | down |
| CEP170P1 | -0.59688172 | 0.483543891 | 0.612590877 | down |
| LACE1 | -0.596880663 | 0.000789437 | 0.005023906 | down |
| RPS4XP11 | -0.596818872 | 0.178465825 | 0.303562659 | down |
| MIB2 | -0.596816015 | 0.000701603 | 0.004575423 | down |
| RPS3P6 | -0.596442303 | 0.110714308 | 0.217348449 | down |
| ADHFE1 | -0.596395363 | 0.057260531 | 0.135034175 | down |
| LRP5 | -0.596295867 | 0.006267592 | 0.02578745 | down |
| RPL37P2 | -0.59626035 | 0.071851849 | 0.159350184 | down |
| CENPUP2 | -0.596042785 | 0.538813442 | 0.66084712 | down |
| FAM222A | -0.595945258 | 0.006094955 | 0.025257492 | down |
| ERAP2 | -0.59587264 | 0.094762906 | 0.194564119 | down |
| PTGES2 | -0.595747879 | 0.00167642 | 0.009116083 | down |
| FFAR3 | -0.595706358 | 0.132481871 | 0.24652955 | down |
| METTL26 | -0.595684021 | 0.015338735 | 0.050820133 | down |
| SCAP | -0.5956616 | 0.000105193 | 0.000987634 | down |
| MTND4LP30 | -0.595611781 | 0.254657414 | 0.389677465 | down |
| RP11-753H16.3 | -0.595611649 | 0.322625527 | 0.461196237 | down |
| RP11-485M7.1 | -0.595581313 | 0.355726534 | 0.494299697 | down |
| PRELID3BP8 | -0.595478781 | 0.805445086 | | down |
| RP11-702H23.4 | -0.59516142 | 0.499877383 | | down |
| RAD9B | -0.595019443 | 0.192472258 | 0.320385424 | down |
| PHOSPHO2 | -0.594971807 | 0.01416125 | 0.047810912 | down |
| ATOX1 | -0.594906914 | 0.05025139 | 0.122765296 | down |
| NXF3 | -0.594833393 | 0.229539075 | 0.361888492 | down |
| C11orf42 | -0.594782991 | 0.506243076 | | down |
| MIR661 | -0.594650113 | 0.609954218 | | down |
| RP11-413H22.3 | -0.594569397 | 0.404414922 | 0.541018065 | down |

| | | | | |
|---|---|---|---|---|
| SERAC1 | -0.594443503 | 0.001094188 | 0.006519991 | down |
| RP11-702F3.3 | -0.594306951 | 0.657558566 | 0.75974655 | down |
| RP11-1348G14.8 | -0.594255311 | 0.078294016 | 0.169505367 | down |
| RP11-157J24.2 | -0.594225884 | 0.171371729 | 0.295358664 | down |
| MRPL11 | -0.594172488 | 0.001428291 | 0.008026859 | down |
| LINC01006 | -0.594130629 | 0.004333947 | 0.019395734 | down |
| EPB41L5 | -0.59411689 | 0.002977098 | 0.014417137 | down |
| RP11-345P4.6 | -0.594078794 | 0.160729671 | 0.282213911 | down |
| RP11-679B17.2 | -0.593795054 | 0.657267196 | | down |
| RPL18 | -0.593738995 | 0.012885907 | 0.044483994 | down |
| ALG14 | -0.593683529 | 0.001679391 | 0.009126279 | down |
| ZNF546 | -0.59357942 | 0.009290162 | 0.034668193 | down |
| RP11-12M5.3 | -0.593488239 | 0.179962785 | 0.305227611 | down |
| C1GALT1P1 | -0.593483976 | 0.298883525 | 0.436435316 | down |
| RP11-603B24.2 | -0.593480743 | 0.744439655 | | down |
| RP11-196G18.3 | -0.593385295 | 0.57137511 | 0.688875184 | down |
| RP11-1415C14.4 | -0.593140193 | 0.512981056 | | down |
| UNC5C | -0.59278701 | 0.017849328 | 0.057027388 | down |
| IQGAP3 | -0.592690555 | 0.028592165 | 0.08107815 | down |
| RP11-100E13.1 | -0.59266814 | 0.523094589 | 0.647302353 | down |
| AC108479.2 | -0.592650222 | 0.411339838 | 0.548015347 | down |
| GHc-857G6.7 | -0.592570449 | 0.371160863 | 0.509534246 | down |
| RP11-480D4.6 | -0.592422032 | 0.229196403 | 0.361639448 | down |
| NA | -0.592397467 | 0.196961573 | 0.325585336 | down |
| RP11-45A17.4 | -0.592338994 | 0.361800917 | 0.500089563 | down |
| MTND4P35 | -0.592291067 | 0.296362819 | 0.433787612 | down |
| WNT16 | -0.592243138 | 0.298763276 | 0.436323345 | down |
| EMC10 | -0.592221877 | 0.00013627 | 0.001221753 | down |
| FBXW11P1 | -0.592218136 | 0.501639574 | | down |
| KCNJ12 | -0.592134861 | 0.111982147 | 0.219092968 | down |
| RP11-549J18.1 | -0.592129685 | 0.08048689 | 0.173006486 | down |
| RP13-977J11.8 | -0.592118733 | 0.387159764 | 0.524878298 | down |
| TRIM60P14 | -0.592089473 | 0.69202993 | | down |
| A1BG | -0.592053134 | 0.317354605 | 0.456070111 | down |
| ANGPTL4 | -0.591759551 | 0.053062026 | 0.127576465 | down |
| NA | -0.591208528 | 0.590400129 | | down |
| AC018766.6 | -0.590900636 | 0.387581483 | 0.525158617 | down |
| CACNG3 | -0.59075577 | 0.512579775 | | down |
| E2F2 | -0.590381642 | 0.00967783 | 0.035728768 | down |
| AC074011.2 | -0.590308389 | 0.397515507 | 0.534527856 | down |
| CYHR1 | -0.590291356 | 0.008373943 | 0.032012566 | down |
| FRMD5 | -0.590259455 | 0.062246374 | 0.143624151 | down |
| XX-C283C717.1 | -0.590159154 | 0.609678216 | | down |
| RP1-310O13.7 | -0.589988093 | 0.321261749 | 0.460034698 | down |
| ARHGEF35 | -0.589868325 | 0.058095921 | 0.136564733 | down |
| TMEM164 | -0.589668518 | 4.91E-05 | 0.00052789 | down |
| LDLRAP1 | -0.589527037 | 8.02E-05 | 0.000788023 | down |
| MST1 | -0.589507895 | 0.061059743 | 0.14160393 | down |
| MIPOL1 | -0.589503881 | 0.042495085 | 0.108712732 | down |
| PPFIBP2 | -0.589323924 | 6.36E-08 | 1.94E-06 | down |
| RP11-227D13.4 | -0.589294985 | 0.478213023 | | down |

| | | | | |
|---|---|---|---|---|
| LINC01239 | -0.589250408 | 0.318810324 | 0.457599905 | down |
| HPR | -0.589186757 | 0.245934203 | 0.380006541 | down |
| SLC25A3P1 | -0.589166407 | 0.785006861 | | down |
| SNORA33 | -0.589083247 | 0.840434561 | | down |
| RP11-56P9.11 | -0.589083247 | 0.840434561 | | down |
| RP11-66H6.3 | -0.589083197 | 0.835734971 | | down |
| ACTG1P16 | -0.589083161 | 0.832088249 | | down |
| OR2T11 | -0.58908314 | 0.829810016 | | down |
| CTD-2272D18.1 | -0.589083138 | 0.829617844 | | down |
| RP11-320G10.1 | -0.589083015 | 0.814267031 | | down |
| CDY4P | -0.589082966 | 0.806975591 | | down |
| AP001604.3 | -0.589082874 | 0.790499921 | | down |
| PMS2P11 | -0.589082696 | 0.740975891 | | down |
| RP11-203H2.2 | -0.589082689 | 0.73828511 | | down |
| RP11-25E2.1 | -0.589082677 | 0.73360973 | | down |
| AC004066.3 | -0.589082638 | 0.715323957 | | down |
| AC003985.1 | -0.589082632 | 0.712213647 | | down |
| ATP5G1P1 | -0.589082628 | 0.710222593 | | down |
| DDX3P1 | -0.589082592 | 0.688906292 | | down |
| NA | -0.589082559 | 0.664395043 | | down |
| RP11-214K3.20 | -0.58908249 | 0.586092502 | | down |
| SNX24 | -0.588995651 | 0.000836318 | 0.005259855 | down |
| RPL34P34 | -0.588991398 | 0.191633848 | 0.319360361 | down |
| BCLAF1P2 | -0.58889943 | 0.436359882 | | down |
| TUBB8P7 | -0.588635638 | 0.441917434 | 0.575570675 | down |
| AC111155.1 | -0.588576619 | 0.5318836 | | down |
| DNAJC28 | -0.588471934 | 0.037949138 | 0.099881201 | down |
| CCDC136 | -0.588039418 | 0.027095552 | 0.077870082 | down |
| PRRT2 | -0.588030451 | 0.026669277 | 0.076918326 | down |
| RP11-283G6.3 | -0.588001221 | 0.160847994 | 0.282372159 | down |
| STX3 | -0.587962335 | 0.000977266 | 0.00594352 | down |
| RP11-490K7.4 | -0.58794334 | 0.43094176 | 0.565973949 | down |
| NANOS2 | -0.587661202 | 0.840783169 | | down |
| IL17C | -0.587472076 | 0.288188777 | 0.425471431 | down |
| KIAA0825 | -0.587434869 | 0.207287906 | 0.337419833 | down |
| RP11-715J22.1 | -0.587391116 | 0.313344307 | 0.451993295 | down |
| RP11-95M15.1 | -0.587315931 | 0.363604913 | 0.502028768 | down |
| USP49 | -0.587263296 | 0.039287714 | 0.102550832 | down |
| PHACTR1 | -0.587029622 | 0.022307163 | 0.067255033 | down |
| SPIB | -0.586671081 | 0.050604408 | 0.1233395 | down |
| NA | -0.586583754 | 0.348199454 | 0.486981808 | down |
| C19orf60 | -0.586507498 | 0.011989495 | 0.042180774 | down |
| NDUFS3 | -0.586438486 | 0.001017306 | 0.006148447 | down |
| HIGD2A | -0.586351326 | 0.014457957 | 0.048538926 | down |
| NA | -0.58616762 | 0.544504205 | 0.665950919 | down |
| AC062028.1 | -0.586058277 | 0.325993553 | 0.464441877 | down |
| NA | -0.585726981 | 0.463595728 | 0.595174346 | down |
| C7 | -0.585631668 | 0.070427712 | 0.157049668 | down |
| NA | -0.585524177 | 0.454423775 | 0.586960786 | down |
| COX6A1 | -0.585501662 | 0.031517135 | 0.086975367 | down |
| CPLX2 | -0.585318334 | 0.083112383 | 0.177116973 | down |

| | | | | |
|---|---|---|---|---|
| TMEM9B-AS1 | -0.585258293 | 0.062292508 | 0.143697489 | down |
| ZNF396 | -0.585182577 | 0.022957466 | 0.06866442 | down |
| GOLIM4 | -0.585098956 | 0.032521684 | 0.089019814 | down |
| COX5A | -0.585055581 | 0.001677899 | 0.009120827 | down |
| ZNF571-AS1 | -0.584962224 | 0.091492785 | 0.189657009 | down |
| RP11-1070N10.7 | -0.584659787 | 0.592383087 | | down |
| STAG2 | -0.584544094 | 0.000156768 | 0.001367977 | down |
| TMEM177 | -0.584343299 | 0.011187427 | 0.039964173 | down |
| NA | -0.5842981 | 0.308812508 | 0.4473032 | down |
| TCF25 | -0.584211507 | 0.002081536 | 0.010830909 | down |
| RP11-793H13.11 | -0.583994141 | 0.048013798 | 0.118746011 | down |
| NA | -0.583918507 | 0.377950762 | 0.516049478 | down |
| COQ3 | -0.583827348 | 0.001252747 | 0.007250534 | down |
| ZNF444 | -0.583799901 | 0.007286573 | 0.028835325 | down |
| TMEM246 | -0.58379254 | 0.036913143 | 0.097796766 | down |
| ARHGEF11 | -0.583686915 | 0.007415768 | 0.029238759 | down |
| PDGFRL | -0.583408525 | 0.140332007 | 0.256783898 | down |
| HPCAL4 | -0.58325346 | 0.219718351 | 0.351222034 | down |
| CD96 | -0.583074611 | 0.021120217 | 0.06464327 | down |
| FTLP17 | -0.583042204 | 0.521904102 | | down |
| NRL | -0.58297927 | 0.009801309 | 0.03606056 | down |
| RP13-225021.2 | -0.582883385 | 0.36653023 | 0.505069679 | down |
| FAM166AP3 | -0.582812462 | 0.842110666 | | down |
| BC14-118882207. | -0.582812462 | 0.842110666 | | down |
| NA | -0.582812462 | 0.842110666 | | down |
| RNU6-1022P | -0.582812462 | 0.842110666 | | down |
| NFU1P2 | -0.582812462 | 0.842110666 | | down |
| LCE2B | -0.582812462 | 0.842110666 | | down |
| LCE1B | -0.582812462 | 0.842110666 | | down |
| AIMP1P2 | -0.582812462 | 0.842110666 | | down |
| RP3-419C19.3 | -0.582812462 | 0.842110666 | | down |
| NA | -0.582812462 | 0.842110666 | | down |
| RNU6-968P | -0.582812462 | 0.842110666 | | down |
| AC116609.3 | -0.582812462 | 0.842110666 | | down |
| AC007131.2 | -0.582812462 | 0.842110666 | | down |
| Y_RNA | -0.582812462 | 0.842110666 | | down |
| RSL24D1P2 | -0.582812462 | 0.842110666 | | down |
| TRIM51JP | -0.582812462 | 0.842110666 | | down |
| NMS | -0.582812462 | 0.842110666 | | down |
| RP11-426K3.1 | -0.582812462 | 0.842110666 | | down |
| AC108051.1 | -0.582812462 | 0.842110666 | | down |
| AC023347.2 | -0.582812462 | 0.842110666 | | down |
| RNU6-268P | -0.582812462 | 0.842110666 | | down |
| RNU1-93P | -0.582812462 | 0.842110666 | | down |
| NA | -0.582812462 | 0.842110666 | | down |
| AC105461.1 | -0.582812462 | 0.842110666 | | down |
| Y_RNA | -0.582812462 | 0.842110666 | | down |
| GRM7-AS2 | -0.582812462 | 0.842110666 | | down |
| RNU6-511P | -0.582812462 | 0.842110666 | | down |
| RP11-17403.4 | -0.582812462 | 0.842110666 | | down |
| RN7SL715P | -0.582812462 | 0.842110666 | | down |

| | | | |
|---|---|---|---|
| OTOL1 | -0.582812462 | 0.842110666 | down |
| RP11-809F4.3 | -0.582812462 | 0.842110666 | down |
| RNU6-4P | -0.582812462 | 0.842110666 | down |
| RN7SL73P | -0.582812462 | 0.842110666 | down |
| ECM1P2 | -0.582812462 | 0.842110666 | down |
| RP11-576E20.1 | -0.582812462 | 0.842110666 | down |
| RP11-415C15.1 | -0.582812462 | 0.842110666 | down |
| RP11-530I17.2 | -0.582812462 | 0.842110666 | down |
| RP11-790I12.3 | -0.582812462 | 0.842110666 | down |
| RNU6-1187P | -0.582812462 | 0.842110666 | down |
| HNRNPA1P56 | -0.582812462 | 0.842110666 | down |
| KRT18P51 | -0.582812462 | 0.842110666 | down |
| AKIRIN2P1 | -0.582812462 | 0.842110666 | down |
| RP11-91J3.2 | -0.582812462 | 0.842110666 | down |
| CTD-2345H1.1 | -0.582812462 | 0.842110666 | down |
| RP11-506H20.2 | -0.582812462 | 0.842110666 | down |
| NA | -0.582812462 | 0.842110666 | down |
| RNU6-448P | -0.582812462 | 0.842110666 | down |
| CTD-2285G11.1 | -0.582812462 | 0.842110666 | down |
| CTC-286N12.1 | -0.582812462 | 0.842110666 | down |
| SNORA57 | -0.582812462 | 0.842110666 | down |
| RPS15AP18 | -0.582812462 | 0.842110666 | down |
| RP1-59B16.1 | -0.582812462 | 0.842110666 | down |
| OR2B7P | -0.582812462 | 0.842110666 | down |
| RP11-349P19.1 | -0.582812462 | 0.842110666 | down |
| RN7SL509P | -0.582812462 | 0.842110666 | down |
| RP1-76C18.1 | -0.582812462 | 0.842110666 | down |
| CTA-444M12.3 | -0.582812462 | 0.842110666 | down |
| RP11-377H23.1 | -0.582812462 | 0.842110666 | down |
| HMGN1P19 | -0.582812462 | 0.842110666 | down |
| RP11-760D2.13 | -0.582812462 | 0.842110666 | down |
| MTND4P2 | -0.582812462 | 0.842110666 | down |
| HINT1P2 | -0.582812462 | 0.842110666 | down |
| MIR183 | -0.582812462 | 0.842110666 | down |
| TAS2R41 | -0.582812462 | 0.842110666 | down |
| CTD-2336O2.4 | -0.582812462 | 0.842110666 | down |
| MIR1322 | -0.582812462 | 0.842110666 | down |
| RN7SL709P | -0.582812462 | 0.842110666 | down |
| RP11-573J24.1 | -0.582812462 | 0.842110666 | down |
| RP11-48D4.2 | -0.582812462 | 0.842110666 | down |
| REXO1L8P | -0.582812462 | 0.842110666 | down |
| RP11-386D6.2 | -0.582812462 | 0.842110666 | down |
| MRPS16P1 | -0.582812462 | 0.842110666 | down |
| RP11-737F9.1 | -0.582812462 | 0.842110666 | down |
| RNU1-35P | -0.582812462 | 0.842110666 | down |
| SNX18P7 | -0.582812462 | 0.842110666 | down |
| RP11-350E12.6 | -0.582812462 | 0.842110666 | down |
| RP11-173G21.1 | -0.582812462 | 0.842110666 | down |
| RP11-508N12.3 | -0.582812462 | 0.842110666 | down |
| RNU6-1020P | -0.582812462 | 0.842110666 | down |
| MIR199B | -0.582812462 | 0.842110666 | down |

| | | | |
|---|---|---|---|
| RP11-573G6.9 | -0.582812462 | 0.842110666 | down |
| RNU6-908P | -0.582812462 | 0.842110666 | down |
| RP11-391J2.3 | -0.582812462 | 0.842110666 | down |
| NACAP2 | -0.582812462 | 0.842110666 | down |
| NA | -0.582812462 | 0.842110666 | down |
| snoMBII-202 | -0.582812462 | 0.842110666 | down |
| LINC01495 | -0.582812462 | 0.842110666 | down |
| OR4C4P | -0.582812462 | 0.842110666 | down |
| OR5B19P | -0.582812462 | 0.842110666 | down |
| RN7SL435P | -0.582812462 | 0.842110666 | down |
| OR5A2 | -0.582812462 | 0.842110666 | down |
| RP11-867G2.5 | -0.582812462 | 0.842110666 | down |
| RP11-89C3.2 | -0.582812462 | 0.842110666 | down |
| RP11-267D19.1 | -0.582812462 | 0.842110666 | down |
| SNORA64 | -0.582812462 | 0.842110666 | down |
| RP11-274M17.2 | -0.582812462 | 0.842110666 | down |
| RP11-411G2.1 | -0.582812462 | 0.842110666 | down |
| RPL27P12 | -0.582812462 | 0.842110666 | down |
| NA | -0.582812462 | 0.842110666 | down |
| RP11-629E24.2 | -0.582812462 | 0.842110666 | down |
| RP13-444H2.1 | -0.582812462 | 0.842110666 | down |
| RN7SL375P | -0.582812462 | 0.842110666 | down |
| CALM2P4 | -0.582812462 | 0.842110666 | down |
| LINC00399 | -0.582812462 | 0.842110666 | down |
| MIR4706 | -0.582812462 | 0.842110666 | down |
| CTD-2014B16.1 | -0.582812462 | 0.842110666 | down |
| RP11-232C2.2 | -0.582812462 | 0.842110666 | down |
| RP11-804L24.2 | -0.582812462 | 0.842110666 | down |
| RP11-12016.1 | -0.582812462 | 0.842110666 | down |
| RNU6-796P | -0.582812462 | 0.842110666 | down |
| Y_RNA | -0.582812462 | 0.842110666 | down |
| SLX1B | -0.582812462 | 0.842110666 | down |
| ABCD1P3 | -0.582812462 | 0.842110666 | down |
| RP11-989E6.11 | -0.582812462 | 0.842110666 | down |
| AGGF1P8 | -0.582812462 | 0.842110666 | down |
| RP11-55L4.2 | -0.582812462 | 0.842110666 | down |
| RP11-1113L8.2 | -0.582812462 | 0.842110666 | down |
| PRELID3BP3 | -0.582812462 | 0.842110666 | down |
| RP11-269G24.3 | -0.582812462 | 0.842110666 | down |
| RP11-797E24.1 | -0.582812462 | 0.842110666 | down |
| RNU1-46P | -0.582812462 | 0.842110666 | down |
| RP11-202D1.3 | -0.582812462 | 0.842110666 | down |
| RP11-775G23.1 | -0.582812462 | 0.842110666 | down |
| RN7SL231P | -0.582812462 | 0.842110666 | down |
| CYP4F9P | -0.582812462 | 0.842110666 | down |
| MIR1323 | -0.582812462 | 0.842110666 | down |
| RP4-726N1.2 | -0.582812462 | 0.842110666 | down |
| NA | -0.582812462 | 0.842110666 | down |
| IMMTP1 | -0.582812462 | 0.842110666 | down |
| AC002472.11 | -0.582812462 | 0.842110666 | down |
| HAUS1P2 | -0.582812462 | 0.842110666 | down |

| | | | | |
|---|---|---|---|---|
| RP11-571E6.4 | -0.582812462 | 0.842110666 | | down |
| RP11-236P24.1 | -0.582812462 | 0.842110666 | | down |
| BTF3P8 | -0.582812462 | 0.842110666 | | down |
| FRMPD3-AS1 | -0.582812462 | 0.842110666 | | down |
| SNORD30 | -0.582812462 | 0.842110666 | | down |
| WNK4 | -0.582788955 | 0.053072785 | 0.127592123 | down |
| COX7B | -0.582594887 | 0.00023213 | 0.001890516 | down |
| SELO | -0.582524712 | 0.005792146 | 0.024273851 | down |
| RHBDL3 | -0.582506936 | 0.12866647 | 0.241527987 | down |
| AMOT | -0.58226781 | 0.006712231 | 0.027141622 | down |
| MIR5480 | -0.582257703 | 0.391627306 | 0.529215615 | down |
| NPNT | -0.581978936 | 0.050601615 | 0.1233395 | down |
| LA16c-380F5.1 | -0.581868201 | 0.492717866 | 0.620862122 | down |
| RP11-88H9.2 | -0.581751873 | 0.157215672 | 0.277895374 | down |
| PHACTR2P1 | -0.581635025 | 0.601466122 | | down |
| NAPSA | -0.581566678 | 0.091889401 | 0.190289341 | down |
| RP13-565O16.2 | -0.58151915 | 0.68358783 | | down |
| ZNF37CP | -0.581471971 | 0.569784806 | | down |
| KRT17P1 | -0.581376249 | 0.379804663 | 0.517733725 | down |
| ACSM4 | -0.581336425 | 0.353854464 | 0.49254393 | down |
| NA | -0.581033977 | 0.618901382 | | down |
| GPSM2 | -0.58102677 | 0.011744246 | 0.041503109 | down |
| TSSC1-IT1 | -0.581024157 | 0.731275189 | | down |
| CTC-412M14.5 | -0.58085601 | 0.567623695 | | down |
| DLG5-AS1 | -0.580707017 | 0.201063747 | 0.330114171 | down |
| PGAP2 | -0.580628292 | 0.000433284 | 0.003100898 | down |
| FAM3C2 | -0.580487537 | 0.268286048 | 0.404082042 | down |
| RP11-37N22.1 | -0.580425307 | 0.432753501 | 0.567707894 | down |
| RP11-218E20.3 | -0.580339073 | 0.317654374 | 0.456435412 | down |
| NA | -0.580283894 | 0.215103474 | 0.346165771 | down |
| RP11-290F5.2 | -0.579910479 | 0.085304884 | 0.180524049 | down |
| RP11-211C9.1 | -0.579882795 | 0.486965056 | 0.615678918 | down |
| RP1-37N7.1 | -0.579674198 | 0.842949756 | | down |
| RP11-401F2.2 | -0.579674127 | 0.834580241 | | down |
| CTD-2228K2.2 | -0.579674118 | 0.83348603 | | down |
| RN7SL74P | -0.579674106 | 0.831808516 | | down |
| NA | -0.5796738 | 0.764721798 | | down |
| NA | -0.579673792 | 0.761851074 | | down |
| TRGV1 | -0.579673706 | 0.720256112 | | down |
| HOTAIR | -0.579673703 | 0.717884588 | | down |
| COX7A2P1 | -0.579673644 | 0.671063568 | | down |
| FFAR2 | -0.579645793 | 0.193041966 | 0.321053913 | down |
| BCL11A | -0.579612735 | 0.025775863 | 0.074973044 | down |
| PTCH2 | -0.57957844 | 0.03558943 | 0.095179707 | down |
| SNX30 | -0.579569067 | 0.000948818 | 0.005806944 | down |
| TRIM11 | -0.579473288 | 4.33E-05 | 0.000473546 | down |
| LA16c-OS12.2 | -0.579465345 | 0.151463885 | 0.270706779 | down |
| RPL7P3 | -0.579278152 | 0.6172493 | 0.728040121 | down |
| HMGN2P4 | -0.579267602 | 0.069973205 | 0.156291352 | down |
| BST1 | -0.57924826 | 0.096047752 | 0.19639164 | down |
| OGFOD3 | -0.579236332 | 0.005875424 | 0.024564602 | down |

| | | | | |
|---|---|---|---|---|
| AC079922.2 | -0.579100975 | 0.144068478 | 0.261550146 | down |
| RPS19P3 | -0.578997317 | 0.099476304 | 0.201224411 | down |
| KIAA1217 | -0.578840624 | 0.003339565 | 0.015748532 | down |
| GLYCTK-AS1 | -0.578829172 | 0.24159118 | 0.375497434 | down |
| RPL7P19 | -0.578720007 | 0.187803001 | 0.314529983 | down |
| NA | -0.5785662 | 0.15272646 | 0.272250727 | down |
| GPR82 | -0.578513719 | 0.073041831 | 0.161272941 | down |
| HIST1H2AI | -0.578326651 | 0.213770482 | 0.344646965 | down |
| KRT2 | -0.578321811 | 0.24254514 | 0.376415424 | down |
| MYLK-AS1 | -0.578294428 | 0.047289402 | 0.117437824 | down |
| HSD11B1L | -0.578225657 | 0.00412697 | 0.018704281 | down |
| GAL3ST3 | -0.578119386 | 0.478693191 | 0.608304635 | down |
| NA | -0.577951148 | 0.630969545 | 0.738885602 | down |
| AC007969.5 | -0.577850268 | 0.039190541 | 0.102350539 | down |
| RP11-18H21.3 | -0.577668751 | 0.368852119 | 0.507222311 | down |
| RP11-170M17.2 | -0.577632283 | 0.091078446 | 0.189092725 | down |
| EIF3B | -0.577366052 | 0.004181659 | 0.018886617 | down |
| TCEAL2 | -0.577292696 | 0.15429039 | 0.274030532 | down |
| KCNK2 | -0.577274874 | 0.237142897 | 0.370230784 | down |
| RP11-1277A3.1 | -0.577213112 | 0.049607787 | 0.121607079 | down |
| RTKN | -0.577207042 | 0.001501485 | 0.008341459 | down |
| NA | -0.577201282 | 0.196247216 | 0.324726076 | down |
| PAPPA-AS2 | -0.577076738 | 0.769814847 | | down |
| CTD-2270P14.5 | -0.576873192 | 0.109053085 | 0.215207556 | down |
| PARD3-AS1 | -0.576835267 | 0.248516459 | 0.382933044 | down |
| RP13-638C3.2 | -0.576779887 | 0.471707555 | 0.602268572 | down |
| NDUFS1 | -0.576737347 | 6.65E-05 | 0.000674606 | down |
| CPLX4 | -0.576676625 | 0.63368867 | | down |
| AP001055.6 | -0.576651714 | 0.128492508 | 0.241367319 | down |
| RP11-486O13.2 | -0.576649613 | 0.636738747 | 0.743554839 | down |
| MIR196A1 | -0.57657342 | 0.400356319 | 0.537361742 | down |
| RASAL1 | -0.576319042 | 0.030928037 | 0.08569602 | down |
| RP11-162D16.2 | -0.576311563 | 0.286252844 | 0.423320557 | down |
| POU3F1 | -0.576151655 | 0.268095281 | 0.403830419 | down |
| RP11-85K15.3 | -0.576004194 | 0.169191791 | 0.292692934 | down |
| TCEB1P28 | -0.575945865 | 0.345015031 | 0.483582108 | down |
| GAPDHP37 | -0.575785957 | 0.768423683 | | down |
| RP3-467K16.4 | -0.575431796 | 0.227477526 | 0.35968488 | down |
| IPO9-AS1 | -0.575280071 | 0.438584904 | 0.572850575 | down |
| U91328.19 | -0.575149991 | 0.008446732 | 0.03221704 | down |
| RP11-313P13.4 | -0.575084574 | 0.14248865 | 0.259434519 | down |
| PYCRL | -0.575038173 | 0.045781079 | 0.114620561 | down |
| NCKIPSD | -0.574583568 | 0.000345539 | 0.002614571 | down |
| PAWRP1 | -0.574551836 | 0.519209601 | | down |
| NA | -0.573964348 | 0.175239995 | 0.299687524 | down |
| MIR3179-1 | -0.573936248 | 0.572585394 | | down |
| NA | -0.573883999 | 0.287377174 | 0.424565294 | down |
| C5orf63 | -0.573872907 | 0.009386016 | 0.034934771 | down |
| ENDOV | -0.573759875 | 8.42E-05 | 0.000819183 | down |
| PRPF31 | -0.573536671 | 0.015140287 | 0.050290192 | down |
| LA16c-358B7.3 | -0.573442996 | 0.1120703 | 0.219179801 | down |

| | | | | |
|---|---|---|---|---|
| ADGRE1 | -0.573429766 | 0.032466909 | 0.088894179 | down |
| NA | -0.573403412 | 0.844626932 | | down |
| C1orf185 | -0.573403412 | 0.844626932 | | down |
| RP11-97C18.1 | -0.573403412 | 0.844626932 | | down |
| RP11-389O22.4 | -0.573403412 | 0.844626932 | | down |
| RNU1-68P | -0.573403412 | 0.844626932 | | down |
| NA | -0.573403412 | 0.844626932 | | down |
| RNU1-74P | -0.573403412 | 0.844626932 | | down |
| OR2L6P | -0.573403412 | 0.844626932 | | down |
| AC007463.2 | -0.573403412 | 0.844626932 | | down |
| RPL7P12 | -0.573403412 | 0.844626932 | | down |
| Vax2os1_3 | -0.573403412 | 0.844626932 | | down |
| AC092642.1 | -0.573403412 | 0.844626932 | | down |
| RN7SL664P | -0.573403412 | 0.844626932 | | down |
| snoU13 | -0.573403412 | 0.844626932 | | down |
| ATP5EP1 | -0.573403412 | 0.844626932 | | down |
| RP11-524L6.2 | -0.573403412 | 0.844626932 | | down |
| IL3 | -0.573403412 | 0.844626932 | | down |
| CTC-436K13.2 | -0.573403412 | 0.844626932 | | down |
| RP11-299J5.1 | -0.573403412 | 0.844626932 | | down |
| RP3-429O6.1 | -0.573403412 | 0.844626932 | | down |
| SMARCE1P2 | -0.573403412 | 0.844626932 | | down |
| KRT18P64 | -0.573403412 | 0.844626932 | | down |
| RP11-16C18.3 | -0.573403412 | 0.844626932 | | down |
| RP11-518I13.1 | -0.573403412 | 0.844626932 | | down |
| SNRPCP19 | -0.573403412 | 0.844626932 | | down |
| RP11-1217F2.1 | -0.573403412 | 0.844626932 | | down |
| MIR4650-2 | -0.573403412 | 0.844626932 | | down |
| BUB3P1 | -0.573403412 | 0.844626932 | | down |
| RP11-744I24.3 | -0.573403412 | 0.844626932 | | down |
| NA | -0.573403412 | 0.844626932 | | down |
| RP11-216N21.1 | -0.573403412 | 0.844626932 | | down |
| RP11-316J7.4 | -0.573403412 | 0.844626932 | | down |
| RP11-347J14.7 | -0.573403412 | 0.844626932 | | down |
| NA | -0.573403412 | 0.844626932 | | down |
| SLC25A6P5 | -0.573403412 | 0.844626932 | | down |
| MTND2P11 | -0.573403412 | 0.844626932 | | down |
| RN7SL57P | -0.573403412 | 0.844626932 | | down |
| NA | -0.573403412 | 0.844626932 | | down |
| TMEM256P1 | -0.573403412 | 0.844626932 | | down |
| NA | -0.573403412 | 0.844626932 | | down |
| RP11-680L20.1 | -0.573403412 | 0.844626932 | | down |
| NA | -0.573403412 | 0.844626932 | | down |
| RNU6-83P | -0.573403412 | 0.844626932 | | down |
| MIR624 | -0.573403412 | 0.844626932 | | down |
| SNORA31 | -0.573403412 | 0.844626932 | | down |
| RPS26P49 | -0.573403412 | 0.844626932 | | down |
| NA | -0.573403412 | 0.844626932 | | down |
| RP11-429B14.3 | -0.573403412 | 0.844626932 | | down |
| RP11-300G22.2 | -0.573403412 | 0.844626932 | | down |
| CTD-2258A20.3 | -0.573403412 | 0.844626932 | | down |

| | | | | |
|---|---|---|---|---|
| RP11-361L15.4 | -0.573403412 | 0.844626932 | | down |
| AC005703.2 | -0.573403412 | 0.844626932 | | down |
| RNU6-1137P | -0.573403412 | 0.844626932 | | down |
| RN7SL756P | -0.573403412 | 0.844626932 | | down |
| RP11-94B19.2 | -0.573403412 | 0.844626932 | | down |
| AIMP1P1 | -0.573403412 | 0.844626932 | | down |
| CDC42P1 | -0.573403412 | 0.844626932 | | down |
| NA | -0.573403412 | 0.844626932 | | down |
| CICP4 | -0.573403412 | 0.844626932 | | down |
| RNU6-859P | -0.573403412 | 0.844626932 | | down |
| FTCD-AS1 | -0.573403412 | 0.844626932 | | down |
| NBEAP3 | -0.573403412 | 0.844626932 | | down |
| RP3-438O4.4 | -0.573403412 | 0.844626932 | | down |
| Y_RNA | -0.573403412 | 0.844626932 | | down |
| NA | -0.573403412 | 0.844626932 | | down |
| NA | -0.57340296 | 0.82864134 | | down |
| RP11-3M1.1 | -0.573402403 | 0.799887693 | | down |
| AC093627.11 | -0.573402116 | 0.778029024 | | down |
| GPR75 | -0.573390063 | 0.290230294 | 0.427422207 | down |
| SLC25A5 | -0.573387575 | 0.011180371 | 0.039950412 | down |
| TIPARP-AS1 | -0.573305742 | 0.024886296 | 0.073051672 | down |
| SNORA7 | -0.573126197 | 0.471152776 | 0.601763552 | down |
| AC007016.3 | -0.573084106 | 0.74404002 | 0.826309138 | down |
| GUSBP1 | -0.573083734 | 0.009696982 | 0.035786282 | down |
| ZNF724 | -0.573023226 | 0.238141217 | 0.371519094 | down |
| TRIM3 | -0.57301168 | 0.001927408 | 0.010178776 | down |
| PRR34 | -0.572876191 | 0.093574808 | 0.19282315 | down |
| DLL3 | -0.572735163 | 0.276303419 | 0.412587471 | down |
| KLHL30-AS1 | -0.572661797 | 0.317158667 | 0.455886764 | down |
| LGR6 | -0.572573039 | 0.117773028 | 0.227066218 | down |
| RNU2-63P | -0.572546232 | 0.284362649 | 0.421250994 | down |
| MBOAT7 | -0.572364317 | 0.007226232 | 0.028645588 | down |
| RP11-478C6.4 | -0.572356795 | 0.18559393 | 0.311821051 | down |
| CATSPER3 | -0.572300717 | 0.148658568 | 0.267371014 | down |
| TMSB4XP2 | -0.572033986 | 0.187133138 | 0.313652887 | down |
| ZNF529-AS1 | -0.572007923 | 0.035648041 | 0.095306659 | down |
| CHCHD6 | -0.571994431 | 0.004521518 | 0.020077518 | down |
| FAM134B | -0.571984796 | 0.005759074 | 0.024158841 | down |
| MAP3K11 | -0.571981421 | 7.36E-05 | 0.0007307 | down |
| NUDT12 | -0.571798971 | 0.032952774 | 0.089880449 | down |
| RNA5-8S5 | -0.571770124 | 0.591698966 | | down |
| MRPL55 | -0.571734787 | 0.021165611 | 0.064762156 | down |
| RP11-44F14.2 | -0.57164948 | 0.213478819 | 0.344362827 | down |
| RP11-834C11.8 | -0.571272574 | 0.299275969 | 0.436838518 | down |
| FBXL20 | -0.571113127 | 0.000293021 | 0.002286039 | down |
| WI2-80269A6.1 | -0.570959376 | 0.53284702 | | down |
| SLC20A1P2 | -0.570918701 | 0.621941806 | | down |
| RGMB-AS1 | -0.570908257 | 0.095058414 | 0.194997609 | down |
| POLN | -0.570889124 | 0.115447119 | 0.223860413 | down |
| SPATA31A6 | -0.570841893 | 0.845311908 | | down |
| SULT1C2P2 | -0.570840162 | 0.836225749 | | down |

| | | | | |
|---|---|---|---|---|
| RNU2-68P | -0.570835987 | 0.816027081 | | down |
| RP11-783L4.1 | -0.570835238 | 0.812631391 | | down |
| AC018641.7 | -0.570810002 | 0.722267418 | | down |
| NA | -0.570809193 | 0.719899003 | | down |
| RP11-435I10.4 | -0.570800885 | 0.696807523 | | down |
| CTD-2339L15.3 | -0.570800199 | 0.69499143 | | down |
| SYS1-DBNDD2 | -0.570793811 | 0.678688809 | | down |
| VN2R17P | -0.570790814 | 0.671376956 | | down |
| RP11-452C8.1 | -0.570783124 | 0.653492228 | | down |
| RNU2-17P | -0.570782161 | 0.65133514 | | down |
| MIR571 | -0.570782007 | 0.650991539 | | down |
| NA | -0.570776015 | 0.192383591 | 0.320291067 | down |
| RP11-231E19.1 | -0.570771067 | 0.627625672 | | down |
| NA | -0.570740665 | 0.144771668 | 0.262319523 | down |
| SOX8 | -0.570717365 | 0.080773828 | 0.173414955 | down |
| DOC2GP | -0.570710251 | 0.066259935 | 0.150129223 | down |
| NA | -0.570675463 | 0.410629993 | 0.547409482 | down |
| RP11-572P18.1 | -0.57061561 | 0.024506066 | 0.072239034 | down |
| C9orf43 | -0.570595722 | 0.092122547 | 0.190613224 | down |
| RP4-631H13.2 | -0.570395283 | 0.811300527 | | down |
| XXbac-B461K10.4 | -0.570291326 | 0.536126644 | 0.658674021 | down |
| TENM3 | -0.570224111 | 0.122524001 | 0.233395327 | down |
| CBFA2T2 | -0.570073522 | 9.51E-07 | 1.94E-05 | down |
| TNIP1 | -0.570051959 | 0.00070859 | 0.004608542 | down |
| AC008063.3 | -0.5700393 | 0.796346478 | | down |
| RP11-21L23.3 | -0.57003544 | 0.185617885 | 0.311843859 | down |
| CTC-429P9.3 | -0.570035085 | 0.02077056 | 0.063839458 | down |
| RP11-466H18.1 | -0.570031475 | 0.001105733 | 0.006574442 | down |
| RP11-277P12.10 | -0.56994955 | 0.686726169 | | down |
| LINC00523 | -0.569896641 | 0.791953943 | | down |
| LIPT1 | -0.569732856 | 0.015172781 | 0.050364716 | down |
| LAMTOR5-AS1 | -0.569636276 | 0.046703107 | 0.116279783 | down |
| MRGPRX2 | -0.569624393 | 0.686042565 | | down |
| ARFGAP1 | -0.569492236 | 0.011357233 | 0.040462077 | down |
| LINC00176 | -0.569326166 | 0.105994122 | 0.210697855 | down |
| TBC1D29 | -0.569298801 | 0.312861568 | 0.451535426 | down |
| RPL36 | -0.568994546 | 0.009176753 | 0.034338817 | down |
| ZNF192P1 | -0.568813829 | 0.083470322 | 0.177658449 | down |
| ASB16 | -0.568778047 | 0.05859864 | 0.137357204 | down |
| RP4-597N16.4 | -0.568483222 | 0.289663973 | 0.426915104 | down |
| RP11-498E2.9 | -0.568393943 | 0.121669638 | 0.232382943 | down |
| GRHL3 | -0.568212817 | 0.273957927 | 0.410267767 | down |
| GALT | -0.56817708 | 1.34E-05 | 0.00018027 | down |
| NA | -0.567898091 | 0.569754181 | 0.687430008 | down |
| NA | -0.567844671 | 0.006807657 | 0.02741679 | down |
| HMGB3P21 | -0.567809693 | 0.813932589 | | down |
| ETV1 | -0.567640381 | 0.07244123 | 0.160325917 | down |
| RP11-231C14.5 | -0.567579799 | 0.645873236 | 0.750836384 | down |
| RP11-35015.1 | -0.566891963 | 0.383721425 | 0.521457778 | down |
| RNF112 | -0.566601443 | 0.004848769 | 0.021192378 | down |
| RP11-826N14.2 | -0.566566302 | 0.609753139 | | down |

| | | | | |
|---|---|---|---|---|
| LINC01422 | -0.56654707 | 0.376836971 | 0.515020241 | down |
| AC002116.7 | -0.566461382 | 0.178135188 | 0.303240613 | down |
| RP11-379F4.6 | -0.56632729 | 0.107000165 | 0.212206282 | down |
| NA | -0.566137293 | 0.216982854 | 0.348257538 | down |
| B3GAT3 | -0.565978763 | 0.022531955 | 0.067783125 | down |
| CTA-398F10.1 | -0.565960323 | 0.57839013 | 0.694863767 | down |
| SCGB1B2P | -0.565909405 | 0.085563506 | 0.180982116 | down |
| FAM220CP | -0.565802538 | 0.566680667 | | down |
| LGALS3 | -0.565787369 | 0.029598578 | 0.083015281 | down |
| RP11-1036E20.7 | -0.565716269 | 0.665332485 | | down |
| RP11-1101H11.1 | -0.56570291 | 0.591928034 | | down |
| TCN2 | -0.565680426 | 0.011914918 | 0.041971133 | down |
| CTD-2325P2.4 | -0.565560037 | 0.235833357 | 0.36881877 | down |
| EEF1A1P29 | -0.565303903 | 0.408668037 | 0.545447468 | down |
| MCM3AP-AS1 | -0.565083483 | 0.113167046 | 0.220606717 | down |
| GBP3 | -0.565071808 | 0.049358166 | 0.121242477 | down |
| CTD-2196E14.3 | -0.565036842 | 0.50182659 | 0.628779634 | down |
| CCL21 | -0.564682283 | 0.137271044 | 0.252660126 | down |
| RP3-510H16.3 | -0.564250937 | 0.330626016 | 0.468951375 | down |
| RP11-345P4.10 | -0.564144999 | 0.472111599 | 0.602528392 | down |
| PHF14 | -0.564090458 | 0.00409383 | 0.018579308 | down |
| RP11-567G24.1 | -0.564073086 | 0.326253715 | 0.46461424 | down |
| KB-1184D12.1 | -0.564030055 | 0.39604987 | 0.533105837 | down |
| ACACB | -0.563998037 | 0.001189207 | 0.00696734 | down |
| RP5-1024N4.2 | -0.563994362 | 0.847144784 | | down |
| CDCP2 | -0.563994362 | 0.847144784 | | down |
| ACTG1P21 | -0.563994362 | 0.847144784 | | down |
| RP11-574F21.3 | -0.563994362 | 0.847144784 | | down |
| NA | -0.563994362 | 0.847144784 | | down |
| RP11-316I3.2 | -0.563994362 | 0.847144784 | | down |
| PROX1-AS1 | -0.563994362 | 0.847144784 | | down |
| RP11-543E8.2 | -0.563994362 | 0.847144784 | | down |
| OR14A16 | -0.563994362 | 0.847144784 | | down |
| OR2T12 | -0.563994362 | 0.847144784 | | down |
| AC098872.3 | -0.563994362 | 0.847144784 | | down |
| GLULP6 | -0.563994362 | 0.847144784 | | down |
| RP11-255E6.5 | -0.563994362 | 0.847144784 | | down |
| OR7E130P | -0.563994362 | 0.847144784 | | down |
| HNRNPA1P24 | -0.563994362 | 0.847144784 | | down |
| RNU2-31P | -0.563994362 | 0.847144784 | | down |
| NA | -0.563994362 | 0.847144784 | | down |
| MIR576 | -0.563994362 | 0.847144784 | | down |
| U6 | -0.563994362 | 0.847144784 | | down |
| RP11-334G8.1 | -0.563994362 | 0.847144784 | | down |
| CUL1P1 | -0.563994362 | 0.847144784 | | down |
| NA | -0.563994362 | 0.847144784 | | down |
| CTC-209L16.1 | -0.563994362 | 0.847144784 | | down |
| RP11-69L16.3 | -0.563994362 | 0.847144784 | | down |
| AP002530.2 | -0.563994362 | 0.847144784 | | down |
| AC074389.7 | -0.563994362 | 0.847144784 | | down |
| MIR148A | -0.563994362 | 0.847144784 | | down |

| | | | |
|---|---|---|---|
| RP11-89N17.2 | -0.563994362 | 0.847144784 | down |
| RN7SL625P | -0.563994362 | 0.847144784 | down |
| RN7SL685P | -0.563994362 | 0.847144784 | down |
| MTND6P5 | -0.563994362 | 0.847144784 | down |
| IFNA16 | -0.563994362 | 0.847144784 | down |
| IFNA11P | -0.563994362 | 0.847144784 | down |
| YRDCP2 | -0.563994362 | 0.847144784 | down |
| PRPF38AP2 | -0.563994362 | 0.847144784 | down |
| RN7SKP219 | -0.563994362 | 0.847144784 | down |
| NA | -0.563994362 | 0.847144784 | down |
| RAG2 | -0.563994362 | 0.847144784 | down |
| OR5J1P | -0.563994362 | 0.847144784 | down |
| RP11-688I9.4 | -0.563994362 | 0.847144784 | down |
| RP11-259P6.3 | -0.563994362 | 0.847144784 | down |
| RP11-90D4.4 | -0.563994362 | 0.847144784 | down |
| RP11-7M8.2 | -0.563994362 | 0.847144784 | down |
| MIR4499 | -0.563994362 | 0.847144784 | down |
| RPL23AP69 | -0.563994362 | 0.847144784 | down |
| ANKRD26P4 | -0.563994362 | 0.847144784 | down |
| Metazoa_SRP | -0.563994362 | 0.847144784 | down |
| LINC00458 | -0.563994362 | 0.847144784 | down |
| RP11-108M12.2 | -0.563994362 | 0.847144784 | down |
| RP11-516J2.1 | -0.563994362 | 0.847144784 | down |
| RP11-353P15.1 | -0.563994362 | 0.847144784 | down |
| VN1R3 | -0.563994362 | 0.847144784 | down |
| IGHV2OR16-5 | -0.563994362 | 0.847144784 | down |
| RP11-488I20.8 | -0.563994362 | 0.847144784 | down |
| RP11-720N19.2 | -0.563994362 | 0.847144784 | down |
| RP11-218M11.7 | -0.563994362 | 0.847144784 | down |
| RP11-401F2.4 | -0.563994362 | 0.847144784 | down |
| RP1-200G19.1 | -0.563994362 | 0.847144784 | down |
| CTD-2378E21.1 | -0.563994362 | 0.847144784 | down |
| RP11-9H20.2 | -0.563994362 | 0.847144784 | down |
| RP11-861L17.2 | -0.563994362 | 0.847144784 | down |
| RP11-94B19.6 | -0.563994362 | 0.847144784 | down |
| AC004004.2 | -0.563994362 | 0.847144784 | down |
| CTC-565M22.1 | -0.563994362 | 0.847144784 | down |
| AC009892.2 | -0.563994362 | 0.847144784 | down |
| NA | -0.563994362 | 0.847144784 | down |
| RN7SKP210 | -0.563994362 | 0.847144784 | down |
| MIR6889 | -0.563994362 | 0.847144784 | down |
| MTND2P24 | -0.563994362 | 0.847144784 | down |
| RP5-1100E15.4 | -0.563994362 | 0.847144784 | down |
| CTD-2193P3.1 | -0.56399436 | 0.847050056 | down |
| CROCCP1 | -0.5639943 | 0.845092995 | down |
| MTND1P3 | -0.563993844 | 0.827071403 | down |
| SLC10A5P1 | -0.563993843 | 0.827001457 | down |
| RPL31P7 | -0.563993806 | 0.825279109 | down |
| CTD-2342N23.1 | -0.563993763 | 0.823147162 | down |
| Metazoa_SRP | -0.563993418 | 0.802909493 | down |
| BMP15 | -0.563993401 | 0.801727884 | down |

| | | | | |
|---|---|---|---|---|
| MIR302B | -0.563993126 | 0.779014036 | | down |
| PRRT3 | -0.563764209 | 0.002767466 | 0.013614825 | down |
| RP11-545M17.3 | -0.56371871 | 0.268770846 | 0.404555573 | down |
| RAPGEF4-AS1 | -0.563687018 | 0.691404804 | | down |
| NA | -0.563394105 | 0.150431754 | 0.269549457 | down |
| RP11-430H10.1 | -0.563205247 | 0.175025377 | 0.299525088 | down |
| CTD-2196E14.8 | -0.563034201 | 0.520155795 | 0.644617538 | down |
| PSMG3-AS1 | -0.562953946 | 0.003160641 | 0.015072747 | down |
| HSD3B7 | -0.562944851 | 0.02654652 | 0.076674066 | down |
| RP11-390K5.1 | -0.562816944 | 0.33230285 | 0.470622405 | down |
| RP5-1050E16.1 | -0.562788808 | 0.836158305 | | down |
| CTD-2353N24.1 | -0.562772771 | 0.813018827 | | down |
| DEFB109P1 | -0.562758419 | 0.794633119 | | down |
| PRR13P1 | -0.562737876 | 0.77107773 | | down |
| MIR5194 | -0.562626974 | 0.675039188 | | down |
| B3GAT2 | -0.562625156 | 0.300705735 | 0.438456836 | down |
| KLK4 | -0.562539563 | 0.319417225 | 0.458223325 | down |
| NA | -0.562517172 | 0.606334655 | | down |
| RP11-692D12.1 | -0.562484753 | 0.223484502 | 0.355558588 | down |
| ADIRF-AS1 | -0.562424661 | 0.025961162 | 0.075396053 | down |
| RP11-757O6.4 | -0.562380775 | 0.539508281 | | down |
| CTBP2P4 | -0.562291917 | 0.729010155 | | down |
| RP11-128L5.1 | -0.562193123 | 0.816426228 | | down |
| MORF4L2-AS1 | -0.562145081 | 0.14149164 | 0.258217171 | down |
| BAIAP2L1 | -0.562036538 | 0.041939077 | 0.107693814 | down |
| LINC00607 | -0.561959228 | 0.39805166 | 0.535081166 | down |
| AC012668.2 | -0.561902626 | 0.836329686 | | down |
| EPB41L3 | -0.561898499 | 0.051492306 | 0.124982618 | down |
| HIST1H4B | -0.561700654 | 0.286383693 | 0.423472373 | down |
| FAM86C1 | -0.561538829 | 0.00846914 | 0.032286113 | down |
| RP13-188A5.1 | -0.561531565 | 0.016302295 | 0.053136806 | down |
| MIR142 | -0.561461731 | 0.252493371 | 0.387490977 | down |
| VN2R1P | -0.561240142 | 0.797592987 | | down |
| ZFP41 | -0.5612247 | 0.003779501 | 0.017415849 | down |
| RP11-417F21.1 | -0.561192891 | 0.482186709 | 0.611335506 | down |
| RP11-297N6.4 | -0.561153991 | 0.273366529 | 0.409647563 | down |
| KRT18P7 | -0.561063305 | 0.683484803 | | down |
| IGSF1 | -0.560962223 | 0.129432334 | 0.242450593 | down |
| MIR5587 | -0.560957874 | 0.412538318 | 0.549197834 | down |
| ACTL10 | -0.560326804 | 0.073016898 | 0.161231787 | down |
| SIRPB3P | -0.560198227 | 0.541734037 | 0.663291932 | down |
| ILDR1 | -0.55968671 | 0.039590086 | 0.103160846 | down |
| PNPLA2 | -0.559664794 | 0.00026641 | 0.002119146 | down |
| LRRC23 | -0.559531996 | 0.023036247 | 0.068845222 | down |
| CTC-242N15.1 | -0.559449233 | 0.382070323 | 0.519925753 | down |
| NAGLU | -0.559376519 | 0.017158992 | 0.055224933 | down |
| CASK | -0.559121804 | 0.000401323 | 0.002921155 | down |
| NA | -0.559087349 | 0.713629625 | | down |
| ADRA2B | -0.558977519 | 0.102511799 | 0.205583665 | down |
| RP11-1094M14.7 | -0.5589473 | 0.51530552 | | down |
| TBRG4 | -0.55893714 | 0.001081128 | 0.006456558 | down |

| | | | | |
|---|---|---|---|---|
| PPL | -0.558892724 | 0.09282443 | 0.191606813 | down |
| ASAP3 | -0.558879844 | 0.003618256 | 0.016809478 | down |
| TRIM52-AS1 | -0.558805704 | 0.002662885 | 0.01320629 | down |
| PHLPP1 | -0.558672858 | 4.66E-05 | 0.000504009 | down |
| NA | -0.558647373 | 0.602437606 | | down |
| ADCY9 | -0.55839762 | 0.015472291 | 0.051155444 | down |
| MRPS25 | -0.558378218 | 0.003132972 | 0.014978766 | down |
| RTCB | -0.558371705 | 0.000334098 | 0.002545683 | down |
| RP11-517C16.2 | -0.55805922 | 0.38216596 | 0.519947165 | down |
| MAPKAPK5-AS1 | -0.558016606 | 0.001197324 | 0.00700397 | down |
| RP11-48G14.3 | -0.557815032 | 0.40903752 | 0.545795135 | down |
| RP11-520P18.1 | -0.557587635 | 0.775011045 | | down |
| TRAJ4 | -0.557529994 | 0.689096813 | | down |
| NA | -0.557487593 | 0.820589927 | | down |
| NA | -0.557324866 | 0.256209005 | 0.391234731 | down |
| HLA-K | -0.557162332 | 0.065164599 | 0.148228741 | down |
| RP11-557J10.3 | -0.557112003 | 0.709906772 | | down |
| AZIN2 | -0.557015828 | 0.017860753 | 0.057055657 | down |
| TNFRSF14 | -0.556959112 | 0.005907184 | 0.024652791 | down |
| SDR9C7 | -0.556919062 | 0.709724683 | | down |
| RP11-303E16.7 | -0.556789417 | 0.644061784 | | down |
| PFN1P8 | -0.55677086 | 0.70805255 | | down |
| NA | -0.556653882 | 0.640123058 | | down |
| RP11-498C9.12 | -0.556626333 | 0.364543508 | 0.50293177 | down |
| LMF1 | -0.556402814 | 0.017106036 | 0.055084002 | down |
| RP1-10C16.1 | -0.556391149 | 0.551779199 | | down |
| FDX2 | -0.556371965 | 0.796436494 | | down |
| EIF3LP2 | -0.556325502 | 0.336174871 | 0.474354015 | down |
| RPL21P110 | -0.556306539 | 0.649760216 | | down |
| AC130689.5 | -0.556300194 | 0.602221119 | | down |
| MTND4P9 | -0.556261988 | 0.614681067 | | down |
| ZNF625 | -0.556227707 | 0.573057818 | | down |
| CTD-3137H5.1 | -0.556211794 | 0.388001439 | 0.525616161 | down |
| RPL35P1 | -0.556177853 | 0.056526168 | 0.133806506 | down |
| OTUB1 | -0.556138389 | 0.011409118 | 0.040603594 | down |
| CHKA | -0.556090697 | 0.00045532 | 0.00323014 | down |
| GSDMB | -0.55598087 | 0.003042924 | 0.014665033 | down |
| RP11-27I1.4 | -0.555904891 | 0.118068088 | 0.227507301 | down |
| TNNC1 | -0.555863309 | 0.073260352 | 0.161603084 | down |
| BAIAP2 | -0.555851339 | 0.01012139 | 0.037007063 | down |
| RP11-211G3.3 | -0.555700826 | 0.755818657 | | down |
| CCDC187 | -0.555571387 | 0.196115461 | 0.324615333 | down |
| QRFP | -0.555388694 | 0.226951904 | 0.359078572 | down |
| CBWD3 | -0.555269567 | 0.019998362 | 0.062093604 | down |
| MIR3183 | -0.555132976 | 0.628930809 | | down |
| PCDHA3 | -0.55507033 | 0.402070945 | 0.538790483 | down |
| C19orf73 | -0.555042818 | 0.045649923 | 0.114373352 | down |
| NLRP7 | -0.555036069 | 0.14777085 | 0.266340357 | down |
| MON1A | -0.554966656 | 0.000171183 | 0.001470278 | down |
| PPP1R14BP3 | -0.554841652 | 0.052481249 | 0.126575149 | down |
| CTD-2384A14.2 | -0.554832326 | 0.768571226 | | down |

| | | | | |
|---|---|---|---|---|
| MIR4740 | -0.554827647 | 0.267101397 | 0.402777202 | down |
| ASIC1 | -0.554650686 | 0.048573189 | 0.119872916 | down |
| QARS | -0.55464823 | 0.000162338 | 0.001407582 | down |
| RANP5 | -0.554585313 | 0.849664197 | | down |
| NA | -0.554585313 | 0.849664197 | | down |
| AC016894.1 | -0.554585313 | 0.849664197 | | down |
| MTCO3P7 | -0.554585313 | 0.849664197 | | down |
| IQCF6 | -0.554585313 | 0.849664197 | | down |
| NA | -0.554585313 | 0.849664197 | | down |
| RP1-20N2.7 | -0.554585313 | 0.849664197 | | down |
| AC005077.7 | -0.554585313 | 0.849664197 | | down |
| AC093106.4 | -0.554585313 | 0.849664197 | | down |
| NA | -0.554585313 | 0.849664197 | | down |
| RP11-350N15.6 | -0.554585313 | 0.849664197 | | down |
| Y_RNA | -0.554585313 | 0.849664197 | | down |
| SNRPCP5 | -0.554585313 | 0.849664197 | | down |
| YPEL5P2 | -0.554585313 | 0.849664197 | | down |
| RP11-471N19.1 | -0.554585313 | 0.849664197 | | down |
| RP11-3L23.2 | -0.554585313 | 0.849664197 | | down |
| HSBP1P1 | -0.554585313 | 0.849664197 | | down |
| IGHV1-12 | -0.554585313 | 0.849664197 | | down |
| CTD-2515H24.4 | -0.554585313 | 0.849664197 | | down |
| CTB-60B18.18 | -0.554585313 | 0.849664197 | | down |
| BCRP8 | -0.554585313 | 0.849664197 | | down |
| RPL21P132 | -0.554585313 | 0.849664197 | | down |
| MTND4P32 | -0.554585313 | 0.849664197 | | down |
| CXorf66 | -0.554585311 | 0.849611822 | | down |
| TMA16P2 | -0.55458527 | 0.848189001 | | down |
| RP11-399K21.11 | -0.554585246 | 0.847335104 | | down |
| RP11-579D7.8 | -0.554584781 | 0.827346823 | | down |
| AP002856.5 | -0.554584758 | 0.826156052 | | down |
| RP11-165M1.2 | -0.55458475 | 0.825691169 | | down |
| Y_RNA | -0.554584719 | 0.82403927 | | down |
| MIR1972-2 | -0.554584707 | 0.823345084 | | down |
| DNAJC19P6 | -0.554584677 | 0.821705962 | | down |
| TPT1P10 | -0.554584391 | 0.802685745 | | down |
| NA | -0.55458439 | 0.802593332 | | down |
| CTC-295J13.3 | -0.554584382 | 0.802047287 | | down |
| FAM86LP | -0.554584371 | 0.801128227 | | down |
| COX6B1P3 | -0.554584138 | 0.779684463 | | down |
| RNU6-199P | -0.554584088 | 0.774064683 | | down |
| RP11-687M24.8 | -0.554583945 | 0.755385234 | | down |
| RP11-329A14.1 | -0.5545838 | 0.730652008 | | down |
| RP11-696N14.1 | -0.554561684 | 0.028607059 | 0.081097422 | down |
| SPINK6 | -0.554559123 | 0.66182414 | | down |
| ACOX3 | -0.554354724 | 0.000434581 | 0.003109442 | down |
| CTSW | -0.554342282 | 0.064497699 | 0.147216704 | down |
| ALDH8A1 | -0.554271931 | 0.151360006 | 0.270569407 | down |
| AC002310.14 | -0.554242945 | 0.062481487 | 0.144000751 | down |
| NTRK3 | -0.554175782 | 0.169160909 | 0.29265636 | down |
| RP11-359G22.2 | -0.554136981 | 0.563126864 | 0.682030224 | down |

| | | | | |
|---|---|---|---|---|
| PRSS42 | -0.55410435 | 0.467284316 | 0.598401176 | down |
| RP11-53B2.2 | -0.55389163 | 0.213204736 | 0.34406849 | down |
| PGBD5 | -0.553889549 | 0.03268537 | 0.08934576 | down |
| NACAP3 | -0.553795031 | 0.469508414 | 0.600321504 | down |
| NA | -0.553723122 | 0.303493202 | 0.441548835 | down |
| IMPA2 | -0.553675236 | 0.00507892 | 0.021958619 | down |
| AC018816.3 | -0.553509766 | 0.15846319 | 0.279524899 | down |
| HCG18 | -0.55345329 | 0.001436637 | 0.008063378 | down |
| EIF4BP2 | -0.55342345 | 0.691213951 | | down |
| PALM3 | -0.553333364 | 0.117208731 | 0.226297738 | down |
| LINC00606 | -0.553303507 | 0.749625292 | | down |
| FCAMR | -0.553217441 | 0.244882234 | 0.378790189 | down |
| RP11-236L14.2 | -0.553148732 | 0.15738427 | 0.278111575 | down |
| TTC19 | -0.553039526 | 1.09E-06 | 2.19E-05 | down |
| FAM207A | -0.552997845 | 0.010635208 | 0.038445939 | down |
| FAM110A | -0.552892518 | 0.003554789 | 0.016591592 | down |
| RNU6-1170P | -0.552821604 | 0.707336072 | 0.798887446 | down |
| ZNF3 | -0.552719641 | 3.72E-09 | 1.64E-07 | down |
| PEBP1 | -0.552686137 | 4.00E-05 | 0.000444354 | down |
| RAD52 | -0.552510515 | 0.000388267 | 0.00284862 | down |
| ABTB2 | -0.552362614 | 0.025740958 | 0.07489457 | down |
| SLC22A31 | -0.552344427 | 0.275671956 | 0.412013145 | down |
| RP11-368I7.4 | -0.552302828 | 0.148290427 | 0.267020533 | down |
| LINC00963 | -0.552162592 | 0.011924686 | 0.042000619 | down |
| RP11-1379J22.2 | -0.551795346 | 0.149403756 | 0.268301641 | down |
| RP11-304L19.1 | -0.551784032 | 0.208179225 | 0.338394018 | down |
| KLF14 | -0.551632213 | 0.485624398 | 0.614444451 | down |
| MAP7 | -0.55162645 | 0.031791226 | 0.087514713 | down |
| GADL1 | -0.55157453 | 0.527568018 | 0.651068126 | down |
| CTD-3051D23.1 | -0.551561644 | 0.313229465 | 0.451918752 | down |
| SERTAD1 | -0.551558664 | 0.012733615 | 0.044104446 | down |
| RP11-120K24.3 | -0.55137846 | 0.195788191 | 0.32432378 | down |
| RP11-85B7.5 | -0.551331297 | 0.43252779 | 0.5675357 | down |
| RP11-313E19.2 | -0.551018875 | 0.578982011 | 0.695352396 | down |
| RP11-371A19.2 | -0.551004399 | 0.554252757 | 0.674228743 | down |
| GAL3ST2 | -0.551002254 | 0.30101656 | 0.438782278 | down |
| AL590762.7 | -0.550888459 | 0.469103696 | | down |
| CA9 | -0.550701371 | 0.209040694 | 0.339225376 | down |
| RP11-342M1.3 | -0.550668527 | 0.174185107 | 0.298682572 | down |
| ZNF581 | -0.550605177 | 0.011420375 | 0.04061479 | down |
| RPL23AP3 | -0.550558786 | 0.43348311 | | down |
| EEF1A2 | -0.550519492 | 0.091843672 | 0.190232507 | down |
| ZNF665 | -0.550420632 | 0.041041357 | 0.105877256 | down |
| DDX39B-AS1 | -0.550121963 | 0.299388727 | 0.436916592 | down |
| RP1-59D14.5 | -0.550107908 | 0.240138991 | 0.373917379 | down |
| Y_RNA | -0.549872731 | 0.45475514 | 0.587223367 | down |
| RP11-73M7.9 | -0.54985918 | 0.740799159 | 0.823963591 | down |
| RNVU1-3 | -0.549804498 | 0.356998969 | 0.495420802 | down |
| RP11-235C23.5 | -0.549694264 | 0.401790189 | 0.538527254 | down |
| HPGDS | -0.549577274 | 0.041590264 | 0.107017633 | down |
| RNU6-1216P | -0.549333164 | 0.784924262 | | down |

| | | | | |
|---|---|---|---|---|
| CTD-3014M21.1 | -0.549318075 | 0.18599519 | 0.312303106 | down |
| SUMO2P17 | -0.549225782 | 0.185815102 | 0.312070485 | down |
| API5P1 | -0.549150512 | 0.629965092 | | down |
| MLIP-IT1 | -0.549145054 | 0.681891213 | | down |
| PRKAG2 | -0.549034994 | 1.54E-05 | 0.000202874 | down |
| RP4-651E10.4 | -0.5490237 | 0.409309088 | 0.546056503 | down |
| ADRA1A | -0.549011293 | 0.303605324 | 0.441634471 | down |
| RP11-508N22.12 | -0.548922515 | 0.294344929 | 0.431759547 | down |
| FAM134A | -0.548857781 | 5.21E-05 | 0.000553174 | down |
| RP4-705D16.3 | -0.548703259 | 0.698972182 | | down |
| RPL21P28 | -0.548333467 | 0.102080953 | 0.20504915 | down |
| RPS20P35 | -0.548327581 | 0.331807994 | 0.470161739 | down |
| RP11-180I4.2 | -0.548311795 | 0.681085927 | | down |
| VRK3 | -0.548279777 | 5.84E-05 | 0.000607573 | down |
| GRM4 | -0.548151771 | 0.179069992 | 0.304194212 | down |
| ZNF628 | -0.548086152 | 0.002328199 | 0.011880022 | down |
| WNT4 | -0.547932155 | 0.113062647 | 0.220446113 | down |
| AC016735.2 | -0.547754666 | 0.347447571 | 0.486156115 | down |
| RPL21P1 | -0.547745859 | 0.115889974 | 0.224429442 | down |
| COX19 | -0.547712781 | 8.04E-07 | 1.68E-05 | down |
| RP11-961A15.3 | -0.547697337 | 0.558490638 | | down |
| RP11-384M15.3 | -0.547578413 | 0.469674909 | | down |
| NA | -0.547493732 | 0.318254057 | 0.457144047 | down |
| RP11-147L13.8 | -0.547437226 | 0.323752147 | 0.462125904 | down |
| RP11-301O19.1 | -0.547282407 | 0.268688569 | 0.404493629 | down |
| PLGLB1 | -0.547254357 | 0.158261292 | 0.279234335 | down |
| RP11-620J15.3 | -0.547181824 | 0.035487908 | 0.094942005 | down |
| CISD1 | -0.547010537 | 1.09E-05 | 0.000151603 | down |
| NA | -0.546999843 | 0.474297402 | 0.604456882 | down |
| UPK3A | -0.546982216 | 0.143773894 | 0.26118895 | down |
| AC009133.12 | -0.546900489 | 0.041966292 | 0.107736074 | down |
| CCDC94 | -0.546704758 | 0.004168365 | 0.018837619 | down |
| YY1 | -0.546501072 | 3.81E-05 | 0.000427266 | down |
| TFPT | -0.546470331 | 0.019001494 | 0.059703082 | down |
| AC093627.10 | -0.546410138 | 0.11321422 | 0.220671271 | down |
| AC012074.2 | -0.546353797 | 0.262986723 | 0.398299758 | down |
| ADGRA3 | -0.546346528 | 0.019477959 | 0.06082484 | down |
| NA | -0.546343563 | 0.487478734 | 0.616224643 | down |
| LINC00857 | -0.54622866 | 0.018327736 | 0.058176282 | down |
| LINC01504 | -0.546151859 | 0.028171898 | 0.080189153 | down |
| PSMB3 | -0.546128452 | 0.02438114 | 0.071962566 | down |
| RP11-805I24.4 | -0.54608038 | 0.4803733 | 0.609731113 | down |
| RP11-402P6.15 | -0.545836905 | 0.455948315 | 0.588322933 | down |
| C1orf226 | -0.545831159 | 0.036455192 | 0.096934224 | down |
| GP9 | -0.545623373 | 0.358001555 | 0.496436755 | down |
| RP11-88I18.2 | -0.545559652 | 0.339738704 | 0.478116597 | down |
| SNORD93 | -0.545537675 | 0.717421677 | | down |
| RP11-180C16.1 | -0.545416289 | 0.318534201 | 0.457371512 | down |
| RNASEH2C | -0.545409994 | 0.012734858 | 0.044104446 | down |
| SPATA6L | -0.545380649 | 0.021232951 | 0.064908706 | down |
| RP11-4L24.4 | -0.54534113 | 0.18672707 | 0.313112021 | down |

| | | | | |
|---|---|---|---|---|
| RP11-739N20.3 | -0.545176263 | 0.852185146 | | down |
| RP11-239E10.3 | -0.545176263 | 0.852185146 | | down |
| HNRNPA1P42 | -0.545176263 | 0.852185146 | | down |
| SCARNA21 | -0.545176263 | 0.852185146 | | down |
| Y_RNA | -0.545176263 | 0.852185146 | | down |
| RNU6-127P | -0.545176263 | 0.852185146 | | down |
| RNU6-454P | -0.545176263 | 0.852185146 | | down |
| MTCO3P28 | -0.545176263 | 0.852185146 | | down |
| RP13-539F13.2 | -0.545176263 | 0.852185146 | | down |
| NA | -0.545176263 | 0.852185146 | | down |
| NCOR1P3 | -0.545176263 | 0.852185146 | | down |
| RNA5SP268 | -0.545176263 | 0.852185146 | | down |
| RP11-358M14.2 | -0.545176263 | 0.852185146 | | down |
| RP11-403H13.1 | -0.545176263 | 0.852185146 | | down |
| AL138479.3 | -0.545176263 | 0.852185146 | | down |
| RP11-925D8.3 | -0.545176263 | 0.852185146 | | down |
| RP11-185O21.1 | -0.545176263 | 0.852185146 | | down |
| RN7SL520P | -0.545176263 | 0.852185146 | | down |
| RP11-1072C15.1 | -0.545176263 | 0.852185146 | | down |
| RP11-752P2.1 | -0.545176263 | 0.852185146 | | down |
| AP001595.1 | -0.545176263 | 0.852185146 | | down |
| NA | -0.54517625 | 0.851703236 | | down |
| RP11-110A12.2 | -0.545176207 | 0.850098804 | | down |
| CTD-2216M2.1 | -0.545176163 | 0.848420656 | | down |
| RP11-732A21.2 | -0.545176099 | 0.84584333 | | down |
| BTF3P13 | -0.545175733 | 0.827996924 | | down |
| AC007790.4 | -0.545175705 | 0.826342851 | | down |
| MIR151B | -0.545175695 | 0.825783139 | | down |
| NPM1P43 | -0.545175682 | 0.82497538 | | down |
| RP11-575L7.2 | -0.545175434 | 0.807739981 | | down |
| RP11-424M22.3 | -0.545175401 | 0.805005558 | | down |
| RP11-235D19.4 | -0.545175378 | 0.803016504 | | down |
| RP4-809F4.1 | -0.545175369 | 0.802223381 | | down |
| NA | -0.54517513 | 0.777405098 | | down |
| AP006547.3 | -0.545174978 | 0.755577425 | | down |
| RP11-165F24.5 | -0.545174845 | 0.730110816 | | down |
| SPC24 | -0.545169263 | 0.051744084 | 0.125330479 | down |
| PDGFA | -0.545135731 | 0.008082921 | 0.031173718 | down |
| SCX | -0.544792514 | 0.023258776 | 0.069399808 | down |
| RP11-179B2.2 | -0.54478202 | 0.051958556 | 0.125636128 | down |
| NUTM2D | -0.544649234 | 0.017836536 | 0.057008605 | down |
| RP11-57C13.6 | -0.544550192 | 0.361743614 | 0.500079379 | down |
| RP11-227D13.5 | -0.54446036 | 0.682248055 | | down |
| ATXN7 | -0.544267331 | 0.272150283 | 0.408395339 | down |
| RP5-908M14.9 | -0.544099922 | 0.072186262 | 0.159879399 | down |
| TOMM20L | -0.544079706 | 0.23706855 | 0.37016462 | down |
| RIPPLY2 | -0.544077317 | 0.660530627 | | down |
| DGKG | -0.543965889 | 0.115714022 | 0.224233235 | down |
| RN7SL806P | -0.543917063 | 0.645950575 | | down |
| MIR10A | -0.543711089 | 0.281627555 | 0.418106353 | down |
| AC007255.8 | -0.543596692 | 0.173175236 | 0.297636052 | down |

| | | | | |
|---|---|---|---|---|
| MMP21 | -0.543451668 | 0.339510971 | 0.478032149 | down |
| RP11-517P14.2 | -0.543436302 | 0.255060105 | 0.390154565 | down |
| EMP2 | -0.543327175 | 0.020708733 | 0.063721136 | down |
| TEX38 | -0.543297927 | 0.473828911 | 0.60407858 | down |
| RPL18AP3 | -0.54318893 | 0.018622786 | 0.058857877 | down |
| SIGLEC8 | -0.543144698 | 0.088430233 | 0.185220156 | down |
| AC004156.3 | -0.543143034 | 0.322277689 | 0.461048558 | down |
| ZNF397 | -0.543141569 | 0.0002782 | 0.002194342 | down |
| CRABP1 | -0.543108065 | 0.259847565 | 0.395044037 | down |
| ESCO2 | -0.542960485 | 0.050933621 | 0.123957043 | down |
| TTC4 | -0.542942247 | 0.118382554 | 0.227865005 | down |
| LINC00235 | -0.542872646 | 0.131747334 | 0.24562314 | down |
| TMED3 | -0.542790293 | 0.036908038 | 0.097792141 | down |
| C20orf203 | -0.542741479 | 0.340358291 | 0.4786876 | down |
| C18orf32 | -0.542728492 | 0.029928046 | 0.083611513 | down |
| ZSCAN16-AS1 | -0.542398012 | 0.025076577 | 0.073477116 | down |
| NA | -0.542292376 | 0.311584493 | 0.450237197 | down |
| MVK | -0.542292026 | 0.003134104 | 0.014978766 | down |
| DPM2 | -0.542160121 | 0.02491601 | 0.073103184 | down |
| RP11-466F5.10 | -0.54204397 | 0.538824255 | | down |
| NPIPB11 | -0.541990294 | 0.060691313 | 0.140936586 | down |
| LUZP1 | -0.541921728 | 0.005865783 | 0.024534536 | down |
| UBL5P2 | -0.541876004 | 0.302700359 | 0.440616744 | down |
| TTLL9 | -0.541697996 | 0.319068923 | 0.457841931 | down |
| FAXC | -0.541650398 | 0.181034936 | 0.306505913 | down |
| RP3-425C14.6 | -0.541505795 | 0.722010484 | | down |
| ZDHHC14 | -0.541504627 | 0.000820208 | 0.005182403 | down |
| BCDIN3D | -0.541480737 | 0.01621854 | 0.052960241 | down |
| POU3F3 | -0.541464359 | 0.31437013 | 0.452994542 | down |
| RN7SL526P | -0.541437984 | 0.710148656 | | down |
| NA | -0.541106438 | 0.179443875 | 0.304605446 | down |
| CDH13 | -0.541094229 | 0.193259679 | 0.321340074 | down |
| NA | -0.54109296 | 0.111800726 | 0.218937623 | down |
| JPH4 | -0.541030686 | 0.173498737 | 0.29796479 | down |
| XXyac-YX65C7_A.[illegible] | -0.541026152 | 0.18398135 | 0.309822076 | down |
| RNU6-531P | -0.540844822 | 0.638084995 | | down |
| CDK2AP2P2 | -0.540814468 | 0.567014228 | 0.685059535 | down |
| MBOAT1 | -0.54079681 | 0.006798605 | 0.027395021 | down |
| SULT1A3 | -0.540670725 | 0.275198707 | 0.411478247 | down |
| HABP2 | -0.540582646 | 0.500678596 | 0.627825843 | down |
| WNT1 | -0.540296811 | 0.229562591 | 0.361888492 | down |
| NA | -0.5402433 | 0.078429681 | 0.169728541 | down |
| RP11-617I14.1 | -0.540044637 | 0.726864862 | | down |
| RP11-89F3.2 | -0.540030495 | 0.341422408 | 0.479779926 | down |
| ESPNL | -0.539862928 | 0.125677442 | 0.237534792 | down |
| NDUFS7 | -0.53980354 | 0.020495728 | 0.063247087 | down |
| CTD-2517O10.6 | -0.539728361 | 0.128941751 | 0.241908073 | down |
| CTB-174O21.2 | -0.539500379 | 0.525346812 | 0.649180258 | down |
| CYBRD1 | -0.539423188 | 0.094935488 | 0.194825259 | down |
| RP11-102N12.3 | -0.539382108 | 0.095873702 | 0.196147736 | down |
| RP11-61O1.2 | -0.539221378 | 0.444843795 | 0.578291159 | down |

| | | | | |
|---|---|---|---|---|
| RNF145 | -0.539115164 | 0.028947235 | 0.081783968 | down |
| IFT74 | -0.539039468 | 0.000791703 | 0.005031931 | down |
| GPR42 | -0.538978326 | 0.329382442 | 0.467822399 | down |
| GTF2IP5 | -0.538901807 | 0.566222608 | | down |
| MSRB2 | -0.538845599 | 0.004318041 | 0.019345546 | down |
| RP11-374M1.5 | -0.538817081 | 0.385017889 | 0.522752958 | down |
| CCDC159 | -0.538415791 | 0.00910292 | 0.034138671 | down |
| FAM53A | -0.538343437 | 0.092508064 | 0.191175181 | down |
| ACAA1 | -0.53829032 | 0.019022204 | 0.059761906 | down |
| CNPPD1 | -0.538131957 | 0.000801307 | 0.005086516 | down |
| RP11-196B3.2 | -0.538130239 | 0.625904037 | | down |
| CTC-329D1.2 | -0.538103817 | 0.079285083 | 0.171025347 | down |
| HSD17B1 | -0.537944429 | 0.01293321 | 0.04456536 | down |
| CTB-5506.10 | -0.5379371 | 0.504913894 | 0.631568051 | down |
| C1orf53 | -0.537880414 | 0.088822592 | 0.185769993 | down |
| LTB4R | -0.537873087 | 0.051711664 | 0.125302462 | down |
| SLC20A2 | -0.537671781 | 0.0018203 | 0.009741557 | down |
| SURF1 | -0.53765837 | 0.003198777 | 0.015223201 | down |
| RP11-280K24.4 | -0.537604384 | 0.710240838 | | down |
| SPOCK1 | -0.537504879 | 0.165406873 | 0.288202987 | down |
| NFKBIB | -0.537502406 | 0.000566787 | 0.003849548 | down |
| HEXDC | -0.537396056 | 0.037253635 | 0.098482025 | down |
| RP11-800A3.3 | -0.537293508 | 0.367368565 | 0.50590004 | down |
| OSBP2 | -0.537283186 | 0.035138703 | 0.094343805 | down |
| MYCL | -0.537198316 | 0.025352446 | 0.074065897 | down |
| Y_RNA | -0.53714312 | 0.467822649 | 0.598911866 | down |
| RP11-390E23.6 | -0.537127965 | 0.002457923 | 0.01240481 | down |
| NAGPA | -0.537084893 | 0.000260107 | 0.002077811 | down |
| ATG10 | -0.536926624 | 0.003971899 | 0.018143792 | down |
| RP11-18H21.1 | -0.53680145 | 0.195602745 | 0.324088065 | down |
| PABPC1P1 | -0.536737567 | 0.590374621 | | down |
| AC004980.7 | -0.536730956 | 0.065801405 | 0.149338073 | down |
| SAMM50 | -0.536675839 | 0.000592175 | 0.003989979 | down |
| RP11-34E5.4 | -0.536622335 | 0.662976083 | | down |
| RP11-497H16.8 | -0.536545599 | 0.352780133 | 0.491578605 | down |
| KPTN | -0.536532331 | 0.018299543 | 0.058111349 | down |
| NA | -0.5364478 | 0.367662984 | 0.506235871 | down |
| MGST2 | -0.536304959 | 0.009279039 | 0.03463945 | down |
| PEX6 | -0.536233046 | 0.004975854 | 0.021615753 | down |
| RP11-109D9.4 | -0.536091515 | 0.57543144 | | down |
| EIF1AXP1 | -0.536069497 | 0.159431433 | 0.280623234 | down |
| TFCP2L1 | -0.535930245 | 0.102999727 | 0.206225662 | down |
| XRCC6P2 | -0.535888057 | 0.121977174 | 0.232663464 | down |
| RP11-350N15.5 | -0.535801207 | 0.03972307 | 0.103409966 | down |
| TNK1 | -0.535793482 | 5.97E-05 | 0.00061746 | down |
| KCTD9P5 | -0.535767213 | 0.854707606 | | down |
| RNA5SP202 | -0.535767178 | 0.853338936 | | down |
| RP11-304F15.6 | -0.535767108 | 0.850404114 | | down |
| KYNUP3 | -0.535766681 | 0.82796133 | | down |
| NXNL1 | -0.535766669 | 0.827159147 | | down |
| AHCYP2 | -0.535766652 | 0.826047667 | | down |

| | | | | |
|---|---|---|---|---|
| AC009166.5 | -0.535766422 | 0.808170143 | | down |
| NA | -0.535766225 | 0.787387011 | | down |
| RP11-273B20.3 | -0.535766213 | 0.785907367 | | down |
| HNRNPA1P51 | -0.53576621 | 0.785489112 | | down |
| RNU6-652P | -0.53576621 | 0.785507276 | | down |
| RP11-678G15.2 | -0.535766207 | 0.785192159 | | down |
| TSEN15P2 | -0.535766191 | 0.783121471 | | down |
| RAD21L1 | -0.535766186 | 0.782485786 | | down |
| RP11-96H17.3 | -0.53576566 | 0.639320945 | | down |
| NCR3 | -0.535669947 | 0.196888587 | 0.325503605 | down |
| LCN8 | -0.535610296 | 0.449237773 | 0.582137098 | down |
| RP11-856M7.6 | -0.535218308 | 0.476739198 | 0.606502052 | down |
| RP11-396B14.2 | -0.535145928 | 0.135172998 | 0.249932935 | down |
| RP11-307C12.11 | -0.535139926 | 0.106533206 | 0.211503578 | down |
| MIR765 | -0.534879305 | 0.690783389 | | down |
| HACE1 | -0.534874636 | 0.108561363 | 0.214509542 | down |
| RNU6-516P | -0.534801296 | 0.09734222 | 0.19818539 | down |
| RP11-442G21.2 | -0.534533932 | 0.532051794 | 0.655131324 | down |
| DFNB59 | -0.534457867 | 0.034821101 | 0.093700205 | down |
| RP11-416N13.1 | -0.534434193 | 0.593009286 | 0.707757159 | down |
| TTC12 | -0.534238572 | 0.004556846 | 0.020201548 | down |
| RP11-483H20.4 | -0.534235901 | 0.483511734 | | down |
| RUSC1-AS1 | -0.534228868 | 0.04694111 | 0.116729199 | down |
| MAP2K6 | -0.534045932 | 0.001168936 | 0.006865982 | down |
| ZNF891 | -0.534044534 | 0.028531258 | 0.08097733 | down |
| MXI1 | -0.533837858 | 0.010591199 | 0.038343997 | down |
| RP3-341D10.1 | -0.533697999 | 0.552697356 | | down |
| RP11-680C21.1 | -0.533685869 | 0.506176092 | 0.632488542 | down |
| MRPL37 | -0.53365249 | 0.000561053 | 0.00381882 | down |
| RP11-96H19.1 | -0.533348801 | 0.037765964 | 0.09952646 | down |
| C12orf60 | -0.533309056 | 0.115616991 | 0.224117483 | down |
| ZNF836 | -0.533289115 | 0.004082346 | 0.018538391 | down |
| SPDYA | -0.533245292 | 0.320799108 | 0.459547437 | down |
| RP11-734K2.4 | -0.533164236 | 0.028794793 | 0.081460524 | down |
| LINC00092 | -0.533152152 | 0.089753367 | 0.187185768 | down |
| NA | -0.532970386 | 0.038785333 | 0.101504054 | down |
| GAK | -0.532840197 | 2.07E-05 | 0.000258239 | down |
| RP11-215P8.1 | -0.532751918 | 0.410941465 | 0.547751791 | down |
| RP11-499P1.2 | -0.532720914 | 0.691740935 | | down |
| AC078941.1 | -0.532698804 | 0.426939905 | 0.562465982 | down |
| RP11-409C19.2 | -0.532658469 | 0.576129426 | | down |
| RP3-426I6.5 | -0.532657237 | 0.352918061 | 0.49170141 | down |
| RP11-469J4.3 | -0.532647149 | 0.375780334 | 0.514043818 | down |
| RP11-818O24.2 | -0.532644786 | 0.635896532 | | down |
| RP11-283G6.5 | -0.532577421 | 0.348871658 | 0.487579666 | down |
| RP11-299L17.1 | -0.532574686 | 0.458939933 | 0.590888851 | down |
| RP5-965F6.2 | -0.532536084 | 0.147260065 | 0.265652318 | down |
| SLC12A7 | -0.532528584 | 0.004570031 | 0.020233128 | down |
| EFNB1 | -0.532483659 | 0.02465749 | 0.072564367 | down |
| ZNF813 | -0.53212911 | 0.009411305 | 0.03501588 | down |
| TFAP2A | -0.532127 | 0.193901797 | 0.322015675 | down |

| | | | | |
|---|---|---|---|---|
| PCNX3 | -0.532085322 | 0.004678858 | 0.020608649 | down |
| RP5-827C21.2 | -0.532011077 | 0.647965818 | | down |
| CTC-559E9.6 | -0.531656748 | 0.082372792 | 0.176042691 | down |
| NXPE1 | -0.531637321 | 0.152272073 | 0.271747085 | down |
| ADGRL3 | -0.531624796 | 0.153651307 | 0.273315563 | down |
| ZNF709 | -0.531436884 | 0.294800455 | 0.432195622 | down |
| RP11-429P3.8 | -0.531240858 | 0.415436586 | 0.551858195 | down |
| RP11-16E12.1 | -0.531210169 | 0.047223469 | 0.117303174 | down |
| LINC00339 | -0.531136053 | 0.254641477 | 0.389672925 | down |
| ARHGEF4 | -0.530901212 | 0.072651312 | 0.16071983 | down |
| NA | -0.53085253 | 0.625318224 | | down |
| RPL28 | -0.530622206 | 0.028547616 | 0.080997693 | down |
| RP11-17M16.2 | -0.530615912 | 0.094208965 | 0.193810884 | down |
| RP11-286H14.4 | -0.530567824 | 0.253758029 | 0.388776452 | down |
| RP3-332B22.1 | -0.530528754 | 0.825883527 | | down |
| RTTN | -0.530453121 | 0.033769458 | 0.091592454 | down |
| SHPRH | -0.530361674 | 0.161985343 | 0.283904308 | down |
| AKR1D1 | -0.530262673 | 0.436049186 | 0.570685735 | down |
| SERPINA1 | -0.530257457 | 0.163993903 | 0.286322223 | down |
| RP11-350G8.5 | -0.53018306 | 0.153730662 | 0.273375791 | down |
| LRIT3 | -0.530170573 | 0.271276909 | 0.407389956 | down |
| ATP5C1P1 | -0.530099749 | 0.387077421 | 0.5248614 | down |
| KIF1A | -0.529886948 | 0.233876973 | 0.366617958 | down |
| SOCS6 | -0.529838657 | 0.022194387 | 0.067042839 | down |
| RP3-395M20.2 | -0.529720216 | 0.386792741 | 0.524499057 | down |
| ARHGAP27 | -0.529395561 | 0.000162051 | 0.00140591 | down |
| FAM50A | -0.529326036 | 0.012124861 | 0.04250634 | down |
| SLC27A1 | -0.529282846 | 0.024927842 | 0.073123617 | down |
| RPS23P2 | -0.529255206 | 0.448765293 | 0.581677572 | down |
| FAHD2A | -0.52918333 | 3.07E-05 | 0.000357105 | down |
| RP11-138I18.2 | -0.528670142 | 0.40850108 | 0.545264968 | down |
| FANK1 | -0.528666642 | 0.005430657 | 0.023120115 | down |
| TMPRSS4-AS1 | -0.528643442 | 0.189520439 | 0.31668757 | down |
| ATAD3A | -0.528631018 | 0.018563008 | 0.05873692 | down |
| PSME2P2 | -0.528410601 | 0.055084495 | 0.131252168 | down |
| RP11-310H4.3 | -0.528367357 | 0.644028782 | | down |
| RP11-779O18.1 | -0.528153119 | 0.548229182 | | down |
| HRAS | -0.527878792 | 0.020232882 | 0.062667702 | down |
| LCLAT1 | -0.52784866 | 0.01234185 | 0.043081043 | down |
| RP11-531A24.5 | -0.527773043 | 0.150402998 | 0.269516145 | down |
| PRKCSH | -0.527613426 | 0.000940563 | 0.005770525 | down |
| RPL7P9 | -0.527531431 | 0.002452966 | 0.012383954 | down |
| RP1-179N16.6 | -0.52739684 | 0.168360568 | 0.291725296 | down |
| C5orf52 | -0.527277364 | 0.195593143 | 0.324088065 | down |
| KCNH8 | -0.527003183 | 0.238897041 | 0.372353645 | down |
| LINC00886 | -0.526960688 | 0.111461243 | 0.218529209 | down |
| H1FNT | -0.526957746 | 0.514950491 | 0.640204112 | down |
| RP11-61I13.3 | -0.526808237 | 0.22673299 | 0.358847388 | down |
| RP11-409K20.7 | -0.5266301 | 0.408209854 | | down |
| BGLAP | -0.526509616 | 0.22084772 | 0.352520927 | down |
| PCDHB9 | -0.526423947 | 0.166619049 | 0.289716538 | down |

| | | | | |
|---|---|---|---|---|
| SMAGP | -0.526400913 | 0.028933028 | 0.081766899 | down |
| RP11-956J14.2 | -0.526358163 | 0.857231553 | | down |
| CTD-2651C21.3 | -0.526358163 | 0.857231553 | | down |
| OR11K2P | -0.526358163 | 0.857231553 | | down |
| RP11-606P2.1 | -0.526358071 | 0.853174655 | | down |
| DDI1 | -0.526358041 | 0.851784845 | | down |
| DPRXP5 | -0.52635804 | 0.851705359 | | down |
| NA | -0.526358022 | 0.850833071 | | down |
| NA | -0.526357672 | 0.830367839 | | down |
| RP11-108M9.2 | -0.52635767 | 0.830191016 | | down |
| RNU6-1101P | -0.526357644 | 0.828339195 | | down |
| RP11-184D12.1 | -0.526357643 | 0.828266249 | | down |
| RNU6-509P | -0.526357437 | 0.810675881 | | down |
| MIR3127 | -0.526357419 | 0.808820136 | | down |
| RP11-357K6.1 | -0.526357408 | 0.807743396 | | down |
| HNRNPA1P28 | -0.526357257 | 0.789884058 | | down |
| LYST-AS1 | -0.526357252 | 0.78913027 | | down |
| PPIAP21 | -0.526357228 | 0.785777335 | | down |
| KRT8P29 | -0.526357103 | 0.765291649 | | down |
| NA | -0.526357088 | 0.762366106 | | down |
| RNU6-304P | -0.526356915 | 0.719052394 | | down |
| NA | -0.526356853 | 0.69660396 | | down |
| CTA-797E19.1 | -0.52635676 | 0.649366149 | | down |
| NA | -0.526246209 | 0.243741707 | 0.377774013 | down |
| HEMGN | -0.526144886 | 0.4304309 | 0.565544843 | down |
| NUCB1 | -0.526088566 | 0.010467283 | 0.037985149 | down |
| CMB9-55F22.1 | -0.526046027 | 0.153641245 | 0.273313847 | down |
| BLVRB | -0.525856613 | 0.005464202 | 0.023216868 | down |
| SLC25A11 | -0.525758047 | 0.005391306 | 0.022991684 | down |
| BTD | -0.525693539 | 0.003279513 | 0.015526576 | down |
| LRFN3 | -0.525484342 | 0.001414608 | 0.007963697 | down |
| RP11-42901.1 | -0.525369209 | 0.613226303 | | down |
| USP8 | -0.525356942 | 0.000262428 | 0.002092462 | down |
| RP4-561L24.3 | -0.525075 | 0.284560919 | 0.421378493 | down |
| IFIT1P1 | -0.524997023 | 0.654811082 | | down |
| RP11-529J17.3 | -0.524970534 | 0.602882029 | | down |
| RMND1 | -0.52482495 | 7.90E-05 | 0.000777432 | down |
| RP11-420K14.6 | -0.524723728 | 0.43050439 | 0.565547612 | down |
| TSPAN3 | -0.524627471 | 0.024230073 | 0.071622227 | down |
| GNMT | -0.524537361 | 0.160584169 | 0.282040849 | down |
| RP11-80A15.1 | -0.524478253 | 0.096943515 | 0.197676866 | down |
| ASGR1 | -0.52420578 | 0.042980227 | 0.109655055 | down |
| AP002954.4 | -0.524066441 | 0.388115835 | 0.525747426 | down |
| RP11-727F15.11 | -0.523893746 | 0.551830844 | 0.671821293 | down |
| CH507-154B10.1 | -0.523854429 | 0.53660289 | 0.65894291 | down |
| AC004854.4 | -0.523817608 | 0.433812516 | 0.568749487 | down |
| CPNE9 | -0.523791192 | 0.208090398 | 0.33830454 | down |
| CLCNKB | -0.523634363 | 0.336400365 | 0.474565083 | down |
| MMP27 | -0.523539005 | 0.397952201 | 0.534971405 | down |
| L3MBTL4 | -0.523445418 | 0.06127398 | 0.142034986 | down |
| GUSBP3 | -0.523278487 | 0.507068603 | 0.633287708 | down |

| | | | | |
|---|---|---|---|---|
| RP11-367N14.3 | -0.523189417 | 0.356769383 | 0.495253862 | down |
| ZNF77 | -0.523115119 | 0.064897258 | 0.147788465 | down |
| PCDHGB9P | -0.523050854 | 0.567752151 | | down |
| U2AF1L4 | -0.523030323 | 0.015365817 | 0.050893023 | down |
| RP11-108M9.3 | -0.522994982 | 0.451033227 | 0.583970838 | down |
| SNRPEP4 | -0.52284316 | 0.141582173 | 0.258316317 | down |
| SIGLEC6 | -0.522796688 | 0.157500549 | 0.278235226 | down |
| AC113167.1 | -0.522603431 | 0.549301185 | 0.669716008 | down |
| AC083873.4 | -0.522599744 | 0.312584715 | 0.451244243 | down |
| RP11-182N22.9 | -0.522487827 | 0.405188166 | 0.541693288 | down |
| SURF2 | -0.522388455 | 0.01155473 | 0.041005233 | down |
| RP11-464F9.20 | -0.522341249 | 0.099608343 | 0.201415606 | down |
| PRKAR2A | -0.522267523 | 0.000973897 | 0.00592543 | down |
| AC068831.16 | -0.522065779 | 0.638113379 | | down |
| NA | -0.521863072 | 0.016819164 | 0.054387576 | down |
| RP5-1142A6.2 | -0.521802957 | 0.243976539 | 0.377932818 | down |
| PTDSS2 | -0.521576179 | 0.017164122 | 0.055235528 | down |
| AC078852.1 | -0.521574343 | 0.434890903 | 0.569690965 | down |
| NA | -0.521164634 | 0.392891429 | 0.530311974 | down |
| USP30 | -0.521151416 | 0.001058064 | 0.006342474 | down |
| CTB-51J22.1 | -0.52113047 | 0.188177967 | 0.315035041 | down |
| C17orf82 | -0.521117945 | 0.200400841 | 0.329511978 | down |
| NA | -0.52097839 | 0.396122726 | 0.533180018 | down |
| HTATIP2 | -0.520859342 | 0.007247479 | 0.028703303 | down |
| CD5L | -0.520857295 | 0.474318337 | 0.604456882 | down |
| ERBIN | -0.52075624 | 0.022669766 | 0.068108657 | down |
| POLR2J4 | -0.520522965 | 0.075435497 | 0.165043991 | down |
| IP6K3 | -0.520425197 | 0.243995314 | 0.377932818 | down |
| DENND1A | -0.520250686 | 7.22E-05 | 0.000719193 | down |
| DDX49 | -0.520093885 | 0.001899537 | 0.010059876 | down |
| MIR4689 | -0.520087378 | 0.858914482 | | down |
| PRAMEF7 | -0.520087378 | 0.858914482 | | down |
| PRAMEF9 | -0.520087378 | 0.858914482 | | down |
| RP11-293F5.8 | -0.520087378 | 0.858914482 | | down |
| SCARNA24 | -0.520087378 | 0.858914482 | | down |
| RNU6-40P | -0.520087378 | 0.858914482 | | down |
| MIR4254 | -0.520087378 | 0.858914482 | | down |
| MIR552 | -0.520087378 | 0.858914482 | | down |
| RNU6-753P | -0.520087378 | 0.858914482 | | down |
| GOT2P1 | -0.520087378 | 0.858914482 | | down |
| RP11-342H21.2 | -0.520087378 | 0.858914482 | | down |
| RP11-339A11.1 | -0.520087378 | 0.858914482 | | down |
| NA | -0.520087378 | 0.858914482 | | down |
| MTCO1P21 | -0.520087378 | 0.858914482 | | down |
| LINC01349 | -0.520087378 | 0.858914482 | | down |
| RP11-478L17.1 | -0.520087378 | 0.858914482 | | down |
| ALX3 | -0.520087378 | 0.858914482 | | down |
| SNORA25 | -0.520087378 | 0.858914482 | | down |
| NUTF2P4 | -0.520087378 | 0.858914482 | | down |
| Y_RNA | -0.520087378 | 0.858914482 | | down |
| RP5-998N21.3 | -0.520087378 | 0.858914482 | | down |

| | | | |
|---|---|---|---|
| RN7SL261P | -0.520087378 | 0.858914482 | down |
| RNU6-1062P | -0.520087378 | 0.858914482 | down |
| RP11-137P24.5 | -0.520087378 | 0.858914482 | down |
| MRPS21P2 | -0.520087378 | 0.858914482 | down |
| OR10J5 | -0.520087378 | 0.858914482 | down |
| RP11-419N10.6 | -0.520087378 | 0.858914482 | down |
| RP11-312J18.3 | -0.520087378 | 0.858914482 | down |
| SIGLEC30P | -0.520087378 | 0.858914482 | down |
| RNA5SP68 | -0.520087378 | 0.858914482 | down |
| NA | -0.520087378 | 0.858914482 | down |
| TEDDM2P | -0.520087378 | 0.858914482 | down |
| RP11-317P15.6 | -0.520087378 | 0.858914482 | down |
| RNU6-41P | -0.520087378 | 0.858914482 | down |
| GS1-120K12.4 | -0.520087378 | 0.858914482 | down |
| RP11-445J9.1 | -0.520087378 | 0.858914482 | down |
| RP11-98G13.1 | -0.520087378 | 0.858914482 | down |
| RP11-2L13.1 | -0.520087378 | 0.858914482 | down |
| MIR6740 | -0.520087378 | 0.858914482 | down |
| NA | -0.520087378 | 0.858914482 | down |
| RP11-323I1.1 | -0.520087378 | 0.858914482 | down |
| VDAC1P10 | -0.520087378 | 0.858914482 | down |
| SNORD116 | -0.520087378 | 0.858914482 | down |
| RP11-224O19.5 | -0.520087378 | 0.858914482 | down |
| RP11-412H9.2 | -0.520087378 | 0.858914482 | down |
| RNU6-1008P | -0.520087378 | 0.858914482 | down |
| HMGB1P26 | -0.520087378 | 0.858914482 | down |
| RP11-66A2.2 | -0.520087378 | 0.858914482 | down |
| MTND4P10 | -0.520087378 | 0.858914482 | down |
| RSL24D1P4 | -0.520087378 | 0.858914482 | down |
| OR2T27 | -0.520087378 | 0.858914482 | down |
| AC093326.1 | -0.520087378 | 0.858914482 | down |
| AC093326.2 | -0.520087378 | 0.858914482 | down |
| AC010729.3 | -0.520087378 | 0.858914482 | down |
| RNU7-138P | -0.520087378 | 0.858914482 | down |
| AC110754.3 | -0.520087378 | 0.858914482 | down |
| RP11-120J4.1 | -0.520087378 | 0.858914482 | down |
| RNU6-936P | -0.520087378 | 0.858914482 | down |
| AL133247.2 | -0.520087378 | 0.858914482 | down |
| Metazoa_SRP | -0.520087378 | 0.858914482 | down |
| AC087073.1 | -0.520087378 | 0.858914482 | down |
| MIR217 | -0.520087378 | 0.858914482 | down |
| RP11-511I11.2 | -0.520087378 | 0.858914482 | down |
| RP11-340F16.2 | -0.520087378 | 0.858914482 | down |
| Y_RNA | -0.520087378 | 0.858914482 | down |
| RN7SKP164 | -0.520087378 | 0.858914482 | down |
| AC012075.2 | -0.520087378 | 0.858914482 | down |
| MIR4780 | -0.520087378 | 0.858914482 | down |
| CH17-132F21.4 | -0.520087378 | 0.858914482 | down |
| NA | -0.520087378 | 0.858914482 | down |
| AC103563.3 | -0.520087378 | 0.858914482 | down |
| AC079395.1 | -0.520087378 | 0.858914482 | down |

| | | | |
|---|---|---|---|
| AC068538.2 | -0.520087378 | 0.858914482 | down |
| NA | -0.520087378 | 0.858914482 | down |
| AC096669.1 | -0.520087378 | 0.858914482 | down |
| SETD6P1 | -0.520087378 | 0.858914482 | down |
| Y_RNA | -0.520087378 | 0.858914482 | down |
| RP11-56018.1 | -0.520087378 | 0.858914482 | down |
| AC097499.1 | -0.520087378 | 0.858914482 | down |
| MIR4783 | -0.520087378 | 0.858914482 | down |
| RNU4-48P | -0.520087378 | 0.858914482 | down |
| AJ239322.3 | -0.520087378 | 0.858914482 | down |
| RN7SKP103 | -0.520087378 | 0.858914482 | down |
| EDDM3CP | -0.520087378 | 0.858914482 | down |
| AHCYP4 | -0.520087378 | 0.858914482 | down |
| RP11-514A9.1 | -0.520087378 | 0.858914482 | down |
| AC062032.1 | -0.520087378 | 0.858914482 | down |
| AC104777.3 | -0.520087378 | 0.858914482 | down |
| PHBP4 | -0.520087378 | 0.858914482 | down |
| RPLP0P7 | -0.520087378 | 0.858914482 | down |
| AC007740.1 | -0.520087378 | 0.858914482 | down |
| RNU6-627P | -0.520087378 | 0.858914482 | down |
| RNA5SP112 | -0.520087378 | 0.858914482 | down |
| AC011998.1 | -0.520087378 | 0.858914482 | down |
| AC011998.2 | -0.520087378 | 0.858914482 | down |
| AC013733.4 | -0.520087378 | 0.858914482 | down |
| RPL23AP33 | -0.520087378 | 0.858914482 | down |
| AC114760.1 | -0.520087378 | 0.858914482 | down |
| UBE2V1P11 | -0.520087378 | 0.858914482 | down |
| AC007557.3 | -0.520087378 | 0.858914482 | down |
| RP11-574016.1 | -0.520087378 | 0.858914482 | down |
| HIGD1AP4 | -0.520087378 | 0.858914482 | down |
| NA | -0.520087378 | 0.858914482 | down |
| NA | -0.520087378 | 0.858914482 | down |
| SNRPGP8 | -0.520087378 | 0.858914482 | down |
| AC007677.2 | -0.520087378 | 0.858914482 | down |
| AC017104.4 | -0.520087378 | 0.858914482 | down |
| RN7SL204P | -0.520087378 | 0.858914482 | down |
| MIR3133 | -0.520087378 | 0.858914482 | down |
| AC133680.1 | -0.520087378 | 0.858914482 | down |
| AC133141.1 | -0.520087378 | 0.858914482 | down |
| HMGB3P12 | -0.520087378 | 0.858914482 | down |
| AC123900.2 | -0.520087378 | 0.858914482 | down |
| RP11-528N21.1 | -0.520087378 | 0.858914482 | down |
| RN7SKP58 | -0.520087378 | 0.858914482 | down |
| RP4-672N11.1 | -0.520087378 | 0.858914482 | down |
| RN7SL870P | -0.520087378 | 0.858914482 | down |
| RNA5SP131 | -0.520087378 | 0.858914482 | down |
| IQCF5-AS1 | -0.520087378 | 0.858914482 | down |
| RP11-875H7.2 | -0.520087378 | 0.858914482 | down |
| RNU6-139P | -0.520087378 | 0.858914482 | down |
| RNU6-739P | -0.520087378 | 0.858914482 | down |
| NA | -0.520087378 | 0.858914482 | down |

| | | | |
|---|---|---|---|
| RP11-118N24.2 | -0.520087378 | 0.858914482 | down |
| RPS18P6 | -0.520087378 | 0.858914482 | down |
| OR5K3 | -0.520087378 | 0.858914482 | down |
| RAP1BP2 | -0.520087378 | 0.858914482 | down |
| MTND3P6 | -0.520087378 | 0.858914482 | down |
| RP11-397K18.1 | -0.520087378 | 0.858914482 | down |
| MIR4445 | -0.520087378 | 0.858914482 | down |
| RP11-274J15.2 | -0.520087378 | 0.858914482 | down |
| RP11-17403.6 | -0.520087378 | 0.858914482 | down |
| SNORA5 | -0.520087378 | 0.858914482 | down |
| RP11-77P16.1 | -0.520087378 | 0.858914482 | down |
| RP11-237P21.1 | -0.520087378 | 0.858914482 | down |
| NA | -0.520087378 | 0.858914482 | down |
| HMGN2P25 | -0.520087378 | 0.858914482 | down |
| GM2AP1 | -0.520087378 | 0.858914482 | down |
| RNU6-505P | -0.520087378 | 0.858914482 | down |
| NA | -0.520087378 | 0.858914482 | down |
| RP11-639F1.3 | -0.520087378 | 0.858914482 | down |
| RP11-79M21.3 | -0.520087378 | 0.858914482 | down |
| RPL6P8 | -0.520087378 | 0.858914482 | down |
| RNU2-20P | -0.520087378 | 0.858914482 | down |
| RN7SKP40 | -0.520087378 | 0.858914482 | down |
| RNU6-1317P | -0.520087378 | 0.858914482 | down |
| MIR4448 | -0.520087378 | 0.858914482 | down |
| RP11-95L3.2 | -0.520087378 | 0.858914482 | down |
| CCT6P4 | -0.520087378 | 0.858914482 | down |
| AC128709.2 | -0.520087378 | 0.858914482 | down |
| RP11-460I19.2 | -0.520087378 | 0.858914482 | down |
| RP3-513G18.2 | -0.520087378 | 0.858914482 | down |
| USP17L6P | -0.520087378 | 0.858914482 | down |
| RP11-608B3.1 | -0.520087378 | 0.858914482 | down |
| RP11-556G22.1 | -0.520087378 | 0.858914482 | down |
| HNRNPA1P65 | -0.520087378 | 0.858914482 | down |
| RP11-802H3.2 | -0.520087378 | 0.858914482 | down |
| snoU13 | -0.520087378 | 0.858914482 | down |
| RP11-324J13.2 | -0.520087378 | 0.858914482 | down |
| RN7SL691P | -0.520087378 | 0.858914482 | down |
| RP11-1281K21.9 | -0.520087378 | 0.858914482 | down |
| MTCO3P42 | -0.520087378 | 0.858914482 | down |
| FCF1P8 | -0.520087378 | 0.858914482 | down |
| RN7SL357P | -0.520087378 | 0.858914482 | down |
| NA | -0.520087378 | 0.858914482 | down |
| MTCYBP16 | -0.520087378 | 0.858914482 | down |
| MTND4LP31 | -0.520087378 | 0.858914482 | down |
| RP11-468N14.8 | -0.520087378 | 0.858914482 | down |
| RP11-618I10.3 | -0.520087378 | 0.858914482 | down |
| RP11-622A1.2 | -0.520087378 | 0.858914482 | down |
| MINOS1P4 | -0.520087378 | 0.858914482 | down |
| MIR575 | -0.520087378 | 0.858914482 | down |
| MIR4452 | -0.520087378 | 0.858914482 | down |
| RP11-147K6.2 | -0.520087378 | 0.858914482 | down |

| | | | |
|---|---|---|---|
| RN7SKP244 | -0.520087378 | 0.858914482 | down |
| RNA5SP164 | -0.520087378 | 0.858914482 | down |
| HMGB3P15 | -0.520087378 | 0.858914482 | down |
| RP11-61G24.1 | -0.520087378 | 0.858914482 | down |
| RP11-255I10.1 | -0.520087378 | 0.858914482 | down |
| RP11-362M19.1 | -0.520087378 | 0.858914482 | down |
| AC096732.2 | -0.520087378 | 0.858914482 | down |
| TMEM248P1 | -0.520087378 | 0.858914482 | down |
| RN7SL205P | -0.520087378 | 0.858914482 | down |
| AARSP1 | -0.520087378 | 0.858914482 | down |
| RP11-404I7.1 | -0.520087378 | 0.858914482 | down |
| RP11-400D2.3 | -0.520087378 | 0.858914482 | down |
| RP11-192C21.2 | -0.520087378 | 0.858914482 | down |
| Metazoa_SRP | -0.520087378 | 0.858914482 | down |
| RN7SKP253 | -0.520087378 | 0.858914482 | down |
| PRMT5P1 | -0.520087378 | 0.858914482 | down |
| RPPH1-3P | -0.520087378 | 0.858914482 | down |
| RP11-392C20.1 | -0.520087378 | 0.858914482 | down |
| FABP5P12 | -0.520087378 | 0.858914482 | down |
| RP11-322J23.1 | -0.520087378 | 0.858914482 | down |
| SNORA51 | -0.520087378 | 0.858914482 | down |
| RP11-45I20.1 | -0.520087378 | 0.858914482 | down |
| RP11-237D3.1 | -0.520087378 | 0.858914482 | down |
| RP11-713C19.1 | -0.520087378 | 0.858914482 | down |
| DBET | -0.520087378 | 0.858914482 | down |
| CTD-2247C11.2 | -0.520087378 | 0.858914482 | down |
| CTD-2195M18.2 | -0.520087378 | 0.858914482 | down |
| RP11-215I16.1 | -0.520087378 | 0.858914482 | down |
| RP11-315A16.4 | -0.520087378 | 0.858914482 | down |
| NA | -0.520087378 | 0.858914482 | down |
| RPL30P7 | -0.520087378 | 0.858914482 | down |
| RP11-419C19.3 | -0.520087378 | 0.858914482 | down |
| RP1-251I12.1 | -0.520087378 | 0.858914482 | down |
| NA | -0.520087378 | 0.858914482 | down |
| RP11-432M8.19 | -0.520087378 | 0.858914482 | down |
| NA | -0.520087378 | 0.858914482 | down |
| BTG4P1 | -0.520087378 | 0.858914482 | down |
| CTD-2533K21.3 | -0.520087378 | 0.858914482 | down |
| RP11-42L13.2 | -0.520087378 | 0.858914482 | down |
| RP11-136H13.2 | -0.520087378 | 0.858914482 | down |
| RP11-1084J3.1 | -0.520087378 | 0.858914482 | down |
| C8orf59P2 | -0.520087378 | 0.858914482 | down |
| RNU1-150P | -0.520087378 | 0.858914482 | down |
| RNU7-161P | -0.520087378 | 0.858914482 | down |
| RP11-503D12.1 | -0.520087378 | 0.858914482 | down |
| RPS17P11 | -0.520087378 | 0.858914482 | down |
| MIR449A | -0.520087378 | 0.858914482 | down |
| NA | -0.520087378 | 0.858914482 | down |
| CTD-2170G1.2 | -0.520087378 | 0.858914482 | down |
| SUMO2P5 | -0.520087378 | 0.858914482 | down |
| RPL5P16 | -0.520087378 | 0.858914482 | down |

| | | | |
|---|---|---|---|
| RP11-313L6.2 | -0.520087378 | 0.858914482 | down |
| SNORA31 | -0.520087378 | 0.858914482 | down |
| CTC-554D6.1 | -0.520087378 | 0.858914482 | down |
| CTB-161J7.1 | -0.520087378 | 0.858914482 | down |
| DDX43P1 | -0.520087378 | 0.858914482 | down |
| RPL7L1P4 | -0.520087378 | 0.858914482 | down |
| RNU4-69P | -0.520087378 | 0.858914482 | down |
| AC008565.1 | -0.520087378 | 0.858914482 | down |
| MIR4460 | -0.520087378 | 0.858914482 | down |
| RNA5SP191 | -0.520087378 | 0.858914482 | down |
| AC005741.2 | -0.520087378 | 0.858914482 | down |
| AC063976.2 | -0.520087378 | 0.858914482 | down |
| SNORA27 | -0.520087378 | 0.858914482 | down |
| RNU7-156P | -0.520087378 | 0.858914482 | down |
| CTC-218H9.1 | -0.520087378 | 0.858914482 | down |
| CTB-23I7.2 | -0.520087378 | 0.858914482 | down |
| RNA5SP198 | -0.520087378 | 0.858914482 | down |
| MRPL57P6 | -0.520087378 | 0.858914482 | down |
| CTC-425K20.1 | -0.520087378 | 0.858914482 | down |
| CTB-162J7.1 | -0.520087378 | 0.858914482 | down |
| RP11-21I4.4 | -0.520087378 | 0.858914482 | down |
| MIR8089 | -0.520087378 | 0.858914482 | down |
| RNU6-705P | -0.520087378 | 0.858914482 | down |
| RP3-400B16.4 | -0.520087378 | 0.858914482 | down |
| RN7SL554P | -0.520087378 | 0.858914482 | down |
| RNU6ATAC21P | -0.520087378 | 0.858914482 | down |
| RP1-62D2.4 | -0.520087378 | 0.858914482 | down |
| RNU6-263P | -0.520087378 | 0.858914482 | down |
| RP3-348I23.3 | -0.520087378 | 0.858914482 | down |
| OR2J2 | -0.520087378 | 0.858914482 | down |
| SNORD45 | -0.520087378 | 0.858914482 | down |
| RN7SL465P | -0.520087378 | 0.858914482 | down |
| SNORA8 | -0.520087378 | 0.858914482 | down |
| RP1-202I21.3 | -0.520087378 | 0.858914482 | down |
| RNU6-250P | -0.520087378 | 0.858914482 | down |
| RP11-213N20.1 | -0.520087378 | 0.858914482 | down |
| B3GNTL1P2 | -0.520087378 | 0.858914482 | down |
| RN7SKP116 | -0.520087378 | 0.858914482 | down |
| DEFB114 | -0.520087378 | 0.858914482 | down |
| RNU6-626P | -0.520087378 | 0.858914482 | down |
| RP11-325M4.2 | -0.520087378 | 0.858914482 | down |
| BECN1P2 | -0.520087378 | 0.858914482 | down |
| SNORA70 | -0.520087378 | 0.858914482 | down |
| RP3-492P14.2 | -0.520087378 | 0.858914482 | down |
| RPL7P29 | -0.520087378 | 0.858914482 | down |
| snoU13 | -0.520087378 | 0.858914482 | down |
| Y_RNA | -0.520087378 | 0.858914482 | down |
| RP11-63K6.4 | -0.520087378 | 0.858914482 | down |
| SNORA18 | -0.520087378 | 0.858914482 | down |
| RP3-399J4.3 | -0.520087378 | 0.858914482 | down |
| NPM1P38 | -0.520087378 | 0.858914482 | down |

| | | | |
|---|---|---|---|
| TAAR9 | -0.520087378 | 0.858914482 | down |
| CCNG1P1 | -0.520087378 | 0.858914482 | down |
| RP11-394G3.2 | -0.520087378 | 0.858914482 | down |
| RAET1E-AS1 | -0.520087378 | 0.858914482 | down |
| RNU7-152P | -0.520087378 | 0.858914482 | down |
| MIR3692 | -0.520087378 | 0.858914482 | down |
| yR211F11.2 | -0.520087378 | 0.858914482 | down |
| RP11-534P19.1 | -0.520087378 | 0.858914482 | down |
| ABC7-481722F1.2 | -0.520087378 | 0.858914482 | down |
| RP11-472F21.1 | -0.520087378 | 0.858914482 | down |
| TAS2R2P | -0.520087378 | 0.858914482 | down |
| SNORA64 | -0.520087378 | 0.858914482 | down |
| GTF3AP5 | -0.520087378 | 0.858914482 | down |
| MIR1302-6 | -0.520087378 | 0.858914482 | down |
| AC004009.1 | -0.520087378 | 0.858914482 | down |
| AC073325.2 | -0.520087378 | 0.858914482 | down |
| DDX43P2 | -0.520087378 | 0.858914482 | down |
| SNORA31 | -0.520087378 | 0.858914482 | down |
| GS1-278J22.2 | -0.520087378 | 0.858914482 | down |
| RP11-775L16.1 | -0.520087378 | 0.858914482 | down |
| RP11-1217F2.19 | -0.520087378 | 0.858914482 | down |
| MTND5P7 | -0.520087378 | 0.858914482 | down |
| RP11-548K12.1 | -0.520087378 | 0.858914482 | down |
| NA | -0.520087378 | 0.858914482 | down |
| RP11-321E8.8 | -0.520087378 | 0.858914482 | down |
| AC004866.3 | -0.520087378 | 0.858914482 | down |
| LINC00972 | -0.520087378 | 0.858914482 | down |
| RP5-1121E10.2 | -0.520087378 | 0.858914482 | down |
| RN7SL252P | -0.520087378 | 0.858914482 | down |
| NA | -0.520087378 | 0.858914482 | down |
| Y_RNA | -0.520087378 | 0.858914482 | down |
| RP11-703P11.1 | -0.520087378 | 0.858914482 | down |
| RAC1P6 | -0.520087378 | 0.858914482 | down |
| AC016831.3 | -0.520087378 | 0.858914482 | down |
| AC105443.2 | -0.520087378 | 0.858914482 | down |
| AC009518.8 | -0.520087378 | 0.858914482 | down |
| AC008154.5 | -0.520087378 | 0.858914482 | down |
| AC083867.4 | -0.520087378 | 0.858914482 | down |
| RP4-813F11.3 | -0.520087378 | 0.858914482 | down |
| U66059.58 | -0.520087378 | 0.858914482 | down |
| TRBVA | -0.520087378 | 0.858914482 | down |
| TAS2R40 | -0.520087378 | 0.858914482 | down |
| PAICSP5 | -0.520087378 | 0.858914482 | down |
| AC073310.4 | -0.520087378 | 0.858914482 | down |
| C7orf33 | -0.520087378 | 0.858914482 | down |
| PAXBP1P1 | -0.520087378 | 0.858914482 | down |
| AC005588.2 | -0.520087378 | 0.858914482 | down |
| AC133633.2 | -0.520087378 | 0.858914482 | down |
| RP11-481A20.4 | -0.520087378 | 0.858914482 | down |
| Y_RNA | -0.520087378 | 0.858914482 | down |
| MTND4P7 | -0.520087378 | 0.858914482 | down |

| | | | |
|---|---|---|---|
| RN7SL474P | -0.520087378 | 0.858914482 | down |
| RPL30P9 | -0.520087378 | 0.858914482 | down |
| RP11-369E15.1 | -0.520087378 | 0.858914482 | down |
| RP11-613H2.3 | -0.520087378 | 0.858914482 | down |
| MIR4288 | -0.520087378 | 0.858914482 | down |
| RP11-566H8.1 | -0.520087378 | 0.858914482 | down |
| RANP9 | -0.520087378 | 0.858914482 | down |
| RP11-139F9.1 | -0.520087378 | 0.858914482 | down |
| NA | -0.520087378 | 0.858914482 | down |
| MTND6P20 | -0.520087378 | 0.858914482 | down |
| RP11-11C20.1 | -0.520087378 | 0.858914482 | down |
| RP11-550I15.1 | -0.520087378 | 0.858914482 | down |
| RNA5SP266 | -0.520087378 | 0.858914482 | down |
| MIR4470 | -0.520087378 | 0.858914482 | down |
| COX6CP8 | -0.520087378 | 0.858914482 | down |
| RNA5SP270 | -0.520087378 | 0.858914482 | down |
| RP11-313C15.2 | -0.520087378 | 0.858914482 | down |
| TPM3P3 | -0.520087378 | 0.858914482 | down |
| RP11-69I13.1 | -0.520087378 | 0.858914482 | down |
| RN7SKP231 | -0.520087378 | 0.858914482 | down |
| RP11-44N17.2 | -0.520087378 | 0.858914482 | down |
| SLC16A14P1 | -0.520087378 | 0.858914482 | down |
| RP11-211F2.1 | -0.520087378 | 0.858914482 | down |
| RP11-114O8.1 | -0.520087378 | 0.858914482 | down |
| MIR2053 | -0.520087378 | 0.858914482 | down |
| RNA5SP276 | -0.520087378 | 0.858914482 | down |
| SNORA31 | -0.520087378 | 0.858914482 | down |
| CDK5P1 | -0.520087378 | 0.858914482 | down |
| RP11-622O11.4 | -0.520087378 | 0.858914482 | down |
| RP11-89K10.2 | -0.520087378 | 0.858914482 | down |
| CTD-2309H9.4 | -0.520087378 | 0.858914482 | down |
| RP11-157E21.3 | -0.520087378 | 0.858914482 | down |
| MIR30D | -0.520087378 | 0.858914482 | down |
| IMP3P1 | -0.520087378 | 0.858914482 | down |
| ATP5HP3 | -0.520087378 | 0.858914482 | down |
| RP11-378I6.2 | -0.520087378 | 0.858914482 | down |
| IFNA2 | -0.520087378 | 0.858914482 | down |
| RP11-572H4.1 | -0.520087378 | 0.858914482 | down |
| SNORD121A | -0.520087378 | 0.858914482 | down |
| FAM220BP | -0.520087378 | 0.858914482 | down |
| MIR1299 | -0.520087378 | 0.858914482 | down |
| RP11-459O16.7 | -0.520087378 | 0.858914482 | down |
| NA | -0.520087378 | 0.858914482 | down |
| FKBP4P8 | -0.520087378 | 0.858914482 | down |
| MTND2P8 | -0.520087378 | 0.858914482 | down |
| RP11-15B24.1 | -0.520087378 | 0.858914482 | down |
| UBE2V1P10 | -0.520087378 | 0.858914482 | down |
| NA | -0.520087378 | 0.858914482 | down |
| OR7E108P | -0.520087378 | 0.858914482 | down |
| LINC00587 | -0.520087378 | 0.858914482 | down |
| OR13C5 | -0.520087378 | 0.858914482 | down |

| | | | |
|---|---|---|---|
| OR13C9 | -0.520087378 | 0.858914482 | down |
| RNA5SP292 | -0.520087378 | 0.858914482 | down |
| RP11-196I18.4 | -0.520087378 | 0.858914482 | down |
| ACTL7A | -0.520087378 | 0.858914482 | down |
| RNU6-1013P | -0.520087378 | 0.858914482 | down |
| RP11-523L1.2 | -0.520087378 | 0.858914482 | down |
| OR1L1 | -0.520087378 | 0.858914482 | down |
| OR1L6 | -0.520087378 | 0.858914482 | down |
| PIGFP2 | -0.520087378 | 0.858914482 | down |
| RNU6-881P | -0.520087378 | 0.858914482 | down |
| MIR3689B | -0.520087378 | 0.858914482 | down |
| LINC00708 | -0.520087378 | 0.858914482 | down |
| NA | -0.520087378 | 0.858914482 | down |
| RP11-49L2.1 | -0.520087378 | 0.858914482 | down |
| EBLN1 | -0.520087378 | 0.858914482 | down |
| GPN3P1 | -0.520087378 | 0.858914482 | down |
| MIR3611 | -0.520087378 | 0.858914482 | down |
| MTND4P18 | -0.520087378 | 0.858914482 | down |
| RP11-20F24.2 | -0.520087378 | 0.858914482 | down |
| TACC1P1 | -0.520087378 | 0.858914482 | down |
| RP11-453N3.1 | -0.520087378 | 0.858914482 | down |
| RP11-542E6.3 | -0.520087378 | 0.858914482 | down |
| RP11-507K13.4 | -0.520087378 | 0.858914482 | down |
| Y_RNA | -0.520087378 | 0.858914482 | down |
| RNU6-1250P | -0.520087378 | 0.858914482 | down |
| RPL5P26 | -0.520087378 | 0.858914482 | down |
| RP11-167P22.5 | -0.520087378 | 0.858914482 | down |
| RPSAP6 | -0.520087378 | 0.858914482 | down |
| RNA5SP321 | -0.520087378 | 0.858914482 | down |
| RPS7P9 | -0.520087378 | 0.858914482 | down |
| RNU6-129P | -0.520087378 | 0.858914482 | down |
| RP11-80H5.6 | -0.520087378 | 0.858914482 | down |
| RNU6-740P | -0.520087378 | 0.858914482 | down |
| CYP2C58P | -0.520087378 | 0.858914482 | down |
| RP11-208C17.4 | -0.520087378 | 0.858914482 | down |
| SORCS3-AS1 | -0.520087378 | 0.858914482 | down |
| RNU6-463P | -0.520087378 | 0.858914482 | down |
| RN7SKP278 | -0.520087378 | 0.858914482 | down |
| SNRPGP12 | -0.520087378 | 0.858914482 | down |
| RP11-5G18.2 | -0.520087378 | 0.858914482 | down |
| Y_RNA | -0.520087378 | 0.858914482 | down |
| FAM24A | -0.520087378 | 0.858914482 | down |
| MRPS21P6 | -0.520087378 | 0.858914482 | down |
| OR4F2P | -0.520087378 | 0.858914482 | down |
| RP11-304M2.3 | -0.520087378 | 0.858914482 | down |
| OR7E117P | -0.520087378 | 0.858914482 | down |
| OR51H2P | -0.520087378 | 0.858914482 | down |
| OR51A5P | -0.520087378 | 0.858914482 | down |
| OR51K1P | -0.520087378 | 0.858914482 | down |
| HMGN2P36 | -0.520087378 | 0.858914482 | down |
| RNA5SP332 | -0.520087378 | 0.858914482 | down |

| | | | |
|---|---|---|---|
| RN7SL188P | -0.520087378 | 0.858914482 | down |
| RP11-787P24.2 | -0.520087378 | 0.858914482 | down |
| MIR3973 | -0.520087378 | 0.858914482 | down |
| OR4A46P | -0.520087378 | 0.858914482 | down |
| OR4A40P | -0.520087378 | 0.858914482 | down |
| OR4A45P | -0.520087378 | 0.858914482 | down |
| CTD-2132H18.3 | -0.520087378 | 0.858914482 | down |
| CBX3P8 | -0.520087378 | 0.858914482 | down |
| OR4C50P | -0.520087378 | 0.858914482 | down |
| TRIM51HP | -0.520087378 | 0.858914482 | down |
| OR5M11 | -0.520087378 | 0.858914482 | down |
| OR5AR1 | -0.520087378 | 0.858914482 | down |
| OR9G2P | -0.520087378 | 0.858914482 | down |
| OR5AZ1P | -0.520087378 | 0.858914482 | down |
| TMA16P1 | -0.520087378 | 0.858914482 | down |
| RNU7-23P | -0.520087378 | 0.858914482 | down |
| RPS3AP41 | -0.520087378 | 0.858914482 | down |
| MIR4692 | -0.520087378 | 0.858914482 | down |
| RP11-263C24.3 | -0.520087378 | 0.858914482 | down |
| CTD-2555I5.1 | -0.520087378 | 0.858914482 | down |
| RNU6-544P | -0.520087378 | 0.858914482 | down |
| U6 | -0.520087378 | 0.858914482 | down |
| RP11-643G5.1 | -0.520087378 | 0.858914482 | down |
| RP11-358N4.6 | -0.520087378 | 0.858914482 | down |
| AP000648.3 | -0.520087378 | 0.858914482 | down |
| RP11-313I2.7 | -0.520087378 | 0.858914482 | down |
| UBTFL1 | -0.520087378 | 0.858914482 | down |
| MIR1261 | -0.520087378 | 0.858914482 | down |
| RP11-379J13.1 | -0.520087378 | 0.858914482 | down |
| RN7SKP53 | -0.520087378 | 0.858914482 | down |
| RP11-37O16.8 | -0.520087378 | 0.858914482 | down |
| OR2AL1P | -0.520087378 | 0.858914482 | down |
| RP11-794P6.6 | -0.520087378 | 0.858914482 | down |
| ACA59 | -0.520087378 | 0.858914482 | down |
| AP000797.3 | -0.520087378 | 0.858914482 | down |
| AP000797.1 | -0.520087378 | 0.858914482 | down |
| AP000797.2 | -0.520087378 | 0.858914482 | down |
| RP11-334E6.4 | -0.520087378 | 0.858914482 | down |
| HMGB1P42 | -0.520087378 | 0.858914482 | down |
| RNU6-592P | -0.520087378 | 0.858914482 | down |
| U8 | -0.520087378 | 0.858914482 | down |
| OR6X1 | -0.520087378 | 0.858914482 | down |
| NA | -0.520087378 | 0.858914482 | down |
| AP002856.7 | -0.520087378 | 0.858914482 | down |
| RNU6ATAC12P | -0.520087378 | 0.858914482 | down |
| AP004372.1 | -0.520087378 | 0.858914482 | down |
| RP3-461F17.2 | -0.520087378 | 0.858914482 | down |
| RP11-20D14.4 | -0.520087378 | 0.858914482 | down |
| RP11-377D9.1 | -0.520087378 | 0.858914482 | down |
| RPL7P40 | -0.520087378 | 0.858914482 | down |
| PSMC1P8 | -0.520087378 | 0.858914482 | down |

| | | | |
|---|---|---|---|
| GYS2 | -0.520087378 | 0.858914482 | down |
| RPS27P22 | -0.520087378 | 0.858914482 | down |
| RP11-153K16.1 | -0.520087378 | 0.858914482 | down |
| NA | -0.520087378 | 0.858914482 | down |
| RNA5SP358 | -0.520087378 | 0.858914482 | down |
| FAM166AP9 | -0.520087378 | 0.858914482 | down |
| RNA5SP360 | -0.520087378 | 0.858914482 | down |
| OR6C74 | -0.520087378 | 0.858914482 | down |
| OR2AP1 | -0.520087378 | 0.858914482 | down |
| RP11-644F5.17 | -0.520087378 | 0.858914482 | down |
| RP11-74M13.3 | -0.520087378 | 0.858914482 | down |
| RP11-1022B3.1 | -0.520087378 | 0.858914482 | down |
| RP11-415I12.1 | -0.520087378 | 0.858914482 | down |
| SNORD83 | -0.520087378 | 0.858914482 | down |
| APOOP3 | -0.520087378 | 0.858914482 | down |
| Metazoa_SRP | -0.520087378 | 0.858914482 | down |
| MIR1251 | -0.520087378 | 0.858914482 | down |
| RNU6-36P | -0.520087378 | 0.858914482 | down |
| MIR4495 | -0.520087378 | 0.858914482 | down |
| AC190387.1 | -0.520087378 | 0.858914482 | down |
| RP11-385N17.3 | -0.520087378 | 0.858914482 | down |
| SNORA38 | -0.520087378 | 0.858914482 | down |
| RNU6-927P | -0.520087378 | 0.858914482 | down |
| RP11-955H22.4 | -0.520087378 | 0.858914482 | down |
| NA | -0.520087378 | 0.858914482 | down |
| Y_RNA | -0.520087378 | 0.858914482 | down |
| SNORA49 | -0.520087378 | 0.858914482 | down |
| NANOGNBP2 | -0.520087378 | 0.858914482 | down |
| AC226150.3 | -0.520087378 | 0.858914482 | down |
| GXYLT1P1 | -0.520087378 | 0.858914482 | down |
| RP11-408E5.6 | -0.520087378 | 0.858914482 | down |
| BASP1P1 | -0.520087378 | 0.858914482 | down |
| FGFR1OP2P1 | -0.520087378 | 0.858914482 | down |
| RNY1P4 | -0.520087378 | 0.858914482 | down |
| RP11-218I21.2 | -0.520087378 | 0.858914482 | down |
| RP11-282M24.1 | -0.520087378 | 0.858914482 | down |
| RNA5SP26 | -0.520087378 | 0.858914482 | down |
| CDKN2AIPNLP3 | -0.520087378 | 0.858914482 | down |
| RPS28P8 | -0.520087378 | 0.858914482 | down |
| RP11-415G4.1 | -0.520087378 | 0.858914482 | down |
| RPL31P53 | -0.520087378 | 0.858914482 | down |
| HMGN2P39 | -0.520087378 | 0.858914482 | down |
| LINC00434 | -0.520087378 | 0.858914482 | down |
| RPS3AP52 | -0.520087378 | 0.858914482 | down |
| RPL12P34 | -0.520087378 | 0.858914482 | down |
| RP11-475F14.1 | -0.520087378 | 0.858914482 | down |
| SNORA68 | -0.520087378 | 0.858914482 | down |
| LINC01034 | -0.520087378 | 0.858914482 | down |
| RP11-52L5.6 | -0.520087378 | 0.858914482 | down |
| HNRNPA1P31 | -0.520087378 | 0.858914482 | down |
| HIGD1AP2 | -0.520087378 | 0.858914482 | down |

| | | | |
|---|---|---|---|
| 3C8-43014700C16. | -0.520087378 | 0.858914482 | down |
| RNY4P27 | -0.520087378 | 0.858914482 | down |
| RP11-383H17.4 | -0.520087378 | 0.858914482 | down |
| FTLP8 | -0.520087378 | 0.858914482 | down |
| SNORA25 | -0.520087378 | 0.858914482 | down |
| RP11-754I20.3 | -0.520087378 | 0.858914482 | down |
| RP11-536C10.13 | -0.520087378 | 0.858914482 | down |
| RP11-536C10.15 | -0.520087378 | 0.858914482 | down |
| MED15P1 | -0.520087378 | 0.858914482 | down |
| ARHGAP42P4 | -0.520087378 | 0.858914482 | down |
| OR4N1P | -0.520087378 | 0.858914482 | down |
| OR4N5 | -0.520087378 | 0.858914482 | down |
| AE000658.30 | -0.520087378 | 0.858914482 | down |
| TRAV31 | -0.520087378 | 0.858914482 | down |
| RP11-30H9.1 | -0.520087378 | 0.858914482 | down |
| RP11-269C4.1 | -0.520087378 | 0.858914482 | down |
| RPL12P5 | -0.520087378 | 0.858914482 | down |
| MRPL57P8 | -0.520087378 | 0.858914482 | down |
| RN7SKP21 | -0.520087378 | 0.858914482 | down |
| RP11-629F19.1 | -0.520087378 | 0.858914482 | down |
| RP11-90P16.2 | -0.520087378 | 0.858914482 | down |
| RP11-557C9.3 | -0.520087378 | 0.858914482 | down |
| RP11-831F12.4 | -0.520087378 | 0.858914482 | down |
| RNA5SP385 | -0.520087378 | 0.858914482 | down |
| RP11-1042B17.1 | -0.520087378 | 0.858914482 | down |
| CTD-2302E22.1 | -0.520087378 | 0.858914482 | down |
| RNU7-116P | -0.520087378 | 0.858914482 | down |
| RPS28P1 | -0.520087378 | 0.858914482 | down |
| RPPH1-2P | -0.520087378 | 0.858914482 | down |
| Y_RNA | -0.520087378 | 0.858914482 | down |
| RP11-332E19.2 | -0.520087378 | 0.858914482 | down |
| RP11-688D15.1 | -0.520087378 | 0.858914482 | down |
| RP11-661G16.1 | -0.520087378 | 0.858914482 | down |
| RNU6-366P | -0.520087378 | 0.858914482 | down |
| RNU6-1258P | -0.520087378 | 0.858914482 | down |
| MIR665 | -0.520087378 | 0.858914482 | down |
| MIR370 | -0.520087378 | 0.858914482 | down |
| MIR496 | -0.520087378 | 0.858914482 | down |
| RN7SL634P | -0.520087378 | 0.858914482 | down |
| IGHVII-43-1D | -0.520087378 | 0.858914482 | down |
| RP11-1360M22.2 | -0.520087378 | 0.858914482 | down |
| RP11-403B2.5 | -0.520087378 | 0.858914482 | down |
| ZNF519P2 | -0.520087378 | 0.858914482 | down |
| IGHD1OR15-1B | -0.520087378 | 0.858914482 | down |
| GOLGA6L1 | -0.520087378 | 0.858914482 | down |
| SNORD115-22 | -0.520087378 | 0.858914482 | down |
| SNORD115-23 | -0.520087378 | 0.858914482 | down |
| SNORD115-37 | -0.520087378 | 0.858914482 | down |
| RP11-1084I9.2 | -0.520087378 | 0.858914482 | down |
| RP11-483E23.7 | -0.520087378 | 0.858914482 | down |
| SNORA18 | -0.520087378 | 0.858914482 | down |

| | | | |
|---|---|---|---|
| Y_RNA | -0.520087378 | 0.858914482 | down |
| MTND5P40 | -0.520087378 | 0.858914482 | down |
| HNRNPA1P74 | -0.520087378 | 0.858914482 | down |
| MESTP2 | -0.520087378 | 0.858914482 | down |
| RP11-745A24.3 | -0.520087378 | 0.858914482 | down |
| RP11-299H22.7 | -0.520087378 | 0.858914482 | down |
| NA | -0.520087378 | 0.858914482 | down |
| CTD-2071N1.2 | -0.520087378 | 0.858914482 | down |
| Metazoa_SRP | -0.520087378 | 0.858914482 | down |
| RNU6-401P | -0.520087378 | 0.858914482 | down |
| CSPG4P5 | -0.520087378 | 0.858914482 | down |
| MIR548AP | -0.520087378 | 0.858914482 | down |
| NA | -0.520087378 | 0.858914482 | down |
| RP11-697E2.12 | -0.520087378 | 0.858914482 | down |
| AC068831.12 | -0.520087378 | 0.858914482 | down |
| RPL7P5 | -0.520087378 | 0.858914482 | down |
| RP11-19H6.1 | -0.520087378 | 0.858914482 | down |
| RP11-204E4.4 | -0.520087378 | 0.858914482 | down |
| CTA-345G4.1 | -0.520087378 | 0.858914482 | down |
| RP11-989E6.3 | -0.520087378 | 0.858914482 | down |
| IGHV1OR16-4 | -0.520087378 | 0.858914482 | down |
| ZNF971P | -0.520087378 | 0.858914482 | down |
| RP11-80F22.15 | -0.520087378 | 0.858914482 | down |
| RP11-352B15.2 | -0.520087378 | 0.858914482 | down |
| RP11-44I10.5 | -0.520087378 | 0.858914482 | down |
| RP11-305A4.2 | -0.520087378 | 0.858914482 | down |
| RP11-152014.3 | -0.520087378 | 0.858914482 | down |
| RP11-46107.2 | -0.520087378 | 0.858914482 | down |
| MT1B | -0.520087378 | 0.858914482 | down |
| GNPATP | -0.520087378 | 0.858914482 | down |
| RNA5SP428 | -0.520087378 | 0.858914482 | down |
| RP11-521L9.1 | -0.520087378 | 0.858914482 | down |
| RN7SL33P | -0.520087378 | 0.858914482 | down |
| RNU6-955P | -0.520087378 | 0.858914482 | down |
| NA | -0.520087378 | 0.858914482 | down |
| RNU6-405P | -0.520087378 | 0.858914482 | down |
| RP1-37N7.6 | -0.520087378 | 0.858914482 | down |
| RP11-822E23.4 | -0.520087378 | 0.858914482 | down |
| NA | -0.520087378 | 0.858914482 | down |
| RP11-19P22.3 | -0.520087378 | 0.858914482 | down |
| H3F3BP2 | -0.520087378 | 0.858914482 | down |
| MIR4725 | -0.520087378 | 0.858914482 | down |
| Y_RNA | -0.520087378 | 0.858914482 | down |
| CTD-2206N4.1 | -0.520087378 | 0.858914482 | down |
| RP11-6N17.10 | -0.520087378 | 0.858914482 | down |
| Metazoa_SRP | -0.520087378 | 0.858914482 | down |
| RP11-613C6.4 | -0.520087378 | 0.858914482 | down |
| BC1D3P1-DHX40P | -0.520087378 | 0.858914482 | down |
| Y_RNA | -0.520087378 | 0.858914482 | down |
| Y_RNA | -0.520087378 | 0.858914482 | down |
| POLR3KP2 | -0.520087378 | 0.858914482 | down |

| | | | |
|---|---|---|---|
| RNU1-80P | -0.520087378 | 0.858914482 | down |
| RP11-672L10.3 | -0.520087378 | 0.858914482 | down |
| MIR4317 | -0.520087378 | 0.858914482 | down |
| RP11-856M7.8 | -0.520087378 | 0.858914482 | down |
| RP11-64C12.9 | -0.520087378 | 0.858914482 | down |
| RNU6-170P | -0.520087378 | 0.858914482 | down |
| NF1P5 | -0.520087378 | 0.858914482 | down |
| RP11-757O6.7 | -0.520087378 | 0.858914482 | down |
| EXOGP1 | -0.520087378 | 0.858914482 | down |
| Metazoa_SRP | -0.520087378 | 0.858914482 | down |
| RP11-322E11.1 | -0.520087378 | 0.858914482 | down |
| AC083760.1 | -0.520087378 | 0.858914482 | down |
| MIR4319 | -0.520087378 | 0.858914482 | down |
| TCEB3C | -0.520087378 | 0.858914482 | down |
| RNU6-1131P | -0.520087378 | 0.858914482 | down |
| ADAD1P2 | -0.520087378 | 0.858914482 | down |
| RP11-397A16.3 | -0.520087378 | 0.858914482 | down |
| RP11-325K19.6 | -0.520087378 | 0.858914482 | down |
| RP11-1096D5.2 | -0.520087378 | 0.858914482 | down |
| RP11-567M16.5 | -0.520087378 | 0.858914482 | down |
| RP11-1137G4.5 | -0.520087378 | 0.858914482 | down |
| MBD3L3 | -0.520087378 | 0.858914482 | down |
| CTC-543D15.3 | -0.520087378 | 0.858914482 | down |
| CTD-3116E22.6 | -0.520087378 | 0.858914482 | down |
| OR7A2P | -0.520087378 | 0.858914482 | down |
| OR10H2 | -0.520087378 | 0.858914482 | down |
| CTD-2542C24.8 | -0.520087378 | 0.858914482 | down |
| VN1R88P | -0.520087378 | 0.858914482 | down |
| AC005357.1 | -0.520087378 | 0.858914482 | down |
| AC011524.3 | -0.520087378 | 0.858914482 | down |
| CTC-525D6.5 | -0.520087378 | 0.858914482 | down |
| CTC-400I9.1 | -0.520087378 | 0.858914482 | down |
| Y_RNA | -0.520087378 | 0.858914482 | down |
| CTB-60E11.11 | -0.520087378 | 0.858914482 | down |
| CTD-2616J11.15 | -0.520087378 | 0.858914482 | down |
| CTD-3073N11.7 | -0.520087378 | 0.858914482 | down |
| RPL19P1 | -0.520087378 | 0.858914482 | down |
| RP4-775C13.3 | -0.520087378 | 0.858914482 | down |
| RPS11P1 | -0.520087378 | 0.858914482 | down |
| RPS3P1 | -0.520087378 | 0.858914482 | down |
| RP11-388K2.1 | -0.520087378 | 0.858914482 | down |
| LLPHP1 | -0.520087378 | 0.858914482 | down |
| RP4-753D10.5 | -0.520087378 | 0.858914482 | down |
| BSNDP1 | -0.520087378 | 0.858914482 | down |
| PCMTD1P7 | -0.520087378 | 0.858914482 | down |
| DUX4L33 | -0.520087378 | 0.858914482 | down |
| 5_8S_rRNA | -0.520087378 | 0.858914482 | down |
| DEFB115 | -0.520087378 | 0.858914482 | down |
| RP5-1121H13.3 | -0.520087378 | 0.858914482 | down |
| RP4-753D4.2 | -0.520087378 | 0.858914482 | down |
| RN7SKP33 | -0.520087378 | 0.858914482 | down |

| | | | |
|---|---|---|---|
| SRMP1 | -0.520087378 | 0.858914482 | down |
| RP5-897D18.1 | -0.520087378 | 0.858914482 | down |
| RP4-614C15.3 | -0.520087378 | 0.858914482 | down |
| NA | -0.520087378 | 0.858914482 | down |
| CH507-254M2.3 | -0.520087378 | 0.858914482 | down |
| MTCO1P1 | -0.520087378 | 0.858914482 | down |
| SNX19P1 | -0.520087378 | 0.858914482 | down |
| OR4K11P | -0.520087378 | 0.858914482 | down |
| TERF1P1 | -0.520087378 | 0.858914482 | down |
| AL157359.3 | -0.520087378 | 0.858914482 | down |
| AP001506.1 | -0.520087378 | 0.858914482 | down |
| CLDN17 | -0.520087378 | 0.858914482 | down |
| KRTAP13-1 | -0.520087378 | 0.858914482 | down |
| KRTAP19-3 | -0.520087378 | 0.858914482 | down |
| KRTAP19-4 | -0.520087378 | 0.858914482 | down |
| KRTAP19-5 | -0.520087378 | 0.858914482 | down |
| KRTAP19-6 | -0.520087378 | 0.858914482 | down |
| KRTAP19-7 | -0.520087378 | 0.858914482 | down |
| KRTAP8-3P | -0.520087378 | 0.858914482 | down |
| AP000290.7 | -0.520087378 | 0.858914482 | down |
| RNU6-992P | -0.520087378 | 0.858914482 | down |
| DSCR4 | -0.520087378 | 0.858914482 | down |
| RPSAP64 | -0.520087378 | 0.858914482 | down |
| RPS26P4 | -0.520087378 | 0.858914482 | down |
| YME1L1P1 | -0.520087378 | 0.858914482 | down |
| LA16c-23H5.4 | -0.520087378 | 0.858914482 | down |
| LA16c-4G1.3 | -0.520087378 | 0.858914482 | down |
| RN7SL168P | -0.520087378 | 0.858914482 | down |
| KB-1592A4.16 | -0.520087378 | 0.858914482 | down |
| IGLVV-66 | -0.520087378 | 0.858914482 | down |
| RP1-213J1P__B.2 | -0.520087378 | 0.858914482 | down |
| RFPL1 | -0.520087378 | 0.858914482 | down |
| MRPS16P3 | -0.520087378 | 0.858914482 | down |
| RP11-706O15.8 | -0.520087378 | 0.858914482 | down |
| VCX2 | -0.520087378 | 0.858914482 | down |
| RNA5SP499 | -0.520087378 | 0.858914482 | down |
| RP11-126O22.5 | -0.520087378 | 0.858914482 | down |
| LOXNC01-138C3.[illegible] | -0.520087378 | 0.858914482 | down |
| RNU6-266P | -0.520087378 | 0.858914482 | down |
| RP11-494I9.1 | -0.520087378 | 0.858914482 | down |
| RP13-60P5.2 | -0.520087378 | 0.858914482 | down |
| GS1-466O4.5 | -0.520087378 | 0.858914482 | down |
| RP11-268G12.2 | -0.520087378 | 0.858914482 | down |
| RP5-1158E12.1 | -0.520087378 | 0.858914482 | down |
| GAGE13 | -0.520087378 | 0.858914482 | down |
| TPMTP3 | -0.520087378 | 0.858914482 | down |
| RP11-485B17.5 | -0.520087378 | 0.858914482 | down |
| VTRNA3-1P | -0.520087378 | 0.858914482 | down |
| SHC1P1 | -0.520087378 | 0.858914482 | down |
| RP13-57D9.3 | -0.520087378 | 0.858914482 | down |
| RP13-278H16.2 | -0.520087378 | 0.858914482 | down |

| | | | | |
|---|---|---|---|---|
| RP3-481A17.1 | -0.520087378 | 0.858914482 | | down |
| NDUFA5P7 | -0.520087378 | 0.858914482 | | down |
| GEMIN8P3 | -0.520087378 | 0.858914482 | | down |
| RP5-998G20.1 | -0.520087378 | 0.858914482 | | down |
| RP11-471G13.5 | -0.520087378 | 0.858914482 | | down |
| LOXNC01-237H1.[illegible] | -0.520087378 | 0.858914482 | | down |
| H2BFWT | -0.520087378 | 0.858914482 | | down |
| TEX13A | -0.520087378 | 0.858914482 | | down |
| EEF1A1P40 | -0.520087378 | 0.858914482 | | down |
| RP6-149D17.1 | -0.520087378 | 0.858914482 | | down |
| RP5-1168A5.1 | -0.520087378 | 0.858914482 | | down |
| RN7SL93P | -0.520087378 | 0.858914482 | | down |
| MIR1912 | -0.520087378 | 0.858914482 | | down |
| MIR448 | -0.520087378 | 0.858914482 | | down |
| RNU6-648P | -0.520087378 | 0.858914482 | | down |
| RP11-197B12.1 | -0.520087378 | 0.858914482 | | down |
| Y_RNA | -0.520087378 | 0.858914482 | | down |
| RP1-138017.1 | -0.520087378 | 0.858914482 | | down |
| OR11N1P | -0.520087378 | 0.858914482 | | down |
| OR2AF1P | -0.520087378 | 0.858914482 | | down |
| RNU1-115P | -0.520087378 | 0.858914482 | | down |
| RP11-265D19.7 | -0.520087378 | 0.858914482 | | down |
| RP11-2906.2 | -0.520087378 | 0.858914482 | | down |
| RN7SKP189 | -0.520087378 | 0.858914482 | | down |
| GS1-115M3.3 | -0.520087378 | 0.858914482 | | down |
| DUTP4 | -0.520087378 | 0.858914482 | | down |
| RP11-316K19.3 | -0.520087378 | 0.858914482 | | down |
| RPSAP60 | -0.520087378 | 0.858914482 | | down |
| RP13-228J13.10 | -0.520087378 | 0.858914482 | | down |
| TBL1YP1 | -0.520087378 | 0.858914482 | | down |
| RBMY1E | -0.520087378 | 0.858914482 | | down |
| TTTY25P | -0.520087378 | 0.858914482 | | down |
| LINC00265-2P | -0.520087378 | 0.858914482 | | down |
| AC006328.9 | -0.520087378 | 0.858914482 | | down |
| CYCSP48 | -0.520087378 | 0.858914482 | | down |
| DLGAP3 | -0.520003233 | 0.041978162 | 0.107757339 | down |
| MIR181A2HG | -0.51993711 | 0.186340957 | 0.312639064 | down |
| IPP | -0.519919237 | 7.40E-05 | 0.000734155 | down |
| NDUFS5 | -0.51989139 | 0.017463503 | 0.056018948 | down |
| SH2D1B | -0.519581147 | 0.182843371 | 0.308632297 | down |
| RP11-301N24.3 | -0.519541001 | 0.828981286 | | down |
| ZDHHC20P1 | -0.519486547 | 0.387137315 | 0.524878298 | down |
| RP11-380B4.3 | -0.519353722 | 0.203750164 | 0.333159351 | down |
| STK4-AS1 | -0.519343158 | 0.248989978 | 0.383524988 | down |
| RPL39P3 | -0.519271872 | 0.004059607 | 0.018463037 | down |
| LINC00476 | -0.519269466 | 0.002315867 | 0.011830242 | down |
| DMD | -0.519162816 | 0.116302821 | 0.225040378 | down |
| AC016700.5 | -0.518912945 | 0.132937132 | 0.247074742 | down |
| RRAS2 | -0.51890885 | 0.006325765 | 0.025963291 | down |
| SFXN4 | -0.51865724 | 0.006223247 | 0.025668758 | down |
| BIRC6-AS2 | -0.518515799 | 0.533183067 | 0.656117406 | down |

| | | | | |
|---|---|---|---|---|
| CORT | -0.518477462 | 0.213393609 | 0.344262342 | down |
| PANK3 | -0.518450814 | 0.040418831 | 0.10475535 | down |
| DENND4C | -0.518027402 | 0.03699187 | 0.097936531 | down |
| SIDT2 | -0.518025639 | 0.000132232 | 0.001191948 | down |
| ZNF763 | -0.518019582 | 0.036340486 | 0.096749097 | down |
| NA | -0.517851437 | 0.742101924 | | down |
| ATP1B1 | -0.517813856 | 0.103582598 | 0.207068706 | down |
| RPS19P1 | -0.517759962 | 0.249753265 | 0.384306637 | down |
| RP11-266K4.13 | -0.517612192 | 0.538284957 | 0.660387654 | down |
| RP11-734J24.1 | -0.517438852 | 0.100288678 | 0.202369068 | down |
| RP11-267M23.4 | -0.517434548 | 0.145481893 | 0.263225414 | down |
| RP3-508I15.21 | -0.517165234 | 0.15818252 | 0.279111742 | down |
| RP11-958N24.2 | -0.517112494 | 0.421005133 | 0.556675795 | down |
| ZNF773 | -0.517069405 | 0.006046789 | 0.025092505 | down |
| ZNF394 | -0.516957725 | 0.000145909 | 0.001289323 | down |
| RN7SL616P | -0.516949113 | 0.859756959 | | down |
| RPL29P22 | -0.516949113 | 0.859756959 | | down |
| KRT18P44 | -0.516949113 | 0.859756959 | | down |
| NA | -0.516949001 | 0.854229028 | | down |
| RP11-290012.1 | -0.516948934 | 0.850637113 | | down |
| RP11-701I24.1 | -0.51694866 | 0.832391517 | | down |
| CTD-2576N18.1 | -0.516948446 | 0.812301197 | | down |
| ZNF680P1 | -0.516948435 | 0.811058391 | | down |
| RP11-10C8.2 | -0.516948416 | 0.808832891 | | down |
| NA | -0.516948282 | 0.790892551 | | down |
| RPS7P8 | -0.516948277 | 0.79013202 | | down |
| PRR27 | -0.516948268 | 0.788700582 | | down |
| TRAJ47 | -0.516948065 | 0.746648523 | | down |
| NA | -0.516947988 | 0.722277862 | | down |
| LINC01154 | -0.516947931 | 0.69934921 | | down |
| SNORA70 | -0.516947853 | 0.655320584 | | down |
| NEUROG3 | -0.516867241 | 0.257709351 | 0.392727353 | down |
| AC105402.4 | -0.516717058 | 0.65719478 | | down |
| AC022154.7 | -0.516662325 | 0.418067086 | 0.554176375 | down |
| RP11-83A24.1 | -0.51659879 | 0.650792546 | 0.754715377 | down |
| ZNF252P-AS1 | -0.516444013 | 0.272344226 | 0.408597719 | down |
| AC156455.1 | -0.516316964 | 0.047792149 | 0.118343964 | down |
| ZNF26 | -0.516276973 | 0.012922296 | 0.044543078 | down |
| RP11-159D12.3 | -0.516258058 | 0.534614777 | 0.657305011 | down |
| A2MP1 | -0.516225982 | 0.255617018 | 0.39064844 | down |
| ACRBP | -0.516147598 | 0.027937682 | 0.079681006 | down |
| PARP10 | -0.516095964 | 0.022612771 | 0.067964996 | down |
| NA | -0.515847215 | 0.860025028 | | down |
| AC093382.1 | -0.515845575 | 0.431373901 | 0.566466956 | down |
| BCKDHA | -0.515776489 | 0.369354798 | 0.507727652 | down |
| AUTS2 | -0.515698377 | 0.006277415 | 0.025807149 | down |
| RP4-756H11.3 | -0.515415337 | 0.011411764 | 0.04060809 | down |
| MRPS12 | -0.515221312 | 0.01010405 | 0.036966158 | down |
| BRIP1 | -0.515213283 | 0.076568451 | 0.166854468 | down |
| RP11-46I8.4 | -0.515180496 | 0.772424039 | | down |
| AC097724.3 | -0.515160221 | 0.148288606 | 0.267020533 | down |

| | | | | |
|---|---|---|---|---|
| RASA4 | -0.515093191 | 0.065231825 | 0.148336813 | down |
| PLEKHA8P1 | -0.514794991 | 0.115824822 | 0.224375585 | down |
| FAM21FP | -0.514665848 | 0.109453741 | 0.215719509 | down |
| QPRT | -0.514585633 | 0.003910583 | 0.017923571 | down |
| RENBP | -0.514404738 | 0.01578897 | 0.051912025 | down |
| AP003068.23 | -0.514381795 | 0.021187481 | 0.064822472 | down |
| NA | -0.514338095 | 0.342805428 | 0.481228216 | down |
| USP34 | -0.514290399 | 0.129377885 | 0.242424696 | down |
| ZNF311 | -0.514218279 | 0.077303312 | 0.167992817 | down |
| CSPG4P12 | -0.514164978 | 0.067820566 | 0.15275516 | down |
| RP5-956018.2 | -0.513585813 | 0.466362268 | 0.59762798 | down |
| RP11-574K11.5 | -0.513565688 | 0.353320481 | 0.492012075 | down |
| CTD-2012J19.1 | -0.513481633 | 0.428247831 | 0.563593579 | down |
| RP5-943J3.2 | -0.513447806 | 0.106329763 | 0.211141532 | down |
| RP11-848P1.9 | -0.51342489 | 0.023058341 | 0.068890692 | down |
| RGN | -0.513368413 | 0.220490224 | 0.352156094 | down |
| USP54 | -0.513326501 | 0.040540709 | 0.104946154 | down |
| KLHL40 | -0.513244767 | 0.61232712 | | down |
| CTD-2033A16.2 | -0.513124331 | 0.216726764 | 0.348017457 | down |
| IGSF6 | -0.512993122 | 0.081080299 | 0.173911367 | down |
| RPS26P28 | -0.512897101 | 0.443658461 | 0.577199793 | down |
| CTD-2588E21.1 | -0.512697422 | 0.338193263 | 0.476602016 | down |
| CH17-472G23.1 | -0.512685444 | 0.45905665 | 0.590988472 | down |
| AC104532.3 | -0.512525952 | 0.69494347 | | down |
| RP5-864K19.4 | -0.512371835 | 0.233450998 | 0.366119783 | down |
| CFL1P2 | -0.512288154 | 0.562078405 | 0.681200516 | down |
| RP4-644L1.2 | -0.512176328 | 0.530204572 | 0.653436138 | down |
| RPS15P5 | -0.512019901 | 0.399454122 | 0.536414412 | down |
| RP11-342D14.1 | -0.512016471 | 0.735355129 | 0.820256798 | down |
| LINC01058 | -0.51192354 | 0.456623587 | 0.588865478 | down |
| TP53I3 | -0.511899469 | 0.01854429 | 0.058702419 | down |
| MAPK15 | -0.511667285 | 0.133162614 | 0.247294305 | down |
| RP3-512E2.2 | -0.511556873 | 0.677394362 | | down |
| TOM1L2 | -0.511467053 | 2.80E-06 | 4.93E-05 | down |
| MTG2 | -0.511360449 | 0.014490473 | 0.048618915 | down |
| FBXL22 | -0.511120667 | 0.095392513 | 0.195386768 | down |
| NLN | -0.511086724 | 0.003168088 | 0.015098675 | down |
| RP11-571M6.18 | -0.511057526 | 0.256485805 | 0.391555946 | down |
| SMPDL3B | -0.510993415 | 0.125953638 | 0.237922106 | down |
| NA | -0.510954681 | 0.246700226 | 0.380935478 | down |
| RP11-354A14.1 | -0.510951162 | 0.457160278 | 0.58935522 | down |
| NA | -0.510698351 | 0.705583955 | | down |
| RP11-5407.10 | -0.510678328 | 0.861440849 | | down |
| SCARNA21 | -0.510678328 | 0.861440849 | | down |
| RP11-91A18.4 | -0.510678328 | 0.861440849 | | down |
| RP11-378I13.1 | -0.510678328 | 0.861440849 | | down |
| GAPDHP74 | -0.510678328 | 0.861440849 | | down |
| PPIAL4F | -0.510678328 | 0.861440849 | | down |
| RNVU1-8 | -0.510678328 | 0.861440849 | | down |
| RNVU1-2 | -0.510678328 | 0.861440849 | | down |
| NA | -0.510678328 | 0.861440849 | | down |

| | | | |
|---|---|---|---|
| RP11-551G24.2 | -0.510678328 | 0.861440849 | down |
| NA | -0.510678328 | 0.861440849 | down |
| KRT8P18 | -0.510678328 | 0.861440849 | down |
| NA | -0.510678328 | 0.861440849 | down |
| EEF1GP3 | -0.510678328 | 0.861440849 | down |
| RP13-1056D16.2 | -0.510678328 | 0.861440849 | down |
| NA | -0.510678328 | 0.861440849 | down |
| MORC1-AS1 | -0.510678328 | 0.861440849 | down |
| GS1-388B5.1 | -0.510678328 | 0.861440849 | down |
| RP11-588L15.1 | -0.510678328 | 0.861440849 | down |
| RP11-218C23.1 | -0.510678328 | 0.861440849 | down |
| FGG | -0.510678328 | 0.861440849 | down |
| RNU2-34P | -0.510678328 | 0.861440849 | down |
| Y_RNA | -0.510678328 | 0.861440849 | down |
| RNU6-1305P | -0.510678328 | 0.861440849 | down |
| CTD-2128F4.1 | -0.510678328 | 0.861440849 | down |
| AC010240.3 | -0.510678328 | 0.861440849 | down |
| RPL23AP52 | -0.510678328 | 0.861440849 | down |
| TAS2R38 | -0.510678328 | 0.861440849 | down |
| SNRPCP6 | -0.510678328 | 0.861440849 | down |
| NA | -0.510678328 | 0.861440849 | down |
| TRBV26OR9-2 | -0.510678328 | 0.861440849 | down |
| RN7SL22P | -0.510678328 | 0.861440849 | down |
| RP11-123N4.4 | -0.510678328 | 0.861440849 | down |
| SNORA74 | -0.510678328 | 0.861440849 | down |
| RP11-428G2.1 | -0.510678328 | 0.861440849 | down |
| RAB5CP1 | -0.510678328 | 0.861440849 | down |
| NDUFA8P1 | -0.510678328 | 0.861440849 | down |
| MIR608 | -0.510678328 | 0.861440849 | down |
| RNU6-43P | -0.510678328 | 0.861440849 | down |
| RP13-631K18.2 | -0.510678328 | 0.861440849 | down |
| MRGPRX3 | -0.510678328 | 0.861440849 | down |
| GLYATL1P4 | -0.510678328 | 0.861440849 | down |
| ARL2-SNX15 | -0.510678328 | 0.861440849 | down |
| RPS3AP40 | -0.510678328 | 0.861440849 | down |
| SNORA70E | -0.510678328 | 0.861440849 | down |
| RP11-665E10.2 | -0.510678328 | 0.861440849 | down |
| TRIM53AP | -0.510678328 | 0.861440849 | down |
| RP11-755E23.3 | -0.510678328 | 0.861440849 | down |
| RP11-680F20.4 | -0.510678328 | 0.861440849 | down |
| RP11-234B24.5 | -0.510678328 | 0.861440849 | down |
| RP11-711K1.8 | -0.510678328 | 0.861440849 | down |
| RP11-443N24.4 | -0.510678328 | 0.861440849 | down |
| RP11-499A10.3 | -0.510678328 | 0.861440849 | down |
| OR10P1 | -0.510678328 | 0.861440849 | down |
| RP11-575G13.2 | -0.510678328 | 0.861440849 | down |
| NA | -0.510678328 | 0.861440849 | down |
| RP11-1033H12.3 | -0.510678328 | 0.861440849 | down |
| RP11-624A21.1 | -0.510678328 | 0.861440849 | down |
| RP11-483K5.2 | -0.510678328 | 0.861440849 | down |
| CTD-2014E2.6 | -0.510678328 | 0.861440849 | down |

| | | | | |
|---|---|---|---|---|
| NA | -0.510678328 | 0.861440849 | | down |
| KRT28 | -0.510678328 | 0.861440849 | | down |
| RN7SL405P | -0.510678328 | 0.861440849 | | down |
| RP11-720L3.1 | -0.510678328 | 0.861440849 | | down |
| LONRF2P1 | -0.510678328 | 0.861440849 | | down |
| AC092316.1 | -0.510678328 | 0.861440849 | | down |
| ZIM2-AS1 | -0.510678328 | 0.861440849 | | down |
| RP5-831C21.1 | -0.510678328 | 0.861440849 | | down |
| RBMX2P1 | -0.510678328 | 0.861440849 | | down |
| LINC01425 | -0.510678328 | 0.861440849 | | down |
| NA | -0.510678328 | 0.861440849 | | down |
| NA | -0.510678328 | 0.861440849 | | down |
| LLOXNC01-39B3.1 | -0.510678328 | 0.861440849 | | down |
| RP13-347D8.1 | -0.510678328 | 0.861440849 | | down |
| TTTY13 | -0.510678328 | 0.861440849 | | down |
| CTD-2260A17.3 | -0.510605975 | 0.334595474 | 0.472850311 | down |
| ANKRD23 | -0.510605127 | 0.127045272 | 0.239322099 | down |
| KLHL36 | -0.510565181 | 0.000118537 | 0.001092095 | down |
| RGS9BP | -0.51054449 | 0.608259542 | 0.720463224 | down |
| NA | -0.510461472 | 0.715599811 | | down |
| ZDHHC1 | -0.510437209 | 0.02660424 | 0.076796483 | down |
| LCOR | -0.510374242 | 0.000289769 | 0.002267734 | down |
| SPDYE12P | -0.510283032 | 0.07742706 | 0.16812797 | down |
| NUDT7 | -0.510134496 | 0.177630343 | 0.302588187 | down |
| CPT1A | -0.510108993 | 0.008095942 | 0.031206679 | down |
| ADAM10 | -0.510026826 | 0.014431138 | 0.048490193 | down |
| NA | -0.509950552 | 0.201616099 | 0.330782005 | down |
| ASAP2 | -0.509384944 | 0.196445923 | 0.324934285 | down |
| RP11-923I11.5 | -0.509364676 | 0.575500356 | | down |
| RP11-661A12.12 | -0.509333759 | 0.286786935 | 0.423922592 | down |
| NA | -0.509324225 | 0.251267067 | 0.386023101 | down |
| CTD-3064M3.7 | -0.509318443 | 0.278680806 | 0.414982462 | down |
| LONRF2 | -0.50925091 | 0.245205746 | 0.379173475 | down |
| ADGRB1 | -0.509079199 | 0.087313775 | 0.183534251 | down |
| HOXA3 | -0.509051392 | 0.028239391 | 0.080322166 | down |
| HAMP | -0.508872859 | 0.312739196 | 0.451423869 | down |
| SLC41A2 | -0.508865132 | 0.012514163 | 0.043495548 | down |
| COMMD7 | -0.508864389 | 0.018988274 | 0.059692759 | down |
| CES1P2 | -0.508827051 | 0.723931071 | | down |
| E2F1 | -0.508802316 | 0.086543749 | 0.182413386 | down |
| FRA10AC1 | -0.508731214 | 0.000242704 | 0.001962803 | down |
| KCTD17 | -0.508688368 | 0.009762348 | 0.035961255 | down |
| TMC5 | -0.508687848 | 0.121380962 | 0.231909038 | down |
| MAGEC3 | -0.508645763 | 0.711468605 | | down |
| RP11-259K5.2 | -0.50864156 | 0.141713914 | 0.258438897 | down |
| CCDC40 | -0.50839194 | 0.047422501 | 0.117670522 | down |
| RP6-91H8.3 | -0.508367018 | 0.464035025 | 0.595560182 | down |
| RP11-87H9.5 | -0.508243234 | 0.372703498 | 0.511068183 | down |
| ATAD3C | -0.508223271 | 0.136275652 | 0.251386979 | down |
| ARAF | -0.50820617 | 0.001237874 | 0.007186607 | down |
| BPI | -0.507959337 | 0.446360873 | 0.579657433 | down |

| | | | | |
|---|---|---|---|---|
| NDUFC2 | -0.507954573 | 0.00951728 | 0.03528346 | down |
| PHF24 | -0.507953078 | 0.109630996 | 0.215983846 | down |
| MOB2 | -0.507848524 | 0.016014387 | 0.052472076 | down |
| RP11-188C12.2 | -0.507817106 | 0.124799605 | 0.236425963 | down |
| CTNND1 | -0.5077417 | 0.020765312 | 0.063829859 | down |
| DAPK3 | -0.507620999 | 0.013102243 | 0.045005706 | down |
| NA | -0.507540063 | 0.862283801 | | down |
| MIR146B | -0.507540063 | 0.862283801 | | down |
| SETP7 | -0.507540063 | 0.862283801 | | down |
| RP11-503G7.2 | -0.50753967 | 0.836184276 | | down |
| UHRF2P1 | -0.507539661 | 0.835374966 | | down |
| OR2L1P | -0.507539655 | 0.834844407 | | down |
| NA | -0.507539452 | 0.813207863 | | down |
| AC062021.1 | -0.507539451 | 0.813082423 | | down |
| RNU6-181P | -0.507539208 | 0.770853632 | | down |
| AC002400.1 | -0.50753914 | 0.752737905 | | down |
| AF196972.4 | -0.507539136 | 0.75165459 | | down |
| RP11-552F3.4 | -0.50753908 | 0.732670306 | | down |
| NA | -0.50753907 | 0.728936594 | | down |
| CHCHD3P3 | -0.507539022 | 0.708082075 | | down |
| GS1-257G1.1 | -0.507538951 | 0.665536563 | | down |
| MARK2P8 | -0.507538948 | 0.663309868 | | down |
| CPA6 | -0.507489744 | 0.247470705 | 0.381791612 | down |
| MIR3197 | -0.507481256 | 0.682441186 | | down |
| RP11-490G8.1 | -0.507464211 | 0.099308452 | 0.201054907 | down |
| UBAC2-AS1 | -0.507340589 | 0.057304823 | 0.135106811 | down |
| POLR2H | -0.507176234 | 0.000365896 | 0.002727106 | down |
| POLE4 | -0.507015199 | 0.003765728 | 0.017373698 | down |
| MAPK6 | -0.506984409 | 0.011017919 | 0.039510903 | down |
| CTD-2651B20.3 | -0.506984325 | 0.177025515 | 0.301941316 | down |
| NA | -0.506973028 | 0.213576041 | 0.344445681 | down |
| TUBGCP2 | -0.506945385 | 6.71E-05 | 0.000679354 | down |
| C14orf2 | -0.506659407 | 0.003794916 | 0.01747616 | down |
| SPATA2 | -0.506592165 | 0.000767004 | 0.004908519 | down |
| NA | -0.506562147 | 0.217615864 | 0.349025711 | down |
| LINC01433 | -0.506328556 | 0.564491615 | 0.682670615 | down |
| FANCA | -0.506293962 | 0.059244903 | 0.138463933 | down |
| NA | -0.506141259 | 0.289500656 | 0.426737204 | down |
| MYL6P5 | -0.505973155 | 0.332044189 | 0.470407678 | down |
| RP11-115H13.1 | -0.505845048 | 0.585266539 | 0.700969116 | down |
| GS1-259H13.2 | -0.505820696 | 0.20339424 | 0.332849095 | down |
| SUMF1 | -0.505713379 | 0.003542553 | 0.016539619 | down |
| TCF7L2 | -0.505524438 | 0.001289116 | 0.007401144 | down |
| CPAMD8 | -0.505466973 | 0.01518645 | 0.050393386 | down |
| RP3-414A15.12 | -0.505221669 | 0.142776198 | 0.259832097 | down |
| BRSK2 | -0.505221646 | 0.116684301 | 0.225502582 | down |
| F11R | -0.50515964 | 0.022738223 | 0.068246471 | down |
| DNAH8 | -0.505069319 | 0.260151062 | 0.395245436 | down |
| RPL10P15 | -0.505065319 | 0.218740872 | 0.35015942 | down |
| BRD3 | -0.504875735 | 0.041557565 | 0.106970141 | down |
| RP5-1186P10.2 | -0.504861785 | 0.536485883 | | down |

| | | | | |
|---|---|---|---|---|
| GFRA2 | -0.50481459 | 0.050051104 | 0.122404181 | down |
| CTD-2325A15.3 | -0.504814194 | 0.466496597 | 0.59774446 | down |
| RP11-490M8.1 | -0.504647169 | 0.042304004 | 0.108367785 | down |
| FBXO48 | -0.504582616 | 0.003274172 | 0.01550831 | down |
| DMGDH | -0.504552085 | 0.199270403 | 0.328335948 | down |
| RP11-893F2.18 | -0.504525451 | 0.328125938 | 0.466594835 | down |
| NA | -0.504386689 | 0.333010352 | 0.471354116 | down |
| CTD-2104P17.1 | -0.504362599 | 0.694262292 | | down |
| RPL12P13 | -0.504320236 | 0.425205828 | 0.560769233 | down |
| AC024084.1 | -0.504210032 | 0.576114823 | | down |
| SRD5A1P1 | -0.504200785 | 0.785266031 | 0.856384356 | down |
| HIST1H2AH | -0.503942189 | 0.331211471 | 0.469537934 | down |
| ZNRF2P2 | -0.503926767 | 0.629075128 | | down |
| NOSTRIN | -0.503900825 | 0.003635173 | 0.016872413 | down |
| CRYM | -0.503882838 | 0.065552523 | 0.148912691 | down |
| RP11-54C4.3 | -0.503656707 | 0.02915771 | 0.08220083 | down |
| MC5R | -0.503615259 | 0.594700247 | | down |
| VMAC | -0.503464074 | 0.002462458 | 0.012421438 | down |
| ARMC7 | -0.503375305 | 0.002673323 | 0.01324494 | down |
| RRM2P3 | -0.503290739 | 0.570340649 | | down |
| RP11-55K13.1 | -0.50321721 | 0.250470591 | 0.385173693 | down |
| NEBL-AS1 | -0.50320619 | 0.430125163 | 0.565321335 | down |
| UBALD2 | -0.503134581 | 0.001508861 | 0.008373148 | down |
| ATP5J2P6 | -0.502950286 | 0.81927725 | | down |
| AC006026.13 | -0.502912188 | 0.360233033 | 0.498518712 | down |
| MIPEPP2 | -0.502763531 | 0.863509971 | | down |
| RP11-175B9.2 | -0.50266872 | 0.56364429 | 0.68227756 | down |
| REG1A | -0.502634966 | 0.463239713 | 0.594895232 | down |
| AC005534.8 | -0.502471731 | 0.200190644 | 0.329238433 | down |
| FCN2 | -0.502413969 | 0.651736691 | | down |
| RP11-348N5.7 | -0.502358828 | 0.161302888 | 0.282988844 | down |
| LINC00648 | -0.502337095 | 0.623273908 | 0.732901812 | down |
| AC002310.12 | -0.502310266 | 0.043453372 | 0.110533665 | down |
| INTS4P1 | -0.502128923 | 0.330853613 | 0.469163447 | down |
| RP1-28O10.1 | -0.502039593 | 0.139364363 | 0.255511254 | down |
| AGTR1 | -0.502029947 | 0.257051198 | 0.392086283 | down |
| RP11-541M12.6 | -0.501932882 | 0.462489046 | 0.594236022 | down |
| NUTM2E | -0.501687853 | 0.457260443 | 0.589368659 | down |
| PRKAB1 | -0.501584427 | 2.61E-05 | 0.000312009 | down |
| RPL9P9 | -0.501577336 | 0.103081055 | 0.2062857 | down |
| CTC-550B14.7 | -0.501575318 | 0.183106265 | 0.3089025 | down |
| SCN2B | -0.501535797 | 0.177517194 | 0.302508606 | down |
| C16orf90 | -0.501532654 | 0.708189127 | 0.799458058 | down |
| NA | -0.501382083 | 0.225697386 | 0.357752601 | down |
| FAM21EP | -0.501379521 | 0.078592138 | 0.169994397 | down |
| RP11-486G15.1 | -0.501269278 | 0.863968633 | | down |
| RP11-98D18.16 | -0.501269278 | 0.863968633 | | down |
| RP11-383G10.1 | -0.501269278 | 0.863968633 | | down |
| RNU6-1283P | -0.501269278 | 0.863968633 | | down |
| RP11-493E12.3 | -0.501269278 | 0.863968633 | | down |
| AC074367.1 | -0.501269278 | 0.863968633 | | down |

| | | | |
|---|---|---|---|
| IQCF2 | -0.501269278 | 0.863968633 | down |
| TUSC7 | -0.501269278 | 0.863968633 | down |
| SNORD74 | -0.501269278 | 0.863968633 | down |
| RP11-681L8.1 | -0.501269278 | 0.863968633 | down |
| FTH1P21 | -0.501269278 | 0.863968633 | down |
| RP11-364P22.2 | -0.501269278 | 0.863968633 | down |
| NA | -0.501269278 | 0.863968633 | down |
| RN7SKP64 | -0.501269278 | 0.863968633 | down |
| RP3-365O12.2 | -0.501269278 | 0.863968633 | down |
| RP11-419M24.4 | -0.501269278 | 0.863968633 | down |
| RNU7-157P | -0.501269278 | 0.863968633 | down |
| RP11-10E6.1 | -0.501269278 | 0.863968633 | down |
| SNORA51 | -0.501269278 | 0.863968633 | down |
| RNU4-31P | -0.501269278 | 0.863968633 | down |
| NA | -0.501269278 | 0.863968633 | down |
| RP11-697N18.2 | -0.501269278 | 0.863968633 | down |
| RP11-678D18.1 | -0.501269278 | 0.863968633 | down |
| RP11-296C13.1 | -0.501269278 | 0.863968633 | down |
| DCAF4L2 | -0.501269278 | 0.863968633 | down |
| RP11-629O1.3 | -0.501269278 | 0.863968633 | down |
| RP11-415H23.4 | -0.501269278 | 0.863968633 | down |
| RP11-168C9.1 | -0.501269278 | 0.863968633 | down |
| OR56B4 | -0.501269278 | 0.863968633 | down |
| OR4P1P | -0.501269278 | 0.863968633 | down |
| RP11-805J14.3 | -0.501269278 | 0.863968633 | down |
| RNU6-311P | -0.501269278 | 0.863968633 | down |
| MIR708 | -0.501269278 | 0.863968633 | down |
| GLULP3 | -0.501269278 | 0.863968633 | down |
| OR7A19P | -0.501269278 | 0.863968633 | down |
| RP5-944M2.1 | -0.501269278 | 0.863968633 | down |
| LINC00407 | -0.501269278 | 0.863968633 | down |
| RN7SL60P | -0.501269278 | 0.863968633 | down |
| MIR4309 | -0.501269278 | 0.863968633 | down |
| RP11-106D4.3 | -0.501269278 | 0.863968633 | down |
| OR4D2 | -0.501269278 | 0.863968633 | down |
| CTD-2514K5.4 | -0.501269278 | 0.863968633 | down |
| RP11-699A5.2 | -0.501269278 | 0.863968633 | down |
| CTC-260E6.7 | -0.501269278 | 0.863968633 | down |
| RP5-1009E24.9 | -0.501269278 | 0.863968633 | down |
| BMP6P1 | -0.501269278 | 0.863968633 | down |
| MTND1P10 | -0.501269278 | 0.863968633 | down |
| RP11-281B1.2 | -0.501269278 | 0.863968633 | down |
| [illegible]LOXNC01-221F2.[illegible] | -0.501269278 | 0.863968633 | down |
| MT-TS1 | -0.501269278 | 0.863968633 | down |
| RP11-239L20.6 | -0.501268632 | 0.847466619 | down |
| LINC01101 | -0.50126861 | 0.846797163 | down |
| ENPP7P5 | -0.501268584 | 0.846018652 | down |
| RP11-47J17.3 | -0.501268046 | 0.825811695 | down |
| RPL7AP3 | -0.501267971 | 0.822332723 | down |
| RNU6-883P | -0.501267602 | 0.801469662 | down |
| RP11-173A16.1 | -0.501177996 | 0.674794948 | down |

| | | | | |
|---|---|---|---|---|
| AC073254.1 | -0.501125335 | 0.326200147 | 0.464569556 | down |
| AC016738.4 | -0.501094613 | 0.456646764 | 0.588870091 | down |
| TSKS | -0.500907543 | 0.24024235 | 0.373981408 | down |
| MDK | -0.500902866 | 0.038576326 | 0.101097972 | down |
| PITX1 | -0.500579176 | 0.109143069 | 0.215304949 | down |
| RP11-312J18.5 | -0.500311076 | 0.256024259 | 0.391052 | down |
| COL11A1 | -0.500309188 | 0.433049324 | 0.568021562 | down |
| RP11-317N8.3 | -0.500255905 | 0.572993256 | 0.690256992 | down |
| RP11-42I10.1 | -0.50025335 | 0.202412317 | 0.331711976 | down |
| TUFM | -0.500217581 | 0.003453614 | 0.016199901 | down |
| RP11-285J16.1 | -0.500207774 | 0.263201075 | 0.398549121 | down |
| ZSCAN23 | -0.50007122 | 0.442713794 | 0.576270232 | down |
| NA | -0.499981885 | 0.715365505 | | down |
| TOLLIP | -0.499541642 | 0.001739075 | 0.00939553 | down |
| COX8A | -0.499494929 | 0.020067062 | 0.062260298 | down |
| RP11-459C13.1 | -0.499443264 | 0.490425249 | 0.618829743 | down |
| ST13P3 | -0.4994341 | 0.523417781 | | down |
| C9orf16 | -0.499407592 | 0.064445585 | 0.147150262 | down |
| CEP135 | -0.499360463 | 0.027346323 | 0.078403753 | down |
| PACSIN1 | -0.499250421 | 0.104343093 | 0.208062911 | down |
| RP11-324E6.10 | -0.499182292 | 0.206491421 | 0.336542707 | down |
| NDRG2 | -0.499163208 | 0.002705338 | 0.013377088 | down |
| ZBTB42 | -0.499113226 | 0.013424958 | 0.045860085 | down |
| RP11-18H7.1 | -0.499002712 | 0.189523449 | 0.31668757 | down |
| RP11-834C11.10 | -0.498987649 | 0.56705888 | | down |
| MUM1 | -0.498878064 | 0.006417697 | 0.026265264 | down |
| RP11-301G23.1 | -0.498752865 | 0.711249393 | 0.801709941 | down |
| MTCO2P33 | -0.498707764 | 0.864656751 | | down |
| CYP4F10P | -0.498707764 | 0.864656751 | | down |
| GAPDHP49 | -0.498706042 | 0.857013833 | | down |
| RP6-24A23.3 | -0.498701375 | 0.837764143 | | down |
| RP11-219J21.1 | -0.498701015 | 0.836357771 | | down |
| RP4-612C19.1 | -0.49870101 | 0.836337966 | | down |
| RP11-33N16.2 | -0.498700354 | 0.833809691 | | down |
| NA | -0.49869613 | 0.818298054 | | down |
| RP13-539J13.1 | -0.498695941 | 0.817630724 | | down |
| RN7SL279P | -0.498695636 | 0.816564872 | | down |
| RP11-417F21.2 | -0.498682654 | 0.775784699 | | down |
| RP11-643G16.4 | -0.498682445 | 0.744594967 | | down |
| TRAJ33 | -0.498675987 | 0.757602843 | | down |
| NA | -0.498675758 | 0.757004034 | | down |
| AC079612.1 | -0.498675578 | 0.756537092 | | down |
| NA | -0.498668248 | 0.738216685 | | down |
| RP5-1115A15.2 | -0.498666514 | 0.734088435 | | down |
| RP4-669P10.16 | -0.498666008 | 0.732899369 | | down |
| SHFM1P1 | -0.498655899 | 0.7102743 | | down |
| RP11-304L19.2 | -0.498648503 | 0.694950175 | | down |
| NA | -0.498648306 | 0.694554223 | | down |
| AC005488.11 | -0.498647189 | 0.692323124 | | down |
| GPR22 | -0.498625276 | 0.652010089 | | down |
| AC007365.4 | -0.498622302 | 0.6469803 | | down |

| | | | | |
|---|---|---|---|---|
| RPSAP12 | -0.498495807 | 0.120714014 | 0.23107562 | down |
| AC104653.1 | -0.498441681 | 0.265094958 | 0.40073021 | down |
| OTP | -0.498413406 | 0.769309636 | | down |
| CTC-429P9.5 | -0.498363409 | 0.235359998 | 0.368155141 | down |
| LINC01443 | -0.498351594 | 0.280823794 | 0.41728424 | down |
| WASL | -0.498345675 | 0.04496297 | 0.113161195 | down |
| NA | -0.498306553 | 0.35861336 | 0.496918171 | down |
| RP11-715J22.3 | -0.49828161 | 0.27535233 | 0.411642819 | down |
| LRRC37A16P | -0.498246514 | 0.076330182 | 0.166431815 | down |
| GGNBP1 | -0.498150497 | 0.385575241 | 0.523367784 | down |
| RPS19 | -0.498148707 | 0.030282077 | 0.084373061 | down |
| RP11-20I23.8 | -0.498065569 | 0.486621441 | 0.61534806 | down |
| RPL29P19 | -0.498025069 | 0.195490529 | 0.324009348 | down |
| CIRBP-AS1 | -0.49773515 | 0.092218731 | 0.190761468 | down |
| CHRNA10 | -0.497701282 | 0.070120316 | 0.156550105 | down |
| CTD-2555A7.1 | -0.497647406 | 0.464968912 | 0.596503949 | down |
| PHKB | -0.497634154 | 8.59E-07 | 1.78E-05 | down |
| RP11-140H17.2 | -0.497494067 | 0.549772686 | | down |
| NA | -0.497370991 | 0.797859896 | | down |
| BMS1P10 | -0.49723915 | 0.19503715 | 0.32344306 | down |
| CTA-27603.4 | -0.497091644 | 0.313435845 | 0.452081381 | down |
| RP11-98J23.2 | -0.49685113 | 0.392516952 | 0.530012553 | down |
| PYDC1 | -0.496719386 | 0.497077817 | 0.624548592 | down |
| RP11-353N4.6 | -0.496649572 | 0.069382286 | 0.155317891 | down |
| SCRN2 | -0.49659472 | 0.007372569 | 0.02910002 | down |
| RP3-475N16.1 | -0.496547652 | 0.09723747 | 0.198114917 | down |
| HDAC10 | -0.496376222 | 0.192446884 | 0.320360936 | down |
| TACC2 | -0.496289952 | 0.02539551 | 0.074147978 | down |
| RP11-318E3.9 | -0.496258418 | 0.31666444 | 0.455296058 | down |
| LINC00371 | -0.496211431 | 0.679292166 | | down |
| PET117 | -0.496143747 | 0.292896945 | 0.430223713 | down |
| RP11-277B15.3 | -0.49606447 | 0.266393387 | 0.402086055 | down |
| MKRN1 | -0.496045877 | 7.55E-08 | 2.26E-06 | down |
| CTA-268H5.12 | -0.495992609 | 0.542974611 | | down |
| TERT | -0.495719675 | 0.257053772 | 0.392086283 | down |
| AC073130.3 | -0.495627039 | 0.494400592 | | down |
| TNXA | -0.495619915 | 0.261044642 | 0.396242369 | down |
| CCT6P2 | -0.495592607 | 0.678341195 | | down |
| POMT1 | -0.495578298 | 0.009910236 | 0.036385573 | down |
| TRBJ2-1 | -0.495573839 | 0.354303037 | 0.49294621 | down |
| PELP1 | -0.495451289 | 0.009703223 | 0.03580052 | down |
| HOXB6 | -0.495321998 | 0.093933401 | 0.193349897 | down |
| SLC25A5P5 | -0.495269582 | 0.439406159 | 0.57341463 | down |
| MIEN1 | -0.495234547 | 0.020278174 | 0.062756228 | down |
| CCDC125 | -0.495220822 | 0.000308674 | 0.002386468 | down |
| RP11-514D23.1 | -0.495182248 | 0.558056063 | 0.677642727 | down |
| TRAV36DV7 | -0.495120946 | 0.289589417 | 0.426833948 | down |
| NA | -0.495119518 | 0.725618337 | | down |
| LINC01192 | -0.495097619 | 0.76140895 | 0.839009958 | down |
| CTD-2666L21.2 | -0.494902075 | 0.478640102 | 0.608302026 | down |
| UGP2 | -0.494872838 | 0.002105707 | 0.010925284 | down |

| | | | | |
|---|---|---|---|---|
| RPS9 | -0.494769828 | 0.011526926 | 0.040925899 | down |
| RP11-400L8.2 | -0.49463723 | 0.373970358 | 0.512355419 | down |
| RGL3 | -0.494631504 | 0.067509524 | 0.152214344 | down |
| SLC2A11 | -0.494558589 | 0.027861626 | 0.079532037 | down |
| TMEM143 | -0.494446222 | 0.003487326 | 0.016332536 | down |
| MYBL1 | -0.494427528 | 0.23939967 | 0.372978827 | down |
| RNU6-5P | -0.494389004 | 0.270096397 | 0.406083978 | down |
| AFDN | -0.494382443 | 0.028452992 | 0.080820809 | down |
| RP11-522N14.2 | -0.494217148 | 0.695519919 | | down |
| OLFM3 | -0.494127106 | 0.411144659 | 0.547872058 | down |
| PPP1R26 | -0.494055994 | 0.001333984 | 0.00760642 | down |
| RP11-362F19.3 | -0.493980668 | 0.57267564 | | down |
| RP5-857K21.4 | -0.493959401 | 0.354751274 | 0.493393714 | down |
| ZNF19 | -0.493796221 | 0.014436707 | 0.048495795 | down |
| SEPW1 | -0.493684401 | 0.009241648 | 0.034517832 | down |
| CTD-2184D3.7 | -0.493631334 | 0.655928292 | | down |
| EPS8L1 | -0.493458504 | 0.125829509 | 0.237747422 | down |
| RNF175 | -0.493382217 | 0.219745458 | 0.351246677 | down |
| FAM149A | -0.493359123 | 0.029318574 | 0.08249254 | down |
| SNHG14 | -0.493335083 | 0.074558813 | 0.163638321 | down |
| ITPRIPL1 | -0.493173635 | 0.090179282 | 0.187744879 | down |
| RP11-290H9.5 | -0.493053399 | 0.649215004 | | down |
| TCP11L1 | -0.492995136 | 0.004278775 | 0.01920686 | down |
| APOM | -0.492987734 | 0.074504598 | 0.163543227 | down |
| NPY1R | -0.492947639 | 0.159501881 | 0.280665017 | down |
| SNORD3B-1 | -0.492934798 | 0.354422998 | 0.493021787 | down |
| LINC00567 | -0.492908165 | 0.648434636 | | down |
| AC093063.2 | -0.492884006 | 0.556594714 | 0.676250873 | down |
| FBXW4 | -0.492867262 | 1.94E-10 | 1.21E-08 | down |
| MIR25 | -0.492733088 | 0.24362626 | 0.377664071 | down |
| AC083884.8 | -0.492671837 | 0.517717864 | 0.642625965 | down |
| CTD-2256P15.2 | -0.492609929 | 0.068145778 | 0.153337538 | down |
| CTH | -0.49248831 | 0.038537821 | 0.101049946 | down |
| DSG3 | -0.492414023 | 0.548618468 | 0.669261119 | down |
| MOB3B | -0.492401356 | 0.03680684 | 0.097601474 | down |
| ZNF563 | -0.492363252 | 0.044498583 | 0.112307533 | down |
| RP1-309F20.3 | -0.492232029 | 0.213362025 | 0.344233587 | down |
| PPP2R2B | -0.492217015 | 0.095911559 | 0.196198466 | down |
| ZNF219 | -0.492146892 | 0.008884753 | 0.033504897 | down |
| SPAG5-AS1 | -0.491894074 | 0.331140019 | 0.469487501 | down |
| NA | -0.491860228 | 0.866497811 | | down |
| RP4-620F22.3 | -0.491860228 | 0.866497811 | | down |
| HIST2H3DP1 | -0.491860228 | 0.866497811 | | down |
| RP1-127D3.4 | -0.491860228 | 0.866497811 | | down |
| AC016907.2 | -0.491860228 | 0.866497811 | | down |
| MIR2355 | -0.491860228 | 0.866497811 | | down |
| AC080002.1 | -0.491860228 | 0.866497811 | | down |
| Y_RNA | -0.491860228 | 0.866497811 | | down |
| NA | -0.491860228 | 0.866497811 | | down |
| RP11-389G6.5 | -0.491860228 | 0.866497811 | | down |
| RTP2 | -0.491860228 | 0.866497811 | | down |

| | | | | |
|---|---|---|---|---|
| RP11-12K22.1 | -0.491860228 | 0.866497811 | | down |
| RNU6-1336P | -0.491860228 | 0.866497811 | | down |
| CTD-2351A8.1 | -0.491860228 | 0.866497811 | | down |
| AC005592.3 | -0.491860228 | 0.866497811 | | down |
| TRBV8-2 | -0.491860228 | 0.866497811 | | down |
| RN7SL457P | -0.491860228 | 0.866497811 | | down |
| RP11-16E18.1 | -0.491860228 | 0.866497811 | | down |
| GOLGA2P1 | -0.491860228 | 0.866497811 | | down |
| RP11-255A11.4 | -0.491860228 | 0.866497811 | | down |
| HSPE1P22 | -0.491860228 | 0.866497811 | | down |
| MIR32 | -0.491860228 | 0.866497811 | | down |
| OR1L4 | -0.491860228 | 0.866497811 | | down |
| RP11-487I5.4 | -0.491860228 | 0.866497811 | | down |
| uc_338 | -0.491860228 | 0.866497811 | | down |
| RP1-68D18.3 | -0.491860228 | 0.866497811 | | down |
| RNU6-1156P | -0.491860228 | 0.866497811 | | down |
| RP11-434C1.2 | -0.491860228 | 0.866497811 | | down |
| TUBB8P4 | -0.491860228 | 0.866497811 | | down |
| RP11-616L12.1 | -0.491860228 | 0.866497811 | | down |
| NA | -0.491860228 | 0.866497811 | | down |
| RPP40P2 | -0.491860228 | 0.866497811 | | down |
| NFYAP1 | -0.491860228 | 0.866497811 | | down |
| EIF4A1P12 | -0.491860228 | 0.866497811 | | down |
| RP11-966I7.3 | -0.491860228 | 0.866497811 | | down |
| IGHVIII-11-1 | -0.491860228 | 0.866497811 | | down |
| RP11-578F21.6 | -0.491860228 | 0.866497811 | | down |
| RNU6-953P | -0.491860228 | 0.866497811 | | down |
| NA | -0.491860228 | 0.866497811 | | down |
| snoU13 | -0.491860228 | 0.866497811 | | down |
| RNU6-686P | -0.491860228 | 0.866497811 | | down |
| RNU6-1005P | -0.491860228 | 0.866497811 | | down |
| RP11-22D3.1 | -0.491860228 | 0.866497811 | | down |
| snoU13 | -0.491860228 | 0.866497811 | | down |
| AC003958.6 | -0.491860228 | 0.866497811 | | down |
| RPL5P33 | -0.491860228 | 0.866497811 | | down |
| RP11-51L5.1 | -0.491860228 | 0.866497811 | | down |
| PPIAP20 | -0.491860228 | 0.866497811 | | down |
| LL22NC03-75A1.9 | -0.491860228 | 0.866497811 | | down |
| NANOGP9 | -0.491860228 | 0.866497811 | | down |
| RP13-210D15.9 | -0.491860228 | 0.866497811 | | down |
| AC008834.1 | -0.491860137 | 0.864415349 | | down |
| BNIP3P24 | -0.491859521 | 0.847175997 | | down |
| RP11-1081K18.1 | -0.491859424 | 0.843843155 | | down |
| Metazoa_SRP | -0.49185932 | 0.839971504 | | down |
| RP11-432I5.6 | -0.49185904 | 0.827981385 | | down |
| DGAT2L6 | -0.491859011 | 0.826557436 | | down |
| LINC01324 | -0.491858629 | 0.804160664 | | down |
| LINC01296 | -0.491786823 | 0.245680739 | 0.379653951 | down |
| FMN1 | -0.491784884 | 0.04407221 | 0.111559986 | down |
| RPL34P18 | -0.491782209 | 0.089451032 | 0.186763679 | down |
| FAR2P2 | -0.49168555 | 0.113241561 | 0.220677438 | down |

| | | | | |
|---|---|---|---|---|
| CCL13 | -0.491537073 | 0.124984543 | 0.236642023 | down |
| ATP6V0B | -0.491279508 | 0.059339754 | 0.138577814 | down |
| PGRMC1 | -0.491183766 | 0.018760948 | 0.059158756 | down |
| DAGLA | -0.491087475 | 0.225752326 | 0.357769698 | down |
| RP11-452L6.1 | -0.491086568 | 0.064742271 | 0.147579512 | down |
| GPR153 | -0.490695429 | 0.016477904 | 0.053578156 | down |
| AC010468.1 | -0.490669982 | 0.149173613 | 0.267936393 | down |
| BMS1P16 | -0.490625368 | 0.821933294 | | down |
| CTD-2228K2.1 | -0.490584116 | 0.782269266 | | down |
| LGALS7 | -0.490499434 | 0.719289446 | | down |
| TTLL11-IT1 | -0.490467434 | 0.699407794 | | down |
| RP11-4K16.2 | -0.490401717 | 0.662999567 | | down |
| MIR6514 | -0.490363792 | 0.644116139 | | down |
| SMG5 | -0.490276729 | 0.000240561 | 0.001949665 | down |
| VWDE | -0.489953422 | 0.337045079 | 0.47531836 | down |
| ALDH5A1 | -0.489885968 | 0.005758979 | 0.024158841 | down |
| CYB561D2 | -0.489867752 | 0.045652309 | 0.114373352 | down |
| KNDC1 | -0.489858748 | 0.186188825 | 0.312471068 | down |
| CCDC129 | -0.489844419 | 0.388584598 | 0.52619262 | down |
| ZC3H12B | -0.489727838 | 0.044483489 | 0.11227942 | down |
| RPL7P1 | -0.489692857 | 0.008380382 | 0.032024953 | down |
| CTC-529I10.2 | -0.489563937 | 0.151073731 | 0.270194745 | down |
| RP11-471J7.1 | -0.489475151 | 0.720118365 | | down |
| GJC2 | -0.489344282 | 0.041931058 | 0.107682427 | down |
| ZNF197-AS1 | -0.4892994 | 0.454845049 | 0.587302933 | down |
| FGD5P1 | -0.489104029 | 0.621790148 | | down |
| RHOBTB2 | -0.489014684 | 0.038405139 | 0.100816426 | down |
| NAA60 | -0.488859423 | 0.003918062 | 0.017942069 | down |
| AC092159.2 | -0.488859184 | 0.491103065 | 0.619389988 | down |
| RP11-139G7.1 | -0.488857082 | 0.856060935 | | down |
| SMIM7 | -0.488755082 | 0.012234766 | 0.042796753 | down |
| RP11-412D9.4 | -0.488535104 | 0.144812704 | 0.262362231 | down |
| CDK3 | -0.488389961 | 0.438608038 | 0.572850575 | down |
| ULK3 | -0.488263661 | 0.009796455 | 0.036047116 | down |
| AMPD1 | -0.488000537 | 0.173904627 | 0.298403401 | down |
| AP000351.13 | -0.487971985 | 0.70056884 | 0.793870563 | down |
| PRDM16 | -0.487756683 | 0.176516532 | 0.301373114 | down |
| MSH4 | -0.487741943 | 0.418510768 | 0.554568761 | down |
| RP11-799B12.2 | -0.487719105 | 0.646225181 | | down |
| RP11-234A1.1 | -0.487472251 | 0.011002373 | 0.039469289 | down |
| RNU6-238P | -0.487241325 | 0.664135101 | 0.765284969 | down |
| WDR25 | -0.487177527 | 0.041763452 | 0.107380501 | down |
| NNT-AS1 | -0.487087801 | 0.018198615 | 0.057851993 | down |
| RP11-83M16.3 | -0.487082276 | 0.667302034 | | down |
| NA | -0.487071762 | 0.294128101 | 0.431525768 | down |
| SIGLEC16 | -0.487053091 | 0.078748882 | 0.170222151 | down |
| CENPM | -0.487014291 | 0.092860592 | 0.19162614 | down |
| NA | -0.486832374 | 0.075084941 | 0.164456657 | down |
| SLC38A7 | -0.486711645 | 0.008657439 | 0.032804148 | down |
| RP11-178L8.3 | -0.486609976 | 0.506774475 | 0.632972991 | down |
| NA | -0.486556318 | 0.558769774 | | down |

| | | | | |
|---|---|---|---|---|
| CUTA | -0.486509733 | 0.055907198 | 0.132728239 | down |
| CGNL1 | -0.486479071 | 0.051504059 | 0.125001045 | down |
| INSL3 | -0.486447787 | 0.242143608 | 0.376180907 | down |
| CTA-292E10.6 | -0.486411322 | 0.080613301 | 0.173156933 | down |
| RP1-95L4.4 | -0.486112999 | 0.101496009 | 0.204201841 | down |
| CD163L1 | -0.486108088 | 0.134551136 | 0.24908832 | down |
| RP11-897M7.4 | -0.486086518 | 0.82423041 | | down |
| RP11-12G12.7 | -0.486084924 | 0.043111739 | 0.109897258 | down |
| CTA-150C2.13 | -0.486072361 | 0.639067418 | | down |
| SYT6 | -0.486017187 | 0.553240801 | | down |
| APBA1 | -0.485979997 | 0.071864908 | 0.159364292 | down |
| OR7E90P | -0.485625534 | 0.857430146 | | down |
| RP11-190A12.9 | -0.485588865 | 0.49475354 | 0.622669879 | down |
| THEM6 | -0.485527036 | 0.001757219 | 0.009471393 | down |
| RALGAPA1P1 | -0.485204409 | 0.401722365 | 0.538527254 | down |
| CTC-523E23.4 | -0.485092557 | 0.399257569 | 0.5361984 | down |
| C12orf57 | -0.485066913 | 0.1342056 | 0.248571175 | down |
| RP11-1E1.2 | -0.485062633 | 0.764326989 | | down |
| CDC42EP4 | -0.485024634 | 0.002861083 | 0.013974699 | down |
| RP11-498D10.8 | -0.4849745 | 0.745476255 | | down |
| NA | -0.484958755 | 0.368074741 | 0.506640285 | down |
| PPM1L | -0.484840091 | 0.036780459 | 0.097568112 | down |
| RP11-503C24.2 | -0.484793207 | 0.785383756 | | down |
| NKAPL | -0.484630515 | 0.300168493 | 0.437822225 | down |
| RP4-752I6.1 | -0.484598142 | 0.704641071 | | down |
| AC096664.1 | -0.484506185 | 0.6645634 | | down |
| NA | -0.484423378 | 0.127399775 | 0.23979444 | down |
| Y_RNA | -0.484036332 | 0.766773325 | | down |
| RP11-264F23.1 | -0.48397569 | 0.322201625 | 0.460985076 | down |
| PKP2 | -0.483855545 | 0.067432232 | 0.152074311 | down |
| NA | -0.483746801 | 0.689751112 | | down |
| GNAO1 | -0.483679503 | 0.07813767 | 0.169279503 | down |
| MYL2 | -0.483514009 | 0.308574101 | 0.447022532 | down |
| CRADD | -0.483453149 | 0.03318793 | 0.090308684 | down |
| RP11-61J19.5 | -0.483422622 | 0.028020743 | 0.079879988 | down |
| RP11-314A20.1 | -0.483359119 | 0.209401836 | 0.339627984 | down |
| MIR4734 | -0.483339146 | 0.617137873 | | down |
| AC008850.3 | -0.483288561 | 0.67495662 | | down |
| RPL13A | -0.483280349 | 0.011417809 | 0.040610473 | down |
| TAF7L | -0.483182662 | 0.498930211 | 0.626116673 | down |
| ZFYVE16 | -0.483167953 | 0.051981052 | 0.125661307 | down |
| ZNF213-AS1 | -0.483162207 | 0.002633826 | 0.013096767 | down |
| PRPS1P2 | -0.483017398 | 0.431688279 | 0.566780696 | down |
| WNT3A | -0.483017189 | 0.610581078 | | down |
| IDH3B | -0.482990712 | 0.001348721 | 0.007674426 | down |
| GRPEL2-AS1 | -0.482964424 | 0.632130505 | | down |
| OPN3 | -0.482912504 | 0.29464544 | 0.432086486 | down |
| RP4-738P11.4 | -0.482812062 | 0.711260407 | | down |
| SLC43A2 | -0.4827522 | 0.022287945 | 0.067217327 | down |
| RP11-550I24.2 | -0.482741409 | 0.033389635 | 0.09071787 | down |
| IGKV1OR2-6 | -0.482451178 | 0.869028356 | | down |

| | | | | |
|---|---|---|---|---|
| RP11-47P18.2 | -0.482451178 | 0.869028356 | | down |
| FTLP10 | -0.482451178 | 0.869028356 | | down |
| SALL4P1 | -0.482451178 | 0.869028356 | | down |
| TRPC7-AS2 | -0.482451178 | 0.869028356 | | down |
| OR6B1 | -0.482451178 | 0.869028356 | | down |
| OR1J2 | -0.482451178 | 0.869028356 | | down |
| MIR920 | -0.482451178 | 0.869028356 | | down |
| RP11-429B14.1 | -0.482451178 | 0.869028356 | | down |
| AC139099.5 | -0.482451178 | 0.869028356 | | down |
| KRTAP10-3 | -0.482451178 | 0.869028356 | | down |
| TBX22 | -0.482451178 | 0.869028356 | | down |
| GPR50 | -0.482451178 | 0.869028356 | | down |
| SHROOM2P1 | -0.482451178 | 0.869028356 | | down |
| Y_RNA | -0.482451155 | 0.86848785 | | down |
| CTB-79E8.2 | -0.482451121 | 0.867683238 | | down |
| RP11-325L12.3 | -0.482450499 | 0.849620962 | | down |
| RPSAP50 | -0.482450463 | 0.848360633 | | down |
| CTD-2540B15.12 | -0.482450431 | 0.847199131 | | down |
| RP5-1003J2.4 | -0.482450403 | 0.846141465 | | down |
| NA | -0.482450005 | 0.828472541 | | down |
| SNORD91A | -0.482449981 | 0.827228352 | | down |
| RP11-1B20.1 | -0.482449968 | 0.82655496 | | down |
| RN7SL494P | -0.482449962 | 0.826205698 | | down |
| RN7SL329P | -0.482449961 | 0.826151215 | | down |
| RNU6-245P | -0.482449917 | 0.823706727 | | down |
| RNA5SP490 | -0.482449678 | 0.808334558 | | down |
| RP4-566L20.1 | -0.482449643 | 0.8057099 | | down |
| RNU6-46P | -0.48244941 | 0.785166906 | | down |
| EML2-AS1 | -0.482449401 | 0.784231195 | | down |
| RP5-1174J21.1 | -0.482449062 | 0.737100115 | | down |
| RPS6KL1 | -0.482440286 | 0.014307507 | 0.048189992 | down |
| RP11-372E1.1 | -0.482433107 | 0.148095135 | 0.266764809 | down |
| RP11-651L5.3 | -0.482419718 | 0.12754859 | 0.240027534 | down |
| RP1-56K13.5 | -0.482370518 | 0.606771075 | | down |
| CTD-2248H3.1 | -0.482277047 | 0.359226786 | 0.497515768 | down |
| INSM1 | -0.482276212 | 0.213781149 | 0.344646965 | down |
| HBEGF | -0.48223972 | 0.124133639 | 0.235569736 | down |
| CTD-2017C7.1 | -0.482056022 | 0.283499463 | 0.420138009 | down |
| RP11-543P15.1 | -0.481699889 | 0.008341541 | 0.031933393 | down |
| CDCP1 | -0.481564117 | 0.116653863 | 0.225458261 | down |
| RP1-121G13.3 | -0.481555101 | 0.705313061 | | down |
| RP11-305F18.1 | -0.481516236 | 0.424552581 | 0.560178206 | down |
| ODCP | -0.481365612 | 0.624379412 | 0.733845193 | down |
| BNIP3P17 | -0.481212794 | 0.609833575 | 0.721701655 | down |
| RP11-92K2.2 | -0.48117654 | 0.165588624 | 0.288413601 | down |
| TPI1P1 | -0.480890777 | 0.073605337 | 0.162185459 | down |
| BSPRY | -0.48088054 | 0.085192579 | 0.180337197 | down |
| LINC01138 | -0.480714396 | 0.063106563 | 0.145051915 | down |
| EGLN3 | -0.480710402 | 0.036589868 | 0.097204339 | down |
| LINC00242 | -0.480587175 | 0.155333292 | 0.275426902 | down |
| TFP1 | -0.480397708 | 0.575096676 | 0.692124841 | down |

| | | | | |
|---|---|---|---|---|
| AC019097.7 | -0.480224813 | 0.419419131 | 0.555160295 | down |
| FAM27E4 | -0.48005191 | 0.256865531 | 0.391903906 | down |
| RNA5SP284 | -0.480036632 | 0.677898685 | | down |
| RFK | -0.480022834 | 0.021795241 | 0.066156419 | down |
| RP11-752L20.5 | -0.479942856 | 0.735188452 | | down |
| RPS24P17 | -0.479919092 | 0.437449387 | | down |
| RP11-467J12.4 | -0.4798313 | 0.121650734 | 0.232365354 | down |
| RPS4XP17 | -0.47976283 | 0.393954635 | 0.531332962 | down |
| UBXN6 | -0.479746598 | 0.000356693 | 0.002677784 | down |
| SAMD12 | -0.479739847 | 0.012241807 | 0.042809385 | down |
| RP11-544L8__B.4 | -0.479722157 | 0.617793849 | | down |
| HOXB-AS1 | -0.479579141 | 0.070868379 | 0.157716265 | down |
| ZNF547 | -0.479414981 | 0.145931382 | 0.263848013 | down |
| RP4-778K6.3 | -0.479383675 | 0.681492375 | | down |
| RNU4-39P | -0.47938286 | 0.535943902 | | down |
| OSBPL1A | -0.479380166 | 0.063839699 | 0.146157353 | down |
| ASS1P9 | -0.479346417 | 0.468485953 | 0.599480045 | down |
| SCHIP1 | -0.479335442 | 0.445686445 | 0.578960721 | down |
| CEBPA | -0.47930727 | 0.087041808 | 0.183141962 | down |
| CECR7 | -0.479306488 | 0.183474317 | 0.309210881 | down |
| FUCA1 | -0.478946265 | 0.02431735 | 0.07180956 | down |
| RP11-474O21.5 | -0.478866846 | 0.30899715 | 0.447419843 | down |
| CNTNAP4 | -0.478802098 | 0.606740877 | | down |
| NA | -0.47870444 | 0.26633443 | 0.402017263 | down |
| CTD-2006C1.13 | -0.478633301 | 0.673545138 | | down |
| CCDC144A | -0.478632969 | 0.585479166 | 0.701109493 | down |
| EIF4EBP1 | -0.478353228 | 0.054717414 | 0.130657287 | down |
| RP11-958F21.1 | -0.478338292 | 0.555276626 | 0.67511355 | down |
| ST6GALNAC1 | -0.478248532 | 0.130014525 | 0.243237834 | down |
| RNA5SP201 | -0.478158176 | 0.656292448 | | down |
| RP11-257O5.4 | -0.478071654 | 0.483770853 | 0.61280089 | down |
| RP5-907C10.3 | -0.477755578 | 0.650491188 | | down |
| SDHDP2 | -0.477719699 | 0.671163638 | | down |
| EIF4EBP2 | -0.477589366 | 0.000997593 | 0.006049998 | down |
| NA | -0.477583444 | 0.769527364 | | down |
| RP5-1157M23.2 | -0.477437838 | 0.308869599 | 0.447342761 | down |
| HTRA4 | -0.477408416 | 0.304250512 | 0.442315996 | down |
| ROR1 | -0.477309617 | 0.216597237 | 0.347861624 | down |
| RP11-145M9.6 | -0.477274909 | 0.236040336 | 0.369011655 | down |
| BDH2 | -0.47722547 | 0.040818828 | 0.105438987 | down |
| KLHDC9 | -0.477067077 | 0.035515467 | 0.095007274 | down |
| RP11-397P13.7 | -0.477006617 | 0.613574789 | | down |
| MED28P3 | -0.476809669 | 0.612387881 | | down |
| GPC2 | -0.476741379 | 0.168048562 | 0.291403151 | down |
| TH2LCRR | -0.476651108 | 0.464034067 | 0.595560182 | down |
| HNRNPA3P1 | -0.476559466 | 0.435609588 | 0.570334007 | down |
| CYP20A1 | -0.476544198 | 0.001949531 | 0.010255946 | down |
| RP11-817O13.8 | -0.476486202 | 0.177848462 | 0.302855478 | down |
| RNF150 | -0.476437532 | 0.057224748 | 0.134974068 | down |
| HIST1H4D | -0.476401581 | 0.40523861 | 0.54173662 | down |
| RIMKLBP2 | -0.476368493 | 0.495868697 | 0.623550943 | down |

| | | | | |
|---|---|---|---|---|
| CTD-2284J15.1 | -0.476309936 | 0.193661682 | 0.321776993 | down |
| KB-208E9.1 | -0.476302193 | 0.274521382 | 0.410883161 | down |
| RP11-371A22.1 | -0.476299433 | 0.004306246 | 0.019304217 | down |
| CTC-479C5.12 | -0.476263388 | 0.118305177 | 0.227770634 | down |
| NA | -0.476232248 | 0.346932783 | 0.485683264 | down |
| SHISA3 | -0.476169596 | 0.26767059 | 0.403426066 | down |
| LINC01560 | -0.476001654 | 0.015908715 | 0.052211688 | down |
| TULP4 | -0.475896828 | 0.001734413 | 0.009378819 | down |
| RP11-39C10.1 | -0.475849509 | 0.379674549 | 0.517650307 | down |
| RP11-1079H9.1 | -0.47564402 | 0.539125896 | 0.661068419 | down |
| CTD-2313J17.5 | -0.47537779 | 0.207421457 | 0.337600642 | down |
| RP4-568C11.4 | -0.475367528 | 0.13589212 | 0.250934399 | down |
| B4GALT7 | -0.475236591 | 0.029801081 | 0.083365333 | down |
| DHRS4L1 | -0.475221202 | 0.439458276 | 0.57341463 | down |
| AGO4 | -0.475204802 | 0.015139036 | 0.050290192 | down |
| EXOC6B | -0.475102809 | 0.054977722 | 0.131080921 | down |
| ALS2CL | -0.475094809 | 0.011340668 | 0.040417441 | down |
| RIMBP2 | -0.475013512 | 0.256415562 | 0.391490454 | down |
| NALT1 | -0.474908378 | 0.186315491 | 0.312614196 | down |
| RP11-2C24.5 | -0.474873072 | 0.397201161 | 0.5342008 | down |
| SFI1 | -0.474703236 | 0.008934683 | 0.033642512 | down |
| RP13-270P17.1 | -0.474603794 | 0.457911234 | 0.589943615 | down |
| TRIM51FP | -0.474535805 | 0.860996991 | | down |
| C5orf58 | -0.47451331 | 0.365802572 | 0.504237973 | down |
| TOLLIP-AS1 | -0.47441832 | 0.042814167 | 0.109324226 | down |
| RP11-713P17.3 | -0.474276792 | 0.156563352 | 0.277084669 | down |
| ACP5 | -0.474237237 | 0.090306626 | 0.187957899 | down |
| RP11-365O16.6 | -0.474164776 | 0.297996733 | 0.435457872 | down |
| AL358852.1 | -0.473966823 | 0.225625771 | 0.357684288 | down |
| MAPKBP1 | -0.473943512 | 0.003098852 | 0.014867661 | down |
| RP5-902P8.10 | -0.47381361 | 0.152747147 | 0.272258224 | down |
| PGM1 | -0.473703303 | 0.004295425 | 0.019264331 | down |
| RP11-644F5.10 | -0.473685337 | 0.278641566 | 0.414944595 | down |
| C3orf33 | -0.47357858 | 0.228108191 | 0.360357686 | down |
| NBAS | -0.473557455 | 0.005386484 | 0.022974378 | down |
| RP5-1063M23.2 | -0.473554504 | 0.705206894 | | down |
| TMEM135 | -0.473518628 | 0.000346086 | 0.002617118 | down |
| PET100 | -0.473417717 | 0.089384245 | 0.186671782 | down |
| KCND2 | -0.473413701 | 0.423130599 | 0.558743659 | down |
| FKBP8 | -0.473152707 | 0.021145798 | 0.064708124 | down |
| GSTP1 | -0.473134679 | 0.025068193 | 0.073470762 | down |
| RNU6-342P | -0.473042128 | 0.871560244 | | down |
| NA | -0.473042128 | 0.871560244 | | down |
| CTD-2057J6.1 | -0.473042128 | 0.871560244 | | down |
| OR2AE1 | -0.473042128 | 0.871560244 | | down |
| RP11-317J10.4 | -0.473042128 | 0.871560244 | | down |
| RP11-369K17.2 | -0.473042128 | 0.871560244 | | down |
| RP11-45F15.1 | -0.473042128 | 0.871560244 | | down |
| RP11-548M13.1 | -0.473042128 | 0.871560244 | | down |
| RP11-357H3.1 | -0.473042128 | 0.871560244 | | down |
| AC005307.4 | -0.473042128 | 0.871560244 | | down |

| | | | | |
|---|---|---|---|---|
| Y_RNA | -0.473042128 | 0.871560244 | | down |
| RP1-31B8.1 | -0.473042128 | 0.871560244 | | down |
| HIGD1AP18 | -0.473042079 | 0.870338269 | | down |
| NUDT19P3 | -0.473042046 | 0.86952239 | | down |
| RNU6-1245P | -0.473042005 | 0.868464412 | | down |
| CTD-2311M21.5 | -0.473041922 | 0.866244982 | | down |
| AC091493.1 | -0.473041913 | 0.86599657 | | down |
| RP11-443B7.2 | -0.473041459 | 0.851383418 | | down |
| RNU7-43P | -0.473041409 | 0.849451446 | | down |
| FRG1FP | -0.473041379 | 0.848283426 | | down |
| GAPDHP59 | -0.473041356 | 0.84734142 | | down |
| RP11-20J15.2 | -0.473040991 | 0.8298327 | | down |
| IQCB2P | -0.473040986 | 0.829553316 | | down |
| TRBV17 | -0.47304098 | 0.829190991 | | down |
| RP11-467L19.14 | -0.473040971 | 0.82866759 | | down |
| RP11-424M21.1 | -0.473040686 | 0.80947212 | | down |
| RP11-831F12.3 | -0.473040684 | 0.809263956 | | down |
| RP11-116D17.4 | -0.473040662 | 0.807480242 | | down |
| AC004510.3 | -0.473040662 | 0.807492666 | | down |
| Y_RNA | -0.473040639 | 0.805590594 | | down |
| CTD-2086O20.1 | -0.473040469 | 0.789413792 | | down |
| RP5-1052M9.1 | -0.47304041 | 0.782838039 | | down |
| RP11-182N22.8 | -0.473040284 | 0.766381362 | | down |
| KRT8P41 | -0.473040283 | 0.766166412 | | down |
| RP13-487P22.1 | -0.473040167 | 0.747163217 | | down |
| MRPL3P1 | -0.473040164 | 0.746509499 | | down |
| RP11-666O2.4 | -0.473039981 | 0.703567505 | | down |
| ZNF454 | -0.472896572 | 0.276767627 | 0.412993275 | down |
| MCRIP1 | -0.47284345 | 0.070639606 | 0.157420551 | down |
| WDR62 | -0.472812591 | 0.122831949 | 0.233774425 | down |
| RP11-209D14.2 | -0.47276914 | 0.546605983 | 0.667703757 | down |
| RP11-365N19.2 | -0.472727909 | 0.449214962 | 0.582137098 | down |
| URGCP | -0.47267964 | 3.30E-05 | 0.000377971 | down |
| EIF4EBP3 | -0.472569134 | 0.388434131 | 0.526036287 | down |
| SEPSECS | -0.472516009 | 0.000319653 | 0.002453044 | down |
| STOML1 | -0.472412968 | 0.00299415 | 0.014474053 | down |
| RP11-348F1.2 | -0.472392093 | 0.293436754 | 0.430763893 | down |
| AFF2 | -0.472366255 | 0.414530053 | 0.5510438 | down |
| TSPEAR-AS2 | -0.472316987 | 0.372333512 | 0.510700696 | down |
| CUL9 | -0.472255778 | 0.003575788 | 0.01666224 | down |
| FOXL1 | -0.472211281 | 0.053423859 | 0.128251412 | down |
| DLEU2 | -0.472054159 | 0.064792733 | 0.147651158 | down |
| AL161668.5 | -0.471982789 | 0.622847698 | | down |
| RAC1 | -0.471946564 | 0.017190432 | 0.055296504 | down |
| SNORA53 | -0.471864314 | 0.564330518 | | down |
| RP11-576N17.5 | -0.471856024 | 0.669456832 | | down |
| RP11-632P5.1 | -0.471610011 | 0.402900464 | 0.539595308 | down |
| PLGRKT | -0.471588291 | 0.018542273 | 0.058702419 | down |
| ENOSF1 | -0.471440295 | 0.016911897 | 0.054599294 | down |
| ACTR3C | -0.471197351 | 0.063220082 | 0.145246169 | down |
| DNPH1 | -0.471178919 | 0.008017124 | 0.031019509 | down |

| | | | | |
|---|---|---|---|---|
| TMEM254 | -0.471131756 | 0.003831196 | 0.017616232 | down |
| FAM181B | -0.471027419 | 0.180451703 | 0.305780653 | down |
| FAHD1 | -0.471006475 | 0.006225052 | 0.02567246 | down |
| MTMR7 | -0.470948589 | 0.132493171 | 0.24652955 | down |
| SHMT1 | -0.470914 | 0.001187101 | 0.006956359 | down |
| CTB-164N12.1 | -0.470874605 | 0.829507308 | | down |
| MYO19 | -0.470869197 | 0.000350356 | 0.002641428 | down |
| TIMM50 | -0.47086588 | 0.005217584 | 0.022413081 | down |
| STOML2 | -0.470764759 | 0.00401934 | 0.018313172 | down |
| PPIP5K1 | -0.470750227 | 0.020088467 | 0.062297472 | down |
| RP11-434H6.6 | -0.470526698 | 0.157156223 | 0.277847251 | down |
| TAF6L | -0.470506854 | 0.016371935 | 0.053331717 | down |
| ADAM20P1 | -0.470478749 | 0.315120567 | 0.45379295 | down |
| AGPAT3 | -0.4703977 | 4.00E-05 | 0.000443928 | down |
| RP11-140K17.3 | -0.470396052 | 0.03019375 | 0.084187309 | down |
| Y_RNA | -0.470391962 | 0.764162803 | 0.841217472 | down |
| AF011889.2 | -0.470299285 | 0.372596996 | 0.510968785 | down |
| RP11-38G5.2 | -0.470135675 | 0.755405601 | | down |
| ALOX12 | -0.470072894 | 0.141199242 | 0.257852543 | down |
| RPL7L1P8 | -0.469787264 | 0.830082725 | | down |
| AAMDC | -0.469735914 | 0.008217854 | 0.031576571 | down |
| PFKFB1 | -0.469724796 | 0.243133012 | 0.377152428 | down |
| NA | -0.469698065 | 0.41622158 | 0.552456416 | down |
| RP11-649A18.12 | -0.469443392 | 0.212011994 | 0.342751202 | down |
| RP11-621L6.3 | -0.469397719 | 0.543519121 | 0.664962678 | down |
| SMARCD2 | -0.4693567 | 0.004492306 | 0.019975239 | down |
| RHOF | -0.469098268 | 0.05855961 | 0.137300211 | down |
| RP11-761N21.1 | -0.468828224 | 0.615557793 | | down |
| ALDH2 | -0.468644397 | 0.008294781 | 0.03179472 | down |
| LINC00994 | -0.468575396 | 0.750862048 | | down |
| ROBO2 | -0.46836707 | 0.173252462 | 0.297660947 | down |
| CNIH2 | -0.468195538 | 0.078292382 | 0.169505367 | down |
| RPS3AP47 | -0.467913442 | 0.081279598 | 0.174228964 | down |
| AP006222.2 | -0.46790047 | 0.318781179 | 0.457599905 | down |
| RNASE3 | -0.46788045 | 0.526328808 | 0.649885294 | down |
| FAM87A | -0.467439583 | 0.417492083 | 0.553731773 | down |
| AP5S1 | -0.467313237 | 0.001436937 | 0.008063378 | down |
| RP1-102E24.6 | -0.467299515 | 0.487106158 | 0.615805487 | down |
| RP11-414H17.5 | -0.46729068 | 0.569390809 | 0.687157159 | down |
| RP5-1007H16.1 | -0.467267314 | 0.449809514 | 0.582804771 | down |
| CINP | -0.46715473 | 0.012749128 | 0.044123351 | down |
| NET1 | -0.467145916 | 0.02732288 | 0.078357629 | down |
| ENO3 | -0.467021389 | 0.011851228 | 0.041795785 | down |
| RORA-AS1 | -0.466884785 | 0.274833209 | 0.411107241 | down |
| RANP4 | -0.466842957 | 0.458257818 | 0.590288882 | down |
| RP11-1072A3.4 | -0.466634609 | 0.335380589 | 0.473615719 | down |
| MRPL14 | -0.466626708 | 0.033819093 | 0.091702242 | down |
| RP5-967N21.11 | -0.466516842 | 0.181593206 | 0.307126001 | down |
| LINC01465 | -0.466288931 | 0.203931494 | 0.333401414 | down |
| TSPAN8 | -0.466271467 | 0.17449041 | 0.298933106 | down |
| RP11-613D13.5 | -0.466265917 | 0.494168288 | 0.622220026 | down |

| | | | | |
|---|---|---|---|---|
| RPS21 | -0.466259441 | 0.02171371 | 0.065962256 | down |
| KRT18P4 | -0.466220212 | 0.312471802 | 0.451167956 | down |
| AC024937.4 | -0.466088631 | 0.500710407 | 0.627825843 | down |
| IGKV6D-41 | -0.465944417 | 0.669134112 | | down |
| NA | -0.465927506 | 0.797815399 | | down |
| RP11-1017G21.5 | -0.465603063 | 0.035012463 | 0.094080532 | down |
| LSM4 | -0.465587111 | 0.000534369 | 0.003672139 | down |
| TIRAP | -0.465557248 | 0.022082135 | 0.066784342 | down |
| FAM216B | -0.465496699 | 0.264517711 | 0.399958234 | down |
| RP11-493E12.1 | -0.465486601 | 0.574907804 | | down |
| MAD2L1BP | -0.465302842 | 0.00147561 | 0.008225088 | down |
| CCDC12 | -0.465068407 | 0.01509053 | 0.050174844 | down |
| CTD-2287O16.1 | -0.464836043 | 0.065843499 | 0.14939975 | down |
| FAM86GP | -0.464813045 | 0.296593738 | 0.433977611 | down |
| FAM98C | -0.46462348 | 0.000658612 | 0.004345046 | down |
| RP11-83N9.5 | -0.464247529 | 0.107226354 | 0.212477148 | down |
| NA | -0.464080383 | 0.279234959 | 0.415519322 | down |
| DEPDC5 | -0.463775001 | 0.005580033 | 0.02359557 | down |
| EIF5B | -0.463705263 | 0.040979624 | 0.105763408 | down |
| RN7SL313P | -0.463633078 | 0.874093448 | | down |
| MIR4736 | -0.463633078 | 0.874093448 | | down |
| RP11-677O4.2 | -0.463633078 | 0.874093448 | | down |
| BNIP3P23 | -0.463633078 | 0.874093448 | | down |
| RP3-445N2.1 | -0.463632937 | 0.870325703 | | down |
| FTLP1 | -0.463632859 | 0.868094965 | | down |
| CTC-321K16.4 | -0.463632827 | 0.867167447 | | down |
| KB-176G8.1 | -0.463632391 | 0.85182671 | | down |
| WASF1P1 | -0.463632373 | 0.851052497 | | down |
| MIR6719 | -0.46363202 | 0.833493923 | | down |
| RP11-330L19.2 | -0.463632007 | 0.832737793 | | down |
| RP11-5N19.3 | -0.463631991 | 0.831783464 | | down |
| C1QTNF9B | -0.46363174 | 0.81405635 | | down |
| RN7SL751P | -0.463631738 | 0.813874381 | | down |
| CTD-2007H13.1 | -0.463631527 | 0.793593822 | | down |
| RP11-1038A11.3 | -0.463631522 | 0.793108938 | | down |
| RN7SL220P | -0.463631517 | 0.792518482 | | down |
| CH17-174L20.1 | -0.463631497 | 0.790249306 | | down |
| RP11-983G14.3 | -0.463631334 | 0.767836933 | | down |
| FOXN3-AS1 | -0.463576105 | 0.056547472 | 0.133827516 | down |
| UBBP4 | -0.463567602 | 0.016066452 | 0.052587482 | down |
| C11orf94 | -0.463500499 | 0.40383927 | 0.540467149 | down |
| RP11-166B2.8 | -0.463363149 | 0.40176882 | 0.538527254 | down |
| MPZL3 | -0.463294134 | 0.00612577 | 0.025360705 | down |
| LOXNC01-116E7.[illegible] | -0.462990459 | 0.306878504 | 0.445188358 | down |
| NA | -0.46259466 | 0.531206529 | | down |
| NA | -0.462563115 | 0.751949827 | | down |
| PLA2G6 | -0.462443612 | 0.008229127 | 0.031595643 | down |
| RP11-960L18.1 | -0.462442636 | 0.254293605 | 0.389319052 | down |
| DGCR2 | -0.462400217 | 0.012927425 | 0.044550535 | down |
| FOXO6 | -0.462077849 | 0.194907313 | 0.323328104 | down |
| UQCRFS1 | -0.462062251 | 0.01512461 | 0.050265906 | down |

| | | | | |
|---|---|---|---|---|
| SYTL2 | -0.462035509 | 0.059263828 | 0.138472524 | down |
| TDRD3 | -0.461870915 | 0.003318564 | 0.015674098 | down |
| CCDC88B | -0.46175943 | 0.127749555 | 0.240257129 | down |
| RP5-968P14.2 | -0.461734573 | 0.181263335 | 0.306724656 | down |
| GRIP2 | -0.461572161 | 0.250006769 | 0.384610716 | down |
| RP11-534L6.2 | -0.461559268 | 0.638142682 | | down |
| LPP | -0.461509005 | 0.041286858 | 0.106382707 | down |
| APEH | -0.461348778 | 0.003044923 | 0.014667576 | down |
| BTBD2 | -0.461016411 | 0.000501132 | 0.003480352 | down |
| LINC01546 | -0.460674862 | 0.502815875 | 0.629694801 | down |
| MIEF2 | -0.460664433 | 0.004813047 | 0.021084384 | down |
| RP11-20B7.1 | -0.460441957 | 0.626595155 | 0.73556929 | down |
| NPM1P25 | -0.460320991 | 0.11108677 | 0.217913482 | down |
| RP11-134K13.4 | -0.460106105 | 0.365575512 | 0.504077776 | down |
| HNRNPA1P27 | -0.460094154 | 0.441750244 | 0.575389938 | down |
| TMEM94 | -0.460020445 | 0.000629091 | 0.004190777 | down |
| LA16c-313F4.1 | -0.459900659 | 0.25092064 | 0.385609193 | down |
| TTC30B | -0.459691286 | 0.004002415 | 0.018249894 | down |
| CLEC16A | -0.45968359 | 0.000325609 | 0.002491112 | down |
| KSR1 | -0.459498269 | 0.045931432 | 0.114870341 | down |
| PIP | -0.459410752 | 0.55150479 | 0.67155933 | down |
| RP11-319G6.1 | -0.459198726 | 0.031219466 | 0.086344255 | down |
| AP2A2 | -0.459143867 | 0.000864975 | 0.005393798 | down |
| RP11-483E17.1 | -0.45908936 | 0.334749317 | 0.472968796 | down |
| PRRC2C | -0.459063646 | 0.007616511 | 0.029861733 | down |
| ATPIF1 | -0.45892598 | 0.012112389 | 0.042469842 | down |
| RNU6-882P | -0.458852704 | 0.597188805 | | down |
| RPL12P47 | -0.458735642 | 0.556921528 | | down |
| CTD-2179L22.1 | -0.458639197 | 0.749284296 | | down |
| CCDC22 | -0.458624315 | 0.007050472 | 0.028138203 | down |
| ZNF821 | -0.457686716 | 0.166247932 | 0.289340854 | down |
| RP3-412A9.16 | -0.457594719 | 0.207610679 | 0.337802574 | down |
| UCK2 | -0.457505483 | 0.001549256 | 0.008554652 | down |
| SERINC5 | -0.457415655 | 0.034793693 | 0.093651828 | down |
| LRRC37A12P | -0.457362293 | 0.875782454 | | down |
| HSPD1P14 | -0.457362293 | 0.875782454 | | down |
| CYP4A43P | -0.457362293 | 0.875782454 | | down |
| NA | -0.457362293 | 0.875782454 | | down |
| MIR378G | -0.457362293 | 0.875782454 | | down |
| RP11-24P14.1 | -0.457362293 | 0.875782454 | | down |
| SNORA42 | -0.457362293 | 0.875782454 | | down |
| PFN1P9 | -0.457362293 | 0.875782454 | | down |
| RNU6-179P | -0.457362293 | 0.875782454 | | down |
| RPS23P10 | -0.457362293 | 0.875782454 | | down |
| RNA5SP64 | -0.457362293 | 0.875782454 | | down |
| RP5-1097F14.3 | -0.457362293 | 0.875782454 | | down |
| AC080094.1 | -0.457362293 | 0.875782454 | | down |
| AC012506.1 | -0.457362293 | 0.875782454 | | down |
| NDUFB4P4 | -0.457362293 | 0.875782454 | | down |
| RP4-614C10.1 | -0.457362293 | 0.875782454 | | down |
| RPS24P6 | -0.457362293 | 0.875782454 | | down |

| | | | |
|---|---|---|---|
| AC103563.4 | -0.457362293 | 0.875782454 | down |
| AC016724.6 | -0.457362293 | 0.875782454 | down |
| AC092646.1 | -0.457362293 | 0.875782454 | down |
| NA | -0.457362293 | 0.875782454 | down |
| MIR375 | -0.457362293 | 0.875782454 | down |
| RPL23AP39 | -0.457362293 | 0.875782454 | down |
| OR7E53P | -0.457362293 | 0.875782454 | down |
| RP11-305F5.2 | -0.457362293 | 0.875782454 | down |
| RNU6-823P | -0.457362293 | 0.875782454 | down |
| CLRN1 | -0.457362293 | 0.875782454 | down |
| MRPL42P6 | -0.457362293 | 0.875782454 | down |
| KCNAB1-AS1 | -0.457362293 | 0.875782454 | down |
| OR4D12P | -0.457362293 | 0.875782454 | down |
| MIR4802 | -0.457362293 | 0.875782454 | down |
| RP11-30P21.2 | -0.457362293 | 0.875782454 | down |
| CSN1S2AP | -0.457362293 | 0.875782454 | down |
| RP11-477G18.1 | -0.457362293 | 0.875782454 | down |
| RP11-124N3.3 | -0.457362293 | 0.875782454 | down |
| GCNT1P2 | -0.457362293 | 0.875782454 | down |
| CTD-2134P3.1 | -0.457362293 | 0.875782454 | down |
| CTD-2316B1.1 | -0.457362293 | 0.875782454 | down |
| MIR3691 | -0.457362293 | 0.875782454 | down |
| RN7SKP293 | -0.457362293 | 0.875782454 | down |
| RP11-457M11.5 | -0.457362293 | 0.875782454 | down |
| RP11-344J7.3 | -0.457362293 | 0.875782454 | down |
| NA | -0.457362293 | 0.875782454 | down |
| RP11-542F9.1 | -0.457362293 | 0.875782454 | down |
| RPL7P27 | -0.457362293 | 0.875782454 | down |
| RN7SL11P | -0.457362293 | 0.875782454 | down |
| RPSAP45 | -0.457362293 | 0.875782454 | down |
| RNU6-200P | -0.457362293 | 0.875782454 | down |
| RPS4XP9 | -0.457362293 | 0.875782454 | down |
| HLFP1 | -0.457362293 | 0.875782454 | down |
| RP11-432L12.1 | -0.457362293 | 0.875782454 | down |
| NA | -0.457362293 | 0.875782454 | down |
| AC004920.3 | -0.457362293 | 0.875782454 | down |
| TRIM60P16 | -0.457362293 | 0.875782454 | down |
| RP11-1217F2.9 | -0.457362293 | 0.875782454 | down |
| CTB-10G5.1 | -0.457362293 | 0.875782454 | down |
| Y_RNA | -0.457362293 | 0.875782454 | down |
| RP11-203P23.2 | -0.457362293 | 0.875782454 | down |
| AC073387.2 | -0.457362293 | 0.875782454 | down |
| MTCYBP24 | -0.457362293 | 0.875782454 | down |
| RN7SKP277 | -0.457362293 | 0.875782454 | down |
| TRBV8-1 | -0.457362293 | 0.875782454 | down |
| RP11-208G20.2 | -0.457362293 | 0.875782454 | down |
| MOS | -0.457362293 | 0.875782454 | down |
| RNU6-1324P | -0.457362293 | 0.875782454 | down |
| RP11-700E23.1 | -0.457362293 | 0.875782454 | down |
| RP11-22C11.1 | -0.457362293 | 0.875782454 | down |
| MIR875 | -0.457362293 | 0.875782454 | down |

| | | | |
|---|---|---|---|
| TRHR | -0.457362293 | 0.875782454 | down |
| SERPINA15P | -0.457362293 | 0.875782454 | down |
| NA | -0.457362293 | 0.875782454 | down |
| GTF3AP1 | -0.457362293 | 0.875782454 | down |
| RNU7-185P | -0.457362293 | 0.875782454 | down |
| RP11-18A15.1 | -0.457362293 | 0.875782454 | down |
| RP11-462B18.1 | -0.457362293 | 0.875782454 | down |
| OR13C6P | -0.457362293 | 0.875782454 | down |
| RP11-392E22.11 | -0.457362293 | 0.875782454 | down |
| RPS15AP27 | -0.457362293 | 0.875782454 | down |
| MIR7150 | -0.457362293 | 0.875782454 | down |
| LINC00701 | -0.457362293 | 0.875782454 | down |
| RN7SKP37 | -0.457362293 | 0.875782454 | down |
| ACTR3BP5 | -0.457362293 | 0.875782454 | down |
| RP11-366I13.3 | -0.457362293 | 0.875782454 | down |
| RP11-107I14.2 | -0.457362293 | 0.875782454 | down |
| KCNK18 | -0.457362293 | 0.875782454 | down |
| NA | -0.457362293 | 0.875782454 | down |
| RNU6-1143P | -0.457362293 | 0.875782454 | down |
| CTD-2302A16.2 | -0.457362293 | 0.875782454 | down |
| CTD-2560E9.5 | -0.457362293 | 0.875782454 | down |
| RP11-107P7.1 | -0.457362293 | 0.875782454 | down |
| AP000438.4 | -0.457362293 | 0.875782454 | down |
| RP11-655M14.14 | -0.457362293 | 0.875782454 | down |
| OSBPL9P2 | -0.457362293 | 0.875782454 | down |
| RP11-178H8.2 | -0.457362293 | 0.875782454 | down |
| RP11-212D19.4 | -0.457362293 | 0.875782454 | down |
| RP11-627K11.5 | -0.457362293 | 0.875782454 | down |
| RNU6-618P | -0.457362293 | 0.875782454 | down |
| RP11-8P13.1 | -0.457362293 | 0.875782454 | down |
| NA | -0.457362293 | 0.875782454 | down |
| RP11-299A16.1 | -0.457362293 | 0.875782454 | down |
| RP11-357K6.3 | -0.457362293 | 0.875782454 | down |
| RP11-495K9.10 | -0.457362293 | 0.875782454 | down |
| MTND3P1 | -0.457362293 | 0.875782454 | down |
| RPL7AP73 | -0.457362293 | 0.875782454 | down |
| HMGA1P6 | -0.457362293 | 0.875782454 | down |
| RNY4P9 | -0.457362293 | 0.875782454 | down |
| LINC00331 | -0.457362293 | 0.875782454 | down |
| MOB1AP1 | -0.457362293 | 0.875782454 | down |
| MEMO1P5 | -0.457362293 | 0.875782454 | down |
| TRAV28 | -0.457362293 | 0.875782454 | down |
| RP11-260G13.1 | -0.457362293 | 0.875782454 | down |
| KRT18P6 | -0.457362293 | 0.875782454 | down |
| CTD-2325K12.1 | -0.457362293 | 0.875782454 | down |
| DPPA5P4 | -0.457362293 | 0.875782454 | down |
| SNORD113 | -0.457362293 | 0.875782454 | down |
| SNORD114-22 | -0.457362293 | 0.875782454 | down |
| CTD-3118D7.1 | -0.457362293 | 0.875782454 | down |
| RN7SL673P | -0.457362293 | 0.875782454 | down |
| RP11-86K22.1 | -0.457362293 | 0.875782454 | down |

| | | | | |
|---|---|---|---|---|
| HMGN2P47 | -0.457362293 | 0.875782454 | | down |
| RP11-282M16.1 | -0.457362293 | 0.875782454 | | down |
| SNORD74 | -0.457362293 | 0.875782454 | | down |
| LINC00052 | -0.457362293 | 0.875782454 | | down |
| Z69720.2 | -0.457362293 | 0.875782454 | | down |
| RP11-66602.1 | -0.457362293 | 0.875782454 | | down |
| RP11-452L6.8 | -0.457362293 | 0.875782454 | | down |
| CTD-2014E2.2 | -0.457362293 | 0.875782454 | | down |
| RP11-104C4.6 | -0.457362293 | 0.875782454 | | down |
| RP11-434E6.2 | -0.457362293 | 0.875782454 | | down |
| RP11-354I13.1 | -0.457362293 | 0.875782454 | | down |
| AC013248.2 | -0.457362293 | 0.875782454 | | down |
| RP11-22N12.2 | -0.457362293 | 0.875782454 | | down |
| RP5-1110E20.1 | -0.457362293 | 0.875782454 | | down |
| KRTAP2-4 | -0.457362293 | 0.875782454 | | down |
| U3 | -0.457362293 | 0.875782454 | | down |
| CTD-2526A2.5 | -0.457362293 | 0.875782454 | | down |
| SNORA70 | -0.457362293 | 0.875782454 | | down |
| SCML2P1 | -0.457362293 | 0.875782454 | | down |
| RP11-454P7.3 | -0.457362293 | 0.875782454 | | down |
| RP11-638L3.3 | -0.457362293 | 0.875782454 | | down |
| AC002314.4 | -0.457362293 | 0.875782454 | | down |
| RN7SL498P | -0.457362293 | 0.875782454 | | down |
| RP11-19D2.1 | -0.457362293 | 0.875782454 | | down |
| NCSTNP1 | -0.457362293 | 0.875782454 | | down |
| XXbac-B33L19.10 | -0.457362293 | 0.875782454 | | down |
| KB-1183D5.13 | -0.457362293 | 0.875782454 | | down |
| Metazoa_SRP | -0.457362293 | 0.875782454 | | down |
| RP6-24A23.8 | -0.457362293 | 0.875782454 | | down |
| RP13-147D17.1 | -0.457362293 | 0.875782454 | | down |
| RP1-137H15.2 | -0.457362293 | 0.875782454 | | down |
| RP1-169P22.2 | -0.457362293 | 0.875782454 | | down |
| MIR892A | -0.457362293 | 0.875782454 | | down |
| RBMY2JP | -0.457362293 | 0.875782454 | | down |
| RP11-473A10.2 | -0.457343759 | 0.865601877 | | down |
| RP11-65M17.3 | -0.45731039 | 0.511357492 | 0.637081671 | down |
| AC107081.5 | -0.457169361 | 0.281498271 | 0.417955722 | down |
| HUS1B | -0.457074291 | 0.340081749 | 0.478410642 | down |
| AC024560.2 | -0.457029863 | 0.398366166 | 0.535336275 | down |
| NA | -0.456956861 | 0.561468265 | 0.68065417 | down |
| GRAMD4 | -0.456827552 | 0.002546975 | 0.012753303 | down |
| KCNN4 | -0.456699254 | 0.111569194 | 0.21869804 | down |
| TCP11L2 | -0.456657155 | 0.082869509 | 0.176789856 | down |
| NUSAP1 | -0.45663819 | 0.188787516 | 0.315826724 | down |
| RNPS1P1 | -0.45659735 | 0.358255987 | 0.496697871 | down |
| TSPAN17 | -0.456565357 | 0.003397978 | 0.015983852 | down |
| ETFA | -0.456480043 | 0.000992531 | 0.006025382 | down |
| FGFBP1 | -0.456299304 | 0.338621283 | 0.477070942 | down |
| RP11-298I3.4 | -0.456268168 | 0.303418853 | 0.441470097 | down |
| TAF1A-AS1 | -0.456177458 | 0.078395478 | 0.169691192 | down |
| RPL23AP87 | -0.456165179 | 0.471575001 | 0.60213744 | down |

| | | | | |
|---|---|---|---|---|
| RP11-629B11.5 | -0.455960966 | 0.409026564 | 0.545795135 | down |
| TRIM24 | -0.45591945 | 1.31E-05 | 0.000177206 | down |
| PPP3CB-AS1 | -0.45587293 | 0.01590533 | 0.052207991 | down |
| CHRNB2 | -0.455857711 | 0.433444002 | 0.568464798 | down |
| EBF2 | -0.455650863 | 0.383266525 | 0.520963646 | down |
| CDT1 | -0.455572289 | 0.055993451 | 0.132883793 | down |
| USP37 | -0.455553987 | 0.155058565 | 0.275138568 | down |
| RP11-54F2.9 | -0.45547988 | 0.744522652 | 0.826532113 | down |
| RP11-1094M14.1 | -0.455456455 | 0.333433794 | 0.471709041 | down |
| MEST | -0.455455975 | 0.019960471 | 0.062002935 | down |
| AC007238.1 | -0.455377502 | 0.208822609 | 0.339018158 | down |
| PADI1 | -0.455363368 | 0.453898953 | 0.586459388 | down |
| PRAM1 | -0.455322316 | 0.103950054 | 0.207587742 | down |
| PARD3B | -0.45526704 | 0.106799256 | 0.211906155 | down |
| LIX1 | -0.455077258 | 0.471113096 | 0.601763552 | down |
| NCAPH2 | -0.455048153 | 0.014820769 | 0.049496562 | down |
| FAM159A | -0.454838468 | 0.213171439 | 0.344051717 | down |
| CEBPZOS | -0.454627731 | 0.022258757 | 0.067169758 | down |
| LGI4 | -0.454274393 | 0.125020819 | 0.236694349 | down |
| CCDC61 | -0.454246706 | 0.010131636 | 0.037038564 | down |
| RP11-38F22.1 | -0.454224028 | 0.876627944 | | down |
| RP11-626K17.3 | -0.454224028 | 0.876627944 | | down |
| HNRNPA3P16 | -0.454224028 | 0.876627944 | | down |
| GSTM5P1 | -0.454223804 | 0.870139602 | | down |
| CTD-2542C24.5 | -0.454223787 | 0.869584359 | | down |
| TPM3P1 | -0.454223311 | 0.851260616 | | down |
| AC079613.1 | -0.454223015 | 0.835052503 | | down |
| RN7SL688P | -0.454222988 | 0.833253624 | | down |
| AC011284.3 | -0.454222752 | 0.815035655 | | down |
| TRGV6 | -0.454222582 | 0.797493437 | | down |
| RP11-54D18.4 | -0.454222442 | 0.778474796 | | down |
| RP4-816N1.6 | -0.454222428 | 0.776254397 | | down |
| RP11-17E2.2 | -0.454222414 | 0.773958286 | | down |
| RPL3P10 | -0.454222401 | 0.771858994 | | down |
| FTH1P8 | -0.454219498 | 0.088160348 | 0.184770806 | down |
| NRDE2 | -0.454212304 | 1.29E-06 | 2.53E-05 | down |
| RP11-582J16.4 | -0.454201625 | 0.094225223 | 0.193817787 | down |
| DUX4L37 | -0.45417898 | 0.617593905 | 0.728332206 | down |
| CTB-1144G6.6 | -0.454087601 | 0.38875706 | 0.526378706 | down |
| RP11-230G5.2 | -0.453965921 | 0.620622795 | 0.730808068 | down |
| UBR5-AS1 | -0.453568082 | 0.290601563 | 0.427804459 | down |
| NOL3 | -0.45352284 | 0.021251624 | 0.064949673 | down |
| RP11-569A11.1 | -0.453506376 | 0.220970569 | 0.352642078 | down |
| RPL24P7 | -0.453436719 | 0.605911825 | | down |
| RP11-298I3.3 | -0.453391814 | 0.501952549 | 0.628867942 | down |
| ZNF775 | -0.453291472 | 0.034467297 | 0.093090207 | down |
| RP11-701H16.4 | -0.453055144 | 0.56019163 | 0.67955491 | down |
| CYP4V2 | -0.453014021 | 0.004502959 | 0.020007133 | down |
| NCK1-AS1 | -0.452966989 | 0.053057985 | 0.127576465 | down |
| NA | -0.452801012 | 0.016221864 | 0.052960241 | down |
| ARHGAP26 | -0.452483066 | 0.007045175 | 0.028133114 | down |

| | | | | |
|---|---|---|---|---|
| SLC7A8 | -0.45233627 | 0.015389209 | 0.05094494 | down |
| RELL1 | -0.452133443 | 0.03154315 | 0.087015188 | down |
| C21orf58 | -0.452126048 | 0.073800872 | 0.16247332 | down |
| RP11-488L18.3 | -0.452091343 | 0.399506543 | 0.536436853 | down |
| LA16c-321D4.2 | -0.452078896 | 0.208304663 | 0.338504044 | down |
| FAM63A | -0.452023257 | 0.00456495 | 0.020219573 | down |
| BTBD6P1 | -0.452013816 | 0.449889829 | 0.582833427 | down |
| AC092620.3 | -0.451995299 | 0.517754097 | 0.642625965 | down |
| HIST1H2BO | -0.451957528 | 0.482629905 | 0.611845768 | down |
| NEDD4 | -0.451954465 | 0.235225912 | 0.368079548 | down |
| HM13-IT1 | -0.451764417 | 0.38584967 | 0.523574702 | down |
| RP11-449D8.1 | -0.451672862 | 0.313938146 | 0.452589139 | down |
| ZDHHC3 | -0.451661999 | 0.000172648 | 0.00148116 | down |
| TALDO1 | -0.451578725 | 0.01240087 | 0.043211753 | down |
| MLYCD | -0.451512237 | 0.000249095 | 0.002006925 | down |
| VWA2 | -0.45138895 | 0.248106659 | 0.382419273 | down |
| FEZF1-AS1 | -0.451375776 | 0.702891338 | | down |
| CCDC160 | -0.451330519 | 0.614488278 | | down |
| RPL8 | -0.451267292 | 0.017417016 | 0.0558937 | down |
| MIR3681HG | -0.451231687 | 0.379638944 | 0.517628216 | down |
| STUM | -0.45113445 | 0.314000926 | 0.45261449 | down |
| ZNF366 | -0.45108466 | 0.344075355 | 0.482559984 | down |
| MYBPC3 | -0.450647635 | 0.239371653 | 0.372954516 | down |
| CTD-2027I19.3 | -0.450549171 | 0.416710879 | 0.552890905 | down |
| ALDH3A1 | -0.450391968 | 0.168292146 | 0.291657201 | down |
| RP6-159A1.2 | -0.450237144 | 0.515076908 | | down |
| RP11-66B24.4 | -0.450179171 | 0.088519245 | 0.185338627 | down |
| RP11-121A14.2 | -0.450100344 | 0.281985484 | 0.418492979 | down |
| EXOSC2 | -0.449909182 | 0.034784932 | 0.093636637 | down |
| CTD-260009.2 | -0.449856742 | 0.518580151 | 0.643281158 | down |
| FBXO43 | -0.449734329 | 0.310503236 | 0.44899362 | down |
| NIPAL4 | -0.449306811 | 0.297072378 | 0.434487515 | down |
| CCR10 | -0.449217118 | 0.200947005 | 0.329976119 | down |
| TRAPPC2L | -0.448956104 | 0.048053615 | 0.118834703 | down |
| AOAH-IT1 | -0.448948706 | 0.640270034 | | down |
| RP11-1006G14.2 | -0.448920155 | 0.598089008 | | down |
| RP13-270P17.2 | -0.44876504 | 0.353206806 | 0.491876578 | down |
| NA | -0.448664084 | 0.44236705 | 0.575943649 | down |
| IL1RAP | -0.448642158 | 0.152419049 | 0.271897595 | down |
| SBF2-AS1 | -0.448566267 | 0.019457126 | 0.06082484 | down |
| OR7E46P | -0.448343771 | 0.821338938 | | down |
| RP11-51J9.6 | -0.448322129 | 0.248799225 | 0.38329012 | down |
| KCNH3 | -0.448274137 | 0.218540666 | 0.350013633 | down |
| RNU6-190P | -0.448186758 | 0.716117462 | | down |
| GALK1 | -0.448094782 | 0.039720038 | 0.103409966 | down |
| RP5-1053E7.2 | -0.447953243 | 0.878317796 | | down |
| HNRNPFP1 | -0.447953243 | 0.878317796 | | down |
| RP11-484D4.3 | -0.447953243 | 0.878317796 | | down |
| KATNBL1P2 | -0.447953243 | 0.878317796 | | down |
| RP11-403I13.9 | -0.447953243 | 0.878317796 | | down |
| RP11-404F10.5 | -0.447953243 | 0.878317796 | | down |

| | | | |
|---|---|---|---|
| RP11-38C18.2 | -0.447953243 | 0.878317796 | down |
| TNR-IT1 | -0.447953243 | 0.878317796 | down |
| FAM58BP | -0.447953243 | 0.878317796 | down |
| RP11-378J18.10 | -0.447953243 | 0.878317796 | down |
| SNRPD2P2 | -0.447953243 | 0.878317796 | down |
| RNU4-73P | -0.447953243 | 0.878317796 | down |
| RP11-182E7.1 | -0.447953243 | 0.878317796 | down |
| RP11-314C9.1 | -0.447953243 | 0.878317796 | down |
| TGFA-IT1 | -0.447953243 | 0.878317796 | down |
| RP11-717A5.1 | -0.447953243 | 0.878317796 | down |
| AC018696.1 | -0.447953243 | 0.878317796 | down |
| NA | -0.447953243 | 0.878317796 | down |
| RP11-77A13.1 | -0.447953243 | 0.878317796 | down |
| MTND4P21 | -0.447953243 | 0.878317796 | down |
| AC104777.4 | -0.447953243 | 0.878317796 | down |
| AC013733.3 | -0.447953243 | 0.878317796 | down |
| RNU6-1051P | -0.447953243 | 0.878317796 | down |
| RPSAP32 | -0.447953243 | 0.878317796 | down |
| THOC7-AS1 | -0.447953243 | 0.878317796 | down |
| ENPP7P2 | -0.447953243 | 0.878317796 | down |
| RP11-779P15.2 | -0.447953243 | 0.878317796 | down |
| RPS3AP14 | -0.447953243 | 0.878317796 | down |
| MARK3P3 | -0.447953243 | 0.878317796 | down |
| SNORA58 | -0.447953243 | 0.878317796 | down |
| NA | -0.447953243 | 0.878317796 | down |
| RNU6-646P | -0.447953243 | 0.878317796 | down |
| NA | -0.447953243 | 0.878317796 | down |
| RP11-141P6.1 | -0.447953243 | 0.878317796 | down |
| RP11-468N14.1 | -0.447953243 | 0.878317796 | down |
| SNORA31 | -0.447953243 | 0.878317796 | down |
| HNRNPA3P13 | -0.447953243 | 0.878317796 | down |
| MIR1255A | -0.447953243 | 0.878317796 | down |
| RP11-170N16.2 | -0.447953243 | 0.878317796 | down |
| STMN1P2 | -0.447953243 | 0.878317796 | down |
| RP11-173E2.2 | -0.447953243 | 0.878317796 | down |
| RP11-91J3.1 | -0.447953243 | 0.878317796 | down |
| NA | -0.447953243 | 0.878317796 | down |
| ROPN1L-AS1 | -0.447953243 | 0.878317796 | down |
| RNU6-760P | -0.447953243 | 0.878317796 | down |
| RP11-94D20.1 | -0.447953243 | 0.878317796 | down |
| SAP18P1 | -0.447953243 | 0.878317796 | down |
| ATP6V1G1P6 | -0.447953243 | 0.878317796 | down |
| RP11-254I22.2 | -0.447953243 | 0.878317796 | down |
| AC034228.2 | -0.447953243 | 0.878317796 | down |
| SNORD45 | -0.447953243 | 0.878317796 | down |
| APOOP1 | -0.447953243 | 0.878317796 | down |
| HLA-Z | -0.447953243 | 0.878317796 | down |
| MYL8P | -0.447953243 | 0.878317796 | down |
| RNU1-88P | -0.447953243 | 0.878317796 | down |
| NA | -0.447953243 | 0.878317796 | down |
| RP11-151M7.1 | -0.447953243 | 0.878317796 | down |

| | | | |
|---|---|---|---|
| RPS3AP23 | -0.447953243 | 0.878317796 | down |
| RP11-235G24.3 | -0.447953243 | 0.878317796 | down |
| NA | -0.447953243 | 0.878317796 | down |
| AC009531.2 | -0.447953243 | 0.878317796 | down |
| HMGN2P30 | -0.447953243 | 0.878317796 | down |
| RNU6-863P | -0.447953243 | 0.878317796 | down |
| RP4-777G9.1 | -0.447953243 | 0.878317796 | down |
| OR7E15P | -0.447953243 | 0.878317796 | down |
| RP11-436P7.2 | -0.447953243 | 0.878317796 | down |
| RP11-705O24.2 | -0.447953243 | 0.878317796 | down |
| KB-1043D8.8 | -0.447953243 | 0.878317796 | down |
| KB-1907C4.2 | -0.447953243 | 0.878317796 | down |
| Y_RNA | -0.447953243 | 0.878317796 | down |
| RP11-26E5.1 | -0.447953243 | 0.878317796 | down |
| SYF2P2 | -0.447953243 | 0.878317796 | down |
| RP11-111F5.1 | -0.447953243 | 0.878317796 | down |
| RP11-574E24.3 | -0.447953243 | 0.878317796 | down |
| RP11-379P1.4 | -0.447953243 | 0.878317796 | down |
| XXyac-YM21GA2.1 | -0.447953243 | 0.878317796 | down |
| RP11-62C3.8 | -0.447953243 | 0.878317796 | down |
| RP11-482E14.2 | -0.447953243 | 0.878317796 | down |
| Y_RNA | -0.447953243 | 0.878317796 | down |
| RP11-526K21.2 | -0.447953243 | 0.878317796 | down |
| MIR6744 | -0.447953243 | 0.878317796 | down |
| OR51A3P | -0.447953243 | 0.878317796 | down |
| OR2D2 | -0.447953243 | 0.878317796 | down |
| RP11-573E11.2 | -0.447953243 | 0.878317796 | down |
| SNORA1 | -0.447953243 | 0.878317796 | down |
| RP11-347H15.1 | -0.447953243 | 0.878317796 | down |
| RNA5SP341 | -0.447953243 | 0.878317796 | down |
| OR10Q1 | -0.447953243 | 0.878317796 | down |
| RN7SL259P | -0.447953243 | 0.878317796 | down |
| RP11-643C9.1 | -0.447953243 | 0.878317796 | down |
| RP11-683O4.1 | -0.447953243 | 0.878317796 | down |
| Y_RNA | -0.447953243 | 0.878317796 | down |
| RP11-708B6.2 | -0.447953243 | 0.878317796 | down |
| NA | -0.447953243 | 0.878317796 | down |
| TSPAN9-IT1 | -0.447953243 | 0.878317796 | down |
| NA | -0.447953243 | 0.878317796 | down |
| UBE2L2 | -0.447953243 | 0.878317796 | down |
| RP11-1060J15.7 | -0.447953243 | 0.878317796 | down |
| RP11-362A1.2 | -0.447953243 | 0.878317796 | down |
| RP11-718L23.1 | -0.447953243 | 0.878317796 | down |
| RNU6-1068P | -0.447953243 | 0.878317796 | down |
| U7 | -0.447953243 | 0.878317796 | down |
| MIR620 | -0.447953243 | 0.878317796 | down |
| RP11-495K9.9 | -0.447953243 | 0.878317796 | down |
| RNU4ATAC12P | -0.447953243 | 0.878317796 | down |
| NA | -0.447953243 | 0.878317796 | down |
| RPL36P19 | -0.447953243 | 0.878317796 | down |
| CTD-2314B22.1 | -0.447953243 | 0.878317796 | down |

| | | | | |
|---|---|---|---|---|
| EDDM3DP | -0.447953243 | 0.878317796 | | down |
| TRAJ41 | -0.447953243 | 0.878317796 | | down |
| RP11-1085N6.4 | -0.447953243 | 0.878317796 | | down |
| CTD-2315A10.1 | -0.447953243 | 0.878317796 | | down |
| SNORD114-6 | -0.447953243 | 0.878317796 | | down |
| RP11-32B5.2 | -0.447953243 | 0.878317796 | | down |
| Y_RNA | -0.447953243 | 0.878317796 | | down |
| RP11-315O8.1 | -0.447953243 | 0.878317796 | | down |
| Y_RNA | -0.447953243 | 0.878317796 | | down |
| RP11-344A16.2 | -0.447953243 | 0.878317796 | | down |
| Metazoa_SRP | -0.447953243 | 0.878317796 | | down |
| RP11-354B3.1 | -0.447953243 | 0.878317796 | | down |
| OR4F15 | -0.447953243 | 0.878317796 | | down |
| RP11-21B23.2 | -0.447953243 | 0.878317796 | | down |
| RP11-324D17.1 | -0.447953243 | 0.878317796 | | down |
| GS1-21A4.2 | -0.447953243 | 0.878317796 | | down |
| RN7SL784P | -0.447953243 | 0.878317796 | | down |
| NMTRQ-TTG12-1 | -0.447953243 | 0.878317796 | | down |
| CTD-2370N5.3 | -0.447953243 | 0.878317796 | | down |
| MIR6779 | -0.447953243 | 0.878317796 | | down |
| FRG2LP | -0.447953243 | 0.878317796 | | down |
| NA | -0.447953243 | 0.878317796 | | down |
| RPIAP1 | -0.447953243 | 0.878317796 | | down |
| RP11-106E15.1 | -0.447953243 | 0.878317796 | | down |
| RNA5SP465 | -0.447953243 | 0.878317796 | | down |
| CTD-3162L10.4 | -0.447953243 | 0.878317796 | | down |
| RN7SL718P | -0.447953243 | 0.878317796 | | down |
| MIR4323 | -0.447953243 | 0.878317796 | | down |
| RNU4-60P | -0.447953243 | 0.878317796 | | down |
| CTB-147C22.6 | -0.447953243 | 0.878317796 | | down |
| MIR518E | -0.447953243 | 0.878317796 | | down |
| RPL21P2 | -0.447953243 | 0.878317796 | | down |
| MACROD2-AS1 | -0.447953243 | 0.878317796 | | down |
| SOCS2P1 | -0.447953243 | 0.878317796 | | down |
| MIR124-3 | -0.447953243 | 0.878317796 | | down |
| AF015262.2 | -0.447953243 | 0.878317796 | | down |
| FAM230A | -0.447953243 | 0.878317796 | | down |
| NA | -0.447953243 | 0.878317796 | | down |
| IGLV3-15 | -0.447953243 | 0.878317796 | | down |
| RP11-126O22.7 | -0.447953243 | 0.878317796 | | down |
| RPL6P30 | -0.447953243 | 0.878317796 | | down |
| RNU6-1189P | -0.447953243 | 0.878317796 | | down |
| RNASEH2CP1 | -0.447953243 | 0.878317796 | | down |
| OFD1P2Y | -0.447953243 | 0.878317796 | | down |
| RP11-731D1.3 | -0.447952571 | 0.866973048 | | down |
| PITRM1-AS1 | -0.447879436 | 0.330154411 | 0.468695974 | down |
| MYO9A | -0.447710989 | 0.062065513 | 0.143316906 | down |
| TMEM41B | -0.447636176 | 0.003981642 | 0.018177244 | down |
| RP11-855A2.2 | -0.447616962 | 0.576061388 | | down |
| SERHL2 | -0.447507079 | 0.09671137 | 0.19740447 | down |
| NONOP2 | -0.447476476 | 0.326195738 | 0.464569556 | down |

| | | | | |
|---|---|---|---|---|
| AC006277.3 | -0.447251522 | 0.181712059 | 0.307257829 | down |
| RP3-522J7.7 | -0.447249859 | 0.448388384 | 0.581406817 | down |
| GUSBP5 | -0.447012393 | 0.361777055 | 0.500079588 | down |
| RPSAP8 | -0.446964458 | 0.314265493 | 0.45290893 | down |
| PIGX | -0.446943756 | 0.001626161 | 0.00890078 | down |
| RP11-15L13.4 | -0.446913523 | 0.629222904 | | down |
| HOXD-AS2 | -0.446802214 | 0.243793756 | 0.377806757 | down |
| PGP | -0.446797411 | 0.026507369 | 0.076571079 | down |
| ZNF229 | -0.446643118 | 0.23604267 | 0.369011655 | down |
| FBXO15 | -0.44650896 | 0.300403325 | 0.438100942 | down |
| SERGEF | -0.446456506 | 0.027125412 | 0.077934002 | down |
| ZCWPW1 | -0.44642251 | 0.021769593 | 0.0660986 | down |
| RP3-441A12.1 | -0.446170592 | 0.573794282 | | down |
| E2F3P1 | -0.446037708 | 0.570495782 | | down |
| RP11-108M9.4 | -0.445925818 | 0.083478017 | 0.177659101 | down |
| HIST1H4F | -0.445799664 | 0.415909066 | 0.552278037 | down |
| RP13-991F5.2 | -0.445600263 | 0.572655691 | 0.689961004 | down |
| RP11-477D19.2 | -0.445464967 | 0.124143777 | 0.235569736 | down |
| IP6K1 | -0.44534635 | 0.009157142 | 0.034286786 | down |
| ARPC1A | -0.445198104 | 0.055716939 | 0.13236022 | down |
| MRPS5 | -0.445068969 | 0.002357901 | 0.011994714 | down |
| HOXD1 | -0.445026053 | 0.381192011 | 0.519043416 | down |
| RP11-326K13.4 | -0.444985926 | 0.149002162 | 0.267724475 | down |
| SLC16A11 | -0.44496538 | 0.133151386 | 0.247289376 | down |
| LINC00604 | -0.444814978 | 0.879163705 | | down |
| LINC00529 | -0.444814978 | 0.879163705 | | down |
| RP11-90C1.1 | -0.444814978 | 0.879163705 | | down |
| C16orf97 | -0.444814978 | 0.879163705 | | down |
| NA | -0.444814813 | 0.874168008 | | down |
| RP11-640N20.8 | -0.44481476 | 0.872434329 | | down |
| AC016723.4 | -0.444814345 | 0.855826937 | | down |
| LINC01507 | -0.444814338 | 0.855501859 | | down |
| RP11-546M21.6 | -0.444814015 | 0.836695064 | | down |
| KRTAP29-1 | -0.444813634 | 0.800880023 | | down |
| CTD-2553L13.5 | -0.444813614 | 0.798329669 | | down |
| MFSD1P1 | -0.444813508 | 0.782701628 | | down |
| NA | -0.444813493 | 0.780224035 | | down |
| RP1-125I3.2 | -0.444813405 | 0.763361599 | | down |
| RP1-506.4 | -0.444813334 | 0.746585822 | | down |
| SPINK13 | -0.444813317 | 0.741996302 | | down |
| RP11-286N3.2 | -0.444813255 | 0.722890706 | | down |
| RPL7P24 | -0.444813253 | 0.722491178 | | down |
| RP11-1399P15.1 | -0.444446533 | 0.240589199 | 0.374308005 | down |
| C1QA | -0.444345405 | 0.101031433 | 0.203485175 | down |
| PKDCC | -0.444198885 | 0.070105557 | 0.156528787 | down |
| FTH1P20 | -0.444138849 | 0.142428348 | 0.259356159 | down |
| HSD17B8 | -0.444074502 | 0.041771214 | 0.107382079 | down |
| AKR7A2 | -0.443991107 | 0.010528772 | 0.038162193 | down |
| RP11-327E2.5 | -0.443977317 | 0.199631631 | 0.328649004 | down |
| RP5-867C24.1 | -0.443975941 | 0.634847181 | 0.742009287 | down |
| RP11-1000B6.5 | -0.443965006 | 0.501324179 | 0.628374832 | down |

| | | | | |
|---|---|---|---|---|
| RP11-767C1.2 | -0.443922522 | 0.321565371 | 0.460315895 | down |
| TMEM192 | -0.443872251 | 0.000505071 | 0.003503911 | down |
| Y_RNA | -0.443572848 | 0.419298986 | 0.555137964 | down |
| LINC00670 | -0.443525856 | 0.636731663 | 0.743554839 | down |
| HMBS | -0.443457497 | 0.029999522 | 0.083780038 | down |
| VTRNA1-3 | -0.442885514 | 0.681862252 | | down |
| MINCR | -0.442868754 | 0.035051549 | 0.094160295 | down |
| ZNF69 | -0.442691789 | 0.027672803 | 0.07909825 | down |
| AC074117.10 | -0.442592758 | 0.021772775 | 0.066101581 | down |
| AC079781.8 | -0.442574583 | 0.607641351 | 0.719872901 | down |
| RP11-848P1.3 | -0.442568466 | 0.13674196 | 0.252035984 | down |
| CTD-2312P21.1 | -0.442255283 | 0.855232122 | | down |
| RREB1 | -0.442233922 | 0.002300398 | 0.011763943 | down |
| UMODL1-AS1 | -0.441943169 | 0.672209964 | 0.771659642 | down |
| RP4-585I14.3 | -0.441936525 | 0.545750571 | | down |
| ZNF600 | -0.441897414 | 0.010750267 | 0.038777888 | down |
| RGPD5 | -0.441853875 | 0.421017966 | 0.556675795 | down |
| RP11-296O14.2 | -0.441824032 | 0.59352073 | | down |
| RP3-477M7.5 | -0.441811648 | 0.601254438 | 0.714334401 | down |
| RP11-473E2.4 | -0.441669747 | 0.448023609 | 0.58119263 | down |
| UBE2V1P4 | -0.441652078 | 0.870363816 | | down |
| FAXDC2 | -0.441575742 | 0.097321413 | 0.198167101 | down |
| CLDN6 | -0.441333624 | 0.582684095 | 0.698678177 | down |
| C8orf82 | -0.441325042 | 0.003664455 | 0.016992573 | down |
| SEMA5B | -0.441320708 | 0.198677241 | 0.327484448 | down |
| WFDC21P | -0.44122962 | 0.260021759 | 0.395150778 | down |
| RP5-1024G6.5 | -0.441205765 | 0.401643706 | 0.538489979 | down |
| UPP2 | -0.441148497 | 0.457217742 | 0.589368659 | down |
| CHID1 | -0.441033191 | 0.087984047 | 0.184506129 | down |
| MAF | -0.440938491 | 0.106799461 | 0.211906155 | down |
| DHRS12 | -0.44086091 | 0.001941886 | 0.010230057 | down |
| NDUFA1 | -0.440694492 | 0.021491733 | 0.065458748 | down |
| CTD-2010I16.1 | -0.440652834 | 0.224478272 | 0.356686333 | down |
| HMGB3 | -0.440604465 | 0.007744766 | 0.030226522 | down |
| RP1-187B23.1 | -0.440582123 | 0.424514066 | 0.560151987 | down |
| ZSCAN12P1 | -0.440560985 | 0.215974657 | 0.347110188 | down |
| LINC01481 | -0.440551939 | 0.387270883 | 0.524957878 | down |
| CYP2R1 | -0.440506292 | 0.007649301 | 0.029962922 | down |
| CPEB1 | -0.440505721 | 0.162106382 | 0.284017037 | down |
| TMEM40 | -0.440491479 | 0.672142001 | | down |
| PCBP1-AS1 | -0.440291402 | 0.006235508 | 0.025691114 | down |
| CTD-2561B21.11 | -0.440287076 | 0.273020714 | 0.409210992 | down |
| P2RX2 | -0.440244378 | 0.312557114 | 0.451244243 | down |
| AP000569.9 | -0.440142226 | 0.495758163 | 0.623490236 | down |
| TMPPE | -0.440078081 | 0.195426692 | 0.323921413 | down |
| PDE1C | -0.43993535 | 0.118077531 | 0.227507301 | down |
| KIRREL3 | -0.439919801 | 0.129543954 | 0.242601559 | down |
| PIWIL4 | -0.439905879 | 0.007492545 | 0.02946414 | down |
| FGFBP2 | -0.439786217 | 0.284423071 | 0.421278186 | down |
| ZNF805 | -0.43964856 | 0.131293256 | 0.245015355 | down |
| WDR46 | -0.439601657 | 0.053013069 | 0.12747916 | down |

| | | | | |
|---|---|---|---|---|
| KIRREL2 | -0.43958368 | 0.377659974 | 0.515746193 | down |
| ARHGAP39 | -0.439576636 | 0.012779618 | 0.044193731 | down |
| PHC3 | -0.4395601 | 0.031247313 | 0.086405363 | down |
| SYN2 | -0.439484286 | 0.241893709 | 0.375870421 | down |
| RPL13AP25 | -0.439293113 | 0.138429464 | 0.254342043 | down |
| SVIL | -0.439284219 | 0.126765743 | 0.238982317 | down |
| RP11-82K18.2 | -0.439262321 | 0.63771685 | | down |
| RP11-157G21.2 | -0.439226008 | 0.503616876 | 0.63033003 | down |
| MROH5 | -0.439104451 | 0.551174527 | 0.671403158 | down |
| ATP5B | -0.439023759 | 0.013791239 | 0.046809059 | down |
| SUPV3L1 | -0.438992928 | 0.000231442 | 0.001885426 | down |
| ACOT7 | -0.438981023 | 0.003225727 | 0.015329601 | down |
| RP4-671G15.2 | -0.438913956 | 0.328219044 | 0.466683056 | down |
| AP5Z1 | -0.438899678 | 0.026257956 | 0.076008674 | down |
| CTD-2313F11.1 | -0.438893348 | 0.463203434 | 0.594874069 | down |
| LOXHD1 | -0.438815819 | 0.378422278 | 0.516505493 | down |
| AC005076.5 | -0.438636407 | 0.232560064 | 0.365277842 | down |
| RP11-360F5.1 | -0.438569517 | 0.332703991 | 0.471046466 | down |
| LINC01004 | -0.438566236 | 0.112836991 | 0.220106127 | down |
| RP5-1024G6.9 | -0.438544193 | 0.880854386 | | down |
| RP11-284F21.8 | -0.438544193 | 0.880854386 | | down |
| LARP7P1 | -0.438544193 | 0.880854386 | | down |
| RN7SKP98 | -0.438544193 | 0.880854386 | | down |
| Y_RNA | -0.438544193 | 0.880854386 | | down |
| OR2AJ1 | -0.438544193 | 0.880854386 | | down |
| OR2T3 | -0.438544193 | 0.880854386 | | down |
| PPIL1P1 | -0.438544193 | 0.880854386 | | down |
| AC092106.2 | -0.438544193 | 0.880854386 | | down |
| AC018799.1 | -0.438544193 | 0.880854386 | | down |
| NA | -0.438544193 | 0.880854386 | | down |
| SNRPCP13 | -0.438544193 | 0.880854386 | | down |
| RN7SL681P | -0.438544193 | 0.880854386 | | down |
| RP11-18D7.3 | -0.438544193 | 0.880854386 | | down |
| PRELID3BP4 | -0.438544193 | 0.880854386 | | down |
| NBPF22P | -0.438544193 | 0.880854386 | | down |
| AC034228.3 | -0.438544193 | 0.880854386 | | down |
| LINC01621 | -0.438544193 | 0.880854386 | | down |
| GJE1 | -0.438544193 | 0.880854386 | | down |
| OSTCP1 | -0.438544193 | 0.880854386 | | down |
| RP11-103H7.3 | -0.438544193 | 0.880854386 | | down |
| CDC42P3 | -0.438544193 | 0.880854386 | | down |
| RP11-74C3.1 | -0.438544193 | 0.880854386 | | down |
| Y_RNA | -0.438544193 | 0.880854386 | | down |
| RN7SKP47 | -0.438544193 | 0.880854386 | | down |
| HSD17B3 | -0.438544193 | 0.880854386 | | down |
| RP11-57G10.1 | -0.438544193 | 0.880854386 | | down |
| RP11-45A12.2 | -0.438544193 | 0.880854386 | | down |
| OR7E5P | -0.438544193 | 0.880854386 | | down |
| RP1-5308.2 | -0.438544193 | 0.880854386 | | down |
| RP11-841C19.1 | -0.438544193 | 0.880854386 | | down |
| RP11-58A17.3 | -0.438544193 | 0.880854386 | | down |

| | | | | |
|---|---|---|---|---|
| OSBPL9P4 | -0.438544193 | 0.880854386 | | down |
| RNU6-1329P | -0.438544193 | 0.880854386 | | down |
| RP11-214K3.18 | -0.438544193 | 0.880854386 | | down |
| uc_338 | -0.438544193 | 0.880854386 | | down |
| RN7SKP4 | -0.438544193 | 0.880854386 | | down |
| RP11-114N19.5 | -0.438544193 | 0.880854386 | | down |
| LA16c-395F10.1 | -0.438544193 | 0.880854386 | | down |
| CTD-2547E10.5 | -0.438544193 | 0.880854386 | | down |
| SHC1P2 | -0.438544193 | 0.880854386 | | down |
| RP11-672L10.5 | -0.438544193 | 0.880854386 | | down |
| RP11-484N16.1 | -0.438544193 | 0.880854386 | | down |
| RNU2-52P | -0.438544193 | 0.880854386 | | down |
| RP4-569M23.4 | -0.438544193 | 0.880854386 | | down |
| RN7SL672P | -0.438544193 | 0.880854386 | | down |
| SEPHS1P4 | -0.438544193 | 0.880854386 | | down |
| ZCCHC13 | -0.438544193 | 0.880854386 | | down |
| NLRP3P1 | -0.438544193 | 0.880854386 | | down |
| BNIP3P26 | -0.438543584 | 0.870434226 | | down |
| AC007879.1 | -0.438543555 | 0.86985532 | | down |
| SNX18P27 | -0.438543503 | 0.868821506 | | down |
| FMR1NB | -0.4385435 | 0.868764286 | | down |
| RP4-665J23.4 | -0.438543485 | 0.868466283 | | down |
| MTNR1B | -0.438543435 | 0.867431953 | | down |
| RNU6-20P | -0.43854343 | 0.867324832 | | down |
| RP11-685B14.3 | -0.438543397 | 0.866628809 | | down |
| NA | -0.438543383 | 0.866333764 | | down |
| RP11-155L15.1 | -0.43854335 | 0.865635005 | | down |
| RP11-400K9.1 | -0.438542072 | 0.824729508 | | down |
| RP11-114O4.1 | -0.438308925 | 0.558997723 | 0.678594189 | down |
| RNY4P10 | -0.438157171 | 0.260752616 | 0.395939129 | down |
| RPL23AP25 | -0.438111019 | 0.32604787 | 0.464477581 | down |
| PSMG3 | -0.437864636 | 0.036368367 | 0.096787954 | down |
| LINC00116 | -0.437815586 | 0.021016942 | 0.064405652 | down |
| C5 | -0.43777014 | 0.08714033 | 0.183307949 | down |
| GS1-166A23.1 | -0.437723866 | 0.62263166 | | down |
| FAM46B | -0.437663901 | 0.265111993 | 0.400735798 | down |
| RP4-673M15.1 | -0.437480233 | 0.293024384 | 0.430305714 | down |
| ZFYVE27 | -0.437373409 | 0.016537765 | 0.053708855 | down |
| FAM72C | -0.437242589 | 0.304498954 | 0.442591514 | down |
| CIPC | -0.437231978 | 0.012828339 | 0.044320909 | down |
| RNU6-216P | -0.437213529 | 0.654802337 | | down |
| RP11-12J10.4 | -0.437092712 | 0.369504027 | 0.507855131 | down |
| ENDOD1 | -0.436980186 | 0.046506331 | 0.115914735 | down |
| ZDHHC23 | -0.43694452 | 0.07345285 | 0.16194448 | down |
| KLRD1 | -0.436784086 | 0.16648922 | 0.289618558 | down |
| AC006445.6 | -0.436721818 | 0.798983371 | 0.866594094 | down |
| MDH2 | -0.436647992 | 0.01165475 | 0.041282163 | down |
| RMND5A | -0.436623343 | 0.012378719 | 0.043164604 | down |
| RP11-760D2.7 | -0.43661178 | 0.747063572 | | down |
| NA | -0.436371514 | 0.551327235 | 0.671453056 | down |
| CYSTM1 | -0.436246554 | 0.024865696 | 0.072998337 | down |

| | | | | |
|---|---|---|---|---|
| ADPGK | -0.436199895 | 0.04664639 | 0.116167451 | down |
| FBLL1 | -0.435995292 | 0.334478598 | 0.472741921 | down |
| TICAM1 | -0.435917194 | 0.008305862 | 0.03182519 | down |
| AC092638.1 | -0.435885442 | 0.858050732 | 0.908969225 | down |
| RP11-390F4.10 | -0.435790735 | 0.554726298 | 0.674608253 | down |
| GLIPR1L2 | -0.435498173 | 0.318203442 | 0.457119733 | down |
| RP11-401E14.2 | -0.435405928 | 0.881700706 | | down |
| RNGTTP1 | -0.435405928 | 0.881700706 | | down |
| RP11-166J5.1 | -0.435405309 | 0.856931473 | | down |
| RP11-265F19.1 | -0.435405045 | 0.840315582 | | down |
| RN7SL75P | -0.435404586 | 0.788997427 | | down |
| BMPR1APS2 | -0.435404568 | 0.785683219 | | down |
| LINC01187 | -0.435404566 | 0.785474625 | | down |
| AC008993.2 | -0.435404482 | 0.76843957 | | down |
| RP11-56B16.5 | -0.435404401 | 0.747691039 | | down |
| RP11-413G15.1 | -0.435404353 | 0.732163013 | | down |
| RBBP4P2 | -0.435404231 | 0.675167422 | | down |
| RP11-458F8.1 | -0.435232949 | 0.441609669 | 0.575256716 | down |
| NA | -0.435034756 | 0.460951897 | 0.592805794 | down |
| ACVRL1 | -0.434866492 | 0.055526595 | 0.131970652 | down |
| PAQR7 | -0.434845445 | 0.003508596 | 0.016412803 | down |
| PCDHGB3 | -0.434765465 | 0.559599059 | 0.679077233 | down |
| COQ8A | -0.434761503 | 0.00098759 | 0.005997805 | down |
| FAM187B | -0.434600981 | 0.540937819 | 0.662665949 | down |
| CLSTN2 | -0.434596345 | 0.344766824 | 0.483304146 | down |
| CCDC38 | -0.43456666 | 0.583738048 | | down |
| PCYT1A | -0.434522542 | 0.000102838 | 0.00096915 | down |
| RP11-1018J11.1 | -0.434483883 | 0.73368339 | | down |
| FALEC | -0.434421789 | 0.50995031 | 0.635926935 | down |
| RP11-500G10.5 | -0.434180328 | 0.361686067 | 0.500022833 | down |
| NA | -0.434057415 | 0.623447313 | | down |
| MRM1 | -0.433929735 | 0.032642903 | 0.08925404 | down |
| FARP2 | -0.433675392 | 0.009577932 | 0.035451447 | down |
| TMEM116 | -0.433604668 | 0.03477688 | 0.093636637 | down |
| FAM90A1 | -0.4335672 | 0.23989352 | 0.373631616 | down |
| NPY2R | -0.433564947 | 0.518192918 | 0.642984679 | down |
| HSPD1P10 | -0.433485864 | 0.653032853 | | down |
| RN7SL702P | -0.433482711 | 0.831018296 | 0.88960069 | down |
| AC013474.4 | -0.433409784 | 0.721291482 | | down |
| BRAT1 | -0.433378845 | 0.010556746 | 0.038235912 | down |
| RP11-561I11.4 | -0.43298336 | 0.757298785 | | down |
| SLC25A4 | -0.432965094 | 0.016280854 | 0.053092583 | down |
| HPS1 | -0.432880087 | 0.001939622 | 0.010223511 | down |
| HMGA1P4 | -0.432829225 | 0.230130562 | 0.3625042 | down |
| CTD-2263F21.1 | -0.432821889 | 0.58470696 | 0.700533776 | down |
| FAM13A | -0.43253445 | 0.060245728 | 0.140138019 | down |
| MRM3 | -0.432330697 | 0.036766712 | 0.097568112 | down |
| RP11-177H2.1 | -0.431943184 | 0.359290947 | 0.497581691 | down |
| RP11-353N4.5 | -0.431828814 | 0.725844464 | 0.813309629 | down |
| PCDH1 | -0.431597277 | 0.095576207 | 0.195659006 | down |
| PDZD4 | -0.431593857 | 0.081213313 | 0.174116944 | down |

| | | | | |
|---|---|---|---|---|
| RP1-182O16.1 | -0.43144359 | 0.564523394 | 0.682681567 | down |
| SGTA | -0.431437757 | 0.017018672 | 0.054885033 | down |
| ZNF576 | -0.431427718 | 0.002944285 | 0.014290482 | down |
| NA | -0.431366043 | 0.576003061 | 0.693021344 | down |
| RP11-352G18.2 | -0.431206496 | 0.465647657 | 0.597043794 | down |
| PDCD4 | -0.4310851 | 0.031105419 | 0.08612397 | down |
| RPS19BP1 | -0.430939539 | 0.038385198 | 0.1007905 | down |
| RP11-156P1.3 | -0.430752722 | 0.00691958 | 0.027759629 | down |
| VPS37B | -0.430742416 | 0.010561612 | 0.038248924 | down |
| RPS4XP3 | -0.430553497 | 0.339537985 | 0.478032149 | down |
| SERF1A | -0.430473786 | 0.564051978 | 0.682347892 | down |
| RASEF | -0.430472571 | 0.142707277 | 0.259747455 | down |
| CHMP4B | -0.430228443 | 0.016911575 | 0.054599294 | down |
| CTD-2373H9.3 | -0.430135695 | 0.541070357 | 0.662765954 | down |
| SEC24B-AS1 | -0.43010124 | 0.161461318 | 0.283184112 | down |
| ZNF879 | -0.429981043 | 0.098086003 | 0.199249213 | down |
| AC133965.1 | -0.429891116 | 0.77492337 | | down |
| MTMR8 | -0.429804438 | 0.179004758 | 0.304117787 | down |
| C1orf127 | -0.429766863 | 0.121688355 | 0.232382943 | down |
| KRT16P1 | -0.429528003 | 0.874370946 | | down |
| EI24 | -0.429419222 | 0.002941927 | 0.014281347 | down |
| NPAS2 | -0.4293938 | 0.034707528 | 0.093478527 | down |
| AMIGO1 | -0.429302917 | 0.04269026 | 0.10906345 | down |
| HSBP1L1 | -0.429201092 | 0.029072043 | 0.082036298 | down |
| RP1-118J21.24 | -0.429135143 | 0.8833922 | | down |
| OSTCP5 | -0.429135143 | 0.8833922 | | down |
| TMEM108-AS1 | -0.429135143 | 0.8833922 | | down |
| MIR8082 | -0.429135143 | 0.8833922 | | down |
| RNU2-61P | -0.429135143 | 0.8833922 | | down |
| NIP7P3 | -0.429135143 | 0.8833922 | | down |
| NA | -0.429135143 | 0.8833922 | | down |
| AC004987.10 | -0.429135143 | 0.8833922 | | down |
| OR2A13P | -0.429135143 | 0.8833922 | | down |
| KB-1639H6.2 | -0.429135143 | 0.8833922 | | down |
| RP11-574F11.3 | -0.429135143 | 0.8833922 | | down |
| RN7SKP225 | -0.429135143 | 0.8833922 | | down |
| MIR4481 | -0.429135143 | 0.8833922 | | down |
| 7SK | -0.429135143 | 0.8833922 | | down |
| OR4D5 | -0.429135143 | 0.8833922 | | down |
| RP11-843B15.1 | -0.429135143 | 0.8833922 | | down |
| RNU6-87P | -0.429135143 | 0.8833922 | | down |
| RP11-495K9.8 | -0.429135143 | 0.8833922 | | down |
| LINC01072 | -0.429135143 | 0.8833922 | | down |
| LINC00445 | -0.429135143 | 0.8833922 | | down |
| TRAJ52 | -0.429135143 | 0.8833922 | | down |
| SNORD56B | -0.429135143 | 0.8833922 | | down |
| CTD-3051D23.3 | -0.429135143 | 0.8833922 | | down |
| RNU6-667P | -0.429135143 | 0.8833922 | | down |
| OR7G1 | -0.429135143 | 0.8833922 | | down |
| CTD-2050I18.1 | -0.429135143 | 0.8833922 | | down |
| HMGB3P1 | -0.429135143 | 0.8833922 | | down |

| | | | | |
|---|---|---|---|---|
| AP001172.2 | -0.429135143 | 0.8833922 | | down |
| PAGE3 | -0.429135143 | 0.8833922 | | down |
| NA | -0.429135034 | 0.881659307 | | down |
| PCGF7P | -0.429134303 | 0.867728379 | | down |
| RP3-354J5.3 | -0.429134292 | 0.867474875 | | down |
| TRAJ55 | -0.429134278 | 0.867153577 | | down |
| RP11-506E9.3 | -0.429134268 | 0.866927115 | | down |
| HOXC-AS3 | -0.429134219 | 0.865785065 | | down |
| ZDHHC4P1 | -0.429133638 | 0.849563928 | | down |
| RP11-58A18.1 | -0.429133605 | 0.848452158 | | down |
| DPYD-IT1 | -0.429133579 | 0.847592514 | | down |
| NA | -0.429133112 | 0.82832926 | | down |
| SETP11 | -0.429133067 | 0.82609221 | | down |
| RP11-20I23.11 | -0.429132794 | 0.810147002 | | down |
| RP11-292F9.1 | -0.429132556 | 0.791912696 | | down |
| RP4-583P15.16 | -0.429050502 | 0.315776286 | 0.454376476 | down |
| KIAA1211L | -0.428921017 | 0.039667703 | 0.103313785 | down |
| ZNF888 | -0.428664888 | 0.060073765 | 0.139816683 | down |
| PRKCA | -0.428662426 | 0.030785879 | 0.085441769 | down |
| PAGE2B | -0.428654006 | 0.473621589 | 0.603993677 | down |
| PNKP | -0.428619631 | 0.032556156 | 0.089081709 | down |
| NA | -0.428587718 | 0.7532921 | | down |
| TMSB10P1 | -0.428559919 | 0.36951515 | 0.507855131 | down |
| AL022341.3 | -0.428490593 | 0.307229447 | 0.445525702 | down |
| MRPL52 | -0.428475417 | 0.046732362 | 0.116323703 | down |
| RP11-887P2.5 | -0.428444908 | 0.524755681 | 0.648823395 | down |
| PCDHB12 | -0.428394356 | 0.369507639 | 0.507855131 | down |
| LRPPRC | -0.428209001 | 9.59E-05 | 0.000913474 | down |
| RP11-295D4.1 | -0.427939557 | 0.2565916 | 0.391604413 | down |
| MATN2 | -0.427863463 | 0.111743921 | 0.21886918 | down |
| CHRNA9 | -0.427770049 | 0.645604994 | | down |
| TMEM191B | -0.427726448 | 0.242368651 | 0.376258138 | down |
| ASB2 | -0.427546459 | 0.046127208 | 0.11525369 | down |
| LRSAM1 | -0.427531267 | 0.008165731 | 0.031424349 | down |
| PGAP1 | -0.427510962 | 0.115570496 | 0.224070727 | down |
| STX8 | -0.426963126 | 0.031779122 | 0.087494157 | down |
| C14orf93 | -0.426918328 | 0.000224247 | 0.001839277 | down |
| CTD-2366F13.2 | -0.426863691 | 0.313985599 | 0.452614113 | down |
| MUTYH | -0.426810071 | 0.355819345 | 0.494391916 | down |
| SORBS3 | -0.426750581 | 0.014347979 | 0.048293826 | down |
| ARNT2 | -0.426652123 | 0.134626982 | 0.249151967 | down |
| HEMK1 | -0.426618277 | 0.013129173 | 0.045054584 | down |
| SLC17A6 | -0.426571594 | 0.876602104 | | down |
| CTD-2527I21.5 | -0.426555454 | 0.826375861 | | down |
| RP11-13J12.2 | -0.426548452 | 0.808327924 | | down |
| RP11-57H14.3 | -0.426540556 | 0.789839632 | | down |
| NA | -0.42653288 | 0.773366566 | | down |
| RP11-712C19.1 | -0.426525441 | 0.758548277 | | down |
| RPS18P13 | -0.42652297 | 0.753841215 | | down |
| NA | -0.426515075 | 0.739433482 | | down |
| PDE6H | -0.426513594 | 0.736829257 | | down |

| | | | | |
|---|---|---|---|---|
| ZNF337 | -0.426510546 | 0.391895534 | 0.529459008 | down |
| RP11-70D24.3 | -0.426502687 | 0.718492989 | | down |
| RP11-90K6.1 | -0.426481527 | 0.68642814 | | down |
| PCDHB6 | -0.426469406 | 0.440520413 | 0.574236172 | down |
| CTD-2622I13.3 | -0.426452057 | 0.358911672 | 0.497193944 | down |
| SNORD91B | -0.426381423 | 0.644902917 | 0.749946775 | down |
| EPHX3 | -0.426381243 | 0.267980906 | 0.403772234 | down |
| EMC3 | -0.426322984 | 0.014888535 | 0.049663277 | down |
| NA | -0.426303979 | 0.340603851 | 0.478988115 | down |
| TBC1D13 | -0.426138201 | 0.000538283 | 0.003695651 | down |
| CELF2-AS1 | -0.426105282 | 0.547053104 | 0.66792634 | down |
| NA | -0.425880864 | 0.467958342 | 0.599009008 | down |
| PDZRN4 | -0.425872125 | 0.40080079 | 0.537718092 | down |
| VPREB3 | -0.425816281 | 0.355312268 | 0.49389293 | down |
| AC005537.2 | -0.425706603 | 0.57169483 | 0.6891626 | down |
| TOMM40L | -0.425669621 | 0.046941891 | 0.116729199 | down |
| RP11-370A5.2 | -0.425624119 | 0.600767588 | | down |
| AKR1E2 | -0.425544092 | 0.209388552 | 0.339627984 | down |
| AC020571.3 | -0.425346136 | 0.297954258 | 0.435416981 | down |
| CERS1 | -0.425174503 | 0.306326518 | 0.444710023 | down |
| BLCAP | -0.42461398 | 5.67E-06 | 8.80E-05 | down |
| CTD-2547G23.4 | -0.424465016 | 0.242288675 | 0.376238334 | down |
| GDE1 | -0.424450953 | 0.0109798 | 0.039421262 | down |
| IQCE | -0.424405302 | 0.001246041 | 0.007222853 | down |
| LTB | -0.424396867 | 0.271608839 | 0.407745763 | down |
| OR52N4 | -0.424162338 | 0.393489817 | 0.530896716 | down |
| C11orf1 | -0.424153269 | 0.008618579 | 0.032706401 | down |
| SMIM8 | -0.424026966 | 0.020579437 | 0.063400481 | down |
| MSS51 | -0.4239788 | 0.189913144 | 0.317215238 | down |
| LRRC37A4P | -0.423974914 | 0.240447208 | 0.374222748 | down |
| SYNE4 | -0.423935846 | 0.130496883 | 0.243988323 | down |
| CTD-2116N20.1 | -0.423898541 | 0.383989325 | 0.521686501 | down |
| C20orf85 | -0.423766493 | 0.829463214 | | down |
| DGKZ | -0.423735978 | 0.009360806 | 0.034866856 | down |
| POLA2 | -0.423677781 | 0.140514791 | 0.257024437 | down |
| RP5-1142A6.8 | -0.423615017 | 0.555889516 | 0.675749328 | down |
| RP11-379F4.4 | -0.423486895 | 0.170043917 | 0.293811793 | down |
| GOLM1 | -0.423393331 | 0.117294727 | 0.226372058 | down |
| RP11-241K18.2 | -0.423390359 | 0.762326067 | | down |
| STK32B | -0.423310086 | 0.152006811 | 0.271386536 | down |
| CH17-11806.3 | -0.42325054 | 0.753789358 | | down |
| LIMA1 | -0.423195553 | 0.058163311 | 0.136648305 | down |
| NPM1P46 | -0.423157212 | 0.438762471 | 0.572938964 | down |
| RP11-244J10.1 | -0.423019189 | 0.364827801 | 0.503242992 | down |
| PTCHD1 | -0.422950968 | 0.362608444 | 0.501021343 | down |
| EFCAB1 | -0.422882449 | 0.366702656 | 0.505191425 | down |
| PLXNB2 | -0.422795145 | 0.011278362 | 0.040219242 | down |
| SMIM12 | -0.422762992 | 0.005455007 | 0.023191751 | down |
| AC142528.1 | -0.422747111 | 0.446650461 | 0.579937182 | down |
| OR52B3P | -0.422732653 | 0.528490489 | 0.651858455 | down |
| NA | -0.422640123 | 0.548898635 | 0.669472276 | down |

| | | | | |
|---|---|---|---|---|
| TOB1 | -0.42252491 | 0.070712203 | 0.157502352 | down |
| SLC25A5-AS1 | -0.422521648 | 0.096820595 | 0.197546891 | down |
| TMEM62 | -0.422512388 | 0.000311028 | 0.002401573 | down |
| NA | -0.422430652 | 0.273775351 | 0.410076111 | down |
| RNF25 | -0.422375483 | 0.039383616 | 0.102738678 | down |
| NBEAL2 | -0.422352763 | 0.054239902 | 0.129723262 | down |
| DDAH1 | -0.422202575 | 0.051180991 | 0.124438107 | down |
| TEX21P | -0.422164383 | 0.377565591 | 0.515687622 | down |
| ETV4 | -0.422145354 | 0.129125193 | 0.24208657 | down |
| RP11-482D24.2 | -0.422124347 | 0.756861566 | | down |
| IGFALS | -0.422115705 | 0.341524894 | 0.479856611 | down |
| CYP51A1P2 | -0.422101499 | 0.63446557 | 0.741765258 | down |
| MOV10 | -0.421961518 | 0.016946587 | 0.054687784 | down |
| ZSCAN31 | -0.421957359 | 0.111110493 | 0.217941088 | down |
| SWI5 | -0.421860759 | 0.02266603 | 0.068104632 | down |
| RNVU1-15 | -0.421846602 | 0.447768983 | 0.581050505 | down |
| RPS26 | -0.421755477 | 0.107123933 | 0.21236763 | down |
| EEPD1 | -0.421703879 | 0.004886117 | 0.021309116 | down |
| NA | -0.421641187 | 0.662392844 | 0.763863302 | down |
| RP11-12M5.4 | -0.421547832 | 0.707913773 | | down |
| GOLGA6L9 | -0.421544289 | 0.077021054 | 0.167524836 | down |
| ZNF44 | -0.421340326 | 0.001164992 | 0.006846833 | down |
| AC026150.8 | -0.421204515 | 0.639692484 | | down |
| RHPN1 | -0.421129707 | 0.113764987 | 0.221484919 | down |
| RP11-296O14.3 | -0.420944427 | 0.129608952 | 0.242675518 | down |
| TMEM234 | -0.420745636 | 0.059281976 | 0.138485941 | down |
| NA | -0.420630341 | 0.48768217 | 0.616404001 | down |
| RP11-761N21.2 | -0.420589097 | 0.109082032 | 0.215207556 | down |
| RP11-313P13.5 | -0.420564666 | 0.489279193 | 0.617902652 | down |
| SLC8B1 | -0.420473044 | 0.001410371 | 0.007943981 | down |
| CCDC57 | -0.420443724 | 0.028125997 | 0.08008885 | down |
| HSPA9P1 | -0.420343716 | 0.592926256 | | down |
| RP11-209D14.4 | -0.420333958 | 0.578588159 | | down |
| SCAI | -0.420316259 | 0.037658975 | 0.099335053 | down |
| CHST8 | -0.420251277 | 0.277095787 | 0.41329891 | down |
| RPRM | -0.420200489 | 0.516056156 | 0.641127699 | down |
| PDIA3P2 | -0.420163053 | 0.623562464 | | down |
| AC034243.1 | -0.419869478 | 0.652634933 | 0.756064005 | down |
| SYTL4 | -0.419841604 | 0.190344236 | 0.317823395 | down |
| ME3 | -0.41981674 | 0.018044915 | 0.057497179 | down |
| RNU6-501P | -0.419726093 | 0.88593121 | | down |
| RN7SKP276 | -0.419726093 | 0.88593121 | | down |
| AK4P6 | -0.419726093 | 0.88593121 | | down |
| RP11-485C11.1 | -0.419726093 | 0.88593121 | | down |
| NA | -0.419726093 | 0.88593121 | | down |
| RP11-53O19.2 | -0.419726093 | 0.88593121 | | down |
| RP11-269M20.2 | -0.419726093 | 0.88593121 | | down |
| RN7SL332P | -0.419726093 | 0.88593121 | | down |
| LY6G6F | -0.419726093 | 0.88593121 | | down |
| RN7SL652P | -0.419726093 | 0.88593121 | | down |
| OR4A44P | -0.419726093 | 0.88593121 | | down |

| | | | | |
|---|---|---|---|---|
| TAS2R13 | -0.419726093 | 0.88593121 | | down |
| RP11-813P10.1 | -0.419726093 | 0.88593121 | | down |
| TRAJ54 | -0.419726093 | 0.88593121 | | down |
| RP11-65J21.1 | -0.419726093 | 0.88593121 | | down |
| CTD-3076M17.1 | -0.419726093 | 0.88593121 | | down |
| RP11-354I13.2 | -0.419726093 | 0.88593121 | | down |
| RP11-728E14.3 | -0.419726093 | 0.88593121 | | down |
| SLC25A51P2 | -0.419726093 | 0.88593121 | | down |
| RN7SL50P | -0.419726093 | 0.88593121 | | down |
| RP11-15F12.3 | -0.419726093 | 0.88593121 | | down |
| ERVK-28 | -0.419726093 | 0.88593121 | | down |
| NA | -0.419726082 | 0.885742709 | | down |
| MIR4641 | -0.419726032 | 0.884945621 | | down |
| RP11-323I15.5 | -0.419725258 | 0.869828709 | | down |
| PRSS58 | -0.419725243 | 0.869480549 | | down |
| MIR6793 | -0.419725199 | 0.868412493 | | down |
| RNU6-869P | -0.419725175 | 0.867842039 | | down |
| NPM1P52 | -0.419725174 | 0.867814274 | | down |
| RP11-691H4.3 | -0.419725169 | 0.867698139 | | down |
| RP1-38C16.2 | -0.419725118 | 0.866399046 | | down |
| ZNF30-AS1 | -0.419724673 | 0.853391441 | | down |
| RNU7-13P | -0.419724641 | 0.852325664 | | down |
| RP11-214K3.5 | -0.419724603 | 0.850979496 | | down |
| ALDH1L1-AS1 | -0.419724592 | 0.850592138 | | down |
| AC005019.2 | -0.419724564 | 0.849581271 | | down |
| CTD-2308G16.1 | -0.419724562 | 0.849539268 | | down |
| RP11-220I1.4 | -0.41972456 | 0.849457255 | | down |
| NA | -0.419724088 | 0.828614711 | | down |
| NA | -0.41972362 | 0.796126366 | | down |
| RP11-214D15.2 | -0.419723576 | 0.792052656 | | down |
| AC138517.6 | -0.419545068 | 0.182931468 | 0.308711622 | down |
| ZNF268 | -0.419536239 | 0.000374603 | 0.002774793 | down |
| SEC14L6 | -0.419371347 | 0.246163403 | 0.380262907 | down |
| RP11-666A8.7 | -0.419349092 | 0.276300359 | 0.412587471 | down |
| ATP5E | -0.419335482 | 0.029256745 | 0.082387257 | down |
| RP11-328J6.1 | -0.419253541 | 0.644356458 | | down |
| RP11-342K2.1 | -0.419110329 | 0.134005779 | 0.248292916 | down |
| PKD1L1 | -0.419107692 | 0.236346786 | 0.369352553 | down |
| NA | -0.419039192 | 0.22534883 | 0.357426896 | down |
| TYMSOS | -0.418979875 | 0.211114944 | 0.341631875 | down |
| TYSND1 | -0.418572979 | 0.000790351 | 0.005025461 | down |
| RP11-463M14.1 | -0.418521053 | 0.878537283 | | down |
| ZNF252P | -0.41851304 | 0.001184188 | 0.006942 | down |
| NA | -0.418506847 | 0.863166918 | | down |
| NA | -0.418470546 | 0.830032732 | | down |
| RP11-390F4.2 | -0.418445301 | 0.810472155 | | down |
| OR5BK1P | -0.418399609 | 0.779726139 | | down |
| ARMC4P1 | -0.418396045 | 0.777523553 | | down |
| RPL7P57 | -0.418340571 | 0.745974073 | | down |
| SNHG5 | -0.418337657 | 0.042079892 | 0.107944697 | down |
| FOXL2 | -0.418217974 | 0.68892651 | | down |

| | | | | |
|---|---|---|---|---|
| DNAJB2 | -0.418205956 | 0.028104582 | 0.080058223 | down |
| PVRL3-AS1 | -0.418078574 | 0.398863636 | 0.535789103 | down |
| SMIM1 | -0.418047229 | 0.140865737 | 0.257433595 | down |
| HIST1H2AL | -0.417940768 | 0.446218617 | 0.579510167 | down |
| CTB-134H23.2 | -0.417900208 | 0.587427027 | | down |
| RP11-586D5.3 | -0.417741151 | 0.284557499 | 0.421378493 | down |
| AC005083.1 | -0.417659205 | 0.193012332 | 0.321053177 | down |
| KIF13B | -0.417654083 | 0.056457459 | 0.133685995 | down |
| RP11-996F15.2 | -0.417489578 | 0.179368928 | 0.304518237 | down |
| HRASLS5 | -0.417451105 | 0.198019765 | 0.326741008 | down |
| RP4-633O19__A.1 | -0.41736472 | 0.390716819 | 0.528382474 | down |
| LETMD1 | -0.417287447 | 0.001658723 | 0.009031295 | down |
| MPC2 | -0.417265808 | 0.001377452 | 0.007800695 | down |
| S100A13 | -0.417184828 | 0.191337898 | 0.319080584 | down |
| GNB2 | -0.417096289 | 0.01168543 | 0.041356705 | down |
| HDHD2 | -0.417011085 | 0.386551223 | 0.524218874 | down |
| TMEM222 | -0.416980651 | 0.022200577 | 0.067048062 | down |
| ZFP30 | -0.416958923 | 0.08618245 | 0.181857241 | down |
| TMEM92-AS1 | -0.416852887 | 0.359200353 | 0.497502094 | down |
| L2HGDH | -0.416558421 | 0.038051334 | 0.10010633 | down |
| IL7 | -0.416510505 | 0.150173225 | 0.269200738 | down |
| RP11-98I9.4 | -0.416475351 | 0.081023909 | 0.173815232 | down |
| TEX43 | -0.4164642 | 0.531897939 | 0.655093125 | down |
| PPM1A | -0.416388389 | 0.023635735 | 0.070245527 | down |
| INO80C | -0.416249957 | 0.002526163 | 0.012676806 | down |
| RAP1GAP2 | -0.416226548 | 0.017777744 | 0.056857591 | down |
| FAM135A | -0.416120049 | 0.023415585 | 0.069749935 | down |
| CTSA | -0.416012807 | 0.057221752 | 0.134974068 | down |
| C9orf69 | -0.415795391 | 0.013153751 | 0.045128617 | down |
| EPHB1 | -0.415575205 | 0.266578843 | 0.40226493 | down |
| CTD-2555C10.3 | -0.415546082 | 0.296754034 | 0.434127584 | down |
| LBX2 | -0.41534864 | 0.220178199 | 0.351844786 | down |
| RP11-867G2.6 | -0.415342097 | 0.78596321 | | down |
| CST6 | -0.415293998 | 0.353658688 | 0.492414572 | down |
| SPTSSB | -0.415200189 | 0.346352575 | 0.485006607 | down |
| MTND5P25 | -0.415057361 | 0.727694043 | | down |
| RP11-15A1.7 | -0.414780837 | 0.429571086 | 0.564889644 | down |
| XXbac-B135H6.18 | -0.414693878 | 0.275780141 | 0.412051847 | down |
| RP11-359G22.4 | -0.41462627 | 0.84959348 | | down |
| UBE2Q2P2 | -0.41453071 | 0.167155517 | 0.290346211 | down |
| AC016739.2 | -0.414516641 | 0.102905641 | 0.206140623 | down |
| NA | -0.414513724 | 0.529444845 | 0.652711504 | down |
| CACNA1H | -0.414493155 | 0.184400473 | 0.310406085 | down |
| MIR8075 | -0.41434655 | 0.711729887 | | down |
| FAM66D | -0.414150049 | 0.286891556 | 0.424056376 | down |
| CTD-2145A24.5 | -0.414131351 | 0.493206089 | 0.621294916 | down |
| SDHAF1 | -0.414105986 | 0.010159115 | 0.037117894 | down |
| RP11-144F15.1 | -0.414078683 | 0.739530369 | | down |
| FMO3 | -0.414021329 | 0.209980743 | 0.340401525 | down |
| GLTP | -0.414003108 | 5.92E-05 | 0.000613495 | down |
| SMIM19 | -0.413816523 | 0.000221398 | 0.001818888 | down |

| | | | | |
|---|---|---|---|---|
| RP11-557H15.4 | -0.413804463 | 0.25337118 | 0.388318277 | down |
| RP11-361D15.2 | -0.413769033 | 0.616487464 | 0.727341424 | down |
| SUCLA2 | -0.413722635 | 0.040968488 | 0.105743751 | down |
| RERG-IT1 | -0.413711783 | 0.749401423 | | down |
| SGPP2 | -0.413620078 | 0.086151522 | 0.181841811 | down |
| KB-1460A1.1 | -0.413573991 | 0.525346476 | 0.649180258 | down |
| RP11-195B17.1 | -0.413537908 | 0.375367992 | 0.513713659 | down |
| GPR20 | -0.413476331 | 0.519040735 | 0.643638362 | down |
| RP11-465B22.3 | -0.413367452 | 0.236441504 | 0.369462139 | down |
| AKT1S1 | -0.413318635 | 0.01387846 | 0.047066763 | down |
| EXOC4 | -0.413235597 | 0.008193419 | 0.031498793 | down |
| RBL1 | -0.413215712 | 0.04474141 | 0.112740997 | down |
| LINC00184 | -0.413209634 | 0.64205787 | | down |
| SCN7A | -0.413156835 | 0.276524753 | 0.412807444 | down |
| IGFL2 | -0.412984948 | 0.392679484 | 0.530112892 | down |
| ADCK2 | -0.412978525 | 0.011884539 | 0.041888679 | down |
| RP11-97N19.3 | -0.412943902 | 0.817983243 | | down |
| PLCE1-AS2 | -0.41293321 | 0.799543242 | | down |
| SNX21 | -0.412747616 | 0.051854506 | 0.125486651 | down |
| FLOT2 | -0.412726405 | 0.035831047 | 0.095681092 | down |
| RP11-307L3.2 | -0.412568039 | 0.738950925 | | down |
| CTD-2623N2.3 | -0.412460788 | 0.235358525 | 0.368155141 | down |
| TOB1-AS1 | -0.412442889 | 0.151242934 | 0.270376217 | down |
| ATP9B | -0.412404701 | 0.013411803 | 0.045834081 | down |
| RP11-211G23.2 | -0.412241312 | 0.439648556 | 0.573564746 | down |
| RP11-472K17.3 | -0.412219344 | 0.505752884 | 0.632140277 | down |
| SNRPD2 | -0.412198999 | 0.067645368 | 0.15242912 | down |
| CLCNKA | -0.412013862 | 0.471501893 | 0.602082747 | down |
| FGFBP3 | -0.411916841 | 0.064615882 | 0.147393483 | down |
| PSD3 | -0.411845027 | 0.09912445 | 0.20084266 | down |
| RP11-635N19.2 | -0.411694794 | 0.749321917 | | down |
| CAP2 | -0.411668684 | 0.402195768 | 0.538915782 | down |
| AC005796.2 | -0.41143359 | 0.525044914 | 0.648957296 | down |
| RP11-2C24.7 | -0.411381328 | 0.519041081 | 0.643638362 | down |
| KRT223P | -0.411296928 | 0.660952684 | 0.762700202 | down |
| AC078852.2 | -0.411254923 | 0.582763324 | 0.698692737 | down |
| RP11-219E7.4 | -0.411174759 | 0.703913157 | | down |
| FOXP2 | -0.411142605 | 0.082769107 | 0.176676025 | down |
| RP11-485G7.6 | -0.410862224 | 0.527308697 | 0.6508283 | down |
| LILRA5 | -0.410859487 | 0.303892566 | 0.441881146 | down |
| RPL26 | -0.41083537 | 0.071868819 | 0.159364292 | down |
| RP11-366L5.1 | -0.410672936 | 0.271393864 | 0.407545222 | down |
| TMEM245 | -0.410600976 | 0.015571839 | 0.051416223 | down |
| RASL11B | -0.410530845 | 0.716603909 | | down |
| MTCH2 | -0.41049232 | 0.004447095 | 0.019817344 | down |
| NA | -0.410364067 | 0.473257239 | 0.603682779 | down |
| RP11-339D23.1 | -0.410317044 | 0.888471393 | | down |
| RP11-310N16.1 | -0.410317044 | 0.888471393 | | down |
| KCNIP4-IT1 | -0.410317044 | 0.888471393 | | down |
| RP11-254A17.1 | -0.410317044 | 0.888471393 | | down |
| RNU4-35P | -0.410317044 | 0.888471393 | | down |

| | | | | |
|---|---|---|---|---|
| SNORD114-19 | -0.410317044 | 0.888471393 | | down |
| RP5-1012F16.2 | -0.410317044 | 0.888471393 | | down |
| RP11-408I18.9 | -0.410316979 | 0.887414292 | | down |
| AC010084.1 | -0.410316942 | 0.886779156 | | down |
| HSP90AA6P | -0.410316938 | 0.886710705 | | down |
| CTD-2503O16.3 | -0.410316917 | 0.886360071 | | down |
| MTND4P4 | -0.410316799 | 0.884260777 | | down |
| LINC00161 | -0.410316181 | 0.871093193 | | down |
| KAT14 | -0.410316165 | 0.001470628 | 0.008210583 | down |
| IGLV3-2 | -0.410316156 | 0.870461181 | | down |
| AC002115.9 | -0.410316125 | 0.869648371 | | down |
| NA | -0.410316091 | 0.868752236 | | down |
| METTL21AP1 | -0.410315599 | 0.853405043 | | down |
| RP11-889D3.2 | -0.410315564 | 0.852073186 | | down |
| SNORA70B | -0.410315542 | 0.851249275 | | down |
| LINC01598 | -0.410315518 | 0.85031561 | | down |
| RP11-90H3.2 | -0.410315174 | 0.834600899 | | down |
| RNU6V | -0.410314886 | 0.816851293 | | down |
| RP11-295B17.6 | -0.410314843 | 0.813685538 | | down |
| RP11-792A8.4 | -0.410314643 | 0.796298607 | | down |
| NA | -0.410235104 | 0.679351584 | 0.777634567 | down |
| MAT1A | -0.40996416 | 0.446436001 | 0.579708831 | down |
| INMT | -0.40994685 | 0.125620918 | 0.237456407 | down |
| RP11-848P1.2 | -0.409903568 | 0.167884483 | 0.291202668 | down |
| CCDC122 | -0.409887549 | 0.060629232 | 0.140866496 | down |
| FAU | -0.40984329 | 0.096169911 | 0.196566352 | down |
| ACAT1 | -0.409838332 | 0.012717569 | 0.044064888 | down |
| HNRNPA1P39 | -0.409699368 | 0.584911432 | | down |
| NA | -0.409671737 | 0.28485975 | 0.421779425 | down |
| CCDC114 | -0.409649814 | 0.32162975 | 0.460385294 | down |
| RP1-102K2.8 | -0.409384011 | 0.38617927 | 0.523832678 | down |
| AC012123.1 | -0.409165106 | 0.495423115 | 0.62332979 | down |
| RP3-395M20.7 | -0.408991849 | 0.465923094 | 0.59729448 | down |
| CCDC183-AS1 | -0.408976435 | 0.149441552 | 0.268353475 | down |
| FDCSP | -0.408958611 | 0.445136807 | 0.578496852 | down |
| PRKAA2 | -0.408891649 | 0.315468247 | 0.454125937 | down |
| CTD-2323K18.3 | -0.408776088 | 0.638465742 | | down |
| CTD-2287O16.4 | -0.408534667 | 0.573020867 | | down |
| SMIM4 | -0.408468552 | 0.053684994 | 0.12876291 | down |
| RP11-130C6.1 | -0.408397491 | 0.73308816 | | down |
| PRICKLE1 | -0.408351601 | 0.128650822 | 0.241517599 | down |
| ARL14EPL | -0.408344674 | 0.596795888 | 0.710780259 | down |
| RP5-1101C3.1 | -0.408212366 | 0.539238126 | 0.661152067 | down |
| FARS2 | -0.408205595 | 0.007227926 | 0.028648524 | down |
| C2orf40 | -0.408169214 | 0.330435879 | 0.468813352 | down |
| CARMN | -0.408141985 | 0.224422164 | 0.356634904 | down |
| NAPSB | -0.408101885 | 0.259725132 | 0.39495783 | down |
| ASGR2 | -0.40795387 | 0.210604149 | 0.341191213 | down |
| CERS6 | -0.407730475 | 0.15320728 | 0.272856847 | down |
| AC140912.1 | -0.407712987 | 0.68385886 | 0.781154775 | down |
| TPCN1 | -0.407623741 | 0.001375562 | 0.00779321 | down |

| | | | | |
|---|---|---|---|---|
| S100A16 | -0.407556345 | 0.112435349 | 0.219606389 | down |
| Metazoa_SRP | -0.407512964 | 0.505007243 | 0.631658518 | down |
| NA | -0.407500933 | 0.153947058 | 0.273647226 | down |
| KIAA1328 | -0.407471945 | 0.037496982 | 0.09901207 | down |
| RP11-666A8.11 | -0.407408636 | 0.866721351 | | down |
| Y_RNA | -0.40719992 | 0.679014413 | 0.777361926 | down |
| RNU4-78P | -0.407122665 | 0.735334125 | | down |
| RNU5B-3P | -0.407032439 | 0.633563387 | | down |
| AC079250.1 | -0.406980808 | 0.207021647 | 0.337096003 | down |
| FAM162A | -0.406902175 | 0.047641894 | 0.118069213 | down |
| XACT | -0.406827924 | 0.62490414 | 0.734330191 | down |
| NXPE2 | -0.406760687 | 0.466749812 | 0.597922115 | down |
| RP11-517H2.6 | -0.406745023 | 0.773853753 | | down |
| VWA5A | -0.406708577 | 0.017734675 | 0.056761541 | down |
| RP11-64D24.2 | -0.406566589 | 0.769752948 | | down |
| PLIN4 | -0.406515541 | 0.409795671 | 0.546564022 | down |
| AC006014.8 | -0.406292387 | 0.675979969 | | down |
| GUF1 | -0.406165666 | 3.54E-06 | 5.96E-05 | down |
| MED19 | -0.406022868 | 0.004401265 | 0.019653924 | down |
| KCNMA1 | -0.406013962 | 0.090392666 | 0.188123944 | down |
| NA | -0.405696694 | 0.57418255 | 0.691318371 | down |
| RP11-1102P22.1 | -0.40564151 | 0.310848614 | 0.44927771 | down |
| RALY | -0.40558234 | 0.009420732 | 0.035033604 | down |
| FZD3 | -0.405534679 | 0.063665139 | 0.145922753 | down |
| AC093627.9 | -0.40552619 | 0.111609721 | 0.218743122 | down |
| GSTK1 | -0.405396488 | 0.012019132 | 0.042249071 | down |
| RLN2 | -0.405337155 | 0.380447969 | 0.518375455 | down |
| CTD-2282P23.2 | -0.405220345 | 0.668259514 | 0.768475421 | down |
| RP11-153M7.1 | -0.405110563 | 0.670022997 | | down |
| SERPIND1 | -0.405039506 | 0.551521489 | 0.67155933 | down |
| RP11-96A15.1 | -0.404888838 | 0.706532578 | | down |
| CERS4 | -0.404635436 | 0.11812608 | 0.227552909 | down |
| RP11-496H15.2 | -0.404616929 | 0.814162361 | | down |
| RP11-166P13.3 | -0.404485125 | 0.039294467 | 0.102559393 | down |
| POLR1C | -0.404474757 | 0.018658264 | 0.05892042 | down |
| RANBP3L | -0.404449181 | 0.54635222 | 0.667586063 | down |
| IGHM | -0.404161511 | 0.233367151 | 0.366099637 | down |
| RPLP1 | -0.404145495 | 0.050910361 | 0.123910474 | down |
| NA | -0.404089221 | 0.501790799 | 0.628774932 | down |
| RP11-4C20.3 | -0.403986496 | 0.631302418 | | down |
| SIM1 | -0.403969529 | 0.770701355 | | down |
| NA | -0.403957738 | 0.593741454 | 0.708230922 | down |
| SLC25A24 | -0.403927323 | 0.121280428 | 0.23179066 | down |
| KLHL25 | -0.403854205 | 0.007441118 | 0.029321518 | down |
| NSDHL | -0.403807761 | 0.016243476 | 0.053016729 | down |
| RP11-27K13.3 | -0.403807733 | 0.323205533 | 0.461761712 | down |
| NFS1 | -0.403732016 | 0.002525299 | 0.012674588 | down |
| MGC16275 | -0.403615284 | 0.333572083 | 0.471779451 | down |
| RP11-973D8.5 | -0.403199819 | 0.422738734 | 0.558275275 | down |
| RP11-365F18.1 | -0.403140082 | 0.720902672 | | down |
| RP11-351I21.11 | -0.403126018 | 0.508685892 | 0.63502731 | down |

| | | | | |
|---|---|---|---|---|
| NLRP2 | -0.403019771 | 0.1571792 | 0.277847251 | down |
| LPAR2 | -0.402933705 | 0.109983525 | 0.216401998 | down |
| C6orf3 | -0.402921008 | 0.189130351 | 0.316241778 | down |
| CTD-3126B10.4 | -0.402877808 | 0.571387618 | 0.688875184 | down |
| FAM102A | -0.402870853 | 0.067927917 | 0.152939625 | down |
| MRGPRE | -0.402858831 | 0.478702786 | 0.608304635 | down |
| RBM10 | -0.402797558 | 0.004462728 | 0.019872271 | down |
| RP11-255G12.3 | -0.402766896 | 0.756466417 | | down |
| RP11-527L4.5 | -0.402762799 | 0.583324848 | 0.699170658 | down |
| UBA52 | -0.40275669 | 0.044939546 | 0.113135892 | down |
| RP11-43A14.1 | -0.402623046 | 0.534797472 | 0.657478323 | down |
| RP4-803A2.2 | -0.402486229 | 0.599523469 | 0.712898089 | down |
| NA | -0.402348379 | 0.442035652 | 0.575587012 | down |
| ALDH7A1 | -0.402139702 | 0.016394484 | 0.05337045 | down |
| RP11-348F1.3 | -0.402074974 | 0.551126552 | 0.671371939 | down |
| TIMM17B | -0.401942185 | 0.063796705 | 0.146114475 | down |
| PPP4C | -0.401926245 | 0.042619243 | 0.108942808 | down |
| OXCT2 | -0.401924637 | 0.260066382 | 0.395150778 | down |
| NA | -0.40163088 | 0.428182203 | 0.563547257 | down |
| OSER1-AS1 | -0.401377151 | 0.015991678 | 0.052411254 | down |
| RPSAP61 | -0.401180748 | 0.336161549 | 0.474354015 | down |
| RP3-426I6.2 | -0.401177512 | 0.525930761 | 0.649581245 | down |
| NDUFA2 | -0.400958879 | 0.025745584 | 0.074900777 | down |
| RP13-130D24.1 | -0.40092102 | 0.829853369 | | down |
| RPSAP22 | -0.400907994 | 0.891012721 | | down |
| CTD-2292M14.1 | -0.400907994 | 0.891012721 | | down |
| RNU7-30P | -0.400907994 | 0.891012721 | | down |
| NA | -0.400907994 | 0.891012721 | | down |
| RP1-107N3.1 | -0.400907994 | 0.891012721 | | down |
| RP11-565N2.2 | -0.400907881 | 0.889069867 | | down |
| RPL23AP40 | -0.400907867 | 0.888827421 | | down |
| FABP9 | -0.400907821 | 0.888008536 | | down |
| RP11-815J4.1 | -0.400907754 | 0.886769459 | | down |
| L22NC03-80A10.1 | -0.400907725 | 0.886225801 | | down |
| CCDC179 | -0.400907686 | 0.885463199 | | down |
| SPIN4-AS1 | -0.400907111 | 0.872369655 | | down |
| AC083864.4 | -0.400907066 | 0.871121749 | | down |
| RP11-815N9.2 | -0.400906551 | 0.853992423 | | down |
| DBIP2 | -0.400906213 | 0.838130355 | | down |
| snoU2_19 | -0.400906182 | 0.836431458 | | down |
| RP11-576N17.3 | -0.400905899 | 0.81761143 | | down |
| MIR744 | -0.400905735 | 0.803145827 | | down |
| RP11-423H2.4 | -0.400905727 | 0.802402644 | | down |
| RP11-324F21.1 | -0.400905711 | 0.800793216 | | down |
| PTTG3P | -0.400905704 | 0.800069425 | | down |
| RP11-187E13.2 | -0.400905574 | 0.785062034 | | down |
| CTD-2531D15.4 | -0.400905258 | 0.727674631 | | down |
| KDM6A | -0.400884161 | 0.043417962 | 0.110481007 | down |
| C22orf15 | -0.400874435 | 0.336201128 | 0.474354015 | down |
| RP11-68606.1 | -0.400341813 | 0.564268252 | 0.682449724 | down |
| RP11-400K9.4 | -0.400264086 | 0.256868946 | 0.391903906 | down |

| | | | | |
|---|---|---|---|---|
| POMZP3 | -0.400095555 | 0.107207617 | 0.212463433 | down |
| CCDC124 | -0.400054774 | 0.040516461 | 0.104937312 | down |
| FTH1P16 | -0.399686464 | 0.237430794 | 0.370622475 | down |
| GNG12 | -0.399587331 | 0.120242829 | 0.230482045 | down |
| TRMU | -0.399547567 | 0.020757948 | 0.063826481 | down |
| COQ5 | -0.399495442 | 2.48E-05 | 0.000298244 | down |
| NTRK2 | -0.399400306 | 0.334649286 | 0.472871938 | down |
| OGDH | -0.399217273 | 0.010336301 | 0.037623433 | down |
| DTYMK | -0.399080653 | 0.062270286 | 0.143657258 | down |
| LRRC37B | -0.3990647 | 0.051279702 | 0.124577292 | down |
| RYR2 | -0.398998676 | 0.334978487 | 0.473203577 | down |
| RP11-346C20.3 | -0.398958741 | 0.355098118 | 0.493725712 | down |
| PAX8-AS1 | -0.398931687 | 0.114291076 | 0.222206762 | down |
| TBC1D7 | -0.398887393 | 0.014958393 | 0.049830937 | down |
| RN7SKP16 | -0.398732502 | 0.707455489 | | down |
| EIF3FP3 | -0.398569246 | 0.086784089 | 0.182746665 | down |
| NFATC4 | -0.398079647 | 0.129758062 | 0.242879024 | down |
| RNF121 | -0.397937299 | 0.007118604 | 0.028342344 | down |
| HINT2 | -0.397874247 | 0.403149413 | 0.539780521 | down |
| NAA10 | -0.397863849 | 0.164074461 | 0.286429582 | down |
| ADORA2A | -0.397841432 | 0.318806199 | 0.457599905 | down |
| STK24-AS1 | -0.397822325 | 0.370633687 | 0.509043131 | down |
| LOC440461 | -0.397785015 | 0.400424275 | 0.537404937 | down |
| AC091493.2 | -0.397760948 | 0.662537475 | 0.764000764 | down |
| NDUFAB1 | -0.397607934 | 0.019280052 | 0.060439348 | down |
| SHARPIN | -0.397522163 | 0.039963714 | 0.103899449 | down |
| FDX1 | -0.397441812 | 0.003261596 | 0.015463373 | down |
| CAT | -0.397371999 | 0.142907343 | 0.260007764 | down |
| DTNA | -0.397344006 | 0.236705938 | 0.369798408 | down |
| RGMB | -0.397229925 | 0.044228792 | 0.111815341 | down |
| CTC-501O10.1 | -0.39706939 | 0.523218568 | 0.647429105 | down |
| RP11-455G16.1 | -0.397038088 | 0.576939515 | 0.693731321 | down |
| RP4-663N10.1 | -0.396983775 | 0.713147888 | | down |
| UCKL1 | -0.396951842 | 0.005145623 | 0.022176889 | down |
| SMARCE1P6 | -0.396903675 | 0.424905261 | 0.560524251 | down |
| NA | -0.396896146 | 0.655866574 | | down |
| SYNJ2BP | -0.396582864 | 0.03134191 | 0.086611142 | down |
| RP11-326I11.4 | -0.39650391 | 0.380250483 | 0.518176873 | down |
| RAI1-AS1 | -0.396495417 | 0.686165403 | | down |
| RP11-1072A3.3 | -0.396350793 | 0.432203954 | 0.567275231 | down |
| GSR | -0.396027644 | 0.00840023 | 0.032082361 | down |
| RP11-4O1.2 | -0.395977907 | 0.045357688 | 0.113850569 | down |
| RP1-101K10.6 | -0.395821935 | 0.459913799 | 0.59183836 | down |
| RP11-147L13.12 | -0.395800188 | 0.143972775 | 0.261420223 | down |
| SSTR3 | -0.395695067 | 0.305916087 | 0.444242975 | down |
| RP11-494K3.2 | -0.39566575 | 0.720328037 | | down |
| MST1R | -0.395651706 | 0.15003305 | 0.26899761 | down |
| POLR3A | -0.395646375 | 0.000104119 | 0.000979695 | down |
| PDCL3P4 | -0.395611801 | 0.249873131 | 0.3844517 | down |
| RP11-248B24.1 | -0.395505516 | 0.567471371 | 0.685474022 | down |
| LINC01468 | -0.395460738 | 0.373813831 | 0.512281896 | down |

| | | | | |
|---|---|---|---|---|
| RP5-115904.2 | -0.395323243 | 0.270344926 | 0.406349933 | down |
| AC087499.7 | -0.39527582 | 0.762437896 | | down |
| GRB14 | -0.395259852 | 0.353045394 | 0.491742967 | down |
| BACE1-AS | -0.395237292 | 0.091527758 | 0.189697845 | down |
| CEBPG | -0.395160371 | 0.027907296 | 0.079624579 | down |
| ALDH1L1 | -0.395106119 | 0.250580144 | 0.385251668 | down |
| NMNAT3 | -0.395080658 | 0.073406968 | 0.161855199 | down |
| NA | -0.395033618 | 0.342355286 | 0.480731081 | down |
| SLC25A5P1 | -0.394949484 | 0.532158331 | 0.655198954 | down |
| TMPRSS5 | -0.394914456 | 0.238291066 | 0.371714267 | down |
| RP1-69D17.3 | -0.394907085 | 0.651199901 | | down |
| LINC00028 | -0.394822991 | 0.582704669 | 0.698678177 | down |
| FDXACB1 | -0.394737479 | 0.136757579 | 0.252035984 | down |
| RP11-120K24.5 | -0.394730794 | 0.546600958 | 0.667703757 | down |
| SLC5A10 | -0.394694062 | 0.387522876 | 0.525109927 | down |
| C3orf52 | -0.394672062 | 0.05165469 | 0.125214903 | down |
| SIRT7 | -0.394639452 | 0.015596927 | 0.051476883 | down |
| MIR4251 | -0.394637208 | 0.892707046 | | down |
| RP1-58B11.1 | -0.394637208 | 0.892707046 | | down |
| PRAMEF32P | -0.394637208 | 0.892707046 | | down |
| RP11-219C24.10 | -0.394637208 | 0.892707046 | | down |
| CROCCP4 | -0.394637208 | 0.892707046 | | down |
| RP1-184J9.2 | -0.394637208 | 0.892707046 | | down |
| RNU6-1208P | -0.394637208 | 0.892707046 | | down |
| RP11-492M19.3 | -0.394637208 | 0.892707046 | | down |
| RNU6-48P | -0.394637208 | 0.892707046 | | down |
| RP5-1125N11.1 | -0.394637208 | 0.892707046 | | down |
| AL355480.2 | -0.394637208 | 0.892707046 | | down |
| RP11-346M5.1 | -0.394637208 | 0.892707046 | | down |
| MTND2P29 | -0.394637208 | 0.892707046 | | down |
| RNA5SP48 | -0.394637208 | 0.892707046 | | down |
| PIGQP1 | -0.394637208 | 0.892707046 | | down |
| RN7SL713P | -0.394637208 | 0.892707046 | | down |
| RNU6-414P | -0.394637208 | 0.892707046 | | down |
| RPS15AP7 | -0.394637208 | 0.892707046 | | down |
| RP4-771M4.2 | -0.394637208 | 0.892707046 | | down |
| MIR6068 | -0.394637208 | 0.892707046 | | down |
| RNU6-1031P | -0.394637208 | 0.892707046 | | down |
| RNU6-622P | -0.394637208 | 0.892707046 | | down |
| NA | -0.394637208 | 0.892707046 | | down |
| uc_338 | -0.394637208 | 0.892707046 | | down |
| RN7SKP272 | -0.394637208 | 0.892707046 | | down |
| MTATP6P13 | -0.394637208 | 0.892707046 | | down |
| RP4-586015.1 | -0.394637208 | 0.892707046 | | down |
| FLJ31662 | -0.394637208 | 0.892707046 | | down |
| RP5-898J17.1 | -0.394637208 | 0.892707046 | | down |
| RP11-272L13.2 | -0.394637208 | 0.892707046 | | down |
| RP11-8202.2 | -0.394637208 | 0.892707046 | | down |
| CYMP | -0.394637208 | 0.892707046 | | down |
| RN7SL432P | -0.394637208 | 0.892707046 | | down |
| RP4-791M13.5 | -0.394637208 | 0.892707046 | | down |

| | | | |
|---|---|---|---|
| RNU6-1042P | -0.394637208 | 0.892707046 | down |
| RP1-52J10.9 | -0.394637208 | 0.892707046 | down |
| MIR190B | -0.394637208 | 0.892707046 | down |
| RP11-419N10.5 | -0.394637208 | 0.892707046 | down |
| RP11-5K23.5 | -0.394637208 | 0.892707046 | down |
| U3 | -0.394637208 | 0.892707046 | down |
| RNU6-755P | -0.394637208 | 0.892707046 | down |
| RP11-38C18.3 | -0.394637208 | 0.892707046 | down |
| QRSL1P1 | -0.394637208 | 0.892707046 | down |
| RP1-102G20.4 | -0.394637208 | 0.892707046 | down |
| Y_RNA | -0.394637208 | 0.892707046 | down |
| MCRIP2P2 | -0.394637208 | 0.892707046 | down |
| RNA5SP73 | -0.394637208 | 0.892707046 | down |
| RP11-463J7.3 | -0.394637208 | 0.892707046 | down |
| RP11-567B20.2 | -0.394637208 | 0.892707046 | down |
| Y_RNA | -0.394637208 | 0.892707046 | down |
| RP11-335O13.8 | -0.394637208 | 0.892707046 | down |
| MIR135B | -0.394637208 | 0.892707046 | down |
| RNU2-19P | -0.394637208 | 0.892707046 | down |
| RP11-459K23.2 | -0.394637208 | 0.892707046 | down |
| RP11-323K10.1 | -0.394637208 | 0.892707046 | down |
| RNU4-57P | -0.394637208 | 0.892707046 | down |
| RPS27P5 | -0.394637208 | 0.892707046 | down |
| RP11-520H14.1 | -0.394637208 | 0.892707046 | down |
| NA | -0.394637208 | 0.892707046 | down |
| RAC1P7 | -0.394637208 | 0.892707046 | down |
| MTRNR2L11 | -0.394637208 | 0.892707046 | down |
| RP11-567G24.3 | -0.394637208 | 0.892707046 | down |
| OR2G2 | -0.394637208 | 0.892707046 | down |
| U6 | -0.394637208 | 0.892707046 | down |
| OR6F1 | -0.394637208 | 0.892707046 | down |
| HSD17B7P1 | -0.394637208 | 0.892707046 | down |
| AC018685.2 | -0.394637208 | 0.892707046 | down |
| MIR4262 | -0.394637208 | 0.892707046 | down |
| AC113608.1 | -0.394637208 | 0.892707046 | down |
| RN7SL140P | -0.394637208 | 0.892707046 | down |
| RNA5SP87 | -0.394637208 | 0.892707046 | down |
| AC012506.3 | -0.394637208 | 0.892707046 | down |
| AC015977.5 | -0.394637208 | 0.892707046 | down |
| Six3os1_4 | -0.394637208 | 0.892707046 | down |
| SNORA75 | -0.394637208 | 0.892707046 | down |
| RNU6-997P | -0.394637208 | 0.892707046 | down |
| RNU7-81P | -0.394637208 | 0.892707046 | down |
| RNU6-612P | -0.394637208 | 0.892707046 | down |
| AC096664.2 | -0.394637208 | 0.892707046 | down |
| RP11-51I5.1 | -0.394637208 | 0.892707046 | down |
| TAF13P2 | -0.394637208 | 0.892707046 | down |
| RP11-335E8.1 | -0.394637208 | 0.892707046 | down |
| NA | -0.394637208 | 0.892707046 | down |
| AC012075.1 | -0.394637208 | 0.892707046 | down |
| RN7SL201P | -0.394637208 | 0.892707046 | down |

| | | | |
|---|---|---|---|
| AC138623.1 | -0.394637208 | 0.892707046 | down |
| PGBD4P5 | -0.394637208 | 0.892707046 | down |
| RP11-165D20.1 | -0.394637208 | 0.892707046 | down |
| AC128677.3 | -0.394637208 | 0.892707046 | down |
| AC073464.6 | -0.394637208 | 0.892707046 | down |
| SNX18P14 | -0.394637208 | 0.892707046 | down |
| RP11-404J24.1 | -0.394637208 | 0.892707046 | down |
| RP11-219H23.2 | -0.394637208 | 0.892707046 | down |
| RP11-38C17.1 | -0.394637208 | 0.892707046 | down |
| HMGB3P11 | -0.394637208 | 0.892707046 | down |
| AC073409.1 | -0.394637208 | 0.892707046 | down |
| RNU7-182P | -0.394637208 | 0.892707046 | down |
| AC079776.6 | -0.394637208 | 0.892707046 | down |
| MTND2P22 | -0.394637208 | 0.892707046 | down |
| MTND1P26 | -0.394637208 | 0.892707046 | down |
| RP1-156L9.1 | -0.394637208 | 0.892707046 | down |
| AC011243.1 | -0.394637208 | 0.892707046 | down |
| RN7SKP93 | -0.394637208 | 0.892707046 | down |
| MRPS18BP2 | -0.394637208 | 0.892707046 | down |
| AC023128.2 | -0.394637208 | 0.892707046 | down |
| AC079150.2 | -0.394637208 | 0.892707046 | down |
| SNORD56 | -0.394637208 | 0.892707046 | down |
| CBX3P6 | -0.394637208 | 0.892707046 | down |
| MTND4P28 | -0.394637208 | 0.892707046 | down |
| OR7E89P | -0.394637208 | 0.892707046 | down |
| KRT18P46 | -0.394637208 | 0.892707046 | down |
| CYP2C56P | -0.394637208 | 0.892707046 | down |
| XIRP2-AS1 | -0.394637208 | 0.892707046 | down |
| RNU6-1290P | -0.394637208 | 0.892707046 | down |
| AC016751.3 | -0.394637208 | 0.892707046 | down |
| snoU13 | -0.394637208 | 0.892707046 | down |
| AC007557.2 | -0.394637208 | 0.892707046 | down |
| Y_RNA | -0.394637208 | 0.892707046 | down |
| RP11-525G12.1 | -0.394637208 | 0.892707046 | down |
| RN7SL807P | -0.394637208 | 0.892707046 | down |
| RNA5SP121 | -0.394637208 | 0.892707046 | down |
| AC006037.2 | -0.394637208 | 0.892707046 | down |
| OR6B2 | -0.394637208 | 0.892707046 | down |
| VENTXP4 | -0.394637208 | 0.892707046 | down |
| RBMS3-AS1 | -0.394637208 | 0.892707046 | down |
| RP11-384L8.2 | -0.394637208 | 0.892707046 | down |
| AC018359.3 | -0.394637208 | 0.892707046 | down |
| RP11-333B11.1 | -0.394637208 | 0.892707046 | down |
| Y_RNA | -0.394637208 | 0.892707046 | down |
| EI24P3 | -0.394637208 | 0.892707046 | down |
| RP11-359I18.1 | -0.394637208 | 0.892707046 | down |
| UBL5P3 | -0.394637208 | 0.892707046 | down |
| RNA5SP134 | -0.394637208 | 0.892707046 | down |
| RP11-436J20.1 | -0.394637208 | 0.892707046 | down |
| RN7SL418P | -0.394637208 | 0.892707046 | down |
| NA | -0.394637208 | 0.892707046 | down |

| | | | |
|---|---|---|---|
| MIR1284 | -0.394637208 | 0.892707046 | down |
| MIR3923 | -0.394637208 | 0.892707046 | down |
| RP11-359D24.1 | -0.394637208 | 0.892707046 | down |
| CADM2-AS2 | -0.394637208 | 0.892707046 | down |
| APOOP2 | -0.394637208 | 0.892707046 | down |
| PSMC1P6 | -0.394637208 | 0.892707046 | down |
| RP11-325B23.2 | -0.394637208 | 0.892707046 | down |
| NA | -0.394637208 | 0.892707046 | down |
| RNU6-1263P | -0.394637208 | 0.892707046 | down |
| MIR548AB | -0.394637208 | 0.892707046 | down |
| MTATP6P22 | -0.394637208 | 0.892707046 | down |
| MTND5P16 | -0.394637208 | 0.892707046 | down |
| LINC00901 | -0.394637208 | 0.892707046 | down |
| RP11-500K19.1 | -0.394637208 | 0.892707046 | down |
| RP11-500K19.2 | -0.394637208 | 0.892707046 | down |
| OR7E97P | -0.394637208 | 0.892707046 | down |
| Y_RNA | -0.394637208 | 0.892707046 | down |
| NPHP3-ACAD11 | -0.394637208 | 0.892707046 | down |
| NDUFS6P1 | -0.394637208 | 0.892707046 | down |
| PISRT1 | -0.394637208 | 0.892707046 | down |
| TRMT112P5 | -0.394637208 | 0.892707046 | down |
| RP11-50102.3 | -0.394637208 | 0.892707046 | down |
| RP11-439C8.1 | -0.394637208 | 0.892707046 | down |
| MIR16-2 | -0.394637208 | 0.892707046 | down |
| LINC01326 | -0.394637208 | 0.892707046 | down |
| TMEM212-IT1 | -0.394637208 | 0.892707046 | down |
| SNORA18 | -0.394637208 | 0.892707046 | down |
| RNU6-1120P | -0.394637208 | 0.892707046 | down |
| RP11-360P21.2 | -0.394637208 | 0.892707046 | down |
| NA | -0.394637208 | 0.892707046 | down |
| RP11-528A4.3 | -0.394637208 | 0.892707046 | down |
| MIR4797 | -0.394637208 | 0.892707046 | down |
| NKX1-1 | -0.394637208 | 0.892707046 | down |
| OR7E83P | -0.394637208 | 0.892707046 | down |
| AC006840.1 | -0.394637208 | 0.892707046 | down |
| AC024132.1 | -0.394637208 | 0.892707046 | down |
| IGBP1P5 | -0.394637208 | 0.892707046 | down |
| MIR4801 | -0.394637208 | 0.892707046 | down |
| RNU7-74P | -0.394637208 | 0.892707046 | down |
| Y_RNA | -0.394637208 | 0.892707046 | down |
| MTND3P22 | -0.394637208 | 0.892707046 | down |
| RP11-61F5.1 | -0.394637208 | 0.892707046 | down |
| RNA5SP161 | -0.394637208 | 0.892707046 | down |
| RP11-593F5.1 | -0.394637208 | 0.892707046 | down |
| RP11-26C10.1 | -0.394637208 | 0.892707046 | down |
| RNU6-191P | -0.394637208 | 0.892707046 | down |
| RP11-468N14.11 | -0.394637208 | 0.892707046 | down |
| RP13-644M16.5 | -0.394637208 | 0.892707046 | down |
| RP13-644M16.4 | -0.394637208 | 0.892707046 | down |
| HSPE1P23 | -0.394637208 | 0.892707046 | down |
| RP11-44F21.4 | -0.394637208 | 0.892707046 | down |

| | | | |
|---|---|---|---|
| RNU6-1000P | -0.394637208 | 0.892707046 | down |
| RNU5A-2P | -0.394637208 | 0.892707046 | down |
| RP11-42A4.1 | -0.394637208 | 0.892707046 | down |
| RP11-710E1.1 | -0.394637208 | 0.892707046 | down |
| RPL23AP94 | -0.394637208 | 0.892707046 | down |
| AC107399.1 | -0.394637208 | 0.892707046 | down |
| PGBD4P4 | -0.394637208 | 0.892707046 | down |
| RNU6-224P | -0.394637208 | 0.892707046 | down |
| RNU6-506P | -0.394637208 | 0.892707046 | down |
| RP11-586L23.1 | -0.394637208 | 0.892707046 | down |
| RP11-54P19.1 | -0.394637208 | 0.892707046 | down |
| RP11-292D4.1 | -0.394637208 | 0.892707046 | down |
| RP11-292D4.4 | -0.394637208 | 0.892707046 | down |
| RP11-423J7.1 | -0.394637208 | 0.892707046 | down |
| RNA5SP168 | -0.394637208 | 0.892707046 | down |
| MTCO3P9 | -0.394637208 | 0.892707046 | down |
| SNORD39 | -0.394637208 | 0.892707046 | down |
| Y_RNA | -0.394637208 | 0.892707046 | down |
| RP11-719L21.1 | -0.394637208 | 0.892707046 | down |
| RP11-366M4.19 | -0.394637208 | 0.892707046 | down |
| RP11-765K14.1 | -0.394637208 | 0.892707046 | down |
| RP11-335L23.2 | -0.394637208 | 0.892707046 | down |
| RNU6-479P | -0.394637208 | 0.892707046 | down |
| RNU4-64P | -0.394637208 | 0.892707046 | down |
| LINC01596 | -0.394637208 | 0.892707046 | down |
| CLUHP4 | -0.394637208 | 0.892707046 | down |
| RP11-445O3.3 | -0.394637208 | 0.892707046 | down |
| CTD-2247C11.5 | -0.394637208 | 0.892707046 | down |
| MTCO1P30 | -0.394637208 | 0.892707046 | down |
| CTC-471C19.2 | -0.394637208 | 0.892707046 | down |
| RP11-417J1.6 | -0.394637208 | 0.892707046 | down |
| MTCYBP37 | -0.394637208 | 0.892707046 | down |
| Y_RNA | -0.394637208 | 0.892707046 | down |
| RP11-432M8.15 | -0.394637208 | 0.892707046 | down |
| HSPD1P15 | -0.394637208 | 0.892707046 | down |
| Y_RNA | -0.394637208 | 0.892707046 | down |
| RP11-5N11.5 | -0.394637208 | 0.892707046 | down |
| RP11-79C6.3 | -0.394637208 | 0.892707046 | down |
| CTD-2007F2.1 | -0.394637208 | 0.892707046 | down |
| CTD-2054H24.1 | -0.394637208 | 0.892707046 | down |
| RP11-182I21.2 | -0.394637208 | 0.892707046 | down |
| RP11-45H22.3 | -0.394637208 | 0.892707046 | down |
| RP11-101B14.4 | -0.394637208 | 0.892707046 | down |
| MIR548AE2 | -0.394637208 | 0.892707046 | down |
| RP11-83M16.1 | -0.394637208 | 0.892707046 | down |
| MIR4803 | -0.394637208 | 0.892707046 | down |
| Y_RNA | -0.394637208 | 0.892707046 | down |
| CTD-2037K23.1 | -0.394637208 | 0.892707046 | down |
| CTC-431G16.3 | -0.394637208 | 0.892707046 | down |
| CTD-2227C6.3 | -0.394637208 | 0.892707046 | down |
| RNU6-620P | -0.394637208 | 0.892707046 | down |

| | | | |
|---|---|---|---|
| RP11-509M23.1 | -0.394637208 | 0.892707046 | down |
| RP11-1072N2.2 | -0.394637208 | 0.892707046 | down |
| PTP4A1P4 | -0.394637208 | 0.892707046 | down |
| CCT7P2 | -0.394637208 | 0.892707046 | down |
| MIR583 | -0.394637208 | 0.892707046 | down |
| RP11-231G15.3 | -0.394637208 | 0.892707046 | down |
| HMGN1P15 | -0.394637208 | 0.892707046 | down |
| RNU6-373P | -0.394637208 | 0.892707046 | down |
| MIR5706 | -0.394637208 | 0.892707046 | down |
| RNU7-53P | -0.394637208 | 0.892707046 | down |
| CTB-49A3.1 | -0.394637208 | 0.892707046 | down |
| GS1-39E22.2 | -0.394637208 | 0.892707046 | down |
| Y_RNA | -0.394637208 | 0.892707046 | down |
| RN7SKP246 | -0.394637208 | 0.892707046 | down |
| NAMPTP2 | -0.394637208 | 0.892707046 | down |
| CTB-33O18.1 | -0.394637208 | 0.892707046 | down |
| NA | -0.394637208 | 0.892707046 | down |
| CTC-571O20.1 | -0.394637208 | 0.892707046 | down |
| RP11-21I4.2 | -0.394637208 | 0.892707046 | down |
| RP11-420L9.4 | -0.394637208 | 0.892707046 | down |
| RP1-140K8.1 | -0.394637208 | 0.892707046 | down |
| RP11-360O19.4 | -0.394637208 | 0.892707046 | down |
| Metazoa_SRP | -0.394637208 | 0.892707046 | down |
| RP4-551N11.1 | -0.394637208 | 0.892707046 | down |
| HIST1H2APS1 | -0.394637208 | 0.892707046 | down |
| OR2J4P | -0.394637208 | 0.892707046 | down |
| Xbac-BPG118E17.1 | -0.394637208 | 0.892707046 | down |
| ZNF70P1 | -0.394637208 | 0.892707046 | down |
| RNU1-105P | -0.394637208 | 0.892707046 | down |
| DEFB110 | -0.394637208 | 0.892707046 | down |
| MIR133B | -0.394637208 | 0.892707046 | down |
| RPL31P28 | -0.394637208 | 0.892707046 | down |
| RPL10P10 | -0.394637208 | 0.892707046 | down |
| KRASP1 | -0.394637208 | 0.892707046 | down |
| NPM1P36 | -0.394637208 | 0.892707046 | down |
| GAPDHP41 | -0.394637208 | 0.892707046 | down |
| RP11-307F22.2 | -0.394637208 | 0.892707046 | down |
| NA | -0.394637208 | 0.892707046 | down |
| U3 | -0.394637208 | 0.892707046 | down |
| MTCYBP36 | -0.394637208 | 0.892707046 | down |
| RN7SL797P | -0.394637208 | 0.892707046 | down |
| RP1-104O17.3 | -0.394637208 | 0.892707046 | down |
| Y_RNA | -0.394637208 | 0.892707046 | down |
| RNU6-527P | -0.394637208 | 0.892707046 | down |
| RP11-697G4.4 | -0.394637208 | 0.892707046 | down |
| RP11-425D10.1 | -0.394637208 | 0.892707046 | down |
| RP11-506B6.5 | -0.394637208 | 0.892707046 | down |
| RP1-276J11.2 | -0.394637208 | 0.892707046 | down |
| Y_RNA | -0.394637208 | 0.892707046 | down |
| RP3-406A7.1 | -0.394637208 | 0.892707046 | down |
| RP3-406A7.3 | -0.394637208 | 0.892707046 | down |

| | | | |
|---|---|---|---|
| NA | -0.394637208 | 0.892707046 | down |
| RN7SL234P | -0.394637208 | 0.892707046 | down |
| RP11-568A7.4 | -0.394637208 | 0.892707046 | down |
| RNU6-215P | -0.394637208 | 0.892707046 | down |
| CTD-2060L22.1 | -0.394637208 | 0.892707046 | down |
| AC004535.2 | -0.394637208 | 0.892707046 | down |
| RBMX2P4 | -0.394637208 | 0.892707046 | down |
| AC098592.6 | -0.394637208 | 0.892707046 | down |
| RN7SKP266 | -0.394637208 | 0.892707046 | down |
| MTCYBP42 | -0.394637208 | 0.892707046 | down |
| AC005017.2 | -0.394637208 | 0.892707046 | down |
| NA | -0.394637208 | 0.892707046 | down |
| SNX2P2 | -0.394637208 | 0.892707046 | down |
| RNU6-241P | -0.394637208 | 0.892707046 | down |
| RNU6-1125P | -0.394637208 | 0.892707046 | down |
| RNU6-1126P | -0.394637208 | 0.892707046 | down |
| RP11-1217F2.22 | -0.394637208 | 0.892707046 | down |
| NA | -0.394637208 | 0.892707046 | down |
| RP11-114G11.3 | -0.394637208 | 0.892707046 | down |
| RP11-368M16.10 | -0.394637208 | 0.892707046 | down |
| RP11-548K12.5 | -0.394637208 | 0.892707046 | down |
| RP11-548K12.4 | -0.394637208 | 0.892707046 | down |
| RNU6-417P | -0.394637208 | 0.892707046 | down |
| snoU2_19 | -0.394637208 | 0.892707046 | down |
| RP11-340I6.5 | -0.394637208 | 0.892707046 | down |
| NA | -0.394637208 | 0.892707046 | down |
| MTCO3P8 | -0.394637208 | 0.892707046 | down |
| MTND2P4 | -0.394637208 | 0.892707046 | down |
| VN1R40P | -0.394637208 | 0.892707046 | down |
| MTCO1P57 | -0.394637208 | 0.892707046 | down |
| RNU6-832P | -0.394637208 | 0.892707046 | down |
| MTCO2P25 | -0.394637208 | 0.892707046 | down |
| RNU6-229P | -0.394637208 | 0.892707046 | down |
| Metazoa_SRP | -0.394637208 | 0.892707046 | down |
| AC005077.8 | -0.394637208 | 0.892707046 | down |
| Y_RNA | -0.394637208 | 0.892707046 | down |
| RNU6-274P | -0.394637208 | 0.892707046 | down |
| AC002075.3 | -0.394637208 | 0.892707046 | down |
| RNU6-1328P | -0.394637208 | 0.892707046 | down |
| ATP5F1P2 | -0.394637208 | 0.892707046 | down |
| BANF1P5 | -0.394637208 | 0.892707046 | down |
| RN7SKP223 | -0.394637208 | 0.892707046 | down |
| RNU6-1272P | -0.394637208 | 0.892707046 | down |
| WBP1LP1 | -0.394637208 | 0.892707046 | down |
| OR6V1 | -0.394637208 | 0.892707046 | down |
| RNU6-267P | -0.394637208 | 0.892707046 | down |
| AC005229.5 | -0.394637208 | 0.892707046 | down |
| RP11-632K21.3 | -0.394637208 | 0.892707046 | down |
| NA | -0.394637208 | 0.892707046 | down |
| RP11-303G3.9 | -0.394637208 | 0.892707046 | down |
| RP11-429O2.1 | -0.394637208 | 0.892707046 | down |

| | | | |
|---|---|---|---|
| RP11-299D14.2 | -0.394637208 | 0.892707046 | down |
| RP11-94H18.2 | -0.394637208 | 0.892707046 | down |
| RNA5SP262 | -0.394637208 | 0.892707046 | down |
| RNA5SP263 | -0.394637208 | 0.892707046 | down |
| RP11-412B14.2 | -0.394637208 | 0.892707046 | down |
| RP11-359P18.1 | -0.394637208 | 0.892707046 | down |
| IGLV8OR8-1 | -0.394637208 | 0.892707046 | down |
| RP11-182E14.1 | -0.394637208 | 0.892707046 | down |
| RNU6ATAC32P | -0.394637208 | 0.892707046 | down |
| TRMT112P7 | -0.394637208 | 0.892707046 | down |
| SNORA3 | -0.394637208 | 0.892707046 | down |
| RP11-403D15.1 | -0.394637208 | 0.892707046 | down |
| RPS20P20 | -0.394637208 | 0.892707046 | down |
| Y_RNA | -0.394637208 | 0.892707046 | down |
| RP11-730G20.1 | -0.394637208 | 0.892707046 | down |
| COX6B1P6 | -0.394637208 | 0.892707046 | down |
| RP11-700E23.3 | -0.394637208 | 0.892707046 | down |
| KB-1083B1.1 | -0.394637208 | 0.892707046 | down |
| RNU7-67P | -0.394637208 | 0.892707046 | down |
| snoU13 | -0.394637208 | 0.892707046 | down |
| RPS12P15 | -0.394637208 | 0.892707046 | down |
| RP11-200A13.2 | -0.394637208 | 0.892707046 | down |
| RP11-58O3.1 | -0.394637208 | 0.892707046 | down |
| RNU4-37P | -0.394637208 | 0.892707046 | down |
| RPL30P16 | -0.394637208 | 0.892707046 | down |
| RP11-775B15.3 | -0.394637208 | 0.892707046 | down |
| MRPS36P3 | -0.394637208 | 0.892707046 | down |
| RP11-1082L8.1 | -0.394637208 | 0.892707046 | down |
| RN7SL260P | -0.394637208 | 0.892707046 | down |
| RP11-429J17.4 | -0.394637208 | 0.892707046 | down |
| RPS27AP14 | -0.394637208 | 0.892707046 | down |
| RP11-459D20.1 | -0.394637208 | 0.892707046 | down |
| MTATP6P11 | -0.394637208 | 0.892707046 | down |
| RNU6-14P | -0.394637208 | 0.892707046 | down |
| RP11-113D19.9 | -0.394637208 | 0.892707046 | down |
| IFNA8 | -0.394637208 | 0.892707046 | down |
| SUMO2P2 | -0.394637208 | 0.892707046 | down |
| NA | -0.394637208 | 0.892707046 | down |
| RMRPP5 | -0.394637208 | 0.892707046 | down |
| RN7SL100P | -0.394637208 | 0.892707046 | down |
| MTATP6P30 | -0.394637208 | 0.892707046 | down |
| RN7SKP114 | -0.394637208 | 0.892707046 | down |
| Y_RNA | -0.394637208 | 0.892707046 | down |
| SNX18P5 | -0.394637208 | 0.892707046 | down |
| RP11-144A16.4 | -0.394637208 | 0.892707046 | down |
| Y_RNA | -0.394637208 | 0.892707046 | down |
| RP11-30P16.1 | -0.394637208 | 0.892707046 | down |
| RP11-70K10.2 | -0.394637208 | 0.892707046 | down |
| MTND3P4 | -0.394637208 | 0.892707046 | down |
| OR13F1 | -0.394637208 | 0.892707046 | down |
| RPL36AP6 | -0.394637208 | 0.892707046 | down |

| | | | |
|---|---|---|---|
| RP11-127L21.1 | -0.394637208 | 0.892707046 | down |
| RP11-542K23.10 | -0.394637208 | 0.892707046 | down |
| OR1N1 | -0.394637208 | 0.892707046 | down |
| RP11-205K6.2 | -0.394637208 | 0.892707046 | down |
| Y_RNA | -0.394637208 | 0.892707046 | down |
| TPRKBP1 | -0.394637208 | 0.892707046 | down |
| RP11-285G1.15 | -0.394637208 | 0.892707046 | down |
| RP11-478B11.2 | -0.394637208 | 0.892707046 | down |
| MRPL50P4 | -0.394637208 | 0.892707046 | down |
| RP11-111N20.4 | -0.394637208 | 0.892707046 | down |
| RP11-65E10.1 | -0.394637208 | 0.892707046 | down |
| RP11-227H15.7 | -0.394637208 | 0.892707046 | down |
| MTCO2P23 | -0.394637208 | 0.892707046 | down |
| RNU6-805P | -0.394637208 | 0.892707046 | down |
| RP11-325D15.2 | -0.394637208 | 0.892707046 | down |
| TPRX1P1 | -0.394637208 | 0.892707046 | down |
| CTSLP6 | -0.394637208 | 0.892707046 | down |
| MARK2P15 | -0.394637208 | 0.892707046 | down |
| NA | -0.394637208 | 0.892707046 | down |
| RP11-113E21.2 | -0.394637208 | 0.892707046 | down |
| SLC16A12-AS1 | -0.394637208 | 0.892707046 | down |
| MIR107 | -0.394637208 | 0.892707046 | down |
| RNU6-657P | -0.394637208 | 0.892707046 | down |
| NA | -0.394637208 | 0.892707046 | down |
| RNU5B-6P | -0.394637208 | 0.892707046 | down |
| BTF3P15 | -0.394637208 | 0.892707046 | down |
| BTBD7P2 | -0.394637208 | 0.892707046 | down |
| RP11-106M7.4 | -0.394637208 | 0.892707046 | down |
| SNRPGP6 | -0.394637208 | 0.892707046 | down |
| RN7SKP167 | -0.394637208 | 0.892707046 | down |
| RNU2-42P | -0.394637208 | 0.892707046 | down |
| NA | -0.394637208 | 0.892707046 | down |
| AP006285.7 | -0.394637208 | 0.892707046 | down |
| RNU6-878P | -0.394637208 | 0.892707046 | down |
| NA | -0.394637208 | 0.892707046 | down |
| OR52M2P | -0.394637208 | 0.892707046 | down |
| OR51F4P | -0.394637208 | 0.892707046 | down |
| HBE1 | -0.394637208 | 0.892707046 | down |
| OR52Q1P | -0.394637208 | 0.892707046 | down |
| OR56A7P | -0.394637208 | 0.892707046 | down |
| RP11-236J17.6 | -0.394637208 | 0.892707046 | down |
| NA | -0.394637208 | 0.892707046 | down |
| RNU7-28P | -0.394637208 | 0.892707046 | down |
| RP11-358H18.3 | -0.394637208 | 0.892707046 | down |
| RP11-485O14.1 | -0.394637208 | 0.892707046 | down |
| RP11-460B17.2 | -0.394637208 | 0.892707046 | down |
| RPL7AP56 | -0.394637208 | 0.892707046 | down |
| RP11-810F22.1 | -0.394637208 | 0.892707046 | down |
| OR4X1 | -0.394637208 | 0.892707046 | down |
| OR4A48P | -0.394637208 | 0.892707046 | down |
| OR4A41P | -0.394637208 | 0.892707046 | down |

| | | | |
|---|---|---|---|
| RP11-163O19.10 | -0.394637208 | 0.892707046 | down |
| RP11-72M10.5 | -0.394637208 | 0.892707046 | down |
| OR4S2 | -0.394637208 | 0.892707046 | down |
| OR5D13 | -0.394637208 | 0.892707046 | down |
| OR5BN1P | -0.394637208 | 0.892707046 | down |
| OR8V1P | -0.394637208 | 0.892707046 | down |
| OR5AL2P | -0.394637208 | 0.892707046 | down |
| OR5M8 | -0.394637208 | 0.892707046 | down |
| CTD-3051L14.13 | -0.394637208 | 0.892707046 | down |
| NA | -0.394637208 | 0.892707046 | down |
| OR9I3P | -0.394637208 | 0.892707046 | down |
| EIF4A2P3 | -0.394637208 | 0.892707046 | down |
| RN7SL42P | -0.394637208 | 0.892707046 | down |
| NA | -0.394637208 | 0.892707046 | down |
| AP001271.3 | -0.394637208 | 0.892707046 | down |
| RP11-876F14.1 | -0.394637208 | 0.892707046 | down |
| AP000857.3 | -0.394637208 | 0.892707046 | down |
| RP11-164N3.2 | -0.394637208 | 0.892707046 | down |
| RP11-164N3.3 | -0.394637208 | 0.892707046 | down |
| RP11-643G5.6 | -0.394637208 | 0.892707046 | down |
| RP11-313I2.6 | -0.394637208 | 0.892707046 | down |
| TRIM49D1 | -0.394637208 | 0.892707046 | down |
| NDUFB11P1 | -0.394637208 | 0.892707046 | down |
| RNU6-277P | -0.394637208 | 0.892707046 | down |
| RNU6-1107P | -0.394637208 | 0.892707046 | down |
| NA | -0.394637208 | 0.892707046 | down |
| RPL34P23 | -0.394637208 | 0.892707046 | down |
| PHBP17 | -0.394637208 | 0.892707046 | down |
| OR10G8 | -0.394637208 | 0.892707046 | down |
| PATE3 | -0.394637208 | 0.892707046 | down |
| NTM-IT | -0.394637208 | 0.892707046 | down |
| Y_RNA | -0.394637208 | 0.892707046 | down |
| RP11-815D16.1 | -0.394637208 | 0.892707046 | down |
| RNU7-60P | -0.394637208 | 0.892707046 | down |
| RP11-158N24.1 | -0.394637208 | 0.892707046 | down |
| AK6P2 | -0.394637208 | 0.892707046 | down |
| NA | -0.394637208 | 0.892707046 | down |
| NF1P12 | -0.394637208 | 0.892707046 | down |
| SNORA22 | -0.394637208 | 0.892707046 | down |
| RP11-478B9.3 | -0.394637208 | 0.892707046 | down |
| RNU6-940P | -0.394637208 | 0.892707046 | down |
| METTL7AP1 | -0.394637208 | 0.892707046 | down |
| OR6C5P | -0.394637208 | 0.892707046 | down |
| OR6C64P | -0.394637208 | 0.892707046 | down |
| NA | -0.394637208 | 0.892707046 | down |
| DUX4L52 | -0.394637208 | 0.892707046 | down |
| RNU1-83P | -0.394637208 | 0.892707046 | down |
| RNU6-166P | -0.394637208 | 0.892707046 | down |
| RAB11AP2 | -0.394637208 | 0.892707046 | down |
| VENTXP3 | -0.394637208 | 0.892707046 | down |
| NA | -0.394637208 | 0.892707046 | down |

| | | | |
|---|---|---|---|
| RNU7-120P | -0.394637208 | 0.892707046 | down |
| MRPS6P4 | -0.394637208 | 0.892707046 | down |
| RP11-575G13.3 | -0.394637208 | 0.892707046 | down |
| RP11-438H24.2 | -0.394637208 | 0.892707046 | down |
| RNU6-361P | -0.394637208 | 0.892707046 | down |
| RPS15AP32 | -0.394637208 | 0.892707046 | down |
| RN7SKP216 | -0.394637208 | 0.892707046 | down |
| SNORD56 | -0.394637208 | 0.892707046 | down |
| NA | -0.394637208 | 0.892707046 | down |
| RNA5SP374 | -0.394637208 | 0.892707046 | down |
| RP11-364C11.2 | -0.394637208 | 0.892707046 | down |
| MIR3612 | -0.394637208 | 0.892707046 | down |
| RP11-669N7.3 | -0.394637208 | 0.892707046 | down |
| LINC00328-2P | -0.394637208 | 0.892707046 | down |
| RP11-408E5.8 | -0.394637208 | 0.892707046 | down |
| LINC00556 | -0.394637208 | 0.892707046 | down |
| RFESDP1 | -0.394637208 | 0.892707046 | down |
| VDAC1P12 | -0.394637208 | 0.892707046 | down |
| RP11-478K15.7 | -0.394637208 | 0.892707046 | down |
| TIMM9P3 | -0.394637208 | 0.892707046 | down |
| COX4I1P2 | -0.394637208 | 0.892707046 | down |
| FKBP1AP3 | -0.394637208 | 0.892707046 | down |
| POLR2KP2 | -0.394637208 | 0.892707046 | down |
| SNRPGP11 | -0.394637208 | 0.892707046 | down |
| RN7SL413P | -0.394637208 | 0.892707046 | down |
| POLR3KP1 | -0.394637208 | 0.892707046 | down |
| STARP1 | -0.394637208 | 0.892707046 | down |
| MIR4704 | -0.394637208 | 0.892707046 | down |
| RIOK3P1 | -0.394637208 | 0.892707046 | down |
| LINC01038 | -0.394637208 | 0.892707046 | down |
| DPPA3P3 | -0.394637208 | 0.892707046 | down |
| RNU6-77P | -0.394637208 | 0.892707046 | down |
| VENTXP2 | -0.394637208 | 0.892707046 | down |
| LIN28AP2 | -0.394637208 | 0.892707046 | down |
| NUS1P4 | -0.394637208 | 0.892707046 | down |
| ARF4P3 | -0.394637208 | 0.892707046 | down |
| RP11-496I2.7 | -0.394637208 | 0.892707046 | down |
| RP11-244H18.4 | -0.394637208 | 0.892707046 | down |
| OR4T1P | -0.394637208 | 0.892707046 | down |
| RP11-14J7.7 | -0.394637208 | 0.892707046 | down |
| OR10G1P | -0.394637208 | 0.892707046 | down |
| SNORD41 | -0.394637208 | 0.892707046 | down |
| RP11-388E23.3 | -0.394637208 | 0.892707046 | down |
| RP11-159D23.2 | -0.394637208 | 0.892707046 | down |
| RP11-73E17.3 | -0.394637208 | 0.892707046 | down |
| RPL23AP70 | -0.394637208 | 0.892707046 | down |
| RP11-322L17.1 | -0.394637208 | 0.892707046 | down |
| RP11-476J6.1 | -0.394637208 | 0.892707046 | down |
| RP11-286B5.1 | -0.394637208 | 0.892707046 | down |
| RP11-326E7.1 | -0.394637208 | 0.892707046 | down |
| RP11-1140I5.1 | -0.394637208 | 0.892707046 | down |

| | | | |
|---|---|---|---|
| MIR4308 | -0.394637208 | 0.892707046 | down |
| RP11-1012A1.4 | -0.394637208 | 0.892707046 | down |
| RNA5SP388 | -0.394637208 | 0.892707046 | down |
| RP11-588P7.2 | -0.394637208 | 0.892707046 | down |
| RP11-241N4.2 | -0.394637208 | 0.892707046 | down |
| Mico1 | -0.394637208 | 0.892707046 | down |
| SNORD113-2 | -0.394637208 | 0.892707046 | down |
| MIR329-2 | -0.394637208 | 0.892707046 | down |
| MIR134 | -0.394637208 | 0.892707046 | down |
| MIR668 | -0.394637208 | 0.892707046 | down |
| U3 | -0.394637208 | 0.892707046 | down |
| RNU4-68P | -0.394637208 | 0.892707046 | down |
| IGHV3-25 | -0.394637208 | 0.892707046 | down |
| IGHVIII-51-1 | -0.394637208 | 0.892707046 | down |
| RNU6-978P | -0.394637208 | 0.892707046 | down |
| IGHD2OR15-2A | -0.394637208 | 0.892707046 | down |
| BCAR1P1 | -0.394637208 | 0.892707046 | down |
| RN7SL400P | -0.394637208 | 0.892707046 | down |
| NA | -0.394637208 | 0.892707046 | down |
| OR4N3P | -0.394637208 | 0.892707046 | down |
| SNORD115-5 | -0.394637208 | 0.892707046 | down |
| SNORD115-46 | -0.394637208 | 0.892707046 | down |
| RP11-30G8.2 | -0.394637208 | 0.892707046 | down |
| SNORD77 | -0.394637208 | 0.892707046 | down |
| RP11-597G23.1 | -0.394637208 | 0.892707046 | down |
| GAPDHP55 | -0.394637208 | 0.892707046 | down |
| CTD-2014N11.1 | -0.394637208 | 0.892707046 | down |
| RP11-627D16.1 | -0.394637208 | 0.892707046 | down |
| RNU6-745P | -0.394637208 | 0.892707046 | down |
| RP11-361M10.4 | -0.394637208 | 0.892707046 | down |
| RNU6-1339P | -0.394637208 | 0.892707046 | down |
| RN7SL346P | -0.394637208 | 0.892707046 | down |
| RN7SKP254 | -0.394637208 | 0.892707046 | down |
| RP11-183E24.1 | -0.394637208 | 0.892707046 | down |
| SNRPCP18 | -0.394637208 | 0.892707046 | down |
| WBP1LP5 | -0.394637208 | 0.892707046 | down |
| RP11-382N13.4 | -0.394637208 | 0.892707046 | down |
| Y_RNA | -0.394637208 | 0.892707046 | down |
| SNORA30 | -0.394637208 | 0.892707046 | down |
| TP53TG3 | -0.394637208 | 0.892707046 | down |
| DUX4L45 | -0.394637208 | 0.892707046 | down |
| PCMTD1P2 | -0.394637208 | 0.892707046 | down |
| RP11-14K3.7 | -0.394637208 | 0.892707046 | down |
| RNA5SP407 | -0.394637208 | 0.892707046 | down |
| RNA5SP409 | -0.394637208 | 0.892707046 | down |
| RNA5SP417 | -0.394637208 | 0.892707046 | down |
| RNA5SP418 | -0.394637208 | 0.892707046 | down |
| RNU6-845P | -0.394637208 | 0.892707046 | down |
| RNA5SP426 | -0.394637208 | 0.892707046 | down |
| RP11-50021.1 | -0.394637208 | 0.892707046 | down |
| RP11-513N24.1 | -0.394637208 | 0.892707046 | down |

| | | | |
|---|---|---|---|
| RN7SKP190 | -0.394637208 | 0.892707046 | down |
| COX6CP16 | -0.394637208 | 0.892707046 | down |
| Y_RNA | -0.394637208 | 0.892707046 | down |
| CH17-53B9.2 | -0.394637208 | 0.892707046 | down |
| MTCO3P13 | -0.394637208 | 0.892707046 | down |
| RP11-846F4.10 | -0.394637208 | 0.892707046 | down |
| RP1-66C13.2 | -0.394637208 | 0.892707046 | down |
| RNU6-990P | -0.394637208 | 0.892707046 | down |
| RP11-40A13.1 | -0.394637208 | 0.892707046 | down |
| RP11-17M24.3 | -0.394637208 | 0.892707046 | down |
| CTC-304I17.3 | -0.394637208 | 0.892707046 | down |
| RNA5SP438 | -0.394637208 | 0.892707046 | down |
| RP11-686D22.9 | -0.394637208 | 0.892707046 | down |
| KRTAP9-1 | -0.394637208 | 0.892707046 | down |
| MIR6780A | -0.394637208 | 0.892707046 | down |
| RP11-1094H24.3 | -0.394637208 | 0.892707046 | down |
| RP11-94C24.11 | -0.394637208 | 0.892707046 | down |
| MIR8059 | -0.394637208 | 0.892707046 | down |
| RP11-1018N14.4 | -0.394637208 | 0.892707046 | down |
| RP11-3K24.2 | -0.394637208 | 0.892707046 | down |
| AC079325.5 | -0.394637208 | 0.892707046 | down |
| RP11-683L23.6 | -0.394637208 | 0.892707046 | down |
| RP11-78F17.3 | -0.394637208 | 0.892707046 | down |
| BOD1P2 | -0.394637208 | 0.892707046 | down |
| LRRC30 | -0.394637208 | 0.892707046 | down |
| RP11-856M7.2 | -0.394637208 | 0.892707046 | down |
| RP11-794M8.2 | -0.394637208 | 0.892707046 | down |
| RPS4XP18 | -0.394637208 | 0.892707046 | down |
| RAC1P1 | -0.394637208 | 0.892707046 | down |
| NA | -0.394637208 | 0.892707046 | down |
| NA | -0.394637208 | 0.892707046 | down |
| MIR4527 | -0.394637208 | 0.892707046 | down |
| NA | -0.394637208 | 0.892707046 | down |
| Y_RNA | -0.394637208 | 0.892707046 | down |
| CTD-2526M8.3 | -0.394637208 | 0.892707046 | down |
| RP11-389J22.3 | -0.394637208 | 0.892707046 | down |
| MIR548AV | -0.394637208 | 0.892707046 | down |
| Y_RNA | -0.394637208 | 0.892707046 | down |
| OR4G3P | -0.394637208 | 0.892707046 | down |
| AC093230.2 | -0.394637208 | 0.892707046 | down |
| Y_RNA | -0.394637208 | 0.892707046 | down |
| CTD-2265O21.7 | -0.394637208 | 0.892707046 | down |
| VN1R78P | -0.394637208 | 0.892707046 | down |
| CTC-457E21.10 | -0.394637208 | 0.892707046 | down |
| RN7SL150P | -0.394637208 | 0.892707046 | down |
| AC005176.3 | -0.394637208 | 0.892707046 | down |
| DNAJC19P3 | -0.394637208 | 0.892707046 | down |
| CEACAMP8 | -0.394637208 | 0.892707046 | down |
| RN7SL836P | -0.394637208 | 0.892707046 | down |
| AC006262.10 | -0.394637208 | 0.892707046 | down |
| RNU6-924P | -0.394637208 | 0.892707046 | down |

| | | | |
|---|---|---|---|
| MIR520A | -0.394637208 | 0.892707046 | down |
| MIR517B | -0.394637208 | 0.892707046 | down |
| TGM3 | -0.394637208 | 0.892707046 | down |
| RN7SL555P | -0.394637208 | 0.892707046 | down |
| RP5-828H9.4 | -0.394637208 | 0.892707046 | down |
| RP4-764O22.1 | -0.394637208 | 0.892707046 | down |
| RP5-859I17.2 | -0.394637208 | 0.892707046 | down |
| NA | -0.394637208 | 0.892707046 | down |
| RN7SKP111 | -0.394637208 | 0.892707046 | down |
| Y_RNA | -0.394637208 | 0.892707046 | down |
| NA | -0.394637208 | 0.892707046 | down |
| ZNF877P | -0.394637208 | 0.892707046 | down |
| RP5-984P4.1 | -0.394637208 | 0.892707046 | down |
| CST12P | -0.394637208 | 0.892707046 | down |
| U6 | -0.394637208 | 0.892707046 | down |
| RN7SL680P | -0.394637208 | 0.892707046 | down |
| HSPE1P1 | -0.394637208 | 0.892707046 | down |
| RNU6-1251P | -0.394637208 | 0.892707046 | down |
| EPPIN-WFDC6 | -0.394637208 | 0.892707046 | down |
| SPINT5P | -0.394637208 | 0.892707046 | down |
| RP5-998H6.2 | -0.394637208 | 0.892707046 | down |
| LINC01523 | -0.394637208 | 0.892707046 | down |
| RNU6-347P | -0.394637208 | 0.892707046 | down |
| BMP7-AS1 | -0.394637208 | 0.892707046 | down |
| RP4-806M20.3 | -0.394637208 | 0.892707046 | down |
| NA | -0.394637208 | 0.892707046 | down |
| SNORA70 | -0.394637208 | 0.892707046 | down |
| MIR548XHG | -0.394637208 | 0.892707046 | down |
| AL157359.4 | -0.394637208 | 0.892707046 | down |
| RPL37P4 | -0.394637208 | 0.892707046 | down |
| C1QBPP1 | -0.394637208 | 0.892707046 | down |
| AP000705.6 | -0.394637208 | 0.892707046 | down |
| AP001342.1 | -0.394637208 | 0.892707046 | down |
| AP000233.3 | -0.394637208 | 0.892707046 | down |
| KRTAP6-2 | -0.394637208 | 0.892707046 | down |
| KRTAP22-1 | -0.394637208 | 0.892707046 | down |
| KRTAP21-2 | -0.394637208 | 0.892707046 | down |
| KRTAP8-1 | -0.394637208 | 0.892707046 | down |
| SNORA70 | -0.394637208 | 0.892707046 | down |
| AP001437.1 | -0.394637208 | 0.892707046 | down |
| SNORA62 | -0.394637208 | 0.892707046 | down |
| YRDCP3 | -0.394637208 | 0.892707046 | down |
| NA | -0.394637208 | 0.892707046 | down |
| OR11H1 | -0.394637208 | 0.892707046 | down |
| KB-67B5.17 | -0.394637208 | 0.892707046 | down |
| AC007064.22 | -0.394637208 | 0.892707046 | down |
| CLCP1 | -0.394637208 | 0.892707046 | down |
| NA | -0.394637208 | 0.892707046 | down |
| NA | -0.394637208 | 0.892707046 | down |
| E2F6P3 | -0.394637208 | 0.892707046 | down |
| NA | -0.394637208 | 0.892707046 | down |

| | | | |
|---|---|---|---|
| Metazoa_SRP | -0.394637208 | 0.892707046 | down |
| L22NC03-48A11.1 | -0.394637208 | 0.892707046 | down |
| LL22NC03-84E4.8 | -0.394637208 | 0.892707046 | down |
| POM121L10P | -0.394637208 | 0.892707046 | down |
| ARL5AP4 | -0.394637208 | 0.892707046 | down |
| CTA-390C10.9 | -0.394637208 | 0.892707046 | down |
| RNU6-114P | -0.394637208 | 0.892707046 | down |
| Y_RNA | -0.394637208 | 0.892707046 | down |
| SETP15 | -0.394637208 | 0.892707046 | down |
| RP11-716A19.3 | -0.394637208 | 0.892707046 | down |
| RP1-258N20.3 | -0.394637208 | 0.892707046 | down |
| GS1-46604.3 | -0.394637208 | 0.892707046 | down |
| RP11-702C7.1 | -0.394637208 | 0.892707046 | down |
| MAGEB10 | -0.394637208 | 0.892707046 | down |
| RP11-12D5.4 | -0.394637208 | 0.892707046 | down |
| SNORD74 | -0.394637208 | 0.892707046 | down |
| PIGFP3 | -0.394637208 | 0.892707046 | down |
| NA | -0.394637208 | 0.892707046 | down |
| GAGE1 | -0.394637208 | 0.892707046 | down |
| PYY3 | -0.394637208 | 0.892707046 | down |
| NA | -0.394637208 | 0.892707046 | down |
| RP11-552J9.1 | -0.394637208 | 0.892707046 | down |
| RNA5SP504 | -0.394637208 | 0.892707046 | down |
| RP13-77O11.8 | -0.394637208 | 0.892707046 | down |
| MTCO1P52 | -0.394637208 | 0.892707046 | down |
| KRT8P27 | -0.394637208 | 0.892707046 | down |
| RP11-291O7.1 | -0.394637208 | 0.892707046 | down |
| RP11-322A17.1 | -0.394637208 | 0.892707046 | down |
| RP5-928E24.4 | -0.394637208 | 0.892707046 | down |
| TPMTP4 | -0.394637208 | 0.892707046 | down |
| RP1-138A5.2 | -0.394637208 | 0.892707046 | down |
| RP13-24G8.2 | -0.394637208 | 0.892707046 | down |
| CCNB1IP1P3 | -0.394637208 | 0.892707046 | down |
| RP3-377O6.2 | -0.394637208 | 0.892707046 | down |
| MTND6P13 | -0.394637208 | 0.892707046 | down |
| TCEAL3-AS1 | -0.394637208 | 0.892707046 | down |
| RP13-483F6.2 | -0.394637208 | 0.892707046 | down |
| QTRT1P1 | -0.394637208 | 0.892707046 | down |
| RPL36AP53 | -0.394637208 | 0.892707046 | down |
| DANT1 | -0.394637208 | 0.892707046 | down |
| RPL12P43 | -0.394637208 | 0.892707046 | down |
| RP4-555N2.4 | -0.394637208 | 0.892707046 | down |
| RP11-417D4.1 | -0.394637208 | 0.892707046 | down |
| LOXNC01-220B11. | -0.394637208 | 0.892707046 | down |
| RP1-203C2.4 | -0.394637208 | 0.892707046 | down |
| RPL7AP72 | -0.394637208 | 0.892707046 | down |
| RN7SL191P | -0.394637208 | 0.892707046 | down |
| OR7L1P | -0.394637208 | 0.892707046 | down |
| RP1-197O17.3 | -0.394637208 | 0.892707046 | down |
| Y_RNA | -0.394637208 | 0.892707046 | down |
| MIR542 | -0.394637208 | 0.892707046 | down |

| | | | | |
|---|---|---|---|---|
| MAGEC1 | -0.394637208 | 0.892707046 | | down |
| RP3-526F5.2 | -0.394637208 | 0.892707046 | | down |
| HNRNPCP10 | -0.394637208 | 0.892707046 | | down |
| MIR509-2 | -0.394637208 | 0.892707046 | | down |
| MIR509-3 | -0.394637208 | 0.892707046 | | down |
| MIR509-1 | -0.394637208 | 0.892707046 | | down |
| RP5-824H1.1 | -0.394637208 | 0.892707046 | | down |
| RNU6-764P | -0.394637208 | 0.892707046 | | down |
| NA | -0.394637208 | 0.892707046 | | down |
| RP13-228J13.9 | -0.394637208 | 0.892707046 | | down |
| RBMY2HP | -0.394637208 | 0.892707046 | | down |
| TSPY1 | -0.394637208 | 0.892707046 | | down |
| RNA5SP519 | -0.394637208 | 0.892707046 | | down |
| RP1-85D24.3 | -0.394637208 | 0.892707046 | | down |
| RP11-295P22.2 | -0.394637208 | 0.892707046 | | down |
| RBMY2FP | -0.394637208 | 0.892707046 | | down |
| RP11-823E8.3 | -0.394612252 | 0.129378165 | 0.242424696 | down |
| HSDL2 | -0.39459674 | 0.022870778 | 0.068482101 | down |
| ARHGAP4 | -0.39448249 | 0.126694786 | 0.238931526 | down |
| RP11-468E2.10 | -0.39437931 | 0.281786604 | 0.418218482 | down |
| DDX43 | -0.394360596 | 0.215770604 | 0.346886345 | down |
| SLC29A3 | -0.394312315 | 0.051962273 | 0.125636128 | down |
| AC111200.7 | -0.394212527 | 0.544222005 | 0.665659973 | down |
| AC114730.5 | -0.394174938 | 0.691421738 | | down |
| RPS27AP12 | -0.3940601 | 0.57215341 | 0.689536216 | down |
| DHX33 | -0.393976682 | 0.021119822 | 0.06464327 | down |
| KANK1 | -0.393916751 | 0.123551241 | 0.234786432 | down |
| ENTPD6 | -0.393880057 | 0.069346998 | 0.155278466 | down |
| PLA2G12AP1 | -0.393699518 | 0.416090324 | 0.552395269 | down |
| RP11-540A21.2 | -0.393697147 | 0.329770379 | 0.46820194 | down |
| RNF135 | -0.393693739 | 0.002901431 | 0.014121268 | down |
| RP11-523H20.3 | -0.393651922 | 0.344339091 | 0.482839711 | down |
| LRRN1 | -0.39360278 | 0.350461573 | 0.489164576 | down |
| YPEL1 | -0.393561337 | 0.135423122 | 0.2502554 | down |
| CYSRT1 | -0.393505556 | 0.253070933 | 0.388040375 | down |
| PRPF6 | -0.393398564 | 0.006147407 | 0.025424672 | down |
| RP11-338K17.5 | -0.393390905 | 0.433910766 | 0.568803833 | down |
| PIANP | -0.393242316 | 0.139715175 | 0.255920176 | down |
| BIRC5 | -0.393115159 | 0.143772939 | 0.26118895 | down |
| RP11-8P13.5 | -0.392991362 | 0.444753382 | 0.578247358 | down |
| KLHDC4 | -0.39285869 | 0.069422643 | 0.155373502 | down |
| RNLS | -0.392742175 | 0.096870216 | 0.197567631 | down |
| AC093388.3 | -0.392425099 | 0.175200039 | 0.299664909 | down |
| HMCN2 | -0.39237627 | 0.098051416 | 0.199212495 | down |
| MLXIP | -0.392355695 | 0.000870863 | 0.005420386 | down |
| RP4-758J18.10 | -0.392249085 | 0.227625055 | 0.359802555 | down |
| TMEM169 | -0.392195565 | 0.240234231 | 0.373981408 | down |
| CPEB3 | -0.392072686 | 0.068229506 | 0.153436868 | down |
| EFHD2 | -0.392041529 | 0.008307997 | 0.031829311 | down |
| ITLN1 | -0.392035272 | 0.388492589 | 0.52609174 | down |
| RP11-134G8.5 | -0.39202651 | 0.074616479 | 0.163729003 | down |

| | | | | |
|---|---|---|---|---|
| RP11-161M6.2 | -0.39184052 | 0.162796351 | 0.284846547 | down |
| EIF3CL | -0.39183727 | 0.175392128 | 0.299896483 | down |
| SCCPDH | -0.391836774 | 0.007674794 | 0.03002332 | down |
| NAT1 | -0.391778962 | 0.046260388 | 0.115435798 | down |
| PPP1R13B | -0.39172953 | 0.000144299 | 0.001278858 | down |
| NUDT13 | -0.391619092 | 0.093021208 | 0.191906143 | down |
| LUZP4P1 | -0.391618062 | 0.819628516 | | down |
| SIAE | -0.391540296 | 0.130298902 | 0.243709159 | down |
| NA | -0.391498665 | 0.888403323 | | down |
| AC005150.1 | -0.391498634 | 0.887778031 | | down |
| RNU6-484P | -0.391498056 | 0.873898204 | | down |
| RN7SL202P | -0.391498031 | 0.873147451 | | down |
| TRAPPC13P1 | -0.391497261 | 0.842141268 | | down |
| RP11-123M21.2 | -0.391496998 | 0.824906001 | | down |
| CTD-2349P21.1 | -0.39149677 | 0.804276936 | | down |
| NA | -0.391496764 | 0.803577527 | | down |
| RNA5SP195 | -0.391496618 | 0.785624281 | | down |
| SCARA5 | -0.391430642 | 0.158821046 | 0.279893214 | down |
| CTD-2027I19.2 | -0.391414317 | 0.399658155 | 0.536568487 | down |
| ATRN | -0.391198335 | 0.183775501 | 0.309596902 | down |
| RP1-20N2.8 | -0.391155785 | 0.570031791 | | down |
| RP5-832C2.5 | -0.391067591 | 0.207874226 | 0.33804812 | down |
| NA | -0.391064389 | 0.221395089 | 0.353057009 | down |
| ZFHX3 | -0.391036493 | 0.097748213 | 0.198815252 | down |
| LINC01366 | -0.390894502 | 0.38138025 | 0.519245399 | down |
| KANSL1-AS1 | -0.390822654 | 0.104064797 | 0.207700974 | down |
| RPL5P5 | -0.390618487 | 0.392037414 | 0.529555439 | down |
| OGFOD2 | -0.390524224 | 0.418188063 | 0.554312281 | down |
| RTN2 | -0.390222533 | 0.102256139 | 0.205263809 | down |
| LGALS12 | -0.390157519 | 0.363032287 | 0.501445544 | down |
| RP11-222N13.1 | -0.390018725 | 0.734736662 | | down |
| RASSF8-AS1 | -0.390010084 | 0.172414262 | 0.296695922 | down |
| STEAP1B | -0.389872046 | 0.355947846 | 0.494478988 | down |
| PLPP6 | -0.389776331 | 0.024253923 | 0.071678621 | down |
| NNAT | -0.389711797 | 0.31467396 | 0.453345376 | down |
| EEF1A1P22 | -0.389437212 | 0.448000713 | 0.581188023 | down |
| RPS13P2 | -0.389395611 | 0.127791196 | 0.240320399 | down |
| AC093642.1 | -0.389345448 | 0.426492361 | 0.562120486 | down |
| B3GNT7 | -0.389321467 | 0.21298684 | 0.343815291 | down |
| SLC7A5P1 | -0.389153691 | 0.520534323 | 0.644931057 | down |
| LINC01427 | -0.389131033 | 0.85880517 | | down |
| RPLP0 | -0.389036269 | 0.04051432 | 0.104937312 | down |
| LINC01554 | -0.388992296 | 0.310770493 | 0.44920802 | down |
| RP11-778D9.12 | -0.388989028 | 0.373421263 | 0.511888873 | down |
| GSTM1 | -0.38895836 | 0.674447421 | 0.773666462 | down |
| LRG1 | -0.388877608 | 0.295723143 | 0.433188986 | down |
| COX7C | -0.388547944 | 0.006553057 | 0.026661161 | down |
| ZNF589 | -0.388427914 | 0.03501072 | 0.094080532 | down |
| HIP1R | -0.388396725 | 0.012307208 | 0.042987384 | down |
| WARS2-IT1 | -0.388396386 | 0.541279714 | 0.662897755 | down |
| SHC4 | -0.38834749 | 0.343455735 | 0.481825929 | down |

| | | | | |
|---|---|---|---|---|
| RP11-687E1.2 | -0.388227967 | 0.551295766 | 0.671453056 | down |
| RP11-566K11.7 | -0.388094837 | 0.692620933 | | down |
| RP5-906C1.1 | -0.388085811 | 0.346649212 | 0.485399371 | down |
| CH17-431G21.1 | -0.388076691 | 0.259830677 | 0.395038351 | down |
| FNBP1P1 | -0.388054097 | 0.462442618 | 0.594211534 | down |
| GOT2 | -0.387970252 | 0.002813857 | 0.013790353 | down |
| RP11-448G4.2 | -0.38792487 | 0.592552525 | 0.70743684 | down |
| RTN4R | -0.38786247 | 0.102332616 | 0.205348725 | down |
| PSEN1 | -0.387605872 | 0.004065618 | 0.018476391 | down |
| RP3-325F22.3 | -0.387552649 | 0.420957422 | 0.556674791 | down |
| LINC01094 | -0.38736001 | 0.244652916 | 0.378688934 | down |
| RP11-426C22.5 | -0.387185317 | 0.325694839 | 0.464174358 | down |
| OXLD1 | -0.38710874 | 0.058420792 | 0.137116261 | down |
| PGM5 | -0.387003435 | 0.231487505 | 0.364107146 | down |
| CNKSR1 | -0.386888472 | 0.055785206 | 0.132501442 | down |
| PGLYRP4 | -0.386866578 | 0.54162598 | 0.663240715 | down |
| TMEM130 | -0.386760734 | 0.249453104 | 0.384041458 | down |
| SNX27 | -0.386706627 | 0.104102625 | 0.207762672 | down |
| POLR2J2 | -0.38668311 | 0.390178852 | 0.527923145 | down |
| RP11-486O13.5 | -0.386655864 | 0.650203968 | | down |
| TOP3A | -0.386574837 | 0.008642011 | 0.032766352 | down |
| RP11-1H15.2 | -0.386354337 | 0.801528435 | 0.868567456 | down |
| MMP11 | -0.386324437 | 0.056671368 | 0.134023502 | down |
| EFCAB10 | -0.38622238 | 0.511013121 | 0.63683749 | down |
| SNAI3-AS1 | -0.3861544 | 0.083025223 | 0.177059185 | down |
| RP11-38M8.1 | -0.386092185 | 0.258563903 | 0.393670192 | down |
| AC004381.8 | -0.385961874 | 0.540409041 | 0.662209366 | down |
| CTD-2528L19.3 | -0.38589642 | 0.734059678 | | down |
| ZNF771 | -0.385878458 | 0.063621297 | 0.145855647 | down |
| RP11-420A6.2 | -0.385628994 | 0.612195709 | 0.723556565 | down |
| C3orf67 | -0.385558217 | 0.396616643 | 0.533534092 | down |
| uc_338 | -0.385518378 | 0.612131765 | | down |
| KCTD3 | -0.385492773 | 0.153607081 | 0.273301619 | down |
| FAM169A | -0.385431425 | 0.391864381 | 0.529446194 | down |
| CCDC169 | -0.38539055 | 0.605050154 | 0.717702963 | down |
| RP11-182I10.1 | -0.385228159 | 0.895250226 | | down |
| SNORA63 | -0.385228159 | 0.895250226 | | down |
| ADH5P3 | -0.385228159 | 0.895250226 | | down |
| RN7SL531P | -0.385228159 | 0.895250226 | | down |
| AC018696.4 | -0.385228159 | 0.895250226 | | down |
| AC105760.3 | -0.385228159 | 0.895250226 | | down |
| RN7SL567P | -0.385228159 | 0.895250226 | | down |
| VPS26BP1 | -0.385228159 | 0.895250226 | | down |
| OR7E43P | -0.385228159 | 0.895250226 | | down |
| NA | -0.385228159 | 0.895250226 | | down |
| RP11-259O2.3 | -0.385228159 | 0.895250226 | | down |
| CTD-2138O14.1 | -0.385228159 | 0.895250226 | | down |
| CTD-2169H9.1 | -0.385228159 | 0.895250226 | | down |
| RP11-1026M7.3 | -0.385228159 | 0.895250226 | | down |
| RP11-528A10.2 | -0.385228159 | 0.895250226 | | down |
| Xbac-BPG299F13.[illegible] | -0.385228159 | 0.895250226 | | down |

| | | | | |
|---|---|---|---|---|
| RP3-497J21.1 | -0.385228159 | 0.895250226 | | down |
| HPVC1 | -0.385228159 | 0.895250226 | | down |
| TUBB3P2 | -0.385228159 | 0.895250226 | | down |
| ENPP7P6 | -0.385228159 | 0.895250226 | | down |
| RP11-134K1.3 | -0.385228159 | 0.895250226 | | down |
| NA | -0.385228159 | 0.895250226 | | down |
| CALML3 | -0.385228159 | 0.895250226 | | down |
| RP11-241I20.1 | -0.385228159 | 0.895250226 | | down |
| RNU7-193P | -0.385228159 | 0.895250226 | | down |
| RP11-167P22.4 | -0.385228159 | 0.895250226 | | down |
| MIR3157 | -0.385228159 | 0.895250226 | | down |
| OR5AP2 | -0.385228159 | 0.895250226 | | down |
| RP11-500M8.7 | -0.385228159 | 0.895250226 | | down |
| CTC-465D4.1 | -0.385228159 | 0.895250226 | | down |
| RP11-560G2.1 | -0.385228159 | 0.895250226 | | down |
| RP11-351O2.2 | -0.385228159 | 0.895250226 | | down |
| HMGB3P7 | -0.385228159 | 0.895250226 | | down |
| RP11-219E7.2 | -0.385228159 | 0.895250226 | | down |
| RP11-463J10.4 | -0.385228159 | 0.895250226 | | down |
| RP11-112N19.2 | -0.385228159 | 0.895250226 | | down |
| CTA-363E6.1 | -0.385228159 | 0.895250226 | | down |
| RP11-405F3.2 | -0.385228159 | 0.895250226 | | down |
| OR1G1 | -0.385228159 | 0.895250226 | | down |
| RP11-637C24.5 | -0.385228159 | 0.895250226 | | down |
| NA | -0.385228159 | 0.895250226 | | down |
| CGB3 | -0.385228159 | 0.895250226 | | down |
| LLNLR-285B5.1 | -0.385228159 | 0.895250226 | | down |
| MIR1289-1 | -0.385228159 | 0.895250226 | | down |
| NA | -0.385228159 | 0.895250226 | | down |
| RP11-11M20.2 | -0.385228159 | 0.895250226 | | down |
| GS1-309P15.4 | -0.385228159 | 0.895250226 | | down |
| RP5-884M20.4 | -0.385228159 | 0.895250226 | | down |
| HMGN1P35 | -0.385228159 | 0.895250226 | | down |
| RP13-36G14.3 | -0.385228159 | 0.895250226 | | down |
| RP11-392B6.1 | -0.385227615 | 0.888905806 | | down |
| CLDN16 | -0.385165211 | 0.596196258 | 0.710291077 | down |
| REL | -0.385136083 | 0.227026897 | 0.359159396 | down |
| NA | -0.385052358 | 0.40462156 | 0.541080258 | down |
| NA | -0.385041136 | 0.858903281 | | down |
| MIR212 | -0.384998197 | 0.585300533 | 0.700976486 | down |
| UBR2 | -0.384953168 | 0.058519816 | 0.137239042 | down |
| CTD-2619J13.14 | -0.384886658 | 0.021265513 | 0.064975193 | down |
| RPS26P3 | -0.384883947 | 0.390612062 | 0.528295165 | down |
| RP11-354P11.3 | -0.38487624 | 0.696483812 | | down |
| CTB-50L17.10 | -0.384850538 | 0.020759964 | 0.063826481 | down |
| EIF4E3 | -0.384766509 | 0.04458501 | 0.112441203 | down |
| DHRS9 | -0.384743768 | 0.311716462 | 0.450337054 | down |
| NTN5 | -0.38451682 | 0.231836766 | 0.36454199 | down |
| WRAP53 | -0.384496995 | 0.07916305 | 0.170884802 | down |
| RASL10A | -0.384434692 | 0.182264036 | 0.307827348 | down |
| STOX1 | -0.38441293 | 0.296928295 | 0.434319071 | down |

| | | | | |
|---|---|---|---|---|
| IL5RA | -0.384349209 | 0.338579647 | 0.477034651 | down |
| REM2 | -0.384330231 | 0.23027948 | 0.362679545 | down |
| GLI2 | -0.384326154 | 0.256927941 | 0.39195405 | down |
| RNPC3 | -0.384263909 | 0.26267848 | 0.397978429 | down |
| TNRC6C | -0.384120082 | 0.026877498 | 0.077391943 | down |
| C15orf41 | -0.38379945 | 0.18022233 | 0.30547812 | down |
| TET1 | -0.383658805 | 0.314299489 | 0.452936198 | down |
| NA | -0.383647745 | 0.7215627 | | down |
| CTD-2517M22.14 | -0.383633904 | 0.341125224 | 0.479481868 | down |
| NQO1 | -0.383587335 | 0.158814001 | 0.279893214 | down |
| DDRGK1 | -0.383536957 | 0.025998028 | 0.075454966 | down |
| RP11-156E8.1 | -0.383535408 | 0.305896116 | 0.444235446 | down |
| WNT5A-AS1 | -0.383417672 | 0.429212547 | 0.564516997 | down |
| LINC01311 | -0.383407927 | 0.255419377 | 0.390537455 | down |
| CTA-212D2.2 | -0.383385407 | 0.708825988 | | down |
| C1RL-AS1 | -0.383297272 | 0.100506266 | 0.202658407 | down |
| VSIG10L | -0.383289548 | 0.166789041 | 0.289905117 | down |
| RP11-467D6.1 | -0.383204056 | 0.203091157 | 0.332534238 | down |
| MED25 | -0.383194153 | 0.014876618 | 0.049650422 | down |
| RSPH1 | -0.383065814 | 0.156060223 | 0.276389884 | down |
| TMSB10 | -0.382856544 | 0.072608663 | 0.160637309 | down |
| NUDT6 | -0.38283372 | 0.128590678 | 0.241491333 | down |
| NACA3P | -0.382811836 | 0.218201808 | 0.349579007 | down |
| ANKRD44 | -0.382804563 | 0.133919864 | 0.248149032 | down |
| ZNF70 | -0.382715198 | 0.083724124 | 0.177968558 | down |
| HHIP-AS1 | -0.382706244 | 0.287971612 | 0.425328642 | down |
| NA | -0.382617439 | 0.633082379 | | down |
| SAMSN1-AS1 | -0.38260169 | 0.690666488 | | down |
| B4GALNT4 | -0.382534039 | 0.139700961 | 0.255912864 | down |
| RP11-654D12.2 | -0.382364198 | 0.61473575 | 0.725873325 | down |
| NA | -0.382159302 | 0.41280641 | 0.549457549 | down |
| RP11-292D4.2 | -0.382089894 | 0.896098711 | | down |
| NA | -0.382089894 | 0.896098711 | | down |
| RNU6-422P | -0.382089894 | 0.896098711 | | down |
| RNU6-597P | -0.382089894 | 0.896098711 | | down |
| NA | -0.382089563 | 0.889653196 | | down |
| RP11-69H7.4 | -0.382089006 | 0.875397449 | | down |
| DPPA2 | -0.382088976 | 0.87447111 | | down |
| AQP7P3 | -0.382088949 | 0.873609391 | | down |
| RN7SKP239 | -0.382088292 | 0.845219077 | | down |
| ACTG1P23 | -0.382088273 | 0.844075276 | | down |
| CAMTA1-IT1 | -0.382088256 | 0.843025375 | | down |
| RN7SL344P | -0.382088247 | 0.842470489 | | down |
| RP11-46021.2 | -0.382088049 | 0.828577 | | down |
| RN7SL449P | -0.382088046 | 0.828331872 | | down |
| RP11-702F3.1 | -0.382087859 | 0.810894359 | | down |
| CTB-35F21.3 | -0.382087858 | 0.810883809 | | down |
| Y_RNA | -0.382087616 | 0.777898955 | | down |
| LDHAP3 | -0.382087531 | 0.761362143 | | down |
| CTD-2218G20.1 | -0.382087378 | 0.719452616 | | down |
| CTD-307407.5 | -0.38204138 | 0.112478794 | 0.219635969 | down |

| | | | | |
|---|---|---|---|---|
| RP11-307C19.2 | -0.381822789 | 0.619917204 | 0.730240834 | down |
| FIBP | -0.381820642 | 0.006611091 | 0.026822907 | down |
| RP11-253E3.1 | -0.381498562 | 0.239563348 | 0.373189206 | down |
| CDKN2AIPNL | -0.381429536 | 0.000958146 | 0.00585449 | down |
| MCF2L-AS1 | -0.381394521 | 0.257885694 | 0.39289644 | down |
| PIK3CB | -0.381336659 | 0.00721167 | 0.02860296 | down |
| AIMP2 | -0.381205717 | 0.052618557 | 0.126825583 | down |
| HLF | -0.381110798 | 0.274190558 | 0.410554753 | down |
| NA | -0.381083616 | 0.779424314 | | down |
| NA | -0.381065298 | 0.764334816 | | down |
| TADA2A | -0.380957677 | 0.000263186 | 0.002097388 | down |
| NA | -0.380952977 | 0.493304691 | 0.621393071 | down |
| MYB | -0.38094328 | 0.119497272 | 0.229520902 | down |
| PEF1 | -0.380847911 | 0.063509034 | 0.145697815 | down |
| KIAA1671 | -0.380746574 | 0.048141629 | 0.119003381 | down |
| BCAS3 | -0.380640943 | 0.005999225 | 0.024950264 | down |
| RRS1-AS1 | -0.380583291 | 0.590076469 | | down |
| RP11-129K12.1 | -0.38056086 | 0.468173435 | 0.599214027 | down |
| AC005082.12 | -0.380486015 | 0.377102355 | 0.515265743 | down |
| BAP1 | -0.38039617 | 0.000532424 | 0.003663469 | down |
| NRADDP | -0.380308537 | 0.328624977 | 0.467097157 | down |
| EWSAT1 | -0.380295108 | 0.605711184 | 0.718209283 | down |
| RP11-325L12.6 | -0.380272795 | 0.342282668 | 0.480674042 | down |
| FASTKD2 | -0.380266895 | 0.009478123 | 0.035173015 | down |
| KRT19P1 | -0.380162041 | 0.477683299 | 0.607420199 | down |
| FSD1 | -0.380129992 | 0.148323039 | 0.267032379 | down |
| ITGB4 | -0.380052431 | 0.085605353 | 0.181057494 | down |
| RP4-555D20.2 | -0.37981314 | 0.429789215 | 0.565003377 | down |
| NTM | -0.37966137 | 0.277087521 | 0.41329891 | down |
| RP11-327P2.5 | -0.379519212 | 0.040268894 | 0.104458526 | down |
| LYRM9 | -0.379179028 | 0.029674933 | 0.083167322 | down |
| ANGPTL6 | -0.379115438 | 0.337153592 | 0.475404446 | down |
| NELFB | -0.379081675 | 0.013373147 | 0.045719483 | down |
| LYSMD1 | -0.379005923 | 0.008273647 | 0.031734129 | down |
| CTC-510F12.4 | -0.37894566 | 0.332117584 | 0.470489471 | down |
| BSCL2 | -0.378837898 | 0.380004819 | 0.517912574 | down |
| NA | -0.378750932 | 0.282307555 | 0.418846824 | down |
| NTPCR | -0.378582017 | 0.000327399 | 0.002502254 | down |
| NECTIN1 | -0.37857997 | 0.108185356 | 0.213935421 | down |
| JTB | -0.37856107 | 0.245367308 | 0.379286654 | down |
| RP11-317N8.5 | -0.378554472 | 0.596326291 | 0.710333323 | down |
| PIH1D1 | -0.378500676 | 0.022016176 | 0.066647781 | down |
| MLEC | -0.378316545 | 0.111914851 | 0.219015767 | down |
| P2RX7 | -0.378099536 | 0.410354439 | 0.547163535 | down |
| ENGASE | -0.378065626 | 0.007886541 | 0.030644495 | down |
| RNA5SP311 | -0.377997625 | 0.72112302 | | down |
| ZNF346-IT1 | -0.377993098 | 0.453883538 | 0.586459388 | down |
| CLEC10A | -0.377792138 | 0.127886051 | 0.24043858 | down |
| LAT | -0.377716484 | 0.318381039 | 0.457260861 | down |
| RPS5 | -0.377541295 | 0.054973988 | 0.131080921 | down |
| CACNA2D4 | -0.377498779 | 0.043597962 | 0.110779531 | down |

| | | | | |
|---|---|---|---|---|
| SERPINE3 | -0.377438588 | 0.598673941 | 0.712255072 | down |
| CTA-293F17.1 | -0.377416326 | 0.16286965 | 0.284938902 | down |
| NA | -0.377378213 | 0.474452808 | 0.604496359 | down |
| RP1-101A2.1 | -0.377239317 | 0.1404816 | 0.256995019 | down |
| CLNK | -0.377199375 | 0.259425523 | 0.394642047 | down |
| AC005077.14 | -0.377163294 | 0.37628551 | 0.514500608 | down |
| LBX2-AS1 | -0.377069814 | 0.050644996 | 0.123384549 | down |
| THBS3 | -0.377068871 | 0.076794074 | 0.167228866 | down |
| PPFIA2 | -0.377043177 | 0.456574494 | 0.588827442 | down |
| NCF1C | -0.376898635 | 0.223508054 | 0.355576576 | down |
| HINFP | -0.376841287 | 0.000181356 | 0.001542658 | down |
| RP11-356I2.1 | -0.376792333 | 0.665656624 | 0.766303457 | down |
| NUDCD3 | -0.376752881 | 0.004063215 | 0.018471402 | down |
| NT5CP1 | -0.376731797 | 0.660979341 | | down |
| AC011742.5 | -0.376669777 | 0.69657537 | 0.790926319 | down |
| LRRC37A3 | -0.376604945 | 0.117049582 | 0.226063105 | down |
| GPX1 | -0.376549023 | 0.09486714 | 0.194698296 | down |
| CASP6 | -0.376521471 | 0.144262841 | 0.261756051 | down |
| GLTSCR2 | -0.376366435 | 0.023449855 | 0.069824326 | down |
| GOT1 | -0.376346923 | 0.041028592 | 0.105871598 | down |
| SRSF8 | -0.376336552 | 0.008867686 | 0.033465742 | down |
| UBQLNL | -0.376146263 | 0.270815525 | 0.406900461 | down |
| BAX | -0.37613606 | 0.03426265 | 0.092629089 | down |
| CENPB | -0.37591041 | 0.006662981 | 0.027004265 | down |
| TMBIM6 | -0.37582468 | 0.031335109 | 0.086600315 | down |
| RP11-810H18.1 | -0.375819109 | 0.897794484 | | down |
| RNU6-1237P | -0.375819109 | 0.897794484 | | down |
| RP4-592A1.4 | -0.375819109 | 0.897794484 | | down |
| RP11-335E6.3 | -0.375819109 | 0.897794484 | | down |
| RPL23AP90 | -0.375819109 | 0.897794484 | | down |
| AMY1A | -0.375819109 | 0.897794484 | | down |
| LCE1D | -0.375819109 | 0.897794484 | | down |
| IGKV2-4 | -0.375819109 | 0.897794484 | | down |
| NA | -0.375819109 | 0.897794484 | | down |
| AC019050.1 | -0.375819109 | 0.897794484 | | down |
| AC073928.2 | -0.375819109 | 0.897794484 | | down |
| AC017101.10 | -0.375819109 | 0.897794484 | | down |
| AC017081.2 | -0.375819109 | 0.897794484 | | down |
| ATG12P2 | -0.375819109 | 0.897794484 | | down |
| AC113618.1 | -0.375819109 | 0.897794484 | | down |
| PPP2R2DP1 | -0.375819109 | 0.897794484 | | down |
| RN7SL762P | -0.375819109 | 0.897794484 | | down |
| NA | -0.375819109 | 0.897794484 | | down |
| NA | -0.375819109 | 0.897794484 | | down |
| NA | -0.375819109 | 0.897794484 | | down |
| RP11-362I1.2 | -0.375819109 | 0.897794484 | | down |
| RNU6-948P | -0.375819109 | 0.897794484 | | down |
| LINC01262 | -0.375819109 | 0.897794484 | | down |
| Y_RNA | -0.375819109 | 0.897794484 | | down |
| MTHFD2P6 | -0.375819109 | 0.897794484 | | down |
| CTC-552D5.1 | -0.375819109 | 0.897794484 | | down |

| | | | |
|---|---|---|---|
| RP11-461O14.3 | -0.375819109 | 0.897794484 | down |
| RP11-278J6.5 | -0.375819109 | 0.897794484 | down |
| CTC-327F10.5 | -0.375819109 | 0.897794484 | down |
| MIR4634 | -0.375819109 | 0.897794484 | down |
| MUC21 | -0.375819109 | 0.897794484 | down |
| NA | -0.375819109 | 0.897794484 | down |
| NA | -0.375819109 | 0.897794484 | down |
| RP11-503C24.3 | -0.375819109 | 0.897794484 | down |
| RP1-39J2.1 | -0.375819109 | 0.897794484 | down |
| AC005086.2 | -0.375819109 | 0.897794484 | down |
| RP11-235P11.1 | -0.375819109 | 0.897794484 | down |
| NA | -0.375819109 | 0.897794484 | down |
| SNORA51 | -0.375819109 | 0.897794484 | down |
| CTD-2571E19.3 | -0.375819109 | 0.897794484 | down |
| NA | -0.375819109 | 0.897794484 | down |
| NA | -0.375819109 | 0.897794484 | down |
| RP11-218D6.4 | -0.375819109 | 0.897794484 | down |
| RNA5SP310 | -0.375819109 | 0.897794484 | down |
| Y_RNA | -0.375819109 | 0.897794484 | down |
| RPL39P25 | -0.375819109 | 0.897794484 | down |
| NA | -0.375819109 | 0.897794484 | down |
| OR51R1P | -0.375819109 | 0.897794484 | down |
| OR51B4 | -0.375819109 | 0.897794484 | down |
| RP5-916O11.3 | -0.375819109 | 0.897794484 | down |
| OR5M4P | -0.375819109 | 0.897794484 | down |
| OR5B3 | -0.375819109 | 0.897794484 | down |
| SNRPGP19 | -0.375819109 | 0.897794484 | down |
| NA | -0.375819109 | 0.897794484 | down |
| RP11-50B3.1 | -0.375819109 | 0.897794484 | down |
| RNU6-333P | -0.375819109 | 0.897794484 | down |
| RSL24D1P5 | -0.375819109 | 0.897794484 | down |
| RP11-116D17.2 | -0.375819109 | 0.897794484 | down |
| IRX1P1 | -0.375819109 | 0.897794484 | down |
| RP11-131P10.2 | -0.375819109 | 0.897794484 | down |
| NA | -0.375819109 | 0.897794484 | down |
| NA | -0.375819109 | 0.897794484 | down |
| RNU6-1204P | -0.375819109 | 0.897794484 | down |
| IGHVII-1-1 | -0.375819109 | 0.897794484 | down |
| IGHVII-51-2 | -0.375819109 | 0.897794484 | down |
| RP11-603B24.6 | -0.375819109 | 0.897794484 | down |
| RP11-456J20.2 | -0.375819109 | 0.897794484 | down |
| TMCO5A | -0.375819109 | 0.897794484 | down |
| AC109631.1 | -0.375819109 | 0.897794484 | down |
| RP11-540O11.8 | -0.375819109 | 0.897794484 | down |
| NA | -0.375819109 | 0.897794484 | down |
| MTND5P32 | -0.375819109 | 0.897794484 | down |
| RP11-272D12.1 | -0.375819109 | 0.897794484 | down |
| SNORD18 | -0.375819109 | 0.897794484 | down |
| RP11-279O17.1 | -0.375819109 | 0.897794484 | down |
| SNORD71 | -0.375819109 | 0.897794484 | down |
| RNA5SP434 | -0.375819109 | 0.897794484 | down |

| | | | | |
|---|---|---|---|---|
| snoU13 | -0.375819109 | 0.897794484 | | down |
| AC006050.2 | -0.375819109 | 0.897794484 | | down |
| CTD-3194G12.1 | -0.375819109 | 0.897794484 | | down |
| RN7SL735P | -0.375819109 | 0.897794484 | | down |
| RP11-123I22.1 | -0.375819109 | 0.897794484 | | down |
| MIR99B | -0.375819109 | 0.897794484 | | down |
| DKKL1P1 | -0.375819109 | 0.897794484 | | down |
| RP4-785G19.5 | -0.375819109 | 0.897794484 | | down |
| MC3R | -0.375819109 | 0.897794484 | | down |
| CXADRP1 | -0.375819109 | 0.897794484 | | down |
| SNORA3 | -0.375819109 | 0.897794484 | | down |
| RNU6-810P | -0.375819109 | 0.897794484 | | down |
| RP1-302D9.1 | -0.375819109 | 0.897794484 | | down |
| NPM1P8 | -0.375819109 | 0.897794484 | | down |
| FRMD8P1 | -0.375819109 | 0.897794484 | | down |
| SAR1AP4 | -0.375819109 | 0.897794484 | | down |
| AGPAT5P1 | -0.375819109 | 0.897794484 | | down |
| GOLGA6L16P | -0.375819109 | 0.897794484 | | down |
| PDSS1P2 | -0.375818416 | 0.889367337 | | down |
| THRAP3P1 | -0.375818331 | 0.888188388 | | down |
| RP11-193I22.2 | -0.375818244 | 0.88693684 | | down |
| Y_RNA | -0.375818235 | 0.886793865 | | down |
| RPL23AP20 | -0.375817405 | 0.872204891 | | down |
| RP11-1094M14.10 | -0.375816693 | 0.853844194 | | down |
| PER3 | -0.375801384 | 0.151543559 | 0.270825685 | down |
| PIK3IP1-AS1 | -0.375756222 | 0.395186368 | 0.532576162 | down |
| ZNF845 | -0.375703468 | 0.003369455 | 0.01586707 | down |
| DST | -0.375702295 | 0.128037345 | 0.240667511 | down |
| CTC-457L16.2 | -0.375497203 | 0.48945008 | 0.61801455 | down |
| ZNF681 | -0.375454685 | 0.20539487 | 0.33521014 | down |
| RP11-133N21.7 | -0.375405437 | 0.737858818 | | down |
| RP11-439E19.9 | -0.375275494 | 0.277288617 | 0.413462664 | down |
| SMAD9-IT1 | -0.375213315 | 0.691730861 | 0.787297945 | down |
| RP11-252I14.2 | -0.375178212 | 0.749249975 | | down |
| RP13-735L24.1 | -0.375157479 | 0.292847978 | 0.43018008 | down |
| ANAPC11 | -0.375109489 | 0.042290829 | 0.108347034 | down |
| MRPL21 | -0.375057857 | 0.016620997 | 0.053915055 | down |
| CHODL | -0.374967685 | 0.183198877 | 0.309006685 | down |
| LMTK2 | -0.374879209 | 0.094638466 | 0.194454798 | down |
| OAZ1 | -0.374704251 | 0.072558261 | 0.160549447 | down |
| SRD5A1 | -0.374360295 | 0.023712964 | 0.070412363 | down |
| ATP8A1 | -0.374344298 | 0.04225047 | 0.108289808 | down |
| SEMA3A | -0.374183216 | 0.200440185 | 0.329548453 | down |
| GUCD1 | -0.37410286 | 0.078260971 | 0.169463426 | down |
| CTC-550B14.6 | -0.373874987 | 0.42086602 | 0.556642971 | down |
| RP11-402G3.3 | -0.373664007 | 0.74322495 | 0.825793284 | down |
| SNORA26 | -0.373525895 | 0.629497996 | | down |
| PDSS1 | -0.373397071 | 0.219392943 | 0.350813858 | down |
| RP11-1396O13.1 | -0.373236391 | 0.79518585 | | down |
| RP11-286N22.10 | -0.373216482 | 0.502135703 | 0.629009551 | down |
| KRTAP5-5 | -0.373112623 | 0.701062382 | 0.794160252 | down |

| | | | | |
|---|---|---|---|---|
| NCBP2-AS1 | -0.372967742 | 0.588649606 | | down |
| AE000661.50 | -0.372861709 | 0.663884087 | 0.765054409 | down |
| ZNF557 | -0.37281863 | 0.006576963 | 0.02671691 | down |
| LINC01011 | -0.372807812 | 0.134481292 | 0.248989705 | down |
| BAK1 | -0.372791383 | 0.05905178 | 0.138152287 | down |
| RP11-178L8.9 | -0.372719051 | 0.56768789 | 0.685652863 | down |
| RP3-488M23.3 | -0.372702048 | 0.512568402 | 0.637936488 | down |
| LINC00276 | -0.372680844 | 0.898643322 | | down |
| OR2U2P | -0.372680844 | 0.898643322 | | down |
| ASTN2 | -0.37268074 | 0.064673929 | 0.147491252 | down |
| RP11-67H24.2 | -0.372680598 | 0.893774998 | | down |
| NA | -0.372680571 | 0.893200366 | | down |
| RP11-201M22.1 | -0.37268057 | 0.893178824 | | down |
| RPL7AP23 | -0.372680548 | 0.89269779 | | down |
| RP11-392A14.9 | -0.372679338 | 0.849108663 | | down |
| HNRNPA1P76 | -0.37267931 | 0.847417314 | | down |
| TMEM174 | -0.372679071 | 0.82963442 | | down |
| PKNOX2-AS1 | -0.372678812 | 0.801121327 | | down |
| RP11-95M15.2 | -0.372678805 | 0.800146477 | | down |
| PGAM1P6 | -0.372678489 | 0.731120953 | | down |
| ALDH3B1 | -0.37267727 | 0.037192532 | 0.098361186 | down |
| RP11-324L17.1 | -0.372585274 | 0.515451743 | 0.640641714 | down |
| SPATA33 | -0.372530104 | 0.048505606 | 0.119750291 | down |
| NEK3 | -0.372507787 | 0.151861524 | 0.271223851 | down |
| ANXA11 | -0.37247925 | 0.014025645 | 0.047464121 | down |
| JUND | -0.372473539 | 0.05241042 | 0.126455081 | down |
| MYLK | -0.372416928 | 0.382418743 | 0.520164284 | down |
| UBE2L5P | -0.372328842 | 0.469728455 | 0.60037955 | down |
| AC098824.6 | -0.372314893 | 0.651498965 | 0.75524308 | down |
| IGHA1 | -0.37212569 | 0.443911438 | 0.577478902 | down |
| SLC25A39 | -0.371980201 | 0.044540289 | 0.112366146 | down |
| ZNF500 | -0.371970457 | 0.006824741 | 0.027474545 | down |
| RNU4-21P | -0.37191675 | 0.774361183 | 0.848681552 | down |
| PLEKHG3 | -0.371866677 | 0.014867833 | 0.049626617 | down |
| LPAR5 | -0.371739018 | 0.052417638 | 0.12645939 | down |
| MIR4506 | -0.371572163 | 0.720329768 | | down |
| RP5-991G20.1 | -0.371539588 | 0.182891853 | 0.308662107 | down |
| FBXO25 | -0.371365249 | 0.000547513 | 0.003746183 | down |
| CTD-2621I17.8 | -0.371283006 | 0.779275645 | | down |
| MSLNL | -0.37123167 | 0.687756971 | 0.783965187 | down |
| POLE | -0.371143429 | 0.004330906 | 0.019394362 | down |
| RP4-798A10.7 | -0.371138848 | 0.684768361 | | down |
| PSMD13 | -0.371100482 | 0.038435147 | 0.100875335 | down |
| HSPD1P6 | -0.370953955 | 0.556566062 | 0.676250873 | down |
| CTD-2228K2.7 | -0.37078355 | 0.232628012 | 0.365327269 | down |
| ANXA4 | -0.370738047 | 0.123481469 | 0.234698377 | down |
| NOC4L | -0.370725665 | 0.087475763 | 0.183745752 | down |
| BORCS8 | -0.370673896 | 0.100247485 | 0.202299532 | down |
| DES | -0.370589604 | 0.342892817 | 0.481328403 | down |
| C9orf40 | -0.370552507 | 0.070966795 | 0.157841753 | down |
| BEAN1 | -0.370421423 | 0.307998398 | 0.44644685 | down |

| | | | | |
|---|---|---|---|---|
| RP4-753F5.1 | -0.370361956 | 0.648725638 | | down |
| RP11-616M22.12 | -0.370325767 | 0.399653211 | 0.536568487 | down |
| TMEM179 | -0.370257454 | 0.38725479 | 0.524957878 | down |
| TRAK1 | -0.370250524 | 0.012953486 | 0.044609645 | down |
| CTPS2 | -0.370133489 | 0.003065416 | 0.014740294 | down |
| SSNA1 | -0.370118172 | 0.046218185 | 0.115359239 | down |
| CA5BP1 | -0.37005025 | 0.160686174 | 0.282154028 | down |
| RP11-543B16.2 | -0.370005443 | 0.481776171 | 0.610979484 | down |
| CTB-178M22.2 | -0.369999307 | 0.551323595 | 0.671453056 | down |
| RP11-135A1.2 | -0.369953831 | 0.308998727 | 0.447419843 | down |
| OXCT1-AS1 | -0.369769215 | 0.617542936 | 0.728300686 | down |
| ST20-AS1 | -0.369768077 | 0.058137207 | 0.136625571 | down |
| RNF5 | -0.369762617 | 0.039047633 | 0.102083805 | down |
| MXD1 | -0.369438208 | 0.16159395 | 0.283400189 | down |
| ZNF664 | -0.369433066 | 0.142700445 | 0.259747455 | down |
| KBTBD12 | -0.369387544 | 0.278536541 | 0.414808755 | down |
| ZNF180 | -0.369358528 | 0.133301083 | 0.247391774 | down |
| RP11-266L9.6 | -0.369171462 | 0.611336722 | | down |
| FLJ20021 | -0.369164683 | 0.129187075 | 0.242172365 | down |
| SLC47A1 | -0.369164196 | 0.186872839 | 0.313303994 | down |
| RP11-92A5.2 | -0.369099832 | 0.682181822 | 0.779858859 | down |
| ZNF462 | -0.369050494 | 0.080801428 | 0.173461816 | down |
| RPL9 | -0.368928332 | 0.053943036 | 0.129197338 | down |
| CYC1 | -0.368922062 | 0.023460985 | 0.069843622 | down |
| PIGCP1 | -0.36870055 | 0.576215395 | 0.693161932 | down |
| ZNF701 | -0.368665632 | 0.00260999 | 0.013008383 | down |
| APRT | -0.368574517 | 0.105686295 | 0.210238961 | down |
| RP11-59H7.4 | -0.368448245 | 0.440748276 | 0.57442302 | down |
| RP11-67P15.1 | -0.368268104 | 0.521045834 | 0.6453519 | down |
| CTD-2270F17.1 | -0.36821005 | 0.730577037 | 0.816997632 | down |
| RP11-61F12.1 | -0.368063255 | 0.506110426 | 0.632466195 | down |
| ANTXRLP1 | -0.368013378 | 0.544123967 | 0.665594253 | down |
| RP1-30M3.5 | -0.367972025 | 0.244835922 | 0.378770734 | down |
| RP11-1415C14.3 | -0.367922938 | 0.439712952 | 0.573591448 | down |
| CTD-3185P2.1 | -0.36781578 | 0.20747046 | 0.337643817 | down |
| PTGDR | -0.367637135 | 0.345179963 | 0.483770435 | down |
| BRI3BP | -0.367571338 | 0.043814743 | 0.111114373 | down |
| TRGC2 | -0.367511715 | 0.246483638 | 0.380633783 | down |
| PRDM15 | -0.367496831 | 0.14865113 | 0.267371014 | down |
| RP11-517I3.2 | -0.367446444 | 0.262392301 | 0.397645109 | down |
| UBL7-AS1 | -0.367440916 | 0.078424789 | 0.169728541 | down |
| TPM1 | -0.367391882 | 0.266905713 | 0.402616621 | down |
| STK11IP | -0.367222415 | 0.00304959 | 0.014678601 | down |
| XXbac-B476C20.14 | -0.367192629 | 0.708956691 | | down |
| RAB37 | -0.366940707 | 0.052728094 | 0.127007365 | down |
| NIPAL3 | -0.3668776 | 0.004854295 | 0.021207278 | down |
| SPPL3 | -0.366795431 | 0.024219682 | 0.071598555 | down |
| GPI | -0.366757837 | 0.008370961 | 0.032005238 | down |
| MGST1 | -0.366594173 | 0.200002252 | 0.32903667 | down |
| H1F0 | -0.366587739 | 0.104269838 | 0.207982375 | down |
| RASGEF1B | -0.366563539 | 0.055970397 | 0.13285728 | down |

| | | | | |
|---|---|---|---|---|
| CLN5 | -0.366480142 | 0.018222703 | 0.057916312 | down |
| NA | -0.366410059 | 0.900339792 | | down |
| LAPTM4BP1 | -0.366410059 | 0.900339792 | | down |
| TRIM43 | -0.366410059 | 0.900339792 | | down |
| IL36RN | -0.366410059 | 0.900339792 | | down |
| TPMTP2 | -0.366410059 | 0.900339792 | | down |
| RP11-314M24.1 | -0.366410059 | 0.900339792 | | down |
| RP11-271C24.2 | -0.366410059 | 0.900339792 | | down |
| RP11-404G16.2 | -0.366410059 | 0.900339792 | | down |
| OR7E162P | -0.366410059 | 0.900339792 | | down |
| CTC-270D5.1 | -0.366410059 | 0.900339792 | | down |
| RP11-277I20.3 | -0.366410059 | 0.900339792 | | down |
| RP4-668E10.3 | -0.366410059 | 0.900339792 | | down |
| AC021876.4 | -0.366410059 | 0.900339792 | | down |
| NA | -0.366410059 | 0.900339792 | | down |
| TAS2R3 | -0.366410059 | 0.900339792 | | down |
| RP11-434I12.4 | -0.366410059 | 0.900339792 | | down |
| RP11-181D18.4 | -0.366410059 | 0.900339792 | | down |
| RP11-297B17.2 | -0.366410059 | 0.900339792 | | down |
| NA | -0.366410059 | 0.900339792 | | down |
| OR4A49P | -0.366410059 | 0.900339792 | | down |
| RP11-697H9.2 | -0.366410059 | 0.900339792 | | down |
| AP001891.1 | -0.366410059 | 0.900339792 | | down |
| TAS2R42 | -0.366410059 | 0.900339792 | | down |
| SLC25A15P2 | -0.366410059 | 0.900339792 | | down |
| TRAJ11 | -0.366410059 | 0.900339792 | | down |
| RP11-297M9.2 | -0.366410059 | 0.900339792 | | down |
| CTD-2319I12.9 | -0.366410059 | 0.900339792 | | down |
| RP11-264B14.1 | -0.366410059 | 0.900339792 | | down |
| NA | -0.366410059 | 0.900339792 | | down |
| RP11-58G13.1 | -0.366410059 | 0.900339792 | | down |
| FUT5 | -0.366410059 | 0.900339792 | | down |
| NDUFA3P1 | -0.366410059 | 0.900339792 | | down |
| DUXA | -0.366410059 | 0.900339792 | | down |
| EIF5P1 | -0.366410059 | 0.900339792 | | down |
| MAGEA6 | -0.366410059 | 0.900339792 | | down |
| RP11-49I11.3 | -0.366409126 | 0.88823678 | | down |
| RP11-109P11.1 | -0.366409097 | 0.887795063 | | down |
| AC007272.3 | -0.366409071 | 0.887379525 | | down |
| RP11-434I12.5 | -0.366409065 | 0.887283522 | | down |
| RP11-710M11.1 | -0.366409036 | 0.886830681 | | down |
| RNU6-1301P | -0.366409018 | 0.886541107 | | down |
| RP11-3K16.1 | -0.366409002 | 0.886285724 | | down |
| RP11-778O17.4 | -0.366408887 | 0.884367483 | | down |
| RNU1-16P | -0.366408303 | 0.872876749 | | down |
| RP11-461F16.3 | -0.366408294 | 0.872660464 | | down |
| RP11-256L6.2 | -0.366408283 | 0.872425792 | | down |
| PGAM1P11 | -0.366408281 | 0.87236446 | | down |
| PHF5CP | -0.366408267 | 0.872039351 | | down |
| TRBV7-5 | -0.366408259 | 0.871856335 | | down |
| RP11-1149M3.2 | -0.366408226 | 0.87109346 | | down |

| | | | | |
|---|---|---|---|---|
| PPIAP13 | -0.366408223 | 0.871012806 | | down |
| RP11-193E15.4 | -0.366407645 | 0.854663527 | | down |
| TRIM16L | -0.366406401 | 0.056261993 | 0.133328232 | down |
| SKIV2L | -0.366324558 | 0.01690572 | 0.054591647 | down |
| MIR4477B | -0.366260566 | 0.290870501 | 0.428012658 | down |
| MLLT3 | -0.366258082 | 0.009003781 | 0.033856018 | down |
| STAG3L1 | -0.366103541 | 0.47948413 | 0.608911202 | down |
| RP11-1348G14.1 | -0.366083321 | 0.531222557 | 0.654502788 | down |
| RP11-673P17.4 | -0.366079357 | 0.819138511 | 0.881327798 | down |
| FAM134C | -0.366028748 | 0.060058539 | 0.139809984 | down |
| RP11-433J8.1 | -0.365978212 | 0.400045446 | 0.536992466 | down |
| AC004160.4 | -0.365919151 | 0.668356366 | | down |
| MCM7 | -0.365787859 | 0.047659926 | 0.118094418 | down |
| PCDHAC2 | -0.365739944 | 0.669858416 | 0.769665988 | down |
| RPL18A | -0.365689648 | 0.087534999 | 0.183844915 | down |
| CXorf23 | -0.365668587 | 0.014388187 | 0.04838038 | down |
| XYLT2 | -0.365545319 | 0.02127267 | 0.064990452 | down |
| PI4KB | -0.365482575 | 0.019416868 | 0.060735073 | down |
| GABPB1-AS1 | -0.365353744 | 0.160651028 | 0.282134646 | down |
| PRKCD | -0.365205808 | 0.035654 | 0.095309971 | down |
| RP5-890E16.2 | -0.365204019 | 0.397918043 | 0.534949422 | down |
| RP11-57407.1 | -0.365122817 | 0.775697938 | | down |
| RP11-22P6.2 | -0.365034139 | 0.560453822 | 0.679812453 | down |
| PMP2 | -0.364927923 | 0.416688864 | 0.552886112 | down |
| RP11-395L14.3 | -0.364835317 | 0.786653046 | 0.857274624 | down |
| RP1-168L15.6 | -0.364796879 | 0.517231245 | 0.642162715 | down |
| FASTKD1 | -0.364773297 | 0.0060368 | 0.025069318 | down |
| CDA | -0.364734028 | 0.278228606 | 0.414464621 | down |
| NDUFV3 | -0.36471756 | 0.003190932 | 0.015195491 | down |
| TTC28 | -0.364530681 | 0.059198463 | 0.138398462 | down |
| RPL10AP6 | -0.364452413 | 0.102740755 | 0.205920164 | down |
| LINC00894 | -0.36436741 | 0.196199407 | 0.324673629 | down |
| HMGB3P24 | -0.364337333 | 0.480648938 | 0.609976324 | down |
| GATSL2 | -0.364334546 | 0.023045446 | 0.068859015 | down |
| TUBB4B | -0.364300628 | 0.051927032 | 0.125601461 | down |
| NPHS1 | -0.364151262 | 0.516143744 | 0.641183487 | down |
| ARHGAP22 | -0.364146991 | 0.06098936 | 0.141506242 | down |
| RP11-297C4.2 | -0.363989387 | 0.700960279 | 0.79407453 | down |
| FAM163A | -0.363682277 | 0.334170447 | 0.472417509 | down |
| NA | -0.363626291 | 0.362705042 | 0.501131767 | down |
| RP11-332H14.1 | -0.36359566 | 0.420218846 | 0.555951895 | down |
| RP11-359E10.1 | -0.363517434 | 0.435702828 | 0.570431226 | down |
| RPL9P32 | -0.363430411 | 0.202494226 | 0.331791913 | down |
| PRSS46 | -0.363270642 | 0.867053322 | | down |
| LLNLR-276E7.1 | -0.363270365 | 0.851820059 | | down |
| YWHAEP1 | -0.363270177 | 0.837952095 | | down |
| RP11-839G9.1 | -0.36327017 | 0.837306552 | | down |
| RN7SL236P | -0.363270169 | 0.837277849 | | down |
| RP11-534L6.3 | -0.363270165 | 0.836930044 | | down |
| RP11-874G11.1 | -0.363269888 | 0.806032796 | | down |
| KRT71 | -0.363269884 | 0.805357577 | | down |

| | | | | |
|---|---|---|---|---|
| NA | -0.36326987 | 0.803325147 | | down |
| LINC00244 | -0.363269792 | 0.79024422 | | down |
| PRMT1P1 | -0.363269785 | 0.789126115 | | down |
| CTD-2588C8.8 | -0.363269547 | 0.722772533 | | down |
| RP11-359M6.1 | -0.363269479 | 0.688124004 | | down |
| UBQLN4P1 | -0.363106553 | 0.674285019 | | down |
| RP11-692M12.5 | -0.363064647 | 0.442211787 | 0.575791407 | down |
| HIST1H2BN | -0.363060654 | 0.298019791 | 0.435470386 | down |
| TMPRSS13 | -0.362959421 | 0.27713248 | 0.413299028 | down |
| DTX1 | -0.362935404 | 0.253856695 | 0.388887954 | down |
| RP1-15005.3 | -0.36277752 | 0.447356282 | 0.580621479 | down |
| ATP23 | -0.362757321 | 0.084732855 | 0.179566474 | down |
| RP11-24H1.1 | -0.362653394 | 0.638680625 | | down |
| POLD4 | -0.362648527 | 0.064074712 | 0.146545645 | down |
| RP11-122G18.10 | -0.362639522 | 0.660390197 | 0.762224376 | down |
| RPS10P14 | -0.362538951 | 0.742877375 | | down |
| SERTAD4 | -0.36236232 | 0.244621519 | 0.378659844 | down |
| AC007773.2 | -0.362328125 | 0.421358045 | 0.557022144 | down |
| OPCML | -0.362224738 | 0.555928084 | 0.675768869 | down |
| MAPK10 | -0.362216247 | 0.125512871 | 0.237298389 | down |
| ELAC1 | -0.362065768 | 0.00770805 | 0.030122638 | down |
| C14orf132 | -0.3620633 | 0.169657784 | 0.293296419 | down |
| CTC-232P5.1 | -0.362062228 | 0.312558734 | 0.451244243 | down |
| RP11-281015.4 | -0.362025848 | 0.533560943 | 0.656414764 | down |
| RP11-142C4.6 | -0.361838154 | 0.39513035 | 0.532536167 | down |
| POLE2 | -0.361773396 | 0.179384143 | 0.304521256 | down |
| RP11-91K8.5 | -0.361656127 | 0.492960698 | 0.621089961 | down |
| KIF1C | -0.361647514 | 0.037931226 | 0.09985155 | down |
| HOXD11 | -0.361327564 | 0.437441683 | 0.572009833 | down |
| RPL24P8 | -0.361293967 | 0.134464346 | 0.248973672 | down |
| GGT5 | -0.36107938 | 0.177002455 | 0.301941316 | down |
| RNASET2 | -0.361063629 | 0.109862747 | 0.216270238 | down |
| GATA3-AS1 | -0.361037715 | 0.668755349 | 0.768957315 | down |
| TBC1D17 | -0.360989453 | 0.034620986 | 0.093361532 | down |
| PKD1P6 | -0.36091559 | 0.363684576 | 0.502068511 | down |
| AC005363.11 | -0.360778257 | 0.587936352 | 0.703295101 | down |
| UBTFL6 | -0.360756558 | 0.660515812 | | down |
| GMDS | -0.36074947 | 0.102187182 | 0.205152803 | down |
| AC097495.2 | -0.360542073 | 0.655638267 | 0.758344803 | down |
| RPL29 | -0.360525336 | 0.03857621 | 0.101097972 | down |
| DEPTOR | -0.360457511 | 0.174543352 | 0.298955616 | down |
| RP11-857B24.5 | -0.360168609 | 0.698262529 | | down |
| LINC00892 | -0.359801353 | 0.315627083 | 0.454271804 | down |
| LINC01031 | -0.359782927 | 0.678446227 | | down |
| KNL1 | -0.359743095 | 0.18179818 | 0.307355295 | down |
| NA | -0.359671761 | 0.645024988 | | down |
| SULT1C4 | -0.359575272 | 0.296990369 | 0.434388718 | down |
| OXGR1 | -0.359568483 | 0.529507971 | 0.652711504 | down |
| LL0XNC01-36H8.1 | -0.359540812 | 0.592506812 | 0.707410374 | down |
| USP32P3 | -0.359426418 | 0.349249322 | 0.48792419 | down |
| AC023590.1 | -0.359403214 | 0.476246883 | 0.606106721 | down |

| | | | | |
|---|---|---|---|---|
| RP11-881M11.1 | -0.359382757 | 0.697781198 | | down |
| AC005682.6 | -0.359373173 | 0.208960046 | 0.339149458 | down |
| GPR55 | -0.359279111 | 0.233594768 | 0.366252021 | down |
| CASP7 | -0.35921485 | 0.15111274 | 0.270223865 | down |
| SCARB1 | -0.359084231 | 0.035878467 | 0.095765162 | down |
| USP32P2 | -0.358922346 | 0.710302657 | 0.801093623 | down |
| ZNF622 | -0.358865429 | 0.024857698 | 0.072989668 | down |
| RCVRN | -0.358788641 | 0.445265275 | 0.57861375 | down |
| UBQLN4 | -0.358497295 | 0.030885695 | 0.085645336 | down |
| ANKRD18EP | -0.358310381 | 0.531735283 | 0.654934151 | down |
| RPL27A | -0.357991638 | 0.049571681 | 0.121568166 | down |
| CACNB1 | -0.357953455 | 0.030320645 | 0.084464855 | down |
| DYNLL2 | -0.357760878 | 0.002681565 | 0.013279206 | down |
| CHD4 | -0.357755359 | 0.132527372 | 0.24653079 | down |
| SCYL3 | -0.357737047 | 0.007868511 | 0.030606154 | down |
| SCAMP2 | -0.357612703 | 0.061038113 | 0.141597477 | down |
| CDPF1 | -0.357582642 | 0.077416195 | 0.168116527 | down |
| LRRC14 | -0.357537106 | 0.09468271 | 0.194479206 | down |
| PLEKHM3 | -0.357533444 | 0.183728031 | 0.309534288 | down |
| CLGN | -0.357354642 | 0.387357473 | 0.525004192 | down |
| C17orf89 | -0.357306545 | 0.088447429 | 0.185230347 | down |
| LIAS | -0.357295902 | 0.001297811 | 0.007442534 | down |
| IL27 | -0.357281512 | 0.452753614 | 0.585407539 | down |
| CEACAMP3 | -0.357222523 | 0.66648774 | 0.766936982 | down |
| RP11-739L10.1 | -0.357052166 | 0.517635324 | 0.642558181 | down |
| RP11-61K9.3 | -0.357035691 | 0.071186577 | 0.158225162 | down |
| RP11-266K22.2 | -0.357001009 | 0.902886126 | | down |
| NA | -0.357001009 | 0.902886126 | | down |
| BARHL2 | -0.357001009 | 0.902886126 | | down |
| PRDX3P4 | -0.357001009 | 0.902886126 | | down |
| CCDC39-AS1 | -0.357001009 | 0.902886126 | | down |
| RP11-415D17.4 | -0.357001009 | 0.902886126 | | down |
| OR51A9P | -0.357001009 | 0.902886126 | | down |
| NA | -0.357001009 | 0.902886126 | | down |
| RP11-809H16.4 | -0.357001009 | 0.902886126 | | down |
| AP001042.1 | -0.357001009 | 0.902886126 | | down |
| KRTAP10-9 | -0.357001009 | 0.902886126 | | down |
| RP5-1023B21.1 | -0.357001009 | 0.902886126 | | down |
| RP11-152P23.2 | -0.357000066 | 0.890379426 | | down |
| RNU6-757P | -0.357000043 | 0.890027056 | | down |
| RP11-473C18.3 | -0.357000032 | 0.889847599 | | down |
| ZNF849P | -0.35700001 | 0.889487717 | | down |
| HMGN2P40 | -0.356999995 | 0.889237699 | | down |
| COX5AP2 | -0.356999935 | 0.888233347 | | down |
| RPL9P16 | -0.356999253 | 0.874574405 | | down |
| FBXO36P1 | -0.356999246 | 0.874418213 | | down |
| OR10G2 | -0.356999228 | 0.873984844 | | down |
| NA | -0.356999205 | 0.873417349 | | down |
| Y_RNA | -0.356999192 | 0.873093888 | | down |
| NA | -0.356999189 | 0.87301407 | | down |
| RP11-829H16.5 | -0.356999147 | 0.871972988 | | down |

| | | | | |
|---|---|---|---|---|
| RAB40AL | -0.356998686 | 0.858364345 | | down |
| RP11-299J3.6 | -0.356998661 | 0.857504136 | | down |
| PCDH20 | -0.356998661 | 0.85749775 | | down |
| CTD-2373N4.5 | -0.35699863 | 0.856392286 | | down |
| MAGED4 | -0.356998618 | 0.855988079 | | down |
| IGFBP7-AS1 | -0.356883902 | 0.552355319 | 0.67235083 | down |
| RPL23AP49 | -0.356785288 | 0.555068186 | 0.674887437 | down |
| POTEH | -0.356172648 | 0.850946342 | 0.90394216 | down |
| TAOK3 | -0.356116342 | 0.023492765 | 0.069903599 | down |
| UNC5B | -0.356101694 | 0.116615146 | 0.225397931 | down |
| LHFPL5 | -0.355998709 | 0.485145292 | 0.613946555 | down |
| RP11-626G11.1 | -0.355970664 | 0.503513093 | 0.630235694 | down |
| ULBP2 | -0.355688077 | 0.497169009 | 0.624610927 | down |
| POM121L9P | -0.355605023 | 0.316318262 | 0.454907178 | down |
| UNC93B1 | -0.355584947 | 0.148785085 | 0.267478404 | down |
| RP11-613F22.6 | -0.355367232 | 0.792745305 | | down |
| AC144831.1 | -0.355259605 | 0.219002063 | 0.350434273 | down |
| CEP70 | -0.355258048 | 0.01967715 | 0.061293966 | down |
| CTD-3092A11.1 | -0.355239984 | 0.373983302 | 0.512355419 | down |
| RP11-137H2.4 | -0.355001517 | 0.43369836 | 0.568674269 | down |
| RP11-42O15.3 | -0.354988912 | 0.392943577 | 0.530333233 | down |
| RPL5P9 | -0.354939208 | 0.622530468 | 0.732201286 | down |
| POLL | -0.354910061 | 0.041849424 | 0.107555527 | down |
| PFDN5 | -0.35485755 | 0.079211528 | 0.170937903 | down |
| NMNAT1 | -0.354820922 | 0.026887749 | 0.077406625 | down |
| RP11-307O1.1 | -0.35478198 | 0.570743568 | 0.688307658 | down |
| PTCD1 | -0.354749923 | 0.097291008 | 0.198149848 | down |
| TIGD5 | -0.354698832 | 0.015921835 | 0.052233634 | down |
| LA16c-425C2.1 | -0.354689856 | 0.707221759 | | down |
| HMGN2P3 | -0.354686527 | 0.133820242 | 0.248071534 | down |
| RP4-639F20.1 | -0.354677578 | 0.032258427 | 0.088465778 | down |
| C1orf189 | -0.354659531 | 0.618707471 | 0.729187481 | down |
| CCDC157 | -0.354637086 | 0.187191215 | 0.313720589 | down |
| DNAJB1P1 | -0.354439508 | 0.903579303 | | down |
| SNORA54 | -0.354399244 | 0.812556836 | | down |
| RP11-29H23.6 | -0.354399153 | 0.812397685 | | down |
| RP11-381E24.4 | -0.354398813 | 0.811805389 | | down |
| RNA5SP155 | -0.354380543 | 0.782428313 | | down |
| RP11-1348G14.6 | -0.354380313 | 0.782084061 | | down |
| RNU5E-10P | -0.354379982 | 0.781592154 | | down |
| NA | -0.354370974 | 0.768616311 | | down |
| DUXB | -0.354368199 | 0.764776306 | | down |
| RP11-288L9.1 | -0.354356667 | 0.749511583 | | down |
| AC011816.1 | -0.354355183 | 0.747622707 | | down |
| NA | -0.35435086 | 0.742207671 | | down |
| ACBD5 | -0.35433535 | 0.2160223 | 0.347110188 | down |
| CKS1BP7 | -0.354304275 | 0.690606292 | | down |
| CTB-60B18.10 | -0.354102469 | 0.483153032 | 0.61225061 | down |
| RP11-353N14.5 | -0.354094558 | 0.454268393 | 0.586861007 | down |
| SIMC1 | -0.354035876 | 0.107441217 | 0.212800049 | down |
| POFUT1 | -0.35399819 | 0.048894102 | 0.120397868 | down |

| | | | | |
|---|---|---|---|---|
| ADRB3 | -0.353959258 | 0.47751162 | 0.607318539 | down |
| C2orf68 | -0.353579156 | 3.47E-05 | 0.000394187 | down |
| RP11-417L19.6 | -0.353492057 | 0.378969993 | 0.516994709 | down |
| NA | -0.353248664 | 0.252751621 | 0.38778826 | down |
| CLUAP1 | -0.353147787 | 0.046600899 | 0.116083028 | down |
| PPP1R32 | -0.353066855 | 0.141039999 | 0.257702714 | down |
| RP11-49O14.2 | -0.352912989 | 0.6138436 | 0.725073316 | down |
| HMG20B | -0.352891161 | 0.006473361 | 0.026419336 | down |
| SNORA18 | -0.352763638 | 0.871616101 | | down |
| AC021016.7 | -0.352727569 | 0.370166635 | 0.50868071 | down |
| ITGAE | -0.352627051 | 0.011495932 | 0.040830333 | down |
| RP11-439E19.10 | -0.352449586 | 0.4379489 | 0.572399041 | down |
| VN1R1 | -0.352270434 | 0.399502144 | 0.536436853 | down |
| NSUN3 | -0.352149018 | 0.011008237 | 0.039482667 | down |
| R3HCC1 | -0.352099917 | 0.030024428 | 0.083802854 | down |
| TMEM117 | -0.352023435 | 0.136948286 | 0.252251506 | down |
| RP11-160E2.21 | -0.35187675 | 0.718347059 | | down |
| SASS6 | -0.351844429 | 0.161958149 | 0.283886668 | down |
| SLC35A3 | -0.351792717 | 0.077760857 | 0.168633404 | down |
| RANGAP1 | -0.351637937 | 0.10888567 | 0.215008944 | down |
| RP11-46309.9 | -0.351618104 | 0.47587606 | 0.605865767 | down |
| SSBP3-AS1 | -0.351583134 | 0.422531646 | 0.558075385 | down |
| RP11-10J21.2 | -0.35154471 | 0.687060032 | | down |
| ZNF526 | -0.351515494 | 0.022992012 | 0.068733532 | down |
| TRIM14 | -0.351337071 | 0.023470466 | 0.069844163 | down |
| MAP10 | -0.350799434 | 0.329077275 | 0.46750509 | down |
| TSC2 | -0.350588926 | 0.000427741 | 0.003071475 | down |
| RP11-363D14.1 | -0.350404539 | 0.478836904 | 0.608397866 | down |
| RP5-899B16.1 | -0.350378427 | 0.711915258 | | down |
| NA | -0.35032614 | 0.337795703 | 0.476132692 | down |
| C7orf25 | -0.350308963 | 0.430091964 | 0.565315492 | down |
| STARD7-AS1 | -0.35028229 | 0.118458245 | 0.227977677 | down |
| RP11-151A6.6 | -0.350174355 | 0.776454202 | | down |
| TUBD1 | -0.350156401 | 0.087440128 | 0.183722722 | down |
| DECR1 | -0.350077792 | 0.027243406 | 0.078183292 | down |
| PF4 | -0.349892504 | 0.471171808 | 0.601763552 | down |
| PSMC5 | -0.349671525 | 0.04078865 | 0.105379156 | down |
| RP11-626G11.6 | -0.349584048 | 0.348405555 | 0.48713219 | down |
| RP11-33N14.3 | -0.349574047 | 0.583871088 | 0.69971372 | down |
| ANKRD36C | -0.349567392 | 0.336248785 | 0.474395797 | down |
| LRRC73 | -0.34955413 | 0.357791631 | 0.496168555 | down |
| BBC3 | -0.349552905 | 0.085456825 | 0.180807385 | down |
| ARHGEF28 | -0.349503507 | 0.095926278 | 0.196215216 | down |
| HOMER3 | -0.349306613 | 0.180723756 | 0.306069026 | down |
| AC007966.1 | -0.349228762 | 0.685237834 | 0.78216198 | down |
| CTD-2554C21.1 | -0.349202224 | 0.619345529 | 0.729653246 | down |
| MIR1302-10 | -0.349188167 | 0.646967798 | | down |
| RPS27AP1 | -0.349148943 | 0.488471748 | 0.617139258 | down |
| SNRPA | -0.349126468 | 0.108200752 | 0.213951783 | down |
| RP11-977G19.11 | -0.349084151 | 0.414712974 | 0.55123818 | down |
| SAR1B | -0.349053398 | 0.057138307 | 0.134851791 | down |

| | | | | |
|---|---|---|---|---|
| STAT6 | -0.348908462 | 0.009663899 | 0.035686108 | down |
| XXbac-... LO9NC01-139C3.1 | -0.348847593 | 0.486720445 | 0.615447351 | down |
| RP11-809N8.4 | -0.348702505 | 0.45375558 | 0.586331926 | down |
| CTC-548K16.2 | -0.348700455 | 0.389935725 | 0.527641712 | down |
| SDS | -0.348617538 | 0.290594139 | 0.427804459 | down |
| AARD | -0.348568253 | 0.306825213 | 0.445154371 | down |
| RBMX2 | -0.348567314 | 0.047022934 | 0.116892025 | down |
| CWF19L2 | -0.348566145 | 0.06968141 | 0.155824957 | down |
| GFOD1 | -0.348561789 | 0.060040475 | 0.139779624 | down |
| STRA13 | -0.348551985 | 0.077537936 | 0.16825928 | down |
| RP11-1280N14.3 | -0.348410956 | 0.315782879 | 0.454376476 | down |
| PKMP1 | -0.348181313 | 0.620387117 | | down |
| RP11-464F9.22 | -0.348172369 | 0.295178959 | 0.432588718 | down |
| ALG6 | -0.348121252 | 0.013974862 | 0.047324251 | down |
| NXPH4 | -0.347997656 | 0.409056546 | 0.545796281 | down |
| NUPL2 | -0.347768777 | 0.16667621 | 0.289776059 | down |
| RP11-295P9.12 | -0.347755632 | 0.382172588 | 0.519947165 | down |
| RP11-513I15.6 | -0.347732576 | 0.633110398 | 0.740699719 | down |
| CHRNA5 | -0.347614018 | 0.362793761 | 0.501230719 | down |
| CTD-2015C24.1 | -0.347591959 | 0.905433459 | | down |
| RP11-535C7.1 | -0.347591959 | 0.905433459 | | down |
| NA | -0.347591959 | 0.905433459 | | down |
| RP11-346J10.1 | -0.347591959 | 0.905433459 | | down |
| GCNT6 | -0.347591959 | 0.905433459 | | down |
| HLA-DQB3 | -0.347591959 | 0.905433459 | | down |
| RP11-1112C15.2 | -0.347591959 | 0.905433459 | | down |
| MTND5P14 | -0.347591959 | 0.905433459 | | down |
| RNU2-50P | -0.347591959 | 0.905433459 | | down |
| RP11-323A7.1 | -0.347591959 | 0.905433459 | | down |
| RP11-556H2.4 | -0.347591959 | 0.905433459 | | down |
| AP000281.1 | -0.347591959 | 0.905433459 | | down |
| RP11-543H23.2 | -0.347591957 | 0.905414677 | | down |
| RPL37P15 | -0.347591921 | 0.905005795 | | down |
| INMT-FAM188B | -0.347591867 | 0.904381056 | | down |
| Y_RNA | -0.347590911 | 0.890891962 | | down |
| RP11-290L1.5 | -0.3475909 | 0.890703428 | | down |
| ATP1B4 | -0.347590874 | 0.890257422 | | down |
| RP11-169K17.2 | -0.347590856 | 0.889940782 | | down |
| RP11-286M16.1 | -0.347590743 | 0.887887273 | | down |
| AC097711.1 | -0.347590189 | 0.875811783 | | down |
| RNU6-26P | -0.347590169 | 0.875316063 | | down |
| RP11-813F20.4 | -0.347589636 | 0.858828326 | | down |
| NA | -0.347589586 | 0.856902827 | | down |
| EIF4A1P11 | -0.347589227 | 0.840490515 | | down |
| RP11-379F4.1 | -0.347589006 | 0.827075759 | | down |
| SEPT5 | -0.347560629 | 0.592040436 | 0.707022134 | down |
| ZNF704 | -0.347505765 | 0.153044515 | 0.272688258 | down |
| USP33 | -0.347371841 | 0.047204027 | 0.117274272 | down |
| MTHFS | -0.347316129 | 0.356998121 | 0.495420802 | down |
| SORBS2 | -0.347262637 | 0.244007959 | 0.377932818 | down |
| PRKAB2 | -0.347145516 | 0.203151099 | 0.332596132 | down |

| | | | | |
|---|---|---|---|---|
| DNAJC16 | -0.346960971 | 0.009091116 | 0.034099064 | down |
| TSPAN6 | -0.34689629 | 0.158369677 | 0.279392752 | down |
| RP11-96K19.2 | -0.34672408 | 0.458975803 | 0.590909711 | down |
| SLC44A1 | -0.34670294 | 0.055288509 | 0.131519238 | down |
| MRPL35 | -0.346495062 | 0.002432729 | 0.012300387 | down |
| BMP7 | -0.346467726 | 0.159346469 | 0.28051096 | down |
| RP11-75C9.2 | -0.34639346 | 0.90575734 | | down |
| ATP5G1P6 | -0.346371874 | 0.886026158 | | down |
| RP11-493E12.2 | -0.346327785 | 0.586497027 | 0.702009551 | down |
| NA | -0.346313112 | 0.844109351 | | down |
| HS1BP3 | -0.346304244 | 0.007992288 | 0.030955307 | down |
| NA | -0.346293163 | 0.832281869 | | down |
| C4orf45 | -0.34626729 | 0.818143822 | | down |
| RP11-516C1.1 | -0.346266732 | 0.817852124 | | down |
| RP11-32B5.1 | -0.346265166 | 0.817035154 | | down |
| RP11-425D10.10 | -0.346264605 | 0.398769215 | 0.535782045 | down |
| ARPIN | -0.346259937 | 0.067596953 | 0.152365732 | down |
| LINC00315 | -0.346238689 | 0.602523631 | 0.715417933 | down |
| KIAA0196-AS1 | -0.346211628 | 0.791171596 | | down |
| RP3-496C20.1 | -0.346170487 | 0.773485161 | | down |
| GADD45G | -0.346153699 | 0.083971193 | 0.178329719 | down |
| RP11-690I21.2 | -0.346138747 | 0.760842816 | | down |
| AC019118.4 | -0.346138244 | 0.760648608 | | down |
| RP11-77K12.9 | -0.346065943 | 0.385643682 | 0.523391793 | down |
| ZNF829 | -0.346024754 | 0.261395253 | 0.396514136 | down |
| GS1-393G12.13 | -0.346004053 | 0.714226467 | | down |
| RP11-46J23.1 | -0.345985471 | 0.563676061 | 0.682288529 | down |
| TEX29 | -0.345976603 | 0.37976268 | 0.517722956 | down |
| NA | -0.345824089 | 0.73305814 | | down |
| WFDC2 | -0.345788716 | 0.405885546 | 0.542456643 | down |
| IFT172 | -0.345670464 | 0.010187696 | 0.03719518 | down |
| RP11-421N8.1 | -0.345572512 | 0.307177619 | 0.445515057 | down |
| PTPN20 | -0.345419654 | 0.520327185 | 0.644727593 | down |
| LINC01012 | -0.345261254 | 0.303525753 | 0.441549212 | down |
| SIVA1 | -0.345260575 | 0.047499601 | 0.117784583 | down |
| RP11-680F20.10 | -0.345176665 | 0.754942873 | | down |
| RP11-452L6.7 | -0.345032499 | 0.306452683 | 0.444807209 | down |
| DIP2C | -0.344991526 | 0.162297086 | 0.2842517 | down |
| EDC3 | -0.344921286 | 0.014048813 | 0.047505067 | down |
| NA | -0.344689915 | 0.023596691 | 0.070158078 | down |
| CCDC116 | -0.344639154 | 0.330346326 | 0.468813352 | down |
| RNF32 | -0.344569602 | 0.167746728 | 0.291030934 | down |
| RP11-411A19.5 | -0.344392727 | 0.739492279 | 0.82289496 | down |
| GRIK5 | -0.344114364 | 0.218806672 | 0.350230562 | down |
| PHPT1 | -0.344008853 | 0.087649514 | 0.183982532 | down |
| RP11-44F14.8 | -0.343982351 | 0.391448648 | 0.529045577 | down |
| RPL7P23 | -0.343798772 | 0.186616476 | 0.312996451 | down |
| RP3-431P23.5 | -0.343748242 | 0.354137812 | 0.492807616 | down |
| ITGA2B | -0.343589327 | 0.288230687 | 0.425491488 | down |
| SLC18A3 | -0.343431672 | 0.545258935 | 0.666528947 | down |
| ACSM5 | -0.343427186 | 0.342703168 | 0.481174595 | down |

| | | | | |
|---|---|---|---|---|
| NA | -0.343310262 | 0.331346537 | 0.469685084 | down |
| MED29 | -0.343190803 | 0.000271696 | 0.002153784 | down |
| FAM3C | -0.343183133 | 0.219160144 | 0.350601996 | down |
| RPL23AP43 | -0.343167948 | 0.593612413 | | down |
| OPTN | -0.342917213 | 0.009722678 | 0.035854687 | down |
| UBE2FP1 | -0.342853307 | 0.474522855 | 0.604527187 | down |
| INSRR | -0.342849098 | 0.431855663 | 0.566975683 | down |
| OLIG1 | -0.342804149 | 0.6599074 | 0.761815866 | down |
| RP11-767N6.7 | -0.342722836 | 0.143003533 | 0.260151265 | down |
| PAPD5 | -0.342717981 | 0.026780682 | 0.077150141 | down |
| AC098823.3 | -0.342693579 | 0.653194191 | 0.756566031 | down |
| Metazoa_SRP | -0.34265424 | 0.814835487 | | down |
| RP11-25G10.2 | -0.342651492 | 0.56390548 | 0.682347892 | down |
| AP1S3 | -0.342644119 | 0.061425853 | 0.142288228 | down |
| ERICH6-AS1 | -0.342641057 | 0.327257235 | 0.465734279 | down |
| TGM1 | -0.342546109 | 0.334602503 | 0.472850311 | down |
| BCAR3 | -0.342301927 | 0.080391768 | 0.172854096 | down |
| LY6G5B | -0.342212635 | 0.511156408 | 0.636908478 | down |
| CACNA1C | -0.342197784 | 0.221484234 | 0.353154126 | down |
| HCG4P7 | -0.342082782 | 0.316246259 | 0.45486895 | down |
| CLEC9A | -0.341974061 | 0.316161443 | 0.454781456 | down |
| TMEM199 | -0.341771773 | 0.074883487 | 0.164147052 | down |
| PSMC1P1 | -0.341652917 | 0.170489362 | 0.294361381 | down |
| RP11-37C7.3 | -0.341572675 | 0.494120299 | 0.622185677 | down |
| LINC00865 | -0.34119529 | 0.17928869 | 0.304462435 | down |
| Metazoa_SRP | -0.341161103 | 0.32249467 | 0.461155469 | down |
| NA | -0.341069601 | 0.886010399 | | down |
| RP11-516A11.1 | -0.340921069 | 0.589725273 | | down |
| DBT | -0.340804051 | 0.044117669 | 0.111642779 | down |
| SPSB2 | -0.340725914 | 0.047906818 | 0.118549744 | down |
| CXorf67 | -0.340675986 | 0.578947238 | 0.695338429 | down |
| AIG1 | -0.340626734 | 0.027189871 | 0.078074403 | down |
| NA | -0.340620588 | 0.807520446 | | down |
| RP13-895J2.2 | -0.340565182 | 0.777741707 | | down |
| CCDC34 | -0.340545197 | 0.075549067 | 0.165206021 | down |
| MPO | -0.340519396 | 0.382123253 | 0.519947165 | down |
| TOPORS-AS1 | -0.340393757 | 0.225336212 | 0.357426896 | down |
| RP11-240G22.4 | -0.340184535 | 0.790331374 | | down |
| RP11-143I21.1 | -0.340118583 | 0.748212967 | | down |
| RP11-247I13.11 | -0.340082664 | 0.525776802 | 0.649524599 | down |
| RP1-100J12.1 | -0.340036774 | 0.646567231 | | down |
| TUBA3E | -0.340031647 | 0.722577483 | 0.810820128 | down |
| LINC00840 | -0.340022305 | 0.475283843 | 0.60529133 | down |
| LINC00243 | -0.339952706 | 0.451992649 | 0.584850777 | down |
| NA | -0.33977999 | 0.747817403 | | down |
| KBTBD3 | -0.339716144 | 0.033353096 | 0.090679313 | down |
| SLC6A3 | -0.339656141 | 0.632879222 | 0.740503016 | down |
| TRIM28 | -0.3395741 | 0.037545592 | 0.099070768 | down |
| TMX2 | -0.339495067 | 0.059391079 | 0.13864379 | down |
| GPR135 | -0.339478857 | 0.47374301 | 0.604064443 | down |
| RP11-677M14.7 | -0.339385632 | 0.15956278 | 0.280755735 | down |

| | | | | |
|---|---|---|---|---|
| RP11-708J19.1 | -0.339297943 | 0.247309773 | 0.381680528 | down |
| LY86-AS1 | -0.339286667 | 0.444106795 | 0.577641272 | down |
| NA | -0.339155638 | 0.771197343 | | down |
| PDHA1 | -0.339082246 | 0.033911961 | 0.091920874 | down |
| IGHD5-12 | -0.339073046 | 0.803455178 | | down |
| AF064858.8 | -0.339059018 | 0.280165793 | 0.416490954 | down |
| DENND1B | -0.339039362 | 0.022819969 | 0.068382521 | down |
| COG4 | -0.338961761 | 0.015706194 | 0.051729543 | down |
| AC006378.2 | -0.338950424 | 0.371923259 | 0.51023116 | down |
| RPL3 | -0.338939232 | 0.02128306 | 0.065002364 | down |
| RP11-206L10.8 | -0.338893808 | 0.466810715 | 0.597922115 | down |
| C20orf27 | -0.338632293 | 0.148918625 | 0.267645647 | down |
| CTDSP1 | -0.33862898 | 0.009600365 | 0.035495121 | down |
| RP1-12G14.7 | -0.338493097 | 0.514427511 | 0.639683652 | down |
| NAPEPLD | -0.338455402 | 0.020746229 | 0.063809672 | down |
| RPL34P33 | -0.338400155 | 0.426092482 | 0.561692007 | down |
| ENDOG | -0.338384513 | 0.162493019 | 0.284473015 | down |
| RPL7AP11 | -0.338365693 | 0.348200746 | 0.486981808 | down |
| RP11-395N3.2 | -0.338278672 | 0.416116686 | 0.552395269 | down |
| RNF103 | -0.338185326 | 0.054024003 | 0.129320145 | down |
| RPL39P6 | -0.338182909 | 0.907981766 | | down |
| RP1-206D15.5 | -0.338182909 | 0.907981766 | | down |
| NA | -0.338182909 | 0.907981766 | | down |
| CTD-2532K18.1 | -0.338182909 | 0.907981766 | | down |
| LINC01445 | -0.338182909 | 0.907981766 | | down |
| RP11-5P18.1 | -0.338182873 | 0.90756891 | | down |
| NA | -0.3381828 | 0.906719662 | | down |
| RP4-683L5.1 | -0.338182775 | 0.906418954 | | down |
| AC020915.2 | -0.338182708 | 0.905600577 | | down |
| AP001625.5 | -0.338182619 | 0.904491371 | | down |
| MIR4298 | -0.338181827 | 0.892476764 | | down |
| NA | -0.338181753 | 0.891109183 | | down |
| RP11-439A17.10 | -0.338181192 | 0.878681951 | | down |
| RP11-619J20.1 | -0.338181186 | 0.87852606 | | down |
| RPL13AP23 | -0.338181122 | 0.876791384 | | down |
| RN7SL692P | -0.338181099 | 0.876169965 | | down |
| SNORA19 | -0.338180727 | 0.864338525 | | down |
| RP1-287H17.1 | -0.33818068 | 0.862596968 | | down |
| RP11-609L3.2 | -0.338180639 | 0.861038541 | | down |
| NA | -0.338180636 | 0.860903363 | | down |
| OR7E36P | -0.338180628 | 0.860570266 | | down |
| TRAJ17 | -0.338180365 | 0.848827741 | | down |
| RNA5SP78 | -0.338180353 | 0.848259803 | | down |
| AC006960.5 | -0.338180331 | 0.847125033 | | down |
| SNORA80E | -0.338180103 | 0.833599421 | | down |
| MIR4999 | -0.338180084 | 0.832304752 | | down |
| RP11-93O14.3 | -0.338180074 | 0.831625747 | | down |
| CTD-2215E18.2 | -0.33817972 | 0.799738124 | | down |
| AC005740.6 | -0.338161912 | 0.371752073 | 0.510042893 | down |
| RP11-108F13.2 | -0.338021791 | 0.765619204 | | down |
| FAM166B | -0.337933682 | 0.315527227 | 0.454160879 | down |

| | | | | |
|---|---|---|---|---|
| RP4-669L17.10 | -0.33792489 | 0.533597893 | 0.656433343 | down |
| RP13-941N14.1 | -0.337916391 | 0.484447074 | 0.613321306 | down |
| RAB32 | -0.337914406 | 0.060330381 | 0.140313203 | down |
| AAK1 | -0.337894384 | 0.008430641 | 0.032167918 | down |
| FTLP12 | -0.337839757 | 0.74963312 | | down |
| SH3D19 | -0.337838695 | 0.277205128 | 0.413358689 | down |
| GRN | -0.337765731 | 0.133845032 | 0.248086874 | down |
| AC106873.4 | -0.337744754 | 0.828271992 | 0.887803804 | down |
| EXTL3 | -0.337709164 | 0.08277509 | 0.176676243 | down |
| SCO1 | -0.337575678 | 0.01082526 | 0.038978201 | down |
| STARD4-AS1 | -0.33748661 | 0.45060144 | 0.583554018 | down |
| SIGLEC1 | -0.337352401 | 0.250585411 | 0.385251668 | down |
| DAAM2 | -0.337283661 | 0.335324788 | 0.473601279 | down |
| PPP4R1-AS1 | -0.337142441 | 0.625968564 | 0.735092227 | down |
| PNPLA4 | -0.336985239 | 0.116840217 | 0.225774857 | down |
| MMAA | -0.336883641 | 0.002980268 | 0.014423194 | down |
| TSHZ1 | -0.336870346 | 0.225338991 | 0.357426896 | down |
| RP5-1120P11.1 | -0.33680695 | 0.524644896 | 0.648739814 | down |
| RPL32 | -0.336746287 | 0.08613274 | 0.181840493 | down |
| UQCRC2 | -0.336642869 | 0.077076525 | 0.167596955 | down |
| RPSAP31 | -0.336577462 | 0.754400589 | | down |
| NKX3-1 | -0.336450218 | 0.288876432 | 0.426130677 | down |
| ARSA | -0.336422287 | 0.168183157 | 0.291536541 | down |
| COL18A1-AS2 | -0.336388966 | 0.816802543 | | down |
| NDUFAF6 | -0.336357156 | 0.018032779 | 0.057473506 | down |
| SLC39A11 | -0.336193652 | 0.070687457 | 0.157481252 | down |
| CNNM4 | -0.33618543 | 0.205888097 | 0.335796211 | down |
| DCTN5 | -0.336179811 | 0.009225409 | 0.034486523 | down |
| RP11-274A11.3 | -0.336129793 | 0.778114107 | | down |
| AC007285.6 | -0.336061292 | 0.513731689 | 0.639059078 | down |
| SNORD17 | -0.336053458 | 0.492464531 | 0.620601623 | down |
| AK3P3 | -0.3360041 | 0.701480777 | | down |
| ITPA | -0.335834414 | 0.086341351 | 0.182091846 | down |
| RP1-12803.5 | -0.335746467 | 0.877576333 | | down |
| PIP5KL1 | -0.335745477 | 0.314735599 | 0.453368957 | down |
| VEZT | -0.335650234 | 0.017975725 | 0.057331495 | down |
| NA | -0.335632404 | 0.769200591 | | down |
| LLPH-AS1 | -0.335338086 | 0.535519318 | 0.65818343 | down |
| LUCAT1 | -0.335255862 | 0.509834161 | 0.635869382 | down |
| C11orf49 | -0.335229398 | 0.000761721 | 0.00488477 | down |
| FBXO32 | -0.335082203 | 0.175151951 | 0.299664909 | down |
| RP11-1193F23.1 | -0.334690532 | 0.642281008 | 0.747991728 | down |
| RTN4IP1 | -0.334626734 | 0.051298734 | 0.124613452 | down |
| SOX2 | -0.334626655 | 0.5163922 | 0.64143909 | down |
| SIAH1 | -0.334561838 | 0.009466214 | 0.035150529 | down |
| FILIP1 | -0.334389339 | 0.305245905 | 0.443462667 | down |
| ZNF16 | -0.334366089 | 0.051168569 | 0.124417975 | down |
| PPM1N | -0.334300837 | 0.208483194 | 0.338668239 | down |
| RP1-127L4.10 | -0.334216109 | 0.81628593 | | down |
| FRG1BP | -0.334163955 | 0.167203548 | 0.290356823 | down |
| PM20D1 | -0.334086438 | 0.490396669 | 0.618829743 | down |

| | | | | |
|---|---|---|---|---|
| GZMM | -0.33408402 | 0.325385296 | 0.463905274 | down |
| MTCYBP18 | -0.33407581 | 0.560196261 | 0.67955491 | down |
| NA | -0.333942021 | 0.853370862 | | down |
| RP11-138I1.3 | -0.333866252 | 0.401036357 | 0.537914028 | down |
| NA | -0.333612358 | 0.766834494 | | down |
| RP11-159D12.2 | -0.333256677 | 0.365419876 | 0.503886303 | down |
| COG7 | -0.333226512 | 0.001657691 | 0.009030751 | down |
| RP11-344N10.5 | -0.333037479 | 0.401644985 | 0.538489979 | down |
| RP11-405L18.4 | -0.333008623 | 0.730189389 | | down |
| ARFGAP2 | -0.332995376 | 0.003594286 | 0.01672398 | down |
| AD000864.6 | -0.332963652 | 0.481151552 | 0.610357931 | down |
| DDB2 | -0.332904012 | 0.016258208 | 0.053030248 | down |
| DBIL5P | -0.332862589 | 0.380749199 | 0.518715319 | down |
| SLC25A38 | -0.332770572 | 0.003077447 | 0.014786315 | down |
| PSMA8 | -0.332768349 | 0.526483227 | 0.649961551 | down |
| EHMT2 | -0.332678702 | 0.039805485 | 0.10358312 | down |
| RP11-90J7.2 | -0.332627673 | 0.856276018 | | down |
| RBM26 | -0.332560444 | 0.068213783 | 0.153422287 | down |
| FAM85B | -0.332542701 | 0.621485176 | 0.731485569 | down |
| SEMA5A | -0.332527979 | 0.126849257 | 0.239012799 | down |
| KCTD2 | -0.332510297 | 0.011635415 | 0.041237986 | down |
| RP11-522I20.3 | -0.332440511 | 0.201898162 | 0.331068026 | down |
| PLP1 | -0.332173484 | 0.43579441 | 0.570501406 | down |
| FAM160A2 | -0.332123279 | 0.017336357 | 0.055688389 | down |
| CALML6 | -0.332064079 | 0.38917173 | 0.526726527 | down |
| RP11-359P5.1 | -0.332052676 | 0.654797465 | 0.757726519 | down |
| MAST3 | -0.331970816 | 0.049679804 | 0.121733955 | down |
| RP3-467L1.6 | -0.331915017 | 0.392897558 | 0.530311974 | down |
| RP3-491M17.1 | -0.331912124 | 0.909680646 | | down |
| PRAMEF31P | -0.331912124 | 0.909680646 | | down |
| MIR1290 | -0.331912124 | 0.909680646 | | down |
| RP11-344H11.5 | -0.331912124 | 0.909680646 | | down |
| SNORA26 | -0.331912124 | 0.909680646 | | down |
| RP11-99H8.1 | -0.331912124 | 0.909680646 | | down |
| RP5-837I24.5 | -0.331912124 | 0.909680646 | | down |
| SNORA2 | -0.331912124 | 0.909680646 | | down |
| NDUFA5P10 | -0.331912124 | 0.909680646 | | down |
| RP11-314N2.2 | -0.331912124 | 0.909680646 | | down |
| RNU6-884P | -0.331912124 | 0.909680646 | | down |
| COX5BP8 | -0.331912124 | 0.909680646 | | down |
| NA | -0.331912124 | 0.909680646 | | down |
| RP11-372M18.2 | -0.331912124 | 0.909680646 | | down |
| RP11-122M14.3 | -0.331912124 | 0.909680646 | | down |
| Y_RNA | -0.331912124 | 0.909680646 | | down |
| RP11-103C3.2 | -0.331912124 | 0.909680646 | | down |
| SNRPEP10 | -0.331912124 | 0.909680646 | | down |
| RP11-100E13.3 | -0.331912124 | 0.909680646 | | down |
| RP4-559A3.6 | -0.331912124 | 0.909680646 | | down |
| NA | -0.331912124 | 0.909680646 | | down |
| RP11-177F15.1 | -0.331912124 | 0.909680646 | | down |
| RP11-467I20.5 | -0.331912124 | 0.909680646 | | down |

| | | | |
|---|---|---|---|
| OR2G3 | -0.331912124 | 0.909680646 | down |
| OR2X1P | -0.331912124 | 0.909680646 | down |
| OR14C36 | -0.331912124 | 0.909680646 | down |
| AC010907.2 | -0.331912124 | 0.909680646 | down |
| MIR7158 | -0.331912124 | 0.909680646 | down |
| RNU6-1288P | -0.331912124 | 0.909680646 | down |
| RP11-32P22.1 | -0.331912124 | 0.909680646 | down |
| AC010145.4 | -0.331912124 | 0.909680646 | down |
| RN7SKP66 | -0.331912124 | 0.909680646 | down |
| RNA5SP96 | -0.331912124 | 0.909680646 | down |
| MIR1285-2 | -0.331912124 | 0.909680646 | down |
| RNU6-640P | -0.331912124 | 0.909680646 | down |
| AC012671.2 | -0.331912124 | 0.909680646 | down |
| IGKV2D-14 | -0.331912124 | 0.909680646 | down |
| TRIM43B | -0.331912124 | 0.909680646 | down |
| Metazoa_SRP | -0.331912124 | 0.909680646 | down |
| BMS1P19 | -0.331912124 | 0.909680646 | down |
| MTND1P28 | -0.331912124 | 0.909680646 | down |
| MTND1P29 | -0.331912124 | 0.909680646 | down |
| SMC4P1 | -0.331912124 | 0.909680646 | down |
| AC104777.1 | -0.331912124 | 0.909680646 | down |
| RPL21P38 | -0.331912124 | 0.909680646 | down |
| NA | -0.331912124 | 0.909680646 | down |
| AC104058.1 | -0.331912124 | 0.909680646 | down |
| AC067945.2 | -0.331912124 | 0.909680646 | down |
| AC109589.1 | -0.331912124 | 0.909680646 | down |
| AC073284.4 | -0.331912124 | 0.909680646 | down |
| AC053503.2 | -0.331912124 | 0.909680646 | down |
| RP11-256I23.3 | -0.331912124 | 0.909680646 | down |
| AC007559.1 | -0.331912124 | 0.909680646 | down |
| MIR4792 | -0.331912124 | 0.909680646 | down |
| SUMO2P10 | -0.331912124 | 0.909680646 | down |
| RNU6-243P | -0.331912124 | 0.909680646 | down |
| MIR4443 | -0.331912124 | 0.909680646 | down |
| Metazoa_SRP | -0.331912124 | 0.909680646 | down |
| COX6CP6 | -0.331912124 | 0.909680646 | down |
| HSPE1P19 | -0.331912124 | 0.909680646 | down |
| RP11-529P9.1 | -0.331912124 | 0.909680646 | down |
| RN7SL752P | -0.331912124 | 0.909680646 | down |
| RN7SL229P | -0.331912124 | 0.909680646 | down |
| RP11-433C9.3 | -0.331912124 | 0.909680646 | down |
| BNIP3P41 | -0.331912124 | 0.909680646 | down |
| RP11-747H12.7 | -0.331912124 | 0.909680646 | down |
| Metazoa_SRP | -0.331912124 | 0.909680646 | down |
| RN7SL193P | -0.331912124 | 0.909680646 | down |
| SYT14P1 | -0.331912124 | 0.909680646 | down |
| RP11-147K6.1 | -0.331912124 | 0.909680646 | down |
| RP11-713M6.2 | -0.331912124 | 0.909680646 | down |
| RNU6-1217P | -0.331912124 | 0.909680646 | down |
| RP11-62N21.1 | -0.331912124 | 0.909680646 | down |
| LINC00613 | -0.331912124 | 0.909680646 | down |

| | | | |
|---|---|---|---|
| Y_RNA | -0.331912124 | 0.909680646 | down |
| ZBTB8OSP1 | -0.331912124 | 0.909680646 | down |
| RP13-577H12.3 | -0.331912124 | 0.909680646 | down |
| AF146191.8 | -0.331912124 | 0.909680646 | down |
| LINC01377 | -0.331912124 | 0.909680646 | down |
| RP11-480D4.4 | -0.331912124 | 0.909680646 | down |
| RP11-417J1.8 | -0.331912124 | 0.909680646 | down |
| AC114973.1 | -0.331912124 | 0.909680646 | down |
| CTC-235G5.2 | -0.331912124 | 0.909680646 | down |
| ST13P12 | -0.331912124 | 0.909680646 | down |
| RPS2P25 | -0.331912124 | 0.909680646 | down |
| CTC-506B8.1 | -0.331912124 | 0.909680646 | down |
| EEF1A1P20 | -0.331912124 | 0.909680646 | down |
| JAKMIP2-AS1 | -0.331912124 | 0.909680646 | down |
| CTC-353G13.1 | -0.331912124 | 0.909680646 | down |
| RP11-844P9.4 | -0.331912124 | 0.909680646 | down |
| OR2Y1 | -0.331912124 | 0.909680646 | down |
| CTD-2593A12.4 | -0.331912124 | 0.909680646 | down |
| RP5-856G1.2 | -0.331912124 | 0.909680646 | down |
| TUBB2BP1 | -0.331912124 | 0.909680646 | down |
| HNRNPA1P37 | -0.331912124 | 0.909680646 | down |
| RPL5P20 | -0.331912124 | 0.909680646 | down |
| RP3-369A17.2 | -0.331912124 | 0.909680646 | down |
| OR2B4P | -0.331912124 | 0.909680646 | down |
| E2F4P1 | -0.331912124 | 0.909680646 | down |
| NA | -0.331912124 | 0.909680646 | down |
| RP1-232L24.2 | -0.331912124 | 0.909680646 | down |
| NACAP7 | -0.331912124 | 0.909680646 | down |
| RNU6-1115P | -0.331912124 | 0.909680646 | down |
| NEPNP | -0.331912124 | 0.909680646 | down |
| RP11-307P5.2 | -0.331912124 | 0.909680646 | down |
| RP11-472G23.3 | -0.331912124 | 0.909680646 | down |
| RNU4-7P | -0.331912124 | 0.909680646 | down |
| RP1-292B18.3 | -0.331912124 | 0.909680646 | down |
| RP1-101K10.4 | -0.331912124 | 0.909680646 | down |
| NA | -0.331912124 | 0.909680646 | down |
| CYP3A54P | -0.331912124 | 0.909680646 | down |
| RN7SL496P | -0.331912124 | 0.909680646 | down |
| NA | -0.331912124 | 0.909680646 | down |
| AC004920.2 | -0.331912124 | 0.909680646 | down |
| CALM1P2 | -0.331912124 | 0.909680646 | down |
| RP5-965K10.3 | -0.331912124 | 0.909680646 | down |
| VN1R34P | -0.331912124 | 0.909680646 | down |
| MTND1P2 | -0.331912124 | 0.909680646 | down |
| RP11-797H7.6 | -0.331912124 | 0.909680646 | down |
| MIR5692A1 | -0.331912124 | 0.909680646 | down |
| AC091654.7 | -0.331912124 | 0.909680646 | down |
| RNU6-392P | -0.331912124 | 0.909680646 | down |
| RP11-500M10.1 | -0.331912124 | 0.909680646 | down |
| MIR129-1 | -0.331912124 | 0.909680646 | down |
| RP11-274B21.13 | -0.331912124 | 0.909680646 | down |

| | | | |
|---|---|---|---|
| RP11-190G13.4 | -0.331912124 | 0.909680646 | down |
| AC009517.2 | -0.331912124 | 0.909680646 | down |
| OR2F2 | -0.331912124 | 0.909680646 | down |
| RP4-545C24.5 | -0.331912124 | 0.909680646 | down |
| RN7SKP174 | -0.331912124 | 0.909680646 | down |
| COX6B1P1 | -0.331912124 | 0.909680646 | down |
| CTD-2281E23.3 | -0.331912124 | 0.909680646 | down |
| RP11-52B19.10 | -0.331912124 | 0.909680646 | down |
| NA | -0.331912124 | 0.909680646 | down |
| RNU7-55P | -0.331912124 | 0.909680646 | down |
| RP11-349F21.5 | -0.331912124 | 0.909680646 | down |
| RP11-1113C11.2 | -0.331912124 | 0.909680646 | down |
| RNU6-895P | -0.331912124 | 0.909680646 | down |
| RNU6-104P | -0.331912124 | 0.909680646 | down |
| NA | -0.331912124 | 0.909680646 | down |
| LINC00251 | -0.331912124 | 0.909680646 | down |
| RP11-744J10.2 | -0.331912124 | 0.909680646 | down |
| RP11-267M23.5 | -0.331912124 | 0.909680646 | down |
| POU5F1P2 | -0.331912124 | 0.909680646 | down |
| KB-1299A7.2 | -0.331912124 | 0.909680646 | down |
| OC90 | -0.331912124 | 0.909680646 | down |
| RP11-408A13.2 | -0.331912124 | 0.909680646 | down |
| RNU6-1260P | -0.331912124 | 0.909680646 | down |
| RN7SL720P | -0.331912124 | 0.909680646 | down |
| IFNA4 | -0.331912124 | 0.909680646 | down |
| RP11-96J15.1 | -0.331912124 | 0.909680646 | down |
| RP11-265B8.5 | -0.331912124 | 0.909680646 | down |
| RP11-92C4.4 | -0.331912124 | 0.909680646 | down |
| OR13C2 | -0.331912124 | 0.909680646 | down |
| RNA5SP293 | -0.331912124 | 0.909680646 | down |
| RNU6-984P | -0.331912124 | 0.909680646 | down |
| RP11-69C17.2 | -0.331912124 | 0.909680646 | down |
| RN7SL445P | -0.331912124 | 0.909680646 | down |
| MIR4293 | -0.331912124 | 0.909680646 | down |
| RP11-123G9.1 | -0.331912124 | 0.909680646 | down |
| RP11-252C24.2 | -0.331912124 | 0.909680646 | down |
| COX6CP15 | -0.331912124 | 0.909680646 | down |
| Y_RNA | -0.331912124 | 0.909680646 | down |
| RPS27P1 | -0.331912124 | 0.909680646 | down |
| OR6L1P | -0.331912124 | 0.909680646 | down |
| AC109309.4 | -0.331912124 | 0.909680646 | down |
| RPS24P14 | -0.331912124 | 0.909680646 | down |
| AC104389.31 | -0.331912124 | 0.909680646 | down |
| NA | -0.331912124 | 0.909680646 | down |
| OR4C10P | -0.331912124 | 0.909680646 | down |
| TYRL | -0.331912124 | 0.909680646 | down |
| OR5M13P | -0.331912124 | 0.909680646 | down |
| TEX40 | -0.331912124 | 0.909680646 | down |
| HMGN2P38 | -0.331912124 | 0.909680646 | down |
| RP11-806N19.2 | -0.331912124 | 0.909680646 | down |
| NA | -0.331912124 | 0.909680646 | down |

| | | | |
|---|---|---|---|
| CLDN25 | -0.331912124 | 0.909680646 | down |
| NA | -0.331912124 | 0.909680646 | down |
| SUPT4H1P2 | -0.331912124 | 0.909680646 | down |
| RP11-443N24.3 | -0.331912124 | 0.909680646 | down |
| TUBB8P5 | -0.331912124 | 0.909680646 | down |
| MIR1293 | -0.331912124 | 0.909680646 | down |
| KRT125P | -0.331912124 | 0.909680646 | down |
| RP11-1136G11.8 | -0.331912124 | 0.909680646 | down |
| LACRT | -0.331912124 | 0.909680646 | down |
| PDCL3P7 | -0.331912124 | 0.909680646 | down |
| CTD-2021H9.2 | -0.331912124 | 0.909680646 | down |
| RP11-12K6.2 | -0.331912124 | 0.909680646 | down |
| RP11-263K4.4 | -0.331912124 | 0.909680646 | down |
| BRWD1P2 | -0.331912124 | 0.909680646 | down |
| SNORD74 | -0.331912124 | 0.909680646 | down |
| NA | -0.331912124 | 0.909680646 | down |
| RN7SL769P | -0.331912124 | 0.909680646 | down |
| NA | -0.331912124 | 0.909680646 | down |
| RP11-367G12.1 | -0.331912124 | 0.909680646 | down |
| RP11-749H20.2 | -0.331912124 | 0.909680646 | down |
| RPL17P51 | -0.331912124 | 0.909680646 | down |
| RN7SKP2 | -0.331912124 | 0.909680646 | down |
| RAC1P3 | -0.331912124 | 0.909680646 | down |
| LINC00400 | -0.331912124 | 0.909680646 | down |
| SMIM2-IT1 | -0.331912124 | 0.909680646 | down |
| HTR2A-AS1 | -0.331912124 | 0.909680646 | down |
| RP11-452B18.2 | -0.331912124 | 0.909680646 | down |
| NA | -0.331912124 | 0.909680646 | down |
| RP11-75N6.3 | -0.331912124 | 0.909680646 | down |
| LINC01040 | -0.331912124 | 0.909680646 | down |
| NA | -0.331912124 | 0.909680646 | down |
| RP11-282A11.2 | -0.331912124 | 0.909680646 | down |
| RP11-199F6.4 | -0.331912124 | 0.909680646 | down |
| NF1P10 | -0.331912124 | 0.909680646 | down |
| OR4K13 | -0.331912124 | 0.909680646 | down |
| RNA5SP380 | -0.331912124 | 0.909680646 | down |
| ARL6IP1P1 | -0.331912124 | 0.909680646 | down |
| LINC00645 | -0.331912124 | 0.909680646 | down |
| Y_RNA | -0.331912124 | 0.909680646 | down |
| RNU6ATAC9P | -0.331912124 | 0.909680646 | down |
| RPSAP13 | -0.331912124 | 0.909680646 | down |
| RN7SL461P | -0.331912124 | 0.909680646 | down |
| RNU6-1162P | -0.331912124 | 0.909680646 | down |
| MIR4708 | -0.331912124 | 0.909680646 | down |
| RP11-226F19.1 | -0.331912124 | 0.909680646 | down |
| RP11-232C2.3 | -0.331912124 | 0.909680646 | down |
| Metazoa_SRP | -0.331912124 | 0.909680646 | down |
| SERPINA2 | -0.331912124 | 0.909680646 | down |
| NA | -0.331912124 | 0.909680646 | down |
| RP11-543C4.3 | -0.331912124 | 0.909680646 | down |
| HMGB3P26 | -0.331912124 | 0.909680646 | down |

| | | | |
|---|---|---|---|
| GRAMD4P5 | -0.331912124 | 0.909680646 | down |
| RP11-854K16.4 | -0.331912124 | 0.909680646 | down |
| NA | -0.331912124 | 0.909680646 | down |
| RP11-580I1.1 | -0.331912124 | 0.909680646 | down |
| RP11-1H8.1 | -0.331912124 | 0.909680646 | down |
| RP11-133K1.8 | -0.331912124 | 0.909680646 | down |
| NA | -0.331912124 | 0.909680646 | down |
| CTD-2651B20.5 | -0.331912124 | 0.909680646 | down |
| RP11-112N19.1 | -0.331912124 | 0.909680646 | down |
| MIR5094 | -0.331912124 | 0.909680646 | down |
| OR4F4 | -0.331912124 | 0.909680646 | down |
| RN7SL219P | -0.331912124 | 0.909680646 | down |
| RP11-382N13.1 | -0.331912124 | 0.909680646 | down |
| RP11-2603.1 | -0.331912124 | 0.909680646 | down |
| PRM2 | -0.331912124 | 0.909680646 | down |
| RP11-23E10.2 | -0.331912124 | 0.909680646 | down |
| RP11-177N22.2 | -0.331912124 | 0.909680646 | down |
| RP11-152014.4 | -0.331912124 | 0.909680646 | down |
| RN7SKP118 | -0.331912124 | 0.909680646 | down |
| RPL35AP33 | -0.331912124 | 0.909680646 | down |
| AC009120.9 | -0.331912124 | 0.909680646 | down |
| CTD-2545H1.1 | -0.331912124 | 0.909680646 | down |
| RP1-59D14.8 | -0.331912124 | 0.909680646 | down |
| YWHAEP2 | -0.331912124 | 0.909680646 | down |
| TNPO1P2 | -0.331912124 | 0.909680646 | down |
| RP11-822E23.5 | -0.331912124 | 0.909680646 | down |
| GTF2IP6 | -0.331912124 | 0.909680646 | down |
| CCL15-CCL14 | -0.331912124 | 0.909680646 | down |
| MIR6783 | -0.331912124 | 0.909680646 | down |
| RP11-618P13.1 | -0.331912124 | 0.909680646 | down |
| Y_RNA | -0.331912124 | 0.909680646 | down |
| RP11-927P21.8 | -0.331912124 | 0.909680646 | down |
| CTD-2582D11.1 | -0.331912124 | 0.909680646 | down |
| RPS4XP19 | -0.331912124 | 0.909680646 | down |
| RNU6-167P | -0.331912124 | 0.909680646 | down |
| RPSAP57 | -0.331912124 | 0.909680646 | down |
| RP11-383D22.1 | -0.331912124 | 0.909680646 | down |
| RP11-866E20.1 | -0.331912124 | 0.909680646 | down |
| RP11-762J3.1 | -0.331912124 | 0.909680646 | down |
| OR10H3 | -0.331912124 | 0.909680646 | down |
| OR1AB1P | -0.331912124 | 0.909680646 | down |
| CTC-451A6.5 | -0.331912124 | 0.909680646 | down |
| ZNF723P | -0.331912124 | 0.909680646 | down |
| VN1R92P | -0.331912124 | 0.909680646 | down |
| CTC-525D6.2 | -0.331912124 | 0.909680646 | down |
| LGALS16 | -0.331912124 | 0.909680646 | down |
| CTD-3187F8.2 | -0.331912124 | 0.909680646 | down |
| MIR524 | -0.331912124 | 0.909680646 | down |
| RN7SKP69 | -0.331912124 | 0.909680646 | down |
| Y_RNA | -0.331912124 | 0.909680646 | down |
| RP5-1004I9.2 | -0.331912124 | 0.909680646 | down |

| | | | | |
|---|---|---|---|---|
| NA | -0.331912124 | 0.909680646 | | down |
| RP1-66N13.1 | -0.331912124 | 0.909680646 | | down |
| RP13-30A9.1 | -0.331912124 | 0.909680646 | | down |
| RP11-358D14.3 | -0.331912124 | 0.909680646 | | down |
| NA | -0.331912124 | 0.909680646 | | down |
| KRTAP7-1 | -0.331912124 | 0.909680646 | | down |
| RPS20P1 | -0.331912124 | 0.909680646 | | down |
| AP001425.14 | -0.331912124 | 0.909680646 | | down |
| TMEM97P1 | -0.331912124 | 0.909680646 | | down |
| KB-1183D5.16 | -0.331912124 | 0.909680646 | | down |
| MIR301B | -0.331912124 | 0.909680646 | | down |
| AP000343.2 | -0.331912124 | 0.909680646 | | down |
| AP000345.4 | -0.331912124 | 0.909680646 | | down |
| CTA-85E5.7 | -0.331912124 | 0.909680646 | | down |
| [illegible]L22NC01-116C6.[illegible] | -0.331912124 | 0.909680646 | | down |
| NA | -0.331912124 | 0.909680646 | | down |
| [illegible]L22NC03-121E8.[illegible] | -0.331912124 | 0.909680646 | | down |
| GS1-519E5.1 | -0.331912124 | 0.909680646 | | down |
| RP11-753E22.3 | -0.331912124 | 0.909680646 | | down |
| RNU6-1321P | -0.331912124 | 0.909680646 | | down |
| RP1-192P9.1 | -0.331912124 | 0.909680646 | | down |
| RP11-38023.3 | -0.331912124 | 0.909680646 | | down |
| RP13-324D24.1 | -0.331912124 | 0.909680646 | | down |
| RP1-19N1.1 | -0.331912124 | 0.909680646 | | down |
| E2F6P4 | -0.331912124 | 0.909680646 | | down |
| PGBD4P6 | -0.331912124 | 0.909680646 | | down |
| MIR513C | -0.331912124 | 0.909680646 | | down |
| RPL12P50 | -0.331912124 | 0.909680646 | | down |
| 5S_rRNA | -0.331912124 | 0.909680646 | | down |
| MAGEA1 | -0.331912124 | 0.909680646 | | down |
| FAM223A | -0.331912124 | 0.909680646 | | down |
| TSPY11P | -0.331912124 | 0.909680646 | | down |
| RP11-109F19.1 | -0.331912124 | 0.909680646 | | down |
| FAM197Y4P | -0.331912124 | 0.909680646 | | down |
| RBMY2OP | -0.331912124 | 0.909680646 | | down |
| RP1-278022.1 | -0.331745877 | 0.499448317 | 0.626614422 | down |
| CCDC77 | -0.331696425 | 0.068536003 | 0.153947782 | down |
| RP11-441015.3 | -0.331619126 | 0.243948172 | 0.377932818 | down |
| CNTNAP3 | -0.331576516 | 0.427608652 | 0.562999007 | down |
| NDUFB4P8 | -0.331515232 | 0.714622845 | | down |
| NA | -0.331422752 | 0.611328809 | 0.72298704 | down |
| AC009487.6 | -0.331298017 | 0.645324093 | 0.750314127 | down |
| C6orf203 | -0.331219871 | 0.06302218 | 0.144895516 | down |
| HACL1 | -0.33103239 | 0.008292381 | 0.03179472 | down |
| C16orf59 | -0.331010574 | 0.288549046 | 0.425689542 | down |
| MFSD10 | -0.330983687 | 0.150917253 | 0.270163734 | down |
| NUAK2 | -0.33097961 | 0.058940137 | 0.1379663 | down |
| FANCC | -0.330886993 | 0.011227008 | 0.040064642 | down |
| ZNF579 | -0.330744496 | 0.092672549 | 0.191436611 | down |
| WDR83 | -0.330722506 | 0.118366224 | 0.227858962 | down |
| LRRC29 | -0.33071257 | 0.126731573 | 0.238948565 | down |

| | | | | |
|---|---|---|---|---|
| SLC7A3 | -0.330707407 | 0.573386177 | 0.690536509 | down |
| CDC42P5 | -0.330687725 | 0.690359271 | | down |
| HNRNPA3P10 | -0.330624229 | 0.563781884 | 0.682347892 | down |
| RP11-484K9.4 | -0.330618732 | 0.637406391 | 0.744045288 | down |
| THAP7-AS1 | -0.330570188 | 0.258714746 | 0.393839979 | down |
| CTC-260F20.3 | -0.330401737 | 0.430150075 | 0.565329346 | down |
| GALNTL6 | -0.330358294 | 0.393082215 | 0.530442062 | down |
| DUTP6 | -0.330321008 | 0.337533169 | 0.475872667 | down |
| SAP18P3 | -0.330203669 | 0.782873543 | | down |
| NFRKB | -0.330193187 | 0.000257718 | 0.002061571 | down |
| C16orf89 | -0.3301148 | 0.177839845 | 0.302855478 | down |
| ALG3 | -0.330104666 | 0.185398212 | 0.311562643 | down |
| PMS2P7 | -0.329648044 | 0.53989884 | 0.661818034 | down |
| UBE2V1 | -0.329593261 | 0.274669404 | 0.411003959 | down |
| TJP1 | -0.329505577 | 0.16730644 | 0.290442908 | down |
| GGA1 | -0.329450088 | 0.005602326 | 0.023658214 | down |
| TMEM120B | -0.329337138 | 0.046208642 | 0.115346259 | down |
| ESRRAP2 | -0.329290982 | 0.784382847 | | down |
| RP11-401P9.6 | -0.329254667 | 0.655913804 | 0.758517103 | down |
| TMEM176A | -0.329246711 | 0.080131065 | 0.172453991 | down |
| RP11-467L13.7 | -0.329226479 | 0.126115082 | 0.238182142 | down |
| RAD54L | -0.329198469 | 0.297919578 | 0.435387479 | down |
| SLC35D1 | -0.32915129 | 0.159687956 | 0.280877288 | down |
| NUDT16L1 | -0.329132489 | 0.029703651 | 0.083216755 | down |
| VPS16 | -0.329108786 | 0.027979335 | 0.079784656 | down |
| RPS15AP12 | -0.329064786 | 0.49131705 | 0.61955111 | down |
| CKMT2-AS1 | -0.328931708 | 0.097972617 | 0.199113123 | down |
| RP11-589P10.5 | -0.328872399 | 0.21336061 | 0.344233587 | down |
| UBXN11 | -0.328844336 | 0.182686092 | 0.308418799 | down |
| ZNF350 | -0.328781032 | 0.107244089 | 0.212484562 | down |
| RN7SKP229 | -0.328773859 | 0.910531019 | | down |
| RNA5SP19 | -0.328773859 | 0.910531019 | | down |
| LSINCT5 | -0.328773859 | 0.910531019 | | down |
| RNA5SP345 | -0.328773859 | 0.910531019 | | down |
| KIRREL3-AS1 | -0.328773859 | 0.910531019 | | down |
| NA | -0.328773859 | 0.910531019 | | down |
| KRTAP26-1 | -0.328773859 | 0.910531019 | | down |
| RP11-791N19.1 | -0.328773859 | 0.910531019 | | down |
| ZIK1P1 | -0.328773859 | 0.910531019 | | down |
| DMRTC1 | -0.328772677 | 0.892654978 | | down |
| AC140542.2 | -0.328772148 | 0.880219437 | | down |
| NA | -0.328772132 | 0.879792759 | | down |
| AC069257.6 | -0.328771749 | 0.867344122 | | down |
| NA | -0.328771701 | 0.865484489 | | down |
| KRT8P26 | -0.3287717 | 0.86545593 | | down |
| PAX7 | -0.32877169 | 0.865070136 | | down |
| RP11-1365D11.1 | -0.328771688 | 0.8649872 | | down |
| OR52I1 | -0.328771669 | 0.864226036 | | down |
| BET1P1 | -0.328771406 | 0.852165583 | | down |
| GAPDHP52 | -0.328771162 | 0.837525433 | | down |
| RPL23AP50 | -0.328771128 | 0.835104781 | | down |

| | | | | |
|---|---|---|---|---|
| KRT18P42 | -0.328771103 | 0.833254918 | | down |
| RPL7P25 | -0.328771103 | 0.833274018 | | down |
| Y_RNA | -0.328770977 | 0.823010608 | | down |
| RP11-977G19.14 | -0.328770968 | 0.822146389 | | down |
| AL355480.1 | -0.328770813 | 0.806144052 | | down |
| PCDHGA11 | -0.328675288 | 0.505114186 | 0.631735102 | down |
| HEPHL1 | -0.328550461 | 0.515720891 | 0.640870184 | down |
| KIF9-AS1 | -0.328487542 | 0.31659779 | 0.455243802 | down |
| SUGT1P3 | -0.328466398 | 0.474057041 | 0.604281495 | down |
| NA | -0.328304923 | 0.442005489 | 0.575587012 | down |
| CNPY3 | -0.328294023 | 0.144625104 | 0.262173486 | down |
| RP11-258F22.1 | -0.328073048 | 0.627820976 | 0.736432804 | down |
| UBXN1 | -0.327960975 | 0.061043045 | 0.141597477 | down |
| MIR9-3HG | -0.327951905 | 0.327032502 | 0.465480599 | down |
| PTTG1 | -0.32775263 | 0.202362098 | 0.331647768 | down |
| RPL7P47 | -0.327709093 | 0.463071849 | 0.594785249 | down |
| PQBP1 | -0.327688081 | 0.126046073 | 0.238081744 | down |
| CORO1B | -0.327512821 | 0.033157816 | 0.090251294 | down |
| FAM46A | -0.327433125 | 0.106308341 | 0.211120127 | down |
| TMEM259 | -0.327405346 | 0.132100948 | 0.246068627 | down |
| RP11-70L8.5 | -0.327396021 | 0.750240697 | 0.831160128 | down |
| TLE2 | -0.327278091 | 0.114750078 | 0.222853998 | down |
| TRPC7-AS1 | -0.327277001 | 0.695362329 | 0.790176096 | down |
| KLHDC3 | -0.327117178 | 0.019213491 | 0.06027463 | down |
| BORCS7 | -0.327112672 | 0.010277259 | 0.037443929 | down |
| LINC01176 | -0.327089481 | 0.212918343 | 0.343790977 | down |
| RP11-689P11.2 | -0.327067057 | 0.432568997 | 0.567564981 | down |
| RP11-10C24.3 | -0.327057334 | 0.191596115 | 0.319336578 | down |
| RP11-534C12.1 | -0.326852545 | 0.419789823 | 0.555553054 | down |
| MIIP | -0.326768843 | 0.136783585 | 0.252040848 | down |
| ZNF682 | -0.326761654 | 0.178736498 | 0.303833843 | down |
| ARRDC5 | -0.326727456 | 0.393020944 | 0.530407028 | down |
| GDF9 | -0.326685186 | 0.483291531 | 0.612374457 | down |
| RCOR2 | -0.3264853 | 0.227551713 | 0.359743405 | down |
| RP11-536C12.1 | -0.32647066 | 0.468770979 | 0.599742593 | down |
| ACP6 | -0.326441861 | 0.035201649 | 0.094436855 | down |
| YIF1B | -0.326373412 | 0.181625319 | 0.307145732 | down |
| ZKSCAN2 | -0.326345729 | 0.137711584 | 0.253346815 | down |
| NA | -0.326271064 | 0.301960046 | 0.439773505 | down |
| MRGPRF-AS1 | -0.326226466 | 0.461041559 | 0.592857926 | down |
| ATP5J2 | -0.326203793 | 0.234893106 | 0.367687216 | down |
| PARD3 | -0.326141715 | 0.050003153 | 0.122326742 | down |
| Z84812.4 | -0.326091035 | 0.806240055 | | down |
| RN7SL535P | -0.326026851 | 0.751604792 | | down |
| Y_RNA | -0.325991336 | 0.581323834 | 0.697356489 | down |
| FAM201A | -0.325733304 | 0.325493015 | 0.464014811 | down |
| ZNF675 | -0.325694843 | 0.219003924 | 0.350434273 | down |
| RGS12 | -0.325620826 | 0.019823992 | 0.061636347 | down |
| GAPDHP63 | -0.325583942 | 0.40082881 | 0.537731671 | down |
| MRPS2 | -0.325521341 | 0.088261653 | 0.18494442 | down |
| BOK-AS1 | -0.325216891 | 0.805906409 | | down |

| | | | | |
|---|---|---|---|---|
| RP11-297B17.3 | -0.325168512 | 0.56404846 | 0.682347892 | down |
| RP11-90M2.5 | -0.325123073 | 0.514446692 | 0.639683652 | down |
| NA | -0.325082743 | 0.75516562 | | down |
| NF1 | -0.325061015 | 0.056779114 | 0.134225487 | down |
| C19orf81 | -0.324998496 | 0.439460024 | 0.57341463 | down |
| MIR4645 | -0.324935061 | 0.706346236 | | down |
| RP11-476H16.1 | -0.324757751 | 0.508449739 | 0.634775149 | down |
| IL2RG | -0.324649002 | 0.347224833 | 0.486024174 | down |
| ANKS3 | -0.324641617 | 0.095202416 | 0.195193524 | down |
| PCCB | -0.324609395 | 0.004290616 | 0.019247022 | down |
| ADH5P4 | -0.324556656 | 0.565108316 | 0.683278905 | down |
| ABCA2 | -0.324489687 | 0.031833637 | 0.087591335 | down |
| SEMA6C | -0.324483866 | 0.199388901 | 0.328387246 | down |
| AC005042.2 | -0.324470205 | 0.747864317 | | down |
| IL1RL2 | -0.324455447 | 0.444771608 | 0.578247358 | down |
| KLHL11 | -0.324385922 | 0.417644608 | 0.553836272 | down |
| SCAMP5 | -0.324350102 | 0.188803061 | 0.315835142 | down |
| MRPL48 | -0.324330803 | 0.008837928 | 0.033370203 | down |
| NA | -0.324307345 | 0.340985442 | 0.479345247 | down |
| NAV3 | -0.324282243 | 0.420809345 | 0.556608081 | down |
| STX10 | -0.324249874 | 0.01777976 | 0.056857591 | down |
| RP11-415F23.2 | -0.324218056 | 0.377222687 | 0.515383284 | down |
| LINC00675 | -0.324194225 | 0.591243722 | 0.706211044 | down |
| ZNF765 | -0.324099921 | 0.045142435 | 0.113485007 | down |
| UQCRHL | -0.324022221 | 0.107088889 | 0.212326179 | down |
| SOWAHC | -0.324002725 | 0.153318948 | 0.272956568 | down |
| TRDV1 | -0.323960845 | 0.532445004 | 0.655498185 | down |
| DENND5B | -0.323917944 | 0.162046209 | 0.283972016 | down |
| KCNT1 | -0.323910085 | 0.438796353 | 0.572958305 | down |
| AC096579.13 | -0.323888564 | 0.594290019 | 0.708643122 | down |
| PCDHGB8P | -0.323476825 | 0.618503938 | 0.729033399 | down |
| THOC6 | -0.323397389 | 0.035242943 | 0.094496852 | down |
| P2RY11 | -0.323358997 | 0.271450574 | 0.407589636 | down |
| C6orf163 | -0.323288564 | 0.358123334 | 0.496564705 | down |
| DNAJC5 | -0.323218363 | 0.006804926 | 0.027409464 | down |
| RBPMS2 | -0.323192463 | 0.328209591 | 0.466683056 | down |
| TMEM261 | -0.32318101 | 0.073786472 | 0.162460828 | down |
| LDHAP5 | -0.323137021 | 0.738252301 | | down |
| RP11-522B15.3 | -0.32313555 | 0.447905151 | 0.581098794 | down |
| ADCY6 | -0.323043534 | 0.240551072 | 0.374306836 | down |
| CRTC1 | -0.323003266 | 0.050963417 | 0.124009467 | down |
| AC008746.12 | -0.322991436 | 0.406687156 | 0.543262146 | down |
| HMOX2 | -0.322985349 | 0.018680741 | 0.058966609 | down |
| RP11-111A22.1 | -0.322982706 | 0.493544299 | 0.621668829 | down |
| CTD-2270L9.4 | -0.322967314 | 0.410494625 | 0.547253306 | down |
| RP11-380G5.3 | -0.322891088 | 0.416390927 | 0.552591158 | down |
| PIGS | -0.322655254 | 0.032179814 | 0.088325447 | down |
| RP11-348A7.1 | -0.322503074 | 0.912230516 | | down |
| RP11-384B12.3 | -0.322503074 | 0.912230516 | | down |
| CNOT7P2 | -0.322503074 | 0.912230516 | | down |
| NA | -0.322503074 | 0.912230516 | | down |

| | | | |
|---|---|---|---|
| AC011242.5 | -0.322503074 | 0.912230516 | down |
| RNU6-1145P | -0.322503074 | 0.912230516 | down |
| AC079896.1 | -0.322503074 | 0.912230516 | down |
| MTND5P27 | -0.322503074 | 0.912230516 | down |
| RN7SL251P | -0.322503074 | 0.912230516 | down |
| AC092570.3 | -0.322503074 | 0.912230516 | down |
| SNRPGP9 | -0.322503074 | 0.912230516 | down |
| AC103564.7 | -0.322503074 | 0.912230516 | down |
| RNA5SP109 | -0.322503074 | 0.912230516 | down |
| AC073465.1 | -0.322503074 | 0.912230516 | down |
| AC073834.3 | -0.322503074 | 0.912230516 | down |
| NA | -0.322503074 | 0.912230516 | down |
| RP11-548H3.1 | -0.322503074 | 0.912230516 | down |
| AC114765.1 | -0.322503074 | 0.912230516 | down |
| RPL23AP28 | -0.322503074 | 0.912230516 | down |
| AC027119.1 | -0.322503074 | 0.912230516 | down |
| RP11-646D13.1 | -0.322503074 | 0.912230516 | down |
| RNA5SP132 | -0.322503074 | 0.912230516 | down |
| VDAC1P7 | -0.322503074 | 0.912230516 | down |
| RP11-481N16.1 | -0.322503074 | 0.912230516 | down |
| SNORA72 | -0.322503074 | 0.912230516 | down |
| RN7SL434P | -0.322503074 | 0.912230516 | down |
| RNA5SP152 | -0.322503074 | 0.912230516 | down |
| MTND5P4 | -0.322503074 | 0.912230516 | down |
| RP11-567N4.2 | -0.322503074 | 0.912230516 | down |
| NA | -0.322503074 | 0.912230516 | down |
| MTND2P33 | -0.322503074 | 0.912230516 | down |
| NA | -0.322503074 | 0.912230516 | down |
| RPL6P12 | -0.322503074 | 0.912230516 | down |
| RP11-451F20.1 | -0.322503074 | 0.912230516 | down |
| CTD-2297D10.1 | -0.322503074 | 0.912230516 | down |
| RP11-159F24.2 | -0.322503074 | 0.912230516 | down |
| CTD-233503.2 | -0.322503074 | 0.912230516 | down |
| RPL5P15 | -0.322503074 | 0.912230516 | down |
| RN7SL169P | -0.322503074 | 0.912230516 | down |
| CDH12P3 | -0.322503074 | 0.912230516 | down |
| CTC-347C20.2 | -0.322503074 | 0.912230516 | down |
| RP11-526F3.1 | -0.322503074 | 0.912230516 | down |
| Y_RNA | -0.322503074 | 0.912230516 | down |
| PPP2R2B-IT1 | -0.322503074 | 0.912230516 | down |
| HIST1H1T | -0.322503074 | 0.912230516 | down |
| RNU6-502P | -0.322503074 | 0.912230516 | down |
| OR2H5P | -0.322503074 | 0.912230516 | down |
| RP11-63K6.5 | -0.322503074 | 0.912230516 | down |
| Y_RNA | -0.322503074 | 0.912230516 | down |
| MIR3146 | -0.322503074 | 0.912230516 | down |
| AC079780.3 | -0.322503074 | 0.912230516 | down |
| AC005162.4 | -0.322503074 | 0.912230516 | down |
| RPL7P31 | -0.322503074 | 0.912230516 | down |
| SNORA22 | -0.322503074 | 0.912230516 | down |
| MAGI2-AS2 | -0.322503074 | 0.912230516 | down |

| | | | |
|---|---|---|---|
| AC003045.1 | -0.322503074 | 0.912230516 | down |
| RP5-1006K12.1 | -0.322503074 | 0.912230516 | down |
| RP5-842K16.2 | -0.322503074 | 0.912230516 | down |
| AC073264.10 | -0.322503074 | 0.912230516 | down |
| RP11-445N20.3 | -0.322503074 | 0.912230516 | down |
| Y_RNA | -0.322503074 | 0.912230516 | down |
| NA | -0.322503074 | 0.912230516 | down |
| CTC-756D1.3 | -0.322503074 | 0.912230516 | down |
| RNU1-124P | -0.322503074 | 0.912230516 | down |
| RNU4-50P | -0.322503074 | 0.912230516 | down |
| RP11-21C17.1 | -0.322503074 | 0.912230516 | down |
| RP11-29B9.1 | -0.322503074 | 0.912230516 | down |
| RP11-62F24.1 | -0.322503074 | 0.912230516 | down |
| IFNWP9 | -0.322503074 | 0.912230516 | down |
| TRBV21OR9-2 | -0.322503074 | 0.912230516 | down |
| RP11-160N1.9 | -0.322503074 | 0.912230516 | down |
| MIR4290HG | -0.322503074 | 0.912230516 | down |
| MIR181B2 | -0.322503074 | 0.912230516 | down |
| RP11-295P9.6 | -0.322503074 | 0.912230516 | down |
| RN7SKP39 | -0.322503074 | 0.912230516 | down |
| PABPC1P8 | -0.322503074 | 0.912230516 | down |
| RP11-753C18.8 | -0.322503074 | 0.912230516 | down |
| RP11-309P22.1 | -0.322503074 | 0.912230516 | down |
| RP13-726E6.1 | -0.322503074 | 0.912230516 | down |
| CTD-2381F24.1 | -0.322503074 | 0.912230516 | down |
| OR5B12 | -0.322503074 | 0.912230516 | down |
| RNU6-1306P | -0.322503074 | 0.912230516 | down |
| AP000860.2 | -0.322503074 | 0.912230516 | down |
| MIR4696 | -0.322503074 | 0.912230516 | down |
| TOMM20P1 | -0.322503074 | 0.912230516 | down |
| FNTAL1 | -0.322503074 | 0.912230516 | down |
| RP11-203F8.1 | -0.322503074 | 0.912230516 | down |
| RNU2-35P | -0.322503074 | 0.912230516 | down |
| RP11-712L6.7 | -0.322503074 | 0.912230516 | down |
| TAS2R63P | -0.322503074 | 0.912230516 | down |
| RP11-59H1.1 | -0.322503074 | 0.912230516 | down |
| NA | -0.322503074 | 0.912230516 | down |
| RP11-428G5.7 | -0.322503074 | 0.912230516 | down |
| RP11-70F11.2 | -0.322503074 | 0.912230516 | down |
| TMEM5-AS1 | -0.322503074 | 0.912230516 | down |
| RP11-1029F8.1 | -0.322503074 | 0.912230516 | down |
| GS1-410F4.5 | -0.322503074 | 0.912230516 | down |
| RNU6-101P | -0.322503074 | 0.912230516 | down |
| RP11-210L7.1 | -0.322503074 | 0.912230516 | down |
| RP11-357K6.2 | -0.322503074 | 0.912230516 | down |
| MIR3908 | -0.322503074 | 0.912230516 | down |
| RP11-337L12.1 | -0.322503074 | 0.912230516 | down |
| CYCSP32 | -0.322503074 | 0.912230516 | down |
| RNU6-60P | -0.322503074 | 0.912230516 | down |
| NA | -0.322503074 | 0.912230516 | down |
| RNU6-66P | -0.322503074 | 0.912230516 | down |

| | | | | |
|---|---|---|---|---|
| NA | -0.322503074 | 0.912230516 | | down |
| RP11-736P16.1 | -0.322503074 | 0.912230516 | | down |
| IGHV3-60 | -0.322503074 | 0.912230516 | | down |
| IGHV1OR15-9 | -0.322503074 | 0.912230516 | | down |
| Metazoa_SRP | -0.322503074 | 0.912230516 | | down |
| AC025918.2 | -0.322503074 | 0.912230516 | | down |
| RP11-798K3.4 | -0.322503074 | 0.912230516 | | down |
| RP11-1006G14.3 | -0.322503074 | 0.912230516 | | down |
| MTND4P34 | -0.322503074 | 0.912230516 | | down |
| FRG2HP | -0.322503074 | 0.912230516 | | down |
| NA | -0.322503074 | 0.912230516 | | down |
| RP11-321A17.5 | -0.322503074 | 0.912230516 | | down |
| RP11-686D22.2 | -0.322503074 | 0.912230516 | | down |
| PSMD7P1 | -0.322503074 | 0.912230516 | | down |
| MIR1250 | -0.322503074 | 0.912230516 | | down |
| PMM2P1 | -0.322503074 | 0.912230516 | | down |
| CXADRP3 | -0.322503074 | 0.912230516 | | down |
| RBM22P1 | -0.322503074 | 0.912230516 | | down |
| HNRNPA1P11 | -0.322503074 | 0.912230516 | | down |
| CTD-3214H19.4 | -0.322503074 | 0.912230516 | | down |
| MIR4748 | -0.322503074 | 0.912230516 | | down |
| BNIP3P19 | -0.322503074 | 0.912230516 | | down |
| Y_RNA | -0.322503074 | 0.912230516 | | down |
| RN7SL443P | -0.322503074 | 0.912230516 | | down |
| LA16c-17H1.3 | -0.322503074 | 0.912230516 | | down |
| KB-1592A4.14 | -0.322503074 | 0.912230516 | | down |
| SNORD56 | -0.322503074 | 0.912230516 | | down |
| RP3-388M5.8 | -0.322503074 | 0.912230516 | | down |
| ATXN3L | -0.322503074 | 0.912230516 | | down |
| GS1-594A7.5 | -0.322503074 | 0.912230516 | | down |
| RP13-13A3.1 | -0.322503074 | 0.912230516 | | down |
| RP11-344N17.3 | -0.322503074 | 0.912230516 | | down |
| RP13-770I1.11 | -0.322503074 | 0.912230516 | | down |
| GHc-210E9.2 | -0.322503074 | 0.912230516 | | down |
| SERPINA7P1 | -0.322503074 | 0.912230516 | | down |
| PRR32 | -0.322503074 | 0.912230516 | | down |
| CTD-2183H9.3 | -0.322503074 | 0.912230516 | | down |
| RP11-143H17.1 | -0.322503074 | 0.912230516 | | down |
| TTTY2B | -0.322503074 | 0.912230516 | | down |
| AC007967.4 | -0.322503074 | 0.912230516 | | down |
| FAM8A4P | -0.322503074 | 0.912230516 | | down |
| ATP6V0E2-AS1 | -0.322455543 | 0.306644934 | 0.444957276 | down |
| NLGN3 | -0.322124365 | 0.234799741 | 0.367619455 | down |
| RP5-858B6.3 | -0.322088713 | 0.748898188 | | down |
| RP11-495O10.1 | -0.322067384 | 0.811278515 | | down |
| CTC-499B15.8 | -0.321981258 | 0.576271351 | 0.693163497 | down |
| ACADM | -0.32197993 | 0.254435926 | 0.389482321 | down |
| UEVLD | -0.321864991 | 0.095095219 | 0.195046474 | down |
| RP11-638I2.2 | -0.321849539 | 0.635781873 | 0.74275501 | down |
| RP11-297K8.2 | -0.32182998 | 0.630251013 | 0.738303171 | down |
| PVALB | -0.321641561 | 0.473666076 | 0.604024771 | down |

| | | | | |
|---|---|---|---|---|
| THOC5 | -0.321572519 | 0.006553477 | 0.026661161 | down |
| RP11-982M15.7 | -0.321331743 | 0.709734684 | | down |
| NA | -0.32118587 | 0.467349249 | 0.598433313 | down |
| CTD-2026D20.3 | -0.321159927 | 0.1196413 | 0.229695394 | down |
| RNU5B-2P | -0.321121156 | 0.378886362 | 0.516951038 | down |
| RP11-143M1.3 | -0.321076048 | 0.782203923 | | down |
| RNASE4 | -0.321035871 | 0.352688271 | 0.491473395 | down |
| METTL2B | -0.320997674 | 0.013560393 | 0.046238616 | down |
| RP11-775D22.2 | -0.320976211 | 0.464164083 | 0.595674927 | down |
| DKFZp779M0652 | -0.320802242 | 0.375271056 | 0.513604392 | down |
| RP11-159F24.6 | -0.320737748 | 0.57809971 | 0.694625968 | down |
| AP001505.10 | -0.320665547 | 0.199333533 | 0.328374487 | down |
| HIGD1AP1 | -0.320529935 | 0.626652028 | 0.735607312 | down |
| ATAD5 | -0.320341684 | 0.206734746 | 0.336774858 | down |
| AC068282.3 | -0.320296935 | 0.178298514 | 0.303371893 | down |
| S100A6 | -0.320237884 | 0.219794849 | 0.351288245 | down |
| ABHD3 | -0.320193494 | 0.154142389 | 0.273864809 | down |
| PPIEL | -0.320166776 | 0.235659241 | 0.368565655 | down |
| FCER1A | -0.320138397 | 0.43945215 | 0.57341463 | down |
| NRF1 | -0.320120558 | 0.007812752 | 0.030424669 | down |
| NA | -0.320115903 | 0.559797173 | 0.67920791 | down |
| CLCN4 | -0.320080532 | 0.305717559 | 0.4439976 | down |
| CDK16 | -0.319992671 | 0.005029335 | 0.021797652 | down |
| PARP4P1 | -0.319880972 | 0.800488782 | | down |
| SWSAP1 | -0.319874947 | 0.112591188 | 0.219783618 | down |
| XRCC6 | -0.319830328 | 0.021753323 | 0.066055876 | down |
| PPA2 | -0.319775801 | 0.015228491 | 0.050506345 | down |
| TSPAN33 | -0.319772017 | 0.201835959 | 0.331002158 | down |
| RP11-395B7.4 | -0.31965661 | 0.528929575 | 0.652189632 | down |
| LIN52 | -0.319614888 | 0.174015599 | 0.298460049 | down |
| CREB3L4 | -0.319545634 | 0.098811176 | 0.20031833 | down |
| RP11-571I18.5 | -0.319416336 | 0.717528769 | | down |
| ZKSCAN3 | -0.319397426 | 0.073732246 | 0.162369831 | down |
| RNU7-29P | -0.319364809 | 0.913081195 | | down |
| NOBOX | -0.319364809 | 0.913081195 | | down |
| FAM220A | -0.319364516 | 0.909421512 | | down |
| KRT6C | -0.319363708 | 0.896297428 | | down |
| LINC01048 | -0.319363694 | 0.896002702 | | down |
| NA | -0.319363676 | 0.895645814 | | down |
| UBL5P4 | -0.319363602 | 0.894098232 | | down |
| RP11-398A8.4 | -0.319363173 | 0.883635593 | | down |
| RP11-221J22.2 | -0.319363152 | 0.883020973 | | down |
| RP11-98D18.1 | -0.319363148 | 0.882915837 | | down |
| IFNL4P1 | -0.319363091 | 0.881234223 | | down |
| RP11-380I10.2 | -0.319362458 | 0.856022122 | | down |
| RP11-32B5.8 | -0.3193622 | 0.839958519 | | down |
| RP11-565F19.4 | -0.319362187 | 0.838976024 | | down |
| RNU6-1209P | -0.319362075 | 0.829856955 | | down |
| NA | -0.319362061 | 0.828676645 | | down |
| RP11-267N12.1 | -0.319362037 | 0.826434936 | | down |
| RP11-318K12.2 | -0.319361903 | 0.812419224 | | down |

| | | | | |
|---|---|---|---|---|
| RP11-49K24.4 | -0.319361903 | 0.812424026 | | down |
| RP4-758J24.4 | -0.319361708 | 0.783978674 | | down |
| RP11-24F11.5 | -0.319344618 | 0.463354165 | 0.594991346 | down |
| RP13-216E22.5 | -0.319275251 | 0.72750972 | | down |
| AC010733.5 | -0.319185272 | 0.56886346 | 0.68679255 | down |
| NSUN5 | -0.31912731 | 0.063439847 | 0.145617447 | down |
| SLC9B1 | -0.319032484 | 0.365070185 | 0.503495207 | down |
| PRELID3B | -0.318902857 | 0.080342925 | 0.172789478 | down |
| RP11-495P10.1 | -0.318875581 | 0.284413048 | 0.421278186 | down |
| RAD50 | -0.318686793 | 0.184784076 | 0.310836282 | down |
| FRG1EP | -0.318551968 | 0.349296814 | 0.48792419 | down |
| FAAH2 | -0.318522398 | 0.093679967 | 0.193013367 | down |
| RP11-358N2.2 | -0.318481032 | 0.365630072 | 0.504106736 | down |
| ZSCAN2 | -0.318478788 | 0.082140777 | 0.175690679 | down |
| KLRC4-KLRK1 | -0.318465304 | 0.755115768 | | down |
| FAM65B | -0.318376246 | 0.310476339 | 0.44899362 | down |
| RPS26P6 | -0.318313465 | 0.40599985 | 0.542585272 | down |
| MIR1244-2 | -0.318299079 | 0.160155689 | 0.281469291 | down |
| RP11-758N13.3 | -0.318256443 | 0.637761872 | 0.744257372 | down |
| ZNF658 | -0.318230139 | 0.275769177 | 0.412051847 | down |
| ST3GAL6 | -0.318208519 | 0.214921528 | 0.345965623 | down |
| STK32C | -0.318197851 | 0.098041497 | 0.199212495 | down |
| NA | -0.318175053 | 0.569309772 | 0.687114562 | down |
| ADD2 | -0.318085371 | 0.296331161 | 0.433783541 | down |
| IKBKE | -0.318051631 | 0.130678248 | 0.244236223 | down |
| ARIH2OS | -0.31797569 | 0.146860208 | 0.265140572 | down |
| RP5-875H18.9 | -0.317965466 | 0.562303536 | 0.681355166 | down |
| AP5B1 | -0.317946212 | 0.039736553 | 0.103425885 | down |
| ACO1 | -0.317699976 | 0.044064058 | 0.111548749 | down |
| CCDC96 | -0.317630687 | 0.06978615 | 0.155966309 | down |
| FZR1 | -0.317593133 | 0.010475511 | 0.038010416 | down |
| AC002066.1 | -0.31755177 | 0.729419468 | | down |
| RP11-251G23.5 | -0.317511913 | 0.30128377 | 0.439065269 | down |
| RP11-120K19.4 | -0.317348513 | 0.478219861 | 0.607870938 | down |
| PLEKHB2 | -0.31715854 | 0.035260955 | 0.094520092 | down |
| ZNF141 | -0.317088396 | 0.122431322 | 0.23329274 | down |
| RP11-802O23.3 | -0.317045082 | 0.619500798 | 0.729807551 | down |
| UACA | -0.31701163 | 0.129545228 | 0.242601559 | down |
| RP11-299G20.2 | -0.316921352 | 0.424379704 | 0.560023887 | down |
| RP11-83M16.6 | -0.316643468 | 0.74349145 | | down |
| SSX2IP | -0.316079782 | 0.132444292 | 0.246524769 | down |
| CTC-575C13.3 | -0.316079071 | 0.829546691 | 0.888668491 | down |
| IMMT | -0.316068607 | 0.000703291 | 0.004582449 | down |
| RP11-567J20.3 | -0.316025929 | 0.634615435 | 0.741911597 | down |
| WWP2 | -0.316019848 | 0.011171785 | 0.039924481 | down |
| RND2 | -0.315999194 | 0.287277718 | 0.424480979 | down |
| FGGY | -0.315811474 | 0.086856128 | 0.182818521 | down |
| SLC6A15 | -0.315734608 | 0.62521765 | 0.734440064 | down |
| SUPT5H | -0.315534997 | 0.02284101 | 0.068418276 | down |
| RP11-379F4.8 | -0.315531193 | 0.49451532 | 0.622474371 | down |
| RPS27AP16 | -0.31550119 | 0.102726299 | 0.205904925 | down |

| | | | | |
|---|---|---|---|---|
| RPUSD2 | -0.315382394 | 0.019381884 | 0.06065722 | down |
| RP3-483K16.4 | -0.315297796 | 0.632690351 | 0.740462348 | down |
| AC068499.10 | -0.315191475 | 0.468925374 | 0.599868496 | down |
| ALMS1 | -0.315133751 | 0.132511627 | 0.24652955 | down |
| MRPL46 | -0.315039478 | 0.359066609 | 0.497385643 | down |
| IL10RB | -0.315010621 | 0.356000348 | 0.494520788 | down |
| PNMAL2 | -0.314875355 | 0.218545947 | 0.350013633 | down |
| THAP5P1 | -0.314813941 | 0.810245517 | | down |
| TMEM159 | -0.314703556 | 0.024384365 | 0.071965015 | down |
| APTX | -0.314666862 | 0.013604988 | 0.046348595 | down |
| PRG2 | -0.314648444 | 0.705926531 | | down |
| PRSS57 | -0.314635436 | 0.581592424 | 0.697539432 | down |
| TRUB2 | -0.314533661 | 0.003768883 | 0.017377583 | down |
| RASAL2-AS1 | -0.314441349 | 0.440821015 | 0.574453549 | down |
| RP11-347C12.10 | -0.314221287 | 0.126815474 | 0.238982317 | down |
| ZNF717 | -0.314109066 | 0.096079227 | 0.196421088 | down |
| PCSK6 | -0.314004944 | 0.323335401 | 0.461815488 | down |
| RP11-259G18.1 | -0.31399373 | 0.508767823 | 0.63509309 | down |
| HLA-DRB9 | -0.313811556 | 0.786218773 | | down |
| URB1-AS1 | -0.313806025 | 0.081168749 | 0.174063804 | down |
| ARHGEF39 | -0.313796741 | 0.093071763 | 0.191970894 | down |
| PGBD4 | -0.31373787 | 0.196168412 | 0.324649321 | down |
| MIR106B | -0.313731387 | 0.737202737 | | down |
| AIFM1 | -0.313679575 | 0.048504735 | 0.119750291 | down |
| RP11-295D4.4 | -0.313679317 | 0.503281014 | 0.630007031 | down |
| SLX4IP | -0.313627786 | 0.091895488 | 0.190289341 | down |
| AC017074.2 | -0.313608005 | 0.469557954 | 0.600321504 | down |
| RUVBL2 | -0.313593779 | 0.040760197 | 0.105341881 | down |
| LRRC27 | -0.313518519 | 0.120120903 | 0.230336508 | down |
| CTD-3220F14.1 | -0.313501808 | 0.520173998 | 0.644617538 | down |
| COPS9 | -0.313451923 | 0.137984042 | 0.253770358 | down |
| LINC01010 | -0.313366245 | 0.589433245 | 0.704615485 | down |
| RP11-250B2.6 | -0.313354741 | 0.237577582 | 0.370755292 | down |
| NA | -0.313319642 | 0.486163671 | 0.614950376 | down |
| CTC-537E7.3 | -0.313316941 | 0.617108402 | 0.727902509 | down |
| SOCS2-AS1 | -0.313136963 | 0.160771829 | 0.282254942 | down |
| RP5-1042K10.13 | -0.313115004 | 0.524966974 | 0.648939386 | down |
| PSMB1 | -0.313094822 | 0.026756752 | 0.077095987 | down |
| RP11-5P18.11 | -0.313094024 | 0.914781291 | | down |
| AC099344.2 | -0.313094024 | 0.914781291 | | down |
| SP9 | -0.313094024 | 0.914781291 | | down |
| AC007563.3 | -0.313094024 | 0.914781291 | | down |
| NPM1P29 | -0.313094024 | 0.914781291 | | down |
| RP11-747H12.3 | -0.313094024 | 0.914781291 | | down |
| MIR548AH | -0.313094024 | 0.914781291 | | down |
| RN7SL382P | -0.313094024 | 0.914781291 | | down |
| RP11-215G15.4 | -0.313094024 | 0.914781291 | | down |
| RP11-1379J22.7 | -0.313094024 | 0.914781291 | | down |
| RP11-795J1.2 | -0.313094024 | 0.914781291 | | down |
| PBOV1 | -0.313094024 | 0.914781291 | | down |
| RP11-359B20.1 | -0.313094024 | 0.914781291 | | down |

| | | | | |
|---|---|---|---|---|
| RP1-84015.2 | -0.313094024 | 0.914781291 | | down |
| SNORA1 | -0.313094024 | 0.914781291 | | down |
| TCF24 | -0.313094024 | 0.914781291 | | down |
| UBE2HP1 | -0.313094024 | 0.914781291 | | down |
| RP11-179D22.1 | -0.313094024 | 0.914781291 | | down |
| RP11-323H21.3 | -0.313094024 | 0.914781291 | | down |
| SLC25A18P1 | -0.313094024 | 0.914781291 | | down |
| RP11-574M7.1 | -0.313094024 | 0.914781291 | | down |
| OR8H1 | -0.313094024 | 0.914781291 | | down |
| OR4D10 | -0.313094024 | 0.914781291 | | down |
| PHBP16 | -0.313094024 | 0.914781291 | | down |
| SETP16 | -0.313094024 | 0.914781291 | | down |
| NA | -0.313094024 | 0.914781291 | | down |
| GSTP1P1 | -0.313094024 | 0.914781291 | | down |
| RP11-83B20.6 | -0.313094024 | 0.914781291 | | down |
| GPC6-AS1 | -0.313094024 | 0.914781291 | | down |
| SEPT7P1 | -0.313094024 | 0.914781291 | | down |
| RP11-545M17.1 | -0.313094024 | 0.914781291 | | down |
| NA | -0.313094024 | 0.914781291 | | down |
| RNU6-237P | -0.313094024 | 0.914781291 | | down |
| RP11-190D6.2 | -0.313094024 | 0.914781291 | | down |
| KRTAP4-3 | -0.313094024 | 0.914781291 | | down |
| MIR3678 | -0.313094024 | 0.914781291 | | down |
| ATP5LP6 | -0.313094024 | 0.914781291 | | down |
| RP4-718D20.3 | -0.313094024 | 0.914781291 | | down |
| RN7SL678P | -0.313094024 | 0.914781291 | | down |
| snoU2-30 | -0.313094024 | 0.914781291 | | down |
| NA | -0.313094024 | 0.914781291 | | down |
| RP11-277P12.9 | -0.313093298 | 0.908538522 | | down |
| MIR3168 | -0.313093288 | 0.908442462 | | down |
| IGHVIV-44-1 | -0.313093203 | 0.907618416 | | down |
| AC140076.1 | -0.313093058 | 0.90615807 | | down |
| GSTM2P1 | -0.313092159 | 0.895221051 | | down |
| CTC-507E2.2 | -0.313092067 | 0.893863425 | | down |
| OR2A1 | -0.313083369 | 0.579912656 | 0.696163976 | down |
| SNRPN | -0.312887714 | 0.242175833 | 0.376200108 | down |
| POU2F1 | -0.312882284 | 0.011449661 | 0.040704487 | down |
| IGSF9B | -0.312783486 | 0.368598009 | 0.507081761 | down |
| RPL23AP65 | -0.312663629 | 0.252634095 | 0.387627738 | down |
| PTPN21 | -0.312490139 | 0.27283226 | 0.40901015 | down |
| RPL31P2 | -0.312343298 | 0.489415082 | 0.617996332 | down |
| ADAM20 | -0.312325782 | 0.721034295 | 0.809720948 | down |
| TBX1 | -0.312139664 | 0.29511482 | 0.432550963 | down |
| USF1 | -0.312097649 | 0.05144115 | 0.12486854 | down |
| RP11-334C17.5 | -0.312029805 | 0.303711765 | 0.441746527 | down |
| RP11-452F19.3 | -0.312024879 | 0.176191147 | 0.301005732 | down |
| TLDC1 | -0.311749394 | 0.010552366 | 0.038231622 | down |
| RABGGTA | -0.311723951 | 0.053946898 | 0.129197338 | down |
| AC006372.6 | -0.311639921 | 0.700452564 | 0.79376874 | down |
| ASPRV1 | -0.311626899 | 0.240915698 | 0.374718949 | down |
| RP11-39402.3 | -0.311613606 | 0.512538308 | 0.637936488 | down |

| | | | | |
|---|---|---|---|---|
| 8-Mar | -0.311581614 | 0.018719764 | 0.059071169 | down |
| FAM53B | -0.311576428 | 0.088970561 | 0.185988834 | down |
| RP3-51008.3 | -0.311561114 | 0.81156867 | 0.87572349 | down |
| FGF4 | -0.311549829 | 0.774734459 | 0.848935812 | down |
| KLHL8 | -0.311364369 | 0.105886798 | 0.210582046 | down |
| SMUG1 | -0.311332658 | 0.017461824 | 0.056018948 | down |
| ADCY5 | -0.311200246 | 0.315502519 | 0.454147071 | down |
| ABCA12 | -0.311113466 | 0.624393711 | 0.733845193 | down |
| CPSF3L | -0.311105959 | 0.014293986 | 0.04816065 | down |
| U3 | -0.311012048 | 0.888658653 | 0.928749694 | down |
| PLAGL2 | -0.310940803 | 0.073385037 | 0.161830597 | down |
| TMEM161A | -0.310935302 | 0.129211581 | 0.242188081 | down |
| RP4-575N6.4 | -0.310761768 | 0.523318967 | 0.647500002 | down |
| ESRP2 | -0.310735007 | 0.261597529 | 0.396760875 | down |
| GLUD1 | -0.310605549 | 0.010826755 | 0.038978912 | down |
| C11orf45 | -0.310513734 | 0.306761953 | 0.445084084 | down |
| BNIP3P27 | -0.310484743 | 0.660660928 | 0.762422099 | down |
| HSPB1 | -0.31035478 | 0.210759877 | 0.341333068 | down |
| NME6 | -0.310227029 | 0.001256929 | 0.007270536 | down |
| SNAPC4 | -0.310190031 | 0.086178088 | 0.181857241 | down |
| CTD-2066L21.1 | -0.309955759 | 0.915632265 | | down |
| PPIAP33 | -0.309955759 | 0.915632265 | | down |
| RP11-344N17.11 | -0.309955759 | 0.915632265 | | down |
| PRYP2 | -0.309955305 | 0.909612412 | | down |
| RN7SL775P | -0.309953807 | 0.873784354 | | down |
| TPD52L3 | -0.309953774 | 0.872462231 | | down |
| RP11-326C3.4 | -0.309953498 | 0.859247619 | | down |
| RP11-460N11.2 | -0.309953289 | 0.846006532 | | down |
| SCOCP1 | -0.309953148 | 0.834576031 | | down |
| RNU6-1149P | -0.309952991 | 0.818310275 | | down |
| ATP5G2P3 | -0.309952985 | 0.817593435 | | down |
| ARGFXP2 | -0.309952806 | 0.791249672 | | down |
| MAGED4B | -0.309952579 | 0.732004743 | | down |
| RP11-503E24.2 | -0.309929057 | 0.439164493 | 0.573307238 | down |
| OAF | -0.309765359 | 0.145741707 | 0.263600255 | down |
| TRBV24-1 | -0.309718218 | 0.546338676 | 0.667586063 | down |
| FBXO33 | -0.309695423 | 0.010952932 | 0.039353012 | down |
| MS4A3 | -0.309456368 | 0.684392652 | 0.781470586 | down |
| COX7A2 | -0.309368984 | 0.067881999 | 0.152847695 | down |
| NA | -0.30935938 | 0.532746015 | 0.655734412 | down |
| G6PC3 | -0.30932516 | 0.089643044 | 0.187030252 | down |
| RP11-98D18.17 | -0.309266352 | 0.621414783 | 0.731431371 | down |
| RNF126 | -0.309228366 | 0.011793924 | 0.041627898 | down |
| PRR7 | -0.309044389 | 0.176880143 | 0.30180526 | down |
| TATDN2 | -0.309004967 | 0.036222485 | 0.096546161 | down |
| RP11-15B17.1 | -0.308835278 | 0.56522254 | 0.683376146 | down |
| CEACAM5 | -0.308828651 | 0.490967454 | 0.619306741 | down |
| CA6 | -0.308761143 | 0.614500997 | 0.72565317 | down |
| CACNA1G | -0.308749869 | 0.489329885 | 0.617940695 | down |
| RP11-546M4.1 | -0.308714198 | 0.830642304 | 0.889341698 | down |
| SLC25A16 | -0.30861733 | 0.001540225 | 0.008515735 | down |

| | | | | |
|---|---|---|---|---|
| AC083899.1 | -0.308590054 | 0.816198392 | 0.879329597 | down |
| ZNF326 | -0.308527197 | 0.050474551 | 0.123149052 | down |
| RP1-28H20.3 | -0.308498936 | 0.563608005 | 0.68227756 | down |
| SDHAP1 | -0.308453234 | 0.003495126 | 0.01635632 | down |
| AF011889.5 | -0.308308457 | 0.536660416 | 0.658959684 | down |
| HOXC4 | -0.308266903 | 0.210256348 | 0.340719618 | down |
| CFAP54 | -0.308252641 | 0.483100927 | 0.612236228 | down |
| SYBU | -0.308243086 | 0.184206875 | 0.310167088 | down |
| RP11-89B16.1 | -0.308239366 | 0.609455587 | 0.72139641 | down |
| RP11-46A10.5 | -0.308164121 | 0.429788942 | 0.565003377 | down |
| SLC31A1 | -0.307979515 | 0.173095535 | 0.297561496 | down |
| GBA2 | -0.307971417 | 0.055044123 | 0.131176778 | down |
| PTGES2-AS1 | -0.30793825 | 0.563955613 | 0.682347892 | down |
| LARGE1 | -0.307901029 | 0.057840217 | 0.136123413 | down |
| LCMT1-AS2 | -0.307836456 | 0.719497233 | 0.808579893 | down |
| MIR31HG | -0.307747346 | 0.64421867 | 0.749521965 | down |
| AC024937.6 | -0.307661078 | 0.512574452 | 0.637936488 | down |
| AJAP1 | -0.307652726 | 0.516722086 | 0.641716202 | down |
| U82695.9 | -0.307638203 | 0.692186033 | 0.787490047 | down |
| RP11-496H1.2 | -0.307557081 | 0.555845174 | 0.675722764 | down |
| KCNC4 | -0.307416472 | 0.13412525 | 0.248452987 | down |
| BNIP1 | -0.307032303 | 0.106741658 | 0.211833435 | down |
| RP11-266K4.14 | -0.306924675 | 0.43534957 | 0.570142648 | down |
| VPS35 | -0.306902373 | 0.015049925 | 0.050069448 | down |
| RP1-41C23.4 | -0.306780847 | 0.637811029 | 0.744257372 | down |
| LINC00672 | -0.306623622 | 0.180132391 | 0.305360129 | down |
| RP11-752G15.10 | -0.306617851 | 0.640732985 | 0.746796827 | down |
| RP4-816N1.8 | -0.306585188 | 0.564156209 | 0.682402332 | down |
| NFIC | -0.306554612 | 0.111089165 | 0.217913482 | down |
| RP11-706P11.2 | -0.306544716 | 0.563967534 | 0.682347892 | down |
| TRAIP | -0.306519425 | 0.143838269 | 0.261258506 | down |
| CHMP6 | -0.306380977 | 0.063852565 | 0.146157353 | down |
| TRANK1 | -0.306289417 | 0.124445907 | 0.235921309 | down |
| DYNC2LI1 | -0.306231496 | 0.061296531 | 0.14206663 | down |
| ADPGK-AS1 | -0.306226712 | 0.526420444 | 0.649918875 | down |
| ZNF790-AS1 | -0.306179161 | 0.221295915 | 0.352936323 | down |
| NELFA | -0.306051998 | 7.36E-05 | 0.000730861 | down |
| DPP7 | -0.306041577 | 0.25655118 | 0.391578117 | down |
| RP11-407G23.5 | -0.30594942 | 0.679003272 | 0.777361926 | down |
| WAC-AS1 | -0.305879674 | 0.012758876 | 0.044146916 | down |
| UXT | -0.305782951 | 0.216800165 | 0.348094493 | down |
| Y_RNA | -0.30575921 | 0.754849522 | 0.834266445 | down |
| WDR11-AS1 | -0.305752142 | 0.605689796 | 0.718209283 | down |
| RP11-203F10.6 | -0.305725122 | 0.489957249 | 0.618378033 | down |
| RP5-881P19.7 | -0.305605895 | 0.614255547 | 0.725391834 | down |
| RP11-785D18.3 | -0.305516896 | 0.753000107 | | down |
| CCR3 | -0.305486972 | 0.573637419 | 0.690783704 | down |
| DYDC2 | -0.305416458 | 0.679723489 | 0.777888497 | down |
| PHBP5 | -0.305342325 | 0.739412111 | | down |
| CCNT2-AS1 | -0.305280928 | 0.292456748 | 0.429766228 | down |
| ARHGAP5 | -0.305166063 | 0.396278607 | 0.533270379 | down |

| | | | | |
|---|---|---|---|---|
| MTFR1L | -0.304869319 | 0.051270005 | 0.124577292 | down |
| PTRHD1 | -0.30481576 | 0.157756529 | 0.278572773 | down |
| AL035610.2 | -0.304811482 | 0.655071835 | 0.75798106 | down |
| MUC2 | -0.304775223 | 0.504144919 | 0.630763751 | down |
| NA | -0.304651437 | 0.65355382 | 0.756778346 | down |
| SCML2 | -0.304612512 | 0.398106954 | 0.535083672 | down |
| SURF6 | -0.304572279 | 0.045926442 | 0.114870341 | down |
| CTD-2292M16.8 | -0.304535807 | 0.468908224 | 0.599868496 | down |
| AP2M1 | -0.304480819 | 0.018650499 | 0.058908283 | down |
| CH17-13I23.3 | -0.304461622 | 0.491137255 | 0.619389988 | down |
| MKS1 | -0.304388608 | 0.053985796 | 0.129249282 | down |
| CTD-3001H11.2 | -0.304371641 | 0.433686013 | 0.568674269 | down |
| MAPKAPK5 | -0.304262256 | 0.033859264 | 0.091795392 | down |
| LRRIQ1 | -0.304176379 | 0.630098596 | 0.738183593 | down |
| CDNF | -0.304087871 | 0.323604978 | 0.462087402 | down |
| RP11-876N24.1 | -0.304079753 | 0.609363669 | 0.721344447 | down |
| SCNN1G | -0.303962475 | 0.596693934 | 0.710731237 | down |
| MURC | -0.303771167 | 0.531267595 | 0.654531437 | down |
| RP11-575F12.3 | -0.303751038 | 0.262602117 | 0.397902864 | down |
| PDE3A | -0.303725441 | 0.254560125 | 0.389594956 | down |
| RP11-415K20.2 | -0.303684974 | 0.917332944 | | down |
| Metazoa_SRP | -0.303684974 | 0.917332944 | | down |
| NDUFAF4P4 | -0.303684974 | 0.917332944 | | down |
| RNU6-307P | -0.303684974 | 0.917332944 | | down |
| AKR1B1P1 | -0.303684974 | 0.917332944 | | down |
| MTND3P10 | -0.303684974 | 0.917332944 | | down |
| NA | -0.303684974 | 0.917332944 | | down |
| NPM1P33 | -0.303684974 | 0.917332944 | | down |
| COX6CP10 | -0.303684974 | 0.917332944 | | down |
| RNU6ATAC16P | -0.303684974 | 0.917332944 | | down |
| OR7E100P | -0.303684974 | 0.917332944 | | down |
| RP11-39E3.3 | -0.303684974 | 0.917332944 | | down |
| RP11-39E3.5 | -0.303684974 | 0.917332944 | | down |
| RN7SL215P | -0.303684974 | 0.917332944 | | down |
| RN7SL253P | -0.303684974 | 0.917332944 | | down |
| CTD-2154B17.1 | -0.303684974 | 0.917332944 | | down |
| RP11-281O15.2 | -0.303684974 | 0.917332944 | | down |
| RP11-451H23.1 | -0.303684974 | 0.917332944 | | down |
| RNU6-141P | -0.303684974 | 0.917332944 | | down |
| SCARNA15 | -0.303684974 | 0.917332944 | | down |
| RP3-406A7.5 | -0.303684974 | 0.917332944 | | down |
| NDUFS5P1 | -0.303684974 | 0.917332944 | | down |
| NA | -0.303684974 | 0.917332944 | | down |
| RP11-726G23.2 | -0.303684974 | 0.917332944 | | down |
| RP11-257P3.3 | -0.303684974 | 0.917332944 | | down |
| KCTD10P1 | -0.303684974 | 0.917332944 | | down |
| RN7SL726P | -0.303684974 | 0.917332944 | | down |
| RP11-424E7.6 | -0.303684974 | 0.917332944 | | down |
| AP006294.2 | -0.303684974 | 0.917332944 | | down |
| OR52E2 | -0.303684974 | 0.917332944 | | down |
| OR10A4 | -0.303684974 | 0.917332944 | | down |

| | | | | |
|---|---|---|---|---|
| PCNAP4 | -0.303684974 | 0.917332944 | | down |
| RP1-17K7.1 | -0.303684974 | 0.917332944 | | down |
| RP11-646J21.4 | -0.303684974 | 0.917332944 | | down |
| RP11-163O19.11 | -0.303684974 | 0.917332944 | | down |
| FAM138D | -0.303684974 | 0.917332944 | | down |
| RP11-200K12.3 | -0.303684974 | 0.917332944 | | down |
| NA | -0.303684974 | 0.917332944 | | down |
| FAR1P1 | -0.303684974 | 0.917332944 | | down |
| CKAP2P1 | -0.303684974 | 0.917332944 | | down |
| RP11-11K13.1 | -0.303684974 | 0.917332944 | | down |
| IGHVII-49-1 | -0.303684974 | 0.917332944 | | down |
| BMS1P15 | -0.303684974 | 0.917332944 | | down |
| RP11-114H24.3 | -0.303684974 | 0.917332944 | | down |
| AC068831.17 | -0.303684974 | 0.917332944 | | down |
| IGHV3OR16-16 | -0.303684974 | 0.917332944 | | down |
| RP11-304F15.5 | -0.303684974 | 0.917332944 | | down |
| RP11-76K13.3 | -0.303684974 | 0.917332944 | | down |
| RNU6-1242P | -0.303684974 | 0.917332944 | | down |
| RP11-94B19.5 | -0.303684974 | 0.917332944 | | down |
| AC007192.4 | -0.303684974 | 0.917332944 | | down |
| RP3-333B15.6 | -0.303684974 | 0.917332944 | | down |
| SNORA71 | -0.303684974 | 0.917332944 | | down |
| PPP1R26P3 | -0.303684974 | 0.917332944 | | down |
| EIF4BP9 | -0.303684974 | 0.917332944 | | down |
| RP5-961K14.2 | -0.303684974 | 0.917332944 | | down |
| AP000654.5 | -0.303684025 | 0.90883509 | | down |
| SNORA36 | -0.303684006 | 0.908636459 | | down |
| OR13Z2P | -0.303684004 | 0.908611393 | | down |
| RP11-307L3.4 | -0.303683929 | 0.907818887 | | down |
| NA | -0.303683897 | 0.907476366 | | down |
| RP1-152L7.8 | -0.30368388 | 0.907290277 | | down |
| RP11-113A11.3 | -0.303683868 | 0.907150961 | | down |
| AP000580.1 | -0.303683847 | 0.906922421 | | down |
| RP11-401E9.3 | -0.303683836 | 0.906802015 | | down |
| CYCSP39 | -0.303683773 | 0.906090336 | | down |
| AC087762.1 | -0.303683012 | 0.896041135 | | down |
| NA | -0.303682994 | 0.895761808 | | down |
| RP11-785H5.2 | -0.303682959 | 0.895226043 | | down |
| CTC-338M12.9 | -0.303682919 | 0.894577735 | | down |
| C8orf22 | -0.303682919 | 0.894585243 | | down |
| RP11-68I18.2 | -0.303682851 | 0.893473423 | | down |
| IRGQ | -0.303395892 | 0.03302957 | 0.090032697 | down |
| SSU72 | -0.303334999 | 0.015788459 | 0.051912025 | down |
| FAM212B | -0.303291897 | 0.157739146 | 0.27855845 | down |
| LRRC3-AS1 | -0.303226834 | 0.686995146 | 0.783574486 | down |
| SUGP1 | -0.30322416 | 0.019528242 | 0.060912221 | down |
| ADI1 | -0.303108526 | 0.013045961 | 0.044866398 | down |
| LINC01144 | -0.303096637 | 0.286228431 | 0.42330529 | down |
| LZIC | -0.303015691 | 0.007176542 | 0.028508797 | down |
| ACSS1 | -0.302994739 | 0.090726734 | 0.188558368 | down |
| PVT1 | -0.302960839 | 0.246716624 | 0.38094122 | down |

| | | | | |
|---|---|---|---|---|
| NAV2 | -0.302926169 | 0.172776664 | 0.297098334 | down |
| KCNH1-IT1 | -0.302875236 | 0.714498685 | | down |
| RRNAD1 | -0.302750272 | 0.008206889 | 0.031538473 | down |
| SNRNP25 | -0.302736396 | 0.13067699 | 0.244236223 | down |
| LINC01063 | -0.30269913 | 0.526669413 | 0.650146103 | down |
| CCDC7 | -0.302621864 | 0.219178761 | 0.350601996 | down |
| CH507-9B2.9 | -0.30239161 | 0.137131472 | 0.25248057 | down |
| NEK1 | -0.302374824 | 0.247928957 | 0.382223809 | down |
| RPL34 | -0.302373651 | 0.092342928 | 0.190939501 | down |
| RRP1 | -0.302358302 | 0.13489783 | 0.249574077 | down |
| RP11-163E9.1 | -0.302344362 | 0.455762402 | 0.588184089 | down |
| SPEG | -0.302225312 | 0.415954847 | 0.552278037 | down |
| NA | -0.302044028 | 0.402799101 | 0.539555763 | down |
| LIPA | -0.301915017 | 0.254156498 | 0.389245421 | down |
| PUF60 | -0.301820731 | 0.014740124 | 0.049271504 | down |
| NCAM2 | -0.301800255 | 0.358277448 | 0.496704705 | down |
| AC074389.5 | -0.301627456 | 0.796236357 | | down |
| LST1 | -0.301619452 | 0.263748797 | 0.39914406 | down |
| ASTE1 | -0.301586718 | 0.132484041 | 0.24652955 | down |
| RP11-111M22.2 | -0.301557866 | 0.224537836 | 0.35672438 | down |
| SH3YL1 | -0.301520952 | 0.148713206 | 0.267413189 | down |
| AC099850.1 | -0.301518204 | 0.329040327 | 0.467474705 | down |
| TRAPPC4 | -0.301302232 | 0.030793619 | 0.085441769 | down |
| RP11-93209.7 | -0.301263317 | 0.607586334 | 0.719864493 | down |
| PAK4 | -0.301239641 | 0.06238328 | 0.14385166 | down |
| RP11-282018.7 | -0.301233654 | 0.677437764 | | down |
| PCDHGA10 | -0.301208573 | 0.416935608 | 0.553140219 | down |
| NPEPL1 | -0.300811426 | 0.176752162 | 0.301689691 | down |
| ATP5SL | -0.300763815 | 0.004584352 | 0.020278605 | down |
| GNB3 | -0.300750341 | 0.290368275 | 0.427533666 | down |
| IRF3 | -0.30070363 | 0.145532221 | 0.263284763 | down |
| C22orf29 | -0.300690111 | 0.002808695 | 0.013773795 | down |
| COL21A1 | -0.300675855 | 0.458486472 | 0.590532777 | down |
| SORCS1 | -0.300675306 | 0.30009449 | 0.437735536 | down |
| CYP2E1 | -0.300659923 | 0.342728135 | 0.481182233 | down |
| RP5-837I24.1 | -0.300545198 | 0.889691839 | | down |
| RP11-681L4.1 | -0.300545182 | 0.889200695 | | down |
| RNU6ATAC | -0.300545168 | 0.888770012 | | down |
| NA | -0.300544194 | 0.836733644 | | down |
| GTF2H4 | -0.300543996 | 0.813336245 | | down |
| CTD-2340D6.2 | -0.300543983 | 0.811520777 | | down |
| CTB-17P3.4 | -0.300543909 | 0.799343145 | | down |
| RNY1P16 | -0.300543901 | 0.797953762 | | down |
| PIK3R2 | -0.300543838 | 0.785169566 | | down |
| RP11-267M23.6 | -0.300543836 | 0.784721911 | | down |
| C8orf76 | -0.300374555 | 0.278318056 | 0.414545024 | down |
| ZC4H2 | -0.300324948 | 0.023147908 | 0.06911705 | down |
| BCAR1 | -0.300242555 | 0.074304411 | 0.16319919 | down |
| RP11-864I4.1 | -0.300213477 | 0.177510452 | 0.302508606 | down |
| RP11-81A22.5 | -0.300207791 | 0.429703675 | 0.564965088 | down |
| HMGN3-AS1 | -0.300151855 | 0.279254958 | 0.415528501 | down |

| | | | | |
|---|---|---|---|---|
| MAP3K13 | -0.300147574 | 0.054751943 | 0.130708573 | down |
| LINC01503 | -0.299969301 | 0.330620121 | 0.468951375 | down |
| SNX15 | -0.299888308 | 0.490272002 | 0.618714329 | down |
| CASC19 | -0.299860184 | 0.493979665 | 0.622060736 | down |
| CTD-2545G14.6 | -0.299795343 | 0.478355217 | 0.608005572 | down |
| ARHGEF18 | -0.299751812 | 0.097233036 | 0.198114917 | down |
| RP11-499P20.2 | -0.29973836 | 0.336830143 | 0.475105776 | down |
| VPS13A | -0.299715657 | 0.09495616 | 0.194827758 | down |
| CTC-359D24.3 | -0.299706881 | 0.550748446 | 0.671031492 | down |
| AC083843.4 | -0.299689755 | 0.455868217 | 0.58827011 | down |
| RP4-640E24.1 | -0.299557647 | 0.543305939 | 0.664783075 | down |
| SPECC1 | -0.29949844 | 0.021576239 | 0.065664154 | down |
| FLJ33360 | -0.299492435 | 0.550004268 | 0.670412538 | down |
| EIF3J-AS1 | -0.299216752 | 0.071512925 | 0.158809545 | down |
| CTC-534B23.1 | -0.299192736 | 0.782851652 | | down |
| NA | -0.299165729 | 0.719209205 | 0.808371427 | down |
| AADACL4 | -0.298900764 | 0.766823332 | | down |
| THOC2 | -0.298792313 | 0.020438831 | 0.063129257 | down |
| ZFP82 | -0.298740442 | 0.153391971 | 0.273048251 | down |
| XKR9 | -0.298695422 | 0.488489838 | 0.617139258 | down |
| RP11-345P4.9 | -0.298683074 | 0.022184203 | 0.067025563 | down |
| KIAA0319L | -0.298649267 | 0.029309522 | 0.08248176 | down |
| SEC23A | -0.298622959 | 0.096849205 | 0.197565013 | down |
| SLC22A15 | -0.298438363 | 0.354823875 | 0.493419509 | down |
| RP11-565F19.2 | -0.298405006 | 0.229505229 | 0.361888492 | down |
| HES6 | -0.298329735 | 0.208445512 | 0.338625335 | down |
| STX1A | -0.298293164 | 0.136051881 | 0.251139268 | down |
| TRIM56 | -0.298274818 | 0.00290134 | 0.014121268 | down |
| CST2 | -0.298054868 | 0.669600102 | 0.769516332 | down |
| TIGAR | -0.297861023 | 0.177030232 | 0.301941316 | down |
| SPATA20 | -0.297671103 | 0.029469267 | 0.082783978 | down |
| RP3-453C12.15 | -0.29760965 | 0.727511186 | 0.8145696 | down |
| PMF1 | -0.297582637 | 0.214997416 | 0.346069239 | down |
| AC011998.4 | -0.297561868 | 0.827870578 | 0.88757605 | down |
| GUK1 | -0.297500067 | 0.077362501 | 0.168057522 | down |
| GCKR | -0.297409977 | 0.57777734 | 0.694377472 | down |
| E2F7 | -0.297159888 | 0.460911097 | 0.592791652 | down |
| CTD-2054N24.2 | -0.297153264 | 0.45376127 | 0.586331926 | down |
| CTD-2623N2.11 | -0.297003925 | 0.638840328 | 0.745197966 | down |
| SUCLG2-AS1 | -0.296954058 | 0.315758223 | 0.454376476 | down |
| TFRC | -0.296947173 | 0.11032541 | 0.2168684 | down |
| SPPL2A | -0.296838867 | 0.091234447 | 0.189299247 | down |
| NA | -0.296613408 | 0.641745622 | 0.747534142 | down |
| RPL5P17 | -0.296595321 | 0.415373425 | 0.551858195 | down |
| ZNF568 | -0.296592926 | 0.224320062 | 0.356585818 | down |
| KCNIP3 | -0.296471536 | 0.306754791 | 0.445084084 | down |
| NA | -0.29632048 | 0.784542301 | | down |
| STYXL1 | -0.296249498 | 0.141130923 | 0.257771511 | down |
| RP11-285F7.2 | -0.296227082 | 0.170335487 | 0.294180235 | down |
| RNU6-650P | -0.296134266 | 0.902511682 | | down |
| CSNK1G1 | -0.295893967 | 0.014051689 | 0.047509446 | down |

| | | | | |
|---|---|---|---|---|
| CCDC65 | -0.295757228 | 0.254528562 | 0.389594956 | down |
| WDTC1 | -0.29573011 | 0.007910219 | 0.030708698 | down |
| MEGF8 | -0.295658267 | 0.032219997 | 0.088390562 | down |
| GFRA3 | -0.295637429 | 0.414375421 | 0.550886993 | down |
| GINS2 | -0.295612948 | 0.194587661 | 0.322904816 | down |
| CYP26C1 | -0.295512245 | 0.62022325 | 0.73051542 | down |
| AC002310.7 | -0.295462679 | 0.745691227 | | down |
| ZSWIM3 | -0.295445235 | 0.150265042 | 0.269333189 | down |
| CCDC120 | -0.295421062 | 0.104867643 | 0.208900906 | down |
| TRAV1-1 | -0.29535984 | 0.670434321 | 0.770180428 | down |
| NSG1 | -0.295177851 | 0.224961354 | 0.357074526 | down |
| CELSR3 | -0.29516715 | 0.225247869 | 0.357398837 | down |
| NA | -0.295153637 | 0.621891742 | 0.731792084 | down |
| RP3-329A5.8 | -0.295121897 | 0.360931667 | 0.499347532 | down |
| AC002511.3 | -0.295073259 | 0.61386516 | 0.725073316 | down |
| AC145124.2 | -0.295035836 | 0.511522517 | 0.637220298 | down |
| AC083900.1 | -0.294987725 | 0.633108017 | 0.740699719 | down |
| INTS3 | -0.294986761 | 0.022066703 | 0.066757831 | down |
| RP11-723J4.3 | -0.294978391 | 0.652762382 | 0.756153339 | down |
| SLC52A2 | -0.294649589 | 0.233307502 | 0.366069072 | down |
| ATE1 | -0.294646471 | 0.009727924 | 0.035865228 | down |
| POTEF | -0.294635956 | 0.650079071 | 0.754091722 | down |
| RP11-731C17.2 | -0.294611735 | 0.363700542 | 0.502068511 | down |
| RP11-44K6.4 | -0.29457983 | 0.785946338 | | down |
| SULT4A1 | -0.294561847 | 0.515018296 | 0.640208933 | down |
| SVILP1 | -0.294335862 | 0.41974357 | 0.555516313 | down |
| NELFE | -0.294292199 | 0.075569378 | 0.165206021 | down |
| RP11-132G19.3 | -0.294275924 | 0.919885449 | | down |
| SMCP | -0.294275924 | 0.919885449 | | down |
| AC093822.1 | -0.294275924 | 0.919885449 | | down |
| AC007271.3 | -0.294275924 | 0.919885449 | | down |
| AC012451.1 | -0.294275924 | 0.919885449 | | down |
| RNU6-136P | -0.294275924 | 0.919885449 | | down |
| RN7SL499P | -0.294275924 | 0.919885449 | | down |
| NA | -0.294275924 | 0.919885449 | | down |
| RPL12P2 | -0.294275924 | 0.919885449 | | down |
| GUSBP4 | -0.294275924 | 0.919885449 | | down |
| RP11-161I2.1 | -0.294275924 | 0.919885449 | | down |
| RNY1P1 | -0.294275924 | 0.919885449 | | down |
| RNA5SP382 | -0.294275924 | 0.919885449 | | down |
| SDCCAG3P1 | -0.294275924 | 0.919885449 | | down |
| CTD-3138B18.4 | -0.294275924 | 0.919885449 | | down |
| RPL5P2 | -0.294275924 | 0.919885449 | | down |
| RN7SL280P | -0.294275924 | 0.919885449 | | down |
| FAM47A | -0.294275924 | 0.919885449 | | down |
| RNU6-494P | -0.294275917 | 0.919831005 | | down |
| NA | -0.294274915 | 0.91068654 | | down |
| CBX1P1 | -0.294274897 | 0.910492001 | | down |
| RPL12P19 | -0.294274772 | 0.909114146 | | down |
| RP11-335K5.2 | -0.294274743 | 0.908780602 | | down |
| RP13-379L11.2 | -0.294274734 | 0.908676061 | | down |

| | | | | |
|---|---|---|---|---|
| RP11-266I3.9 | -0.294274701 | 0.90830364 | | down |
| RP11-69M1.1 | -0.294274683 | 0.908088891 | | down |
| CTD-2123J17.2 | -0.294274633 | 0.907495908 | | down |
| ATP5G1P5 | -0.294274628 | 0.907441338 | | down |
| RP11-490G2.2 | -0.294273894 | 0.897172358 | | down |
| SCARNA22 | -0.29427388 | 0.896937223 | | down |
| LINC00272 | -0.294273859 | 0.896589655 | | down |
| RP4-799G3.2 | -0.294273814 | 0.895845312 | | down |
| RP4-769N13.7 | -0.294273197 | 0.883399541 | | down |
| RPSAP20 | -0.294273193 | 0.883318653 | | down |
| CTC-348L5.1 | -0.294273154 | 0.882370627 | | down |
| DAB1-AS1 | -0.294273139 | 0.881991405 | | down |
| RP11-147G7.3 | -0.294272732 | 0.87039817 | | down |
| NA | -0.294272303 | 0.853526864 | | down |
| GLOD4 | -0.294230738 | 0.068618177 | 0.154055038 | down |
| EXOSC6 | -0.294201452 | 0.02369405 | 0.070363155 | down |
| CAPG | -0.294186627 | 0.165145315 | 0.287897518 | down |
| RP11-165A20.3 | -0.294101088 | 0.492332937 | 0.620533237 | down |
| AC008982.2 | -0.294091444 | 0.532832475 | 0.655787099 | down |
| FHIT | -0.293855222 | 0.113666952 | 0.221322742 | down |
| RP11-83J16.1 | -0.293739116 | 0.447370743 | 0.580621479 | down |
| ZNF212 | -0.293737676 | 0.027453776 | 0.07862929 | down |
| LINC00921 | -0.293729979 | 0.340683149 | 0.479054785 | down |
| LA16c-325D7.2 | -0.293665991 | 0.584740869 | 0.700533776 | down |
| RP5-837J1.6 | -0.293591061 | 0.569518768 | 0.687201167 | down |
| GPBP1L1 | -0.293544086 | 0.039710766 | 0.103403559 | down |
| INTS6 | -0.293524254 | 0.047856054 | 0.118472915 | down |
| HMGB3P32 | -0.293475798 | 0.743157044 | 0.825753907 | down |
| ANAPC2 | -0.293299829 | 0.052983636 | 0.127449187 | down |
| DIS3 | -0.293267989 | 0.023466549 | 0.069844144 | down |
| RP11-296A18.6 | -0.29314074 | 0.7312807 | | down |
| AFG3L2 | -0.293062834 | 0.280334743 | 0.416681197 | down |
| NA | -0.293034663 | 0.470451145 | 0.601098674 | down |
| NABP2 | -0.293025288 | 0.045693714 | 0.114439975 | down |
| LINC00877 | -0.292926352 | 0.406092391 | 0.542661686 | down |
| IL20RB | -0.292912033 | 0.279141292 | 0.41542109 | down |
| MUCL1 | -0.292910577 | 0.814573942 | | down |
| RNF103-CHMP3 | -0.292861654 | 0.62735222 | 0.736084124 | down |
| RP11-517O13.3 | -0.292814183 | 0.771588291 | | down |
| LINC00634 | -0.292589633 | 0.484071402 | 0.613078206 | down |
| NPLOC4 | -0.292438097 | 0.00853947 | 0.032488268 | down |
| AC008063.2 | -0.292358543 | 0.623120676 | 0.732778988 | down |
| RP11-453E17.3 | -0.29234296 | 0.686724613 | | down |
| TSNARE1 | -0.292315872 | 0.112177484 | 0.21931804 | down |
| SCGN | -0.292249403 | 0.408956015 | 0.545759096 | down |
| OR7E12P | -0.292202641 | 0.598303474 | 0.71204888 | down |
| BBS1 | -0.292202603 | 0.450963413 | 0.583922113 | down |
| SLC16A13 | -0.292166062 | 0.222006031 | 0.353843459 | down |
| RNU7-140P | -0.292134035 | 0.6020542 | 0.715030098 | down |
| B4GALT6 | -0.292048748 | 0.281369896 | 0.417785764 | down |
| ASIP | -0.292011557 | 0.479191377 | 0.608682652 | down |

| | | | | |
|---|---|---|---|---|
| CSRNP3 | -0.291966186 | 0.521976168 | 0.646237771 | down |
| RBM42 | -0.29190904 | 0.054642448 | 0.130509397 | down |
| GPR62 | -0.291615838 | 0.636843941 | 0.743648777 | down |
| U3 | -0.291602771 | 0.669193078 | 0.769195685 | down |
| XPNPEP1 | -0.291431083 | 0.063136312 | 0.145098092 | down |
| RP11-474D14.2 | -0.291347096 | 0.732030642 | 0.81795331 | down |
| NRSN1 | -0.291339441 | 0.59729525 | 0.711149183 | down |
| NUDT22 | -0.291283339 | 0.080895879 | 0.173652171 | down |
| NDUFB1 | -0.291150858 | 0.096394495 | 0.196911474 | down |
| AC069363.1 | -0.291147878 | 0.667128508 | 0.767527337 | down |
| AZGP1P2 | -0.291137659 | 0.920736988 | | down |
| CTA-481E9.3 | -0.291137659 | 0.920736988 | | down |
| RP11-50B3.2 | -0.29113565 | 0.869623561 | | down |
| CTB-4E7.1 | -0.291135453 | 0.857181223 | | down |
| ZNF807 | -0.291135015 | 0.808025748 | | down |
| RP11-1260E13.1 | -0.290999413 | 0.751503006 | | down |
| RP11-1167A19.2 | -0.290968659 | 0.767907126 | | down |
| RP11-536C5.7 | -0.290835386 | 0.506368184 | 0.632675943 | down |
| NA | -0.290801465 | 0.684296799 | 0.781462081 | down |
| RAB6B | -0.290789702 | 0.233197331 | 0.365915319 | down |
| PARGP1 | -0.290783905 | 0.426855985 | 0.562427033 | down |
| GOLGA6L4 | -0.290760746 | 0.563637912 | 0.68227756 | down |
| ZSCAN5A | -0.290668402 | 0.072835688 | 0.160949943 | down |
| RP5-1056L3.3 | -0.290557449 | 0.390211823 | 0.527943979 | down |
| TAZ | -0.290523286 | 0.171820005 | 0.295995427 | down |
| UBL7 | -0.290310923 | 0.073833459 | 0.16251352 | down |
| SCAMP4 | -0.29019656 | 0.070723905 | 0.157511417 | down |
| RP11-156K23.3 | -0.290162871 | 0.23745669 | 0.370643641 | down |
| SCML4 | -0.290123919 | 0.297162732 | 0.4345562 | down |
| RP11-111H13.1 | -0.290110032 | 0.540309465 | 0.662114352 | down |
| CSPG4P13 | -0.290035422 | 0.721086494 | 0.809720948 | down |
| VDAC1P1 | -0.290035422 | 0.621603764 | 0.731550645 | down |
| RP1-111C20.4 | -0.289998651 | 0.269203069 | 0.405004357 | down |
| RP11-274B21.14 | -0.289830041 | 0.47889018 | 0.608435997 | down |
| OVOS2 | -0.289753287 | 0.333473585 | 0.47173124 | down |
| NMB | -0.28968857 | 0.275760524 | 0.412051847 | down |
| RP11-834C11.4 | -0.289680965 | 0.191333487 | 0.319080584 | down |
| HIBCH | -0.289669822 | 0.027436694 | 0.078595352 | down |
| U1 | -0.289386525 | 0.462223759 | 0.594022184 | down |
| GAS5-AS1 | -0.289287773 | 0.2793936 | 0.415652455 | down |
| AC012442.5 | -0.289145539 | 0.576282713 | 0.693163497 | down |
| IL18 | -0.289097881 | 0.202114878 | 0.331369136 | down |
| ATP13A1 | -0.288962944 | 0.212799236 | 0.343635595 | down |
| NSUN5P1 | -0.288927201 | 0.238220095 | 0.371622853 | down |
| RP11-176H8.3 | -0.288919071 | 0.707665925 | | down |
| CTD-2562G15.3 | -0.288823811 | 0.540606281 | 0.662424043 | down |
| KCNE1 | -0.288743425 | 0.610922154 | 0.722619889 | down |
| OPA3 | -0.288677834 | 0.001820265 | 0.009741557 | down |
| ATP5G3 | -0.288656322 | 0.069730335 | 0.155911154 | down |
| KMT2C | -0.288539668 | 0.18796071 | 0.314776565 | down |
| SPSB3 | -0.288514691 | 0.502558118 | 0.62943372 | down |

| | | | | |
|---|---|---|---|---|
| CTA-126B4.7 | -0.288471903 | 0.537132496 | 0.659296838 | down |
| RP1-199J3.6 | -0.28844521 | 0.202276788 | 0.331548111 | down |
| GPX2 | -0.288443026 | 0.423907845 | 0.559563558 | down |
| CAMTA1 | -0.288417976 | 0.008798863 | 0.033264509 | down |
| CD1E | -0.288367666 | 0.457288404 | 0.589368659 | down |
| PSTK | -0.288249448 | 0.1559037 | 0.276226382 | down |
| NOL12 | -0.288194731 | 0.231916455 | 0.364629126 | down |
| CLUHP3 | -0.288168304 | 0.160497497 | 0.281921585 | down |
| SLC25A13 | -0.28815286 | 0.042838622 | 0.109358786 | down |
| RP1-225E12.2 | -0.287895916 | 0.644435233 | 0.749686832 | down |
| LRTOMT | -0.287887709 | 0.059310804 | 0.138524785 | down |
| AIM1 | -0.287783982 | 0.121157021 | 0.231669636 | down |
| RP11-958N24.1 | -0.287779666 | 0.505561489 | 0.63203601 | down |
| HAUS8 | -0.287755993 | 0.118995912 | 0.228748683 | down |
| HMGN1P38 | -0.287688012 | 0.541780765 | 0.663310795 | down |
| DPY19L2P2 | -0.287665649 | 0.495755485 | 0.623490236 | down |
| RNA5SP383 | -0.287551557 | 0.535792237 | 0.658351068 | down |
| LA16c-60H5.7 | -0.287442083 | 0.692471981 | 0.787696055 | down |
| AC105760.2 | -0.287396062 | 0.385639936 | 0.523391793 | down |
| LINC00294 | -0.287355829 | 0.225035535 | 0.357137373 | down |
| PALM | -0.287231128 | 0.267626257 | 0.403379484 | down |
| NUP50-AS1 | -0.287048869 | 0.21685777 | 0.348149773 | down |
| UNC13B | -0.28690785 | 0.091515487 | 0.189685499 | down |
| USP46-AS1 | -0.286796099 | 0.270022098 | 0.406012908 | down |
| LENG9 | -0.286755659 | 0.196392538 | 0.324894962 | down |
| DYNC1I2P1 | -0.286741571 | 0.545246388 | 0.666528947 | down |
| CCDC138 | -0.286726049 | 0.322514123 | 0.461155469 | down |
| WDR59 | -0.286694992 | 0.037524235 | 0.09904051 | down |
| POU2F2 | -0.286632604 | 0.371597066 | 0.509900079 | down |
| PLCXD2 | -0.286442219 | 0.384391906 | 0.522091791 | down |
| CYP11A1 | -0.28633725 | 0.366555267 | 0.505081015 | down |
| ABCC5 | -0.286316502 | 0.041350581 | 0.106482973 | down |
| DUSP27 | -0.286269947 | 0.698738777 | 0.792603935 | down |
| RP11-217B1.2 | -0.286132054 | 0.515557586 | 0.640720253 | down |
| ESPL1 | -0.286130491 | 0.36799653 | 0.506584489 | down |
| LA16c-444G7.2 | -0.286111236 | 0.736016396 | 0.820560584 | down |
| RP11-253I19.4 | -0.286032841 | 0.876969238 | | down |
| CDK2AP2 | -0.286014397 | 0.130524826 | 0.244025381 | down |
| RP1-267L14.6 | -0.285932025 | 0.69291266 | | down |
| RP11-864N7.2 | -0.285924423 | 0.179977357 | 0.305228133 | down |
| MCM8 | -0.285897716 | 0.148699134 | 0.26740389 | down |
| RP11-405M12.4 | -0.28586322 | 0.453643597 | 0.586255516 | down |
| ACOT2 | -0.285819438 | 0.046057811 | 0.115111959 | down |
| RP11-139H15.6 | -0.285804025 | 0.57716871 | 0.693853318 | down |
| STRIP2 | -0.285707415 | 0.461722619 | 0.59353048 | down |
| RP11-983P16.4 | -0.285612645 | 0.149775523 | 0.268712278 | down |
| AB019441.29 | -0.285571729 | 0.275204497 | 0.411478247 | down |
| PPP1R7 | -0.285368277 | 0.021006504 | 0.0643868 | down |
| DNAH1 | -0.285364044 | 0.113626 | 0.221271684 | down |
| GSDMA | -0.285282329 | 0.473887605 | 0.604127772 | down |
| PRR19 | -0.285212015 | 0.390560085 | 0.528248647 | down |

| | | | | |
|---|---|---|---|---|
| RPS20P5 | -0.284866874 | 0.922438779 | | down |
| RP1-140J1.1 | -0.284866874 | 0.922438779 | | down |
| ICE2P1 | -0.284866874 | 0.922438779 | | down |
| CTC-250P20.1 | -0.284866874 | 0.922438779 | | down |
| RP11-395D3.1 | -0.284866874 | 0.922438779 | | down |
| Y_RNA | -0.284866874 | 0.922438779 | | down |
| PRO1804 | -0.284866874 | 0.922438779 | | down |
| IGKV2OR2-1 | -0.284865683 | 0.911124219 | | down |
| NA | -0.284865668 | 0.910939725 | | down |
| AC104651.2 | -0.284865623 | 0.910408114 | | down |
| RP11-354E23.3 | -0.284865533 | 0.909317944 | | down |
| RP11-717F1.2 | -0.284864892 | 0.900129511 | | down |
| CTD-3030D20.1 | -0.284864842 | 0.899291985 | | down |
| KRT8P14 | -0.284864816 | 0.898856624 | | down |
| SFTPA1 | -0.284864791 | 0.898421589 | | down |
| RPL12P9 | -0.284864213 | 0.886529499 | | down |
| SNORA14 | -0.284864202 | 0.886259752 | | down |
| RPL7P59 | -0.284864183 | 0.885809424 | | down |
| RP11-319F12.2 | -0.284864163 | 0.885293575 | | down |
| TRAJ12 | -0.284864161 | 0.885242806 | | down |
| CDC42EP2 | -0.284864157 | 0.885160691 | | down |
| MIR4294 | -0.284864119 | 0.884178051 | | down |
| FAM25G | -0.284863745 | 0.873089796 | | down |
| RP11-797H7.5 | -0.284863721 | 0.872283529 | | down |
| ATP5J2-PTCD1 | -0.284863682 | 0.870914029 | | down |
| KRT8P42 | -0.284862929 | 0.830861124 | | down |
| ATPAF2 | -0.284793365 | 0.033106396 | 0.09017666 | down |
| Xbac-BPG154L12. | -0.284760477 | 0.381740317 | 0.519547274 | down |
| RETN | -0.284729548 | 0.671923387 | 0.771389616 | down |
| LINC01093 | -0.284726445 | 0.792926888 | 0.862005213 | down |
| RMDN3 | -0.284698777 | 0.172322987 | 0.296657794 | down |
| SCN4B | -0.284529542 | 0.380970362 | 0.518851929 | down |
| VANGL2 | -0.284510309 | 0.19825673 | 0.326988647 | down |
| CTD-3222D19.12 | -0.284492848 | 0.428958462 | 0.564306333 | down |
| CASP5 | -0.284476815 | 0.441948381 | 0.575570675 | down |
| MLPH | -0.284376014 | 0.273597196 | 0.409891002 | down |
| RP11-684B2.3 | -0.28437286 | 0.723582432 | | down |
| ANKEF1 | -0.284316314 | 0.213558808 | 0.344436376 | down |
| RP11-778D9.4 | -0.284226751 | 0.27444265 | 0.410809375 | down |
| ZSCAN20 | -0.284139351 | 0.238509037 | 0.371938415 | down |
| KIAA0232 | -0.284118689 | 0.083693164 | 0.177945402 | down |
| IQSEC2 | -0.283928639 | 0.024577339 | 0.072385215 | down |
| SLC9A7 | -0.28392532 | 0.293955166 | 0.431398448 | down |
| ANXA9 | -0.28377787 | 0.166429371 | 0.289531212 | down |
| DIO3 | -0.283685985 | 0.477864431 | 0.60752196 | down |
| RP11-333E1.2 | -0.283598193 | 0.514266664 | 0.639592153 | down |
| IL11RA | -0.283476313 | 0.14982246 | 0.268747529 | down |
| NIPA1 | -0.283413021 | 0.102763473 | 0.20593822 | down |
| CTD-2561B21.10 | -0.28341219 | 0.728576454 | | down |
| RP11-475C16.1 | -0.283331705 | 0.112777925 | 0.22001561 | down |
| NRARP | -0.283249596 | 0.248975629 | 0.383522549 | down |

| | | | | |
|---|---|---|---|---|
| RP1-257A7.4 | -0.283244073 | 0.433034421 | 0.568021562 | down |
| USP27X | -0.283232039 | 0.191372742 | 0.319120978 | down |
| TTLL7 | -0.283126857 | 0.417641981 | 0.553836272 | down |
| NPIPB9 | -0.283106174 | 0.707126215 | 0.798740507 | down |
| BTBD10P2 | -0.283027487 | 0.759341345 | | down |
| ARTN | -0.282863463 | 0.427900644 | 0.563284711 | down |
| RP11-84A12.1 | -0.282860504 | 0.779583785 | | down |
| RP11-364L4.1 | -0.282769455 | 0.428049614 | 0.563453245 | down |
| TNFSF10 | -0.282715662 | 0.179542636 | 0.304712106 | down |
| TEX9 | -0.282710791 | 0.356163221 | 0.49457237 | down |
| UBA7 | -0.282597615 | 0.111621918 | 0.218743122 | down |
| NA | -0.282446646 | 0.469686213 | 0.600351099 | down |
| RNU7-115P | -0.282305379 | 0.923133866 | | down |
| CTBP1-AS | -0.282284472 | 0.505354071 | 0.631881876 | down |
| RP11-370F5.4 | -0.282272512 | 0.861635962 | | down |
| RN7SL693P | -0.282272379 | 0.861433865 | | down |
| PLIN1 | -0.282264457 | 0.537844034 | 0.66003538 | down |
| OR10AC1 | -0.282256188 | 0.838771715 | | down |
| FAM60CP | -0.282236218 | 0.814626339 | | down |
| RP11-360D2.2 | -0.282209941 | 0.787164177 | | down |
| ARID4B | -0.282184372 | 0.087783031 | 0.184191918 | down |
| EEF1A1P1 | -0.282151805 | 0.737151486 | | down |
| RRP7BP | -0.28207701 | 0.137196597 | 0.252554044 | down |
| RP5-1031D4.3 | -0.282026047 | 0.36741778 | 0.505944623 | down |
| UCP3 | -0.281931515 | 0.358527698 | 0.496845302 | down |
| CABLES1 | -0.28190971 | 0.139342694 | 0.255487116 | down |
| TTC1 | -0.281775267 | 0.064146102 | 0.146644764 | down |
| PKP1 | -0.28176747 | 0.395195478 | 0.532576162 | down |
| ZNF404 | -0.281746652 | 0.352964383 | 0.49170141 | down |
| RP11-35015.2 | -0.281742892 | 0.691293532 | 0.786951458 | down |
| RP11-665C16.9 | -0.28169643 | 0.51031385 | 0.636184063 | down |
| RP11-7807.2 | -0.281690512 | 0.392425021 | 0.529936054 | down |
| ZNF490 | -0.281659664 | 0.139421249 | 0.255599952 | down |
| RP4-665N4.8 | -0.281522734 | 0.559967044 | 0.679347249 | down |
| COQ7 | -0.281400398 | 0.00346621 | 0.016249316 | down |
| RP3-518E13.2 | -0.281397453 | 0.775947243 | | down |
| CTC-398G3.2 | -0.281384378 | 0.702529812 | | down |
| RP11-456N14.6 | -0.281349674 | 0.627648575 | 0.736316821 | down |
| GSK3B | -0.281333702 | 0.036805982 | 0.097601474 | down |
| UCN | -0.281291857 | 0.332292319 | 0.470622405 | down |
| RP11-707023.1 | -0.281205841 | 0.54204759 | 0.663460832 | down |
| KIAA0513 | -0.281124405 | 0.066218831 | 0.150058724 | down |
| RP1-122P22.4 | -0.281109167 | 0.63719703 | 0.7438652 | down |
| AC013439.4 | -0.281070881 | 0.690610752 | 0.786340767 | down |
| ADAT1 | -0.280936687 | 0.092281228 | 0.190838189 | down |
| SOX5 | -0.280693896 | 0.359957099 | 0.498205697 | down |
| CTD-2245F17.3 | -0.280593151 | 0.385456595 | 0.523230378 | down |
| P2RX5-TAX1BP3 | -0.280589447 | 0.417132142 | 0.553327656 | down |
| AC008592.4 | -0.28057055 | 0.629227951 | 0.737536071 | down |
| AC008427.2 | -0.280423664 | 0.780783857 | | down |
| RANGRF | -0.280393504 | 0.466220587 | 0.597522878 | down |

| | | | | |
|---|---|---|---|---|
| MYL12B | -0.280329686 | 0.105985751 | 0.210695158 | down |
| AC004057.1 | -0.280321444 | 0.206948524 | 0.337013467 | down |
| CCDC25 | -0.28022033 | 0.054236678 | 0.129723262 | down |
| RP11-8L8.2 | -0.280066179 | 0.594075305 | 0.7084947 | down |
| TTC23 | -0.279995041 | 0.027935117 | 0.079681006 | down |
| ALKBH3 | -0.279857348 | 0.069906521 | 0.156165626 | down |
| CTSZ | -0.279688168 | 0.078169906 | 0.169325708 | down |
| MT2P1 | -0.279495682 | 0.569149525 | 0.68700395 | down |
| RASSF6 | -0.279417246 | 0.34103545 | 0.479393115 | down |
| CH507-9B2.1 | -0.279362311 | 0.500731023 | 0.627825843 | down |
| LAMTOR1 | -0.279238991 | 0.030340398 | 0.084504209 | down |
| SLC39A10 | -0.279174756 | 0.222562877 | 0.35446772 | down |
| SNORA81 | -0.27916529 | 0.688105679 | | down |
| CTC-338M12.4 | -0.279115599 | 0.43507993 | 0.569888887 | down |
| RPL23AP42 | -0.279021036 | 0.65776746 | 0.759958682 | down |
| ZNF365 | -0.278966172 | 0.654371118 | 0.757491171 | down |
| AC006946.16 | -0.278939467 | 0.511994943 | 0.637505742 | down |
| RP11-6B6.3 | -0.278913788 | 0.741204238 | | down |
| ALOX12-AS1 | -0.278836081 | 0.226792001 | 0.358891372 | down |
| LLOXNC01-7P3.1 | -0.278799863 | 0.408162853 | 0.544924028 | down |
| RP5-1085F17.3 | -0.278793607 | 0.118543418 | 0.228112371 | down |
| NA | -0.278745452 | 0.440286066 | 0.574055226 | down |
| ALOX12B | -0.2787257 | 0.579289046 | 0.695637719 | down |
| PYROXD1 | -0.278642556 | 0.251591975 | 0.386403707 | down |
| AC079922.3 | -0.278498107 | 0.170855264 | 0.294722143 | down |
| CTD-2302E22.4 | -0.278497755 | 0.706357541 | 0.798029788 | down |
| SEPSECS-AS1 | -0.278478364 | 0.25302255 | 0.388005793 | down |
| RGS5 | -0.278282583 | 0.437831875 | 0.572270984 | down |
| RHOT2 | -0.278157139 | 0.104751543 | 0.20873883 | down |
| RN7SL32P | -0.278109076 | 0.740906542 | 0.824040134 | down |
| MFN2 | -0.278041403 | 0.118162217 | 0.2275829 | down |
| AC012354.8 | -0.277906117 | 0.668848879 | 0.769005997 | down |
| OSBPL2 | -0.277612273 | 0.000853621 | 0.005343931 | down |
| DLGAP1-AS2 | -0.277544564 | 0.322643202 | 0.46119956 | down |
| RP11-894J14.5 | -0.277508992 | 0.47145613 | 0.602049898 | down |
| GPR137 | -0.277339247 | 0.183261267 | 0.309036104 | down |
| RP11-325E5.1 | -0.277289703 | 0.679676391 | | down |
| RP3-508I15.22 | -0.277137932 | 0.513573462 | 0.638888704 | down |
| NA | -0.277129034 | 0.470598622 | 0.601210401 | down |
| PTPN18 | -0.277085221 | 0.092069526 | 0.19054466 | down |
| RP11-297D21.4 | -0.276956861 | 0.257655259 | 0.392664837 | down |
| TFEB | -0.276769175 | 0.233454134 | 0.366119783 | down |
| FBXL6 | -0.2767377 | 0.146543412 | 0.264731542 | down |
| IPO5P1 | -0.276600418 | 0.184167605 | 0.310118346 | down |
| HSPA4L | -0.276565931 | 0.409033405 | 0.545795135 | down |
| PDLIM5 | -0.276555515 | 0.086348819 | 0.182091944 | down |
| CYCS | -0.27646825 | 0.148862489 | 0.267585535 | down |
| CDAN1 | -0.276448399 | 0.057987259 | 0.136351977 | down |
| NBPF2P | -0.276400426 | 0.666346607 | 0.766862693 | down |
| DGUOK-AS1 | -0.276363203 | 0.363776379 | 0.502146253 | down |
| CTD-2547L24.4 | -0.276342088 | 0.474363393 | 0.604456882 | down |

| | | | | |
|---|---|---|---|---|
| VPS53 | -0.276335347 | 0.007053111 | 0.028144995 | down |
| NA | -0.276306361 | 0.730938187 | 0.817127698 | down |
| SOD1 | -0.27615159 | 0.092621626 | 0.191344574 | down |
| ZNF213 | -0.27597969 | 0.023654376 | 0.07028007 | down |
| CCL5 | -0.275739255 | 0.278080862 | 0.414314973 | down |
| TLE3 | -0.275660127 | 0.031765548 | 0.087476088 | down |
| FADS2P1 | -0.275630127 | 0.816970357 | | down |
| GIMAP3P | -0.275603194 | 0.702187854 | 0.795045482 | down |
| TNK2-AS1 | -0.275559453 | 0.572342458 | 0.689676193 | down |
| ERV3-1 | -0.275524535 | 0.333503588 | 0.471741139 | down |
| RNU6-110P | -0.275457824 | 0.924992909 | | down |
| RP11-174F8.1 | -0.275457824 | 0.924992909 | | down |
| MTND4P5 | -0.275457824 | 0.924992909 | | down |
| RP11-632K21.7 | -0.275457824 | 0.924992909 | | down |
| SDHCP4 | -0.275457824 | 0.924992909 | | down |
| NA | -0.275457824 | 0.924992909 | | down |
| AC010907.5 | -0.275457792 | 0.924738817 | | down |
| RP11-167H9.3 | -0.2754577 | 0.924006403 | | down |
| RP4-800M22.2 | -0.275457682 | 0.923862773 | | down |
| TBC1D26 | -0.275457643 | 0.923541356 | | down |
| RP11-119F7.6 | -0.27545763 | 0.923434583 | | down |
| CGB2 | -0.275457512 | 0.922439481 | | down |
| Metazoa_SRP | -0.275456667 | 0.913969255 | | down |
| CTC-28708.1 | -0.275456637 | 0.913619075 | | down |
| RP11-368I23.3 | -0.275456608 | 0.91327121 | | down |
| TSNAX-DISC1 | -0.275456585 | 0.912988576 | | down |
| IGHV3-38 | -0.275456555 | 0.912626263 | | down |
| RP11-161D15.3 | -0.275456488 | 0.911790884 | | down |
| NA | -0.275456453 | 0.911345717 | | down |
| RNU4-82P | -0.275455755 | 0.900748458 | | down |
| RP11-430C7.2 | -0.275455685 | 0.899455305 | | down |
| RP11-1394016.1 | -0.27545566 | 0.899000006 | | down |
| Y_RNA | -0.275455205 | 0.889072122 | | down |
| RNU6-1262P | -0.275455191 | 0.888716408 | | down |
| RP1-12208.7 | -0.275455176 | 0.8883168 | | down |
| RNU6-341P | -0.275454772 | 0.876232775 | | down |
| NA | -0.275454448 | 0.863125014 | | down |
| NA | -0.275454207 | 0.850070989 | | down |
| RP11-172C16.5 | -0.275454168 | 0.847578412 | | down |
| PCDHA13 | -0.275438744 | 0.791697084 | | down |
| LOC401913 | -0.27542316 | 0.728701821 | | down |
| SPATA41 | -0.275324382 | 0.342962209 | 0.481380828 | down |
| PP12613 | -0.275306573 | 0.780128011 | | down |
| DGCR8 | -0.275243619 | 0.058609365 | 0.13736327 | down |
| DMC1 | -0.275194921 | 0.392854777 | 0.530301879 | down |
| RP11-234B24.6 | -0.275168573 | 0.697813793 | 0.791883573 | down |
| MKKS | -0.275084791 | 0.062455986 | 0.143964066 | down |
| CCNA1 | -0.274954213 | 0.619797879 | 0.730128901 | down |
| FAM214B | -0.274934649 | 0.153139174 | 0.272759861 | down |
| INPP5K | -0.274872768 | 0.042140959 | 0.108064441 | down |
| PES1 | -0.274742908 | 0.122689066 | 0.233635692 | down |

| | | | | |
|---|---|---|---|---|
| GCC2-AS1 | -0.274669333 | 0.509542411 | 0.635637444 | down |
| TMEM44-AS1 | -0.274662248 | 0.113221697 | 0.220671271 | down |
| LINC00211 | -0.274658844 | 0.694532971 | 0.789442668 | down |
| AGO1 | -0.274637675 | 0.001501017 | 0.008340401 | down |
| AC091633.2 | -0.274614366 | 0.599653885 | 0.712968511 | down |
| TMEM251 | -0.274595622 | 0.12183555 | 0.232526189 | down |
| CWF19L1 | -0.274455286 | 0.084171931 | 0.178640726 | down |
| ASB16-AS1 | -0.274406261 | 0.084385975 | 0.178982862 | down |
| RP11-423H2.3 | -0.274398729 | 0.426446963 | 0.562085309 | down |
| CTC-661I16.2 | -0.274252381 | 0.920037076 | | down |
| RP11-550C4.6 | -0.274250987 | 0.919011636 | | down |
| RNU1-36P | -0.274219759 | 0.898462259 | | down |
| CTC-575C13.4 | -0.274201332 | 0.887999609 | | down |
| RP11-118F2.2 | -0.274155091 | 0.865259772 | | down |
| KCNAB3 | -0.274151612 | 0.320470261 | 0.459295354 | down |
| C20orf96 | -0.274080872 | 0.101121396 | 0.203625417 | down |
| RP11-133K1.7 | -0.274068176 | 0.830812251 | | down |
| OR56B2P | -0.274054045 | 0.704870336 | 0.797006513 | down |
| CTB-83J4.1 | -0.274042209 | 0.821884542 | | down |
| MIR1285-1 | -0.274006802 | 0.810444542 | | down |
| AC074366.3 | -0.273998897 | 0.807992577 | | down |
| RAPGEF2 | -0.2739973 | 0.236725904 | 0.369810371 | down |
| RP11-857B24.1 | -0.273966801 | 0.798375943 | | down |
| U1 | -0.273951453 | 0.793954181 | | down |
| SNF8 | -0.273948332 | 0.137300062 | 0.252698056 | down |
| SLC9A5 | -0.273928596 | 0.312977503 | 0.451659354 | down |
| PPAN | -0.273914126 | 0.275097312 | 0.411379406 | down |
| ZHX1-C8orf76 | -0.273886989 | 0.776453748 | | down |
| AC009403.2 | -0.273883691 | 0.215519542 | 0.346612555 | down |
| KIAA1841 | -0.273849628 | 0.119464419 | 0.229473115 | down |
| RPL3P3 | -0.27381945 | 0.75967206 | | down |
| MOK | -0.273746239 | 0.116325634 | 0.225070024 | down |
| SLIRP | -0.273744731 | 0.08381937 | 0.178095413 | down |
| MIPEP | -0.273674328 | 0.078135275 | 0.169279503 | down |
| ATP5J | -0.273581369 | 0.153469168 | 0.273162719 | down |
| YWHAZP3 | -0.273579598 | 0.687270271 | 0.783588783 | down |
| RPL39P38 | -0.273564294 | 0.520075487 | 0.644548631 | down |
| ZNF345 | -0.273525389 | 0.232587049 | 0.365301129 | down |
| PDSS2 | -0.27344143 | 0.009297798 | 0.034688073 | down |
| AOC2 | -0.273242541 | 0.480305823 | 0.609671221 | down |
| EEF1A1P28 | -0.273175142 | 0.909264704 | | down |
| AC012512.1 | -0.2731736 | 0.807287271 | | down |
| FTL | -0.2731083 | 0.172727518 | 0.297063613 | down |
| NLRX1 | -0.273082871 | 0.017367163 | 0.055763497 | down |
| SLC14A2-AS1 | -0.273077925 | 0.746972231 | 0.828302713 | down |
| THUMPD3-AS1 | -0.272980211 | 0.099534702 | 0.201307344 | down |
| RP11-583F2.2 | -0.272924512 | 0.7775049 | | down |
| AC027612.1 | -0.272788389 | 0.831779963 | | down |
| TMEM176B | -0.272767188 | 0.141386623 | 0.258116284 | down |
| MMS22L | -0.272767018 | 0.251739214 | 0.386570557 | down |
| PELI2 | -0.272641121 | 0.277779835 | 0.413971266 | down |

| | | | | |
|---|---|---|---|---|
| ATP10B | -0.272588148 | 0.436535123 | 0.570998356 | down |
| RP11-707G18.1 | -0.27256579 | 0.479292948 | 0.608719873 | down |
| C9orf153 | -0.272387693 | 0.797895323 | | down |
| RP3-472M2.2 | -0.272368592 | 0.874021717 | 0.919790832 | down |
| EIF4A1P4 | -0.272203846 | 0.562140347 | 0.68122558 | down |
| CTC-559E9.8 | -0.272143247 | 0.466723971 | 0.597922115 | down |
| AGBL5 | -0.272131342 | 0.002392276 | 0.012140802 | down |
| DGKZP1 | -0.271863456 | 0.378969924 | 0.516994709 | down |
| RP11-196G11.2 | -0.271849382 | 0.43629159 | 0.570853781 | down |
| EGLN1 | -0.271825471 | 0.490419314 | 0.618829743 | down |
| SALL2 | -0.271659503 | 0.524124352 | 0.648309607 | down |
| MFSD4A | -0.271480944 | 0.30565946 | 0.443934681 | down |
| MIR3176 | -0.271252983 | 0.673008131 | 0.772369314 | down |
| NA | -0.271240794 | 0.536573761 | 0.658934072 | down |
| RP1-8B1.4 | -0.271236368 | 0.451216567 | 0.584149202 | down |
| AC241377.1 | -0.271108939 | 0.628805115 | 0.737213021 | down |
| PPP1R2P3 | -0.271050124 | 0.562293198 | 0.681355166 | down |
| NA | -0.270964463 | 0.779611457 | | down |
| LGALS7B | -0.270864156 | 0.682232524 | 0.779887145 | down |
| HMGN1P37 | -0.270853535 | 0.752004156 | | down |
| CTD-2270N23.1 | -0.270754215 | 0.375865824 | 0.514097294 | down |
| BMPR1A | -0.270715126 | 0.135546197 | 0.250452027 | down |
| CTD-2233K9.1 | -0.270713095 | 0.524187749 | 0.648361331 | down |
| RNASE1 | -0.270660252 | 0.370303752 | 0.50882259 | down |
| NA | -0.270644536 | 0.531715525 | 0.654934151 | down |
| RP11-240M16.1 | -0.270568952 | 0.690003177 | 0.785899441 | down |
| ZNRF3 | -0.270540492 | 0.40176306 | 0.538527254 | down |
| CYS1 | -0.270535418 | 0.363555215 | 0.502006292 | down |
| MIR4263 | -0.270529094 | 0.655272232 | 0.758037771 | down |
| PTPRZ1 | -0.270488737 | 0.597950865 | 0.71176053 | down |
| RNF7 | -0.270443347 | 0.018364009 | 0.058272949 | down |
| CHTF8 | -0.270433884 | 0.630649105 | 0.738599754 | down |
| B3GALT6 | -0.270426442 | 0.174620512 | 0.299030186 | down |
| SMTN | -0.270420526 | 0.320620547 | 0.459373197 | down |
| RP11-141O11.2 | -0.270410766 | 0.837010715 | 0.893866631 | down |
| ZNF785 | -0.270395536 | 0.087272627 | 0.183460594 | down |
| PLPP4 | -0.270272146 | 0.604693107 | 0.717402246 | down |
| RP11-573D15.9 | -0.27015422 | 0.512016208 | 0.6375058 | down |
| GNL1 | -0.270113475 | 0.146189582 | 0.264213035 | down |
| FAM86DP | -0.270105404 | 0.161634786 | 0.283409544 | down |
| RNF181 | -0.270081277 | 0.175167718 | 0.299664909 | down |
| MSLN | -0.270073919 | 0.552473037 | 0.672415826 | down |
| GNGT2 | -0.269935815 | 0.308945883 | 0.447398532 | down |
| C1orf122 | -0.26990269 | 0.203354139 | 0.332801598 | down |
| AP1AR | -0.269841741 | 0.324347875 | 0.462799561 | down |
| BRF1 | -0.269775504 | 0.040529701 | 0.104946154 | down |
| GAS6-AS1 | -0.269759341 | 0.402867761 | 0.539575563 | down |
| RP11-250B2.4 | -0.269596181 | 0.817084156 | 0.879879737 | down |
| TADA3 | -0.269518013 | 0.015842695 | 0.052036506 | down |
| DUS1L | -0.269503077 | 0.109812698 | 0.216185878 | down |
| STARD3 | -0.269492094 | 0.038262065 | 0.100581458 | down |

| | | | | |
|---|---|---|---|---|
| PYM1 | -0.269443462 | 0.056208033 | 0.133221376 | down |
| RP11-12A2.1 | -0.269392629 | 0.635439485 | 0.742441623 | down |
| COMMD6 | -0.269254936 | 0.129210855 | 0.242188081 | down |
| ITM2C | -0.269247007 | 0.330339378 | 0.468813352 | down |
| RP11-34P13.9 | -0.269187039 | 0.926695574 | | down |
| RP4-736L20.3 | -0.269187039 | 0.926695574 | | down |
| PRAMEF1 | -0.269187039 | 0.926695574 | | down |
| RP11-174G17 | -0.269187039 | 0.926695574 | | down |
| RNU6-135P | -0.269187039 | 0.926695574 | | down |
| NA | -0.269187039 | 0.926695574 | | down |
| GTF2F2P2 | -0.269187039 | 0.926695574 | | down |
| CYP4A27P | -0.269187039 | 0.926695574 | | down |
| NA | -0.269187039 | 0.926695574 | | down |
| NA | -0.269187039 | 0.926695574 | | down |
| RP11-5P4.2 | -0.269187039 | 0.926695574 | | down |
| COX6B1P7 | -0.269187039 | 0.926695574 | | down |
| MTND2P30 | -0.269187039 | 0.926695574 | | down |
| DPYD-AS2 | -0.269187039 | 0.926695574 | | down |
| AMYP1 | -0.269187039 | 0.926695574 | | down |
| GAPDHP64 | -0.269187039 | 0.926695574 | | down |
| RP11-344P13.3 | -0.269187039 | 0.926695574 | | down |
| NUDT4P1 | -0.269187039 | 0.926695574 | | down |
| RP1-140J1.4 | -0.269187039 | 0.926695574 | | down |
| RNA5SP62 | -0.269187039 | 0.926695574 | | down |
| RP11-466F5.4 | -0.269187039 | 0.926695574 | | down |
| RP4-800F24.1 | -0.269187039 | 0.926695574 | | down |
| RP4-612B18.1 | -0.269187039 | 0.926695574 | | down |
| Metazoa_SRP | -0.269187039 | 0.926695574 | | down |
| RP11-293B7.1 | -0.269187039 | 0.926695574 | | down |
| RP11-38J22.2 | -0.269187039 | 0.926695574 | | down |
| RP5-1051D14.2 | -0.269187039 | 0.926695574 | | down |
| AC108488.4 | -0.269187039 | 0.926695574 | | down |
| MIR4765 | -0.269187039 | 0.926695574 | | down |
| SNORD75 | -0.269187039 | 0.926695574 | | down |
| AC017078.1 | -0.269187039 | 0.926695574 | | down |
| U6 | -0.269187039 | 0.926695574 | | down |
| AC097463.2 | -0.269187039 | 0.926695574 | | down |
| RNA5SP95 | -0.269187039 | 0.926695574 | | down |
| AC114752.1 | -0.269187039 | 0.926695574 | | down |
| IGKV1OR2-118 | -0.269187039 | 0.926695574 | | down |
| RNU6-1180P | -0.269187039 | 0.926695574 | | down |
| AC108059.1 | -0.269187039 | 0.926695574 | | down |
| MED15P4 | -0.269187039 | 0.926695574 | | down |
| Y_RNA | -0.269187039 | 0.926695574 | | down |
| UBE2V1P6 | -0.269187039 | 0.926695574 | | down |
| MIR933 | -0.269187039 | 0.926695574 | | down |
| RPL21P32 | -0.269187039 | 0.926695574 | | down |
| RPL23AP36 | -0.269187039 | 0.926695574 | | down |
| Y_RNA | -0.269187039 | 0.926695574 | | down |
| PKP4P1 | -0.269187039 | 0.926695574 | | down |
| RNU6-1027P | -0.269187039 | 0.926695574 | | down |

| | | | |
|---|---|---|---|
| MIR1471 | -0.269187039 | 0.926695574 | down |
| AC144525.1 | -0.269187039 | 0.926695574 | down |
| NA | -0.269187039 | 0.926695574 | down |
| RNF10P1 | -0.269187039 | 0.926695574 | down |
| CYCSP12 | -0.269187039 | 0.926695574 | down |
| CRIP1P1 | -0.269187039 | 0.926695574 | down |
| RN7SL110P | -0.269187039 | 0.926695574 | down |
| RN7SL296P | -0.269187039 | 0.926695574 | down |
| RNU7-73P | -0.269187039 | 0.926695574 | down |
| RNA5SP130 | -0.269187039 | 0.926695574 | down |
| RP11-1008M1.1 | -0.269187039 | 0.926695574 | down |
| MIR6827 | -0.269187039 | 0.926695574 | down |
| RNU6-1284P | -0.269187039 | 0.926695574 | down |
| RP11-88H10.2 | -0.269187039 | 0.926695574 | down |
| RP11-78O22.1 | -0.269187039 | 0.926695574 | down |
| RN7SKP46 | -0.269187039 | 0.926695574 | down |
| TERC | -0.269187039 | 0.926695574 | down |
| ATP13A4-AS1 | -0.269187039 | 0.926695574 | down |
| RP11-747H12.5 | -0.269187039 | 0.926695574 | down |
| AC006296.2 | -0.269187039 | 0.926695574 | down |
| RNU7-11P | -0.269187039 | 0.926695574 | down |
| MTCO3P39 | -0.269187039 | 0.926695574 | down |
| Y_RNA | -0.269187039 | 0.926695574 | down |
| RPS23P3 | -0.269187039 | 0.926695574 | down |
| RP11-468N14.7 | -0.269187039 | 0.926695574 | down |
| RP11-297P16.3 | -0.269187039 | 0.926695574 | down |
| RPL7L1P13 | -0.269187039 | 0.926695574 | down |
| NDUFB2P1 | -0.269187039 | 0.926695574 | down |
| RP11-171N4.1 | -0.269187039 | 0.926695574 | down |
| RP11-234O6.2 | -0.269187039 | 0.926695574 | down |
| RP11-6E9.5 | -0.269187039 | 0.926695574 | down |
| NDUFB5P1 | -0.269187039 | 0.926695574 | down |
| DCAF13P2 | -0.269187039 | 0.926695574 | down |
| NA | -0.269187039 | 0.926695574 | down |
| U3 | -0.269187039 | 0.926695574 | down |
| CTD-2194L12.3 | -0.269187039 | 0.926695574 | down |
| RNU6ATAC36P | -0.269187039 | 0.926695574 | down |
| CTC-498M16.3 | -0.269187039 | 0.926695574 | down |
| AC011366.3 | -0.269187039 | 0.926695574 | down |
| MIR548F3 | -0.269187039 | 0.926695574 | down |
| CTC-505O3.2 | -0.269187039 | 0.926695574 | down |
| HMGB1P22 | -0.269187039 | 0.926695574 | down |
| HNRNPA1P13 | -0.269187039 | 0.926695574 | down |
| RNU6-1148P | -0.269187039 | 0.926695574 | down |
| CH17-140K24.5 | -0.269187039 | 0.926695574 | down |
| RN7SL655P | -0.269187039 | 0.926695574 | down |
| RP11-281O15.5 | -0.269187039 | 0.926695574 | down |
| U4 | -0.269187039 | 0.926695574 | down |
| NA | -0.269187039 | 0.926695574 | down |
| RP3-470L22.1 | -0.269187039 | 0.926695574 | down |
| OR2W2P | -0.269187039 | 0.926695574 | down |

| | | | |
|---|---|---|---|
| RN7SL353P | -0.269187039 | 0.926695574 | down |
| RNU6-754P | -0.269187039 | 0.926695574 | down |
| RP1-152L7.9 | -0.269187039 | 0.926695574 | down |
| RPL26P20 | -0.269187039 | 0.926695574 | down |
| Y_RNA | -0.269187039 | 0.926695574 | down |
| RP1-105O18.1 | -0.269187039 | 0.926695574 | down |
| RP11-282C5.1 | -0.269187039 | 0.926695574 | down |
| RP11-472E5.3 | -0.269187039 | 0.926695574 | down |
| RNU6-293P | -0.269187039 | 0.926695574 | down |
| AC005094.2 | -0.269187039 | 0.926695574 | down |
| TRGVB | -0.269187039 | 0.926695574 | down |
| AC004692.5 | -0.269187039 | 0.926695574 | down |
| NA | -0.269187039 | 0.926695574 | down |
| RP11-760D2.12 | -0.269187039 | 0.926695574 | down |
| MIR4283-2 | -0.269187039 | 0.926695574 | down |
| VN1R35P | -0.269187039 | 0.926695574 | down |
| AC003984.1 | -0.269187039 | 0.926695574 | down |
| RP11-458K10.3 | -0.269187039 | 0.926695574 | down |
| Metazoa_SRP | -0.269187039 | 0.926695574 | down |
| NA | -0.269187039 | 0.926695574 | down |
| DEFB107B | -0.269187039 | 0.926695574 | down |
| RP11-326L2.1 | -0.269187039 | 0.926695574 | down |
| RNU6-1276P | -0.269187039 | 0.926695574 | down |
| RP11-359B20.5 | -0.269187039 | 0.926695574 | down |
| RP11-738G5.2 | -0.269187039 | 0.926695574 | down |
| RNU6-1331P | -0.269187039 | 0.926695574 | down |
| TRAPPC2P2 | -0.269187039 | 0.926695574 | down |
| RP11-267M23.7 | -0.269187039 | 0.926695574 | down |
| Y_RNA | -0.269187039 | 0.926695574 | down |
| RNU4-83P | -0.269187039 | 0.926695574 | down |
| HMGB1P46 | -0.269187039 | 0.926695574 | down |
| RNU6-442P | -0.269187039 | 0.926695574 | down |
| RP11-343P9.1 | -0.269187039 | 0.926695574 | down |
| CYP11B2 | -0.269187039 | 0.926695574 | down |
| IFNWP4 | -0.269187039 | 0.926695574 | down |
| RN7SL151P | -0.269187039 | 0.926695574 | down |
| RP11-49E18.1 | -0.269187039 | 0.926695574 | down |
| NUTF2P3 | -0.269187039 | 0.926695574 | down |
| RP11-540H22.2 | -0.269187039 | 0.926695574 | down |
| RN7SKP128 | -0.269187039 | 0.926695574 | down |
| NA | -0.269187039 | 0.926695574 | down |
| MIR5699 | -0.269187039 | 0.926695574 | down |
| RP11-336A10.7 | -0.269187039 | 0.926695574 | down |
| MTND2P16 | -0.269187039 | 0.926695574 | down |
| AC063961.1 | -0.269187039 | 0.926695574 | down |
| RP11-748L13.6 | -0.269187039 | 0.926695574 | down |
| U3 | -0.269187039 | 0.926695574 | down |
| RN7SL63P | -0.269187039 | 0.926695574 | down |
| ARL6IP1P2 | -0.269187039 | 0.926695574 | down |
| RP11-40C11.2 | -0.269187039 | 0.926695574 | down |
| RP11-443O13.3 | -0.269187039 | 0.926695574 | down |

| | | | |
|---|---|---|---|
| NA | -0.269187039 | 0.926695574 | down |
| RPL7P34 | -0.269187039 | 0.926695574 | down |
| RP11-429G19.3 | -0.269187039 | 0.926695574 | down |
| RP11-108L7.4 | -0.269187039 | 0.926695574 | down |
| BUB1P1 | -0.269187039 | 0.926695574 | down |
| AC068580.7 | -0.269187039 | 0.926695574 | down |
| OR52E5 | -0.269187039 | 0.926695574 | down |
| RP11-494M8.1 | -0.269187039 | 0.926695574 | down |
| RP11-231N3.1 | -0.269187039 | 0.926695574 | down |
| RNA5SP338 | -0.269187039 | 0.926695574 | down |
| RP11-960D24.1 | -0.269187039 | 0.926695574 | down |
| MIR5582 | -0.269187039 | 0.926695574 | down |
| OR4R1P | -0.269187039 | 0.926695574 | down |
| OR5D17P | -0.269187039 | 0.926695574 | down |
| OR5B10P | -0.269187039 | 0.926695574 | down |
| RANP3 | -0.269187039 | 0.926695574 | down |
| MTND6P25 | -0.269187039 | 0.926695574 | down |
| RP11-358N4.3 | -0.269187039 | 0.926695574 | down |
| PGAM1P9 | -0.269187039 | 0.926695574 | down |
| OR10D4P | -0.269187039 | 0.926695574 | down |
| RP11-697E14.2 | -0.269187039 | 0.926695574 | down |
| RNU6-781P | -0.269187039 | 0.926695574 | down |
| Y_RNA | -0.269187039 | 0.926695574 | down |
| RNU6-251P | -0.269187039 | 0.926695574 | down |
| RP11-56H16.1 | -0.269187039 | 0.926695574 | down |
| RN7SKP289 | -0.269187039 | 0.926695574 | down |
| CHCHD3P2 | -0.269187039 | 0.926695574 | down |
| RP11-20L19.1 | -0.269187039 | 0.926695574 | down |
| MIR5700 | -0.269187039 | 0.926695574 | down |
| LSM3P2 | -0.269187039 | 0.926695574 | down |
| RPL30P12 | -0.269187039 | 0.926695574 | down |
| RNA5SP33 | -0.269187039 | 0.926695574 | down |
| RP11-340C20.3 | -0.269187039 | 0.926695574 | down |
| RPL7P45 | -0.269187039 | 0.926695574 | down |
| TRAV32 | -0.269187039 | 0.926695574 | down |
| NKX2-1-AS1 | -0.269187039 | 0.926695574 | down |
| RP11-280K24.1 | -0.269187039 | 0.926695574 | down |
| SCARNA20 | -0.269187039 | 0.926695574 | down |
| RNU6-91P | -0.269187039 | 0.926695574 | down |
| MIR411 | -0.269187039 | 0.926695574 | down |
| RPL23AP11 | -0.269187039 | 0.926695574 | down |
| IGHVII-26-2 | -0.269187039 | 0.926695574 | down |
| RNA5SP389 | -0.269187039 | 0.926695574 | down |
| RP11-467L19.11 | -0.269187039 | 0.926695574 | down |
| RP11-275E15.3 | -0.269187039 | 0.926695574 | down |
| SNORA18 | -0.269187039 | 0.926695574 | down |
| RNU6-1332P | -0.269187039 | 0.926695574 | down |
| CTD-2651B20.8 | -0.269187039 | 0.926695574 | down |
| Y_RNA | -0.269187039 | 0.926695574 | down |
| RN7SL577P | -0.269187039 | 0.926695574 | down |
| RP11-355N15.2 | -0.269187039 | 0.926695574 | down |

| | | | |
|---|---|---|---|
| Metazoa_SRP | -0.269187039 | 0.926695574 | down |
| MIR1276 | -0.269187039 | 0.926695574 | down |
| RP11-133L19.2 | -0.269187039 | 0.926695574 | down |
| RP11-461F11.3 | -0.269187039 | 0.926695574 | down |
| RN7SL850P | -0.269187039 | 0.926695574 | down |
| RP11-483K5.3 | -0.269187039 | 0.926695574 | down |
| RP11-429K17.1 | -0.269187039 | 0.926695574 | down |
| RP11-146F11.3 | -0.269187039 | 0.926695574 | down |
| LINC01566 | -0.269187039 | 0.926695574 | down |
| C2orf69P2 | -0.269187039 | 0.926695574 | down |
| RP11-93O14.1 | -0.269187039 | 0.926695574 | down |
| RP11-169E6.4 | -0.269187039 | 0.926695574 | down |
| LINC00919 | -0.269187039 | 0.926695574 | down |
| NA | -0.269187039 | 0.926695574 | down |
| RNU6-21P | -0.269187039 | 0.926695574 | down |
| RP11-256I9.3 | -0.269187039 | 0.926695574 | down |
| RP11-77K12.7 | -0.269187039 | 0.926695574 | down |
| NA | -0.269187039 | 0.926695574 | down |
| RN7SL105P | -0.269187039 | 0.926695574 | down |
| OR1P1 | -0.269187039 | 0.926695574 | down |
| OR3A4P | -0.269187039 | 0.926695574 | down |
| RP11-219A15.2 | -0.269187039 | 0.926695574 | down |
| RP1-48J14.1 | -0.269187039 | 0.926695574 | down |
| MIR144 | -0.269187039 | 0.926695574 | down |
| RPL35AP35 | -0.269187039 | 0.926695574 | down |
| SNORD63 | -0.269187039 | 0.926695574 | down |
| KRT41P | -0.269187039 | 0.926695574 | down |
| RNU7-134P | -0.269187039 | 0.926695574 | down |
| RN7SKP94 | -0.269187039 | 0.926695574 | down |
| UBE2V2P2 | -0.269187039 | 0.926695574 | down |
| RP11-219G17.8 | -0.269187039 | 0.926695574 | down |
| RN7SKP72 | -0.269187039 | 0.926695574 | down |
| Y_RNA | -0.269187039 | 0.926695574 | down |
| RP11-107K17.2 | -0.269187039 | 0.926695574 | down |
| RP11-46D1.2 | -0.269187039 | 0.926695574 | down |
| Y_RNA | -0.269187039 | 0.926695574 | down |
| RP11-267C16.2 | -0.269187039 | 0.926695574 | down |
| OR7A8P | -0.269187039 | 0.926695574 | down |
| AC078899.3 | -0.269187039 | 0.926695574 | down |
| BNIP3P20 | -0.269187039 | 0.926695574 | down |
| VN1R79P | -0.269187039 | 0.926695574 | down |
| RN7SL402P | -0.269187039 | 0.926695574 | down |
| PSG10P | -0.269187039 | 0.926695574 | down |
| AC138472.4 | -0.269187039 | 0.926695574 | down |
| MIR4531 | -0.269187039 | 0.926695574 | down |
| SIGLEC20P | -0.269187039 | 0.926695574 | down |
| MIR522 | -0.269187039 | 0.926695574 | down |
| AC009892.5 | -0.269187039 | 0.926695574 | down |
| RP11-416N4.1 | -0.269187039 | 0.926695574 | down |
| PGAM3P | -0.269187039 | 0.926695574 | down |
| RNF11P2 | -0.269187039 | 0.926695574 | down |

| | | | | |
|---|---|---|---|---|
| RPL17P1 | -0.269187039 | 0.926695574 | | down |
| RP5-984P4.4 | -0.269187039 | 0.926695574 | | down |
| KRT18P3 | -0.269187039 | 0.926695574 | | down |
| CST9 | -0.269187039 | 0.926695574 | | down |
| HMGB3P2 | -0.269187039 | 0.926695574 | | down |
| Metazoa_SRP | -0.269187039 | 0.926695574 | | down |
| ATP5J2LP | -0.269187039 | 0.926695574 | | down |
| IGKV1OR22-1 | -0.269187039 | 0.926695574 | | down |
| L22NC03-22A12.9 | -0.269187039 | 0.926695574 | | down |
| MIR4764 | -0.269187039 | 0.926695574 | | down |
| RP11-126O22.3 | -0.269187039 | 0.926695574 | | down |
| uc_338 | -0.269187039 | 0.926695574 | | down |
| RP11-1037J10.1 | -0.269187039 | 0.926695574 | | down |
| SSX4 | -0.269187039 | 0.926695574 | | down |
| SPANXN5 | -0.269187039 | 0.926695574 | | down |
| RP13-212L9.1 | -0.269187039 | 0.926695574 | | down |
| Y_RNA | -0.269187039 | 0.926695574 | | down |
| MTND5P26 | -0.269187039 | 0.926695574 | | down |
| SNORD96B | -0.269187039 | 0.926695574 | | down |
| NA | -0.269187039 | 0.926695574 | | down |
| RNU7-69P | -0.269187039 | 0.926695574 | | down |
| RN7SL190P | -0.269187039 | 0.926695574 | | down |
| RP11-265D19.8 | -0.269187039 | 0.926695574 | | down |
| snoU13 | -0.269187039 | 0.926695574 | | down |
| XXyac-YR29IB3.1 | -0.269187039 | 0.926695574 | | down |
| Metazoa_SRP | -0.269187039 | 0.926695574 | | down |
| MAGEA5 | -0.269187039 | 0.926695574 | | down |
| USP9YP4 | -0.269187039 | 0.926695574 | | down |
| RBMY2QP | -0.269187039 | 0.926695574 | | down |
| SLC9B1P1 | -0.269187039 | 0.926695574 | | down |
| MTND1P12 | -0.269187039 | 0.926695574 | | down |
| DPPA2P1 | -0.269187039 | 0.926695574 | | down |
| AC010723.1 | -0.269187039 | 0.926695574 | | down |
| AC010086.5 | -0.269187039 | 0.926695574 | | down |
| USP9YP3 | -0.269187039 | 0.926695574 | | down |
| TSPY22P | -0.269187039 | 0.926695574 | | down |
| NA | -0.269181712 | 0.646377339 | 0.751277062 | down |
| GOLGA8H | -0.269149444 | 0.463986075 | 0.595548239 | down |
| PLA2G12A | -0.26912212 | 0.13264455 | 0.246683556 | down |
| SNORA25 | -0.268994111 | 0.921728055 | | down |
| TRAF2 | -0.268969209 | 0.102918153 | 0.206151943 | down |
| LY86 | -0.26888933 | 0.386415175 | 0.524081686 | down |
| GABRE | -0.268885384 | 0.331366541 | 0.46969128 | down |
| MFAP5 | -0.268881437 | 0.417413406 | 0.553651865 | down |
| bac-BPGBPG55C20 | -0.268877984 | 0.601435458 | 0.71452121 | down |
| RP4-665J23.1 | -0.268848227 | 0.323758448 | 0.462125904 | down |
| CBWD5 | -0.268692054 | 0.159077094 | 0.280229708 | down |
| RP11-379K17.2 | -0.268560409 | 0.754854484 | | down |
| INCA1 | -0.268521387 | 0.238733691 | 0.372153532 | down |
| ANKUB1 | -0.268485663 | 0.84768137 | | down |
| RP11-4B16.4 | -0.268409807 | 0.417842964 | 0.554001501 | down |

| | | | | |
|---|---|---|---|---|
| UBE2J2 | -0.268391704 | 0.0285814 | 0.081055274 | down |
| MCPH1 | -0.268280917 | 0.0194417 | 0.060793757 | down |
| AC009892.10 | -0.268046063 | 0.848424343 | | down |
| NRSN2-AS1 | -0.267942246 | 0.191897231 | 0.319693935 | down |
| RNU6-892P | -0.267872078 | 0.791311553 | | down |
| TRAJ44 | -0.267840011 | 0.859526952 | | down |
| OAS1 | -0.267815305 | 0.265135544 | 0.400751235 | down |
| ZADH2 | -0.267764106 | 0.023804585 | 0.070635551 | down |
| PSD4 | -0.267759318 | 0.144270421 | 0.261756051 | down |
| LRRFIP2 | -0.267576899 | 0.018564965 | 0.05873692 | down |
| PPP3R2 | -0.267336553 | 0.834378148 | | down |
| KMT2E-AS1 | -0.267334909 | 0.313450514 | 0.452081381 | down |
| DNAJC3-AS1 | -0.267258472 | 0.296742475 | 0.434127584 | down |
| AC000036.4 | -0.267087719 | 0.815404248 | | down |
| RP11-115C21.2 | -0.267057488 | 0.102387507 | 0.205428807 | down |
| ZNF683 | -0.2668367 | 0.549622035 | 0.670000991 | down |
| EMBP1 | -0.266812477 | 0.518364109 | 0.643117413 | down |
| ACOT13 | -0.266761038 | 0.192990768 | 0.321035083 | down |
| RPUSD1 | -0.266760865 | 0.227671858 | 0.359857603 | down |
| CCDC93 | -0.26653903 | 0.092268522 | 0.190825048 | down |
| C1orf101 | -0.266348908 | 0.588672337 | 0.703923252 | down |
| CTD-2044J15.1 | -0.266318047 | 0.722220224 | 0.81066967 | down |
| IGLC4 | -0.266247979 | 0.71597195 | 0.805461741 | down |
| LOR | -0.266149065 | 0.586924613 | 0.702448435 | down |
| RP11-126L15.4 | -0.266088023 | 0.450717892 | 0.583654513 | down |
| POMGNT1 | -0.266071381 | 0.058437857 | 0.137141604 | down |
| NDUFB4P5 | -0.266048774 | 0.927547812 | | down |
| MTND2P23 | -0.266048774 | 0.927547812 | | down |
| RP11-223C24.2 | -0.266048774 | 0.927547812 | | down |
| CTD-2154H6.1 | -0.266048774 | 0.927547812 | | down |
| ZNF90P3 | -0.266048774 | 0.927547812 | | down |
| RN7SL379P | -0.266048774 | 0.927547812 | | down |
| NA | -0.266048599 | 0.926145064 | | down |
| RP11-480D4.7 | -0.266047468 | 0.914576945 | | down |
| NA | -0.266047385 | 0.91349233 | | down |
| HIGD1AP16 | -0.266046711 | 0.902884018 | | down |
| CTD-2207P18.2 | -0.266046686 | 0.902424514 | | down |
| RP4-694A7.2 | -0.266046234 | 0.892486552 | | down |
| RP11-212I21.3 | -0.266046204 | 0.891724225 | | down |
| RP11-107M16.2 | -0.266046194 | 0.891452309 | | down |
| C3orf67-AS1 | -0.26604619 | 0.891355972 | | down |
| RP11-65E22.2 | -0.26604618 | 0.89108992 | | down |
| SNORA70 | -0.266045836 | 0.880687826 | | down |
| CACNA1C-IT2 | -0.266045779 | 0.878627967 | | down |
| SNRPGP4 | -0.266045536 | 0.868650599 | | down |
| AKAP17BP | -0.266045103 | 0.842347616 | | down |
| TDGF1P6 | -0.266044956 | 0.828997257 | | down |
| Y_RNA | -0.266044746 | 0.802414575 | | down |
| NA | -0.266036252 | 0.70794315 | 0.799302668 | down |
| C11orf71 | -0.266028112 | 0.090766884 | 0.188628363 | down |
| GAPDHP65 | -0.265999048 | 0.42702168 | 0.562497341 | down |

| | | | | |
|---|---|---|---|---|
| RP11-424C20.2 | -0.265932288 | 0.703756042 | 0.796230036 | down |
| CRIP1 | -0.26592686 | 0.287744041 | 0.425023695 | down |
| ICA1L | -0.265859574 | 0.350133118 | 0.488865108 | down |
| COQ8B | -0.265706423 | 0.0414292 | 0.106676285 | down |
| POLR2E | -0.265599223 | 0.047061575 | 0.116959051 | down |
| ALG1L6P | -0.265572663 | 0.522400326 | 0.646523125 | down |
| NA | -0.265441648 | 0.829451028 | 0.88861172 | down |
| RIC3 | -0.265423985 | 0.429748476 | 0.564999266 | down |
| DDAH2 | -0.265232366 | 0.1961591 | 0.324649321 | down |
| GSTM3 | -0.265141528 | 0.187349479 | 0.313927929 | down |
| ACTRT3 | -0.265064863 | 0.3226935 | 0.461249513 | down |
| LPCAT4 | -0.265008308 | 0.185720334 | 0.311963643 | down |
| PTPA | -0.264958945 | 0.122327026 | 0.23314908 | down |
| NA | -0.264954789 | 0.561558959 | 0.680713228 | down |
| GLI1 | -0.264844302 | 0.15405991 | 0.273773322 | down |
| TRAF3IP2-AS1 | -0.26476842 | 0.173735495 | 0.298269098 | down |
| NA | -0.264683342 | 0.431621017 | 0.566717151 | down |
| ABHD17AP4 | -0.264670821 | 0.6959423 | 0.790596351 | down |
| KIF2C | -0.264594211 | 0.333128149 | 0.47145884 | down |
| ATP5A1 | -0.264572205 | 0.013598304 | 0.046336334 | down |
| ZNF517 | -0.264556591 | 0.053937175 | 0.129194648 | down |
| NA | -0.264515233 | 0.72739388 | 0.814498965 | down |
| CCDC15 | -0.264386247 | 0.378932292 | 0.516990226 | down |
| LAGE3 | -0.264299964 | 0.232743819 | 0.365438143 | down |
| RITA1 | -0.264276516 | 0.094722861 | 0.194508485 | down |
| RP11-477J21.6 | -0.26421726 | 0.800634213 | | down |
| NDUFA6 | -0.263968744 | 0.039565808 | 0.103124415 | down |
| FAM131B | -0.263958735 | 0.512386394 | 0.637913842 | down |
| CORO6 | -0.263871334 | 0.4625882 | 0.594338003 | down |
| CTC-444N24.7 | -0.263750082 | 0.734072534 | | down |
| RTF1 | -0.263677513 | 0.032319604 | 0.08861199 | down |
| VTI1A | -0.263639513 | 0.048060696 | 0.118842432 | down |
| RP11-428K3.1 | -0.263592296 | 0.481260847 | 0.6104708 | down |
| NELL2 | -0.263457191 | 0.510790989 | 0.636613477 | down |
| OR2B11 | -0.263159841 | 0.759159334 | 0.837397035 | down |
| FAM27B | -0.263155898 | 0.424995724 | 0.560543729 | down |
| DUS2 | -0.263128277 | 0.209738051 | 0.340063141 | down |
| UGDH | -0.263115735 | 0.211642069 | 0.342295261 | down |
| RP11-173A8.2 | -0.262941114 | 0.827997096 | | down |
| RP1-170O19.14 | -0.262880686 | 0.649859492 | 0.753978812 | down |
| MRPS18C | -0.262850035 | 0.212740854 | 0.343560552 | down |
| CRYGS | -0.262590301 | 0.352385156 | 0.491142124 | down |
| C9orf152 | -0.262515974 | 0.384436792 | 0.522129152 | down |
| CCDC58P5 | -0.262369692 | 0.892560432 | | down |
| GLB1L3 | -0.26221055 | 0.593626897 | 0.70816471 | down |
| NA | -0.262186219 | 0.589187212 | 0.704482851 | down |
| CTA-246H3.11 | -0.262157649 | 0.705350755 | 0.797334566 | down |
| PPIC | -0.262140613 | 0.211691821 | 0.342325695 | down |
| DDX52 | -0.262037612 | 0.043781562 | 0.111082574 | down |
| RPS12 | -0.261998961 | 0.133231998 | 0.247347349 | down |
| PTGR2 | -0.261987908 | 0.375633944 | 0.513913762 | down |

| | | | | |
|---|---|---|---|---|
| DNAJC9-AS1 | -0.261934977 | 0.377564086 | 0.515687622 | down |
| SIRT3 | -0.261897442 | 0.048128007 | 0.118985729 | down |
| RP11-345K20.2 | -0.261842277 | 0.424997524 | 0.560543729 | down |
| ZNF528-AS1 | -0.261807707 | 0.159480733 | 0.280663813 | down |
| SNORD88A | -0.261784698 | 0.693252937 | 0.78831578 | down |
| SFXN2 | -0.261673518 | 0.062486347 | 0.144000905 | down |
| SMDT1 | -0.261623417 | 0.115801853 | 0.224345555 | down |
| TBC1D8B | -0.26161579 | 0.347448546 | 0.486156115 | down |
| ZNF823 | -0.261589907 | 0.261101636 | 0.396288838 | down |
| HTT | -0.261330693 | 0.004116624 | 0.018665839 | down |
| IMPDH2 | -0.261283582 | 0.096671142 | 0.197349173 | down |
| LINC00398 | -0.261269345 | 0.771180582 | | down |
| DDIT3 | -0.261238856 | 0.150810084 | 0.270036246 | down |
| PUDP | -0.261063004 | 0.162222696 | 0.284163932 | down |
| CDKL3 | -0.261035555 | 0.49092597 | 0.619306741 | down |
| RP11-410E4.1 | -0.261002512 | 0.659834564 | 0.761786856 | down |
| GPD2 | -0.260937402 | 0.112406477 | 0.21956571 | down |
| RP11-783K16.13 | -0.260912302 | 0.687033053 | 0.783574486 | down |
| AC009970.1 | -0.260869111 | 0.759190552 | | down |
| CLPTM1 | -0.260850128 | 0.051975659 | 0.12565838 | down |
| AC018643.4 | -0.260827706 | 0.877681415 | 0.921821598 | down |
| CCDC152 | -0.260808022 | 0.423170366 | 0.558771612 | down |
| RAB27B | -0.260805939 | 0.360139805 | 0.498435614 | down |
| GGT7 | -0.260696845 | 0.098882598 | 0.200433731 | down |
| RP11-383C6.2 | -0.260678934 | 0.775482824 | 0.84961893 | down |
| NA | -0.260669059 | 0.484174334 | 0.613156874 | down |
| PP2D1 | -0.26052519 | 0.605083274 | 0.717702963 | down |
| NA | -0.260471712 | 0.546786627 | 0.667775299 | down |
| ARRDC3-AS1 | -0.26038184 | 0.436519421 | 0.570998356 | down |
| RP5-1172A22.1 | -0.260137115 | 0.548968222 | 0.669502791 | down |
| KLHL22 | -0.260110002 | 0.17206432 | 0.296297383 | down |
| CERK | -0.260089837 | 0.062125377 | 0.143444115 | down |
| CTC-332L22.1 | -0.26004054 | 0.501759981 | 0.628774843 | down |
| DAZAP1 | -0.260030654 | 0.082707296 | 0.176573604 | down |
| RP11-1105G2.3 | -0.259949963 | 0.443008829 | 0.576554354 | down |
| RP5-934G17.6 | -0.259777989 | 0.929250978 | | down |
| RP5-893G23.1 | -0.259777989 | 0.929250978 | | down |
| RNFT1P2 | -0.259777989 | 0.929250978 | | down |
| RNU7-70P | -0.259777989 | 0.929250978 | | down |
| RN7SL420P | -0.259777989 | 0.929250978 | | down |
| LINC01527 | -0.259777989 | 0.929250978 | | down |
| VHLL | -0.259777989 | 0.929250978 | | down |
| RN7SL861P | -0.259777989 | 0.929250978 | | down |
| RNU6-704P | -0.259777989 | 0.929250978 | | down |
| RP11-385M4.3 | -0.259777989 | 0.929250978 | | down |
| LINC00210 | -0.259777989 | 0.929250978 | | down |
| MIR3126 | -0.259777989 | 0.929250978 | | down |
| ATP6V1B1-AS1 | -0.259777989 | 0.929250978 | | down |
| ACTP1 | -0.259777989 | 0.929250978 | | down |
| MED15P9 | -0.259777989 | 0.929250978 | | down |
| AC097721.1 | -0.259777989 | 0.929250978 | | down |

| | | | |
|---|---|---|---|
| AC078882.1 | -0.259777989 | 0.929250978 | down |
| UBQLN4P2 | -0.259777989 | 0.929250978 | down |
| ALDH7A1P2 | -0.259777989 | 0.929250978 | down |
| AC017048.1 | -0.259777989 | 0.929250978 | down |
| AC019185.2 | -0.259777989 | 0.929250978 | down |
| RP11-70C1.3 | -0.259777989 | 0.929250978 | down |
| RP11-719N22.2 | -0.259777989 | 0.929250978 | down |
| PRICKLE2-AS2 | -0.259777989 | 0.929250978 | down |
| RP11-227H4.5 | -0.259777989 | 0.929250978 | down |
| GMFBP1 | -0.259777989 | 0.929250978 | down |
| RP11-789L4.1 | -0.259777989 | 0.929250978 | down |
| RP11-451G4.3 | -0.259777989 | 0.929250978 | down |
| RP11-262M14.2 | -0.259777989 | 0.929250978 | down |
| RNU6-998P | -0.259777989 | 0.929250978 | down |
| HNRNPA1P67 | -0.259777989 | 0.929250978 | down |
| RP11-431J17.1 | -0.259777989 | 0.929250978 | down |
| ATP5LP4 | -0.259777989 | 0.929250978 | down |
| RP11-796L2.1 | -0.259777989 | 0.929250978 | down |
| RP11-45H22.1 | -0.259777989 | 0.929250978 | down |
| Metazoa_SRP | -0.259777989 | 0.929250978 | down |
| SETP22 | -0.259777989 | 0.929250978 | down |
| RN7SKP57 | -0.259777989 | 0.929250978 | down |
| GPR151 | -0.259777989 | 0.929250978 | down |
| KRT18P41 | -0.259777989 | 0.929250978 | down |
| Y_RNA | -0.259777989 | 0.929250978 | down |
| SNORA73 | -0.259777989 | 0.929250978 | down |
| RP11-346C16.4 | -0.259777989 | 0.929250978 | down |
| U8 | -0.259777989 | 0.929250978 | down |
| RP5-894D12.4 | -0.259777989 | 0.929250978 | down |
| CTD-2526L21.3 | -0.259777989 | 0.929250978 | down |
| CTA-313A17.5 | -0.259777989 | 0.929250978 | down |
| RP11-401L13.7 | -0.259777989 | 0.929250978 | down |
| MIR3907 | -0.259777989 | 0.929250978 | down |
| MAPK6PS4 | -0.259777989 | 0.929250978 | down |
| NA | -0.259777989 | 0.929250978 | down |
| RP11-659E9.4 | -0.259777989 | 0.929250978 | down |
| KB-1930G5.4 | -0.259777989 | 0.929250978 | down |
| FAM74A1 | -0.259777989 | 0.929250978 | down |
| NA | -0.259777989 | 0.929250978 | down |
| TMEM161BP1 | -0.259777989 | 0.929250978 | down |
| GAPDHP21 | -0.259777989 | 0.929250978 | down |
| RN7SKP158 | -0.259777989 | 0.929250978 | down |
| RP1-65P5.5 | -0.259777989 | 0.929250978 | down |
| RP11-313M3.1 | -0.259777989 | 0.929250978 | down |
| RNU6-126P | -0.259777989 | 0.929250978 | down |
| MTCYBP41 | -0.259777989 | 0.929250978 | down |
| RP11-684N3.1 | -0.259777989 | 0.929250978 | down |
| RP11-6K23.1 | -0.259777989 | 0.929250978 | down |
| RP11-71J4.2 | -0.259777989 | 0.929250978 | down |
| RNA5SP370 | -0.259777989 | 0.929250978 | down |
| MIR3657 | -0.259777989 | 0.929250978 | down |

| | | | | |
|---|---|---|---|---|
| RP11-386I8.5 | -0.259777989 | 0.929250978 | | down |
| TRAJ61 | -0.259777989 | 0.929250978 | | down |
| BTF3P2 | -0.259777989 | 0.929250978 | | down |
| Y_RNA | -0.259777989 | 0.929250978 | | down |
| CTD-2207P18.1 | -0.259777989 | 0.929250978 | | down |
| RP11-697E2.10 | -0.259777989 | 0.929250978 | | down |
| RP11-697E2.11 | -0.259777989 | 0.929250978 | | down |
| Y_RNA | -0.259777989 | 0.929250978 | | down |
| RP11-72I8.2 | -0.259777989 | 0.929250978 | | down |
| SLX1B-SULT1A4 | -0.259777989 | 0.929250978 | | down |
| RPL10P14 | -0.259777989 | 0.929250978 | | down |
| RP11-44F14.4 | -0.259777989 | 0.929250978 | | down |
| RP11-744D14.1 | -0.259777989 | 0.929250978 | | down |
| TUFMP1 | -0.259777989 | 0.929250978 | | down |
| RNU6-233P | -0.259777989 | 0.929250978 | | down |
| NA | -0.259777989 | 0.929250978 | | down |
| MIR5089 | -0.259777989 | 0.929250978 | | down |
| CTD-2124B20.3 | -0.259777989 | 0.929250978 | | down |
| SNORA73 | -0.259777989 | 0.929250978 | | down |
| RNY4P37 | -0.259777989 | 0.929250978 | | down |
| MIR1539 | -0.259777989 | 0.929250978 | | down |
| RP11-93B14.4 | -0.259777989 | 0.929250978 | | down |
| KRTAP6-3 | -0.259777989 | 0.929250978 | | down |
| SOCS2P2 | -0.259777989 | 0.929250978 | | down |
| L22NC03-102D1.1 | -0.259777989 | 0.929250978 | | down |
| SPECC1L-ADORA2A | -0.259777989 | 0.929250978 | | down |
| SNORA7 | -0.259777989 | 0.929250978 | | down |
| RPS11P7 | -0.259777989 | 0.929250978 | | down |
| SPACA5B | -0.259777989 | 0.929250978 | | down |
| RP11-325E14.2 | -0.259777989 | 0.929250978 | | down |
| RP11-1007I13.4 | -0.259777989 | 0.929250978 | | down |
| TTTY7 | -0.259777989 | 0.929250978 | | down |
| OFD1P16Y | -0.259777989 | 0.929250978 | | down |
| NA | -0.259742888 | 0.681012361 | 0.778996207 | down |
| SIGLEC17P | -0.259713791 | 0.386413514 | 0.524081686 | down |
| FAM86B1 | -0.259632424 | 0.36638161 | 0.5049112 | down |
| WDPCP | -0.259238953 | 0.265898128 | 0.401600813 | down |
| LINC00996 | -0.258918064 | 0.255858727 | 0.39089853 | down |
| ZDHHC24 | -0.258667123 | 0.222024416 | 0.353853989 | down |
| NDUFS4 | -0.258627021 | 0.029561124 | 0.082964448 | down |
| TET3 | -0.258617791 | 0.243312276 | 0.377391535 | down |
| DUX4L27 | -0.258575886 | 0.680825963 | 0.778871953 | down |
| GRIN2D | -0.258552679 | 0.481766252 | 0.610979484 | down |
| RP11-434H6.7 | -0.258423705 | 0.392070217 | 0.529575939 | down |
| BECN1 | -0.258411727 | 0.289405552 | 0.426659817 | down |
| MSH3 | -0.258408722 | 0.051063049 | 0.124211662 | down |
| ZBTB45 | -0.258340861 | 0.051831313 | 0.125450735 | down |
| NA | -0.258298606 | 0.623886733 | 0.733434634 | down |
| RAD51 | -0.258179459 | 0.20932236 | 0.339554073 | down |
| AC062017.1 | -0.258136994 | 0.286297051 | 0.423365093 | down |
| 3C7-42404400C24. | -0.258122778 | 0.5946987 | 0.708852155 | down |

| | | | | |
|---|---|---|---|---|
| TMTC4 | -0.258033155 | 0.159726611 | 0.280928832 | down |
| ADIPOR2 | -0.257969749 | 0.089591388 | 0.186961426 | down |
| TSNAX | -0.257938885 | 0.044687484 | 0.112661808 | down |
| RNA5SP105 | -0.257919463 | 0.894453622 | | down |
| ZNF487 | -0.257864945 | 0.051950209 | 0.125627179 | down |
| ATG2A | -0.25782399 | 0.098196922 | 0.199420598 | down |
| RP11-409K20.6 | -0.257750462 | 0.845393974 | | down |
| THNSL1 | -0.257450195 | 0.203083486 | 0.332534238 | down |
| GPHN | -0.257437417 | 0.243996139 | 0.377932818 | down |
| AANAT | -0.257432817 | 0.547980839 | 0.668805374 | down |
| COL13A1 | -0.257403741 | 0.292520376 | 0.429838706 | down |
| RSF1 | -0.257264419 | 0.089625615 | 0.187006873 | down |
| PRKXP1 | -0.257204899 | 0.656874389 | 0.759218825 | down |
| RP11-298I3.1 | -0.256969179 | 0.411882282 | 0.548616389 | down |
| CCSER1 | -0.256942042 | 0.490794487 | 0.619191633 | down |
| RPS27AP11 | -0.25685515 | 0.483534018 | 0.612590877 | down |
| RP11-293M10.6 | -0.256835638 | 0.3567323 | 0.495225266 | down |
| FXYD7 | -0.256796665 | 0.509493055 | 0.635631714 | down |
| THAP4 | -0.256771257 | 0.113982597 | 0.221763389 | down |
| RP11-679C8.2 | -0.256759287 | 0.61510187 | 0.726134405 | down |
| B9D2 | -0.256678317 | 0.234998342 | 0.367761756 | down |
| NPTN-IT1 | -0.256672122 | 0.670687011 | 0.770382343 | down |
| RP11-661A12.5 | -0.256645506 | 0.714326642 | 0.804002159 | down |
| ELOVL6 | -0.256644705 | 0.270937451 | 0.40698189 | down |
| RP3-331H24.4 | -0.256639725 | 0.930103463 | | down |
| RP11-616M22.9 | -0.256639725 | 0.930103463 | | down |
| CLIC4P2 | -0.256639725 | 0.930103463 | | down |
| NF1P6 | -0.256639725 | 0.930103463 | | down |
| IGHV3-33-2 | -0.256639448 | 0.927844861 | | down |
| RPS17P13 | -0.256639349 | 0.926976745 | | down |
| RP11-390P2.2 | -0.256639277 | 0.926332389 | | down |
| RP11-159H22.1 | -0.256639231 | 0.925904328 | | down |
| CASC6 | -0.256638411 | 0.9168812 | | down |
| NA | -0.256637732 | 0.906230284 | | down |
| CTD-3037G24.3 | -0.256637731 | 0.9062084 | | down |
| SNORA67 | -0.256637259 | 0.895807322 | | down |
| RP11-16K12.2 | -0.256637258 | 0.895781238 | | down |
| RP11-523O18.1 | -0.256637254 | 0.895695981 | | down |
| MIR3613 | -0.256637251 | 0.895618705 | | down |
| CMB9-55A18.1 | -0.256636388 | 0.861812838 | | down |
| AC128709.4 | -0.256636341 | 0.858873661 | | down |
| RPS24P7 | -0.256636193 | 0.848299816 | | down |
| LL22NC03-32F9.1 | -0.256636189 | 0.84799674 | | down |
| RPL23AP37 | -0.256635778 | 0.798115333 | | down |
| AC141928.1 | -0.256627209 | 0.60381021 | 0.716622237 | down |
| RP13-554M15.8 | -0.256568923 | 0.589391099 | 0.704614468 | down |
| HENMT1 | -0.256561087 | 0.038300106 | 0.10063612 | down |
| DOCK9-AS2 | -0.256523342 | 0.398916429 | 0.535836062 | down |
| AP001628.6 | -0.256485578 | 0.574784639 | 0.691804731 | down |
| EDA2R | -0.256482452 | 0.563131167 | 0.682030224 | down |
| RP4-555D20.1 | -0.256462568 | 0.704301671 | 0.796508296 | down |

| | | | | |
|---|---|---|---|---|
| AP003068.18 | -0.256417219 | 0.401395318 | 0.538251325 | down |
| RP5-1112D6.8 | -0.256250001 | 0.49821368 | 0.625504881 | down |
| TMEM51 | -0.256113195 | 0.217050806 | 0.348329385 | down |
| MSMO1 | -0.256109976 | 0.149879237 | 0.268802042 | down |
| C6orf141 | -0.255949973 | 0.40963783 | 0.546377757 | down |
| MYO9B | -0.255860208 | 0.091129074 | 0.18916587 | down |
| ATAD3B | -0.255841135 | 0.260055465 | 0.395150778 | down |
| ELP5 | -0.25573913 | 0.119970245 | 0.230121053 | down |
| DMXL2 | -0.255699338 | 0.179262029 | 0.304451578 | down |
| SERPINB2 | -0.255676936 | 0.778833266 | 0.851687655 | down |
| ZMYM1 | -0.255489511 | 0.231160337 | 0.363687744 | down |
| IZUMO4 | -0.255479077 | 0.427402814 | 0.562875998 | down |
| ELF4 | -0.255425931 | 0.068972863 | 0.154654877 | down |
| SLC31A2 | -0.255325992 | 0.682304782 | 0.779905628 | down |
| RP11-814H16.2 | -0.25530506 | 0.775174438 | | down |
| PCNPP5 | -0.255177248 | 0.747328803 | 0.828606361 | down |
| PRORSD1P | -0.255122504 | 0.282638539 | 0.41912866 | down |
| MEFV | -0.255026367 | 0.486184802 | 0.614951214 | down |
| NDUFA8 | -0.255007874 | 0.098998246 | 0.200641076 | down |
| TMC7 | -0.25498749 | 0.399221967 | 0.536174555 | down |
| Xbac-BPG181B23. | -0.254907936 | 0.229596634 | 0.361909825 | down |
| CFL1 | -0.254894085 | 0.105669674 | 0.210238961 | down |
| AATF | -0.254744883 | 0.073813549 | 0.162489321 | down |
| OR2B6 | -0.254661475 | 0.715283662 | 0.804835793 | down |
| SLC25A45 | -0.254627664 | 0.178970001 | 0.304075933 | down |
| TPK1 | -0.254607397 | 0.210734436 | 0.341333068 | down |
| TMEM68 | -0.254515592 | 0.132537083 | 0.24653079 | down |
| RAB9B | -0.254302697 | 0.458917086 | 0.590884758 | down |
| RP11-456H18.2 | -0.254297012 | 0.69343277 | 0.78843075 | down |
| SPRYD4 | -0.254238313 | 0.08259341 | 0.176388712 | down |
| TRAV35 | -0.254235012 | 0.665263452 | 0.766056309 | down |
| RP11-146F11.1 | -0.254177062 | 0.413298535 | 0.549890434 | down |
| AF129075.5 | -0.254146479 | 0.537490282 | 0.659628203 | down |
| NA | -0.254086921 | 0.840736517 | | down |
| RP11-276E15.4 | -0.253881034 | 0.671880116 | 0.771389616 | down |
| TMEM97 | -0.25386779 | 0.172062293 | 0.296297383 | down |
| RP11-307L14.1 | -0.253832482 | 0.500370771 | 0.627478899 | down |
| PPM1H | -0.253769294 | 0.298934771 | 0.43646772 | down |
| RP11-20J15.3 | -0.253761991 | 0.6689378 | 0.769033525 | down |
| NR2F1-AS1 | -0.253693418 | 0.465403733 | 0.596883366 | down |
| ARHGEF9 | -0.253628707 | 0.06853881 | 0.153947782 | down |
| CSPP1 | -0.253456774 | 0.193044811 | 0.321053913 | down |
| LINC00298 | -0.253414717 | 0.784155237 | | down |
| GTF2IP14 | -0.25332838 | 0.661610979 | 0.763196017 | down |
| TSGA10 | -0.253314617 | 0.230279407 | 0.362679545 | down |
| SIT1 | -0.253268008 | 0.485006711 | 0.613822891 | down |
| NA | -0.253250609 | 0.594940592 | 0.709076291 | down |
| RP3-442L6.4 | -0.253232471 | 0.86139675 | | down |
| FOXD3-AS1 | -0.253131246 | 0.507420754 | 0.633674834 | down |
| RP11-651P23.5 | -0.252916929 | 0.775914122 | | down |
| MRPL38 | -0.252757873 | 0.429352687 | 0.564651876 | down |

| | | | | |
|---|---|---|---|---|
| LINC01330 | -0.252591854 | 0.6662907 | 0.766827727 | down |
| HAGHL | -0.252549658 | 0.355246168 | 0.493823896 | down |
| RP11-109J4.1 | -0.25254341 | 0.717115992 | 0.806356258 | down |
| USP20 | -0.252542529 | 0.131349881 | 0.245079855 | down |
| PRAMENP | -0.252451791 | 0.649279107 | 0.753454674 | down |
| RP11-7D5.2 | -0.252406063 | 0.730596223 | | down |
| ZFPM1 | -0.252392931 | 0.140285168 | 0.256713826 | down |
| OSCP1 | -0.252357205 | 0.267083309 | 0.402777202 | down |
| LRGUK | -0.252276372 | 0.573072261 | 0.690305744 | down |
| NEFH | -0.252017154 | 0.416465033 | 0.55265993 | down |
| COG5 | -0.252015426 | 0.244154934 | 0.378073953 | down |
| GPR108 | -0.252011623 | 0.131187551 | 0.244853003 | down |
| FOXO4 | -0.251951106 | 0.236499558 | 0.369533634 | down |
| DOCK6 | -0.251895183 | 0.132666975 | 0.24670781 | down |
| SLC25A33 | -0.251753809 | 0.045276104 | 0.113706869 | down |
| RP11-796E2.4 | -0.251575206 | 0.533123044 | 0.656103504 | down |
| BCAS4 | -0.251525967 | 0.489547929 | 0.618086147 | down |
| CTB-33G10.1 | -0.251297407 | 0.304528977 | 0.442613731 | down |
| TCAP | -0.251273959 | 0.476611608 | 0.606391088 | down |
| AC105399.2 | -0.251270415 | 0.884777552 | | down |
| GIGYF1 | -0.251145516 | 0.094543697 | 0.194307657 | down |
| UBL4A | -0.250998504 | 0.014064347 | 0.04753185 | down |
| USP40 | -0.250957831 | 0.003114481 | 0.014923681 | down |
| HNRNPUP1 | -0.250934659 | 0.588437324 | 0.703698244 | down |
| CTB-133G6.1 | -0.25083789 | 0.541824435 | 0.663310795 | down |
| NFIB | -0.250836495 | 0.153813671 | 0.27349103 | down |
| LRFN4 | -0.250784484 | 0.348857314 | 0.487579666 | down |
| SLC38A6 | -0.250605639 | 0.14692268 | 0.265209385 | down |
| TMEM87B | -0.250555387 | 0.148445087 | 0.267138916 | down |
| STAU2 | -0.25046895 | 0.111244327 | 0.218160872 | down |
| RP11-293P20.4 | -0.250368939 | 0.931807112 | | down |
| RP5-864K19.6 | -0.250368939 | 0.931807112 | | down |
| RN7SL62P | -0.250368939 | 0.931807112 | | down |
| RP4-784A16.2 | -0.250368939 | 0.931807112 | | down |
| NA | -0.250368939 | 0.931807112 | | down |
| RP11-25L19.1 | -0.250368939 | 0.931807112 | | down |
| CYCSP51 | -0.250368939 | 0.931807112 | | down |
| OR6K3 | -0.250368939 | 0.931807112 | | down |
| Metazoa_SRP | -0.250368939 | 0.931807112 | | down |
| LINC01347 | -0.250368939 | 0.931807112 | | down |
| MIR1301 | -0.250368939 | 0.931807112 | | down |
| RNU6-647P | -0.250368939 | 0.931807112 | | down |
| U51244.2 | -0.250368939 | 0.931807112 | | down |
| RP11-191H23.1 | -0.250368939 | 0.931807112 | | down |
| AC103563.5 | -0.250368939 | 0.931807112 | | down |
| AC103563.7 | -0.250368939 | 0.931807112 | | down |
| RNA5SP102 | -0.250368939 | 0.931807112 | | down |
| RNA5SP107 | -0.250368939 | 0.931807112 | | down |
| AC073465.3 | -0.250368939 | 0.931807112 | | down |
| RP11-369J9.1 | -0.250368939 | 0.931807112 | | down |
| OR6B3 | -0.250368939 | 0.931807112 | | down |

| | | | |
|---|---|---|---|
| AC034187.2 | -0.250368939 | 0.931807112 | down |
| AC115283.1 | -0.250368939 | 0.931807112 | down |
| NA | -0.250368939 | 0.931807112 | down |
| RNA5SP133 | -0.250368939 | 0.931807112 | down |
| PPATP1 | -0.250368939 | 0.931807112 | down |
| OR5H4P | -0.250368939 | 0.931807112 | down |
| RP11-88I21.2 | -0.250368939 | 0.931807112 | down |
| AC006427.2 | -0.250368939 | 0.931807112 | down |
| NA | -0.250368939 | 0.931807112 | down |
| LYPLA1P2 | -0.250368939 | 0.931807112 | down |
| RP11-335L23.4 | -0.250368939 | 0.931807112 | down |
| RP11-321E2.11 | -0.250368939 | 0.931807112 | down |
| CTD-2265D6.2 | -0.250368939 | 0.931807112 | down |
| CTD-2176I21.2 | -0.250368939 | 0.931807112 | down |
| RNU6-390P | -0.250368939 | 0.931807112 | down |
| MIR5003 | -0.250368939 | 0.931807112 | down |
| RP1-15D7.1 | -0.250368939 | 0.931807112 | down |
| OR2W6P | -0.250368939 | 0.931807112 | down |
| GPR53P | -0.250368939 | 0.931807112 | down |
| RN7SL748P | -0.250368939 | 0.931807112 | down |
| RP11-570K4.1 | -0.250368939 | 0.931807112 | down |
| LYPLA1P3 | -0.250368939 | 0.931807112 | down |
| AC060834.2 | -0.250368939 | 0.931807112 | down |
| AC098592.7 | -0.250368939 | 0.931807112 | down |
| MIR1183 | -0.250368939 | 0.931807112 | down |
| VN1R28P | -0.250368939 | 0.931807112 | down |
| MTCO1P8 | -0.250368939 | 0.931807112 | down |
| RP11-561N12.1 | -0.250368939 | 0.931807112 | down |
| RP11-746P2.5 | -0.250368939 | 0.931807112 | down |
| DDX43P3 | -0.250368939 | 0.931807112 | down |
| RP11-13M3.1 | -0.250368939 | 0.931807112 | down |
| RP5-1121A15.3 | -0.250368939 | 0.931807112 | down |
| DEFA7P | -0.250368939 | 0.931807112 | down |
| RP11-597M17.3 | -0.250368939 | 0.931807112 | down |
| NA | -0.250368939 | 0.931807112 | down |
| NA | -0.250368939 | 0.931807112 | down |
| RNU6ATAC8P | -0.250368939 | 0.931807112 | down |
| KB-1433G4.1 | -0.250368939 | 0.931807112 | down |
| LINC00583 | -0.250368939 | 0.931807112 | down |
| RP11-281A20.1 | -0.250368939 | 0.931807112 | down |
| MIR4672 | -0.250368939 | 0.931807112 | down |
| RN7SKP151 | -0.250368939 | 0.931807112 | down |
| WIZP1 | -0.250368939 | 0.931807112 | down |
| NA | -0.250368939 | 0.931807112 | down |
| CTD-2530H12.2 | -0.250368939 | 0.931807112 | down |
| NA | -0.250368939 | 0.931807112 | down |
| RP11-727A23.7 | -0.250368939 | 0.931807112 | down |
| RP11-136I14.2 | -0.250368939 | 0.931807112 | down |
| RP11-993B23.3 | -0.250368939 | 0.931807112 | down |
| HOXC12 | -0.250368939 | 0.931807112 | down |
| RP11-110L15.2 | -0.250368939 | 0.931807112 | down |

| | | | | |
|---|---|---|---|---|
| RP13-895J2.10 | -0.250368939 | 0.931807112 | | down |
| RP11-144H23.2 | -0.250368939 | 0.931807112 | | down |
| NA | -0.250368939 | 0.931807112 | | down |
| HSPD1P9 | -0.250368939 | 0.931807112 | | down |
| TRDD3 | -0.250368939 | 0.931807112 | | down |
| TRAJ9 | -0.250368939 | 0.931807112 | | down |
| CTD-2540L5.9 | -0.250368939 | 0.931807112 | | down |
| Y_RNA | -0.250368939 | 0.931807112 | | down |
| AB019438.66 | -0.250368939 | 0.931807112 | | down |
| DNM1P28 | -0.250368939 | 0.931807112 | | down |
| RP11-108K3.1 | -0.250368939 | 0.931807112 | | down |
| RP11-621H8.2 | -0.250368939 | 0.931807112 | | down |
| RP11-342A23.1 | -0.250368939 | 0.931807112 | | down |
| Y_RNA | -0.250368939 | 0.931807112 | | down |
| CTD-2349B8.1 | -0.250368939 | 0.931807112 | | down |
| NA | -0.250368939 | 0.931807112 | | down |
| RBM22P12 | -0.250368939 | 0.931807112 | | down |
| RP11-26L20.5 | -0.250368939 | 0.931807112 | | down |
| RP11-328J14.1 | -0.250368939 | 0.931807112 | | down |
| AC005358.3 | -0.250368939 | 0.931807112 | | down |
| RNA5SP437 | -0.250368939 | 0.931807112 | | down |
| TBC1D3I | -0.250368939 | 0.931807112 | | down |
| RNU6-1158P | -0.250368939 | 0.931807112 | | down |
| OR10B1P | -0.250368939 | 0.931807112 | | down |
| BNIP3P18 | -0.250368939 | 0.931807112 | | down |
| AC025811.3 | -0.250368939 | 0.931807112 | | down |
| NA | -0.250368939 | 0.931807112 | | down |
| RP5-955M13.4 | -0.250368939 | 0.931807112 | | down |
| CH507-42P11.1 | -0.250368939 | 0.931807112 | | down |
| LINC00319 | -0.250368939 | 0.931807112 | | down |
| RP1-225L15.1 | -0.250368939 | 0.931807112 | | down |
| AP000357.4 | -0.250368939 | 0.931807112 | | down |
| RP11-126O22.2 | -0.250368939 | 0.931807112 | | down |
| MAGEB4 | -0.250368939 | 0.931807112 | | down |
| RNU6-29P | -0.250368939 | 0.931807112 | | down |
| CNOT7P1 | -0.250368939 | 0.931807112 | | down |
| HSPA8P20 | -0.250368939 | 0.931807112 | | down |
| RP13-210D15.1 | -0.250368939 | 0.931807112 | | down |
| XGY1 | -0.250368939 | 0.931807112 | | down |
| RNU6-890P | -0.250368423 | 0.928820189 | | down |
| AC017006.3 | -0.250368399 | 0.928675999 | | down |
| RP11-508N12.2 | -0.250368298 | 0.928041587 | | down |
| RN7SL265P | -0.250368297 | 0.928034613 | | down |
| CTC-455F18.3 | -0.250368264 | 0.927822688 | | down |
| NA | -0.250368126 | 0.926915017 | | down |
| MDN1 | -0.250270404 | 0.217864305 | 0.349243153 | down |
| BRCA2 | -0.250175111 | 0.261925836 | 0.397038336 | down |
| RP11-159N11.4 | -0.249993182 | 0.438642559 | 0.572850575 | down |
| FH | -0.249972926 | 0.120271934 | 0.230523124 | down |
| HS6ST2 | -0.249902538 | 0.603108901 | 0.715914809 | down |
| AC009303.2 | -0.249808204 | 0.430313777 | 0.565445553 | down |

| | | | | |
|---|---|---|---|---|
| RPS11 | -0.249773321 | 0.180082297 | 0.305326892 | down |
| TUSC5 | -0.249718945 | 0.676130941 | 0.775124127 | down |
| DPH6 | -0.249709158 | 0.261380124 | 0.396511207 | down |
| NA | -0.249643455 | 0.724537204 | 0.812152254 | down |
| CD1D | -0.24962941 | 0.357156609 | 0.495561303 | down |
| NA | -0.249542822 | 0.696792628 | 0.791023529 | down |
| SMG1P2 | -0.249463307 | 0.128652897 | 0.241517599 | down |
| TEX22 | -0.249427557 | 0.494224448 | 0.62226466 | down |
| LINC01561 | -0.249422133 | 0.787724441 | | down |
| TSEN2 | -0.249296043 | 0.136490543 | 0.251732466 | down |
| LCORL | -0.249103498 | 0.395612167 | 0.53285089 | down |
| SERTAD3 | -0.249009487 | 0.097257816 | 0.198129498 | down |
| NFASC | -0.248931251 | 0.305989955 | 0.444328767 | down |
| 10-Mar | -0.248905686 | 0.714133951 | 0.803879561 | down |
| KIF17 | -0.248856324 | 0.358453416 | 0.49683403 | down |
| BRPF3 | -0.248836193 | 0.314842998 | 0.453458439 | down |
| LPP-AS2 | -0.248829556 | 0.32325877 | 0.461793851 | down |
| ST7L | -0.248712052 | 0.069605552 | 0.155729614 | down |
| RPL41P1 | -0.248691879 | 0.179286509 | 0.304462435 | down |
| RP5-1115A15.1 | -0.248648511 | 0.493731753 | 0.621826736 | down |
| RNF207 | -0.248432789 | 0.153638002 | 0.273313847 | down |
| RP11-680H20.1 | -0.248397368 | 0.426905341 | 0.562465982 | down |
| C9orf64 | -0.248335901 | 0.419901569 | 0.555651988 | down |
| DMKN | -0.248220085 | 0.373789205 | 0.512281896 | down |
| LRRN2 | -0.248186294 | 0.392556333 | 0.530041907 | down |
| TBC1D10A | -0.2480988 | 0.059272921 | 0.138475556 | down |
| RP11-927P21.4 | -0.248001136 | 0.441389589 | 0.575019895 | down |
| HMGA1 | -0.247935402 | 0.175134083 | 0.299664909 | down |
| RP11-386G11.8 | -0.24789907 | 0.860586044 | | down |
| AC114271.2 | -0.247635962 | 0.626350234 | 0.735396711 | down |
| RP11-398K22.12 | -0.247484323 | 0.353497282 | 0.492235463 | down |
| MAK | -0.247475986 | 0.610197896 | 0.721999517 | down |
| RP11-428C19.5 | -0.24732896 | 0.819705898 | | down |
| ZNF572 | -0.247326763 | 0.362810027 | 0.501230719 | down |
| RP11-732A19.1 | -0.247291479 | 0.686402668 | 0.783045328 | down |
| RP11-572O17.1 | -0.2472328 | 0.523965971 | 0.64816707 | down |
| AP000997.1 | -0.247230675 | 0.932659834 | | down |
| SNORD13P1 | -0.247230333 | 0.929821662 | | down |
| RNU1-84P | -0.247230228 | 0.928879821 | | down |
| LINC00616 | -0.247229243 | 0.917610369 | | down |
| CTB-78F1.2 | -0.247228256 | 0.898387234 | | down |
| NA | -0.247228233 | 0.89777968 | | down |
| ANP32D | -0.247227899 | 0.887218753 | | down |
| RNU1-28P | -0.24722742 | 0.864049169 | | down |
| TMED4 | -0.247195831 | 0.102177444 | 0.205146961 | down |
| DCLRE1CP1 | -0.247135131 | 0.658668784 | 0.760706691 | down |
| BTBD6 | -0.24701562 | 0.08762436 | 0.183968292 | down |
| DBH | -0.246986633 | 0.601029823 | 0.714145194 | down |
| RP13-39P12.2 | -0.246883521 | 0.719597106 | | down |
| EEF1A1P25 | -0.246880989 | 0.563936958 | 0.682347892 | down |
| CEP41 | -0.246830954 | 0.122739932 | 0.23367331 | down |

| | | | | |
|---|---|---|---|---|
| MED23 | -0.246645127 | 0.146161871 | 0.264185252 | down |
| AP4B1 | -0.246531555 | 0.072095188 | 0.159724787 | down |
| CTC-559E9.1 | -0.246384019 | 0.348234659 | 0.486983834 | down |
| RP11-98D18.3 | -0.246360643 | 0.473757505 | 0.604064443 | down |
| VPS33B | -0.246316646 | 0.271574884 | 0.407715161 | down |
| MICAL2 | -0.24626054 | 0.236561667 | 0.369611458 | down |
| RPL13AP7 | -0.246241235 | 0.448762205 | 0.581677572 | down |
| RP11-196B3.3 | -0.246178012 | 0.818599648 | | down |
| XL22NC03-24A12.9 | -0.246062994 | 0.646921818 | 0.751735413 | down |
| LOH12CR2 | -0.246040073 | 0.404767648 | 0.541203348 | down |
| GPR17 | -0.245905747 | 0.553369719 | 0.673340077 | down |
| ATG7 | -0.245903721 | 0.13916958 | 0.255263178 | down |
| GOLGA8M | -0.245833919 | 0.726107305 | 0.8135738 | down |
| FBXO27 | -0.24571461 | 0.429648439 | 0.564917187 | down |
| PARS2 | -0.245681368 | 0.199242611 | 0.328326295 | down |
| RP5-899E9.1 | -0.245626234 | 0.482697987 | 0.611880441 | down |
| RP11-793J2.1 | -0.245620499 | 0.613922924 | 0.725113037 | down |
| RP11-61A14.4 | -0.245596436 | 0.696773757 | 0.791023529 | down |
| SPATC1L | -0.245584841 | 0.393806424 | 0.531228458 | down |
| RP11-264M12.4 | -0.245484496 | 0.910148963 | | down |
| ATIC | -0.245469563 | 0.096665654 | 0.197349173 | down |
| ALS2 | -0.24545386 | 0.310462855 | 0.44899362 | down |
| TTC30A | -0.245422912 | 0.308426584 | 0.446938132 | down |
| NA | -0.245171811 | 0.617702935 | 0.728403602 | down |
| PLEKHF2 | -0.245114545 | 0.269830112 | 0.405846101 | down |
| ISY1 | -0.245019079 | 0.150672507 | 0.269856387 | down |
| APTR | -0.244964508 | 0.199852382 | 0.328862141 | down |
| RP11-417N10.5 | -0.244829713 | 0.765946684 | 0.842532939 | down |
| ADAM18 | -0.244803808 | 0.911605935 | | down |
| DENND4A | -0.244770031 | 0.12488435 | 0.236556164 | down |
| C8orf31 | -0.244686308 | 0.41287117 | 0.549495058 | down |
| ATP5F1 | -0.244677572 | 0.051526476 | 0.125045351 | down |
| GDAP2 | -0.244635261 | 0.090479842 | 0.188266249 | down |
| ADSSL1 | -0.244585882 | 0.37953539 | 0.517578015 | down |
| RP11-161H23.10 | -0.244520787 | 0.812573352 | | down |
| NT5M | -0.244502492 | 0.322719403 | 0.461259789 | down |
| NME2P1 | -0.24441538 | 0.495738906 | 0.623490236 | down |
| SERPINA9 | -0.244389002 | 0.760371791 | 0.838242032 | down |
| HNRNPKP2 | -0.244319951 | 0.500882483 | 0.62793713 | down |
| RPS27 | -0.244312754 | 0.16616556 | 0.289239751 | down |
| RUSC1 | -0.244253157 | 0.052863432 | 0.127221159 | down |
| SAXO2 | -0.24418449 | 0.525470538 | 0.649275855 | down |
| VPS33A | -0.24416553 | 0.020442835 | 0.063129257 | down |
| RP11-505P4.6 | -0.244013808 | 0.606112716 | 0.718515247 | down |
| PAQR6 | -0.243970747 | 0.138560899 | 0.254504104 | down |
| ASCC2 | -0.243731972 | 0.005336958 | 0.022805601 | down |
| RP11-416I2.1 | -0.243679434 | 0.733139744 | 0.818674985 | down |
| NEURL1B | -0.243650477 | 0.265796803 | 0.401508329 | down |
| C9orf85 | -0.243495656 | 0.057015377 | 0.134625116 | down |
| FAAP100 | -0.243484535 | 0.126257081 | 0.2383754 | down |
| CCNG1 | -0.24345154 | 0.307548833 | 0.44588125 | down |

| | | | | |
|---|---|---|---|---|
| EPHB6 | -0.243165879 | 0.353542415 | 0.492275493 | down |
| RFXANK | -0.243013483 | 0.080379795 | 0.172840722 | down |
| CH507-9B2.5 | -0.243006899 | 0.281038847 | 0.417417991 | down |
| RP11-57206.1 | -0.242957906 | 0.716009559 | 0.80547389 | down |
| SLC35F1 | -0.242869299 | 0.590960079 | 0.70601259 | down |
| LINC00694 | -0.242770746 | 0.580372222 | 0.696599895 | down |
| MKRN3 | -0.242594286 | 0.641550147 | 0.747372338 | down |
| SDHB | -0.242507065 | 0.206170073 | 0.336091897 | down |
| COG8 | -0.242252005 | 0.193410548 | 0.321515981 | down |
| SUCLA2-AS1 | -0.242138421 | 0.567423788 | 0.685444103 | down |
| CAMK2D | -0.24209472 | 0.056882429 | 0.134395699 | down |
| BMF | -0.242028562 | 0.125131483 | 0.236843758 | down |
| B3GLCT | -0.24198753 | 0.193144205 | 0.321165846 | down |
| HTT-AS | -0.241860781 | 0.789918581 | | down |
| GIGYF2 | -0.24175081 | 0.171268451 | 0.295231473 | down |
| FOXF2 | -0.241698775 | 0.486997324 | 0.615693806 | down |
| AC245100.1 | -0.241671918 | 0.395104151 | 0.532524746 | down |
| PAK5 | -0.241596854 | 0.842451278 | 0.898144359 | down |
| RFX3-AS1 | -0.241507274 | 0.548637967 | 0.669261119 | down |
| NPDC1 | -0.241463804 | 0.322061088 | 0.460827896 | down |
| VAV2 | -0.241404717 | 0.118887781 | 0.228584698 | down |
| NXPE4 | -0.241289214 | 0.61185121 | 0.723348591 | down |
| RP1-68D18.2 | -0.241097565 | 0.705133228 | 0.79716544 | down |
| CIDECP | -0.241081986 | 0.073955017 | 0.162681544 | down |
| RP11-312J18.6 | -0.24095989 | 0.934363949 | | down |
| RN7SL269P | -0.24095989 | 0.934363949 | | down |
| AC008280.1 | -0.24095989 | 0.934363949 | | down |
| ADIPOQ-AS1 | -0.24095989 | 0.934363949 | | down |
| RP11-317B7.3 | -0.24095989 | 0.934363949 | | down |
| RP11-269F21.1 | -0.24095989 | 0.934363949 | | down |
| RP11-241J12.3 | -0.24095989 | 0.934363949 | | down |
| RNA5SP280 | -0.24095989 | 0.934363949 | | down |
| RNU4-5P | -0.24095989 | 0.934363949 | | down |
| RAD1P1 | -0.24095989 | 0.934363949 | | down |
| RP11-236J17.7 | -0.24095989 | 0.934363949 | | down |
| Y_RNA | -0.24095989 | 0.934363949 | | down |
| RP11-443N24.2 | -0.24095989 | 0.934363949 | | down |
| BTBD10P1 | -0.24095989 | 0.934363949 | | down |
| RN7SL441P | -0.24095989 | 0.934363949 | | down |
| RP11-780K2.1 | -0.24095989 | 0.934363949 | | down |
| RP11-83B20.4 | -0.24095989 | 0.934363949 | | down |
| ATP8A2P2 | -0.24095989 | 0.934363949 | | down |
| NA | -0.24095989 | 0.934363949 | | down |
| RP11-804M7.1 | -0.24095989 | 0.934363949 | | down |
| NA | -0.24095989 | 0.934363949 | | down |
| RP11-91I8.2 | -0.24095989 | 0.934363949 | | down |
| SPINT3 | -0.24095989 | 0.934363949 | | down |
| RP1-290F12.3 | -0.24095989 | 0.934363949 | | down |
| FRMD7 | -0.24095989 | 0.934363949 | | down |
| KLF3P1 | -0.240958907 | 0.92835921 | | down |
| RNU1-61P | -0.240958861 | 0.928040351 | | down |

| | | | | |
|---|---|---|---|---|
| RNU6-942P | -0.240958793 | 0.927550159 | | down |
| RPL5P13 | -0.240958736 | 0.927139904 | | down |
| PRSS3P1 | -0.240958504 | 0.925377433 | | down |
| RP11-259018.4 | -0.24095758 | 0.916814577 | | down |
| RP11-1157N2__B.2 | -0.240957571 | 0.916714906 | | down |
| Metazoa_SRP | -0.240956756 | 0.906028836 | | down |
| ANKRD6 | -0.240720908 | 0.360831883 | 0.49923247 | down |
| RP11-1094M14.11 | -0.24058033 | 0.100821367 | 0.203157421 | down |
| HLA-A | -0.240499502 | 0.260070717 | 0.395150778 | down |
| CTC-428G20.6 | -0.240240969 | 0.609125627 | 0.721147903 | down |
| KRT18P15 | -0.240146321 | 0.732427469 | 0.818157726 | down |
| HLA-U | -0.239784056 | 0.776146186 | | down |
| PTPRJ | -0.239609556 | 0.207211538 | 0.337332075 | down |
| MAP4K3 | -0.239513436 | 0.327529941 | 0.466012008 | down |
| LINC01117 | -0.239465509 | 0.627771253 | 0.73640323 | down |
| AC005300.5 | -0.239419297 | 0.812154087 | 0.876166375 | down |
| SUPT6H | -0.239412062 | 0.028025033 | 0.079884639 | down |
| PGRMC2 | -0.239221328 | 0.119609937 | 0.229649856 | down |
| STAC3 | -0.239192516 | 0.287477296 | 0.424671446 | down |
| HNRNPA3P12 | -0.239152014 | 0.661966284 | 0.763488619 | down |
| AKAP3 | -0.239065462 | 0.431039795 | 0.566077703 | down |
| LOXNC01-250H12. | -0.239043094 | 0.655745552 | 0.758410107 | down |
| SLC25A46 | -0.239027599 | 0.163257504 | 0.285386749 | down |
| TMEM147 | -0.23890215 | 0.370389858 | 0.508871085 | down |
| SPRYD7 | -0.23883973 | 0.095372756 | 0.195386768 | down |
| CLCA3P | -0.238806932 | 0.789570913 | 0.859581422 | down |
| NDUFC1 | -0.238729584 | 0.123228592 | 0.234321508 | down |
| RP11-564D11.3 | -0.238635059 | 0.557436261 | 0.677081667 | down |
| NDUFAF5 | -0.238606009 | 0.004228727 | 0.019056226 | down |
| NAIF1 | -0.238568823 | 0.02505684 | 0.073444654 | down |
| RP11-113C12.4 | -0.238442827 | 0.67958348 | 0.777776316 | down |
| C21orf33 | -0.238353991 | 0.664282402 | 0.765366639 | down |
| CRNKL1 | -0.238331129 | 0.009331017 | 0.034781482 | down |
| RP4-737E23.5 | -0.238313749 | 0.730867645 | 0.817079241 | down |
| PDE6G | -0.238235666 | 0.378565554 | 0.516677577 | down |
| NA | -0.238205698 | 0.707879448 | 0.79929082 | down |
| RADIL | -0.238103893 | 0.511134695 | 0.636908478 | down |
| GULP1 | -0.238060341 | 0.411616088 | 0.548286134 | down |
| NA | -0.237973061 | 0.849414011 | | down |
| FGF20 | -0.237939003 | 0.856474963 | | down |
| RP1-78014.1 | -0.237931343 | 0.69532564 | 0.790164292 | down |
| RP11-762L8.6 | -0.237925771 | 0.506393661 | 0.632681463 | down |
| RP11-509J21.2 | -0.237892864 | 0.767796377 | 0.843887849 | down |
| PGBD4P1 | -0.237862387 | 0.711705072 | 0.802193479 | down |
| RN7SL604P | -0.237821625 | 0.935216901 | | down |
| RP11-466J24.1 | -0.237821625 | 0.935216901 | | down |
| SAR1P3 | -0.237821625 | 0.935216901 | | down |
| RP3-461F17.1 | -0.237818077 | 0.829122994 | | down |
| BORCS5 | -0.237779435 | 0.130465817 | 0.243960603 | down |
| CLEC3B | -0.237667917 | 0.453275398 | 0.585830081 | down |
| ZNF684 | -0.237624502 | 0.283433045 | 0.420060301 | down |

| | | | | |
|---|---|---|---|---|
| LINC01132 | -0.237443561 | 0.568307286 | 0.686263027 | down |
| UBE2F | -0.237429815 | 0.116462513 | 0.225207298 | down |
| EIF4E2 | -0.237160832 | 0.043054914 | 0.109780347 | down |
| RP4-647C14.3 | -0.237093162 | 0.576235268 | 0.693161932 | down |
| A4GNT | -0.237047943 | 0.86815766 | | down |
| IFI27L1 | -0.237034624 | 0.266090402 | 0.401749839 | down |
| MATN3 | -0.237032603 | 0.656451172 | 0.758934044 | down |
| ZNF425 | -0.236960056 | 0.397855128 | 0.534912713 | down |
| PSMD6-AS2 | -0.236832881 | 0.688400627 | 0.784579813 | down |
| GBA | -0.236768567 | 0.208885698 | 0.339065423 | down |
| ENTPD7 | -0.23676606 | 0.362086135 | 0.500414731 | down |
| GOLGA4 | -0.236462704 | 0.352515107 | 0.491300454 | down |
| C11orf24 | -0.236452184 | 0.169697083 | 0.293330601 | down |
| RP11-1082L8.3 | -0.236196789 | 0.596303955 | 0.710333323 | down |
| CDC20 | -0.236152404 | 0.391433368 | 0.529045577 | down |
| HIST4H4 | -0.236081269 | 0.474020569 | 0.604271639 | down |
| GLE1 | -0.236050681 | 0.00286219 | 0.013975563 | down |
| KCNMB1 | -0.236050432 | 0.51933349 | 0.643921232 | down |
| KIAA0368 | -0.23588471 | 0.007339252 | 0.02899796 | down |
| NA | -0.235760264 | 0.565760008 | 0.683819193 | down |
| POLH | -0.23574267 | 0.206725197 | 0.336774858 | down |
| FLCN | -0.23574183 | 0.067200054 | 0.151726686 | down |
| NHLRC1 | -0.2357012 | 0.355711953 | 0.494299697 | down |
| PRKAR2A-AS1 | -0.235643633 | 0.419309873 | 0.555137964 | down |
| LLNLR-304G9.1 | -0.235606803 | 0.750771286 | 0.831405164 | down |
| LRP2BP | -0.235549968 | 0.381558816 | 0.519370834 | down |
| SLC4A5 | -0.235505834 | 0.439380699 | 0.57341463 | down |
| PSTPIP1 | -0.235394637 | 0.453685276 | 0.58628416 | down |
| NDUFS2 | -0.235334375 | 0.166851723 | 0.289992286 | down |
| MSRB1 | -0.235324076 | 0.21786023 | 0.349243153 | down |
| AC008753.3 | -0.235155357 | 0.695898522 | 0.790596351 | down |
| NA | -0.235131382 | 0.650960563 | 0.754822816 | down |
| CCL28 | -0.235091354 | 0.351519397 | 0.490276625 | down |
| RP11-133K1.12 | -0.235085911 | 0.624081821 | 0.73359342 | down |
| RP4-706A16.3 | -0.235072895 | 0.263766681 | 0.39914406 | down |
| COX6A1P2 | -0.234922147 | 0.368410585 | 0.506986125 | down |
| KLHDC2 | -0.234844946 | 0.385367754 | 0.523133418 | down |
| AP000925.2 | -0.234762089 | 0.812042712 | | down |
| ZNF23 | -0.234691383 | 0.639445369 | 0.745643185 | down |
| Y_RNA | -0.234596973 | 0.914787327 | | down |
| AC005330.2 | -0.234596665 | 0.67362512 | 0.772870737 | down |
| CTA-217C2.2 | -0.234469858 | 0.676269149 | 0.775252988 | down |
| GHRL | -0.234353298 | 0.443106793 | 0.57665687 | down |
| RP11-115D19.1 | -0.23429696 | 0.696842973 | 0.791050793 | down |
| RP5-1092A11.5 | -0.234056803 | 0.732679013 | 0.818282156 | down |
| COX15 | -0.234046834 | 0.015727656 | 0.051792101 | down |
| RP11-347C12.3 | -0.233937059 | 0.648196357 | 0.752663343 | down |
| AC011997.1 | -0.233892694 | 0.827644076 | | down |
| TICRR | -0.23381215 | 0.30111605 | 0.438884713 | down |
| ABCG1 | -0.233778525 | 0.185213038 | 0.311372644 | down |
| LINC01415 | -0.233765797 | 0.663429136 | 0.764706118 | down |

| | | | | |
|---|---|---|---|---|
| TSSK6 | -0.233727719 | 0.379110775 | 0.517116322 | down |
| KLRF1 | -0.233565708 | 0.51898738 | 0.643638362 | down |
| AK2 | -0.233495222 | 0.03751235 | 0.099033504 | down |
| RP11-600F24.7 | -0.233432106 | 0.392836417 | 0.530300921 | down |
| RP11-486F17.1 | -0.233417288 | 0.909455448 | 0.94326922 | down |
| COX16 | -0.233205942 | 0.272090582 | 0.408326146 | down |
| CLIC1P1 | -0.23317705 | 0.687166046 | 0.783576051 | down |
| EBF1 | -0.233132282 | 0.411552743 | 0.548226063 | down |
| ACAP3 | -0.233110665 | 0.17906328 | 0.304194212 | down |
| DDX28 | -0.233109903 | 0.114393794 | 0.222320147 | down |
| RUNDC1 | -0.23308351 | 0.016303258 | 0.053136806 | down |
| CLIP1 | -0.232967556 | 0.257566566 | 0.392640671 | down |
| ARVCF | -0.23290402 | 0.314723015 | 0.453368957 | down |
| GOLGB1 | -0.232765604 | 0.143419321 | 0.260702449 | down |
| ARRDC1-AS1 | -0.232684793 | 0.056074027 | 0.132966698 | down |
| RN7SL600P | -0.232653792 | 0.754416557 | 0.833959171 | down |
| FBXW9 | -0.232507674 | 0.292947293 | 0.430255597 | down |
| SNX7 | -0.232456638 | 0.280775991 | 0.417233845 | down |
| ABR | -0.2323014 | 0.131063881 | 0.244713395 | down |
| RP11-830F9.5 | -0.23228104 | 0.647355731 | 0.752006944 | down |
| ATP5G2 | -0.232220587 | 0.19341898 | 0.321515981 | down |
| C4orf33 | -0.232185728 | 0.313836759 | 0.4524864 | down |
| RP11-411B10.2 | -0.232146434 | 0.688775332 | 0.784824294 | down |
| MCRS1 | -0.231987395 | 0.037242559 | 0.098461408 | down |
| UCHL3 | -0.231920874 | 0.47334221 | 0.6037399 | down |
| SLC45A4 | -0.231763727 | 0.20855668 | 0.338714357 | down |
| RP11-253M7.1 | -0.231722143 | 0.618481033 | 0.729033399 | down |
| VPS50 | -0.231665939 | 0.175967393 | 0.300691865 | down |
| RP11-147C23.1 | -0.231605632 | 0.872962224 | 0.919062167 | down |
| NBR1 | -0.231553056 | 0.099289997 | 0.201054907 | down |
| RPL23AP85 | -0.23155084 | 0.936921464 | | down |
| RP4-641G12.4 | -0.23155084 | 0.936921464 | | down |
| NA | -0.23155084 | 0.936921464 | | down |
| NIFKP9 | -0.23155084 | 0.936921464 | | down |
| AC008277.1 | -0.23155084 | 0.936921464 | | down |
| AC069155.1 | -0.23155084 | 0.936921464 | | down |
| NAALADL2-AS1 | -0.23155084 | 0.936921464 | | down |
| RP11-395I6.2 | -0.23155084 | 0.936921464 | | down |
| RP11-324J13.1 | -0.23155084 | 0.936921464 | | down |
| LINC01218 | -0.23155084 | 0.936921464 | | down |
| RPL5P14 | -0.23155084 | 0.936921464 | | down |
| RP11-1250I15.2 | -0.23155084 | 0.936921464 | | down |
| RP11-252I14.1 | -0.23155084 | 0.936921464 | | down |
| NA | -0.23155084 | 0.936921464 | | down |
| RP5-874C20.7 | -0.23155084 | 0.936921464 | | down |
| RP1-72A23.4 | -0.23155084 | 0.936921464 | | down |
| NCOA7-AS1 | -0.23155084 | 0.936921464 | | down |
| TRGVA | -0.23155084 | 0.936921464 | | down |
| RP11-705O24.3 | -0.23155084 | 0.936921464 | | down |
| RP11-7F18.1 | -0.23155084 | 0.936921464 | | down |
| RN7SL760P | -0.23155084 | 0.936921464 | | down |

| | | | | |
|---|---|---|---|---|
| NRBF2P4 | -0.23155084 | 0.936921464 | | down |
| Y_RNA | -0.23155084 | 0.936921464 | | down |
| RP11-643G5.5 | -0.23155084 | 0.936921464 | | down |
| NIFKP3 | -0.23155084 | 0.936921464 | | down |
| RN7SKP172 | -0.23155084 | 0.936921464 | | down |
| Y_RNA | -0.23155084 | 0.936921464 | | down |
| PPIAP25 | -0.23155084 | 0.936921464 | | down |
| NA | -0.23155084 | 0.936921464 | | down |
| NA | -0.23155084 | 0.936921464 | | down |
| RNA5SP36 | -0.23155084 | 0.936921464 | | down |
| IGHV3-62 | -0.23155084 | 0.936921464 | | down |
| RP11-429P3.2 | -0.23155084 | 0.936921464 | | down |
| RP11-566K11.4 | -0.23155084 | 0.936921464 | | down |
| RP4-673D20.4 | -0.23155084 | 0.936921464 | | down |
| MIR3194 | -0.23155084 | 0.936921464 | | down |
| AP001476.4 | -0.23155084 | 0.936921464 | | down |
| BMS1P22 | -0.23155084 | 0.936921464 | | down |
| Y_RNA | -0.23155084 | 0.936921464 | | down |
| IPO7P1 | -0.23155084 | 0.936921464 | | down |
| RP11-355K23B.1 | -0.23155084 | 0.936921464 | | down |
| RP11-530C5.1 | -0.231549792 | 0.930503667 | | down |
| RP11-340I6.6 | -0.231549711 | 0.929925836 | | down |
| RP11-517C16.4 | -0.231549654 | 0.929507129 | | down |
| RP11-92B11.3 | -0.231549653 | 0.929497081 | | down |
| RN7SL388P | -0.231549621 | 0.929260476 | | down |
| RN7SKP235 | -0.231548497 | 0.918981143 | | down |
| TCEA1P4 | -0.231548494 | 0.918948511 | | down |
| RN7SL68P | -0.231548481 | 0.918797452 | | down |
| OR8G3P | -0.231548479 | 0.918777155 | | down |
| AC079199.2 | -0.231548462 | 0.91859157 | | down |
| AC017079.4 | -0.231548393 | 0.917788868 | | down |
| MIR3193 | -0.231547744 | 0.908907604 | | down |
| TRAJ58 | -0.23154774 | 0.908854783 | | down |
| FAM35CP | -0.231547737 | 0.908795056 | | down |
| VDAC1P11 | -0.231547188 | 0.898482226 | | down |
| RNU1-103P | -0.23135208 | 0.770189155 | 0.84558973 | down |
| NA | -0.231025634 | 0.665075137 | 0.76594566 | down |
| RP11-486I11.2 | -0.230958112 | 0.46407239 | 0.595582695 | down |
| KRT15 | -0.230954616 | 0.554699204 | 0.674608253 | down |
| FAM185A | -0.230819754 | 0.212010601 | 0.342751202 | down |
| RP11-133K1.11 | -0.230729601 | 0.804872356 | | down |
| RP11-363E7.4 | -0.230683978 | 0.395036225 | 0.532457082 | down |
| RP11-598F7.6 | -0.230453067 | 0.681126191 | 0.779046946 | down |
| GDAP1 | -0.230440162 | 0.386008167 | 0.523695163 | down |
| CTD-2260A17.2 | -0.230283826 | 0.423635525 | 0.559262928 | down |
| RP11-489E7.4 | -0.230205733 | 0.583037229 | 0.698906649 | down |
| EIF2B3 | -0.230136127 | 0.229542151 | 0.361888492 | down |
| LINC01598 | -0.230031166 | 0.808231835 | | down |
| RP11-148L24.1 | -0.230015591 | 0.707619142 | 0.799117032 | down |
| ARF5 | -0.230006533 | 0.1272985 | 0.239648859 | down |
| EMB | -0.229847215 | 0.423201575 | 0.558788262 | down |

| | | | | |
|---|---|---|---|---|
| LINC00683 | -0.229816244 | 0.468205181 | 0.599222852 | down |
| DTX2P1 | -0.229663145 | 0.740386826 | 0.823614551 | down |
| AC073333.8 | -0.229629766 | 0.834462998 | | down |
| LRP6 | -0.229594869 | 0.330264713 | 0.468813352 | down |
| RHBDL2 | -0.229527605 | 0.504746189 | 0.631437146 | down |
| GAPDHP71 | -0.229476123 | 0.643720325 | 0.749203278 | down |
| ENKUR | -0.229426091 | 0.630851318 | 0.738790603 | down |
| SLC39A1 | -0.229333268 | 0.583105476 | 0.698935604 | down |
| KDELR1 | -0.229283145 | 0.238952811 | 0.372398499 | down |
| POMT2 | -0.229258946 | 0.07378979 | 0.162460828 | down |
| RP11-512F24.1 | -0.229171443 | 0.609742133 | 0.721650291 | down |
| MAMDC2 | -0.229162317 | 0.653889373 | 0.75707936 | down |
| ATP5C1 | -0.229112904 | 0.180516702 | 0.305839045 | down |
| C10orf35 | -0.22910415 | 0.268574107 | 0.404382102 | down |
| RET | -0.229101053 | 0.51813024 | 0.642941184 | down |
| CTD-2020K17.3 | -0.229058833 | 0.570108274 | 0.687719143 | down |
| VPS25 | -0.229034208 | 0.028265703 | 0.080364794 | down |
| SNX8 | -0.228947943 | 0.097998496 | 0.199152246 | down |
| HSD17B10 | -0.228947817 | 0.244899624 | 0.378797585 | down |
| ZFYVE28 | -0.228851756 | 0.267888488 | 0.40365323 | down |
| EML3 | -0.228839219 | 0.291748489 | 0.428977223 | down |
| ISL2 | -0.228820746 | 0.45788684 | 0.589937486 | down |
| TMEM127 | -0.22872869 | 0.180112138 | 0.305343023 | down |
| MIR573 | -0.228719898 | 0.783848126 | 0.85546495 | down |
| ZNF669 | -0.228682521 | 0.227691143 | 0.359869151 | down |
| DOCK1 | -0.22857035 | 0.390351118 | 0.528013552 | down |
| AC092159.3 | -0.228411352 | 0.925207796 | | down |
| RP13-539F13.3 | -0.228411234 | 0.923551225 | | down |
| NA | -0.228409815 | 0.887369427 | | down |
| RP11-195B3.1 | -0.228409651 | 0.878891359 | | down |
| RP1-91J24.3 | -0.228409625 | 0.877357621 | | down |
| Y_RNA | -0.228409618 | 0.876964248 | | down |
| MYL6P3 | -0.228409578 | 0.874469614 | | down |
| RN7SL328P | -0.228409342 | 0.855760014 | | down |
| RP11-983P16.2 | -0.228409342 | 0.855774797 | | down |
| CH507-42P11.6 | -0.228409241 | 0.844857978 | | down |
| ATP5A1P3 | -0.228409169 | 0.835285707 | | down |
| RP4-569M23.2 | -0.228300927 | 0.593481726 | 0.708067865 | down |
| HIST1H2AG | -0.228285254 | 0.399702644 | 0.536604238 | down |
| RP11-351D16.3 | -0.228222283 | 0.562933614 | 0.681939337 | down |
| ZDHHC9 | -0.228144665 | 0.34292708 | 0.481354008 | down |
| HNRNPH1P1 | -0.228100537 | 0.721686626 | | down |
| SYT12 | -0.227902629 | 0.606631016 | 0.71898782 | down |
| ADCY10P1 | -0.227901893 | 0.394502318 | 0.531928357 | down |
| DGCR9 | -0.227735313 | 0.628484045 | 0.736894109 | down |
| AC087294.2 | -0.227601092 | 0.579375791 | 0.695686274 | down |
| PTENP1 | -0.227533103 | 0.553509693 | 0.673483121 | down |
| BIN1 | -0.22750327 | 0.163235621 | 0.285379749 | down |
| CLPP | -0.227472682 | 0.086429079 | 0.182235613 | down |
| RP11-770J1.5 | -0.22744345 | 0.647274608 | 0.751957996 | down |
| NA | -0.227347568 | 0.799120459 | | down |

| | | | | |
|---|---|---|---|---|
| PLBD1-AS1 | -0.227341144 | 0.435903979 | 0.570570259 | down |
| GDF5 | -0.227231976 | 0.792248255 | 0.861559582 | down |
| KLF4 | -0.227202786 | 0.440570793 | 0.574276927 | down |
| PCDHGA9 | -0.227140123 | 0.678723993 | 0.77719991 | down |
| AF131215.9 | -0.227101765 | 0.367030283 | 0.505596415 | down |
| TMUB2 | -0.227017711 | 0.097753259 | 0.198815252 | down |
| CARD9 | -0.22697963 | 0.2678532 | 0.403625729 | down |
| ZNRF2P1 | -0.226943215 | 0.533847337 | 0.656632662 | down |
| SYCE3 | -0.226887849 | 0.652309591 | 0.755745386 | down |
| DAB2IP | -0.226841223 | 0.268050532 | 0.403816387 | down |
| USMG5 | -0.226702386 | 0.186066928 | 0.312354445 | down |
| RP11-568K15.1 | -0.22667987 | 0.231617772 | 0.364273902 | down |
| SREK1IP1 | -0.226672716 | 0.215093006 | 0.346165771 | down |
| HARBI1 | -0.226625206 | 0.314342696 | 0.452976736 | down |
| SPEF2 | -0.226569352 | 0.37124701 | 0.509605939 | down |
| MARK1 | -0.22649688 | 0.471584807 | 0.60213744 | down |
| RER1 | -0.226470984 | 0.180165342 | 0.305398756 | down |
| CYP4F29P | -0.226416158 | 0.592968913 | 0.707757159 | down |
| SEC62-AS1 | -0.226361448 | 0.835728337 | | down |
| RP11-142A22.4 | -0.226345067 | 0.877322857 | | down |
| DNM2 | -0.226290667 | 0.022073575 | 0.066771896 | down |
| MECR | -0.226262965 | 0.060658726 | 0.140891439 | down |
| RPS2P55 | -0.226144975 | 0.326188524 | 0.464569556 | down |
| NKAPP1 | -0.226135807 | 0.455420787 | 0.587837786 | down |
| TARS2 | -0.226101356 | 0.031889955 | 0.087708522 | down |
| ZNF382 | -0.226065041 | 0.545314891 | 0.666562536 | down |
| CTD-2256P15.3 | -0.226058624 | 0.764412218 | | down |
| LPIN2 | -0.225907717 | 0.304432877 | 0.442538289 | down |
| NCBP2-AS2 | -0.225754725 | 0.226089008 | 0.358147114 | down |
| COX10-AS1 | -0.225554616 | 0.314215161 | 0.452858117 | down |
| CHCHD2P4 | -0.225416755 | 0.846578395 | 0.901105409 | down |
| DACH1 | -0.225414033 | 0.432358456 | 0.567412635 | down |
| FMR1 | -0.225281474 | 0.03829442 | 0.10063612 | down |
| CLPB | -0.225235601 | 0.261685974 | 0.396794863 | down |
| RARRES3 | -0.22522989 | 0.348647681 | 0.487379998 | down |
| SAMD15 | -0.225104028 | 0.579977899 | 0.69621448 | down |
| RARS | -0.225100237 | 0.039970371 | 0.103899449 | down |
| NA | -0.224973098 | 0.645277361 | 0.750288828 | down |
| PVR | -0.224953031 | 0.294032486 | 0.431448694 | down |
| RP11-849F2.10 | -0.224946968 | 0.797206238 | | down |
| RP11-268P4.5 | -0.224632732 | 0.784104586 | 0.855500804 | down |
| RP11-364B14.3 | -0.2243982 | 0.817333464 | | down |
| NSUN6 | -0.224077347 | 0.30161649 | 0.439443569 | down |
| LYSMD4 | -0.224076929 | 0.121320618 | 0.231852722 | down |
| RP11-472M19.2 | -0.224014233 | 0.838936632 | | down |
| ARHGEF12 | -0.223957599 | 0.291945466 | 0.429161835 | down |
| FLAD1 | -0.223908701 | 0.118856409 | 0.228539005 | down |
| AC009302.2 | -0.223791565 | 0.764293267 | | down |
| NA | -0.223689906 | 0.760486835 | 0.838307337 | down |
| DBI | -0.223546577 | 0.14464502 | 0.262184916 | down |
| LASP1 | -0.223472554 | 0.469114865 | 0.600050814 | down |

| | | | | |
|---|---|---|---|---|
| TUBGCP4 | -0.223356894 | 0.373956759 | 0.512355419 | down |
| CLEC11A | -0.223352737 | 0.429117552 | 0.564466187 | down |
| MTR | -0.223266284 | 0.368356107 | 0.506957896 | down |
| CUX1 | -0.223237217 | 0.00554707 | 0.023482622 | down |
| CPEB2 | -0.223181924 | 0.310135423 | 0.448635402 | down |
| SETP12 | -0.223075467 | 0.816733258 | 0.87971656 | down |
| APC | -0.223070089 | 0.287340631 | 0.424532182 | down |
| RP11-343H5.4 | -0.223051696 | 0.626487619 | 0.735500529 | down |
| TMEFF1 | -0.222994339 | 0.846917372 | | down |
| LINC00960 | -0.222797258 | 0.556459847 | 0.676196394 | down |
| RP11-307B6.3 | -0.222762101 | 0.709346355 | 0.800315516 | down |
| ZNF428 | -0.222657215 | 0.347886639 | 0.48665578 | down |
| RP11-106D4.2 | -0.222640483 | 0.585634382 | 0.701211517 | down |
| MYL3 | -0.22263483 | 0.390538806 | 0.528243647 | down |
| FEM1C | -0.222556108 | 0.16097746 | 0.282549909 | down |
| RBM12B-AS1 | -0.222504518 | 0.664644647 | 0.76564967 | down |
| TMBIM1 | -0.222367326 | 0.196154324 | 0.324649321 | down |
| PAK1 | -0.222304987 | 0.139000987 | 0.25504202 | down |
| NDNF | -0.222295733 | 0.627853295 | 0.736441961 | down |
| CDKL5 | -0.222265172 | 0.442816159 | 0.576378506 | down |
| MANBAL | -0.222221041 | 0.059475002 | 0.138819312 | down |
| POPDC2 | -0.222154551 | 0.528932651 | 0.652189632 | down |
| GDNF | -0.222145941 | 0.613522525 | 0.724754088 | down |
| RP11-367J7.4 | -0.22214179 | 0.93947963 | | down |
| RP11-389C8.1 | -0.22214179 | 0.93947963 | | down |
| RPS27AP10 | -0.22214179 | 0.93947963 | | down |
| Y_RNA | -0.22214179 | 0.93947963 | | down |
| RP11-36B6.1 | -0.22214179 | 0.93947963 | | down |
| RP11-90B22.1 | -0.22214179 | 0.93947963 | | down |
| MIR5093 | -0.22214179 | 0.93947963 | | down |
| SLURP1 | -0.22214177 | 0.939375727 | | down |
| KRT19P4 | -0.222141604 | 0.938482589 | | down |
| RP11-266K4.1 | -0.222140501 | 0.931338808 | | down |
| MIR4511 | -0.222140355 | 0.930195405 | | down |
| OPN5 | -0.222140349 | 0.930148914 | | down |
| SPTLC1P4 | -0.222140298 | 0.92972928 | | down |
| RP11-553D4.2 | -0.222139498 | 0.92208828 | | down |
| HERC2P7 | -0.222139451 | 0.921554391 | | down |
| SLC25A14P1 | -0.222139399 | 0.920960406 | | down |
| PHBP10 | -0.222139391 | 0.920858489 | | down |
| RPS3AP29 | -0.222139384 | 0.920785551 | | down |
| AC007919.18 | -0.222139362 | 0.920522874 | | down |
| TREML5P | -0.22213936 | 0.920498344 | | down |
| Y_RNA | -0.222139304 | 0.919827326 | | down |
| NA | -0.222138733 | 0.911806156 | | down |
| MIR5091 | -0.222138649 | 0.910425942 | | down |
| RP1-283K11.2 | -0.222138157 | 0.900673933 | | down |
| ATP13A2 | -0.222105731 | 0.28261076 | 0.419110405 | down |
| USMG5P1 | -0.222034337 | 0.612512867 | 0.723789049 | down |
| ZNF662 | -0.221928745 | 0.322604978 | 0.461196237 | down |
| U47924.32 | -0.221915927 | 0.666937335 | 0.767366165 | down |

| | | | | |
|---|---|---|---|---|
| UROD | -0.221844261 | 0.181810621 | 0.307355295 | down |
| RP11-216P16.7 | -0.221839819 | 0.894582463 | | down |
| EEF1E1P1 | -0.221792063 | 0.814522116 | 0.878007311 | down |
| HPSE2 | -0.221768489 | 0.495752847 | 0.623490236 | down |
| CSAG3 | -0.221629668 | 0.768393322 | 0.84426598 | down |
| INPP5A | -0.221612925 | 0.304543704 | 0.442613731 | down |
| UBE2L3 | -0.221539919 | 0.061751765 | 0.142789966 | down |
| ANXA8L1 | -0.221463177 | 0.858199347 | | down |
| RP11-809C18.3 | -0.221405722 | 0.716065896 | 0.805507105 | down |
| NSFL1C | -0.221370572 | 0.044341694 | 0.112043889 | down |
| HGD | -0.221320689 | 0.463766294 | 0.595317006 | down |
| PLEKHB1 | -0.221134694 | 0.152077128 | 0.271463681 | down |
| ACRC | -0.221097287 | 0.358505284 | 0.496845302 | down |
| PCDHAC1 | -0.220963715 | 0.756174524 | 0.835144965 | down |
| LNPEP | -0.220932368 | 0.266176481 | 0.401839415 | down |
| RPL37A | -0.220913715 | 0.228229957 | 0.360531092 | down |
| NA | -0.220779698 | 0.729669634 | 0.816256264 | down |
| KRT8P49 | -0.220762188 | 0.785275829 | | down |
| TDRD7 | -0.220747646 | 0.279501571 | 0.415771906 | down |
| NA | -0.220741069 | 0.287086745 | 0.424261395 | down |
| BRAP | -0.220725732 | 0.003144922 | 0.015016853 | down |
| FBXO31 | -0.220533648 | 0.092457884 | 0.191121598 | down |
| RP11-477N3.1 | -0.220532061 | 0.444912795 | 0.578339974 | down |
| ZNF747 | -0.220362632 | 0.139839927 | 0.256070627 | down |
| AC008746.10 | -0.220351938 | 0.756649746 | 0.835424628 | down |
| DENND6B | -0.220195884 | 0.41048472 | 0.547253306 | down |
| MRAP | -0.220123147 | 0.837383399 | | down |
| KIAA0101 | -0.220085659 | 0.40123762 | 0.538111912 | down |
| Y_RNA | -0.219963728 | 0.611105865 | 0.722808732 | down |
| CD164 | -0.219820657 | 0.2507823 | 0.385475464 | down |
| ARMC6 | -0.219609447 | 0.189039401 | 0.316177685 | down |
| RP11-669M16.2 | -0.219550742 | 0.913483932 | | down |
| RP11-552D8.1 | -0.219535893 | 0.872733959 | | down |
| LINC00539 | -0.219417515 | 0.505982264 | 0.63237785 | down |
| KDM1B | -0.219334146 | 0.161241987 | 0.282915041 | down |
| RP11-54G14.1 | -0.219303672 | 0.621338023 | 0.731369674 | down |
| RP1-234P15.4 | -0.219222666 | 0.486463613 | 0.615226165 | down |
| FUT4 | -0.219185684 | 0.348293415 | 0.487003282 | down |
| TELO2 | -0.219179406 | 0.278074918 | 0.414314973 | down |
| RPS3 | -0.219097909 | 0.148420268 | 0.267110251 | down |
| ZNF692 | -0.219072064 | 0.270179273 | 0.406188255 | down |
| BTF3P14 | -0.219001807 | 0.919414366 | | down |
| SAA4 | -0.219001395 | 0.91003487 | | down |
| RP11-379K17.9 | -0.219001103 | 0.900964578 | | down |
| RP11-15E18.5 | -0.219000554 | 0.872388142 | | down |
| NA | -0.219000378 | 0.85624449 | | down |
| AC079781.5 | -0.219000374 | 0.855862037 | | down |
| PPP1R8P1 | -0.219000363 | 0.854660981 | | down |
| GNG4 | -0.218990728 | 0.390661979 | 0.528338892 | down |
| CTD-2589M5.4 | -0.218979648 | 0.575500778 | 0.692500215 | down |
| TDH | -0.218967826 | 0.661344059 | 0.763005296 | down |

| | | | | |
|---|---|---|---|---|
| HNRNPA1P12 | -0.218870757 | 0.814558253 | | down |
| BPIFB2 | -0.21884246 | 0.878706402 | 0.922307357 | down |
| GCSHP5 | -0.218642978 | 0.773502329 | | down |
| FABP3 | -0.218439428 | 0.431305812 | 0.5664023 | down |
| NME7 | -0.218408756 | 0.437411442 | 0.571995184 | down |
| RNY3P8 | -0.218304268 | 0.575720226 | 0.692736533 | down |
| RPL12P25 | -0.21828993 | 0.759665713 | | down |
| FABP5 | -0.218222013 | 0.297354081 | 0.434751375 | down |
| COLCA2 | -0.218175804 | 0.440988468 | 0.574596997 | down |
| RP11-74J13.9 | -0.218151817 | 0.520850776 | 0.645179946 | down |
| CEBPB-AS1 | -0.218139454 | 0.421342463 | 0.557022144 | down |
| FAM156A | -0.218127861 | 0.627098159 | 0.735929733 | down |
| TPTEP1 | -0.21806229 | 0.429973869 | 0.565221392 | down |
| CDH6 | -0.217828317 | 0.521138029 | 0.645439482 | down |
| NA | -0.21772291 | 0.638676845 | 0.745074478 | down |
| RP1-27K12.4 | -0.21771688 | 0.759579928 | | down |
| HMBOX1 | -0.217507411 | 0.205511706 | 0.335377703 | down |
| SREBF1 | -0.217084924 | 0.221027617 | 0.352695652 | down |
| BAMBI | -0.21706745 | 0.335375409 | 0.473615719 | down |
| MLST8 | -0.217059385 | 0.163638631 | 0.285884691 | down |
| ACTN4 | -0.21681441 | 0.21917064 | 0.350601996 | down |
| CMAS | -0.21673846 | 0.406814296 | 0.543311198 | down |
| RP11-68606.2 | -0.21672622 | 0.363819671 | 0.502146253 | down |
| ARHGAP12 | -0.216672053 | 0.25558123 | 0.390633488 | down |
| NA | -0.216583536 | 0.766188697 | 0.84270659 | down |
| AC003090.1 | -0.21657357 | 0.560723337 | 0.68004611 | down |
| IDH1-AS1 | -0.216483357 | 0.51644958 | 0.641483842 | down |
| CTD-3224K15.2 | -0.216457448 | 0.796469145 | 0.864491293 | down |
| ATXN7L1 | -0.216434376 | 0.149540101 | 0.268493371 | down |
| TRIM31 | -0.216113911 | 0.545116258 | 0.666455312 | down |
| NA | -0.216020377 | 0.730802887 | 0.817079241 | down |
| RP11-351I24.1 | -0.216013915 | 0.750727346 | 0.831405164 | down |
| GLULP4 | -0.215996451 | 0.745643455 | 0.827256719 | down |
| SNTA1 | -0.21572025 | 0.236084518 | 0.369038672 | down |
| RPL21 | -0.21558423 | 0.211030462 | 0.341574463 | down |
| AC000067.1 | -0.215500096 | 0.838674915 | | down |
| LINC01136 | -0.215496308 | 0.619194717 | 0.729569865 | down |
| APOBEC3D | -0.215191123 | 0.491119992 | 0.619389988 | down |
| DCUN1D2-AS | -0.215162268 | 0.791909191 | | down |
| FAM117B | -0.215114299 | 0.173868943 | 0.298386004 | down |
| VARS | -0.215101021 | 0.297400387 | 0.434797919 | down |
| STX16-NPEPL1 | -0.215059103 | 0.633150305 | 0.740717564 | down |
| RP11-456I15.2 | -0.214829618 | 0.884130767 | 0.926147093 | down |
| Z69720.3 | -0.214785001 | 0.777574281 | 0.85095347 | down |
| ZNF347 | -0.214639968 | 0.40170784 | 0.538527254 | down |
| AUH | -0.214596689 | 0.381287587 | 0.519142766 | down |
| CLDN11 | -0.21453134 | 0.529491814 | 0.652711504 | down |
| AP001062.9 | -0.21451356 | 0.803195338 | 0.869624157 | down |
| RP1-278E11.3 | -0.214468935 | 0.484362138 | 0.613239616 | down |
| RP11-85A1.3 | -0.214438585 | 0.609443003 | 0.72139641 | down |
| HMGN2P5 | -0.214417942 | 0.431510437 | 0.566598408 | down |

| | | | | |
|---|---|---|---|---|
| METTL21A | -0.214284402 | 0.104290932 | 0.208000316 | down |
| TET2 | -0.214254743 | 0.293288288 | 0.430570486 | down |
| TPTE | -0.214215874 | 0.887500448 | 0.927962117 | down |
| NUBP2 | -0.214178598 | 0.250458645 | 0.385173693 | down |
| TMOD4 | -0.214111781 | 0.525174599 | 0.649047538 | down |
| TOR2A | -0.214107755 | 0.195754038 | 0.324286429 | down |
| GPR63 | -0.21389779 | 0.519723008 | 0.64424467 | down |
| RPS3A | -0.213849323 | 0.222060504 | 0.353892732 | down |
| KDM4A | -0.213791627 | 0.1052463 | 0.209557945 | down |
| NIT1 | -0.213678354 | 0.277064527 | 0.41329891 | down |
| RP11-263K4.5 | -0.213582175 | 0.773409999 | 0.847886517 | down |
| HLCS | -0.213536288 | 0.124975219 | 0.236639282 | down |
| SRC | -0.213523084 | 0.190535182 | 0.318042058 | down |
| RP11-231G3.1 | -0.213454763 | 0.539447137 | 0.661327367 | down |
| RP11-278C7.4 | -0.213377006 | 0.497669332 | 0.625004281 | down |
| POLR1B | -0.213376476 | 0.226070154 | 0.35813613 | down |
| PIAS2 | -0.213359277 | 0.122466381 | 0.233320285 | down |
| EEF1DP4 | -0.21325968 | 0.713215468 | 0.803336644 | down |
| GFPT1 | -0.213209084 | 0.24518595 | 0.379162377 | down |
| HDGF | -0.213200187 | 0.035876719 | 0.095765162 | down |
| RP11-96D1.10 | -0.21317495 | 0.647199369 | 0.751957996 | down |
| DYM | -0.21307913 | 0.011855617 | 0.041806357 | down |
| IFNGR2 | -0.21304167 | 0.091450699 | 0.189616618 | down |
| AC011290.5 | -0.213025106 | 0.710674355 | 0.80145266 | down |
| EEF1B2P4 | -0.212905374 | 0.881841636 | | down |
| FOXD4 | -0.212864003 | 0.663415903 | 0.764706118 | down |
| YWHAZP2 | -0.212809768 | 0.775372384 | 0.849541881 | down |
| RAD23BP2 | -0.21273274 | 0.94203842 | | down |
| U95743.1 | -0.212732582 | 0.941207772 | | down |
| LRRC37A13P | -0.212731201 | 0.931951188 | | down |
| RP11-140A10.3 | -0.212731105 | 0.93113422 | | down |
| RP11-280G9.1 | -0.212731087 | 0.93098089 | | down |
| RP11-87N3.6 | -0.212730472 | 0.924898286 | | down |
| RNU6-856P | -0.212730376 | 0.923795702 | | down |
| RP11-511I11.1 | -0.212729662 | 0.913705836 | | down |
| RP11-680F20.9 | -0.212729209 | 0.904748115 | | down |
| RP11-256L6.3 | -0.212729204 | 0.904639343 | | down |
| TRAV40 | -0.21272883 | 0.894658675 | | down |
| VN2R19P | -0.21272883 | 0.894657601 | | down |
| AC103563.8 | -0.212551977 | 0.690988343 | 0.786723275 | down |
| AOC4P | -0.212415209 | 0.703638457 | 0.796171647 | down |
| CLEC12B | -0.212357683 | 0.756415812 | 0.835268149 | down |
| PAXBP1-AS1 | -0.212233411 | 0.514176748 | 0.639521648 | down |
| PSMC3 | -0.212112461 | 0.222170261 | 0.354030091 | down |
| RNASEL | -0.212072349 | 0.068130503 | 0.15332283 | down |
| RHBDD3 | -0.212063784 | 0.348930795 | 0.487639632 | down |
| RP11-374F3.5 | -0.2119197 | 0.716471238 | 0.805818398 | down |
| RP11-545P7.9 | -0.211714856 | 0.685382615 | 0.782208368 | down |
| RP11-553K8.2 | -0.211695795 | 0.747270996 | 0.828572845 | down |
| CCND2-AS2 | -0.211687239 | 0.469860862 | 0.600523237 | down |
| ZNF33B | -0.211640402 | 0.085611195 | 0.181057494 | down |

| | | | | |
|---|---|---|---|---|
| TXNL4A | -0.211636566 | 0.267374966 | 0.403081614 | down |
| HSDL1 | -0.211509173 | 0.159149647 | 0.280313556 | down |
| ZNF316 | -0.211464895 | 0.13228689 | 0.246292826 | down |
| UBE2FP3 | -0.211339143 | 0.626306162 | 0.735373704 | down |
| MT1L | -0.211334773 | 0.483808654 | 0.612814477 | down |
| RP11-758P17.3 | -0.211273831 | 0.655889083 | 0.758517103 | down |
| C1orf220 | -0.211203818 | 0.670235215 | 0.77004003 | down |
| SLC25A3 | -0.21110448 | 0.053119951 | 0.127686433 | down |
| RP13-258O15.1 | -0.211095726 | 0.46696224 | 0.598065204 | down |
| LRRC55 | -0.211085659 | 0.608438471 | 0.72061834 | down |
| TMEM185A | -0.211019665 | 0.191575178 | 0.319336578 | down |
| PRRT3-AS1 | -0.210988479 | 0.571175946 | 0.68871647 | down |
| RP11-94H18.1 | -0.210972986 | 0.795614851 | 0.863948807 | down |
| WWOX | -0.210830848 | 0.21079676 | 0.341356567 | down |
| NEDD8 | -0.210819655 | 0.400538645 | 0.53751041 | down |
| PLCL2 | -0.210633485 | 0.190619167 | 0.318111551 | down |
| KCNIP1 | -0.210429416 | 0.662269663 | 0.763750566 | down |
| IFT27 | -0.210386689 | 0.217619319 | 0.349025711 | down |
| HAGH | -0.210310464 | 0.049509721 | 0.121455871 | down |
| ARL9 | -0.21018198 | 0.623784296 | 0.733380234 | down |
| TIGD2 | -0.210162432 | 0.438227745 | 0.572589126 | down |
| SNORA12 | -0.210152202 | 0.914177383 | | down |
| VIT | -0.210151349 | 0.568882538 | 0.68679255 | down |
| ANKRD20A18P | -0.210130774 | 0.888932684 | | down |
| HSP90AA4P | -0.210104756 | 0.864172786 | | down |
| DNAJA1P3 | -0.21010335 | 0.862960864 | | down |
| RP11-368J21.3 | -0.210098745 | 0.618417923 | 0.728989214 | down |
| RP11-49K24.8 | -0.210077925 | 0.84267828 | | down |
| RP3-415N12.1 | -0.210075047 | 0.840549242 | | down |
| AP001258.5 | -0.210073576 | 0.839472791 | | down |
| PXYLP1 | -0.20988066 | 0.218667419 | 0.350133937 | down |
| GMEB1 | -0.209877375 | 0.09881233 | 0.20031833 | down |
| RP11-468E2.11 | -0.209851184 | 0.370837945 | 0.50920802 | down |
| AC093159.1 | -0.209758902 | 0.769382413 | 0.844951025 | down |
| LINC00618 | -0.209726269 | 0.671243133 | 0.770870738 | down |
| RP1-40E16.12 | -0.209667658 | 0.60514192 | 0.717704242 | down |
| ZACN | -0.2095996 | 0.708682326 | 0.799820019 | down |
| RP11-710C12.1 | -0.209391097 | 0.52606605 | 0.649668218 | down |
| SEMA6A-AS1 | -0.209330957 | 0.616159009 | 0.727039559 | down |
| RP11-667K14.3 | -0.209289767 | 0.4094476 | 0.546173702 | down |
| RP4-796I17.5 | -0.209244855 | 0.757202399 | | down |
| IGBP1 | -0.209112429 | 0.031086558 | 0.086095551 | down |
| LHFPL1 | -0.208945033 | 0.759217213 | 0.837399389 | down |
| PITPNM1 | -0.208887413 | 0.102516668 | 0.205583665 | down |
| PGR | -0.208833795 | 0.622385011 | 0.732201286 | down |
| CTC-281F24.1 | -0.208653861 | 0.679562437 | 0.777776316 | down |
| AKR1A1 | -0.208557508 | 0.142527579 | 0.259489673 | down |
| DCAF8 | -0.208549106 | 0.194081251 | 0.322206836 | down |
| RFWD2 | -0.208475231 | 0.0801739 | 0.172504468 | down |
| CCDC173 | -0.208382895 | 0.663938481 | 0.765087747 | down |
| SLX4 | -0.208379124 | 0.430362889 | 0.565485355 | down |

| | | | | |
|---|---|---|---|---|
| LMO3 | -0.208372483 | 0.550096792 | 0.670498114 | down |
| RP11-528A4.2 | -0.208350422 | 0.71109337 | 0.801624299 | down |
| CPA5 | -0.20830844 | 0.724238092 | 0.811963924 | down |
| AC010504.2 | -0.207874018 | 0.684663189 | 0.781713948 | down |
| AC091729.9 | -0.207740716 | 0.366694991 | 0.505191425 | down |
| CX3CR1 | -0.207725605 | 0.648612079 | 0.752958418 | down |
| AP001610.5 | -0.20760329 | 0.834204411 | | down |
| CDIP1 | -0.207570828 | 0.126802971 | 0.238982317 | down |
| RP11-977G19.12 | -0.207477386 | 0.657151889 | 0.75939349 | down |
| ZNF587B | -0.207442214 | 0.203834943 | 0.333279839 | down |
| RP4-614C10.2 | -0.207325417 | 0.434269503 | 0.569075455 | down |
| PFN4 | -0.207193914 | 0.581430179 | 0.697413601 | down |
| CLASP2 | -0.20705042 | 0.29947862 | 0.436985705 | down |
| PCCA | -0.207028562 | 0.353867622 | 0.49254393 | down |
| U1 | -0.206909281 | 0.72942218 | 0.816070604 | down |
| EFL1 | -0.206825271 | 0.299745204 | 0.437310975 | down |
| BCKDK | -0.206816901 | 0.244000944 | 0.377932818 | down |
| TRIM25 | -0.206791789 | 0.106946534 | 0.21214193 | down |
| NCOA1 | -0.206777607 | 0.220786464 | 0.352441874 | down |
| ZNF789 | -0.206771488 | 0.282583363 | 0.419109646 | down |
| RP9P | -0.206702788 | 0.303750417 | 0.441747374 | down |
| ACSM1 | -0.206688161 | 0.556925199 | 0.676597683 | down |
| UQCRB | -0.206615499 | 0.165708343 | 0.288560914 | down |
| ABCA8 | -0.206601853 | 0.547047854 | 0.66792634 | down |
| COL6A5 | -0.20657919 | 0.668485249 | 0.768676165 | down |
| SCAMP3 | -0.206570959 | 0.210928983 | 0.341479281 | down |
| MAML3 | -0.20646441 | 0.299416712 | 0.436916592 | down |
| RPL23AP24 | -0.206461955 | 0.943744093 | | down |
| RNU7-179P | -0.206461955 | 0.943744093 | | down |
| RP1-8B22.2 | -0.206461955 | 0.943744093 | | down |
| MIR1256 | -0.206461955 | 0.943744093 | | down |
| PPP1R11P1 | -0.206461955 | 0.943744093 | | down |
| RP11-415K20.1 | -0.206461955 | 0.943744093 | | down |
| SCARNA18 | -0.206461955 | 0.943744093 | | down |
| RP4-785J1.1 | -0.206461955 | 0.943744093 | | down |
| RN7SKP91 | -0.206461955 | 0.943744093 | | down |
| NA | -0.206461955 | 0.943744093 | | down |
| RNU6-605P | -0.206461955 | 0.943744093 | | down |
| UBE2V1P8 | -0.206461955 | 0.943744093 | | down |
| RP3-393B19.1 | -0.206461955 | 0.943744093 | | down |
| AGBL4-IT1 | -0.206461955 | 0.943744093 | | down |
| RP4-657D16.6 | -0.206461955 | 0.943744093 | | down |
| MIR1273F | -0.206461955 | 0.943744093 | | down |
| MIR4422 | -0.206461955 | 0.943744093 | | down |
| PGBD4P8 | -0.206461955 | 0.943744093 | | down |
| RNA5SP49 | -0.206461955 | 0.943744093 | | down |
| RP11-510C10.4 | -0.206461955 | 0.943744093 | | down |
| RP11-375A5.1 | -0.206461955 | 0.943744093 | | down |
| RPL36AP10 | -0.206461955 | 0.943744093 | | down |
| RN7SL653P | -0.206461955 | 0.943744093 | | down |
| RP4-775D17.1 | -0.206461955 | 0.943744093 | | down |

| | | | |
|---|---|---|---|
| RNU6-970P | -0.206461955 | 0.943744093 | down |
| RP5-837021.2 | -0.206461955 | 0.943744093 | down |
| RP11-305E17.4 | -0.206461955 | 0.943744093 | down |
| RP11-84O12.2 | -0.206461955 | 0.943744093 | down |
| MRPL57P1 | -0.206461955 | 0.943744093 | down |
| NOTCH2P1 | -0.206461955 | 0.943744093 | down |
| RP11-763B22.7 | -0.206461955 | 0.943744093 | down |
| Y_RNA | -0.206461955 | 0.943744093 | down |
| RP11-301M17.2 | -0.206461955 | 0.943744093 | down |
| PMF1-BGLAP | -0.206461955 | 0.943744093 | down |
| RP11-85G21.1 | -0.206461955 | 0.943744093 | down |
| OR6K4P | -0.206461955 | 0.943744093 | down |
| RNU6-157P | -0.206461955 | 0.943744093 | down |
| RP5-1114G22.1 | -0.206461955 | 0.943744093 | down |
| PTP4A1P7 | -0.206461955 | 0.943744093 | down |
| NA | -0.206461955 | 0.943744093 | down |
| MIR488 | -0.206461955 | 0.943744093 | down |
| RNA5SP72 | -0.206461955 | 0.943744093 | down |
| NA | -0.206461955 | 0.943744093 | down |
| RP11-134C1.1 | -0.206461955 | 0.943744093 | down |
| HNRNPA1P46 | -0.206461955 | 0.943744093 | down |
| RP3-419C19.2 | -0.206461955 | 0.943744093 | down |
| MIR5191 | -0.206461955 | 0.943744093 | down |
| RP5-1051D14.1 | -0.206461955 | 0.943744093 | down |
| IPO8P1 | -0.206461955 | 0.943744093 | down |
| SNORA72 | -0.206461955 | 0.943744093 | down |
| NDUFA3P3 | -0.206461955 | 0.943744093 | down |
| RP11-261C10.4 | -0.206461955 | 0.943744093 | down |
| Y_RNA | -0.206461955 | 0.943744093 | down |
| Y_RNA | -0.206461955 | 0.943744093 | down |
| OR2M5 | -0.206461955 | 0.943744093 | down |
| OR2AS2P | -0.206461955 | 0.943744093 | down |
| RN7SKP112 | -0.206461955 | 0.943744093 | down |
| AC011897.2 | -0.206461955 | 0.943744093 | down |
| CDKN2AIPNLP2 | -0.206461955 | 0.943744093 | down |
| NA | -0.206461955 | 0.943744093 | down |
| MIR558 | -0.206461955 | 0.943744093 | down |
| ATP6V0E1P3 | -0.206461955 | 0.943744093 | down |
| Y_RNA | -0.206461955 | 0.943744093 | down |
| RP11-89K21.2 | -0.206461955 | 0.943744093 | down |
| NA | -0.206461955 | 0.943744093 | down |
| MIR8485 | -0.206461955 | 0.943744093 | down |
| Y_RNA | -0.206461955 | 0.943744093 | down |
| RP11-474G23.1 | -0.206461955 | 0.943744093 | down |
| TCEB1P21 | -0.206461955 | 0.943744093 | down |
| MRPL53 | -0.206461955 | 0.943744093 | down |
| AC092660.1 | -0.206461955 | 0.943744093 | down |
| AC013262.1 | -0.206461955 | 0.943744093 | down |
| AC106874.1 | -0.206461955 | 0.943744093 | down |
| IGKV3D-25 | -0.206461955 | 0.943744093 | down |
| AC073464.7 | -0.206461955 | 0.943744093 | down |

| | | | |
|---|---|---|---|
| AC092570.4 | -0.206461955 | 0.943744093 | down |
| AC093166.2 | -0.206461955 | 0.943744093 | down |
| NA | -0.206461955 | 0.943744093 | down |
| SEPHS1P7 | -0.206461955 | 0.943744093 | down |
| DPP10-AS2 | -0.206461955 | 0.943744093 | down |
| RP11-98C1.2 | -0.206461955 | 0.943744093 | down |
| AC013399.3 | -0.206461955 | 0.943744093 | down |
| Y_RNA | -0.206461955 | 0.943744093 | down |
| RNU6-675P | -0.206461955 | 0.943744093 | down |
| MTATP6P4 | -0.206461955 | 0.943744093 | down |
| RNU6-617P | -0.206461955 | 0.943744093 | down |
| AC110620.1 | -0.206461955 | 0.943744093 | down |
| RNU6-512P | -0.206461955 | 0.943744093 | down |
| Y_RNA | -0.206461955 | 0.943744093 | down |
| AC114763.1 | -0.206461955 | 0.943744093 | down |
| AC117947.1 | -0.206461955 | 0.943744093 | down |
| NA | -0.206461955 | 0.943744093 | down |
| RPSAP25 | -0.206461955 | 0.943744093 | down |
| RNU6-629P | -0.206461955 | 0.943744093 | down |
| AC019178.3 | -0.206461955 | 0.943744093 | down |
| Y_RNA | -0.206461955 | 0.943744093 | down |
| Y_RNA | -0.206461955 | 0.943744093 | down |
| TPT1P2 | -0.206461955 | 0.943744093 | down |
| AC013404.1 | -0.206461955 | 0.943744093 | down |
| AC122136.2 | -0.206461955 | 0.943744093 | down |
| AC009955.8 | -0.206461955 | 0.943744093 | down |
| AC009310.1 | -0.206461955 | 0.943744093 | down |
| NA | -0.206461955 | 0.943744093 | down |
| ECEL1P3 | -0.206461955 | 0.943744093 | down |
| UGT1A11P | -0.206461955 | 0.943744093 | down |
| RP11-315F22.1 | -0.206461955 | 0.943744093 | down |
| RP11-118M12.1 | -0.206461955 | 0.943744093 | down |
| RNU6-1194P | -0.206461955 | 0.943744093 | down |
| RNA5SP123 | -0.206461955 | 0.943744093 | down |
| AC027124.2 | -0.206461955 | 0.943744093 | down |
| HDAC11-AS1 | -0.206461955 | 0.943744093 | down |
| RNU6-815P | -0.206461955 | 0.943744093 | down |
| RNU6-788P | -0.206461955 | 0.943744093 | down |
| AC099535.4 | -0.206461955 | 0.943744093 | down |
| AC098614.1 | -0.206461955 | 0.943744093 | down |
| AC104306.4 | -0.206461955 | 0.943744093 | down |
| AC104308.2 | -0.206461955 | 0.943744093 | down |
| EIF4BP4 | -0.206461955 | 0.943744093 | down |
| U3 | -0.206461955 | 0.943744093 | down |
| uc_338 | -0.206461955 | 0.943744093 | down |
| AMIGO3 | -0.206461955 | 0.943744093 | down |
| U3 | -0.206461955 | 0.943744093 | down |
| RP11-154D3.1 | -0.206461955 | 0.943744093 | down |
| RNU7-119P | -0.206461955 | 0.943744093 | down |
| RP11-520D19.1 | -0.206461955 | 0.943744093 | down |
| NA | -0.206461955 | 0.943744093 | down |

| | | | |
|---|---|---|---|
| NDUFA5P5 | -0.206461955 | 0.943744093 | down |
| OR5AC1 | -0.206461955 | 0.943744093 | down |
| OR5AC4P | -0.206461955 | 0.943744093 | down |
| OR5H3P | -0.206461955 | 0.943744093 | down |
| Y_RNA | -0.206461955 | 0.943744093 | down |
| Y_RNA | -0.206461955 | 0.943744093 | down |
| NDUFA4P2 | -0.206461955 | 0.943744093 | down |
| MIR5682 | -0.206461955 | 0.943744093 | down |
| RP11-592P9.3 | -0.206461955 | 0.943744093 | down |
| Y_RNA | -0.206461955 | 0.943744093 | down |
| RNA5SP139 | -0.206461955 | 0.943744093 | down |
| RNU6-726P | -0.206461955 | 0.943744093 | down |
| RP11-305O4.2 | -0.206461955 | 0.943744093 | down |
| RP11-312P21.2 | -0.206461955 | 0.943744093 | down |
| ATP5LP5 | -0.206461955 | 0.943744093 | down |
| RPL9P15 | -0.206461955 | 0.943744093 | down |
| RP11-674E16.4 | -0.206461955 | 0.943744093 | down |
| RP11-6F2.3 | -0.206461955 | 0.943744093 | down |
| RNA5SP146 | -0.206461955 | 0.943744093 | down |
| RP11-538P18.1 | -0.206461955 | 0.943744093 | down |
| MIR551B | -0.206461955 | 0.943744093 | down |
| RNU4-38P | -0.206461955 | 0.943744093 | down |
| MIR6828 | -0.206461955 | 0.943744093 | down |
| RP11-373E16.1 | -0.206461955 | 0.943744093 | down |
| MIR569 | -0.206461955 | 0.943744093 | down |
| Y_RNA | -0.206461955 | 0.943744093 | down |
| RP11-145M9.2 | -0.206461955 | 0.943744093 | down |
| FAUP2 | -0.206461955 | 0.943744093 | down |
| RP11-338L18.1 | -0.206461955 | 0.943744093 | down |
| EHHADH-AS1 | -0.206461955 | 0.943744093 | down |
| SNORA48 | -0.206461955 | 0.943744093 | down |
| RP11-747H12.1 | -0.206461955 | 0.943744093 | down |
| RP11-747H12.4 | -0.206461955 | 0.943744093 | down |
| AC006499.5 | -0.206461955 | 0.943744093 | down |
| RP11-141E13.1 | -0.206461955 | 0.943744093 | down |
| RP11-556G22.3 | -0.206461955 | 0.943744093 | down |
| RNA5SP158 | -0.206461955 | 0.943744093 | down |
| MIR5591 | -0.206461955 | 0.943744093 | down |
| PRDX4P1 | -0.206461955 | 0.943744093 | down |
| RP11-362I1.1 | -0.206461955 | 0.943744093 | down |
| RP11-1281K21.10 | -0.206461955 | 0.943744093 | down |
| RP11-633G11.1 | -0.206461955 | 0.943744093 | down |
| TMPRSS11A | -0.206461955 | 0.943744093 | down |
| TMPRSS11F | -0.206461955 | 0.943744093 | down |
| RP11-598G2.1 | -0.206461955 | 0.943744093 | down |
| RP11-562F9.2 | -0.206461955 | 0.943744093 | down |
| RP11-428L21.2 | -0.206461955 | 0.943744093 | down |
| RP11-554D13.1 | -0.206461955 | 0.943744093 | down |
| RP11-1299A16.1 | -0.206461955 | 0.943744093 | down |
| NDUFS5P4 | -0.206461955 | 0.943744093 | down |
| AC004069.1 | -0.206461955 | 0.943744093 | down |

| | | | |
|---|---|---|---|
| EXOC7P1 | -0.206461955 | 0.943744093 | down |
| TUBB4BP5 | -0.206461955 | 0.943744093 | down |
| RP11-728C8.1 | -0.206461955 | 0.943744093 | down |
| RP11-209D20.2 | -0.206461955 | 0.943744093 | down |
| RP11-404I7.2 | -0.206461955 | 0.943744093 | down |
| PES1P1 | -0.206461955 | 0.943744093 | down |
| CTD-2012I17.1 | -0.206461955 | 0.943744093 | down |
| RP11-782E2.2 | -0.206461955 | 0.943744093 | down |
| RP11-625B21.1 | -0.206461955 | 0.943744093 | down |
| RP11-793B23.1 | -0.206461955 | 0.943744093 | down |
| MTND1P22 | -0.206461955 | 0.943744093 | down |
| RP11-612J15.3 | -0.206461955 | 0.943744093 | down |
| Y_RNA | -0.206461955 | 0.943744093 | down |
| RP11-597D13.1 | -0.206461955 | 0.943744093 | down |
| RP11-344G13.1 | -0.206461955 | 0.943744093 | down |
| RP11-344G13.2 | -0.206461955 | 0.943744093 | down |
| NA | -0.206461955 | 0.943744093 | down |
| CCNHP1 | -0.206461955 | 0.943744093 | down |
| RP11-386B13.1 | -0.206461955 | 0.943744093 | down |
| DUX4 | -0.206461955 | 0.943744093 | down |
| CTD-2194D22.2 | -0.206461955 | 0.943744093 | down |
| CTD-2287N17.1 | -0.206461955 | 0.943744093 | down |
| CTD-2008N3.1 | -0.206461955 | 0.943744093 | down |
| RP11-404K5.4 | -0.206461955 | 0.943744093 | down |
| RP11-315A16.3 | -0.206461955 | 0.943744093 | down |
| RNU6-429P | -0.206461955 | 0.943744093 | down |
| CTD-2313D3.1 | -0.206461955 | 0.943744093 | down |
| NA | -0.206461955 | 0.943744093 | down |
| RP11-432M8.10 | -0.206461955 | 0.943744093 | down |
| RP11-432M8.14 | -0.206461955 | 0.943744093 | down |
| RP11-432M8.18 | -0.206461955 | 0.943744093 | down |
| RP11-124N3.2 | -0.206461955 | 0.943744093 | down |
| CTD-2333M24.1 | -0.206461955 | 0.943744093 | down |
| Y_RNA | -0.206461955 | 0.943744093 | down |
| CTD-2533K21.4 | -0.206461955 | 0.943744093 | down |
| CTD-2269E23.3 | -0.206461955 | 0.943744093 | down |
| SUCLG2P4 | -0.206461955 | 0.943744093 | down |
| SNORA18 | -0.206461955 | 0.943744093 | down |
| HPRT1P2 | -0.206461955 | 0.943744093 | down |
| RP11-1084J3.3 | -0.206461955 | 0.943744093 | down |
| RP11-447H19.2 | -0.206461955 | 0.943744093 | down |
| RP11-287J9.1 | -0.206461955 | 0.943744093 | down |
| RP11-434D9.2 | -0.206461955 | 0.943744093 | down |
| RP11-232L2.1 | -0.206461955 | 0.943744093 | down |
| Y_RNA | -0.206461955 | 0.943744093 | down |
| CTD-2218K11.2 | -0.206461955 | 0.943744093 | down |
| RP11-1072N2.4 | -0.206461955 | 0.943744093 | down |
| CTC-498M16.4 | -0.206461955 | 0.943744093 | down |
| RP11-61G23.2 | -0.206461955 | 0.943744093 | down |
| CTC-458G6.2 | -0.206461955 | 0.943744093 | down |
| MRPS35P2 | -0.206461955 | 0.943744093 | down |

| | | | |
|---|---|---|---|
| MTCYBP22 | -0.206461955 | 0.943744093 | down |
| RNU6-47P | -0.206461955 | 0.943744093 | down |
| RP11-231G15.1 | -0.206461955 | 0.943744093 | down |
| CTC-313D10.1 | -0.206461955 | 0.943744093 | down |
| AK3P4 | -0.206461955 | 0.943744093 | down |
| FABP5P6 | -0.206461955 | 0.943744093 | down |
| RNU6-752P | -0.206461955 | 0.943744093 | down |
| MRPS5P3 | -0.206461955 | 0.943744093 | down |
| CTB-88F18.4 | -0.206461955 | 0.943744093 | down |
| MIR4633 | -0.206461955 | 0.943744093 | down |
| RP11-114H7.3 | -0.206461955 | 0.943744093 | down |
| MEIKIN | -0.206461955 | 0.943744093 | down |
| MIR6830 | -0.206461955 | 0.943744093 | down |
| AC011431.1 | -0.206461955 | 0.943744093 | down |
| RNU6-888P | -0.206461955 | 0.943744093 | down |
| MIR5197 | -0.206461955 | 0.943744093 | down |
| CTC-359M8.3 | -0.206461955 | 0.943744093 | down |
| RN7SL868P | -0.206461955 | 0.943744093 | down |
| RNA5SP197 | -0.206461955 | 0.943744093 | down |
| MIR3141 | -0.206461955 | 0.943744093 | down |
| CTC-280C13.1 | -0.206461955 | 0.943744093 | down |
| CTD-2313P7.1 | -0.206461955 | 0.943744093 | down |
| SNORA40 | -0.206461955 | 0.943744093 | down |
| CTB-79E8.4 | -0.206461955 | 0.943744093 | down |
| CTB-14A14.1 | -0.206461955 | 0.943744093 | down |
| RP1-90J20.14 | -0.206461955 | 0.943744093 | down |
| RNU6-793P | -0.206461955 | 0.943744093 | down |
| UQCRFS1P3 | -0.206461955 | 0.943744093 | down |
| HNRNPA1P58 | -0.206461955 | 0.943744093 | down |
| HIST1H2BA | -0.206461955 | 0.943744093 | down |
| HIST1H4G | -0.206461955 | 0.943744093 | down |
| OR2AD1P | -0.206461955 | 0.943744093 | down |
| OR2P1P | -0.206461955 | 0.943744093 | down |
| Y_RNA | -0.206461955 | 0.943744093 | down |
| RP11-552E20.1 | -0.206461955 | 0.943744093 | down |
| DEFB113 | -0.206461955 | 0.943744093 | down |
| RP4-753D5.3 | -0.206461955 | 0.943744093 | down |
| SLC25A20P1 | -0.206461955 | 0.943744093 | down |
| RPL31P33 | -0.206461955 | 0.943744093 | down |
| ERHP2 | -0.206461955 | 0.943744093 | down |
| RP11-160A9.3 | -0.206461955 | 0.943744093 | down |
| RP5-1046G13.2 | -0.206461955 | 0.943744093 | down |
| RP11-554D15.2 | -0.206461955 | 0.943744093 | down |
| NA | -0.206461955 | 0.943744093 | down |
| RP11-35J1.2 | -0.206461955 | 0.943744093 | down |
| MIR4464 | -0.206461955 | 0.943744093 | down |
| COPS5P1 | -0.206461955 | 0.943744093 | down |
| RP11-793L10.1 | -0.206461955 | 0.943744093 | down |
| RNU6-117P | -0.206461955 | 0.943744093 | down |
| MIR587 | -0.206461955 | 0.943744093 | down |
| RNU6-1299P | -0.206461955 | 0.943744093 | down |

| | | | |
|---|---|---|---|
| FEM1AP3 | -0.206461955 | 0.943744093 | down |
| RP1-249H1.3 | -0.206461955 | 0.943744093 | down |
| Y_RNA | -0.206461955 | 0.943744093 | down |
| RPL35AP3 | -0.206461955 | 0.943744093 | down |
| RP11-137J7.2 | -0.206461955 | 0.943744093 | down |
| RP1-12G14.5 | -0.206461955 | 0.943744093 | down |
| RNU6-813P | -0.206461955 | 0.943744093 | down |
| RP11-306O13.1 | -0.206461955 | 0.943744093 | down |
| RNA5SP225 | -0.206461955 | 0.943744093 | down |
| RP11-52J3.3 | -0.206461955 | 0.943744093 | down |
| RP11-317M22.1 | -0.206461955 | 0.943744093 | down |
| RP3-495K2.1 | -0.206461955 | 0.943744093 | down |
| RPSAP73 | -0.206461955 | 0.943744093 | down |
| MIR3683 | -0.206461955 | 0.943744093 | down |
| NA | -0.206461955 | 0.943744093 | down |
| SNORD65 | -0.206461955 | 0.943744093 | down |
| HMGB3P20 | -0.206461955 | 0.943744093 | down |
| RPL35P4 | -0.206461955 | 0.943744093 | down |
| NA | -0.206461955 | 0.943744093 | down |
| RP11-239C9.1 | -0.206461955 | 0.943744093 | down |
| AC007327.6 | -0.206461955 | 0.943744093 | down |
| AC005160.3 | -0.206461955 | 0.943744093 | down |
| AC004938.5 | -0.206461955 | 0.943744093 | down |
| RP11-52M17.1 | -0.206461955 | 0.943744093 | down |
| RP11-111G20.3 | -0.206461955 | 0.943744093 | down |
| NA | -0.206461955 | 0.943744093 | down |
| RP11-310H4.6 | -0.206461955 | 0.943744093 | down |
| NA | -0.206461955 | 0.943744093 | down |
| NA | -0.206461955 | 0.943744093 | down |
| RP11-1217F2.17 | -0.206461955 | 0.943744093 | down |
| RP11-1217F2.18 | -0.206461955 | 0.943744093 | down |
| RP11-368M16.6 | -0.206461955 | 0.943744093 | down |
| SEPT7P4 | -0.206461955 | 0.943744093 | down |
| SAPCD2P1 | -0.206461955 | 0.943744093 | down |
| RP11-328P23.2 | -0.206461955 | 0.943744093 | down |
| RP4-736H5.3 | -0.206461955 | 0.943744093 | down |
| Y_RNA | -0.206461955 | 0.943744093 | down |
| AC004945.1 | -0.206461955 | 0.943744093 | down |
| RN7SL869P | -0.206461955 | 0.943744093 | down |
| DYNLL1P7 | -0.206461955 | 0.943744093 | down |
| SOCS5P1 | -0.206461955 | 0.943744093 | down |
| C7orf62 | -0.206461955 | 0.943744093 | down |
| GS1-306C12.1 | -0.206461955 | 0.943744093 | down |
| AP1S2P1 | -0.206461955 | 0.943744093 | down |
| CTB-152G17.5 | -0.206461955 | 0.943744093 | down |
| RP11-458K10.2 | -0.206461955 | 0.943744093 | down |
| RP4-745K6.1 | -0.206461955 | 0.943744093 | down |
| RP11-332K15.1 | -0.206461955 | 0.943744093 | down |
| AC000099.1 | -0.206461955 | 0.943744093 | down |
| Y_RNA | -0.206461955 | 0.943744093 | down |
| AC078842.4 | -0.206461955 | 0.943744093 | down |

| | | | |
|---|---|---|---|
| RNY4 | -0.206461955 | 0.943744093 | down |
| RP11-476H24.1 | -0.206461955 | 0.943744093 | down |
| AC008060.5 | -0.206461955 | 0.943744093 | down |
| AC006003.3 | -0.206461955 | 0.943744093 | down |
| MIR595 | -0.206461955 | 0.943744093 | down |
| SEPT14P8 | -0.206461955 | 0.943744093 | down |
| RP11-1049H7.2 | -0.206461955 | 0.943744093 | down |
| OR7E157P | -0.206461955 | 0.943744093 | down |
| SNORA70 | -0.206461955 | 0.943744093 | down |
| RP11-1236K1.8 | -0.206461955 | 0.943744093 | down |
| OR7E8P | -0.206461955 | 0.943744093 | down |
| RP11-3G21.1 | -0.206461955 | 0.943744093 | down |
| MTND4LP26 | -0.206461955 | 0.943744093 | down |
| RP11-108A14.2 | -0.206461955 | 0.943744093 | down |
| RP11-618M23.2 | -0.206461955 | 0.943744093 | down |
| TMEM97P2 | -0.206461955 | 0.943744093 | down |
| MTND1P6 | -0.206461955 | 0.943744093 | down |
| MTCYBP19 | -0.206461955 | 0.943744093 | down |
| RP11-465K16.1 | -0.206461955 | 0.943744093 | down |
| RP11-726G23.11 | -0.206461955 | 0.943744093 | down |
| RP11-401N18.1 | -0.206461955 | 0.943744093 | down |
| RP11-759A9.1 | -0.206461955 | 0.943744093 | down |
| CYCSP22 | -0.206461955 | 0.943744093 | down |
| RP11-463C14.1 | -0.206461955 | 0.943744093 | down |
| SLC2A13P1 | -0.206461955 | 0.943744093 | down |
| RNA5SP269 | -0.206461955 | 0.943744093 | down |
| RP11-159H10.1 | -0.206461955 | 0.943744093 | down |
| HIGD1AP6 | -0.206461955 | 0.943744093 | down |
| ACTBP6 | -0.206461955 | 0.943744093 | down |
| KB-1589B1.4 | -0.206461955 | 0.943744093 | down |
| KB-1562D12.2 | -0.206461955 | 0.943744093 | down |
| ADI1P2 | -0.206461955 | 0.943744093 | down |
| RP11-778D12.2 | -0.206461955 | 0.943744093 | down |
| RP11-393K19.1 | -0.206461955 | 0.943744093 | down |
| RP11-973F15.2 | -0.206461955 | 0.943744093 | down |
| RPL23AP56 | -0.206461955 | 0.943744093 | down |
| RP11-279E1.1 | -0.206461955 | 0.943744093 | down |
| RP11-272P10.3 | -0.206461955 | 0.943744093 | down |
| RNU6-246P | -0.206461955 | 0.943744093 | down |
| RP11-570H19.2 | -0.206461955 | 0.943744093 | down |
| MIR3152 | -0.206461955 | 0.943744093 | down |
| Y_RNA | -0.206461955 | 0.943744093 | down |
| IFNA13 | -0.206461955 | 0.943744093 | down |
| SNORD39 | -0.206461955 | 0.943744093 | down |
| RN7SKP120 | -0.206461955 | 0.943744093 | down |
| RP11-373A6.1 | -0.206461955 | 0.943744093 | down |
| LINC01243 | -0.206461955 | 0.943744093 | down |
| NDUFA5P4 | -0.206461955 | 0.943744093 | down |
| SNX18P8 | -0.206461955 | 0.943744093 | down |
| RP11-157L3.11 | -0.206461955 | 0.943744093 | down |
| RNA5SP283 | -0.206461955 | 0.943744093 | down |

| | | | |
|---|---|---|---|
| PTGER4P3 | -0.206461955 | 0.943744093 | down |
| ATP5A1P10 | -0.206461955 | 0.943744093 | down |
| AL359314.1 | -0.206461955 | 0.943744093 | down |
| RN7SKP59 | -0.206461955 | 0.943744093 | down |
| RP11-345K9.3 | -0.206461955 | 0.943744093 | down |
| RP11-547C13.1 | -0.206461955 | 0.943744093 | down |
| TRMT112P4 | -0.206461955 | 0.943744093 | down |
| RNU6-432P | -0.206461955 | 0.943744093 | down |
| RP11-85O21.5 | -0.206461955 | 0.943744093 | down |
| RP11-123K19.2 | -0.206461955 | 0.943744093 | down |
| MIR3689C | -0.206461955 | 0.943744093 | down |
| MIR3689F | -0.206461955 | 0.943744093 | down |
| Y_RNA | -0.206461955 | 0.943744093 | down |
| RP11-139J15.5 | -0.206461955 | 0.943744093 | down |
| RP11-51E20.1 | -0.206461955 | 0.943744093 | down |
| RNA5SP305 | -0.206461955 | 0.943744093 | down |
| RNU2-24P | -0.206461955 | 0.943744093 | down |
| RNU6-1244P | -0.206461955 | 0.943744093 | down |
| RP11-8L18.3 | -0.206461955 | 0.943744093 | down |
| SS18L2P1 | -0.206461955 | 0.943744093 | down |
| RP11-508N22.11 | -0.206461955 | 0.943744093 | down |
| KSR1P1 | -0.206461955 | 0.943744093 | down |
| RP11-71J2.1 | -0.206461955 | 0.943744093 | down |
| RP11-388P9.3 | -0.206461955 | 0.943744093 | down |
| MTND1P20 | -0.206461955 | 0.943744093 | down |
| SNORA71 | -0.206461955 | 0.943744093 | down |
| RP11-20E23.1 | -0.206461955 | 0.943744093 | down |
| RP11-93H12.3 | -0.206461955 | 0.943744093 | down |
| RP11-57C13.4 | -0.206461955 | 0.943744093 | down |
| Y_RNA | -0.206461955 | 0.943744093 | down |
| RP11-236B18.2 | -0.206461955 | 0.943744093 | down |
| CPEB3_ribozyme | -0.206461955 | 0.943744093 | down |
| MIR609 | -0.206461955 | 0.943744093 | down |
| PTGES3P5 | -0.206461955 | 0.943744093 | down |
| MIR548E | -0.206461955 | 0.943744093 | down |
| RN7SL384P | -0.206461955 | 0.943744093 | down |
| TAF9BP2 | -0.206461955 | 0.943744093 | down |
| GNG10P1 | -0.206461955 | 0.943744093 | down |
| FANK1-AS1 | -0.206461955 | 0.943744093 | down |
| RNU6-1025P | -0.206461955 | 0.943744093 | down |
| AP006285.6 | -0.206461955 | 0.943744093 | down |
| INS | -0.206461955 | 0.943744093 | down |
| Y_RNA | -0.206461955 | 0.943744093 | down |
| OR51G2 | -0.206461955 | 0.943744093 | down |
| OR51G1 | -0.206461955 | 0.943744093 | down |
| HIGD1AP5 | -0.206461955 | 0.943744093 | down |
| RP11-1081L13.5 | -0.206461955 | 0.943744093 | down |
| RP11-583F24.3 | -0.206461955 | 0.943744093 | down |
| EIF4A2P5 | -0.206461955 | 0.943744093 | down |
| RP1-130L23.1 | -0.206461955 | 0.943744093 | down |
| RP4-743O11.1 | -0.206461955 | 0.943744093 | down |

| | | | |
|---|---|---|---|
| KRT18P14 | -0.206461955 | 0.943744093 | down |
| RP11-514F3.4 | -0.206461955 | 0.943744093 | down |
| RP11-111A24.1 | -0.206461955 | 0.943744093 | down |
| GTF2IP11 | -0.206461955 | 0.943744093 | down |
| OR4A3P | -0.206461955 | 0.943744093 | down |
| OR4A9P | -0.206461955 | 0.943744093 | down |
| OR4A50P | -0.206461955 | 0.943744093 | down |
| OR4C16 | -0.206461955 | 0.943744093 | down |
| OR5L2 | -0.206461955 | 0.943744093 | down |
| OR8I2 | -0.206461955 | 0.943744093 | down |
| OR8H3 | -0.206461955 | 0.943744093 | down |
| OR5M12P | -0.206461955 | 0.943744093 | down |
| OR2AH1P | -0.206461955 | 0.943744093 | down |
| OR5G1P | -0.206461955 | 0.943744093 | down |
| OR5BL1P | -0.206461955 | 0.943744093 | down |
| MS4A5 | -0.206461955 | 0.943744093 | down |
| AP000439.5 | -0.206461955 | 0.943744093 | down |
| ART2P | -0.206461955 | 0.943744093 | down |
| CTD-2530H12.7 | -0.206461955 | 0.943744093 | down |
| MIR5579 | -0.206461955 | 0.943744093 | down |
| DLG2-AS1_1 | -0.206461955 | 0.943744093 | down |
| RP11-882I15.1 | -0.206461955 | 0.943744093 | down |
| PTP4A1P6 | -0.206461955 | 0.943744093 | down |
| RP11-164N3.1 | -0.206461955 | 0.943744093 | down |
| UBTFL10 | -0.206461955 | 0.943744093 | down |
| RP11-313I2.13 | -0.206461955 | 0.943744093 | down |
| RP11-313I2.8 | -0.206461955 | 0.943744093 | down |
| RP11-81M19.1 | -0.206461955 | 0.943744093 | down |
| TRIM64 | -0.206461955 | 0.943744093 | down |
| TUBB4BP4 | -0.206461955 | 0.943744093 | down |
| JRKL-AS1 | -0.206461955 | 0.943744093 | down |
| RP11-49K4.2 | -0.206461955 | 0.943744093 | down |
| RP11-99C10.1 | -0.206461955 | 0.943744093 | down |
| AP000619.6 | -0.206461955 | 0.943744093 | down |
| RP11-681H10.1 | -0.206461955 | 0.943744093 | down |
| AP000641.1 | -0.206461955 | 0.943744093 | down |
| GNG5P3 | -0.206461955 | 0.943744093 | down |
| AP000770.1 | -0.206461955 | 0.943744093 | down |
| RNU6-376P | -0.206461955 | 0.943744093 | down |
| RP11-158I9.1 | -0.206461955 | 0.943744093 | down |
| ACA64 | -0.206461955 | 0.943744093 | down |
| RP11-109E10.1 | -0.206461955 | 0.943744093 | down |
| SLC5A4P1 | -0.206461955 | 0.943744093 | down |
| RP11-476M19.3 | -0.206461955 | 0.943744093 | down |
| SNORA75 | -0.206461955 | 0.943744093 | down |
| RP11-705C15.5 | -0.206461955 | 0.943744093 | down |
| TAS2R7 | -0.206461955 | 0.943744093 | down |
| RP11-434C1.4 | -0.206461955 | 0.943744093 | down |
| RNU6-318P | -0.206461955 | 0.943744093 | down |
| RP11-72J9.1 | -0.206461955 | 0.943744093 | down |
| RN7SKP134 | -0.206461955 | 0.943744093 | down |

| | | | |
|---|---|---|---|
| RP11-233G1.4 | -0.206461955 | 0.943744093 | down |
| RP11-268P4.2 | -0.206461955 | 0.943744093 | down |
| RP11-153K16.2 | -0.206461955 | 0.943744093 | down |
| RN7SKP262 | -0.206461955 | 0.943744093 | down |
| RP11-77I22.4 | -0.206461955 | 0.943744093 | down |
| RP11-367O10.1 | -0.206461955 | 0.943744093 | down |
| ZNF75BP | -0.206461955 | 0.943744093 | down |
| RP11-474C8.3 | -0.206461955 | 0.943744093 | down |
| RP11-133N21.12 | -0.206461955 | 0.943744093 | down |
| OR6C7P | -0.206461955 | 0.943744093 | down |
| OR6C71P | -0.206461955 | 0.943744093 | down |
| OR6C76 | -0.206461955 | 0.943744093 | down |
| RP11-348M3.6 | -0.206461955 | 0.943744093 | down |
| SNORA70 | -0.206461955 | 0.943744093 | down |
| RP11-453D16.2 | -0.206461955 | 0.943744093 | down |
| RPL7P43 | -0.206461955 | 0.943744093 | down |
| RP11-136F16.2 | -0.206461955 | 0.943744093 | down |
| RP11-228G3.1 | -0.206461955 | 0.943744093 | down |
| RP11-248E9.4 | -0.206461955 | 0.943744093 | down |
| RP11-13A1.2 | -0.206461955 | 0.943744093 | down |
| snoMe28S-Am2634 | -0.206461955 | 0.943744093 | down |
| NA | -0.206461955 | 0.943744093 | down |
| Y_RNA | -0.206461955 | 0.943744093 | down |
| RNU5E-5P | -0.206461955 | 0.943744093 | down |
| RP11-650K20.2 | -0.206461955 | 0.943744093 | down |
| SNORD74 | -0.206461955 | 0.943744093 | down |
| GLULP5 | -0.206461955 | 0.943744093 | down |
| RP11-324E6.4 | -0.206461955 | 0.943744093 | down |
| RP11-407A16.8 | -0.206461955 | 0.943744093 | down |
| RP11-407A16.4 | -0.206461955 | 0.943744093 | down |
| RNU1-104P | -0.206461955 | 0.943744093 | down |
| RP11-553N19.1 | -0.206461955 | 0.943744093 | down |
| RP11-474D1.4 | -0.206461955 | 0.943744093 | down |
| RP11-386I8.6 | -0.206461955 | 0.943744093 | down |
| RP11-341D18.6 | -0.206461955 | 0.943744093 | down |
| LONRF2P2 | -0.206461955 | 0.943744093 | down |
| RN7SL166P | -0.206461955 | 0.943744093 | down |
| RN7SL766P | -0.206461955 | 0.943744093 | down |
| MTCO3P2 | -0.206461955 | 0.943744093 | down |
| C1QTNF9-AS1 | -0.206461955 | 0.943744093 | down |
| RNU6-73P | -0.206461955 | 0.943744093 | down |
| KATNBL1P1 | -0.206461955 | 0.943744093 | down |
| Y_RNA | -0.206461955 | 0.943744093 | down |
| RNU6-71P | -0.206461955 | 0.943744093 | down |
| LINC00437 | -0.206461955 | 0.943744093 | down |
| RN7SKP5 | -0.206461955 | 0.943744093 | down |
| RN7SL618P | -0.206461955 | 0.943744093 | down |
| RP11-490P13.2 | -0.206461955 | 0.943744093 | down |
| RNU6-80P | -0.206461955 | 0.943744093 | down |
| RNY1P5 | -0.206461955 | 0.943744093 | down |
| RNU6-38P | -0.206461955 | 0.943744093 | down |

| | | | |
|---|---|---|---|
| RP11-30L8.1 | -0.206461955 | 0.943744093 | down |
| SNORD38 | -0.206461955 | 0.943744093 | down |
| RNU6-75P | -0.206461955 | 0.943744093 | down |
| LINC01039 | -0.206461955 | 0.943744093 | down |
| MIR4705 | -0.206461955 | 0.943744093 | down |
| LINC00370 | -0.206461955 | 0.943744093 | down |
| NF1P11 | -0.206461955 | 0.943744093 | down |
| OR4K3 | -0.206461955 | 0.943744093 | down |
| OR11G1P | -0.206461955 | 0.943744093 | down |
| AE000660.21 | -0.206461955 | 0.943744093 | down |
| TRAJ59 | -0.206461955 | 0.943744093 | down |
| RN7SKP205 | -0.206461955 | 0.943744093 | down |
| OR7K1P | -0.206461955 | 0.943744093 | down |
| CTD-2591A6.2 | -0.206461955 | 0.943744093 | down |
| RP11-966I7.2 | -0.206461955 | 0.943744093 | down |
| RNU11-5P | -0.206461955 | 0.943744093 | down |
| RNU2-14P | -0.206461955 | 0.943744093 | down |
| RNU6-659P | -0.206461955 | 0.943744093 | down |
| RP11-270M14.5 | -0.206461955 | 0.943744093 | down |
| EEF1A1P2 | -0.206461955 | 0.943744093 | down |
| RP11-371E8.2 | -0.206461955 | 0.943744093 | down |
| RP11-404P21.6 | -0.206461955 | 0.943744093 | down |
| PEBP1P1 | -0.206461955 | 0.943744093 | down |
| C14orf177 | -0.206461955 | 0.943744093 | down |
| RP11-1127D7.2 | -0.206461955 | 0.943744093 | down |
| SNORD113-9 | -0.206461955 | 0.943744093 | down |
| RP11-1017G21.4 | -0.206461955 | 0.943744093 | down |
| IGHVII-15-1 | -0.206461955 | 0.943744093 | down |
| IGHV3-76 | -0.206461955 | 0.943744093 | down |
| NA | -0.206461955 | 0.943744093 | down |
| RP11-810K23.6 | -0.206461955 | 0.943744093 | down |
| GRAMD4P6 | -0.206461955 | 0.943744093 | down |
| RP11-467N20.8 | -0.206461955 | 0.943744093 | down |
| AC023449.2 | -0.206461955 | 0.943744093 | down |
| ABCB10P4 | -0.206461955 | 0.943744093 | down |
| DNM1P32 | -0.206461955 | 0.943744093 | down |
| RP11-1H8.6 | -0.206461955 | 0.943744093 | down |
| H3F3AP1 | -0.206461955 | 0.943744093 | down |
| RP11-129I12.3 | -0.206461955 | 0.943744093 | down |
| RP11-23P13.4 | -0.206461955 | 0.943744093 | down |
| SNORA41 | -0.206461955 | 0.943744093 | down |
| RP11-485O10.2 | -0.206461955 | 0.943744093 | down |
| RNA5SP394 | -0.206461955 | 0.943744093 | down |
| RNU2-53P | -0.206461955 | 0.943744093 | down |
| RP11-1O10.1 | -0.206461955 | 0.943744093 | down |
| MIR629 | -0.206461955 | 0.943744093 | down |
| RP11-326A19.2 | -0.206461955 | 0.943744093 | down |
| MIR5009 | -0.206461955 | 0.943744093 | down |
| RP11-76E17.1 | -0.206461955 | 0.943744093 | down |
| NA | -0.206461955 | 0.943744093 | down |
| NA | -0.206461955 | 0.943744093 | down |

| | | | |
|---|---|---|---|
| RNU6-1186P | -0.206461955 | 0.943744093 | down |
| SNRPCP20 | -0.206461955 | 0.943744093 | down |
| MTCO3P24 | -0.206461955 | 0.943744093 | down |
| TMF1P1 | -0.206461955 | 0.943744093 | down |
| CTD-2576D5.3 | -0.206461955 | 0.943744093 | down |
| HSPE1P16 | -0.206461955 | 0.943744093 | down |
| SLX1A | -0.206461955 | 0.943744093 | down |
| RP11-17M15.1 | -0.206461955 | 0.943744093 | down |
| RP11-652G5.1 | -0.206461955 | 0.943744093 | down |
| RP11-812E19.13 | -0.206461955 | 0.943744093 | down |
| RNA5-8SP2 | -0.206461955 | 0.943744093 | down |
| RARRES2P6 | -0.206461955 | 0.943744093 | down |
| RARRES2P10 | -0.206461955 | 0.943744093 | down |
| C1QL1P1 | -0.206461955 | 0.943744093 | down |
| RP11-352B15.5 | -0.206461955 | 0.943744093 | down |
| CTC-527H23.2 | -0.206461955 | 0.943744093 | down |
| NA | -0.206461955 | 0.943744093 | down |
| AC137527.2 | -0.206461955 | 0.943744093 | down |
| AC009052.12 | -0.206461955 | 0.943744093 | down |
| RP11-510M2.7 | -0.206461955 | 0.943744093 | down |
| MTCYBP28 | -0.206461955 | 0.943744093 | down |
| MIR3182 | -0.206461955 | 0.943744093 | down |
| NA | -0.206461955 | 0.943744093 | down |
| MIR4520-1 | -0.206461955 | 0.943744093 | down |
| CTC-297N7.10 | -0.206461955 | 0.943744093 | down |
| TBC1D3P4 | -0.206461955 | 0.943744093 | down |
| RP11-160E2.11 | -0.206461955 | 0.943744093 | down |
| AC004702.2 | -0.206461955 | 0.943744093 | down |
| RNU6-1057P | -0.206461955 | 0.943744093 | down |
| HNRNPA1P19 | -0.206461955 | 0.943744093 | down |
| RP11-822E23.2 | -0.206461955 | 0.943744093 | down |
| MTCYBP13 | -0.206461955 | 0.943744093 | down |
| RPS29P22 | -0.206461955 | 0.943744093 | down |
| RNU6-711P | -0.206461955 | 0.943744093 | down |
| RNU6-1134P | -0.206461955 | 0.943744093 | down |
| RP11-118G23.1 | -0.206461955 | 0.943744093 | down |
| RP11-118G23.4 | -0.206461955 | 0.943744093 | down |
| AC011193.1 | -0.206461955 | 0.943744093 | down |
| TBC1D3C | -0.206461955 | 0.943744093 | down |
| KRTAP4-4 | -0.206461955 | 0.943744093 | down |
| KRTAP9-9 | -0.206461955 | 0.943744093 | down |
| RNU6-453P | -0.206461955 | 0.943744093 | down |
| CCDC103 | -0.206461955 | 0.943744093 | down |
| RP11-312B18.1 | -0.206461955 | 0.943744093 | down |
| RPL36AP48 | -0.206461955 | 0.943744093 | down |
| RNU1-109P | -0.206461955 | 0.943744093 | down |
| RNU7-25P | -0.206461955 | 0.943744093 | down |
| RP11-146G7.2 | -0.206461955 | 0.943744093 | down |
| RP11-128P17.4 | -0.206461955 | 0.943744093 | down |
| RP11-449D8.3 | -0.206461955 | 0.943744093 | down |
| CTD-2006O16.2 | -0.206461955 | 0.943744093 | down |

| | | | |
|---|---|---|---|
| RP11-687D19.1 | -0.206461955 | 0.943744093 | down |
| RP11-49K24.9 | -0.206461955 | 0.943744093 | down |
| MIR4320 | -0.206461955 | 0.943744093 | down |
| RP11-289E15.1 | -0.206461955 | 0.943744093 | down |
| CTD-2526M8.2 | -0.206461955 | 0.943744093 | down |
| MRPS5P4 | -0.206461955 | 0.943744093 | down |
| RP11-640A1.4 | -0.206461955 | 0.943744093 | down |
| RP11-2803.2 | -0.206461955 | 0.943744093 | down |
| RP11-94B19.4 | -0.206461955 | 0.943744093 | down |
| RNU6-655P | -0.206461955 | 0.943744093 | down |
| AC005559.3 | -0.206461955 | 0.943744093 | down |
| SNORD38 | -0.206461955 | 0.943744093 | down |
| MIR6885 | -0.206461955 | 0.943744093 | down |
| OR7E19P | -0.206461955 | 0.943744093 | down |
| CTD-2538G9.3 | -0.206461955 | 0.943744093 | down |
| HAPLN4 | -0.206461955 | 0.943744093 | down |
| CTC-559E9.9 | -0.206461955 | 0.943744093 | down |
| ZNF209P | -0.206461955 | 0.943744093 | down |
| BNIP3P35 | -0.206461955 | 0.943744093 | down |
| CTD-2528L19.4 | -0.206461955 | 0.943744093 | down |
| AC067969.2 | -0.206461955 | 0.943744093 | down |
| Y_RNA | -0.206461955 | 0.943744093 | down |
| AC005626.3 | -0.206461955 | 0.943744093 | down |
| APOC4-APOC2 | -0.206461955 | 0.943744093 | down |
| CGB5 | -0.206461955 | 0.943744093 | down |
| MIR6801 | -0.206461955 | 0.943744093 | down |
| MIR518F | -0.206461955 | 0.943744093 | down |
| MIR518B | -0.206461955 | 0.943744093 | down |
| CTD-3093M3.1 | -0.206461955 | 0.943744093 | down |
| AC008746.3 | -0.206461955 | 0.943744093 | down |
| RP5-1164C1.2 | -0.206461955 | 0.943744093 | down |
| RP5-1068H6.1 | -0.206461955 | 0.943744093 | down |
| RP4-775C13.2 | -0.206461955 | 0.943744093 | down |
| RP5-988G17.1 | -0.206461955 | 0.943744093 | down |
| RP5-986I17.2 | -0.206461955 | 0.943744093 | down |
| RP5-974N19.1 | -0.206461955 | 0.943744093 | down |
| RPL15P1 | -0.206461955 | 0.943744093 | down |
| RNU7-137P | -0.206461955 | 0.943744093 | down |
| RN7SL638P | -0.206461955 | 0.943744093 | down |
| 5_8S_rRNA | -0.206461955 | 0.943744093 | down |
| CFTRP3 | -0.206461955 | 0.943744093 | down |
| RNA5SP482 | -0.206461955 | 0.943744093 | down |
| GLRXP1 | -0.206461955 | 0.943744093 | down |
| RNU6-1018P | -0.206461955 | 0.943744093 | down |
| RP4-730D4.1 | -0.206461955 | 0.943744093 | down |
| PGBD4P2 | -0.206461955 | 0.943744093 | down |
| RP3-461P17.6 | -0.206461955 | 0.943744093 | down |
| WFDC11 | -0.206461955 | 0.943744093 | down |
| RP4-719C8.1 | -0.206461955 | 0.943744093 | down |
| CH507-210P18.3 | -0.206461955 | 0.943744093 | down |
| bP-2168N6.1 | -0.206461955 | 0.943744093 | down |

| | | | |
|---|---|---|---|
| RN7SL52P | -0.206461955 | 0.943744093 | down |
| AF254982.2 | -0.206461955 | 0.943744093 | down |
| GXYLT1P2 | -0.206461955 | 0.943744093 | down |
| NF1P3 | -0.206461955 | 0.943744093 | down |
| VDAC2P1 | -0.206461955 | 0.943744093 | down |
| Y_RNA | -0.206461955 | 0.943744093 | down |
| PPIAP1 | -0.206461955 | 0.943744093 | down |
| RNU4-45P | -0.206461955 | 0.943744093 | down |
| KRTAP21-4P | -0.206461955 | 0.943744093 | down |
| KRTAP8-2P | -0.206461955 | 0.943744093 | down |
| MIR802 | -0.206461955 | 0.943744093 | down |
| MYL6P2 | -0.206461955 | 0.943744093 | down |
| RNU6-1150P | -0.206461955 | 0.943744093 | down |
| LA16c-60G3.6 | -0.206461955 | 0.943744093 | down |
| AC008103.3 | -0.206461955 | 0.943744093 | down |
| IGLV10-67 | -0.206461955 | 0.943744093 | down |
| CTA-992D9.10 | -0.206461955 | 0.943744093 | down |
| RP1-205F14P.1 | -0.206461955 | 0.943744093 | down |
| RN7SL162P | -0.206461955 | 0.943744093 | down |
| RFPL3-AS1_1 | -0.206461955 | 0.943744093 | down |
| RP4-754E20__A.5 | -0.206461955 | 0.943744093 | down |
| MIR651 | -0.206461955 | 0.943744093 | down |
| RN7SKP20 | -0.206461955 | 0.943744093 | down |
| RP11-699A7.1 | -0.206461955 | 0.943744093 | down |
| GS1-433024.1 | -0.206461955 | 0.943744093 | down |
| MAGEB5 | -0.206461955 | 0.943744093 | down |
| MIR4666B | -0.206461955 | 0.943744093 | down |
| HMGB1P16 | -0.206461955 | 0.943744093 | down |
| RP11-49208.1 | -0.206461955 | 0.943744093 | down |
| AL121578.5 | -0.206461955 | 0.943744093 | down |
| RP11-75A9.1 | -0.206461955 | 0.943744093 | down |
| NA | -0.206461955 | 0.943744093 | down |
| GAGE12J | -0.206461955 | 0.943744093 | down |
| HMGB1P15 | -0.206461955 | 0.943744093 | down |
| RP11-363G10.3 | -0.206461955 | 0.943744093 | down |
| SSXP4 | -0.206461955 | 0.943744093 | down |
| RNA5SP505 | -0.206461955 | 0.943744093 | down |
| PPP1R11P2 | -0.206461955 | 0.943744093 | down |
| COX6CP12 | -0.206461955 | 0.943744093 | down |
| CXorf49B | -0.206461955 | 0.943744093 | down |
| YWHAZP8 | -0.206461955 | 0.943744093 | down |
| CYLC1 | -0.206461955 | 0.943744093 | down |
| RP11-345E19.2 | -0.206461955 | 0.943744093 | down |
| RP1-249C1.1 | -0.206461955 | 0.943744093 | down |
| CALM1P1 | -0.206461955 | 0.943744093 | down |
| RP11-471G13.2 | -0.206461955 | 0.943744093 | down |
| RPS29P28 | -0.206461955 | 0.943744093 | down |
| RP11-390F10.3 | -0.206461955 | 0.943744093 | down |
| RP3-347M6.1 | -0.206461955 | 0.943744093 | down |
| RP1-232L22__A.1 | -0.206461955 | 0.943744093 | down |
| HMGB1P12 | -0.206461955 | 0.943744093 | down |

| | | | | |
|---|---|---|---|---|
| DPRXP7 | -0.206461955 | 0.943744093 | | down |
| RP5-1170D6.1 | -0.206461955 | 0.943744093 | | down |
| CTD-2230M5.3 | -0.206461955 | 0.943744093 | | down |
| RP13-347D8.7 | -0.206461955 | 0.943744093 | | down |
| U3 | -0.206461955 | 0.943744093 | | down |
| RP3-384D21.2 | -0.206461955 | 0.943744093 | | down |
| MTCYBP38 | -0.206461955 | 0.943744093 | | down |
| OR13H1 | -0.206461955 | 0.943744093 | | down |
| OR13K1P | -0.206461955 | 0.943744093 | | down |
| AL691477.1 | -0.206461955 | 0.943744093 | | down |
| Y_RNA | -0.206461955 | 0.943744093 | | down |
| RP1-171K16.5 | -0.206461955 | 0.943744093 | | down |
| SPANXD | -0.206461955 | 0.943744093 | | down |
| RP3-406C18.2 | -0.206461955 | 0.943744093 | | down |
| HNRNPH1P2 | -0.206461955 | 0.943744093 | | down |
| MIR513A1 | -0.206461955 | 0.943744093 | | down |
| MIR513A2 | -0.206461955 | 0.943744093 | | down |
| SPRY3 | -0.206461955 | 0.943744093 | | down |
| ATP5JP1 | -0.206461955 | 0.943744093 | | down |
| RNU6-521P | -0.206461955 | 0.943744093 | | down |
| RP11-131M6.1 | -0.206461955 | 0.943744093 | | down |
| RCC2P1 | -0.206461955 | 0.943744093 | | down |
| AC006386.1 | -0.206461955 | 0.943744093 | | down |
| CICP1 | -0.206461955 | 0.943744093 | | down |
| CSPG4P4Y | -0.206461955 | 0.943744093 | | down |
| FAM58CP | -0.206461955 | 0.943744093 | | down |
| MT-TH | -0.206461955 | 0.943744093 | | down |
| MUT | -0.20637427 | 0.355693068 | 0.494299697 | down |
| AF127577.11 | -0.206350495 | 0.772737666 | | down |
| CALHM3 | -0.20634268 | 0.841746936 | | down |
| RPL12P4 | -0.206321583 | 0.361535197 | 0.499906271 | down |
| PRSS12 | -0.206180514 | 0.566629829 | 0.684760331 | down |
| PHKA2 | -0.206114859 | 0.259754106 | 0.394981899 | down |
| RP4-800J21.3 | -0.206107067 | 0.636090338 | 0.743003975 | down |
| TTLL5 | -0.206089074 | 0.200690458 | 0.329753575 | down |
| CALM1 | -0.206076001 | 0.197664728 | 0.326316781 | down |
| NDP | -0.206028268 | 0.664641431 | 0.76564967 | down |
| CTD-2529O21.1 | -0.205862817 | 0.880722404 | | down |
| PRR34-AS1 | -0.205806239 | 0.491370129 | 0.619579643 | down |
| ARFIP2 | -0.205655104 | 0.052862124 | 0.127221159 | down |
| CCDC154 | -0.20559797 | 0.545739194 | 0.666999137 | down |
| NA | -0.205447767 | 0.731316626 | 0.817429864 | down |
| RHOG | -0.205416527 | 0.322471583 | 0.461155469 | down |
| UVSSA | -0.205333169 | 0.168113061 | 0.29149817 | down |
| DCBLD2 | -0.205294618 | 0.353928203 | 0.49254393 | down |
| AC046143.3 | -0.205273942 | 0.565261856 | 0.683382045 | down |
| RP11-394I13.1 | -0.205145549 | 0.770830348 | 0.845885091 | down |
| NEU1 | -0.205085061 | 0.255185186 | 0.390306151 | down |
| TBX2-AS1 | -0.205061133 | 0.472322985 | 0.602695764 | down |
| TXNDC17 | -0.204981423 | 0.326536028 | 0.464928128 | down |
| LONP1 | -0.204935094 | 0.222616453 | 0.354496672 | down |

| | | | | |
|---|---|---|---|---|
| GPR89A | -0.204884574 | 0.25406237 | 0.389123666 | down |
| RP11-377G16.2 | -0.20484605 | 0.767210555 | 0.843480812 | down |
| STRBP | -0.20480836 | 0.255612151 | 0.39064844 | down |
| MIR132 | -0.204560194 | 0.823265713 | 0.88418514 | down |
| RPL26P37 | -0.204540754 | 0.748565833 | | down |
| RP11-186N15.3 | -0.204513146 | 0.627405228 | 0.736088826 | down |
| ANKRD26P1 | -0.204492781 | 0.830837884 | 0.889517841 | down |
| LRIG2 | -0.204491866 | 0.137553847 | 0.253087622 | down |
| CTB-41I6.1 | -0.204408619 | 0.699444983 | 0.793015777 | down |
| ZNF567 | -0.204310331 | 0.494443022 | 0.622435522 | down |
| TYW5 | -0.204194945 | 0.154591775 | 0.27446846 | down |
| RP11-342M1.7 | -0.20411141 | 0.863975913 | | down |
| RP11-1182P23.5 | -0.204101477 | 0.831156114 | | down |
| RP4-742C19.13 | -0.204002155 | 0.634276543 | 0.741601979 | down |
| FTLP15 | -0.203953247 | 0.79252036 | 0.861730726 | down |
| METTL18 | -0.203833673 | 0.283174809 | 0.419743256 | down |
| VPS9D1 | -0.20382301 | 0.300760915 | 0.438473453 | down |
| ZNF777 | -0.203804484 | 0.13768133 | 0.253306667 | down |
| BFSP2-AS1 | -0.203761683 | 0.665949468 | 0.766583072 | down |
| FLYWCH2 | -0.203725087 | 0.123641773 | 0.234884188 | down |
| EPT1 | -0.203706536 | 0.288366279 | 0.425551022 | down |
| ZNF766 | -0.20368425 | 0.03529596 | 0.094561752 | down |
| TAF4 | -0.203337152 | 0.126109804 | 0.238182142 | down |
| MICAL1 | -0.203334799 | 0.265882906 | 0.401598011 | down |
| ZFYVE9P2 | -0.20332369 | 0.944597809 | | down |
| RPLP2P1 | -0.20332369 | 0.944597809 | | down |
| OR6T1 | -0.20332369 | 0.944597809 | | down |
| GTF2IP3 | -0.20332369 | 0.944597809 | | down |
| Metazoa_SRP | -0.20332369 | 0.944597809 | | down |
| MAP1LC3P | -0.20332369 | 0.944597809 | | down |
| CTC-429P9.4 | -0.20332369 | 0.944597809 | | down |
| CTD-250103.2 | -0.203323461 | 0.94339111 | | down |
| Y_RNA | -0.203322239 | 0.935297015 | | down |
| NA | -0.203321348 | 0.92656925 | | down |
| C16orf92 | -0.203321208 | 0.924846988 | | down |
| Y_RNA | -0.203320272 | 0.909080843 | | down |
| ATP6V1E1P1 | -0.203320247 | 0.90851726 | | down |
| NA | -0.203320241 | 0.908375337 | | down |
| CTD-3096M3.2 | -0.203320229 | 0.908097335 | | down |
| RP11-408H20.3 | -0.203319879 | 0.898700404 | | down |
| RP11-198M15.1 | -0.203319641 | 0.890361457 | | down |
| RP11-20B24.6 | -0.203319385 | 0.878613418 | | down |
| TIPIN | -0.203305652 | 0.460142232 | 0.592030886 | down |
| MTMR4 | -0.203205894 | 0.281751066 | 0.418207057 | down |
| RNU6-1223P | -0.203181558 | 0.794511635 | 0.863289928 | down |
| CEP350 | -0.203030294 | 0.266870928 | 0.402588766 | down |
| C17orf100 | -0.202925127 | 0.444444274 | 0.577971855 | down |
| TATDN2P2 | -0.202911865 | 0.709716169 | 0.800612495 | down |
| CTD-2583A14.11 | -0.202831101 | 0.525889043 | 0.649556422 | down |
| AC053503.6 | -0.202603445 | 0.738606021 | 0.822333863 | down |
| IGHV3-35 | -0.202580352 | 0.842775153 | | down |

| | | | | |
|---|---|---|---|---|
| CDC42BPA | -0.202464952 | 0.339706558 | 0.478116597 | down |
| ZNF613 | -0.202376363 | 0.360722063 | 0.499103512 | down |
| RP11-34F13.2 | -0.202350676 | 0.862968971 | | down |
| GON4L | -0.202251371 | 0.247445518 | 0.381783494 | down |
| RP11-968A15.2 | -0.202175445 | 0.61210802 | 0.723554879 | down |
| RP3-508I15.20 | -0.202146772 | 0.608974063 | 0.721035884 | down |
| AP000962.1 | -0.202117575 | 0.940701029 | | down |
| RP11-57H14.4 | -0.202077951 | 0.493135609 | 0.621232179 | down |
| CLIC4P3 | -0.20204667 | 0.909616621 | | down |
| BAIAP2-AS1 | -0.202012118 | 0.214916363 | 0.345965623 | down |
| FRG1GP | -0.201983056 | 0.646459291 | 0.751314182 | down |
| RP11-121G22.3 | -0.201970788 | 0.885142429 | | down |
| C2orf74 | -0.201921389 | 0.729082163 | 0.81575095 | down |
| AC073326.3 | -0.201908347 | 0.868458629 | | down |
| RP11-730A19.7 | -0.201905198 | 0.867675606 | | down |
| SNORA9 | -0.201838121 | 0.85203824 | | down |
| LRRTM2 | -0.201815574 | 0.656185138 | 0.758743267 | down |
| RN7SKP70 | -0.20178807 | 0.841442924 | | down |
| NGB | -0.201758222 | 0.727572108 | 0.814577098 | down |
| RP11-44K6.3 | -0.201685296 | 0.821825143 | | down |
| AC022007.5 | -0.201586049 | 0.420220729 | 0.555951895 | down |
| TPRXL | -0.201548026 | 0.701577042 | 0.794537619 | down |
| SMAD2 | -0.201383634 | 0.161108077 | 0.282696593 | down |
| NA | -0.201196501 | 0.577179541 | 0.693853318 | down |
| GTSF1 | -0.201095278 | 0.610375532 | 0.722115475 | down |
| AC007566.10 | -0.201082167 | 0.469574985 | 0.600321504 | down |
| TWF2 | -0.201002499 | 0.288398494 | 0.425551022 | down |
| PRKCQ-AS1 | -0.20098647 | 0.381705484 | 0.519523402 | down |
| RP11-190A12.8 | -0.200910985 | 0.563519959 | 0.682269561 | down |
| SYCE2 | -0.200910301 | 0.589438856 | 0.704615485 | down |
| RP11-152L20.3 | -0.200881826 | 0.86947208 | 0.916608273 | down |
| RP11-466A19.4 | -0.200876677 | 0.821282353 | | down |
| ABCB7 | -0.200829908 | 0.109607554 | 0.215951824 | down |
| SUGT1 | -0.200798337 | 0.098355067 | 0.199633809 | down |
| JAGN1 | -0.200715061 | 0.14912107 | 0.267906084 | down |
| LDHAL6A | -0.200653291 | 0.783907083 | 0.855465772 | down |
| NUDC | -0.200644633 | 0.214213484 | 0.345103217 | down |
| CTC1 | -0.200608727 | 0.168341087 | 0.291708363 | down |
| AC025750.5 | -0.200550274 | 0.918367397 | | down |
| RSAD1 | -0.20046935 | 0.018891462 | 0.059445622 | down |
| ZBED9 | -0.200460216 | 0.643637065 | 0.749164419 | down |
| LINC01481 | -0.200395147 | 0.476927941 | 0.60669079 | down |
| CHAF1B | -0.200236438 | 0.225835496 | 0.357858737 | down |
| COX10 | -0.200146429 | 0.233920666 | 0.366654397 | down |
| AC079610.2 | -0.200021493 | 0.819453562 | | down |
| TTC7B | -0.199891697 | 0.450792827 | 0.58372639 | down |
| CCM2 | -0.199866484 | 0.299339086 | 0.436888196 | down |
| BMP2 | -0.199740796 | 0.379153633 | 0.517151303 | down |
| STMN2 | -0.199720969 | 0.428898368 | 0.564251984 | down |
| AC124789.1 | -0.199711678 | 0.54184414 | 0.663310795 | down |
| CIAPIN1P | -0.199687888 | 0.891645584 | | down |

| | | | | |
|---|---|---|---|---|
| RABGAP1L | -0.199670592 | 0.145441227 | 0.263171621 | down |
| MUC5B | -0.199654586 | 0.704341517 | 0.796519739 | down |
| DPP9 | -0.199435079 | 0.032461284 | 0.088894179 | down |
| ATP6V1E2 | -0.199363984 | 0.280989669 | 0.417368377 | down |
| ZNF678 | -0.19930504 | 0.16406889 | 0.286429582 | down |
| ATP6V0C | -0.199262545 | 0.690160196 | 0.785980394 | down |
| IGSF8 | -0.199228445 | 0.213807495 | 0.344670943 | down |
| RP3-449O17.1 | -0.19914217 | 0.550803932 | 0.671060557 | down |
| ANXA2R | -0.199023039 | 0.451377569 | 0.584301597 | down |
| BFSP1 | -0.198948806 | 0.521815267 | 0.646118837 | down |
| CTD-3065J16.9 | -0.198910281 | 0.463544761 | 0.595153517 | down |
| FBL | -0.198821578 | 0.22497634 | 0.357074526 | down |
| PTGS1 | -0.198704795 | 0.260030985 | 0.395150778 | down |
| GALNT7 | -0.198595123 | 0.434075028 | 0.568970459 | down |
| MIR4458HG | -0.198534703 | 0.333480888 | 0.47173124 | down |
| AC068580.5 | -0.19847959 | 0.710816836 | 0.801490775 | down |
| GIPC3 | -0.198403243 | 0.352582722 | 0.491370927 | down |
| RP11-141B14.2 | -0.198318195 | 0.87280101 | | down |
| CTD-2006C1.2 | -0.198147795 | 0.395608376 | 0.53285089 | down |
| PIGV | -0.198066438 | 0.233356478 | 0.366099637 | down |
| TMEM79 | -0.198019642 | 0.319186607 | 0.457936222 | down |
| UBA1 | -0.198011404 | 0.144132779 | 0.261635261 | down |
| RP11-307E17.11 | -0.197924911 | 0.694617252 | 0.789508597 | down |
| MIR1244-4 | -0.197872696 | 0.539154901 | 0.661077005 | down |
| LL22NC03-2H8.4 | -0.197846145 | 0.723959513 | 0.811745427 | down |
| CEACAM21 | -0.197685001 | 0.514560197 | 0.639771832 | down |
| RP11-798G7.5 | -0.197614301 | 0.767788034 | 0.843887849 | down |
| NA | -0.197449481 | 0.764608265 | 0.841561776 | down |
| TSTA3 | -0.197338062 | 0.339704568 | 0.478116597 | down |
| MGAT3 | -0.197233354 | 0.52487711 | 0.648876143 | down |
| CH507-513H4.6 | -0.197226025 | 0.883228464 | | down |
| LNPK | -0.197220792 | 0.27339071 | 0.409663366 | down |
| RP11-54O7.17 | -0.197156936 | 0.617779981 | 0.728406963 | down |
| TOMM7 | -0.197131852 | 0.238612399 | 0.37202279 | down |
| ELOVL1 | -0.197090383 | 0.172588869 | 0.29687736 | down |
| AC114730.11 | -0.197059134 | 0.703764099 | 0.796230036 | down |
| SCARNA11 | -0.197052905 | 0.946303866 | | down |
| CROCCP5 | -0.197052905 | 0.946303866 | | down |
| SLC25A24P2 | -0.197052905 | 0.946303866 | | down |
| HIST2H3C | -0.197052905 | 0.946303866 | | down |
| Y_RNA | -0.197052905 | 0.946303866 | | down |
| RP3-332O11.2 | -0.197052905 | 0.946303866 | | down |
| MIR4424 | -0.197052905 | 0.946303866 | | down |
| RP11-38O14.6 | -0.197052905 | 0.946303866 | | down |
| C4BPAP2 | -0.197052905 | 0.946303866 | | down |
| OR3D1P | -0.197052905 | 0.946303866 | | down |
| RNU6-939P | -0.197052905 | 0.946303866 | | down |
| ABCD1P5 | -0.197052905 | 0.946303866 | | down |
| RNU7-96P | -0.197052905 | 0.946303866 | | down |
| RNU6-1275P | -0.197052905 | 0.946303866 | | down |
| AC093899.3 | -0.197052905 | 0.946303866 | | down |

| | | | |
|---|---|---|---|
| RN7SL267P | -0.197052905 | 0.946303866 | down |
| SNORD11B | -0.197052905 | 0.946303866 | down |
| MIR4777 | -0.197052905 | 0.946303866 | down |
| AY269186.2 | -0.197052905 | 0.946303866 | down |
| NA | -0.197052905 | 0.946303866 | down |
| RP11-572B2.1 | -0.197052905 | 0.946303866 | down |
| RNU7-110P | -0.197052905 | 0.946303866 | down |
| CLUHP10 | -0.197052905 | 0.946303866 | down |
| RNU6-1127P | -0.197052905 | 0.946303866 | down |
| RP11-158I23.1 | -0.197052905 | 0.946303866 | down |
| AC069213.4 | -0.197052905 | 0.946303866 | down |
| RNU6-1279P | -0.197052905 | 0.946303866 | down |
| RNU1-63P | -0.197052905 | 0.946303866 | down |
| RP11-584P21.4 | -0.197052905 | 0.946303866 | down |
| Y_RNA | -0.197052905 | 0.946303866 | down |
| RP11-102N12.2 | -0.197052905 | 0.946303866 | down |
| RN7SKP13 | -0.197052905 | 0.946303866 | down |
| CTD-219904.4 | -0.197052905 | 0.946303866 | down |
| CTC-529L17.2 | -0.197052905 | 0.946303866 | down |
| CTB-88F18.3 | -0.197052905 | 0.946303866 | down |
| RNU6-1311P | -0.197052905 | 0.946303866 | down |
| FBXL21 | -0.197052905 | 0.946303866 | down |
| NA | -0.197052905 | 0.946303866 | down |
| NA | -0.197052905 | 0.946303866 | down |
| LINC01015 | -0.197052905 | 0.946303866 | down |
| TMEM183AP1 | -0.197052905 | 0.946303866 | down |
| RNU1-87P | -0.197052905 | 0.946303866 | down |
| SMIM11P1 | -0.197052905 | 0.946303866 | down |
| RP1-191J18.65 | -0.197052905 | 0.946303866 | down |
| RPL7P28 | -0.197052905 | 0.946303866 | down |
| RNU4-18P | -0.197052905 | 0.946303866 | down |
| TUBB8P2 | -0.197052905 | 0.946303866 | down |
| NA | -0.197052905 | 0.946303866 | down |
| CYCSP19 | -0.197052905 | 0.946303866 | down |
| AC011625.1 | -0.197052905 | 0.946303866 | down |
| OR2AO1P | -0.197052905 | 0.946303866 | down |
| RP11-175E9.2 | -0.197052905 | 0.946303866 | down |
| RP11-726G23.10 | -0.197052905 | 0.946303866 | down |
| GYG1P1 | -0.197052905 | 0.946303866 | down |
| RP11-219B4.6 | -0.197052905 | 0.946303866 | down |
| RP11-203L2.4 | -0.197052905 | 0.946303866 | down |
| RP11-270F18.2 | -0.197052905 | 0.946303866 | down |
| RP11-229P13.22 | -0.197052905 | 0.946303866 | down |
| Y_RNA | -0.197052905 | 0.946303866 | down |
| RPL7P37 | -0.197052905 | 0.946303866 | down |
| GAPDHP28 | -0.197052905 | 0.946303866 | down |
| SNORA25 | -0.197052905 | 0.946303866 | down |
| RP11-430L3.1 | -0.197052905 | 0.946303866 | down |
| LINC01493 | -0.197052905 | 0.946303866 | down |
| OR4A18P | -0.197052905 | 0.946303866 | down |
| RP11-429A20.2 | -0.197052905 | 0.946303866 | down |

| | | | | |
|---|---|---|---|---|
| RP11-318G8.4 | -0.197052905 | 0.946303866 | | down |
| RP11-609L23.2 | -0.197052905 | 0.946303866 | | down |
| RNU4-65P | -0.197052905 | 0.946303866 | | down |
| NA | -0.197052905 | 0.946303866 | | down |
| GALNT4 | -0.197052905 | 0.946303866 | | down |
| PTP4A1P2 | -0.197052905 | 0.946303866 | | down |
| RNU6-53P | -0.197052905 | 0.946303866 | | down |
| TRAJ30 | -0.197052905 | 0.946303866 | | down |
| PSMA3P | -0.197052905 | 0.946303866 | | down |
| NA | -0.197052905 | 0.946303866 | | down |
| MIR345 | -0.197052905 | 0.946303866 | | down |
| RD3L | -0.197052905 | 0.946303866 | | down |
| IGHVIII-13-1 | -0.197052905 | 0.946303866 | | down |
| IGHVIII-76-1 | -0.197052905 | 0.946303866 | | down |
| RP11-507J18.1 | -0.197052905 | 0.946303866 | | down |
| DNM1P38 | -0.197052905 | 0.946303866 | | down |
| LA16c-335H7.2 | -0.197052905 | 0.946303866 | | down |
| RP11-95J11.1 | -0.197052905 | 0.946303866 | | down |
| RNU6-944P | -0.197052905 | 0.946303866 | | down |
| VN1R64P | -0.197052905 | 0.946303866 | | down |
| RP11-46309.6 | -0.197052905 | 0.946303866 | | down |
| RP11-46C24.3 | -0.197052905 | 0.946303866 | | down |
| RP1-4G17.2 | -0.197052905 | 0.946303866 | | down |
| RPL7AP65 | -0.197052905 | 0.946303866 | | down |
| NA | -0.197052905 | 0.946303866 | | down |
| NA | -0.197052905 | 0.946303866 | | down |
| RPL9P30 | -0.197052905 | 0.946303866 | | down |
| AC104984.4 | -0.197052905 | 0.946303866 | | down |
| MIR4733 | -0.197052905 | 0.946303866 | | down |
| WDR45BP1 | -0.197052905 | 0.946303866 | | down |
| RP11-481C4.1 | -0.197052905 | 0.946303866 | | down |
| RP11-178C3.7 | -0.197052905 | 0.946303866 | | down |
| HMGN1P28 | -0.197052905 | 0.946303866 | | down |
| Y_RNA | -0.197052905 | 0.946303866 | | down |
| RNU2-69P | -0.197052905 | 0.946303866 | | down |
| PPAN-P2RY11 | -0.197052905 | 0.946303866 | | down |
| RNA5SP464 | -0.197052905 | 0.946303866 | | down |
| CTC-260E6.12 | -0.197052905 | 0.946303866 | | down |
| RP5-1178H5.2 | -0.197052905 | 0.946303866 | | down |
| Y_RNA | -0.197052905 | 0.946303866 | | down |
| MIR4756 | -0.197052905 | 0.946303866 | | down |
| LA16c-60G3.8 | -0.197052905 | 0.946303866 | | down |
| NA | -0.197052905 | 0.946303866 | | down |
| RNU6-409P | -0.197052905 | 0.946303866 | | down |
| Y_RNA | -0.197052905 | 0.946303866 | | down |
| TUBB4BP3 | -0.197052905 | 0.946303866 | | down |
| RNU6-972P | -0.197052905 | 0.946303866 | | down |
| RP11-1114A5.6 | -0.197052905 | 0.946303866 | | down |
| MIR513B | -0.197052905 | 0.946303866 | | down |
| CDKN1A | -0.196825248 | 0.395894827 | 0.532992659 | down |
| SYNGR2 | -0.196782745 | 0.244602926 | 0.378650573 | down |

| | | | | |
|---|---|---|---|---|
| MFSD6 | -0.196615733 | 0.243612315 | 0.37766194 | down |
| ATP6AP1L | -0.196400129 | 0.395362045 | 0.532728944 | down |
| GAGE10 | -0.196345488 | 0.887837891 | | down |
| LINC00467 | -0.196297318 | 0.215730083 | 0.346863705 | down |
| AC004448.2 | -0.196252943 | 0.880627453 | | down |
| PCDHB10 | -0.196251803 | 0.732173955 | 0.818000854 | down |
| RP11-506M12.1 | -0.196230872 | 0.74778935 | 0.828933447 | down |
| RP11-89N17.1 | -0.196221141 | 0.854359658 | | down |
| FOXD4L1 | -0.196215319 | 0.714557279 | 0.80417137 | down |
| GOLGA8VP | -0.196183452 | 0.607949979 | 0.720178088 | down |
| COL27A1 | -0.196172058 | 0.303278828 | 0.441309106 | down |
| RP11-490N5.3 | -0.196141203 | 0.803480862 | | down |
| LIMCH1 | -0.196070602 | 0.537868832 | 0.660037733 | down |
| ZNF124 | -0.195984862 | 0.330165144 | 0.468695974 | down |
| USP19 | -0.195972147 | 0.014276513 | 0.048112571 | down |
| MAP2K7 | -0.195942009 | 0.089890094 | 0.187376534 | down |
| RABL2A | -0.19589288 | 0.173984453 | 0.298440703 | down |
| GSN | -0.195879673 | 0.341130506 | 0.479481868 | down |
| DHX34 | -0.195868103 | 0.313211902 | 0.451918752 | down |
| TBC1D3L | -0.195797691 | 0.534267062 | 0.656960653 | down |
| SLC37A1 | -0.195724989 | 0.360454344 | 0.498802004 | down |
| ABHD15 | -0.195615635 | 0.402936698 | 0.539615776 | down |
| RP11-422P24.12 | -0.195559288 | 0.597875035 | 0.71176053 | down |
| REEP4 | -0.195551554 | 0.327471226 | 0.465950534 | down |
| RP11-830F9.6 | -0.195441491 | 0.57218837 | 0.689536216 | down |
| TNXB | -0.195393805 | 0.546304811 | 0.667582435 | down |
| LINC00982 | -0.195387218 | 0.546626335 | 0.667703757 | down |
| SAMD10 | -0.195279417 | 0.30644936 | 0.444807209 | down |
| ASB1 | -0.195194372 | 0.193118751 | 0.321141293 | down |
| RP11-215D10.1 | -0.195122461 | 0.733695303 | 0.819068486 | down |
| ERGIC3 | -0.195073455 | 0.259545753 | 0.394784964 | down |
| NLRP14 | -0.195072451 | 0.782551598 | 0.854509712 | down |
| HIST2H2AC | -0.195022581 | 0.490141308 | 0.618575378 | down |
| BRWD3 | -0.195021676 | 0.426172719 | 0.56177313 | down |
| PLEKHH2 | -0.194901395 | 0.554707587 | 0.674608253 | down |
| C2orf66 | -0.194865822 | 0.75256864 | 0.832425048 | down |
| ZNF417 | -0.194838963 | 0.290683271 | 0.427819957 | down |
| RP11-394I13.2 | -0.194712068 | 0.67310498 | 0.772450956 | down |
| GAPDHP42 | -0.194615755 | 0.859774903 | | down |
| CETP | -0.194605794 | 0.64379441 | 0.749260478 | down |
| RPL6P27 | -0.194550782 | 0.17444885 | 0.29891304 | down |
| AC098820.4 | -0.194540036 | 0.831206528 | | down |
| RP1-30M3.6 | -0.194474899 | 0.607634262 | 0.719872901 | down |
| FRAT2 | -0.194357472 | 0.339984564 | 0.47831872 | down |
| PRIMA1 | -0.194354077 | 0.58988968 | 0.705042191 | down |
| NUTM2B-AS1 | -0.194329366 | 0.174652061 | 0.299039524 | down |
| CLTCL1 | -0.19428222 | 0.398844809 | 0.535789103 | down |
| CTD-2349P21.12 | -0.194219895 | 0.751098031 | 0.83152735 | down |
| CAPN11 | -0.19412807 | 0.610453001 | 0.722178688 | down |
| UBE3B | -0.194096873 | 0.072756406 | 0.160857567 | down |
| LRRC37A9P | -0.194013305 | 0.882244907 | | down |

| | | | | |
|---|---|---|---|---|
| HAR1A | -0.193998504 | 0.492245357 | 0.620474911 | down |
| RP11-665I14.1 | -0.19391464 | 0.947157769 | | down |
| NA | -0.19391464 | 0.947157769 | | down |
| RPL23AP78 | -0.19391464 | 0.947157769 | | down |
| NDUFB10P2 | -0.193914434 | 0.946094969 | | down |
| DHFRP2 | -0.193914423 | 0.946036316 | | down |
| RP11-522B15.7 | -0.1939143 | 0.945363078 | | down |
| RP3-425P12.5 | -0.193914245 | 0.945059453 | | down |
| Y_RNA | -0.193913198 | 0.937992701 | | down |
| ELMO1-AS1 | -0.19391313 | 0.937433556 | | down |
| UBE2E2-AS1 | -0.1939131 | 0.937179263 | | down |
| MIR1180 | -0.193912308 | 0.929214283 | | down |
| RP11-697N18.1 | -0.193912246 | 0.928455329 | | down |
| CTC-513N18.6 | -0.193912225 | 0.928187329 | | down |
| Y_RNA | -0.193911728 | 0.920981287 | | down |
| CTD-3236F5.1 | -0.193910972 | 0.904104196 | | down |
| RP11-102C16.3 | -0.193910971 | 0.904060006 | | down |
| RP11-630I5.1 | -0.193910938 | 0.903043024 | | down |
| NA | -0.193910911 | 0.902217874 | | down |
| COX6CP2 | -0.193910494 | 0.885448429 | | down |
| Y_RNA | -0.1939102 | 0.866659993 | | down |
| GOLGA6L5P | -0.193888107 | 0.655106312 | 0.75799176 | down |
| P2RX1 | -0.193872359 | 0.38715178 | 0.524878298 | down |
| AC114730.2 | -0.193817007 | 0.752481746 | 0.832425048 | down |
| GPANK1 | -0.193708776 | 0.067567448 | 0.152322083 | down |
| RP11-162J8.3 | -0.193693233 | 0.759860837 | 0.837924719 | down |
| SLC9A6 | -0.193680733 | 0.263699824 | 0.3991233 | down |
| CTD-2621I17.3 | -0.193654919 | 0.673294465 | 0.772570719 | down |
| URM1 | -0.193438571 | 0.168654169 | 0.29209608 | down |
| KB-1517D11.4 | -0.193428504 | 0.732458672 | 0.818157726 | down |
| OR8R1P | -0.193390727 | 0.876324959 | | down |
| RP11-391L3.5 | -0.193231883 | 0.847841524 | 0.901785937 | down |
| RP11-472G21.2 | -0.193196846 | 0.82460036 | | down |
| FPGS | -0.193057072 | 0.282923911 | 0.419451888 | down |
| RP11-424M24.3 | -0.192981213 | 0.811169732 | | down |
| DEFB109P3 | -0.192947616 | 0.795829493 | | down |
| CTC-471J1.2 | -0.192919224 | 0.519520739 | 0.644073661 | down |
| MTCO3P43 | -0.192868675 | 0.83152669 | | down |
| OFD1 | -0.192737087 | 0.224083289 | 0.356322554 | down |
| BAZ1A | -0.192660066 | 0.368929001 | 0.507281597 | down |
| LINGO4 | -0.192354168 | 0.67322499 | 0.772529661 | down |
| C9orf170 | -0.19193932 | 0.775910431 | 0.849976409 | down |
| RP11-455F5.3 | -0.191856809 | 0.551526259 | 0.67155933 | down |
| CTD-2260A17.1 | -0.1916992 | 0.706027134 | 0.797828985 | down |
| TMEM129 | -0.191675517 | 0.224776514 | 0.356914834 | down |
| RP11-973H7.3 | -0.191668531 | 0.510624383 | 0.636485042 | down |
| NA | -0.191616307 | 0.439244552 | 0.573307238 | down |
| SLC35A4 | -0.19155527 | 0.048791321 | 0.120252826 | down |
| RPL4P2 | -0.191494362 | 0.675265571 | 0.774368442 | down |
| HIST1H2BL | -0.191478186 | 0.721937817 | 0.810443514 | down |
| SNX12 | -0.191436771 | 0.335469191 | 0.473674047 | down |

| | | | | |
|---|---|---|---|---|
| RP11-413N19.2 | -0.191397522 | 0.80183682 | 0.868709479 | down |
| RTN3 | -0.191347439 | 0.189253163 | 0.316394304 | down |
| SMAD4 | -0.191277304 | 0.011973208 | 0.042141876 | down |
| COQ9 | -0.191260941 | 0.451804616 | 0.584658564 | down |
| RP11-296P7.4 | -0.191246349 | 0.772849775 | 0.847426958 | down |
| MED17 | -0.191116783 | 0.415563198 | 0.552001978 | down |
| NA | -0.191028209 | 0.855447506 | | down |
| NT5DC1 | -0.190994615 | 0.285370974 | 0.422390656 | down |
| RP11-490B18.6 | -0.190967432 | 0.638724056 | 0.745091265 | down |
| ITGB6 | -0.19090564 | 0.593443389 | 0.708050243 | down |
| TRIM2 | -0.19086876 | 0.3291506 | 0.467560254 | down |
| RP5-921G16.2 | -0.190707055 | 0.889407653 | | down |
| GAPDHP60 | -0.190699148 | 0.72047548 | 0.809355163 | down |
| AC009404.2 | -0.190504584 | 0.417882652 | 0.554029672 | down |
| AC024560.3 | -0.190501044 | 0.283530798 | 0.420163722 | down |
| RP11-266K4.9 | -0.190494015 | 0.630391625 | 0.738410308 | down |
| MANSC1 | -0.190314975 | 0.361512093 | 0.499897331 | down |
| RPS18P12 | -0.190258978 | 0.499451989 | 0.626614422 | down |
| MRPL24 | -0.190212697 | 0.323772206 | 0.462125904 | down |
| AC004967.7 | -0.190074603 | 0.731026978 | 0.817196552 | down |
| KB-1471A8.1 | -0.190065722 | 0.819338093 | | down |
| RP11-131M11.2 | -0.189936052 | 0.634644228 | 0.741916389 | down |
| FAM133DP | -0.189851638 | 0.708422525 | 0.799633555 | down |
| TREML1 | -0.189846224 | 0.690205241 | 0.785980603 | down |
| RP11-440L14.1 | -0.189795156 | 0.426575798 | 0.562156473 | down |
| RP11-375N15.2 | -0.189763175 | 0.696173169 | 0.790678815 | down |
| RP6-159A1.3 | -0.189651407 | 0.85781698 | | down |
| DEDD2 | -0.189494862 | 0.25438438 | 0.389438181 | down |
| CEND1 | -0.189408346 | 0.553068324 | 0.673027856 | down |
| NA | -0.189296993 | 0.705458192 | 0.797366011 | down |
| ZNF511 | -0.189248356 | 0.434972447 | 0.569772943 | down |
| KIF19 | -0.189248356 | 0.403649555 | 0.540318501 | down |
| KCNH2 | -0.18913181 | 0.531562549 | 0.654841119 | down |
| RP1-111B22.3 | -0.189081042 | 0.741559746 | 0.824430914 | down |
| RP4-545C24.1 | -0.188809701 | 0.383023831 | 0.520775128 | down |
| PITPNM2 | -0.188678461 | 0.207728517 | 0.337885096 | down |
| C20orf196 | -0.188666817 | 0.227307472 | 0.359501685 | down |
| DBNDD2 | -0.188519281 | 0.605093156 | 0.717702963 | down |
| MRPS18B | -0.188479488 | 0.035300562 | 0.094561752 | down |
| TIMM8B | -0.18844069 | 0.325985156 | 0.464441877 | down |
| TMCO1 | -0.188435705 | 0.265174641 | 0.400790165 | down |
| RBM6 | -0.188431392 | 0.234177486 | 0.366943651 | down |
| RP4-765C7.2 | -0.188326412 | 0.833574428 | 0.891400965 | down |
| HOXA9 | -0.18823584 | 0.488465967 | 0.617139258 | down |
| ZNF461 | -0.188228064 | 0.274752199 | 0.411047459 | down |
| UPF2 | -0.188151925 | 0.269469652 | 0.405344526 | down |
| SPATA12 | -0.187934249 | 0.681495692 | 0.779371039 | down |
| RP11-295G24.5 | -0.187775716 | 0.581224479 | 0.697265143 | down |
| ANKS6 | -0.187751401 | 0.242609672 | 0.376496125 | down |
| RP4-669L17.8 | -0.187643855 | 0.948864194 | | down |
| RNA5SP44 | -0.187643855 | 0.948864194 | | down |

| | | | |
|---|---|---|---|
| RP11-67L3.5 | -0.187643855 | 0.948864194 | down |
| RP11-290M5.2 | -0.187643855 | 0.948864194 | down |
| RNU6-125P | -0.187643855 | 0.948864194 | down |
| MIR4742 | -0.187643855 | 0.948864194 | down |
| MIR4666A | -0.187643855 | 0.948864194 | down |
| AC106053.1 | -0.187643855 | 0.948864194 | down |
| RN7SL414P | -0.187643855 | 0.948864194 | down |
| TRIM64FP | -0.187643855 | 0.948864194 | down |
| AC108059.4 | -0.187643855 | 0.948864194 | down |
| CDK7P1 | -0.187643855 | 0.948864194 | down |
| SNORA70F | -0.187643855 | 0.948864194 | down |
| MTND4P30 | -0.187643855 | 0.948864194 | down |
| VWC2L-IT1 | -0.187643855 | 0.948864194 | down |
| RP11-316O14.1 | -0.187643855 | 0.948864194 | down |
| NA | -0.187643855 | 0.948864194 | down |
| VPS26AP1 | -0.187643855 | 0.948864194 | down |
| RP11-768G7.2 | -0.187643855 | 0.948864194 | down |
| HMGB3P14 | -0.187643855 | 0.948864194 | down |
| PLCH1-AS2 | -0.187643855 | 0.948864194 | down |
| LINC01209 | -0.187643855 | 0.948864194 | down |
| OR7E35P | -0.187643855 | 0.948864194 | down |
| RP11-22A3.1 | -0.187643855 | 0.948864194 | down |
| RP11-18M17.1 | -0.187643855 | 0.948864194 | down |
| RP11-57B24.1 | -0.187643855 | 0.948864194 | down |
| R3HDM2P1 | -0.187643855 | 0.948864194 | down |
| CTD-2275D10.2 | -0.187643855 | 0.948864194 | down |
| RP11-546B8.3 | -0.187643855 | 0.948864194 | down |
| NA | -0.187643855 | 0.948864194 | down |
| OR2B8P | -0.187643855 | 0.948864194 | down |
| MIR4642 | -0.187643855 | 0.948864194 | down |
| snoU13 | -0.187643855 | 0.948864194 | down |
| RP4-725G10.4 | -0.187643855 | 0.948864194 | down |
| RP11-632K21.6 | -0.187643855 | 0.948864194 | down |
| RNU6-665P | -0.187643855 | 0.948864194 | down |
| RP11-642D21.2 | -0.187643855 | 0.948864194 | down |
| MTCO1P47 | -0.187643855 | 0.948864194 | down |
| ZNHIT1P1 | -0.187643855 | 0.948864194 | down |
| RP11-265B7.1 | -0.187643855 | 0.948864194 | down |
| RP11-452D2.2 | -0.187643855 | 0.948864194 | down |
| RP11-330M2.3 | -0.187643855 | 0.948864194 | down |
| MIR147A | -0.187643855 | 0.948864194 | down |
| RNA5SP307 | -0.187643855 | 0.948864194 | down |
| RP11-351M16.3 | -0.187643855 | 0.948864194 | down |
| RSU1P3 | -0.187643855 | 0.948864194 | down |
| RP11-399K21.12 | -0.187643855 | 0.948864194 | down |
| LINC00678 | -0.187643855 | 0.948864194 | down |
| RP11-242C24.2 | -0.187643855 | 0.948864194 | down |
| RP11-793H13.3 | -0.187643855 | 0.948864194 | down |
| RP11-158L12.2 | -0.187643855 | 0.948864194 | down |
| RP11-407A16.3 | -0.187643855 | 0.948864194 | down |
| RP11-341D18.2 | -0.187643855 | 0.948864194 | down |

| | | | | |
|---|---|---|---|---|
| RXFP2 | -0.187643855 | 0.948864194 | | down |
| ARHGEF7-IT1 | -0.187643855 | 0.948864194 | | down |
| RN7SL189P | -0.187643855 | 0.948864194 | | down |
| TRAJ46 | -0.187643855 | 0.948864194 | | down |
| NF1P2 | -0.187643855 | 0.948864194 | | down |
| RP11-566K19.11 | -0.187643855 | 0.948864194 | | down |
| AC124312.1 | -0.187643855 | 0.948864194 | | down |
| RNU7-195P | -0.187643855 | 0.948864194 | | down |
| MIR3177 | -0.187643855 | 0.948864194 | | down |
| AC003009.1 | -0.187643855 | 0.948864194 | | down |
| AC135048.13 | -0.187643855 | 0.948864194 | | down |
| RP11-249C24.11 | -0.187643855 | 0.948864194 | | down |
| RP11-403P17.2 | -0.187643855 | 0.948864194 | | down |
| SRP68P2 | -0.187643855 | 0.948864194 | | down |
| Y_RNA | -0.187643855 | 0.948864194 | | down |
| CTD-2033D24.2 | -0.187643855 | 0.948864194 | | down |
| RNU6-288P | -0.187643855 | 0.948864194 | | down |
| RP11-666A8.9 | -0.187643855 | 0.948864194 | | down |
| RNU6-625P | -0.187643855 | 0.948864194 | | down |
| SNRPCP4 | -0.187643855 | 0.948864194 | | down |
| RN7SL745P | -0.187643855 | 0.948864194 | | down |
| RP11-2N1.3 | -0.187643855 | 0.948864194 | | down |
| PLCB1-IT1 | -0.187643855 | 0.948864194 | | down |
| NA | -0.187643855 | 0.948864194 | | down |
| ANKRD62P1 | -0.187643855 | 0.948864194 | | down |
| L22NC03-22A12.1 | -0.187643855 | 0.948864194 | | down |
| CTA-929C8.8 | -0.187643855 | 0.948864194 | | down |
| GS1-600G8.3 | -0.187643855 | 0.948864194 | | down |
| RP11-382F24.1 | -0.187643855 | 0.948864194 | | down |
| SPANXN1 | -0.187643855 | 0.948864194 | | down |
| HIST1H4C | -0.187637294 | 0.6866828 | 0.783275676 | down |
| FTX | -0.187635748 | 0.423332062 | 0.558935991 | down |
| SSH3 | -0.187575913 | 0.274660071 | 0.411003959 | down |
| ZNF324 | -0.187558697 | 0.114179068 | 0.222075219 | down |
| RPRD2 | -0.187418972 | 0.302844747 | 0.440762854 | down |
| RPL5P6 | -0.187418672 | 0.750738149 | 0.831405164 | down |
| AP000704.5 | -0.187218763 | 0.578198819 | 0.694717269 | down |
| SDHAP3 | -0.187214216 | 0.334139808 | 0.472396423 | down |
| SMG9 | -0.187196799 | 0.320380266 | 0.45918828 | down |
| ZNF280C | -0.187070772 | 0.603871978 | 0.716650723 | down |
| C12orf10 | -0.187056403 | 0.378184374 | 0.516344982 | down |
| NA | -0.187037661 | 0.772100527 | 0.846759928 | down |
| RPL9P3 | -0.186951297 | 0.676546073 | 0.775452092 | down |
| RP11-388C12.5 | -0.186851952 | 0.760433758 | | down |
| IVD | -0.186726614 | 0.211064523 | 0.341574463 | down |
| LINCR-0001 | -0.186636427 | 0.642087175 | 0.747852959 | down |
| RP11-411H5.1 | -0.186473046 | 0.875077281 | 0.920450297 | down |
| PDCD6IP | -0.186469231 | 0.255440405 | 0.390537455 | down |
| RPS15AP38 | -0.186455281 | 0.683722927 | 0.781077562 | down |
| OBP2B | -0.186444817 | 0.853319234 | | down |
| LRRN4 | -0.186419663 | 0.807881562 | 0.873096083 | down |

| | | | | |
|---|---|---|---|---|
| XKR7 | -0.186410809 | 0.743162025 | 0.825753907 | down |
| F10 | -0.186286741 | 0.567522073 | 0.685487263 | down |
| MEIS1-AS2 | -0.186021383 | 0.757173666 | 0.835851177 | down |
| C10orf76 | -0.186009134 | 0.002686724 | 0.013300369 | down |
| PHOSPHO1 | -0.186008468 | 0.428761895 | 0.564121847 | down |
| COL11A2 | -0.185884127 | 0.525061453 | 0.648957296 | down |
| LINC00460 | -0.185850169 | 0.763012834 | 0.840351831 | down |
| UQCC2 | -0.185840091 | 0.419594432 | 0.55536478 | down |
| PYGO1 | -0.18583789 | 0.590309468 | 0.705443808 | down |
| PCNT | -0.185832222 | 0.152757851 | 0.272258224 | down |
| SENP3 | -0.185780351 | 0.238451686 | 0.371887586 | down |
| ZFYVE1 | -0.185552359 | 0.073567396 | 0.162149428 | down |
| FNIP2 | -0.185430268 | 0.342122698 | 0.480518034 | down |
| AP001350.4 | -0.185428586 | 0.649797615 | 0.753969009 | down |
| CLCN6 | -0.18541332 | 0.183382755 | 0.309133914 | down |
| LINC01259 | -0.185406198 | 0.863931609 | | down |
| RAB1C | -0.185404302 | 0.79783502 | | down |
| TTLL3 | -0.185379646 | 0.389610275 | 0.527248822 | down |
| HAUS4 | -0.185361224 | 0.337685436 | 0.476027381 | down |
| PARN | -0.185311854 | 0.073946212 | 0.162674086 | down |
| RP1-292L20.3 | -0.185304369 | 0.608760738 | 0.720857938 | down |
| TMEM38B | -0.185285721 | 0.356030239 | 0.494524849 | down |
| BAHCC1 | -0.185202572 | 0.384254814 | 0.521952782 | down |
| RP11-51F16.1 | -0.185139233 | 0.546566455 | 0.667703757 | down |
| ZSCAN30 | -0.185112868 | 0.436013699 | 0.570685735 | down |
| AVL9 | -0.185084942 | 0.182313825 | 0.307894129 | down |
| LAMP1 | -0.185036838 | 0.237542206 | 0.370730169 | down |
| SMIM14 | -0.185023597 | 0.241322265 | 0.3751571 | down |
| NODAL | -0.18498261 | 0.784217882 | 0.855551838 | down |
| AC138035.2 | -0.184930675 | 0.79098719 | | down |
| CLHC1 | -0.184910136 | 0.440014213 | 0.573834587 | down |
| C9orf66 | -0.184877736 | 0.59287026 | 0.707703686 | down |
| SPIN2A | -0.184850017 | 0.71705742 | 0.806320575 | down |
| ACP2 | -0.184773981 | 0.273228385 | 0.409460974 | down |
| ZNF846 | -0.184604932 | 0.255479466 | 0.390537553 | down |
| LINC00378 | -0.18456174 | 0.914512995 | 0.946684065 | down |
| PPM1J | -0.184545245 | 0.499766703 | 0.626878375 | down |
| RP3-435K13.1 | -0.18450559 | 0.949718276 | | down |
| RP11-348J12.2 | -0.18450559 | 0.949718276 | | down |
| NA | -0.18450559 | 0.949718276 | | down |
| NA | -0.18450559 | 0.949718276 | | down |
| GAGE2A | -0.18450559 | 0.949718276 | | down |
| NA | -0.184505174 | 0.947533386 | | down |
| Y_RNA | -0.184505006 | 0.9465643 | | down |
| CNN3P1 | -0.184504061 | 0.939899062 | | down |
| RP11-803B1.3 | -0.184504043 | 0.9397517 | | down |
| PSMG4 | -0.184503044 | 0.132506744 | 0.24652955 | down |
| NA | -0.184502365 | 0.917024516 | | down |
| RP1-124C6.1 | -0.184502341 | 0.916496387 | | down |
| RP11-559M23.1 | -0.184502301 | 0.91556845 | | down |
| NA | -0.184502291 | 0.915336944 | | down |

| | | | | |
|---|---|---|---|---|
| NA | -0.184502027 | 0.908335866 | | down |
| RNU7-61P | -0.18450202 | 0.90811486 | | down |
| NA | -0.184502015 | 0.907975636 | | down |
| VN2R10P | -0.184502012 | 0.907883158 | | down |
| KRT77 | -0.184501997 | 0.907431357 | | down |
| RP11-15B17.4 | -0.18450178 | 0.89990649 | | down |
| RP11-302K17.3 | -0.184501288 | 0.872609908 | | down |
| RP11-495K9.5 | -0.184497168 | 0.861800002 | | down |
| BMP8B | -0.184347952 | 0.502431821 | 0.629328009 | down |
| FAM3D | -0.184226765 | 0.595995856 | 0.710136804 | down |
| VDAC2 | -0.184068463 | 0.192058603 | 0.319920361 | down |
| PTPN4 | -0.183977567 | 0.210941086 | 0.341479281 | down |
| RPL35 | -0.183975954 | 0.329349197 | 0.467802916 | down |
| DMWD | -0.183960997 | 0.230724475 | 0.363154135 | down |
| RBM44 | -0.183954688 | 0.728780092 | 0.815655978 | down |
| NA | -0.183946689 | 0.899360734 | | down |
| PDRG1 | -0.183945668 | 0.211183535 | 0.341687658 | down |
| GPM6B | -0.183776051 | 0.557104717 | 0.676706319 | down |
| LINC01374 | -0.183704012 | 0.741196383 | 0.824179434 | down |
| LEF1-AS1 | -0.18350126 | 0.684951936 | 0.781895055 | down |
| PRH2 | -0.18348166 | 0.65073994 | 0.754712633 | down |
| PEX19 | -0.183472142 | 0.175233796 | 0.299687524 | down |
| DOT1L | -0.183272269 | 0.243969714 | 0.377932818 | down |
| CACNA2D2 | -0.183188836 | 0.566939104 | 0.685059535 | down |
| IFI27L2 | -0.183087345 | 0.468579323 | 0.599573986 | down |
| CTC-367J11.1 | -0.183078506 | 0.850884428 | | down |
| PKIA-AS1 | -0.183005267 | 0.822064866 | 0.883343108 | down |
| TSPY26P | -0.182788498 | 0.376012816 | 0.514221359 | down |
| GRIN2C | -0.182766994 | 0.575151607 | 0.692163223 | down |
| ZFAND2A | -0.182416042 | 0.466785625 | 0.597922115 | down |
| KIAA0586 | -0.182322619 | 0.270468643 | 0.40646058 | down |
| XYLT1 | -0.182266102 | 0.415741545 | 0.552186085 | down |
| LINC00863 | -0.182239923 | 0.38246227 | 0.520199939 | down |
| NUP214 | -0.182192395 | 0.039618575 | 0.103217175 | down |
| Y_RNA | -0.182153905 | 0.851460592 | | down |
| RP11-927P21.1 | -0.182145724 | 0.721790115 | 0.810310379 | down |
| QRSL1 | -0.182134277 | 0.335485364 | 0.473674622 | down |
| TPRA1 | -0.182113583 | 0.265560176 | 0.401171055 | down |
| RPS24 | -0.182059824 | 0.259507653 | 0.394746996 | down |
| C10orf54 | -0.181800438 | 0.385645191 | 0.523391793 | down |
| COPE | -0.181783993 | 0.398099508 | 0.535083672 | down |
| INAFM2 | -0.181747319 | 0.424788627 | 0.560465043 | down |
| SLC25A1P1 | -0.181740049 | 0.824108519 | | down |
| BROX | -0.181696574 | 0.096464958 | 0.197029657 | down |
| FAIM2 | -0.181636106 | 0.476007038 | 0.606006841 | down |
| NA | -0.181626069 | 0.782155372 | 0.854201236 | down |
| NFE4 | -0.181617523 | 0.788735304 | 0.85898298 | down |
| NA | -0.181498555 | 0.718057827 | 0.807354866 | down |
| PLA2G4C | -0.181213548 | 0.292927779 | 0.430247968 | down |
| KBTBD7 | -0.181203769 | 0.391868427 | 0.529446194 | down |
| SNHG17 | -0.18109177 | 0.29631504 | 0.433781078 | down |

| | | | | |
|---|---|---|---|---|
| HMGCLL1 | -0.181067729 | 0.710087527 | 0.800881059 | down |
| SIDT1 | -0.181024598 | 0.309235788 | 0.44765732 | down |
| UBE2D3P1 | -0.180950594 | 0.717019775 | 0.806308422 | down |
| CPT1C | -0.180946561 | 0.534634621 | 0.657305011 | down |
| SENP7 | -0.180924665 | 0.43985555 | 0.573736638 | down |
| AP000223.42 | -0.18076975 | 0.706179589 | 0.797881222 | down |
| PTGER4 | -0.180730329 | 0.440325426 | 0.57405672 | down |
| SPRED2 | -0.180669552 | 0.476144289 | 0.606106721 | down |
| GALNT10 | -0.180665541 | 0.128392166 | 0.241193913 | down |
| PSMB6 | -0.180649813 | 0.274614808 | 0.410985171 | down |
| HSCB | -0.180572482 | 0.427865402 | 0.563276805 | down |
| DPCD | -0.180454055 | 0.418737825 | 0.55477176 | down |
| PDPR | -0.180364162 | 0.177451039 | 0.302491243 | down |
| MCUR1 | -0.180286666 | 0.256529605 | 0.391565079 | down |
| PSMB7 | -0.180259032 | 0.134281527 | 0.248696474 | down |
| GHITM | -0.180231937 | 0.302737088 | 0.440648858 | down |
| MED9 | -0.180179737 | 0.142091968 | 0.25892924 | down |
| CTB-193M12.3 | -0.180164335 | 0.833727298 | 0.891529044 | down |
| OXNAD1 | -0.180140769 | 0.284749475 | 0.421636926 | down |
| NPPA | -0.180094238 | 0.702959032 | 0.795618805 | down |
| SLC25A44 | -0.180084943 | 0.159616947 | 0.280829543 | down |
| GSKIP | -0.180008152 | 0.349209805 | 0.487893386 | down |
| MIR325HG | -0.179983991 | 0.863323161 | | down |
| USP7 | -0.179832828 | 0.035185526 | 0.094410461 | down |
| ARHGAP21 | -0.179770426 | 0.322520261 | 0.461155469 | down |
| FAM180B | -0.179723092 | 0.73086081 | 0.817079241 | down |
| ZFP3 | -0.179704223 | 0.447166872 | 0.580458582 | down |
| GALE | -0.179613876 | 0.419205065 | 0.55510657 | down |
| PLEK2 | -0.179503969 | 0.514147932 | 0.639521648 | down |
| RP11-298E9.5 | -0.179503888 | 0.934957397 | | down |
| MKI67 | -0.179467424 | 0.517615653 | 0.642558181 | down |
| C1orf21 | -0.17945814 | 0.382217755 | 0.519985067 | down |
| CAPN1 | -0.179325426 | 0.143150632 | 0.260310297 | down |
| SMAD7 | -0.178907863 | 0.403342785 | 0.539971064 | down |
| RP11-203B9.4 | -0.178742844 | 0.643965821 | 0.749314838 | down |
| SLC12A2 | -0.178646845 | 0.517257662 | 0.642168975 | down |
| RPS8 | -0.178590895 | 0.299873217 | 0.437476494 | down |
| PPIE | -0.178358457 | 0.133894463 | 0.248117267 | down |
| Y_RNA | -0.178234805 | 0.951425049 | | down |
| ARL5AP3 | -0.178234805 | 0.951425049 | | down |
| RP3-333A15.1 | -0.178234805 | 0.951425049 | | down |
| CHIAP2 | -0.178234805 | 0.951425049 | | down |
| RNU6-152P | -0.178234805 | 0.951425049 | | down |
| RP11-176D17.3 | -0.178234805 | 0.951425049 | | down |
| NA | -0.178234805 | 0.951425049 | | down |
| RP11-439E19.6 | -0.178234805 | 0.951425049 | | down |
| AC106875.1 | -0.178234805 | 0.951425049 | | down |
| SDHCP3 | -0.178234805 | 0.951425049 | | down |
| RP11-391A7.1 | -0.178234805 | 0.951425049 | | down |
| AC009299.4 | -0.178234805 | 0.951425049 | | down |
| MIR4447 | -0.178234805 | 0.951425049 | | down |

| | | | | |
|---|---|---|---|---|
| RP11-588P8.1 | -0.178234805 | 0.951425049 | | down |
| MIR4459 | -0.178234805 | 0.951425049 | | down |
| RP11-185E12.2 | -0.178234805 | 0.951425049 | | down |
| AC010468.2 | -0.178234805 | 0.951425049 | | down |
| LINC01623 | -0.178234805 | 0.951425049 | | down |
| UBD | -0.178234805 | 0.951425049 | | down |
| NPM1P51 | -0.178234805 | 0.951425049 | | down |
| RNU6-130P | -0.178234805 | 0.951425049 | | down |
| NA | -0.178234805 | 0.951425049 | | down |
| RPL35P6 | -0.178234805 | 0.951425049 | | down |
| NRG1-IT1 | -0.178234805 | 0.951425049 | | down |
| RNU2-46P | -0.178234805 | 0.951425049 | | down |
| RP11-253D19.2 | -0.178234805 | 0.951425049 | | down |
| NA | -0.178234805 | 0.951425049 | | down |
| RP11-477L16.2 | -0.178234805 | 0.951425049 | | down |
| RP11-167N4.5 | -0.178234805 | 0.951425049 | | down |
| TRIM49C | -0.178234805 | 0.951425049 | | down |
| RNU6-275P | -0.178234805 | 0.951425049 | | down |
| Y_RNA | -0.178234805 | 0.951425049 | | down |
| RN7SL387P | -0.178234805 | 0.951425049 | | down |
| RP11-465L8.1 | -0.178234805 | 0.951425049 | | down |
| RPL21P108 | -0.178234805 | 0.951425049 | | down |
| RN7SL683P | -0.178234805 | 0.951425049 | | down |
| RP11-16L14.2 | -0.178234805 | 0.951425049 | | down |
| RP11-313P18.2 | -0.178234805 | 0.951425049 | | down |
| MIR4312 | -0.178234805 | 0.951425049 | | down |
| RP11-692C24.2 | -0.178234805 | 0.951425049 | | down |
| RP11-713H12.2 | -0.178234805 | 0.951425049 | | down |
| IL6STP1 | -0.178234805 | 0.951425049 | | down |
| RN7SL404P | -0.178234805 | 0.951425049 | | down |
| RNA5SP452 | -0.178234805 | 0.951425049 | | down |
| AC002398.11 | -0.178234805 | 0.951425049 | | down |
| NDUFB4P10 | -0.178234805 | 0.951425049 | | down |
| APOBEC3B-AS1 | -0.178234805 | 0.951425049 | | down |
| CHCHD2P1 | -0.178234805 | 0.951425049 | | down |
| AC069257.8 | -0.178234041 | 0.948490563 | | down |
| RP11-256I23.2 | -0.17823404 | 0.948484222 | | down |
| RP11-1021O19.2 | -0.178234037 | 0.948474502 | | down |
| RP11-552M6.1 | -0.178233952 | 0.948111914 | | down |
| RNU6-681P | -0.178233907 | 0.947920245 | | down |
| CTA-250D13.1 | -0.178233907 | 0.947918895 | | down |
| RP11-1012A1.10 | -0.178233879 | 0.947797211 | | down |
| CTD-3060P21.1 | -0.178233707 | 0.947026801 | | down |
| SETP10 | -0.178233696 | 0.946977801 | | down |
| AC007128.1 | -0.178233545 | 0.946273987 | | down |
| RP11-322E11.3 | -0.178232475 | 0.940315171 | | down |
| RP11-574H6.1 | -0.178232363 | 0.939570109 | | down |
| RNA5SP474 | -0.178232347 | 0.939464399 | | down |
| LLNLR-268E12.1 | -0.178219016 | 0.718644109 | 0.807953583 | down |
| DNMT3A | -0.178184495 | 0.108350327 | 0.214148887 | down |
| ZCCHC14 | -0.177952834 | 0.427570368 | 0.562999007 | down |

| | | | | |
|---|---|---|---|---|
| VPS13D | -0.177946563 | 0.142866433 | 0.259949073 | down |
| AC009506.1 | -0.177915419 | 0.508272428 | 0.634580154 | down |
| AFF3 | -0.177875908 | 0.562537045 | 0.681573254 | down |
| ILKAP | -0.177855126 | 0.303532015 | 0.441549212 | down |
| FBXO2 | -0.177824006 | 0.45958751 | 0.59154516 | down |
| ZNF419 | -0.177798017 | 0.413460518 | 0.550035768 | down |
| NEK8 | -0.177774413 | 0.418902109 | 0.554894422 | down |
| CTDNEP1 | -0.177731953 | 0.244702712 | 0.378707478 | down |
| FTLP3 | -0.177665055 | 0.442035112 | 0.575587012 | down |
| TTLL4 | -0.17762556 | 0.111287609 | 0.218217264 | down |
| CAPN15 | -0.177573616 | 0.216729018 | 0.348017457 | down |
| TMA7 | -0.177474343 | 0.378393292 | 0.516489395 | down |
| SPECC1L | -0.177403791 | 0.178861219 | 0.304011454 | down |
| RP11-999E24.3 | -0.17735925 | 0.669785085 | 0.769622144 | down |
| THRA | -0.176970302 | 0.448969911 | 0.581892585 | down |
| AK8 | -0.176966743 | 0.564015826 | 0.682347892 | down |
| TAX1BP3 | -0.17696525 | 0.574641411 | 0.691715476 | down |
| LINC00900 | -0.176914277 | 0.660629093 | 0.762414645 | down |
| RP11-644K8.1 | -0.17688925 | 0.849812983 | | down |
| ORAI1 | -0.176882597 | 0.374867198 | 0.513132932 | down |
| ZNF383 | -0.176850133 | 0.142176435 | 0.259041132 | down |
| F7 | -0.176735254 | 0.723962146 | 0.811745427 | down |
| DHODH | -0.176651188 | 0.104382824 | 0.208128323 | down |
| PIN1 | -0.176620667 | 0.255540121 | 0.390610399 | down |
| FGD4 | -0.176401644 | 0.403995315 | 0.540603743 | down |
| LINC00662 | -0.17635164 | 0.278801824 | 0.415018679 | down |
| FAM21A | -0.176251763 | 0.167314209 | 0.290442908 | down |
| RPL29P33 | -0.176198657 | 0.760422747 | 0.838267448 | down |
| HGH1 | -0.176153441 | 0.390791523 | 0.528442727 | down |
| TAPBP | -0.176139589 | 0.426523727 | 0.562132173 | down |
| HMP19 | -0.176109651 | 0.655638605 | 0.758344803 | down |
| ZBTB8OS | -0.176023883 | 0.087660477 | 0.183992691 | down |
| ADAM15 | -0.175878271 | 0.469474719 | 0.600321504 | down |
| HMGB1P24 | -0.175849937 | 0.691871659 | 0.787370913 | down |
| ROCK2 | -0.17567068 | 0.352931668 | 0.49170141 | down |
| KRT18P25 | -0.175667429 | 0.842057124 | 0.897819726 | down |
| RP11-39404.3 | -0.175523018 | 0.728444539 | 0.815371548 | down |
| BOLA1 | -0.175121674 | 0.181518008 | 0.307067964 | down |
| LINC01506 | -0.175111016 | 0.647447092 | 0.752083995 | down |
| TMPRSS11B | -0.175095913 | 0.948915755 | | down |
| RP11-429E11.2 | -0.175093789 | 0.928186449 | | down |
| RP11-526N18.1 | -0.175093706 | 0.926782674 | | down |
| RP11-1212A22.7 | -0.175093701 | 0.92668318 | | down |
| RP11-788M5.3 | -0.175093363 | 0.919910257 | | down |
| MIR320B2 | -0.175092868 | 0.905412121 | | down |
| AC009303.1 | -0.175092854 | 0.904912574 | | down |
| RP5-1116H23.6 | -0.175092847 | 0.904624979 | | down |
| U3 | -0.175092679 | 0.897384531 | | down |
| RP11-126K15.1 | -0.175092668 | 0.896848601 | | down |
| IGHV3OR15-7 | -0.175092552 | 0.890670345 | | down |
| RP4-742J24.2 | -0.175092509 | 0.888076839 | | down |

| | | | | |
|---|---|---|---|---|
| NA | -0.175092215 | 0.863439325 | | down |
| RP11-302I18.1 | -0.175089179 | 0.908119286 | 0.942371367 | down |
| STK35 | -0.175052988 | 0.333282762 | 0.47156199 | down |
| TMEM11 | -0.175023782 | 0.080502417 | 0.173006486 | down |
| AC010136.2 | -0.175014294 | 0.738547967 | 0.822299701 | down |
| RP11-12M9.3 | -0.174998738 | 0.5365723 | 0.658934072 | down |
| RP11-1246C19.1 | -0.174985732 | 0.581655777 | 0.697584253 | down |
| RP11-161M6.6 | -0.174769595 | 0.712696147 | 0.802983676 | down |
| RP11-70F11.8 | -0.174750298 | 0.874867512 | | down |
| RPS7P1 | -0.174739849 | 0.35883938 | 0.497116722 | down |
| FAM35A | -0.174684935 | 0.267999198 | 0.403779546 | down |
| RPS11P5 | -0.174549554 | 0.401637311 | 0.538489979 | down |
| SYT1 | -0.174517675 | 0.674248626 | 0.773527025 | down |
| RP11-638I2.4 | -0.17451304 | 0.828273387 | | down |
| NUTM2HP | -0.174464906 | 0.728472753 | 0.815372751 | down |
| RP4-61404.13 | -0.174441921 | 0.508236759 | 0.634561991 | down |
| BAHD1 | -0.174388001 | 0.284987158 | 0.421926486 | down |
| PARPBP | -0.174345777 | 0.546998107 | 0.66792634 | down |
| CTD-2516F10.2 | -0.174273255 | 0.574674315 | 0.691727369 | down |
| RP11-79P5.9 | -0.174244836 | 0.66728292 | 0.767646196 | down |
| NRIP1 | -0.174200419 | 0.639871793 | 0.74599566 | down |
| HCCS | -0.174021339 | 0.336106344 | 0.474343108 | down |
| AMBRA1 | -0.173991783 | 0.04844096 | 0.119635034 | down |
| RP11-467P9.1 | -0.173959731 | 0.737287765 | 0.821535942 | down |
| SRL | -0.173948696 | 0.637458481 | 0.7440574 | down |
| ABHD5 | -0.173883948 | 0.261286889 | 0.39642982 | down |
| TRMT2B | -0.173872554 | 0.274817016 | 0.411107241 | down |
| COL6A2 | -0.173623741 | 0.526259868 | 0.64985415 | down |
| ECHDC3 | -0.173614666 | 0.437596818 | 0.572088195 | down |
| NA | -0.173614148 | 0.701647295 | 0.794583164 | down |
| UBE2Q2P9 | -0.173560046 | 0.879440294 | | down |
| RP11-596C23.6 | -0.173311554 | 0.637121276 | 0.743831733 | down |
| ASB7 | -0.173307173 | 0.340669849 | 0.479054785 | down |
| HMGB1P10 | -0.173210384 | 0.385259413 | 0.52303361 | down |
| OMG | -0.173183472 | 0.634362785 | 0.741673951 | down |
| NUDT2 | -0.173036431 | 0.274221865 | 0.410581168 | down |
| ZNF7 | -0.172788389 | 0.065875988 | 0.149452198 | down |
| XG | -0.172761883 | 0.739259484 | 0.822817469 | down |
| AP001372.2 | -0.172723822 | 0.389768167 | 0.527438735 | down |
| JOSD1 | -0.172693042 | 0.161007549 | 0.282569707 | down |
| RP11-73M18.8 | -0.172656193 | 0.380834437 | 0.518784394 | down |
| FAM32A | -0.172613903 | 0.176115185 | 0.30091018 | down |
| ADAT3 | -0.172578244 | 0.50546676 | 0.631943878 | down |
| PRDX2 | -0.172571838 | 0.339733704 | 0.478116597 | down |
| BEX1 | -0.172569159 | 0.617728266 | 0.728404884 | down |
| TTTY10 | -0.172494608 | 0.807613308 | 0.872960396 | down |
| ZNF195 | -0.172400477 | 0.11431914 | 0.222218183 | down |
| FAM83A | -0.1723932 | 0.723048351 | 0.811161243 | down |
| MRPL1 | -0.172280179 | 0.131599184 | 0.245408621 | down |
| HUNK | -0.172240517 | 0.517478651 | 0.642416784 | down |
| DVL3 | -0.172183683 | 0.150058882 | 0.26902787 | down |

| | | | | |
|---|---|---|---|---|
| MCHR1 | -0.172169649 | 0.709409381 | 0.800356569 | down |
| AMFR | -0.172102899 | 0.164587619 | 0.287124387 | down |
| GPR141 | -0.172082607 | 0.718732185 | 0.808022368 | down |
| PLEKHA3P1 | -0.171825771 | 0.745299164 | 0.827088542 | down |
| CCDC91 | -0.171808032 | 0.275725064 | 0.412031028 | down |
| MACROD2 | -0.17178926 | 0.589667475 | 0.704832689 | down |
| C14orf79 | -0.171757093 | 0.439147762 | 0.573307238 | down |
| CCNE2 | -0.171754354 | 0.63962172 | 0.745819878 | down |
| C1orf147 | -0.171732965 | 0.763575241 | 0.840698937 | down |
| HRNR | -0.171633328 | 0.818796367 | 0.881212138 | down |
| RP11-2B6.2 | -0.171569021 | 0.456873286 | 0.589086349 | down |
| MAP1LC3C | -0.171491775 | 0.699175991 | 0.792980124 | down |
| CD40LG | -0.171411212 | 0.647059356 | 0.751837076 | down |
| MEPCE | -0.171325893 | 0.252064894 | 0.386971778 | down |
| PCP2 | -0.171296018 | 0.659856914 | 0.761786856 | down |
| SLC8A1 | -0.171241947 | 0.559100821 | 0.678691922 | down |
| UQCRH | -0.171128026 | 0.234552294 | 0.367348075 | down |
| RP1-186E20.1 | -0.171126682 | 0.891353191 | | down |
| TMEM208 | -0.171058428 | 0.546276966 | 0.667582435 | down |
| ALG1L9P | -0.171020519 | 0.694187792 | 0.789139885 | down |
| RPS20P14 | -0.170954282 | 0.551001597 | 0.671246937 | down |
| AC115617.2 | -0.170947394 | 0.680249426 | 0.77836058 | down |
| CTC-378H22.1 | -0.170896123 | 0.713725354 | 0.803656567 | down |
| FASTKD3 | -0.170604925 | 0.396462289 | 0.53338905 | down |
| NA | -0.170553563 | 0.776533987 | 0.850290699 | down |
| NARF | -0.17039434 | 0.244700977 | 0.378707478 | down |
| ZNF592 | -0.170388965 | 0.148403629 | 0.267096306 | down |
| PKN2 | -0.170324705 | 0.376990955 | 0.515171649 | down |
| CTD-2012K14.8 | -0.170265879 | 0.687582144 | 0.783825382 | down |
| EVA1A | -0.170262357 | 0.477411329 | 0.607228566 | down |
| RPL36AL | -0.170201742 | 0.371487641 | 0.509796497 | down |
| PEX7 | -0.170080012 | 0.321139176 | 0.459881097 | down |
| EIF2B4 | -0.170059083 | 0.353926294 | 0.49254393 | down |
| AC093390.1 | -0.169990523 | 0.87886813 | 0.922323737 | down |
| CNIH4 | -0.169947782 | 0.16914061 | 0.292638093 | down |
| RP9 | -0.169925571 | 0.42140049 | 0.557022144 | down |
| RP11-982M15.6 | -0.169904065 | 0.777372092 | 0.850833047 | down |
| PSMA2 | -0.169869437 | 0.286153498 | 0.423236139 | down |
| MYO1E | -0.169784104 | 0.339192515 | 0.477696522 | down |
| LINC00304 | -0.169742414 | 0.781013359 | 0.853357289 | down |
| VPS4B | -0.169622285 | 0.237088197 | 0.37016462 | down |
| KMT5C | -0.169543167 | 0.190733676 | 0.318284969 | down |
| KDELR2 | -0.169536365 | 0.369034086 | 0.507402868 | down |
| TCTA | -0.169267744 | 0.275515218 | 0.411840351 | down |
| PERP | -0.16926533 | 0.485056645 | 0.613860229 | down |
| EFHD1 | -0.169155614 | 0.652727209 | 0.75614175 | down |
| NA | -0.169095744 | 0.872055566 | 0.918365082 | down |
| SLC40A1 | -0.169089019 | 0.517803632 | 0.642640239 | down |
| EFHB | -0.169076359 | 0.628204692 | 0.736681568 | down |
| MATN1 | -0.169041981 | 0.684365623 | 0.781470586 | down |
| PDZD2 | -0.169017861 | 0.530507309 | 0.653746271 | down |

| | | | | |
|---|---|---|---|---|
| MIR762HG | -0.1689915 | 0.597950844 | 0.71176053 | down |
| HOXB3 | -0.168950883 | 0.396424717 | 0.533371437 | down |
| RCN3 | -0.168929773 | 0.385302612 | 0.523068622 | down |
| TPPP3 | -0.16889799 | 0.540111225 | 0.66195242 | down |
| TCL1A | -0.168875211 | 0.766841563 | 0.843208576 | down |
| IFI35 | -0.16884854 | 0.411228194 | 0.547915201 | down |
| RP11-242O24.3 | -0.168825755 | 0.953986406 | | down |
| OR10J3 | -0.168825755 | 0.953986406 | | down |
| ATP5BP1 | -0.168825755 | 0.953986406 | | down |
| NA | -0.168825755 | 0.953986406 | | down |
| RP11-297K7.1 | -0.168825755 | 0.953986406 | | down |
| FAM159B | -0.168825755 | 0.953986406 | | down |
| HNRNPA3P7 | -0.168825755 | 0.953986406 | | down |
| RP1-111D6.4 | -0.168825755 | 0.953986406 | | down |
| S100A11P1 | -0.168825755 | 0.953986406 | | down |
| Metazoa_SRP | -0.168825755 | 0.953986406 | | down |
| KB-1205A7.1 | -0.168825755 | 0.953986406 | | down |
| NA | -0.168825755 | 0.953986406 | | down |
| KRT18P67 | -0.168825755 | 0.953986406 | | down |
| RP11-843B15.2 | -0.168825755 | 0.953986406 | | down |
| LINC00368 | -0.168825755 | 0.953986406 | | down |
| AL132709.1 | -0.168825755 | 0.953986406 | | down |
| LINC00226 | -0.168825755 | 0.953986406 | | down |
| RP11-178C3.6 | -0.168825755 | 0.953986406 | | down |
| MIR3975 | -0.168825755 | 0.953986406 | | down |
| CTD-2616J11.11 | -0.168825755 | 0.953986406 | | down |
| MIMT1 | -0.168825755 | 0.953986406 | | down |
| NA | -0.168825755 | 0.953986406 | | down |
| H2BFM | -0.168825755 | 0.953986406 | | down |
| CTXN2 | -0.168824599 | 0.949439525 | | down |
| RP3-403L10.3 | -0.168824553 | 0.94923243 | | down |
| RP11-143N13.3 | -0.168824535 | 0.949147562 | | down |
| RFPL3 | -0.168824456 | 0.948776567 | | down |
| AC092580.1 | -0.168823258 | 0.941979709 | | down |
| RP11-466F5.3 | -0.168823204 | 0.941607067 | | down |
| RP5-1068H6.6 | -0.168823186 | 0.941485104 | | down |
| snoU13 | -0.168822352 | 0.934560079 | | down |
| NA | -0.168822304 | 0.934079703 | | down |
| RP11-385E5.5 | -0.16882228 | 0.933837538 | | down |
| RP11-956E11.1 | -0.168822237 | 0.933395487 | | down |
| CD99 | -0.168822215 | 0.933163129 | | down |
| RNU6-1215P | -0.168821562 | 0.925052899 | | down |
| MTCO2P11 | -0.168821101 | 0.917162228 | | down |
| RP11-133N21.10 | -0.168808688 | 0.842483967 | | down |
| ZNF2 | -0.168735037 | 0.425992009 | 0.561608177 | down |
| RP11-478C1.7 | -0.168729531 | 0.868104962 | 0.915581753 | down |
| NUBPL | -0.168684109 | 0.296698253 | 0.434088255 | down |
| UCK1 | -0.168518327 | 0.058338697 | 0.136974666 | down |
| TSR3 | -0.168456209 | 0.270344613 | 0.406349933 | down |
| FAR2 | -0.168291732 | 0.486385573 | 0.615153363 | down |
| KRT8P46 | -0.16810463 | 0.848254809 | 0.90209081 | down |

| | | | | |
|---|---|---|---|---|
| IL21-AS1 | -0.168090758 | 0.777598282 | 0.85095347 | down |
| RP11-25705.2 | -0.167983712 | 0.325658153 | 0.46416213 | down |
| BCORL1 | -0.167954226 | 0.398279 | 0.535243079 | down |
| ITIH3 | -0.167894114 | 0.657956018 | 0.760147302 | down |
| RPL39P5 | -0.16779626 | 0.813317292 | 0.877137796 | down |
| RP11-1C8.6 | -0.167774295 | 0.776145391 | 0.850099505 | down |
| TOMM22 | -0.167770763 | 0.135324251 | 0.250118843 | down |
| PINX1 | -0.167728804 | 0.421830012 | 0.557382994 | down |
| REEP3 | -0.167709154 | 0.447087061 | 0.580458582 | down |
| SNRNP35 | -0.167543954 | 0.313964847 | 0.452605915 | down |
| CBX3P2 | -0.167543588 | 0.65464739 | 0.757663475 | down |
| TMEM134 | -0.167423651 | 0.294148883 | 0.431535184 | down |
| RP11-178F10.1 | -0.167347937 | 0.915772148 | | down |
| ATPAF1 | -0.167222889 | 0.126393729 | 0.238573424 | down |
| PDLIM1 | -0.167093327 | 0.253315847 | 0.388257387 | down |
| WRB | -0.167027274 | 0.232805185 | 0.365471783 | down |
| ABTB1 | -0.167018297 | 0.393405214 | 0.530806406 | down |
| SMARCA4 | -0.166988768 | 0.060004008 | 0.13970555 | down |
| PRKAG2-AS1 | -0.166916124 | 0.446100788 | 0.579448857 | down |
| TMEM170A | -0.166915343 | 0.382696114 | 0.520447313 | down |
| RP11-672L10.6 | -0.16690249 | 0.675168812 | 0.774316593 | down |
| HNRNPA1P8 | -0.166842626 | 0.792158636 | | down |
| RP11-341N2.1 | -0.166839049 | 0.728998892 | 0.815748918 | down |
| RP11-387M24.5 | -0.166775296 | 0.768067621 | 0.844031588 | down |
| GAPDHP1 | -0.166728519 | 0.503714521 | 0.63035651 | down |
| RP11-477H21.2 | -0.166722227 | 0.68537576 | 0.782208368 | down |
| SLC5A6 | -0.166638043 | 0.323971635 | 0.462350551 | down |
| AGL | -0.166629519 | 0.178752975 | 0.303844661 | down |
| RPS14 | -0.166607321 | 0.286141336 | 0.423236139 | down |
| GABARAP | -0.166566392 | 0.682759728 | 0.780270649 | down |
| YOD1 | -0.166565473 | 0.310131088 | 0.448635402 | down |
| TK1 | -0.166336516 | 0.484351208 | 0.613239616 | down |
| ERICH6 | -0.166287353 | 0.829737084 | 0.888784261 | down |
| MRPL34 | -0.166060277 | 0.193722044 | 0.321841689 | down |
| CCDC106 | -0.165900735 | 0.349604482 | 0.488285857 | down |
| CSNK2B | -0.165873331 | 0.598057399 | 0.711859137 | down |
| HADHAP1 | -0.165861206 | 0.804655655 | 0.870797295 | down |
| GPR146 | -0.165821021 | 0.711127359 | 0.801632536 | down |
| P4HTM | -0.165773114 | 0.353725853 | 0.492462444 | down |
| KIRREL-IT1 | -0.165754665 | 0.890574086 | | down |
| RAET1M | -0.16568749 | 0.95484082 | | down |
| MIR4472-2 | -0.16568749 | 0.95484082 | | down |
| MTOR-AS1 | -0.165685928 | 0.944897818 | | down |
| NA | -0.165684849 | 0.932285402 | | down |
| RP11-473E2.2 | -0.165684473 | 0.925319529 | | down |
| RP11-271C24.3 | -0.165683981 | 0.911825599 | | down |
| RP11-474L23.3 | -0.165683415 | 0.88069606 | | down |
| XXYLT1-AS1 | -0.165683405 | 0.879813726 | | down |
| RPL12P41 | -0.165683312 | 0.870499661 | | down |
| HNRNPDP1 | -0.165683207 | 0.856790079 | | down |
| ENPP5 | -0.165588281 | 0.622503732 | 0.732201286 | down |

| | | | | |
|---|---|---|---|---|
| CPSF1 | -0.165585038 | 0.232364186 | 0.365027431 | down |
| B4GALNT1 | -0.165555539 | 0.602261539 | 0.715219799 | down |
| CD79B | -0.165548186 | 0.723676125 | 0.811681413 | down |
| NKPD1 | -0.165545502 | 0.684464421 | 0.781516705 | down |
| RP11-950C14.3 | -0.165332786 | 0.815237503 | | down |
| CBWD1 | -0.165290243 | 0.203177485 | 0.332604359 | down |
| ANO2 | -0.165284356 | 0.656485019 | 0.758943971 | down |
| RP11-755F10.1 | -0.165280974 | 0.632853538 | 0.740503016 | down |
| RP11-1166P10.1 | -0.165202497 | 0.863670988 | 0.912798789 | down |
| AP000355.2 | -0.165150572 | 0.885264247 | | down |
| TYRO3P | -0.165076998 | 0.852110289 | | down |
| PSMD4 | -0.165056621 | 0.343648972 | 0.482029493 | down |
| VSTM1 | -0.16504499 | 0.79113134 | 0.860781153 | down |
| PPIH | -0.164990545 | 0.444484826 | 0.577987054 | down |
| OPHN1 | -0.164853995 | 0.561715987 | 0.680848634 | down |
| ATP4A | -0.164632308 | 0.778607827 | 0.851627114 | down |
| ZMYND12 | -0.164456536 | 0.580391711 | 0.696599895 | down |
| CTNS | -0.164387261 | 0.336070828 | 0.474322918 | down |
| MOSPD3 | -0.164240802 | 0.241320323 | 0.3751571 | down |
| CTA-204B4.2 | -0.16412259 | 0.43528101 | 0.570102561 | down |
| CNOT10 | -0.164106923 | 0.052123743 | 0.125935321 | down |
| TRIM73 | -0.164091552 | 0.654801411 | 0.757726519 | down |
| RPS15AP36 | -0.164089975 | 0.907965624 | | down |
| NA | -0.164068507 | 0.683020371 | 0.780490797 | down |
| PTPRK | -0.164065466 | 0.332597999 | 0.470947982 | down |
| LPGAT1 | -0.164063333 | 0.401058835 | 0.537920163 | down |
| RP11-399E6.4 | -0.164043126 | 0.809694988 | 0.87426689 | down |
| MCOLN2 | -0.164035935 | 0.644044941 | 0.749377879 | down |
| TNF | -0.164002833 | 0.6398514 | 0.74599566 | down |
| 5S_rRNA | -0.163990171 | 0.821619849 | 0.883178595 | down |
| VAPB | -0.163902173 | 0.14070097 | 0.257239698 | down |
| STMN4 | -0.16371986 | 0.734305775 | 0.819500004 | down |
| RP11-507K13.6 | -0.163675986 | 0.693184937 | 0.788268291 | down |
| RP11-352M15.2 | -0.163628977 | 0.330383684 | 0.468813352 | down |
| MAATS1 | -0.16356054 | 0.689066681 | 0.785041119 | down |
| PADI6 | -0.163292917 | 0.93078708 | | down |
| PPCDC | -0.163240251 | 0.444113158 | 0.577641272 | down |
| EAPP | -0.16322968 | 0.231238631 | 0.363734734 | down |
| KDM4B | -0.163158579 | 0.194464594 | 0.322738602 | down |
| KDM5B | -0.163049557 | 0.225767398 | 0.357769698 | down |
| HCFC1R1 | -0.163035527 | 0.436465216 | 0.570956631 | down |
| SLC35C2 | -0.162993185 | 0.32462058 | 0.463056723 | down |
| TRIL | -0.162958689 | 0.641490901 | 0.747372338 | down |
| UBE2D4 | -0.162937314 | 0.275667971 | 0.412013145 | down |
| AC074212.5 | -0.162876872 | 0.678858495 | 0.777242659 | down |
| ZNF736 | -0.162868773 | 0.514404844 | 0.639683652 | down |
| GTF2IP1 | -0.162792401 | 0.613958365 | 0.725126391 | down |
| RP3-507I15.1 | -0.162674384 | 0.902503985 | 0.938177163 | down |
| VPS26B | -0.162646117 | 0.210021562 | 0.340428753 | down |
| ST6GALNAC2 | -0.162640835 | 0.73667205 | 0.821154419 | down |
| NA | -0.162570104 | 0.696046573 | 0.790654589 | down |

| | | | | |
|---|---|---|---|---|
| NUTM2A-AS1 | -0.16252927 | 0.136268658 | 0.251386979 | down |
| ACTG1P14 | -0.162448862 | 0.738979691 | 0.822597474 | down |
| CIT | -0.162432305 | 0.406513026 | 0.543101981 | down |
| PRG4 | -0.162264947 | 0.698952452 | 0.792816381 | down |
| MTG1 | -0.162210892 | 0.585079687 | 0.700798713 | down |
| FAM86EP | -0.161996281 | 0.515851941 | 0.640953504 | down |
| NISCH | -0.16197575 | 0.099457738 | 0.201208977 | down |
| ZNF493 | -0.161970495 | 0.380654202 | 0.518614275 | down |
| RP3-476K8.3 | -0.161904338 | 0.750139677 | 0.831078857 | down |
| RP11-676J12.7 | -0.161673127 | 0.726584134 | 0.813895593 | down |
| ZNF230 | -0.161383028 | 0.508919669 | 0.635177074 | down |
| SCGB3A1 | -0.161348816 | 0.742828514 | 0.825566525 | down |
| PRDM10 | -0.161295485 | 0.493927065 | 0.622020569 | down |
| NDUFB8 | -0.161250565 | 0.594477569 | 0.708749368 | down |
| ELMOD3 | -0.161212172 | 0.047209455 | 0.11727806 | down |
| ALOX15B | -0.161127263 | 0.647915743 | 0.752424745 | down |
| LRRC6 | -0.160979177 | 0.488747984 | 0.617361549 | down |
| TMEM189 | -0.160976458 | 0.295936241 | 0.433455068 | down |
| DBNL | -0.160938904 | 0.249352955 | 0.383926622 | down |
| DZANK1 | -0.160808401 | 0.597601115 | 0.711485156 | down |
| PAWR | -0.160806489 | 0.299239448 | 0.436806431 | down |
| RP11-134G8.7 | -0.160797729 | 0.552475858 | 0.672415826 | down |
| SLC38A9 | -0.160646304 | 0.421640993 | 0.557241982 | down |
| HNRNPKP1 | -0.160560311 | 0.8516133 | | down |
| MAST4 | -0.160557423 | 0.372142005 | 0.510461325 | down |
| DUSP26 | -0.160459402 | 0.582318259 | 0.698326339 | down |
| KDM3A | -0.160178624 | 0.420368653 | 0.556074137 | down |
| RP11-592N21.1 | -0.160106712 | 0.525966883 | 0.649599155 | down |
| MRAP2 | -0.15982375 | 0.578739832 | 0.695200493 | down |
| IL17D | -0.159732636 | 0.611508803 | 0.72310691 | down |
| ING5 | -0.159684747 | 0.101403896 | 0.204084851 | down |
| GRK2 | -0.159628772 | 0.268499779 | 0.40429045 | down |
| TNFRSF18 | -0.159485984 | 0.655193597 | 0.75800802 | down |
| LIG1 | -0.15941886 | 0.358366919 | 0.496782896 | down |
| MIR4711 | -0.159416705 | 0.956548239 | | down |
| RP11-398M15.1 | -0.159416705 | 0.956548239 | | down |
| AC074375.1 | -0.159416705 | 0.956548239 | | down |
| AC010987.6 | -0.159416705 | 0.956548239 | | down |
| RN7SL124P | -0.159416705 | 0.956548239 | | down |
| IQCF1 | -0.159416705 | 0.956548239 | | down |
| Y_RNA | -0.159416705 | 0.956548239 | | down |
| RP11-21G20.4 | -0.159416705 | 0.956548239 | | down |
| RP11-93017.1 | -0.159416705 | 0.956548239 | | down |
| CTC-311G1.1 | -0.159416705 | 0.956548239 | | down |
| RP1-93H18.6 | -0.159416705 | 0.956548239 | | down |
| MIR1913 | -0.159416705 | 0.956548239 | | down |
| HOXA11-AS1_5 | -0.159416705 | 0.956548239 | | down |
| CLIC4P1 | -0.159416705 | 0.956548239 | | down |
| CYP2C61P | -0.159416705 | 0.956548239 | | down |
| CTD-2063L20.1 | -0.159416705 | 0.956548239 | | down |
| DDX39AP1 | -0.159416705 | 0.956548239 | | down |

| | | | | |
|---|---|---|---|---|
| AKR1B1P4 | -0.159416705 | 0.956548239 | | down |
| RP11-463C8.7 | -0.159416705 | 0.956548239 | | down |
| GCNT1P1 | -0.159416705 | 0.956548239 | | down |
| APOOP5 | -0.159416626 | 0.956282321 | | down |
| TEN1 | -0.159416482 | 0.955789722 | | down |
| CTA-299D3.8 | -0.159415338 | 0.951162456 | | down |
| LSP1P2 | -0.15941532 | 0.951077412 | | down |
| RNU6-298P | -0.159415301 | 0.950986005 | | down |
| RPL23AP55 | -0.159415297 | 0.95097071 | | down |
| RN7SKP161 | -0.15941525 | 0.95074318 | | down |
| IGKV1-32 | -0.15941524 | 0.950693651 | | down |
| RP11-196H14.2 | -0.159415235 | 0.950669934 | | down |
| CNTN4-AS1 | -0.159415217 | 0.950581364 | | down |
| NA | -0.159415209 | 0.950543019 | | down |
| RP11-76C10.5 | -0.159414136 | 0.944295005 | | down |
| GHSR | -0.15941403 | 0.943536018 | | down |
| RP1-164F3.8 | -0.15941399 | 0.943243602 | | down |
| NA | -0.159413977 | 0.943150702 | | down |
| NA | -0.159413277 | 0.937112531 | | down |
| FOXN3-AS2 | -0.159413255 | 0.936895829 | | down |
| MIR93 | -0.159413188 | 0.936205621 | | down |
| AC000032.2 | -0.159413171 | 0.936025706 | | down |
| AC005086.3 | -0.159413143 | 0.935719446 | | down |
| RP11-458F8.3 | -0.159412594 | 0.928886072 | | down |
| RP11-144A16.8 | -0.159412561 | 0.928401944 | | down |
| CTC-248O19.1 | -0.159412216 | 0.842742255 | 0.898263324 | down |
| RP11-517A5.7 | -0.159412159 | 0.921576956 | | down |
| RP11-378J18.6 | -0.159412147 | 0.921349662 | | down |
| RP11-466F5.9 | -0.159411498 | 0.904319577 | | down |
| HMGB1P31 | -0.159373292 | 0.712695106 | 0.802983676 | down |
| N4BP3 | -0.159372458 | 0.4835232 | 0.612590877 | down |
| LECT1 | -0.159319655 | 0.879912673 | | down |
| NA | -0.159307733 | 0.855973076 | | down |
| CS | -0.159294956 | 0.144411357 | 0.261903586 | down |
| AP4S1 | -0.159260171 | 0.309913914 | 0.448466122 | down |
| EMG1 | -0.15921962 | 0.257552139 | 0.392640671 | down |
| MRPS17 | -0.159210016 | 0.333343312 | 0.471603243 | down |
| TRPT1 | -0.159148663 | 0.372624039 | 0.510982547 | down |
| MIOX | -0.159032957 | 0.715067751 | 0.804655437 | down |
| AC084219.4 | -0.15895432 | 0.69236958 | 0.787639214 | down |
| PAPPA | -0.158940061 | 0.61091496 | 0.722619889 | down |
| CD300LF | -0.158879244 | 0.576342842 | 0.693208069 | down |
| GNAQ | -0.158867079 | 0.251108235 | 0.385838277 | down |
| LIN7C | -0.158837967 | 0.294685887 | 0.432086486 | down |
| CHL1 | -0.158791884 | 0.606699238 | 0.719036522 | down |
| CD9 | -0.15870551 | 0.540157624 | 0.661982283 | down |
| DAAM1 | -0.158686951 | 0.415420355 | 0.551858195 | down |
| ZNF283 | -0.158653556 | 0.374984266 | 0.513235264 | down |
| CCDC9 | -0.158621875 | 0.384946641 | 0.522679843 | down |
| NUB1 | -0.158579588 | 0.105935666 | 0.21064939 | down |
| CTB-96E2.10 | -0.158571579 | 0.818635785 | 0.881168884 | down |

| | | | | |
|---|---|---|---|---|
| CENPT | -0.158423159 | 0.430941954 | 0.565973949 | down |
| HIST1H2AC | -0.158341766 | 0.435772013 | 0.570496944 | down |
| MILR1 | -0.158080164 | 0.528714967 | 0.652081792 | down |
| RP13-631K18.5 | -0.157960384 | 0.882637848 | | down |
| BRWD1 | -0.157958863 | 0.242421998 | 0.376302067 | down |
| SETD4 | -0.15794635 | 0.239300541 | 0.372863056 | down |
| CD6 | -0.157895208 | 0.620722688 | 0.730845806 | down |
| RP11-537H15.3 | -0.157871742 | 0.825195188 | 0.885401959 | down |
| GAL3ST4 | -0.157752523 | 0.368460992 | 0.506986125 | down |
| ABCD4 | -0.157670947 | 0.15695642 | 0.277584099 | down |
| BFSP2 | -0.157665304 | 0.722096078 | 0.810560604 | down |
| CDCA3 | -0.157634869 | 0.541402062 | 0.662993542 | down |
| NA | -0.157624701 | 0.900118287 | 0.936267616 | down |
| CHMP7 | -0.157585239 | 0.307001617 | 0.445324284 | down |
| AUNIP | -0.157553575 | 0.570649191 | 0.688233483 | down |
| CHCHD2P2 | -0.157530471 | 0.82011362 | | down |
| SERP2 | -0.15748041 | 0.456291732 | 0.588589103 | down |
| HEXA-AS1 | -0.157332659 | 0.698175623 | 0.792084752 | down |
| SLC16A10 | -0.15732013 | 0.61167707 | 0.723256558 | down |
| CCAR2 | -0.157212711 | 0.125329305 | 0.237010993 | down |
| CYP4F34P | -0.15718068 | 0.925611125 | | down |
| WASF2 | -0.157153065 | 0.284533246 | 0.421378493 | down |
| CBY1 | -0.157099645 | 0.402009676 | 0.538738512 | down |
| RPLP0P2 | -0.157019078 | 0.74002709 | 0.823275343 | down |
| MBOAT2 | -0.156994339 | 0.348801529 | 0.487572379 | down |
| TMEM9B | -0.156911779 | 0.288373251 | 0.425551022 | down |
| SEC14L1P1 | -0.156902834 | 0.753344644 | 0.833001601 | down |
| RP11-109N23.4 | -0.156832764 | 0.723715939 | 0.811681413 | down |
| PRSS16 | -0.156637172 | 0.50600587 | 0.632381047 | down |
| ZNF43 | -0.156594723 | 0.483441977 | 0.612539253 | down |
| CLTA | -0.156563402 | 0.349032584 | 0.487734012 | down |
| MRPS33 | -0.15655798 | 0.239985001 | 0.373716341 | down |
| AC025335.1 | -0.156531214 | 0.558509536 | 0.678111153 | down |
| RCN1 | -0.156523971 | 0.587808117 | 0.703169702 | down |
| GCC2 | -0.156489852 | 0.379064919 | 0.51707725 | down |
| GALNT16 | -0.156418095 | 0.528047689 | 0.651499518 | down |
| NTAN1P3 | -0.15627844 | 0.957402805 | | down |
| CTXN3 | -0.156277945 | 0.954897152 | | down |
| AC008992.1 | -0.156275861 | 0.93560646 | | down |
| CTD-2616J11.2 | -0.156275272 | 0.923394884 | | down |
| RP11-183I6.2 | -0.156275261 | 0.923110158 | | down |
| NA | -0.156274848 | 0.908521012 | | down |
| DNAJC19P9 | -0.156274439 | 0.880766353 | | down |
| MIR4258 | -0.156274373 | 0.873374641 | | down |
| NA | -0.156213653 | 0.674611701 | 0.773795821 | down |
| BEX2 | -0.156174407 | 0.401004364 | 0.537895129 | down |
| POLR2F | -0.156148291 | 0.189103693 | 0.316214803 | down |
| ABCF1 | -0.156097141 | 0.072724166 | 0.160809953 | down |
| ZNF605 | -0.156049161 | 0.375566466 | 0.513868244 | down |
| DPP9-AS1 | -0.156040185 | 0.737934249 | 0.822012405 | down |
| METTL8 | -0.155983725 | 0.235879132 | 0.368851956 | down |

| | | | | |
|---|---|---|---|---|
| AC011899.9 | -0.155967376 | 0.657275057 | 0.759477398 | down |
| EEF1B2P3 | -0.155857033 | 0.426818514 | 0.562402327 | down |
| STK16 | -0.155777458 | 0.17924491 | 0.304439712 | down |
| ZBTB24 | -0.155686538 | 0.289488563 | 0.426737204 | down |
| ARID1B | -0.155492104 | 0.395656275 | 0.532886409 | down |
| LRR1 | -0.155451445 | 0.37434539 | 0.512708834 | down |
| LINC01539 | -0.155436136 | 0.81027012 | 0.874674537 | down |
| SNRK-AS1 | -0.155342635 | 0.713910966 | 0.803783209 | down |
| KCNK12 | -0.155320714 | 0.681912656 | 0.779610481 | down |
| COL4A3 | -0.155276784 | 0.669327641 | 0.769291494 | down |
| LINC01125 | -0.155245803 | 0.653266302 | 0.756620385 | down |
| MYL7 | -0.155173152 | 0.831667853 | 0.890027392 | down |
| FHOD3 | -0.155107699 | 0.709580388 | 0.80048938 | down |
| RAET1G | -0.155062609 | 0.751430529 | 0.831772855 | down |
| PYGM | -0.155038569 | 0.536982903 | 0.65917888 | down |
| UBTF | -0.155020721 | 0.101014861 | 0.203465439 | down |
| LRRIQ3 | -0.155016433 | 0.678207838 | 0.77685296 | down |
| RP11-545A16.4 | -0.154945232 | 0.9024596 | | down |
| ZNF18 | -0.154821572 | 0.249655872 | 0.384236697 | down |
| BRCA1 | -0.154727915 | 0.479619308 | 0.609005642 | down |
| PLIN3 | -0.154708806 | 0.620684801 | 0.730829839 | down |
| RP11-697N18.4 | -0.154583993 | 0.742365874 | 0.825143926 | down |
| PRR14L | -0.154495103 | 0.295670188 | 0.433153653 | down |
| RP11-113C12.2 | -0.15437259 | 0.836802203 | | down |
| RP3-395M20.3 | -0.154371349 | 0.818154437 | | down |
| MXD3 | -0.154253419 | 0.555040028 | 0.674880514 | down |
| UNC45A | -0.154197677 | 0.27633142 | 0.412608776 | down |
| BTBD11 | -0.154141908 | 0.539359469 | 0.661246874 | down |
| FAM76A | -0.154109889 | 0.346836589 | 0.485605689 | down |
| RP11-472N13.3 | -0.154073556 | 0.67837046 | 0.77695037 | down |
| TIGD7 | -0.153986623 | 0.688001733 | 0.784184683 | down |
| RP11-6N17.4 | -0.153835025 | 0.372444113 | 0.510805758 | down |
| TUT1 | -0.153812429 | 0.419212254 | 0.55510657 | down |
| RP11-666F17.1 | -0.153719877 | 0.801976051 | 0.868730831 | down |
| PKD1P5 | -0.153592832 | 0.757721711 | 0.836148942 | down |
| ZNF491 | -0.153518669 | 0.514544656 | 0.639771832 | down |
| RMND5B | -0.153433789 | 0.080681897 | 0.17325795 | down |
| IDH1 | -0.153416844 | 0.521669678 | 0.646018045 | down |
| ZNF587 | -0.153314463 | 0.380177606 | 0.518123883 | down |
| JPH3 | -0.153284164 | 0.72894979 | 0.815735515 | down |
| RBBP5 | -0.153251027 | 0.136772556 | 0.252035984 | down |
| ZNF525 | -0.153155629 | 0.641148526 | 0.747078277 | down |
| HSBP1 | -0.153141616 | 0.136883254 | 0.252162639 | down |
| RP11-185E8.2 | -0.153122963 | 0.639307565 | 0.745569306 | down |
| DDX60 | -0.153101904 | 0.549126167 | 0.669603661 | down |
| ASH1L-AS1 | -0.152871919 | 0.417573441 | 0.553815233 | down |
| RNF111 | -0.152685396 | 0.519208267 | 0.643792537 | down |
| NAT6 | -0.152612245 | 0.412310424 | 0.549040616 | down |
| MT1X | -0.152607602 | 0.641425751 | 0.747343336 | down |
| FRAT1 | -0.152543776 | 0.279925934 | 0.416256382 | down |
| NIFK-AS1 | -0.152370183 | 0.417913725 | 0.554046419 | down |

| | | | | |
|---|---|---|---|---|
| RP11-89K11.1 | -0.152355426 | 0.623214306 | 0.73286041 | down |
| GPC3 | -0.152350597 | 0.640872155 | 0.746838137 | down |
| CTD-2501E16.2 | -0.152332952 | 0.863073168 | | down |
| C19orf54 | -0.152296883 | 0.342315667 | 0.480697916 | down |
| SLC35F2 | -0.15223903 | 0.454355461 | 0.586900546 | down |
| AJ003147.11 | -0.152113214 | 0.711761595 | 0.802227091 | down |
| PDE8A | -0.152072117 | 0.433737691 | 0.568701021 | down |
| ZNF672 | -0.152071919 | 0.31076901 | 0.44920802 | down |
| LONP2 | -0.152029781 | 0.215092103 | 0.346165771 | down |
| ARID2 | -0.152003791 | 0.269957658 | 0.405976969 | down |
| LTBR | -0.151999404 | 0.412556447 | 0.549197834 | down |
| RP11-6F2.5 | -0.151955992 | 0.881781209 | | down |
| NDC1 | -0.151907187 | 0.403735074 | 0.540351771 | down |
| RP11-387A1.6 | -0.151891839 | 0.929313549 | | down |
| SMG1P7 | -0.151728536 | 0.566907932 | 0.685059535 | down |
| ZNF30 | -0.15161998 | 0.543409432 | 0.664855554 | down |
| RP11-325P15.2 | -0.15142871 | 0.897377954 | | down |
| TBC1D27 | -0.151336721 | 0.73785016 | 0.821949211 | down |
| UBXN10-AS1 | -0.151335044 | 0.653829719 | 0.757039466 | down |
| SPAG7 | -0.151304354 | 0.347407166 | 0.486156115 | down |
| RP11-173P15.9 | -0.151293255 | 0.770178182 | 0.84558973 | down |
| AC090804.1 | -0.151208073 | 0.802669429 | 0.869211373 | down |
| NA | -0.151016201 | 0.817728424 | 0.880472808 | down |
| PPP6R2 | -0.150986223 | 0.136689909 | 0.251976417 | down |
| IPMK | -0.150951109 | 0.436395484 | 0.570890269 | down |
| TMEM175 | -0.150914165 | 0.469673008 | 0.600351099 | down |
| OLFM4 | -0.150847416 | 0.733303617 | 0.818745499 | down |
| PITPNA-AS1 | -0.150627683 | 0.830641031 | | down |
| TMPO-AS1 | -0.15059144 | 0.610039295 | 0.721916677 | down |
| ZNF445 | -0.150574796 | 0.443545077 | 0.577077269 | down |
| NDUFB5 | -0.15056309 | 0.224970392 | 0.357074526 | down |
| CAND2 | -0.150528804 | 0.733487044 | 0.818910619 | down |
| CPSF3 | -0.150451529 | 0.343097111 | 0.481484375 | down |
| RPL36A | -0.150442611 | 0.650407956 | 0.754385849 | down |
| RP5-991G20.2 | -0.150136469 | 0.774429348 | 0.848725299 | down |
| NA | -0.150120746 | 0.84332055 | 0.898560932 | down |
| MMP17 | -0.150094968 | 0.62099998 | 0.731057694 | down |
| RP13-131K19.6 | -0.150062701 | 0.781869749 | 0.854026006 | down |
| RP11-391L3.1 | -0.150060342 | 0.764948393 | 0.841743636 | down |
| RPL23AP31 | -0.150007655 | 0.95911052 | | down |
| RP11-417J1.1 | -0.150007655 | 0.95911052 | | down |
| RP11-624D11.2 | -0.150007655 | 0.95911052 | | down |
| OR10AG1 | -0.150007655 | 0.95911052 | | down |
| NA | -0.150007655 | 0.95911052 | | down |
| RP11-47G4.1 | -0.150007655 | 0.95911052 | | down |
| NA | -0.150007644 | 0.959075916 | | down |
| RNU6-208P | -0.150007486 | 0.958558761 | | down |
| HMMR-AS1 | -0.150006204 | 0.953489772 | | down |
| PPP1R3A | -0.150006147 | 0.953221206 | | down |
| BSNDP2 | -0.150006101 | 0.95299822 | | down |
| FAM138E | -0.150005905 | 0.952013527 | | down |

| | | | | |
|---|---|---|---|---|
| MIR1273H | -0.15000496 | 0.94622839 | | down |
| RNA5SP323 | -0.150004237 | 0.940039446 | | down |
| NA | -0.150004204 | 0.939703556 | | down |
| ACTL7B | -0.150004201 | 0.939679605 | | down |
| MIR4518 | -0.150004199 | 0.939658292 | | down |
| RP1-32I10.10 | -0.150004177 | 0.939430214 | | down |
| RSL24D1P1 | -0.150003648 | 0.933035004 | | down |
| AC022173.2 | -0.150003614 | 0.932554975 | | down |
| NA | -0.150003607 | 0.932461383 | | down |
| RPSAP53 | -0.150003582 | 0.93208511 | | down |
| RP11-677M24.1 | -0.150003565 | 0.931842069 | | down |
| RP11-444E17.6 | -0.15000287 | 0.918399294 | | down |
| RP11-44D5.1 | -0.149961747 | 0.859010514 | | down |
| AC008278.3 | -0.149898417 | 0.889056318 | | down |
| AARS2 | -0.149852274 | 0.33409416 | 0.472354115 | down |
| AC009299.3 | -0.149796635 | 0.544912278 | 0.666368629 | down |
| NTN1 | -0.149593763 | 0.713227321 | 0.803336644 | down |
| PROSER3 | -0.149494082 | 0.425403027 | 0.560980052 | down |
| SAP30L | -0.149488612 | 0.424494888 | 0.560151283 | down |
| CTC-471F3.5 | -0.149404699 | 0.766807145 | 0.843201591 | down |
| MCCC1 | -0.14917925 | 0.346845043 | 0.485605689 | down |
| GPR89B | -0.148996984 | 0.452636207 | 0.585331305 | down |
| TUBA4A | -0.148876882 | 0.445084661 | 0.578454106 | down |
| DNAJA2 | -0.148640464 | 0.146618651 | 0.264819693 | down |
| SDHD | -0.148555842 | 0.427138817 | 0.562602307 | down |
| ZNF570 | -0.148551986 | 0.478821313 | 0.608397866 | down |
| RP11-244F12.3 | -0.148520416 | 0.801604503 | 0.868590388 | down |
| HNRNPA1P49 | -0.148472814 | 0.832383169 | 0.890539133 | down |
| PAG1 | -0.14847137 | 0.498372965 | 0.62560029 | down |
| UBE4A | -0.148462144 | 0.638878745 | 0.745213846 | down |
| RP1-159A19.3 | -0.148449348 | 0.692660056 | 0.787820508 | down |
| SCARNA7 | -0.148389776 | 0.809800728 | 0.87434964 | down |
| NA | -0.148377163 | 0.655337323 | 0.758054692 | down |
| ALYREF | -0.148368999 | 0.423919123 | 0.559563558 | down |
| TMPRSS3 | -0.14834858 | 0.666284147 | 0.766827727 | down |
| RP11-216B9.9 | -0.148346443 | 0.765583864 | 0.84225719 | down |
| AC104843.3 | -0.148340204 | 0.735499171 | 0.820256798 | down |
| RNU6-30P | -0.14831957 | 0.814754773 | 0.878152193 | down |
| HTR2B | -0.148267021 | 0.82150464 | 0.883146148 | down |
| CAST | -0.148231638 | 0.301657776 | 0.439466546 | down |
| RBM24 | -0.148199513 | 0.738337989 | 0.822262612 | down |
| DLD | -0.148136555 | 0.429180436 | 0.564499476 | down |
| ING4 | -0.14811673 | 0.161626278 | 0.283409544 | down |
| RP11-23F23.2 | -0.147951902 | 0.823571121 | 0.884386688 | down |
| MLX | -0.147854602 | 0.444919845 | 0.578339974 | down |
| GPN2 | -0.147761803 | 0.224668199 | 0.356849193 | down |
| FLJ38576 | -0.14774673 | 0.710877367 | 0.801501093 | down |
| UBN2 | -0.14774517 | 0.338432363 | 0.476871866 | down |
| NECTIN2 | -0.147507394 | 0.550266337 | 0.670595945 | down |
| C19orf18 | -0.147415357 | 0.604523754 | 0.717254232 | down |
| RP11-60A14.1 | -0.147378233 | 0.861721087 | 0.911475437 | down |

| | | | | |
|---|---|---|---|---|
| SPANXA2-OT1 | -0.147322371 | 0.883474567 | 0.925653353 | down |
| JARID2 | -0.147269149 | 0.419418532 | 0.555160295 | down |
| MTFR2 | -0.147225852 | 0.581441083 | 0.697413601 | down |
| LINC00544 | -0.147197021 | 0.818573755 | 0.881161945 | down |
| ALDH3B2 | -0.147187232 | 0.801858982 | 0.868709479 | down |
| RP11-308D16.2 | -0.147154222 | 0.58756044 | 0.703051049 | down |
| RN7SL558P | -0.146937349 | 0.882950184 | | down |
| TSSC2 | -0.14693014 | 0.539971696 | 0.661862414 | down |
| UTAT33 | -0.146908756 | 0.795123895 | 0.863811329 | down |
| THYN1 | -0.146894577 | 0.312033551 | 0.450665129 | down |
| HRG | -0.146867322 | 0.945090413 | | down |
| NA | -0.146866864 | 0.938806978 | | down |
| RPS3AP53 | -0.146866633 | 0.934681407 | | down |
| RP11-25K21.1 | -0.14686661 | 0.934219074 | | down |
| DDX11L8 | -0.146866141 | 0.922128207 | | down |
| AC005304.1 | -0.146866099 | 0.920690446 | | down |
| RP11-168K11.2 | -0.14686582 | 0.908512433 | | down |
| RP11-532F6.5 | -0.146865727 | 0.903078947 | | down |
| RP11-351I24.3 | -0.146865555 | 0.88963297 | | down |
| RP11-474I11.8 | -0.14686553 | 0.887159752 | | down |
| RP13-228J13.1 | -0.146865393 | 0.870119989 | | down |
| RP11-94I2.4 | -0.146699331 | 0.687147653 | 0.783576051 | down |
| MYO1G | -0.146679703 | 0.631149064 | 0.739038214 | down |
| NA | -0.146673042 | 0.869429052 | | down |
| RP11-27909.4 | -0.146654631 | 0.739544678 | 0.82289496 | down |
| UPK2 | -0.146543208 | 0.788430799 | 0.85871361 | down |
| SUOX | -0.146503847 | 0.228390057 | 0.360746063 | down |
| CEP78 | -0.146458917 | 0.377858599 | 0.515947087 | down |
| TTN-AS1 | -0.146370361 | 0.631458907 | 0.739314578 | down |
| TMEM221 | -0.146364235 | 0.727546493 | 0.814577098 | down |
| AP1B1 | -0.14633579 | 0.223568636 | 0.355635946 | down |
| FRYL | -0.146334053 | 0.310517556 | 0.44899362 | down |
| NA | -0.146244562 | 0.724125141 | 0.811897885 | down |
| MRPS11 | -0.146156437 | 0.313232462 | 0.451918752 | down |
| LA16c-316G12.2 | -0.146127231 | 0.82344005 | 0.884277545 | down |
| S100A11P2 | -0.146115615 | 0.861456188 | 0.911262025 | down |
| RAPH1 | -0.145995177 | 0.682301363 | 0.779905628 | down |
| RP11-274E7.2 | -0.145992076 | 0.577831011 | 0.694414196 | down |
| ATAT1 | -0.145991826 | 0.308545734 | 0.447022532 | down |
| RP5-89003.9 | -0.145989546 | 0.551951694 | 0.671941193 | down |
| RNF180 | -0.145884563 | 0.672481439 | 0.771912293 | down |
| CR2 | -0.145863239 | 0.794939632 | 0.863642377 | down |
| PKN1 | -0.145753177 | 0.4396617 | 0.573564746 | down |
| DDX19B | -0.145707571 | 0.20573983 | 0.335662282 | down |
| RNF170 | -0.145405462 | 0.215829228 | 0.346962027 | down |
| RPP38 | -0.145404432 | 0.312912706 | 0.451587536 | down |
| UBE4B | -0.145372136 | 0.278246736 | 0.414464621 | down |
| NA | -0.145281947 | 0.825987044 | 0.886125861 | down |
| ARHGAP18 | -0.145237576 | 0.471754079 | 0.602302378 | down |
| ZNF696 | -0.145185956 | 0.425531175 | 0.561075161 | down |
| RP5-827C21.4 | -0.145139875 | 0.62698537 | 0.735883603 | down |

| | | | | |
|---|---|---|---|---|
| SARM1 | -0.145106392 | 0.547725257 | 0.668529662 | down |
| PHACTR4 | -0.145104971 | 0.370196032 | 0.508697841 | down |
| GP1BA | -0.145100724 | 0.684141216 | 0.781355212 | down |
| ARHGAP6 | -0.145055115 | 0.647887838 | 0.752421423 | down |
| RP11-309L24.10 | -0.144967454 | 0.783853502 | 0.85546495 | down |
| FAM193B | -0.144901925 | 0.412515847 | 0.549192454 | down |
| CTD-2554C21.2 | -0.144841156 | 0.699328284 | 0.792989384 | down |
| RP11-252A24.2 | -0.144805556 | 0.63999861 | 0.746114557 | down |
| MPPE1 | -0.144784359 | 0.369105713 | 0.507461709 | down |
| GLS | -0.144738852 | 0.536264281 | 0.658715567 | down |
| IRGM | -0.144662937 | 0.820687994 | 0.882584032 | down |
| SRPK1 | -0.144628357 | 0.293626337 | 0.430979026 | down |
| CELF1 | -0.144549655 | 0.178508352 | 0.303588112 | down |
| CTD-2616J11.3 | -0.144513724 | 0.829302525 | 0.888508846 | down |
| MTMR14 | -0.144351244 | 0.333824748 | 0.47208429 | down |
| ADGRE3 | -0.144321203 | 0.723375473 | 0.811420725 | down |
| TMEM119 | -0.144241405 | 0.32441357 | 0.462849598 | down |
| RARA | -0.144215977 | 0.27346746 | 0.409757936 | down |
| NANOS1 | -0.144114671 | 0.739177179 | 0.822756341 | down |
| URAHP | -0.144077994 | 0.726847545 | 0.814160302 | down |
| CHCHD7 | -0.144031514 | 0.26338243 | 0.398763442 | down |
| PSMD1 | -0.143908248 | 0.086690502 | 0.182621289 | down |
| TTC39A | -0.143905404 | 0.579436907 | 0.695731853 | down |
| RP11-660L16.2 | -0.143897647 | 0.522129169 | 0.646373924 | down |
| RP11-365H22.2 | -0.143890289 | 0.751703764 | 0.831981985 | down |
| PRAMEF13 | -0.14373687 | 0.960818423 | | down |
| MINOS1-NBL1 | -0.14373687 | 0.960818423 | | down |
| RP11-293F5.1 | -0.14373687 | 0.960818423 | | down |
| MIR4418 | -0.14373687 | 0.960818423 | | down |
| MIR4684 | -0.14373687 | 0.960818423 | | down |
| MIR6731 | -0.14373687 | 0.960818423 | | down |
| MIR30E | -0.14373687 | 0.960818423 | | down |
| RNU6-1026P | -0.14373687 | 0.960818423 | | down |
| MIR1273G | -0.14373687 | 0.960818423 | | down |
| RP11-334A14.2 | -0.14373687 | 0.960818423 | | down |
| SGO1P1 | -0.14373687 | 0.960818423 | | down |
| RP11-262K1.1 | -0.14373687 | 0.960818423 | | down |
| RNA5SP22 | -0.14373687 | 0.960818423 | | down |
| RP11-115A15.4 | -0.14373687 | 0.960818423 | | down |
| CAPNS1P1 | -0.14373687 | 0.960818423 | | down |
| RNU6-750P | -0.14373687 | 0.960818423 | | down |
| SEPT2P1 | -0.14373687 | 0.960818423 | | down |
| Y_RNA | -0.14373687 | 0.960818423 | | down |
| RPS15AP9 | -0.14373687 | 0.960818423 | | down |
| RP11-196G18.23 | -0.14373687 | 0.960818423 | | down |
| PYHIN5P | -0.14373687 | 0.960818423 | | down |
| FMO10P | -0.14373687 | 0.960818423 | | down |
| HMGB1P11 | -0.14373687 | 0.960818423 | | down |
| RP11-444D13.1 | -0.14373687 | 0.960818423 | | down |
| RP11-541F9.1 | -0.14373687 | 0.960818423 | | down |
| RP11-142L4.2 | -0.14373687 | 0.960818423 | | down |

| | | | |
|---|---|---|---|
| Y_RNA | -0.14373687 | 0.960818423 | down |
| MIR6769B | -0.14373687 | 0.960818423 | down |
| SEPT14P17 | -0.14373687 | 0.960818423 | down |
| RP11-551G24.3 | -0.14373687 | 0.960818423 | down |
| OR2L5 | -0.14373687 | 0.960818423 | down |
| MIR4757 | -0.14373687 | 0.960818423 | down |
| AC010872.1 | -0.14373687 | 0.960818423 | down |
| AC066692.3 | -0.14373687 | 0.960818423 | down |
| AC013267.1 | -0.14373687 | 0.960818423 | down |
| SNORD53 | -0.14373687 | 0.960818423 | down |
| MIR4431 | -0.14373687 | 0.960818423 | down |
| RN7SL211P | -0.14373687 | 0.960818423 | down |
| AC006461.2 | -0.14373687 | 0.960818423 | down |
| NA | -0.14373687 | 0.960818423 | down |
| AC007099.2 | -0.14373687 | 0.960818423 | down |
| MIR4264 | -0.14373687 | 0.960818423 | down |
| MTND4P25 | -0.14373687 | 0.960818423 | down |
| AC009237.5 | -0.14373687 | 0.960818423 | down |
| ARHGAP42P2 | -0.14373687 | 0.960818423 | down |
| MTND6P10 | -0.14373687 | 0.960818423 | down |
| NCKAP5-IT1 | -0.14373687 | 0.960818423 | down |
| MTCO2P5 | -0.14373687 | 0.960818423 | down |
| AC009480.3 | -0.14373687 | 0.960818423 | down |
| AC073551.1 | -0.14373687 | 0.960818423 | down |
| RNU1-133P | -0.14373687 | 0.960818423 | down |
| AC079354.6 | -0.14373687 | 0.960818423 | down |
| AC108066.1 | -0.14373687 | 0.960818423 | down |
| AC007563.4 | -0.14373687 | 0.960818423 | down |
| MIR3132 | -0.14373687 | 0.960818423 | down |
| AC114812.5 | -0.14373687 | 0.960818423 | down |
| AC114814.2 | -0.14373687 | 0.960818423 | down |
| RPS8P6 | -0.14373687 | 0.960818423 | down |
| AC083855.5 | -0.14373687 | 0.960818423 | down |
| SNORA25 | -0.14373687 | 0.960818423 | down |
| SDAD1P3 | -0.14373687 | 0.960818423 | down |
| RP11-852E15.1 | -0.14373687 | 0.960818423 | down |
| Y_RNA | -0.14373687 | 0.960818423 | down |
| RP11-157F20.1 | -0.14373687 | 0.960818423 | down |
| UQCRHP4 | -0.14373687 | 0.960818423 | down |
| RP11-119D18.1 | -0.14373687 | 0.960818423 | down |
| MIR4796 | -0.14373687 | 0.960818423 | down |
| RNU6-232P | -0.14373687 | 0.960818423 | down |
| RPL21P42 | -0.14373687 | 0.960818423 | down |
| Y_RNA | -0.14373687 | 0.960818423 | down |
| RP11-263C16.2 | -0.14373687 | 0.960818423 | down |
| RNA5SP149 | -0.14373687 | 0.960818423 | down |
| MIR28 | -0.14373687 | 0.960818423 | down |
| MIR378D1 | -0.14373687 | 0.960818423 | down |
| FAM90A26 | -0.14373687 | 0.960818423 | down |
| MIR572 | -0.14373687 | 0.960818423 | down |
| RP11-442P12.2 | -0.14373687 | 0.960818423 | down |

| | | | |
|---|---|---|---|
| U2 | -0.14373687 | 0.960818423 | down |
| Y_RNA | -0.14373687 | 0.960818423 | down |
| RP11-231C18.1 | -0.14373687 | 0.960818423 | down |
| RPL17P20 | -0.14373687 | 0.960818423 | down |
| RPL21P47 | -0.14373687 | 0.960818423 | down |
| DPP3P1 | -0.14373687 | 0.960818423 | down |
| RNU6-891P | -0.14373687 | 0.960818423 | down |
| RNU2-16P | -0.14373687 | 0.960818423 | down |
| DSPP | -0.14373687 | 0.960818423 | down |
| CRYZP2 | -0.14373687 | 0.960818423 | down |
| ABT1P1 | -0.14373687 | 0.960818423 | down |
| PCNAP1 | -0.14373687 | 0.960818423 | down |
| RP11-729M20.1 | -0.14373687 | 0.960818423 | down |
| KRT19P3 | -0.14373687 | 0.960818423 | down |
| CUL4AP1 | -0.14373687 | 0.960818423 | down |
| LINC01256 | -0.14373687 | 0.960818423 | down |
| NA | -0.14373687 | 0.960818423 | down |
| RP11-292D4.3 | -0.14373687 | 0.960818423 | down |
| RP11-171N4.4 | -0.14373687 | 0.960818423 | down |
| RP11-287F9.1 | -0.14373687 | 0.960818423 | down |
| RP11-332J15.4 | -0.14373687 | 0.960818423 | down |
| CTD-2296D1.4 | -0.14373687 | 0.960818423 | down |
| KRT18P56 | -0.14373687 | 0.960818423 | down |
| CTD-2335O3.3 | -0.14373687 | 0.960818423 | down |
| RPEP1 | -0.14373687 | 0.960818423 | down |
| NA | -0.14373687 | 0.960818423 | down |
| MTCYBP40 | -0.14373687 | 0.960818423 | down |
| Y_RNA | -0.14373687 | 0.960818423 | down |
| PGAM5P1 | -0.14373687 | 0.960818423 | down |
| RP11-492L8.1 | -0.14373687 | 0.960818423 | down |
| RP11-436H11.5 | -0.14373687 | 0.960818423 | down |
| snoZ6 | -0.14373687 | 0.960818423 | down |
| GS1-39E22.1 | -0.14373687 | 0.960818423 | down |
| SPINK7 | -0.14373687 | 0.960818423 | down |
| GLRXP3 | -0.14373687 | 0.960818423 | down |
| CTC-430J12.2 | -0.14373687 | 0.960818423 | down |
| RP11-91H12.4 | -0.14373687 | 0.960818423 | down |
| BRD2-IT1 | -0.14373687 | 0.960818423 | down |
| GUCA1A | -0.14373687 | 0.960818423 | down |
| LINC01626 | -0.14373687 | 0.960818423 | down |
| RNU6-155P | -0.14373687 | 0.960818423 | down |
| DBIP1 | -0.14373687 | 0.960818423 | down |
| RNA5SP210 | -0.14373687 | 0.960818423 | down |
| RP3-393K13.1 | -0.14373687 | 0.960818423 | down |
| RP3-417O22.3 | -0.14373687 | 0.960818423 | down |
| Y_RNA | -0.14373687 | 0.960818423 | down |
| Y_RNA | -0.14373687 | 0.960818423 | down |
| RNY3P11 | -0.14373687 | 0.960818423 | down |
| GPRC6A | -0.14373687 | 0.960818423 | down |
| RP3-323K23.3 | -0.14373687 | 0.960818423 | down |
| RP11-753B14.1 | -0.14373687 | 0.960818423 | down |

| | | | |
|---|---|---|---|
| RP1-95L4.3 | -0.14373687 | 0.960818423 | down |
| RP11-106E7.1 | -0.14373687 | 0.960818423 | down |
| SSR1P1 | -0.14373687 | 0.960818423 | down |
| RP3-393E18.3 | -0.14373687 | 0.960818423 | down |
| NA | -0.14373687 | 0.960818423 | down |
| PSMC1P2 | -0.14373687 | 0.960818423 | down |
| AC005105.2 | -0.14373687 | 0.960818423 | down |
| RNA5SP229 | -0.14373687 | 0.960818423 | down |
| AC004869.2 | -0.14373687 | 0.960818423 | down |
| NA | -0.14373687 | 0.960818423 | down |
| RP11-548K12.6 | -0.14373687 | 0.960818423 | down |
| RNU6-973P | -0.14373687 | 0.960818423 | down |
| RN7SL43P | -0.14373687 | 0.960818423 | down |
| RP11-3P22.1 | -0.14373687 | 0.960818423 | down |
| RNU6-1198P | -0.14373687 | 0.960818423 | down |
| RPL7P30 | -0.14373687 | 0.960818423 | down |
| GS1-293C5.1 | -0.14373687 | 0.960818423 | down |
| RP11-758P17.2 | -0.14373687 | 0.960818423 | down |
| RN7SL416P | -0.14373687 | 0.960818423 | down |
| CTB-20D2.1 | -0.14373687 | 0.960818423 | down |
| AC009784.4 | -0.14373687 | 0.960818423 | down |
| RNU6-797P | -0.14373687 | 0.960818423 | down |
| RNY3 | -0.14373687 | 0.960818423 | down |
| RP11-728K20.3 | -0.14373687 | 0.960818423 | down |
| NA | -0.14373687 | 0.960818423 | down |
| RNU6-1086P | -0.14373687 | 0.960818423 | down |
| RP11-962G15.2 | -0.14373687 | 0.960818423 | down |
| CTA-392C11.1 | -0.14373687 | 0.960818423 | down |
| Y_RNA | -0.14373687 | 0.960818423 | down |
| RP11-643N23.2 | -0.14373687 | 0.960818423 | down |
| NA | -0.14373687 | 0.960818423 | down |
| RN7SKP294 | -0.14373687 | 0.960818423 | down |
| RP11-787D18.2 | -0.14373687 | 0.960818423 | down |
| RPL31P41 | -0.14373687 | 0.960818423 | down |
| CTD-2320G14.2 | -0.14373687 | 0.960818423 | down |
| RP11-48B3.2 | -0.14373687 | 0.960818423 | down |
| RPS26P34 | -0.14373687 | 0.960818423 | down |
| RNU6-1224P | -0.14373687 | 0.960818423 | down |
| RP11-122A21.2 | -0.14373687 | 0.960818423 | down |
| RP11-299M14.2 | -0.14373687 | 0.960818423 | down |
| IFNNP1 | -0.14373687 | 0.960818423 | down |
| KRT18P66 | -0.14373687 | 0.960818423 | down |
| AL353662.1 | -0.14373687 | 0.960818423 | down |
| NA | -0.14373687 | 0.960818423 | down |
| OR1N2 | -0.14373687 | 0.960818423 | down |
| RP11-85O21.4 | -0.14373687 | 0.960818423 | down |
| RP11-473E2.3 | -0.14373687 | 0.960818423 | down |
| NA | -0.14373687 | 0.960818423 | down |
| SNORD115 | -0.14373687 | 0.960818423 | down |
| TCEB1P30 | -0.14373687 | 0.960818423 | down |
| RP11-449O16.2 | -0.14373687 | 0.960818423 | down |

| | | | |
|---|---|---|---|
| ARL4AP1 | -0.14373687 | 0.960818423 | down |
| DNAJC19P1 | -0.14373687 | 0.960818423 | down |
| RP11-369F10.2 | -0.14373687 | 0.960818423 | down |
| C1DP2 | -0.14373687 | 0.960818423 | down |
| SNORA19 | -0.14373687 | 0.960818423 | down |
| NA | -0.14373687 | 0.960818423 | down |
| MIR4484 | -0.14373687 | 0.960818423 | down |
| KRTAP5-2 | -0.14373687 | 0.960818423 | down |
| OR51F1 | -0.14373687 | 0.960818423 | down |
| OR52E7P | -0.14373687 | 0.960818423 | down |
| RP11-35J10.4 | -0.14373687 | 0.960818423 | down |
| RP11-98J9.3 | -0.14373687 | 0.960818423 | down |
| RNA5SP339 | -0.14373687 | 0.960818423 | down |
| CTD-3012A18.1 | -0.14373687 | 0.960818423 | down |
| RP4-710M3.2 | -0.14373687 | 0.960818423 | down |
| OR4A2P | -0.14373687 | 0.960818423 | down |
| OR8U1 | -0.14373687 | 0.960818423 | down |
| OR5AL1 | -0.14373687 | 0.960818423 | down |
| OR5M1 | -0.14373687 | 0.960818423 | down |
| AP000445.2 | -0.14373687 | 0.960818423 | down |
| OR10V1 | -0.14373687 | 0.960818423 | down |
| C11orf98 | -0.14373687 | 0.960818423 | down |
| AP003774.5 | -0.14373687 | 0.960818423 | down |
| MIR3163 | -0.14373687 | 0.960818423 | down |
| MIR3165 | -0.14373687 | 0.960818423 | down |
| AP000593.6 | -0.14373687 | 0.960818423 | down |
| MIR326 | -0.14373687 | 0.960818423 | down |
| RNU7-59P | -0.14373687 | 0.960818423 | down |
| RP11-720D4.3 | -0.14373687 | 0.960818423 | down |
| RNU6-16P | -0.14373687 | 0.960818423 | down |
| OSBPL9P3 | -0.14373687 | 0.960818423 | down |
| RP11-676F20.1 | -0.14373687 | 0.960818423 | down |
| RP11-573M3.6 | -0.14373687 | 0.960818423 | down |
| RP11-360K13.2 | -0.14373687 | 0.960818423 | down |
| RNU7-159P | -0.14373687 | 0.960818423 | down |
| CASP1P1 | -0.14373687 | 0.960818423 | down |
| RP11-386B1.1 | -0.14373687 | 0.960818423 | down |
| NA | -0.14373687 | 0.960818423 | down |
| OR8A3P | -0.14373687 | 0.960818423 | down |
| RP11-283I3.1 | -0.14373687 | 0.960818423 | down |
| RP11-283I3.4 | -0.14373687 | 0.960818423 | down |
| RP11-476M19.1 | -0.14373687 | 0.960818423 | down |
| NA | -0.14373687 | 0.960818423 | down |
| GOT2P3 | -0.14373687 | 0.960818423 | down |
| HSPE1P12 | -0.14373687 | 0.960818423 | down |
| ZKSCAN7P1 | -0.14373687 | 0.960818423 | down |
| RP11-946L16.2 | -0.14373687 | 0.960818423 | down |
| RP11-73B8.1 | -0.14373687 | 0.960818423 | down |
| RP11-161H23.5 | -0.14373687 | 0.960818423 | down |
| VTI1BP3 | -0.14373687 | 0.960818423 | down |
| OR10U1P | -0.14373687 | 0.960818423 | down |

| | | | |
|---|---|---|---|
| OR10AE3P | -0.14373687 | 0.960818423 | down |
| Y_RNA | -0.14373687 | 0.960818423 | down |
| RP11-43N5.2 | -0.14373687 | 0.960818423 | down |
| RP11-81H14.3 | -0.14373687 | 0.960818423 | down |
| RNU7-4P | -0.14373687 | 0.960818423 | down |
| CTD-2021H9.1 | -0.14373687 | 0.960818423 | down |
| RNU6-808P | -0.14373687 | 0.960818423 | down |
| RN7SKP11 | -0.14373687 | 0.960818423 | down |
| MIR4304 | -0.14373687 | 0.960818423 | down |
| LINC00442 | -0.14373687 | 0.960818423 | down |
| RNU6-59P | -0.14373687 | 0.960818423 | down |
| TPTE2P6 | -0.14373687 | 0.960818423 | down |
| RPL29P28 | -0.14373687 | 0.960818423 | down |
| LINC00428 | -0.14373687 | 0.960818423 | down |
| PPIAP26 | -0.14373687 | 0.960818423 | down |
| CTAGE16P | -0.14373687 | 0.960818423 | down |
| LINC01069 | -0.14373687 | 0.960818423 | down |
| RPL29P29 | -0.14373687 | 0.960818423 | down |
| MTND6P18 | -0.14373687 | 0.960818423 | down |
| DAOA-AS1 | -0.14373687 | 0.960818423 | down |
| LINC00396 | -0.14373687 | 0.960818423 | down |
| OR4K1 | -0.14373687 | 0.960818423 | down |
| RPPH1 | -0.14373687 | 0.960818423 | down |
| RP11-306B9.1 | -0.14373687 | 0.960818423 | down |
| RP11-134E15.1 | -0.14373687 | 0.960818423 | down |
| ILF2P2 | -0.14373687 | 0.960818423 | down |
| LINC00609 | -0.14373687 | 0.960818423 | down |
| RPL29P3 | -0.14373687 | 0.960818423 | down |
| RN7SKP193 | -0.14373687 | 0.960818423 | down |
| RP11-486O13.4 | -0.14373687 | 0.960818423 | down |
| NA | -0.14373687 | 0.960818423 | down |
| U3 | -0.14373687 | 0.960818423 | down |
| NA | -0.14373687 | 0.960818423 | down |
| MIR4710 | -0.14373687 | 0.960818423 | down |
| HERC2P11 | -0.14373687 | 0.960818423 | down |
| BUB1B-PAK6 | -0.14373687 | 0.960818423 | down |
| RN7SL568P | -0.14373687 | 0.960818423 | down |
| MIR1272 | -0.14373687 | 0.960818423 | down |
| RP11-325L12.5 | -0.14373687 | 0.960818423 | down |
| RP11-81A1.8 | -0.14373687 | 0.960818423 | down |
| RNU7-79P | -0.14373687 | 0.960818423 | down |
| RP11-382N13.6 | -0.14373687 | 0.960818423 | down |
| CTD-2135D7.3 | -0.14373687 | 0.960818423 | down |
| RP11-419L9.1 | -0.14373687 | 0.960818423 | down |
| CTA-363E6.7 | -0.14373687 | 0.960818423 | down |
| RP11-143N13.2 | -0.14373687 | 0.960818423 | down |
| RP11-143N13.4 | -0.14373687 | 0.960818423 | down |
| U4atac | -0.14373687 | 0.960818423 | down |
| AC026471.6 | -0.14373687 | 0.960818423 | down |
| RP11-19N8.3 | -0.14373687 | 0.960818423 | down |
| ENPP7P13 | -0.14373687 | 0.960818423 | down |

| | | | |
|---|---|---|---|
| RP11-104C4.4 | -0.14373687 | 0.960818423 | down |
| RP11-598D12.2 | -0.14373687 | 0.960818423 | down |
| RP11-46D6.4 | -0.14373687 | 0.960818423 | down |
| RP11-355E10.1 | -0.14373687 | 0.960818423 | down |
| RP11-403P17.5 | -0.14373687 | 0.960818423 | down |
| RNU6-22P | -0.14373687 | 0.960818423 | down |
| RNU7-90P | -0.14373687 | 0.960818423 | down |
| RN7SL134P | -0.14373687 | 0.960818423 | down |
| RP5-1142A6.5 | -0.14373687 | 0.960818423 | down |
| MIR6864 | -0.14373687 | 0.960818423 | down |
| MIR6865 | -0.14373687 | 0.960818423 | down |
| RP11-960B9.2 | -0.14373687 | 0.960818423 | down |
| Metazoa_SRP | -0.14373687 | 0.960818423 | down |
| SNORA74 | -0.14373687 | 0.960818423 | down |
| RNU6-862P | -0.14373687 | 0.960818423 | down |
| MTND1P14 | -0.14373687 | 0.960818423 | down |
| RP11-822E23.7 | -0.14373687 | 0.960818423 | down |
| NCOR1P2 | -0.14373687 | 0.960818423 | down |
| CTD-2008P7.8 | -0.14373687 | 0.960818423 | down |
| RN7SL656P | -0.14373687 | 0.960818423 | down |
| RP11-995C19.2 | -0.14373687 | 0.960818423 | down |
| RP11-63A1.2 | -0.14373687 | 0.960818423 | down |
| MIR4729 | -0.14373687 | 0.960818423 | down |
| Y_RNA | -0.14373687 | 0.960818423 | down |
| RPL23AP74 | -0.14373687 | 0.960818423 | down |
| RNA5SP445 | -0.14373687 | 0.960818423 | down |
| NA | -0.14373687 | 0.960818423 | down |
| RNU6-120P | -0.14373687 | 0.960818423 | down |
| MIR924 | -0.14373687 | 0.960818423 | down |
| MIR4529 | -0.14373687 | 0.960818423 | down |
| RP11-456O19.5 | -0.14373687 | 0.960818423 | down |
| RN7SL551P | -0.14373687 | 0.960818423 | down |
| CTC-312O10.2 | -0.14373687 | 0.960818423 | down |
| MTCO1P27 | -0.14373687 | 0.960818423 | down |
| MIR181C | -0.14373687 | 0.960818423 | down |
| CTD-3233P19.7 | -0.14373687 | 0.960818423 | down |
| NA | -0.14373687 | 0.960818423 | down |
| CTD-2293H3.3 | -0.14373687 | 0.960818423 | down |
| SYCN | -0.14373687 | 0.960818423 | down |
| CTD-2126E3.5 | -0.14373687 | 0.960818423 | down |
| RN7SL317P | -0.14373687 | 0.960818423 | down |
| CTD-2105E13.15 | -0.14373687 | 0.960818423 | down |
| RP4-717M23.2 | -0.14373687 | 0.960818423 | down |
| RN7SKP74 | -0.14373687 | 0.960818423 | down |
| NKX2-4 | -0.14373687 | 0.960818423 | down |
| Y_RNA | -0.14373687 | 0.960818423 | down |
| DEFB119 | -0.14373687 | 0.960818423 | down |
| RP11-346K17.3 | -0.14373687 | 0.960818423 | down |
| Metazoa_SRP | -0.14373687 | 0.960818423 | down |
| NA | -0.14373687 | 0.960818423 | down |
| MIR3617 | -0.14373687 | 0.960818423 | down |

| | | | | |
|---|---|---|---|---|
| RP11-560A15.6 | -0.14373687 | 0.960818423 | | down |
| RNA5-8S5 | -0.14373687 | 0.960818423 | | down |
| RNA5-8S5 | -0.14373687 | 0.960818423 | | down |
| KRTAP21-1 | -0.14373687 | 0.960818423 | | down |
| EXOSC3P1 | -0.14373687 | 0.960818423 | | down |
| MRPL20P1 | -0.14373687 | 0.960818423 | | down |
| MIR6814 | -0.14373687 | 0.960818423 | | down |
| MIR5692B | -0.14373687 | 0.960818423 | | down |
| AP001631.10 | -0.14373687 | 0.960818423 | | down |
| CRYAA | -0.14373687 | 0.960818423 | | down |
| RP5-1170K4.7 | -0.14373687 | 0.960818423 | | down |
| RP1-127B20.4 | -0.14373687 | 0.960818423 | | down |
| RNU6-800P | -0.14373687 | 0.960818423 | | down |
| MPC1L | -0.14373687 | 0.960818423 | | down |
| MIR500B | -0.14373687 | 0.960818423 | | down |
| RNY4P23 | -0.14373687 | 0.960818423 | | down |
| RNU6-562P | -0.14373687 | 0.960818423 | | down |
| HDGFP1 | -0.14373687 | 0.960818423 | | down |
| RP11-85L21.4 | -0.14373687 | 0.960818423 | | down |
| SAGE4P | -0.14373687 | 0.960818423 | | down |
| CT45A10 | -0.14373687 | 0.960818423 | | down |
| SPANXN3 | -0.14373687 | 0.960818423 | | down |
| RNU6-383P | -0.14373687 | 0.960818423 | | down |
| LL0XNC01-37G1.2 | -0.14373687 | 0.960818423 | | down |
| SNX3P1Y | -0.14373687 | 0.960818423 | | down |
| RNA5-8SP6 | -0.14373687 | 0.960818423 | | down |
| ARSFP1 | -0.14373687 | 0.960818423 | | down |
| CDY7P | -0.14373687 | 0.960818423 | | down |
| NA | -0.14373687 | 0.960818423 | | down |
| OFD1P15Y | -0.14373687 | 0.960818423 | | down |
| MT-TV | -0.14373687 | 0.960818423 | | down |
| MT-TS2 | -0.14373687 | 0.960818423 | | down |
| RP11-359B12.2 | -0.143564511 | 0.641526212 | 0.747372338 | down |
| NA | -0.143432393 | 0.755716548 | 0.834917554 | down |
| XCL1 | -0.143416505 | 0.723236567 | 0.811295203 | down |
| RP4-635E18.8 | -0.143098078 | 0.724541235 | 0.812152254 | down |
| FUZ | -0.143061966 | 0.301273824 | 0.439065269 | down |
| RP11-367N14.2 | -0.143033984 | 0.697468768 | 0.791633959 | down |
| SNRPD3 | -0.14301058 | 0.185855678 | 0.312121185 | down |
| RP11-454E5.4 | -0.142892839 | 0.543701922 | 0.665105071 | down |
| USE1 | -0.142852876 | 0.383101864 | 0.520857652 | down |
| RP11-415F23.4 | -0.142826983 | 0.699422537 | 0.793015777 | down |
| ACAD9 | -0.142822542 | 0.319408857 | 0.458223325 | down |
| SLC25A24P1 | -0.142806259 | 0.899148064 | | down |
| ZNHIT3 | -0.142736176 | 0.323798842 | 0.462125904 | down |
| GLDC | -0.142726681 | 0.67573569 | 0.774730131 | down |
| ACKR4 | -0.142665077 | 0.738812926 | 0.822472786 | down |
| RP11-457K10.1 | -0.142662763 | 0.944528792 | 0.966434732 | down |
| BANP | -0.142628015 | 0.33973556 | 0.478116597 | down |
| TRBJ2-2P | -0.14257656 | 0.886514227 | | down |
| RP11-53I6.4 | -0.142492913 | 0.882609294 | | down |

| | | | | |
|---|---|---|---|---|
| RP11-95D17.1 | -0.142370205 | 0.509102256 | 0.635325781 | down |
| ATL1 | -0.142181084 | 0.758553385 | 0.836882267 | down |
| PTTG1IP | -0.142112087 | 0.392602483 | 0.530056577 | down |
| RP11-2H3.6 | -0.142046327 | 0.776034077 | 0.850018878 | down |
| RPA3 | -0.141902668 | 0.364315746 | 0.502686795 | down |
| EPDR1 | -0.141844649 | 0.664270636 | 0.765366639 | down |
| TRAF7 | -0.141776698 | 0.198725206 | 0.327545539 | down |
| DCUN1D2 | -0.141774956 | 0.298297411 | 0.435791267 | down |
| PPM1K | -0.14176764 | 0.497850019 | 0.625205064 | down |
| BPIFB4 | -0.141698629 | 0.889441203 | 0.929279855 | down |
| ZXDA | -0.141689084 | 0.629697687 | 0.737880979 | down |
| AK4P3 | -0.141676811 | 0.814454005 | 0.878007311 | down |
| RP11-732A21.3 | -0.141661825 | 0.737743597 | 0.82189145 | down |
| COG3 | -0.141518494 | 0.258092131 | 0.393131209 | down |
| CXorf40A | -0.141451529 | 0.178238304 | 0.303347397 | down |
| LINC00936 | -0.141250001 | 0.556581534 | 0.676250873 | down |
| CTA-390C10.10 | -0.141144112 | 0.67117358 | 0.770823358 | down |
| METTL15 | -0.141013521 | 0.230502482 | 0.362898867 | down |
| LZTR1 | -0.140999964 | 0.409324275 | 0.546056503 | down |
| TEPSIN | -0.140927408 | 0.48958257 | 0.61810391 | down |
| PPP1R14A | -0.140923579 | 0.605872979 | 0.71831608 | down |
| RRP8 | -0.140876653 | 0.274514519 | 0.410883161 | down |
| EXOC1 | -0.140787479 | 0.263615464 | 0.399035823 | down |
| C1QTNF2 | -0.140761058 | 0.584464006 | 0.700326013 | down |
| RP11-111K18.2 | -0.140759455 | 0.8437753 | | down |
| MTIF2 | -0.140707489 | 0.233320635 | 0.36607056 | down |
| NSUN4 | -0.140613237 | 0.335777502 | 0.473953454 | down |
| SNX33 | -0.140611571 | 0.317897336 | 0.456740834 | down |
| OCRL | -0.140603168 | 0.571014939 | 0.688564031 | down |
| NA | -0.140599068 | 0.924368703 | | down |
| PRELID3BP1 | -0.140598605 | 0.961673224 | | down |
| GABRA6 | -0.140598605 | 0.961673224 | | down |
| RN7SKP209 | -0.140598605 | 0.961673224 | | down |
| RP11-164D18.2 | -0.140598605 | 0.961673224 | | down |
| BX842568.4 | -0.140596916 | 0.955095619 | | down |
| CTC-551A13.1 | -0.140596909 | 0.955056584 | | down |
| RP11-114H24.7 | -0.140596852 | 0.954770365 | | down |
| TEX19 | -0.1405968 | 0.954506136 | | down |
| IGHV3-79 | -0.140595965 | 0.949480591 | | down |
| AC006994.1 | -0.140595931 | 0.949236965 | | down |
| AC006946.12 | -0.140595899 | 0.949007875 | | down |
| PRB1 | -0.140595143 | 0.942444059 | | down |
| RP11-21B23.1 | -0.140595101 | 0.94199463 | | down |
| RP11-83B20.8 | -0.140594692 | 0.93706769 | | down |
| L22NC03-30E12.1 | -0.14059467 | 0.936762824 | | down |
| AC007349.5 | -0.140594655 | 0.936556108 | | down |
| RNU4-8P | -0.14059464 | 0.936338872 | | down |
| GSTO3P | -0.140594581 | 0.93549453 | | down |
| RN7SL753P | -0.140594272 | 0.930400481 | | down |
| CYCSP24 | -0.140594243 | 0.929870167 | | down |
| RP13-672B3.2 | -0.140594227 | 0.929567212 | | down |

| | | | | |
|---|---|---|---|---|
| LRRC20 | -0.140586029 | 0.476042504 | 0.606026313 | down |
| ZNF787 | -0.140493021 | 0.325987847 | 0.464441877 | down |
| PPP2R5D | -0.140407055 | 0.712776412 | 0.803009394 | down |
| AURKC | -0.140301799 | 0.503891096 | 0.630524952 | down |
| TCIRG1 | -0.139987799 | 0.551716865 | 0.67173697 | down |
| CFAP53 | -0.139880941 | 0.721351301 | 0.809906144 | down |
| RP11-274B21.3 | -0.139755219 | 0.524383081 | 0.648549535 | down |
| DDX56 | -0.139754233 | 0.391084886 | 0.528649073 | down |
| RAB15 | -0.139685334 | 0.513323161 | 0.638630215 | down |
| SLC6A17 | -0.139662999 | 0.810035495 | 0.874508837 | down |
| ADCYAP1R1 | -0.139301763 | 0.679065296 | 0.777390556 | down |
| IST1 | -0.139262669 | 0.070395916 | 0.156999744 | down |
| RP11-120B7.1 | -0.139154905 | 0.716116202 | 0.805533534 | down |
| CCDC28A | -0.139004139 | 0.38318304 | 0.520897301 | down |
| C17orf53 | -0.138718067 | 0.58976479 | 0.704920964 | down |
| CHRM3-AS2 | -0.138698045 | 0.705332471 | 0.797334566 | down |
| CD1C | -0.138550401 | 0.714418857 | 0.804074327 | down |
| NA | -0.138460541 | 0.823640877 | 0.884398374 | down |
| ZC3HAV1L | -0.138236385 | 0.580886416 | 0.696915242 | down |
| RP13-977J11.2 | -0.138068929 | 0.64046914 | 0.746518267 | down |
| RP11-261C10.8 | -0.138037123 | 0.962370873 | | down |
| GS1-18A18.2 | -0.138034458 | 0.959585447 | | down |
| CEP89 | -0.138003052 | 0.218549627 | 0.350013633 | down |
| RN7SL337P | -0.137987348 | 0.921013919 | | down |
| Y_RNA | -0.137985983 | 0.920137613 | | down |
| RP5-935K16.2 | -0.137944935 | 0.897146863 | | down |
| TMBIM4 | -0.137940259 | 0.370974127 | 0.509324438 | down |
| RP11-667K14.5 | -0.137936097 | 0.892836012 | | down |
| ABHD10 | -0.137931241 | 0.240649306 | 0.374382132 | down |
| CENPJ | -0.137878061 | 0.467223441 | 0.598348724 | down |
| CTC-508F8.1 | -0.137793963 | 0.823735408 | 0.884436659 | down |
| FIZ1 | -0.137648173 | 0.390982141 | 0.528621331 | down |
| DUSP16 | -0.137480032 | 0.442271072 | 0.575843644 | down |
| CD2BP2 | -0.137364292 | 0.321311187 | 0.460048169 | down |
| HMCES | -0.137276276 | 0.531974284 | 0.65511193 | down |
| NA | -0.137182444 | 0.633330454 | 0.740841429 | down |
| FER | -0.137166571 | 0.652178553 | 0.755700631 | down |
| ZNF608 | -0.137149222 | 0.563929193 | 0.682347892 | down |
| DDX51 | -0.137141362 | 0.390729378 | 0.528382474 | down |
| RPL37P23 | -0.136714509 | 0.721261055 | 0.809835082 | down |
| ZNF658B | -0.136712286 | 0.799416689 | 0.866970184 | down |
| RP11-218C14.8 | -0.136678512 | 0.799585709 | 0.867090887 | down |
| SYNPO | -0.1365028 | 0.403585843 | 0.540272382 | down |
| RP11-69L16.5 | -0.136494105 | 0.598794809 | 0.712344804 | down |
| CCDC137 | -0.136429909 | 0.44257389 | 0.576163011 | down |
| TRNAU1AP | -0.136377236 | 0.283177208 | 0.419743256 | down |
| KB-1460A1.5 | -0.13634351 | 0.470533885 | 0.601178824 | down |
| RP13-12804.3 | -0.13631792 | 0.842649785 | 0.898260365 | down |
| RPS29 | -0.136236346 | 0.437019937 | 0.571582734 | down |
| RP11-482G13.1 | -0.1361196 | 0.870263177 | 0.917156255 | down |
| ELMOD2 | -0.135952795 | 0.45603423 | 0.588355142 | down |

| | | | | |
|---|---|---|---|---|
| PIEZO2 | -0.135924283 | 0.722874464 | 0.811030999 | down |
| ITGB1BP2 | -0.135899104 | 0.667537244 | 0.767909368 | down |
| ASCL2 | -0.135863854 | 0.591697211 | 0.706668429 | down |
| CD3D | -0.135792722 | 0.683561431 | 0.780960552 | down |
| FIBCD1 | -0.135762201 | 0.732194517 | 0.818000854 | down |
| APCDD1L | -0.135617856 | 0.768717726 | 0.84449729 | down |
| KIAA0556 | -0.135549625 | 0.426010197 | 0.561608177 | down |
| RP11-624C23.1 | -0.135524843 | 0.785358976 | 0.856454631 | down |
| NA | -0.135329528 | 0.77199737 | 0.846677702 | down |
| RP11-200A1.1 | -0.135304343 | 0.90958464 | 0.943370648 | down |
| CKS1B | -0.135230682 | 0.365809175 | 0.504237973 | down |
| SCN3B | -0.135104444 | 0.661157923 | 0.762819841 | down |
| MOCS3 | -0.135094577 | 0.23001817 | 0.362384158 | down |
| RUFY2 | -0.135084693 | 0.533211393 | 0.656119065 | down |
| RABEP2 | -0.135051486 | 0.36578983 | 0.504237973 | down |
| HOXA-AS2 | -0.134997939 | 0.561320015 | 0.680560877 | down |
| ZNF441 | -0.134982689 | 0.637059487 | 0.743813743 | down |
| RP11-45M22.3 | -0.134980205 | 0.903115298 | | down |
| LAS1L | -0.134967791 | 0.341239098 | 0.479602362 | down |
| RP11-667K14.14 | -0.134785696 | 0.862781548 | 0.912281102 | down |
| CPM | -0.134670963 | 0.49858133 | 0.625775702 | down |
| AL023806.1 | -0.134657737 | 0.768243456 | 0.84416306 | down |
| NYNRIN | -0.134610395 | 0.572373392 | 0.689676193 | down |
| ACHE | -0.134579179 | 0.598160929 | 0.711939796 | down |
| RP11-49K24.3 | -0.134551825 | 0.788960119 | 0.85916553 | down |
| ZNF480 | -0.13446505 | 0.201421934 | 0.330545525 | down |
| AC073052.1 | -0.134451978 | 0.837315752 | 0.894097045 | down |
| LINC00452 | -0.134446464 | 0.872289325 | | down |
| PANK2 | -0.134340479 | 0.372381508 | 0.51074321 | down |
| HNRNPCL3 | -0.13432782 | 0.963381393 | | down |
| Y_RNA | -0.13432782 | 0.963381393 | | down |
| RP1-34M23.5 | -0.13432782 | 0.963381393 | | down |
| CYP4A26P | -0.13432782 | 0.963381393 | | down |
| RP4-700A9.1 | -0.13432782 | 0.963381393 | | down |
| COX6CP13 | -0.13432782 | 0.963381393 | | down |
| HNRNPA3P14 | -0.13432782 | 0.963381393 | | down |
| UBE2WP1 | -0.13432782 | 0.963381393 | | down |
| RP11-12L8.2 | -0.13432782 | 0.963381393 | | down |
| SNORA40 | -0.13432782 | 0.963381393 | | down |
| NBPF17P | -0.13432782 | 0.963381393 | | down |
| RP11-640M9.4 | -0.13432782 | 0.963381393 | | down |
| RP11-554K11.2 | -0.13432782 | 0.963381393 | | down |
| RNU1-132P | -0.13432782 | 0.963381393 | | down |
| MIR4261 | -0.13432782 | 0.963381393 | | down |
| CISD1P1 | -0.13432782 | 0.963381393 | | down |
| IGKV2D-23 | -0.13432782 | 0.963381393 | | down |
| NA | -0.13432782 | 0.963381393 | | down |
| MTCYBP11 | -0.13432782 | 0.963381393 | | down |
| MTND5P24 | -0.13432782 | 0.963381393 | | down |
| AC023128.1 | -0.13432782 | 0.963381393 | | down |
| uc_338 | -0.13432782 | 0.963381393 | | down |

| | | | |
|---|---|---|---|
| MTCO3P17 | -0.13432782 | 0.963381393 | down |
| RP11-95H11.1 | -0.13432782 | 0.963381393 | down |
| RNU6-107P | -0.13432782 | 0.963381393 | down |
| RNU4-56P | -0.13432782 | 0.963381393 | down |
| RP11-389G6.4 | -0.13432782 | 0.963381393 | down |
| MTCO3P35 | -0.13432782 | 0.963381393 | down |
| RPL7L1P7 | -0.13432782 | 0.963381393 | down |
| RNU6-486P | -0.13432782 | 0.963381393 | down |
| RP11-78H24.1 | -0.13432782 | 0.963381393 | down |
| FGF12-AS1 | -0.13432782 | 0.963381393 | down |
| RP11-317G22.2 | -0.13432782 | 0.963381393 | down |
| RN7SL728P | -0.13432782 | 0.963381393 | down |
| RP11-785F11.1 | -0.13432782 | 0.963381393 | down |
| RNU6-1282P | -0.13432782 | 0.963381393 | down |
| MIR3140 | -0.13432782 | 0.963381393 | down |
| NA | -0.13432782 | 0.963381393 | down |
| MIR4277 | -0.13432782 | 0.963381393 | down |
| RP11-191F9.1 | -0.13432782 | 0.963381393 | down |
| CTC-232H10.1 | -0.13432782 | 0.963381393 | down |
| RNU6-272P | -0.13432782 | 0.963381393 | down |
| LINC01332 | -0.13432782 | 0.963381393 | down |
| TRMT112P2 | -0.13432782 | 0.963381393 | down |
| CTC-254B4.1 | -0.13432782 | 0.963381393 | down |
| RP11-2N5.1 | -0.13432782 | 0.963381393 | down |
| IL9 | -0.13432782 | 0.963381393 | down |
| RP11-118M9.3 | -0.13432782 | 0.963381393 | down |
| CTB-99A3.1 | -0.13432782 | 0.963381393 | down |
| CTC-224D3.1 | -0.13432782 | 0.963381393 | down |
| MIR7853 | -0.13432782 | 0.963381393 | down |
| RP11-191A15.4 | -0.13432782 | 0.963381393 | down |
| RPL7AP7 | -0.13432782 | 0.963381393 | down |
| RP11-793K1.1 | -0.13432782 | 0.963381393 | down |
| RCC2P7 | -0.13432782 | 0.963381393 | down |
| RP5-991C6.2 | -0.13432782 | 0.963381393 | down |
| RNU6-344P | -0.13432782 | 0.963381393 | down |
| RNA5SP212 | -0.13432782 | 0.963381393 | down |
| RP11-346C16.2 | -0.13432782 | 0.963381393 | down |
| RP1-177A13.1 | -0.13432782 | 0.963381393 | down |
| RP11-277I20.2 | -0.13432782 | 0.963381393 | down |
| RP11-291C6.1 | -0.13432782 | 0.963381393 | down |
| NA | -0.13432782 | 0.963381393 | down |
| RPL23AP47 | -0.13432782 | 0.963381393 | down |
| RP11-740N7.4 | -0.13432782 | 0.963381393 | down |
| AC079448.1 | -0.13432782 | 0.963381393 | down |
| RNU7-143P | -0.13432782 | 0.963381393 | down |
| AC004692.4 | -0.13432782 | 0.963381393 | down |
| AC092657.2 | -0.13432782 | 0.963381393 | down |
| CTB-152G17.4 | -0.13432782 | 0.963381393 | down |
| RPL3P8 | -0.13432782 | 0.963381393 | down |
| RP11-264K23.1 | -0.13432782 | 0.963381393 | down |
| RP11-286H14.1 | -0.13432782 | 0.963381393 | down |

| | | | |
|---|---|---|---|
| RNU6-729P | -0.13432782 | 0.963381393 | down |
| RP11-465K4.4 | -0.13432782 | 0.963381393 | down |
| SRPK2P | -0.13432782 | 0.963381393 | down |
| IARS2P1 | -0.13432782 | 0.963381393 | down |
| RP11-31K23.1 | -0.13432782 | 0.963381393 | down |
| RP11-771F20.1 | -0.13432782 | 0.963381393 | down |
| RNU6-1255P | -0.13432782 | 0.963381393 | down |
| RNA5SP278 | -0.13432782 | 0.963381393 | down |
| AC145123.2 | -0.13432782 | 0.963381393 | down |
| RNU6-1327P | -0.13432782 | 0.963381393 | down |
| NOP56P2 | -0.13432782 | 0.963381393 | down |
| NA | -0.13432782 | 0.963381393 | down |
| OSTCP8 | -0.13432782 | 0.963381393 | down |
| RPS19P6 | -0.13432782 | 0.963381393 | down |
| Y_RNA | -0.13432782 | 0.963381393 | down |
| RP11-100G15.10 | -0.13432782 | 0.963381393 | down |
| RP11-19J3.5 | -0.13432782 | 0.963381393 | down |
| NA | -0.13432782 | 0.963381393 | down |
| RP11-631M21.1 | -0.13432782 | 0.963381393 | down |
| RP11-89K18.1 | -0.13432782 | 0.963381393 | down |
| RP11-445P17.5 | -0.13432782 | 0.963381393 | down |
| RP11-144G6.13 | -0.13432782 | 0.963381393 | down |
| RP11-399K21.13 | -0.13432782 | 0.963381393 | down |
| RP11-93H12.4 | -0.13432782 | 0.963381393 | down |
| RNA5SP324 | -0.13432782 | 0.963381393 | down |
| NA | -0.13432782 | 0.963381393 | down |
| PTGES3P4 | -0.13432782 | 0.963381393 | down |
| RP11-438N5.4 | -0.13432782 | 0.963381393 | down |
| OR52A5 | -0.13432782 | 0.963381393 | down |
| OR52B1P | -0.13432782 | 0.963381393 | down |
| OR4D6 | -0.13432782 | 0.963381393 | down |
| SNORA43 | -0.13432782 | 0.963381393 | down |
| RP11-939C17.4 | -0.13432782 | 0.963381393 | down |
| RP11-535A19.1 | -0.13432782 | 0.963381393 | down |
| RP11-686G23.2 | -0.13432782 | 0.963381393 | down |
| RP11-856F16.2 | -0.13432782 | 0.963381393 | down |
| RP11-60C6.8 | -0.13432782 | 0.963381393 | down |
| RNU6-893P | -0.13432782 | 0.963381393 | down |
| RN7SL778P | -0.13432782 | 0.963381393 | down |
| AC007618.3 | -0.13432782 | 0.963381393 | down |
| RP11-476M19.2 | -0.13432782 | 0.963381393 | down |
| RP11-318G8.2 | -0.13432782 | 0.963381393 | down |
| RP11-820K3.4 | -0.13432782 | 0.963381393 | down |
| MARK3P1 | -0.13432782 | 0.963381393 | down |
| RP11-812D23.1 | -0.13432782 | 0.963381393 | down |
| MIR331 | -0.13432782 | 0.963381393 | down |
| MIR6761 | -0.13432782 | 0.963381393 | down |
| RP11-87C12.6 | -0.13432782 | 0.963381393 | down |
| RP13-895J2.11 | -0.13432782 | 0.963381393 | down |
| LAMTOR3P1 | -0.13432782 | 0.963381393 | down |
| ANKRD26P2 | -0.13432782 | 0.963381393 | down |

| | | | |
|---|---|---|---|
| LINC00333 | -0.13432782 | 0.963381393 | down |
| GPC5-AS2 | -0.13432782 | 0.963381393 | down |
| GPC5-AS1 | -0.13432782 | 0.963381393 | down |
| FGF14-AS1 | -0.13432782 | 0.963381393 | down |
| TRAJ50 | -0.13432782 | 0.963381393 | down |
| TRAJ20 | -0.13432782 | 0.963381393 | down |
| RNA5SP386 | -0.13432782 | 0.963381393 | down |
| RN7SL706P | -0.13432782 | 0.963381393 | down |
| HIF1AP1 | -0.13432782 | 0.963381393 | down |
| IGHVIII-22-2 | -0.13432782 | 0.963381393 | down |
| FAM30C | -0.13432782 | 0.963381393 | down |
| Y_RNA | -0.13432782 | 0.963381393 | down |
| RP11-108K3.4 | -0.13432782 | 0.963381393 | down |
| Y_RNA | -0.13432782 | 0.963381393 | down |
| U6 | -0.13432782 | 0.963381393 | down |
| RP11-462G12.4 | -0.13432782 | 0.963381393 | down |
| IGHV1OR16-1 | -0.13432782 | 0.963381393 | down |
| NA | -0.13432782 | 0.963381393 | down |
| RP11-133M8.3 | -0.13432782 | 0.963381393 | down |
| AC005324.6 | -0.13432782 | 0.963381393 | down |
| RNU6-767P | -0.13432782 | 0.963381393 | down |
| AC005730.2 | -0.13432782 | 0.963381393 | down |
| MIR2909 | -0.13432782 | 0.963381393 | down |
| Metazoa_SRP | -0.13432782 | 0.963381393 | down |
| AC102948.2 | -0.13432782 | 0.963381393 | down |
| Y_RNA | -0.13432782 | 0.963381393 | down |
| RNU6-446P | -0.13432782 | 0.963381393 | down |
| TRMT112P3 | -0.13432782 | 0.963381393 | down |
| RP11-269G24.7 | -0.13432782 | 0.963381393 | down |
| AC015933.2 | -0.13432782 | 0.963381393 | down |
| AKR1B10P2 | -0.13432782 | 0.963381393 | down |
| AC139085.2 | -0.13432782 | 0.963381393 | down |
| LINC00905 | -0.13432782 | 0.963381393 | down |
| NA | -0.13432782 | 0.963381393 | down |
| BNIP3P34 | -0.13432782 | 0.963381393 | down |
| CTD-2540B15.10 | -0.13432782 | 0.963381393 | down |
| CTC-490E21.13 | -0.13432782 | 0.963381393 | down |
| CYP2A7P1 | -0.13432782 | 0.963381393 | down |
| RFPL4AP1 | -0.13432782 | 0.963381393 | down |
| RNU105B | -0.13432782 | 0.963381393 | down |
| CST13P | -0.13432782 | 0.963381393 | down |
| RN7SL197P | -0.13432782 | 0.963381393 | down |
| MIR298 | -0.13432782 | 0.963381393 | down |
| NA | -0.13432782 | 0.963381393 | down |
| FEM1AP1 | -0.13432782 | 0.963381393 | down |
| NA | -0.13432782 | 0.963381393 | down |
| SLC6A6P1 | -0.13432782 | 0.963381393 | down |
| AP000563.2 | -0.13432782 | 0.963381393 | down |
| AP000281.2 | -0.13432782 | 0.963381393 | down |
| RNU6-696P | -0.13432782 | 0.963381393 | down |
| KRTAP10-7 | -0.13432782 | 0.963381393 | down |

| | | | | |
|---|---|---|---|---|
| AP001468.58 | -0.13432782 | 0.963381393 | | down |
| NA | -0.13432782 | 0.963381393 | | down |
| LA16c-3G11.7 | -0.13432782 | 0.963381393 | | down |
| KB-67B5.12 | -0.13432782 | 0.963381393 | | down |
| RNU6-225P | -0.13432782 | 0.963381393 | | down |
| RP3-412A9.12 | -0.13432782 | 0.963381393 | | down |
| LINC00229 | -0.13432782 | 0.963381393 | | down |
| MIR548AM | -0.13432782 | 0.963381393 | | down |
| RP11-87M18.2 | -0.13432782 | 0.963381393 | | down |
| RBM22P6 | -0.13432782 | 0.963381393 | | down |
| RNA5SP508 | -0.13432782 | 0.963381393 | | down |
| HNRNPH3P1 | -0.13432782 | 0.963381393 | | down |
| U3 | -0.13432782 | 0.963381393 | | down |
| RP3-378P9.2 | -0.13432782 | 0.963381393 | | down |
| RPS7P12 | -0.13432782 | 0.963381393 | | down |
| MIR505 | -0.13432782 | 0.963381393 | | down |
| RP13-150K15.1 | -0.13432782 | 0.963381393 | | down |
| AFF2-IT1 | -0.13432782 | 0.963381393 | | down |
| RP11-218L14.4 | -0.13432782 | 0.963381393 | | down |
| TAF9P1 | -0.13432782 | 0.963381393 | | down |
| RP11-256K9.1 | -0.13432782 | 0.963381393 | | down |
| TRIM66 | -0.133913006 | 0.393948143 | 0.531332962 | down |
| CACNA1C-AS1 | -0.133910714 | 0.772165873 | 0.846800682 | down |
| RP11-445P17.8 | -0.13378155 | 0.878966193 | | down |
| CTD-2012K14.6 | -0.133776366 | 0.764936558 | 0.841743636 | down |
| MED6 | -0.133647238 | 0.290249895 | 0.427422207 | down |
| RP11-70D24.2 | -0.133627934 | 0.722597155 | 0.810820128 | down |
| HIST1H1D | -0.133535822 | 0.766618153 | 0.843101193 | down |
| SCAMP1-AS1 | -0.133400609 | 0.364836094 | 0.503242992 | down |
| AREL1 | -0.133350912 | 0.224846674 | 0.356991704 | down |
| TEFM | -0.133320564 | 0.586020312 | 0.701589729 | down |
| ADAP2 | -0.133258516 | 0.440115045 | 0.573906962 | down |
| CHCHD1 | -0.133210543 | 0.395931653 | 0.532995354 | down |
| PTPMT1 | -0.133205819 | 0.670527112 | 0.770228123 | down |
| IL9RP3 | -0.133111751 | 0.813887878 | 0.877532655 | down |
| CCDC110 | -0.132987052 | 0.733195983 | 0.818707355 | down |
| AC112721.2 | -0.132879527 | 0.821974453 | 0.88330875 | down |
| FAM177A1 | -0.13283706 | 0.288356052 | 0.425551022 | down |
| RASAL2 | -0.132744228 | 0.646139479 | 0.75108777 | down |
| COMMD9 | -0.13262619 | 0.399379648 | 0.536338375 | down |
| AC015971.2 | -0.132603195 | 0.846786221 | 0.90119891 | down |
| RP11-568J23.5 | -0.132531413 | 0.861805559 | 0.911532695 | down |
| JRK | -0.132453087 | 0.448410849 | 0.581406817 | down |
| SLC46A1 | -0.132439658 | 0.544187568 | 0.665644951 | down |
| LINC00869 | -0.132397544 | 0.488676897 | 0.617297709 | down |
| AICDA | -0.132364179 | 0.791213976 | 0.860801017 | down |
| CLIC1 | -0.132278688 | 0.373821433 | 0.512281896 | down |
| CGB7 | -0.132271205 | 0.868239453 | 0.915653587 | down |
| CRYZ | -0.132254545 | 0.640393851 | 0.746459471 | down |
| ZNF284 | -0.132241447 | 0.605792442 | 0.718248939 | down |
| NA | -0.132240433 | 0.768744387 | 0.84449729 | down |

| | | | | |
|---|---|---|---|---|
| U2AF1L5 | -0.132090519 | 0.789120821 | 0.859307677 | down |
| PNMA5 | -0.131957813 | 0.768022898 | 0.844031588 | down |
| ZNF48 | -0.13183478 | 0.394814904 | 0.532230403 | down |
| ARPC1B | -0.131706304 | 0.64283026 | 0.748428302 | down |
| C7orf43 | -0.1315998 | 0.451039918 | 0.583970838 | down |
| TTC21B | -0.131546718 | 0.383906149 | 0.521620674 | down |
| PTMAP8 | -0.131438471 | 0.852925136 | | down |
| RPS20 | -0.13135273 | 0.437704476 | 0.572154249 | down |
| PLA2G2A | -0.13131992 | 0.755376491 | 0.834633933 | down |
| SLAIN1 | -0.131213406 | 0.703902312 | 0.796296448 | down |
| RNU6-725P | -0.131189555 | 0.964236324 | | down |
| HMGN1P10 | -0.131189555 | 0.964236324 | | down |
| MED28P2 | -0.131189555 | 0.964236324 | | down |
| XX-FYM637E10_5.1 | -0.131189555 | 0.964236324 | | down |
| RP11-804F13.1 | -0.131189555 | 0.964236324 | | down |
| OR4F6 | -0.131189555 | 0.964236324 | | down |
| HYPM | -0.131189555 | 0.964236324 | | down |
| RP11-522B15.6 | -0.131189377 | 0.963696074 | | down |
| NA | -0.131189242 | 0.963273956 | | down |
| Y_RNA | -0.131189204 | 0.963150487 | | down |
| RP11-379I19.3 | -0.131189101 | 0.962811509 | | down |
| RP11-57G22.1 | -0.131187936 | 0.958206782 | | down |
| CLDN24 | -0.131187907 | 0.958070074 | | down |
| RP11-230L22.4 | -0.131187893 | 0.958003944 | | down |
| CTD-2324A24.2 | -0.131187762 | 0.957362506 | | down |
| MIR3135A | -0.131187588 | 0.95646227 | | down |
| SNRPFP2 | -0.131186906 | 0.952298404 | | down |
| AC022153.1 | -0.13118679 | 0.951465194 | | down |
| RP11-178C3.2 | -0.131185708 | 0.940724804 | | down |
| RP4-613A2.1 | -0.131185315 | 0.934603066 | | down |
| BCLAF1P1 | -0.131185031 | 0.928780655 | | down |
| RP11-112J1.3 | -0.131185029 | 0.928723516 | | down |
| AC015969.3 | -0.131185009 | 0.928255149 | | down |
| RP11-298J23.5 | -0.131185 | 0.928033885 | | down |
| ENPP7P1 | -0.13118498 | 0.92755444 | | down |
| TCL1B | -0.131175352 | 0.892653131 | | down |
| METTL15P1 | -0.131138263 | 0.868651024 | | down |
| SELM | -0.13112283 | 0.668943188 | 0.769033525 | down |
| LINC00313 | -0.130986861 | 0.834393759 | 0.891832726 | down |
| TSPYL1 | -0.130964427 | 0.093459865 | 0.192639146 | down |
| LGMN | -0.130912532 | 0.517738345 | 0.642625965 | down |
| C1QB | -0.130889327 | 0.633181105 | 0.740724754 | down |
| TTC16 | -0.130774831 | 0.791017658 | 0.860719804 | down |
| FAM217B | -0.130739436 | 0.401569023 | 0.538460222 | down |
| DNHD1 | -0.130680444 | 0.520075438 | 0.644548631 | down |
| DIAPH3 | -0.130594772 | 0.640311446 | 0.746408349 | down |
| NAPB | -0.130580971 | 0.358527177 | 0.496845302 | down |
| MBL1P | -0.130487656 | 0.771545094 | 0.846336149 | down |
| USP3 | -0.130452641 | 0.131487924 | 0.245276492 | down |
| DISP1 | -0.130396329 | 0.606228246 | 0.718595495 | down |
| CARNMT1 | -0.130396002 | 0.318750844 | 0.457599905 | down |

| | | | | |
|---|---|---|---|---|
| PSPHP1 | -0.130391325 | 0.908947264 | 0.94287234 | down |
| B4GAT1 | -0.130335536 | 0.421511769 | 0.557144724 | down |
| S1PR2 | -0.130313091 | 0.611364029 | 0.722988115 | down |
| KCNK9 | -0.130269147 | 0.786739319 | 0.857337544 | down |
| RP11-392E22.9 | -0.130218799 | 0.796421988 | 0.864473116 | down |
| RPL26P30 | -0.130189153 | 0.778933304 | 0.851766048 | down |
| ADIPOR1P1 | -0.130053568 | 0.954112195 | 0.972674267 | down |
| KLHL23 | -0.130004408 | 0.670862719 | 0.770525251 | down |
| HAR1B | -0.13000002 | 0.787917259 | 0.858309867 | down |
| RP11-369K17.1 | -0.129933832 | 0.947166327 | | down |
| PRPF3 | -0.12989327 | 0.204572035 | 0.334230368 | down |
| LINC01422 | -0.129878309 | 0.712390022 | 0.80278481 | down |
| RP11-430H12.2 | -0.129866409 | 0.931859131 | | down |
| RP5-837J1.1 | -0.129863262 | 0.931227262 | | down |
| UNGP3 | -0.129810982 | 0.921479545 | | down |
| NA | -0.129810036 | 0.921314415 | | down |
| RP11-90L20.2 | -0.1297829 | 0.916724478 | | down |
| RNU1-134P | -0.12977604 | 0.915604974 | | down |
| OR2G6 | -0.12977036 | 0.914689682 | | down |
| RTN3P1 | -0.129739996 | 0.909960894 | | down |
| HIST1H4K | -0.129731308 | 0.798437858 | 0.86622132 | down |
| OR2L13 | -0.129692056 | 0.902985256 | | down |
| RP1-313L4.4 | -0.129656496 | 0.898136936 | | down |
| NA | -0.129543228 | 0.884128329 | | down |
| LINC00649 | -0.129512596 | 0.537224217 | 0.659373912 | down |
| PNMA3 | -0.129410099 | 0.747025381 | 0.828331077 | down |
| VSTM2B | -0.129401027 | 0.882299352 | | down |
| SMARCB1 | -0.129247613 | 0.339338637 | 0.477835115 | down |
| WDR31 | -0.129246016 | 0.6160943 | 0.726991758 | down |
| KLHL14 | -0.129138215 | 0.784625964 | 0.8559038 | down |
| CES1 | -0.129118097 | 0.700853075 | 0.7940637 | down |
| TMEM9 | -0.128952231 | 0.538700039 | 0.660735006 | down |
| NA | -0.128874259 | 0.801770149 | 0.868707215 | down |
| SCG3 | -0.128829668 | 0.74935487 | 0.830423729 | down |
| KEAP1 | -0.12881075 | 0.175966163 | 0.300691865 | down |
| ZFYVE26 | -0.128723677 | 0.170704712 | 0.294563917 | down |
| CNOT1 | -0.128650808 | 0.681634645 | 0.77946123 | down |
| SOS2 | -0.128587675 | 0.557886767 | 0.677494519 | down |
| XRCC3 | -0.128582685 | 0.534938436 | 0.657624714 | down |
| RP11-360L9.4 | -0.128453794 | 0.817771676 | 0.880487824 | down |
| ACOT1 | -0.128412737 | 0.623802819 | 0.733380234 | down |
| ID3 | -0.128373985 | 0.534016311 | 0.656759837 | down |
| RN7SL453P | -0.128338217 | 0.95790717 | | down |
| RNF141 | -0.128180001 | 0.439869849 | 0.573736638 | down |
| RP11-218M22.2 | -0.128068559 | 0.907592483 | | down |
| PRRT1 | -0.128042062 | 0.577748396 | 0.694370462 | down |
| RP11-425M5.7 | -0.127894598 | 0.875854339 | 0.920880762 | down |
| COX11 | -0.127874028 | 0.311603577 | 0.450237197 | down |
| ARHGAP31-AS1 | -0.127819944 | 0.777652059 | 0.85095347 | down |
| SYAP1 | -0.127729224 | 0.224757387 | 0.356903327 | down |
| NA | -0.127677494 | 0.9213574 | | down |

| | | | | |
|---|---|---|---|---|
| PTMAP1 | -0.127657227 | 0.887978764 | 0.928203813 | down |
| SARS | -0.127615656 | 0.301861146 | 0.43967543 | down |
| SIPA1L2 | -0.127551109 | 0.555009717 | 0.674880514 | down |
| GLB1 | -0.12737944 | 0.342243304 | 0.480663698 | down |
| NUP155 | -0.127205988 | 0.401387681 | 0.538251325 | down |
| TRIP4 | -0.127201262 | 0.427339355 | 0.562817095 | down |
| FAM160B2 | -0.127149774 | 0.144610134 | 0.262173486 | down |
| ZDHHC13 | -0.127016948 | 0.489190613 | 0.617816754 | down |
| EFCAB13 | -0.126953296 | 0.697730494 | 0.791818953 | down |
| RBMS3 | -0.126772842 | 0.633694119 | 0.741180602 | down |
| CD3G | -0.126752261 | 0.646954823 | 0.751744689 | down |
| CBFA2T3 | -0.126692597 | 0.472219193 | 0.602623827 | down |
| ZNF780A | -0.126682557 | 0.475583341 | 0.605544409 | down |
| PROCA1 | -0.126388459 | 0.613461768 | 0.724710811 | down |
| PLK1 | -0.126277698 | 0.536382391 | 0.658796381 | down |
| ARAP2 | -0.126260174 | 0.587447351 | 0.702962051 | down |
| HMCN1 | -0.126152155 | 0.764302492 | 0.841309594 | down |
| RP1-302G2.5 | -0.126065469 | 0.741485278 | 0.824409136 | down |
| NF2 | -0.126016987 | 0.100557671 | 0.20274845 | down |
| SLC39A14 | -0.125735841 | 0.601047643 | 0.714145194 | down |
| RP4-773N10.4 | -0.125667787 | 0.59919234 | 0.712617159 | down |
| SEPT7P2 | -0.125630752 | 0.592644971 | 0.707519094 | down |
| EGOT | -0.125628257 | 0.857741476 | 0.908810521 | down |
| RP11-177H13.2 | -0.125516806 | 0.793095743 | 0.862106752 | down |
| RP4-655C5.4 | -0.125482554 | 0.889087051 | 0.92900676 | down |
| ZNF815P | -0.125416434 | 0.598870187 | 0.712375021 | down |
| ANAPC16 | -0.125349415 | 0.122703914 | 0.233649157 | down |
| CTTN | -0.1252527 | 0.37450171 | 0.512808412 | down |
| SKP1 | -0.125148422 | 0.330842511 | 0.469163447 | down |
| CD63 | -0.125099585 | 0.582912067 | 0.698815297 | down |
| RBM26-AS1 | -0.125064249 | 0.69217933 | 0.787490047 | down |
| RP11-424G14.1 | -0.125062672 | 0.870552856 | 0.91736137 | down |
| CD7 | -0.125019253 | 0.700276717 | 0.793659274 | down |
| B3GNT6 | -0.124927192 | 0.796229961 | 0.864382428 | down |
| RP3-477M7.6 | -0.12491877 | 0.965944743 | | down |
| RP1-134O19.3 | -0.12491877 | 0.965944743 | | down |
| RP11-420K8.1 | -0.12491877 | 0.965944743 | | down |
| CYP46A4P | -0.12491877 | 0.965944743 | | down |
| NA | -0.12491877 | 0.965944743 | | down |
| RP11-182I10.2 | -0.12491877 | 0.965944743 | | down |
| RP11-12L8.1 | -0.12491877 | 0.965944743 | | down |
| SUPT4H1P1 | -0.12491877 | 0.965944743 | | down |
| RP11-521O16.1 | -0.12491877 | 0.965944743 | | down |
| RNA5SP118 | -0.12491877 | 0.965944743 | | down |
| RPL7L1P9 | -0.12491877 | 0.965944743 | | down |
| IQCA1-AS1 | -0.12491877 | 0.965944743 | | down |
| EIF2S2P2 | -0.12491877 | 0.965944743 | | down |
| RP11-79E3.1 | -0.12491877 | 0.965944743 | | down |
| RP11-83C7.3 | -0.12491877 | 0.965944743 | | down |
| Metazoa_SRP | -0.12491877 | 0.965944743 | | down |
| AC004066.2 | -0.12491877 | 0.965944743 | | down |

| | | | |
|---|---|---|---|
| RNU6-583P | -0.12491877 | 0.965944743 | down |
| RP11-366M4.12 | -0.12491877 | 0.965944743 | down |
| RN7SKP79 | -0.12491877 | 0.965944743 | down |
| MIR580 | -0.12491877 | 0.965944743 | down |
| RP11-232L2.2 | -0.12491877 | 0.965944743 | down |
| CTD-2372A4.1 | -0.12491877 | 0.965944743 | down |
| NA | -0.12491877 | 0.965944743 | down |
| POLD2P1 | -0.12491877 | 0.965944743 | down |
| RN7SL803P | -0.12491877 | 0.965944743 | down |
| RN7SKP110 | -0.12491877 | 0.965944743 | down |
| NA | -0.12491877 | 0.965944743 | down |
| AC006041.1 | -0.12491877 | 0.965944743 | down |
| NA | -0.12491877 | 0.965944743 | down |
| AC079781.7 | -0.12491877 | 0.965944743 | down |
| RNU6-1322P | -0.12491877 | 0.965944743 | down |
| RNU6-517P | -0.12491877 | 0.965944743 | down |
| PRELID3BP10 | -0.12491877 | 0.965944743 | down |
| OR2A3P | -0.12491877 | 0.965944743 | down |
| RP11-402L5.2 | -0.12491877 | 0.965944743 | down |
| RP11-513H8.1 | -0.12491877 | 0.965944743 | down |
| NA | -0.12491877 | 0.965944743 | down |
| NA | -0.12491877 | 0.965944743 | down |
| RP11-49014.3 | -0.12491877 | 0.965944743 | down |
| RNA5SP288 | -0.12491877 | 0.965944743 | down |
| RP11-205A8.4 | -0.12491877 | 0.965944743 | down |
| RP11-452K12.6 | -0.12491877 | 0.965944743 | down |
| RP11-264E18.1 | -0.12491877 | 0.965944743 | down |
| OR52E4 | -0.12491877 | 0.965944743 | down |
| DEFB108B | -0.12491877 | 0.965944743 | down |
| CBX3P7 | -0.12491877 | 0.965944743 | down |
| U4 | -0.12491877 | 0.965944743 | down |
| RP11-474P2.4 | -0.12491877 | 0.965944743 | down |
| RPL29P25 | -0.12491877 | 0.965944743 | down |
| OR7E155P | -0.12491877 | 0.965944743 | down |
| RP11-78J21.6 | -0.12491877 | 0.965944743 | down |
| TRAJ48 | -0.12491877 | 0.965944743 | down |
| TRAJ43 | -0.12491877 | 0.965944743 | down |
| RPL7AP4 | -0.12491877 | 0.965944743 | down |
| RNU4-22P | -0.12491877 | 0.965944743 | down |
| IGHVII-31-1 | -0.12491877 | 0.965944743 | down |
| RP11-433P17.1 | -0.12491877 | 0.965944743 | down |
| ACA64 | -0.12491877 | 0.965944743 | down |
| RP11-1113L8.7 | -0.12491877 | 0.965944743 | down |
| RP5-1028K7.1 | -0.12491877 | 0.965944743 | down |
| RP11-670E13.3 | -0.12491877 | 0.965944743 | down |
| NA | -0.12491877 | 0.965944743 | down |
| RP11-742D12.1 | -0.12491877 | 0.965944743 | down |
| RP11-102.1 | -0.12491877 | 0.965944743 | down |
| RP11-1137G4.3 | -0.12491877 | 0.965944743 | down |
| AC003002.6 | -0.12491877 | 0.965944743 | down |
| PROKR2 | -0.12491877 | 0.965944743 | down |

| | | | | |
|---|---|---|---|---|
| EIF4E2P1 | -0.12491877 | 0.965944743 | | down |
| RPL8P2 | -0.12491877 | 0.965944743 | | down |
| GGTLC5P | -0.12491877 | 0.965944743 | | down |
| GYG2-AS1 | -0.12491877 | 0.965944743 | | down |
| RP11-126O22.1 | -0.12491877 | 0.965944743 | | down |
| CBX1P4 | -0.12491877 | 0.965944743 | | down |
| RP5-1174J21.2 | -0.12491877 | 0.965944743 | | down |
| Metazoa_SRP | -0.12491877 | 0.965944743 | | down |
| RP1-172N19.5 | -0.12491877 | 0.965944743 | | down |
| MIR548AN | -0.12491877 | 0.965944743 | | down |
| RPL3P12 | -0.12491877 | 0.965944743 | | down |
| STARD7 | -0.12491717 | 0.211871574 | 0.342579493 | down |
| GRWD1 | -0.124900657 | 0.460961555 | 0.592805794 | down |
| PPP1R13L | -0.124732048 | 0.374223054 | 0.512624188 | down |
| PIAS4 | -0.124654864 | 0.169672416 | 0.293304837 | down |
| RP4-536B24.2 | -0.124579857 | 0.939540094 | | down |
| TMEM44 | -0.12446897 | 0.462450209 | 0.594211534 | down |
| NA | -0.124244497 | 0.934042388 | | down |
| RP11-818O24.3 | -0.124136351 | 0.824486986 | 0.884907888 | down |
| ART4 | -0.124081697 | 0.773297869 | 0.847794525 | down |
| SLC35F5 | -0.124081636 | 0.558592219 | 0.678184136 | down |
| IP6K2 | -0.124067065 | 0.386042706 | 0.523718372 | down |
| STX5 | -0.124066706 | 0.426538648 | 0.562132173 | down |
| PACSIN2 | -0.124024505 | 0.303107151 | 0.441102022 | down |
| AC067945.3 | -0.123970695 | 0.920293055 | | down |
| PCDHB8 | -0.123947543 | 0.855022095 | 0.907200305 | down |
| FABP5P7 | -0.123924232 | 0.65998001 | 0.761870415 | down |
| Y_RNA | -0.123908714 | 0.865803282 | | down |
| POLQ | -0.123829124 | 0.698656531 | 0.792540563 | down |
| TMEM110 | -0.123804697 | 0.775010026 | 0.849175828 | down |
| ADGRF5P1 | -0.123768846 | 0.919268058 | | down |
| RPS3AP5 | -0.123704473 | 0.635371548 | 0.742403601 | down |
| AC079807.2 | -0.12370041 | 0.630521802 | 0.738508573 | down |
| CDCA2 | -0.123686891 | 0.619003227 | 0.729450204 | down |
| LINC00174 | -0.123675662 | 0.590641139 | 0.705687679 | down |
| CUEDC1 | -0.123655816 | 0.488472765 | 0.617139258 | down |
| RP11-265E18.1 | -0.12358482 | 0.839761308 | 0.896167293 | down |
| EPB41 | -0.123555397 | 0.654489196 | 0.757569479 | down |
| CAMK1D | -0.123510825 | 0.396754152 | 0.533671282 | down |
| TMEM223 | -0.123500794 | 0.51153239 | 0.637220298 | down |
| RPS27A | -0.123473556 | 0.437547595 | 0.572088195 | down |
| DMPK | -0.123460725 | 0.61926853 | 0.729591143 | down |
| RP11-843A23.1 | -0.12344345 | 0.915264819 | | down |
| NCOA4 | -0.12335621 | 0.58810425 | 0.703394337 | down |
| CTA-722E9.1 | -0.123305995 | 0.861150177 | | down |
| RP11-1212A22.1 | -0.123260884 | 0.789266541 | 0.859370199 | down |
| ANKRD17 | -0.123242991 | 0.449985646 | 0.582895249 | down |
| RP11-540O11.4 | -0.123203178 | 0.886406019 | | down |
| RP11-585P4.6 | -0.123155131 | 0.759283993 | 0.837442301 | down |
| RP11-159H3.1 | -0.123027281 | 0.754660649 | 0.834119069 | down |
| NPRL2 | -0.122972383 | 0.25929378 | 0.39452154 | down |

| | | | | |
|---|---|---|---|---|
| AC005772.2 | -0.122829901 | 0.956437513 | 0.974005996 | down |
| RPL5P34 | -0.122678831 | 0.60035891 | 0.713591733 | down |
| RP11-1398P2.1 | -0.122666192 | 0.625122393 | 0.734414311 | down |
| RP11-820I16.1 | -0.122627183 | 0.89674457 | | down |
| DNAJC30 | -0.122581301 | 0.563494591 | 0.682269561 | down |
| RPL23AP2 | -0.122545054 | 0.823872129 | 0.884551844 | down |
| PCDHGB6 | -0.122460301 | 0.73396595 | 0.819239262 | down |
| FAM35DP | -0.122456233 | 0.847848068 | 0.901785937 | down |
| CCNF | -0.122346272 | 0.596271368 | 0.710333323 | down |
| RP5-882C2.2 | -0.122281376 | 0.602053162 | 0.715030098 | down |
| RP11-303G3.6 | -0.122214177 | 0.753090502 | 0.832812539 | down |
| TRBJ1-1 | -0.12208803 | 0.878856217 | | down |
| NFKBID | -0.122004343 | 0.648726178 | 0.753045719 | down |
| DUTP8 | -0.121780505 | 0.966799794 | | down |
| RP11-5N11.3 | -0.121780505 | 0.966799794 | | down |
| RP11-85O21.2 | -0.121780505 | 0.966799794 | | down |
| RP5-862K6.4 | -0.121780505 | 0.966799794 | | down |
| RP11-284H19.1 | -0.121780072 | 0.965502619 | | down |
| CTB-49A3.2 | -0.121777915 | 0.955614653 | | down |
| RP11-478C19.2 | -0.121777235 | 0.950166582 | | down |
| RP11-146F11.4 | -0.121777154 | 0.949371952 | | down |
| RP11-127B16.1 | -0.121776796 | 0.945352518 | | down |
| RP11-404P21.1 | -0.121776769 | 0.945008983 | | down |
| AC093627.7 | -0.121776745 | 0.944703406 | | down |
| KB-1466C5.1 | -0.121776737 | 0.944595461 | | down |
| AMMECR1-IT1 | -0.121776675 | 0.943759932 | | down |
| RP11-108K14.12 | -0.121776406 | 0.93966045 | | down |
| RP11-513D5.5 | -0.121776374 | 0.939113491 | | down |
| CLEC3A | -0.121776362 | 0.938905486 | | down |
| NA | -0.121776101 | 0.933724739 | | down |
| AL355480.3 | -0.121776097 | 0.933635708 | | down |
| RPSAP29 | -0.121775518 | 0.914776693 | | down |
| RWDD4P1 | -0.121775431 | 0.910319953 | | down |
| CTC-436P18.1 | -0.121775322 | 0.903517657 | | down |
| RP11-424D14.1 | -0.121736513 | 0.946549287 | | down |
| DCTN2 | -0.121711466 | 0.29019169 | 0.42739934 | down |
| RASL11A | -0.121663191 | 0.440814849 | 0.574453549 | down |
| RP11-425A6.5 | -0.121641851 | 0.890498862 | | down |
| C8orf58 | -0.121617981 | 0.506432586 | 0.632703785 | down |
| TTC27 | -0.121597052 | 0.357227221 | 0.495614621 | down |
| CASC10 | -0.121544659 | 0.727038267 | 0.814252505 | down |
| RP11-807C20.2 | -0.121535441 | 0.864788557 | 0.913466784 | down |
| RP11-344P13.6 | -0.121391887 | 0.81901715 | 0.881327798 | down |
| RP11-325L12.7 | -0.121244086 | 0.857694629 | 0.908810521 | down |
| CTD-2540L5.6 | -0.121148646 | 0.869797312 | 0.916822455 | down |
| GSTO1 | -0.121130178 | 0.535429735 | 0.658120974 | down |
| CTD-3093B17.2 | -0.120932384 | 0.931756956 | | down |
| RBM14 | -0.12083395 | 0.460174339 | 0.592046842 | down |
| MED22 | -0.120725144 | 0.295646854 | 0.433153653 | down |
| MXD4 | -0.120701875 | 0.302109302 | 0.439969552 | down |
| RP11-359I18.5 | -0.120674949 | 0.842690579 | 0.898263324 | down |

| | | | | |
|---|---|---|---|---|
| KCNV2 | -0.120663058 | 0.921145852 | | down |
| LILRA4 | -0.120618249 | 0.765040048 | 0.841782208 | down |
| RP11-453N18.1 | -0.120595747 | 0.900515321 | 0.936615741 | down |
| RXRG | -0.12045611 | 0.699220227 | 0.792989384 | down |
| LAMA2 | -0.120311484 | 0.694395239 | 0.789315976 | down |
| POP4 | -0.120310746 | 0.140822367 | 0.257397201 | down |
| METAP1D | -0.120297116 | 0.513308456 | 0.638630215 | down |
| ERMP1 | -0.120285828 | 0.526093486 | 0.649675396 | down |
| CLDN10-AS1 | -0.120138813 | 0.93064593 | | down |
| C15orf53 | -0.120090979 | 0.781502389 | 0.853798465 | down |
| PLEKHA1 | -0.120082174 | 0.572411332 | 0.689694247 | down |
| PDCD11 | -0.119819961 | 0.536172926 | 0.658684143 | down |
| FBXL12 | -0.119788984 | 0.554445423 | 0.674375867 | down |
| ZNF518A | -0.119704387 | 0.496144231 | 0.623792989 | down |
| LEMD2 | -0.119684568 | 0.420872778 | 0.556642971 | down |
| KCNK1 | -0.119645549 | 0.688845759 | 0.784826368 | down |
| RP1-223B1.1 | -0.119604058 | 0.847645109 | 0.901761611 | down |
| TAS1R1 | -0.119528656 | 0.813983323 | 0.877584016 | down |
| TMEM178B | -0.119506323 | 0.738952953 | 0.822597474 | down |
| NDOR1 | -0.119395191 | 0.466292903 | 0.597590068 | down |
| PPP2R1B | -0.119321686 | 0.230622094 | 0.363012009 | down |
| STK32A | -0.119279227 | 0.759596621 | 0.837664105 | down |
| LTBP3 | -0.119256817 | 0.547022903 | 0.66792634 | down |
| NFE2 | -0.119195357 | 0.770336762 | 0.84568108 | down |
| RP11-195F19.9 | -0.119168016 | 0.650573702 | 0.754548962 | down |
| MADD | -0.119086725 | 0.319950942 | 0.458791815 | down |
| LRRC61 | -0.118938644 | 0.643926455 | 0.749298052 | down |
| RP11-834C11.7 | -0.118802871 | 0.752955239 | 0.832709427 | down |
| WDR90 | -0.118794876 | 0.398415526 | 0.535354715 | down |
| HID1 | -0.118783062 | 0.54865868 | 0.669261119 | down |
| SPEF1 | -0.118586144 | 0.812615607 | 0.876538373 | down |
| PHKA2-AS1 | -0.118579295 | 0.77629053 | 0.850113821 | down |
| PFN2 | -0.118491656 | 0.615620635 | 0.726604061 | down |
| AC145110.1 | -0.118472789 | 0.853674011 | 0.906121956 | down |
| SNRNP70 | -0.118455162 | 0.517932448 | 0.642741118 | down |
| HLA-F | -0.11832973 | 0.548118302 | 0.668873528 | down |
| NQO2 | -0.118231669 | 0.412166532 | 0.548897663 | down |
| PPP1R3E | -0.118201205 | 0.384288418 | 0.521974828 | down |
| LLPH | -0.118100452 | 0.364113201 | 0.502522716 | down |
| PIN4 | -0.118043189 | 0.351085708 | 0.489769072 | down |
| MOB3A | -0.118001479 | 0.521195765 | 0.64548438 | down |
| SLC28A3 | -0.117954382 | 0.786472572 | 0.85714013 | down |
| CSK | -0.117950318 | 0.593437201 | 0.708050243 | down |
| TMEM203 | -0.117905259 | 0.349814341 | 0.488545424 | down |
| PDGFC | -0.117852587 | 0.588007228 | 0.70335188 | down |
| RP11-599B13.7 | -0.11764327 | 0.892969274 | 0.931508242 | down |
| CRP | -0.117626778 | 0.900995964 | | down |
| HEIH | -0.117524119 | 0.677388793 | 0.776240327 | down |
| CXorf57 | -0.117517837 | 0.723897885 | 0.811733963 | down |
| PPID | -0.117434065 | 0.580585372 | 0.696733964 | down |
| TMEM231 | -0.117412025 | 0.625114515 | 0.734414311 | down |

| | | | | |
|---|---|---|---|---|
| C2-AS1 | -0.117314625 | 0.889913315 | | down |
| PIGT | -0.11730228 | 0.450695528 | 0.583650708 | down |
| GFOD1-AS1 | -0.117253597 | 0.902262033 | | down |
| THG1L | -0.117140263 | 0.277563875 | 0.413749892 | down |
| RP11-77P16.4 | -0.117083652 | 0.754520379 | 0.833994712 | down |
| TPO | -0.117037504 | 0.700718018 | 0.79400966 | down |
| RP11-49I11.4 | -0.116907224 | 0.787457044 | 0.857964077 | down |
| TBC1D22B | -0.116798388 | 0.309850374 | 0.448395773 | down |
| RP11-815J4.7 | -0.116681617 | 0.814999343 | 0.878352773 | down |
| FRAS1 | -0.116617806 | 0.750317597 | 0.831214671 | down |
| CDK19 | -0.116434157 | 0.395251193 | 0.532627353 | down |
| ZNF518B | -0.116308607 | 0.536992514 | 0.65917888 | down |
| ZNF619 | -0.116296807 | 0.49887626 | 0.62607512 | down |
| RP11-326C3.16 | -0.11621451 | 0.843277064 | 0.898560932 | down |
| LINC00910 | -0.116143613 | 0.595179168 | 0.709276225 | down |
| DUPD1 | -0.116122222 | 0.895347472 | 0.933243806 | down |
| RP11-1376P16.2 | -0.116120407 | 0.857749672 | 0.908810521 | down |
| ANKRD46 | -0.116060755 | 0.574395987 | 0.691447754 | down |
| FAT1 | -0.115948213 | 0.734850124 | 0.819944927 | down |
| MIR4786 | -0.115937844 | 0.900280525 | | down |
| ELOVL7 | -0.115915294 | 0.764959869 | 0.841743636 | down |
| AC005154.6 | -0.115800036 | 0.443226871 | 0.576738192 | down |
| E2F5 | -0.115778781 | 0.696761288 | 0.791023529 | down |
| AP000254.8 | -0.115735039 | 0.671266151 | 0.770870738 | down |
| RAB20 | -0.115640336 | 0.484781994 | 0.613616036 | down |
| TRIOBP | -0.115566118 | 0.323223887 | 0.461765975 | down |
| AC017104.6 | -0.115539774 | 0.773809648 | 0.848180743 | down |
| RNU6-695P | -0.11550972 | 0.968508444 | | down |
| AMY1C | -0.11550972 | 0.968508444 | | down |
| GAPDHP27 | -0.11550972 | 0.968508444 | | down |
| UQCRBP2 | -0.11550972 | 0.968508444 | | down |
| VN1R17P | -0.11550972 | 0.968508444 | | down |
| RP11-254F7.3 | -0.11550972 | 0.968508444 | | down |
| AC097523.3 | -0.11550972 | 0.968508444 | | down |
| PGAM1P4 | -0.11550972 | 0.968508444 | | down |
| RNU6ATAC26P | -0.11550972 | 0.968508444 | | down |
| PSMD12P | -0.11550972 | 0.968508444 | | down |
| RP11-503I22.2 | -0.11550972 | 0.968508444 | | down |
| RP11-206P5.1 | -0.11550972 | 0.968508444 | | down |
| SULT1D1P | -0.11550972 | 0.968508444 | | down |
| RPL3P13 | -0.11550972 | 0.968508444 | | down |
| NA | -0.11550972 | 0.968508444 | | down |
| NA | -0.11550972 | 0.968508444 | | down |
| RP11-746P2.3 | -0.11550972 | 0.968508444 | | down |
| SNORA48 | -0.11550972 | 0.968508444 | | down |
| OR2R1P | -0.11550972 | 0.968508444 | | down |
| MIR1205 | -0.11550972 | 0.968508444 | | down |
| MAPKAPK5P1 | -0.11550972 | 0.968508444 | | down |
| OR10Y1P | -0.11550972 | 0.968508444 | | down |
| RP11-707G14.8 | -0.11550972 | 0.968508444 | | down |
| NA | -0.11550972 | 0.968508444 | | down |

| | | | | |
|---|---|---|---|---|
| U6 | -0.11550972 | 0.968508444 | | down |
| RP11-248E9.6 | -0.11550972 | 0.968508444 | | down |
| RP11-203I16.7 | -0.11550972 | 0.968508444 | | down |
| RPL7L1P12 | -0.11550972 | 0.968508444 | | down |
| LINC00911 | -0.11550972 | 0.968508444 | | down |
| SERPINA13P | -0.11550972 | 0.968508444 | | down |
| KRT8P24 | -0.11550972 | 0.968508444 | | down |
| NA | -0.11550972 | 0.968508444 | | down |
| NA | -0.11550972 | 0.968508444 | | down |
| RP11-345J4.1 | -0.11550972 | 0.968508444 | | down |
| RP11-480G7.3 | -0.11550972 | 0.968508444 | | down |
| RP11-686D22.6 | -0.11550972 | 0.968508444 | | down |
| KRTAP4-11 | -0.11550972 | 0.968508444 | | down |
| RP11-286N3.1 | -0.11550972 | 0.968508444 | | down |
| RP11-513M1.1 | -0.11550972 | 0.968508444 | | down |
| RP11-57J16.1 | -0.11550972 | 0.968508444 | | down |
| RP11-776B3.1 | -0.11550972 | 0.968508444 | | down |
| CTC-43909.3 | -0.11550972 | 0.968508444 | | down |
| MIR643 | -0.11550972 | 0.968508444 | | down |
| RP11-476I15.6 | -0.11550972 | 0.968508444 | | down |
| MIR185 | -0.11550972 | 0.968508444 | | down |
| H2AFZP6 | -0.11550972 | 0.968508444 | | down |
| RP1-180M12.1 | -0.11550972 | 0.968508444 | | down |
| AL590762.10 | -0.11550972 | 0.968508444 | | down |
| AL590763.5 | -0.11550972 | 0.968508444 | | down |
| RP11-635016.2 | -0.11550972 | 0.968508444 | | down |
| RP13-228J13.6 | -0.11550972 | 0.968508444 | | down |
| RNU6-689P | -0.115509133 | 0.96723211 | | down |
| TMED11P | -0.115509027 | 0.966983999 | | down |
| RP11-739B23.1 | -0.115508739 | 0.966281318 | | down |
| HFE | -0.115444027 | 0.639406184 | 0.745626432 | down |
| SDF4 | -0.115443657 | 0.455175085 | 0.587653313 | down |
| COL28A1 | -0.115401585 | 0.760749991 | 0.838474366 | down |
| LANCL2 | -0.115366109 | 0.257586216 | 0.392640671 | down |
| KLK8 | -0.115302401 | 0.937332419 | 0.961888691 | down |
| TRGV9 | -0.115250792 | 0.841184195 | 0.897151514 | down |
| DIS3L2 | -0.115246173 | 0.27571125 | 0.412030879 | down |
| RP11-429J17.2 | -0.115135022 | 0.750761368 | 0.831405164 | down |
| USP9X | -0.115098835 | 0.477931847 | 0.607581958 | down |
| RFC2 | -0.115089766 | 0.384795553 | 0.522522855 | down |
| C15orf61 | -0.114870309 | 0.593996915 | 0.708457456 | down |
| CST5 | -0.114754791 | 0.934402381 | | down |
| HUS1 | -0.114657603 | 0.397881903 | 0.534924773 | down |
| RP4-584D14.6 | -0.114651868 | 0.835542523 | 0.892838226 | down |
| OBFC1 | -0.114635996 | 0.357455767 | 0.49586301 | down |
| CTD-233602.1 | -0.114630044 | 0.468242475 | 0.599245055 | down |
| CD8B | -0.114547109 | 0.707846061 | 0.799283161 | down |
| CARS2 | -0.114345746 | 0.294184201 | 0.431565924 | down |
| RP11-317J9.1 | -0.114288193 | 0.887042283 | | down |
| RP11-531F16.4 | -0.114189595 | 0.84983211 | 0.903256657 | down |
| CYFIP2 | -0.114111693 | 0.57707785 | 0.693786591 | down |

| | | | | |
|---|---|---|---|---|
| GLIS2-AS1 | -0.114053537 | 0.848616989 | 0.902380152 | down |
| ZRSR2 | -0.114001561 | 0.551309152 | 0.671453056 | down |
| DHCR7 | -0.113990222 | 0.597120021 | 0.711047331 | down |
| BCL11B | -0.113769045 | 0.644692072 | 0.749769 | down |
| RP11-23J18.1 | -0.11375989 | 0.774687477 | 0.848915292 | down |
| HPCAL1 | -0.11369119 | 0.335435899 | 0.4736493 | down |
| DDHD2 | -0.113610484 | 0.617854981 | 0.728439942 | down |
| FBRSL1 | -0.11358439 | 0.406710993 | 0.543269833 | down |
| RP11-27M5.1 | -0.113522071 | 0.950925951 | | down |
| ZNF580 | -0.113480655 | 0.545103892 | 0.666455312 | down |
| RP11-378A13.1 | -0.113446504 | 0.59559228 | 0.70971223 | down |
| RP11-1151B14.5 | -0.113428113 | 0.894016545 | | down |
| CTD-3199J23.6 | -0.11322029 | 0.802590495 | 0.869157223 | down |
| ANAPC15 | -0.113134285 | 0.511947374 | 0.637499353 | down |
| CCDC130 | -0.113006481 | 0.511726454 | 0.637329917 | down |
| MT2A | -0.112996334 | 0.665338587 | 0.766102886 | down |
| SLC30A6 | -0.112913508 | 0.495971681 | 0.623628241 | down |
| TRPM2-AS | -0.112817666 | 0.766677572 | 0.843120826 | down |
| RP11-791M20.1 | -0.112808097 | 0.917175639 | | down |
| RAD51-AS1 | -0.11268171 | 0.579750532 | 0.696057493 | down |
| POP5 | -0.112549721 | 0.472226493 | 0.602623827 | down |
| RPS2P4 | -0.112520108 | 0.848941831 | 0.902629733 | down |
| SGCA | -0.112412096 | 0.70676272 | 0.798359934 | down |
| RP11-907D1.1 | -0.112371456 | 0.969363606 | | down |
| RN7SL740P | -0.112371456 | 0.969363606 | | down |
| Y_RNA | -0.112371456 | 0.969363606 | | down |
| PRAMEF33P | -0.112370926 | 0.967835829 | | down |
| MEG8 | -0.112369629 | 0.962851345 | | down |
| NA | -0.112368895 | 0.958749677 | | down |
| RP3-347M6.3 | -0.112367817 | 0.949140898 | | down |
| RP11-295M3.2 | -0.112367002 | 0.934690893 | | down |
| RP11-510I6.2 | -0.112366939 | 0.932980523 | | down |
| NBEA | -0.112361292 | 0.752647798 | 0.832476179 | down |
| TLR7 | -0.112284531 | 0.627293688 | 0.736044933 | down |
| MTMR10 | -0.112272843 | 0.333900534 | 0.472147016 | down |
| EIF4A2P2 | -0.112238684 | 0.900044226 | | down |
| SLC25A37 | -0.11223146 | 0.519389436 | 0.643937449 | down |
| HERC2 | -0.112216043 | 0.494371835 | 0.622371984 | down |
| CTDP1 | -0.112171261 | 0.239121789 | 0.37260386 | down |
| PRPH | -0.112151204 | 0.783961471 | 0.855487057 | down |
| PDCD4-AS1 | -0.111935737 | 0.511717551 | 0.637329917 | down |
| NA | -0.111922498 | 0.73172923 | 0.817829433 | down |
| LILRB5 | -0.111728062 | 0.727386403 | 0.814498965 | down |
| NCOA6 | -0.111603692 | 0.563546845 | 0.682269561 | down |
| MIR647 | -0.111560202 | 0.817498626 | 0.880288474 | down |
| KIAA0930 | -0.111544246 | 0.470068564 | 0.600737587 | down |
| SF3A2 | -0.111457476 | 0.500284819 | 0.6273973 | down |
| TNRC6B | -0.111403589 | 0.452380409 | 0.585076066 | down |
| XXbac-B476C20.1 | -0.11130099 | 0.934064099 | | down |
| YIPF3 | -0.111296142 | 0.55971272 | 0.679160297 | down |
| EPB41L1 | -0.111035371 | 0.585321432 | 0.700976486 | down |

| | | | | |
|---|---|---|---|---|
| FAT3 | -0.110973488 | 0.810832357 | 0.875117553 | down |
| STAG3 | -0.110964392 | 0.752447725 | 0.832425048 | down |
| GMCL1 | -0.11096387 | 0.526364831 | 0.649885294 | down |
| GATB | -0.110897646 | 0.387339266 | 0.525003199 | down |
| ELOVL4 | -0.110873697 | 0.745405537 | 0.827124587 | down |
| NPM1P28 | -0.110850676 | 0.946976432 | 0.967983122 | down |
| CTB-113P19.4 | -0.110830297 | 0.867875891 | | down |
| PIP4K2C | -0.110821279 | 0.55273796 | 0.672653081 | down |
| CHD9 | -0.110628032 | 0.433658886 | 0.568672148 | down |
| TSC1 | -0.110618433 | 0.379878658 | 0.51778104 | down |
| MLF2 | -0.110590101 | 0.354391532 | 0.493021787 | down |
| GHRLOS | -0.110587947 | 0.784886101 | 0.856094319 | down |
| RP3-395C13.1 | -0.110545648 | 0.796253764 | 0.864382428 | down |
| SPATS2L | -0.110451972 | 0.570412826 | 0.688003651 | down |
| LA16c-306A4.1 | -0.110436734 | 0.779720649 | 0.852377191 | down |
| CTD-2035E11.5 | -0.110395638 | 0.7314632 | 0.817562515 | down |
| CXorf65 | -0.110346356 | 0.910614652 | | down |
| RP11-122C5.3 | -0.110343172 | 0.907830302 | | down |
| TUBB2A | -0.110223179 | 0.525869102 | 0.649556422 | down |
| KMT2A | -0.11021469 | 0.476201989 | 0.606106721 | down |
| CAB39 | -0.110163655 | 0.544977359 | 0.666393967 | down |
| MGAT5 | -0.11016183 | 0.546827349 | 0.667786394 | down |
| RP13-228J13.8 | -0.110097531 | 0.909191315 | | down |
| GDPD5 | -0.110065004 | 0.665641511 | 0.766303457 | down |
| AC139100.4 | -0.110020257 | 0.808702215 | 0.87363451 | down |
| RP11-56B16.2 | -0.109783358 | 0.793359707 | 0.862237702 | down |
| TNFRSF1A | -0.109730316 | 0.553808858 | 0.673819841 | down |
| RP11-631N16.2 | -0.109547553 | 0.634703117 | 0.741927494 | down |
| PAXIP1-AS1 | -0.109434729 | 0.52559544 | 0.649380656 | down |
| TBC1D31 | -0.109423076 | 0.618776992 | 0.729240809 | down |
| RP11-503P10.1 | -0.109275333 | 0.857018601 | | down |
| RNU6-722P | -0.109268252 | 0.774129957 | 0.848459085 | down |
| CTD-3220F14.3 | -0.109084906 | 0.799571102 | 0.867090887 | down |
| GDI1 | -0.109068185 | 0.388413774 | 0.52603243 | down |
| NA | -0.109014809 | 0.826308041 | 0.886343632 | down |
| REPIN1 | -0.108858068 | 0.363328817 | 0.501739795 | down |
| RP13-554M15.7 | -0.108819063 | 0.699318128 | 0.792989384 | down |
| ZNF263 | -0.10878269 | 0.463470848 | 0.59509031 | down |
| RPL4P5 | -0.10873538 | 0.634167639 | 0.741503505 | down |
| RP11-973H7.1 | -0.108689077 | 0.808891589 | 0.873744837 | down |
| TCAIM | -0.108626286 | 0.495113083 | 0.62307018 | down |
| FAM114A1 | -0.108547817 | 0.449995753 | 0.582895249 | down |
| TMEM138 | -0.108539582 | 0.344877146 | 0.483413687 | down |
| PLCH2 | -0.108377358 | 0.685270556 | 0.782169614 | down |
| AC002117.1 | -0.108312443 | 0.864257849 | | down |
| SAP30BP | -0.108251597 | 0.370734249 | 0.509130296 | down |
| RBM38 | -0.107973347 | 0.672825061 | 0.772190709 | down |
| CTIF | -0.107955399 | 0.517808378 | 0.642640239 | down |
| BOK | -0.107916831 | 0.540866563 | 0.662634874 | down |
| RIN3 | -0.107903069 | 0.355555833 | 0.494162901 | down |
| AC018816.1 | -0.107896601 | 0.948446628 | | down |

| | | | | |
|---|---|---|---|---|
| CNOT6 | -0.107801775 | 0.573274153 | 0.690456952 | down |
| AK4P1 | -0.107772286 | 0.859904705 | 0.910355437 | down |
| KIAA0391 | -0.107726462 | 0.526371613 | 0.649885294 | down |
| RP11-67L2.2 | -0.107715534 | 0.62527325 | 0.734476659 | down |
| RP11-335G20.7 | -0.107706121 | 0.916766672 | | down |
| AC007292.3 | -0.107659257 | 0.792989425 | 0.862022371 | down |
| SLC6A16 | -0.107591127 | 0.73560951 | 0.820274362 | down |
| KMT5B | -0.107485054 | 0.432215994 | 0.567275231 | down |
| GINS1 | -0.107423291 | 0.643897844 | 0.74929378 | down |
| RRM1 | -0.107418737 | 0.327752871 | 0.466174656 | down |
| TMEM101 | -0.107322197 | 0.487999671 | 0.616753413 | down |
| CACNA1C-AS2 | -0.107278066 | 0.789291082 | 0.859370199 | down |
| GLTSCR1 | -0.107239079 | 0.234911949 | 0.367687216 | down |
| APOL6 | -0.10721795 | 0.591986839 | 0.70698623 | down |
| NA | -0.107146202 | 0.826274402 | 0.886339193 | down |
| WNT10A | -0.107100405 | 0.730629445 | 0.816997632 | down |
| BST2 | -0.107081581 | 0.659208534 | 0.761140234 | down |
| IRF2BP1 | -0.106750663 | 0.173415227 | 0.297872453 | down |
| BCHE | -0.106636279 | 0.826042073 | 0.886153255 | down |
| SRP19 | -0.106563182 | 0.518855292 | 0.643540783 | down |
| HDAC4 | -0.106526042 | 0.5638236 | 0.682347892 | down |
| LMOD1 | -0.1064795 | 0.74153541 | 0.824430914 | down |
| KLHL24 | -0.106335363 | 0.528351187 | 0.651740149 | down |
| DDA1 | -0.106195766 | 0.323318281 | 0.46181299 | down |
| FOXD3 | -0.106153902 | 0.824378981 | 0.884874674 | down |
| OOSP1P1 | -0.10610067 | 0.97107247 | | down |
| RPL26P9 | -0.10610067 | 0.97107247 | | down |
| FAM91A3P | -0.10610067 | 0.97107247 | | down |
| GS1-304P7.2 | -0.10610067 | 0.97107247 | | down |
| RP11-177F11.1 | -0.10610067 | 0.97107247 | | down |
| AC008069.2 | -0.10610067 | 0.97107247 | | down |
| IGKV2-14 | -0.10610067 | 0.97107247 | | down |
| RP11-615J4.3 | -0.10610067 | 0.97107247 | | down |
| HNRNPA1P23 | -0.10610067 | 0.97107247 | | down |
| RP11-764I5.1 | -0.10610067 | 0.97107247 | | down |
| RP11-438D8.2 | -0.10610067 | 0.97107247 | | down |
| MTND5P15 | -0.10610067 | 0.97107247 | | down |
| ALG1L14P | -0.10610067 | 0.97107247 | | down |
| RP11-640B6.1 | -0.10610067 | 0.97107247 | | down |
| RP11-533F5.2 | -0.10610067 | 0.97107247 | | down |
| NA | -0.10610067 | 0.97107247 | | down |
| RP11-321E2.4 | -0.10610067 | 0.97107247 | | down |
| CTD-2113L7.1 | -0.10610067 | 0.97107247 | | down |
| CTD-2061E19.1 | -0.10610067 | 0.97107247 | | down |
| RNU6-402P | -0.10610067 | 0.97107247 | | down |
| RNA5SP190 | -0.10610067 | 0.97107247 | | down |
| CTB-95B16.1 | -0.10610067 | 0.97107247 | | down |
| CTC-535M15.2 | -0.10610067 | 0.97107247 | | down |
| NA | -0.10610067 | 0.97107247 | | down |
| DOCK4-AS1 | -0.10610067 | 0.97107247 | | down |
| AC009362.2 | -0.10610067 | 0.97107247 | | down |

| | | | |
|---|---|---|---|
| RNA5SP256 | -0.10610067 | 0.97107247 | down |
| RP11-386G21.2 | -0.10610067 | 0.97107247 | down |
| RNU1-101P | -0.10610067 | 0.97107247 | down |
| NA | -0.10610067 | 0.97107247 | down |
| RP11-275N1.3 | -0.10610067 | 0.97107247 | down |
| RP11-285G1.2 | -0.10610067 | 0.97107247 | down |
| RP11-93L14.1 | -0.10610067 | 0.97107247 | down |
| NA | -0.10610067 | 0.97107247 | down |
| OR7E87P | -0.10610067 | 0.97107247 | down |
| TDGP1 | -0.10610067 | 0.97107247 | down |
| RP11-582E3.2 | -0.10610067 | 0.97107247 | down |
| MREGP1 | -0.10610067 | 0.97107247 | down |
| RP11-73B8.3 | -0.10610067 | 0.97107247 | down |
| RP11-769N19.2 | -0.10610067 | 0.97107247 | down |
| RP11-117N2.2 | -0.10610067 | 0.97107247 | down |
| MIR6762 | -0.10610067 | 0.97107247 | down |
| RP11-116D17.1 | -0.10610067 | 0.97107247 | down |
| RPS21P8 | -0.10610067 | 0.97107247 | down |
| RNU6-273P | -0.10610067 | 0.97107247 | down |
| RP1-292L20.2 | -0.10610067 | 0.97107247 | down |
| NT5CP2 | -0.10610067 | 0.97107247 | down |
| RP11-439M15.1 | -0.10610067 | 0.97107247 | down |
| RP11-456J20.1 | -0.10610067 | 0.97107247 | down |
| RP11-139F4.2 | -0.10610067 | 0.97107247 | down |
| UBE2Q2P11 | -0.10610067 | 0.97107247 | down |
| NA | -0.10610067 | 0.97107247 | down |
| RP11-883G14.2 | -0.10610067 | 0.97107247 | down |
| ZNF415P1 | -0.10610067 | 0.97107247 | down |
| RP11-17E3.1 | -0.10610067 | 0.97107247 | down |
| CTC-503J8.8 | -0.10610067 | 0.97107247 | down |
| CTD-2528A14.1 | -0.10610067 | 0.97107247 | down |
| ZNF92P2 | -0.10610067 | 0.97107247 | down |
| TGIF1P1 | -0.10610067 | 0.97107247 | down |
| LINC01370 | -0.10610067 | 0.97107247 | down |
| CH507-145C22.1 | -0.10610067 | 0.97107247 | down |
| MTND6P21 | -0.10610067 | 0.97107247 | down |
| RNU4-6P | -0.10610067 | 0.97107247 | down |
| EIF1AX-AS1 | -0.10610067 | 0.97107247 | down |
| RPL7P55 | -0.10610067 | 0.97107247 | down |
| AC002368.4 | -0.10610067 | 0.97107247 | down |
| TRIM60P3Y | -0.10610067 | 0.97107247 | down |
| RNU6-1278P | -0.106099608 | 0.968781607 | down |
| C6orf229 | -0.106099477 | 0.96845878 | down |
| RNY3P13 | -0.106099358 | 0.968159012 | down |
| RNU6-553P | -0.106099299 | 0.968006301 | down |
| Y_RNA | -0.106098044 | 0.964132192 | down |
| RP11-74E24.2 | -0.106098043 | 0.964127015 | down |
| TAS2R43 | -0.106098016 | 0.964027434 | down |
| MTCO1P11 | -0.106097958 | 0.963812803 | down |
| TRAJ21 | -0.106097929 | 0.963703621 | down |
| AP004289.1 | -0.106097867 | 0.963462966 | down |

| | | | | |
|---|---|---|---|---|
| HMGN2P21 | -0.106097811 | 0.963246589 | | down |
| RP11-57C13.5 | -0.106097766 | 0.963067142 | | down |
| RPS27AP6 | -0.106096877 | 0.958888869 | | down |
| AC004980.9 | -0.106096783 | 0.958355899 | | down |
| TAF8 | -0.106001478 | 0.495938545 | 0.623612675 | down |
| PPP1R14B | -0.105965926 | 0.595166619 | 0.709276225 | down |
| RP1-292B18.1 | -0.105943362 | 0.722465245 | 0.810732671 | down |
| AGAP7P | -0.105938996 | 0.782995567 | 0.854839159 | down |
| CBR4 | -0.105912262 | 0.486560128 | 0.615310308 | down |
| CCNB2 | -0.10589794 | 0.619218927 | 0.729569865 | down |
| AC137932.4 | -0.105891422 | 0.818006166 | 0.880625665 | down |
| TRIM41 | -0.105825859 | 0.258168066 | 0.393226939 | down |
| ABHD14A | -0.105800899 | 0.728539857 | 0.815417482 | down |
| SMCO4 | -0.105793524 | 0.535052912 | 0.657738531 | down |
| GPRC5D | -0.105764101 | 0.779936468 | 0.852459664 | down |
| TMEM99 | -0.105730534 | 0.539648119 | 0.661546763 | down |
| IKZF4 | -0.10565706 | 0.724432624 | 0.812121409 | down |
| TGOLN2 | -0.105642113 | 0.524614025 | 0.64872834 | down |
| SPATA24 | -0.105635593 | 0.546750056 | 0.667775299 | down |
| PHYHIP | -0.1055931 | 0.750793952 | 0.831405164 | down |
| WBP1L | -0.105485443 | 0.350674632 | 0.489416484 | down |
| FBXL17 | -0.105411575 | 0.453010469 | 0.585588441 | down |
| DIO2 | -0.105409865 | 0.771267069 | 0.84620626 | down |
| COLQ | -0.105393971 | 0.77728157 | 0.850764964 | down |
| NHLRC3 | -0.105392168 | 0.530328145 | 0.653561622 | down |
| RP1-221C16.8 | -0.105383027 | 0.837621376 | 0.894391606 | down |
| DGKD | -0.105362935 | 0.530521497 | 0.653746271 | down |
| COMT | -0.105278573 | 0.434470842 | 0.569289632 | down |
| TAS2R5 | -0.105173783 | 0.878627263 | 0.922307357 | down |
| TRBV21-1 | -0.105126572 | 0.848596099 | 0.902380152 | down |
| SESTD1 | -0.105103613 | 0.708189858 | 0.799458058 | down |
| CGRRF1 | -0.105017633 | 0.51435047 | 0.639654993 | down |
| CTD-2262B20.1 | -0.105006035 | 0.805509794 | 0.871591696 | down |
| FAF1 | -0.104840615 | 0.198030456 | 0.326741008 | down |
| RNU6-8 | -0.104810357 | 0.811199917 | 0.875388466 | down |
| RPS4Y1 | -0.104660972 | 0.910166561 | 0.943746131 | down |
| C14orf1 | -0.104650595 | 0.502514996 | 0.62940595 | down |
| RP11-385H1.1 | -0.104626344 | 0.954858778 | | down |
| RP11-318C24.1 | -0.104604168 | 0.789527071 | 0.85956484 | down |
| CTD-2020K17.1 | -0.104595855 | 0.72179225 | 0.810310379 | down |
| TREX2 | -0.104569429 | 0.743874674 | 0.82627073 | down |
| CA11 | -0.104545303 | 0.580561194 | 0.696733964 | down |
| ARFGEF3 | -0.104353151 | 0.696122448 | 0.79065521 | down |
| RP11-102M11.1 | -0.103912947 | 0.87873514 | 0.922307357 | down |
| GK-AS1 | -0.103863282 | 0.876967629 | 0.921567522 | down |
| CAMKK1 | -0.103763852 | 0.413994264 | 0.550575169 | down |
| ELP3 | -0.103730299 | 0.172364765 | 0.296687289 | down |
| RP11-50I19.2 | -0.103693593 | 0.739172141 | 0.822756341 | down |
| RPL10 | -0.103685772 | 0.507655612 | 0.63390458 | down |
| ZNF519 | -0.103681266 | 0.740616071 | 0.823808561 | down |
| ERLIN2 | -0.103654727 | 0.602850598 | 0.71574959 | down |

| | | | | |
|---|---|---|---|---|
| SIRT5 | -0.10364437 | 0.448108052 | 0.581228923 | down |
| RP11-574K11.27 | -0.103612715 | 0.915758194 | | down |
| PITPNM3 | -0.103504212 | 0.752932408 | 0.832709427 | down |
| HN1L | -0.103495347 | 0.449092887 | 0.582026861 | down |
| CRYZL1 | -0.103446972 | 0.278488746 | 0.414758137 | down |
| RP11-141M3.6 | -0.103428827 | 0.840687875 | 0.896805373 | down |
| CCDC74B | -0.103426041 | 0.787655621 | 0.858074166 | down |
| RNF39 | -0.103291932 | 0.752573864 | 0.832425048 | down |
| RP11-661013.1 | -0.10326973 | 0.916586319 | | down |
| MIR1254-1 | -0.103257523 | 0.892496406 | 0.931176622 | down |
| THAP6 | -0.103250459 | 0.590053188 | 0.70518151 | down |
| COX6B1P2 | -0.102962406 | 0.971927736 | | down |
| CTC-260E6.9 | -0.102958491 | 0.948185167 | | down |
| RP11-405A12.1 | -0.10295787 | 0.934075859 | | down |
| DYNLL1P2 | -0.102957552 | 0.920256041 | | down |
| RSAD2 | -0.102952845 | 0.72039536 | 0.809347027 | down |
| CEACAM3 | -0.10292817 | 0.814561902 | 0.878007311 | down |
| PDIK1L | -0.102841435 | 0.520753695 | 0.645149644 | down |
| RP11-331F9.4 | -0.102777781 | 0.874500468 | 0.920093678 | down |
| KRT23 | -0.102751602 | 0.853335429 | 0.905794574 | down |
| OGFR-AS1 | -0.102739364 | 0.850635763 | 0.903886994 | down |
| MGA | -0.10273047 | 0.568012175 | 0.685961805 | down |
| KIF24 | -0.102642235 | 0.75919231 | 0.837399389 | down |
| RP4-655J12.4 | -0.10260461 | 0.822313171 | 0.883509527 | down |
| H2AFY2 | -0.102567223 | 0.34078651 | 0.479132852 | down |
| ATOH8 | -0.102494881 | 0.65196748 | 0.755611271 | down |
| LINC01473 | -0.102289617 | 0.782987141 | 0.854839159 | down |
| RPS18 | -0.102182886 | 0.515490712 | 0.640663645 | down |
| CTD-2008E3.1 | -0.102060816 | 0.831912293 | 0.890162153 | down |
| IPO13 | -0.101869166 | 0.525006644 | 0.648946746 | down |
| SORD | -0.101767468 | 0.516933382 | 0.64187248 | down |
| NA | -0.101763193 | 0.853296495 | 0.905794574 | down |
| ATP5H | -0.101755136 | 0.460383735 | 0.59229088 | down |
| ADNP | -0.1016852 | 0.34742018 | 0.486156115 | down |
| RPL21P134 | -0.10154513 | 0.856581766 | 0.908245724 | down |
| EMC1 | -0.101543317 | 0.54656122 | 0.667703757 | down |
| GTF2F1 | -0.101513365 | 0.256510589 | 0.391555946 | down |
| TTC28-AS1 | -0.101476141 | 0.437336061 | 0.571921504 | down |
| H6PD | -0.101423069 | 0.540849942 | 0.662634874 | down |
| STXBP6 | -0.101299858 | 0.704364812 | 0.796519739 | down |
| TSC22D4 | -0.101226389 | 0.553858833 | 0.673853356 | down |
| PAQR3 | -0.101171526 | 0.7603315 | 0.838228371 | down |
| STX4 | -0.101158609 | 0.477574692 | 0.607333505 | down |
| CH507-513H4.1 | -0.101079582 | 0.886068733 | 0.927214542 | down |
| CCDC71 | -0.100960267 | 0.455435367 | 0.587837786 | down |
| RPL41 | -0.100913962 | 0.484979755 | 0.613814633 | down |
| TCEB1P2 | -0.100902586 | 0.905019116 | | down |
| MZF1 | -0.100885037 | 0.520864458 | 0.645179946 | down |
| PPP1R37 | -0.100814339 | 0.288181899 | 0.425471431 | down |
| RGP1 | -0.100786832 | 0.565366499 | 0.68345355 | down |
| RPP14 | -0.100773409 | 0.445030382 | 0.578445289 | down |

| | | | | |
|---|---|---|---|---|
| DLST | -0.100770274 | 0.407015998 | 0.543556414 | down |
| TRGV10 | -0.100759662 | 0.855232883 | 0.907327827 | down |
| ORMDL2 | -0.100758121 | 0.659050008 | 0.761001516 | down |
| EIF1AD | -0.100735364 | 0.377586787 | 0.515693128 | down |
| SETD2 | -0.100664884 | 0.635178171 | 0.74228064 | down |
| DENND1C | -0.100495348 | 0.632710338 | 0.740462348 | down |
| SRP54-AS1 | -0.100484857 | 0.732123714 | 0.817996462 | down |
| HSD17B7 | -0.100469946 | 0.459675344 | 0.591614136 | down |
| RP11-109E24.2 | -0.100364668 | 0.887400321 | 0.927922012 | down |
| FAM210A | -0.100309813 | 0.509195395 | 0.63536284 | down |
| LINC01534 | -0.100228171 | 0.682326569 | 0.779905628 | down |
| RP11-9M16.2 | -0.100125401 | 0.930234625 | 0.957155008 | down |
| FLJ37453 | -0.100120456 | 0.600132946 | 0.713396938 | down |
| ZNF565 | -0.100076521 | 0.699689714 | 0.793203448 | down |
| CCNB1IP1 | -0.100074251 | 0.396594093 | 0.533527647 | down |
| MDH1 | -0.100052898 | 0.61282589 | 0.724101979 | down |
| GFRA1 | -0.100046145 | 0.723158913 | 0.811238383 | down |
| ZNF530 | -0.100044461 | 0.622153693 | 0.732042984 | down |
| CENPI | -0.099963757 | 0.756959784 | 0.835676481 | down |
| HIST2H2BA | -0.099949457 | 0.915351655 | | down |
| SSSCA1 | -0.099943663 | 0.540807512 | 0.662634874 | down |
| HLA-J | -0.099873557 | 0.76738584 | 0.843560067 | down |
| SNCG | -0.099827049 | 0.741270568 | 0.824231419 | down |
| CSE1L-AS1 | -0.099769522 | 0.901637713 | | down |
| NEK2 | -0.099639689 | 0.657045459 | 0.759337965 | down |
| RP11-500G22.5 | -0.099475889 | 0.805757322 | 0.871738314 | down |
| ZC3H3 | -0.099475106 | 0.551456459 | 0.671555991 | down |
| RHOD | -0.099446453 | 0.746796113 | 0.828137985 | down |
| RP11-206L10.2 | -0.099445947 | 0.721051218 | 0.809720948 | down |
| SOX9 | -0.099392208 | 0.655325953 | 0.758054692 | down |
| MAL | -0.099371089 | 0.726272939 | 0.813694184 | down |
| RP11-111J6.2 | -0.099355346 | 0.811023754 | 0.875229801 | down |
| PCDHB18P | -0.099273381 | 0.849001286 | 0.902661004 | down |
| LILRB3 | -0.099264668 | 0.750564751 | 0.831365852 | down |
| CAMKMT | -0.099247348 | 0.544789875 | 0.666246062 | down |
| SFXN3 | -0.099226894 | 0.659220811 | 0.761140234 | down |
| RP11-25I15.1 | -0.099224752 | 0.903340603 | | down |
| LIMD1-AS1 | -0.099198237 | 0.815280473 | 0.87856121 | down |
| RP11-385D13.3 | -0.099052172 | 0.849782678 | 0.903236071 | down |
| UQCC1 | -0.098712211 | 0.270495199 | 0.406480156 | down |
| RP11-79P5.10 | -0.098703619 | 0.866881945 | | down |
| MRPS31P5 | -0.098669335 | 0.875565951 | 0.920738657 | down |
| NA | -0.098619006 | 0.922349992 | | down |
| HOXD9 | -0.098607994 | 0.692641886 | 0.787820508 | down |
| TMEM181 | -0.098492757 | 0.653325188 | 0.756632537 | down |
| CYB5D2 | -0.098486022 | 0.476418362 | 0.606170892 | down |
| ZNF471 | -0.098387082 | 0.732765215 | 0.818348009 | down |
| FST | -0.098348676 | 0.756131071 | 0.835144965 | down |
| AMY2B | -0.09831207 | 0.727472217 | 0.814556324 | down |
| FAM84A | -0.09830106 | 0.696236898 | 0.790714001 | down |
| AP000688.14 | -0.098141066 | 0.853303143 | 0.905794574 | down |

| | | | | |
|---|---|---|---|---|
| C3orf62 | -0.098106341 | 0.592953589 | 0.707757159 | down |
| BCL7B | -0.098077221 | 0.477311922 | 0.607127832 | down |
| LINC00852 | -0.098053104 | 0.764643635 | 0.841561776 | down |
| GTF3C1 | -0.097915153 | 0.484063825 | 0.613078206 | down |
| ZNF878 | -0.097867002 | 0.874569239 | 0.920093678 | down |
| ATP6V1H | -0.097733913 | 0.180888469 | 0.306296183 | down |
| CILP | -0.097654193 | 0.753676798 | 0.833276872 | down |
| NOMO3 | -0.097509432 | 0.833753547 | 0.891529044 | down |
| SEC31B | -0.097499052 | 0.686399328 | 0.783045328 | down |
| ZNF791 | -0.09745184 | 0.453090855 | 0.585641957 | down |
| HIST1H2BH | -0.097434224 | 0.742524621 | 0.825289842 | down |
| PFN1P2 | -0.097365903 | 0.80788414 | 0.873096083 | down |
| ANAPC1 | -0.097168562 | 0.590546118 | 0.705607545 | down |
| CCDC60 | -0.097103548 | 0.819458464 | 0.88157733 | down |
| RNF123 | -0.096861517 | 0.749527679 | 0.830523331 | down |
| PPP1R12C | -0.096833136 | 0.465648055 | 0.597043794 | down |
| NA | -0.096792356 | 0.922719573 | 0.951987576 | down |
| HIST3H3 | -0.09669162 | 0.973636796 | | down |
| NA | -0.09669162 | 0.973636796 | | down |
| RP4-718N17.2 | -0.09669162 | 0.973636796 | | down |
| RP11-45K10.2 | -0.09669162 | 0.973636796 | | down |
| RP11-989F5.3 | -0.09669162 | 0.973636796 | | down |
| PSG8 | -0.09669162 | 0.973636796 | | down |
| RP11-481F24.3 | -0.096690327 | 0.927926854 | | down |
| ULK4P1 | -0.096690216 | 0.970684833 | | down |
| RP11-4M23.4 | -0.096690201 | 0.970647171 | | down |
| LINC00500 | -0.0966902 | 0.970646148 | | down |
| COX11P1 | -0.096690196 | 0.970634605 | | down |
| Metazoa_SRP | -0.09669017 | 0.97057024 | | down |
| RP11-230C9.3 | -0.096690022 | 0.970193861 | | down |
| RP11-152P17.3 | -0.096689816 | 0.969645529 | | down |
| NA | -0.096688807 | 0.9664334 | | down |
| RP11-357G3.2 | -0.096688726 | 0.966130516 | | down |
| RP11-153M7.5 | -0.096688722 | 0.966112967 | | down |
| RNU6ATAC35P | -0.096687791 | 0.961889061 | | down |
| NA | -0.096687786 | 0.961863151 | | down |
| RN7SL338P | -0.096687781 | 0.961834453 | | down |
| AL022345.10 | -0.096687767 | 0.961756519 | | down |
| TRAJ23 | -0.09668776 | 0.961717314 | | down |
| NA | -0.096687729 | 0.961546566 | | down |
| RP11-242D8.3 | -0.096687697 | 0.961368585 | | down |
| RP11-113D6.6 | -0.096687056 | 0.957146521 | | down |
| RP11-10A14.9 | -0.096687048 | 0.95708347 | | down |
| RP11-503N18.4 | -0.096686549 | 0.952603925 | | down |
| LIN37 | -0.09664904 | 0.745635809 | 0.827256719 | down |
| CTC-523E23.5 | -0.096644086 | 0.864074364 | 0.913096626 | down |
| ROBO3 | -0.096598548 | 0.652151125 | 0.755700631 | down |
| CISD2 | -0.096551651 | 0.549028479 | 0.669542603 | down |
| AFG3L1P | -0.096541297 | 0.506481704 | 0.632738838 | down |
| SMC4 | -0.096511789 | 0.802144933 | 0.86876865 | down |
| RP11-521D12.5 | -0.096386405 | 0.877086978 | 0.921628468 | down |

| | | | | |
|---|---|---|---|---|
| HIC2 | -0.09636228 | 0.640905629 | 0.746838137 | down |
| PITPNA | -0.096235937 | 0.545415381 | 0.666658246 | down |
| RPS20P22 | -0.096230021 | 0.890620374 | 0.930188365 | down |
| DTNB | -0.096181878 | 0.669407263 | 0.769353576 | down |
| PHB2 | -0.096173352 | 0.41300841 | 0.549580329 | down |
| FLNA | -0.096131499 | 0.735144111 | 0.820090215 | down |
| RP11-817013.9 | -0.09611161 | 0.850724615 | 0.903913468 | down |
| CTD-2377D24.6 | -0.096068938 | 0.745410532 | 0.827124587 | down |
| WSB2 | -0.096008264 | 0.666399497 | 0.766894185 | down |
| SNORA2 | -0.095965389 | 0.90243907 | 0.938161918 | down |
| SLC25A51 | -0.095889443 | 0.59962079 | 0.712957378 | down |
| C5AR2 | -0.095797078 | 0.770346232 | 0.84568108 | down |
| STK39 | -0.095590122 | 0.465599527 | 0.597043794 | down |
| RELB | -0.095571737 | 0.592207832 | 0.707165823 | down |
| RP11-733018.1 | -0.095511655 | 0.756187717 | 0.835144965 | down |
| NA | -0.095495944 | 0.602921813 | 0.715805854 | down |
| ELOVL3 | -0.095495056 | 0.855804645 | 0.907613921 | down |
| OTUD5 | -0.095493174 | 0.379779531 | 0.517722956 | down |
| CCDC186 | -0.095427122 | 0.671363081 | 0.770923116 | down |
| CAMKK2 | -0.095327716 | 0.353102646 | 0.491777113 | down |
| RPL15P18 | -0.095283417 | 0.79536304 | 0.863854539 | down |
| TESMIN | -0.095221047 | 0.69900179 | 0.792842411 | down |
| RASSF10 | -0.095193848 | 0.842280881 | 0.897994563 | down |
| PIK3R6 | -0.095101746 | 0.647971235 | 0.752460103 | down |
| PEX5 | -0.095045697 | 0.489964355 | 0.618378033 | down |
| ATP5L | -0.095040941 | 0.377543473 | 0.515687622 | down |
| CPNE2 | -0.094987881 | 0.540006289 | 0.661877813 | down |
| ZNF155 | -0.09498765 | 0.830230353 | 0.889185824 | down |
| DOPEY1 | -0.094941845 | 0.744308592 | 0.826414597 | down |
| COG2 | -0.094896909 | 0.788165277 | 0.858508484 | down |
| RP11-546K22.3 | -0.094883378 | 0.806780704 | 0.872499891 | down |
| LRP1 | -0.094875028 | 0.67321392 | 0.772529661 | down |
| PPP1CA | -0.094730014 | 0.598626572 | 0.712254434 | down |
| NA | -0.094623924 | 0.776593555 | 0.850290699 | down |
| ID2 | -0.094568148 | 0.666051448 | 0.766640476 | down |
| TRMT10A | -0.094567094 | 0.673312199 | 0.772570719 | down |
| AACS | -0.094394501 | 0.444501479 | 0.577987054 | down |
| DND1P1 | -0.094361478 | 0.801706495 | 0.868669573 | down |
| QDPR | -0.094249129 | 0.435423033 | 0.570189147 | down |
| RP11-18B16.2 | -0.094235788 | 0.925307828 | | down |
| ZRANB3 | -0.094172771 | 0.700326324 | 0.793685556 | down |
| FAM21C | -0.094161419 | 0.426992703 | 0.562491973 | down |
| OLMALINC | -0.094107148 | 0.531166153 | 0.654460133 | down |
| RP5-837J1.4 | -0.093892003 | 0.889210842 | 0.929071484 | down |
| TPSD1 | -0.093857183 | 0.86076074 | 0.910844452 | down |
| SLC25A53 | -0.093821368 | 0.560736996 | 0.68004611 | down |
| SCML2P2 | -0.093808883 | 0.891745385 | 0.930805968 | down |
| ATG13 | -0.093761969 | 0.51914292 | 0.643738078 | down |
| CTD-2341M24.1 | -0.093675544 | 0.720703101 | 0.80951114 | down |
| PLEKHA8 | -0.093640533 | 0.657034725 | 0.759337965 | down |
| POMC | -0.093615421 | 0.741813651 | 0.824652162 | down |

| | | | | |
|---|---|---|---|---|
| PKI55 | -0.093558881 | 0.62084372 | 0.730946973 | down |
| AC096921.2 | -0.093549633 | 0.953948248 | | down |
| MKRN5P | -0.093549368 | 0.949741407 | | down |
| DNM3-IT1 | -0.093549328 | 0.948998338 | | down |
| CTD-2313N18.7 | -0.093549162 | 0.945509159 | | down |
| SNORA14B | -0.093549002 | 0.941397367 | | down |
| RP11-736K20.4 | -0.09346375 | 0.782517021 | 0.854503012 | down |
| RP11-196B3.4 | -0.093463327 | 0.908180828 | 0.942372085 | down |
| PCDHB3 | -0.093455611 | 0.834272637 | 0.89180069 | down |
| RP11-552F3.10 | -0.093416519 | 0.808618077 | 0.87358586 | down |
| CNOT2 | -0.093389888 | 0.328706369 | 0.467154879 | down |
| LINC00427 | -0.093380319 | 0.883724378 | 0.925850515 | down |
| RPS7P11 | -0.093352606 | 0.732678747 | 0.818282156 | down |
| FAM200B | -0.093079377 | 0.657507475 | 0.759716736 | down |
| HSP90B2P | -0.093068046 | 0.847832832 | 0.901785937 | down |
| SCNM1 | -0.093019374 | 0.46128274 | 0.593091909 | down |
| ADNP-AS1 | -0.092962104 | 0.77875193 | 0.851660708 | down |
| CNTROB | -0.092871051 | 0.399801841 | 0.536693987 | down |
| TAPT1-AS1 | -0.092812758 | 0.740602671 | 0.823808561 | down |
| PROSC | -0.09277086 | 0.656758702 | 0.759114317 | down |
| SYNJ2 | -0.092752004 | 0.549162369 | 0.669603661 | down |
| RP11-755F10.3 | -0.092725134 | 0.831032251 | 0.88960069 | down |
| GKAP1 | -0.092692376 | 0.651593973 | 0.755316621 | down |
| RP13-104F24.2 | -0.092677446 | 0.772282606 | 0.846897785 | down |
| HMGCS1 | -0.09266931 | 0.736356283 | 0.82089381 | down |
| TEP1 | -0.092656553 | 0.502996359 | 0.629772571 | down |
| MIR4292 | -0.092474618 | 0.818016944 | 0.880625665 | down |
| FOCAD | -0.092404992 | 0.344571024 | 0.483074748 | down |
| KANTR | -0.092395925 | 0.674520864 | 0.773721168 | down |
| ZNF334 | -0.092294342 | 0.769533798 | 0.845086389 | down |
| ALPK1 | -0.092294004 | 0.495393958 | 0.623319209 | down |
| CASP8AP2 | -0.092233642 | 0.784660114 | 0.855909976 | down |
| ATP6AP1 | -0.091947267 | 0.637654536 | 0.744219287 | down |
| FKBP1C | -0.091822473 | 0.736784331 | 0.821190104 | down |
| PXN | -0.091752557 | 0.580762696 | 0.696794634 | down |
| RPL38 | -0.091734124 | 0.613228178 | 0.724491836 | down |
| NLE1 | -0.091733517 | 0.594654231 | 0.708852155 | down |
| PPP6R3 | -0.091657915 | 0.172537062 | 0.296805239 | down |
| AKR1C2 | -0.091630002 | 0.714257808 | 0.803954803 | down |
| RPL32P1 | -0.09160075 | 0.846171405 | 0.90076383 | down |
| CNST | -0.091590049 | 0.501995449 | 0.628886301 | down |
| ATXN2 | -0.091568502 | 0.509634845 | 0.635726356 | down |
| BCL7A | -0.091327346 | 0.755972204 | 0.835138583 | down |
| LA16c-385E7.1 | -0.091307314 | 0.859646295 | 0.910113936 | down |
| LYPLAL1 | -0.091241504 | 0.676481278 | 0.77543699 | down |
| RP11-230F18.5 | -0.091232586 | 0.851178814 | 0.904144261 | down |
| MRPS16 | -0.0909706 | 0.321931165 | 0.460688755 | down |
| RP11-264L1.2 | -0.09094331 | 0.94162335 | | down |
| RP11-13N13.5 | -0.090861232 | 0.884504175 | 0.926414932 | down |
| RP11-563P16.1 | -0.090675186 | 0.894118486 | 0.932512785 | down |
| MRPL4 | -0.090624061 | 0.60680866 | 0.719084896 | down |

| | | | | |
|---|---|---|---|---|
| PON2 | -0.090505429 | 0.619055245 | 0.729482891 | down |
| MCTP2 | -0.09044648 | 0.637202595 | 0.7438652 | down |
| RPS2P41 | -0.090405672 | 0.933074685 | 0.959091855 | down |
| INTS5 | -0.090237072 | 0.636071836 | 0.743003975 | down |
| CTD-2595P9.4 | -0.090227589 | 0.824985725 | 0.885272917 | down |
| EFNA3 | -0.090203241 | 0.768780936 | 0.844506561 | down |
| VDAC1 | -0.090140073 | 0.504808048 | 0.631488238 | down |
| GNAZ | -0.090101069 | 0.750843505 | 0.831429392 | down |
| PCBP3 | -0.090092343 | 0.699560844 | 0.79308728 | down |
| RP11-905K4.1 | -0.090086652 | 0.919712645 | 0.949954081 | down |
| FOXN3 | -0.090002105 | 0.618607499 | 0.729126861 | down |
| GPX1P2 | -0.089854512 | 0.900555927 | | down |
| ZNF799 | -0.089773563 | 0.681652389 | 0.77946123 | down |
| AK3 | -0.089734973 | 0.641335151 | 0.747266755 | down |
| IGLC5 | -0.089684723 | 0.870683222 | 0.917410121 | down |
| RRBP1 | -0.089653472 | 0.706734502 | 0.798358076 | down |
| RP11-88H12.2 | -0.089612425 | 0.919910252 | | down |
| KIF1B | -0.089591757 | 0.635808827 | 0.742757617 | down |
| AUP1 | -0.089548351 | 0.575034304 | 0.6920775 | down |
| ARMCX5-GPRASP2 | -0.089404619 | 0.698124563 | 0.792056734 | down |
| LRFN1 | -0.089305704 | 0.704296632 | 0.796508296 | down |
| GLIPR2 | -0.089260253 | 0.649718117 | 0.753905882 | down |
| EEF1B2 | -0.089141289 | 0.53630304 | 0.658736244 | down |
| AC083949.1 | -0.089117143 | 0.858185899 | 0.909080359 | down |
| TCOF1 | -0.088943959 | 0.56542183 | 0.683492937 | down |
| CHTF18 | -0.088905126 | 0.694961113 | 0.789809792 | down |
| KDM1A | -0.088844792 | 0.365629895 | 0.504106736 | down |
| HIST1H2BG | -0.0887116 | 0.795199125 | 0.863830586 | down |
| MMP25-AS1 | -0.088676516 | 0.679264064 | 0.777588476 | down |
| RP11-1277A3.3 | -0.088493759 | 0.820723786 | 0.882590931 | down |
| CTD-2012K14.7 | -0.088449026 | 0.843304873 | 0.898560932 | down |
| GS1-166A23.2 | -0.088439504 | 0.864819904 | 0.913466784 | down |
| TMEM209 | -0.088435948 | 0.636093904 | 0.743003975 | down |
| TRBC1 | -0.088392368 | 0.800066477 | 0.867518307 | down |
| NA | -0.088368587 | 0.901730286 | | down |
| DRG1 | -0.088314922 | 0.337972783 | 0.47633599 | down |
| RP11-977G19.5 | -0.088173582 | 0.897182021 | 0.934539979 | down |
| SPA17 | -0.08816533 | 0.776911363 | 0.850514685 | down |
| VTCN1 | -0.088103409 | 0.952486156 | | down |
| PCNPP1 | -0.087997595 | 0.890544657 | 0.930141619 | down |
| CEP131 | -0.087919528 | 0.643405317 | 0.748946391 | down |
| RPLP0P6 | -0.087881236 | 0.536921131 | 0.65917888 | down |
| SPTAN1 | -0.087863953 | 0.421027297 | 0.556675795 | down |
| BRE | -0.087846174 | 0.60898301 | 0.721035884 | down |
| ORAI3 | -0.087823879 | 0.66211371 | 0.763600027 | down |
| RP11-771K4.3 | -0.087677863 | 0.816090132 | 0.879244492 | down |
| PARP4 | -0.08756481 | 0.729712317 | 0.816273619 | down |
| C19orf47 | -0.087545486 | 0.538972291 | 0.660987979 | down |
| NA | -0.087474748 | 0.870297153 | 0.917156255 | down |
| PCBP3-OT1 | -0.08735839 | 0.862549551 | 0.912126947 | down |
| GSK3A | -0.087297326 | 0.463727553 | 0.595310092 | down |

| | | | | |
|---|---|---|---|---|
| NA | -0.087282571 | 0.976201394 | | down |
| RP11-756P10.4 | -0.087282571 | 0.976201394 | | down |
| RP1-232L24.4 | -0.087282571 | 0.976201394 | | down |
| NA | -0.087282571 | 0.976201394 | | down |
| RP11-751K21.1 | -0.087282571 | 0.976201394 | | down |
| CTD-307407.7 | -0.087282571 | 0.976201394 | | down |
| H19_3 | -0.087282571 | 0.976201394 | | down |
| FAM166AP2 | -0.087282571 | 0.976201394 | | down |
| RP11-141M1.1 | -0.087282571 | 0.976201394 | | down |
| MIR548X2 | -0.087282571 | 0.976201394 | | down |
| RP11-179A9.3 | -0.087282571 | 0.976201394 | | down |
| MIR138-2 | -0.087282571 | 0.976201394 | | down |
| AC007787.2 | -0.087282571 | 0.976201394 | | down |
| CTD-3233P19.8 | -0.087282571 | 0.976201394 | | down |
| MT-TM | -0.087282571 | 0.976201394 | | down |
| Y_RNA | -0.08728226 | 0.975668498 | | down |
| RP11-192H23.7 | -0.087281003 | 0.973078161 | | down |
| RP5-1021I20.4 | -0.087281001 | 0.973073944 | | down |
| RNU6ATAC40P | -0.087279672 | 0.969171118 | | down |
| CTD-2525I3.3 | -0.087279623 | 0.968992602 | | down |
| LINC01307 | -0.08727954 | 0.968684582 | | down |
| GML | -0.087279509 | 0.96856718 | | down |
| MIR1273C | -0.087279417 | 0.968210816 | | down |
| LINC00517 | -0.087278774 | 0.965315489 | | down |
| AC009133.15 | -0.087278717 | 0.965018129 | | down |
| RP11-445O16.2 | -0.087278701 | 0.964934458 | | down |
| ALDOAP2 | -0.087278699 | 0.964924576 | | down |
| RP11-256I23.1 | -0.087278093 | 0.961178947 | | down |
| RP1-224A6.8 | -0.087278077 | 0.961066241 | | down |
| MTAPP1 | -0.087278031 | 0.960727539 | | down |
| PPIAP2 | -0.087277956 | 0.960151753 | | down |
| RP13-444K19.1 | -0.087277175 | 0.952198951 | | down |
| BCAP31P2 | -0.087276893 | 0.947888338 | | down |
| AC002398.13 | -0.087230793 | 0.806210826 | 0.872042058 | down |
| PSMD9 | -0.087210648 | 0.715707535 | 0.80522458 | down |
| SARS2 | -0.087108856 | 0.830601966 | 0.889330202 | down |
| ZSWIM4 | -0.08696376 | 0.766152051 | 0.842697134 | down |
| IFRD1 | -0.086824625 | 0.484542309 | 0.613390179 | down |
| ITIH5 | -0.086768007 | 0.652298881 | 0.755745386 | down |
| LINC00589 | -0.086758485 | 0.87187706 | 0.918209282 | down |
| KCNQ5 | -0.086736373 | 0.850501624 | 0.90378981 | down |
| SLC44A2 | -0.086717239 | 0.582461998 | 0.698465689 | down |
| ABCA3 | -0.086676634 | 0.697928924 | 0.791904054 | down |
| NA | -0.086674041 | 0.861399328 | 0.911262025 | down |
| ZC3H10 | -0.086603254 | 0.56905028 | 0.686911753 | down |
| ZNF808 | -0.086547806 | 0.66599297 | 0.766602536 | down |
| THAP8 | -0.08652571 | 0.548017931 | 0.668805374 | down |
| FAHD2B | -0.086520636 | 0.713413517 | 0.803433556 | down |
| LOXL4 | -0.08648265 | 0.750520841 | 0.831347865 | down |
| Xbac-BPG248L24.[illegible] | -0.086455198 | 0.916543167 | 0.94806148 | down |
| C14orf28 | -0.086442349 | 0.617790313 | 0.728406963 | down |

| | | | | |
|---|---|---|---|---|
| APLP2 | -0.086439962 | 0.676509684 | 0.775439966 | down |
| BAALC-AS1 | -0.086349854 | 0.762359972 | 0.839848305 | down |
| RP11-347E10.1 | -0.086321409 | 0.864777496 | 0.913466784 | down |
| BACH2 | -0.086180349 | 0.82241695 | 0.883577562 | down |
| SHFM1 | -0.086168669 | 0.551596682 | 0.671617861 | down |
| RP1-193H18.3 | -0.086115925 | 0.899743917 | 0.936066642 | down |
| PGLYRP3 | -0.085994105 | 0.942346014 | | down |
| NAPA-AS1 | -0.085914117 | 0.791778608 | 0.861267071 | down |
| RP4-717I23.2 | -0.085888016 | 0.862984612 | 0.912298019 | down |
| RP11-637A17.2 | -0.085867139 | 0.845588846 | 0.900339203 | down |
| CTC-543D15.8 | -0.08576863 | 0.807434962 | 0.872861831 | down |
| RP1-223H12.5 | -0.08518448 | 0.959792455 | 0.975807887 | down |
| VPS37D | -0.085155584 | 0.734847208 | 0.819944927 | down |
| NA | -0.085036469 | 0.945652987 | | down |
| ALKBH4 | -0.085029454 | 0.53664861 | 0.658959684 | down |
| CYB5RL | -0.084924231 | 0.744334277 | 0.826414597 | down |
| PLXNA3 | -0.084849356 | 0.624952966 | 0.73433276 | down |
| C12orf73 | -0.084631725 | 0.670420556 | 0.770180428 | down |
| HMGN2 | -0.084589956 | 0.576093082 | 0.693101896 | down |
| CHEK1 | -0.084569514 | 0.638684866 | 0.745074478 | down |
| RFC5 | -0.084567936 | 0.625996047 | 0.735095769 | down |
| C12orf49 | -0.08449362 | 0.42694171 | 0.562465982 | down |
| RP11-873E20.1 | -0.084487818 | 0.838769622 | 0.895363103 | down |
| AKAP7 | -0.084215069 | 0.707558424 | 0.799078498 | down |
| RNA5SP208 | -0.084141875 | 0.968562583 | | down |
| RP11-368J22.2 | -0.084141405 | 0.965499741 | | down |
| RP11-309L24.6 | -0.084140439 | 0.954705479 | | down |
| PRM1 | -0.084140423 | 0.954426414 | | down |
| SERBP1P6 | -0.084140115 | 0.947933869 | | down |
| BX842568.2 | -0.084139987 | 0.944286793 | | down |
| RP11-254G11.1 | -0.084139566 | 0.922955577 | | down |
| PRMT1 | -0.084124486 | 0.661604075 | 0.763196017 | down |
| CHMP3 | -0.084030506 | 0.691417701 | 0.787062987 | down |
| SNRPD1 | -0.084002986 | 0.578821499 | 0.695242998 | down |
| BEND3 | -0.083990114 | 0.692015251 | 0.78741502 | down |
| AC073257.2 | -0.083969237 | 0.882548264 | 0.924876348 | down |
| GLCE | -0.083940617 | 0.703659491 | 0.796171647 | down |
| DPY19L1P1 | -0.083838107 | 0.71575423 | 0.805246961 | down |
| TRIM49 | -0.083805324 | 0.969175801 | | down |
| CDK9 | -0.083730257 | 0.452205454 | 0.585076015 | down |
| RALGAPA1 | -0.083715661 | 0.738482881 | 0.822262612 | down |
| BIK | -0.083628772 | 0.760916502 | 0.838627124 | down |
| OVGP1 | -0.083543312 | 0.697963632 | 0.791904054 | down |
| LZTS3 | -0.083508541 | 0.770375699 | 0.84568108 | down |
| C19orf43 | -0.083492699 | 0.583573331 | 0.699440585 | down |
| SREBF2 | -0.083413549 | 0.573867387 | 0.690977548 | down |
| ACTA1 | -0.08338979 | 0.849750582 | 0.903236071 | down |
| CC2D2A | -0.083298243 | 0.722333119 | 0.810725783 | down |
| CTD-2353F22.1 | -0.083275195 | 0.859076189 | 0.909702701 | down |
| NKAP | -0.0831913 | 0.469131726 | 0.600050814 | down |
| RP11-43F13.1 | -0.083023716 | 0.569476299 | 0.687201167 | down |

| | | | | |
|---|---|---|---|---|
| NECTIN4 | -0.082899137 | 0.83107947 | 0.889619539 | down |
| BEND4 | -0.082855024 | 0.847004456 | 0.90136731 | down |
| ALG11 | -0.082733393 | 0.752231866 | 0.832338661 | down |
| RPS6 | -0.082569015 | 0.595679146 | 0.709787588 | down |
| SMU1 | -0.082544416 | 0.259916827 | 0.395095494 | down |
| TAC1 | -0.082537251 | 0.864134179 | 0.913127718 | down |
| FAM184A | -0.082322311 | 0.813857816 | 0.877531733 | down |
| GCDH | -0.082243533 | 0.514359771 | 0.639654993 | down |
| AC007389.1 | -0.082228141 | 0.9522391 | 0.971640987 | down |
| NA | -0.082178409 | 0.8096597 | 0.87426689 | down |
| WDR70 | -0.082176192 | 0.241565485 | 0.375476922 | down |
| CRY2 | -0.082111129 | 0.594672747 | 0.708852155 | down |
| DTNBP1 | -0.082065354 | 0.457492006 | 0.589529909 | down |
| FOXRED1 | -0.081927162 | 0.492211393 | 0.620458131 | down |
| TOMM5 | -0.081891437 | 0.823634343 | 0.884398374 | down |
| PMS2P4 | -0.081890402 | 0.73836827 | 0.822262612 | down |
| KDM4A-AS1 | -0.081848707 | 0.832211056 | 0.890386701 | down |
| TARBP2 | -0.081779986 | 0.651612685 | 0.755316621 | down |
| JPH2 | -0.081753871 | 0.819117507 | 0.881327798 | down |
| CDC25A | -0.081713334 | 0.713323428 | 0.803384655 | down |
| KIF9 | -0.081500403 | 0.675591108 | 0.774623488 | down |
| LINC01431 | -0.081351011 | 0.783646477 | 0.85541787 | down |
| ELMSAN1 | -0.081258524 | 0.742351341 | 0.825143926 | down |
| DEAF1 | -0.081224816 | 0.428691234 | 0.56405358 | down |
| EHBP1L1 | -0.081155059 | 0.582736662 | 0.698688653 | down |
| ZNF561-AS1 | -0.081114332 | 0.656738625 | 0.759114317 | down |
| WWC2-AS2 | -0.081041868 | 0.874534157 | 0.920093678 | down |
| FNDC8 | -0.08093022 | 0.918073369 | | down |
| FFAR1 | -0.080920897 | 0.892288333 | 0.931056531 | down |
| RP11-180M15.6 | -0.080903628 | 0.834546271 | 0.891932275 | down |
| DMTN | -0.080550328 | 0.614907974 | 0.725991086 | down |
| CTNNA1 | -0.080529978 | 0.653712289 | 0.756932671 | down |
| MED7 | -0.080469397 | 0.585072526 | 0.700798713 | down |
| ARHGEF10L | -0.080464224 | 0.691108287 | 0.786800204 | down |
| TRAV30 | -0.080455669 | 0.909728533 | 0.943454747 | down |
| KLHDC8B | -0.08044455 | 0.632892972 | 0.740503016 | down |
| HLA-DQB2 | -0.080374913 | 0.823903916 | 0.884554362 | down |
| CTD-228808.1 | -0.080363526 | 0.87987502 | 0.922976331 | down |
| PMP22 | -0.080329209 | 0.752969544 | 0.832709427 | down |
| HECTD4 | -0.080324406 | 0.690178896 | 0.785980394 | down |
| MAP3K2 | -0.080252393 | 0.591665485 | 0.70665863 | down |
| LINC01583 | -0.080193537 | 0.962297852 | | down |
| ZEB2-AS1 | -0.080113817 | 0.878061363 | 0.922039621 | down |
| SPAG8 | -0.080100728 | 0.796423625 | 0.864473116 | down |
| NKX2-3 | -0.080097837 | 0.787659924 | 0.858074166 | down |
| PRND | -0.08004669 | 0.876483483 | 0.921329624 | down |
| PLPP7 | -0.080033689 | 0.722456515 | 0.810732671 | down |
| AP000442.1 | -0.080012457 | 0.85176912 | 0.904643363 | down |
| NIPAL2 | -0.079987816 | 0.687334351 | 0.78360237 | down |
| RP11-88E10.4 | -0.079843028 | 0.870278073 | 0.917156255 | down |
| NA | -0.079803066 | 0.83173362 | 0.890066068 | down |

| | | | | |
|---|---|---|---|---|
| ZNF764 | -0.079732906 | 0.549950538 | 0.670374243 | down |
| HIST1H1C | -0.079719886 | 0.7778067 | 0.851050848 | down |
| PARP3 | -0.079624279 | 0.682875709 | 0.780355171 | down |
| ZNF543 | -0.079552859 | 0.786894469 | 0.857437383 | down |
| RP11-164H13.1 | -0.079495207 | 0.896638509 | 0.934265359 | down |
| RP11-1020A11.1 | -0.079474835 | 0.834778617 | 0.892117932 | down |
| SUPT16HP1 | -0.079462393 | 0.915874588 | | down |
| FAM58A | -0.079281798 | 0.637052136 | 0.743813743 | down |
| RP11-399J13.2 | -0.079278995 | 0.890822028 | 0.930334296 | down |
| PPP1CC | -0.07923337 | 0.539069902 | 0.661068419 | down |
| S100A1 | -0.079111891 | 0.841226365 | 0.897156825 | down |
| ICA1 | -0.07906163 | 0.496311119 | 0.623950593 | down |
| RP11-741G21.1 | -0.079005234 | 0.964224166 | 0.977960666 | down |
| AC097523.1 | -0.078888331 | 0.875542841 | 0.920738657 | down |
| TMEM59 | -0.078770832 | 0.668798012 | 0.768976941 | down |
| SPATA5 | -0.078607836 | 0.710841539 | 0.801490775 | down |
| RP11-212I21.4 | -0.078571948 | 0.895212408 | 0.933177452 | down |
| C3orf18 | -0.078450175 | 0.744030954 | 0.826309138 | down |
| MIEF1 | -0.078420497 | 0.410978864 | 0.547759684 | down |
| ANKMY2 | -0.078221964 | 0.707710985 | 0.799160672 | down |
| DDX41 | -0.078079867 | 0.500126794 | 0.627225305 | down |
| CMPK2 | -0.078057597 | 0.771924503 | 0.846655772 | down |
| RP11-804N13.1 | -0.077873521 | 0.978766239 | | down |
| TRBV26 | -0.077873521 | 0.978766239 | | down |
| RPL34P21 | -0.077873521 | 0.978766239 | | down |
| RP11-290D2.5 | -0.077873521 | 0.978766239 | | down |
| CTD-2553L13.4 | -0.077873521 | 0.978766239 | | down |
| RP11-436M15.3 | -0.077873385 | 0.978558319 | | down |
| ATP6V0E1P1 | -0.077871768 | 0.975476722 | | down |
| RP11-746M1.8 | -0.077870576 | 0.972109365 | | down |
| KATNBL1P6 | -0.077870498 | 0.971837746 | | down |
| MIR8058 | -0.077870478 | 0.971766478 | | down |
| RP11-127O4.3 | -0.077870407 | 0.971509905 | | down |
| NA | -0.077869771 | 0.968850796 | | down |
| MACC1-AS1 | -0.077869672 | 0.968362265 | | down |
| AC007193.10 | -0.077869086 | 0.96494366 | | down |
| MIR103A2 | -0.077869069 | 0.964821878 | | down |
| RP11-214N16.2 | -0.077869044 | 0.964648554 | | down |
| RP11-179B15.5 | -0.077867775 | 0.949904829 | | down |
| CTD-2587M2.1 | -0.077864798 | 0.843067758 | 0.898482768 | down |
| QPCTL | -0.077797547 | 0.778722702 | 0.851659745 | down |
| NA | -0.077619841 | 0.906026018 | 0.940946651 | down |
| DNAJC15 | -0.077611174 | 0.64470551 | 0.749769 | down |
| RP11-613F7.1 | -0.077570131 | 0.836150093 | 0.8931566 | down |
| FIG4 | -0.077548885 | 0.283724266 | 0.420408949 | down |
| LENG1 | -0.077478474 | 0.635683307 | 0.742668739 | down |
| ELP6 | -0.077432831 | 0.605339382 | 0.717881755 | down |
| BAG6 | -0.077363798 | 0.452874574 | 0.585488347 | down |
| RPS2P46 | -0.077306056 | 0.691780538 | 0.787297945 | down |
| ORC2 | -0.077300003 | 0.585365429 | 0.701001233 | down |
| REPS2 | -0.077227729 | 0.712654348 | 0.802983676 | down |

| | | | | |
|---|---|---|---|---|
| KIAA1324L | -0.077198199 | 0.767229518 | 0.843480812 | down |
| MKNK1 | -0.077128841 | 0.439567718 | 0.573491945 | down |
| NA | -0.076923406 | 0.807829908 | 0.873096083 | down |
| KHDC1 | -0.076758131 | 0.798215642 | 0.866011511 | down |
| PDPK1 | -0.076705654 | 0.756386856 | 0.835268149 | down |
| FTSJ3 | -0.076599095 | 0.46954855 | 0.600321504 | down |
| AC096670.3 | -0.076595861 | 0.878746109 | 0.922307357 | down |
| PTPRO | -0.076110078 | 0.735511854 | 0.820256798 | down |
| LINC00494 | -0.076055369 | 0.854925337 | 0.907129677 | down |
| KTN1 | -0.076043019 | 0.615350625 | 0.726360777 | down |
| PRMT9 | -0.075949793 | 0.735026803 | 0.82000764 | down |
| PRIMPOL | -0.075899719 | 0.814496498 | 0.878007311 | down |
| L3MBTL2 | -0.075797451 | 0.625524695 | 0.734714568 | down |
| RPS4X | -0.07576206 | 0.633354816 | 0.740841429 | down |
| RPL36AP43 | -0.075647435 | 0.897452546 | 0.934724547 | down |
| SLMAP | -0.07536549 | 0.761855989 | 0.839416215 | down |
| SPDYE19P | -0.075345299 | 0.944070078 | | down |
| NIT2 | -0.075323239 | 0.43266609 | 0.567618008 | down |
| RPL31 | -0.075282373 | 0.61245909 | 0.723786651 | down |
| AGO3 | -0.075256403 | 0.660527531 | 0.762326718 | down |
| PSKH1 | -0.075253351 | 0.736969995 | 0.821364637 | down |
| TMEM186 | -0.07509396 | 0.64623483 | 0.751159787 | down |
| RBM5 | -0.075047551 | 0.506174914 | 0.632488542 | down |
| PRDX1P1 | -0.074942772 | 0.900926812 | 0.936913989 | down |
| HSPD1P11 | -0.074847101 | 0.946289182 | | down |
| ODF3B | -0.074775969 | 0.789176456 | 0.859307677 | down |
| RP11-266L9.3 | -0.074731088 | 0.950823776 | | down |
| CDCA4P1 | -0.074730969 | 0.947033963 | | down |
| PES1P2 | -0.074730966 | 0.946910338 | | down |
| TLR9 | -0.074730881 | 0.94360612 | | down |
| RP11-686D22.5 | -0.074730646 | 0.929564741 | | down |
| RP11-752G15.8 | -0.07469146 | 0.946365857 | 0.967622373 | down |
| XIST | -0.074587788 | 0.925823768 | 0.95411962 | down |
| ZNF585A | -0.074491391 | 0.743949755 | 0.826309138 | down |
| RPL21P131 | -0.074459066 | 0.958007335 | 0.975058416 | down |
| POPDC3 | -0.074427939 | 0.890345926 | 0.929966382 | down |
| MTND5P1 | -0.074419608 | 0.925304808 | | down |
| RP11-666A8.8 | -0.074373201 | 0.8850304 | 0.926701751 | down |
| GRHPR | -0.074325681 | 0.564286249 | 0.682449724 | down |
| RP1-128M12.3 | -0.074249754 | 0.948669771 | | down |
| AC010642.1 | -0.074231766 | 0.873697891 | 0.919675546 | down |
| RP11-1081L13.4 | -0.074198465 | 0.884622352 | 0.926468224 | down |
| LDLRAD4-AS1 | -0.074067041 | 0.926103938 | 0.95427742 | down |
| RP11-70L8.4 | -0.074066864 | 0.911640302 | 0.944719966 | down |
| MED14 | -0.074031208 | 0.67881371 | 0.777231461 | down |
| RPS3AP34 | -0.07398021 | 0.909914018 | | down |
| RAB11B | -0.073946976 | 0.60064337 | 0.713777763 | down |
| CMPK1 | -0.07394148 | 0.629030658 | 0.737391123 | down |
| PCSK2 | -0.073857196 | 0.857949285 | 0.908902976 | down |
| TCAF1P1 | -0.073841662 | 0.917316697 | 0.948560449 | down |
| PPP2R5A | -0.07382203 | 0.421732739 | 0.557314203 | down |

| | | | | |
|---|---|---|---|---|
| GTF2IP12 | -0.073768994 | 0.755521447 | 0.834732702 | down |
| RP1-153P14.7 | -0.073745928 | 0.899832365 | 0.936099847 | down |
| RP4-669P10.20 | -0.073668514 | 0.836108546 | 0.8931566 | down |
| SNORD14A | -0.073507193 | 0.828373788 | 0.887862085 | down |
| LYL1 | -0.073492822 | 0.804527242 | 0.870721054 | down |
| RP11-356B19.11 | -0.073471659 | 0.852510145 | 0.905174399 | down |
| PAX5 | -0.073456748 | 0.866860421 | 0.914839858 | down |
| TOP1MT | -0.073247771 | 0.608419586 | 0.72061834 | down |
| IREB2 | -0.073229954 | 0.704205797 | 0.796489256 | down |
| RNU6-1011P | -0.073213751 | 0.928144736 | | down |
| TRAK2 | -0.072949907 | 0.716814553 | 0.806168167 | down |
| GLRX5 | -0.072944725 | 0.45804567 | 0.590066208 | down |
| TGFB2 | -0.072941846 | 0.862494918 | 0.912101278 | down |
| PIGBOS1 | -0.072773676 | 0.648153113 | 0.752642219 | down |
| RP11-211A18.2 | -0.072762532 | 0.95917722 | 0.975608382 | down |
| ZSWIM7 | -0.07273411 | 0.580664736 | 0.696733964 | down |
| IL27RA | -0.072698997 | 0.754460467 | 0.833959171 | down |
| RP11-413M3.4 | -0.072553191 | 0.886877791 | 0.927660602 | down |
| LY6G5C | -0.072517584 | 0.802942317 | 0.869412872 | down |
| RNF220 | -0.072500561 | 0.373443795 | 0.511896404 | down |
| RP11-59C5.3 | -0.072492772 | 0.847736146 | 0.901785937 | down |
| SEC61A1 | -0.072482562 | 0.771410053 | 0.846249813 | down |
| FNBP1L | -0.072430335 | 0.766938079 | 0.843252978 | down |
| BCKDHB | -0.072265303 | 0.744721553 | 0.826661285 | down |
| CDC37L1-AS1 | -0.072219179 | 0.781881218 | 0.854026006 | down |
| RP11-613D13.4 | -0.072132183 | 0.866587055 | 0.914625922 | down |
| SORD2P | -0.072130173 | 0.855674488 | 0.907574025 | down |
| NA | -0.072073958 | 0.857737724 | 0.908810521 | down |
| ENKD1 | -0.072028811 | 0.723759029 | 0.811699439 | down |
| OSER1 | -0.071924938 | 0.603198615 | 0.715993015 | down |
| TUBAP2 | -0.071862863 | 0.846238473 | 0.900775507 | down |
| SOCS7 | -0.071828155 | 0.627172706 | 0.735959722 | down |
| ZNF707 | -0.071741563 | 0.521764905 | 0.64610934 | down |
| TMEM150C | -0.071710507 | 0.806974692 | 0.872590554 | down |
| RNU11 | -0.071602735 | 0.980475736 | | down |
| GJA9 | -0.071602735 | 0.980475736 | | down |
| RP5-850O15.3 | -0.071602735 | 0.980475736 | | down |
| RNU6-1176P | -0.071602735 | 0.980475736 | | down |
| RP11-131O15.2 | -0.071602735 | 0.980475736 | | down |
| RN7SL370P | -0.071602735 | 0.980475736 | | down |
| Y_RNA | -0.071602735 | 0.980475736 | | down |
| MIR942 | -0.071602735 | 0.980475736 | | down |
| GAPDHP33 | -0.071602735 | 0.980475736 | | down |
| NA | -0.071602735 | 0.980475736 | | down |
| NA | -0.071602735 | 0.980475736 | | down |
| MRPS10P1 | -0.071602735 | 0.980475736 | | down |
| RP11-203F10.5 | -0.071602735 | 0.980475736 | | down |
| ATP5G2P1 | -0.071602735 | 0.980475736 | | down |
| UBBP2 | -0.071602735 | 0.980475736 | | down |
| RP5-1087E8.2 | -0.071602735 | 0.980475736 | | down |
| SNORA51 | -0.071602735 | 0.980475736 | | down |

| | | | |
|---|---|---|---|
| RP11-80B9.4 | -0.071602735 | 0.980475736 | down |
| AC013480.2 | -0.071602735 | 0.980475736 | down |
| AC009236.2 | -0.071602735 | 0.980475736 | down |
| AC097463.1 | -0.071602735 | 0.980475736 | down |
| AC007403.3 | -0.071602735 | 0.980475736 | down |
| IGKV1-35 | -0.071602735 | 0.980475736 | down |
| MIR4266 | -0.071602735 | 0.980475736 | down |
| MIR4436B1 | -0.071602735 | 0.980475736 | down |
| RPL12P15 | -0.071602735 | 0.980475736 | down |
| CYP4F30P | -0.071602735 | 0.980475736 | down |
| RP11-470B22.1 | -0.071602735 | 0.980475736 | down |
| MTATP6P17 | -0.071602735 | 0.980475736 | down |
| NA | -0.071602735 | 0.980475736 | down |
| RP11-118M12.2 | -0.071602735 | 0.980475736 | down |
| RN7SL147P | -0.071602735 | 0.980475736 | down |
| NDUFB4P1 | -0.071602735 | 0.980475736 | down |
| PDZRN3-AS1 | -0.071602735 | 0.980475736 | down |
| THAP12P2 | -0.071602735 | 0.980475736 | down |
| Y_RNA | -0.071602735 | 0.980475736 | down |
| RNU6-42P | -0.071602735 | 0.980475736 | down |
| RN7SKP292 | -0.071602735 | 0.980475736 | down |
| RNU6-1112P | -0.071602735 | 0.980475736 | down |
| RPL12P20 | -0.071602735 | 0.980475736 | down |
| RNU6-469P | -0.071602735 | 0.980475736 | down |
| PMPCAP1 | -0.071602735 | 0.980475736 | down |
| RN7SL254P | -0.071602735 | 0.980475736 | down |
| RP11-360A10.1 | -0.071602735 | 0.980475736 | down |
| RNU6-1196P | -0.071602735 | 0.980475736 | down |
| RNU6-1285P | -0.071602735 | 0.980475736 | down |
| RNU6-128P | -0.071602735 | 0.980475736 | down |
| Y_RNA | -0.071602735 | 0.980475736 | down |
| SPCS2P3 | -0.071602735 | 0.980475736 | down |
| NA | -0.071602735 | 0.980475736 | down |
| RP11-414H23.3 | -0.071602735 | 0.980475736 | down |
| RP11-213H15.1 | -0.071602735 | 0.980475736 | down |
| HMGN1P13 | -0.071602735 | 0.980475736 | down |
| AC005754.7 | -0.071602735 | 0.980475736 | down |
| CTB-180C19.1 | -0.071602735 | 0.980475736 | down |
| MIR378E | -0.071602735 | 0.980475736 | down |
| MIR3912 | -0.071602735 | 0.980475736 | down |
| CTC-209H22.3 | -0.071602735 | 0.980475736 | down |
| RNU6-226P | -0.071602735 | 0.980475736 | down |
| Y_RNA | -0.071602735 | 0.980475736 | down |
| HCG4P8 | -0.071602735 | 0.980475736 | down |
| MIR3925 | -0.071602735 | 0.980475736 | down |
| MYB-AS1 | -0.071602735 | 0.980475736 | down |
| SNORD56 | -0.071602735 | 0.980475736 | down |
[truncated: 740,276 more chars]
